# Mortality, morbidity, and risk factors in China and its provinces, 1990–2017: a systematic analysis for the Global Burden of Disease Study 2017

**DOI:** 10.1016/S0140-6736(19)30427-1

**Published:** 2019-09-28

**Authors:** Maigeng Zhou, Haidong Wang, Xinying Zeng, Peng Yin, Jun Zhu, Wanqing Chen, Xiaohong Li, Lijun Wang, Limin Wang, Yunning Liu, Jiangmei Liu, Mei Zhang, Jinlei Qi, Shicheng Yu, Ashkan Afshin, Emmanuela Gakidou, Scott Glenn, Varsha Sarah Krish, Molly Katherine Miller-Petrie, W Cliff Mountjoy-Venning, Erin C Mullany, Sofia Boston Redford, Hongyan Liu, Mohsen Naghavi, Simon I Hay, Linhong Wang, Christopher J L Murray, Xiaofeng Liang

**Affiliations:** a National Center for Chronic and Noncommunicable Disease Control and Prevention, Chinese Center for Disease Control and Prevention, Beijing, China; b Chinese Center for Disease Control and Prevention, Beijing, China; c Institute for Health Metrics and Evaluation, University of Washington, Seattle, WA, USA; d China Population and Development Research Center, Beijing, China; e National Office for Maternal and Child Health Surveillance, Chengdu, China; f National Cancer Center/National Clinical Research Center for Cancer/Cancer Hospital, Chinese Academy of Medical Science and Peking Union Medical College, Beijing, China

## Abstract

**Background:**

Public health is a priority for the Chinese Government. Evidence-based decision making for health at the province level in China, which is home to a fifth of the global population, is of paramount importance. This analysis uses data from the Global Burden of Diseases, Injuries, and Risk Factors Study (GBD) 2017 to help inform decision making and monitor progress on health at the province level.

**Methods:**

We used the methods in GBD 2017 to analyse health patterns in the 34 province-level administrative units in China from 1990 to 2017. We estimated all-cause and cause-specific mortality, years of life lost (YLLs), years lived with disability (YLDs), disability-adjusted life-years (DALYs), summary exposure values (SEVs), and attributable risk. We compared the observed results with expected values estimated based on the Socio-demographic Index (SDI).

**Findings:**

Stroke and ischaemic heart disease were the leading causes of death and DALYs at the national level in China in 2017. Age-standardised DALYs per 100 000 population decreased by 33·1% (95% uncertainty interval [UI] 29·8 to 37·4) for stroke and increased by 4·6% (–3·3 to 10·7) for ischaemic heart disease from 1990 to 2017. Age-standardised stroke, ischaemic heart disease, lung cancer, chronic obstructive pulmonary disease, and liver cancer were the five leading causes of YLLs in 2017. Musculoskeletal disorders, mental health disorders, and sense organ diseases were the three leading causes of YLDs in 2017, and high systolic blood pressure, smoking, high-sodium diet, and ambient particulate matter pollution were among the leading four risk factors contributing to deaths and DALYs. All provinces had higher than expected DALYs per 100 000 population for liver cancer, with the observed to expected ratio ranging from 2·04 to 6·88. The all-cause age-standardised DALYs per 100 000 population were lower than expected in all provinces in 2017, and among the top 20 level 3 causes were lower than expected for ischaemic heart disease, Alzheimer's disease, headache disorder, and low back pain. The largest percentage change at the national level in age-standardised SEVs among the top ten leading risk factors was in high body-mass index (185%, 95% UI 113·1 to 247·7]), followed by ambient particulate matter pollution (88·5%, 66·4 to 116·4).

**Interpretation:**

China has made substantial progress in reducing the burden of many diseases and disabilities. Strategies targeting chronic diseases, particularly in the elderly, should be prioritised in the expanding Chinese health-care system.

**Funding:**

China National Key Research and Development Program and Bill & Melinda Gates Foundation.

## Introduction

Over the past four decades, China has seen rapid demographic and epidemiological transitions, along with an economic boom that has already lifted millions out of poverty. Fundamental transformations in overall population health, as measured by life expectancy, child mortality, disease profile, and risk factors, have occurred and are continuing.^1^ However, few studies have systematically documented these transitions.^1^, ^2^, ^3^, ^4^ Such information is crucial in helping population health professionals and policy makers understand the health priorities in China at the national and subnational levels.

The Healthy China 2030 plan^5^, ^6^ is the signature national domestic population health policy and has made population health the ultimate goal of economic development and political reform. Two of the goals are to improve longevity and healthy life expectancy and to increase disease prevention.^7^ Other targets include improvements in infant mortality, the environment (eg, air quality), health services and insurance (eg, chronic disease mortality), lifestyle (eg, regular exercise), and the health industry. Health equity is also a priority,^5^ but health problems and access to health-care providers and services remain heterogeneous between the provinces in mainland China.^2^ Close examination of population health metrics at the national and province levels will be crucial to developing evidence-based policies and achieving the Healthy China 2030 goals.

**Research in context**

**Evidence before this study**

A detailed analysis of 240 causes of death at the province level in China was done in the Global Burden of Disease, Injuries, and Risk Factors Study (GBD) 2013. Data in China are primarily available from the Disease Surveillance Point system, the Maternal and Child Surveillance System, the Chinese Center for Disease Control and Prevention cause-of-death reporting system (reported by community health workers and hospitals at province level), cancer registries, and reports from Hong Kong and Macau. However, existing data on death, disability, and risks have not been consistently analysed over time. In this study, we apply the suite of methodologies developed for GBD 2017 to analyse systematically the burden of disease in China at the subnational level.

**Added value of this study**

This is the first comprehensive subnational assessment of health to include data on mortality, morbidity, combined health loss, and risks in China. Major improvements have been made to the GBD methods since the 2013 subnational analysis of population health in China. These include additional causes of death and disability, greater disaggregation of age groups, substantially expanded data sources, the development of data quality ratings, improvements to estimation models, the development of the Socio-demographic Index, consideration of summary exposure values for risk factors, and a fully updated time series of estimates, among other improvements.

**Implications of all the available evidence**

Subnational analysis of health patterns, including mortality, disability, and risk, provide important information for government planning at the province level. Our study identifies areas where the disease burden is being successfully reduced. It also reveals differences in health outcomes between provinces that can guide interventions, particularly in the western rural regions where health outcomes are typically poorer than in other regions. Health factors that need increased attention include high blood pressure, diet, non-communicable diseases, tobacco smoking and exposure to ambient air pollution, and chronic illnesses in the elderly. Health policies in China affect the largest population on the planet, magnifying the importance of evidence-based, data-driven decision making for health.

China is positioned to transform into a much more active participant in improving health in Asia, Africa, and beyond. The Belt and Road Initiative, which China announced in 2013, is a plan to integrate economic development across 65 countries in Asia, Africa, and Europe through promotion of trade, infrastructure, and commercial ties, and includes a substantial health component.^8^ The Beijing Communique, which has been signed by health ministers and representatives from international organisations, including WHO and The Global Fund to Fight AIDS, Tuberculosis and Malaria, has laid out key population health priorities for the countries involved in the Belt and Road Initiative.^9^ An internally consistent and comparable assessment of mortality, disability, and related risk factors for China and its partner countries will be paramount for the initiative's success. As China is transitioning from being an aid recipient to an aid donor, its experience in improving population health and tackling important health issues could help to shape its bilateral and multilateral health aid policies.

This analysis of the Global Burden of Diseases, Injuries, and Risk Factors Study (GBD) 2017 is the result of a long-term close collaboration between the Chinese Center for Disease Control and Prevention and the Institute for Health Metrics and Evaluation (the GBD coordinating centre), along with other agencies of the Chinese Government, including the Center for Health Statistics and Information at the National Health Commission, the National Office for Maternal and Child Health Surveillance, and the National Office for Cancer Control and Prevention, and individual researchers at academic institutions worldwide. Through such collaboration, we can bring together health information from otherwise mostly siloed sources and analyse the information with the methods developed through the GBD collaboration. Here we present national and provincial results for mortality, causes of death, morbidity, and risk factors from 1990 to 2017.

## Methods

### Overview

We employed the suite of estimation methods for burden of disease used in GBD 2017 to assess the state of population health at the province level in China. The GBD 2017 methods have been described in full detail elsewhere.^10^, ^11^, ^12^, ^13^, ^14^, ^15^, ^16^ Metrics generated in evaluating the burden of disease in China include all-cause mortality; cause-specific mortality and years of life lost (YLLs) for 282 causes of death; years lived with disability (YLDs) for 354 diseases and injuries and 3484 sequelae; combined health loss for 359 diseases and injuries presented as disability-adjusted life-years (DALYs); and attributable deaths, YLLs, DALYs, and summary exposure values (SEVs) for 84 risk factors or clusters of risks. Metrics were also analysed in relation to the Socio-demographic Index (SDI), a composite indicator based on fertility, income per person, and years of education. Uncertainty is reported with each estimate and propagated throughout the GBD modelling process.

To ensure transparency and replicability, our study follows the Guidelines for Accurate and Transparent Health Estimates Reporting (GATHER),^17^ and all data sources (except those protected by data usage agreement), code, and results are publicly available online. A comprehensive explanation of inclusion and exclusion criteria and limitations have been previously described elsewhere.^18^

### Geographical units and time periods

All relevant burden of disease metrics were estimated separately for the two sexes and for all age groups that are standard in GBD 2017. Our estimation processes cover the years from 1950 to 2017 for all demographic indicators, and from 1990 to 2017 for all other burden of disease metrics for mortality, morbidity, and associated risk factors. This analysis includes 34 province-level administrative units in China (Xinjiang Production and Construction Corps is excluded). These province-level units consist of 23 provinces, five autonomous regions, four municipalities, and two special administrative regions but are all termed provinces throughout the Article. Because of province-level administrative changes since 1950 (eg, the creation of Chongqing Municipality in 1997), we have used empirical data sources, including censuses and the Disease Surveillance Point system, to separate data to match the current province boundaries. Estimates are provided for the following age groups: early neonatal (0–6 days); late neonatal (7–27 days); postneonatal (28–365 days); and childhood and adult (1–4 years and all subsequent 5-year age groups from 5–9 to 90–94 years, and ≥95 years). In this Article, the discussion is focused on the province-level burden of disease for mainland China and the two SARs

### All-cause and cause-specific mortality

For all-cause mortality, to ensure consistency we scaled each province-level estimate for the 32 provinces in mainland China to match the separately estimated all-cause mortality for mainland China as a whole. Hong Kong and Macau special administrative regions were not included in this scaling process because the mortality and demographic data collection processes are separate from that on the mainland. Data for all-cause mortality in China were derived primarily from the Disease Surveillance Point system; censuses; surveys (including the Annual Survey on Population Change and the Intercensal Survey), which provide data for children younger than 5 years, adults, and age pattern of mortality; and the Maternal and Child Health Surveillance system and One-per-Thousand Population Fertility Sample Survey, which provide data on children younger than 5 years. Standard GBD data processing procedures and analytical models were applied to generate all-cause mortality estimates. Detailed descriptions of these methods can be found in previous GBD publications.^10^, ^11^, ^18^

Cause-specific mortality was estimated primarily with the GBD cause of death ensemble modelling tool, CODEm.^11^ Unlike all-cause mortality, we estimated cause-specific mortality only at the province level. Data on causes of death for the provinces were primarily derived from surveillance systems, including the Disease Surveillance Point system and the Maternal and Child Surveillance System, as well as from surveys, the China Cancer Registry, and the Chinese Center for Disease Control and Prevention cause-of-death reporting system. The cause-of-death data in the Disease Surveillance Point system are a rich source for understanding mortality levels and trends in China from the early 1990s onwards. Since 1978, this national system has included 605 surveillance points with 323·8 million population covered.

Since the SARS epidemic in 2003, China has been establishing a national electronic cause-of-death reporting system. Over the past 15 years, and especially since 2008, coverage and completeness of recording have increased substantially. In 2016, the system covered more than 80% of the deaths nationally. Given the heterogeneous nature of the epidemiological profiles across provinces and between urban and rural regions, and the fact that completeness of reported deaths in and out of hospital differ, we stratified the 2852 counties in China by urban and rural status, as defined in the 2010 census in China (appendix). We then reweighted reported in-hospital and out-of-hospital deaths in counties without Disease Surveillance Points by using the fractions of deaths reported by the system in provinces with urban or rural status. Province-level cause-specific mortality was generated from these estimates by combining stratum-level mortality weighted by the size, age, and sex of the population in each stratum per year. Causes of death for Hong Kong and Macau were based on medical certification data reported to WHO. All cause-of-death data for China were mapped from the codes in the International Classification of Diseases ninth or tenth edition to the GBD cause hierarchy. To ensure consistency across estimates, cause-specific mortality estimates were scaled to match all-cause mortality estimates with the GBD CoDCorrect process.

### Fertility and population

To ensure that estimates of key demographic indicators, such as population and fertility, were internally consistent with estimates of burden of disease, we estimated these factors at the national and subnational levels. Age-specific fertility rates for women aged 10–54 years in 5-year age groups were estimated from 1950 to 2017 by methods detailed by Murray and colleagues.^12^ Input data on age-specific fertility were extracted from censuses, annual surveys on population change, and surveys on fertility, including the One-per-Thousand Population Fertility Sample Survey. To ensure internal consistency, we scaled estimates of age-specific fertility rates across the 32 mainland provinces to estimated age-specific fertility rates for the mainland as one unit.

We estimated population sizes for all 34 provinces and for mainland China as one unit. Because we were unable to parse out the effects of boundary changes on some provinces in the 1953 census, we only included censuses from 1964, 1982, 1990, 2000, and 2010. To estimate populations, we used a Bayesian hierarchical model developed by Wheldon and colleagues^19^, ^20^, ^21^ and improved by Murray and colleagues.^12^ This method uses the estimates of age-specific vital rates on mortality and fertility to ensure maximum internal consistency.

### Morbidity and disability-adjusted life-years

Prevalence and incidence of non-fatal outcomes were primarily estimated with the Bayesian meta-regression method DisMod-MR 2.1.^13^ Briefly, this meta-analysis tool uses a compartmental model structure with a series of differential equations that synthesise sparse and heterogeneous epidemiological data.^14^ Data on non-fatal outcomes were derived primarily from published studies, national surveys, cancer registries, the Chinese Center for Disease Control and Prevention cause-of-death reporting system, and hospital inpatient data. DALYs represent a combined measure of health loss from fatal and non-fatal outcomes. They were estimated as the sum of YLLs and YLDs.^14^, ^22^

### Risk factors

Exposures, attributable mortality, and attributable DALYs were estimated for 84 risk factors categorised as behavioural, environmental and occupational, or metabolic. Briefly, data on relative risks and exposures were derived from over 46 000 empirical data points primarily extracted from randomised controlled trials and cohort and pooled cohort studies. Statistical models were used to pool empirical data and adjust for biases while accounting for effects of relevant covariates. With the counterfactual scenario of theoretical minimum risk exposure level, we estimated attributable burden based on the estimated exposure per risk. Finally, we computed a summary exposure value (SEV) for each risk included in our analysis, by province over time. Each SEV ranges from 0 to 100% and indicates what percentage of the population is exposed to the maximum possible level of a specific risk.^15^

### Socio-demographic Index

The SDI is a combined measure of the rescaled total fertility rates among women younger than 25 years, years of educational attainment in the population older than 15 years, and lag-distributed income per person.^12^ Each factor is scaled from 0 to 1, and the geometric mean of the three values produces a final index score of 0–1. The national SDI for China in 2017 was 0·71 and at the province level was 0·47–0·86.^12^

To generate expected values based on the SDI, we used Gaussian regression to estimate the relationship between SDI and health metrics for each age, sex, year, and province. We then compared observed values with expected values.

### Role of the funding source

The funder of the study had no role in study design, data collection, data analysis, data interpretation, or writing of the report. The corresponding author had full access to all the data in the study and had responsibility for the decision to submit for publication.

## Results

Mortality, YLLs, and percentage changes from 1990 to 2017 for the 25 leading causes of death in China in 2017 are shown in table 1. Stroke, ischaemic heart disease, lung cancer, chronic obstructive pulmonary disease, and liver cancer were the five leading causes of YLLs in 2017. Stroke became the top leading cause of YLLs in 2017, despite a decrease from 1990 in age-standardised mortality of 33·5% (95% uncertainty interval [UI] 30·1–38·7). By contrast, age-standardised mortality from ischaemic heart disease increased by 20·6% (10·1–27·5). For chronic obstructive pulmonary disease, age-standardised mortality declined by 68·6% (62·0–70·9) from 1990 to 2017, but it remained the fourth leading cause of YLLs. Increases were seen in YLLs and age-standardised mortality for lung cancer (12·6%, 1·9–20·7 and 28·2%, 16·5–36·8, respectively), leading to a change in ranking from 13th in 1990 to third in 2017. Age-standardised mortality decreased by more than 50% from 1990 to 2017 for seven among the top 25 causes: chronic obstructive pulmonary disease, neonatal disorders, lower respiratory infections, self-harm, cirrhosis and other chronic liver diseases, congenital birth defects, and drowning. Increases in age-standardised mortality were seen for seven causes. Among them, ischaemic heart disease, lung cancer, and pancreatic cancer increased by more than 20% and were the only three causes that had significant increases. All other causes decreased by less than 50%.

**Table 1:** Deaths and YLLs with percentage changes from 1990 and 2017 for the 25 leading causes of death in China

	**YLL rank**		**YLL per 100 000 population (95% UI)**		**Percentage change in YLL (95% UI)**		**Deaths per 100 000 population (95% UI)**		**Percentage change in mortality (95% UI)**	
	1990	2017	1990	2017	All ages	Age standardised	1990	2017	All ages	Age standardised
Stroke	3	1	2298 (2223 to 2464)	2633 (2541 to 2730)	14·6 (7·1 to 20·5)	−38·8 (−43·0 to −35·6)	106 (102 to 115)	149 (145 to 155)	41·0 (30·6 to 48·3)	−33·5 (−38·7 to −30·1)
Ischaemic heart disease	7	2	1077 (1032 to 1155)	2057 (1984 to 2134)	91·1 (77·1 to 102·7)	4·8 (−3·3 to 11·1)	49 (47 to 53)	124 (120 to 128)	155·4 (134·4 to 170·5)	20·6 (10·1 to 27·5)
Tracheal, bronchus, and lung cancer	13	3	526 (501 to 562)	1065 (1015 to 1112)	102·7 (83·3 to 117·5)	12·6 (1·9 to 20·7)	20 (19 to 21)	49 (47 to 51)	144·0 (121·4 to 161·1)	28·2 (16·5 to 36·8)
Chronic obstructive pulmonary disease	4	4	1764 (1535 to 1846)	952 (909 to 1045)	−46·1 (−50·0 to −35·1)	−72·6 (−74·5 to −66·9)	99 (85 to 103)	68 (65 to 76)	−30·7 (−35·7 to −16·3)	−68·6 (−70·9 to −62·0)
Liver cancer	11	5	644 (599 to 682)	781 (736 to 826)	21·2 (10·8 to 35·2)	−28·5 (−34·6 to −20·2)	20 (19 to 22)	30 (28 to 31)	44·5 (32·7 to 60·6)	−20·3 (−26·7 to −11·9)
Road injuries	5	6	1087 (1020 to 1194)	751 (714 to 784)	−31·0 (−38·6 to −24·8)	−33·3 (−40·4 to −27·7)	19 (18 to 21)	19 (18 to 19)	−4·9 (−16·2 to 3·8)	−21·8 (−30·7 to −14·9)
Stomach cancer	12	7	613 (591 to 648)	541 (519 to 566)	−11·7 (−18·1 to −5·8)	−50·8 (−54·5 to −47·6)	24 (23 to 25)	25 (24 to 26)	5·8 (−2·4 to 12·6)	−44·9 (−49·4 to −41·4)
Alzheimer's disease and other dementias	28	8	179 (171 to 194)	374 (360 to 387)	109·0 (91·0 to 122·5)	−10·7 (−18·1 to −5·3)	14 (13 to 15)	35 (33 to 36)	154·6 (133·1 to 170·0)	−6·3 (−13·7 to −1·1)
Neonatal disorders	2	9	2352 (2191 to 2526)	336 (302 to 365)	−85·7 (−87·6 to −84·0)	−76·7 (−79·7 to −73·9)	27 (25 to 29)	4 (3 to 4)	−85·7 (−87·6 to −84·0)	−76·7 (−79·7 to −73·9)
Hypertensive heart disease	17	10	329 (212 to 367)	312 (198 to 347)	−5·3 (−16·6 to 12·8)	−51·3 (−57·8 to −42·0)	18 (12 to 20)	21 (13 to 23)	20·4 (4·2 to 42·6)	−45·2 (−54·0 to −34·6)
Oesophageal cancer	16	11	337 (322 to 357)	312 (296 to 327)	−7·6 (−15·0 to −0·6)	−50·3 (−54·3 to −46·6)	14 (13 to 15)	15 (14 to 16)	6·9 (−1·5 to 15·0)	−45·2 (−49·6 to −41·1)
Lower respiratory infection	1	12	3142 (2868 to 3380)	302 (282 to 356)	−90·4 (−91·4 to −88·2)	−88·7 (−89·9 to −86·4)	45 (41 to 47)	13 (12 to 16)	−71·5 (−74·3 to −63·3)	−76·8 (−78·8 to −70·7)
Self-harm	9	13	902 (754 to 954)	295 (278 to 329)	−67·3 (−70·3 to −59·1)	−71·9 (−74·4 to −64·7)	19 (16 to 20)	9 (9 to 10)	−52·5 (−56·8 to −41·4)	−65·6 (−68·7 to −57·6)
Cirrhosis and other chronic liver diseases	15	14	421 (334 to 455)	292 (268 to 371)	−30·6 (−40·1 to 1·4)	−57·3 (−63·1 to −38·2)	13 (10 to 14)	11 (10 to 14)	−13·5 (−25·8 to 26·3)	−51·2 (−58·2 to −29·1)
Colon and rectum cancer	29	15	174 (163 to 190)	286 (270 to 300)	64·7 (45·0 to 81·1)	−4·7 (−16·1 to 4·7)	6 (6 to 7)	13 (13 to 14)	108·9 (83·3 to 129·5)	8·2 (−5·6 to 18·6)
Chronic kidney disease	20	16	286 (274 to 306)	264 (244 to 275)	−7·7 (−14·8 to −1·9)	−38·8 (−44·1 to −34·9)	8 (8 to 9)	12 (11 to 13)	47·4 (29·0 to 57·3)	−19·0 (−30·2 to −13·6)
Congenital disorders	6	17	1080 (986 to 1196)	260 (239 to 288)	−76·0 (−79·2 to −71·8)	−61·5 (−66·8 to −54·8)	13 (12 to 14)	3 (3 to 4)	−74·8 (−78·1 to −70·6)	−60·8 (−66·2 to −54·2)
Falls	22	18	243 (213 to 317)	229 (168 to 257)	−5·5 (−42·7 to 13·9)	−18·9 (−50·1 to −2·3)	5 (5 to 7)	10 (7 to 11)	78·2 (15·2 to 110·6)	12·8 (−23·9 to 33·4)
Drowning	8	19	1050 (974 to 1125)	218 (208 to 228)	−79·2 (−80·7 to −77·6)	−71·4 (−73·4 to −69·2)	14 (13 to 15)	5 (4 to 5)	−68·7 (−70·9 to −66·4)	−65·3 (−67·5 to −62·9)
Diabetes mellitus	34	20	139 (132 to 147)	215 (207 to 224)	55·3 (44·0 to 65·8)	−10·4 (−17·0 to −4·5)	5 (5 to 6)	11 (10 to 11)	103·3 (87·6 to 117·6)	6·1 (−2·4 to 13·7)
Breast cancer	39	21	108 (98 to 133)	169 (144 to 183)	56·5 (−1·2 to 80·6)	−7·3 (−42·3 to 6·7)	3 (3 to 4)	6 (5 to 7)	86·0 (13·6 to 114·8)	2·5 (−38·1 to 18·2)
Leukaemia	18	22	320 (226 to 367)	157 (132 to 169)	−50·8 (−59·6 to −37·8)	−48·4 (−58·5 to −34·8)	6 (4 to 6)	4 (4 to 5)	−24·9 (−35·9 to −12·4)	−38·9 (−48·5 to −30·5)
Brain and central nervous system cancer	30	23	150 (100 to 203)	135 (114 to 159)	−10·1 (−30·2 to 31·0)	−23·7 (−40·9 to 11·2)	3 (2 to 4)	4 (4 to 5)	35·5 (8·9 to 73·0)	−7·9 (−26 to 15·8)
Pancreatic cancer	57	24	56 (53 to 59)	133 (126 to 139)	137·4 (118·8 to 155·4)	32·7 (22·5 to 42·6)	2 (2 to 2)	6 (6 to 6)	184·8 (163·4 to 205·2)	47·5 (36·4 to 57·8)
Other malignant neoplasms	35	25	132 (108 to 141)	119 (103 to 126)	−10·0 (−18·4 to 8·6)	−31·8 (−37·5 to −18·3)	3 (3 to 4)	5 (4 to 5)	33·5 (17·8 to 49·5)	−19·3 (−28·7 to −11·3)

Causes are presented by YLL ranking in 2017. YLL=years of life lost. UI=uncertainty interval.

The top three causes of YLDs in 1990 and 2017 were musculoskeletal disorders, mental disorders, and sense organ diseases (table 2). Age-standardised YLDs for transport injuries increased by 32·1% (95% UI 24·5–40·0) from 1990 to 2017, unintentional injuries increased by 27·8% (19·7–36·2), and cardiovascular diseases increased by 20·9% (17·6–24·4). Of the 22 leading causes of YLDs in 2017, the age-standardised YLD rate increased for 11 from 1990.

**Table 2:** YLDs and percentage changes from 1990 to 2017 for the leading 22 causes of disability and injury in China

	**YLD rank**		**YLDs per 100 000 population (95% UI)**		**Percentage change (95% UI)**	
	1990	2017	1990	2017	All-age YLD	Age-standardised YLD
Other musculoskeletal disorders	2	1	1316 (956 to 1754)	1774 (1291 to 2364)	34·8 (29·1 to 40·4)	−7·0 (−9·7 to −4·4)
Mental health disorders	1	2	1388 (1034 to 1800)	1574 (1169 to 2031)	13·4 (10·4 to 16·5)	−3·8 (−5·3 to −2·4)
Sense organ disease	3	3	664 (442 to 970)	1007 (671 to 1462)	51·6 (49·6 to 53·7)	−4·2 (−5·5 to −3·3)
Other cardiovascular diseases	10	4	351 (256 to 449)	729 (532 to 937)	107·6 (99·9 to 115·4)	20·9 (17·6 to 24·4)
Neurological disorders	6	5	550 (376 to 766)	694 (480 to 943)	26·1 (18·5 to 35·5)	0·4 (−4·8 to 6·4)
Other non-communicable diseases	4	6	646 (444 to 902)	686 (467 to 984)	6·3 (2·1 to 10·9)	−13·1 (−15·0 to −11·1)
Chronic respiratory diseases	5	7	626 (520 to 716)	625 (527 to 718)	−0·2 (−5·3 to 4·6)	−32·0 (−35·1 to −28·6)
Diabetes and kidney diseases	11	8	343 (243 to 470)	577 (400 to 792)	68·3 (60·3 to 77·0)	5·0 (−0·3 to 10·2)
Skin and subcutaneous diseases	7	9	515 (333 to 778)	499 (325 to 751)	−3·0 (−5·8 to −0·2)	2·4 (1·1 to 4·1)
Substance use disorders	8	10	469 (330 to 614)	490 (345 to 642)	4·4 (−2·1 to 11·4)	0·6 (−4·5 to 6·4)
Maternal and neonatal disorders	12	11	312 (231 to 411)	459 (336 to 612)	47·2 (33·8 to 62·3)	66·4 (51·0 to 83·9)
Unintentional injuries	15	12	203 (147 to 271)	348 (244 to 481)	71·6 (59·2 to 84·8)	27·8 (19·7 to 36·2)
Digestive diseases	13	13	257 (176 to 359)	337 (233 to 470)	31·3 (26·6 to 35·9)	1·4 (−1·1 to 4·1)
Transport injuries	17	14	139 (100 to 184)	254 (179 to 342)	83·0 (70·9 to 95·7)	32·1 (24·5 to 40·0)
Respiratory infections and tuberculosis	16	15	175 (119 to 247)	143 (98 to 201)	−18·2 (−20·9 to −15·2)	−19·9 (−23·7 to −16·6)
Neoplasms	21	16	48 (35 to 63)	139 (102 to 183)	188·1 (155·6 to 215·3)	87·3 (63·1 to 112·0)
Neglected tropical diseases and malaria	14	17	204 (123 to 320)	122 (70 to 207)	−40·3 (−53·3 to −26·9)	−46·8 (−57·9 to −35·3)
Self-harm and interpersonal violence	18	18	111 (84 to 143)	117 (90 to 152)	5·9 (1·1 to 11·1)	−6·9 (−11·4 to −3·2)
Nutritional deficiencies	9	19	445 (299 to 650)	117 (76 to 178)	−73·7 (−76·9 to −70·0)	−72·7 (−76·1 to −69·0)
Enteric infections	19	20	61 (42 to 84)	48 (33 to 66)	−21·6 (−26·6 to −16·8)	−9·7 (−15·5 to −2·8)
Other infectious diseases	20	21	52 (36 to 72)	30 (21 to 42)	−42·6 (−46·5 to −38·9)	−44·8 (−48·0 to −41·8)
HIV/AIDS and sexually transmitted diseases other than HIV	22	22	20 (12 to 34)	27 (17 to 43)	37·8 (16·3 to 84·8)	22·9 (6·4 to 60·0)

Causes are presented by YLD ranking in 2017. YLD=years lived with disability. UI=uncertainty interval.

**Figure 1:**
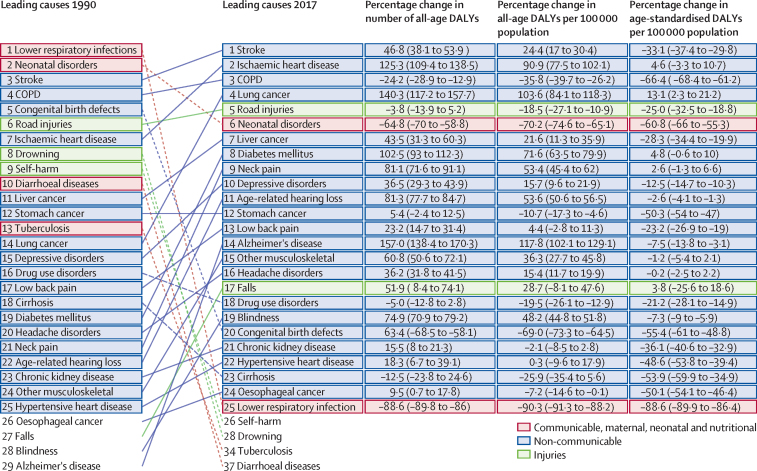
Top 25 causes of DALYs in China, 1990–2017 Causes are ranked by number of DALYs in 2017. COPD=chronic obstructive pulmonary disease. DALY=disability-adjusted life-years.

The 25 leading causes of DALYs, ranked by number of DALYs in 2017, are shown in figure 1. For 18 causes, the number of DALYs increased from 1990 to 2017. Stroke and ischaemic heart disease were the leading causes of all-age DALYs in 2017, replacing lower respiratory infections and neonatal disorders in 1990. The absolute numbers and rates per 100 000 population for all-age DALYs increased substantially for stroke and ischaemic heart disease between 1990 and 2017 (DALY counts increased by 46·8%, 95% UI 38·1–53·9 and 125·3%, 109·4–138·5, respectively), but age-standardised stroke decreased by 33·1% (29·8–37·4) during this period. High systolic blood pressure accounted for 2·54 million deaths (95% UI 2·26 million–2·82 million) in 2017, with 95·7% of these deaths (95·6–96·4) being due to cardiovascular diseases. The age-standardised DALYs per 100 000 population for chronic obstructive pulmonary disease decreased by 66·4% (95% UI 61·2–68·4) between 1990 and 2017, and increased by 13·1% (2·3–21·2) for lung cancer. Lower respiratory infections declined from being the leading cause of all-age DALYs in 1990 to the 25th leading cause in 2017. This change is indicative of a large decline in DALYs from communicable, maternal, neonatal, and nutritional conditions as a whole. Age-standardised DALY rates for injuries also declined over this period, with the greatest reductions seen in drowning (71·3%, 95% UI 69·1–73·3) and self-harm (71·8%, 64·7–74·3), leading them to fall outside the top 25 causes. By contrast, falls increased in rank from 27th in 1990 to 17th in 2017. Age-standardised DALYs per 100 000 population decreased from 1990 to 2017 for 17 of 21 non-communicable causes, with chronic obstructive pulmonary disease, congenital defects, cirrhosis, and stomach cancer showing the largest declines.

**Figure 2:**
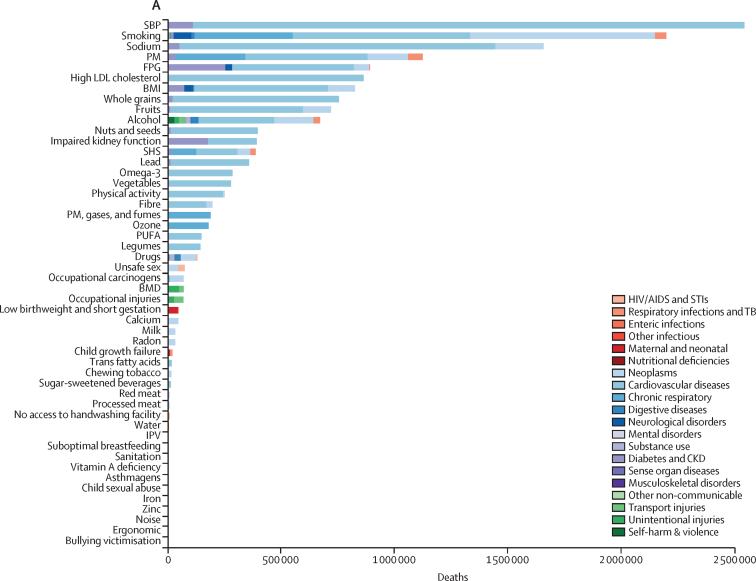

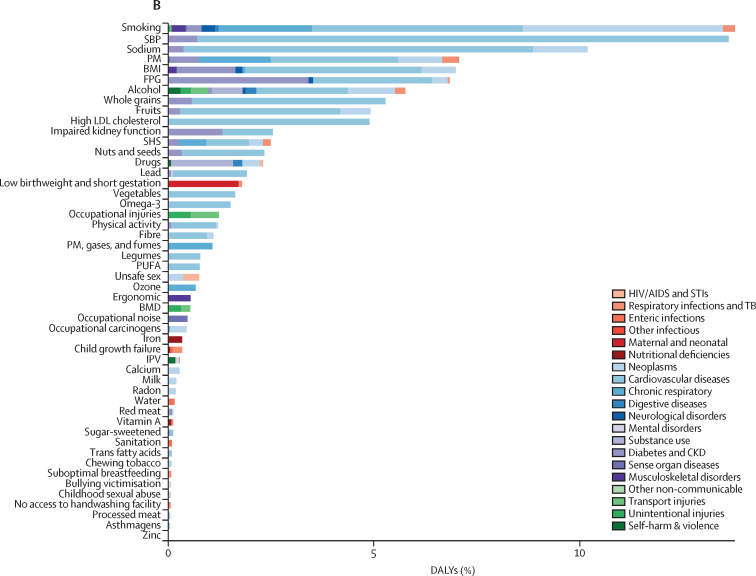
Number of deaths and percentage of DALYs related to the leading level 3 risk factors in China in 2017 (A) Risk factors and related deaths. (B) Risk factors as percentages of DALYs. Alcohol=alcohol use disorders. Asthmagens=occupational asthmagens. BMD=low bone mineral density. BMI=high body-mass index. Calcium=diet low in calcium. CKD=chronic kidney disease. Drugs=drug use disorders. Ergonomic=occupational ergonomic factors. Fibre=diet low in fibre. FPG=high fasting plasma glucose. Fruits=diet low in fruits. IPV=intimate partner violence. Iron=iron-deficiency anaemia. Milk=diet low in milk. Nuts and seeds=diet low in nuts and seeds. Lead=lead exposure. Legumes=diet low in legumes. Noise=occupational noise. Omega 3=diet low in seafood omega-3 fatty acids. Ozone=ambient ozone pollution. Physical activity=low physical activity. PM=particulate matter pollution. PM, gases, and fumes=occupational particulate matter, gases, and fumes. Processed meat=diet high in processed meat. PUFA=diet low in polyunsaturated fatty acids. Radon=residential radon. Red meat=diet high in red meat. Sanitation=unsafe sanitation. SBP=high systolic blood pressure. SHS=second hand smoke. Sodium=diet high in sodium. Sugar-sweetened beverages=diet high in sugar-sweetened beverages. TB=tuberculosis. Vegetables=diet low in vegetables. Water=unsafe water. Whole grains=diet low in whole grains. Zinc=zinc deficiency.

The numbers of deaths and percentages of DALYs by leading level 3 risk factors, ranked by values in 2017, are shown in figure 2. High systolic blood pressure, smoking, diet high in sodium, and particulate matter pollution were the top four risk factors for both number of deaths and percentage of DALYs in 2017. High systolic blood pressure accounted for 2·54 million (95% UI 2·26 million to 2·82 million) deaths in 2017, of which 95·7% (95·6–96·4) were due to cardiovascular diseases. Alcohol use was the tenth leading contributor to the number of deaths. Each of the eight following risks accounted for more than 5% of DALYs in 2017: smoking, high systolic blood pressure, diet high in sodium, particulate matter pollution, high body-mass index, high fasting plasma glucose, alcohol use, and diet low in whole grains.

**Figure 3:**
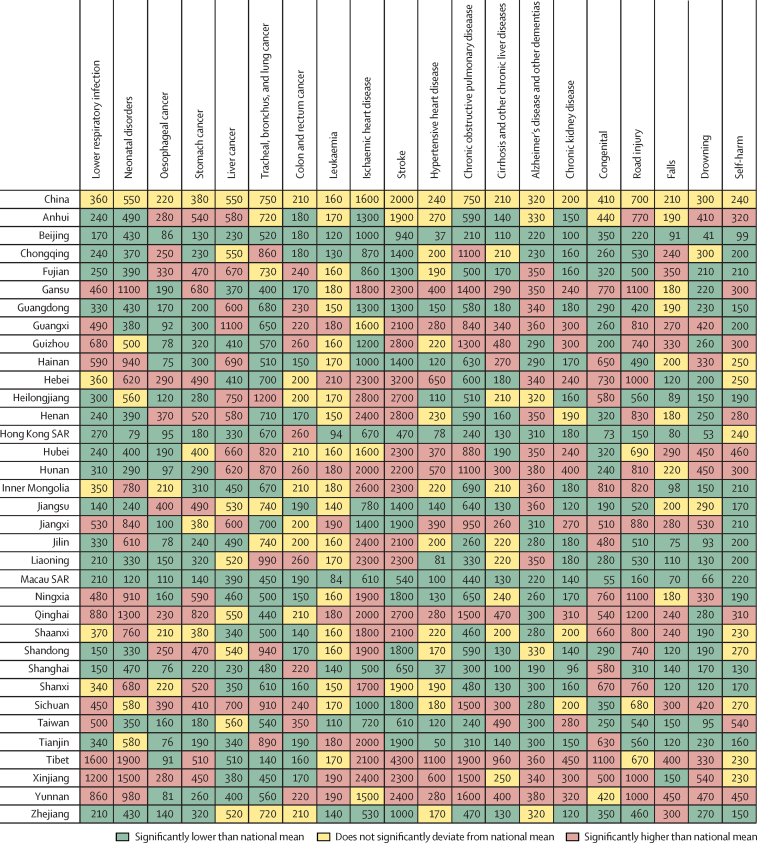
Age-standardised YLLs per 100 000 population for the top 20 level 3 causes in China, 2017, compared with the national averages SAR=special administration region.

When province-level age-standardised YLLs were compared with the national average for the top 20 causes, Beijing and Macau were the only two provinces with significantly lower values for all top 20 causes (figure 3). In Shanghai, age-standardised YLLs were significantly lower than the national mean for all top 20 causes except for colon and rectum cancer and congenital birth defects, which were significantly higher. In Hong Kong, all were significantly lower except colon and rectum cancer, which was significantly higher, and self-harm, which did not differ significantly from the national average.

**Figure 4:**
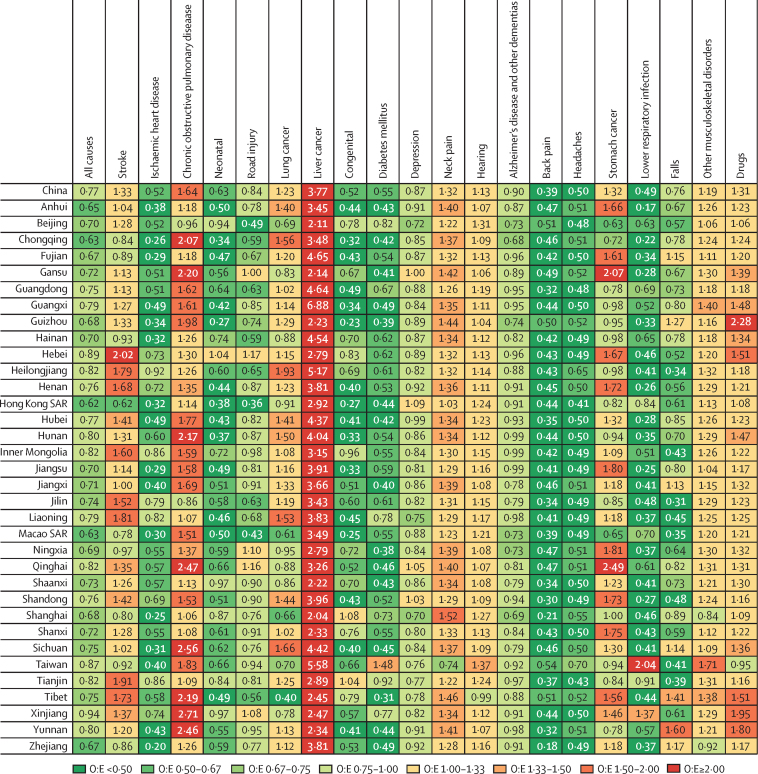
Ratio of observed to expected age-standardised DALYs per 100 000 population for the top 20 level 3 causes in China and provinces in 2017 O:E=ratio of observed to expected. SAR=special administration region.

In all provinces, the ratio of observed to expected age-standardised all-cause DALYs per 100 000 population was lower than expected based on the SDI (figure 4). 12 provinces had an observed age-standardised all-cause DALYs per 100 000 population at least 30% lower than expected. These provinces have widely varying levels of social and economic development (appendix). Thus, although lower-than-expected values are unsurprising for the 16 provinces with high and high-middle SDI values and good public health intervention programmes, such as Zhejiang, it is noteworthy that the remaining 18 provinces with lower levels of economic development, such as Ningxia, also achieved these results. Age-standardised DALYs for ischaemic heart disease, Alzheimer's disease, headache disorder, and low back pain were lower than expected in all provinces in 2017 (figure 4). All provinces had higher than expected age-standardised DALYs for liver cancer, with the observed-to-expected ratio ranging from 2·04 to 6·88. For lower respiratory infections, congenital birth defects, diabetes mellitus, and neonatal disorders, nearly all provinces had age-standardised DALYs lower than expected.

The percentage changes in age-standardised SEVs for the top ten risk factors from 1990 to 2017 in China were greatest for high body-mass index and ambient particulate matter pollution (appendix). Age-standardised SEVs for high body-mass index, ambient particulate matter pollution, alcohol use, high blood pressure, and high LDL cholesterol increased for all mainland provinces, with high body-mass index increasing the most by 185% (95% UI 113·1–247·7), followed by ambient particulate matter pollution (88·5%, 66·4–116·4). Conversely, age-standardised SEVs for low fruit consumption decreased in all 34 provinces during the time period. High fasting plasma glucose increased in all provinces except Hainan. We found substantial heterogeneity between provinces in the change of SEVs by risk. The age-standardised SEV for high sodium consumption increased in 16 of 34 provinces.

In 2017, smoking was the leading risk factor for DALYs in China overall and in 21 provinces, and was second or third in all remaining provinces (appendix). High systolic blood pressure and diet high in sodium were the second and third leading risks for DALYs at the national level. Smoking, high blood pressure, and diet high in sodium were the top three risk factors for DALYs in all provinces in mainland China except seven, where diet high in sodium ranked between fourth and sixth. Notably, high systolic blood pressure or smoking was the first or second leading risk factor for DALYs in all provinces except Xinjiang. Diet low in vegetables was ranked much lower in Beijing, Shanghai, Guangdong, Jiangsu, Zhejiang, and Fujian than in the other provinces.

## Discussion

Over the period 1990 to 2017, health outcomes underwent dramatic changes across the provinces of China. The rates of YLLs, YLDs, and DALYs due to communicable, maternal, neonatal, and nutritional conditions largely decreased during the study period, while those caused by non-communicable diseases generally increased. The national burden of disease has, therefore, shifted considerably, with communicable, maternal, neonatal, and nutritional conditions accounting for a much smaller proportion of DALYs in 2017 than in 1990. Exposure to some risk factors is rising, particularly high fasting plasma glucose, high systolic blood pressure, high body-mass index, and, in many provinces, ambient particulate matter pollution.

Compared with other locations at a similar level of development as measured by SDI, China has unusually high levels of stroke, chronic obstructive pulmonary disease, lung cancer, liver cancer, neck pain, and stomach cancer. The top two leading causes of disease burden by all-age DALYs are stroke and ischaemic heart disease, which have seen significant increases in numbers and crude rates of all-age DALYs between 1990 and 2017, even though age-standardised DALYs for stroke have significantly decreased during the same time period. At the province level the disease burden from these two disorders shows substantial heterogeneity. The provinces with higher YLL rates due to stroke and ischaemic heart disease are those with poorer economic performance, except Shandong. Further investigation using the associated risk factors analysed in our study for these two diseases will be necessary for policy makers at the national and provincial levels to develop targeted intervention programmes to ease the associated burden.

The decreasing burden of communicable, maternal, neonatal, and nutritional conditions has occurred alongside rapid declines in maternal mortality and mortality among children younger than 5 years.^23^, ^24^ Rapid and sustained economic growth and increasing levels of educational attainment are likely to have contributed to these changes.^25^, ^26^ The observed burden indicates that China has been more successful than expected based on the SDI in lessening these health burdens. China has used a range of national programmes to target interventions for communicable, maternal, neonatal, and nutritional conditions,^4^, ^27^ particularly in women and children. One of the most ambitious among such programmes is the Reducing Maternal Mortality and Eliminating Neonatal Tetanus Program, which aims to improve obstetric care quality systematically in every county of China. Maternal mortality has declined in 98·6% of all counties in China since the launch of this programme in 2000.^28^ These factors constitute credible explanations for the greater-than-expected progress.

Age-standardised DALYs per 100 000 population have also reduced rapidly in many provinces for several non-communicable diseases and injuries, most notably self-harm, stomach cancer, oesophageal cancer, and chronic obstructive pulmonary disease. Suicide rates have consistently declined since 1990, particularly among young women. This trend is partly explained by urbanisation and development accompanied by improved opportunities for women and young people.^29^, ^30^ Declines in some cancers alongside increases in others reinforces the heterogeneous burden of cancers and the need for specific, targeted approaches for cancer prevention and control according to province and cancer type. For instance, DALYs for liver cancer were higher than expected in all provinces, whereas the greatest relative difference in the observed to expected ratio across provinces was for lung cancer. Reduced disease burden from chronic obstructive pulmonary disease is consistent with previous findings from the region, which attributed declines primarily to improvements in access to health care, diagnosis, and treatment.^3^ Declines in mortality varied across provinces, highlighting remaining health disparities and the need to continue to implement interventions to improve care and reduce related exposures, such as to tobacco and pollution.

An important aspect of the transition underway in China is the rising mean age of the population, which has been driven by a rapid decline in fertility rate from the 1970s and declining age-specific mortality rates.^12^ With the mean population age rising, age-related conditions, such as musculoskeletal disorders, have increasing importance for the health system. Musculoskeletal disorders can cause important limitations to general activity and work and introduce considerable economic and medical burdens to individuals, families, and governments. However, these disorders receive little attention from public health policy makers. In addition, a growing portion of the working-age population spends most of its time in office environments, leading to increased prevalence of low back and neck pain, which suggests the need for relevant policies to prevent this occupational hazard.^31^

High systolic blood pressure and smoking are the leading risk factors. Government policies, such as Healthy China 2020 and 2030, have been put in place partly to promote risk reduction and encourage healthier lifestyles.^5^ Healthy China 2030 is a national health policy issued by the central Government of China that aims to adopt the most pertinent health policies or strategies for improving the overall health of the Chinese population between 2016 and 2030.^5^ We have found some slow progress in reducing tobacco consumption, but, surprisingly, no progress in lowering systolic blood pressure, which has increased since 1990 despite reductions in dietary sodium in 16 provinces. Given the huge burden of elevated systolic blood pressure and tobacco consumption, policies to address these two risk factors need to be intensified.

Many diet-related risks increased from 1990 to 2017, particularly high body-mass index. Changing lifestyles, including increased consumption of red meat and decreasing levels of physical activity, have probably driven the rapidly rising rates of diabetes in China.^32^, ^33^ The Chinese government has started to deal with the challenges posed by non-communicable diseases, many of which result from dietary risks. A wide range of control and prevention strategies, policies, regulations, and plans have been put in place to address the rising incidence of these disorders, including the national Medium-to-Long Term Plan for the Prevention and Treatment of Chronic Diseases (2017–2025);^34^ the Development of National Demonstration Areas for Comprehensive Prevention and Control of Non-communicable Diseases;^35^ the Healthy Life Style for All 2017–2025 Action Protocol;^36^ the Air Pollution Prevention and Control Action Plan;^37^ and salt reduction programmes.^38^ The government has also included hypertension and diabetes management in the National Basic Public Health Service Project since 2009.^39^ These national polices provide guidance on how to involve multiple departments and motivate societies to participate and work together against non-communicable diseases.

Environmental overhauls and the national transition to a greener economy might eventually reduce pollution-related cancers and chronic obstructive pulmonary disease.^40^, ^41^, ^42^ Particulate matter pollution is the fourth leading risk factor at the national level for deaths and DALYs. Tackling air pollution has become a priority for the Chinese government. The State Counsel issued a National Air Pollution Prevention and Control Action Plan in 2013, in which they identified ten detailed strategies and measures for implementation, including all related government agencies.^43^ Pollution control targets have been set for heavily polluted areas, such as the Beijing, Tianjin, and Hebei region, the Yangtze River Delta, and the Pearl River Delta to reduce concentrations of particular matter smaller than 2·5 μm in diameter by 25%, 20%, and 15%, respectively, over 5 years. Beijing in particular needs to reduce concentration to 60 μg/m^3^ per year. Although evaluation of the action plan is needed, our findings suggest that more stringent regulations should be put in place to reduce the burden caused by ambient air pollution.

Mortality rates from liver cancer was much higher than expected across provinces, but is declining.^44^ Increased coverage of hepatitis B vaccination could contribute to further declines in liver cancer.^45^ Compared with the 1992 pre-recombinant vaccine survey in China,^46^ hepatitis B prevalence had declined by 46% in 2006 and by 52% in 2014. Among children younger than 5 years, the decline was 97% over this time period.^47^ China's hepatitis B prevention programme, which was targeted toward interrupting perinatal transmission, has been highly successful and is increasingly effective.^47^ Vaccination against hepatitis B virus has been included in all childhood vaccinations since 1992, but has only been provided free of charge since 2005.^48^ The urban, coastal, and wealthier provinces and cities in eastern China generally had better health outcomes than those in the west, although health burden by cause varied substantially between provinces. Although various risks are often intensified in urban areas, such as pollution, rapid disease spread, and sedentary lifestyle behaviours,^49^ these factors seem to be offset at least partly by better access to health care and greater wealth than in rural areas. In addition, cities attract younger, working-age populations—both permanent and migrant—and past national health policies tended to focus on densely populated urban areas. The western region of China is more ethnically diverse and dispersed than the east, which increases the difficulty of providing access to care.^50^, ^51^ Of note, risks related to poor diet and sedentary lifestyles are not confined to urban areas. Providing prevention and care for rural, elderly, and migrant populations will be a major challenge as China moves toward universal health coverage.

Overall, China and its provinces have outperformed expected improvements based on SDI. Using SDI in our analysis has helped to identify areas where potential health policies and intervention programmes at the national and province levels have helped improve the population health in China. However, the marked diversity in health outcomes between provinces indicates that health equity remains a challenge. Differences between provinces remain large compared with those in some other nations. Improving health equity has long been a government priority, and Healthy China 2030 includes justice and equity as one of four core principles.^5^, ^52^ Equity at the subnational level can only be achieved if health patterns and trends can be monitored and analysed at that or even more granular levels. China's vast and growing economy puts it in the unique position to prevent and treat the disease burden if equipped with sufficient data to guide investments. Reducing inequalities will require targeted strategies to lessen major risk factors and diseases in each province and needs further and much more detailed analysis.

Our results provide important evidence for tailored, province-specific policy development and interventions. These findings can be used by governments at the subnational level to identify major health problems and facilitate priority setting. Although it was not the objective of this study to investigate the drivers behind the heterogeneity in disease burden and its trends at the provincead level in China in the past three decades, our findings help to evaluate the effectiveness of various national and provincial public health policies and intervention programmes in the same period.

This study is subject to all the limitations of the GBD studies.^10^, ^11^, ^12^, ^13^, ^14^, ^15^, ^16^ First, for the estimation of all-cause mortality in China, we used death distribution methods to evaluate the completeness of the Disease Surveillance Point system, but these methods have wide uncertainty. Second, although uncertainty was propagated in each step of our analytical process, some uncertainties might have not been fully captured in our study. Third, although we have strong evidence on fatal outcomes, we have less information on non-fatal outcomes. Fourth, comorbidity adjustment in our study assumes independent distributions of comorbid conditions, but they vary by cause, age, sex, and location.^13^ Fifth, the current DisMod-MR 2.1 tool does not capture the cohort nature of some diseases, including lung cancer. Sixth, the uncertainty of DALYs might be underestimated because we assume independence of YLLs from YLDs. Seventh, the time trends of certain diseases might be affected by changes in diagnostic technology over time. Eighth, for all-cause mortality, the censuses provide adequate data on mortality, fertility, and population for each province, but the Disease Surveillance Point system covers fewer counties and districts in remote and poorer provinces. Lack of more robust cause-specific mortality data from these provinces affects the precision of our estimates. Ninth, at the province level, we assume that in-hospital and out-of-hospital data in the Disease Surveillance Point System are representative. The accuracy of our estimates is determined by data availability, and although overall completeness of death registration and the percentage of cause-of-death data that were well certified were both over 60% in China in 2016, data sparsity in some regions remains an issue.. Tenth, we used alcohol product sales data to produce estimates of alcohol use. As alcohol consumption among Chinese residents in 1990 was mainly of homemade products, the estimated change in alcohol use between 1990 and 2017 might not be accurate.

China has been successful in improving the health of its more than 1 billion citizens, but health outcomes continue to depend on geography. Health is better overall in more industrialised, wealthier eastern provinces than in more rural, poorer western provinces. Improvements in the disease burden, particularly of communicable, maternal, neonatal, and nutritional conditions and some non-communicable diseases, are very encouraging. Nevertheless, the burden of ischaemic heart disease and other chronic diseases continues to grow and needs increased public health attention and investment. The potential effect of major environmental policy changes remains to be seen, but similar overhauls of diet and lifestyle behaviours will probably be needed to reduce leading risks for non-communicable diseases in China. The levels and trends in burden of disease differ significantly at the province level. Continued monitoring and analysis of health trends could maximise the effectiveness of investments in health and have the potential to guide other countries in the region and the world in successful approaches to evidence-based health policies. In addition, future research needs to investigate the drivers of the observed heterogeneity in the epidemiological and demographic transitions in China. Rigorous evaluations of the various public health programmes and interventions can also help guide appropriate policies in China and other developing countries.
