# Department of Error

**DOI:** 10.1016/S0140-6736(20)31450-1

**Published:** 2020-07-04

*Zhou M, Wang H, Zeng X, et al. Mortality, morbidity, and risk factors in China and its provinces, 1990–2017: a systematic analysis for the Global Burden of Disease Study 2017.* Lancet *2019; **394:** 1145–58*—The appendix of this Article has been corrected as of July 2, 2020.

