# Supplementary material for: Mortality, morbidity, and risk factors in China and its provinces, 1990–2017: a systematic analysis for the Global Burden of Disease Study 2017
Source: Lancet. 2019 Sep 28;394(10204):1145–58. doi: 10.1016/S0140-6736(19)30427-1 (PMC6891889; doi:10.1016/S0140-6736(19)30427-1)
Supplement: Supplementary appendix [file mmc1.pdf]

THE LANCET

Supplementary appendix

This appendix formed part of the original submission and has been peer reviewed. We post it as supplied by the authors.

Supplement to: Zhou M, Wang H, Zeng X, et al. Mortality, morbidity, and risk factors in China and its provinces, 1990–2017: a systematic analysis for the Global Burden of Disease Study 2017. *Lancet* 2019; published online June 24. http://dx.doi.org/10.1016/S0140-6736(19)30427-1.

Supplemental Results Appendix to Mortality, morbidity, and risk factors in China and its provinces, 1990–2017: a systematic analysis for the Global Burden of Disease Study 2017

Table of Contents

Appendix Table 1: DALY numbers and rates for China and provinces in 2017 for all causes, both sexes….3

Appendix Table 2: China Cause of Death data sources and data quality rating from 0 to 5 stars…………211

Appendix Table 3: 5q0 (per 1,000) for China and provinces from 1990 to 2017…………………………………227

Appendix Table 4: 45q15 for China and provinces from 1990 to 2017…………………………………………………228

Appendix Table 5: Province, County, Code, and Urbanicity…………………………………………………………………229

Appendix Table 6: China Non-fatal data sources…………………………………………………………………………………287

Appendix Table 7: Summary Exposure Values for the top 10 risk factors in China in 1990 and 2017, all ages, both sexes……………………………………………………………………………………………424

Appendix Table 8a: DSP 2004……425

Appendix Table 8b: DSP 2006……428

Appendix Table 8c: DSP 2013……431

Appendix Table 9: Death rate from DSP and estimated death rate, by cause, location, sex, and age……441

Appendix Table 10: China and 34 provinces SDI values and quintile groupings, both sexes, all ages, 2017…………………………………………………………………………………451

Appendix Figure 1a: DSP in 2004……………………………………………………………………………………………………452

Appendix Figure 1b: DSP in 2006……………………………………………………………………………………………………453

Appendix Figure 1c: DSP in 2013……………………………………………………………………………………………………454

Appendix Figure 2: Percentage Change in age−standardised Summary Exposure Value for the leading 10 risk factors in China and provinces, from 1990 to 2017, both sexes……..455

Appendix Figure 3: Ranking of risk factors in 2017 for China and provinces according to number of DALYs related to each risk factor……………………………………………………………456

Appendix Figure 4: China SDI values for 1990, 2000, 2007, and 2017 both sexes, all−ages…………………457

DALY numbers and rates for China and proviences in 2017 for all caues, both sexes					
		1990		2017	
Location name	Cause name	Number (in thousands)	Rate (per 100,000)	Number (in thousands)	Rate (per 100,000)
China	All causes	410000 (383000 to 441000)	34251.11 (31964.11 to 36851.26)	371000 (334000 to 414000)	26300.21 (23672.16 to 29296.18)
Taiwan	All causes	4690 (4260 to 5210)	22971.34 (20864.49 to 25527.59)	6190 (5490 to 6960)	26231.09 (23290.73 to 29503.23)
Hong Kong Special Administrative Region of China	All causes	1180 (1040 to 1320)	20432.98 (18108.74 to 22947.94)	1640 (1400 to 1930)	21848.02 (18632.38 to 25663.5)
Macau Special Administrative Region of China	All causes	70.1 (62.6 to 78.9)	19753.24 (17650.57 to 22220.19)	120 (101 to 142)	17506.34 (14805.39 to 20722.38)
Anhui	All causes	22800 (20500 to 25100)	38420.93 (34575.51 to 42297.29)	16500 (14400 to 18700)	26941.4 (23563.25 to 30618.32)
Beijing	All causes	2590 (2290 to 2940)	23133.1 (20381.97 to 26255.27)	4620 (3880 to 5440)	19187.76 (16094 to 22570.12)
Chongqing	All causes	5870 (5170 to 6580)	37207.43 (32773.4 to 41678.71)	7250 (6160 to 8510)	27139.97 (23046.31 to 31850.81)
Fujian	All causes	9120 (8340 to 9990)	28826.78 (26359.65 to 31573.65)	8730 (7530 to 10200)	22160.37 (19114.18 to 25883.43)
Gansu	All causes	12700 (11600 to 13800)	53883.09 (49237.01 to 58520.46)	7510 (6560 to 8550)	28260.76 (24683.07 to 32152)
Guangdong	All causes	18700 (16800 to 20800)	28435.73 (25583.4 to 31628.08)	24200 (20700 to 28200)	20673.62 (17713.16 to 24147.97)
Guangxi	All causes	14600 (13200 to 16100)	33630.63 (30325.46 to 37092.04)	13200 (11500 to 15100)	27374.58 (23875.6 to 31343.07)
Guizhou	All causes	16300 (14600 to 18200)	49043.49 (43889.53 to 54968.34)	9770 (8450 to 11100)	28882.29 (24969.77 to 32865.05)
Hainan	All causes	2330 (2070 to 2600)	33399.35 (29748.07 to 37298.44)	2190 (1870 to 2530)	22804.98 (19482.11 to 26378.85)
Hebei	All causes	18500 (16800 to 20300)	29018.89 (26333.01 to 31904.64)	22500 (19600 to 25500)	29439.9 (25687.42 to 33388.1)
Heilongjiang	All causes	10200 (9230 to 11300)	27886.66 (25333.87 to 30932.55)	11900 (10300 to 13700)	30370.82 (26267.1 to 34839.93)
Henan	All causes	27800 (25200 to 30500)	31517.55 (28564.68 to 34536.25)	25600 (22300 to 29200)	26565.67 (23172.84 to 30270.75)
Hubei	All causes	17700 (16000 to 19400)	31865.88 (28830.52 to 34955.63)	16400 (14000 to 18700)	29624.55 (25375.54 to 33881.09)
Hunan	All causes	21900 (20000 to 23900)	35225.41 (32241.39 to 38460.76)	20000 (17300 to 22600)	29806.88 (25837.43 to 33721.69)
Inner Mongolia	All causes	7930 (7150 to 8750)	34879.21 (31437.51 to 38477.09)	6950 (6040 to 7950)	26845.86 (23334.53 to 30691.87)
Jiangsu	All causes	18900 (17100 to 21000)	27516.8 (24854.46 to 30458.9)	20700 (18100 to 23600)	25301.37 (22111.41 to 28956.54)
Jiangxi	All causes	19400 (17500 to 21700)	49187.49 (44163.87 to 54774.63)	11900 (10400 to 13600)	24956.15 (21839.09 to 28522.92)
Jilin	All causes	7510 (6840 to 8220)	28958.89 (26372.58 to 31692.62)	7420 (6330 to 8570)	26620.81 (22689.4 to 30738.84)
Liaoning	All causes	10500 (9400 to 11800)	25852.55 (23042.11 to 28823.37)	13500 (11700 to 15500)	30180.31 (26204.17 to 34811.23)
Ningxia	All causes	2210 (1900 to 2580)	44828.91 (38463.45 to 52251.31)	1630 (1400 to 1860)	23197.54 (19973.12 to 26445.39)
Qinghai	All causes	2430 (2110 to 2780)	51266.32 (44611.16 to 58615.22)	1750 (1540 to 2010)	27890.04 (24509.77 to 31916.63)
Shaanxi	All causes	10300 (9210 to 11400)	29789.25 (26771.38 to 33035.48)	10200 (8750 to 11800)	25786.99 (22149.01 to 29901.51)
Shandong	All causes	25200 (22700 to 27800)	29417.19 (26491.5 to 32484.99)	26200 (22700 to 30100)	26395.5 (22798.77 to 30266.47)
Shanghai	All causes	3380 (2980 to 3820)	24215.25 (21364.44 to 27313.3)	5510 (4650 to 6510)	19706.17 (16641.94 to 23313.95)
Shanxi	All causes	9990 (9050 to 11100)	33003.31 (29899.89 to 36722.78)	9220 (7890 to 10700)	23871.49 (20420.29 to 27656.6)
Sichuan	All causes	43300 (38500 to 48700)	38821.84 (34485.35 to 43654.95)	25500 (22100 to 29100)	30565.22 (26435.48 to 34870.19)
Tianjin	All causes	2400 (2140 to 2670)	26043.16 (23241.75 to 29055.71)	3650 (3130 to 4210)	23933.38 (20481.43 to 27554.07)
Tibet	All causes	1630 (1460 to 1800)	69827.85 (62726.25 to 77396.79)	1060 (938 to 1190)	30673.64 (27145.73 to 34516.71)

Xinjiang	All causes	7450<br>(6690 to 8280)	47251.69<br>(42441.58 to 52470.09)	6960<br>(6090 to 7860)	28544.67<br>(25004.55 to 32258.19)
Yunnan	All causes	21200<br>(18800 to 23900)	54472.32<br>(48192.12 to 61293.03)	14000<br>(12300 to 15900)	28572.24<br>(25042.17 to 32380.41)
Zhejiang	All causes	13800<br>(12500 to 15300)	31221.47<br>(28434.94 to 34632.72)	13200<br>(11300 to 15400)	21652.2<br>(18475.07 to 25124.31)
China	Communicable, maternal, neonatal, and nutritional diseases	114000<br>(108000 to 121000)	9531.15<br>(8986.93 to 10145.07)	27300<br>(23800 to 31000)	1930.13<br>(1683.12 to 2196.13)
Taiwan	Communicable, maternal, neonatal, and nutritional diseases	357<br>(319 to 404)	1751.02<br>(1565.09 to 1979.95)	380<br>(344 to 424)	1609.4<br>(1457.51 to 1798.97)
Hong Kong Special Administrative Region of China	Communicable, maternal, neonatal, and nutritional diseases	111<br>(88 to 129)	1924.74<br>(1527.09 to 2237.97)	99.8<br>(70.1 to 122)	1328.66<br>(933.46 to 1621.83)
Macau Special Administrative Region of China	Communicable, maternal, neonatal, and nutritional diseases	9.95<br>(8.73 to 11.2)	2803.97<br>(2460.86 to 3162.61)	7.66<br>(6.18 to 9.19)	1120.05<br>(903.24 to 1343.44)
Anhui	Communicable, maternal, neonatal, and nutritional diseases	6890<br>(5850 to 8000)	11627.68<br>(9865.11 to 13508.68)	1180<br>(964 to 1460)	1924.71<br>(1576.35 to 2390.14)
Beijing	Communicable, maternal, neonatal, and nutritional diseases	296<br>(257 to 340)	2636.85<br>(2288.28 to 3027.53)	299<br>(251 to 353)	1240.28<br>(1040.82 to 1465.61)
Chongqing	Communicable, maternal, neonatal, and nutritional diseases	1550<br>(1210 to 1940)	9825.8<br>(7647.4 to 12312.06)	459<br>(391 to 538)	1715.7<br>(1464.28 to 2013.12)
Fujian	Communicable, maternal, neonatal, and nutritional diseases	2400<br>(2090 to 2730)	7579.22<br>(6619.9 to 8621.63)	609<br>(515 to 717)	1544.44<br>(1305.54 to 1819.46)
Gansu	Communicable, maternal, neonatal, and nutritional diseases	5010<br>(4350 to 5670)	21162.53<br>(18411.92 to 23991.62)	644<br>(542 to 748)	2422.06<br>(2039.67 to 2814.48)
Guangdong	Communicable, maternal, neonatal, and nutritional diseases	4430<br>(3930 to 5000)	6736.82<br>(5964.5 to 7600.49)	2250<br>(1900 to 2670)	1929.69<br>(1630.4 to 2285.86)
Guangxi	Communicable, maternal, neonatal, and nutritional diseases	4120<br>(3560 to 4720)	9469.33<br>(8189.78 to 10861.31)	1290<br>(1130 to 1470)	2673.67<br>(2347.88 to 3038.94)
Guizhou	Communicable, maternal, neonatal, and nutritional diseases	6780<br>(5660 to 8040)	20438.02<br>(17063.41 to 24226.59)	839<br>(721 to 959)	2479.99<br>(2130.04 to 2833.88)
Hainan	Communicable, maternal, neonatal, and nutritional diseases	766<br>(643 to 907)	10999.67<br>(9231.74 to 13021.25)	262<br>(225 to 302)	2728.48<br>(2346.06 to 3147.93)
Hebei	Communicable, maternal, neonatal, and nutritional diseases	4040<br>(3520 to 4590)	6354.51<br>(5530.91 to 7211.52)	1910<br>(1550 to 2350)	2497.77<br>(2024.13 to 3080.79)
Heilongjiang	Communicable, maternal, neonatal, and nutritional diseases	1710<br>(1470 to 1990)	4687.92<br>(4039.17 to 5465.87)	553<br>(464 to 657)	1409.86<br>(1181.84 to 1674.59)
Henan	Communicable, maternal, neonatal, and nutritional diseases	7370<br>(6340 to 8570)	8343.74<br>(7177.75 to 9709.6)	1520<br>(1280 to 1820)	1582.32<br>(1329.43 to 1894.59)
Hubei	Communicable, maternal, neonatal, and nutritional diseases	4260<br>(3640 to 4930)	7666.12<br>(6553.62 to 8875.77)	968<br>(744 to 1270)	1749.62<br>(1344.55 to 2303.77)
Hunan	Communicable, maternal, neonatal, and nutritional diseases	4980<br>(4280 to 5750)	8009.11<br>(6890.72 to 9253.88)	1110<br>(948 to 1280)	1649.67<br>(1411.57 to 1913.08)
Inner Mongolia	Communicable, maternal, neonatal, and nutritional diseases	1960<br>(1700 to 2250)	8624.88<br>(7483.18 to 9912.85)	401<br>(339 to 471)	1548.95<br>(1309.37 to 1818.04)
Jiangsu	Communicable, maternal, neonatal, and nutritional diseases	3110<br>(2680 to 3620)	4524.92<br>(3898.48 to 5260.93)	1010<br>(839 to 1220)	1239.29<br>(1027.08 to 1493.09)
Jiangxi	Communicable, maternal, neonatal, and nutritional diseases	6820<br>(5560 to 8160)	17249.31<br>(14069.96 to 20648.7)	1110<br>(952 to 1290)	2336.31<br>(2002.02 to 2707.33)

Jilin	Communicable, maternal, neonatal, and nutritional diseases	1580 (1400 to 1800)	6087.84 (5378.65 to 6928.34)	414 (355 to 478)	1483.21 (1273.39 to 1713.99)
Liaoning	Communicable, maternal, neonatal, and nutritional diseases	1340 (1170 to 1550)	3286.96 (2860.98 to 3807.71)	558 (470 to 657)	1249.47 (1053.41 to 1472.11)
Ningxia	Communicable, maternal, neonatal, and nutritional diseases	871 (682 to 1090)	17645.59 (13808.79 to 22145.97)	155 (130 to 182)	2203.73 (1854.13 to 2597.02)
Qinghai	Communicable, maternal, neonatal, and nutritional diseases	1070 (857 to 1330)	22676.61 (18101.44 to 28072.87)	180 (151 to 215)	2862 (2411.32 to 3416.83)
Shaanxi	Communicable, maternal, neonatal, and nutritional diseases	3940 (3410 to 4500)	11446.56 (9913.92 to 13073.56)	988 (771 to 1250)	2500.54 (1951.69 to 3156.83)
Shandong	Communicable, maternal, neonatal, and nutritional diseases	3440 (2950 to 4050)	4017.63 (3445.84 to 4732.39)	1200 (981 to 1460)	1207.92 (987.07 to 1468.22)
Shanghai	Communicable, maternal, neonatal, and nutritional diseases	404 (343 to 477)	2889.45 (2457.43 to 3412.9)	356 (292 to 431)	1273.45 (1044.76 to 1541.46)
Shanxi	Communicable, maternal, neonatal, and nutritional diseases	2520 (2220 to 2880)	8318.67 (7322.1 to 9527.04)	706 (586 to 847)	1827.95 (1517.91 to 2193.02)
Sichuan	Communicable, maternal, neonatal, and nutritional diseases	15400 (12400 to 18900)	13828.25 (11120.84 to 16891.11)	2010 (1690 to 2400)	2410.14 (2024.43 to 2879.65)
Tianjin	Communicable, maternal, neonatal, and nutritional diseases	307 (266 to 349)	3332.66 (2889.38 to 3792.57)	205 (172 to 241)	1345.83 (1126.98 to 1576.44)
Tibet	Communicable, maternal, neonatal, and nutritional diseases	770 (665 to 898)	33057.83 (28528.9 to 38543.03)	223 (187 to 264)	6449.35 (5402.24 to 7651.02)
Xinjiang	Communicable, maternal, neonatal, and nutritional diseases	3690 (3130 to 4310)	23386.87 (19874.03 to 27332.57)	1120 (964 to 1310)	4603.01 (3956.3 to 5363.79)
Yunnan	Communicable, maternal, neonatal, and nutritional diseases	9170 (7420 to 11000)	23534.58 (19049.48 to 28249.21)	1640 (1420 to 1880)	3327.55 (2893 to 3826.31)
Zhejiang	Communicable, maternal, neonatal, and nutritional diseases	2930 (2560 to 3400)	6653.23 (5800.68 to 7720.57)	982 (786 to 1240)	1604.47 (1285.34 to 2028.93)
China	Tuberculosis	6980 (6580 to 7390)	582.87 (549.94 to 617.57)	1720 (1490 to 1960)	121.6 (105.27 to 138.82)
Taiwan	Tuberculosis	57.4 (54.1 to 60.9)	281.44 (265.25 to 298.56)	19.5 (17 to 22.3)	82.59 (71.94 to 94.58)
Hong Kong Special Administrative Region of China	Tuberculosis	12.7 (11.4 to 14.1)	219.92 (197.85 to 245.2)	5.78 (4.82 to 6.82)	76.93 (64.25 to 90.77)
Macau Special Administrative Region of China	Tuberculosis	1.02 (0.917 to 1.11)	287.06 (258.4 to 314.04)	0.503 (0.419 to 0.597)	73.49 (61.3 to 87.25)
Anhui	Tuberculosis	298 (260 to 339)	502.98 (439.08 to 571.88)	59 (49.3 to 70.2)	96.54 (80.57 to 114.84)
Beijing	Tuberculosis	24.1 (20.9 to 27.7)	214.65 (186.61 to 247.11)	9.24 (7.53 to 11.3)	38.34 (31.24 to 46.9)
Chongqing	Tuberculosis	95.1 (81.7 to 110)	602.43 (517.48 to 698.02)	31.3 (25.2 to 38.3)	117.15 (94.41 to 143.21)
Fujian	Tuberculosis	129 (115 to 144)	406.53 (364.15 to 456.04)	34.5 (28.5 to 41.6)	87.59 (72.27 to 105.53)
Gansu	Tuberculosis	196 (172 to 223)	827.07 (725.43 to 941.97)	31.9 (27.1 to 37.5)	120.04 (101.86 to 141.05)
Guangdong	Tuberculosis	395 (349 to 451)	600.35 (530.55 to 685.24)	129 (106 to 156)	110.32 (90.49 to 133.84)
Guangxi	Tuberculosis	463 (406 to 529)	1064.03 (932.74 to 1216.87)	118 (98.8 to 139)	243.63 (204.45 to 286.68)
Guizhou	Tuberculosis	543 (468 to 630)	1635.65 (1410.64 to 1897.45)	92.2 (77.1 to 108)	272.42 (227.97 to 317.85)
Hainan	Tuberculosis	80.5 (70 to 93.1)	1155.44 (1005.12 to 1336.99)	19.5 (16.1 to 23.4)	203.8 (167.91 to 243.9)

Hebei	Tuberculosis	129 (113 to 146)	203.35 (178.33 to 230.18)	45.6 (37 to 55.1)	59.69 (48.38 to 72.14)
Heilongjiang	Tuberculosis	193 (170 to 216)	530.05 (467.23 to 591.98)	54.3 (45.1 to 64.3)	138.28 (114.91 to 163.85)
Henan	Tuberculosis	244 (213 to 278)	276.02 (241.86 to 315.25)	66.4 (53.7 to 80.6)	68.92 (55.72 to 83.66)
Hubei	Tuberculosis	284 (252 to 315)	511.17 (454.27 to 566.52)	76.3 (63.1 to 90)	138.05 (114.15 to 162.81)
Hunan	Tuberculosis	444 (396 to 493)	713.75 (637.3 to 792.64)	106 (89 to 124)	158.13 (132.55 to 185.15)
Inner Mongolia	Tuberculosis	120 (105 to 136)	527.93 (463.7 to 599.5)	32.1 (25.8 to 39.4)	123.95 (99.48 to 152.01)
Jiangsu	Tuberculosis	196 (174 to 219)	284.46 (253.48 to 318.3)	59.3 (48.3 to 72.7)	72.66 (59.14 to 88.99)
Jiangxi	Tuberculosis	441 (390 to 499)	1114.4 (985.53 to 1261.47)	81.3 (65.9 to 98.5)	171.11 (138.61 to 207.1)
Jilin	Tuberculosis	189 (163 to 210)	726.61 (629.57 to 808.54)	43.6 (35.8 to 51.7)	156.15 (128.48 to 185.29)
Liaoning	Tuberculosis	192 (164 to 222)	470.29 (402.03 to 544.52)	62.1 (51.6 to 73.7)	139.14 (115.52 to 165)
Ningxia	Tuberculosis	31.3 (25.7 to 38.2)	632.99 (520.76 to 773.44)	7.93 (6.26 to 9.9)	112.96 (89.21 to 141.05)
Qinghai	Tuberculosis	47.7 (40.3 to 56.1)	1006.15 (850.67 to 1185.09)	10.9 (9.19 to 12.8)	173.73 (146.28 to 204.4)
Shaanxi	Tuberculosis	84.3 (68.5 to 102)	245.02 (198.98 to 295.82)	25.8 (21.5 to 30.7)	65.38 (54.48 to 77.64)
Shandong	Tuberculosis	179 (156 to 204)	209.38 (182.08 to 238.84)	49.5 (39.5 to 60.6)	49.82 (39.71 to 60.95)
Shanghai	Tuberculosis	33.2 (29 to 38.1)	237.73 (207.46 to 272.57)	11.9 (9.68 to 14.4)	42.57 (34.66 to 51.58)
Shanxi	Tuberculosis	92.9 (79.9 to 105)	306.87 (264.01 to 347.61)	25.6 (20.4 to 31.8)	66.4 (52.92 to 82.35)
Sichuan	Tuberculosis	588 (495 to 696)	526.89 (443.79 to 624.06)	104 (84.8 to 126)	124.77 (101.6 to 151.39)
Tianjin	Tuberculosis	19.5 (16.9 to 22.1)	211.59 (183.23 to 240.59)	6.6 (5.36 to 7.97)	43.27 (35.14 to 52.24)
Tibet	Tuberculosis	124 (110 to 141)	5322.22 (4728.01 to 6059.72)	27.8 (23.8 to 32.1)	804.02 (688.59 to 929.3)
Xinjiang	Tuberculosis	452 (403 to 499)	2865.45 (2553.5 to 3165.14)	149 (125 to 177)	611.86 (512.55 to 727.28)
Yunnan	Tuberculosis	502 (433 to 583)	1287.45 (1110.44 to 1495.11)	102 (85.7 to 118)	206.89 (174.24 to 240.89)
Zhejiang	Tuberculosis	156 (139 to 175)	353.27 (315.47 to 397.1)	38.6 (31 to 47.1)	63.16 (50.64 to 76.99)
China	HIV/AIDS	195 (154 to 230)	16.32 (12.9 to 19.18)	1520 (1410 to 1650)	107.39 (99.62 to 116.56)
Taiwan	HIV/AIDS	1.58 (1.46 to 1.71)	7.74 (7.14 to 8.4)	9.64 (8.68 to 10.8)	40.89 (36.81 to 45.88)
Hong Kong Special Administrative Region of China	HIV/AIDS	0.563 (0.51 to 0.62)	9.77 (8.85 to 10.75)	0.828 (0.745 to 0.93)	11.03 (9.92 to 12.38)
Macau Special Administrative Region of China	HIV/AIDS	0.00652 (0.00157 to 0.0124)	1.84 (0.44 to 3.48)	0.0365 (0.0289 to 0.0429)	5.33 (4.22 to 6.27)
Anhui	HIV/AIDS	2.11 (0.693 to 2.91)	3.57 (1.17 to 4.91)	18.3 (15.4 to 20.9)	29.95 (25.22 to 34.26)
Beijing	HIV/AIDS	0.313 (0.0801 to 0.469)	2.79 (0.71 to 4.18)	4.25 (3.49 to 4.91)	17.62 (14.48 to 20.39)
Chongqing	HIV/AIDS	23.9 (21.9 to 26)	151.48 (138.77 to 164.71)	60.5 (56.8 to 65.6)	226.28 (212.54 to 245.54)
Fujian	HIV/AIDS	1.87 (0.585 to 2.54)	5.9 (1.85 to 8.02)	14 (11.5 to 16)	35.45 (29.26 to 40.59)
Gansu	HIV/AIDS	2.2 (2 to 2.43)	9.32 (8.46 to 10.28)	4.26 (3.9 to 4.67)	16.05 (14.69 to 17.59)
Guangdong	HIV/AIDS	8.75 (2.23 to 12.5)	13.29 (3.39 to 19.02)	101 (84.2 to 117)	86.52 (72.05 to 100.06)
Guangxi	HIV/AIDS	29.5 (7.33 to 42.2)	67.75 (16.85 to 97.03)	281 (229 to 328)	582.24 (474.31 to 678.39)

Guizhou	HIV/AIDS	12.8 (3.86 to 17.6)	38.49 (11.64 to 52.98)	74.4 (61.3 to 85.4)	219.99 (181.22 to 252.53)
Hainan	HIV/AIDS	0.767 (0.228 to 1.06)	11.01 (3.27 to 15.24)	6.08 (4.97 to 7.04)	63.38 (51.84 to 73.46)
Hebei	HIV/AIDS	0.845 (0.222 to 1.19)	1.33 (0.35 to 1.87)	10 (8.37 to 11.5)	13.11 (10.95 to 15.07)
Heilongjiang	HIV/AIDS	0.966 (0.296 to 1.37)	2.65 (0.81 to 3.76)	8.28 (6.89 to 9.43)	21.1 (17.55 to 24.03)
Henan	HIV/AIDS	12.6 (3.46 to 17.6)	14.24 (3.92 to 19.9)	87.2 (70.7 to 101)	90.51 (73.41 to 104.99)
Hubei	HIV/AIDS	3.29 (0.956 to 4.57)	5.92 (1.72 to 8.22)	26.5 (22.3 to 30.5)	47.85 (40.28 to 55.14)
Hunan	HIV/AIDS	11.1 (3.01 to 15.3)	17.79 (4.84 to 24.63)	77.1 (63.6 to 88.8)	114.83 (94.7 to 132.22)
Inner Mongolia	HIV/AIDS	0.532 (0.185 to 0.717)	2.34 (0.81 to 3.15)	3.16 (2.64 to 3.62)	12.19 (10.19 to 13.97)
Jiangsu	HIV/AIDS	2.81 (0.743 to 4.08)	4.08 (1.08 to 5.92)	21.3 (17.8 to 24.6)	26.14 (21.78 to 30.09)
Jiangxi	HIV/AIDS	4.61 (1.34 to 6.34)	11.67 (3.39 to 16.04)	29.1 (23.4 to 33.6)	61.13 (49.32 to 70.66)
Jilin	HIV/AIDS	1.35 (0.418 to 1.88)	5.19 (1.61 to 7.26)	10 (8.39 to 11.4)	35.96 (30.08 to 40.99)
Liaoning	HIV/AIDS	1.56 (0.418 to 2.31)	3.83 (1.02 to 5.66)	14.9 (12.6 to 17.1)	33.47 (28.25 to 38.36)
Ningxia	HIV/AIDS	0.191 (0.0599 to 0.26)	3.86 (1.21 to 5.26)	2.15 (1.74 to 2.51)	30.6 (24.82 to 35.74)
Qinghai	HIV/AIDS	0.339 (0.114 to 0.459)	7.16 (2.4 to 9.7)	3.09 (2.54 to 3.57)	49.13 (40.46 to 56.75)
Shaanxi	HIV/AIDS	0.776 (0.223 to 1.1)	2.25 (0.65 to 3.18)	11.3 (9.4 to 12.9)	28.6 (23.8 to 32.56)
Shandong	HIV/AIDS	1.64 (0.447 to 2.31)	1.91 (0.52 to 2.7)	10.2 (8.43 to 11.7)	10.23 (8.48 to 11.75)
Shanghai	HIV/AIDS	0.476 (0.0957 to 0.723)	3.41 (0.69 to 5.18)	5.83 (4.81 to 6.76)	20.87 (17.2 to 24.21)
Shanxi	HIV/AIDS	1.52 (0.477 to 2.1)	5.01 (1.58 to 6.92)	11.2 (9.39 to 12.9)	29.09 (24.32 to 33.39)
Sichuan	HIV/AIDS	36 (9.36 to 52.5)	32.23 (8.38 to 47.01)	289 (239 to 334)	346.18 (286.14 to 400.61)
Tianjin	HIV/AIDS	0.258 (0.0672 to 0.375)	2.8 (0.73 to 4.07)	3.5 (2.92 to 4.06)	22.95 (19.13 to 26.59)
Tibet	HIV/AIDS	0.182 (0.0692 to 0.242)	7.79 (2.97 to 10.38)	1.21 (1.01 to 1.38)	34.89 (29.11 to 39.88)
Xinjiang	HIV/AIDS	5.46 (1.69 to 7.47)	34.63 (10.74 to 47.38)	88.7 (73.3 to 102)	364.21 (300.97 to 417.13)
Yunnan	HIV/AIDS	19.5 (5.4 to 26.9)	50.06 (13.86 to 69)	224 (186 to 259)	455.25 (378.71 to 525.96)
Zhejiang	HIV/AIDS	6.7 (6.17 to 7.26)	15.2 (13.99 to 16.46)	14.2 (13 to 15.7)	23.2 (21.18 to 25.69)
China	Diarrheal diseases	8570 (7480 to 9930)	715.88 (625.19 to 829.43)	979 (754 to 1250)	69.3 (53.4 to 88.25)
Taiwan	Diarrheal diseases	15.8 (12.2 to 20.2)	77.27 (59.96 to 99.2)	24.4 (17.6 to 32.5)	103.65 (74.5 to 137.95)
Hong Kong Special Administrative Region of China	Diarrheal diseases	3.71 (2.73 to 4.9)	64.29 (47.41 to 84.96)	3.69 (2.66 to 4.96)	49.15 (35.47 to 66.03)
Macau Special Administrative Region of China	Diarrheal diseases	0.494 (0.369 to 0.624)	139.28 (103.94 to 175.86)	0.35 (0.251 to 0.466)	51.19 (36.67 to 68.13)
Anhui	Diarrheal diseases	440 (314 to 627)	741.93 (529.49 to 1057.43)	35.6 (25.9 to 46.6)	58.16 (42.28 to 76.17)
Beijing	Diarrheal diseases	16.1 (12.2 to 20.6)	143.77 (108.79 to 183.47)	21.4 (15.1 to 28.9)	88.68 (62.53 to 119.83)
Chongqing	Diarrheal diseases	131 (87.1 to 188)	830.06 (551.69 to 1193.98)	22.4 (16.4 to 29.8)	83.86 (61.28 to 111.66)
Fujian	Diarrheal diseases	158 (116 to 208)	498.61 (367.57 to 657.78)	17.5 (13.3 to 22.9)	44.39 (33.66 to 58.2)
Gansu	Diarrheal diseases	568 (415 to 779)	2402.2 (1754.29 to 3292.32)	21.4 (16.6 to 26.9)	80.56 (62.51 to 101.34)

Guangdong	Diarrheal diseases	252<br>(188 to 335)	383.51<br>(285.39 to 508.48)	63.5<br>(47.7 to 83.4)	54.32<br>(40.81 to 71.34)
Guangxi	Diarrheal diseases	280<br>(203 to 374)	644.21<br>(466.56 to 858.89)	32.3<br>(24.5 to 41)	66.87<br>(50.62 to 84.91)
Guizhou	Diarrheal diseases	385<br>(270 to 524)	1160.6<br>(812.31 to 1577.44)	34.5<br>(26.8 to 43.8)	101.96<br>(79.12 to 129.46)
Hainan	Diarrheal diseases	63.7<br>(44.2 to 91.1)	913.92<br>(634.6 to 1308.04)	10.3<br>(7.91 to 12.8)	106.93<br>(82.52 to 133.63)
Hebei	Diarrheal diseases	245<br>(179 to 323)	384.49<br>(281.39 to 508.27)	44.5<br>(34 to 56.2)	58.18<br>(44.52 to 73.5)
Heilongjiang	Diarrheal diseases	51.8<br>(39.1 to 68.3)	142.03<br>(107.27 to 187.48)	20.3<br>(14.1 to 27.8)	51.65<br>(35.99 to 70.85)
Henan	Diarrheal diseases	616<br>(447 to 845)	698.32<br>(506.37 to 957.83)	65.4<br>(47.6 to 86.6)	67.89<br>(49.43 to 89.9)
Hubei	Diarrheal diseases	232<br>(164 to 316)	416.91<br>(295.64 to 569.38)	36.4<br>(26 to 48.6)	65.83<br>(46.92 to 87.87)
Hunan	Diarrheal diseases	348<br>(244 to 460)	559.7<br>(392.48 to 739.23)	45.5<br>(33.9 to 59.7)	67.74<br>(50.44 to 88.89)
Inner Mongolia	Diarrheal diseases	135<br>(97.2 to 182)	592.13<br>(427.22 to 800.76)	11.1<br>(8.33 to 14.3)	42.75<br>(32.17 to 55.3)
Jiangsu	Diarrheal diseases	144<br>(109 to 191)	208.79<br>(158.38 to 276.86)	44.8<br>(32.2 to 60.2)	54.9<br>(39.39 to 73.78)
Jiangxi	Diarrheal diseases	584<br>(408 to 804)	1478.38<br>(1033.1 to 2032.61)	36.3<br>(28 to 46.1)	76.36<br>(58.82 to 97.02)
Jilin	Diarrheal diseases	48.9<br>(35.8 to 66.3)	188.4<br>(137.77 to 255.63)	10.9<br>(8.03 to 14.4)	39.25<br>(28.8 to 51.61)
Liaoning	Diarrheal diseases	54.5<br>(40.9 to 71.3)	133.5<br>(100.19 to 174.69)	26<br>(18.1 to 35.6)	58.31<br>(40.48 to 79.72)
Ningxia	Diarrheal diseases	65.1<br>(44 to 92.6)	1317.46<br>(890.1 to 1875.5)	4.15<br>(3.05 to 5.33)	59.14<br>(43.47 to 75.96)
Qinghai	Diarrheal diseases	142<br>(98.2 to 201)	2994.99<br>(2074.18 to 4239.6)	6.55<br>(5.09 to 8.38)	104.28<br>(81.01 to 133.38)
Shaanxi	Diarrheal diseases	406<br>(299 to 541)	1178.69<br>(869.02 to 1570.77)	25.1<br>(19.2 to 32)	63.59<br>(48.64 to 81.08)
Shandong	Diarrheal diseases	185<br>(142 to 240)	215.71<br>(165.31 to 279.87)	56<br>(39.7 to 76.3)	56.36<br>(39.93 to 76.8)
Shanghai	Diarrheal diseases	11.7<br>(8.85 to 14.9)	83.82<br>(63.32 to 106.79)	9.62<br>(6.96 to 12.7)	34.43<br>(24.91 to 45.6)
Shanxi	Diarrheal diseases	138<br>(98.8 to 186)	454.44<br>(326.11 to 612.6)	17.5<br>(13.1 to 22.8)	45.22<br>(33.93 to 59.14)
Sichuan	Diarrheal diseases	1030<br>(741 to 1450)	924.32<br>(663.82 to 1302.59)	55.2<br>(40.6 to 72.5)	66.07<br>(48.68 to 86.86)
Tianjin	Diarrheal diseases	7.8<br>(6.06 to 9.91)	84.84<br>(65.84 to 107.76)	5.07<br>(3.59 to 6.82)	33.21<br>(23.55 to 44.67)
Tibet	Diarrheal diseases	120<br>(89.5 to 162)	5132.94<br>(3838.85 to 6964.61)	18.7<br>(14.1 to 25.2)	542.29<br>(407.01 to 727.79)
Xinjiang	Diarrheal diseases	467<br>(335 to 613)	2958.35<br>(2126.75 to 3884.5)	51.4<br>(37.6 to 69.6)	210.96<br>(154.5 to 285.51)
Yunnan	Diarrheal diseases	1110<br>(805 to 1460)	2845.78<br>(2066.74 to 3747.35)	81.9<br>(63.7 to 104)	166.51<br>(129.48 to 211.61)
Zhejiang	Diarrheal diseases	131<br>(91.3 to 179)	296.62<br>(206.99 to 406.3)	43.6<br>(31.1 to 58.7)	71.33<br>(50.83 to 95.94)
China	Other intestinal infectious diseases	33.8<br>(26.9 to 41)	2.83<br>(2.25 to 3.42)	2.95<br>(1.57 to 4.66)	0.21<br>(0.11 to 0.33)
Taiwan	Other intestinal infectious diseases	0.0642<br>(0.0283 to 0.113)	0.31<br>(0.14 to 0.55)	0.0207<br>(0.0115 to 0.0311)	0.09<br>(0.05 to 0.13)
Hong Kong Special Administrative Region of China	Other intestinal infectious diseases	0.00868<br>(0.00417 to 0.0151)	0.15<br>(0.07 to 0.26)	0.00438<br>(0.0024 to 0.00647)	0.06<br>(0.03 to 0.09)
Macau Special Administrative Region of China	Other intestinal infectious diseases	0.00064<br>(0.000293 to 0.00116)	0.18<br>(0.08 to 0.33)	0.000272<br>(0.000153 to 0.000404)	0.04<br>(0.02 to 0.06)
Anhui	Other intestinal infectious diseases	2.66<br>(1.5 to 4.64)	4.48<br>(2.54 to 7.84)	0.149<br>(0.069 to 0.258)	0.24<br>(0.11 to 0.42)
Beijing	Other intestinal infectious diseases	0.0447<br>(0.0172 to 0.0821)	0.4<br>(0.15 to 0.73)	0.0159<br>(0.00651 to 0.03)	0.07<br>(0.03 to 0.12)
Chongqing	Other intestinal infectious diseases	0.515<br>(0.235 to 0.934)	3.26<br>(1.49 to 5.92)	0.0483<br>(0.0225 to 0.0829)	0.18<br>(0.08 to 0.31)

Fujian	Other intestinal infectious diseases	0.607<br>(0.333 to 0.963)	1.92<br>(1.05 to 3.04)	0.0594<br>(0.0275 to 0.102)	0.15<br>(0.07 to 0.26)
Gansu	Other intestinal infectious diseases	1.89<br>(0.987 to 3.3)	8.01<br>(4.17 to 13.97)	0.107<br>(0.038 to 0.209)	0.4<br>(0.14 to 0.79)
Guangdong	Other intestinal infectious diseases	0.823<br>(0.485 to 1.27)	1.25<br>(0.74 to 1.93)	0.159<br>(0.0753 to 0.266)	0.14<br>(0.06 to 0.23)
Guangxi	Other intestinal infectious diseases	0.925<br>(0.547 to 1.49)	2.13<br>(1.26 to 3.42)	0.114<br>(0.0589 to 0.178)	0.24<br>(0.12 to 0.37)
Guizhou	Other intestinal infectious diseases	2.03<br>(1.18 to 3.37)	6.1<br>(3.55 to 10.16)	0.0963<br>(0.0521 to 0.152)	0.28<br>(0.15 to 0.45)
Hainan	Other intestinal infectious diseases	0.158<br>(0.047 to 0.304)	2.27<br>(0.67 to 4.36)	0.0294<br>(0.00768 to 0.0626)	0.31<br>(0.08 to 0.65)
Hebei	Other intestinal infectious diseases	1.19<br>(0.693 to 1.92)	1.88<br>(1.09 to 3.02)	0.217<br>(0.104 to 0.363)	0.28<br>(0.14 to 0.48)
Heilongjiang	Other intestinal infectious diseases	0.45<br>(0.224 to 0.715)	1.24<br>(0.62 to 1.96)	0.0656<br>(0.0332 to 0.104)	0.17<br>(0.08 to 0.26)
Henan	Other intestinal infectious diseases	2.66<br>(1.47 to 4.54)	3.02<br>(1.67 to 5.14)	0.188<br>(0.0948 to 0.305)	0.2<br>(0.1 to 0.32)
Hubei	Other intestinal infectious diseases	1.3<br>(0.72 to 2.24)	2.34<br>(1.3 to 4.02)	0.0947<br>(0.0497 to 0.154)	0.17<br>(0.09 to 0.28)
Hunan	Other intestinal infectious diseases	1.29<br>(0.791 to 2.09)	2.07<br>(1.27 to 3.37)	0.126<br>(0.0726 to 0.183)	0.19<br>(0.11 to 0.27)
Inner Mongolia	Other intestinal infectious diseases	0.712<br>(0.361 to 1.23)	3.13<br>(1.59 to 5.41)	0.0596<br>(0.0239 to 0.112)	0.23<br>(0.09 to 0.43)
Jiangsu	Other intestinal infectious diseases	0.763<br>(0.423 to 1.22)	1.11<br>(0.61 to 1.77)	0.0788<br>(0.0453 to 0.118)	0.1<br>(0.06 to 0.14)
Jiangxi	Other intestinal infectious diseases	2.12<br>(1.2 to 3.55)	5.37<br>(3.04 to 8.99)	0.159<br>(0.0627 to 0.284)	0.33<br>(0.13 to 0.6)
Jilin	Other intestinal infectious diseases	0.386<br>(0.178 to 0.633)	1.49<br>(0.69 to 2.44)	0.0472<br>(0.023 to 0.0781)	0.17<br>(0.08 to 0.28)
Liaoning	Other intestinal infectious diseases	0.288<br>(0.138 to 0.471)	0.71<br>(0.34 to 1.16)	0.0547<br>(0.0308 to 0.0832)	0.12<br>(0.07 to 0.19)
Ningxia	Other intestinal infectious diseases	0.348<br>(0.123 to 0.638)	7.05<br>(2.49 to 12.92)	0.0243<br>(0.00719 to 0.0503)	0.35<br>(0.1 to 0.72)
Qinghai	Other intestinal infectious diseases	0.343<br>(0.129 to 0.613)	7.24<br>(2.73 to 12.93)	0.0305<br>(0.0101 to 0.0618)	0.49<br>(0.16 to 0.98)
Shaanxi	Other intestinal infectious diseases	1.19<br>(0.664 to 2.03)	3.47<br>(1.93 to 5.89)	0.103<br>(0.0386 to 0.192)	0.26<br>(0.1 to 0.49)
Shandong	Other intestinal infectious diseases	1.06<br>(0.611 to 1.61)	1.23<br>(0.71 to 1.88)	0.126<br>(0.0719 to 0.195)	0.13<br>(0.07 to 0.2)
Shanghai	Other intestinal infectious diseases	0.058<br>(0.0194 to 0.112)	0.42<br>(0.14 to 0.8)	0.0226<br>(0.00885 to 0.0443)	0.08<br>(0.03 to 0.16)
Shanxi	Other intestinal infectious diseases	0.666<br>(0.343 to 1.14)	2.2<br>(1.13 to 3.76)	0.0865<br>(0.0348 to 0.164)	0.22<br>(0.09 to 0.42)
Sichuan	Other intestinal infectious diseases	4.79<br>(2.61 to 8.27)	4.29<br>(2.34 to 7.41)	0.201<br>(0.099 to 0.34)	0.24<br>(0.12 to 0.41)
Tianjin	Other intestinal infectious diseases	0.0648<br>(0.0236 to 0.125)	0.71<br>(0.26 to 1.36)	0.0195<br>(0.00857 to 0.0358)	0.13<br>(0.06 to 0.23)
Tibet	Other intestinal infectious diseases	0.295<br>(0.134 to 0.506)	12.67<br>(5.74 to 21.72)	0.0476<br>(0.0122 to 0.104)	1.38<br>(0.35 to 3.01)
Xinjiang	Other intestinal infectious diseases	0.722<br>(0.376 to 1.19)	4.57<br>(2.38 to 7.57)	0.125<br>(0.0431 to 0.242)	0.51<br>(0.18 to 0.99)
Yunnan	Other intestinal infectious diseases	2.72<br>(1.62 to 4.49)	6.99<br>(4.15 to 11.51)	0.215<br>(0.102 to 0.353)	0.44<br>(0.21 to 0.72)
Zhejiang	Other intestinal infectious diseases	0.762<br>(0.393 to 1.31)	1.73<br>(0.89 to 2.97)	0.0738<br>(0.0352 to 0.128)	0.12<br>(0.06 to 0.21)
China	Lower respiratory infections	37700<br>(34400 to 40500)	3148.38<br>(2874.8 to 3384.83)	4290<br>(4020 to 5060)	304.02<br>(284.31 to 358.16)
Taiwan	Lower respiratory infections	92.8<br>(89.6 to 96)	454.85<br>(439.38 to 470.7)	174<br>(163 to 185)	737.96<br>(692.69 to 785.27)
Hong Kong Special Administrative Region of China	Lower respiratory infections	29.3<br>(11.4 to 34.6)	507.53<br>(197.23 to 600.8)	38.1<br>(13.2 to 49)	506.95<br>(175.93 to 652.63)
Macau Special Administrative Region of China	Lower respiratory infections	2.4<br>(1.73 to 2.76)	677.37<br>(486.37 to 778.35)	1.4<br>(0.748 to 1.71)	204.75<br>(109.34 to 249.85)
Anhui	Lower respiratory infections	2180<br>(1770 to 2660)	3683.39<br>(2979.68 to 4487.5)	139<br>(112 to 206)	227.23<br>(183.39 to 336.9)

Beijing	Lower respiratory infections	68.6 (56 to 80.1)	611.25 (499.22 to 714.33)	35.2 (29.5 to 41.5)	145.9 (122.36 to 172.03)
Chongqing	Lower respiratory infections	462 (341 to 614)	2924.85 (2163.73 to 3888.84)	65.8 (52.7 to 94.1)	246.23 (197.14 to 352.16)
Fujian	Lower respiratory infections	710 (585 to 838)	2243.41 (1848.25 to 2648.4)	92.9 (79.4 to 112)	235.6 (201.43 to 284.96)
Gansu	Lower respiratory infections	1550 (1270 to 1860)	6569.14 (5367.97 to 7884.07)	104 (82.7 to 136)	389.97 (311.32 to 513.54)
Guangdong	Lower respiratory infections	1380 (1130 to 1620)	2090.71 (1711 to 2459.49)	326 (268 to 381)	279.38 (229.05 to 326.18)
Guangxi	Lower respiratory infections	1340 (1040 to 1640)	3076.61 (2397.51 to 3767.87)	228 (175 to 269)	471.09 (361.31 to 556.97)
Guizhou	Lower respiratory infections	3030 (2330 to 3720)	9120.21 (7032.11 to 11197.95)	199 (136 to 239)	589.05 (402.02 to 707.91)
Hainan	Lower respiratory infections	199 (158 to 246)	2850.21 (2268.99 to 3525.67)	50 (41.2 to 59.8)	521.7 (429.9 to 623.36)
Hebei	Lower respiratory infections	957 (784 to 1160)	1502.9 (1232.24 to 1820.51)	240 (198 to 312)	314.4 (259.68 to 407.87)
Heilongjiang	Lower respiratory infections	383 (301 to 479)	1049.86 (825.99 to 1314.78)	81.2 (66.1 to 113)	206.87 (168.46 to 288.03)
Henan	Lower respiratory infections	2200 (1790 to 2650)	2491.04 (2027.84 to 2997.49)	203 (157 to 322)	210.87 (162.86 to 334.46)
Hubei	Lower respiratory infections	1210 (974 to 1470)	2172.59 (1753.79 to 2648.9)	112 (88.9 to 182)	203.12 (160.72 to 329.99)
Hunan	Lower respiratory infections	1650 (1320 to 2020)	2653.4 (2117.54 to 3254.49)	201 (171 to 243)	299.84 (254.88 to 362.2)
Inner Mongolia	Lower respiratory infections	595 (490 to 719)	2615.63 (2152.59 to 3161.73)	65.4 (53.4 to 87.2)	252.54 (206.27 to 336.82)
Jiangsu	Lower respiratory infections	655 (534 to 791)	952.46 (776.62 to 1149.26)	121 (96.5 to 196)	147.78 (118.2 to 239.92)
Jiangxi	Lower respiratory infections	2570 (2010 to 3180)	6509.35 (5082.25 to 8038.02)	215 (180 to 258)	453.21 (378.95 to 543.77)
Jilin	Lower respiratory infections	378 (308 to 456)	1457.22 (1185.98 to 1755.52)	73.5 (61.7 to 87)	263.5 (221.23 to 312.04)
Liaoning	Lower respiratory infections	250 (208 to 295)	613.88 (509.94 to 724.28)	98.1 (81.8 to 122)	219.62 (183.18 to 274.01)
Ningxia	Lower respiratory infections	345 (260 to 442)	6989.15 (5274.22 to 8945.69)	27.3 (21.5 to 34.5)	389 (306.72 to 491.83)
Qinghai	Lower respiratory infections	400 (303 to 515)	8453.39 (6405.12 to 10872.49)	42 (33 to 53.8)	668.65 (525.2 to 856.96)
Shaanxi	Lower respiratory infections	1270 (1040 to 1530)	3704.59 (3009.38 to 4446.54)	117 (91.6 to 159)	296.4 (231.77 to 403.1)
Shandong	Lower respiratory infections	690 (563 to 841)	806.16 (657.2 to 982.13)	143 (112 to 253)	143.53 (112.69 to 254.83)
Shanghai	Lower respiratory infections	58.6 (49.9 to 69.2)	419.31 (357.42 to 495.31)	34.2 (27.8 to 49)	122.45 (99.39 to 175.52)
Shanxi	Lower respiratory infections	712 (595 to 850)	2352.48 (1963.45 to 2805.66)	105 (84.9 to 136)	272.6 (219.79 to 351.79)
Sichuan	Lower respiratory infections	6370 (4960 to 7980)	5704.97 (4441.85 to 7152.45)	322 (267 to 396)	385.71 (319.97 to 473.7)
Tianjin	Lower respiratory infections	94.4 (71 to 112)	1025.95 (772.21 to 1213.62)	42.9 (32.6 to 50.8)	281.39 (213.57 to 332.8)
Tibet	Lower respiratory infections	251 (200 to 308)	10790.49 (8595.02 to 13201.46)	52.7 (41.9 to 65.8)	1524.83 (1210.94 to 1902.45)
Xinjiang	Lower respiratory infections	1270 (1020 to 1540)	8048.11 (6437.41 to 9787.32)	258 (207 to 320)	1057.78 (847.66 to 1314.68)
Yunnan	Lower respiratory infections	3700 (2890 to 4570)	9496.09 (7417.9 to 11724.15)	338 (279 to 408)	687.11 (567.88 to 830.66)
Zhejiang	Lower respiratory infections	726 (610 to 867)	1646.56 (1383.27 to 1965.27)	122 (105 to 143)	199.26 (170.82 to 232.98)
China	Upper respiratory infections	1660 (1080 to 2300)	138.8 (90.52 to 192.18)	1080 (660 to 1700)	76.7 (46.72 to 120.2)
Taiwan	Upper respiratory infections	17.7 (11.4 to 27)	86.95 (56.04 to 132.51)	16.1 (9.51 to 25.6)	68.07 (40.33 to 108.45)
Hong Kong Special Administrative Region of China	Upper respiratory infections	5.08 (3.14 to 7.77)	88.16 (54.48 to 134.73)	5.48 (3.27 to 8.65)	72.94 (43.57 to 115.17)

Macau Special Administrative Region of China	Upper respiratory infections	0.344 (0.214 to 0.523)	96.94 (60.18 to 147.36)	0.537 (0.324 to 0.833)	78.47 (47.32 to 121.83)
Anhui	Upper respiratory infections	74.8 (50.3 to 105)	126.23 (84.88 to 177.64)	45.9 (27.3 to 71.4)	75.06 (44.72 to 116.75)
Beijing	Upper respiratory infections	10.1 (6.38 to 15.5)	90.4 (56.89 to 137.93)	17.9 (10.4 to 28.4)	74.25 (43.24 to 117.84)
Chongqing	Upper respiratory infections	27.4 (15.1 to 39.5)	173.75 (95.71 to 250.45)	20.1 (12.2 to 31.3)	75.35 (45.83 to 117.07)
Fujian	Upper respiratory infections	41.6 (26.8 to 58.7)	131.52 (84.67 to 185.38)	30.3 (18.1 to 47.2)	76.99 (45.99 to 119.83)
Gansu	Upper respiratory infections	55.8 (28.1 to 83.5)	236 (118.78 to 352.85)	21.4 (13.1 to 33.2)	80.4 (49.25 to 124.78)
Guangdong	Upper respiratory infections	72.4 (48.1 to 105)	109.97 (73.02 to 159.33)	89.8 (53.1 to 141)	76.82 (45.46 to 121.03)
Guangxi	Upper respiratory infections	52.5 (36.4 to 73.8)	120.73 (83.77 to 169.62)	37.2 (22.3 to 58.2)	76.9 (46.16 to 120.52)
Guizhou	Upper respiratory infections	69.4 (37.2 to 107)	209.12 (112.18 to 321.38)	28 (17.9 to 42.4)	82.76 (52.91 to 125.49)
Hainan	Upper respiratory infections	10.3 (6.04 to 14.8)	147.59 (86.69 to 212.26)	7.89 (4.94 to 12.2)	82.32 (51.54 to 127.14)
Hebei	Upper respiratory infections	90.6 (53.3 to 127)	142.29 (83.75 to 200.06)	63.3 (39.6 to 97.1)	82.78 (51.82 to 127.09)
Heilongjiang	Upper respiratory infections	35 (22 to 52.4)	95.99 (60.39 to 143.68)	28.6 (17 to 44.9)	72.81 (43.25 to 114.41)
Henan	Upper respiratory infections	128 (82.2 to 176)	145.39 (93.12 to 199.79)	74.1 (44.6 to 116)	76.95 (46.26 to 120.96)
Hubei	Upper respiratory infections	62.8 (42 to 89.3)	113.02 (75.6 to 160.8)	40.8 (24.1 to 64.2)	73.71 (43.65 to 116.09)
Hunan	Upper respiratory infections	86.8 (52.4 to 122)	139.51 (84.31 to 196.53)	52.6 (32.3 to 82.4)	78.36 (48.04 to 122.67)
Inner Mongolia	Upper respiratory infections	39.8 (21.8 to 57)	175.03 (96.07 to 250.78)	19.8 (12 to 30.9)	76.37 (46.45 to 119.25)
Jiangsu	Upper respiratory infections	76.4 (50.6 to 109)	111.01 (73.48 to 158.42)	60 (35.4 to 94.4)	73.51 (43.3 to 115.59)
Jiangxi	Upper respiratory infections	79 (43.2 to 110)	199.76 (109.17 to 278.73)	37.5 (22.9 to 58.8)	78.83 (48.09 to 123.62)
Jilin	Upper respiratory infections	28.5 (18.4 to 41.3)	109.68 (70.84 to 159.12)	20.5 (12.4 to 32.2)	73.62 (44.52 to 115.51)
Liaoning	Upper respiratory infections	46.9 (28.2 to 68.6)	115 (69.25 to 168.1)	32.9 (19.7 to 52)	73.61 (44.07 to 116.4)
Ningxia	Upper respiratory infections	10.9 (5.21 to 15.9)	221.06 (105.44 to 321.63)	5.53 (3.35 to 8.72)	78.85 (47.78 to 124.18)
Qinghai	Upper respiratory infections	15.7 (5.53 to 25.8)	331.28 (116.82 to 543.86)	5.37 (3.36 to 8.18)	85.55 (53.53 to 130.21)
Shaanxi	Upper respiratory infections	50.9 (32 to 71.2)	147.8 (93.01 to 206.89)	30.3 (18.4 to 47.3)	76.57 (46.45 to 119.66)
Shandong	Upper respiratory infections	92.8 (61.7 to 136)	108.45 (72.06 to 158.92)	73.5 (43.7 to 114)	73.94 (44 to 115.14)
Shanghai	Upper respiratory infections	11.4 (6.95 to 17.9)	81.95 (49.78 to 128.15)	20.3 (12 to 32.2)	72.61 (43.05 to 115.31)
Shanxi	Upper respiratory infections	40.2 (25.9 to 56.7)	132.87 (85.39 to 187.29)	29.5 (17.7 to 46.6)	76.45 (45.77 to 120.6)
Sichuan	Upper respiratory infections	164 (107 to 229)	147.39 (95.48 to 204.93)	62.3 (37.2 to 97.2)	74.65 (44.6 to 116.38)
Tianjin	Upper respiratory infections	8.71 (5.64 to 13.1)	94.74 (61.31 to 142.46)	11.3 (6.68 to 18)	74.22 (43.76 to 118.17)
Tibet	Upper respiratory infections	5.84 (2.64 to 9.49)	250.42 (113.24 to 407.4)	3.24 (2.1 to 4.82)	93.65 (60.63 to 139.32)
Xinjiang	Upper respiratory infections	36.4 (15.2 to 51.3)	230.75 (96.31 to 325.55)	22 (13.8 to 33.6)	90.19 (56.78 to 138.02)
Yunnan	Upper respiratory infections	77.2 (40.5 to 113)	198.05 (104.05 to 289.39)	39.9 (24.4 to 61.8)	81.07 (49.72 to 125.73)
Zhejiang	Upper respiratory infections	53 (31.4 to 78.4)	120.28 (71.18 to 177.7)	45.7 (27.2 to 71.9)	74.74 (44.5 to 117.43)
China	Otitis media	398 (245 to 619)	33.27 (20.46 to 51.75)	270 (164 to 425)	19.08 (11.62 to 30.05)
Taiwan	Otitis media	4.29 (2.57 to 6.81)	21.03 (12.57 to 33.37)	3.26 (1.96 to 5.16)	13.8 (8.32 to 21.88)

Hong Kong Special Administrative Region of China	Otitis media	1.05 (0.65 to 1.66)	18.23 (11.27 to 28.74)	0.972 (0.587 to 1.55)	12.94 (7.81 to 20.68)
Macau Special Administrative Region of China	Otitis media	0.0633 (0.0384 to 0.102)	17.84 (10.83 to 28.69)	0.0788 (0.0474 to 0.127)	11.53 (6.93 to 18.59)
Anhui	Otitis media	20.9 (12.8 to 33.2)	35.31 (21.52 to 55.96)	13 (7.84 to 20.5)	21.29 (12.82 to 33.57)
Beijing	Otitis media	2.68 (1.64 to 4.26)	23.89 (14.6 to 37.99)	3.31 (1.99 to 5.29)	13.74 (8.26 to 21.94)
Chongqing	Otitis media	5.16 (3.13 to 8.25)	32.7 (19.81 to 52.27)	5.31 (3.19 to 8.38)	19.86 (11.95 to 31.37)
Fujian	Otitis media	10.5 (6.36 to 16.7)	33.25 (20.11 to 52.89)	6.84 (4.15 to 10.8)	17.35 (10.52 to 27.5)
Gansu	Otitis media	8.27 (5.02 to 13.2)	34.95 (21.2 to 55.61)	6.14 (3.8 to 9.79)	23.1 (14.3 to 36.83)
Guangdong	Otitis media	20.6 (12.5 to 32.3)	31.28 (19 to 49.13)	19.7 (11.9 to 31.3)	16.86 (10.17 to 26.79)
Guangxi	Otitis media	16.5 (10.3 to 25.5)	37.93 (23.76 to 58.56)	10.7 (6.53 to 17)	22.18 (13.52 to 35.14)
Guizhou	Otitis media	12.9 (7.88 to 20.5)	38.87 (23.75 to 61.83)	8.4 (5.11 to 13.2)	24.82 (15.11 to 39.01)
Hainan	Otitis media	2.34 (1.4 to 3.73)	33.53 (20.16 to 53.49)	2.02 (1.21 to 3.2)	21.06 (12.64 to 33.34)
Hebei	Otitis media	21.2 (13.2 to 33.4)	33.26 (20.76 to 52.5)	15.1 (9.27 to 23.4)	19.7 (12.13 to 30.67)
Heilongjiang	Otitis media	11.1 (6.71 to 17.8)	30.34 (18.4 to 48.81)	7.22 (4.42 to 11.4)	18.4 (11.27 to 29.07)
Henan	Otitis media	31.3 (19.2 to 48.9)	35.44 (21.71 to 55.39)	20.5 (12.6 to 32.5)	21.34 (13.11 to 33.79)
Hubei	Otitis media	17.8 (10.6 to 28.3)	32.05 (19.17 to 50.86)	10.6 (6.45 to 16.6)	19.17 (11.66 to 30.06)
Hunan	Otitis media	24 (15.6 to 36)	38.66 (25.08 to 57.89)	14 (8.59 to 22.3)	20.92 (12.79 to 33.16)
Inner Mongolia	Otitis media	7.87 (4.91 to 12.3)	34.59 (21.57 to 53.91)	4.67 (2.86 to 7.36)	18.05 (11.03 to 28.42)
Jiangsu	Otitis media	20.1 (12.2 to 32)	29.25 (17.66 to 46.43)	12.9 (7.79 to 20.4)	15.81 (9.54 to 25)
Jiangxi	Otitis media	14.4 (8.85 to 22.7)	36.35 (22.4 to 57.3)	10.7 (6.53 to 16.9)	22.54 (13.74 to 35.59)
Jilin	Otitis media	8.23 (5.01 to 12.9)	31.73 (19.31 to 49.77)	5.1 (3.1 to 8.07)	18.28 (11.12 to 28.92)
Liaoning	Otitis media	11.3 (6.81 to 18)	27.58 (16.68 to 44.21)	7.44 (4.53 to 11.8)	16.66 (10.15 to 26.41)
Ningxia	Otitis media	1.71 (1.03 to 2.7)	34.62 (20.88 to 54.57)	1.52 (0.923 to 2.4)	21.68 (13.15 to 34.25)
Qinghai	Otitis media	1.67 (1.04 to 2.59)	35.18 (21.88 to 54.74)	1.38 (0.836 to 2.18)	21.92 (13.31 to 34.76)
Shaanxi	Otitis media	10.9 (6.54 to 17.2)	31.59 (19.02 to 50.1)	7.63 (4.58 to 12)	19.32 (11.6 to 30.46)
Shandong	Otitis media	26 (15.8 to 41.6)	30.4 (18.5 to 48.55)	16.7 (10.3 to 26.3)	16.83 (10.34 to 26.43)
Shanghai	Otitis media	2.89 (1.73 to 4.63)	20.7 (12.36 to 33.16)	3.86 (2.32 to 6.17)	13.83 (8.3 to 22.07)
Shanxi	Otitis media	9.91 (5.97 to 16)	32.71 (19.73 to 52.78)	7.85 (4.68 to 12.5)	20.33 (12.12 to 32.3)
Sichuan	Otitis media	38.8 (23.6 to 61.7)	34.79 (21.11 to 55.28)	17 (10.2 to 26.9)	20.36 (12.26 to 32.27)
Tianjin	Otitis media	2.31 (1.39 to 3.62)	25.08 (15.07 to 39.38)	2.25 (1.34 to 3.54)	14.73 (8.8 to 23.18)
Tibet	Otitis media	0.82 (0.492 to 1.29)	35.2 (21.12 to 55.43)	0.852 (0.522 to 1.37)	24.65 (15.09 to 39.52)
Xinjiang	Otitis media	6.3 (4.17 to 9.4)	39.95 (26.42 to 59.61)	5.11 (3.11 to 8.1)	20.98 (12.77 to 33.22)
Yunnan	Otitis media	15.5 (9.85 to 23.8)	39.72 (25.29 to 61.05)	11.1 (6.74 to 17.6)	22.52 (13.71 to 35.73)
Zhejiang	Otitis media	13.2 (8.06 to 21.3)	29.98 (18.27 to 48.19)	9.48 (5.75 to 15.1)	15.49 (9.4 to 24.63)

China	Meningitis	2700 (2270 to 2990)	225.61 (189.5 to 249.63)	372 (296 to 406)	26.31 (20.98 to 28.77)
Taiwan	Meningitis	13.1 (10.9 to 14.1)	64.01 (53.53 to 69.33)	4.8 (4.27 to 6.12)	20.35 (18.11 to 25.97)
Hong Kong Special Administrative Region of China	Meningitis	1.87 (1.18 to 2.2)	32.44 (20.4 to 38.12)	0.814 (0.569 to 0.993)	10.84 (7.58 to 13.22)
Macau Special Administrative Region of China	Meningitis	0.226 (0.0919 to 0.29)	63.73 (25.88 to 81.8)	0.0907 (0.0364 to 0.126)	13.27 (5.32 to 18.36)
Anhui	Meningitis	148 (114 to 191)	249.5 (192.78 to 322.86)	12.7 (10.1 to 17.3)	20.82 (16.59 to 28.34)
Beijing	Meningitis	4.38 (3.55 to 6.19)	39.03 (31.64 to 55.2)	2.13 (1.67 to 3.12)	8.84 (6.91 to 12.94)
Chongqing	Meningitis	32.9 (23.9 to 44.6)	208.31 (151.74 to 282.47)	5.23 (4.29 to 6.49)	19.58 (16.06 to 24.28)
Fujian	Meningitis	44.1 (35 to 56)	139.36 (110.5 to 176.89)	7.01 (5.76 to 8.86)	17.78 (14.63 to 22.47)
Gansu	Meningitis	130 (99.1 to 164)	548.83 (419.12 to 692.96)	12.3 (9.28 to 15.4)	46.41 (34.9 to 57.78)
Guangdong	Meningitis	75.8 (61.4 to 91.2)	115.22 (93.27 to 138.59)	21.5 (17.4 to 26.7)	18.37 (14.88 to 22.85)
Guangxi	Meningitis	87.2 (63.6 to 106)	200.47 (146.25 to 243.78)	16.1 (11.4 to 19.4)	33.34 (23.65 to 40.06)
Guizhou	Meningitis	134 (99.2 to 168)	403.57 (298.86 to 507.21)	10.5 (8.1 to 13)	30.97 (23.95 to 38.42)
Hainan	Meningitis	10.6 (7.94 to 14.9)	152.71 (114.03 to 214.37)	2.87 (2.25 to 3.75)	29.95 (23.48 to 39.08)
Hebei	Meningitis	165 (90.7 to 214)	259.11 (142.45 to 335.59)	39.7 (22.3 to 51)	52.01 (29.13 to 66.7)
Heilongjiang	Meningitis	60.1 (38.2 to 77.7)	164.86 (104.66 to 213.04)	9.83 (6.38 to 12.1)	25.06 (16.26 to 30.86)
Henan	Meningitis	277 (175 to 359)	314.25 (198.22 to 406.64)	31.3 (19.1 to 39.8)	32.48 (19.79 to 41.37)
Hubei	Meningitis	79.2 (59.6 to 113)	142.58 (107.32 to 202.7)	8.21 (6.53 to 12.3)	14.84 (11.8 to 22.28)
Hunan	Meningitis	117 (85.8 to 144)	187.54 (137.93 to 231.36)	14.8 (11.9 to 18)	22.07 (17.67 to 26.78)
Inner Mongolia	Meningitis	62 (47.5 to 78)	272.48 (208.79 to 342.97)	7.36 (5.86 to 9.15)	28.43 (22.64 to 35.33)
Jiangsu	Meningitis	61.2 (50.3 to 75.7)	88.98 (73.15 to 110.03)	9.31 (7.71 to 12.5)	11.41 (9.44 to 15.34)
Jiangxi	Meningitis	162 (124 to 212)	409.3 (313.52 to 536.78)	17.7 (14.4 to 21.6)	37.19 (30.33 to 45.48)
Jilin	Meningitis	48.7 (32 to 65)	187.72 (123.23 to 250.57)	9.16 (4.75 to 11.9)	32.84 (17.04 to 42.54)
Liaoning	Meningitis	30 (24.2 to 37.4)	73.66 (59.39 to 91.81)	6.47 (5.27 to 8.04)	14.48 (11.81 to 18.01)
Ningxia	Meningitis	20.9 (15.2 to 28.2)	423.04 (307.66 to 571.51)	2.27 (1.79 to 2.97)	32.3 (25.44 to 42.39)
Qinghai	Meningitis	33.9 (22.1 to 46.1)	716.64 (467.13 to 972.57)	4.22 (2.78 to 5.43)	67.2 (44.3 to 86.42)
Shaanxi	Meningitis	93.8 (72 to 117)	272.74 (209.2 to 339.79)	13 (9.82 to 16.6)	33.02 (24.85 to 42.11)
Shandong	Meningitis	113 (81.6 to 139)	132.1 (95.29 to 162.55)	18.1 (13.3 to 21.7)	18.17 (13.43 to 21.87)
Shanghai	Meningitis	4.38 (3.36 to 7.34)	31.34 (24.07 to 52.54)	2.21 (1.63 to 3.75)	7.91 (5.82 to 13.42)
Shanxi	Meningitis	65.4 (45.9 to 83.3)	215.96 (151.73 to 275.15)	11.5 (8.22 to 14.5)	29.82 (21.29 to 37.43)
Sichuan	Meningitis	310 (235 to 409)	277.96 (210.14 to 366.54)	21.1 (17.1 to 25.7)	25.23 (20.48 to 30.83)
Tianjin	Meningitis	5.72 (4.72 to 7.43)	62.18 (51.37 to 80.77)	2.12 (1.69 to 2.98)	13.87 (11.11 to 19.53)
Tibet	Meningitis	11.9 (8.98 to 15.4)	512.36 (385.47 to 660.52)	3.51 (2.7 to 4.68)	101.44 (78.21 to 135.53)
Xinjiang	Meningitis	87.4 (54.8 to 116)	554.27 (347.68 to 738.27)	19 (11.7 to 24.8)	78 (47.88 to 101.81)

Yunnan	Meningitis	178 (136 to 225)	455.49 (349.77 to 576.66)	22.4 (18.3 to 27.4)	45.54 (37.12 to 55.72)
Zhejiang	Meningitis	45.4 (35.3 to 68.2)	103.06 (80 to 154.76)	7.1 (5.59 to 10.2)	11.61 (9.13 to 16.7)
China	Encephalitis	895 (621 to 1090)	74.77 (51.85 to 91.09)	306 (263 to 360)	21.69 (18.59 to 25.5)
Taiwan	Encephalitis	4.41 (3.93 to 4.92)	21.6 (19.27 to 24.11)	3.24 (2.9 to 3.67)	13.74 (12.28 to 15.57)
Hong Kong Special Administrative Region of China	Encephalitis	0.889 (0.713 to 1.04)	15.42 (12.37 to 18.12)	0.673 (0.519 to 0.831)	8.97 (6.91 to 11.07)
Macau Special Administrative Region of China	Encephalitis	0.0509 (0.0423 to 0.0603)	14.35 (11.92 to 16.98)	0.0593 (0.0354 to 0.0772)	8.68 (5.18 to 11.29)
Anhui	Encephalitis	64.7 (42.3 to 85.8)	109.19 (71.35 to 144.83)	16.9 (13.7 to 20.6)	27.6 (22.46 to 33.65)
Beijing	Encephalitis	4.42 (3.6 to 5.34)	39.43 (32.12 to 47.64)	3.77 (2.97 to 4.84)	15.64 (12.32 to 20.07)
Chongqing	Encephalitis	9.23 (6.74 to 13.2)	58.47 (42.72 to 83.89)	3.63 (2.85 to 5.72)	13.57 (10.66 to 21.39)
Fujian	Encephalitis	22 (15.5 to 27.2)	69.5 (48.88 to 85.86)	7.54 (6.13 to 9.32)	19.12 (15.55 to 23.65)
Gansu	Encephalitis	21.7 (15.7 to 29.4)	91.63 (66.44 to 124.21)	5.4 (4.3 to 7.49)	20.33 (16.17 to 28.19)
Guangdong	Encephalitis	48.6 (29.3 to 62.2)	73.91 (44.5 to 94.49)	30.4 (24.8 to 37.1)	26.03 (21.24 to 31.76)
Guangxi	Encephalitis	39.6 (23.3 to 51)	91.05 (53.52 to 117.34)	14.3 (12 to 17.2)	29.58 (24.75 to 35.59)
Guizhou	Encephalitis	35.5 (23.3 to 50.7)	106.91 (70.15 to 152.83)	9.56 (7.33 to 12.1)	28.27 (21.65 to 35.63)
Hainan	Encephalitis	6.13 (2.98 to 9.76)	88.03 (42.8 to 140.1)	3.7 (2.71 to 4.81)	38.6 (28.27 to 50.19)
Hebei	Encephalitis	40.6 (32.2 to 50.6)	63.77 (50.64 to 79.5)	23.2 (17.8 to 28.6)	30.36 (23.23 to 37.38)
Heilongjiang	Encephalitis	21.9 (17.3 to 28.2)	59.96 (47.49 to 77.39)	8.98 (7.2 to 11)	22.88 (18.35 to 27.96)
Henan	Encephalitis	80.6 (53.6 to 109)	91.26 (60.7 to 123.3)	24.6 (20 to 30.6)	25.56 (20.76 to 31.83)
Hubei	Encephalitis	27.3 (21.3 to 36.1)	49.12 (38.25 to 64.99)	8.57 (6.68 to 14)	15.49 (12.08 to 25.23)
Hunan	Encephalitis	30.1 (23.7 to 39.7)	48.37 (38.05 to 63.79)	11.4 (9.18 to 16.7)	16.97 (13.67 to 24.92)
Inner Mongolia	Encephalitis	17.5 (11.5 to 23)	76.82 (50.77 to 101.36)	5.14 (4.08 to 6.38)	19.83 (15.75 to 24.64)
Jiangsu	Encephalitis	33.3 (26.9 to 41.2)	48.37 (39.03 to 59.94)	12.7 (10 to 18)	15.56 (12.24 to 22.05)
Jiangxi	Encephalitis	80.1 (26 to 108)	202.49 (65.82 to 272.19)	20.2 (14.8 to 24.6)	42.46 (31.22 to 51.76)
Jilin	Encephalitis	16.4 (12.2 to 21.3)	63.01 (47 to 81.92)	5.3 (4.29 to 6.44)	19 (15.38 to 23.09)
Liaoning	Encephalitis	12.6 (10.1 to 15.6)	30.86 (24.84 to 38.28)	5.17 (4.26 to 6.85)	11.58 (9.54 to 15.34)
Ningxia	Encephalitis	3.78 (2.7 to 5.42)	76.53 (54.64 to 109.82)	1.18 (0.897 to 1.75)	16.77 (12.78 to 24.94)
Qinghai	Encephalitis	5.08 (3.21 to 7.1)	107.21 (67.67 to 149.85)	1.52 (1.22 to 1.9)	24.18 (19.37 to 30.18)
Shaanxi	Encephalitis	32.7 (16.6 to 44)	95.17 (48.14 to 127.98)	9.08 (7.19 to 11.4)	22.98 (18.2 to 28.81)
Shandong	Encephalitis	40.9 (33.4 to 50.5)	47.81 (39.07 to 58.98)	16.6 (13.1 to 24.4)	16.68 (13.22 to 24.51)
Shanghai	Encephalitis	5.98 (4.09 to 7.39)	42.82 (29.28 to 52.9)	4.96 (3.93 to 6.13)	17.75 (14.08 to 21.96)
Shanxi	Encephalitis	16.2 (12.7 to 20.5)	53.6 (41.92 to 67.59)	6.45 (5.2 to 8.86)	16.71 (13.47 to 22.93)
Sichuan	Encephalitis	86.9 (52.7 to 125)	77.84 (47.24 to 112.15)	14.4 (11.6 to 17.9)	17.2 (13.84 to 21.46)
Tianjin	Encephalitis	3.06 (2.56 to 3.71)	33.29 (27.88 to 40.33)	2.18 (1.79 to 2.9)	14.29 (11.7 to 19.03)

Tibet	Encephalitis	1.45<br>(1.05 to 1.98)	62.02<br>(45.26 to 84.81)	0.807<br>(0.628 to 1.12)	23.36<br>(18.17 to 32.42)
Xinjiang	Encephalitis	10.7<br>(7.44 to 14.2)	67.53<br>(47.15 to 90.08)	5.24<br>(3.67 to 6.9)	21.5<br>(15.04 to 28.32)
Yunnan	Encephalitis	51<br>(23.8 to 69.5)	130.91<br>(61.11 to 178.44)	13<br>(10.5 to 16)	26.5<br>(21.45 to 32.58)
Zhejiang	Encephalitis	24.3<br>(18.4 to 31.9)	55.08<br>(41.76 to 72.31)	9.77<br>(7.93 to 12.5)	15.97<br>(12.96 to 20.45)
China	Diphtheria	23.5<br>(18.9 to 29.9)	1.97<br>(1.58 to 2.5)	1.23<br>(1.06 to 1.42)	0.09<br>(0.08 to 0.1)
Taiwan	Diphtheria	0.00117<br>(0.00114 to 0.0012)	0.01<br>(0.01 to 0.01)	0.00104<br>(0.000964 to 0.00114)	0<br>(0 to 0)
Hong Kong Special Administrative Region of China	Diphtheria	0.00368<br>(0.00222 to 0.00554)	0.06<br>(0.04 to 0.1)	0.00185<br>(0.00108 to 0.00281)	0.02<br>(0.01 to 0.04)
Macau Special Administrative Region of China	Diphtheria	0.000299<br>(0.000172 to 0.00048)	0.08<br>(0.05 to 0.14)	0.000129<br>(7.87e-05 to 0.000196)	0.02<br>(0.01 to 0.03)
Anhui	Diphtheria	1.03<br>(0.443 to 1.91)	1.74<br>(0.75 to 3.22)	0.0549<br>(0.0311 to 0.0904)	0.09<br>(0.05 to 0.15)
Beijing	Diphtheria	0.0215<br>(0.0117 to 0.0354)	0.19<br>(0.1 to 0.32)	0.00827<br>(0.00447 to 0.0144)	0.03<br>(0.02 to 0.06)
Chongqing	Diphtheria	0.241<br>(0.108 to 0.455)	1.53<br>(0.69 to 2.89)	0.0172<br>(0.00971 to 0.0277)	0.06<br>(0.04 to 0.1)
Fujian	Diphtheria	0.3<br>(0.14 to 0.551)	0.95<br>(0.44 to 1.74)	0.0277<br>(0.0157 to 0.0466)	0.07<br>(0.04 to 0.12)
Gansu	Diphtheria	0.962<br>(0.439 to 1.78)	4.07<br>(1.86 to 7.51)	0.0333<br>(0.0184 to 0.0561)	0.13<br>(0.07 to 0.21)
Guangdong	Diphtheria	0.495<br>(0.218 to 0.899)	0.75<br>(0.33 to 1.37)	0.0842<br>(0.045 to 0.14)	0.07<br>(0.04 to 0.12)
Guangxi	Diphtheria	0.883<br>(0.392 to 1.59)	2.03<br>(0.9 to 3.66)	0.0568<br>(0.032 to 0.0963)	0.12<br>(0.07 to 0.2)
Guizhou	Diphtheria	2.8<br>(1.24 to 5.22)	8.42<br>(3.75 to 15.72)	0.0331<br>(0.0194 to 0.0526)	0.1<br>(0.06 to 0.16)
Hainan	Diphtheria	0.104<br>(0.0506 to 0.196)	1.5<br>(0.73 to 2.81)	0.0113<br>(0.0058 to 0.0194)	0.12<br>(0.06 to 0.2)
Hebei	Diphtheria	0.556<br>(0.251 to 1.07)	0.87<br>(0.4 to 1.69)	0.102<br>(0.0553 to 0.171)	0.13<br>(0.07 to 0.22)
Heilongjiang	Diphtheria	0.249<br>(0.137 to 0.447)	0.68<br>(0.38 to 1.22)	0.0287<br>(0.017 to 0.045)	0.07<br>(0.04 to 0.11)
Henan	Diphtheria	1.33<br>(0.598 to 2.47)	1.51<br>(0.68 to 2.8)	0.0852<br>(0.0493 to 0.141)	0.09<br>(0.05 to 0.15)
Hubei	Diphtheria	0.716<br>(0.331 to 1.34)	1.29<br>(0.6 to 2.42)	0.0392<br>(0.0223 to 0.0605)	0.07<br>(0.04 to 0.11)
Hunan	Diphtheria	0.902<br>(0.46 to 1.56)	1.45<br>(0.74 to 2.51)	0.0596<br>(0.0335 to 0.0943)	0.09<br>(0.05 to 0.14)
Inner Mongolia	Diphtheria	0.393<br>(0.189 to 0.699)	1.73<br>(0.83 to 3.07)	0.0256<br>(0.0138 to 0.0426)	0.1<br>(0.05 to 0.16)
Jiangsu	Diphtheria	0.349<br>(0.17 to 0.625)	0.51<br>(0.25 to 0.91)	0.0363<br>(0.0205 to 0.0574)	0.04<br>(0.03 to 0.07)
Jiangxi	Diphtheria	1.45<br>(0.624 to 2.76)	3.67<br>(1.58 to 6.98)	0.0636<br>(0.0336 to 0.112)	0.13<br>(0.07 to 0.24)
Jilin	Diphtheria	0.207<br>(0.107 to 0.355)	0.8<br>(0.41 to 1.37)	0.0196<br>(0.0116 to 0.0308)	0.07<br>(0.04 to 0.11)
Liaoning	Diphtheria	0.143<br>(0.0815 to 0.237)	0.35<br>(0.2 to 0.58)	0.0239<br>(0.014 to 0.0371)	0.05<br>(0.03 to 0.08)
Ningxia	Diphtheria	0.175<br>(0.0651 to 0.349)	3.54<br>(1.32 to 7.07)	0.00859<br>(0.00441 to 0.015)	0.12<br>(0.06 to 0.21)
Qinghai	Diphtheria	0.441<br>(0.185 to 0.946)	9.31<br>(3.9 to 19.98)	0.0119<br>(0.00618 to 0.021)	0.19<br>(0.1 to 0.33)
Shaanxi	Diphtheria	0.789<br>(0.314 to 1.58)	2.29<br>(0.91 to 4.59)	0.0389<br>(0.0215 to 0.0685)	0.1<br>(0.05 to 0.17)
Shandong	Diphtheria	0.502<br>(0.259 to 0.862)	0.59<br>(0.3 to 1.01)	0.0611<br>(0.0346 to 0.0955)	0.06<br>(0.03 to 0.1)
Shanghai	Diphtheria	0.0256<br>(0.0135 to 0.0453)	0.18<br>(0.1 to 0.32)	0.0111<br>(0.00573 to 0.0192)	0.04<br>(0.02 to 0.07)
Shanxi	Diphtheria	0.268<br>(0.133 to 0.478)	0.88<br>(0.44 to 1.58)	0.0298<br>(0.0168 to 0.0489)	0.08<br>(0.04 to 0.13)

Sichuan	Diphtheria	3.21<br>(1.26 to 6.6)	2.88<br>(1.13 to 5.92)	0.0713<br>(0.0411 to 0.114)	0.09<br>(0.05 to 0.14)
Tianjin	Diphtheria	0.0296<br>(0.0152 to 0.0504)	0.32<br>(0.17 to 0.55)	0.00992<br>(0.00538 to 0.0158)	0.06<br>(0.04 to 0.1)
Tibet	Diphtheria	0.426<br>(0.182 to 0.812)	18.29<br>(7.81 to 34.84)	0.0162<br>(0.0082 to 0.0301)	0.47<br>(0.24 to 0.87)
Xinjiang	Diphtheria	1.12<br>(0.469 to 2.13)	7.09<br>(2.97 to 13.51)	0.0524<br>(0.0265 to 0.092)	0.22<br>(0.11 to 0.38)
Yunnan	Diphtheria	3.18<br>(1.38 to 6.24)	8.17<br>(3.54 to 16.02)	0.077<br>(0.0406 to 0.128)	0.16<br>(0.08 to 0.26)
Zhejiang	Diphtheria	0.239<br>(0.111 to 0.454)	0.54<br>(0.25 to 1.03)	0.031<br>(0.017 to 0.0519)	0.05<br>(0.03 to 0.08)
China	Whooping cough	3050<br>(302 to 8300)	255.12<br>(25.19 to 693.01)	70.9<br>(9.47 to 228)	5.02<br>(0.67 to 16.12)
Taiwan	Whooping cough	0.288<br>(0.169 to 0.454)	1.41<br>(0.83 to 2.22)	0.0939<br>(0.0457 to 0.146)	0.4<br>(0.19 to 0.62)
Hong Kong Special Administrative Region of China	Whooping cough	0.786<br>(0.0276 to 4.25)	13.64<br>(0.48 to 73.76)	0.138<br>(0.00846 to 0.9)	1.84<br>(0.11 to 11.99)
Macau Special Administrative Region of China	Whooping cough	0.0629<br>(0.00322 to 0.37)	17.71<br>(0.91 to 104.15)	0.00716<br>(0.000688 to 0.0354)	1.05<br>(0.1 to 5.18)
Anhui	Whooping cough	189<br>(7.79 to 770)	318.6<br>(13.15 to 1300.3)	4.33<br>(0.234 to 19.6)	7.08<br>(0.38 to 32.13)
Beijing	Whooping cough	4.12<br>(0.228 to 16.5)	36.73<br>(2.03 to 146.92)	0.278<br>(0.0282 to 1.16)	1.15<br>(0.12 to 4.82)
Chongqing	Whooping cough	49.1<br>(1.58 to 215)	310.97<br>(10.01 to 1359.65)	0.987<br>(0.0781 to 4.9)	3.69<br>(0.29 to 18.34)
Fujian	Whooping cough	67.3<br>(3.04 to 271)	212.82<br>(9.6 to 856.79)	1.06<br>(0.0989 to 4.49)	2.69<br>(0.25 to 11.39)
Gansu	Whooping cough	125<br>(4.29 to 557)	529.6<br>(18.13 to 2354.91)	2.8<br>(0.163 to 13.4)	10.54<br>(0.61 to 50.38)
Guangdong	Whooping cough	93.1<br>(4.53 to 351)	141.39<br>(6.89 to 534)	4.05<br>(0.287 to 19.7)	3.47<br>(0.25 to 16.86)
Guangxi	Whooping cough	129<br>(5.05 to 505)	297.09<br>(11.62 to 1160.41)	4.39<br>(0.26 to 18.2)	9.08<br>(0.54 to 37.57)
Guizhou	Whooping cough	210<br>(7.85 to 824)	633.41<br>(23.64 to 2483.91)	4.77<br>(0.255 to 20.1)	14.11<br>(0.75 to 59.38)
Hainan	Whooping cough	14.8<br>(0.65 to 60.4)	211.8<br>(9.33 to 867)	0.908<br>(0.0544 to 4.06)	9.47<br>(0.57 to 42.31)
Hebei	Whooping cough	119<br>(6.28 to 480)	186.24<br>(9.87 to 754.67)	4.01<br>(0.27 to 17.7)	5.24<br>(0.35 to 23.16)
Heilongjiang	Whooping cough	62.8<br>(2.32 to 266)	172.35<br>(6.37 to 730.34)	1.53<br>(0.0783 to 7.41)	3.9<br>(0.2 to 18.88)
Henan	Whooping cough	271<br>(9.17 to 1140)	306.52<br>(10.39 to 1294.32)	5.38<br>(0.352 to 22.5)	5.59<br>(0.37 to 23.41)
Hubei	Whooping cough	148<br>(5.68 to 571)	266.57<br>(10.23 to 1027.6)	2.76<br>(0.179 to 11.6)	4.99<br>(0.32 to 20.91)
Hunan	Whooping cough	160<br>(8.36 to 585)	257.25<br>(13.45 to 941.06)	3.64<br>(0.269 to 14.9)	5.41<br>(0.4 to 22.24)
Inner Mongolia	Whooping cough	61.1<br>(2 to 243)	268.77<br>(8.79 to 1067.22)	0.911<br>(0.0577 to 4.28)	3.52<br>(0.22 to 16.53)
Jiangsu	Whooping cough	86<br>(4.01 to 340)	124.94<br>(5.83 to 493.46)	1.42<br>(0.109 to 5.99)	1.74<br>(0.13 to 7.34)
Jiangxi	Whooping cough	171<br>(5.79 to 747)	431.52<br>(14.65 to 1890.21)	3.7<br>(0.254 to 15.3)	7.79<br>(0.53 to 32.22)
Jilin	Whooping cough	56.5<br>(2.41 to 214)	217.61<br>(9.27 to 823.5)	0.994<br>(0.0649 to 4.74)	3.56<br>(0.23 to 17)
Liaoning	Whooping cough	35.1<br>(1.59 to 143)	86.02<br>(3.89 to 350.15)	0.715<br>(0.0549 to 3.52)	1.6<br>(0.12 to 7.89)
Ningxia	Whooping cough	19.9<br>(0.715 to 83.2)	403.64<br>(14.47 to 1684.27)	0.529<br>(0.0345 to 2.52)	7.54<br>(0.49 to 35.93)
Qinghai	Whooping cough	26.6<br>(1 to 100)	560.9<br>(21.15 to 2120.26)	0.793<br>(0.0499 to 3.27)	12.62<br>(0.79 to 52.11)
Shaanxi	Whooping cough	115<br>(5.17 to 511)	332.99<br>(15.03 to 1483.81)	2.41<br>(0.154 to 10.3)	6.09<br>(0.39 to 26.1)
Shandong	Whooping cough	139<br>(6.29 to 580)	161.82<br>(7.35 to 677.8)	2.72<br>(0.193 to 11.9)	2.73<br>(0.19 to 11.94)

Shanghai	Whooping cough	3.24<br>(0.19 to 13.3)	23.22<br>(1.36 to 95.18)	0.318<br>(0.0301 to 1.44)	1.14<br>(0.11 to 5.14)
Shanxi	Whooping cough	69<br>(3.05 to 275)	227.98<br>(10.07 to 909.56)	2.09<br>(0.116 to 8.88)	5.42<br>(0.3 to 23)
Sichuan	Whooping cough	304<br>(12.1 to 1310)	272.76<br>(10.87 to 1173.68)	3.43<br>(0.268 to 14.4)	4.1<br>(0.32 to 17.28)
Tianjin	Whooping cough	4.82<br>(0.272 to 21.7)	52.42<br>(2.96 to 235.7)	0.258<br>(0.0213 to 1.18)	1.69<br>(0.14 to 7.75)
Tibet	Whooping cough	10.4<br>(0.458 to 43.8)	447.27<br>(19.63 to 1881.56)	1.2<br>(0.0671 to 5.22)	34.6<br>(1.94 to 150.95)
Xinjiang	Whooping cough	64.8<br>(2.5 to 288)	411.01<br>(15.83 to 1825.82)	2.53<br>(0.16 to 11.1)	10.36<br>(0.66 to 45.43)
Yunnan	Whooping cough	191<br>(7.55 to 773)	489.79<br>(19.38 to 1984.61)	5.03<br>(0.347 to 20.6)	10.23<br>(0.71 to 41.93)
Zhejiang	Whooping cough	54.6<br>(2.34 to 221)	123.75<br>(5.3 to 501.88)	0.856<br>(0.0877 to 3.72)	1.4<br>(0.14 to 6.08)
China	Tetanus	1680<br>(1390 to 2060)	140.27<br>(115.87 to 172.11)	22.9<br>(16.7 to 29.7)	1.62<br>(1.18 to 2.1)
Taiwan	Tetanus	0.49<br>(0.379 to 0.532)	2.4<br>(1.86 to 2.61)	0.0121<br>(0.00705 to 0.0254)	0.05<br>(0.03 to 0.11)
Hong Kong Special Administrative Region of China	Tetanus	0.391<br>(0.254 to 0.491)	6.79<br>(4.41 to 8.52)	0.0833<br>(0.0458 to 0.11)	1.11<br>(0.61 to 1.47)
Macau Special Administrative Region of China	Tetanus	0.122<br>(0.0673 to 0.187)	34.42<br>(18.95 to 52.56)	0.0551<br>(0.0105 to 0.0952)	8.05<br>(1.53 to 13.92)
Anhui	Tetanus	65.4<br>(40 to 114)	110.34<br>(67.55 to 192.3)	0.78<br>(0.592 to 1.22)	1.28<br>(0.97 to 2)
Beijing	Tetanus	0.467<br>(0.307 to 0.808)	4.17<br>(2.74 to 7.2)	0.126<br>(0.0995 to 0.15)	0.52<br>(0.41 to 0.62)
Chongqing	Tetanus	27.3<br>(17 to 41.7)	172.84<br>(107.45 to 264.41)	0.39<br>(0.281 to 0.527)	1.46<br>(1.05 to 1.97)
Fujian	Tetanus	26.2<br>(16.7 to 40.1)	82.94<br>(52.69 to 126.85)	0.386<br>(0.298 to 0.565)	0.98<br>(0.76 to 1.43)
Gansu	Tetanus	79.7<br>(48.5 to 124)	336.86<br>(205.15 to 524.62)	0.501<br>(0.362 to 0.886)	1.88<br>(1.36 to 3.33)
Guangdong	Tetanus	70.4<br>(48.6 to 101)	106.97<br>(73.91 to 152.71)	2.04<br>(1.29 to 2.63)	1.74<br>(1.1 to 2.25)
Guangxi	Tetanus	145<br>(100 to 199)	333.73<br>(230.3 to 456.48)	2.24<br>(0.864 to 3.05)	4.64<br>(1.79 to 6.31)
Guizhou	Tetanus	120<br>(77.8 to 181)	362<br>(234.48 to 545.49)	0.844<br>(0.589 to 1.53)	2.49<br>(1.74 to 4.54)
Hainan	Tetanus	17.7<br>(10.9 to 27.6)	253.57<br>(156 to 395.9)	0.391<br>(0.207 to 0.559)	4.08<br>(2.16 to 5.83)
Hebei	Tetanus	61.5<br>(40.5 to 89.8)	96.64<br>(63.66 to 141.04)	1.67<br>(1.06 to 2.96)	2.19<br>(1.39 to 3.87)
Heilongjiang	Tetanus	5.7<br>(3.1 to 11.3)	15.63<br>(8.5 to 31.08)	0.316<br>(0.236 to 0.57)	0.81<br>(0.6 to 1.45)
Henan	Tetanus	84.6<br>(54.6 to 136)	95.83<br>(61.84 to 153.98)	1.4<br>(1 to 1.82)	1.46<br>(1.04 to 1.89)
Hubei	Tetanus	66.5<br>(43.4 to 102)	119.67<br>(78.07 to 182.73)	0.878<br>(0.51 to 1.17)	1.59<br>(0.92 to 2.11)
Hunan	Tetanus	58.3<br>(39.3 to 86)	93.77<br>(63.23 to 138.29)	0.962<br>(0.599 to 1.39)	1.43<br>(0.89 to 2.08)
Inner Mongolia	Tetanus	7.49<br>(4.66 to 13.5)	32.94<br>(20.51 to 59.55)	0.257<br>(0.194 to 0.354)	0.99<br>(0.75 to 1.37)
Jiangsu	Tetanus	35.9<br>(24.4 to 52.6)	52.12<br>(35.44 to 76.45)	0.846<br>(0.56 to 1.09)	1.04<br>(0.69 to 1.34)
Jiangxi	Tetanus	125<br>(77.1 to 197)	315.4<br>(195.1 to 497.61)	1.01<br>(0.775 to 1.38)	2.13<br>(1.63 to 2.91)
Jilin	Tetanus	4.73<br>(2.7 to 8.49)	18.23<br>(10.39 to 32.73)	0.226<br>(0.171 to 0.363)	0.81<br>(0.61 to 1.3)
Liaoning	Tetanus	10.9<br>(6.79 to 16.5)	26.74<br>(16.66 to 40.46)	0.562<br>(0.327 to 0.749)	1.26<br>(0.73 to 1.68)
Ningxia	Tetanus	7.86<br>(4.57 to 13.5)	159.2<br>(92.54 to 273.42)	0.0839<br>(0.0608 to 0.146)	1.2<br>(0.87 to 2.09)
Qinghai	Tetanus	9.92<br>(5.84 to 16.7)	209.45<br>(123.28 to 353.55)	0.115<br>(0.0798 to 0.278)	1.83<br>(1.27 to 4.43)

Shaanxi	Tetanus	49.2 (29.6 to 77.8)	142.92 (86 to 226.14)	0.631 (0.453 to 0.893)	1.6 (1.15 to 2.26)
Shandong	Tetanus	26 (18.1 to 37.7)	30.41 (21.13 to 44.06)	0.913 (0.674 to 1.19)	0.92 (0.68 to 1.2)
Shanghai	Tetanus	1.09 (0.734 to 1.63)	7.78 (5.25 to 11.68)	0.185 (0.146 to 0.227)	0.66 (0.52 to 0.81)
Shanxi	Tetanus	10.3 (6.73 to 16.8)	34.16 (22.23 to 55.49)	0.344 (0.265 to 0.502)	0.89 (0.69 to 1.3)
Sichuan	Tetanus	334 (210 to 516)	299.05 (188.24 to 462.14)	1.6 (1.12 to 2.13)	1.91 (1.34 to 2.55)
Tianjin	Tetanus	1.26 (0.852 to 1.83)	13.75 (9.27 to 19.87)	0.115 (0.0902 to 0.138)	0.76 (0.59 to 0.91)
Tibet	Tetanus	3.9 (1.98 to 10.4)	167.42 (84.9 to 445.48)	0.125 (0.0536 to 0.633)	3.61 (1.55 to 18.3)
Xinjiang	Tetanus	62.1 (39.1 to 97.2)	393.54 (247.65 to 616.35)	0.724 (0.445 to 1.18)	2.97 (1.83 to 4.83)
Yunnan	Tetanus	143 (89 to 224)	366.46 (228.35 to 574.6)	1.68 (1.2 to 2.37)	3.42 (2.45 to 4.82)
Zhejiang	Tetanus	17.6 (11.3 to 26.6)	39.86 (25.54 to 60.22)	0.439 (0.335 to 0.621)	0.72 (0.55 to 1.02)
China	Measles	2550 (932 to 5530)	212.78 (77.88 to 462.11)	121 (43.3 to 269)	8.6 (3.07 to 19.06)
Taiwan	Measles	0.805 (0.371 to 1.51)	3.95 (1.82 to 7.42)	0.0605 (0.024 to 0.102)	0.26 (0.1 to 0.43)
Hong Kong Special Administrative Region of China	Measles	0.353 (0.116 to 0.83)	6.13 (2.02 to 14.4)	0.21 (0.0655 to 0.469)	2.8 (0.87 to 6.24)
Macau Special Administrative Region of China	Measles	0.0211 (0.00708 to 0.049)	5.94 (1.99 to 13.82)	0.0116 (0.00371 to 0.0273)	1.69 (0.54 to 3.99)
Anhui	Measles	161 (56.3 to 362)	272.41 (94.99 to 611.25)	7.16 (2.45 to 15.7)	11.71 (4.01 to 25.62)
Beijing	Measles	2.23 (0.769 to 5)	19.92 (6.86 to 44.58)	0.33 (0.103 to 0.764)	1.37 (0.43 to 3.17)
Chongqing	Measles	36.7 (13.3 to 85.8)	232.72 (84.26 to 543.64)	1.68 (0.539 to 3.79)	6.27 (2.02 to 14.17)
Fujian	Measles	60.3 (21.3 to 134)	190.52 (67.35 to 422.56)	1.85 (0.574 to 4.27)	4.7 (1.46 to 10.82)
Gansu	Measles	125 (45 to 266)	528.76 (190.26 to 1126.26)	5.74 (1.93 to 12.8)	21.6 (7.26 to 48.18)
Guangdong	Measles	73.8 (26.1 to 164)	112.11 (39.71 to 249.33)	5.42 (1.81 to 12.7)	4.63 (1.55 to 10.86)
Guangxi	Measles	93.8 (33.3 to 211)	215.7 (76.55 to 486.28)	6.62 (2.2 to 15)	13.69 (4.56 to 30.96)
Guizhou	Measles	241 (84.8 to 531)	725.93 (255.48 to 1601.05)	10.8 (3.76 to 23.5)	32.04 (11.13 to 69.45)
Hainan	Measles	11.7 (4.14 to 27.2)	167.9 (59.42 to 391.12)	1.29 (0.445 to 2.86)	13.45 (4.64 to 29.78)
Hebei	Measles	81.5 (29.3 to 177)	128.01 (45.98 to 278.46)	5.77 (1.97 to 13.1)	7.55 (2.58 to 17.11)
Heilongjiang	Measles	43.7 (15.7 to 95.6)	119.86 (43.06 to 262.26)	1.97 (0.641 to 4.59)	5.02 (1.63 to 11.7)
Henan	Measles	181 (64.7 to 404)	204.64 (73.27 to 457.58)	7.28 (2.44 to 16.3)	7.56 (2.53 to 16.92)
Hubei	Measles	94.9 (34 to 209)	170.85 (61.17 to 375.81)	3.63 (1.15 to 8.79)	6.57 (2.08 to 15.9)
Hunan	Measles	120 (41.9 to 267)	192.64 (67.32 to 429.86)	5.53 (1.83 to 12.2)	8.24 (2.73 to 18.24)
Inner Mongolia	Measles	42.7 (15 to 93.4)	187.94 (66.03 to 410.63)	1.36 (0.462 to 3.12)	5.27 (1.78 to 12.07)
Jiangsu	Measles	57.1 (20.8 to 130)	82.93 (30.2 to 188.5)	2.16 (0.708 to 5.09)	2.64 (0.87 to 6.23)
Jiangxi	Measles	155 (51.8 to 348)	392.2 (130.93 to 881.29)	6.65 (2.19 to 14.9)	13.98 (4.61 to 31.4)
Jilin	Measles	36.6 (12.7 to 82.7)	140.93 (48.87 to 318.68)	1.33 (0.443 to 3.04)	4.76 (1.59 to 10.92)
Liaoning	Measles	19.8 (6.9 to 42.7)	48.55 (16.91 to 104.79)	0.855 (0.279 to 1.91)	1.91 (0.63 to 4.27)

Ningxia	Measles	20<br>(6.9 to 47.5)	405.58<br>(139.73 to 962.72)	0.9<br>(0.296 to 2.06)	12.83<br>(4.22 to 29.42)
Qinghai	Measles	36<br>(11.9 to 81.4)	760.61<br>(250.42 to 1718.23)	2.04<br>(0.674 to 4.53)	32.53<br>(10.72 to 72.04)
Shaanxi	Measles	86.3<br>(30.1 to 191)	250.8<br>(87.35 to 553.74)	3.63<br>(1.2 to 8.48)	9.19<br>(3.03 to 21.46)
Shandong	Measles	82.2<br>(29.2 to 181)	96.04<br>(34.1 to 211.74)	3.47<br>(1.08 to 7.84)	3.49<br>(1.09 to 7.88)
Shanghai	Measles	2.59<br>(0.918 to 5.77)	18.51<br>(6.57 to 41.33)	0.41<br>(0.13 to 0.987)	1.47<br>(0.46 to 3.53)
Shanxi	Measles	45.2<br>(16.3 to 100)	149.21<br>(53.87 to 331.82)	2.6<br>(0.877 to 5.8)	6.72<br>(2.27 to 15.02)
Sichuan	Measles	268<br>(92.5 to 608)	239.9<br>(82.9 to 544.98)	5.71<br>(1.78 to 12.8)	6.84<br>(2.14 to 15.32)
Tianjin	Measles	3.14<br>(1.11 to 7.14)	34.17<br>(12.1 to 77.66)	0.318<br>(0.1 to 0.725)	2.08<br>(0.66 to 4.75)
Tibet	Measles	29.8<br>(10.2 to 67.3)	1277.8<br>(438.88 to 2889.64)	6.23<br>(2.09 to 14)	180.27<br>(60.5 to 405.7)
Xinjiang	Measles	61.1<br>(21.2 to 135)	387.62<br>(134.46 to 853.45)	4.73<br>(1.6 to 10.4)	19.42<br>(6.57 to 42.88)
Yunnan	Measles	235<br>(81.5 to 537)	603.02<br>(209.03 to 1377.25)	12.4<br>(4.06 to 29.2)	25.16<br>(8.26 to 59.38)
Zhejiang	Measles	39.7<br>(14 to 90.2)	90.09<br>(31.74 to 204.54)	1.38<br>(0.432 to 3.24)	2.26<br>(0.71 to 5.3)
China	Varicella and herpes zoster	217<br>(187 to 252)	18.11<br>(15.66 to 21.02)	99<br>(65.4 to 144)	7.01<br>(4.63 to 10.21)
Taiwan	Varicella and herpes zoster	1.67<br>(1.09 to 2.45)	8.18<br>(5.34 to 12.03)	2.2<br>(1.38 to 3.22)	9.32<br>(5.83 to 13.67)
Hong Kong Special Administrative Region of China	Varicella and herpes zoster	0.361<br>(0.246 to 0.51)	6.26<br>(4.26 to 8.85)	0.539<br>(0.339 to 0.816)	7.18<br>(4.51 to 10.87)
Macau Special Administrative Region of China	Varicella and herpes zoster	0.0224<br>(0.016 to 0.0315)	6.32<br>(4.51 to 8.87)	0.0417<br>(0.026 to 0.0638)	6.1<br>(3.81 to 9.34)
Anhui	Varicella and herpes zoster	11.3<br>(7.81 to 16.5)	19.06<br>(13.18 to 27.79)	4.27<br>(2.76 to 6.32)	6.98<br>(4.51 to 10.33)
Beijing	Varicella and herpes zoster	0.903<br>(0.66 to 1.25)	8.05<br>(5.89 to 11.15)	1.47<br>(0.922 to 2.24)	6.09<br>(3.82 to 9.29)
Chongqing	Varicella and herpes zoster	2.84<br>(1.82 to 4.27)	17.99<br>(11.53 to 27.03)	1.94<br>(1.27 to 2.92)	7.24<br>(4.76 to 10.91)
Fujian	Varicella and herpes zoster	4.72<br>(3.15 to 6.94)	14.92<br>(9.94 to 21.93)	2.46<br>(1.58 to 3.67)	6.24<br>(4.02 to 9.32)
Gansu	Varicella and herpes zoster	6.88<br>(4.29 to 10.6)	29.09<br>(18.12 to 44.71)	1.88<br>(1.26 to 2.74)	7.07<br>(4.74 to 10.3)
Guangdong	Varicella and herpes zoster	10.6<br>(7.43 to 14.5)	16.12<br>(11.28 to 22.11)	10.3<br>(6.46 to 15.4)	8.84<br>(5.53 to 13.16)
Guangxi	Varicella and herpes zoster	9.27<br>(6.42 to 13.8)	21.32<br>(14.77 to 31.65)	3.35<br>(2.24 to 4.78)	6.93<br>(4.63 to 9.88)
Guizhou	Varicella and herpes zoster	13.4<br>(8 to 21.3)	40.29<br>(24.1 to 64.05)	2.72<br>(1.92 to 3.79)	8.04<br>(5.69 to 11.2)
Hainan	Varicella and herpes zoster	1.16<br>(0.805 to 1.74)	16.63<br>(11.55 to 25.05)	0.638<br>(0.432 to 0.916)	6.66<br>(4.51 to 9.56)
Hebei	Varicella and herpes zoster	8.6<br>(6.04 to 12.2)	13.51<br>(9.49 to 19.22)	5.03<br>(3.31 to 7.5)	6.58<br>(4.34 to 9.81)
Heilongjiang	Varicella and herpes zoster	5.19<br>(3.59 to 7.17)	14.23<br>(9.84 to 19.67)	2.82<br>(1.8 to 4.18)	7.18<br>(4.59 to 10.65)
Henan	Varicella and herpes zoster	15.8<br>(10.5 to 23.2)	17.9<br>(11.93 to 26.28)	6.24<br>(4.04 to 9.33)	6.48<br>(4.19 to 9.69)
Hubei	Varicella and herpes zoster	9.54<br>(6.28 to 14.4)	17.17<br>(11.31 to 25.91)	3.91<br>(2.58 to 5.84)	7.07<br>(4.66 to 10.55)
Hunan	Varicella and herpes zoster	11.8<br>(7.99 to 17.2)	19<br>(12.85 to 27.69)	4.65<br>(3.06 to 6.89)	6.92<br>(4.56 to 10.27)
Inner Mongolia	Varicella and herpes zoster	4<br>(2.61 to 5.98)	17.58<br>(11.48 to 26.28)	1.73<br>(1.12 to 2.58)	6.7<br>(4.32 to 9.96)
Jiangsu	Varicella and herpes zoster	8.01<br>(5.75 to 11.3)	11.65<br>(8.36 to 16.44)	5.54<br>(3.53 to 8.28)	6.78<br>(4.33 to 10.14)
Jiangxi	Varicella and herpes zoster	9.75<br>(6.29 to 14.7)	24.67<br>(15.92 to 37.08)	3.12<br>(2.06 to 4.5)	6.57<br>(4.34 to 9.47)

Jilin	Varicella and herpes zoster	4.02 (2.7 to 5.66)	15.5 (10.41 to 21.81)	1.99 (1.25 to 2.99)	7.13 (4.49 to 10.71)
Liaoning	Varicella and herpes zoster	4.16 (3.01 to 5.68)	10.19 (7.37 to 13.92)	3.22 (2.06 to 4.83)	7.22 (4.62 to 10.83)
Ningxia	Varicella and herpes zoster	1.12 (0.665 to 1.77)	22.61 (13.47 to 35.92)	0.435 (0.288 to 0.64)	6.2 (4.11 to 9.12)
Qinghai	Varicella and herpes zoster	2.07 (1.2 to 3.44)	43.8 (25.23 to 72.6)	0.466 (0.329 to 0.656)	7.42 (5.23 to 10.44)
Shaanxi	Varicella and herpes zoster	7.25 (4.9 to 10.8)	21.08 (14.24 to 31.28)	2.7 (1.82 to 3.94)	6.84 (4.6 to 9.98)
Shandong	Varicella and herpes zoster	10.5 (7.41 to 14.9)	12.25 (8.66 to 17.38)	6.74 (4.3 to 10)	6.78 (4.32 to 10.06)
Shanghai	Varicella and herpes zoster	1.08 (0.759 to 1.47)	7.76 (5.44 to 10.54)	1.81 (1.14 to 2.72)	6.48 (4.08 to 9.72)
Shanxi	Varicella and herpes zoster	4.44 (3 to 6.3)	14.67 (9.92 to 20.8)	2.49 (1.62 to 3.68)	6.45 (4.2 to 9.54)
Sichuan	Varicella and herpes zoster	22.8 (15.5 to 33.3)	20.43 (13.9 to 29.84)	5.99 (3.89 to 8.8)	7.17 (4.66 to 10.54)
Tianjin	Varicella and herpes zoster	0.769 (0.557 to 1.04)	8.36 (6.06 to 11.35)	0.96 (0.599 to 1.44)	6.29 (3.93 to 9.46)
Tibet	Varicella and herpes zoster	1.15 (0.718 to 1.75)	49.2 (30.81 to 75.21)	0.397 (0.281 to 0.554)	11.49 (8.13 to 16.02)
Xinjiang	Varicella and herpes zoster	5.68 (3.46 to 8.83)	36 (21.95 to 55.97)	1.71 (1.19 to 2.42)	7.03 (4.87 to 9.92)
Yunnan	Varicella and herpes zoster	13.1 (7.9 to 20.4)	33.57 (20.28 to 52.47)	3.51 (2.4 to 4.94)	7.14 (4.89 to 10.04)
Zhejiang	Varicella and herpes zoster	4.56 (3.21 to 6.31)	10.34 (7.29 to 14.31)	3.89 (2.44 to 5.87)	6.35 (3.99 to 9.59)
China	Neglected tropical diseases and malaria	2900 (1840 to 4320)	241.95 (153.78 to 360.69)	1800 (1060 to 3000)	127.46 (75.09 to 212.52)
Taiwan	Neglected tropical diseases and malaria	14.5 (9.29 to 22)	71.17 (45.51 to 107.63)	10.2 (6.25 to 15.6)	43.41 (26.49 to 66.03)
Hong Kong Special Administrative Region of China	Neglected tropical diseases and malaria	6.03 (3.67 to 9.43)	104.7 (63.65 to 163.65)	6.72 (4.31 to 9.75)	89.48 (57.37 to 129.89)
Macau Special Administrative Region of China	Neglected tropical diseases and malaria	0.352 (0.219 to 0.546)	99.32 (61.68 to 153.9)	0.554 (0.342 to 0.812)	81 (50.01 to 118.69)
Anhui	Neglected tropical diseases and malaria	129 (87 to 183)	218.46 (146.91 to 309.68)	47.9 (31.8 to 69.5)	78.3 (51.95 to 113.7)
Beijing	Neglected tropical diseases and malaria	11.5 (6.85 to 18.2)	102.96 (61.03 to 162.18)	15.1 (8.95 to 23.8)	62.61 (37.15 to 98.75)
Chongqing	Neglected tropical diseases and malaria	34.2 (21.2 to 52.1)	216.41 (134.52 to 329.82)	24.1 (13.9 to 40.3)	90.24 (52.04 to 150.77)
Fujian	Neglected tropical diseases and malaria	73.7 (40.8 to 123)	233.04 (128.94 to 389.2)	46.5 (22.9 to 87.2)	117.95 (57.98 to 221.18)
Gansu	Neglected tropical diseases and malaria	101 (35.2 to 382)	428.66 (148.78 to 1617.09)	21.9 (13.7 to 32.3)	82.26 (51.44 to 121.57)
Guangdong	Neglected tropical diseases and malaria	285 (177 to 443)	432.98 (269.2 to 673.12)	417 (258 to 639)	357.17 (221.15 to 547.04)
Guangxi	Neglected tropical diseases and malaria	126 (89.6 to 174)	290.48 (206.1 to 400.48)	81.7 (56 to 112)	169.15 (115.87 to 232.74)
Guizhou	Neglected tropical diseases and malaria	122 (73.2 to 197)	367.39 (220.47 to 593.27)	49.9 (23.9 to 96.2)	147.55 (70.6 to 284.44)
Hainan	Neglected tropical diseases and malaria	16.7 (12.1 to 22.5)	240.14 (174.21 to 323.55)	8.74 (5.9 to 12.4)	91.14 (61.5 to 129.04)
Hebei	Neglected tropical diseases and malaria	101 (65.7 to 151)	159.14 (103.24 to 236.65)	53.2 (33.5 to 76.5)	69.59 (43.89 to 100.13)
Heilongjiang	Neglected tropical diseases and malaria	56.2 (36.1 to 84.1)	154.15 (99.11 to 230.61)	33.9 (21.1 to 51.3)	86.28 (53.63 to 130.7)
Henan	Neglected tropical diseases and malaria	168 (111 to 243)	190.17 (125.91 to 275.74)	71.7 (47.5 to 104)	74.43 (49.35 to 107.68)
Hubei	Neglected tropical diseases and malaria	227 (109 to 422)	408.01 (196.84 to 759.96)	214 (63 to 497)	386.91 (113.91 to 899.34)
Hunan	Neglected tropical diseases and malaria	143 (98.8 to 202)	230.58 (158.82 to 324.69)	65 (44.2 to 92.5)	96.86 (65.79 to 137.7)
Inner Mongolia	Neglected tropical diseases and malaria	22 (13.8 to 34.7)	96.87 (60.63 to 152.6)	5.43 (3.59 to 8.06)	20.98 (13.85 to 31.12)

Jiangsu	Neglected tropical diseases and malaria	113 (74.3 to 167)	164.42 (108 to 243.17)	57.6 (36 to 87.4)	70.57 (44.08 to 107)
Jiangxi	Neglected tropical diseases and malaria	96 (68.9 to 137)	242.82 (174.17 to 345.3)	37 (24.5 to 53.3)	77.74 (51.44 to 112.11)
Jilin	Neglected tropical diseases and malaria	38.3 (24.6 to 57.4)	147.77 (94.83 to 221.34)	20.4 (13 to 30.5)	73.1 (46.74 to 109.18)
Liaoning	Neglected tropical diseases and malaria	53.6 (34.3 to 79.7)	131.44 (84.08 to 195.44)	31.1 (20.6 to 45.5)	69.68 (46.13 to 101.85)
Ningxia	Neglected tropical diseases and malaria	7.61 (5.03 to 11.2)	154.08 (101.88 to 227.56)	2.82 (1.77 to 4.42)	40.21 (25.22 to 62.95)
Qinghai	Neglected tropical diseases and malaria	6.92 (4.35 to 10.8)	146.01 (91.9 to 227.95)	1.68 (1.11 to 2.57)	26.78 (17.65 to 40.85)
Shaanxi	Neglected tropical diseases and malaria	69.1 (45.4 to 98.2)	200.68 (131.88 to 285.43)	29.2 (19.1 to 43.2)	74.01 (48.35 to 109.25)
Shandong	Neglected tropical diseases and malaria	130 (84 to 193)	152.19 (98.09 to 225.24)	72.5 (46 to 107)	72.98 (46.3 to 107.95)
Shanghai	Neglected tropical diseases and malaria	21.5 (12.5 to 34.3)	153.56 (89.61 to 245.66)	23.1 (12.8 to 40.1)	82.57 (45.73 to 143.48)
Shanxi	Neglected tropical diseases and malaria	96.5 (44.7 to 182)	318.73 (147.51 to 600.64)	69.6 (26.2 to 151)	180.23 (67.72 to 390.52)
Sichuan	Neglected tropical diseases and malaria	238 (159 to 347)	213.26 (142.4 to 310.97)	62.7 (41.1 to 92.5)	75.07 (49.26 to 110.83)
Tianjin	Neglected tropical diseases and malaria	10.8 (6.73 to 16.6)	116.97 (73.2 to 180.88)	10.4 (6.35 to 16.1)	67.85 (41.62 to 105.42)
Tibet	Neglected tropical diseases and malaria	6.16 (4.2 to 8.92)	264.25 (180.39 to 382.78)	2.69 (1.89 to 3.72)	77.69 (54.7 to 107.72)
Xinjiang	Neglected tropical diseases and malaria	92.2 (13 to 475)	584.29 (82.51 to 3011.63)	8.83 (4.08 to 22.3)	36.23 (16.76 to 91.62)
Yunnan	Neglected tropical diseases and malaria	106 (76.6 to 145)	272.16 (196.63 to 370.99)	42.7 (29.1 to 59.8)	86.92 (59.2 to 121.54)
Zhejiang	Neglected tropical diseases and malaria	186 (74 to 385)	421.55 (167.74 to 873.58)	164 (48.9 to 388)	268.78 (79.85 to 633.91)
China	Malaria	16.3 (11.7 to 23)	1.36 (0.98 to 1.93)	4.36 (3 to 6.39)	0.31 (0.21 to 0.45)
Taiwan	Malaria	0 (0 to 0)	0 (0 to 0)	0 (0 to 0)	0 (0 to 0)
Hong Kong Special Administrative Region of China	Malaria	0.0221 (0.0149 to 0.0322)	0.38 (0.26 to 0.56)	0 (0 to 0)	0 (0 to 0)
Macau Special Administrative Region of China	Malaria	0.0013 (0.000846 to 0.00187)	0.37 (0.24 to 0.53)	0 (0 to 0)	0 (0 to 0)
Anhui	Malaria	0.845 (0.559 to 1.65)	1.43 (0.94 to 2.78)	0.242 (0.162 to 0.381)	0.39 (0.26 to 0.62)
Beijing	Malaria	0.0412 (0.0273 to 0.0588)	0.37 (0.24 to 0.52)	0 (0 to 0)	0 (0 to 0)
Chongqing	Malaria	0.353 (0.187 to 0.639)	2.24 (1.18 to 4.05)	0.115 (0.0729 to 0.168)	0.43 (0.27 to 0.63)
Fujian	Malaria	0.446 (0.299 to 0.948)	1.41 (0.95 to 3)	0.134 (0.0935 to 0.227)	0.34 (0.24 to 0.58)
Gansu	Malaria	0.496 (0.302 to 0.809)	2.1 (1.28 to 3.42)	0.138 (0.0859 to 0.19)	0.52 (0.32 to 0.71)
Guangdong	Malaria	0.879 (0.587 to 1.98)	1.34 (0.89 to 3.01)	0.339 (0.236 to 0.65)	0.29 (0.2 to 0.56)
Guangxi	Malaria	0.174 (0.114 to 0.25)	0.4 (0.26 to 0.57)	0 (0 to 0)	0 (0 to 0)
Guizhou	Malaria	0.707 (0.455 to 1.35)	2.13 (1.37 to 4.08)	0.186 (0.12 to 0.281)	0.55 (0.35 to 0.83)
Hainan	Malaria	0.157 (0.0748 to 0.348)	2.26 (1.07 to 5)	0.0705 (0.0502 to 0.0909)	0.74 (0.52 to 0.95)
Hebei	Malaria	0.212 (0.143 to 0.301)	0.33 (0.23 to 0.47)	0 (0 to 0)	0 (0 to 0)
Heilongjiang	Malaria	0.155 (0.103 to 0.224)	0.42 (0.28 to 0.62)	0 (0 to 0)	0 (0 to 0)
Henan	Malaria	1.81 (1.02 to 4.35)	2.05 (1.15 to 4.93)	0.407 (0.26 to 0.575)	0.42 (0.27 to 0.6)
Hubei	Malaria	0.831 (0.557 to 1.61)	1.5 (1 to 2.9)	0.224 (0.156 to 0.364)	0.41 (0.28 to 0.66)

Hunan	Malaria	0.262<br>(0.172 to 0.374)	0.42<br>(0.28 to 0.6)	0<br>(0 to 0)	0<br>(0 to 0)
Inner Mongolia	Malaria	0.0944<br>(0.0613 to 0.136)	0.42<br>(0.27 to 0.6)	0<br>(0 to 0)	0<br>(0 to 0)
Jiangsu	Malaria	0.954<br>(0.646 to 1.6)	1.39<br>(0.94 to 2.33)	0.245<br>(0.171 to 0.425)	0.3<br>(0.21 to 0.52)
Jiangxi	Malaria	0.811<br>(0.496 to 1.52)	2.05<br>(1.26 to 3.84)	0.221<br>(0.14 to 0.312)	0.46<br>(0.3 to 0.66)
Jilin	Malaria	0.107<br>(0.0713 to 0.152)	0.41<br>(0.27 to 0.59)	0<br>(0 to 0)	0<br>(0 to 0)
Liaoning	Malaria	0.729<br>(0.443 to 1.44)	1.79<br>(1.09 to 3.53)	0.2<br>(0.126 to 0.3)	0.45<br>(0.28 to 0.67)
Ningxia	Malaria	0.0215<br>(0.0143 to 0.0301)	0.44<br>(0.29 to 0.61)	0<br>(0 to 0)	0<br>(0 to 0)
Qinghai	Malaria	0.0217<br>(0.014 to 0.031)	0.46<br>(0.3 to 0.65)	0<br>(0 to 0)	0<br>(0 to 0)
Shaanxi	Malaria	0.539<br>(0.324 to 1.03)	1.57<br>(0.94 to 2.99)	0.207<br>(0.118 to 0.284)	0.52<br>(0.3 to 0.72)
Shandong	Malaria	1.19<br>(0.815 to 2.15)	1.4<br>(0.95 to 2.51)	0.345<br>(0.241 to 0.595)	0.35<br>(0.24 to 0.6)
Shanghai	Malaria	0.182<br>(0.115 to 0.332)	1.3<br>(0.82 to 2.37)	0.0861<br>(0.0508 to 0.146)	0.31<br>(0.18 to 0.52)
Shanxi	Malaria	0.119<br>(0.0768 to 0.171)	0.39<br>(0.25 to 0.57)	0<br>(0 to 0)	0<br>(0 to 0)
Sichuan	Malaria	1.92<br>(1.19 to 3.76)	1.72<br>(1.06 to 3.37)	0.345<br>(0.224 to 0.516)	0.41<br>(0.27 to 0.62)
Tianjin	Malaria	0.0354<br>(0.0238 to 0.0501)	0.39<br>(0.26 to 0.54)	0<br>(0 to 0)	0<br>(0 to 0)
Tibet	Malaria	0.17<br>(0.0354 to 0.279)	7.28<br>(1.52 to 11.96)	0.0775<br>(0.0166 to 0.116)	2.24<br>(0.48 to 3.35)
Xinjiang	Malaria	0.0714<br>(0.0475 to 0.102)	0.45<br>(0.3 to 0.65)	0<br>(0 to 0)	0<br>(0 to 0)
Yunnan	Malaria	1.39<br>(0.625 to 2.97)	3.56<br>(1.6 to 7.63)	0.604<br>(0.328 to 0.793)	1.23<br>(0.67 to 1.61)
Zhejiang	Malaria	0.579<br>(0.38 to 1.19)	1.31<br>(0.86 to 2.69)	0.174<br>(0.119 to 0.307)	0.28<br>(0.2 to 0.5)
China	Chagas disease	0<br>(0 to 0)	0<br>(0 to 0)	0<br>(0 to 0)	0<br>(0 to 0)
Taiwan	Chagas disease	0<br>(0 to 0)	0<br>(0 to 0)	0<br>(0 to 0)	0<br>(0 to 0)
Hong Kong Special Administrative Region of China	Chagas disease	0<br>(0 to 0)	0<br>(0 to 0)	0<br>(0 to 0)	0<br>(0 to 0)
Macau Special Administrative Region of China	Chagas disease	0<br>(0 to 0)	0<br>(0 to 0)	0<br>(0 to 0)	0<br>(0 to 0)
Anhui	Chagas disease	0<br>(0 to 0)	0<br>(0 to 0)	0<br>(0 to 0)	0<br>(0 to 0)
Beijing	Chagas disease	0<br>(0 to 0)	0<br>(0 to 0)	0<br>(0 to 0)	0<br>(0 to 0)
Chongqing	Chagas disease	0<br>(0 to 0)	0<br>(0 to 0)	0<br>(0 to 0)	0<br>(0 to 0)
Fujian	Chagas disease	0<br>(0 to 0)	0<br>(0 to 0)	0<br>(0 to 0)	0<br>(0 to 0)
Gansu	Chagas disease	0<br>(0 to 0)	0<br>(0 to 0)	0<br>(0 to 0)	0<br>(0 to 0)
Guangdong	Chagas disease	0<br>(0 to 0)	0<br>(0 to 0)	0<br>(0 to 0)	0<br>(0 to 0)
Guangxi	Chagas disease	0<br>(0 to 0)	0<br>(0 to 0)	0<br>(0 to 0)	0<br>(0 to 0)
Guizhou	Chagas disease	0<br>(0 to 0)	0<br>(0 to 0)	0<br>(0 to 0)	0<br>(0 to 0)
Hainan	Chagas disease	0<br>(0 to 0)	0<br>(0 to 0)	0<br>(0 to 0)	0<br>(0 to 0)
Hebei	Chagas disease	0<br>(0 to 0)	0<br>(0 to 0)	0<br>(0 to 0)	0<br>(0 to 0)
Heilongjiang	Chagas disease	0<br>(0 to 0)	0<br>(0 to 0)	0<br>(0 to 0)	0<br>(0 to 0)

Henan	Chagas disease	0<br>(0 to 0)	0<br>(0 to 0)	0<br>(0 to 0)	0<br>(0 to 0)
Hubei	Chagas disease	0<br>(0 to 0)	0<br>(0 to 0)	0<br>(0 to 0)	0<br>(0 to 0)
Hunan	Chagas disease	0<br>(0 to 0)	0<br>(0 to 0)	0<br>(0 to 0)	0<br>(0 to 0)
Inner Mongolia	Chagas disease	0<br>(0 to 0)	0<br>(0 to 0)	0<br>(0 to 0)	0<br>(0 to 0)
Jiangsu	Chagas disease	0<br>(0 to 0)	0<br>(0 to 0)	0<br>(0 to 0)	0<br>(0 to 0)
Jiangxi	Chagas disease	0<br>(0 to 0)	0<br>(0 to 0)	0<br>(0 to 0)	0<br>(0 to 0)
Jilin	Chagas disease	0<br>(0 to 0)	0<br>(0 to 0)	0<br>(0 to 0)	0<br>(0 to 0)
Liaoning	Chagas disease	0<br>(0 to 0)	0<br>(0 to 0)	0<br>(0 to 0)	0<br>(0 to 0)
Ningxia	Chagas disease	0<br>(0 to 0)	0<br>(0 to 0)	0<br>(0 to 0)	0<br>(0 to 0)
Qinghai	Chagas disease	0<br>(0 to 0)	0<br>(0 to 0)	0<br>(0 to 0)	0<br>(0 to 0)
Shaanxi	Chagas disease	0<br>(0 to 0)	0<br>(0 to 0)	0<br>(0 to 0)	0<br>(0 to 0)
Shandong	Chagas disease	0<br>(0 to 0)	0<br>(0 to 0)	0<br>(0 to 0)	0<br>(0 to 0)
Shanghai	Chagas disease	0<br>(0 to 0)	0<br>(0 to 0)	0<br>(0 to 0)	0<br>(0 to 0)
Shanxi	Chagas disease	0<br>(0 to 0)	0<br>(0 to 0)	0<br>(0 to 0)	0<br>(0 to 0)
Sichuan	Chagas disease	0<br>(0 to 0)	0<br>(0 to 0)	0<br>(0 to 0)	0<br>(0 to 0)
Tianjin	Chagas disease	0<br>(0 to 0)	0<br>(0 to 0)	0<br>(0 to 0)	0<br>(0 to 0)
Tibet	Chagas disease	0<br>(0 to 0)	0<br>(0 to 0)	0<br>(0 to 0)	0<br>(0 to 0)
Xinjiang	Chagas disease	0<br>(0 to 0)	0<br>(0 to 0)	0<br>(0 to 0)	0<br>(0 to 0)
Yunnan	Chagas disease	0<br>(0 to 0)	0<br>(0 to 0)	0<br>(0 to 0)	0<br>(0 to 0)
Zhejiang	Chagas disease	0<br>(0 to 0)	0<br>(0 to 0)	0<br>(0 to 0)	0<br>(0 to 0)
China	Leishmaniasis	140<br>(0.246 to 869)	11.66<br>(0.02 to 72.61)	4.48<br>(0.0138 to 26.1)	0.32<br>(0 to 1.85)
Taiwan	Leishmaniasis	0.436<br>(0.168 to 0.829)	2.14<br>(0.82 to 4.07)	9.61e-06<br>(0 to 6.13e-05)	0<br>(0 to 0)
Hong Kong Special Administrative Region of China	Leishmaniasis	0<br>(0 to 0)	0<br>(0 to 0)	0<br>(0 to 0)	0<br>(0 to 0)
Macau Special Administrative Region of China	Leishmaniasis	0<br>(0 to 0)	0<br>(0 to 0)	0<br>(0 to 0)	0<br>(0 to 0)
Anhui	Leishmaniasis	0<br>(0 to 0)	0<br>(0 to 0)	0<br>(0 to 0)	0<br>(0 to 0)
Beijing	Leishmaniasis	0<br>(0 to 0)	0<br>(0 to 0)	0<br>(0 to 0)	0<br>(0 to 0)
Chongqing	Leishmaniasis	0<br>(0 to 0)	0<br>(0 to 0)	0<br>(0 to 0)	0<br>(0 to 0)
Fujian	Leishmaniasis	0<br>(0 to 0)	0<br>(0 to 0)	0<br>(0 to 0)	0<br>(0 to 0)
Gansu	Leishmaniasis	51.4<br>(0.0779 to 329)	217.37<br>(0.33 to 1389.26)	1.32<br>(0.003 to 7.86)	4.97<br>(0.01 to 29.56)
Guangdong	Leishmaniasis	0<br>(0 to 0)	0<br>(0 to 0)	0<br>(0 to 0)	0<br>(0 to 0)
Guangxi	Leishmaniasis	0<br>(0 to 0)	0<br>(0 to 0)	0<br>(0 to 0)	0<br>(0 to 0)
Guizhou	Leishmaniasis	0<br>(0 to 0)	0<br>(0 to 0)	0<br>(0 to 0)	0<br>(0 to 0)
Hainan	Leishmaniasis	0<br>(0 to 0)	0<br>(0 to 0)	0<br>(0 to 0)	0<br>(0 to 0)

Hebei	Leishmaniasis	0<br>(0 to 0)	0<br>(0 to 0)	0<br>(0 to 0)	0<br>(0 to 0)
Heilongjiang	Leishmaniasis	0<br>(0 to 0)	0<br>(0 to 0)	0<br>(0 to 0)	0<br>(0 to 0)
Henan	Leishmaniasis	0<br>(0 to 0)	0<br>(0 to 0)	0<br>(0 to 0)	0<br>(0 to 0)
Hubei	Leishmaniasis	0<br>(0 to 0)	0<br>(0 to 0)	0<br>(0 to 0)	0<br>(0 to 0)
Hunan	Leishmaniasis	0<br>(0 to 0)	0<br>(0 to 0)	0<br>(0 to 0)	0<br>(0 to 0)
Inner Mongolia	Leishmaniasis	0.302<br>(0.000502 to 2.03)	1.33<br>(0 to 8.91)	0.00877<br>(1.95e-05 to 0.0501)	0.03<br>(0 to 0.19)
Jiangsu	Leishmaniasis	0<br>(0 to 0)	0<br>(0 to 0)	0<br>(0 to 0)	0<br>(0 to 0)
Jiangxi	Leishmaniasis	0<br>(0 to 0)	0<br>(0 to 0)	0<br>(0 to 0)	0<br>(0 to 0)
Jilin	Leishmaniasis	0<br>(0 to 0)	0<br>(0 to 0)	0<br>(0 to 0)	0<br>(0 to 0)
Liaoning	Leishmaniasis	0<br>(0 to 0)	0<br>(0 to 0)	0<br>(0 to 0)	0<br>(0 to 0)
Ningxia	Leishmaniasis	0<br>(0 to 0)	0<br>(0 to 0)	0<br>(0 to 0)	0<br>(0 to 0)
Qinghai	Leishmaniasis	0<br>(0 to 0)	0<br>(0 to 0)	0<br>(0 to 0)	0<br>(0 to 0)
Shaanxi	Leishmaniasis	0.508<br>(0.00099 to 3.45)	1.48<br>(0 to 10.01)	0.0163<br>(4.04e-05 to 0.0921)	0.04<br>(0 to 0.23)
Shandong	Leishmaniasis	0<br>(0 to 0)	0<br>(0 to 0)	0<br>(0 to 0)	0<br>(0 to 0)
Shanghai	Leishmaniasis	0<br>(0 to 0)	0<br>(0 to 0)	0<br>(0 to 0)	0<br>(0 to 0)
Shanxi	Leishmaniasis	0.519<br>(0.000978 to 3.53)	1.71<br>(0 to 11.65)	0.0157<br>(3.78e-05 to 0.0882)	0.04<br>(0 to 0.23)
Sichuan	Leishmaniasis	11.8<br>(0.0245 to 77.4)	10.61<br>(0.02 to 69.31)	0.401<br>(0.00104 to 2.25)	0.48<br>(0 to 2.69)
Tianjin	Leishmaniasis	0<br>(0 to 0)	0<br>(0 to 0)	0<br>(0 to 0)	0<br>(0 to 0)
Tibet	Leishmaniasis	0<br>(0 to 0)	0<br>(0 to 0)	0<br>(0 to 0)	0<br>(0 to 0)
Xinjiang	Leishmaniasis	75<br>(0.144 to 461)	475.29<br>(0.92 to 2920.39)	2.71<br>(0.00897 to 16)	11.13<br>(0.04 to 65.75)
Yunnan	Leishmaniasis	0<br>(0 to 0)	0<br>(0 to 0)	0<br>(0 to 0)	0<br>(0 to 0)
Zhejiang	Leishmaniasis	0<br>(0 to 0)	0<br>(0 to 0)	0<br>(0 to 0)	0<br>(0 to 0)
China	African trypanosomiasis	0<br>(0 to 0)	0<br>(0 to 0)	0<br>(0 to 0)	0<br>(0 to 0)
Taiwan	African trypanosomiasis	0<br>(0 to 0)	0<br>(0 to 0)	0<br>(0 to 0)	0<br>(0 to 0)
Hong Kong Special Administrative Region of China	African trypanosomiasis	0<br>(0 to 0)	0<br>(0 to 0)	0<br>(0 to 0)	0<br>(0 to 0)
Macau Special Administrative Region of China	African trypanosomiasis	0<br>(0 to 0)	0<br>(0 to 0)	0<br>(0 to 0)	0<br>(0 to 0)
Anhui	African trypanosomiasis	0<br>(0 to 0)	0<br>(0 to 0)	0<br>(0 to 0)	0<br>(0 to 0)
Beijing	African trypanosomiasis	0<br>(0 to 0)	0<br>(0 to 0)	0<br>(0 to 0)	0<br>(0 to 0)
Chongqing	African trypanosomiasis	0<br>(0 to 0)	0<br>(0 to 0)	0<br>(0 to 0)	0<br>(0 to 0)
Fujian	African trypanosomiasis	0<br>(0 to 0)	0<br>(0 to 0)	0<br>(0 to 0)	0<br>(0 to 0)
Gansu	African trypanosomiasis	0<br>(0 to 0)	0<br>(0 to 0)	0<br>(0 to 0)	0<br>(0 to 0)
Guangdong	African trypanosomiasis	0<br>(0 to 0)	0<br>(0 to 0)	0<br>(0 to 0)	0<br>(0 to 0)
Guangxi	African trypanosomiasis	0<br>(0 to 0)	0<br>(0 to 0)	0<br>(0 to 0)	0<br>(0 to 0)

Guizhou	African trypanosomiasis	0<br>(0 to 0)	0<br>(0 to 0)	0<br>(0 to 0)	0<br>(0 to 0)
Hainan	African trypanosomiasis	0<br>(0 to 0)	0<br>(0 to 0)	0<br>(0 to 0)	0<br>(0 to 0)
Hebei	African trypanosomiasis	0<br>(0 to 0)	0<br>(0 to 0)	0<br>(0 to 0)	0<br>(0 to 0)
Heilongjiang	African trypanosomiasis	0<br>(0 to 0)	0<br>(0 to 0)	0<br>(0 to 0)	0<br>(0 to 0)
Henan	African trypanosomiasis	0<br>(0 to 0)	0<br>(0 to 0)	0<br>(0 to 0)	0<br>(0 to 0)
Hubei	African trypanosomiasis	0<br>(0 to 0)	0<br>(0 to 0)	0<br>(0 to 0)	0<br>(0 to 0)
Hunan	African trypanosomiasis	0<br>(0 to 0)	0<br>(0 to 0)	0<br>(0 to 0)	0<br>(0 to 0)
Inner Mongolia	African trypanosomiasis	0<br>(0 to 0)	0<br>(0 to 0)	0<br>(0 to 0)	0<br>(0 to 0)
Jiangsu	African trypanosomiasis	0<br>(0 to 0)	0<br>(0 to 0)	0<br>(0 to 0)	0<br>(0 to 0)
Jiangxi	African trypanosomiasis	0<br>(0 to 0)	0<br>(0 to 0)	0<br>(0 to 0)	0<br>(0 to 0)
Jilin	African trypanosomiasis	0<br>(0 to 0)	0<br>(0 to 0)	0<br>(0 to 0)	0<br>(0 to 0)
Liaoning	African trypanosomiasis	0<br>(0 to 0)	0<br>(0 to 0)	0<br>(0 to 0)	0<br>(0 to 0)
Ningxia	African trypanosomiasis	0<br>(0 to 0)	0<br>(0 to 0)	0<br>(0 to 0)	0<br>(0 to 0)
Qinghai	African trypanosomiasis	0<br>(0 to 0)	0<br>(0 to 0)	0<br>(0 to 0)	0<br>(0 to 0)
Shaanxi	African trypanosomiasis	0<br>(0 to 0)	0<br>(0 to 0)	0<br>(0 to 0)	0<br>(0 to 0)
Shandong	African trypanosomiasis	0<br>(0 to 0)	0<br>(0 to 0)	0<br>(0 to 0)	0<br>(0 to 0)
Shanghai	African trypanosomiasis	0<br>(0 to 0)	0<br>(0 to 0)	0<br>(0 to 0)	0<br>(0 to 0)
Shanxi	African trypanosomiasis	0<br>(0 to 0)	0<br>(0 to 0)	0<br>(0 to 0)	0<br>(0 to 0)
Sichuan	African trypanosomiasis	0<br>(0 to 0)	0<br>(0 to 0)	0<br>(0 to 0)	0<br>(0 to 0)
Tianjin	African trypanosomiasis	0<br>(0 to 0)	0<br>(0 to 0)	0<br>(0 to 0)	0<br>(0 to 0)
Tibet	African trypanosomiasis	0<br>(0 to 0)	0<br>(0 to 0)	0<br>(0 to 0)	0<br>(0 to 0)
Xinjiang	African trypanosomiasis	0<br>(0 to 0)	0<br>(0 to 0)	0<br>(0 to 0)	0<br>(0 to 0)
Yunnan	African trypanosomiasis	0<br>(0 to 0)	0<br>(0 to 0)	0<br>(0 to 0)	0<br>(0 to 0)
Zhejiang	African trypanosomiasis	0<br>(0 to 0)	0<br>(0 to 0)	0<br>(0 to 0)	0<br>(0 to 0)
China	Schistosomiasis	77.9<br>(64.5 to 99.5)	6.51<br>(5.39 to 8.32)	33.3<br>(20.2 to 54.8)	2.36<br>(1.43 to 3.88)
Taiwan	Schistosomiasis	0<br>(0 to 0)	0<br>(0 to 0)	0<br>(0 to 0)	0<br>(0 to 0)
Hong Kong Special Administrative Region of China	Schistosomiasis	0.00288<br>(0.00129 to 0.00487)	0.05<br>(0.02 to 0.08)	0.002<br>(0.000819 to 0.00356)	0.03<br>(0.01 to 0.05)
Macau Special Administrative Region of China	Schistosomiasis	0.000193<br>(9.26e-05 to 0.000324)	0.05<br>(0.03 to 0.09)	0.000111<br>(5.17e-05 to 0.000193)	0.02<br>(0.01 to 0.03)
Anhui	Schistosomiasis	9.15<br>(6.97 to 11.8)	15.45<br>(11.77 to 19.85)	2.67<br>(1.81 to 4.15)	4.37<br>(2.96 to 6.78)
Beijing	Schistosomiasis	0<br>(0 to 0)	0<br>(0 to 0)	0<br>(0 to 0)	0<br>(0 to 0)
Chongqing	Schistosomiasis	0<br>(0 to 0)	0<br>(0 to 0)	0<br>(0 to 0)	0<br>(0 to 0)
Fujian	Schistosomiasis	0.246<br>(0.141 to 0.369)	0.78<br>(0.45 to 1.17)	0.123<br>(0.0617 to 0.204)	0.31<br>(0.16 to 0.52)
Gansu	Schistosomiasis	0<br>(0 to 0)	0<br>(0 to 0)	0<br>(0 to 0)	0<br>(0 to 0)

Guangdong	Schistosomiasis	0.11<br>(0.0537 to 0.186)	0.17<br>(0.08 to 0.28)	0.0335<br>(0.0152 to 0.0581)	0.03<br>(0.01 to 0.05)
Guangxi	Schistosomiasis	0.113<br>(0.0576 to 0.191)	0.26<br>(0.13 to 0.44)	0.0305<br>(0.0139 to 0.0502)	0.06<br>(0.03 to 0.1)
Guizhou	Schistosomiasis	0<br>(0 to 0)	0<br>(0 to 0)	0<br>(0 to 0)	0<br>(0 to 0)
Hainan	Schistosomiasis	0<br>(0 to 0)	0<br>(0 to 0)	0<br>(0 to 0)	0<br>(0 to 0)
Hebei	Schistosomiasis	0<br>(0 to 0)	0<br>(0 to 0)	0<br>(0 to 0)	0<br>(0 to 0)
Heilongjiang	Schistosomiasis	0<br>(0 to 0)	0<br>(0 to 0)	0<br>(0 to 0)	0<br>(0 to 0)
Henan	Schistosomiasis	0<br>(0 to 0)	0<br>(0 to 0)	0<br>(0 to 0)	0<br>(0 to 0)
Hubei	Schistosomiasis	10.8<br>(7.91 to 15.7)	19.52<br>(14.23 to 28.19)	8.16<br>(4.4 to 14.4)	14.75<br>(7.95 to 25.98)
Hunan	Schistosomiasis	25.3<br>(17.7 to 37.7)	40.73<br>(28.41 to 60.56)	15.7<br>(8.29 to 28)	23.41<br>(12.35 to 41.68)
Inner Mongolia	Schistosomiasis	0<br>(0 to 0)	0<br>(0 to 0)	0<br>(0 to 0)	0<br>(0 to 0)
Jiangsu	Schistosomiasis	2.29<br>(1.73 to 2.94)	3.33<br>(2.51 to 4.27)	0.541<br>(0.357 to 0.815)	0.66<br>(0.44 to 1)
Jiangxi	Schistosomiasis	10.5<br>(8.19 to 13.1)	26.45<br>(20.73 to 33.16)	2.01<br>(1.44 to 2.87)	4.23<br>(3.02 to 6.04)
Jilin	Schistosomiasis	0<br>(0 to 0)	0<br>(0 to 0)	0<br>(0 to 0)	0<br>(0 to 0)
Liaoning	Schistosomiasis	0<br>(0 to 0)	0<br>(0 to 0)	0<br>(0 to 0)	0<br>(0 to 0)
Ningxia	Schistosomiasis	0<br>(0 to 0)	0<br>(0 to 0)	0<br>(0 to 0)	0<br>(0 to 0)
Qinghai	Schistosomiasis	0<br>(0 to 0)	0<br>(0 to 0)	0<br>(0 to 0)	0<br>(0 to 0)
Shaanxi	Schistosomiasis	0<br>(0 to 0)	0<br>(0 to 0)	0<br>(0 to 0)	0<br>(0 to 0)
Shandong	Schistosomiasis	0<br>(0 to 0)	0<br>(0 to 0)	0<br>(0 to 0)	0<br>(0 to 0)
Shanghai	Schistosomiasis	0.0109<br>(0.00534 to 0.0174)	0.08<br>(0.04 to 0.12)	0.00491<br>(0.00228 to 0.00855)	0.02<br>(0.01 to 0.03)
Shanxi	Schistosomiasis	0<br>(0 to 0)	0<br>(0 to 0)	0<br>(0 to 0)	0<br>(0 to 0)
Sichuan	Schistosomiasis	11.5<br>(8.79 to 14.9)	10.33<br>(7.87 to 13.33)	2.57<br>(1.72 to 3.85)	3.08<br>(2.06 to 4.61)
Tianjin	Schistosomiasis	0<br>(0 to 0)	0<br>(0 to 0)	0<br>(0 to 0)	0<br>(0 to 0)
Tibet	Schistosomiasis	0<br>(0 to 0)	0<br>(0 to 0)	0<br>(0 to 0)	0<br>(0 to 0)
Xinjiang	Schistosomiasis	0<br>(0 to 0)	0<br>(0 to 0)	0<br>(0 to 0)	0<br>(0 to 0)
Yunnan	Schistosomiasis	7.57<br>(5.83 to 9.95)	19.43<br>(14.95 to 25.53)	1.39<br>(1.02 to 1.88)	2.83<br>(2.07 to 3.82)
Zhejiang	Schistosomiasis	0.286<br>(0.161 to 0.43)	0.65<br>(0.36 to 0.97)	0.0774<br>(0.0354 to 0.133)	0.13<br>(0.06 to 0.22)
China	Cysticercosis	182<br>(122 to 252)	15.2<br>(10.17 to 21.09)	145<br>(82.8 to 225)	10.25<br>(5.86 to 15.92)
Taiwan	Cysticercosis	3.68<br>(1.97 to 6.02)	18.04<br>(9.67 to 29.5)	4.62<br>(2.08 to 8.33)	19.58<br>(8.8 to 35.3)
Hong Kong Special Administrative Region of China	Cysticercosis	0.598<br>(0.294 to 1.04)	10.38<br>(5.11 to 18.08)	0.695<br>(0.279 to 1.39)	9.25<br>(3.72 to 18.52)
Macau Special Administrative Region of China	Cysticercosis	0.0327<br>(0.0162 to 0.0576)	9.22<br>(4.56 to 16.23)	0.055<br>(0.0226 to 0.11)	8.05<br>(3.31 to 16.14)
Anhui	Cysticercosis	6.23<br>(3.51 to 10.1)	10.51<br>(5.92 to 17.09)	4.64<br>(2.1 to 8.33)	7.58<br>(3.44 to 13.63)
Beijing	Cysticercosis	1.32<br>(0.625 to 2.25)	11.75<br>(5.57 to 20.05)	1.93<br>(0.771 to 3.79)	8.02<br>(3.2 to 15.72)
Chongqing	Cysticercosis	2.83<br>(1.55 to 4.5)	17.95<br>(9.82 to 28.52)	3.38<br>(1.56 to 6.03)	12.66<br>(5.85 to 22.56)

Fujian	Cysticercosis	3.99<br>(2.18 to 6.64)	12.6<br>(6.9 to 21)	3.54<br>(1.5 to 6.82)	8.99<br>(3.79 to 17.32)
Gansu	Cysticercosis	4.42<br>(2.45 to 6.99)	18.68<br>(10.35 to 29.55)	2.53<br>(1.19 to 4.6)	9.54<br>(4.47 to 17.32)
Guangdong	Cysticercosis	6.24<br>(3.3 to 10.9)	9.48<br>(5.02 to 16.55)	7.77<br>(3.22 to 16)	6.65<br>(2.75 to 13.66)
Guangxi	Cysticercosis	6.63<br>(3.76 to 10.8)	15.26<br>(8.65 to 24.93)	4.79<br>(2.21 to 8.52)	9.9<br>(4.57 to 17.64)
Guizhou	Cysticercosis	7.71<br>(4.66 to 11.6)	23.24<br>(14.05 to 34.84)	3.99<br>(2.1 to 6.68)	11.81<br>(6.2 to 19.75)
Hainan	Cysticercosis	0.932<br>(0.506 to 1.57)	13.38<br>(7.26 to 22.56)	0.769<br>(0.349 to 1.41)	8.02<br>(3.64 to 14.69)
Hebei	Cysticercosis	9.26<br>(5.3 to 14.7)	14.54<br>(8.33 to 23.12)	9.21<br>(4.16 to 16.2)	12.05<br>(5.44 to 21.22)
Heilongjiang	Cysticercosis	7.11<br>(3.93 to 11.4)	19.49<br>(10.78 to 31.4)	6.62<br>(2.97 to 12.1)	16.87<br>(7.58 to 30.9)
Henan	Cysticercosis	12.3<br>(6.59 to 19.8)	13.91<br>(7.47 to 22.4)	8.49<br>(3.85 to 15.7)	8.82<br>(4 to 16.32)
Hubei	Cysticercosis	6.96<br>(3.77 to 11.3)	12.53<br>(6.78 to 20.29)	5.13<br>(2.26 to 9.42)	9.27<br>(4.09 to 17.03)
Hunan	Cysticercosis	11.6<br>(6.34 to 19)	18.72<br>(10.19 to 30.55)	7.44<br>(3.26 to 14)	11.07<br>(4.85 to 20.92)
Inner Mongolia	Cysticercosis	3.13<br>(1.79 to 5.07)	13.74<br>(7.86 to 22.3)	2.37<br>(1.08 to 4.56)	9.17<br>(4.19 to 17.59)
Jiangsu	Cysticercosis	12.6<br>(6.7 to 20.2)	18.28<br>(9.73 to 29.39)	11.2<br>(4.75 to 21.2)	13.67<br>(5.82 to 25.96)
Jiangxi	Cysticercosis	7.39<br>(4.29 to 11.4)	18.7<br>(10.84 to 28.75)	4.73<br>(2.19 to 9.08)	9.96<br>(4.6 to 19.11)
Jilin	Cysticercosis	3.97<br>(2.13 to 6.74)	15.28<br>(8.21 to 25.97)	3.72<br>(1.69 to 6.86)	13.35<br>(6.07 to 24.59)
Liaoning	Cysticercosis	5.07<br>(2.63 to 8.55)	12.42<br>(6.44 to 20.96)	5.64<br>(2.45 to 10.5)	12.63<br>(5.49 to 23.54)
Ningxia	Cysticercosis	1.33<br>(0.781 to 2.09)	26.88<br>(15.81 to 42.41)	1.56<br>(0.698 to 2.83)	22.23<br>(9.94 to 40.34)
Qinghai	Cysticercosis	0.827<br>(0.474 to 1.26)	17.46<br>(10 to 26.58)	0.43<br>(0.207 to 0.758)	6.84<br>(3.29 to 12.07)
Shaanxi	Cysticercosis	2.77<br>(1.57 to 4.3)	8.05<br>(4.56 to 12.51)	2.33<br>(1.09 to 4.15)	5.89<br>(2.76 to 10.51)
Shandong	Cysticercosis	11<br>(5.95 to 18.1)	12.87<br>(6.95 to 21.14)	9.67<br>(4.21 to 18)	9.73<br>(4.23 to 18.08)
Shanghai	Cysticercosis	1.77<br>(0.901 to 3.04)	12.68<br>(6.45 to 21.75)	2.31<br>(0.941 to 4.39)	8.26<br>(3.37 to 15.72)
Shanxi	Cysticercosis	4.17<br>(2.24 to 6.8)	13.78<br>(7.4 to 22.46)	3.64<br>(1.71 to 6.72)	9.43<br>(4.43 to 17.4)
Sichuan	Cysticercosis	19.8<br>(11.1 to 30.6)	17.7<br>(9.98 to 27.42)	10.3<br>(4.78 to 18.6)	12.34<br>(5.72 to 22.31)
Tianjin	Cysticercosis	1.24<br>(0.64 to 2.11)	13.5<br>(6.96 to 22.93)	1.47<br>(0.603 to 2.78)	9.63<br>(3.95 to 18.21)
Tibet	Cysticercosis	1.15<br>(0.698 to 1.71)	49.45<br>(29.95 to 73.38)	1.24<br>(0.736 to 1.93)	35.84<br>(21.29 to 55.82)
Xinjiang	Cysticercosis	2.49<br>(1.47 to 3.77)	15.78<br>(9.31 to 23.93)	2.39<br>(1.09 to 4.21)	9.81<br>(4.47 to 17.29)
Yunnan	Cysticercosis	9.94<br>(5.99 to 15.3)	25.51<br>(15.36 to 39.21)	5.46<br>(2.61 to 9.73)	11.11<br>(5.32 to 19.79)
Zhejiang	Cysticercosis	5.24<br>(2.57 to 8.87)	11.89<br>(5.84 to 20.12)	5.32<br>(2.14 to 10.2)	8.7<br>(3.5 to 16.74)
China	Cystic echinococcosis	2.69<br>(2.13 to 3.36)	0.22<br>(0.18 to 0.28)	1.18<br>(0.716 to 1.91)	0.08<br>(0.05 to 0.14)
Taiwan	Cystic echinococcosis	0.0124<br>(0.00713 to 0.0184)	0.06<br>(0.03 to 0.09)	0.00374<br>(0.00202 to 0.00565)	0.02<br>(0.01 to 0.02)
Hong Kong Special Administrative Region of China	Cystic echinococcosis	0<br>(0 to 0)	0<br>(0 to 0)	0<br>(0 to 0)	0<br>(0 to 0)
Macau Special Administrative Region of China	Cystic echinococcosis	0<br>(0 to 0)	0<br>(0 to 0)	0<br>(0 to 0)	0<br>(0 to 0)
Anhui	Cystic echinococcosis	0<br>(0 to 0)	0<br>(0 to 0)	0<br>(0 to 0)	0<br>(0 to 0)

Beijing	Cystic echinococcosis	0<br>(0 to 0)	0<br>(0 to 0)	0<br>(0 to 0)	0<br>(0 to 0)
Chongqing	Cystic echinococcosis	0<br>(0 to 0)	0<br>(0 to 0)	0<br>(0 to 0)	0<br>(0 to 0)
Fujian	Cystic echinococcosis	0<br>(0 to 0)	0<br>(0 to 0)	0<br>(0 to 0)	0<br>(0 to 0)
Gansu	Cystic echinococcosis	0.869<br>(0.553 to 1.2)	3.67<br>(2.34 to 5.09)	0.202<br>(0.136 to 0.281)	0.76<br>(0.51 to 1.06)
Guangdong	Cystic echinococcosis	0<br>(0 to 0)	0<br>(0 to 0)	0<br>(0 to 0)	0<br>(0 to 0)
Guangxi	Cystic echinococcosis	0<br>(0 to 0)	0<br>(0 to 0)	0<br>(0 to 0)	0<br>(0 to 0)
Guizhou	Cystic echinococcosis	0<br>(0 to 0)	0<br>(0 to 0)	0<br>(0 to 0)	0<br>(0 to 0)
Hainan	Cystic echinococcosis	0<br>(0 to 0)	0<br>(0 to 0)	0<br>(0 to 0)	0<br>(0 to 0)
Hebei	Cystic echinococcosis	0<br>(0 to 0)	0<br>(0 to 0)	0<br>(0 to 0)	0<br>(0 to 0)
Heilongjiang	Cystic echinococcosis	0.204<br>(0.124 to 0.293)	0.56<br>(0.34 to 0.8)	0.0693<br>(0.0406 to 0.108)	0.18<br>(0.1 to 0.27)
Henan	Cystic echinococcosis	0<br>(0 to 0)	0<br>(0 to 0)	0<br>(0 to 0)	0<br>(0 to 0)
Hubei	Cystic echinococcosis	0<br>(0 to 0)	0<br>(0 to 0)	0<br>(0 to 0)	0<br>(0 to 0)
Hunan	Cystic echinococcosis	0<br>(0 to 0)	0<br>(0 to 0)	0<br>(0 to 0)	0<br>(0 to 0)
Inner Mongolia	Cystic echinococcosis	0.164<br>(0.0968 to 0.243)	0.72<br>(0.43 to 1.07)	0.0551<br>(0.0326 to 0.0856)	0.21<br>(0.13 to 0.33)
Jiangsu	Cystic echinococcosis	0<br>(0 to 0)	0<br>(0 to 0)	0<br>(0 to 0)	0<br>(0 to 0)
Jiangxi	Cystic echinococcosis	0<br>(0 to 0)	0<br>(0 to 0)	0<br>(0 to 0)	0<br>(0 to 0)
Jilin	Cystic echinococcosis	0<br>(0 to 0)	0<br>(0 to 0)	0<br>(0 to 0)	0<br>(0 to 0)
Liaoning	Cystic echinococcosis	0<br>(0 to 0)	0<br>(0 to 0)	0<br>(0 to 0)	0<br>(0 to 0)
Ningxia	Cystic echinococcosis	0.152<br>(0.0731 to 0.256)	3.08<br>(1.48 to 5.19)	0.0564<br>(0.036 to 0.0842)	0.8<br>(0.51 to 1.2)
Qinghai	Cystic echinococcosis	0.112<br>(0.0607 to 0.195)	2.36<br>(1.28 to 4.11)	0.0993<br>(0.047 to 0.19)	1.58<br>(0.75 to 3.02)
Shaanxi	Cystic echinococcosis	0<br>(0 to 0)	0<br>(0 to 0)	0<br>(0 to 0)	0<br>(0 to 0)
Shandong	Cystic echinococcosis	0<br>(0 to 0)	0<br>(0 to 0)	0<br>(0 to 0)	0<br>(0 to 0)
Shanghai	Cystic echinococcosis	0<br>(0 to 0)	0<br>(0 to 0)	0<br>(0 to 0)	0<br>(0 to 0)
Shanxi	Cystic echinococcosis	0<br>(0 to 0)	0<br>(0 to 0)	0<br>(0 to 0)	0<br>(0 to 0)
Sichuan	Cystic echinococcosis	0.82<br>(0.578 to 1.09)	0.73<br>(0.52 to 0.98)	0.265<br>(0.172 to 0.381)	0.32<br>(0.21 to 0.46)
Tianjin	Cystic echinococcosis	0<br>(0 to 0)	0<br>(0 to 0)	0<br>(0 to 0)	0<br>(0 to 0)
Tibet	Cystic echinococcosis	0.0758<br>(0.0392 to 0.12)	3.25<br>(1.68 to 5.17)	0.0326<br>(0.0184 to 0.0542)	0.94<br>(0.53 to 1.57)
Xinjiang	Cystic echinococcosis	0.288<br>(0.146 to 0.54)	1.83<br>(0.93 to 3.42)	0.397<br>(0.172 to 0.807)	1.63<br>(0.71 to 3.31)
Yunnan	Cystic echinococcosis	0<br>(0 to 0)	0<br>(0 to 0)	0<br>(0 to 0)	0<br>(0 to 0)
Zhejiang	Cystic echinococcosis	0<br>(0 to 0)	0<br>(0 to 0)	0<br>(0 to 0)	0<br>(0 to 0)
China	Lymphatic filariasis	0<br>(0 to 0)	0<br>(0 to 0)	0<br>(0 to 0)	0<br>(0 to 0)
Taiwan	Lymphatic filariasis	0<br>(0 to 0)	0<br>(0 to 0)	0<br>(0 to 0)	0<br>(0 to 0)
Hong Kong Special Administrative Region of China	Lymphatic filariasis	0<br>(0 to 0)	0<br>(0 to 0)	0<br>(0 to 0)	0<br>(0 to 0)

Macau Special Administrative Region of China	Lymphatic filariasis	0<br>(0 to 0)	0<br>(0 to 0)	0<br>(0 to 0)	0<br>(0 to 0)
Anhui	Lymphatic filariasis	0<br>(0 to 0)	0<br>(0 to 0)	0<br>(0 to 0)	0<br>(0 to 0)
Beijing	Lymphatic filariasis	0<br>(0 to 0)	0<br>(0 to 0)	0<br>(0 to 0)	0<br>(0 to 0)
Chongqing	Lymphatic filariasis	0<br>(0 to 0)	0<br>(0 to 0)	0<br>(0 to 0)	0<br>(0 to 0)
Fujian	Lymphatic filariasis	0<br>(0 to 0)	0<br>(0 to 0)	0<br>(0 to 0)	0<br>(0 to 0)
Gansu	Lymphatic filariasis	0<br>(0 to 0)	0<br>(0 to 0)	0<br>(0 to 0)	0<br>(0 to 0)
Guangdong	Lymphatic filariasis	0<br>(0 to 0)	0<br>(0 to 0)	0<br>(0 to 0)	0<br>(0 to 0)
Guangxi	Lymphatic filariasis	0<br>(0 to 0)	0<br>(0 to 0)	0<br>(0 to 0)	0<br>(0 to 0)
Guizhou	Lymphatic filariasis	0<br>(0 to 0)	0<br>(0 to 0)	0<br>(0 to 0)	0<br>(0 to 0)
Hainan	Lymphatic filariasis	0<br>(0 to 0)	0<br>(0 to 0)	0<br>(0 to 0)	0<br>(0 to 0)
Hebei	Lymphatic filariasis	0<br>(0 to 0)	0<br>(0 to 0)	0<br>(0 to 0)	0<br>(0 to 0)
Heilongjiang	Lymphatic filariasis	0<br>(0 to 0)	0<br>(0 to 0)	0<br>(0 to 0)	0<br>(0 to 0)
Henan	Lymphatic filariasis	0<br>(0 to 0)	0<br>(0 to 0)	0<br>(0 to 0)	0<br>(0 to 0)
Hubei	Lymphatic filariasis	0<br>(0 to 0)	0<br>(0 to 0)	0<br>(0 to 0)	0<br>(0 to 0)
Hunan	Lymphatic filariasis	0<br>(0 to 0)	0<br>(0 to 0)	0<br>(0 to 0)	0<br>(0 to 0)
Inner Mongolia	Lymphatic filariasis	0<br>(0 to 0)	0<br>(0 to 0)	0<br>(0 to 0)	0<br>(0 to 0)
Jiangsu	Lymphatic filariasis	0<br>(0 to 0)	0<br>(0 to 0)	0<br>(0 to 0)	0<br>(0 to 0)
Jiangxi	Lymphatic filariasis	0<br>(0 to 0)	0<br>(0 to 0)	0<br>(0 to 0)	0<br>(0 to 0)
Jilin	Lymphatic filariasis	0<br>(0 to 0)	0<br>(0 to 0)	0<br>(0 to 0)	0<br>(0 to 0)
Liaoning	Lymphatic filariasis	0<br>(0 to 0)	0<br>(0 to 0)	0<br>(0 to 0)	0<br>(0 to 0)
Ningxia	Lymphatic filariasis	0<br>(0 to 0)	0<br>(0 to 0)	0<br>(0 to 0)	0<br>(0 to 0)
Qinghai	Lymphatic filariasis	0<br>(0 to 0)	0<br>(0 to 0)	0<br>(0 to 0)	0<br>(0 to 0)
Shaanxi	Lymphatic filariasis	0<br>(0 to 0)	0<br>(0 to 0)	0<br>(0 to 0)	0<br>(0 to 0)
Shandong	Lymphatic filariasis	0<br>(0 to 0)	0<br>(0 to 0)	0<br>(0 to 0)	0<br>(0 to 0)
Shanghai	Lymphatic filariasis	0<br>(0 to 0)	0<br>(0 to 0)	0<br>(0 to 0)	0<br>(0 to 0)
Shanxi	Lymphatic filariasis	0<br>(0 to 0)	0<br>(0 to 0)	0<br>(0 to 0)	0<br>(0 to 0)
Sichuan	Lymphatic filariasis	0<br>(0 to 0)	0<br>(0 to 0)	0<br>(0 to 0)	0<br>(0 to 0)
Tianjin	Lymphatic filariasis	0<br>(0 to 0)	0<br>(0 to 0)	0<br>(0 to 0)	0<br>(0 to 0)
Tibet	Lymphatic filariasis	0<br>(0 to 0)	0<br>(0 to 0)	0<br>(0 to 0)	0<br>(0 to 0)
Xinjiang	Lymphatic filariasis	0<br>(0 to 0)	0<br>(0 to 0)	0<br>(0 to 0)	0<br>(0 to 0)
Yunnan	Lymphatic filariasis	0<br>(0 to 0)	0<br>(0 to 0)	0<br>(0 to 0)	0<br>(0 to 0)
Zhejiang	Lymphatic filariasis	0<br>(0 to 0)	0<br>(0 to 0)	0<br>(0 to 0)	0<br>(0 to 0)
China	Onchocerciasis	0<br>(0 to 0)	0<br>(0 to 0)	0<br>(0 to 0)	0<br>(0 to 0)
Taiwan	Onchocerciasis	0<br>(0 to 0)	0<br>(0 to 0)	0<br>(0 to 0)	0<br>(0 to 0)

Hong Kong Special Administrative Region of China	Onchocerciasis	0<br>(0 to 0)	0<br>(0 to 0)	0<br>(0 to 0)	0<br>(0 to 0)
Macau Special Administrative Region of China	Onchocerciasis	0<br>(0 to 0)	0<br>(0 to 0)	0<br>(0 to 0)	0<br>(0 to 0)
Anhui	Onchocerciasis	0<br>(0 to 0)	0<br>(0 to 0)	0<br>(0 to 0)	0<br>(0 to 0)
Beijing	Onchocerciasis	0<br>(0 to 0)	0<br>(0 to 0)	0<br>(0 to 0)	0<br>(0 to 0)
Chongqing	Onchocerciasis	0<br>(0 to 0)	0<br>(0 to 0)	0<br>(0 to 0)	0<br>(0 to 0)
Fujian	Onchocerciasis	0<br>(0 to 0)	0<br>(0 to 0)	0<br>(0 to 0)	0<br>(0 to 0)
Gansu	Onchocerciasis	0<br>(0 to 0)	0<br>(0 to 0)	0<br>(0 to 0)	0<br>(0 to 0)
Guangdong	Onchocerciasis	0<br>(0 to 0)	0<br>(0 to 0)	0<br>(0 to 0)	0<br>(0 to 0)
Guangxi	Onchocerciasis	0<br>(0 to 0)	0<br>(0 to 0)	0<br>(0 to 0)	0<br>(0 to 0)
Guizhou	Onchocerciasis	0<br>(0 to 0)	0<br>(0 to 0)	0<br>(0 to 0)	0<br>(0 to 0)
Hainan	Onchocerciasis	0<br>(0 to 0)	0<br>(0 to 0)	0<br>(0 to 0)	0<br>(0 to 0)
Hebei	Onchocerciasis	0<br>(0 to 0)	0<br>(0 to 0)	0<br>(0 to 0)	0<br>(0 to 0)
Heilongjiang	Onchocerciasis	0<br>(0 to 0)	0<br>(0 to 0)	0<br>(0 to 0)	0<br>(0 to 0)
Henan	Onchocerciasis	0<br>(0 to 0)	0<br>(0 to 0)	0<br>(0 to 0)	0<br>(0 to 0)
Hubei	Onchocerciasis	0<br>(0 to 0)	0<br>(0 to 0)	0<br>(0 to 0)	0<br>(0 to 0)
Hunan	Onchocerciasis	0<br>(0 to 0)	0<br>(0 to 0)	0<br>(0 to 0)	0<br>(0 to 0)
Inner Mongolia	Onchocerciasis	0<br>(0 to 0)	0<br>(0 to 0)	0<br>(0 to 0)	0<br>(0 to 0)
Jiangsu	Onchocerciasis	0<br>(0 to 0)	0<br>(0 to 0)	0<br>(0 to 0)	0<br>(0 to 0)
Jiangxi	Onchocerciasis	0<br>(0 to 0)	0<br>(0 to 0)	0<br>(0 to 0)	0<br>(0 to 0)
Jilin	Onchocerciasis	0<br>(0 to 0)	0<br>(0 to 0)	0<br>(0 to 0)	0<br>(0 to 0)
Liaoning	Onchocerciasis	0<br>(0 to 0)	0<br>(0 to 0)	0<br>(0 to 0)	0<br>(0 to 0)
Ningxia	Onchocerciasis	0<br>(0 to 0)	0<br>(0 to 0)	0<br>(0 to 0)	0<br>(0 to 0)
Qinghai	Onchocerciasis	0<br>(0 to 0)	0<br>(0 to 0)	0<br>(0 to 0)	0<br>(0 to 0)
Shaanxi	Onchocerciasis	0<br>(0 to 0)	0<br>(0 to 0)	0<br>(0 to 0)	0<br>(0 to 0)
Shandong	Onchocerciasis	0<br>(0 to 0)	0<br>(0 to 0)	0<br>(0 to 0)	0<br>(0 to 0)
Shanghai	Onchocerciasis	0<br>(0 to 0)	0<br>(0 to 0)	0<br>(0 to 0)	0<br>(0 to 0)
Shanxi	Onchocerciasis	0<br>(0 to 0)	0<br>(0 to 0)	0<br>(0 to 0)	0<br>(0 to 0)
Sichuan	Onchocerciasis	0<br>(0 to 0)	0<br>(0 to 0)	0<br>(0 to 0)	0<br>(0 to 0)
Tianjin	Onchocerciasis	0<br>(0 to 0)	0<br>(0 to 0)	0<br>(0 to 0)	0<br>(0 to 0)
Tibet	Onchocerciasis	0<br>(0 to 0)	0<br>(0 to 0)	0<br>(0 to 0)	0<br>(0 to 0)
Xinjiang	Onchocerciasis	0<br>(0 to 0)	0<br>(0 to 0)	0<br>(0 to 0)	0<br>(0 to 0)
Yunnan	Onchocerciasis	0<br>(0 to 0)	0<br>(0 to 0)	0<br>(0 to 0)	0<br>(0 to 0)
Zhejiang	Onchocerciasis	0<br>(0 to 0)	0<br>(0 to 0)	0<br>(0 to 0)	0<br>(0 to 0)

China	Trachoma	27.2<br>(18.2 to 37.8)	2.28<br>(1.52 to 3.15)	19.3<br>(12.4 to 27.8)	1.37<br>(0.88 to 1.97)
Taiwan	Trachoma	0<br>(0 to 0)	0<br>(0 to 0)	0<br>(0 to 0)	0<br>(0 to 0)
Hong Kong Special Administrative Region of China	Trachoma	0<br>(0 to 0)	0<br>(0 to 0)	0<br>(0 to 0)	0<br>(0 to 0)
Macau Special Administrative Region of China	Trachoma	0<br>(0 to 0)	0<br>(0 to 0)	0<br>(0 to 0)	0<br>(0 to 0)
Anhui	Trachoma	3.87<br>(2.4 to 5.75)	6.52<br>(4.06 to 9.7)	2.91<br>(1.62 to 4.72)	4.76<br>(2.65 to 7.71)
Beijing	Trachoma	0<br>(0 to 0)	0<br>(0 to 0)	0<br>(0 to 0)	0<br>(0 to 0)
Chongqing	Trachoma	0<br>(0 to 0)	0<br>(0 to 0)	0<br>(0 to 0)	0<br>(0 to 0)
Fujian	Trachoma	1.19<br>(0.705 to 1.84)	3.75<br>(2.23 to 5.82)	0.947<br>(0.496 to 1.54)	2.4<br>(1.26 to 3.9)
Gansu	Trachoma	1.08<br>(0.665 to 1.58)	4.58<br>(2.81 to 6.69)	1.05<br>(0.616 to 1.75)	3.94<br>(2.32 to 6.57)
Guangdong	Trachoma	0<br>(0 to 0)	0<br>(0 to 0)	0<br>(0 to 0)	0<br>(0 to 0)
Guangxi	Trachoma	0<br>(0 to 0)	0<br>(0 to 0)	0<br>(0 to 0)	0<br>(0 to 0)
Guizhou	Trachoma	0<br>(0 to 0)	0<br>(0 to 0)	0<br>(0 to 0)	0<br>(0 to 0)
Hainan	Trachoma	0<br>(0 to 0)	0<br>(0 to 0)	0<br>(0 to 0)	0<br>(0 to 0)
Hebei	Trachoma	2.35<br>(1.44 to 3.72)	3.7<br>(2.26 to 5.85)	1.63<br>(0.926 to 2.72)	2.13<br>(1.21 to 3.56)
Heilongjiang	Trachoma	0<br>(0 to 0)	0<br>(0 to 0)	0<br>(0 to 0)	0<br>(0 to 0)
Henan	Trachoma	7.13<br>(4.49 to 10.6)	8.08<br>(5.08 to 12.06)	4.76<br>(2.72 to 7.59)	4.94<br>(2.83 to 7.88)
Hubei	Trachoma	0<br>(0 to 0)	0<br>(0 to 0)	0<br>(0 to 0)	0<br>(0 to 0)
Hunan	Trachoma	4.62<br>(2.82 to 7.03)	7.43<br>(4.54 to 11.3)	2.76<br>(1.57 to 4.38)	4.11<br>(2.33 to 6.53)
Inner Mongolia	Trachoma	0<br>(0 to 0)	0<br>(0 to 0)	0<br>(0 to 0)	0<br>(0 to 0)
Jiangsu	Trachoma	0<br>(0 to 0)	0<br>(0 to 0)	0<br>(0 to 0)	0<br>(0 to 0)
Jiangxi	Trachoma	0<br>(0 to 0)	0<br>(0 to 0)	0<br>(0 to 0)	0<br>(0 to 0)
Jilin	Trachoma	0<br>(0 to 0)	0<br>(0 to 0)	0<br>(0 to 0)	0<br>(0 to 0)
Liaoning	Trachoma	0<br>(0 to 0)	0<br>(0 to 0)	0<br>(0 to 0)	0<br>(0 to 0)
Ningxia	Trachoma	0<br>(0 to 0)	0<br>(0 to 0)	0<br>(0 to 0)	0<br>(0 to 0)
Qinghai	Trachoma	0<br>(0 to 0)	0<br>(0 to 0)	0<br>(0 to 0)	0<br>(0 to 0)
Shaanxi	Trachoma	0<br>(0 to 0)	0<br>(0 to 0)	0<br>(0 to 0)	0<br>(0 to 0)
Shandong	Trachoma	3.67<br>(2.15 to 5.71)	4.28<br>(2.51 to 6.67)	3.19<br>(1.78 to 5.26)	3.21<br>(1.79 to 5.29)
Shanghai	Trachoma	0<br>(0 to 0)	0<br>(0 to 0)	0<br>(0 to 0)	0<br>(0 to 0)
Shanxi	Trachoma	0<br>(0 to 0)	0<br>(0 to 0)	0<br>(0 to 0)	0<br>(0 to 0)
Sichuan	Trachoma	0<br>(0 to 0)	0<br>(0 to 0)	0<br>(0 to 0)	0<br>(0 to 0)
Tianjin	Trachoma	0<br>(0 to 0)	0<br>(0 to 0)	0<br>(0 to 0)	0<br>(0 to 0)
Tibet	Trachoma	0<br>(0 to 0)	0<br>(0 to 0)	0<br>(0 to 0)	0<br>(0 to 0)
Xinjiang	Trachoma	0<br>(0 to 0)	0<br>(0 to 0)	0<br>(0 to 0)	0<br>(0 to 0)

Yunnan	Trachoma	3.33<br>(2.06 to 5)	8.53<br>(5.28 to 12.83)	2.11<br>(1.2 to 3.36)	4.28<br>(2.45 to 6.84)
Zhejiang	Trachoma	0<br>(0 to 0)	0<br>(0 to 0)	0<br>(0 to 0)	0<br>(0 to 0)
China	Dengue	3.83<br>(0.396 to 9)	0.32<br>(0.03 to 0.75)	25.8<br>(11.6 to 47.7)	1.82<br>(0.82 to 3.38)
Taiwan	Dengue	0.698<br>(0.069 to 1.58)	3.42<br>(0.34 to 7.76)	4.2<br>(2.13 to 7.3)	17.83<br>(9.04 to 30.97)
Hong Kong Special Administrative Region of China	Dengue	0.244<br>(0.0275 to 0.566)	4.24<br>(0.48 to 9.82)	1.54<br>(0.716 to 2.7)	20.45<br>(9.54 to 36.01)
Macau Special Administrative Region of China	Dengue	0.0113<br>(0.000386 to 0.0267)	3.18<br>(0.11 to 7.52)	0.11<br>(0.0526 to 0.196)	16.04<br>(7.69 to 28.62)
Anhui	Dengue	0<br>(0 to 0)	0<br>(0 to 0)	0<br>(0 to 0)	0<br>(0 to 0)
Beijing	Dengue	0<br>(0 to 0)	0<br>(0 to 0)	0<br>(0 to 0)	0<br>(0 to 0)
Chongqing	Dengue	0<br>(0 to 0)	0<br>(0 to 0)	0<br>(0 to 0)	0<br>(0 to 0)
Fujian	Dengue	0.388<br>(0.0415 to 0.886)	1.23<br>(0.13 to 2.8)	2.16<br>(0.851 to 4.38)	5.48<br>(2.16 to 11.12)
Gansu	Dengue	0<br>(0 to 0)	0<br>(0 to 0)	0<br>(0 to 0)	0<br>(0 to 0)
Guangdong	Dengue	1.62<br>(0.133 to 3.67)	2.47<br>(0.2 to 5.57)	13.3<br>(6.43 to 23.7)	11.34<br>(5.51 to 20.3)
Guangxi	Dengue	0.593<br>(0.0382 to 1.45)	1.36<br>(0.09 to 3.34)	3.2<br>(1.35 to 6.13)	6.62<br>(2.79 to 12.68)
Guizhou	Dengue	0<br>(0 to 0)	0<br>(0 to 0)	0<br>(0 to 0)	0<br>(0 to 0)
Hainan	Dengue	0.248<br>(0.0381 to 0.525)	3.56<br>(0.55 to 7.54)	1.5<br>(0.724 to 2.66)	15.64<br>(7.55 to 27.71)
Hebei	Dengue	0<br>(0 to 0)	0<br>(0 to 0)	0<br>(0 to 0)	0<br>(0 to 0)
Heilongjiang	Dengue	0<br>(0 to 0)	0<br>(0 to 0)	0<br>(0 to 0)	0<br>(0 to 0)
Henan	Dengue	0<br>(0 to 0)	0<br>(0 to 0)	0<br>(0 to 0)	0<br>(0 to 0)
Hubei	Dengue	0<br>(0 to 0)	0<br>(0 to 0)	0<br>(0 to 0)	0<br>(0 to 0)
Hunan	Dengue	0<br>(0 to 0)	0<br>(0 to 0)	0<br>(0 to 0)	0<br>(0 to 0)
Inner Mongolia	Dengue	0<br>(0 to 0)	0<br>(0 to 0)	0<br>(0 to 0)	0<br>(0 to 0)
Jiangsu	Dengue	0<br>(0 to 0)	0<br>(0 to 0)	0<br>(0 to 0)	0<br>(0 to 0)
Jiangxi	Dengue	0<br>(0 to 0)	0<br>(0 to 0)	0<br>(0 to 0)	0<br>(0 to 0)
Jilin	Dengue	0<br>(0 to 0)	0<br>(0 to 0)	0<br>(0 to 0)	0<br>(0 to 0)
Liaoning	Dengue	0<br>(0 to 0)	0<br>(0 to 0)	0<br>(0 to 0)	0<br>(0 to 0)
Ningxia	Dengue	0<br>(0 to 0)	0<br>(0 to 0)	0<br>(0 to 0)	0<br>(0 to 0)
Qinghai	Dengue	0<br>(0 to 0)	0<br>(0 to 0)	0<br>(0 to 0)	0<br>(0 to 0)
Shaanxi	Dengue	0<br>(0 to 0)	0<br>(0 to 0)	0<br>(0 to 0)	0<br>(0 to 0)
Shandong	Dengue	0<br>(0 to 0)	0<br>(0 to 0)	0<br>(0 to 0)	0<br>(0 to 0)
Shanghai	Dengue	0<br>(0 to 0)	0<br>(0 to 0)	0<br>(0 to 0)	0<br>(0 to 0)
Shanxi	Dengue	0<br>(0 to 0)	0<br>(0 to 0)	0<br>(0 to 0)	0<br>(0 to 0)
Sichuan	Dengue	0<br>(0 to 0)	0<br>(0 to 0)	0<br>(0 to 0)	0<br>(0 to 0)
Tianjin	Dengue	0<br>(0 to 0)	0<br>(0 to 0)	0<br>(0 to 0)	0<br>(0 to 0)

Tibet	Dengue	0 (0 to 0)	0 (0 to 0)	0 (0 to 0)	0 (0 to 0)
Xinjiang	Dengue	0 (0 to 0)	0 (0 to 0)	0 (0 to 0)	0 (0 to 0)
Yunnan	Dengue	0.38 (0.0623 to 0.889)	0.98 (0.16 to 2.28)	1.81 (0.592 to 4.29)	3.69 (1.2 to 8.72)
Zhejiang	Dengue	0.344 (0.0284 to 0.877)	0.78 (0.06 to 1.99)	2.19 (0.752 to 4.96)	3.58 (1.23 to 8.1)
China	Yellow fever	0 (0 to 0)	0 (0 to 0)	0 (0 to 0)	0 (0 to 0)
Taiwan	Yellow fever	0 (0 to 0)	0 (0 to 0)	0 (0 to 0)	0 (0 to 0)
Hong Kong Special Administrative Region of China	Yellow fever	0 (0 to 0)	0 (0 to 0)	0 (0 to 0)	0 (0 to 0)
Macau Special Administrative Region of China	Yellow fever	0 (0 to 0)	0 (0 to 0)	0 (0 to 0)	0 (0 to 0)
Anhui	Yellow fever	0 (0 to 0)	0 (0 to 0)	0 (0 to 0)	0 (0 to 0)
Beijing	Yellow fever	0 (0 to 0)	0 (0 to 0)	0 (0 to 0)	0 (0 to 0)
Chongqing	Yellow fever	0 (0 to 0)	0 (0 to 0)	0 (0 to 0)	0 (0 to 0)
Fujian	Yellow fever	0 (0 to 0)	0 (0 to 0)	0 (0 to 0)	0 (0 to 0)
Gansu	Yellow fever	0 (0 to 0)	0 (0 to 0)	0 (0 to 0)	0 (0 to 0)
Guangdong	Yellow fever	0 (0 to 0)	0 (0 to 0)	0 (0 to 0)	0 (0 to 0)
Guangxi	Yellow fever	0 (0 to 0)	0 (0 to 0)	0 (0 to 0)	0 (0 to 0)
Guizhou	Yellow fever	0 (0 to 0)	0 (0 to 0)	0 (0 to 0)	0 (0 to 0)
Hainan	Yellow fever	0 (0 to 0)	0 (0 to 0)	0 (0 to 0)	0 (0 to 0)
Hebei	Yellow fever	0 (0 to 0)	0 (0 to 0)	0 (0 to 0)	0 (0 to 0)
Heilongjiang	Yellow fever	0 (0 to 0)	0 (0 to 0)	0 (0 to 0)	0 (0 to 0)
Henan	Yellow fever	0 (0 to 0)	0 (0 to 0)	0 (0 to 0)	0 (0 to 0)
Hubei	Yellow fever	0 (0 to 0)	0 (0 to 0)	0 (0 to 0)	0 (0 to 0)
Hunan	Yellow fever	0 (0 to 0)	0 (0 to 0)	0 (0 to 0)	0 (0 to 0)
Inner Mongolia	Yellow fever	0 (0 to 0)	0 (0 to 0)	0 (0 to 0)	0 (0 to 0)
Jiangsu	Yellow fever	0 (0 to 0)	0 (0 to 0)	0 (0 to 0)	0 (0 to 0)
Jiangxi	Yellow fever	0 (0 to 0)	0 (0 to 0)	0 (0 to 0)	0 (0 to 0)
Jilin	Yellow fever	0 (0 to 0)	0 (0 to 0)	0 (0 to 0)	0 (0 to 0)
Liaoning	Yellow fever	0 (0 to 0)	0 (0 to 0)	0 (0 to 0)	0 (0 to 0)
Ningxia	Yellow fever	0 (0 to 0)	0 (0 to 0)	0 (0 to 0)	0 (0 to 0)
Qinghai	Yellow fever	0 (0 to 0)	0 (0 to 0)	0 (0 to 0)	0 (0 to 0)
Shaanxi	Yellow fever	0 (0 to 0)	0 (0 to 0)	0 (0 to 0)	0 (0 to 0)
Shandong	Yellow fever	0 (0 to 0)	0 (0 to 0)	0 (0 to 0)	0 (0 to 0)
Shanghai	Yellow fever	0 (0 to 0)	0 (0 to 0)	0 (0 to 0)	0 (0 to 0)
Shanxi	Yellow fever	0 (0 to 0)	0 (0 to 0)	0 (0 to 0)	0 (0 to 0)

Sichuan	Yellow fever	0<br>(0 to 0)	0<br>(0 to 0)	0<br>(0 to 0)	0<br>(0 to 0)
Tianjin	Yellow fever	0<br>(0 to 0)	0<br>(0 to 0)	0<br>(0 to 0)	0<br>(0 to 0)
Tibet	Yellow fever	0<br>(0 to 0)	0<br>(0 to 0)	0<br>(0 to 0)	0<br>(0 to 0)
Xinjiang	Yellow fever	0<br>(0 to 0)	0<br>(0 to 0)	0<br>(0 to 0)	0<br>(0 to 0)
Yunnan	Yellow fever	0<br>(0 to 0)	0<br>(0 to 0)	0<br>(0 to 0)	0<br>(0 to 0)
Zhejiang	Yellow fever	0<br>(0 to 0)	0<br>(0 to 0)	0<br>(0 to 0)	0<br>(0 to 0)
China	Rabies	109<br>(84.8 to 239)	9.06<br>(7.08 to 20)	36.2<br>(29 to 40)	2.56<br>(2.05 to 2.83)
Taiwan	Rabies	0.000609<br>(0.000501 to 0.000762)	0<br>(0 to 0)	0.000796<br>(0.000632 to 0.00101)	0<br>(0 to 0)
Hong Kong Special Administrative Region of China	Rabies	0.0619<br>(0.0484 to 0.0818)	1.07<br>(0.84 to 1.42)	0.136<br>(0.0895 to 0.187)	1.8<br>(1.19 to 2.49)
Macau Special Administrative Region of China	Rabies	0.00493<br>(0.00341 to 0.00711)	1.39<br>(0.96 to 2)	0.00312<br>(0.00193 to 0.00545)	0.46<br>(0.28 to 0.8)
Anhui	Rabies	4.98<br>(3.22 to 9.59)	8.4<br>(5.43 to 16.18)	1.37<br>(0.944 to 1.77)	2.23<br>(1.54 to 2.89)
Beijing	Rabies	0.137<br>(0.0911 to 0.246)	1.22<br>(0.81 to 2.19)	0.124<br>(0.0906 to 0.165)	0.51<br>(0.38 to 0.68)
Chongqing	Rabies	2.05<br>(1.35 to 3.81)	13<br>(8.53 to 24.17)	0.834<br>(0.583 to 1.09)	3.12<br>(2.18 to 4.07)
Fujian	Rabies	0.572<br>(0.223 to 3.39)	1.81<br>(0.71 to 10.71)	0.145<br>(0.104 to 0.251)	0.37<br>(0.26 to 0.64)
Gansu	Rabies	1.09<br>(0.376 to 8.04)	4.59<br>(1.59 to 34)	0.186<br>(0.134 to 0.32)	0.7<br>(0.5 to 1.2)
Guangdong	Rabies	8.66<br>(5.9 to 12.7)	13.16<br>(8.96 to 19.36)	4.45<br>(2.96 to 5.74)	3.81<br>(2.54 to 4.91)
Guangxi	Rabies	14.3<br>(9.35 to 22.9)	32.85<br>(21.5 to 52.6)	5.65<br>(3.92 to 7.4)	11.69<br>(8.11 to 15.3)
Guizhou	Rabies	11.4<br>(7.06 to 24.8)	34.38<br>(21.26 to 74.68)	2.34<br>(1.67 to 3.04)	6.92<br>(4.95 to 8.98)
Hainan	Rabies	4.63<br>(2.99 to 6.92)	66.43<br>(42.91 to 99.3)	0.96<br>(0.645 to 1.3)	10.01<br>(6.73 to 13.52)
Hebei	Rabies	4.24<br>(2.64 to 8.78)	6.66<br>(4.15 to 13.8)	1.71<br>(1.26 to 2.28)	2.24<br>(1.65 to 2.98)
Heilongjiang	Rabies	0.317<br>(0.0692 to 2.78)	0.87<br>(0.19 to 7.63)	0.0424<br>(0.0213 to 0.204)	0.11<br>(0.05 to 0.52)
Henan	Rabies	7.15<br>(4.73 to 12.9)	8.1<br>(5.36 to 14.64)	2.75<br>(1.96 to 3.6)	2.86<br>(2.04 to 3.73)
Hubei	Rabies	5.29<br>(3.53 to 9.59)	9.53<br>(6.35 to 17.27)	1.6<br>(1.16 to 2.06)	2.9<br>(2.1 to 3.72)
Hunan	Rabies	9.19<br>(6.17 to 18.2)	14.79<br>(9.92 to 29.28)	3.43<br>(2.38 to 4.44)	5.11<br>(3.54 to 6.61)
Inner Mongolia	Rabies	1.3<br>(0.525 to 7.12)	5.73<br>(2.31 to 31.32)	0.241<br>(0.18 to 0.338)	0.93<br>(0.7 to 1.3)
Jiangsu	Rabies	2.19<br>(1.5 to 3.63)	3.18<br>(2.18 to 5.27)	0.974<br>(0.713 to 1.25)	1.19<br>(0.87 to 1.53)
Jiangxi	Rabies	4.57<br>(2.4 to 19.2)	11.56<br>(6.08 to 48.49)	0.991<br>(0.757 to 1.3)	2.08<br>(1.59 to 2.74)
Jilin	Rabies	0.192<br>(0.0444 to 1.57)	0.74<br>(0.17 to 6.05)	0.0261<br>(0.0118 to 0.123)	0.09<br>(0.04 to 0.44)
Liaoning	Rabies	0.291<br>(0.111 to 1.73)	0.71<br>(0.27 to 4.25)	0.0658<br>(0.0427 to 0.19)	0.15<br>(0.1 to 0.43)
Ningxia	Rabies	0.393<br>(0.216 to 1.06)	7.95<br>(4.37 to 21.45)	0.121<br>(0.0891 to 0.161)	1.73<br>(1.27 to 2.3)
Qinghai	Rabies	0.186<br>(0.0129 to 2.29)	3.94<br>(0.27 to 48.31)	0.013<br>(0.00619 to 0.0659)	0.21<br>(0.1 to 1.05)
Shaanxi	Rabies	2.57<br>(1.58 to 5.42)	7.46<br>(4.59 to 15.74)	1.16<br>(0.841 to 1.5)	2.93<br>(2.13 to 3.8)
Shandong	Rabies	2.79<br>(1.93 to 4.82)	3.25<br>(2.25 to 5.63)	1.21<br>(0.9 to 1.55)	1.22<br>(0.91 to 1.56)

Shanghai	Rabies	0.0705<br>(0.0443 to 0.186)	0.5<br>(0.32 to 1.33)	0.0625<br>(0.0457 to 0.0967)	0.22<br>(0.16 to 0.35)
Shanxi	Rabies	2.54<br>(1.53 to 6.92)	8.39<br>(5.05 to 22.84)	1<br>(0.764 to 1.31)	2.6<br>(1.98 to 3.39)
Sichuan	Rabies	8.44<br>(4.81 to 27.3)	7.56<br>(4.31 to 24.5)	1.64<br>(1.19 to 2.09)	1.96<br>(1.42 to 2.5)
Tianjin	Rabies	0.151<br>(0.102 to 0.278)	1.64<br>(1.11 to 3.02)	0.124<br>(0.0933 to 0.161)	0.81<br>(0.61 to 1.05)
Tibet	Rabies	0.123<br>(0.018 to 1.24)	5.29<br>(0.77 to 53.1)	0.0194<br>(0.01 to 0.0717)	0.56<br>(0.29 to 2.08)
Xinjiang	Rabies	0.261<br>(0.0176 to 3.06)	1.65<br>(0.11 to 19.41)	0.0234<br>(0.00602 to 0.198)	0.1<br>(0.02 to 0.81)
Yunnan	Rabies	7.35<br>(4.14 to 17.4)	18.85<br>(10.61 to 44.53)	2.28<br>(1.75 to 2.95)	4.64<br>(3.56 to 6)
Zhejiang	Rabies	1.02<br>(0.702 to 1.86)	2.32<br>(1.59 to 4.21)	0.468<br>(0.347 to 0.633)	0.76<br>(0.57 to 1.03)
China	Intestinal nematode infections	882<br>(512 to 1450)	73.66<br>(42.74 to 121.42)	112<br>(60.3 to 192)	7.92<br>(4.27 to 13.57)
Taiwan	Intestinal nematode infections	8.64<br>(4.39 to 14.9)	42.33<br>(21.51 to 72.82)	0.687<br>(0.354 to 1.25)	2.91<br>(1.5 to 5.28)
Hong Kong Special Administrative Region of China	Intestinal nematode infections	1.15<br>(0.618 to 2)	19.89<br>(10.73 to 34.7)	0.441<br>(0.228 to 0.779)	5.87<br>(3.04 to 10.38)
Macau Special Administrative Region of China	Intestinal nematode infections	0.0676<br>(0.0381 to 0.114)	19.05<br>(10.73 to 32.07)	0.0375<br>(0.0197 to 0.0656)	5.49<br>(2.88 to 9.6)
Anhui	Intestinal nematode infections	57.5<br>(32.3 to 100)	97.11<br>(54.52 to 169.25)	6.79<br>(3.26 to 12.3)	11.1<br>(5.33 to 20.13)
Beijing	Intestinal nematode infections	3.01<br>(1.47 to 5.42)	26.88<br>(13.12 to 48.35)	0.898<br>(0.436 to 1.67)	3.72<br>(1.81 to 6.95)
Chongqing	Intestinal nematode infections	13<br>(6.96 to 22.7)	82.15<br>(44.11 to 143.82)	2.11<br>(1.02 to 4.01)	7.91<br>(3.83 to 15)
Fujian	Intestinal nematode infections	24.6<br>(13.7 to 41.5)	77.85<br>(43.32 to 131.22)	2.38<br>(1.17 to 4.44)	6.03<br>(2.97 to 11.26)
Gansu	Intestinal nematode infections	24.9<br>(14.1 to 42.5)	105.28<br>(59.49 to 179.65)	3.71<br>(1.86 to 6.88)	13.97<br>(7.01 to 25.87)
Guangdong	Intestinal nematode infections	37.1<br>(20.4 to 63.2)	56.38<br>(31.06 to 96.04)	5.57<br>(2.72 to 10.1)	4.77<br>(2.32 to 8.62)
Guangxi	Intestinal nematode infections	36.2<br>(20.6 to 61.4)	83.24<br>(47.39 to 141.09)	2.6<br>(1.36 to 4.83)	5.39<br>(2.82 to 9.99)
Guizhou	Intestinal nematode infections	43.2<br>(24.3 to 69.6)	130.28<br>(73.19 to 209.71)	5.15<br>(2.67 to 9.38)	15.22<br>(7.89 to 27.74)
Hainan	Intestinal nematode infections	4.62<br>(2.58 to 7.71)	66.28<br>(37.03 to 110.69)	0.696<br>(0.356 to 1.29)	7.26<br>(3.72 to 13.45)
Hebei	Intestinal nematode infections	39.7<br>(21.3 to 68.1)	62.41<br>(33.4 to 107)	5.21<br>(2.55 to 9.96)	6.81<br>(3.33 to 13.04)
Heilongjiang	Intestinal nematode infections	18.5<br>(9.79 to 31.6)	50.63<br>(26.85 to 86.75)	4.55<br>(2.25 to 8.65)	11.59<br>(5.72 to 22.04)
Henan	Intestinal nematode infections	71.8<br>(39 to 123)	81.34<br>(44.18 to 139.45)	10.9<br>(5.22 to 20.2)	11.34<br>(5.42 to 21)
Hubei	Intestinal nematode infections	38.4<br>(20.6 to 69.3)	69.06<br>(37.05 to 124.8)	3.7<br>(1.86 to 7.04)	6.69<br>(3.36 to 12.73)
Hunan	Intestinal nematode infections	47<br>(24.5 to 84.1)	75.61<br>(39.41 to 135.25)	3.74<br>(1.79 to 7.01)	5.57<br>(2.66 to 10.45)
Inner Mongolia	Intestinal nematode infections	14.4<br>(7.49 to 24.5)	63.43<br>(32.94 to 107.81)	1.69<br>(0.872 to 3.04)	6.51<br>(3.37 to 11.73)
Jiangsu	Intestinal nematode infections	38.1<br>(19.9 to 66.6)	55.29<br>(28.94 to 96.74)	4.46<br>(2.15 to 8.13)	5.47<br>(2.64 to 9.96)
Jiangxi	Intestinal nematode infections	40.4<br>(23.8 to 68.8)	102.18<br>(60.25 to 174.07)	5.03<br>(2.37 to 9.13)	10.58<br>(4.99 to 19.21)
Jilin	Intestinal nematode infections	13.2<br>(6.83 to 23.3)	51.01<br>(26.3 to 89.76)	1.84<br>(0.884 to 3.37)	6.59<br>(3.17 to 12.08)
Liaoning	Intestinal nematode infections	18.3<br>(9.29 to 32.6)	44.89<br>(22.78 to 79.81)	3.77<br>(1.78 to 7.21)	8.45<br>(4 to 16.15)
Ningxia	Intestinal nematode infections	4.71<br>(2.64 to 8.08)	95.37<br>(53.55 to 163.6)	0.727<br>(0.353 to 1.43)	10.36<br>(5.03 to 20.31)
Qinghai	Intestinal nematode infections	4.93<br>(2.8 to 8.03)	104.11<br>(59.06 to 169.49)	0.709<br>(0.338 to 1.37)	11.28<br>(5.39 to 21.8)

Shaanxi	Intestinal nematode infections	26.5<br>(14.1 to 46)	76.99<br>(41.09 to 133.58)	2.97<br>(1.47 to 5.48)	7.52<br>(3.71 to 13.88)
Shandong	Intestinal nematode infections	46.8<br>(26.1 to 78.5)	54.69<br>(30.46 to 91.69)	8.68<br>(4.24 to 16.1)	8.74<br>(4.27 to 16.25)
Shanghai	Intestinal nematode infections	4.45<br>(2.23 to 8.32)	31.87<br>(15.95 to 59.58)	1.25<br>(0.581 to 2.52)	4.46<br>(2.08 to 9.01)
Shanxi	Intestinal nematode infections	18.7<br>(9.74 to 32.8)	61.63<br>(32.16 to 108.48)	3.15<br>(1.5 to 5.96)	8.16<br>(3.89 to 15.43)
Sichuan	Intestinal nematode infections	97.5<br>(56.4 to 168)	87.36<br>(50.54 to 150.79)	6.88<br>(3.48 to 12.6)	8.24<br>(4.16 to 15.14)
Tianjin	Intestinal nematode infections	3.21<br>(1.59 to 6.13)	34.85<br>(17.26 to 66.63)	0.652<br>(0.319 to 1.2)	4.28<br>(2.09 to 7.84)
Tibet	Intestinal nematode infections	4.01<br>(2.3 to 6.56)	171.93<br>(98.72 to 281.73)	0.894<br>(0.445 to 1.66)	25.87<br>(12.87 to 48.06)
Xinjiang	Intestinal nematode infections	11.2<br>(6.31 to 19.3)	70.71<br>(40.02 to 122.42)	1.9<br>(0.93 to 3.41)	7.78<br>(3.82 to 13.99)
Yunnan	Intestinal nematode infections	48.4<br>(28.5 to 78.6)	124.15<br>(73.16 to 201.71)	5.41<br>(2.72 to 9.43)	11<br>(5.54 to 19.19)
Zhejiang	Intestinal nematode infections	26.2<br>(14 to 44.4)	59.35<br>(31.8 to 100.69)	3.35<br>(1.66 to 6.22)	5.48<br>(2.72 to 10.17)
China	Food-borne trematodiases	1200<br>(493 to 2420)	100.19<br>(41.17 to 202.56)	1350<br>(684 to 2540)	95.39<br>(48.44 to 179.6)
Taiwan	Food-borne trematodiases	0<br>(0 to 0)	0<br>(0 to 0)	0<br>(0 to 0)	0<br>(0 to 0)
Hong Kong Special Administrative Region of China	Food-borne trematodiases	3.49<br>(1.69 to 6.56)	60.47<br>(29.33 to 113.78)	3.58<br>(2.08 to 5.95)	47.64<br>(27.74 to 79.27)
Macau Special Administrative Region of China	Food-borne trematodiases	0.197<br>(0.0927 to 0.371)	55.55<br>(26.11 to 104.65)	0.323<br>(0.18 to 0.545)	47.29<br>(26.31 to 79.73)
Anhui	Food-borne trematodiases	32.1<br>(12.8 to 67.7)	54.19<br>(21.56 to 114.21)	25.9<br>(14.5 to 44.3)	42.41<br>(23.65 to 72.41)
Beijing	Food-borne trematodiases	6.02<br>(2.65 to 11.8)	53.7<br>(23.64 to 105.35)	11.6<br>(6.34 to 19.7)	47.94<br>(26.29 to 81.62)
Chongqing	Food-borne trematodiases	12.5<br>(4.48 to 26.9)	79.31<br>(28.41 to 170.6)	15.9<br>(7.37 to 31.3)	59.68<br>(27.59 to 117.05)
Fujian	Food-borne trematodiases	37.2<br>(10.5 to 83.2)	117.55<br>(33.22 to 263.01)	35.4<br>(13.2 to 76.3)	89.73<br>(33.38 to 193.62)
Gansu	Food-borne trematodiases	13.3<br>(5.49 to 27.3)	56.03<br>(23.19 to 115.61)	11.4<br>(6.38 to 19.1)	42.91<br>(24 to 71.92)
Guangdong	Food-borne trematodiases	220<br>(124 to 372)	333.8<br>(187.79 to 564.9)	382<br>(231 to 601)	326.89<br>(197.49 to 514.79)
Guangxi	Food-borne trematodiases	60.6<br>(37.3 to 90.4)	139.41<br>(85.87 to 207.82)	62.7<br>(40.5 to 88.9)	129.8<br>(83.74 to 183.93)
Guizhou	Food-borne trematodiases	51.9<br>(15 to 118)	156.25<br>(45.26 to 356.4)	36.5<br>(12 to 82.4)	107.84<br>(35.5 to 243.69)
Hainan	Food-borne trematodiases	3.6<br>(1.49 to 7.47)	51.7<br>(21.35 to 107.24)	4.19<br>(2.34 to 7.19)	43.66<br>(24.45 to 74.98)
Hebei	Food-borne trematodiases	34.3<br>(13.9 to 71)	53.87<br>(21.86 to 111.6)	32.8<br>(18.3 to 55.7)	42.86<br>(23.89 to 72.82)
Heilongjiang	Food-borne trematodiases	21.9<br>(8.94 to 46.3)	60.07<br>(24.53 to 127.05)	20<br>(10.8 to 35.4)	51.07<br>(27.41 to 90.11)
Henan	Food-borne trematodiases	49.4<br>(19.7 to 104)	55.95<br>(22.33 to 117.97)	40<br>(22.3 to 68.8)	41.57<br>(23.17 to 71.43)
Hubei	Food-borne trematodiases	146<br>(34.3 to 343)	263.49<br>(61.67 to 616.76)	192<br>(45.4 to 474)	347.12<br>(82.03 to 857.94)
Hunan	Food-borne trematodiases	35.2<br>(14.2 to 72.7)	56.61<br>(22.9 to 116.89)	29.2<br>(16.2 to 49.8)	43.46<br>(24.09 to 74.21)
Inner Mongolia	Food-borne trematodiases	0<br>(0 to 0)	0<br>(0 to 0)	0<br>(0 to 0)	0<br>(0 to 0)
Jiangsu	Food-borne trematodiases	39<br>(16.4 to 80.7)	56.64<br>(23.86 to 117.26)	36.8<br>(20.9 to 63.6)	45.06<br>(25.54 to 77.87)
Jiangxi	Food-borne trematodiases	21.7<br>(9.01 to 45)	54.93<br>(22.8 to 113.75)	20.2<br>(11.4 to 34.4)	42.47<br>(23.93 to 72.32)
Jilin	Food-borne trematodiases	15.7<br>(7.05 to 31.7)	60.67<br>(27.16 to 122.17)	13.3<br>(7.75 to 22.3)	47.72<br>(27.79 to 79.92)
Liaoning	Food-borne trematodiases	22<br>(9.54 to 43.7)	53.96<br>(23.4 to 107.12)	18.9<br>(11.1 to 30.1)	42.32<br>(24.76 to 67.41)

Ningxia	Food-borne trematodiases	0 (0 to 0)	0 (0 to 0)	0 (0 to 0)	0 (0 to 0)
Qinghai	Food-borne trematodiases	0 (0 to 0)	0 (0 to 0)	0 (0 to 0)	0 (0 to 0)
Shaanxi	Food-borne trematodiases	19 (7.66 to 40)	55.1 (22.26 to 116.27)	17.8 (9.91 to 30.7)	45.07 (25.08 to 77.73)
Shandong	Food-borne trematodiases	48.3 (19.7 to 99.3)	56.38 (23.03 to 116.05)	43.9 (24.2 to 75.1)	44.13 (24.3 to 75.59)
Shanghai	Food-borne trematodiases	10.7 (4 to 22.8)	76.73 (28.66 to 163.29)	17.7 (8.33 to 34)	63.27 (29.8 to 121.69)
Shanxi	Food-borne trematodiases	63.9 (15.6 to 148)	211 (51.4 to 489.23)	59.9 (17.3 to 139)	155.21 (44.79 to 358.79)
Sichuan	Food-borne trematodiases	64.9 (26.2 to 137)	58.19 (23.52 to 122.76)	36.2 (20.3 to 62.1)	43.39 (24.31 to 74.35)
Tianjin	Food-borne trematodiases	4.87 (2.06 to 9.48)	52.98 (22.36 to 103.07)	7.47 (4.11 to 12.9)	48.94 (26.92 to 84.58)
Tibet	Food-borne trematodiases	0 (0 to 0)	0 (0 to 0)	0 (0 to 0)	0 (0 to 0)
Xinjiang	Food-borne trematodiases	0 (0 to 0)	0 (0 to 0)	0 (0 to 0)	0 (0 to 0)
Yunnan	Food-borne trematodiases	21.5 (8.89 to 45.4)	55.27 (22.82 to 116.47)	21.5 (12 to 36.6)	43.63 (24.33 to 74.45)
Zhejiang	Food-borne trematodiases	140 (30.1 to 336)	317.08 (68.23 to 761.71)	150 (36.1 to 372)	245.79 (59.03 to 608.76)
China	Other neglected tropical diseases	255 (182 to 346)	21.34 (15.22 to 28.92)	70.4 (50.1 to 97.7)	4.99 (3.55 to 6.92)
Taiwan	Other neglected tropical diseases	1.06 (0.558 to 1.79)	5.18 (2.74 to 8.79)	0.725 (0.355 to 1.46)	3.07 (1.51 to 6.2)
Hong Kong Special Administrative Region of China	Other neglected tropical diseases	0.469 (0.242 to 0.822)	8.14 (4.2 to 14.26)	0.329 (0.187 to 0.582)	4.38 (2.48 to 7.75)
Macau Special Administrative Region of China	Other neglected tropical diseases	0.0366 (0.0211 to 0.0646)	10.31 (5.94 to 18.2)	0.024 (0.0139 to 0.041)	3.51 (2.03 to 5.99)
Anhui	Other neglected tropical diseases	14.6 (9.9 to 21.3)	24.71 (16.71 to 35.95)	3.28 (1.96 to 5.35)	5.36 (3.2 to 8.74)
Beijing	Other neglected tropical diseases	0.998 (0.597 to 1.59)	8.9 (5.32 to 14.19)	0.562 (0.274 to 0.994)	2.33 (1.14 to 4.12)
Chongqing	Other neglected tropical diseases	3.41 (2.03 to 5.52)	21.62 (12.84 to 34.99)	1.69 (0.885 to 2.93)	6.34 (3.31 to 10.97)
Fujian	Other neglected tropical diseases	5.04 (3.48 to 6.99)	15.93 (11 to 22.11)	1.66 (0.992 to 2.83)	4.2 (2.52 to 7.19)
Gansu	Other neglected tropical diseases	3.84 (2.49 to 6.47)	16.22 (10.53 to 27.35)	1.29 (0.726 to 2.14)	4.87 (2.73 to 8.06)
Guangdong	Other neglected tropical diseases	10.6 (6.76 to 14.9)	16.06 (10.28 to 22.62)	3.87 (2.24 to 5.96)	3.31 (1.91 to 5.1)
Guangxi	Other neglected tropical diseases	7.64 (5.29 to 10.8)	17.57 (12.17 to 24.73)	2.7 (1.58 to 4.71)	5.59 (3.27 to 9.76)
Guizhou	Other neglected tropical diseases	6.96 (5.01 to 9.8)	20.98 (15.1 to 29.54)	1.73 (1.08 to 2.94)	5.13 (3.2 to 8.68)
Hainan	Other neglected tropical diseases	2.54 (1.59 to 3.75)	36.4 (22.87 to 53.89)	0.549 (0.341 to 0.849)	5.73 (3.56 to 8.85)
Hebei	Other neglected tropical diseases	11.1 (7.09 to 16.2)	17.47 (11.14 to 25.47)	2.61 (1.36 to 4.66)	3.41 (1.78 to 6.09)
Heilongjiang	Other neglected tropical diseases	8.01 (4.86 to 11.5)	21.97 (13.33 to 31.66)	2.5 (1.37 to 3.87)	6.37 (3.5 to 9.86)
Henan	Other neglected tropical diseases	18.2 (12.3 to 26.1)	20.61 (13.96 to 29.57)	4.24 (2.61 to 6.57)	4.4 (2.71 to 6.83)
Hubei	Other neglected tropical diseases	17.9 (11.9 to 25.9)	32.25 (21.33 to 46.61)	3.13 (1.67 to 5.04)	5.66 (3.01 to 9.11)
Hunan	Other neglected tropical diseases	10 (6.4 to 14.7)	16.13 (10.29 to 23.65)	2.72 (1.62 to 4.52)	4.04 (2.42 to 6.73)
Inner Mongolia	Other neglected tropical diseases	2.59 (1.73 to 4.01)	11.37 (7.61 to 17.63)	1.04 (0.62 to 1.66)	4.02 (2.39 to 6.43)
Jiangsu	Other neglected tropical diseases	18 (11.5 to 26.6)	26.16 (16.73 to 38.68)	3.37 (1.78 to 6.19)	4.12 (2.18 to 7.58)
Jiangxi	Other neglected tropical diseases	10.6 (5.53 to 15.1)	26.83 (13.99 to 38.08)	3.74 (2.13 to 5.79)	7.87 (4.49 to 12.18)

Jilin	Other neglected tropical diseases	5.07 (3.32 to 7.45)	19.53 (12.79 to 28.7)	1.46 (0.939 to 2.19)	5.24 (3.37 to 7.86)
Liaoning	Other neglected tropical diseases	7.15 (4.68 to 10.4)	17.53 (11.48 to 25.48)	2.5 (1.48 to 3.92)	5.59 (3.31 to 8.78)
Ningxia	Other neglected tropical diseases	0.999 (0.665 to 1.49)	20.23 (13.46 to 30.09)	0.351 (0.218 to 0.563)	5 (3.11 to 8.03)
Qinghai	Other neglected tropical diseases	0.831 (0.55 to 1.22)	17.55 (11.62 to 25.67)	0.426 (0.263 to 0.644)	6.79 (4.18 to 10.25)
Shaanxi	Other neglected tropical diseases	17.2 (8.32 to 24.4)	49.9 (24.18 to 70.92)	4.72 (1.96 to 7.17)	11.94 (4.97 to 18.15)
Shandong	Other neglected tropical diseases	16.4 (11.2 to 23.5)	19.18 (13.06 to 27.47)	5.48 (3.32 to 8.46)	5.52 (3.34 to 8.51)
Shanghai	Other neglected tropical diseases	4.23 (2.65 to 6.13)	30.24 (19 to 43.91)	1.66 (0.844 to 2.6)	5.94 (3.02 to 9.31)
Shanxi	Other neglected tropical diseases	6.57 (4.27 to 9.67)	21.69 (14.09 to 31.93)	1.82 (0.995 to 3.03)	4.7 (2.58 to 7.85)
Sichuan	Other neglected tropical diseases	21.1 (14.2 to 31.2)	18.91 (12.74 to 27.95)	3.97 (2.34 to 6.42)	4.75 (2.8 to 7.69)
Tianjin	Other neglected tropical diseases	1.24 (0.813 to 1.82)	13.47 (8.84 to 19.77)	0.625 (0.337 to 1.15)	4.1 (2.21 to 7.56)
Tibet	Other neglected tropical diseases	0.627 (0.334 to 0.863)	26.92 (14.36 to 37.05)	0.42 (0.277 to 0.597)	12.16 (8.01 to 17.27)
Xinjiang	Other neglected tropical diseases	2.91 (1.97 to 4.31)	18.45 (12.49 to 27.32)	1.39 (0.801 to 2.32)	5.7 (3.29 to 9.54)
Yunnan	Other neglected tropical diseases	6.14 (3.26 to 9.05)	15.77 (8.35 to 23.23)	2.17 (1.46 to 3.11)	4.42 (2.97 to 6.33)
Zhejiang	Other neglected tropical diseases	12.4 (8.62 to 17.4)	28.02 (19.56 to 39.56)	2.42 (1.53 to 3.92)	3.95 (2.5 to 6.4)
China	Maternal disorders	1430 (1320 to 1540)	119.21 (109.88 to 128.37)	164 (146 to 184)	11.62 (10.36 to 13.05)
Taiwan	Maternal disorders	3.95 (3.5 to 4.7)	19.35 (17.17 to 23.04)	1.69 (1.48 to 1.98)	7.16 (6.26 to 8.4)
Hong Kong Special Administrative Region of China	Maternal disorders	0.702 (0.579 to 0.853)	12.17 (10.04 to 14.8)	0.471 (0.355 to 0.61)	6.27 (4.73 to 8.12)
Macau Special Administrative Region of China	Maternal disorders	0.169 (0.138 to 0.207)	47.7 (39.01 to 58.29)	0.0552 (0.0398 to 0.0763)	8.08 (5.83 to 11.16)
Anhui	Maternal disorders	85 (67.6 to 106)	143.53 (114.09 to 179.29)	6.1 (4.7 to 7.94)	9.97 (7.69 to 12.98)
Beijing	Maternal disorders	7.95 (6.19 to 10.3)	70.88 (55.16 to 91.97)	2.43 (1.82 to 3.22)	10.1 (7.54 to 13.38)
Chongqing	Maternal disorders	20.1 (15.5 to 25.7)	127.29 (98 to 162.85)	2.71 (2.05 to 3.48)	10.13 (7.65 to 13.03)
Fujian	Maternal disorders	29.2 (23.6 to 35.8)	92.14 (74.68 to 113.07)	2.8 (2.11 to 3.68)	7.11 (5.35 to 9.35)
Gansu	Maternal disorders	54.8 (44.3 to 67.1)	231.63 (187.12 to 283.72)	5.59 (4.2 to 7.17)	21.02 (15.79 to 26.99)
Guangdong	Maternal disorders	59.7 (47 to 76.5)	90.66 (71.46 to 116.22)	13.6 (10.4 to 17.6)	11.66 (8.87 to 15.08)
Guangxi	Maternal disorders	83 (63.8 to 105)	190.76 (146.61 to 240.56)	7.17 (5.41 to 9.18)	14.83 (11.2 to 19)
Guizhou	Maternal disorders	108 (82.3 to 140)	324.41 (247.94 to 422.77)	6.9 (5.18 to 9.28)	20.41 (15.3 to 27.43)
Hainan	Maternal disorders	8.73 (6.71 to 11.3)	125.25 (96.32 to 161.85)	1.49 (1.1 to 1.99)	15.58 (11.46 to 20.77)
Hebei	Maternal disorders	42 (34 to 51)	66.01 (53.45 to 80.21)	9.2 (7.12 to 11.8)	12.04 (9.31 to 15.39)
Heilongjiang	Maternal disorders	20.5 (15.6 to 26.1)	56.19 (42.75 to 71.67)	1.94 (1.52 to 2.48)	4.95 (3.86 to 6.31)
Henan	Maternal disorders	92 (75.9 to 111)	104.22 (85.96 to 125.83)	16.1 (12.5 to 20.6)	16.72 (12.97 to 21.38)
Hubei	Maternal disorders	37.1 (29.3 to 45.3)	66.69 (52.83 to 81.49)	5.24 (3.82 to 7.25)	9.47 (6.91 to 13.11)
Hunan	Maternal disorders	81.7 (65.8 to 100)	131.35 (105.77 to 161.06)	7.74 (6 to 9.92)	11.53 (8.94 to 14.77)
Inner Mongolia	Maternal disorders	26.6 (20.3 to 34)	117.03 (89.05 to 149.42)	1.79 (1.39 to 2.34)	6.92 (5.39 to 9.03)

Jiangsu	Maternal disorders	30.2 (24.1 to 36.4)	43.81 (35.09 to 52.96)	5.01 (3.67 to 6.84)	6.13 (4.49 to 8.38)
Jiangxi	Maternal disorders	87.6 (70.4 to 109)	221.71 (178.06 to 274.79)	4.78 (3.66 to 6.15)	10.06 (7.7 to 12.95)
Jilin	Maternal disorders	24 (18.5 to 30.8)	92.44 (71.41 to 118.5)	2.08 (1.57 to 2.69)	7.46 (5.64 to 9.65)
Liaoning	Maternal disorders	17.2 (13 to 22.1)	42.06 (31.87 to 54.11)	2.36 (1.78 to 3.08)	5.29 (3.99 to 6.91)
Ningxia	Maternal disorders	10.3 (7.8 to 13.4)	209.04 (157.85 to 272.05)	0.821 (0.591 to 1.08)	11.69 (8.42 to 15.41)
Qinghai	Maternal disorders	16.5 (13 to 20.8)	349.11 (274.08 to 438.24)	2.13 (1.59 to 2.88)	33.93 (25.35 to 45.79)
Shaanxi	Maternal disorders	19.6 (16.1 to 23.6)	56.83 (46.74 to 68.62)	5.78 (4.24 to 7.56)	14.62 (10.74 to 19.15)
Shandong	Maternal disorders	49.5 (38.1 to 62.9)	57.8 (44.54 to 73.51)	7.98 (6.08 to 10.3)	8.03 (6.12 to 10.34)
Shanghai	Maternal disorders	4.43 (3.52 to 5.5)	31.68 (25.23 to 39.34)	1.33 (0.953 to 1.95)	4.76 (3.41 to 7)
Shanxi	Maternal disorders	56 (44.3 to 70.9)	184.95 (146.32 to 234.16)	5.26 (4 to 6.6)	13.62 (10.37 to 17.1)
Sichuan	Maternal disorders	179 (142 to 219)	160.27 (127.67 to 196.36)	8.65 (6.68 to 11)	10.36 (8 to 13.2)
Tianjin	Maternal disorders	5.27 (3.98 to 6.86)	57.34 (43.33 to 74.54)	1.07 (0.803 to 1.35)	7 (5.26 to 8.85)
Tibet	Maternal disorders	12.4 (9.76 to 15.4)	531.55 (418.87 to 659.95)	3.44 (2.55 to 4.47)	99.47 (73.79 to 129.39)
Xinjiang	Maternal disorders	48.4 (39.2 to 59.1)	307.07 (248.25 to 374.88)	10.4 (7.64 to 13.8)	42.59 (31.36 to 56.75)
Yunnan	Maternal disorders	80.6 (61.7 to 104)	206.88 (158.4 to 265.87)	8.37 (6.42 to 10.9)	17.03 (13.05 to 22.26)
Zhejiang	Maternal disorders	29.3 (23.7 to 36.3)	66.48 (53.85 to 82.34)	3.35 (2.48 to 4.4)	5.47 (4.06 to 7.2)
China	Neonatal disorders	31800 (29600 to 34100)	2656.23 (2472.85 to 2851.12)	11200 (9350 to 13300)	792.13 (661.8 to 943.15)
Taiwan	Neonatal disorders	76.4 (66.7 to 87.7)	374.53 (327.15 to 429.75)	68.4 (55.1 to 85.9)	290.24 (233.47 to 364.26)
Hong Kong Special Administrative Region of China	Neonatal disorders	28.6 (24.8 to 32.5)	495.34 (430.83 to 564.65)	22 (16.1 to 29.8)	293.59 (214.65 to 396.26)
Macau Special Administrative Region of China	Neonatal disorders	3.11 (2.67 to 3.58)	877.63 (752.76 to 1008.28)	2.67 (2.04 to 3.51)	390.93 (298.14 to 513.01)
Anhui	Neonatal disorders	2270 (1870 to 2720)	3836.13 (3156.94 to 4582.55)	616 (458 to 843)	1007.78 (748.97 to 1379.3)
Beijing	Neonatal disorders	96.8 (80.8 to 113)	863.23 (720.05 to 1011.38)	148 (119 to 184)	613.58 (494.57 to 764.04)
Chongqing	Neonatal disorders	464 (338 to 613)	2938.27 (2144.79 to 3887.16)	147 (119 to 176)	548.46 (446.58 to 656.95)
Fujian	Neonatal disorders	772 (648 to 901)	2439.04 (2049.67 to 2847.05)	266 (216 to 324)	674.05 (547.26 to 823.19)
Gansu	Neonatal disorders	1660 (1390 to 1930)	7005.47 (5891 to 8156.27)	326 (261 to 392)	1227.19 (980.97 to 1473.12)
Guangdong	Neonatal disorders	1080 (911 to 1280)	1642.95 (1383.82 to 1939.35)	816 (662 to 999)	698.41 (566.82 to 854.88)
Guangxi	Neonatal disorders	776 (647 to 929)	1784.49 (1487.77 to 2137.05)	322 (260 to 394)	665.58 (538.55 to 816)
Guizhou	Neonatal disorders	1080 (850 to 1370)	3258.8 (2560.05 to 4139.4)	192 (154 to 238)	567.79 (455.19 to 703.45)
Hainan	Neonatal disorders	203 (160 to 249)	2909.97 (2297.75 to 3572.64)	113 (93.1 to 135)	1174.01 (970.72 to 1405.11)
Hebei	Neonatal disorders	1540 (1320 to 1800)	2417.92 (2074.68 to 2822.86)	1190 (869 to 1580)	1553.23 (1136.58 to 2073.48)
Heilongjiang	Neonatal disorders	537 (438 to 651)	1473.27 (1202.88 to 1786.38)	212 (164 to 265)	540.93 (418.42 to 675.34)
Henan	Neonatal disorders	2300 (1940 to 2720)	2601.49 (2193 to 3087.01)	669 (526 to 838)	694.88 (546 to 870.46)
Hubei	Neonatal disorders	1230 (1020 to 1460)	2207.34 (1836.69 to 2626.71)	308 (243 to 379)	557.08 (438.59 to 685.93)

Hunan	Neonatal disorders	1200 (985 to 1460)	1931.38 (1584.58 to 2340.53)	347 (272 to 442)	517.17 (404.77 to 657.85)
Inner Mongolia	Neonatal disorders	689 (575 to 815)	3030.49 (2530.2 to 3583.03)	195 (157 to 238)	754.38 (606.5 to 920.21)
Jiangsu	Neonatal disorders	996 (814 to 1190)	1446.82 (1182.4 to 1730.3)	440 (344 to 558)	538.85 (421.53 to 683.54)
Jiangxi	Neonatal disorders	1730 (1360 to 2150)	4379.52 (3443.44 to 5450.43)	462 (382 to 550)	971.58 (803.09 to 1158.02)
Jilin	Neonatal disorders	513 (431 to 604)	1977.62 (1662.58 to 2326.32)	153 (125 to 185)	547.57 (447.08 to 662.57)
Liaoning	Neonatal disorders	375 (312 to 451)	920.41 (764.79 to 1104.55)	177 (139 to 219)	395.61 (310.99 to 490.96)
Ningxia	Neonatal disorders	276 (210 to 351)	5592.21 (4253.86 to 7108.15)	80.5 (64.8 to 99.1)	1147.19 (923.12 to 1412.54)
Qinghai	Neonatal disorders	277 (209 to 349)	5852.78 (4402.69 to 7369.07)	79.7 (62.5 to 101)	1268.28 (995.06 to 1602.22)
Shaanxi	Neonatal disorders	1230 (1050 to 1430)	3583.94 (3056.27 to 4169.53)	589 (414 to 808)	1490.96 (1048.12 to 2045.83)
Shandong	Neonatal disorders	1140 (960 to 1350)	1329.37 (1121.13 to 1581.57)	512 (404 to 640)	515.19 (406.19 to 644.23)
Shanghai	Neonatal disorders	112 (93 to 134)	803.18 (665.9 to 957.12)	164 (133 to 202)	587.95 (475.05 to 723.43)
Shanxi	Neonatal disorders	889 (768 to 1030)	2936.52 (2534.93 to 3395.43)	315 (258 to 375)	815.27 (668.26 to 971.62)
Sichuan	Neonatal disorders	4370 (3400 to 5480)	3918.74 (3048.65 to 4906.32)	845 (623 to 1150)	1012.47 (745.82 to 1375.98)
Tianjin	Neonatal disorders	94.2 (78.6 to 113)	1024.26 (854.53 to 1228.35)	89 (72.3 to 107)	583.1 (473.77 to 701.75)
Tibet	Neonatal disorders	136 (105 to 168)	5835.83 (4486.13 to 7227.79)	76.6 (59.6 to 96.5)	2215.1 (1724.07 to 2793.25)
Xinjiang	Neonatal disorders	772 (615 to 943)	4895.13 (3899.4 to 5977.08)	388 (306 to 483)	1590.89 (1254.57 to 1981.81)
Yunnan	Neonatal disorders	1970 (1510 to 2500)	5049.73 (3873.78 to 6421.51)	544 (435 to 665)	1106.92 (883.76 to 1353.49)
Zhejiang	Neonatal disorders	985 (835 to 1150)	2232.81 (1894.46 to 2618.36)	385 (306 to 482)	629.98 (500.16 to 788.06)
China	Nutritional deficiencies	8000 (6180 to 10500)	668.23 (516.38 to 880.33)	1890 (1310 to 2750)	133.57 (92.43 to 194.91)
Taiwan	Nutritional deficiencies	35.8 (21.9 to 55.9)	175.68 (107.1 to 273.98)	19.2 (10.5 to 35.9)	81.53 (44.39 to 152.15)
Hong Kong Special Administrative Region of China	Nutritional deficiencies	12.3 (7.31 to 19.9)	213.18 (126.9 to 344.67)	8.55 (4.89 to 14.5)	113.82 (65.18 to 193.2)
Macau Special Administrative Region of China	Nutritional deficiencies	0.899 (0.573 to 1.51)	253.32 (161.44 to 425.91)	0.66 (0.386 to 1.13)	96.51 (56.45 to 165.89)
Anhui	Nutritional deficiencies	583 (443 to 770)	983.16 (747.89 to 1299.84)	102 (62.6 to 160)	166.89 (102.38 to 261.89)
Beijing	Nutritional deficiencies	27.3 (17.7 to 41.4)	243.77 (157.5 to 369.11)	17.2 (9.66 to 29.7)	71.56 (40.07 to 123.14)
Chongqing	Nutritional deficiencies	89 (58.7 to 134)	563.79 (371.92 to 852.27)	45.7 (25.2 to 78.8)	171.13 (94.39 to 294.82)
Fujian	Nutritional deficiencies	187 (141 to 247)	591.48 (445.59 to 780.8)	45.8 (28.4 to 74.5)	116.15 (72.07 to 189.01)
Gansu	Nutritional deficiencies	203 (155 to 261)	860.07 (655.98 to 1101.42)	44.6 (28.6 to 69.6)	167.65 (107.62 to 261.92)
Guangdong	Nutritional deficiencies	345 (260 to 457)	523.64 (395.78 to 694.3)	92.1 (60.6 to 143)	78.85 (51.84 to 122.09)
Guangxi	Nutritional deficiencies	310 (234 to 409)	713.76 (537.8 to 940.71)	65.9 (41.5 to 107)	136.37 (85.95 to 220.55)
Guizhou	Nutritional deficiencies	486 (382 to 608)	1465.16 (1149.51 to 1832.01)	55.2 (35 to 90.2)	163.24 (103.51 to 266.74)
Hainan	Nutritional deficiencies	97.8 (75.9 to 125)	1404.24 (1088.99 to 1790.37)	21.6 (15.8 to 30.6)	225.15 (164.68 to 319.52)
Hebei	Nutritional deficiencies	306 (211 to 437)	480.63 (331.55 to 686.26)	94.8 (58.3 to 154)	124.09 (76.26 to 200.96)
Heilongjiang	Nutritional deficiencies	157 (101 to 238)	430.65 (277.73 to 652.87)	53.2 (30 to 91.4)	135.64 (76.5 to 232.82)

Henan	Nutritional deficiencies	471<br>(321 to 669)	533.79<br>(363.76 to 758.16)	104<br>(58.8 to 174)	107.92<br>(61.09 to 181.09)
Hubei	Nutritional deficiencies	412<br>(284 to 596)	741.64<br>(510.51 to 1073.55)	66.4<br>(37.9 to 110)	120.03<br>(68.47 to 198.93)
Hunan	Nutritional deficiencies	307<br>(217 to 423)	494.09<br>(348.98 to 680.65)	74.2<br>(43.2 to 122)	110.44<br>(64.34 to 181.62)
Inner Mongolia	Nutritional deficiencies	80.6<br>(55.4 to 116)	354.36<br>(243.44 to 511.46)	26.9<br>(15.6 to 44.4)	104.07<br>(60.17 to 171.47)
Jiangsu	Nutritional deficiencies	481<br>(327 to 694)	698.61<br>(474.61 to 1008.85)	105<br>(56.2 to 184)	128.07<br>(68.78 to 225.22)
Jiangxi	Nutritional deficiencies	304<br>(231 to 398)	769.05<br>(585.24 to 1007.92)	85<br>(51.7 to 135)	178.88<br>(108.76 to 284.5)
Jilin	Nutritional deficiencies	123<br>(85.1 to 179)	472.73<br>(327.79 to 690.95)	34.4<br>(21.1 to 55.5)	123.2<br>(75.79 to 198.82)
Liaoning	Nutritional deficiencies	160<br>(103 to 240)	391.99<br>(252.36 to 588.34)	59.1<br>(34.2 to 101)	132.42<br>(76.65 to 225.15)
Ningxia	Nutritional deficiencies	35<br>(25.5 to 47.2)	708.8<br>(516.01 to 956.07)	10.9<br>(6.67 to 16.9)	155<br>(95.09 to 240.12)
Qinghai	Nutritional deficiencies	30.5<br>(22.8 to 39.9)	644.13<br>(481.89 to 841.33)	10.5<br>(6.34 to 16.1)	166.44<br>(100.91 to 256.82)
Shaanxi	Nutritional deficiencies	312<br>(230 to 424)	907.93<br>(668.84 to 1232.58)	78.9<br>(45.7 to 126)	199.66<br>(115.79 to 319)
Shandong	Nutritional deficiencies	394<br>(253 to 584)	460.75<br>(295.74 to 682.23)	144<br>(81.6 to 230)	145.18<br>(82.12 to 231.34)
Shanghai	Nutritional deficiencies	87.8<br>(55.1 to 130)	628.29<br>(394.61 to 932.68)	34.5<br>(17.7 to 62.2)	123.53<br>(63.46 to 222.54)
Shanxi	Nutritional deficiencies	193<br>(135 to 272)	637.58<br>(445.71 to 899.26)	59.4<br>(34.6 to 101)	153.9<br>(89.54 to 261.2)
Sichuan	Nutritional deficiencies	759<br>(560 to 1030)	680.48<br>(501.53 to 918.8)	116<br>(72.3 to 183)	138.6<br>(86.61 to 219.44)
Tianjin	Nutritional deficiencies	32.6<br>(21.5 to 48.5)	354.51<br>(234.22 to 527)	17.3<br>(9.51 to 29.4)	113.15<br>(62.3 to 192.41)
Tibet	Nutritional deficiencies	34.3<br>(26.3 to 42.3)	1473.81<br>(1130.18 to 1813.45)	15.7<br>(11 to 22.9)	452.85<br>(318.48 to 662.44)
Xinjiang	Nutritional deficiencies	183<br>(143 to 232)	1158.09<br>(908.74 to 1473.02)	68.2<br>(47.7 to 99.8)	280.02<br>(195.83 to 409.45)
Yunnan	Nutritional deficiencies	490<br>(374 to 623)	1257.5<br>(958.77 to 1598.31)	65.1<br>(48.6 to 90.7)	132.43<br>(98.9 to 184.53)
Zhejiang	Nutritional deficiencies	303<br>(210 to 438)	687.59<br>(476.53 to 993.39)	64.2<br>(39.2 to 105)	104.91<br>(64.1 to 172.39)
China	Protein-energy malnutrition	2600<br>(2330 to 2890)	217.17<br>(194.73 to 241.14)	178<br>(164 to 207)	12.59<br>(11.59 to 14.63)
Taiwan	Protein-energy malnutrition	4.12<br>(3.29 to 4.49)	20.21<br>(16.11 to 21.98)	1.56<br>(1.39 to 1.74)	6.63<br>(5.89 to 7.39)
Hong Kong Special Administrative Region of China	Protein-energy malnutrition	0.711<br>(0.618 to 0.804)	12.33<br>(10.73 to 13.95)	0.31<br>(0.26 to 0.372)	4.13<br>(3.46 to 4.96)
Macau Special Administrative Region of China	Protein-energy malnutrition	0.142<br>(0.121 to 0.167)	39.88<br>(34.12 to 46.96)	0.0766<br>(0.0663 to 0.0897)	11.21<br>(9.69 to 13.12)
Anhui	Protein-energy malnutrition	258<br>(197 to 334)	435.28<br>(332.82 to 563.01)	12.8<br>(10.9 to 16.3)	20.97<br>(17.87 to 26.71)
Beijing	Protein-energy malnutrition	3.26<br>(2.48 to 4.31)	29.05<br>(22.13 to 38.46)	0.852<br>(0.698 to 1.04)	3.54<br>(2.9 to 4.33)
Chongqing	Protein-energy malnutrition	13.4<br>(9.03 to 18.7)	84.96<br>(57.2 to 118.53)	0.772<br>(0.585 to 1.52)	2.89<br>(2.19 to 5.69)
Fujian	Protein-energy malnutrition	76.2<br>(60.3 to 97.2)	240.93<br>(190.47 to 307.26)	7.03<br>(5.96 to 8.3)	17.83<br>(15.12 to 21.07)
Gansu	Protein-energy malnutrition	110<br>(81.1 to 149)	464.11<br>(342.88 to 628.12)	3.59<br>(2.75 to 5.15)	13.51<br>(10.34 to 19.39)
Guangdong	Protein-energy malnutrition	128<br>(101 to 157)	193.77<br>(154 to 238.83)	22.3<br>(18.8 to 26.7)	19.09<br>(16.13 to 22.89)
Guangxi	Protein-energy malnutrition	134<br>(102 to 170)	307.44<br>(234.62 to 391.63)	11.4<br>(8.78 to 17.2)	23.56<br>(18.16 to 35.57)
Guizhou	Protein-energy malnutrition	321<br>(236 to 424)	966.52<br>(709.73 to 1277.97)	9.14<br>(7.18 to 12.8)	27.01<br>(21.21 to 37.92)
Hainan	Protein-energy malnutrition	50.1<br>(37.5 to 65.2)	719.24<br>(538.94 to 935.54)	8.36<br>(6.58 to 10.7)	87.2<br>(68.58 to 111.74)

Hebei	Protein-energy malnutrition	39.2 (28.9 to 52.6)	61.54 (45.36 to 82.65)	8.28 (6.52 to 11.1)	10.83 (8.54 to 14.55)
Heilongjiang	Protein-energy malnutrition	12.1 (8.74 to 16.7)	33.1 (23.97 to 45.9)	1.3 (1.03 to 2.18)	3.31 (2.62 to 5.56)
Henan	Protein-energy malnutrition	99.1 (72.5 to 131)	112.31 (82.11 to 148.45)	4.61 (3.67 to 7.08)	4.79 (3.81 to 7.35)
Hubei	Protein-energy malnutrition	68.5 (50.4 to 91.3)	123.34 (90.72 to 164.32)	3.69 (3.04 to 5.07)	6.67 (5.5 to 9.16)
Hunan	Protein-energy malnutrition	70.3 (52.9 to 93.1)	113.05 (85.12 to 149.65)	3.89 (2.34 to 9.92)	5.79 (3.48 to 14.77)
Inner Mongolia	Protein-energy malnutrition	21.3 (15.8 to 28.1)	93.59 (69.53 to 123.63)	1.09 (0.88 to 1.36)	4.2 (3.4 to 5.25)
Jiangsu	Protein-energy malnutrition	57.9 (45.3 to 74.9)	84.18 (65.84 to 108.79)	6.05 (5.19 to 7.01)	7.41 (6.35 to 8.58)
Jiangxi	Protein-energy malnutrition	134 (99.7 to 179)	339.64 (252.27 to 452.16)	7.45 (6.04 to 9.74)	15.68 (12.71 to 20.48)
Jilin	Protein-energy malnutrition	23.5 (17.4 to 31.1)	90.47 (66.9 to 119.7)	2.46 (2.07 to 3.07)	8.83 (7.41 to 10.99)
Liaoning	Protein-energy malnutrition	13.6 (9.23 to 20.7)	33.39 (22.63 to 50.74)	2.4 (1.41 to 6.73)	5.37 (3.15 to 15.06)
Ningxia	Protein-energy malnutrition	16.4 (11.5 to 22.5)	331.96 (231.9 to 456.47)	0.689 (0.533 to 0.94)	9.82 (7.59 to 13.39)
Qinghai	Protein-energy malnutrition	15.6 (11.4 to 20.8)	329.17 (239.99 to 439.57)	0.791 (0.596 to 1.15)	12.6 (9.49 to 18.29)
Shaanxi	Protein-energy malnutrition	84.4 (63.3 to 109)	245.3 (184.02 to 317.61)	4.86 (3.93 to 6.2)	12.3 (9.95 to 15.69)
Shandong	Protein-energy malnutrition	22.6 (16.9 to 30.7)	26.42 (19.73 to 35.88)	1.75 (1.47 to 2.09)	1.77 (1.48 to 2.1)
Shanghai	Protein-energy malnutrition	1.65 (1.24 to 2.36)	11.78 (8.91 to 16.88)	0.392 (0.32 to 0.482)	1.4 (1.15 to 1.73)
Shanxi	Protein-energy malnutrition	30.2 (22.5 to 40.6)	99.88 (74.14 to 134.1)	1.83 (1.42 to 2.58)	4.74 (3.67 to 6.68)
Sichuan	Protein-energy malnutrition	247 (181 to 330)	221.3 (162.04 to 295.44)	6.02 (4.75 to 9.78)	7.21 (5.69 to 11.71)
Tianjin	Protein-energy malnutrition	2.09 (1.55 to 2.9)	22.73 (16.84 to 31.58)	0.359 (0.293 to 0.453)	2.35 (1.92 to 2.97)
Tibet	Protein-energy malnutrition	20.9 (15.5 to 27)	898.94 (666.36 to 1159.96)	2.7 (1.92 to 3.69)	78.08 (55.54 to 106.72)
Xinjiang	Protein-energy malnutrition	113 (83.8 to 147)	715.25 (531.3 to 931.62)	20.3 (15.2 to 26.7)	83.3 (62.5 to 109.76)
Yunnan	Protein-energy malnutrition	377 (276 to 492)	967.43 (707.87 to 1262.43)	17 (13.9 to 21.4)	34.65 (28.28 to 43.59)
Zhejiang	Protein-energy malnutrition	35.4 (26.6 to 46.6)	80.2 (60.39 to 105.69)	3.26 (2.7 to 4.2)	5.33 (4.41 to 6.87)
China	Iodine deficiency	138 (74.2 to 230)	11.5 (6.2 to 19.25)	150 (72.3 to 287)	10.6 (5.12 to 20.34)
Taiwan	Iodine deficiency	1.19 (0.628 to 2.04)	5.85 (3.08 to 9.99)	1.26 (0.508 to 2.67)	5.34 (2.15 to 11.34)
Hong Kong Special Administrative Region of China	Iodine deficiency	0.44 (0.214 to 0.8)	7.64 (3.7 to 13.88)	0.852 (0.414 to 1.6)	11.35 (5.51 to 21.34)
Macau Special Administrative Region of China	Iodine deficiency	0.0271 (0.0131 to 0.0494)	7.64 (3.68 to 13.91)	0.0735 (0.0354 to 0.139)	10.75 (5.17 to 20.28)
Anhui	Iodine deficiency	5.24 (2.79 to 8.86)	8.85 (4.71 to 14.95)	4.87 (2.34 to 9.21)	7.97 (3.83 to 15.06)
Beijing	Iodine deficiency	0.45 (0.176 to 0.855)	4.01 (1.57 to 7.62)	1.04 (0.452 to 2.15)	4.31 (1.88 to 8.92)
Chongqing	Iodine deficiency	0.963 (0.459 to 1.74)	6.1 (2.91 to 11.03)	1.2 (0.516 to 2.46)	4.5 (1.93 to 9.2)
Fujian	Iodine deficiency	2.76 (1.52 to 4.61)	8.72 (4.79 to 14.57)	3.04 (1.45 to 5.71)	7.7 (3.67 to 14.48)
Gansu	Iodine deficiency	4.9 (2.5 to 8.33)	20.71 (10.55 to 35.23)	5.51 (2.64 to 10)	20.74 (9.92 to 37.64)
Guangdong	Iodine deficiency	3.67 (1.72 to 6.42)	5.58 (2.61 to 9.76)	5.75 (2.67 to 11.9)	4.92 (2.29 to 10.2)
Guangxi	Iodine deficiency	2.89 (1.35 to 5.42)	6.64 (3.1 to 12.46)	1.98 (0.865 to 4.12)	4.1 (1.79 to 8.53)

Guizhou	Iodine deficiency	8.27<br>(4.43 to 13.3)	24.91<br>(13.34 to 40.22)	7.09<br>(3.34 to 13)	20.96<br>(9.88 to 38.3)
Hainan	Iodine deficiency	1.81<br>(0.971 to 3.04)	26.02<br>(13.94 to 43.65)	2.72<br>(1.27 to 4.98)	28.42<br>(13.25 to 51.94)
Hebei	Iodine deficiency	9.19<br>(4.87 to 15.2)	14.45<br>(7.65 to 23.82)	10.1<br>(4.91 to 18.5)	13.28<br>(6.43 to 24.21)
Heilongjiang	Iodine deficiency	4.85<br>(2.64 to 7.91)	13.3<br>(7.23 to 21.69)	5.34<br>(2.58 to 9.87)	13.6<br>(6.56 to 25.15)
Henan	Iodine deficiency	4.1<br>(1.94 to 7.52)	4.65<br>(2.2 to 8.52)	3.48<br>(1.44 to 7.16)	3.61<br>(1.49 to 7.44)
Hubei	Iodine deficiency	3.47<br>(1.66 to 6.15)	6.25<br>(2.98 to 11.06)	2.75<br>(1.21 to 5.63)	4.96<br>(2.19 to 10.18)
Hunan	Iodine deficiency	3.23<br>(1.43 to 6.03)	5.19<br>(2.3 to 9.7)	2.56<br>(1.06 to 5.37)	3.81<br>(1.57 to 8)
Inner Mongolia	Iodine deficiency	3.26<br>(1.75 to 5.41)	14.33<br>(7.68 to 23.78)	3.48<br>(1.66 to 6.43)	13.45<br>(6.42 to 24.82)
Jiangsu	Iodine deficiency	6.24<br>(3.35 to 10.4)	9.07<br>(4.87 to 15.07)	7.06<br>(3.39 to 13.3)	8.65<br>(4.15 to 16.3)
Jiangxi	Iodine deficiency	6.29<br>(3.46 to 10.4)	15.91<br>(8.74 to 26.38)	6.4<br>(3.07 to 11.9)	13.45<br>(6.46 to 25.08)
Jilin	Iodine deficiency	1.96<br>(1.05 to 3.37)	7.55<br>(4.06 to 12.98)	2.03<br>(0.966 to 4)	7.26<br>(3.46 to 14.35)
Liaoning	Iodine deficiency	3.79<br>(1.99 to 6.45)	9.3<br>(4.88 to 15.8)	4.28<br>(2.08 to 8.13)	9.59<br>(4.65 to 18.22)
Ningxia	Iodine deficiency	0.84<br>(0.46 to 1.37)	17<br>(9.31 to 27.76)	1.13<br>(0.54 to 2.12)	16.05<br>(7.69 to 30.23)
Qinghai	Iodine deficiency	0.599<br>(0.304 to 1.05)	12.65<br>(6.43 to 22.23)	0.61<br>(0.294 to 1.17)	9.71<br>(4.69 to 18.55)
Shaanxi	Iodine deficiency	6.43<br>(3.5 to 10.6)	18.69<br>(10.18 to 30.69)	7.08<br>(3.37 to 13)	17.93<br>(8.54 to 32.91)
Shandong	Iodine deficiency	15.5<br>(7.76 to 26.6)	18.07<br>(9.06 to 31.11)	19.4<br>(9.18 to 35.6)	19.56<br>(9.24 to 35.81)
Shanghai	Iodine deficiency	0.697<br>(0.325 to 1.25)	4.99<br>(2.33 to 8.94)	1.35<br>(0.61 to 2.82)	4.84<br>(2.18 to 10.11)
Shanxi	Iodine deficiency	2.9<br>(1.55 to 4.94)	9.57<br>(5.12 to 16.3)	3.18<br>(1.52 to 6.03)	8.24<br>(3.93 to 15.61)
Sichuan	Iodine deficiency	18<br>(9.68 to 28.8)	16.12<br>(8.67 to 25.8)	11.7<br>(5.66 to 21.6)	14.06<br>(6.78 to 25.92)
Tianjin	Iodine deficiency	0.753<br>(0.374 to 1.3)	8.19<br>(4.06 to 14.14)	1.3<br>(0.622 to 2.46)	8.52<br>(4.07 to 16.15)
Tibet	Iodine deficiency	1.4<br>(0.757 to 2.36)	60.2<br>(32.51 to 101.35)	2.64<br>(1.24 to 4.82)	76.49<br>(35.75 to 139.46)
Xinjiang	Iodine deficiency	6.45<br>(3.45 to 11)	40.88<br>(21.85 to 69.88)	12.5<br>(5.88 to 23.1)	51.36<br>(24.12 to 94.69)
Yunnan	Iodine deficiency	3.29<br>(1.63 to 5.62)	8.45<br>(4.19 to 14.43)	3.08<br>(1.49 to 6.12)	6.27<br>(3.03 to 12.44)
Zhejiang	Iodine deficiency	3.03<br>(1.57 to 5.03)	6.88<br>(3.56 to 11.41)	3.97<br>(1.9 to 7.8)	6.48<br>(3.11 to 12.74)
China	Vitamin A deficiency	1260<br>(786 to 1950)	105.37<br>(65.71 to 162.6)	263<br>(161 to 398)	18.64<br>(11.43 to 28.18)
Taiwan	Vitamin A deficiency	4.69<br>(1.68 to 10.1)	22.99<br>(8.23 to 49.64)	0.9<br>(0.227 to 2.2)	3.82<br>(0.96 to 9.33)
Hong Kong Special Administrative Region of China	Vitamin A deficiency	1.87<br>(0.8 to 3.96)	32.52<br>(13.88 to 68.67)	0.866<br>(0.313 to 1.84)	11.53<br>(4.17 to 24.52)
Macau Special Administrative Region of China	Vitamin A deficiency	0.154<br>(0.0543 to 0.346)	43.3<br>(15.29 to 97.4)	0.0716<br>(0.0276 to 0.16)	10.47<br>(4.04 to 23.42)
Anhui	Vitamin A deficiency	88.6<br>(47 to 152)	149.6<br>(79.36 to 256.29)	14.7<br>(6.68 to 28)	23.99<br>(10.92 to 45.85)
Beijing	Vitamin A deficiency	4.81<br>(2.14 to 9.5)	42.89<br>(19.07 to 84.7)	2.11<br>(0.775 to 5.16)	8.75<br>(3.21 to 21.4)
Chongqing	Vitamin A deficiency	12.4<br>(6.71 to 21.8)	78.49<br>(42.5 to 138.39)	6.67<br>(2.41 to 14.2)	24.95<br>(9 to 52.99)
Fujian	Vitamin A deficiency	37.7<br>(20.8 to 60.7)	119.3<br>(65.89 to 191.83)	7.44<br>(2.88 to 18.9)	18.87<br>(7.31 to 47.92)
Gansu	Vitamin A deficiency	28.8<br>(15.5 to 48)	121.64<br>(65.7 to 202.99)	8.01<br>(2.9 to 16.9)	30.13<br>(10.92 to 63.46)

Guangdong	Vitamin A deficiency	64.7 (34.3 to 111)	98.34 (52.12 to 168.11)	15.5 (6.78 to 38.1)	13.29 (5.8 to 32.6)
Guangxi	Vitamin A deficiency	49.6 (24.9 to 84.3)	114.1 (57.31 to 193.72)	8.62 (3.66 to 17.8)	17.83 (7.58 to 36.8)
Guizhou	Vitamin A deficiency	45.9 (25.5 to 74.7)	138.21 (76.94 to 225.12)	6.71 (2.67 to 14.1)	19.82 (7.88 to 41.71)
Hainan	Vitamin A deficiency	9.02 (4.73 to 14.8)	129.42 (67.96 to 212.32)	1.93 (0.828 to 4.47)	20.09 (8.64 to 46.63)
Hebei	Vitamin A deficiency	78.2 (45.1 to 125)	122.86 (70.82 to 196)	19.4 (7.63 to 44.6)	25.42 (9.98 to 58.32)
Heilongjiang	Vitamin A deficiency	29.3 (15.7 to 48.7)	80.25 (42.97 to 133.7)	5.25 (2.44 to 10.6)	13.37 (6.22 to 27.07)
Henan	Vitamin A deficiency	95.6 (51.1 to 163)	108.3 (57.86 to 184.37)	21.7 (8.41 to 47.2)	22.53 (8.73 to 49.06)
Hubei	Vitamin A deficiency	61.6 (32 to 104)	110.82 (57.6 to 187.3)	11.2 (4.69 to 22.2)	20.34 (8.48 to 40.16)
Hunan	Vitamin A deficiency	53.4 (28.6 to 91.3)	85.83 (45.92 to 146.9)	11.7 (4.95 to 25.5)	17.5 (7.38 to 37.95)
Inner Mongolia	Vitamin A deficiency	23.4 (12 to 37.8)	102.94 (52.57 to 166.43)	3.38 (1.38 to 7.51)	13.05 (5.33 to 29)
Jiangsu	Vitamin A deficiency	62 (30.4 to 113)	90.06 (44.21 to 163.87)	12 (4.39 to 26.6)	14.74 (5.38 to 32.54)
Jiangxi	Vitamin A deficiency	57.1 (31.6 to 95.8)	144.39 (79.9 to 242.36)	12.2 (5.18 to 24.5)	25.62 (10.9 to 51.59)
Jilin	Vitamin A deficiency	25.2 (14.1 to 40.9)	97.19 (54.35 to 157.48)	3.43 (1.63 to 6.54)	12.29 (5.86 to 23.45)
Liaoning	Vitamin A deficiency	27.4 (11.9 to 52.3)	67.27 (29.22 to 128.23)	6.26 (2.82 to 11.9)	14.03 (6.31 to 26.68)
Ningxia	Vitamin A deficiency	6.28 (3.17 to 10.9)	127.24 (64.21 to 220.63)	2.21 (0.934 to 4.58)	31.44 (13.31 to 65.24)
Qinghai	Vitamin A deficiency	5.41 (2.96 to 9.23)	114.12 (62.39 to 194.95)	1.82 (0.757 to 3.66)	29.04 (12.05 to 58.21)
Shaanxi	Vitamin A deficiency	40.6 (22.9 to 65.3)	117.85 (66.64 to 189.71)	8.22 (3.45 to 16.5)	20.8 (8.72 to 41.76)
Shandong	Vitamin A deficiency	89.5 (46 to 151)	104.61 (53.69 to 176.72)	19.4 (8.65 to 38.5)	19.52 (8.71 to 38.73)
Shanghai	Vitamin A deficiency	8.77 (4 to 15.9)	62.78 (28.61 to 114.01)	2.7 (1.02 to 6.67)	9.67 (3.64 to 23.87)
Shanxi	Vitamin A deficiency	38.5 (21.4 to 63)	127 (70.76 to 207.94)	8.63 (3.44 to 18.5)	22.34 (8.91 to 47.95)
Sichuan	Vitamin A deficiency	93.8 (52.2 to 159)	84.08 (46.76 to 142.88)	11.6 (5.18 to 23.5)	13.88 (6.21 to 28.16)
Tianjin	Vitamin A deficiency	6.96 (3.83 to 11.9)	75.72 (41.68 to 129.44)	1.79 (0.757 to 3.89)	11.73 (4.96 to 25.47)
Tibet	Vitamin A deficiency	4.84 (2.88 to 7.5)	207.72 (123.52 to 322.03)	2.61 (1.33 to 4.91)	75.48 (38.51 to 142.1)
Xinjiang	Vitamin A deficiency	19.7 (10.5 to 33.8)	124.73 (66.68 to 214.46)	6.96 (3.21 to 13.5)	28.56 (13.18 to 55.57)
Yunnan	Vitamin A deficiency	35.7 (19.9 to 56.6)	91.73 (50.96 to 145.12)	8.02 (3.83 to 15.5)	16.31 (7.79 to 31.6)
Zhejiang	Vitamin A deficiency	54.4 (31.7 to 88.8)	123.31 (71.86 to 201.4)	10.1 (4.7 to 20.3)	16.55 (7.68 to 33.12)
China	Dietary iron deficiency	3810 (2550 to 5600)	318.65 (213.33 to 467.78)	1230 (804 to 1860)	87.14 (56.91 to 131.48)
Taiwan	Dietary iron deficiency	25.4 (13.7 to 42.4)	124.43 (66.92 to 207.99)	15.3 (7.22 to 31.3)	65 (30.6 to 132.85)
Hong Kong Special Administrative Region of China	Dietary iron deficiency	9.01 (5.05 to 15.2)	156.32 (87.6 to 263.61)	6.37 (3.34 to 11.3)	84.86 (44.42 to 149.93)
Macau Special Administrative Region of China	Dietary iron deficiency	0.57 (0.311 to 1.02)	160.51 (87.6 to 286.49)	0.433 (0.205 to 0.854)	63.34 (29.94 to 124.84)
Anhui	Dietary iron deficiency	225 (136 to 344)	380.26 (230.21 to 581.06)	67.8 (35.9 to 116)	110.85 (58.68 to 188.96)
Beijing	Dietary iron deficiency	18.6 (10.8 to 30.1)	165.45 (95.94 to 268)	12.9 (6.64 to 23.7)	53.55 (27.56 to 98.23)
Chongqing	Dietary iron deficiency	59.9 (35.6 to 95.1)	379.78 (225.78 to 602.7)	36.3 (19.3 to 64.3)	135.85 (72.33 to 240.57)

Fujian	Dietary iron deficiency	67.9<br>(41.3 to 101)	214.57<br>(130.55 to 320.44)	26.8<br>(13 to 50)	67.93<br>(32.9 to 126.75)
Gansu	Dietary iron deficiency	50.4<br>(30.7 to 77.5)	212.92<br>(129.81 to 327.63)	25<br>(13.3 to 42.8)	93.97<br>(50.19 to 160.91)
Guangdong	Dietary iron deficiency	142<br>(88.6 to 213)	216.16<br>(134.59 to 323.64)	42.3<br>(21.1 to 81.3)	36.2<br>(18.1 to 69.58)
Guangxi	Dietary iron deficiency	117<br>(71.1 to 176)	268.69<br>(163.4 to 404.43)	40<br>(20.3 to 72.1)	82.77<br>(42.09 to 149.24)
Guizhou	Dietary iron deficiency	96.7<br>(61.8 to 146)	291.34<br>(186.26 to 441.26)	28.9<br>(15.3 to 54.9)	85.56<br>(45.29 to 162.27)
Hainan	Dietary iron deficiency	33.7<br>(20.1 to 51.3)	484.2<br>(288.47 to 735.99)	7.71<br>(4.06 to 13.4)	80.4<br>(42.34 to 139.71)
Hebei	Dietary iron deficiency	176<br>(111 to 261)	276.52<br>(175.1 to 409.63)	55.5<br>(30.1 to 104)	72.68<br>(39.36 to 135.51)
Heilongjiang	Dietary iron deficiency	107<br>(64.8 to 172)	294.37<br>(177.75 to 472.56)	40.6<br>(20.9 to 72.7)	103.37<br>(53.36 to 185.19)
Henan	Dietary iron deficiency	265<br>(165 to 416)	300.75<br>(186.52 to 470.98)	72<br>(37.1 to 127)	74.77<br>(38.56 to 131.97)
Hubei	Dietary iron deficiency	273<br>(171 to 416)	492.12<br>(308.05 to 749.48)	46.8<br>(25 to 82)	84.6<br>(45.2 to 148.19)
Hunan	Dietary iron deficiency	163<br>(101 to 246)	262.22<br>(162.24 to 395.47)	51.4<br>(27.7 to 92.1)	76.56<br>(41.31 to 137.13)
Inner Mongolia	Dietary iron deficiency	30.1<br>(16.3 to 54.5)	132.39<br>(71.84 to 239.8)	18.2<br>(9.64 to 32.7)	70.19<br>(37.23 to 126.39)
Jiangsu	Dietary iron deficiency	352<br>(226 to 528)	511.14<br>(328.64 to 766.87)	77.6<br>(37.2 to 149)	95.08<br>(45.57 to 182.46)
Jiangxi	Dietary iron deficiency	101<br>(63.6 to 153)	254.67<br>(160.96 to 386.72)	56.9<br>(30.8 to 94.4)	119.6<br>(64.72 to 198.64)
Jilin	Dietary iron deficiency	68.4<br>(41.5 to 107)	263.71<br>(159.83 to 413.09)	25.6<br>(14.2 to 43.7)	91.75<br>(50.9 to 156.52)
Liaoning	Dietary iron deficiency	112<br>(66.7 to 175)	274.23<br>(163.6 to 429.64)	44.8<br>(23.8 to 80.9)	100.45<br>(53.41 to 181.28)
Ningxia	Dietary iron deficiency	10.6<br>(6.47 to 16.8)	213.71<br>(131 to 340.72)	6.56<br>(3.66 to 11)	93.41<br>(52.16 to 157.07)
Qinghai	Dietary iron deficiency	7.81<br>(4.75 to 11.9)	164.96<br>(100.27 to 251.84)	6.79<br>(3.71 to 11)	108.15<br>(58.98 to 174.79)
Shaanxi	Dietary iron deficiency	173<br>(115 to 257)	502.85<br>(332.86 to 745.48)	55.4<br>(28.4 to 98.2)	140.33<br>(71.99 to 248.66)
Shandong	Dietary iron deficiency	263<br>(161 to 402)	307.79<br>(188.58 to 469.51)	102<br>(55.4 to 167)	102.66<br>(55.76 to 168.54)
Shanghai	Dietary iron deficiency	76.5<br>(47.1 to 115)	547.31<br>(337.21 to 822.16)	29.8<br>(14.6 to 56.4)	106.78<br>(52.08 to 201.94)
Shanxi	Dietary iron deficiency	117<br>(75 to 175)	387.83<br>(247.54 to 579.39)	44.1<br>(23.5 to 75.5)	114.17<br>(60.71 to 195.59)
Sichuan	Dietary iron deficiency	382<br>(250 to 568)	341.97<br>(223.83 to 508.61)	82.9<br>(48.4 to 137)	99.28<br>(57.97 to 164.2)
Tianjin	Dietary iron deficiency	22.6<br>(14.4 to 33.9)	245.32<br>(156.65 to 368.47)	13.6<br>(6.89 to 24.7)	89.06<br>(45.12 to 162.13)
Tibet	Dietary iron deficiency	5.22<br>(3.25 to 7.68)	224.07<br>(139.38 to 329.57)	5.92<br>(3.54 to 9.7)	171.19<br>(102.55 to 280.59)
Xinjiang	Dietary iron deficiency	39.4<br>(24.9 to 59.4)	249.54<br>(157.82 to 376.66)	26.6<br>(14.3 to 46.5)	109.15<br>(58.54 to 190.69)
Yunnan	Dietary iron deficiency	39.8<br>(21.1 to 74.8)	102.21<br>(54.09 to 191.97)	27.7<br>(16.1 to 48)	56.4<br>(32.66 to 97.66)
Zhejiang	Dietary iron deficiency	208<br>(137 to 303)	470.68<br>(311.33 to 687.87)	45.4<br>(25.3 to 78.5)	74.15<br>(41.42 to 128.31)
China	Other nutritional deficiencies	186<br>(159 to 220)	15.54<br>(13.28 to 18.34)	65<br>(59.5 to 75.6)	4.6<br>(4.21 to 5.35)
Taiwan	Other nutritional deficiencies	0.446<br>(0.236 to 1.32)	2.19<br>(1.16 to 6.49)	0.177<br>(0.0513 to 0.279)	0.75<br>(0.22 to 1.18)
Hong Kong Special Administrative Region of China	Other nutritional deficiencies	0.252<br>(0.211 to 0.294)	4.37<br>(3.67 to 5.1)	0.146<br>(0.122 to 0.176)	1.95<br>(1.62 to 2.34)
Macau Special Administrative Region of China	Other nutritional deficiencies	0.0071<br>(0.00571 to 0.00947)	2<br>(1.61 to 2.67)	0.00509<br>(0.00384 to 0.00649)	0.74<br>(0.56 to 0.95)
Anhui	Other nutritional deficiencies	5.43<br>(3.48 to 8.09)	9.17<br>(5.87 to 13.65)	1.9<br>(1.46 to 3.07)	3.11<br>(2.39 to 5.02)

Beijing	Other nutritional deficiencies	0.265 (0.183 to 0.566)	2.36 (1.63 to 5.04)	0.34 (0.272 to 0.434)	1.41 (1.13 to 1.8)
Chongqing	Other nutritional deficiencies	2.28 (1.49 to 3.29)	14.46 (9.46 to 20.83)	0.785 (0.627 to 1.07)	2.94 (2.35 to 3.99)
Fujian	Other nutritional deficiencies	2.52 (1.73 to 3.59)	7.97 (5.46 to 11.34)	1.5 (1.25 to 1.9)	3.82 (3.16 to 4.82)
Gansu	Other nutritional deficiencies	9.62 (6.03 to 14.3)	40.69 (25.51 to 60.49)	2.47 (1.91 to 3.19)	9.29 (7.19 to 11.99)
Guangdong	Other nutritional deficiencies	6.44 (4.35 to 9.71)	9.79 (6.62 to 14.75)	6.25 (5.12 to 7.76)	5.35 (4.38 to 6.65)
Guangxi	Other nutritional deficiencies	7.34 (5.24 to 9.82)	16.88 (12.04 to 22.58)	3.92 (2.97 to 5.78)	8.11 (6.14 to 11.97)
Guizhou	Other nutritional deficiencies	14.7 (10.4 to 20.4)	44.18 (31.47 to 61.61)	3.34 (2.58 to 4.73)	9.88 (7.62 to 14)
Hainan	Other nutritional deficiencies	3.16 (2.08 to 4.59)	45.36 (29.84 to 65.84)	0.866 (0.611 to 1.25)	9.03 (6.37 to 13.08)
Hebei	Other nutritional deficiencies	3.34 (2.07 to 5.07)	5.26 (3.26 to 7.96)	1.44 (1.07 to 2.1)	1.88 (1.4 to 2.75)
Heilongjiang	Other nutritional deficiencies	3.51 (2.43 to 5.04)	9.63 (6.66 to 13.83)	0.782 (0.621 to 1.16)	1.99 (1.58 to 2.95)
Henan	Other nutritional deficiencies	6.87 (4.45 to 10)	7.78 (5.04 to 11.38)	2.14 (1.7 to 2.98)	2.22 (1.76 to 3.09)
Hubei	Other nutritional deficiencies	5.06 (3.45 to 7.42)	9.11 (6.21 to 13.36)	1.91 (1.57 to 2.56)	3.45 (2.83 to 4.63)
Hunan	Other nutritional deficiencies	17.3 (12.6 to 23.5)	27.79 (20.2 to 37.76)	4.56 (3.64 to 6.84)	6.8 (5.43 to 10.19)
Inner Mongolia	Other nutritional deficiencies	2.53 (1.74 to 3.6)	11.11 (7.63 to 15.84)	0.824 (0.661 to 1.03)	3.18 (2.55 to 3.98)
Jiangsu	Other nutritional deficiencies	2.86 (2.03 to 4.25)	4.16 (2.95 to 6.17)	1.78 (1.5 to 2.14)	2.19 (1.84 to 2.62)
Jiangxi	Other nutritional deficiencies	5.71 (3.79 to 8.35)	14.44 (9.58 to 21.11)	2.15 (1.63 to 2.9)	4.53 (3.44 to 6.09)
Jilin	Other nutritional deficiencies	3.58 (2.4 to 5.24)	13.8 (9.23 to 20.21)	0.856 (0.684 to 1.09)	3.07 (2.45 to 3.9)
Liaoning	Other nutritional deficiencies	3.18 (2.04 to 4.92)	7.8 (4.99 to 12.06)	1.33 (0.871 to 3.13)	2.99 (1.95 to 7)
Ningxia	Other nutritional deficiencies	0.933 (0.605 to 1.46)	18.89 (12.25 to 29.56)	0.3 (0.225 to 0.408)	4.28 (3.21 to 5.81)
Qinghai	Other nutritional deficiencies	1.1 (0.718 to 1.63)	23.23 (15.16 to 34.46)	0.436 (0.326 to 0.599)	6.94 (5.19 to 9.54)
Shaanxi	Other nutritional deficiencies	8 (5.36 to 11.6)	23.25 (15.56 to 33.65)	3.28 (2.64 to 4.09)	8.31 (6.68 to 10.34)
Shandong	Other nutritional deficiencies	3.3 (2.41 to 4.68)	3.85 (2.82 to 5.47)	1.66 (1.41 to 1.94)	1.67 (1.42 to 1.95)
Shanghai	Other nutritional deficiencies	0.2 (0.147 to 0.374)	1.43 (1.05 to 2.67)	0.234 (0.189 to 0.286)	0.84 (0.68 to 1.02)
Shanxi	Other nutritional deficiencies	4.03 (2.72 to 5.8)	13.3 (8.97 to 19.17)	1.7 (1.36 to 2.22)	4.4 (3.51 to 5.75)
Sichuan	Other nutritional deficiencies	19 (12.4 to 28.3)	17 (11.1 to 25.36)	3.48 (2.77 to 4.91)	4.17 (3.32 to 5.88)
Tianjin	Other nutritional deficiencies	0.235 (0.164 to 0.397)	2.55 (1.79 to 4.31)	0.226 (0.182 to 0.285)	1.48 (1.19 to 1.87)
Tibet	Other nutritional deficiencies	1.93 (1.39 to 2.67)	82.89 (59.7 to 114.57)	1.78 (1.3 to 2.48)	51.6 (37.54 to 71.69)
Xinjiang	Other nutritional deficiencies	4.37 (2.97 to 6.36)	27.69 (18.84 to 40.34)	1.86 (1.28 to 2.74)	7.64 (5.26 to 11.25)
Yunnan	Other nutritional deficiencies	34.2 (23.5 to 48.6)	87.68 (60.32 to 124.61)	9.24 (7.47 to 11.6)	18.79 (15.18 to 23.49)
Zhejiang	Other nutritional deficiencies	2.87 (1.93 to 4.29)	6.5 (4.37 to 9.73)	1.47 (1.2 to 1.86)	2.4 (1.96 to 3.04)
China	Sexually transmitted infections excluding HIV	966 (470 to 1760)	80.67 (39.28 to 146.94)	486 (267 to 818)	34.43 (18.9 to 57.93)
Taiwan	Sexually transmitted infections excluding HIV	5.86 (3.24 to 9.78)	28.72 (15.87 to 47.93)	5.3 (3.03 to 9.1)	22.46 (12.87 to 38.58)
Hong Kong Special Administrative Region of China	Sexually transmitted infections excluding HIV	2.78 (1.51 to 4.61)	48.26 (26.16 to 79.92)	2.06 (1.19 to 3.25)	27.41 (15.88 to 43.26)

Macau Special Administrative Region of China	Sexually transmitted infections excluding HIV	0.269<br>(0.148 to 0.442)	75.72<br>(41.69 to 124.48)	0.279<br>(0.161 to 0.424)	40.8<br>(23.59 to 61.93)
Anhui	Sexually transmitted infections excluding HIV	58.9<br>(26.8 to 112)	99.4<br>(45.19 to 189.44)	19.7<br>(10.6 to 33.7)	32.29<br>(17.33 to 55.12)
Beijing	Sexually transmitted infections excluding HIV	6.75<br>(3.74 to 11.3)	60.21<br>(33.39 to 100.32)	11.2<br>(6.07 to 19.5)	46.61<br>(25.19 to 80.8)
Chongqing	Sexually transmitted infections excluding HIV	13.5<br>(6.46 to 24.7)	85.34<br>(40.94 to 156.79)	7.79<br>(4.51 to 12.7)	29.14<br>(16.89 to 47.36)
Fujian	Sexually transmitted infections excluding HIV	23.3<br>(11.9 to 41.4)	73.52<br>(37.65 to 130.73)	14.4<br>(8.44 to 23.9)	36.46<br>(21.41 to 60.77)
Gansu	Sexually transmitted infections excluding HIV	26.8<br>(12 to 52.8)	113.15<br>(50.74 to 223.07)	9.03<br>(4.73 to 15.9)	33.98<br>(17.8 to 60)
Guangdong	Sexually transmitted infections excluding HIV	51.2<br>(25.2 to 91.9)	77.84<br>(38.33 to 139.66)	47.6<br>(25.9 to 81.8)	40.73<br>(22.15 to 69.99)
Guangxi	Sexually transmitted infections excluding HIV	24.6<br>(13.4 to 42.8)	56.67<br>(30.77 to 98.4)	14.7<br>(8.61 to 24.8)	30.38<br>(17.81 to 51.33)
Guizhou	Sexually transmitted infections excluding HIV	25.1<br>(12.9 to 45.4)	75.63<br>(38.92 to 136.91)	8.12<br>(4.74 to 13.3)	24.01<br>(14.01 to 39.45)
Hainan	Sexually transmitted infections excluding HIV	5.33<br>(2.49 to 10.1)	76.49<br>(35.77 to 145.13)	3.7<br>(1.88 to 6.84)	38.56<br>(19.56 to 71.32)
Hebei	Sexually transmitted infections excluding HIV	48.1<br>(22.6 to 90.8)	75.6<br>(35.49 to 142.69)	26.3<br>(13.3 to 48.6)	34.4<br>(17.37 to 63.56)
Heilongjiang	Sexually transmitted infections excluding HIV	21.9<br>(10.8 to 38.9)	60.01<br>(29.54 to 106.69)	9.79<br>(5.53 to 16.3)	24.93<br>(14.1 to 41.65)
Henan	Sexually transmitted infections excluding HIV	69.4<br>(31.8 to 130)	78.61<br>(36.02 to 147.8)	26.9<br>(14.5 to 44.9)	27.88<br>(15.01 to 46.63)
Hubei	Sexually transmitted infections excluding HIV	39<br>(19.4 to 74)	70.22<br>(34.91 to 133.24)	14.2<br>(8.08 to 23.7)	25.63<br>(14.6 to 42.82)
Hunan	Sexually transmitted infections excluding HIV	46.2<br>(26.8 to 78.4)	74.3<br>(43.09 to 126.03)	17.9<br>(10.8 to 28.5)	26.7<br>(16.13 to 42.45)
Inner Mongolia	Sexually transmitted infections excluding HIV	19<br>(8.91 to 35.4)	83.77<br>(39.18 to 155.52)	8.49<br>(4.5 to 14.6)	32.79<br>(17.39 to 56.4)
Jiangsu	Sexually transmitted infections excluding HIV	41.5<br>(20.7 to 73.8)	60.27<br>(30.14 to 107.2)	22.9<br>(13.2 to 37.5)	28.07<br>(16.11 to 45.88)
Jiangxi	Sexually transmitted infections excluding HIV	34.7<br>(16.9 to 65.1)	87.85<br>(42.63 to 164.78)	17<br>(9.04 to 30.4)	35.84<br>(19.02 to 63.9)
Jilin	Sexually transmitted infections excluding HIV	20.4<br>(9.84 to 37.8)	78.56<br>(37.92 to 145.56)	8.72<br>(4.68 to 15.1)	31.28<br>(16.8 to 54.23)
Liaoning	Sexually transmitted infections excluding HIV	21.7<br>(11.1 to 37.7)	53.25<br>(27.11 to 92.44)	10.7<br>(6.32 to 17.8)	24.07<br>(14.14 to 39.88)
Ningxia	Sexually transmitted infections excluding HIV	4.95<br>(2.16 to 9.92)	100.13<br>(43.65 to 200.81)	2.49<br>(1.24 to 4.51)	35.46<br>(17.68 to 64.2)
Qinghai	Sexually transmitted infections excluding HIV	5.23<br>(2.4 to 9.92)	110.5<br>(50.74 to 209.34)	2.39<br>(1.3 to 4.13)	38.07<br>(20.64 to 65.8)
Shaanxi	Sexually transmitted infections excluding HIV	26.9<br>(12.4 to 49.4)	78.24<br>(36.17 to 143.59)	13.6<br>(7.21 to 24.4)	34.43<br>(18.24 to 61.81)
Shandong	Sexually transmitted infections excluding HIV	49.4<br>(27.2 to 84.4)	57.76<br>(31.74 to 98.59)	30.6<br>(19.5 to 46.7)	30.77<br>(19.6 to 46.97)
Shanghai	Sexually transmitted infections excluding HIV	25.2<br>(11.7 to 44.9)	180.61<br>(83.95 to 321.09)	27<br>(12.9 to 50.6)	96.56<br>(46.15 to 181.06)
Shanxi	Sexually transmitted infections excluding HIV	24.6<br>(11.8 to 44.4)	81.08<br>(39.01 to 146.49)	13.4<br>(7 to 23.2)	34.69<br>(18.11 to 60.11)
Sichuan	Sexually transmitted infections excluding HIV	114<br>(50.8 to 216)	101.94<br>(45.51 to 193.49)	29.9<br>(15.7 to 51.6)	35.79<br>(18.75 to 61.84)
Tianjin	Sexually transmitted infections excluding HIV	5.87<br>(2.89 to 10.4)	63.84<br>(31.45 to 113.57)	5.64<br>(2.93 to 10.2)	36.98<br>(19.23 to 66.68)
Tibet	Sexually transmitted infections excluding HIV	2.35<br>(1.21 to 4.29)	100.76<br>(51.83 to 184.17)	2<br>(1.01 to 3.83)	57.85<br>(29.3 to 110.73)
Xinjiang	Sexually transmitted infections excluding HIV	19<br>(8.46 to 35.4)	120.58<br>(53.66 to 224.31)	12.6<br>(5.76 to 25.5)	51.71<br>(23.65 to 104.63)
Yunnan	Sexually transmitted infections excluding HIV	52.2<br>(22.7 to 100)	134.07<br>(58.25 to 256.67)	23.7<br>(11.5 to 44)	48.15<br>(23.46 to 89.53)
Zhejiang	Sexually transmitted infections excluding HIV	35.5<br>(17.3 to 66.4)	80.55<br>(39.31 to 150.65)	21.6<br>(12.1 to 36.6)	35.23<br>(19.84 to 59.87)
China	Acute hepatitis	1160<br>(798 to 1300)	96.54<br>(66.63 to 108.6)	424<br>(358 to 543)	30.01<br>(25.37 to 38.46)
Taiwan	Acute hepatitis	3.47<br>(2.52 to 4.82)	16.99<br>(12.37 to 23.6)	9.73<br>(8.06 to 11.5)	41.26<br>(34.17 to 48.62)

Hong Kong Special Administrative Region of China	Acute hepatitis	1.52<br>(1.21 to 2.14)	26.4<br>(21.03 to 37.1)	1.43<br>(1.12 to 1.93)	19.1<br>(14.92 to 25.66)
Macau Special Administrative Region of China	Acute hepatitis	0.154<br>(0.104 to 0.188)	43.46<br>(29.43 to 52.89)	0.151<br>(0.107 to 0.201)	22.08<br>(15.68 to 29.45)
Anhui	Acute hepatitis	49.7<br>(39.5 to 61.3)	83.93<br>(66.61 to 103.5)	15.5<br>(11.9 to 23.6)	25.42<br>(19.46 to 38.64)
Beijing	Acute hepatitis	3.68<br>(2.89 to 4.9)	32.78<br>(25.81 to 43.67)	3.1<br>(2.2 to 5.1)	12.84<br>(9.13 to 21.17)
Chongqing	Acute hepatitis	14.6<br>(10.3 to 18)	92.79<br>(65.58 to 114.29)	6.45<br>(4.95 to 9.06)	24.13<br>(18.54 to 33.91)
Fujian	Acute hepatitis	13.4<br>(11.1 to 19.4)	42.5<br>(34.94 to 61.25)	7.26<br>(5.04 to 12.7)	18.41<br>(12.8 to 32.13)
Gansu	Acute hepatitis	61.2<br>(30.2 to 79.5)	258.81<br>(127.69 to 336.11)	12.9<br>(9.69 to 15.5)	48.42<br>(36.46 to 58.45)
Guangdong	Acute hepatitis	57.5<br>(35 to 72.1)	87.4<br>(53.11 to 109.59)	28.5<br>(22.5 to 36.9)	24.38<br>(19.26 to 31.58)
Guangxi	Acute hepatitis	45.3<br>(30.2 to 55.5)	104.22<br>(69.39 to 127.56)	17.6<br>(13.6 to 24.5)	36.45<br>(28.2 to 50.7)
Guizhou	Acute hepatitis	48.1<br>(33.1 to 58.3)	144.83<br>(99.63 to 175.73)	15.4<br>(12.2 to 19.1)	45.42<br>(36.14 to 56.47)
Hainan	Acute hepatitis	7.54<br>(4.47 to 9.68)	108.18<br>(64.21 to 138.89)	3.79<br>(2.83 to 4.76)	39.48<br>(29.56 to 49.59)
Hebei	Acute hepatitis	43.5<br>(30.1 to 52.7)	68.29<br>(47.34 to 82.85)	24.3<br>(19.1 to 34.5)	31.77<br>(24.94 to 45.17)
Heilongjiang	Acute hepatitis	20.4<br>(16.7 to 25.7)	55.97<br>(45.83 to 70.55)	10.9<br>(8.05 to 17.9)	27.85<br>(20.51 to 45.61)
Henan	Acute hepatitis	62.1<br>(49.5 to 75.5)	70.34<br>(56.03 to 85.51)	26.3<br>(20.2 to 39.7)	27.33<br>(21.02 to 41.19)
Hubei	Acute hepatitis	44.7<br>(32.7 to 52.9)	80.38<br>(58.93 to 95.16)	17.5<br>(13.7 to 24.3)	31.63<br>(24.77 to 43.95)
Hunan	Acute hepatitis	76<br>(44.6 to 92.6)	122.24<br>(71.77 to 148.83)	31.8<br>(22.9 to 39.5)	47.38<br>(34.17 to 58.83)
Inner Mongolia	Acute hepatitis	14<br>(11.2 to 19.5)	61.71<br>(49.17 to 85.54)	5.63<br>(4.03 to 10.1)	21.75<br>(15.57 to 39.11)
Jiangsu	Acute hepatitis	36.5<br>(30 to 44.5)	52.99<br>(43.63 to 64.61)	16.3<br>(12.3 to 24.9)	19.96<br>(15.05 to 30.51)
Jiangxi	Acute hepatitis	85.9<br>(42.9 to 111)	217.21<br>(108.41 to 280.33)	20.7<br>(15 to 25.4)	43.65<br>(31.54 to 53.5)
Jilin	Acute hepatitis	21.2<br>(14.6 to 26)	81.87<br>(56.37 to 100.26)	8.06<br>(6.41 to 11.1)	28.9<br>(22.97 to 39.78)
Liaoning	Acute hepatitis	23.5<br>(18.2 to 28.8)	57.62<br>(44.61 to 70.61)	11.5<br>(8.65 to 18.2)	25.79<br>(19.37 to 40.77)
Ningxia	Acute hepatitis	5.47<br>(4.07 to 6.95)	110.67<br>(82.32 to 140.67)	1.88<br>(1.44 to 2.61)	26.73<br>(20.56 to 37.17)
Qinghai	Acute hepatitis	10.5<br>(5.62 to 13.6)	222.65<br>(118.71 to 287.26)	3.35<br>(2.66 to 4.12)	53.36<br>(42.26 to 65.61)
Shaanxi	Acute hepatitis	33.2<br>(19.8 to 42.5)	96.56<br>(57.56 to 123.39)	14.4<br>(11.5 to 17.9)	36.53<br>(28.99 to 45.37)
Shandong	Acute hepatitis	52.4<br>(41.9 to 64.5)	61.25<br>(49 to 75.36)	22<br>(16.2 to 35.1)	22.17<br>(16.28 to 35.33)
Shanghai	Acute hepatitis	7.62<br>(4.83 to 9.24)	54.54<br>(34.6 to 66.14)	4.95<br>(3.81 to 6.59)	17.72<br>(13.63 to 23.59)
Shanxi	Acute hepatitis	28.6<br>(18.6 to 35.2)	94.53<br>(61.43 to 116.19)	9.98<br>(8.09 to 13.2)	25.83<br>(20.94 to 34.29)
Sichuan	Acute hepatitis	117<br>(80.2 to 145)	104.48<br>(71.84 to 129.96)	29.2<br>(23.3 to 37.8)	35.03<br>(27.9 to 45.33)
Tianjin	Acute hepatitis	3.3<br>(2.66 to 4.57)	35.83<br>(28.91 to 49.66)	2.5<br>(1.73 to 4.54)	16.39<br>(11.32 to 29.73)
Tibet	Acute hepatitis	16.1<br>(3.89 to 21.5)	692.58<br>(166.96 to 921.91)	4.65<br>(2.17 to 5.98)	134.44<br>(62.72 to 173.14)
Xinjiang	Acute hepatitis	13.2<br>(10.2 to 16.1)	83.87<br>(64.65 to 102)	7.79<br>(6.02 to 10.8)	31.99<br>(24.72 to 44.39)
Yunnan	Acute hepatitis	68.4<br>(43.5 to 85)	175.59<br>(111.57 to 218.16)	20.1<br>(16.2 to 26.6)	40.87<br>(32.95 to 54.12)
Zhejiang	Acute hepatitis	69.3<br>(22.8 to 88.8)	157.16<br>(51.65 to 201.37)	17.8<br>(13.7 to 21.9)	29.11<br>(22.4 to 35.75)

China	Leprosy	1.65<br>(1.1 to 2.37)	0.14<br>(0.09 to 0.2)	1.27<br>(0.852 to 1.84)	0.09<br>(0.06 to 0.13)
Taiwan	Leprosy	0<br>(0 to 0)	0<br>(0 to 0)	4.7e-06<br>(3.01e-06 to 6.99e-06)	0<br>(0 to 0)
Hong Kong Special Administrative Region of China	Leprosy	0.00405<br>(0.000397 to 0.0132)	0.07<br>(0.01 to 0.23)	0.00424<br>(0.00156 to 0.00992)	0.06<br>(0.02 to 0.13)
Macau Special Administrative Region of China	Leprosy	0.000685<br>(5.97e-05 to 0.00207)	0.19<br>(0.02 to 0.58)	0.00105<br>(0.000378 to 0.00236)	0.15<br>(0.06 to 0.34)
Anhui	Leprosy	0.0806<br>(0.0528 to 0.118)	0.14<br>(0.09 to 0.2)	0.06<br>(0.039 to 0.0868)	0.1<br>(0.06 to 0.14)
Beijing	Leprosy	0.0163<br>(0.0106 to 0.0238)	0.15<br>(0.09 to 0.21)	0.0195<br>(0.013 to 0.0282)	0.08<br>(0.05 to 0.12)
Chongqing	Leprosy	0.0232<br>(0.015 to 0.034)	0.15<br>(0.1 to 0.22)	0.0269<br>(0.0178 to 0.0393)	0.1<br>(0.07 to 0.15)
Fujian	Leprosy	0.0424<br>(0.028 to 0.0616)	0.13<br>(0.09 to 0.19)	0.0357<br>(0.0237 to 0.0524)	0.09<br>(0.06 to 0.13)
Gansu	Leprosy	0.0309<br>(0.0201 to 0.0451)	0.13<br>(0.09 to 0.19)	0.0242<br>(0.016 to 0.0352)	0.09<br>(0.06 to 0.13)
Guangdong	Leprosy	0.0896<br>(0.059 to 0.129)	0.14<br>(0.09 to 0.2)	0.0919<br>(0.061 to 0.132)	0.08<br>(0.05 to 0.11)
Guangxi	Leprosy	0.0587<br>(0.0383 to 0.0866)	0.13<br>(0.09 to 0.2)	0.0417<br>(0.0277 to 0.0609)	0.09<br>(0.06 to 0.13)
Guizhou	Leprosy	0.0441<br>(0.0291 to 0.0651)	0.13<br>(0.09 to 0.2)	0.0307<br>(0.0204 to 0.045)	0.09<br>(0.06 to 0.13)
Hainan	Leprosy	0.0094<br>(0.00595 to 0.0137)	0.13<br>(0.09 to 0.2)	0.00847<br>(0.00555 to 0.0122)	0.09<br>(0.06 to 0.13)
Hebei	Leprosy	0.0889<br>(0.0583 to 0.129)	0.14<br>(0.09 to 0.2)	0.0658<br>(0.0436 to 0.0962)	0.09<br>(0.06 to 0.13)
Heilongjiang	Leprosy	0.0489<br>(0.0314 to 0.0717)	0.13<br>(0.09 to 0.2)	0.041<br>(0.0269 to 0.0599)	0.1<br>(0.07 to 0.15)
Henan	Leprosy	0.121<br>(0.0789 to 0.18)	0.14<br>(0.09 to 0.2)	0.0802<br>(0.0529 to 0.117)	0.08<br>(0.05 to 0.12)
Hubei	Leprosy	0.0755<br>(0.0491 to 0.11)	0.14<br>(0.09 to 0.2)	0.0546<br>(0.036 to 0.0791)	0.1<br>(0.07 to 0.14)
Hunan	Leprosy	0.0856<br>(0.0567 to 0.126)	0.14<br>(0.09 to 0.2)	0.0608<br>(0.0395 to 0.0885)	0.09<br>(0.06 to 0.13)
Inner Mongolia	Leprosy	0.0305<br>(0.0198 to 0.0444)	0.13<br>(0.09 to 0.2)	0.0253<br>(0.0166 to 0.0368)	0.1<br>(0.06 to 0.14)
Jiangsu	Leprosy	0.0976<br>(0.0629 to 0.142)	0.14<br>(0.09 to 0.21)	0.0766<br>(0.051 to 0.11)	0.09<br>(0.06 to 0.13)
Jiangxi	Leprosy	0.0523<br>(0.0337 to 0.0765)	0.13<br>(0.09 to 0.19)	0.0407<br>(0.027 to 0.0589)	0.09<br>(0.06 to 0.12)
Jilin	Leprosy	0.0352<br>(0.0229 to 0.0518)	0.14<br>(0.09 to 0.2)	0.0289<br>(0.0191 to 0.0418)	0.1<br>(0.07 to 0.15)
Liaoning	Leprosy	0.0577<br>(0.0378 to 0.0851)	0.14<br>(0.09 to 0.21)	0.0459<br>(0.0305 to 0.0663)	0.1<br>(0.07 to 0.15)
Ningxia	Leprosy	0.00634<br>(0.00413 to 0.00921)	0.13<br>(0.08 to 0.19)	0.00604<br>(0.00398 to 0.00878)	0.09<br>(0.06 to 0.13)
Qinghai	Leprosy	0.00606<br>(0.00394 to 0.00891)	0.13<br>(0.08 to 0.19)	0.00523<br>(0.00345 to 0.0076)	0.08<br>(0.05 to 0.12)
Shaanxi	Leprosy	0.0474<br>(0.0308 to 0.0695)	0.14<br>(0.09 to 0.2)	0.0363<br>(0.0241 to 0.0531)	0.09<br>(0.06 to 0.13)
Shandong	Leprosy	0.12<br>(0.0787 to 0.175)	0.14<br>(0.09 to 0.2)	0.0923<br>(0.061 to 0.134)	0.09<br>(0.06 to 0.13)
Shanghai	Leprosy	0.0217<br>(0.0142 to 0.0315)	0.16<br>(0.1 to 0.23)	0.0234<br>(0.0154 to 0.034)	0.08<br>(0.06 to 0.12)
Shanxi	Leprosy	0.0415<br>(0.0268 to 0.0618)	0.14<br>(0.09 to 0.2)	0.0338<br>(0.0221 to 0.049)	0.09<br>(0.06 to 0.13)
Sichuan	Leprosy	0.158<br>(0.101 to 0.237)	0.14<br>(0.09 to 0.21)	0.0821<br>(0.0546 to 0.119)	0.1<br>(0.07 to 0.14)
Tianjin	Leprosy	0.0133<br>(0.00873 to 0.0197)	0.15<br>(0.09 to 0.21)	0.013<br>(0.00867 to 0.0188)	0.09<br>(0.06 to 0.12)
Tibet	Leprosy	0.00298<br>(0.00194 to 0.00444)	0.13<br>(0.08 to 0.19)	0.00224<br>(0.00148 to 0.00326)	0.06<br>(0.04 to 0.09)
Xinjiang	Leprosy	0.0206<br>(0.0133 to 0.0302)	0.13<br>(0.08 to 0.19)	0.02<br>(0.0131 to 0.0291)	0.08<br>(0.05 to 0.12)

Yunnan	Leprosy	0.0516 (0.0331 to 0.0766)	0.13 (0.08 to 0.2)	0.0413 (0.0268 to 0.0607)	0.08 (0.05 to 0.12)
Zhejiang	Leprosy	0.0631 (0.0415 to 0.0928)	0.14 (0.09 to 0.21)	0.0545 (0.0364 to 0.0784)	0.09 (0.06 to 0.13)
China	Other unspecified infectious diseases	749 (478 to 880)	62.55 (39.93 to 73.55)	195 (161 to 241)	13.81 (11.4 to 17.08)
Taiwan	Other unspecified infectious diseases	2.63 (1.8 to 4.12)	12.9 (8.81 to 20.2)	5.81 (2.52 to 7.38)	24.62 (10.68 to 31.29)
Hong Kong Special Administrative Region of China	Other unspecified infectious diseases	0.799 (0.559 to 1.16)	13.86 (9.7 to 20.18)	0.78 (0.578 to 1.11)	10.38 (7.7 to 14.8)
Macau Special Administrative Region of China	Other unspecified infectious diseases	0.0741 (0.0541 to 0.101)	20.87 (15.24 to 28.53)	0.0811 (0.0491 to 0.112)	11.86 (7.18 to 16.39)
Anhui	Other unspecified infectious diseases	39.3 (28.9 to 50.5)	66.24 (48.79 to 85.3)	7.96 (6.11 to 10.9)	13.01 (10 to 17.76)
Beijing	Other unspecified infectious diseases	2.52 (1.94 to 3.18)	22.5 (17.33 to 28.39)	2.1 (1.59 to 2.92)	8.72 (6.61 to 12.13)
Chongqing	Other unspecified infectious diseases	9.99 (6.96 to 13.1)	63.29 (44.12 to 83.01)	3.66 (2.69 to 5.02)	13.7 (10.05 to 18.78)
Fujian	Other unspecified infectious diseases	13.8 (10.6 to 17.3)	43.76 (33.45 to 54.59)	4.45 (3.39 to 6.12)	11.29 (8.6 to 15.54)
Gansu	Other unspecified infectious diseases	21.2 (11 to 28.2)	89.51 (46.51 to 119.12)	4.39 (3.43 to 5.6)	16.51 (12.91 to 21.06)
Guangdong	Other unspecified infectious diseases	28.6 (20.4 to 35.2)	43.38 (30.94 to 53.53)	13.4 (10.5 to 18.3)	11.44 (8.97 to 15.66)
Guangxi	Other unspecified infectious diseases	34.5 (19.1 to 43.8)	79.41 (43.88 to 100.6)	9.57 (7.4 to 11.8)	19.8 (15.31 to 24.52)
Guizhou	Other unspecified infectious diseases	44.8 (20.8 to 60.6)	134.97 (62.79 to 182.48)	5.76 (4.54 to 7.29)	17.02 (13.41 to 21.55)
Hainan	Other unspecified infectious diseases	8.24 (3.21 to 11.3)	118.23 (46.12 to 161.96)	3.61 (1.44 to 4.98)	37.69 (15.07 to 51.94)
Hebei	Other unspecified infectious diseases	33.9 (21 to 43)	53.27 (33.04 to 67.49)	10.3 (8.11 to 13.3)	13.47 (10.61 to 17.41)
Heilongjiang	Other unspecified infectious diseases	16 (10.3 to 21.5)	43.76 (28.14 to 58.87)	4.54 (3.37 to 6.32)	11.56 (8.58 to 16.1)
Henan	Other unspecified infectious diseases	47.3 (32 to 62.4)	53.59 (36.3 to 70.67)	11.7 (9.08 to 15.5)	12.12 (9.43 to 16.14)
Hubei	Other unspecified infectious diseases	26.5 (19.8 to 35)	47.7 (35.64 to 63.06)	5.8 (4.16 to 8.25)	10.48 (7.53 to 14.92)
Hunan	Other unspecified infectious diseases	30.4 (22 to 38.2)	48.9 (35.36 to 61.46)	8.14 (6.35 to 11.5)	12.13 (9.46 to 17.18)
Inner Mongolia	Other unspecified infectious diseases	13.2 (6.22 to 18.2)	58.2 (27.35 to 80.17)	3.42 (2.66 to 4.46)	13.19 (10.29 to 17.21)
Jiangsu	Other unspecified infectious diseases	27.7 (21.1 to 36.8)	40.26 (30.71 to 53.49)	7.96 (5.78 to 12.2)	9.75 (7.07 to 14.91)
Jiangxi	Other unspecified infectious diseases	60.7 (21.5 to 83.5)	153.56 (54.46 to 211.17)	11.1 (7.55 to 14)	23.38 (15.88 to 29.52)
Jilin	Other unspecified infectious diseases	15.9 (8.25 to 21)	61.26 (31.79 to 80.95)	3.79 (2.88 to 4.87)	13.6 (10.32 to 17.45)
Liaoning	Other unspecified infectious diseases	12.6 (9.36 to 16.2)	30.83 (22.93 to 39.75)	4.64 (3.4 to 6.66)	10.39 (7.61 to 14.92)
Ningxia	Other unspecified infectious diseases	2.73 (1.96 to 3.91)	55.34 (39.64 to 79.14)	0.923 (0.694 to 1.24)	13.15 (9.88 to 17.68)
Qinghai	Other unspecified infectious diseases	4.34 (2.3 to 5.9)	91.54 (48.48 to 124.65)	1.27 (0.948 to 1.67)	20.26 (15.09 to 26.57)
Shaanxi	Other unspecified infectious diseases	27.6 (15.7 to 35.5)	80.09 (45.61 to 103.12)	6.88 (5.04 to 9.15)	17.4 (12.76 to 23.17)
Shandong	Other unspecified infectious diseases	27.8 (21 to 35.3)	32.43 (24.58 to 41.18)	10.2 (7.28 to 15.1)	10.23 (7.32 to 15.15)
Shanghai	Other unspecified infectious diseases	7.04 (4.18 to 9.1)	50.37 (29.89 to 65.11)	3.95 (2.56 to 5.34)	14.13 (9.16 to 19.13)
Shanxi	Other unspecified infectious diseases	14.1 (11.2 to 17.4)	46.59 (36.92 to 57.61)	4.97 (3.79 to 6.69)	12.86 (9.82 to 17.32)
Sichuan	Other unspecified infectious diseases	71.3 (47.4 to 92.2)	63.91 (42.44 to 82.62)	11.3 (8.89 to 14.8)	13.58 (10.64 to 17.74)
Tianjin	Other unspecified infectious diseases	2.08 (1.58 to 2.76)	22.59 (17.2 to 30.01)	1.46 (1.03 to 2.21)	9.58 (6.74 to 14.47)

Tibet	Other unspecified infectious diseases	1.23<br>(0.885 to 2.5)	52.77<br>(37.98 to 107.2)	0.788<br>(0.562 to 1.22)	22.8<br>(16.27 to 35.38)
Xinjiang	Other unspecified infectious diseases	18.8<br>(6.38 to 25.5)	118.99<br>(40.46 to 161.82)	7.26<br>(3.74 to 9.39)	29.81<br>(15.34 to 38.54)
Yunnan	Other unspecified infectious diseases	62.6<br>(22.3 to 83.8)	160.66<br>(57.31 to 214.95)	12.8<br>(8.08 to 16.1)	25.94<br>(16.43 to 32.65)
Zhejiang	Other unspecified infectious diseases	21.2<br>(15.9 to 27)	48<br>(36.06 to 61.24)	6.17<br>(4.61 to 8.6)	10.09<br>(7.54 to 14.05)
China	Non-communicable diseases	238000<br>(217000 to 263000)	19888.11<br>(18125.43 to 21943.42)	307000<br>(275000 to 344000)	21734.98<br>(19437.7 to 24359.77)
Taiwan	Non-communicable diseases	3420<br>(3040 to 3860)	16752.38<br>(14902.77 to 18940.58)	5220<br>(4610 to 5910)	22153.71<br>(19559.42 to 25076.75)
Hong Kong Special Administrative Region of China	Non-communicable diseases	965<br>(850 to 1090)	16740.75<br>(14738.73 to 18912.88)	1410<br>(1200 to 1650)	18782.84<br>(15983.95 to 22014.14)
Macau Special Administrative Region of China	Non-communicable diseases	52.9<br>(46.3 to 60.2)	14892.95<br>(13058.32 to 16961.88)	101<br>(85.5 to 120)	14816.94<br>(12500.63 to 17565.47)
Anhui	Non-communicable diseases	12000<br>(10700 to 13400)	20218.54<br>(17989.4 to 22602.1)	13600<br>(11900 to 15600)	22273.62<br>(19448.91 to 25434.98)
Beijing	Non-communicable diseases	2070<br>(1810 to 2350)	18418.79<br>(16109.36 to 20950.99)	3910<br>(3270 to 4600)	16203.26<br>(13575.63 to 19078.75)
Chongqing	Non-communicable diseases	3440<br>(3070 to 3830)	21794.01<br>(19484.17 to 24278.98)	6180<br>(5210 to 7290)	23116.78<br>(19483.58 to 27268.79)
Fujian	Non-communicable diseases	5310<br>(4770 to 5910)	16778.12<br>(15074.06 to 18667.68)	7130<br>(6130 to 8330)	18094.98<br>(15559.3 to 21125.12)
Gansu	Non-communicable diseases	5970<br>(5430 to 6550)	25233.86<br>(22966.53 to 27710.92)	6090<br>(5310 to 6980)	22916.9<br>(19978.49 to 26278.92)
Guangdong	Non-communicable diseases	12200<br>(10800 to 13700)	18500.85<br>(16348.04 to 20816.78)	19600<br>(16700 to 23000)	16769.74<br>(14256.7 to 19651.31)
Guangxi	Non-communicable diseases	8420<br>(7480 to 9450)	19356.54<br>(17195.53 to 21730.78)	10600<br>(9190 to 12300)	22031.57<br>(19016.03 to 25437.53)
Guizhou	Non-communicable diseases	7370<br>(6520 to 8250)	22198.95<br>(19653.94 to 24860.29)	7900<br>(6790 to 9040)	23367.6<br>(20087.18 to 26723.64)
Hainan	Non-communicable diseases	1220<br>(1080 to 1380)	17519.26<br>(15480.35 to 19852.31)	1700<br>(1420 to 1990)	17691.67<br>(14856.55 to 20717.96)
Hebei	Non-communicable diseases	12000<br>(10700 to 13400)	18852.1<br>(16862.02 to 21078.7)	18400<br>(16100 to 21000)	24091.54<br>(21016 to 27491.79)
Heilongjiang	Non-communicable diseases	7230<br>(6500 to 8070)	19827.34<br>(17826.74 to 22125.53)	10600<br>(9140 to 12200)	27032.79<br>(23290.15 to 31043.76)
Henan	Non-communicable diseases	16600<br>(14900 to 18500)	18846.95<br>(16914.32 to 20993.3)	21600<br>(18800 to 24700)	22404.72<br>(19470.58 to 25616.38)
Hubei	Non-communicable diseases	10500<br>(9390 to 11700)	18860.37<br>(16896.43 to 21001.68)	13700<br>(11700 to 15700)	24693.3<br>(21109.54 to 28374.07)
Hunan	Non-communicable diseases	13600<br>(12300 to 15000)	21859.18<br>(19798.92 to 24120.18)	16900<br>(14700 to 19100)	25179.25<br>(21843.78 to 28496.8)
Inner Mongolia	Non-communicable diseases	4870<br>(4340 to 5430)	21427.18<br>(19094.04 to 23864.79)	5930<br>(5150 to 6800)	22910.85<br>(19881.36 to 26247.78)
Jiangsu	Non-communicable diseases	13200<br>(11800 to 14600)	19122.21<br>(17131.91 to 21269.7)	17500<br>(15300 to 20000)	21394.81<br>(18734.88 to 24536.21)
Jiangxi	Non-communicable diseases	9430<br>(8590 to 10300)	23853.83<br>(21735.96 to 26101.22)	9270<br>(8060 to 10700)	19499.9<br>(16945.38 to 22458.64)
Jilin	Non-communicable diseases	5090<br>(4590 to 5650)	19603.49<br>(17688.74 to 21784.17)	6520<br>(5540 to 7540)	23369.91<br>(19851.2 to 27044.91)
Liaoning	Non-communicable diseases	7920<br>(6990 to 8910)	19422.01<br>(17138.96 to 21841.62)	12000<br>(10400 to 13800)	26808.8<br>(23240.39 to 30858.21)
Ningxia	Non-communicable diseases	979<br>(869 to 1110)	19826.18<br>(17594.9 to 22454.66)	1270<br>(1080 to 1460)	18044.52<br>(15340.35 to 20816.89)
Qinghai	Non-communicable diseases	1010<br>(905 to 1120)	21360.58<br>(19099.61 to 23738.08)	1360<br>(1180 to 1560)	21598.8<br>(18785.49 to 24852.09)
Shaanxi	Non-communicable diseases	5060<br>(4460 to 5720)	14706.5<br>(12972.53 to 16621.59)	8130<br>(6940 to 9430)	20575.53<br>(17555.7 to 23870.82)
Shandong	Non-communicable diseases	17900<br>(16000 to 20000)	20944.29<br>(18663.61 to 23387.5)	22600<br>(19500 to 25900)	22746.33<br>(19592.13 to 26084.23)
Shanghai	Non-communicable diseases	2630<br>(2310 to 2990)	18810.63<br>(16519.1 to 21404.6)	4490<br>(3780 to 5300)	16060.58<br>(13543.51 to 18964.38)
Shanxi	Non-communicable diseases	6260<br>(5590 to 7020)	20662.77<br>(18471.26 to 23194.11)	7540<br>(6400 to 8860)	19518.03<br>(16580.06 to 22931.37)

Sichuan	Non-communicable diseases	21800 (19500 to 24300)	19542.06 (17433.35 to 21747.01)	20900 (18000 to 23800)	25039.7 (21529.63 to 28537.83)
Tianjin	Non-communicable diseases	1830 (1620 to 2050)	19906.23 (17573.03 to 22320.91)	3150 (2680 to 3640)	20615.1 (17528.13 to 23871.02)
Tibet	Non-communicable diseases	693 (624 to 763)	29758.47 (26784.01 to 32748.13)	726 (633 to 819)	21014.46 (18315.31 to 23705.05)
Xinjiang	Non-communicable diseases	2910 (2620 to 3230)	18461.77 (16626.48 to 20504.7)	5110 (4430 to 5850)	20972.47 (18198.12 to 24027.19)
Yunnan	Non-communicable diseases	8870 (7960 to 9900)	22770.43 (20436.68 to 25407.67)	10500 (9110 to 12100)	21438.57 (18527.31 to 24521.48)
Zhejiang	Non-communicable diseases	8700 (7810 to 9700)	19735.58 (17706.66 to 22005.74)	10600 (9020 to 12300)	17302.66 (14741.95 to 20108.52)
China	Neoplasms	43000 (41200 to 45200)	3593.41 (3444.01 to 3775.41)	63200 (60600 to 65900)	4477.65 (4287.05 to 4662.28)
Taiwan	Neoplasms	582 (574 to 590)	2853.44 (2815.15 to 2893.55)	1210 (1150 to 1270)	5117.63 (4858.71 to 5392.06)
Hong Kong Special Administrative Region of China	Neoplasms	237 (218 to 256)	4112.57 (3785.11 to 4439.51)	324 (275 to 384)	4316.23 (3667.97 to 5111.62)
Macau Special Administrative Region of China	Neoplasms	9 (8.35 to 9.79)	2536.64 (2351.93 to 2758.46)	18.5 (16.1 to 21.2)	2704.18 (2358.58 to 3099.24)
Anhui	Neoplasms	2690 (2360 to 3010)	4533.03 (3984.78 to 5074.67)	3100 (2600 to 3620)	5068.22 (4257.95 to 5920.09)
Beijing	Neoplasms	353 (312 to 400)	3145.9 (2783.11 to 3565.33)	644 (533 to 759)	2670.41 (2211.24 to 3150.75)
Chongqing	Neoplasms	625 (548 to 721)	3963.19 (3472.05 to 4567.48)	1280 (1040 to 1570)	4787.41 (3881.66 to 5869.31)
Fujian	Neoplasms	1070 (987 to 1150)	3371.59 (3118.93 to 3642.79)	1790 (1480 to 2150)	4535.32 (3760.72 to 5445.83)
Gansu	Neoplasms	966 (874 to 1080)	4083.74 (3696.65 to 4548.29)	1000 (841 to 1170)	3767.35 (3164.06 to 4389.61)
Guangdong	Neoplasms	2370 (2050 to 2700)	3594.75 (3115.53 to 4101.64)	4030 (3400 to 4780)	3453.24 (2909.14 to 4093.24)
Guangxi	Neoplasms	1480 (1290 to 1710)	3405.33 (2975.59 to 3935.88)	2220 (1860 to 2650)	4594.55 (3838.9 to 5486.48)
Guizhou	Neoplasms	1040 (900 to 1270)	3126.12 (2710.98 to 3827.51)	1220 (1000 to 1440)	3597.5 (2966.75 to 4257.24)
Hainan	Neoplasms	227 (196 to 267)	3265.35 (2812.67 to 3833.05)	317 (261 to 388)	3309.79 (2726.91 to 4042.67)
Hebei	Neoplasms	2100 (1940 to 2260)	3295.99 (3043.79 to 3550.78)	3340 (2870 to 3880)	4370.93 (3749.53 to 5076.91)
Heilongjiang	Neoplasms	1330 (1220 to 1440)	3635.39 (3339.11 to 3961.74)	2300 (1920 to 2730)	5855.03 (4893.04 to 6945.58)
Henan	Neoplasms	3020 (2800 to 3250)	3423.87 (3166.77 to 3686.03)	4270 (3660 to 4990)	4433.12 (3804.22 to 5183.55)
Hubei	Neoplasms	1710 (1560 to 1870)	3072.72 (2816.19 to 3367.46)	2950 (2500 to 3450)	5326.09 (4517.54 to 6239.13)
Hunan	Neoplasms	2210 (2030 to 2460)	3553.07 (3270.91 to 3953.57)	3350 (2870 to 3920)	4989.29 (4280.12 to 5832.01)
Inner Mongolia	Neoplasms	797 (705 to 898)	3505.66 (3099.5 to 3949.74)	1080 (917 to 1260)	4167.91 (3539.49 to 4879.73)
Jiangsu	Neoplasms	3000 (2750 to 3260)	4353.97 (3997.24 to 4738.63)	4510 (3770 to 5280)	5522.89 (4622.32 to 6461.01)
Jiangxi	Neoplasms	1750 (1570 to 1920)	4419.1 (3979.54 to 4846.82)	1800 (1550 to 2100)	3792.17 (3259.21 to 4414.46)
Jilin	Neoplasms	912 (835 to 1000)	3513.88 (3217.67 to 3860.34)	1250 (1010 to 1480)	4477.19 (3628.87 to 5295.38)
Liaoning	Neoplasms	1510 (1330 to 1710)	3711.48 (3254.76 to 4189.16)	2680 (2230 to 3170)	6010.98 (5001.81 to 7106.2)
Ningxia	Neoplasms	160 (139 to 182)	3245.27 (2808.61 to 3695.23)	227 (188 to 273)	3230.94 (2675.89 to 3894.51)
Qinghai	Neoplasms	164 (143 to 197)	3464.5 (3028.13 to 4163.21)	243 (199 to 287)	3864.35 (3165.18 to 4562.7)
Shaanxi	Neoplasms	696 (637 to 756)	2022.88 (1849.75 to 2195.94)	1410 (1150 to 1700)	3569.01 (2908.41 to 4314.22)
Shandong	Neoplasms	3560 (3150 to 3980)	4159.15 (3685.46 to 4648.08)	5100 (4310 to 6060)	5135.99 (4338.97 to 6099.25)

Shanghai	Neoplasms	558<br>(493 to 631)	3991.84<br>(3528.61 to 4513.66)	897<br>(748 to 1080)	3209.96<br>(2678.35 to 3857.59)
Shanxi	Neoplasms	1170<br>(1040 to 1310)	3869.25<br>(3437.24 to 4322.94)	1440<br>(1170 to 1740)	3733.55<br>(3032.02 to 4496.41)
Sichuan	Neoplasms	3740<br>(3400 to 4160)	3353.16<br>(3043.31 to 3724.97)	4960<br>(4120 to 5830)	5936.02<br>(4936.28 to 6986.11)
Tianjin	Neoplasms	319<br>(283 to 357)	3473.14<br>(3072.38 to 3883.35)	606<br>(515 to 718)	3971.09<br>(3371.92 to 4703.88)
Tibet	Neoplasms	72.3<br>(61.4 to 96.4)	3101.1<br>(2634.54 to 4135.61)	74.4<br>(61.7 to 92.7)	2153.04<br>(1784.75 to 2682.87)
Xinjiang	Neoplasms	414<br>(370 to 488)	2623<br>(2343.35 to 3095.15)	770<br>(653 to 912)	3160.18<br>(2678.46 to 3741.11)
Yunnan	Neoplasms	1100<br>(934 to 1380)	2825.7<br>(2395.47 to 3552.83)	1530<br>(1300 to 1830)	3107.87<br>(2637.77 to 3723.17)
Zhejiang	Neoplasms	1670<br>(1530 to 1830)	3788.03<br>(3470.9 to 4138.77)	2520<br>(2110 to 3020)	4115.05<br>(3446.98 to 4934.02)
China	Esophageal cancer	4080<br>(3890 to 4310)	340.71<br>(325 to 360.44)	4460<br>(4250 to 4690)	316.11<br>(300.73 to 332.1)
Taiwan	Esophageal cancer	20.6<br>(19.9 to 21.3)	100.84<br>(97.57 to 104.41)	60.9<br>(55.9 to 66.4)	258.45<br>(237.2 to 281.47)
Hong Kong Special Administrative Region of China	Esophageal cancer	13.7<br>(12.3 to 15.3)	237.41<br>(213.36 to 266.16)	13.1<br>(10.8 to 16)	174.22<br>(143.59 to 212.8)
Macau Special Administrative Region of China	Esophageal cancer	0.523<br>(0.472 to 0.58)	147.32<br>(133.12 to 163.52)	1.07<br>(0.857 to 1.29)	156.09<br>(125.26 to 188.19)
Anhui	Esophageal cancer	296<br>(258 to 339)	499.13<br>(435.82 to 571.63)	262<br>(220 to 310)	429.28<br>(359.06 to 507.1)
Beijing	Esophageal cancer	21.5<br>(18.2 to 25.1)	191.8<br>(162.32 to 223.77)	28.6<br>(22.7 to 35.1)	118.47<br>(94.16 to 145.56)
Chongqing	Esophageal cancer	71.4<br>(61 to 84.3)	452.71<br>(386.32 to 534.06)	118<br>(94.5 to 147)	441.82<br>(353.68 to 551.18)
Fujian	Esophageal cancer	132<br>(118 to 151)	418.79<br>(371.49 to 477.83)	173<br>(142 to 211)	437.82<br>(359.23 to 534.46)
Gansu	Esophageal cancer	82.5<br>(72.4 to 94.7)	348.93<br>(306.16 to 400.35)	66.5<br>(55.2 to 79.1)	250.26<br>(207.61 to 297.75)
Guangdong	Esophageal cancer	173<br>(147 to 205)	263.28<br>(222.76 to 311.34)	218<br>(178 to 265)	186.24<br>(152.68 to 226.75)
Guangxi	Esophageal cancer	50.6<br>(42.8 to 60.5)	116.25<br>(98.44 to 139.08)	58.5<br>(48.4 to 70.9)	121.15<br>(100.1 to 146.72)
Guizhou	Esophageal cancer	31.4<br>(26.3 to 37.5)	94.74<br>(79.32 to 112.84)	36.2<br>(29.6 to 43.3)	106.99<br>(87.5 to 128.04)
Hainan	Esophageal cancer	8.25<br>(6.89 to 9.81)	118.41<br>(98.85 to 140.9)	8.77<br>(6.87 to 11.2)	91.49<br>(71.61 to 116.72)
Hebei	Esophageal cancer	418<br>(376 to 461)	656.86<br>(590.27 to 724.88)	310<br>(257 to 368)	405.15<br>(336.48 to 481.29)
Heilongjiang	Esophageal cancer	48.7<br>(42.5 to 55.4)	133.68<br>(116.57 to 151.93)	79.5<br>(63.9 to 99.8)	202.49<br>(162.73 to 254.17)
Henan	Esophageal cancer	695<br>(633 to 764)	787.16<br>(717.65 to 865.36)	474<br>(397 to 558)	491.86<br>(412.39 to 579.31)
Hubei	Esophageal cancer	122<br>(109 to 135)	218.83<br>(196.87 to 242.87)	173<br>(145 to 208)	313.65<br>(262.87 to 375.23)
Hunan	Esophageal cancer	69.3<br>(61.9 to 78)	111.4<br>(99.48 to 125.38)	98.6<br>(81.3 to 119)	146.88<br>(121.04 to 177.14)
Inner Mongolia	Esophageal cancer	64.9<br>(55 to 76)	285.55<br>(241.78 to 334.12)	80.5<br>(66.5 to 98.1)	310.88<br>(256.63 to 378.71)
Jiangsu	Esophageal cancer	318<br>(282 to 358)	462.56<br>(410.37 to 520.59)	552<br>(459 to 654)	676.15<br>(561.88 to 801.22)
Jiangxi	Esophageal cancer	80.5<br>(71 to 91.1)	203.6<br>(179.53 to 230.47)	61.5<br>(52.3 to 72.6)	129.44<br>(109.99 to 152.74)
Jilin	Esophageal cancer	28.9<br>(25.3 to 33.1)	111.44<br>(97.41 to 127.62)	37.2<br>(29.1 to 45.4)	133.21<br>(104.16 to 162.89)
Liaoning	Esophageal cancer	78.2<br>(64.7 to 93.4)	191.66<br>(158.54 to 229.06)	123<br>(98.1 to 152)	275.46<br>(219.61 to 340.01)
Ningxia	Esophageal cancer	9.31<br>(7.99 to 10.9)	188.43<br>(161.75 to 220.91)	12.4<br>(9.86 to 15.6)	176.06<br>(140.52 to 221.6)
Qinghai	Esophageal cancer	11.7<br>(10 to 13.8)	246.69<br>(211.34 to 290.52)	15.9<br>(12.7 to 19.2)	252.61<br>(202.47 to 305.25)

Shaanxi	Esophageal cancer	65.8<br>(57.5 to 74.5)	191.15<br>(167.15 to 216.46)	122<br>(97.4 to 152)	308.25<br>(246.48 to 384.45)
Shandong	Esophageal cancer	351<br>(305 to 406)	410.34<br>(356.69 to 473.98)	386<br>(318 to 464)	388.29<br>(320.16 to 466.73)
Shanghai	Esophageal cancer	30.5<br>(26.4 to 35.6)	218.62<br>(188.78 to 254.6)	32.7<br>(26.3 to 40.2)	117.21<br>(93.98 to 143.92)
Shanxi	Esophageal cancer	144<br>(126 to 164)	475.81<br>(416.37 to 541.29)	115<br>(92.3 to 140)	298.14<br>(238.84 to 363.7)
Sichuan	Esophageal cancer	467<br>(415 to 537)	418.43<br>(371.91 to 480.77)	544<br>(432 to 656)	651.79<br>(517.97 to 786.13)
Tianjin	Esophageal cancer	16.1<br>(13.8 to 18.4)	175.51<br>(150.51 to 200.17)	16.4<br>(13.5 to 20)	107.68<br>(88.45 to 130.9)
Tibet	Esophageal cancer	3.22<br>(2.76 to 3.76)	138.39<br>(118.63 to 161.24)	2.54<br>(2.09 to 3.05)	73.55<br>(60.47 to 88.27)
Xinjiang	Esophageal cancer	42.5<br>(37.7 to 47.4)	269.59<br>(239.01 to 300.57)	74<br>(61.8 to 88.8)	303.72<br>(253.42 to 364.52)
Yunnan	Esophageal cancer	34.5<br>(28.7 to 42.6)	88.63<br>(73.6 to 109.21)	48.9<br>(40.3 to 60.2)	99.49<br>(81.95 to 122.42)
Zhejiang	Esophageal cancer	97.4<br>(84.6 to 113)	220.93<br>(191.83 to 256.76)	122<br>(97.7 to 151)	200.15<br>(159.64 to 246.67)
China	Stomach cancer	7410<br>(7140 to 7840)	619.45<br>(596.49 to 655)	7810<br>(7480 to 8190)	553.28<br>(529.25 to 579.65)
Taiwan	Stomach cancer	63.3<br>(61.6 to 65)	310.19<br>(301.71 to 318.49)	68.3<br>(64.1 to 73)	289.59<br>(271.6 to 309.54)
Hong Kong Special Administrative Region of China	Stomach cancer	24.3<br>(22.1 to 26.7)	421.82<br>(383.82 to 464)	24.5<br>(20.6 to 29.2)	325.61<br>(273.93 to 388.3)
Macau Special Administrative Region of China	Stomach cancer	1.04<br>(0.953 to 1.13)	293.53<br>(268.65 to 317.89)	1.28<br>(1.07 to 1.54)	187.33<br>(156.5 to 224.78)
Anhui	Stomach cancer	559<br>(490 to 634)	943.03<br>(827.39 to 1070.07)	517<br>(434 to 614)	845.54<br>(709.17 to 1003.95)
Beijing	Stomach cancer	41<br>(35.5 to 46.9)	365.6<br>(316.29 to 417.72)	42.3<br>(34.5 to 50.9)	175.5<br>(142.95 to 211.35)
Chongqing	Stomach cancer	71.7<br>(61.3 to 84.8)	454.43<br>(388.47 to 537.22)	105<br>(84.5 to 129)	391.57<br>(316.09 to 481.04)
Fujian	Stomach cancer	206<br>(186 to 232)	652.16<br>(587.18 to 733.64)	240<br>(197 to 290)	608.15<br>(500.67 to 734.9)
Gansu	Stomach cancer	294<br>(262 to 334)	1242.71<br>(1107.02 to 1410.98)	248<br>(206 to 292)	933.14<br>(775.07 to 1099.35)
Guangdong	Stomach cancer	231<br>(198 to 269)	350.71<br>(300.65 to 408.91)	266<br>(224 to 317)	227.37<br>(191.95 to 271.45)
Guangxi	Stomach cancer	173<br>(149 to 201)	398.09<br>(343.66 to 462.67)	190<br>(159 to 228)	392.66<br>(328.94 to 470.85)
Guizhou	Stomach cancer	139<br>(117 to 167)	419.57<br>(353.74 to 503.31)	145<br>(120 to 172)	428.89<br>(353.94 to 509.46)
Hainan	Stomach cancer	38.2<br>(32.5 to 44.5)	548.13<br>(467.12 to 638.96)	35.3<br>(28.5 to 43.7)	367.78<br>(296.94 to 455.33)
Hebei	Stomach cancer	415<br>(373 to 459)	652.04<br>(585.8 to 720.46)	529<br>(433 to 630)	691.83<br>(567.2 to 824.77)
Heilongjiang	Stomach cancer	162<br>(143 to 185)	443.54<br>(390.97 to 506.37)	184<br>(153 to 224)	469.63<br>(388.73 to 570.41)
Henan	Stomach cancer	703<br>(643 to 769)	796.58<br>(728.14 to 871.23)	679<br>(571 to 796)	705.18<br>(593.14 to 826.79)
Hubei	Stomach cancer	280<br>(252 to 308)	503.89<br>(453.81 to 554.59)	358<br>(302 to 426)	647.67<br>(545.73 to 771.01)
Hunan	Stomach cancer	263<br>(236 to 293)	422.74<br>(379.44 to 471.63)	288<br>(240 to 342)	429.55<br>(357.69 to 509.81)
Inner Mongolia	Stomach cancer	111<br>(97 to 128)	489.97<br>(426.61 to 564.84)	116<br>(97.1 to 138)	449.14<br>(374.91 to 532.78)
Jiangsu	Stomach cancer	621<br>(562 to 691)	901.73<br>(817.21 to 1004.6)	661<br>(547 to 785)	809.53<br>(669.89 to 961)
Jiangxi	Stomach cancer	282<br>(253 to 315)	714.13<br>(640.67 to 796.72)	230<br>(194 to 270)	483.29<br>(408.75 to 568.39)
Jilin	Stomach cancer	122<br>(107 to 142)	470.78<br>(412.93 to 546.31)	110<br>(88.9 to 131)	394.78<br>(318.78 to 468.22)
Liaoning	Stomach cancer	211<br>(182 to 242)	517.04<br>(447.34 to 592.47)	254<br>(209 to 304)	569.4<br>(467.37 to 680.2)

Ningxia	Stomach cancer	34.3 (29.4 to 39.9)	694.06 (594.71 to 808.78)	46.5 (37.5 to 57.6)	662.78 (534.43 to 820.58)
Qinghai	Stomach cancer	42.3 (36.8 to 49.8)	893.28 (776.94 to 1050.82)	58.1 (46.5 to 69.8)	925.18 (739.71 to 1110.34)
Shaanxi	Stomach cancer	115 (103 to 130)	334.65 (299.55 to 378.34)	214 (172 to 263)	542.75 (435.72 to 666.41)
Shandong	Stomach cancer	703 (619 to 799)	821.61 (723.56 to 933.07)	728 (606 to 875)	732.29 (609.28 to 880.72)
Shanghai	Stomach cancer	102 (88.4 to 118)	727.85 (632.55 to 842.99)	94 (77.8 to 114)	336.6 (278.32 to 406.94)
Shanxi	Stomach cancer	285 (249 to 326)	941.92 (823.16 to 1075.2)	269 (213 to 329)	695.8 (551.76 to 851.53)
Sichuan	Stomach cancer	621 (559 to 707)	556.18 (500.66 to 633.29)	569 (462 to 676)	680.97 (553.92 to 809.29)
Tianjin	Stomach cancer	36.9 (31.9 to 41.9)	400.78 (347.31 to 455.7)	42.9 (35.9 to 51.6)	281 (235.27 to 338.12)
Tibet	Stomach cancer	20 (17.2 to 23)	857.15 (738.4 to 988.74)	15 (12.4 to 17.8)	433.01 (360.14 to 515.85)
Xinjiang	Stomach cancer	70.6 (62.8 to 79.4)	447.4 (397.85 to 503.47)	121 (98.7 to 149)	495.94 (405.25 to 610.87)
Yunnan	Stomach cancer	122 (103 to 147)	313.18 (264.61 to 377.43)	153 (128 to 183)	311.61 (260.48 to 371.22)
Zhejiang	Stomach cancer	314 (281 to 352)	712.11 (637.01 to 797.35)	281 (231 to 338)	460.01 (377.17 to 552.42)
China	Liver cancer	7770 (7220 to 8230)	649.16 (603.02 to 687.45)	11200 (10500 to 11800)	789.61 (745.17 to 835.93)
Taiwan	Liver cancer	112 (109 to 116)	548.98 (531.9 to 567.65)	214 (199 to 229)	906.2 (845.94 to 968.93)
Hong Kong Special Administrative Region of China	Liver cancer	32 (28.7 to 36)	555.52 (497.96 to 625.23)	43.7 (35.5 to 54.7)	581.3 (473.04 to 727.88)
Macau Special Administrative Region of China	Liver cancer	1.46 (1.27 to 1.68)	411.16 (358.7 to 473.66)	3.69 (2.52 to 4.83)	540.19 (368.75 to 706.85)
Anhui	Liver cancer	513 (407 to 628)	865.12 (686.88 to 1059.35)	543 (432 to 652)	887.88 (706.15 to 1066.15)
Beijing	Liver cancer	45 (38 to 54.7)	401.18 (338.82 to 487.76)	76.1 (59.7 to 106)	315.78 (247.56 to 441.13)
Chongqing	Liver cancer	132 (103 to 162)	836.69 (651.43 to 1024.87)	233 (184 to 297)	872.89 (689.14 to 1109.62)
Fujian	Liver cancer	189 (163 to 211)	595.83 (514.56 to 667.04)	361 (282 to 454)	915.36 (716.07 to 1151.85)
Gansu	Liver cancer	125 (105 to 149)	527.82 (443.1 to 631.54)	137 (113 to 168)	516.95 (426.47 to 633.29)
Guangdong	Liver cancer	532 (403 to 643)	808.28 (612.44 to 977.62)	845 (674 to 1060)	723.16 (577.23 to 904.78)
Guangxi	Liver cancer	421 (326 to 508)	968.36 (750.44 to 1168.8)	659 (486 to 833)	1363.37 (1004.9 to 1723.87)
Guizhou	Liver cancer	165 (136 to 201)	498.44 (409.56 to 606.42)	185 (146 to 234)	547.12 (431.75 to 692.67)
Hainan	Liver cancer	51.1 (36.6 to 70.4)	733.59 (525.48 to 1011.15)	83.1 (63.4 to 107)	866.68 (661.08 to 1110.78)
Hebei	Liver cancer	242 (210 to 276)	380.08 (329.84 to 433.61)	436 (358 to 535)	569.99 (468.4 to 700.01)
Heilongjiang	Liver cancer	270 (231 to 314)	740.09 (634.73 to 861.56)	497 (398 to 611)	1265.05 (1014.39 to 1556.03)
Henan	Liver cancer	407 (362 to 460)	461.34 (409.56 to 521.18)	758 (630 to 908)	786.87 (653.68 to 942.56)
Hubei	Liver cancer	312 (272 to 351)	560.96 (489.34 to 632.68)	584 (481 to 707)	1055.72 (870.27 to 1279.03)
Hunan	Liver cancer	423 (372 to 474)	680.77 (598.59 to 763.01)	606 (496 to 737)	903.07 (739.2 to 1097.7)
Inner Mongolia	Liver cancer	140 (115 to 165)	617.05 (505.15 to 726.09)	175 (142 to 217)	674.58 (549.82 to 836.12)
Jiangsu	Liver cancer	644 (491 to 727)	935.26 (713.32 to 1056.6)	702 (568 to 859)	859.53 (695.07 to 1052.12)
Jiangxi	Liver cancer	352 (281 to 419)	890.65 (710.9 to 1058.98)	364 (301 to 436)	765.19 (632.99 to 917.44)

Jilin	Liver cancer	183<br>(152 to 217)	705.91<br>(584.34 to 836.21)	225<br>(179 to 272)	805.69<br>(642.23 to 974.51)
Liaoning	Liver cancer	241<br>(199 to 290)	590.47<br>(487.57 to 711.05)	411<br>(329 to 503)	920.58<br>(735.91 to 1127.57)
Ningxia	Liver cancer	26<br>(21.5 to 30.7)	525.47<br>(434.7 to 621.4)	39<br>(30.8 to 48.8)	555.6<br>(438.89 to 695.84)
Qinghai	Liver cancer	24.5<br>(21 to 28.7)	516.89<br>(442.96 to 605.39)	42.2<br>(33.9 to 51.7)	672.48<br>(539.27 to 822.47)
Shaanxi	Liver cancer	89.6<br>(75.7 to 106)	260.41<br>(219.96 to 308.04)	192<br>(151 to 244)	485.76<br>(382.12 to 618.36)
Shandong	Liver cancer	697<br>(571 to 804)	814.11<br>(667.59 to 939.81)	835<br>(684 to 1020)	840.58<br>(687.82 to 1025.89)
Shanghai	Liver cancer	72<br>(61.2 to 85.5)	515.1<br>(438.27 to 611.67)	98.5<br>(78 to 128)	352.41<br>(279.25 to 457.15)
Shanxi	Liver cancer	160<br>(132 to 191)	529.75<br>(436.16 to 631.34)	183<br>(144 to 236)	473.32<br>(372.72 to 611.9)
Sichuan	Liver cancer	728<br>(620 to 830)	651.99<br>(555.25 to 743.7)	920<br>(720 to 1130)	1101.84<br>(862.9 to 1356.35)
Tianjin	Liver cancer	40.2<br>(34 to 47.2)	436.89<br>(369.14 to 512.82)	76.7<br>(62.2 to 97.4)	502.65<br>(407.84 to 638.47)
Tibet	Liver cancer	15.5<br>(12.5 to 18.6)	663.37<br>(534.99 to 799.89)	16.9<br>(13.5 to 20.8)	488.54<br>(391.11 to 602.55)
Xinjiang	Liver cancer	47.1<br>(40.7 to 56.1)	298.7<br>(258.28 to 355.39)	109<br>(88 to 142)	448.83<br>(361.27 to 584.03)
Yunnan	Liver cancer	157<br>(131 to 193)	403.81<br>(336.42 to 494.91)	246<br>(203 to 319)	500.27<br>(412.36 to 648.66)
Zhejiang	Liver cancer	293<br>(256 to 330)	664.75<br>(580.56 to 747.79)	468<br>(373 to 583)	765.57<br>(610 to 952.99)
China	Larynx cancer	309<br>(296 to 325)	25.79<br>(24.73 to 27.16)	465<br>(443 to 489)	32.94<br>(31.35 to 34.63)
Taiwan	Larynx cancer	5.12<br>(4.88 to 5.36)	25.09<br>(23.94 to 26.29)	6.89<br>(6.23 to 7.61)	29.22<br>(26.41 to 32.26)
Hong Kong Special Administrative Region of China	Larynx cancer	2.61<br>(2.32 to 3)	45.36<br>(40.28 to 52.01)	2.09<br>(1.69 to 2.57)	27.89<br>(22.44 to 34.24)
Macau Special Administrative Region of China	Larynx cancer	0.0881<br>(0.0782 to 0.0992)	24.82<br>(22.03 to 27.95)	0.13<br>(0.101 to 0.166)	18.98<br>(14.7 to 24.29)
Anhui	Larynx cancer	13.2<br>(10.9 to 15.7)	22.28<br>(18.36 to 26.57)	15.8<br>(12.9 to 19.1)	25.81<br>(21.13 to 31.16)
Beijing	Larynx cancer	2.33<br>(1.95 to 2.81)	20.8<br>(17.39 to 25.06)	3.6<br>(2.81 to 4.49)	14.95<br>(11.65 to 18.62)
Chongqing	Larynx cancer	3.41<br>(2.86 to 4.12)	21.64<br>(18.13 to 26.14)	6.86<br>(5.3 to 8.83)	25.65<br>(19.83 to 33.04)
Fujian	Larynx cancer	4.47<br>(3.93 to 5.15)	14.12<br>(12.42 to 16.26)	8.72<br>(6.95 to 11)	22.13<br>(17.64 to 27.97)
Gansu	Larynx cancer	3.88<br>(3.28 to 4.48)	16.41<br>(13.87 to 18.94)	4.18<br>(3.37 to 5.1)	15.74<br>(12.68 to 19.18)
Guangdong	Larynx cancer	19.2<br>(15.8 to 23.2)	29.24<br>(24.07 to 35.28)	32.4<br>(25.9 to 40.5)	27.69<br>(22.13 to 34.65)
Guangxi	Larynx cancer	15.3<br>(12.8 to 18.2)	35.18<br>(29.46 to 41.84)	23.9<br>(19.3 to 29.8)	49.46<br>(39.97 to 61.62)
Guizhou	Larynx cancer	15.7<br>(13.3 to 18.7)	47.45<br>(40.2 to 56.26)	18.8<br>(15 to 23)	55.58<br>(44.45 to 67.85)
Hainan	Larynx cancer	4.06<br>(3.22 to 4.9)	58.24<br>(46.26 to 70.29)	5.16<br>(3.95 to 6.75)	53.77<br>(41.19 to 70.38)
Hebei	Larynx cancer	14.4<br>(12.8 to 16.3)	22.69<br>(20.06 to 25.62)	24.4<br>(19.8 to 29.9)	31.9<br>(25.86 to 39.08)
Heilongjiang	Larynx cancer	16.7<br>(14.4 to 19.3)	45.79<br>(39.42 to 52.94)	27.5<br>(22.3 to 33.8)	70.11<br>(56.87 to 86.03)
Henan	Larynx cancer	12.5<br>(11 to 14)	14.12<br>(12.44 to 15.84)	19.8<br>(16.2 to 23.9)	20.53<br>(16.82 to 24.77)
Hubei	Larynx cancer	15.5<br>(13.6 to 17.5)	27.83<br>(24.49 to 31.53)	23.5<br>(19.4 to 28.8)	42.43<br>(35.02 to 52)
Hunan	Larynx cancer	22.7<br>(20.2 to 25.6)	36.48<br>(32.55 to 41.2)	43.8<br>(35.2 to 53.4)	65.29<br>(52.35 to 79.49)
Inner Mongolia	Larynx cancer	5.63<br>(4.76 to 6.63)	24.78<br>(20.93 to 29.15)	8.34<br>(6.89 to 10.2)	32.21<br>(26.62 to 39.49)

Jiangsu	Larynx cancer	12.6 (11.1 to 14.4)	18.34 (16.06 to 20.95)	17.5 (14.2 to 21.7)	21.47 (17.42 to 26.52)
Jiangxi	Larynx cancer	14.1 (12.4 to 15.9)	35.62 (31.39 to 40.29)	15 (12.6 to 18.1)	31.65 (26.51 to 38.09)
Jilin	Larynx cancer	11.1 (9.77 to 12.6)	42.72 (37.66 to 48.62)	14.2 (11.4 to 17.1)	50.83 (40.88 to 61.18)
Liaoning	Larynx cancer	15.2 (12.6 to 18)	37.19 (30.78 to 44.22)	25.4 (20.4 to 31)	56.87 (45.58 to 69.47)
Ningxia	Larynx cancer	0.557 (0.471 to 0.65)	11.28 (9.54 to 13.15)	0.89 (0.695 to 1.12)	12.68 (9.9 to 15.94)
Qinghai	Larynx cancer	0.567 (0.481 to 0.67)	11.96 (10.16 to 14.15)	0.915 (0.729 to 1.13)	14.56 (11.61 to 17.92)
Shaanxi	Larynx cancer	3.99 (3.47 to 4.7)	11.59 (10.07 to 13.67)	8.81 (6.92 to 11.3)	22.3 (17.52 to 28.58)
Shandong	Larynx cancer	21.2 (18.1 to 24.7)	24.74 (21.14 to 28.91)	29.3 (24 to 35.8)	29.45 (24.14 to 36.07)
Shanghai	Larynx cancer	3.52 (2.93 to 4.24)	25.21 (20.99 to 30.36)	4.81 (3.71 to 6.15)	17.21 (13.29 to 22.03)
Shanxi	Larynx cancer	8.93 (7.77 to 10.3)	29.51 (25.64 to 34.07)	11.2 (8.79 to 13.9)	28.94 (22.76 to 36)
Sichuan	Larynx cancer	22.8 (19.9 to 26.1)	20.45 (17.82 to 23.4)	33.8 (26.3 to 41.3)	40.43 (31.45 to 49.48)
Tianjin	Larynx cancer	2.99 (2.56 to 3.49)	32.49 (27.8 to 37.98)	4.67 (3.77 to 5.7)	30.63 (24.72 to 37.35)
Tibet	Larynx cancer	0.262 (0.21 to 0.32)	11.25 (9.01 to 13.74)	0.28 (0.222 to 0.351)	8.11 (6.43 to 10.16)
Xinjiang	Larynx cancer	1.65 (1.43 to 1.9)	10.44 (9.06 to 12.05)	3.15 (2.36 to 4.04)	12.93 (9.7 to 16.57)
Yunnan	Larynx cancer	9.8 (8.21 to 11.9)	25.14 (21.07 to 30.54)	14.8 (12.3 to 18.1)	30.19 (24.92 to 36.79)
Zhejiang	Larynx cancer	7.75 (6.75 to 8.84)	17.57 (15.31 to 20.04)	11.6 (9.15 to 14.6)	18.98 (14.95 to 23.8)
China	Tracheal, bronchus, and lung cancer	6350 (6050 to 6790)	530.38 (505.81 to 567.41)	15300 (14500 to 15900)	1079.87 (1028.5 to 1127.14)
Taiwan	Tracheal, bronchus, and lung cancer	89.2 (87.1 to 91.5)	437.08 (426.96 to 448.24)	209 (196 to 222)	884.75 (829.73 to 941.4)
Hong Kong Special Administrative Region of China	Tracheal, bronchus, and lung cancer	60.4 (54.9 to 66.3)	1048.31 (952.18 to 1149.75)	92 (76.8 to 108)	1225 (1023.2 to 1440.67)
Macau Special Administrative Region of China	Tracheal, bronchus, and lung cancer	1.97 (1.79 to 2.16)	555.89 (502.99 to 607.43)	4.14 (3.39 to 5.2)	604.95 (496.04 to 761)
Anhui	Tracheal, bronchus, and lung cancer	324 (280 to 377)	547.64 (473.21 to 636.47)	685 (576 to 818)	1119.86 (941.61 to 1337.13)
Beijing	Tracheal, bronchus, and lung cancer	76.1 (65.8 to 87.6)	678.22 (586.25 to 781.02)	170 (139 to 201)	703.88 (576.67 to 832.94)
Chongqing	Tracheal, bronchus, and lung cancer	116 (98.2 to 136)	734.8 (622.37 to 863.26)	396 (317 to 494)	1480.75 (1186.99 to 1848.17)
Fujian	Tracheal, bronchus, and lung cancer	120 (107 to 135)	380.56 (338.66 to 427.84)	377 (311 to 456)	955.45 (789.38 to 1156.03)
Gansu	Tracheal, bronchus, and lung cancer	82 (72.1 to 93.7)	346.89 (304.75 to 396.16)	144 (120 to 172)	542.65 (451.97 to 646.94)
Guangdong	Tracheal, bronchus, and lung cancer	375 (317 to 437)	569.09 (480.98 to 663.65)	891 (744 to 1060)	762.98 (636.87 to 907.04)
Guangxi	Tracheal, bronchus, and lung cancer	167 (144 to 198)	384.58 (330.28 to 455.31)	414 (348 to 497)	857.46 (719.16 to 1028.1)
Guizhou	Tracheal, bronchus, and lung cancer	144 (121 to 171)	432.47 (365.28 to 514.95)	265 (218 to 317)	784.48 (644.17 to 938.3)
Hainan	Tracheal, bronchus, and lung cancer	31.5 (25.4 to 38.2)	452.21 (365.07 to 548.38)	59.8 (48.1 to 75)	623.6 (501.73 to 782.47)
Hebei	Tracheal, bronchus, and lung cancer	283 (253 to 315)	444.29 (397.54 to 494.4)	747 (619 to 884)	977.17 (809.69 to 1156.84)
Heilongjiang	Tracheal, bronchus, and lung cancer	307 (275 to 347)	842.08 (754.7 to 951.4)	753 (626 to 893)	1918.39 (1594.24 to 2275.08)
Henan	Tracheal, bronchus, and lung cancer	291 (263 to 325)	329.79 (297.5 to 367.95)	907 (758 to 1070)	942.21 (786.97 to 1113.63)
Hubei	Tracheal, bronchus, and lung cancer	299 (268 to 332)	538.03 (482.89 to 596.82)	751 (628 to 891)	1357.37 (1135 to 1610.69)

Hunan	Tracheal, bronchus, and lung cancer	339 (305 to 383)	545.27 (490.44 to 616.03)	885 (742 to 1070)	1318.57 (1104.37 to 1590.24)
Inner Mongolia	Tracheal, bronchus, and lung cancer	126 (108 to 146)	553.95 (473.03 to 642.39)	247 (208 to 292)	953.81 (804.11 to 1127.58)
Jiangsu	Tracheal, bronchus, and lung cancer	450 (405 to 499)	653.31 (588.07 to 724.69)	1010 (841 to 1200)	1238.83 (1030.21 to 1469.82)
Jiangxi	Tracheal, bronchus, and lung cancer	240 (212 to 272)	607.41 (535.34 to 686.99)	418 (356 to 492)	878.51 (748.57 to 1035.75)
Jilin	Tracheal, bronchus, and lung cancer	169 (148 to 195)	652.49 (569.75 to 751.01)	337 (273 to 398)	1207.93 (980.48 to 1425.64)
Liaoning	Tracheal, bronchus, and lung cancer	332 (282 to 392)	812.81 (691.63 to 961.67)	795 (661 to 946)	1780.95 (1480.92 to 2118.38)
Ningxia	Tracheal, bronchus, and lung cancer	16.9 (14.5 to 19.6)	341.88 (292.88 to 397.54)	39.6 (32 to 48.1)	564.65 (456.21 to 685.23)
Qinghai	Tracheal, bronchus, and lung cancer	13.2 (11.4 to 15.4)	279.1 (240 to 324.91)	31.3 (25.4 to 37.3)	498.08 (403.61 to 594.22)
Shaanxi	Tracheal, bronchus, and lung cancer	76.8 (67.6 to 86.8)	223.11 (196.49 to 252.28)	288 (230 to 358)	728.89 (582.97 to 905.15)
Shandong	Tracheal, bronchus, and lung cancer	642 (559 to 733)	749.86 (652.55 to 855.91)	1460 (1220 to 1730)	1470.82 (1227.17 to 1737.88)
Shanghai	Tracheal, bronchus, and lung cancer	113 (98.8 to 130)	810.45 (707.04 to 933.73)	207 (170 to 251)	739.38 (609.17 to 897.39)
Shanxi	Tracheal, bronchus, and lung cancer	154 (134 to 178)	509.98 (441.28 to 587.26)	318 (253 to 387)	822.13 (654.64 to 1002.84)
Sichuan	Tracheal, bronchus, and lung cancer	444 (391 to 505)	397.66 (350.16 to 452.77)	1260 (1020 to 1480)	1508.43 (1226.91 to 1778.62)
Tianjin	Tracheal, bronchus, and lung cancer	88 (76.8 to 99.3)	956.7 (834.72 to 1079.45)	193 (163 to 232)	1266.04 (1067.31 to 1517.41)
Tibet	Tracheal, bronchus, and lung cancer	2.87 (2.43 to 3.35)	123.31 (104.49 to 143.61)	4.09 (3.37 to 4.94)	118.42 (97.54 to 142.94)
Xinjiang	Tracheal, bronchus, and lung cancer	40.3 (35.9 to 45.7)	255.37 (227.5 to 289.69)	121 (99.8 to 143)	495.17 (409.58 to 586.49)
Yunnan	Tracheal, bronchus, and lung cancer	158 (134 to 190)	405.87 (344.58 to 487.65)	337 (284 to 403)	684.52 (576.6 to 818.81)
Zhejiang	Tracheal, bronchus, and lung cancer	265 (236 to 305)	601.82 (534.32 to 691)	645 (530 to 774)	1053.64 (866.82 to 1265.26)
China	Breast cancer	1360 (1220 to 1650)	113.28 (102.11 to 137.61)	2640 (2220 to 2860)	186.79 (157.32 to 202.54)
Taiwan	Breast cancer	25 (24.1 to 26.1)	122.75 (118.3 to 127.7)	73.1 (66.6 to 80.3)	309.99 (282.24 to 340.36)
Hong Kong Special Administrative Region of China	Breast cancer	8.74 (6.76 to 9.9)	151.57 (117.29 to 171.67)	20.4 (13.8 to 27)	271.38 (184.01 to 359.87)
Macau Special Administrative Region of China	Breast cancer	0.403 (0.331 to 0.493)	113.46 (93.39 to 138.98)	1.05 (0.71 to 1.59)	152.99 (103.85 to 232.13)
Anhui	Breast cancer	63.2 (51.9 to 81.7)	106.67 (87.51 to 137.9)	118 (87.3 to 148)	193.77 (142.72 to 242.65)
Beijing	Breast cancer	17.2 (14.1 to 21.1)	153.75 (125.61 to 188.36)	36.8 (22.7 to 48.6)	152.65 (94.3 to 201.81)
Chongqing	Breast cancer	18.3 (13.7 to 25)	115.9 (87.09 to 158.51)	47.6 (34.2 to 61.7)	177.99 (127.84 to 231)
Fujian	Breast cancer	32.6 (26.3 to 41.3)	103.16 (83.08 to 130.46)	65.8 (44.1 to 86.1)	166.97 (111.95 to 218.46)
Gansu	Breast cancer	24.4 (20.2 to 30)	103.17 (85.43 to 126.95)	46.2 (36 to 57.6)	173.73 (135.6 to 216.64)
Guangdong	Breast cancer	72.6 (56.1 to 95.7)	110.27 (85.19 to 145.34)	187 (134 to 241)	159.97 (114.52 to 206.22)
Guangxi	Breast cancer	47.1 (36.3 to 64.8)	108.2 (83.56 to 149.01)	94.2 (67.1 to 121)	194.92 (138.83 to 251.09)
Guizhou	Breast cancer	37 (28.7 to 57.5)	111.6 (86.39 to 173.26)	54.7 (40.4 to 72)	161.8 (119.54 to 212.72)
Hainan	Breast cancer	7.27 (4.82 to 10.6)	104.35 (69.26 to 152.38)	13.1 (9.19 to 17.7)	136.53 (95.87 to 185.1)
Hebei	Breast cancer	60.9 (51.3 to 70.8)	95.75 (80.6 to 111.32)	143 (111 to 181)	187.6 (144.62 to 237.15)
Heilongjiang	Breast cancer	50.6 (38.6 to 69)	138.92 (105.93 to 189.29)	87.8 (67.7 to 111)	223.79 (172.43 to 282.12)

Henan	Breast cancer	79.4<br>(67.5 to 94.6)	89.93<br>(76.45 to 107.21)	181<br>(141 to 227)	187.49<br>(146.37 to 235.52)
Hubei	Breast cancer	54.9<br>(46.7 to 71.5)	98.77<br>(84.08 to 128.61)	116<br>(87.9 to 148)	210.05<br>(158.93 to 267.47)
Hunan	Breast cancer	91.6<br>(71.7 to 164)	147.31<br>(115.26 to 264.47)	138<br>(108 to 173)	206<br>(160.22 to 257.48)
Inner Mongolia	Breast cancer	25.5<br>(19.5 to 33.8)	112.13<br>(85.86 to 148.53)	51.7<br>(39.4 to 66)	199.65<br>(152.22 to 254.75)
Jiangsu	Breast cancer	82.9<br>(69.7 to 104)	120.42<br>(101.27 to 151.15)	167<br>(121 to 216)	204.75<br>(147.98 to 264.46)
Jiangxi	Breast cancer	45.8<br>(34.1 to 72.8)	115.97<br>(86.24 to 184.27)	74.1<br>(56.8 to 92.3)	155.96<br>(119.46 to 194.26)
Jilin	Breast cancer	38.9<br>(27.3 to 51.9)	149.8<br>(105.22 to 200.18)	63.8<br>(47.5 to 82.5)	228.93<br>(170.17 to 295.64)
Liaoning	Breast cancer	62.4<br>(47.4 to 83.4)	152.87<br>(116.13 to 204.46)	115<br>(85.7 to 148)	258.01<br>(191.99 to 332.57)
Ningxia	Breast cancer	4.62<br>(3.48 to 6.15)	93.61<br>(70.49 to 124.62)	10.1<br>(7.17 to 13.4)	143.2<br>(102.12 to 190.3)
Qinghai	Breast cancer	4.27<br>(3.27 to 7.18)	90.09<br>(68.96 to 151.69)	9.38<br>(6.96 to 12.7)	149.26<br>(110.83 to 201.99)
Shaanxi	Breast cancer	28.8<br>(20.8 to 35.2)	83.74<br>(60.49 to 102.41)	67.1<br>(49.2 to 86.6)	169.87<br>(124.4 to 219.26)
Shandong	Breast cancer	109<br>(86.1 to 142)	126.93<br>(100.56 to 165.89)	212<br>(158 to 273)	213.36<br>(159.45 to 274.76)
Shanghai	Breast cancer	22.3<br>(18.1 to 26.9)	159.47<br>(129.6 to 192.71)	43.6<br>(25.5 to 58.1)	156.19<br>(91.44 to 207.98)
Shanxi	Breast cancer	32.4<br>(25.5 to 43)	106.95<br>(84.15 to 142.06)	62.5<br>(45.5 to 83.8)	161.9<br>(117.67 to 217)
Sichuan	Breast cancer	104<br>(87.2 to 142)	93.55<br>(78.13 to 126.9)	160<br>(118 to 207)	191.12<br>(141.58 to 247.43)
Tianjin	Breast cancer	12.8<br>(10.1 to 16.1)	139.48<br>(110.27 to 175.57)	25.1<br>(18.8 to 31.8)	164.69<br>(123.35 to 208.53)
Tibet	Breast cancer	2.38<br>(1.79 to 4.79)	102<br>(76.92 to 205.48)	4.3<br>(3.29 to 5.55)	124.54<br>(95.12 to 160.43)
Xinjiang	Breast cancer	13.8<br>(11.4 to 20.8)	87.8<br>(72.33 to 132.03)	36.6<br>(27.7 to 48.3)	150.18<br>(113.62 to 198.41)
Yunnan	Breast cancer	39.6<br>(30.7 to 58.2)	101.71<br>(78.75 to 149.46)	80.4<br>(63.8 to 101)	163.45<br>(129.7 to 205.21)
Zhejiang	Breast cancer	60.1<br>(46.5 to 81.6)	136.26<br>(105.52 to 185.15)	104<br>(68.7 to 137)	170.12<br>(112.29 to 223.1)
China	Cervical cancer	800<br>(726 to 1200)	66.86<br>(60.66 to 100.31)	1340<br>(841 to 1460)	95<br>(59.56 to 103.47)
Taiwan	Cervical cancer	28.7<br>(27.6 to 29.9)	140.8<br>(135.45 to 146.4)	19.7<br>(17.8 to 21.8)	83.63<br>(75.58 to 92.61)
Hong Kong Special Administrative Region of China	Cervical cancer	4.93<br>(2.63 to 5.7)	85.49<br>(45.61 to 98.81)	5.85<br>(3.55 to 7.76)	77.83<br>(47.28 to 103.36)
Macau Special Administrative Region of China	Cervical cancer	0.185<br>(0.153 to 0.214)	52.2<br>(43.14 to 60.34)	0.28<br>(0.204 to 0.381)	40.88<br>(29.87 to 55.75)
Anhui	Cervical cancer	40.2<br>(33.5 to 58.6)	67.79<br>(56.58 to 98.95)	62.9<br>(37.1 to 80.5)	102.85<br>(60.64 to 131.67)
Beijing	Cervical cancer	4.54<br>(3.52 to 8.91)	40.52<br>(31.35 to 79.41)	8.83<br>(6.17 to 11.9)	36.63<br>(25.59 to 49.21)
Chongqing	Cervical cancer	13<br>(9.84 to 18.7)	82.64<br>(62.33 to 118.4)	27<br>(14.5 to 36.5)	101.18<br>(54.12 to 136.71)
Fujian	Cervical cancer	24.6<br>(20.3 to 31.4)	77.84<br>(64.1 to 99.18)	38.7<br>(18.4 to 51.4)	98.14<br>(46.58 to 130.43)
Gansu	Cervical cancer	16.8<br>(13.7 to 25.7)	71.01<br>(58.05 to 108.76)	29.9<br>(19.3 to 38.2)	112.37<br>(72.55 to 143.61)
Guangdong	Cervical cancer	37.6<br>(29 to 58.8)	57.16<br>(44.07 to 89.37)	80.8<br>(43.6 to 105)	69.16<br>(37.34 to 89.64)
Guangxi	Cervical cancer	31.7<br>(24.7 to 43.6)	72.9<br>(56.88 to 100.18)	59.2<br>(30 to 78.3)	122.55<br>(62.01 to 161.97)
Guizhou	Cervical cancer	32.1<br>(24.9 to 47.5)	96.65<br>(75 to 143.01)	41.7<br>(26.4 to 57.1)	123.19<br>(78.03 to 168.91)
Hainan	Cervical cancer	7.41<br>(4.65 to 9.59)	106.4<br>(66.7 to 137.73)	7.57<br>(4.3 to 10.4)	78.96<br>(44.86 to 108.16)

Hebei	Cervical cancer	20.9<br>(17 to 36.2)	32.87<br>(26.71 to 56.85)	44.5<br>(33 to 58.6)	58.24<br>(43.22 to 76.69)
Heilongjiang	Cervical cancer	27.5<br>(21.5 to 39.9)	75.31<br>(59.08 to 109.56)	36.9<br>(27.1 to 47.3)	93.95<br>(68.96 to 120.51)
Henan	Cervical cancer	35.5<br>(29 to 60.3)	40.17<br>(32.81 to 68.27)	87.6<br>(58.3 to 111)	90.95<br>(60.49 to 115.45)
Hubei	Cervical cancer	29.9<br>(25 to 45.8)	53.9<br>(44.93 to 82.5)	74.8<br>(38.2 to 97.4)	135.28<br>(69.14 to 176.14)
Hunan	Cervical cancer	91.3<br>(74.3 to 110)	146.74<br>(119.41 to 176.39)	115<br>(53.6 to 148)	171.38<br>(79.87 to 220.73)
Inner Mongolia	Cervical cancer	14<br>(10.5 to 25.3)	61.43<br>(46.07 to 111.04)	19.9<br>(14.3 to 25.9)	76.89<br>(55.34 to 99.84)
Jiangsu	Cervical cancer	39.3<br>(32.2 to 61.9)	57.13<br>(46.76 to 89.98)	72.3<br>(43 to 94.9)	88.53<br>(52.69 to 116.24)
Jiangxi	Cervical cancer	41.2<br>(33 to 57)	104.32<br>(83.36 to 144.07)	43.5<br>(25.5 to 55.9)	91.56<br>(53.67 to 117.49)
Jilin	Cervical cancer	16.9<br>(13.4 to 25.4)	65.18<br>(51.48 to 97.96)	23<br>(16.4 to 30.1)	82.49<br>(58.95 to 108.09)
Liaoning	Cervical cancer	30.3<br>(23.6 to 39.3)	74.27<br>(57.74 to 96.25)	50.5<br>(31.3 to 67.1)	113.19<br>(70.11 to 150.18)
Ningxia	Cervical cancer	2.78<br>(2.12 to 4.36)	56.35<br>(42.86 to 88.37)	5.61<br>(3.62 to 7.59)	79.98<br>(51.51 to 108.17)
Qinghai	Cervical cancer	3.71<br>(2.8 to 6.62)	78.4<br>(59.09 to 139.86)	8.21<br>(5.68 to 11.1)	130.69<br>(90.44 to 176.8)
Shaanxi	Cervical cancer	9.99<br>(8.05 to 13.8)	29.02<br>(23.38 to 40.14)	40.2<br>(22.8 to 53.6)	101.87<br>(57.83 to 135.67)
Shandong	Cervical cancer	45.4<br>(35.2 to 84)	53.02<br>(41.12 to 98.15)	79.1<br>(56.8 to 102)	79.61<br>(57.15 to 102.24)
Shanghai	Cervical cancer	6.58<br>(5.3 to 9.78)	47.07<br>(37.93 to 70.02)	9.87<br>(6.77 to 13.1)	35.31<br>(24.23 to 46.98)
Shanxi	Cervical cancer	27.6<br>(21.9 to 33.9)	91.3<br>(72.17 to 112.02)	43<br>(19.1 to 59.2)	111.38<br>(49.4 to 153.39)
Sichuan	Cervical cancer	59.9<br>(48.9 to 101)	53.63<br>(43.79 to 90.26)	107<br>(62.3 to 141)	127.85<br>(74.61 to 169.44)
Tianjin	Cervical cancer	4.24<br>(3.19 to 8.88)	46.1<br>(34.65 to 96.58)	7.38<br>(5.47 to 9.68)	48.34<br>(35.86 to 63.44)
Tibet	Cervical cancer	1.65<br>(1.14 to 5.52)	70.98<br>(49.09 to 237.04)	2.13<br>(1.56 to 3.84)	61.68<br>(45.1 to 111.1)
Xinjiang	Cervical cancer	13<br>(10.7 to 16.7)	82.36<br>(67.82 to 105.58)	25.5<br>(18.1 to 33.5)	104.83<br>(74.23 to 137.36)
Yunnan	Cervical cancer	25.2<br>(18.2 to 54.1)	64.73<br>(46.74 to 138.95)	42.3<br>(31.4 to 54.7)	86.12<br>(63.89 to 111.27)
Zhejiang	Cervical cancer	40.4<br>(32.4 to 60.2)	91.6<br>(73.52 to 136.58)	40.8<br>(24 to 53.5)	66.73<br>(39.14 to 87.46)
China	Uterine cancer	370<br>(337 to 396)	30.91<br>(28.16 to 33.1)	353<br>(328 to 384)	24.98<br>(23.22 to 27.21)
Taiwan	Uterine cancer	2.52<br>(2.36 to 2.69)	12.33<br>(11.57 to 13.18)	9.74<br>(8.63 to 11)	41.28<br>(36.61 to 46.66)
Hong Kong Special Administrative Region of China	Uterine cancer	1.34<br>(1.16 to 1.54)	23.27<br>(20.13 to 26.7)	2.32<br>(1.73 to 3.08)	30.92<br>(23.05 to 40.95)
Macau Special Administrative Region of China	Uterine cancer	0.0658<br>(0.0555 to 0.076)	18.53<br>(15.65 to 21.41)	0.137<br>(0.101 to 0.182)	20.03<br>(14.81 to 26.64)
Anhui	Uterine cancer	18<br>(15 to 21.3)	30.39<br>(25.28 to 35.92)	13.4<br>(10.3 to 16.9)	21.98<br>(16.86 to 27.64)
Beijing	Uterine cancer	3.15<br>(2.54 to 3.84)	28.07<br>(22.63 to 34.26)	3.39<br>(2.53 to 4.43)	14.07<br>(10.5 to 18.37)
Chongqing	Uterine cancer	5.21<br>(4.03 to 6.66)	33.02<br>(25.57 to 42.2)	5.64<br>(4.18 to 7.55)	21.09<br>(15.62 to 28.25)
Fujian	Uterine cancer	6.39<br>(5.32 to 7.68)	20.18<br>(16.81 to 24.26)	5.88<br>(4.35 to 7.75)	14.93<br>(11.04 to 19.66)
Gansu	Uterine cancer	5.86<br>(4.94 to 7.01)	24.76<br>(20.88 to 29.64)	5.77<br>(4.4 to 7.48)	21.69<br>(16.57 to 28.14)
Guangdong	Uterine cancer	25.8<br>(20.1 to 32.1)	39.15<br>(30.59 to 48.73)	26.9<br>(20.1 to 34.6)	22.99<br>(17.18 to 29.59)
Guangxi	Uterine cancer	13.3<br>(10.3 to 16.3)	30.53<br>(23.77 to 37.38)	12.4<br>(9.12 to 15.8)	25.61<br>(18.88 to 32.74)

Guizhou	Uterine cancer	14.6<br>(11 to 18.4)	43.88<br>(33.01 to 55.34)	12.5<br>(9.12 to 16.9)	36.81<br>(26.97 to 49.9)
Hainan	Uterine cancer	2.6<br>(1.78 to 3.5)	37.37<br>(25.49 to 50.22)	2.78<br>(1.91 to 3.8)	29.04<br>(19.97 to 39.6)
Hebei	Uterine cancer	9.89<br>(8.27 to 11.8)	15.54<br>(13 to 18.52)	21.7<br>(15.7 to 28.8)	28.39<br>(20.49 to 37.67)
Heilongjiang	Uterine cancer	9.34<br>(7.53 to 11.4)	25.62<br>(20.67 to 31.22)	8.52<br>(6.49 to 11.3)	21.7<br>(16.53 to 28.81)
Henan	Uterine cancer	14<br>(11.9 to 16.6)	15.87<br>(13.45 to 18.84)	18.9<br>(14.7 to 24.1)	19.64<br>(15.28 to 25.07)
Hubei	Uterine cancer	10.5<br>(8.9 to 12.2)	18.84<br>(16.02 to 21.93)	12.7<br>(9.67 to 16.4)	22.89<br>(17.48 to 29.59)
Hunan	Uterine cancer	52<br>(42.4 to 61.9)	83.63<br>(68.17 to 99.55)	43.6<br>(32.6 to 54.7)	64.93<br>(48.6 to 81.53)
Inner Mongolia	Uterine cancer	6.34<br>(5.07 to 7.84)	27.86<br>(22.27 to 34.48)	5.41<br>(4.16 to 7.03)	20.89<br>(16.06 to 27.16)
Jiangsu	Uterine cancer	14.3<br>(11.9 to 16.9)	20.76<br>(17.35 to 24.54)	14.6<br>(11 to 19.2)	17.85<br>(13.52 to 23.53)
Jiangxi	Uterine cancer	17.9<br>(14.4 to 21.7)	45.23<br>(36.47 to 54.93)	9.34<br>(7.23 to 11.8)	19.65<br>(15.21 to 24.9)
Jilin	Uterine cancer	9.5<br>(7.53 to 11.5)	36.61<br>(29.04 to 44.3)	7.47<br>(5.57 to 9.76)	26.78<br>(19.96 to 34.99)
Liaoning	Uterine cancer	12.9<br>(9.83 to 16.2)	31.54<br>(24.1 to 39.67)	13.4<br>(9.96 to 17.6)	29.92<br>(22.3 to 39.51)
Ningxia	Uterine cancer	1.11<br>(0.862 to 1.39)	22.4<br>(17.45 to 28.24)	1.26<br>(0.927 to 1.7)	17.99<br>(13.21 to 24.17)
Qinghai	Uterine cancer	1.47<br>(1.15 to 1.81)	31.1<br>(24.26 to 38.2)	1.76<br>(1.29 to 2.37)	28.03<br>(20.48 to 37.72)
Shaanxi	Uterine cancer	4.59<br>(3.66 to 5.83)	13.34<br>(10.62 to 16.94)	8.69<br>(6.5 to 11.4)	21.99<br>(16.44 to 28.89)
Shandong	Uterine cancer	24.4<br>(19.7 to 30.5)	28.55<br>(23.01 to 35.6)	20<br>(15.4 to 25.8)	20.13<br>(15.55 to 25.98)
Shanghai	Uterine cancer	3.74<br>(3.04 to 4.57)	26.79<br>(21.78 to 32.69)	4.23<br>(3.09 to 5.63)	15.16<br>(11.06 to 20.16)
Shanxi	Uterine cancer	16.9<br>(13.2 to 21.3)	55.72<br>(43.69 to 70.44)	13.3<br>(9.38 to 17.9)	34.44<br>(24.3 to 46.22)
Sichuan	Uterine cancer	30.8<br>(26.1 to 36.7)	27.64<br>(23.35 to 32.86)	25.3<br>(18.7 to 32.8)	30.34<br>(22.45 to 39.33)
Tianjin	Uterine cancer	1.99<br>(1.58 to 2.54)	21.63<br>(17.2 to 27.58)	2.47<br>(1.83 to 3.32)	16.2<br>(12.02 to 21.78)
Tibet	Uterine cancer	1.03<br>(0.815 to 1.28)	44.06<br>(34.99 to 54.84)	0.76<br>(0.575 to 0.975)	21.98<br>(16.64 to 28.22)
Xinjiang	Uterine cancer	4.7<br>(4.03 to 5.58)	29.83<br>(25.56 to 35.39)	8.73<br>(6.44 to 11.6)	35.81<br>(26.43 to 47.5)
Yunnan	Uterine cancer	11.4<br>(9 to 14.4)	29.36<br>(23.1 to 37.07)	9.59<br>(7.34 to 12.5)	19.51<br>(14.92 to 25.49)
Zhejiang	Uterine cancer	15<br>(12.2 to 18)	33.94<br>(27.64 to 40.92)	10.2<br>(7.55 to 13.4)	16.62<br>(12.34 to 21.98)
China	Prostate cancer	376<br>(300 to 445)	31.38<br>(25.06 to 37.15)	882<br>(751 to 1130)	62.41<br>(53.14 to 80.28)
Taiwan	Prostate cancer	8.87<br>(6.63 to 10.7)	43.46<br>(32.51 to 52.28)	23.2<br>(17.1 to 28.5)	98.56<br>(72.59 to 120.76)
Hong Kong Special Administrative Region of China	Prostate cancer	2.76<br>(2.19 to 3.28)	47.9<br>(37.92 to 56.96)	8.17<br>(6.09 to 10.7)	108.82<br>(81.14 to 142.75)
Macau Special Administrative Region of China	Prostate cancer	0.13<br>(0.0998 to 0.152)	36.63<br>(28.13 to 42.95)	0.365<br>(0.242 to 0.469)	53.39<br>(35.32 to 68.6)
Anhui	Prostate cancer	32<br>(23.3 to 40.5)	54.03<br>(39.38 to 68.39)	42.4<br>(32.9 to 57)	69.34<br>(53.81 to 93.21)
Beijing	Prostate cancer	4.24<br>(3.23 to 5.32)	37.76<br>(28.82 to 47.44)	14.3<br>(10.7 to 18.7)	59.27<br>(44.25 to 77.77)
Chongqing	Prostate cancer	4.38<br>(3.4 to 5.42)	27.78<br>(21.56 to 34.33)	23.5<br>(17.2 to 32.1)	87.74<br>(64.51 to 120.25)
Fujian	Prostate cancer	7.66<br>(5.91 to 9.35)	24.21<br>(18.69 to 29.55)	22<br>(17 to 29.6)	55.82<br>(43.07 to 75.16)
Gansu	Prostate cancer	6<br>(4.46 to 7.2)	25.35<br>(18.87 to 30.45)	14.7<br>(11.2 to 18.8)	55.35<br>(42.32 to 70.9)

Guangdong	Prostate cancer	18<br>(13.9 to 22.3)	27.31<br>(21.16 to 33.91)	59.1<br>(45.9 to 78.1)	50.61<br>(39.31 to 66.85)
Guangxi	Prostate cancer	12.8<br>(9.74 to 15.8)	29.34<br>(22.4 to 36.44)	28.3<br>(22.3 to 38)	58.6<br>(46.1 to 78.67)
Guizhou	Prostate cancer	10<br>(8 to 12.5)	30.24<br>(24.11 to 37.52)	19.8<br>(15.4 to 27)	58.6<br>(45.59 to 79.96)
Hainan	Prostate cancer	1.76<br>(1.38 to 2.24)	25.22<br>(19.87 to 32.19)	5.06<br>(3.72 to 6.83)	52.75<br>(38.78 to 71.25)
Hebei	Prostate cancer	19.7<br>(15.1 to 23.3)	30.92<br>(23.79 to 36.58)	38.6<br>(29.9 to 50.1)	50.53<br>(39.11 to 65.49)
Heilongjiang	Prostate cancer	9.19<br>(7.65 to 11.6)	25.2<br>(20.99 to 31.91)	26.1<br>(20.1 to 36.1)	66.6<br>(51.24 to 91.97)
Henan	Prostate cancer	24.5<br>(19 to 29.3)	27.76<br>(21.55 to 33.21)	52<br>(39.9 to 68.1)	53.98<br>(41.38 to 70.7)
Hubei	Prostate cancer	16.1<br>(12.4 to 19.3)	28.92<br>(22.26 to 34.68)	39.6<br>(30.3 to 54.5)	71.59<br>(54.71 to 98.48)
Hunan	Prostate cancer	19.1<br>(14.9 to 23.4)	30.69<br>(24.04 to 37.63)	44.9<br>(34.5 to 59.9)	66.86<br>(51.31 to 89.16)
Inner Mongolia	Prostate cancer	5.94<br>(4.56 to 7.34)	26.11<br>(20.03 to 32.26)	15.2<br>(11.7 to 19.8)	58.71<br>(45.21 to 76.33)
Jiangsu	Prostate cancer	24.7<br>(19.5 to 29.5)	35.84<br>(28.33 to 42.92)	64.4<br>(49.8 to 84)	78.82<br>(60.99 to 102.86)
Jiangxi	Prostate cancer	10<br>(7.83 to 12.1)	25.38<br>(19.82 to 30.6)	24.9<br>(19.6 to 32.8)	52.28<br>(41.23 to 68.97)
Jilin	Prostate cancer	7.48<br>(6.14 to 9.27)	28.81<br>(23.65 to 35.71)	17.6<br>(13.1 to 22.8)	63.15<br>(46.9 to 81.82)
Liaoning	Prostate cancer	14.1<br>(11.1 to 17.7)	34.68<br>(27.3 to 43.29)	34.4<br>(26.6 to 45.7)	76.94<br>(59.61 to 102.4)
Ningxia	Prostate cancer	1.34<br>(0.943 to 1.63)	27.1<br>(19.09 to 32.91)	3.12<br>(2.24 to 4.16)	44.43<br>(31.95 to 59.32)
Qinghai	Prostate cancer	0.949<br>(0.723 to 1.15)	20.04<br>(15.26 to 24.27)	2.49<br>(1.9 to 3.35)	39.7<br>(30.26 to 53.28)
Shaanxi	Prostate cancer	12.1<br>(9.07 to 15)	35.16<br>(26.35 to 43.71)	23.3<br>(16.9 to 31.7)	58.92<br>(42.81 to 80.24)
Shandong	Prostate cancer	27.8<br>(21.4 to 34.6)	32.47<br>(25 to 40.48)	66.4<br>(51.9 to 87.4)	66.85<br>(52.24 to 87.98)
Shanghai	Prostate cancer	5.86<br>(4.37 to 7.25)	41.94<br>(31.26 to 51.87)	17.1<br>(12.3 to 22.2)	61.02<br>(44.06 to 79.52)
Shanxi	Prostate cancer	10.4<br>(7.93 to 12.4)	34.19<br>(26.2 to 40.96)	21.7<br>(16 to 29.4)	56.14<br>(41.44 to 76.23)
Sichuan	Prostate cancer	34.4<br>(26.6 to 41.2)	30.84<br>(23.79 to 36.95)	62.9<br>(46.4 to 83.9)	75.36<br>(55.51 to 100.49)
Tianjin	Prostate cancer	3.67<br>(2.85 to 4.5)	39.91<br>(31.04 to 48.88)	9.44<br>(7.37 to 12.4)	61.82<br>(48.28 to 81.5)
Tibet	Prostate cancer	0.482<br>(0.358 to 0.596)	20.69<br>(15.35 to 25.58)	0.865<br>(0.662 to 1.13)	25.04<br>(19.15 to 32.67)
Xinjiang	Prostate cancer	4.31<br>(3.16 to 5.09)	27.31<br>(20.04 to 32.28)	11.6<br>(8.38 to 14.8)	47.53<br>(34.38 to 60.81)
Yunnan	Prostate cancer	10.1<br>(7.77 to 12.9)	26.01<br>(19.94 to 33.21)	24.4<br>(19.4 to 34)	49.64<br>(39.49 to 69.07)
Zhejiang	Prostate cancer	13.7<br>(10.7 to 16.6)	31.06<br>(24.32 to 37.73)	42.9<br>(32.6 to 58.5)	70.15<br>(53.25 to 95.63)
China	Colon and rectum cancer	2120<br>(1990 to 2330)	177.38<br>(166.54 to 194.44)	4250<br>(4010 to 4470)	301.18<br>(284.17 to 316.17)
Taiwan	Colon and rectum cancer	47.5<br>(46.3 to 48.8)	232.95<br>(227.04 to 239.12)	139<br>(131 to 148)	588.91<br>(553.69 to 628.79)
Hong Kong Special Administrative Region of China	Colon and rectum cancer	26.9<br>(21.8 to 29.6)	466.99<br>(378.68 to 513.25)	37.4<br>(31 to 44.5)	497.4<br>(412.15 to 592.92)
Macau Special Administrative Region of China	Colon and rectum cancer	0.733<br>(0.662 to 0.835)	206.51<br>(186.59 to 235.22)	1.77<br>(1.39 to 2.25)	258.24<br>(203.6 to 328.89)
Anhui	Colon and rectum cancer	103<br>(87.7 to 120)	174.06<br>(148.01 to 202.13)	178<br>(150 to 210)	291.52<br>(246.02 to 342.71)
Beijing	Colon and rectum cancer	27.8<br>(24.4 to 31.8)	247.69<br>(217.53 to 283.16)	61.2<br>(47.9 to 73.2)	253.77<br>(198.79 to 303.83)
Chongqing	Colon and rectum cancer	32.9<br>(27.7 to 39.7)	208.22<br>(175.46 to 251.36)	84.8<br>(68.5 to 104)	317.39<br>(256.47 to 389.93)

Fujian	Colon and rectum cancer	49.7 (43.7 to 58.3)	157.13 (138.05 to 184.41)	126 (103 to 153)	320.31 (260.72 to 387.98)
Gansu	Colon and rectum cancer	41.2 (35.6 to 47.9)	174.33 (150.35 to 202.53)	63.5 (53.2 to 73.9)	238.97 (200.19 to 277.95)
Guangdong	Colon and rectum cancer	145 (123 to 173)	220.37 (186.53 to 262.5)	311 (259 to 373)	266.23 (221.71 to 319.29)
Guangxi	Colon and rectum cancer	69 (58 to 83.3)	158.62 (133.41 to 191.58)	141 (117 to 171)	292.66 (242.72 to 352.98)
Guizhou	Colon and rectum cancer	73.9 (62.3 to 90.8)	222.55 (187.55 to 273.53)	119 (97.8 to 142)	351.86 (289.06 to 420.84)
Hainan	Colon and rectum cancer	9.74 (7.49 to 12.4)	139.79 (107.49 to 177.33)	18.4 (14.7 to 22.8)	192.16 (152.91 to 237.32)
Hebei	Colon and rectum cancer	83.7 (75.3 to 93.1)	131.44 (118.38 to 146.25)	218 (182 to 258)	285.41 (238.11 to 337.59)
Heilongjiang	Colon and rectum cancer	63.8 (55.5 to 74.4)	175.09 (152.39 to 204.24)	133 (110 to 156)	337.59 (281.04 to 398.32)
Henan	Colon and rectum cancer	94.1 (84.3 to 108)	106.66 (95.48 to 122.58)	219 (184 to 264)	227.35 (190.74 to 274.25)
Hubei	Colon and rectum cancer	88.1 (79.3 to 98.7)	158.65 (142.72 to 177.65)	195 (164 to 231)	353.24 (295.83 to 417.63)
Hunan	Colon and rectum cancer	134 (117 to 159)	216.08 (188.74 to 256.38)	267 (225 to 317)	398.34 (334.9 to 472.76)
Inner Mongolia	Colon and rectum cancer	40.4 (34.6 to 47.2)	177.72 (152.2 to 207.67)	77.8 (64.9 to 93.4)	300.57 (250.75 to 360.55)
Jiangsu	Colon and rectum cancer	122 (110 to 137)	177.87 (160.44 to 199.8)	265 (221 to 312)	324.72 (270.5 to 381.94)
Jiangxi	Colon and rectum cancer	90.9 (78.8 to 110)	230.02 (199.43 to 277.38)	120 (102 to 142)	252.55 (214.61 to 298.03)
Jilin	Colon and rectum cancer	51.5 (44.9 to 60)	198.49 (173.21 to 231.1)	93.3 (75.8 to 111)	334.65 (271.68 to 398.14)
Liaoning	Colon and rectum cancer	90.8 (77.7 to 106)	222.68 (190.56 to 260.84)	208 (171 to 247)	465.16 (383.72 to 553.89)
Ningxia	Colon and rectum cancer	5.85 (4.97 to 6.88)	118.48 (100.61 to 139.26)	12.4 (10 to 15.2)	176.49 (142.71 to 216.99)
Qinghai	Colon and rectum cancer	7.46 (6.37 to 8.84)	157.57 (134.43 to 186.73)	15.3 (12.4 to 18.4)	243.63 (198.07 to 293.04)
Shaanxi	Colon and rectum cancer	30.8 (27.6 to 34.4)	89.41 (80.11 to 100.11)	82.8 (66.8 to 102)	209.69 (168.97 to 259.18)
Shandong	Colon and rectum cancer	145 (125 to 172)	169.96 (145.74 to 201.48)	276 (231 to 330)	277.24 (232.47 to 332.18)
Shanghai	Colon and rectum cancer	46.6 (39.6 to 53.7)	333.72 (283.57 to 384.25)	96.6 (73.1 to 117)	345.84 (261.64 to 419.22)
Shanxi	Colon and rectum cancer	50.4 (43.5 to 59.1)	166.58 (143.57 to 195.14)	84.1 (67.8 to 103)	217.85 (175.58 to 265.95)
Sichuan	Colon and rectum cancer	184 (164 to 209)	164.64 (147.06 to 187.36)	331 (272 to 388)	396.07 (326.21 to 464.45)
Tianjin	Colon and rectum cancer	17.4 (15 to 20)	188.7 (162.65 to 216.95)	42.5 (35.9 to 50.9)	278.45 (235.5 to 333.69)
Tibet	Colon and rectum cancer	3.44 (2.89 to 4.05)	147.56 (124.15 to 173.68)	4.68 (3.91 to 5.67)	135.53 (113.09 to 164.15)
Xinjiang	Colon and rectum cancer	17.5 (15.6 to 20.5)	111.06 (98.77 to 129.96)	47.9 (38.7 to 59.3)	196.69 (158.98 to 243.36)
Yunnan	Colon and rectum cancer	68.2 (57.8 to 85.2)	175.08 (148.25 to 218.71)	131 (111 to 154)	265.54 (225.01 to 313.45)
Zhejiang	Colon and rectum cancer	106 (92.2 to 126)	240.46 (209.17 to 286.15)	191 (152 to 231)	312.81 (248.09 to 377.07)
China	Lip and oral cavity cancer	205 (194 to 215)	17.09 (16.22 to 17.99)	510 (478 to 538)	36.09 (33.86 to 38.1)
Taiwan	Lip and oral cavity cancer	12.1 (11.6 to 12.6)	59.29 (56.99 to 61.65)	66.3 (60.1 to 72.2)	281.2 (254.71 to 306.34)
Hong Kong Special Administrative Region of China	Lip and oral cavity cancer	3.36 (3 to 3.81)	58.35 (52.05 to 66.13)	2.92 (2.37 to 3.58)	38.87 (31.52 to 47.69)
Macau Special Administrative Region of China	Lip and oral cavity cancer	0.0562 (0.0495 to 0.0633)	15.82 (13.93 to 17.83)	0.131 (0.0931 to 0.179)	19.21 (13.62 to 26.19)
Anhui	Lip and oral cavity cancer	11.6 (9.7 to 13.8)	19.53 (16.36 to 23.35)	20.1 (16.8 to 24.2)	32.87 (27.42 to 39.56)

Beijing	Lip and oral cavity cancer	2.12 (1.81 to 2.47)	18.88 (16.16 to 22.04)	4.47 (3.59 to 5.36)	18.54 (14.91 to 22.25)
Chongqing	Lip and oral cavity cancer	2.93 (2.51 to 3.43)	18.54 (15.93 to 21.74)	8.87 (7.04 to 11.1)	33.19 (26.35 to 41.67)
Fujian	Lip and oral cavity cancer	4.77 (4.28 to 5.34)	15.08 (13.54 to 16.87)	11.9 (9.7 to 14.4)	30.2 (24.61 to 36.48)
Gansu	Lip and oral cavity cancer	3.8 (3.3 to 4.32)	16.05 (13.94 to 18.28)	9.36 (7.54 to 11.3)	35.2 (28.36 to 42.65)
Guangdong	Lip and oral cavity cancer	11.9 (10.2 to 13.8)	18.06 (15.48 to 21.02)	34.2 (28.1 to 41.7)	29.26 (24.09 to 35.7)
Guangxi	Lip and oral cavity cancer	6.77 (5.8 to 7.97)	15.57 (13.33 to 18.34)	16.3 (13.1 to 20.1)	33.64 (27.1 to 41.61)
Guizhou	Lip and oral cavity cancer	7.33 (6.21 to 8.7)	22.09 (18.72 to 26.21)	17.6 (13.6 to 22.1)	52.08 (40.22 to 65.38)
Hainan	Lip and oral cavity cancer	1.08 (0.874 to 1.35)	15.49 (12.54 to 19.38)	2.22 (1.77 to 2.83)	23.11 (18.41 to 29.47)
Hebei	Lip and oral cavity cancer	12.6 (11.1 to 14.4)	19.82 (17.41 to 22.59)	37.1 (29.6 to 46.4)	48.54 (38.78 to 60.73)
Heilongjiang	Lip and oral cavity cancer	5.01 (4.22 to 5.85)	13.74 (11.59 to 16.04)	12.4 (10.1 to 15.2)	31.67 (25.71 to 38.82)
Henan	Lip and oral cavity cancer	10.9 (9.63 to 12.2)	12.32 (10.91 to 13.83)	33.2 (27.3 to 39.9)	34.52 (28.39 to 41.43)
Hubei	Lip and oral cavity cancer	8.11 (7.22 to 9.07)	14.6 (13 to 16.33)	19.1 (15.8 to 22.9)	34.59 (28.6 to 41.45)
Hunan	Lip and oral cavity cancer	13.4 (12 to 14.9)	21.52 (19.3 to 23.96)	40.1 (31.9 to 49.8)	59.76 (47.54 to 74.12)
Inner Mongolia	Lip and oral cavity cancer	3.99 (3.34 to 4.71)	17.54 (14.68 to 20.69)	13.7 (10.9 to 16.9)	52.72 (41.96 to 65.12)
Jiangsu	Lip and oral cavity cancer	11.6 (10.3 to 12.8)	16.81 (15.02 to 18.65)	24 (19.7 to 28.7)	29.33 (24.17 to 35.17)
Jiangxi	Lip and oral cavity cancer	5.69 (5.04 to 6.39)	14.39 (12.75 to 16.16)	13 (10.8 to 15.8)	27.41 (22.73 to 33.18)
Jilin	Lip and oral cavity cancer	6.08 (5.13 to 7.3)	23.44 (19.76 to 28.13)	16.9 (13.2 to 20.7)	60.68 (47.43 to 74.37)
Liaoning	Lip and oral cavity cancer	7.73 (6.46 to 9.26)	18.96 (15.85 to 22.69)	22 (17.9 to 26.8)	49.37 (40.08 to 59.93)
Ningxia	Lip and oral cavity cancer	0.503 (0.431 to 0.585)	10.17 (8.73 to 11.84)	1.05 (0.841 to 1.3)	14.89 (11.98 to 18.47)
Qinghai	Lip and oral cavity cancer	0.625 (0.543 to 0.723)	13.19 (11.45 to 15.26)	1.72 (1.37 to 2.12)	27.45 (21.76 to 33.75)
Shaanxi	Lip and oral cavity cancer	3.96 (3.46 to 4.54)	11.5 (10.05 to 13.21)	13.9 (10.9 to 17.4)	35.23 (27.64 to 44.03)
Shandong	Lip and oral cavity cancer	12.9 (11.2 to 15.1)	15.09 (13.12 to 17.6)	27.3 (22.5 to 32.8)	27.46 (22.68 to 33.02)
Shanghai	Lip and oral cavity cancer	3.2 (2.76 to 3.72)	22.91 (19.74 to 26.62)	6.04 (4.95 to 7.47)	21.62 (17.71 to 26.74)
Shanxi	Lip and oral cavity cancer	5.45 (4.74 to 6.28)	17.99 (15.64 to 20.73)	13.8 (10.7 to 17.1)	35.71 (27.76 to 44.24)
Sichuan	Lip and oral cavity cancer	19.4 (17.2 to 21.8)	17.42 (15.39 to 19.51)	45.2 (35.8 to 54.5)	54.13 (42.93 to 65.25)
Tianjin	Lip and oral cavity cancer	1.72 (1.48 to 2)	18.68 (16.11 to 21.77)	3.64 (3 to 4.4)	23.87 (19.68 to 28.85)
Tibet	Lip and oral cavity cancer	0.283 (0.243 to 0.327)	12.14 (10.42 to 14.02)	0.503 (0.409 to 0.616)	14.56 (11.83 to 17.81)
Xinjiang	Lip and oral cavity cancer	2.19 (1.94 to 2.46)	13.87 (12.32 to 15.62)	5.49 (4.43 to 6.66)	22.52 (18.19 to 27.33)
Yunnan	Lip and oral cavity cancer	5.96 (5.03 to 7.01)	15.28 (12.91 to 18)	18.8 (15.3 to 23.3)	38.32 (31.15 to 47.46)
Zhejiang	Lip and oral cavity cancer	7.66 (6.76 to 8.63)	17.37 (15.33 to 19.57)	12.6 (10.2 to 15.4)	20.61 (16.7 to 25.13)
China	Nasopharynx cancer	920 (848 to 1000)	76.87 (70.84 to 83.76)	817 (763 to 878)	57.81 (54.05 to 62.18)
Taiwan	Nasopharynx cancer	25.3 (24.5 to 26.3)	124.12 (119.88 to 128.76)	24.9 (23 to 27.1)	105.69 (97.34 to 115.12)
Hong Kong Special Administrative Region of China	Nasopharynx cancer	9.6 (8.55 to 10.7)	166.52 (148.31 to 185.98)	4.68 (3.74 to 5.87)	62.37 (49.8 to 78.14)

Macau Special Administrative Region of China	Nasopharynx cancer	0.337 (0.294 to 0.375)	94.83 (82.87 to 105.68)	0.499 (0.361 to 0.706)	72.94 (52.82 to 103.23)
Anhui	Nasopharynx cancer	38.4 (32.5 to 45)	64.77 (54.89 to 76.03)	25.6 (21 to 30.8)	41.81 (34.42 to 50.32)
Beijing	Nasopharynx cancer	3.5 (2.94 to 4.2)	31.18 (26.21 to 37.45)	3.51 (2.74 to 4.3)	14.56 (11.36 to 17.83)
Chongqing	Nasopharynx cancer	14.8 (12.3 to 17.8)	93.49 (77.82 to 112.66)	13.3 (10.5 to 16.7)	49.94 (39.25 to 62.57)
Fujian	Nasopharynx cancer	30.9 (27.1 to 35)	97.52 (85.52 to 110.51)	34.4 (27.5 to 42.1)	87.39 (69.88 to 106.77)
Gansu	Nasopharynx cancer	7.47 (6.36 to 8.76)	31.58 (26.89 to 37.03)	4.35 (3.59 to 5.23)	16.38 (13.51 to 19.67)
Guangdong	Nasopharynx cancer	185 (154 to 224)	281.08 (233.51 to 340.31)	195 (158 to 239)	166.52 (135.29 to 204.14)
Guangxi	Nasopharynx cancer	93.4 (79.3 to 111)	214.68 (182.27 to 255.12)	92.6 (74.6 to 114)	191.65 (154.43 to 236.8)
Guizhou	Nasopharynx cancer	29.3 (24.6 to 35.5)	88.31 (74.26 to 107.02)	19.3 (15.2 to 24.3)	57.07 (44.81 to 71.91)
Hainan	Nasopharynx cancer	12.3 (9.1 to 15.5)	177 (130.69 to 221.84)	11.2 (8.72 to 14.4)	116.42 (90.99 to 150.31)
Hebei	Nasopharynx cancer	12.2 (10.8 to 13.8)	19.23 (17.01 to 21.63)	14.2 (11.7 to 17.4)	18.56 (15.27 to 22.77)
Heilongjiang	Nasopharynx cancer	10.6 (9.03 to 12.3)	29.08 (24.76 to 33.78)	8.87 (7.28 to 11)	22.6 (18.54 to 28.03)
Henan	Nasopharynx cancer	17.2 (15.4 to 19.2)	19.49 (17.42 to 21.73)	17.3 (14.4 to 20.6)	18 (14.93 to 21.42)
Hubei	Nasopharynx cancer	38.7 (34.2 to 44)	69.71 (61.57 to 79.16)	36.9 (30.4 to 44.7)	66.79 (54.91 to 80.92)
Hunan	Nasopharynx cancer	74.6 (65.5 to 84.2)	119.99 (105.31 to 135.39)	73.1 (59.1 to 90.7)	108.91 (88.07 to 135.06)
Inner Mongolia	Nasopharynx cancer	6.4 (5.42 to 7.54)	28.13 (23.82 to 33.14)	4.52 (3.68 to 5.54)	17.44 (14.2 to 21.38)
Jiangsu	Nasopharynx cancer	43.4 (37.7 to 49.4)	63.09 (54.83 to 71.82)	36.9 (30.3 to 45)	45.19 (37.1 to 55.12)
Jiangxi	Nasopharynx cancer	63.5 (55.2 to 73)	160.53 (139.61 to 184.77)	35.3 (29.2 to 42.3)	74.18 (61.44 to 88.99)
Jilin	Nasopharynx cancer	8.15 (6.82 to 9.52)	31.41 (26.28 to 36.69)	5.69 (4.49 to 6.96)	20.39 (16.1 to 24.95)
Liaoning	Nasopharynx cancer	16.2 (13.1 to 19.6)	39.83 (32.07 to 48)	14.3 (11.5 to 17.3)	31.94 (25.82 to 38.85)
Ningxia	Nasopharynx cancer	1.52 (1.3 to 1.8)	30.86 (26.24 to 36.39)	1.38 (1.1 to 1.72)	19.63 (15.63 to 24.46)
Qinghai	Nasopharynx cancer	1.25 (1.07 to 1.47)	26.4 (22.56 to 30.94)	1.13 (0.903 to 1.39)	18.05 (14.37 to 22.13)
Shaanxi	Nasopharynx cancer	5.61 (4.86 to 6.53)	16.29 (14.12 to 18.97)	7.3 (5.83 to 9)	18.48 (14.77 to 22.78)
Shandong	Nasopharynx cancer	31.7 (26.7 to 36.8)	37.08 (31.24 to 42.94)	21.4 (17.8 to 25.9)	21.57 (17.94 to 26.08)
Shanghai	Nasopharynx cancer	9.65 (8.13 to 11.5)	69.03 (58.16 to 82.39)	11 (8.61 to 13.9)	39.34 (30.81 to 49.93)
Shanxi	Nasopharynx cancer	6.43 (5.44 to 7.52)	21.24 (17.96 to 24.84)	4.24 (3.38 to 5.18)	10.97 (8.74 to 13.41)
Sichuan	Nasopharynx cancer	81.7 (71.7 to 93.4)	73.21 (64.28 to 83.69)	60.8 (49.2 to 74.2)	72.88 (58.95 to 88.93)
Tianjin	Nasopharynx cancer	3.32 (2.82 to 3.83)	36.14 (30.61 to 41.66)	3.51 (2.84 to 4.3)	22.99 (18.64 to 28.21)
Tibet	Nasopharynx cancer	0.156 (0.133 to 0.184)	6.71 (5.7 to 7.9)	0.12 (0.0981 to 0.146)	3.46 (2.84 to 4.22)
Xinjiang	Nasopharynx cancer	4.12 (3.57 to 4.72)	26.09 (22.61 to 29.95)	5.2 (4.12 to 6.39)	21.36 (16.89 to 26.22)
Yunnan	Nasopharynx cancer	20.2 (16.9 to 24.9)	51.75 (43.28 to 63.77)	15.1 (12.3 to 18.4)	30.65 (25.11 to 37.41)
Zhejiang	Nasopharynx cancer	38.5 (33.6 to 43.7)	87.36 (76.14 to 99.14)	34.2 (27 to 42.2)	55.97 (44.18 to 69.05)
China	Other pharynx cancer	81.9 (59.1 to 92.2)	6.84 (4.94 to 7.7)	133 (115 to 151)	9.4 (8.17 to 10.68)
Taiwan	Other pharynx cancer	5.36 (5.11 to 5.63)	26.26 (25.06 to 27.6)	26.6 (24 to 29.5)	112.64 (101.61 to 125.02)

Hong Kong Special Administrative Region of China	Other pharynx cancer	1.49<br>(0.484 to 1.78)	25.88<br>(8.4 to 30.94)	0.836<br>(0.617 to 1.07)	11.13<br>(8.21 to 14.26)
Macau Special Administrative Region of China	Other pharynx cancer	0.0331<br>(0.0221 to 0.0385)	9.32<br>(6.23 to 10.86)	0.0626<br>(0.0385 to 0.0902)	9.15<br>(5.62 to 13.19)
Anhui	Other pharynx cancer	3.77<br>(2.55 to 4.66)	6.37<br>(4.3 to 7.86)	4.17<br>(3.32 to 5.63)	6.83<br>(5.43 to 9.21)
Beijing	Other pharynx cancer	0.998<br>(0.652 to 1.25)	8.9<br>(5.82 to 11.17)	2.14<br>(1.42 to 2.77)	8.86<br>(5.87 to 11.51)
Chongqing	Other pharynx cancer	1.49<br>(0.91 to 1.87)	9.46<br>(5.76 to 11.83)	2.78<br>(1.99 to 3.65)	10.4<br>(7.45 to 13.67)
Fujian	Other pharynx cancer	1.45<br>(1.16 to 1.67)	4.59<br>(3.66 to 5.29)	3<br>(2.24 to 4.09)	7.62<br>(5.69 to 10.37)
Gansu	Other pharynx cancer	1.18<br>(0.97 to 1.78)	5<br>(4.1 to 7.53)	1.29<br>(0.979 to 2.41)	4.87<br>(3.69 to 9.08)
Guangdong	Other pharynx cancer	6.96<br>(3.57 to 8.82)	10.58<br>(5.43 to 13.4)	13<br>(7.88 to 16.6)	11.13<br>(6.75 to 14.23)
Guangxi	Other pharynx cancer	4.17<br>(2.23 to 5.11)	9.6<br>(5.14 to 11.74)	6.36<br>(4.27 to 8.1)	13.16<br>(8.84 to 16.76)
Guizhou	Other pharynx cancer	2.12<br>(1.59 to 2.69)	6.4<br>(4.78 to 8.1)	2.62<br>(1.94 to 3.58)	7.76<br>(5.74 to 10.6)
Hainan	Other pharynx cancer	0.557<br>(0.334 to 0.783)	8<br>(4.79 to 11.24)	0.958<br>(0.577 to 1.3)	10<br>(6.02 to 13.59)
Hebei	Other pharynx cancer	3.26<br>(2.66 to 3.89)	5.13<br>(4.17 to 6.11)	6.58<br>(4.66 to 9.13)	8.61<br>(6.1 to 11.95)
Heilongjiang	Other pharynx cancer	2.32<br>(1.84 to 2.9)	6.35<br>(5.04 to 7.96)	4.69<br>(3.57 to 6)	11.96<br>(9.09 to 15.29)
Henan	Other pharynx cancer	3.36<br>(2.88 to 4.12)	3.81<br>(3.26 to 4.66)	5.58<br>(4.29 to 9.69)	5.8<br>(4.46 to 10.06)
Hubei	Other pharynx cancer	3.55<br>(2.58 to 4.25)	6.39<br>(4.64 to 7.64)	6.11<br>(4.69 to 7.86)	11.05<br>(8.47 to 14.21)
Hunan	Other pharynx cancer	5.97<br>(3.79 to 6.96)	9.6<br>(6.09 to 11.19)	11<br>(6.94 to 13.8)	16.31<br>(10.34 to 20.6)
Inner Mongolia	Other pharynx cancer	1.55<br>(1.11 to 1.96)	6.83<br>(4.86 to 8.61)	2.42<br>(1.88 to 3.16)	9.34<br>(7.24 to 12.18)
Jiangsu	Other pharynx cancer	4.29<br>(3.56 to 5.16)	6.23<br>(5.17 to 7.5)	6.53<br>(5.07 to 9.28)	8<br>(6.2 to 11.36)
Jiangxi	Other pharynx cancer	3.51<br>(2.15 to 4.21)	8.88<br>(5.43 to 10.65)	3.9<br>(2.98 to 4.84)	8.21<br>(6.28 to 10.18)
Jilin	Other pharynx cancer	2.11<br>(1.24 to 2.8)	8.12<br>(4.76 to 10.8)	3.7<br>(2.17 to 4.73)	13.27<br>(7.77 to 16.96)
Liaoning	Other pharynx cancer	3.32<br>(2.15 to 4.3)	8.14<br>(5.26 to 10.55)	7.48<br>(4.61 to 9.47)	16.74<br>(10.32 to 21.21)
Ningxia	Other pharynx cancer	0.203<br>(0.165 to 0.246)	4.1<br>(3.35 to 4.97)	0.336<br>(0.253 to 0.511)	4.78<br>(3.61 to 7.27)
Qinghai	Other pharynx cancer	0.218<br>(0.181 to 0.313)	4.6<br>(3.82 to 6.6)	0.358<br>(0.262 to 0.682)	5.7<br>(4.17 to 10.86)
Shaanxi	Other pharynx cancer	1.34<br>(0.949 to 1.63)	3.9<br>(2.76 to 4.73)	2.64<br>(2.03 to 3.72)	6.68<br>(5.13 to 9.42)
Shandong	Other pharynx cancer	6.19<br>(4.53 to 7.69)	7.23<br>(5.29 to 8.99)	9.5<br>(7.33 to 12.5)	9.56<br>(7.38 to 12.56)
Shanghai	Other pharynx cancer	1.05<br>(0.827 to 1.26)	7.53<br>(5.92 to 9)	1.69<br>(1.27 to 2.29)	6.05<br>(4.54 to 8.21)
Shanxi	Other pharynx cancer	1.8<br>(1.47 to 2.2)	5.96<br>(4.85 to 7.27)	2.23<br>(1.7 to 3.48)	5.78<br>(4.39 to 9.02)
Sichuan	Other pharynx cancer	7.21<br>(4.7 to 8.67)	6.46<br>(4.21 to 7.77)	9.76<br>(7.12 to 12.3)	11.68<br>(8.53 to 14.74)
Tianjin	Other pharynx cancer	0.632<br>(0.508 to 0.764)	6.88<br>(5.52 to 8.3)	1.24<br>(0.933 to 1.81)	8.13<br>(6.11 to 11.87)
Tibet	Other pharynx cancer	0.102<br>(0.0805 to 0.208)	4.39<br>(3.45 to 8.91)	0.116<br>(0.0842 to 0.292)	3.34<br>(2.44 to 8.45)
Xinjiang	Other pharynx cancer	0.755<br>(0.634 to 0.901)	4.78<br>(4.02 to 5.71)	1.8<br>(1.27 to 2.43)	7.38<br>(5.2 to 9.98)
Yunnan	Other pharynx cancer	2.03<br>(1.63 to 2.65)	5.21<br>(4.17 to 6.79)	3.02<br>(2.33 to 5.06)	6.14<br>(4.73 to 10.3)
Zhejiang	Other pharynx cancer	2.91<br>(2.09 to 3.41)	6.6<br>(4.75 to 7.73)	4.84<br>(3.59 to 6.58)	7.91<br>(5.87 to 10.76)

China	Gallbladder and biliary tract cancer	284 (253 to 415)	23.71 (21.16 to 34.63)	582 (442 to 637)	41.24 (31.32 to 45.12)
Taiwan	Gallbladder and biliary tract cancer	8.14 (7.79 to 8.9)	39.87 (38.18 to 43.6)	15.2 (13.5 to 16.5)	64.66 (57.05 to 69.76)
Hong Kong Special Administrative Region of China	Gallbladder and biliary tract cancer	3.28 (1.49 to 4.03)	56.91 (25.78 to 69.87)	3.48 (2.48 to 4.53)	46.36 (33.08 to 60.28)
Macau Special Administrative Region of China	Gallbladder and biliary tract cancer	0.0913 (0.0782 to 0.117)	25.74 (22.03 to 33)	0.192 (0.14 to 0.273)	28.04 (20.41 to 39.86)
Anhui	Gallbladder and biliary tract cancer	18.4 (14.8 to 26.3)	31.01 (24.94 to 44.43)	32.2 (19.1 to 39.1)	52.59 (31.28 to 64.02)
Beijing	Gallbladder and biliary tract cancer	4.44 (2.75 to 5.41)	39.61 (24.48 to 48.24)	12.3 (4.51 to 16.4)	50.85 (18.73 to 68.25)
Chongqing	Gallbladder and biliary tract cancer	3 (2.22 to 5.99)	19.01 (14.09 to 37.95)	6.8 (4.58 to 13.5)	25.43 (17.12 to 50.41)
Fujian	Gallbladder and biliary tract cancer	5.85 (4.86 to 8.81)	18.48 (15.36 to 27.85)	12.5 (10 to 15.9)	31.73 (25.43 to 40.31)
Gansu	Gallbladder and biliary tract cancer	4.9 (3.75 to 9.84)	20.7 (15.87 to 41.61)	8.87 (7.2 to 12)	33.36 (27.09 to 45.16)
Guangdong	Gallbladder and biliary tract cancer	14.4 (11.4 to 23.7)	21.91 (17.38 to 35.96)	30.6 (24.5 to 38.8)	26.18 (20.94 to 33.18)
Guangxi	Gallbladder and biliary tract cancer	8.73 (6.82 to 15.9)	20.07 (15.68 to 36.59)	10.5 (7.37 to 20.5)	21.7 (15.25 to 42.51)
Guizhou	Gallbladder and biliary tract cancer	6.64 (4.79 to 14.5)	20 (14.45 to 43.73)	8.93 (6.31 to 17)	26.41 (18.66 to 50.16)
Hainan	Gallbladder and biliary tract cancer	1.17 (0.805 to 2.2)	16.78 (11.56 to 31.65)	1.85 (1.31 to 3.19)	19.34 (13.69 to 33.29)
Hebei	Gallbladder and biliary tract cancer	11.3 (9.09 to 19.8)	17.72 (14.28 to 31.11)	20.5 (15.6 to 33.3)	26.77 (20.44 to 43.6)
Heilongjiang	Gallbladder and biliary tract cancer	7.96 (6.56 to 12)	21.82 (18 to 32.86)	15 (12 to 20.3)	38.13 (30.59 to 51.61)
Henan	Gallbladder and biliary tract cancer	16.7 (13.9 to 26.4)	18.92 (15.74 to 29.91)	38.8 (28.5 to 47.2)	40.29 (29.61 to 48.99)
Hubei	Gallbladder and biliary tract cancer	12.5 (10.6 to 19.1)	22.55 (18.99 to 34.33)	23.4 (18.7 to 29.9)	42.26 (33.74 to 54.02)
Hunan	Gallbladder and biliary tract cancer	14.1 (11.4 to 24)	22.75 (18.37 to 38.6)	25.5 (20.3 to 34.6)	37.99 (30.24 to 51.49)
Inner Mongolia	Gallbladder and biliary tract cancer	4.43 (3.53 to 7.36)	19.49 (15.5 to 32.37)	9.7 (7.77 to 12.4)	37.45 (30.01 to 48.07)
Jiangsu	Gallbladder and biliary tract cancer	21.2 (18.1 to 27.4)	30.81 (26.33 to 39.85)	56 (27.6 to 70.6)	68.56 (33.76 to 86.41)
Jiangxi	Gallbladder and biliary tract cancer	8.51 (6.69 to 16.3)	21.52 (16.92 to 41.22)	13.6 (11.1 to 18.6)	28.6 (23.33 to 39.17)
Jilin	Gallbladder and biliary tract cancer	5.67 (4.53 to 8.62)	21.85 (17.45 to 33.2)	9.93 (7.76 to 12.8)	35.61 (27.81 to 45.97)
Liaoning	Gallbladder and biliary tract cancer	13.6 (10 to 16.7)	33.32 (24.56 to 40.91)	32.9 (16.6 to 41.8)	73.69 (37.12 to 93.72)
Ningxia	Gallbladder and biliary tract cancer	1.01 (0.833 to 1.32)	20.47 (16.87 to 26.71)	2.47 (1.55 to 3.17)	35.26 (22.06 to 45.15)
Qinghai	Gallbladder and biliary tract cancer	1.04 (0.837 to 1.6)	21.98 (17.68 to 33.77)	2.69 (1.83 to 3.36)	42.88 (29.1 to 53.47)
Shaanxi	Gallbladder and biliary tract cancer	7.89 (5.67 to 10)	22.92 (16.48 to 29.18)	24.9 (10.2 to 32.6)	62.98 (25.78 to 82.55)
Shandong	Gallbladder and biliary tract cancer	20 (16.1 to 33.1)	23.41 (18.8 to 38.71)	43.4 (31.8 to 52.9)	43.68 (31.97 to 53.21)
Shanghai	Gallbladder and biliary tract cancer	8.01 (3.71 to 9.96)	57.36 (26.52 to 71.31)	21.3 (5.28 to 29.4)	76.4 (18.91 to 105.22)
Shanxi	Gallbladder and biliary tract cancer	6.69 (5.38 to 10.6)	22.08 (17.76 to 34.87)	11.8 (8.83 to 15.3)	30.61 (22.87 to 39.71)
Sichuan	Gallbladder and biliary tract cancer	21.8 (17.3 to 39)	19.52 (15.47 to 34.95)	33.1 (26.2 to 43.8)	39.63 (31.37 to 52.51)
Tianjin	Gallbladder and biliary tract cancer	3.15 (2.29 to 3.71)	34.24 (24.89 to 40.31)	8.62 (3.88 to 11.3)	56.46 (25.44 to 73.92)
Tibet	Gallbladder and biliary tract cancer	0.458 (0.296 to 1.35)	19.65 (12.72 to 57.79)	0.592 (0.382 to 1.46)	17.13 (11.06 to 42.3)
Xinjiang	Gallbladder and biliary tract cancer	3.17 (2.66 to 4.63)	20.11 (16.84 to 29.36)	7.56 (6.03 to 9.62)	31.03 (24.74 to 39.46)

Yunnan	Gallbladder and biliary tract cancer	7.95 (5.84 to 15.9)	20.39 (14.98 to 40.83)	14.8 (11.8 to 21.8)	30.09 (23.92 to 44.41)
Zhejiang	Gallbladder and biliary tract cancer	15.7 (10.4 to 18.7)	35.66 (23.69 to 42.42)	37.9 (16.2 to 49.3)	61.89 (26.55 to 80.49)
China	Pancreatic cancer	674 (644 to 710)	56.32 (53.81 to 59.29)	1890 (1800 to 1970)	133.87 (127.52 to 139.79)
Taiwan	Pancreatic cancer	13.3 (12.9 to 13.7)	65.08 (63.07 to 67.12)	46.3 (43.4 to 49.6)	196.53 (183.91 to 210.35)
Hong Kong Special Administrative Region of China	Pancreatic cancer	6.39 (5.84 to 7.02)	110.78 (101.3 to 121.79)	11.8 (9.85 to 14)	157 (131.11 to 186.33)
Macau Special Administrative Region of China	Pancreatic cancer	0.176 (0.156 to 0.199)	49.59 (43.94 to 56.03)	0.527 (0.413 to 0.668)	77.08 (60.44 to 97.63)
Anhui	Pancreatic cancer	39.4 (34.2 to 45.4)	66.55 (57.69 to 76.64)	96.3 (80.9 to 114)	157.56 (132.28 to 186.06)
Beijing	Pancreatic cancer	11.7 (10.2 to 13.3)	103.97 (90.68 to 118.69)	29.7 (24.4 to 35.3)	123.22 (101.13 to 146.58)
Chongqing	Pancreatic cancer	7.77 (6.47 to 9.22)	49.24 (40.98 to 58.4)	30.6 (24.8 to 37.4)	114.53 (92.84 to 140.03)
Fujian	Pancreatic cancer	11 (9.9 to 12.3)	34.82 (31.29 to 38.98)	36.6 (30.7 to 43.7)	92.9 (77.92 to 110.93)
Gansu	Pancreatic cancer	12.4 (10.6 to 14.3)	52.38 (44.62 to 60.65)	28.3 (23.8 to 33.2)	106.35 (89.51 to 125.02)
Guangdong	Pancreatic cancer	27.3 (23.2 to 32.3)	41.47 (35.24 to 49.09)	89.9 (75.6 to 106)	76.96 (64.69 to 90.81)
Guangxi	Pancreatic cancer	13.4 (11.4 to 15.8)	30.82 (26.25 to 36.34)	43.3 (36.2 to 52)	89.55 (74.99 to 107.53)
Guizhou	Pancreatic cancer	13.2 (11.2 to 15.5)	39.64 (33.85 to 46.58)	30.1 (24.4 to 35.7)	88.98 (72.08 to 105.66)
Hainan	Pancreatic cancer	2.26 (1.73 to 2.83)	32.39 (24.83 to 40.6)	6.17 (4.92 to 7.74)	64.34 (51.27 to 80.68)
Hebei	Pancreatic cancer	27.1 (24 to 30.3)	42.52 (37.7 to 47.64)	94.8 (78.9 to 114)	124.02 (103.28 to 148.56)
Heilongjiang	Pancreatic cancer	31 (27.3 to 35.4)	85.07 (74.9 to 97.19)	90.7 (75.5 to 108)	231.09 (192.33 to 275.06)
Henan	Pancreatic cancer	26.9 (23.9 to 30.1)	30.48 (27.07 to 34.1)	93.5 (79.5 to 109)	97.11 (82.51 to 113.64)
Hubei	Pancreatic cancer	27.6 (24.7 to 30.8)	49.69 (44.49 to 55.43)	70.6 (59.2 to 83.3)	127.67 (107.03 to 150.63)
Hunan	Pancreatic cancer	28.8 (25.7 to 32.5)	46.35 (41.37 to 52.33)	73.9 (62 to 86.9)	110 (92.39 to 129.37)
Inner Mongolia	Pancreatic cancer	15.6 (13.3 to 18)	68.63 (58.56 to 79.27)	44.8 (37.2 to 53.3)	172.92 (143.84 to 205.68)
Jiangsu	Pancreatic cancer	70.6 (63.6 to 78.6)	102.56 (92.43 to 114.16)	201 (168 to 238)	246.75 (205.74 to 291.97)
Jiangxi	Pancreatic cancer	21.5 (18.7 to 24.8)	54.33 (47.19 to 62.66)	43.3 (36.8 to 50.8)	91.14 (77.39 to 106.83)
Jilin	Pancreatic cancer	19.3 (16.6 to 22.6)	74.2 (63.92 to 87.22)	49 (39.8 to 57.8)	175.66 (142.8 to 207.4)
Liaoning	Pancreatic cancer	38.1 (32.4 to 45.1)	93.5 (79.33 to 110.48)	116 (95.3 to 136)	259.25 (213.33 to 304.83)
Ningxia	Pancreatic cancer	2.37 (2.06 to 2.74)	48.03 (41.63 to 55.45)	7.41 (6.06 to 9)	105.62 (86.38 to 128.27)
Qinghai	Pancreatic cancer	2.41 (2.08 to 2.8)	50.82 (43.86 to 59.08)	7.27 (5.85 to 8.65)	115.7 (93.18 to 137.75)
Shaanxi	Pancreatic cancer	11.3 (9.57 to 12.9)	32.76 (27.81 to 37.4)	43.6 (35.2 to 53.1)	110.37 (89.2 to 134.44)
Shandong	Pancreatic cancer	51.6 (44.7 to 59.5)	60.26 (52.2 to 69.54)	133 (113 to 159)	134.14 (113.44 to 160.23)
Shanghai	Pancreatic cancer	21.7 (18.7 to 24.9)	154.99 (133.78 to 178.34)	60.7 (50.2 to 73.3)	217.13 (179.64 to 262.31)
Shanxi	Pancreatic cancer	14.9 (12.9 to 17.2)	49.19 (42.64 to 56.83)	36.8 (30 to 45.1)	95.27 (77.67 to 116.74)
Sichuan	Pancreatic cancer	46 (40.8 to 51.8)	41.21 (36.55 to 46.38)	122 (100 to 144)	145.64 (119.81 to 172.44)
Tianjin	Pancreatic cancer	9.33 (8.1 to 10.7)	101.45 (88.03 to 116.17)	28.1 (23.6 to 33.7)	183.96 (154.69 to 220.95)

Tibet	Pancreatic cancer	0.883 (0.745 to 1.02)	37.91 (31.97 to 43.9)	1.91 (1.62 to 2.28)	55.37 (46.73 to 65.91)
Xinjiang	Pancreatic cancer	5.83 (5.21 to 6.48)	36.97 (33.02 to 41.09)	22.5 (18.7 to 26.7)	92.3 (76.76 to 109.54)
Yunnan	Pancreatic cancer	14 (12 to 16.7)	35.98 (30.69 to 42.78)	42.9 (36.4 to 51.2)	87.15 (74.06 to 104.2)
Zhejiang	Pancreatic cancer	42.5 (37.3 to 49.3)	96.29 (84.53 to 111.86)	104 (85.9 to 124)	169.52 (140.33 to 203.34)
China	Malignant skin melanoma	96.9 (64.3 to 129)	8.1 (5.37 to 10.75)	144 (94.9 to 164)	10.2 (6.72 to 11.64)
Taiwan	Malignant skin melanoma	1.67 (1.34 to 2.28)	8.21 (6.56 to 11.19)	4.04 (2.7 to 5.19)	17.13 (11.46 to 22.01)
Hong Kong Special Administrative Region of China	Malignant skin melanoma	0.428 (0.318 to 0.612)	7.42 (5.53 to 10.61)	0.803 (0.413 to 1.38)	10.69 (5.5 to 18.39)
Macau Special Administrative Region of China	Malignant skin melanoma	0.0243 (0.0193 to 0.0355)	6.83 (5.43 to 10)	0.0552 (0.0248 to 0.0995)	8.08 (3.62 to 14.55)
Anhui	Malignant skin melanoma	5.57 (4.3 to 7.93)	9.4 (7.26 to 13.39)	6.08 (4.01 to 7.56)	9.94 (6.57 to 12.37)
Beijing	Malignant skin melanoma	0.886 (0.701 to 1.18)	7.9 (6.25 to 10.56)	1.67 (0.826 to 2.77)	6.91 (3.43 to 11.48)
Chongqing	Malignant skin melanoma	1.06 (0.755 to 2.1)	6.69 (4.78 to 13.32)	1.84 (1.27 to 3.18)	6.87 (4.75 to 11.88)
Fujian	Malignant skin melanoma	1.88 (1.5 to 2.85)	5.96 (4.73 to 9.02)	3.1 (2.19 to 4.64)	7.86 (5.56 to 11.77)
Gansu	Malignant skin melanoma	2.59 (1.26 to 3.67)	10.96 (5.34 to 15.54)	2.95 (1.75 to 3.84)	11.09 (6.59 to 14.44)
Guangdong	Malignant skin melanoma	4.75 (3.24 to 6.54)	7.22 (4.92 to 9.93)	9.1 (5.77 to 13.2)	7.79 (4.94 to 11.3)
Guangxi	Malignant skin melanoma	2.95 (2.14 to 5.23)	6.77 (4.93 to 12.03)	4.27 (3.34 to 5.78)	8.84 (6.92 to 11.96)
Guizhou	Malignant skin melanoma	4.5 (1.6 to 6.53)	13.56 (4.83 to 19.67)	4.8 (2.38 to 7.02)	14.19 (7.05 to 20.76)
Hainan	Malignant skin melanoma	0.28 (0.155 to 0.676)	4.02 (2.23 to 9.7)	0.498 (0.289 to 0.957)	5.19 (3.01 to 9.98)
Hebei	Malignant skin melanoma	5.95 (3.4 to 7.48)	9.34 (5.34 to 11.75)	11.9 (4.94 to 17.2)	15.58 (6.46 to 22.57)
Heilongjiang	Malignant skin melanoma	2.41 (1.77 to 4.21)	6.62 (4.87 to 11.56)	3.58 (2.71 to 5.31)	9.11 (6.91 to 13.53)
Henan	Malignant skin melanoma	4.81 (3.83 to 7.62)	5.45 (4.34 to 8.63)	8.44 (6.19 to 10.8)	8.76 (6.43 to 11.2)
Hubei	Malignant skin melanoma	3.38 (2.65 to 5.27)	6.08 (4.77 to 9.49)	5.03 (3.88 to 7.21)	9.09 (7.01 to 13.04)
Hunan	Malignant skin melanoma	7.18 (3.4 to 9.91)	11.54 (5.47 to 15.93)	9.77 (5.14 to 13.4)	14.54 (7.66 to 19.91)
Inner Mongolia	Malignant skin melanoma	2.14 (1.15 to 2.89)	9.41 (5.05 to 12.7)	3.14 (1.77 to 4.08)	12.12 (6.84 to 15.77)
Jiangsu	Malignant skin melanoma	5.54 (4.17 to 7.19)	8.06 (6.05 to 10.44)	8.3 (4.96 to 11)	10.16 (6.07 to 13.5)
Jiangxi	Malignant skin melanoma	3.61 (1.89 to 5.88)	9.14 (4.78 to 14.88)	4.1 (2.94 to 5.14)	8.61 (6.18 to 10.81)
Jilin	Malignant skin melanoma	3.7 (1.38 to 5.87)	14.26 (5.34 to 22.61)	5.33 (1.89 to 7.9)	19.13 (6.78 to 28.33)
Liaoning	Malignant skin melanoma	3.86 (2.5 to 5.28)	9.45 (6.14 to 12.94)	6.06 (3.44 to 7.85)	13.58 (7.7 to 17.58)
Ningxia	Malignant skin melanoma	0.281 (0.203 to 0.445)	5.68 (4.11 to 9.01)	0.478 (0.359 to 0.689)	6.81 (5.12 to 9.82)
Qinghai	Malignant skin melanoma	0.384 (0.201 to 0.66)	8.12 (4.24 to 13.94)	0.641 (0.401 to 0.829)	10.2 (6.39 to 13.2)
Shaanxi	Malignant skin melanoma	1.9 (1.44 to 2.55)	5.53 (4.2 to 7.41)	3.94 (2.39 to 5.08)	9.96 (6.04 to 12.85)
Shandong	Malignant skin melanoma	6.56 (4.77 to 10)	7.67 (5.57 to 11.69)	9.2 (6.55 to 12.4)	9.26 (6.59 to 12.51)
Shanghai	Malignant skin melanoma	1.31 (0.887 to 1.63)	9.41 (6.35 to 11.64)	2.36 (0.989 to 3.27)	8.46 (3.54 to 11.7)
Shanxi	Malignant skin melanoma	2.51 (1.57 to 3.47)	8.28 (5.19 to 11.46)	3.45 (2.24 to 4.47)	8.92 (5.81 to 11.57)

Sichuan	Malignant skin melanoma	7.14 (5.28 to 11.3)	6.4 (4.73 to 10.14)	8.37 (6.02 to 10.7)	10.02 (7.21 to 12.87)
Tianjin	Malignant skin melanoma	0.626 (0.477 to 0.99)	6.81 (5.19 to 10.76)	1.16 (0.821 to 1.91)	7.58 (5.38 to 12.5)
Tibet	Malignant skin melanoma	0.216 (0.102 to 0.535)	9.28 (4.36 to 22.96)	0.279 (0.195 to 0.386)	8.07 (5.65 to 11.16)
Xinjiang	Malignant skin melanoma	1.1 (0.729 to 1.57)	6.97 (4.62 to 9.98)	2.36 (1.58 to 3.1)	9.69 (6.5 to 12.72)
Yunnan	Malignant skin melanoma	3.89 (1.83 to 5.78)	9.98 (4.69 to 14.84)	5.87 (3.41 to 7.76)	11.94 (6.93 to 15.78)
Zhejiang	Malignant skin melanoma	3.5 (2.25 to 4.7)	7.95 (5.1 to 10.66)	5.12 (2.96 to 7.65)	8.38 (4.83 to 12.51)
China	Non-melanoma skin cancer	143 (135 to 152)	11.94 (11.28 to 12.72)	327 (308 to 343)	23.12 (21.77 to 24.3)
Taiwan	Non-melanoma skin cancer	3.22 (3.07 to 3.38)	15.78 (15.05 to 16.59)	3.26 (3.02 to 3.52)	13.84 (12.82 to 14.91)
Hong Kong Special Administrative Region of China	Non-melanoma skin cancer	0.454 (0.403 to 0.512)	7.87 (6.98 to 8.89)	1.29 (1.07 to 1.53)	17.12 (14.3 to 20.4)
Macau Special Administrative Region of China	Non-melanoma skin cancer	0.0334 (0.0301 to 0.0382)	9.42 (8.48 to 10.75)	0.0743 (0.0518 to 0.091)	10.86 (7.58 to 13.31)
Anhui	Non-melanoma skin cancer	7.08 (6 to 8.28)	11.96 (10.12 to 13.97)	14.3 (12.1 to 16.6)	23.35 (19.73 to 27.14)
Beijing	Non-melanoma skin cancer	0.559 (0.474 to 0.653)	4.98 (4.23 to 5.82)	1.65 (1.34 to 2.02)	6.87 (5.56 to 8.37)
Chongqing	Non-melanoma skin cancer	1.56 (1.32 to 1.85)	9.88 (8.37 to 11.71)	5.26 (4.29 to 6.36)	19.68 (16.06 to 23.8)
Fujian	Non-melanoma skin cancer	2.53 (2.22 to 2.87)	8 (7.02 to 9.08)	6.14 (5.1 to 7.36)	15.57 (12.94 to 18.67)
Gansu	Non-melanoma skin cancer	3.03 (2.67 to 3.51)	12.82 (11.29 to 14.85)	6.69 (5.54 to 7.84)	25.17 (20.84 to 29.5)
Guangdong	Non-melanoma skin cancer	5.07 (4.3 to 5.98)	7.7 (6.53 to 9.08)	13.5 (11.3 to 16)	11.55 (9.64 to 13.67)
Guangxi	Non-melanoma skin cancer	4.11 (3.5 to 4.87)	9.46 (8.05 to 11.21)	9.68 (8.09 to 11.5)	20.03 (16.74 to 23.84)
Guizhou	Non-melanoma skin cancer	9.94 (8.43 to 11.7)	29.94 (25.41 to 35.1)	17.5 (14.2 to 21.1)	51.85 (42.04 to 62.33)
Hainan	Non-melanoma skin cancer	0.554 (0.445 to 0.708)	7.95 (6.39 to 10.17)	1.13 (0.921 to 1.42)	11.84 (9.6 to 14.79)
Hebei	Non-melanoma skin cancer	11.2 (9.77 to 12.5)	17.55 (15.35 to 19.62)	27.8 (22.8 to 33.8)	36.42 (29.87 to 44.22)
Heilongjiang	Non-melanoma skin cancer	2.47 (2.12 to 2.97)	6.79 (5.82 to 8.13)	5.32 (4.45 to 6.39)	13.55 (11.33 to 16.28)
Henan	Non-melanoma skin cancer	8.01 (7.13 to 8.94)	9.08 (8.07 to 10.13)	23.2 (19.6 to 27.5)	24.07 (20.36 to 28.56)
Hubei	Non-melanoma skin cancer	4.34 (3.85 to 4.82)	7.8 (6.93 to 8.68)	11.5 (9.63 to 13.6)	20.81 (17.41 to 24.64)
Hunan	Non-melanoma skin cancer	14.5 (12.5 to 16.7)	23.29 (20.17 to 26.83)	30.6 (25.4 to 36.1)	45.55 (37.8 to 53.76)
Inner Mongolia	Non-melanoma skin cancer	2.97 (2.56 to 3.44)	13.07 (11.25 to 15.13)	7.73 (6.35 to 9.25)	29.85 (24.51 to 35.72)
Jiangsu	Non-melanoma skin cancer	6.41 (5.68 to 7.14)	9.32 (8.26 to 10.37)	14.3 (12.1 to 16.9)	17.49 (14.76 to 20.74)
Jiangxi	Non-melanoma skin cancer	5.56 (4.88 to 6.44)	14.06 (12.35 to 16.3)	10.1 (8.58 to 11.8)	21.22 (18.06 to 24.8)
Jilin	Non-melanoma skin cancer	5.61 (4.8 to 6.63)	21.62 (18.52 to 25.54)	12.3 (9.83 to 14.9)	44.1 (35.26 to 53.58)
Liaoning	Non-melanoma skin cancer	4.43 (3.77 to 5.29)	10.86 (9.25 to 12.96)	11.3 (9.38 to 13.3)	25.23 (21.02 to 29.87)
Ningxia	Non-melanoma skin cancer	0.289 (0.246 to 0.335)	5.86 (4.99 to 6.79)	0.78 (0.642 to 0.945)	11.11 (9.15 to 13.47)
Qinghai	Non-melanoma skin cancer	0.5 (0.434 to 0.579)	10.56 (9.16 to 12.23)	1.38 (1.13 to 1.65)	21.97 (18.01 to 26.23)
Shaanxi	Non-melanoma skin cancer	2.07 (1.79 to 2.34)	6 (5.21 to 6.8)	7.81 (6.38 to 9.44)	19.78 (16.15 to 23.88)
Shandong	Non-melanoma skin cancer	10.1 (8.76 to 11.6)	11.78 (10.23 to 13.52)	19.5 (16.5 to 23.1)	19.66 (16.6 to 23.2)

Shanghai	Non-melanoma skin cancer	1.09<br>(0.929 to 1.28)	7.82<br>(6.65 to 9.16)	2.67<br>(2.19 to 3.25)	9.54<br>(7.85 to 11.61)
Shanxi	Non-melanoma skin cancer	3.48<br>(3.04 to 3.99)	11.5<br>(10.03 to 13.17)	7.76<br>(6.25 to 9.48)	20.1<br>(16.19 to 24.55)
Sichuan	Non-melanoma skin cancer	12.3<br>(10.9 to 13.7)	11.05<br>(9.79 to 12.31)	26.5<br>(22.1 to 31.1)	31.77<br>(26.46 to 37.21)
Tianjin	Non-melanoma skin cancer	0.487<br>(0.413 to 0.559)	5.29<br>(4.49 to 6.08)	1.28<br>(1.06 to 1.54)	8.39<br>(6.94 to 10.1)
Tibet	Non-melanoma skin cancer	0.247<br>(0.209 to 0.289)	10.61<br>(8.98 to 12.39)	0.453<br>(0.375 to 0.538)	13.09<br>(10.85 to 15.57)
Xinjiang	Non-melanoma skin cancer	1.09<br>(0.959 to 1.22)	6.92<br>(6.08 to 7.73)	2.76<br>(2.21 to 3.31)	11.34<br>(9.05 to 13.6)
Yunnan	Non-melanoma skin cancer	6.46<br>(5.41 to 7.68)	16.57<br>(13.87 to 19.7)	15.6<br>(13.1 to 18.5)	31.76<br>(26.68 to 37.66)
Zhejiang	Non-melanoma skin cancer	4.41<br>(3.83 to 5.07)	10<br>(8.68 to 11.49)	8.63<br>(7.18 to 10.3)	14.11<br>(11.74 to 16.82)
China	Ovarian cancer	263<br>(242 to 301)	21.94<br>(20.18 to 25.11)	693<br>(650 to 735)	49.05<br>(46.01 to 52.01)
Taiwan	Ovarian cancer	5.32<br>(5.04 to 5.6)	26.07<br>(24.73 to 27.46)	18<br>(16.2 to 19.8)	76.14<br>(68.49 to 83.94)
Hong Kong Special Administrative Region of China	Ovarian cancer	2.45<br>(2.15 to 2.78)	42.47<br>(37.34 to 48.2)	4.87<br>(3.67 to 6.34)	64.9<br>(48.91 to 84.48)
Macau Special Administrative Region of China	Ovarian cancer	0.0689<br>(0.058 to 0.0824)	19.42<br>(16.33 to 23.21)	0.197<br>(0.142 to 0.254)	28.75<br>(20.76 to 37.08)
Anhui	Ovarian cancer	11.1<br>(9.22 to 13.4)	18.77<br>(15.57 to 22.67)	31.4<br>(24.6 to 38.8)	51.31<br>(40.17 to 63.38)
Beijing	Ovarian cancer	5.79<br>(4.68 to 7.11)	51.66<br>(41.7 to 63.38)	12.5<br>(9.49 to 16.1)	51.67<br>(39.36 to 66.65)
Chongqing	Ovarian cancer	2.89<br>(2.17 to 3.92)	18.28<br>(13.77 to 24.86)	12.8<br>(9.74 to 16.7)	47.85<br>(36.45 to 62.32)
Fujian	Ovarian cancer	6.51<br>(5.33 to 8.09)	20.56<br>(16.83 to 25.57)	17.5<br>(13.3 to 22.6)	44.4<br>(33.64 to 57.28)
Gansu	Ovarian cancer	3.97<br>(3.26 to 4.81)	16.8<br>(13.77 to 20.32)	12.2<br>(9.41 to 15.4)	45.73<br>(35.4 to 57.96)
Guangdong	Ovarian cancer	16.7<br>(13.2 to 21.3)	25.31<br>(20.04 to 32.42)	49<br>(37.5 to 62.2)	41.9<br>(32.06 to 53.24)
Guangxi	Ovarian cancer	8.09<br>(6.35 to 10.3)	18.6<br>(14.59 to 23.7)	21.7<br>(16.5 to 27.7)	44.86<br>(34.23 to 57.39)
Guizhou	Ovarian cancer	5.64<br>(4.39 to 7.39)	16.99<br>(13.22 to 22.26)	12.1<br>(9.08 to 15.8)	35.85<br>(26.85 to 46.64)
Hainan	Ovarian cancer	1.13<br>(0.78 to 1.79)	16.24<br>(11.2 to 25.68)	2.82<br>(2.03 to 3.77)	29.39<br>(21.18 to 39.28)
Hebei	Ovarian cancer	8.17<br>(6.86 to 9.63)	12.84<br>(10.78 to 15.13)	28.9<br>(22.4 to 37.1)	37.76<br>(29.35 to 48.54)
Heilongjiang	Ovarian cancer	12<br>(9.6 to 15.7)	32.96<br>(26.35 to 42.98)	25.3<br>(19.6 to 32.3)	64.51<br>(50.02 to 82.35)
Henan	Ovarian cancer	10.5<br>(8.99 to 12.2)	11.89<br>(10.18 to 13.81)	46.1<br>(35.8 to 57.5)	47.88<br>(37.13 to 59.71)
Hubei	Ovarian cancer	9.72<br>(8.33 to 11.2)	17.5<br>(14.99 to 20.2)	30.8<br>(24 to 38.6)	55.74<br>(43.37 to 69.77)
Hunan	Ovarian cancer	16.1<br>(13.3 to 20.3)	25.88<br>(21.42 to 32.7)	30.6<br>(23.9 to 38.1)	45.55<br>(35.61 to 56.71)
Inner Mongolia	Ovarian cancer	6.66<br>(5.28 to 8.4)	29.28<br>(23.2 to 36.93)	14.2<br>(11.1 to 18.1)	54.87<br>(42.7 to 69.79)
Jiangsu	Ovarian cancer	16.6<br>(14 to 19.4)	24.08<br>(20.41 to 28.13)	49.6<br>(37.9 to 63.6)	60.69<br>(46.45 to 77.85)
Jiangxi	Ovarian cancer	10.6<br>(8.58 to 13.7)	26.78<br>(21.71 to 34.56)	19.8<br>(15.9 to 24.9)	41.67<br>(33.46 to 52.37)
Jilin	Ovarian cancer	6.91<br>(5.59 to 8.8)	26.62<br>(21.52 to 33.91)	15.9<br>(11.8 to 20.9)	56.86<br>(42.22 to 74.97)
Liaoning	Ovarian cancer	14.7<br>(11.3 to 19)	36.06<br>(27.67 to 46.51)	35.9<br>(27 to 46.7)	80.4<br>(60.45 to 104.56)
Ningxia	Ovarian cancer	0.871<br>(0.661 to 1.12)	17.64<br>(13.38 to 22.77)	2.68<br>(1.95 to 3.56)	38.24<br>(27.84 to 50.68)
Qinghai	Ovarian cancer	0.925<br>(0.709 to 1.23)	19.53<br>(14.98 to 25.88)	2.51<br>(1.87 to 3.28)	39.97<br>(29.83 to 52.29)

Shaanxi	Ovarian cancer	4.27 (3.46 to 5.09)	12.42 (10.06 to 14.81)	17.9 (13.1 to 23.5)	45.33 (33.13 to 59.41)
Shandong	Ovarian cancer	19.7 (15.9 to 24.5)	23.02 (18.55 to 28.57)	55.1 (42.8 to 69.9)	55.44 (43.04 to 70.3)
Shanghai	Ovarian cancer	7.11 (5.75 to 8.71)	50.86 (41.17 to 62.35)	14 (10.3 to 18.4)	50.17 (36.85 to 65.82)
Shanxi	Ovarian cancer	6.12 (4.87 to 7.61)	20.21 (16.09 to 25.14)	16.2 (12.1 to 21.8)	41.84 (31.26 to 56.45)
Sichuan	Ovarian cancer	16.6 (13.9 to 20.1)	14.85 (12.49 to 18.04)	41.8 (31.2 to 54.5)	50.1 (37.41 to 65.3)
Tianjin	Ovarian cancer	3.96 (3.14 to 4.92)	43.06 (34.1 to 53.51)	8.72 (6.64 to 11.3)	57.13 (43.5 to 73.95)
Tibet	Ovarian cancer	0.416 (0.33 to 0.516)	17.84 (14.16 to 22.13)	1.07 (0.825 to 1.36)	30.83 (23.86 to 39.24)
Xinjiang	Ovarian cancer	3 (2.5 to 3.56)	19.03 (15.87 to 22.58)	9.32 (6.92 to 12.2)	38.23 (28.4 to 49.88)
Yunnan	Ovarian cancer	6.62 (5.1 to 8.91)	16.99 (13.08 to 22.87)	20.6 (16.1 to 25.8)	41.86 (32.68 to 52.55)
Zhejiang	Ovarian cancer	16.8 (13.7 to 21.7)	38.12 (31.06 to 49.11)	29 (21.9 to 37)	47.35 (35.84 to 60.53)
China	Testicular cancer	38.6 (33 to 48.9)	3.22 (2.75 to 4.09)	21.9 (19.7 to 24.1)	1.55 (1.4 to 1.71)
Taiwan	Testicular cancer	0.411 (0.36 to 0.468)	2.01 (1.77 to 2.29)	0.725 (0.611 to 0.862)	3.08 (2.59 to 3.66)
Hong Kong Special Administrative Region of China	Testicular cancer	0.16 (0.13 to 0.192)	2.77 (2.26 to 3.34)	0.101 (0.075 to 0.132)	1.35 (1 to 1.76)
Macau Special Administrative Region of China	Testicular cancer	0.0099 (0.00795 to 0.012)	2.79 (2.24 to 3.39)	0.0103 (0.00747 to 0.015)	1.51 (1.09 to 2.19)
Anhui	Testicular cancer	1.99 (1.45 to 2.82)	3.36 (2.44 to 4.76)	0.98 (0.744 to 1.24)	1.6 (1.22 to 2.03)
Beijing	Testicular cancer	0.321 (0.245 to 0.425)	2.86 (2.18 to 3.78)	0.224 (0.165 to 0.294)	0.93 (0.69 to 1.22)
Chongqing	Testicular cancer	0.713 (0.523 to 1)	4.52 (3.31 to 6.34)	0.401 (0.3 to 0.536)	1.5 (1.12 to 2.01)
Fujian	Testicular cancer	0.786 (0.601 to 1.08)	2.48 (1.9 to 3.41)	0.637 (0.481 to 0.85)	1.62 (1.22 to 2.16)
Gansu	Testicular cancer	1.19 (0.877 to 1.6)	5.03 (3.71 to 6.76)	0.379 (0.285 to 0.488)	1.43 (1.07 to 1.84)
Guangdong	Testicular cancer	2.36 (1.74 to 3.22)	3.59 (2.65 to 4.89)	1.68 (1.24 to 2.21)	1.44 (1.06 to 1.89)
Guangxi	Testicular cancer	1.78 (1.34 to 2.49)	4.1 (3.08 to 5.74)	0.939 (0.711 to 1.24)	1.94 (1.47 to 2.58)
Guizhou	Testicular cancer	1.15 (0.849 to 1.64)	3.47 (2.56 to 4.94)	0.458 (0.335 to 0.612)	1.36 (0.99 to 1.81)
Hainan	Testicular cancer	0.252 (0.142 to 0.39)	3.62 (2.03 to 5.6)	0.144 (0.106 to 0.196)	1.5 (1.1 to 2.04)
Hebei	Testicular cancer	1.56 (1.26 to 1.95)	2.46 (1.98 to 3.07)	1.35 (0.928 to 1.82)	1.76 (1.21 to 2.38)
Heilongjiang	Testicular cancer	1.44 (1.03 to 2.01)	3.96 (2.83 to 5.52)	0.665 (0.51 to 0.856)	1.69 (1.3 to 2.18)
Henan	Testicular cancer	1.24 (0.959 to 1.62)	1.41 (1.09 to 1.84)	1.02 (0.773 to 1.31)	1.06 (0.8 to 1.36)
Hubei	Testicular cancer	1.84 (1.42 to 2.54)	3.31 (2.56 to 4.58)	1.05 (0.823 to 1.37)	1.91 (1.49 to 2.48)
Hunan	Testicular cancer	1.83 (1.42 to 2.5)	2.95 (2.29 to 4.02)	1.22 (0.925 to 1.6)	1.82 (1.38 to 2.39)
Inner Mongolia	Testicular cancer	0.954 (0.704 to 1.3)	4.2 (3.1 to 5.73)	0.455 (0.346 to 0.595)	1.76 (1.34 to 2.3)
Jiangsu	Testicular cancer	2.06 (1.61 to 2.68)	3 (2.35 to 3.9)	1.22 (0.924 to 1.58)	1.49 (1.13 to 1.94)
Jiangxi	Testicular cancer	1.12 (0.856 to 1.56)	2.84 (2.16 to 3.94)	0.471 (0.367 to 0.592)	0.99 (0.77 to 1.24)
Jilin	Testicular cancer	0.881 (0.612 to 1.28)	3.39 (2.36 to 4.92)	0.333 (0.244 to 0.438)	1.19 (0.87 to 1.57)
Liaoning	Testicular cancer	1.97 (1.34 to 2.84)	4.84 (3.29 to 6.97)	0.984 (0.732 to 1.27)	2.2 (1.64 to 2.84)

Ningxia	Testicular cancer	0.0972<br>(0.0695 to 0.138)	1.97<br>(1.41 to 2.8)	0.0616<br>(0.0466 to 0.0795)	0.88<br>(0.66 to 1.13)
Qinghai	Testicular cancer	0.163<br>(0.12 to 0.223)	3.44<br>(2.53 to 4.71)	0.0943<br>(0.0712 to 0.126)	1.5<br>(1.13 to 2)
Shaanxi	Testicular cancer	0.666<br>(0.51 to 0.921)	1.93<br>(1.48 to 2.68)	0.557<br>(0.419 to 0.729)	1.41<br>(1.06 to 1.84)
Shandong	Testicular cancer	2.24<br>(1.66 to 3.05)	2.61<br>(1.93 to 3.56)	1.11<br>(0.818 to 1.44)	1.11<br>(0.82 to 1.45)
Shanghai	Testicular cancer	0.717<br>(0.56 to 0.907)	5.13<br>(4.01 to 6.49)	0.508<br>(0.372 to 0.69)	1.82<br>(1.33 to 2.47)
Shanxi	Testicular cancer	0.889<br>(0.653 to 1.24)	2.94<br>(2.15 to 4.1)	0.403<br>(0.301 to 0.524)	1.04<br>(0.78 to 1.36)
Sichuan	Testicular cancer	4.3<br>(3.27 to 5.89)	3.85<br>(2.93 to 5.28)	1.75<br>(1.31 to 2.29)	2.1<br>(1.57 to 2.74)
Tianjin	Testicular cancer	0.366<br>(0.282 to 0.475)	3.98<br>(3.07 to 5.16)	0.345<br>(0.256 to 0.447)	2.26<br>(1.67 to 2.93)
Tibet	Testicular cancer	0.119<br>(0.087 to 0.166)	5.11<br>(3.73 to 7.13)	0.0523<br>(0.0391 to 0.0674)	1.51<br>(1.13 to 1.95)
Xinjiang	Testicular cancer	0.534<br>(0.401 to 0.7)	3.38<br>(2.54 to 4.44)	0.509<br>(0.353 to 0.698)	2.09<br>(1.45 to 2.86)
Yunnan	Testicular cancer	1.57<br>(1.1 to 2.43)	4.03<br>(2.83 to 6.24)	0.853<br>(0.651 to 1.12)	1.73<br>(1.32 to 2.27)
Zhejiang	Testicular cancer	1.26<br>(0.974 to 1.69)	2.86<br>(2.21 to 3.82)	0.884<br>(0.645 to 1.14)	1.44<br>(1.05 to 1.87)
China	Kidney cancer	218<br>(197 to 263)	18.2<br>(16.43 to 21.93)	438<br>(390 to 474)	31.02<br>(27.61 to 33.53)
Taiwan	Kidney cancer	3.44<br>(3.22 to 4.13)	16.88<br>(15.81 to 20.26)	17.4<br>(14 to 18.9)	73.75<br>(59.33 to 80.13)
Hong Kong Special Administrative Region of China	Kidney cancer	1.33<br>(1.15 to 1.51)	23.12<br>(19.96 to 26.26)	2.59<br>(2.08 to 3.28)	34.48<br>(27.72 to 43.72)
Macau Special Administrative Region of China	Kidney cancer	0.0438<br>(0.0368 to 0.0769)	12.34<br>(10.36 to 21.66)	0.12<br>(0.0917 to 0.214)	17.53<br>(13.4 to 31.3)
Anhui	Kidney cancer	14.1<br>(11.3 to 17.3)	23.74<br>(19.12 to 29.14)	18.5<br>(14.9 to 21.9)	30.28<br>(24.42 to 35.88)
Beijing	Kidney cancer	2.99<br>(2.56 to 3.47)	26.7<br>(22.79 to 30.92)	10.1<br>(6.29 to 12.4)	41.88<br>(26.1 to 51.24)
Chongqing	Kidney cancer	2.47<br>(2 to 3.56)	15.62<br>(12.67 to 22.57)	6.23<br>(4.97 to 8.36)	23.31<br>(18.61 to 31.28)
Fujian	Kidney cancer	4.82<br>(4.17 to 5.64)	15.25<br>(13.19 to 17.82)	11.4<br>(8.93 to 14)	28.95<br>(22.66 to 35.48)
Gansu	Kidney cancer	5.56<br>(4.51 to 7.12)	23.52<br>(19.08 to 30.1)	8.01<br>(6.32 to 9.55)	30.13<br>(23.78 to 35.94)
Guangdong	Kidney cancer	10.3<br>(8.58 to 12.7)	15.71<br>(13.04 to 19.31)	27.6<br>(22.2 to 34)	23.66<br>(18.99 to 29.09)
Guangxi	Kidney cancer	6.44<br>(5.39 to 8.52)	14.8<br>(12.39 to 19.6)	13.4<br>(10.6 to 16.4)	27.73<br>(21.99 to 33.89)
Guizhou	Kidney cancer	6.49<br>(5.21 to 8.26)	19.56<br>(15.7 to 24.89)	8.56<br>(6.73 to 10.8)	25.32<br>(19.9 to 31.9)
Hainan	Kidney cancer	1.21<br>(0.875 to 1.65)	17.33<br>(12.57 to 23.71)	1.91<br>(1.46 to 2.45)	19.87<br>(15.25 to 25.54)
Hebei	Kidney cancer	9.46<br>(8.03 to 13.8)	14.86<br>(12.62 to 21.61)	21.5<br>(17.4 to 28.9)	28.13<br>(22.77 to 37.83)
Heilongjiang	Kidney cancer	8.75<br>(7.07 to 11)	24.01<br>(19.38 to 30.17)	17.4<br>(13.7 to 21)	44.45<br>(34.87 to 53.39)
Henan	Kidney cancer	12.9<br>(10.9 to 18)	14.65<br>(12.36 to 20.39)	25.4<br>(21 to 31)	26.42<br>(21.85 to 32.24)
Hubei	Kidney cancer	8.25<br>(6.99 to 11.6)	14.85<br>(12.59 to 20.82)	16.4<br>(13.5 to 20.8)	29.63<br>(24.33 to 37.54)
Hunan	Kidney cancer	9.72<br>(8.38 to 12.5)	15.63<br>(13.47 to 20.14)	22<br>(17.5 to 27.5)	32.79<br>(26.11 to 40.92)
Inner Mongolia	Kidney cancer	4.98<br>(4.05 to 6.07)	21.91<br>(17.8 to 26.7)	10.4<br>(7.97 to 12.7)	40.33<br>(30.78 to 48.9)
Jiangsu	Kidney cancer	10.5<br>(9.03 to 14.6)	15.24<br>(13.12 to 21.16)	25.4<br>(20.7 to 31.4)	31.06<br>(25.4 to 38.39)
Jiangxi	Kidney cancer	8.88<br>(7.21 to 11.3)	22.47<br>(18.23 to 28.51)	12.3<br>(10.1 to 14.5)	25.86<br>(21.19 to 30.46)

Jilin	Kidney cancer	6.31<br>(5.04 to 8.02)	24.31<br>(19.42 to 30.9)	11<br>(8.3 to 13.2)	39.29<br>(29.77 to 47.22)
Liaoning	Kidney cancer	10.4<br>(8.1 to 12.9)	25.37<br>(19.85 to 31.64)	26.8<br>(17.7 to 32.3)	59.92<br>(39.57 to 72.25)
Ningxia	Kidney cancer	1.37<br>(1.05 to 1.79)	27.84<br>(21.27 to 36.14)	1.79<br>(1.42 to 2.18)	25.46<br>(20.28 to 31.02)
Qinghai	Kidney cancer	1.37<br>(1.04 to 1.72)	28.92<br>(21.93 to 36.3)	2.36<br>(1.67 to 2.9)	37.6<br>(26.58 to 46.13)
Shaanxi	Kidney cancer	6.8<br>(5.45 to 8.18)	19.77<br>(15.83 to 23.77)	13.2<br>(9.42 to 16.1)	33.35<br>(23.84 to 40.81)
Shandong	Kidney cancer	14.1<br>(11.6 to 21.1)	16.46<br>(13.57 to 24.62)	31.9<br>(26.4 to 39.7)	32.1<br>(26.52 to 39.91)
Shanghai	Kidney cancer	3.41<br>(2.79 to 3.93)	24.37<br>(19.97 to 28.16)	12.6<br>(6.17 to 15.4)	44.96<br>(22.07 to 55.29)
Shanxi	Kidney cancer	5.27<br>(4.46 to 6.99)	17.39<br>(14.73 to 23.08)	10.2<br>(8.2 to 12.6)	26.43<br>(21.23 to 32.51)
Sichuan	Kidney cancer	17.5<br>(14.5 to 23.5)	15.64<br>(12.98 to 21.09)	21.9<br>(17.7 to 28)	26.23<br>(21.16 to 33.59)
Tianjin	Kidney cancer	2.43<br>(2.02 to 2.79)	26.43<br>(21.96 to 30.37)	9.24<br>(5.05 to 11.5)	60.51<br>(33.11 to 75.22)
Tibet	Kidney cancer	0.593<br>(0.416 to 0.784)	25.44<br>(17.86 to 33.62)	0.996<br>(0.671 to 1.24)	28.82<br>(19.4 to 35.87)
Xinjiang	Kidney cancer	3.72<br>(3 to 4.56)	23.58<br>(19.02 to 28.91)	6.4<br>(5.06 to 8.53)	26.27<br>(20.77 to 34.99)
Yunnan	Kidney cancer	7.13<br>(5.7 to 9.97)	18.3<br>(14.63 to 25.59)	11.5<br>(9.49 to 14.3)	23.41<br>(19.31 to 29.04)
Zhejiang	Kidney cancer	8.35<br>(7.12 to 9.89)	18.94<br>(16.15 to 22.44)	19<br>(14.3 to 23.1)	31.06<br>(23.41 to 37.7)
China	Bladder cancer	355<br>(292 to 382)	29.63<br>(24.4 to 31.89)	582<br>(548 to 672)	41.24<br>(38.76 to 47.61)
Taiwan	Bladder cancer	8.64<br>(8.31 to 8.99)	42.34<br>(40.75 to 44.06)	17.7<br>(16.4 to 19)	74.97<br>(69.74 to 80.6)
Hong Kong Special Administrative Region of China	Bladder cancer	3.36<br>(2.18 to 3.88)	58.35<br>(37.86 to 67.28)	3.64<br>(2.99 to 4.43)	48.41<br>(39.8 to 58.99)
Macau Special Administrative Region of China	Bladder cancer	0.114<br>(0.1 to 0.127)	32.24<br>(28.28 to 35.9)	0.228<br>(0.164 to 0.291)	33.31<br>(24 to 42.57)
Anhui	Bladder cancer	16.4<br>(12.3 to 20)	27.68<br>(20.8 to 33.68)	22.8<br>(18.9 to 27.9)	37.21<br>(30.87 to 45.62)
Beijing	Bladder cancer	5.72<br>(3.84 to 6.89)	51.03<br>(34.2 to 61.46)	9.58<br>(7.08 to 11.9)	39.74<br>(29.38 to 49.38)
Chongqing	Bladder cancer	4.31<br>(3.6 to 4.98)	27.34<br>(22.78 to 31.58)	11.7<br>(9.19 to 15.2)	43.69<br>(34.38 to 56.69)
Fujian	Bladder cancer	5.44<br>(4.74 to 6.17)	17.18<br>(14.99 to 19.49)	11.2<br>(8.84 to 14.9)	28.36<br>(22.43 to 37.75)
Gansu	Bladder cancer	6.79<br>(6 to 7.62)	28.69<br>(25.38 to 32.22)	9.63<br>(7.95 to 11.8)	36.22<br>(29.93 to 44.26)
Guangdong	Bladder cancer	18.7<br>(15.5 to 21.9)	28.35<br>(23.57 to 33.25)	32.6<br>(26.9 to 41)	27.94<br>(23 to 35.09)
Guangxi	Bladder cancer	9.2<br>(7.79 to 11.4)	21.15<br>(17.91 to 26.27)	15.4<br>(12 to 24.6)	31.97<br>(24.79 to 50.85)
Guizhou	Bladder cancer	10.8<br>(8.89 to 12.6)	32.48<br>(26.8 to 38.08)	15<br>(12.1 to 18.5)	44.24<br>(35.65 to 54.74)
Hainan	Bladder cancer	1.28<br>(1.09 to 1.56)	18.39<br>(15.62 to 22.34)	2.46<br>(1.87 to 3.38)	25.64<br>(19.53 to 35.21)
Hebei	Bladder cancer	16.1<br>(13.9 to 18.1)	25.29<br>(21.82 to 28.38)	33.8<br>(26.3 to 45.8)	44.19<br>(34.37 to 59.97)
Heilongjiang	Bladder cancer	12.8<br>(9.16 to 15.1)	35.07<br>(25.12 to 41.44)	22.7<br>(18.6 to 27.4)	57.93<br>(47.34 to 69.82)
Henan	Bladder cancer	15.6<br>(13.7 to 18.3)	17.72<br>(15.5 to 20.72)	30.3<br>(24.2 to 46.1)	31.47<br>(25.11 to 47.87)
Hubei	Bladder cancer	16.8<br>(13 to 19.5)	30.25<br>(23.37 to 35.17)	30.2<br>(24.7 to 36.4)	54.6<br>(44.71 to 65.86)
Hunan	Bladder cancer	18.3<br>(16.2 to 20.6)	29.38<br>(26.1 to 33.18)	30.1<br>(23.8 to 41.8)	44.79<br>(35.45 to 62.23)
Inner Mongolia	Bladder cancer	7.35<br>(5.49 to 8.74)	32.33<br>(24.13 to 38.42)	11.5<br>(9.49 to 13.8)	44.37<br>(36.66 to 53.4)

Jiangsu	Bladder cancer	23.8 (18.1 to 27.7)	34.66 (26.3 to 40.3)	40.3 (32.5 to 48.6)	49.29 (39.78 to 59.47)
Jiangxi	Bladder cancer	12.2 (10.8 to 13.7)	30.78 (27.39 to 34.76)	14.7 (12.2 to 18.6)	30.96 (25.67 to 39.15)
Jilin	Bladder cancer	10.5 (7.3 to 12.4)	40.42 (28.14 to 47.98)	15.5 (12.1 to 18.6)	55.66 (43.34 to 66.54)
Liaoning	Bladder cancer	19.6 (11.8 to 24.9)	48.17 (29.02 to 61.01)	34.4 (25.5 to 41.5)	76.95 (57.2 to 93.03)
Ningxia	Bladder cancer	1.15 (0.811 to 1.38)	23.19 (16.43 to 27.95)	2.08 (1.64 to 2.6)	29.61 (23.41 to 37.05)
Qinghai	Bladder cancer	1.01 (0.873 to 1.17)	21.41 (18.42 to 24.7)	1.84 (1.44 to 2.59)	29.33 (22.91 to 41.17)
Shaanxi	Bladder cancer	6.23 (4.6 to 7.52)	18.1 (13.38 to 21.85)	13.4 (10.5 to 18.3)	33.84 (26.69 to 46.19)
Shandong	Bladder cancer	31.8 (24.8 to 37.4)	37.18 (28.95 to 43.75)	46.3 (38.6 to 56.4)	46.56 (38.81 to 56.71)
Shanghai	Bladder cancer	7.49 (4.89 to 9.04)	53.63 (34.99 to 64.73)	11.4 (8.02 to 14.5)	40.87 (28.72 to 51.83)
Shanxi	Bladder cancer	9.34 (8.02 to 10.6)	30.84 (26.49 to 34.87)	12.3 (9.56 to 16.5)	31.87 (24.76 to 42.59)
Sichuan	Bladder cancer	27.5 (22.4 to 31.8)	24.66 (20.07 to 28.52)	41.3 (32.7 to 50.4)	49.43 (39.17 to 60.37)
Tianjin	Bladder cancer	4.61 (3.03 to 5.49)	50.1 (32.95 to 59.65)	8.22 (6.43 to 10.1)	53.88 (42.15 to 65.95)
Tibet	Bladder cancer	0.483 (0.398 to 0.702)	20.73 (17.08 to 30.14)	0.516 (0.393 to 0.941)	14.94 (11.36 to 27.23)
Xinjiang	Bladder cancer	2.97 (2.57 to 3.42)	18.8 (16.29 to 21.7)	6.69 (5.07 to 10.2)	27.46 (20.82 to 41.72)
Yunnan	Bladder cancer	11.3 (9.49 to 13.1)	28.92 (24.36 to 33.71)	17.8 (14.8 to 22.2)	36.17 (30.02 to 45.22)
Zhejiang	Bladder cancer	15.6 (12.2 to 18)	35.33 (27.56 to 40.86)	23.1 (18.5 to 28)	37.73 (30.25 to 45.8)
China	Brain and central nervous system cancer	1810 (1210 to 2450)	151.34 (101.47 to 204.58)	1960 (1660 to 2310)	138.7 (117.26 to 163.71)
Taiwan	Brain and central nervous system cancer	14 (11.8 to 15)	68.82 (57.78 to 73.66)	20.3 (16.3 to 22.5)	86.26 (69.04 to 95.35)
Hong Kong Special Administrative Region of China	Brain and central nervous system cancer	5.13 (3.94 to 8.13)	88.94 (68.4 to 141.12)	7.59 (5.03 to 15.5)	101.12 (66.99 to 205.92)
Macau Special Administrative Region of China	Brain and central nervous system cancer	0.291 (0.191 to 0.487)	82.01 (53.7 to 137.18)	0.483 (0.251 to 1.07)	70.67 (36.66 to 156.16)
Anhui	Brain and central nervous system cancer	113 (76.9 to 141)	191.43 (129.77 to 238.73)	94.9 (58.7 to 122)	155.2 (95.93 to 199.69)
Beijing	Brain and central nervous system cancer	14 (11.5 to 18.8)	124.63 (102.12 to 167.6)	23.4 (17.2 to 39.4)	97.28 (71.4 to 163.5)
Chongqing	Brain and central nervous system cancer	23.7 (13.6 to 33.6)	150.38 (86 to 213.16)	31.6 (24.4 to 41)	118.35 (91.19 to 153.5)
Fujian	Brain and central nervous system cancer	43.8 (28.5 to 54.7)	138.48 (90.22 to 173.04)	49.9 (37.7 to 62)	126.49 (95.58 to 157.27)
Gansu	Brain and central nervous system cancer	44.4 (27.1 to 64.4)	187.88 (114.41 to 272.45)	33.1 (26.9 to 41.3)	124.65 (101.4 to 155.24)
Guangdong	Brain and central nervous system cancer	82.7 (62.3 to 113)	125.58 (94.64 to 170.94)	128 (100 to 187)	109.94 (85.58 to 160.04)
Guangxi	Brain and central nervous system cancer	66.9 (41.2 to 97.5)	153.8 (94.82 to 224.26)	73 (58.5 to 94.7)	151.09 (121.02 to 195.94)
Guizhou	Brain and central nervous system cancer	53.4 (29.8 to 96.7)	160.78 (89.78 to 291.4)	39.1 (30.5 to 51.3)	115.63 (90.21 to 151.55)
Hainan	Brain and central nervous system cancer	9.2 (5.32 to 12.9)	132.11 (76.4 to 184.81)	11.2 (8.88 to 14)	116.46 (92.58 to 145.86)
Hebei	Brain and central nervous system cancer	78.3 (63.8 to 103)	123.01 (100.19 to 161.33)	114 (88 to 152)	149.59 (115.14 to 198.62)
Heilongjiang	Brain and central nervous system cancer	62.3 (42.8 to 78.7)	170.98 (117.46 to 215.97)	63 (51 to 76.9)	160.61 (129.83 to 196.02)
Henan	Brain and central nervous system cancer	117 (88 to 155)	132.24 (99.67 to 175.26)	139 (112 to 168)	144.15 (116.81 to 174.46)
Hubei	Brain and central nervous system cancer	80.6 (51.1 to 102)	145.02 (92.07 to 183.17)	91.1 (69.5 to 110)	164.69 (125.63 to 198.57)

Hunan	Brain and central nervous system cancer	102<br>(59.9 to 160)	164.74<br>(96.38 to 256.64)	108<br>(82.6 to 136)	160.65<br>(123.07 to 201.91)
Inner Mongolia	Brain and central nervous system cancer	37<br>(24.5 to 57)	162.84<br>(107.59 to 250.67)	33.4<br>(25.9 to 47.1)	128.89<br>(100.05 to 181.8)
Jiangsu	Brain and central nervous system cancer	99.9<br>(70.6 to 120)	145.18<br>(102.64 to 173.92)	121<br>(79.7 to 153)	148.28<br>(97.66 to 186.82)
Jiangxi	Brain and central nervous system cancer	83.6<br>(37.6 to 123)	211.53<br>(95.18 to 310.56)	61.6<br>(48.5 to 73.9)	129.6<br>(101.96 to 155.5)
Jilin	Brain and central nervous system cancer	37.6<br>(28 to 51.5)	144.96<br>(107.78 to 198.63)	35.2<br>(26.9 to 49)	126.21<br>(96.41 to 175.7)
Liaoning	Brain and central nervous system cancer	63.4<br>(46.2 to 79.1)	155.54<br>(113.36 to 193.93)	78.2<br>(61 to 94.4)	175.21<br>(136.69 to 211.52)
Ningxia	Brain and central nervous system cancer	10.6<br>(4.63 to 14.8)	214.47<br>(93.75 to 298.87)	9.05<br>(6.33 to 11.4)	129.01<br>(90.13 to 162.92)
Qinghai	Brain and central nervous system cancer	9.16<br>(4.43 to 17.2)	193.33<br>(93.47 to 363.74)	8.35<br>(6.36 to 12.1)	132.97<br>(101.16 to 192.59)
Shaanxi	Brain and central nervous system cancer	39.5<br>(31.6 to 52.2)	114.7<br>(91.88 to 151.81)	49.5<br>(39.4 to 61.9)	125.41<br>(99.65 to 156.72)
Shandong	Brain and central nervous system cancer	127<br>(91.9 to 159)	148.84<br>(107.35 to 185.76)	158<br>(121 to 193)	159.02<br>(122.16 to 194.22)
Shanghai	Brain and central nervous system cancer	19.1<br>(14.8 to 22.4)	136.65<br>(105.67 to 160.26)	29.9<br>(19.6 to 46.5)	106.99<br>(70.33 to 166.27)
Shanxi	Brain and central nervous system cancer	43.2<br>(29.9 to 56.6)	142.6<br>(98.59 to 186.98)	45.3<br>(36 to 59.7)	117.36<br>(93.19 to 154.44)
Sichuan	Brain and central nervous system cancer	161<br>(92.9 to 217)	144<br>(83.28 to 194.62)	129<br>(94.2 to 159)	154.21<br>(112.8 to 191.03)
Tianjin	Brain and central nervous system cancer	15.9<br>(10.7 to 19.6)	173.06<br>(116.06 to 212.76)	23.8<br>(19.2 to 29.4)	155.92<br>(125.85 to 192.86)
Tibet	Brain and central nervous system cancer	3.42<br>(1.87 to 9.38)	146.94<br>(80.13 to 402.34)	3.26<br>(2.17 to 5.71)	94.2<br>(62.88 to 165.25)
Xinjiang	Brain and central nervous system cancer	24.8<br>(15.9 to 48.5)	157.43<br>(100.79 to 307.73)	33.2<br>(23.6 to 49.8)	136.27<br>(96.82 to 204.36)
Yunnan	Brain and central nervous system cancer	72.3<br>(35.9 to 131)	185.57<br>(92.17 to 336.33)	61.4<br>(49.1 to 83.3)	124.88<br>(99.88 to 169.42)
Zhejiang	Brain and central nervous system cancer	66.5<br>(39.2 to 84.3)	150.74<br>(88.88 to 191.25)	71<br>(48.4 to 96.7)	116.11<br>(79.03 to 158.03)
China	Thyroid cancer	97.4<br>(89.7 to 110)	8.14<br>(7.49 to 9.21)	173<br>(159 to 191)	12.22<br>(11.27 to 13.51)
Taiwan	Thyroid cancer	2.66<br>(2.49 to 2.84)	13.05<br>(12.22 to 13.9)	4.89<br>(4.4 to 5.49)	20.72<br>(18.68 to 23.28)
Hong Kong Special Administrative Region of China	Thyroid cancer	0.653<br>(0.462 to 0.755)	11.32<br>(8.01 to 13.1)	0.923<br>(0.74 to 1.2)	12.29<br>(9.86 to 16.01)
Macau Special Administrative Region of China	Thyroid cancer	0.0265<br>(0.0221 to 0.0304)	7.47<br>(6.24 to 8.56)	0.0562<br>(0.0411 to 0.0753)	8.22<br>(6.01 to 11.01)
Anhui	Thyroid cancer	4.82<br>(3.95 to 5.86)	8.14<br>(6.67 to 9.89)	6.64<br>(5.44 to 8.02)	10.86<br>(8.9 to 13.11)
Beijing	Thyroid cancer	1.17<br>(0.814 to 1.46)	10.39<br>(7.26 to 13.03)	1.91<br>(1.4 to 2.42)	7.91<br>(5.79 to 10.05)
Chongqing	Thyroid cancer	0.932<br>(0.721 to 1.46)	5.9<br>(4.57 to 9.27)	2.03<br>(1.53 to 2.94)	7.58<br>(5.74 to 10.99)
Fujian	Thyroid cancer	3.16<br>(2.06 to 3.84)	9.98<br>(6.53 to 12.14)	4.47<br>(3.5 to 5.58)	11.35<br>(8.89 to 14.17)
Gansu	Thyroid cancer	2.46<br>(2.02 to 2.93)	10.42<br>(8.52 to 12.4)	4.06<br>(3.27 to 4.93)	15.29<br>(12.31 to 18.55)
Guangdong	Thyroid cancer	5.92<br>(4.47 to 7.53)	8.99<br>(6.79 to 11.44)	11.5<br>(8.84 to 14)	9.83<br>(7.57 to 11.96)
Guangxi	Thyroid cancer	3.45<br>(2.84 to 4.2)	7.94<br>(6.53 to 9.66)	5.85<br>(4.75 to 7.17)	12.1<br>(9.84 to 14.85)
Guizhou	Thyroid cancer	4.25<br>(3.5 to 5.29)	12.81<br>(10.54 to 15.92)	5.87<br>(4.58 to 7.37)	17.36<br>(13.53 to 21.79)
Hainan	Thyroid cancer	0.497<br>(0.348 to 0.645)	7.13<br>(5 to 9.25)	0.742<br>(0.559 to 0.958)	7.74<br>(5.83 to 9.99)
Hebei	Thyroid cancer	5.25<br>(4.52 to 6.31)	8.25<br>(7.1 to 9.92)	13.5<br>(10.7 to 17.3)	17.7<br>(13.97 to 22.65)
Heilongjiang	Thyroid cancer	2.97<br>(2.29 to 3.72)	8.15<br>(6.27 to 10.21)	4.66<br>(3.78 to 5.7)	11.88<br>(9.63 to 14.53)

Henan	Thyroid cancer	4.97<br>(4.28 to 6.26)	5.63<br>(4.85 to 7.09)	11.9<br>(9.76 to 14.4)	12.38<br>(10.14 to 14.96)
Hubei	Thyroid cancer	3.49<br>(2.95 to 4.19)	6.27<br>(5.31 to 7.54)	6.02<br>(4.82 to 7.55)	10.89<br>(8.72 to 13.65)
Hunan	Thyroid cancer	5.5<br>(4.52 to 8.56)	8.85<br>(7.27 to 13.76)	10.3<br>(8.22 to 14)	15.41<br>(12.24 to 20.78)
Inner Mongolia	Thyroid cancer	2.31<br>(1.79 to 2.85)	10.16<br>(7.87 to 12.52)	4.5<br>(3.59 to 5.61)	17.38<br>(13.88 to 21.67)
Jiangsu	Thyroid cancer	4.76<br>(4.05 to 5.8)	6.91<br>(5.89 to 8.42)	8.52<br>(6.78 to 10.4)	10.43<br>(8.31 to 12.76)
Jiangxi	Thyroid cancer	3.21<br>(2.53 to 4.68)	8.13<br>(6.39 to 11.84)	4.08<br>(3.31 to 5.18)	8.59<br>(6.96 to 10.9)
Jilin	Thyroid cancer	3<br>(2.42 to 3.63)	11.57<br>(9.32 to 14)	5.61<br>(4.4 to 6.92)	20.11<br>(15.76 to 24.82)
Liaoning	Thyroid cancer	4.21<br>(3.2 to 5.31)	10.33<br>(7.85 to 13.03)	8.29<br>(6.4 to 10.1)	18.57<br>(14.33 to 22.73)
Ningxia	Thyroid cancer	0.344<br>(0.261 to 0.432)	6.97<br>(5.29 to 8.74)	0.611<br>(0.464 to 0.768)	8.71<br>(6.61 to 10.95)
Qinghai	Thyroid cancer	0.484<br>(0.375 to 0.604)	10.22<br>(7.92 to 12.76)	0.975<br>(0.759 to 1.22)	15.51<br>(12.08 to 19.45)
Shaanxi	Thyroid cancer	1.69<br>(1.35 to 2.2)	4.92<br>(3.93 to 6.38)	4.98<br>(3.97 to 6.14)	12.61<br>(10.05 to 15.54)
Shandong	Thyroid cancer	7.37<br>(5.97 to 9.12)	8.61<br>(6.97 to 10.66)	12.4<br>(10.1 to 15)	12.46<br>(10.14 to 15.12)
Shanghai	Thyroid cancer	1.68<br>(1.05 to 2.11)	12.02<br>(7.5 to 15.11)	2.84<br>(1.7 to 3.68)	10.18<br>(6.07 to 13.16)
Shanxi	Thyroid cancer	2.21<br>(1.77 to 3.01)	7.31<br>(5.86 to 9.95)	4.02<br>(3.07 to 5.2)	10.4<br>(7.94 to 13.46)
Sichuan	Thyroid cancer	5.5<br>(4.45 to 8.4)	4.93<br>(3.99 to 7.53)	8.06<br>(6.32 to 11.3)	9.66<br>(7.57 to 13.48)
Tianjin	Thyroid cancer	0.835<br>(0.643 to 1.03)	9.08<br>(6.99 to 11.21)	1.39<br>(1.1 to 1.73)	9.12<br>(7.22 to 11.36)
Tibet	Thyroid cancer	0.208<br>(0.157 to 0.385)	8.92<br>(6.76 to 16.53)	0.264<br>(0.202 to 0.405)	7.63<br>(5.84 to 11.73)
Xinjiang	Thyroid cancer	1.22<br>(1 to 1.48)	7.75<br>(6.35 to 9.36)	2.84<br>(2.27 to 3.58)	11.67<br>(9.33 to 14.68)
Yunnan	Thyroid cancer	3.45<br>(2.74 to 4.68)	8.85<br>(7.03 to 12.01)	6.6<br>(5.31 to 8.39)	13.41<br>(10.8 to 17.07)
Zhejiang	Thyroid cancer	5.38<br>(3.28 to 6.76)	12.2<br>(7.43 to 15.33)	6.13<br>(4.6 to 7.73)	10.01<br>(7.52 to 12.64)
China	Mesothelioma	38.6<br>(28.8 to 51.7)	3.23<br>(2.4 to 4.32)	77.2<br>(70.8 to 82.2)	5.47<br>(5.01 to 5.82)
Taiwan	Mesothelioma	0.615<br>(0.495 to 0.8)	3.02<br>(2.43 to 3.92)	1.16<br>(1.07 to 1.25)	4.91<br>(4.55 to 5.29)
Hong Kong Special Administrative Region of China	Mesothelioma	0.237<br>(0.178 to 0.291)	4.11<br>(3.1 to 5.04)	0.332<br>(0.272 to 0.407)	4.43<br>(3.62 to 5.42)
Macau Special Administrative Region of China	Mesothelioma	0.00901<br>(0.00771 to 0.0105)	2.54<br>(2.17 to 2.96)	0.0148<br>(0.00971 to 0.0219)	2.17<br>(1.42 to 3.2)
Anhui	Mesothelioma	1.29<br>(0.974 to 1.65)	2.18<br>(1.64 to 2.79)	2.34<br>(1.92 to 2.86)	3.83<br>(3.15 to 4.68)
Beijing	Mesothelioma	0.811<br>(0.508 to 1.15)	7.23<br>(4.53 to 10.24)	1.23<br>(0.998 to 1.5)	5.08<br>(4.14 to 6.2)
Chongqing	Mesothelioma	0.29<br>(0.166 to 0.44)	1.84<br>(1.05 to 2.79)	0.605<br>(0.482 to 0.751)	2.26<br>(1.8 to 2.81)
Fujian	Mesothelioma	0.401<br>(0.285 to 0.574)	1.27<br>(0.9 to 1.82)	0.701<br>(0.558 to 0.866)	1.78<br>(1.42 to 2.2)
Gansu	Mesothelioma	0.983<br>(0.64 to 1.41)	4.16<br>(2.71 to 5.98)	1.94<br>(1.58 to 2.34)	7.31<br>(5.96 to 8.79)
Guangdong	Mesothelioma	2.13<br>(1.41 to 3.19)	3.23<br>(2.14 to 4.84)	4.23<br>(3.44 to 5.23)	3.62<br>(2.94 to 4.48)
Guangxi	Mesothelioma	1.38<br>(0.923 to 2.03)	3.18<br>(2.12 to 4.66)	3.44<br>(2.73 to 4.31)	7.12<br>(5.64 to 8.91)
Guizhou	Mesothelioma	1.48<br>(0.984 to 2.11)	4.46<br>(2.96 to 6.36)	2.68<br>(2 to 3.42)	7.91<br>(5.9 to 10.11)
Hainan	Mesothelioma	0.0921<br>(0.0544 to 0.141)	1.32<br>(0.78 to 2.02)	0.144<br>(0.108 to 0.184)	1.5<br>(1.13 to 1.92)

Hebei	Mesothelioma	2.61 (1.94 to 3.76)	4.1 (3.05 to 5.91)	8.53 (7.12 to 10.2)	11.16 (9.31 to 13.37)
Heilongjiang	Mesothelioma	1.96 (1.37 to 2.78)	5.36 (3.75 to 7.63)	2.99 (2.44 to 3.66)	7.62 (6.23 to 9.33)
Henan	Mesothelioma	1.56 (1.24 to 2.12)	1.77 (1.4 to 2.41)	4.66 (3.83 to 5.57)	4.84 (3.98 to 5.79)
Hubei	Mesothelioma	0.927 (0.707 to 1.27)	1.67 (1.27 to 2.29)	1.77 (1.44 to 2.17)	3.2 (2.61 to 3.93)
Hunan	Mesothelioma	3.72 (2.3 to 5.46)	5.99 (3.69 to 8.78)	6.63 (5.42 to 8.16)	9.88 (8.07 to 12.16)
Inner Mongolia	Mesothelioma	1.05 (0.713 to 1.57)	4.64 (3.13 to 6.91)	2.05 (1.65 to 2.53)	7.91 (6.38 to 9.78)
Jiangsu	Mesothelioma	1.36 (1.07 to 1.83)	1.98 (1.55 to 2.66)	2.29 (1.82 to 2.78)	2.8 (2.23 to 3.4)
Jiangxi	Mesothelioma	1.41 (0.768 to 2.12)	3.58 (1.94 to 5.36)	2.08 (1.69 to 2.51)	4.39 (3.55 to 5.29)
Jilin	Mesothelioma	2.97 (1.98 to 4.32)	11.43 (7.62 to 16.63)	3.42 (2.69 to 4.18)	12.26 (9.66 to 15)
Liaoning	Mesothelioma	1.75 (1.31 to 2.42)	4.3 (3.2 to 5.92)	4.22 (3.42 to 5.1)	9.45 (7.67 to 11.43)
Ningxia	Mesothelioma	0.0665 (0.0446 to 0.0959)	1.35 (0.9 to 1.94)	0.141 (0.113 to 0.174)	2.01 (1.61 to 2.48)
Qinghai	Mesothelioma	0.143 (0.0983 to 0.204)	3.03 (2.08 to 4.32)	0.438 (0.349 to 0.545)	6.97 (5.56 to 8.68)
Shaanxi	Mesothelioma	0.646 (0.47 to 0.825)	1.88 (1.37 to 2.4)	2.36 (1.86 to 2.87)	5.96 (4.72 to 7.27)
Shandong	Mesothelioma	2.44 (1.58 to 3.6)	2.85 (1.85 to 4.2)	4.27 (3.5 to 5.19)	4.3 (3.52 to 5.23)
Shanghai	Mesothelioma	0.464 (0.278 to 0.645)	3.32 (1.99 to 4.62)	0.878 (0.695 to 1.09)	3.14 (2.49 to 3.91)
Shanxi	Mesothelioma	0.995 (0.63 to 1.46)	3.28 (2.08 to 4.82)	1.92 (1.53 to 2.36)	4.96 (3.95 to 6.1)
Sichuan	Mesothelioma	1.65 (1.32 to 2.19)	1.48 (1.18 to 1.97)	3.26 (2.6 to 4)	3.9 (3.12 to 4.8)
Tianjin	Mesothelioma	0.291 (0.199 to 0.418)	3.16 (2.17 to 4.54)	0.585 (0.476 to 0.712)	3.83 (3.12 to 4.66)
Tibet	Mesothelioma	0.218 (0.112 to 0.336)	9.37 (4.79 to 14.4)	0.327 (0.265 to 0.404)	9.47 (7.66 to 11.68)
Xinjiang	Mesothelioma	0.346 (0.274 to 0.456)	2.19 (1.74 to 2.89)	1.11 (0.903 to 1.35)	4.55 (3.71 to 5.55)
Yunnan	Mesothelioma	1.97 (1.28 to 2.85)	5.07 (3.28 to 7.32)	4.14 (3.34 to 5.1)	8.42 (6.8 to 10.37)
Zhejiang	Mesothelioma	0.976 (0.563 to 1.46)	2.21 (1.28 to 3.32)	1.48 (1.18 to 1.82)	2.43 (1.93 to 2.98)
China	Hodgkin lymphoma	256 (142 to 314)	21.38 (11.82 to 26.21)	90.9 (64.6 to 106)	6.44 (4.57 to 7.5)
Taiwan	Hodgkin lymphoma	1.06 (0.902 to 1.34)	5.2 (4.42 to 6.58)	1.04 (0.709 to 1.22)	4.43 (3.01 to 5.19)
Hong Kong Special Administrative Region of China	Hodgkin lymphoma	0.363 (0.274 to 0.431)	6.29 (4.76 to 7.47)	0.397 (0.261 to 0.521)	5.29 (3.48 to 6.94)
Macau Special Administrative Region of China	Hodgkin lymphoma	0.0327 (0.0174 to 0.0422)	9.23 (4.91 to 11.9)	0.035 (0.0185 to 0.0485)	5.11 (2.7 to 7.09)
Anhui	Hodgkin lymphoma	17.2 (7.18 to 24.1)	28.98 (12.11 to 40.59)	4.14 (2.83 to 5.14)	6.76 (4.62 to 8.4)
Beijing	Hodgkin lymphoma	1.53 (0.775 to 2.1)	13.66 (6.91 to 18.69)	0.98 (0.582 to 1.3)	4.06 (2.41 to 5.38)
Chongqing	Hodgkin lymphoma	2.98 (1.83 to 3.77)	18.91 (11.57 to 23.92)	1.32 (0.97 to 1.97)	4.95 (3.63 to 7.36)
Fujian	Hodgkin lymphoma	5.76 (3.01 to 7.61)	18.2 (9.53 to 24.05)	2.19 (1.51 to 2.77)	5.55 (3.83 to 7.04)
Gansu	Hodgkin lymphoma	6 (4.13 to 7.25)	25.35 (17.47 to 30.64)	1.48 (1.06 to 2.43)	5.57 (4 to 9.14)
Guangdong	Hodgkin lymphoma	12.5 (6.24 to 16.9)	18.95 (9.49 to 25.75)	5.6 (4.09 to 6.97)	4.79 (3.5 to 5.96)
Guangxi	Hodgkin lymphoma	11.2 (5.92 to 14.9)	25.87 (13.62 to 34.18)	3.36 (2.39 to 4.52)	6.96 (4.95 to 9.35)

Guizhou	Hodgkin lymphoma	6.14<br>(4.5 to 9.7)	18.5<br>(13.57 to 29.22)	1.8<br>(0.985 to 3.74)	5.33<br>(2.91 to 11.06)
Hainan	Hodgkin lymphoma	0.714<br>(0.527 to 1.09)	10.24<br>(7.57 to 15.7)	0.386<br>(0.254 to 0.664)	4.03<br>(2.65 to 6.93)
Hebei	Hodgkin lymphoma	12.6<br>(6.54 to 16.7)	19.81<br>(10.28 to 26.23)	7.18<br>(3.05 to 10.5)	9.4<br>(3.99 to 13.67)
Heilongjiang	Hodgkin lymphoma	6.07<br>(4.06 to 7.57)	16.65<br>(11.13 to 20.76)	2.8<br>(1.96 to 3.61)	7.13<br>(4.99 to 9.19)
Henan	Hodgkin lymphoma	15.3<br>(9.44 to 19.4)	17.36<br>(10.69 to 22.02)	6.03<br>(4.21 to 7.66)	6.26<br>(4.37 to 7.95)
Hubei	Hodgkin lymphoma	11.1<br>(5.87 to 14.7)	20.01<br>(10.56 to 26.39)	4.2<br>(2.59 to 5.27)	7.6<br>(4.68 to 9.53)
Hunan	Hodgkin lymphoma	11.8<br>(7.57 to 14.3)	18.95<br>(12.17 to 22.96)	4.44<br>(3.11 to 6.64)	6.61<br>(4.64 to 9.89)
Inner Mongolia	Hodgkin lymphoma	4.13<br>(2.84 to 5.14)	18.17<br>(12.51 to 22.62)	1.35<br>(1.02 to 2)	5.2<br>(3.95 to 7.74)
Jiangsu	Hodgkin lymphoma	15.1<br>(6.08 to 21)	21.92<br>(8.84 to 30.47)	6.08<br>(3.09 to 8.3)	7.45<br>(3.79 to 10.16)
Jiangxi	Hodgkin lymphoma	16.3<br>(5.54 to 23.8)	41.11<br>(14 to 60.11)	3.37<br>(2.26 to 4.15)	7.09<br>(4.74 to 8.73)
Jilin	Hodgkin lymphoma	4.77<br>(2.95 to 6.19)	18.38<br>(11.37 to 23.85)	1.66<br>(1.22 to 2.22)	5.95<br>(4.38 to 7.96)
Liaoning	Hodgkin lymphoma	8.39<br>(3.83 to 12.1)	20.57<br>(9.39 to 29.6)	3.92<br>(1.93 to 5.27)	8.77<br>(4.32 to 11.8)
Ningxia	Hodgkin lymphoma	1.07<br>(0.572 to 1.47)	21.58<br>(11.58 to 29.83)	0.34<br>(0.255 to 0.494)	4.84<br>(3.63 to 7.04)
Qinghai	Hodgkin lymphoma	0.816<br>(0.567 to 1.36)	17.23<br>(11.96 to 28.72)	0.329<br>(0.18 to 0.682)	5.23<br>(2.87 to 10.86)
Shaanxi	Hodgkin lymphoma	4.82<br>(3.05 to 6.55)	14.02<br>(8.87 to 19.04)	2.06<br>(1.57 to 3.02)	5.22<br>(3.97 to 7.64)
Shandong	Hodgkin lymphoma	19.7<br>(8.36 to 27.8)	23.07<br>(9.77 to 32.48)	6.47<br>(4.01 to 8.23)	6.51<br>(4.03 to 8.28)
Shanghai	Hodgkin lymphoma	1.54<br>(0.866 to 1.99)	11<br>(6.2 to 14.28)	1.2<br>(0.697 to 1.6)	4.29<br>(2.5 to 5.72)
Shanxi	Hodgkin lymphoma	4.82<br>(3.12 to 6.03)	15.91<br>(10.3 to 19.91)	1.81<br>(1.35 to 2.62)	4.69<br>(3.48 to 6.79)
Sichuan	Hodgkin lymphoma	24.6<br>(13.6 to 32.1)	22<br>(12.22 to 28.78)	5.78<br>(4.36 to 7.64)	6.93<br>(5.22 to 9.15)
Tianjin	Hodgkin lymphoma	1.4<br>(0.747 to 1.83)	15.21<br>(8.13 to 19.93)	0.868<br>(0.548 to 1.12)	5.69<br>(3.59 to 7.35)
Tibet	Hodgkin lymphoma	0.743<br>(0.515 to 1)	31.89<br>(22.1 to 43)	0.264<br>(0.152 to 0.54)	7.63<br>(4.4 to 15.63)
Xinjiang	Hodgkin lymphoma	4.4<br>(2.62 to 5.85)	27.92<br>(16.63 to 37.09)	2.07<br>(1.4 to 2.71)	8.48<br>(5.75 to 11.14)
Yunnan	Hodgkin lymphoma	11<br>(6.78 to 14.1)	28.31<br>(17.39 to 36.16)	2.96<br>(2.07 to 5.15)	6.03<br>(4.21 to 10.47)
Zhejiang	Hodgkin lymphoma	11<br>(3.42 to 16)	24.96<br>(7.77 to 36.25)	4.04<br>(1.99 to 5.72)	6.6<br>(3.25 to 9.34)
China	Non-Hodgkin lymphoma	746<br>(698 to 791)	62.29<br>(58.31 to 66.09)	1160<br>(1100 to 1220)	81.98<br>(77.9 to 86.17)
Taiwan	Non-Hodgkin lymphoma	17<br>(16.5 to 17.6)	83.49<br>(80.75 to 86.17)	29.7<br>(27.8 to 31.7)	125.99<br>(117.91 to 134.6)
Hong Kong Special Administrative Region of China	Non-Hodgkin lymphoma	5.05<br>(4.6 to 5.55)	87.67<br>(79.78 to 96.34)	6.36<br>(5.3 to 7.66)	84.7<br>(70.53 to 101.94)
Macau Special Administrative Region of China	Non-Hodgkin lymphoma	0.174<br>(0.154 to 0.196)	49.09<br>(43.51 to 55.36)	0.36<br>(0.232 to 0.447)	52.59<br>(33.89 to 65.4)
Anhui	Non-Hodgkin lymphoma	54.3<br>(47.2 to 62.3)	91.7<br>(79.65 to 105.19)	60.1<br>(51 to 70.8)	98.36<br>(83.36 to 115.81)
Beijing	Non-Hodgkin lymphoma	7.65<br>(6.71 to 8.69)	68.23<br>(59.86 to 77.44)	15.3<br>(12.6 to 18.3)	63.64<br>(52.43 to 75.73)
Chongqing	Non-Hodgkin lymphoma	9.18<br>(7.54 to 10.8)	58.16<br>(47.76 to 68.27)	18.7<br>(15 to 22.7)	69.84<br>(56.29 to 85.06)
Fujian	Non-Hodgkin lymphoma	18.7<br>(16.7 to 21.1)	59.08<br>(52.66 to 66.66)	30.9<br>(25.5 to 37.2)	78.4<br>(64.69 to 94.41)
Gansu	Non-Hodgkin lymphoma	17<br>(14.5 to 19.8)	71.72<br>(61.39 to 83.71)	19.4<br>(16.1 to 22.9)	72.92<br>(60.67 to 86.14)

Guangdong	Non-Hodgkin lymphoma	34<br>(29.3 to 39.1)	51.67<br>(44.51 to 59.46)	69.3<br>(57.8 to 82.7)	59.33<br>(49.43 to 70.75)
Guangxi	Non-Hodgkin lymphoma	27.3<br>(23.6 to 31.3)	62.71<br>(54.28 to 72.07)	41.7<br>(34.6 to 50.7)	86.23<br>(71.7 to 105.03)
Guizhou	Non-Hodgkin lymphoma	23<br>(19.1 to 27.4)	69.21<br>(57.42 to 82.6)	23.7<br>(18.8 to 29.6)	69.97<br>(55.67 to 87.46)
Hainan	Non-Hodgkin lymphoma	3.25<br>(2.37 to 4.22)	46.71<br>(34.09 to 60.56)	4.56<br>(3.7 to 5.65)	47.55<br>(38.6 to 58.97)
Hebei	Non-Hodgkin lymphoma	35.9<br>(31.6 to 40.3)	56.41<br>(49.73 to 63.25)	69.3<br>(58.4 to 84.1)	90.69<br>(76.37 to 110)
Heilongjiang	Non-Hodgkin lymphoma	19.2<br>(16.5 to 22.5)	52.77<br>(45.26 to 61.7)	31.2<br>(25.8 to 37.7)	79.52<br>(65.79 to 96.16)
Henan	Non-Hodgkin lymphoma	40.3<br>(35.8 to 45.3)	45.63<br>(40.51 to 51.31)	68.6<br>(57.9 to 81)	71.23<br>(60.14 to 84.13)
Hubei	Non-Hodgkin lymphoma	34.5<br>(30.8 to 38.6)	62.17<br>(55.37 to 69.56)	54.7<br>(45.7 to 64.8)	98.91<br>(82.65 to 117.09)
Hunan	Non-Hodgkin lymphoma	47<br>(41.6 to 52.5)	75.6<br>(66.91 to 84.44)	69.3<br>(57.6 to 83.5)	103.17<br>(85.75 to 124.39)
Inner Mongolia	Non-Hodgkin lymphoma	15.5<br>(13.2 to 18)	68.21<br>(57.85 to 79.16)	23.4<br>(19.5 to 28.2)	90.45<br>(75.13 to 108.78)
Jiangsu	Non-Hodgkin lymphoma	42.5<br>(38.5 to 47)	61.71<br>(55.95 to 68.32)	79.5<br>(66.1 to 94.7)	97.37<br>(81.01 to 115.96)
Jiangxi	Non-Hodgkin lymphoma	35.1<br>(29.4 to 41.1)	88.66<br>(74.25 to 103.88)	38.3<br>(32.3 to 45.4)	80.61<br>(67.93 to 95.53)
Jilin	Non-Hodgkin lymphoma	15.5<br>(13.1 to 18.4)	59.76<br>(50.34 to 71.02)	24.6<br>(19.6 to 29.5)	88.14<br>(70.22 to 105.85)
Liaoning	Non-Hodgkin lymphoma	25.3<br>(21.2 to 30)	61.9<br>(51.96 to 73.49)	52.7<br>(43.8 to 62.7)	117.94<br>(98.12 to 140.38)
Ningxia	Non-Hodgkin lymphoma	3.66<br>(2.98 to 4.5)	74.04<br>(60.24 to 91.12)	4.13<br>(3.38 to 4.99)	58.82<br>(48.13 to 71.08)
Qinghai	Non-Hodgkin lymphoma	3.11<br>(2.58 to 3.69)	65.65<br>(54.46 to 77.99)	4.33<br>(3.5 to 5.28)	68.91<br>(55.72 to 84.07)
Shaanxi	Non-Hodgkin lymphoma	16.9<br>(14.9 to 19.3)	49.19<br>(43.33 to 55.94)	28<br>(22.8 to 33.7)	70.9<br>(57.61 to 85.2)
Shandong	Non-Hodgkin lymphoma	44.4<br>(38.7 to 50.7)	51.85<br>(45.22 to 59.21)	78.2<br>(66 to 92.7)	78.65<br>(66.46 to 93.24)
Shanghai	Non-Hodgkin lymphoma	10.7<br>(9.41 to 12.1)	76.61<br>(67.33 to 86.43)	22.1<br>(18.1 to 26.9)	79.15<br>(64.79 to 96.36)
Shanxi	Non-Hodgkin lymphoma	18<br>(15.6 to 20.6)	59.29<br>(51.4 to 68.08)	28.2<br>(23 to 33.9)	73.06<br>(59.54 to 87.79)
Sichuan	Non-Hodgkin lymphoma	67.1<br>(57.6 to 77)	60.13<br>(51.59 to 68.99)	83<br>(68.5 to 98.3)	99.38<br>(81.99 to 117.72)
Tianjin	Non-Hodgkin lymphoma	6.51<br>(5.64 to 7.44)	70.76<br>(61.34 to 80.84)	15.3<br>(12.8 to 18.2)	100.15<br>(83.88 to 118.99)
Tibet	Non-Hodgkin lymphoma	1.61<br>(1.32 to 1.96)	68.93<br>(56.83 to 83.98)	2.41<br>(1.97 to 2.92)	69.66<br>(57.01 to 84.46)
Xinjiang	Non-Hodgkin lymphoma	9.79<br>(8.23 to 11.3)	62.06<br>(52.19 to 71.49)	15.3<br>(12.5 to 18.4)	62.62<br>(51.42 to 75.46)
Yunnan	Non-Hodgkin lymphoma	27.1<br>(22.5 to 32.3)	69.5<br>(57.85 to 82.89)	35.2<br>(29.6 to 42.7)	71.62<br>(60.1 to 86.76)
Zhejiang	Non-Hodgkin lymphoma	26.5<br>(23.1 to 30.2)	60.11<br>(52.34 to 68.43)	39.9<br>(33 to 47.9)	65.21<br>(53.96 to 78.34)
China	Multiple myeloma	163<br>(139 to 184)	13.65<br>(11.57 to 15.38)	315<br>(270 to 338)	22.28<br>(19.12 to 23.94)
Taiwan	Multiple myeloma	2.77<br>(2.55 to 3.58)	13.58<br>(12.48 to 17.55)	8.86<br>(6.84 to 9.88)	37.55<br>(29.02 to 41.89)
Hong Kong Special Administrative Region of China	Multiple myeloma	1.18<br>(0.894 to 1.34)	20.54<br>(15.51 to 23.33)	2.23<br>(1.75 to 2.86)	29.66<br>(23.28 to 38.04)
Macau Special Administrative Region of China	Multiple myeloma	0.0449<br>(0.0398 to 0.0542)	12.65<br>(11.22 to 15.27)	0.141<br>(0.0963 to 0.196)	20.67<br>(14.09 to 28.66)
Anhui	Multiple myeloma	9.02<br>(6.07 to 11.3)	15.22<br>(10.24 to 19)	12.5<br>(10.3 to 14.9)	20.48<br>(16.8 to 24.34)
Beijing	Multiple myeloma	2.25<br>(1.65 to 2.68)	20.04<br>(14.74 to 23.88)	5.87<br>(4.12 to 7.24)	24.38<br>(17.09 to 30.03)
Chongqing	Multiple myeloma	2.26<br>(1.85 to 2.77)	14.32<br>(11.73 to 17.53)	5.59<br>(4.4 to 7.19)	20.91<br>(16.46 to 26.9)

Fujian	Multiple myeloma	3.36<br>(2.78 to 4.05)	10.63<br>(8.8 to 12.8)	7.31<br>(5.95 to 8.91)	18.55<br>(15.1 to 22.61)
Gansu	Multiple myeloma	3.08<br>(2.29 to 4.08)	13<br>(9.69 to 17.24)	4.85<br>(3.93 to 5.95)	18.26<br>(14.8 to 22.38)
Guangdong	Multiple myeloma	9.72<br>(7.42 to 11.7)	14.77<br>(11.27 to 17.72)	22.1<br>(16.9 to 27)	18.93<br>(14.42 to 23.09)
Guangxi	Multiple myeloma	5.33<br>(4.45 to 6.38)	12.26<br>(10.22 to 14.67)	9.35<br>(7.65 to 11.3)	19.35<br>(15.83 to 23.47)
Guizhou	Multiple myeloma	4.22<br>(3.44 to 5.19)	12.7<br>(10.36 to 15.63)	6.17<br>(4.92 to 7.7)	18.24<br>(14.54 to 22.77)
Hainan	Multiple myeloma	0.663<br>(0.504 to 0.925)	9.52<br>(7.24 to 13.29)	1.39<br>(1.08 to 1.81)	14.48<br>(11.3 to 18.86)
Hebei	Multiple myeloma	6.88<br>(5.98 to 8.93)	10.81<br>(9.4 to 14.04)	15.9<br>(12.9 to 20.4)	20.81<br>(16.88 to 26.75)
Heilongjiang	Multiple myeloma	4.2<br>(3.52 to 5.4)	11.53<br>(9.65 to 14.8)	8.9<br>(7.32 to 11.2)	22.68<br>(18.65 to 28.6)
Henan	Multiple myeloma	8.94<br>(7.81 to 10.5)	10.13<br>(8.85 to 11.85)	17.8<br>(14.8 to 22.1)	18.52<br>(15.36 to 22.95)
Hubei	Multiple myeloma	6.64<br>(5.79 to 7.66)	11.95<br>(10.41 to 13.79)	14.4<br>(11.5 to 17.4)	26.05<br>(20.71 to 31.45)
Hunan	Multiple myeloma	11<br>(6.87 to 12.9)	17.75<br>(11.05 to 20.73)	17.9<br>(13.6 to 21.8)	26.71<br>(20.28 to 32.5)
Inner Mongolia	Multiple myeloma	2.92<br>(2.37 to 3.5)	12.86<br>(10.43 to 15.41)	5.5<br>(4.54 to 6.86)	21.25<br>(17.54 to 26.48)
Jiangsu	Multiple myeloma	9.81<br>(8.56 to 11.2)	14.26<br>(12.43 to 16.28)	21.3<br>(16.7 to 25.5)	26.03<br>(20.4 to 31.25)
Jiangxi	Multiple myeloma	7.45<br>(4.48 to 8.98)	18.84<br>(11.33 to 22.71)	10.8<br>(7.61 to 13.3)	22.76<br>(16 to 28.06)
Jilin	Multiple myeloma	3.48<br>(2.89 to 4.46)	13.41<br>(11.12 to 17.21)	6.27<br>(4.93 to 7.71)	22.49<br>(17.68 to 27.63)
Liaoning	Multiple myeloma	6.07<br>(4.99 to 7.65)	14.87<br>(12.24 to 18.76)	13.4<br>(10.3 to 16.1)	29.94<br>(23.06 to 36.12)
Ningxia	Multiple myeloma	0.587<br>(0.405 to 0.714)	11.89<br>(8.2 to 14.45)	1.21<br>(0.95 to 1.51)	17.22<br>(13.54 to 21.48)
Qinghai	Multiple myeloma	0.544<br>(0.442 to 0.653)	11.49<br>(9.34 to 13.78)	1.01<br>(0.807 to 1.28)	16.06<br>(12.84 to 20.45)
Shaanxi	Multiple myeloma	3.86<br>(2.84 to 4.44)	11.2<br>(8.25 to 12.91)	8.22<br>(6.42 to 10.2)	20.81<br>(16.26 to 25.87)
Shandong	Multiple myeloma	11.2<br>(9.37 to 14.3)	13.06<br>(10.94 to 16.75)	21.1<br>(17.1 to 26.7)	21.2<br>(17.19 to 26.86)
Shanghai	Multiple myeloma	2.93<br>(2.02 to 3.42)	21<br>(14.42 to 24.49)	8.34<br>(4.71 to 10.7)	29.86<br>(16.87 to 38.19)
Shanxi	Multiple myeloma	3.93<br>(3.3 to 4.83)	12.96<br>(10.9 to 15.95)	6.62<br>(5.19 to 8.58)	17.15<br>(13.43 to 22.21)
Sichuan	Multiple myeloma	14.3<br>(11.9 to 16.3)	12.85<br>(10.67 to 14.61)	21.2<br>(16.8 to 25.7)	25.42<br>(20.16 to 30.81)
Tianjin	Multiple myeloma	1.41<br>(1.2 to 1.69)	15.35<br>(13.1 to 18.35)	3.84<br>(2.95 to 4.73)	25.18<br>(19.34 to 31)
Tibet	Multiple myeloma	0.247<br>(0.19 to 0.423)	10.6<br>(8.16 to 18.16)	0.391<br>(0.316 to 0.541)	11.31<br>(9.15 to 15.66)
Xinjiang	Multiple myeloma	2.01<br>(1.5 to 2.39)	12.77<br>(9.51 to 15.15)	5.22<br>(3.82 to 6.62)	21.42<br>(15.68 to 27.16)
Yunnan	Multiple myeloma	4.58<br>(3.74 to 5.69)	11.75<br>(9.59 to 14.6)	8.11<br>(6.71 to 9.95)	16.49<br>(13.65 to 20.24)
Zhejiang	Multiple myeloma	9.23<br>(4.93 to 11.1)	20.93<br>(11.18 to 25.08)	19.7<br>(10.9 to 25.8)	32.2<br>(17.82 to 42.22)
China	Leukemia	3870<br>(2740 to 4440)	323.49<br>(228.52 to 370.56)	2300<br>(1930 to 2480)	163.16<br>(136.89 to 175.37)
Taiwan	Leukemia	28.3<br>(27.2 to 29.4)	138.48<br>(133.32 to 144.06)	31<br>(28.7 to 33.2)	131.6<br>(121.52 to 140.77)
Hong Kong Special Administrative Region of China	Leukemia	7.89<br>(6.86 to 9.93)	136.93<br>(119.09 to 172.21)	8.78<br>(6.64 to 11.4)	116.95<br>(88.41 to 152.43)
Macau Special Administrative Region of China	Leukemia	0.458<br>(0.379 to 0.649)	129.09<br>(106.67 to 182.87)	0.632<br>(0.404 to 0.962)	92.43<br>(59.11 to 140.68)
Anhui	Leukemia	249<br>(150 to 307)	419.51<br>(253.53 to 518.84)	108<br>(76.9 to 129)	175.9<br>(125.7 to 210.43)

Beijing	Leukemia	25.7<br>(19.8 to 30.7)	229.54<br>(176.45 to 273.45)	26.9<br>(17.8 to 33.1)	111.7<br>(74.03 to 137.46)
Chongqing	Leukemia	53.8<br>(32.3 to 68.8)	340.97<br>(204.55 to 435.79)	36.9<br>(29.7 to 45.4)	138.24<br>(111.29 to 169.94)
Fujian	Leukemia	98.5<br>(64.8 to 118)	311.3<br>(204.79 to 373.7)	63.6<br>(45 to 77.5)	161.41<br>(114.12 to 196.7)
Gansu	Leukemia	113<br>(73.5 to 139)	476.36<br>(310.74 to 587.45)	45.3<br>(37.6 to 53.2)	170.44<br>(141.47 to 200.21)
Guangdong	Leukemia	195<br>(133 to 238)	296.12<br>(201.96 to 361.5)	161<br>(126 to 198)	137.55<br>(107.85 to 169.23)
Guangxi	Leukemia	142<br>(103 to 181)	326.67<br>(236.83 to 415.83)	88.3<br>(72.1 to 108)	182.7<br>(149.2 to 222.69)
Guizhou	Leukemia	131<br>(84.7 to 195)	393.64<br>(255.25 to 587.56)	55.1<br>(44.4 to 69.5)	162.83<br>(131.33 to 205.4)
Hainan	Leukemia	20.4<br>(12.3 to 27.5)	293.44<br>(176.26 to 395.26)	15.2<br>(12.1 to 18.4)	158.27<br>(126.01 to 192.16)
Hebei	Leukemia	179<br>(147 to 210)	281.18<br>(231.43 to 330.2)	152<br>(110 to 181)	198.32<br>(143.66 to 236.92)
Heilongjiang	Leukemia	106<br>(86.8 to 129)	289.53<br>(238.24 to 353.66)	59.3<br>(49.4 to 76.5)	151.02<br>(125.86 to 194.97)
Henan	Leukemia	241<br>(200 to 300)	272.64<br>(226.52 to 339.6)	153<br>(128 to 185)	158.73<br>(133.12 to 192.35)
Hubei	Leukemia	138<br>(112 to 183)	248.48<br>(201.14 to 329.44)	91.7<br>(74.5 to 108)	165.85<br>(134.78 to 194.57)
Hunan	Leukemia	214<br>(140 to 276)	343.75<br>(225.27 to 443.25)	130<br>(97.4 to 157)	193.35<br>(145.04 to 233.08)
Inner Mongolia	Leukemia	96.4<br>(58.8 to 120)	423.82<br>(258.4 to 528.6)	41.4<br>(33.9 to 49.5)	159.81<br>(130.98 to 191.12)
Jiangsu	Leukemia	180<br>(132 to 210)	261.99<br>(192.02 to 305.45)	131<br>(86.5 to 159)	160<br>(105.94 to 194.49)
Jiangxi	Leukemia	203<br>(96.9 to 264)	513.58<br>(245.2 to 666.66)	85.3<br>(67.1 to 100)	179.49<br>(141.05 to 210.92)
Jilin	Leukemia	85<br>(62 to 105)	327.38<br>(238.75 to 403.85)	43.1<br>(34.6 to 52)	154.38<br>(123.91 to 186.43)
Liaoning	Leukemia	118<br>(85 to 147)	290.46<br>(208.39 to 359.33)	79.8<br>(59.2 to 96.5)	178.69<br>(132.59 to 216.21)
Ningxia	Leukemia	22.5<br>(12.3 to 30.2)	455<br>(248.3 to 612.26)	10.7<br>(8.6 to 12.7)	151.95<br>(122.47 to 181.16)
Qinghai	Leukemia	20.8<br>(13.1 to 32.1)	439.44<br>(277.03 to 676.74)	10.5<br>(8.44 to 15.1)	167.38<br>(134.36 to 240.18)
Shaanxi	Leukemia	95.9<br>(73.5 to 115)	278.68<br>(213.6 to 334.63)	61.1<br>(49.3 to 74.3)	154.6<br>(124.77 to 187.95)
Shandong	Leukemia	250<br>(186 to 300)	292.34<br>(217.45 to 349.97)	161<br>(126 to 191)	161.67<br>(127.02 to 191.99)
Shanghai	Leukemia	28.4<br>(21.6 to 33.3)	203.33<br>(154.69 to 238.4)	34.9<br>(20.7 to 43)	124.79<br>(74.08 to 153.79)
Shanxi	Leukemia	97.4<br>(66.7 to 118)	321.52<br>(220.36 to 389.32)	56.1<br>(45.2 to 67.4)	145.14<br>(117.09 to 174.5)
Sichuan	Leukemia	356<br>(227 to 455)	318.69<br>(203.29 to 407.7)	146<br>(115 to 176)	174.31<br>(137.63 to 210.29)
Tianjin	Leukemia	23.9<br>(17.8 to 28.5)	260.26<br>(193.26 to 309.35)	26.7<br>(18.8 to 32.8)	174.97<br>(123 to 215.08)
Tibet	Leukemia	7.55<br>(5.58 to 17.6)	323.88<br>(239.64 to 753.68)	5.73<br>(3.66 to 9.33)	165.79<br>(105.83 to 269.86)
Xinjiang	Leukemia	61.6<br>(41 to 90.7)	390.57<br>(259.9 to 574.9)	44.6<br>(35.5 to 59.4)	182.9<br>(145.76 to 243.83)
Yunnan	Leukemia	189<br>(101 to 260)	485.51<br>(260.26 to 666.1)	90.2<br>(75.5 to 111)	183.48<br>(153.57 to 226.4)
Zhejiang	Leukemia	123<br>(78 to 153)	279.15<br>(176.93 to 345.99)	83.6<br>(56.2 to 102)	136.6<br>(91.92 to 166.72)
China	Other malignant neoplasms	1600<br>(1310 to 1710)	133.49<br>(109.41 to 142.78)	2020<br>(1750 to 2220)	143.24<br>(123.9 to 157.11)
Taiwan	Other malignant neoplasms	24.6<br>(21.8 to 25.8)	120.41<br>(106.95 to 126.51)	39.3<br>(34 to 44.2)	166.76<br>(144.01 to 187.29)
Hong Kong Special Administrative Region of China	Other malignant neoplasms	5.14<br>(4.57 to 6.05)	89.18<br>(79.24 to 104.97)	8.73<br>(7.13 to 11.1)	116.24<br>(94.89 to 147.59)

Macau Special Administrative Region of China	Other malignant neoplasms	0.287<br>(0.24 to 0.357)	80.87<br>(67.65 to 100.58)	0.559<br>(0.378 to 0.872)	81.73<br>(55.31 to 127.46)
Anhui	Other malignant neoplasms	97.8<br>(73.4 to 118)	165.11<br>(123.94 to 199.17)	86.2<br>(63.9 to 105)	141.03<br>(104.49 to 171.42)
Beijing	Other malignant neoplasms	14.4<br>(11.8 to 16.8)	128.54<br>(104.82 to 149.99)	27<br>(19.9 to 37.3)	111.92<br>(82.38 to 154.87)
Chongqing	Other malignant neoplasms	18.7<br>(15.3 to 23.3)	118.41<br>(97.17 to 147.59)	28.8<br>(22.4 to 38.4)	107.66<br>(83.64 to 143.52)
Fujian	Other malignant neoplasms	37.4<br>(30.2 to 42.9)	118.23<br>(95.3 to 135.54)	50.4<br>(40.6 to 61.8)	127.95<br>(103.11 to 156.92)
Gansu	Other malignant neoplasms	39.3<br>(29.5 to 45.6)	166.14<br>(124.88 to 192.66)	32.3<br>(27.4 to 38.2)	121.56<br>(103.04 to 143.55)
Guangdong	Other malignant neoplasms	81.6<br>(61 to 97.5)	123.99<br>(92.63 to 148.13)	190<br>(139 to 259)	163.01<br>(119.19 to 221.32)
Guangxi	Other malignant neoplasms	56.6<br>(44.3 to 66.5)	130.07<br>(101.89 to 152.83)	66.5<br>(52 to 80.7)	137.69<br>(107.71 to 167.08)
Guizhou	Other malignant neoplasms	49.3<br>(37.2 to 60.9)	148.67<br>(112 to 183.37)	39.7<br>(32.7 to 47.6)	117.38<br>(96.64 to 140.79)
Hainan	Other malignant neoplasms	6.89<br>(5.62 to 8.76)	98.89<br>(80.61 to 125.69)	9.55<br>(7.73 to 12)	99.61<br>(80.67 to 124.87)
Hebei	Other malignant neoplasms	82.5<br>(62.9 to 96.9)	129.66<br>(98.86 to 152.29)	129<br>(88 to 168)	169.24<br>(115.14 to 220.15)
Heilongjiang	Other malignant neoplasms	54.9<br>(42.9 to 64)	150.64<br>(117.65 to 175.63)	74<br>(54.1 to 92.5)	188.45<br>(137.89 to 235.77)
Henan	Other malignant neoplasms	94.1<br>(83.4 to 108)	106.57<br>(94.44 to 121.79)	122<br>(102 to 145)	126.98<br>(106.09 to 150.86)
Hubei	Other malignant neoplasms	63.1<br>(52.9 to 71.5)	113.6<br>(95.22 to 128.77)	87.7<br>(71 to 110)	158.54<br>(128.38 to 198.33)
Hunan	Other malignant neoplasms	93.9<br>(69.9 to 108)	151.02<br>(112.36 to 173.2)	104<br>(82 to 126)	154.27<br>(122.06 to 187.3)
Inner Mongolia	Other malignant neoplasms	36<br>(25.7 to 43)	158.28<br>(113.07 to 189.01)	40.9<br>(31.8 to 50.9)	158.13<br>(122.67 to 196.53)
Jiangsu	Other malignant neoplasms	88.1<br>(72.6 to 100)	127.97<br>(105.5 to 145.62)	126<br>(90.7 to 154)	154<br>(111.06 to 188.62)
Jiangxi	Other malignant neoplasms	68.8<br>(39.8 to 83.6)	173.98<br>(100.66 to 211.46)	55.3<br>(46 to 65.5)	116.43<br>(96.7 to 137.85)
Jilin	Other malignant neoplasms	40.7<br>(28 to 49.2)	156.88<br>(107.89 to 189.5)	47.3<br>(32.8 to 59.6)	169.47<br>(117.52 to 213.82)
Liaoning	Other malignant neoplasms	56.9<br>(46.3 to 66.9)	139.57<br>(113.4 to 164.12)	89.5<br>(64.7 to 112)	200.5<br>(144.83 to 251.2)
Ningxia	Other malignant neoplasms	8.15<br>(5 to 10.5)	164.98<br>(101.17 to 212.78)	7.85<br>(6.2 to 9.74)	111.87<br>(88.32 to 138.83)
Qinghai	Other malignant neoplasms	8.02<br>(4.8 to 10.1)	169.23<br>(101.28 to 212.31)	7.77<br>(6.1 to 9.4)	123.7<br>(97.09 to 149.7)
Shaanxi	Other malignant neoplasms	37.6<br>(31.2 to 43.8)	109.37<br>(90.54 to 127.4)	47.2<br>(38.3 to 56.7)	119.34<br>(97 to 143.57)
Shandong	Other malignant neoplasms	110<br>(91.5 to 127)	128.55<br>(106.86 to 148.29)	142<br>(116 to 172)	143.38<br>(116.85 to 172.68)
Shanghai	Other malignant neoplasms	18.5<br>(13.9 to 21.9)	132.36<br>(99.37 to 156.78)	36.1<br>(23.3 to 46.8)	129.36<br>(83.56 to 167.55)
Shanxi	Other malignant neoplasms	42.5<br>(32.7 to 50.6)	140.41<br>(108.02 to 167.23)	49.8<br>(37.7 to 61.7)	128.99<br>(97.68 to 159.73)
Sichuan	Other malignant neoplasms	138<br>(113 to 158)	123.55<br>(101.6 to 141.28)	116<br>(94.2 to 137)	138.47<br>(112.86 to 164.09)
Tianjin	Other malignant neoplasms	12.5<br>(9.99 to 14.6)	136.16<br>(108.62 to 158.89)	34<br>(24.4 to 47.8)	222.94<br>(159.63 to 313.2)
Tibet	Other malignant neoplasms	3.04<br>(2.3 to 5.02)	130.58<br>(98.49 to 215.55)	2.97<br>(2.31 to 4.43)	85.82<br>(66.78 to 128.26)
Xinjiang	Other malignant neoplasms	22<br>(15.1 to 29.1)	139.69<br>(95.52 to 184.37)	29.3<br>(22.7 to 36.8)	120.34<br>(93.04 to 150.9)
Yunnan	Other malignant neoplasms	60.3<br>(43.6 to 78)	154.8<br>(111.86 to 200.06)	54.3<br>(44.7 to 68.7)	110.52<br>(90.95 to 139.79)
Zhejiang	Other malignant neoplasms	50.6<br>(39.3 to 58.1)	114.83<br>(89.21 to 131.78)	79.8<br>(61.2 to 112)	130.42<br>(100.09 to 183.48)
China	Other neoplasms	206<br>(140 to 243)	17.23<br>(11.7 to 20.28)	387<br>(329 to 561)	27.43<br>(23.27 to 39.73)
Taiwan	Other neoplasms	1.43<br>(1 to 2.34)	7<br>(4.93 to 11.49)	6.89<br>(3.25 to 10.1)	29.21<br>(13.79 to 42.66)

Hong Kong Special Administrative Region of China	Other neoplasms	1.35 (1.1 to 1.73)	23.5 (19.16 to 29.94)	2.44 (1.58 to 4.14)	32.5 (20.97 to 55.09)
Macau Special Administrative Region of China	Other neoplasms	0.0898 (0.0693 to 0.109)	25.32 (19.53 to 30.79)	0.213 (0.148 to 0.291)	31.08 (21.71 to 42.61)
Anhui	Other neoplasms	10.4 (5.39 to 14)	17.49 (9.1 to 23.67)	13.8 (10.6 to 18.2)	22.59 (17.39 to 29.71)
Beijing	Other neoplasms	3.43 (2.25 to 4.52)	30.59 (20.09 to 40.32)	8.53 (6.69 to 12)	35.41 (27.78 to 49.66)
Chongqing	Other neoplasms	2.27 (1.55 to 2.89)	14.4 (9.81 to 18.34)	6.19 (4.66 to 9.07)	23.14 (17.43 to 33.95)
Fujian	Other neoplasms	6.49 (2.86 to 10.1)	20.5 (9.04 to 31.96)	12.7 (8.81 to 16)	32.17 (22.36 to 40.55)
Gansu	Other neoplasms	5.63 (2.65 to 8.21)	23.79 (11.19 to 34.7)	6.53 (4.62 to 8.04)	24.58 (17.37 to 30.24)
Guangdong	Other neoplasms	10.3 (7.24 to 12.8)	15.59 (11 to 19.52)	29.8 (21.5 to 48)	25.5 (18.41 to 41.05)
Guangxi	Other neoplasms	6.6 (4.68 to 8.05)	15.17 (10.77 to 18.52)	13.4 (9.88 to 22.3)	27.77 (20.45 to 46.06)
Guizhou	Other neoplasms	5.09 (3.11 to 6.92)	15.33 (9.38 to 20.84)	7.59 (5.52 to 9.72)	22.44 (16.32 to 28.73)
Hainan	Other neoplasms	1.75 (0.668 to 2.76)	25.08 (9.59 to 39.64)	3.91 (1.88 to 5.81)	40.77 (19.64 to 60.63)
Hebei	Other neoplasms	8.61 (6.23 to 11.4)	13.53 (9.78 to 17.95)	21 (14.8 to 38.5)	27.48 (19.38 to 50.36)
Heilongjiang	Other neoplasms	6.92 (5.17 to 8.52)	18.97 (14.19 to 23.36)	11.8 (8.85 to 19.1)	30.13 (22.55 to 48.59)
Henan	Other neoplasms	15.3 (8.48 to 20.5)	17.31 (9.61 to 23.22)	27.5 (21.8 to 38.8)	28.59 (22.64 to 40.33)
Hubei	Other neoplasms	6.78 (5.36 to 9.69)	12.2 (9.65 to 17.45)	14.5 (9.79 to 25.2)	26.31 (17.7 to 45.5)
Hunan	Other neoplasms	10.2 (7.7 to 12.4)	16.45 (12.38 to 19.99)	20.4 (14.9 to 33.1)	30.37 (22.15 to 49.25)
Inner Mongolia	Other neoplasms	4.39 (2.94 to 5.58)	19.3 (12.95 to 24.52)	7.35 (5.56 to 11.4)	28.37 (21.46 to 44)
Jiangsu	Other neoplasms	10.3 (7.72 to 13.4)	14.99 (11.22 to 19.46)	23.1 (16.1 to 37.9)	28.32 (19.68 to 46.38)
Jiangxi	Other neoplasms	8.54 (4.53 to 12.6)	21.61 (11.47 to 31.97)	11.5 (8.73 to 14.4)	24.27 (18.36 to 30.24)
Jilin	Other neoplasms	4.73 (3.31 to 5.89)	18.23 (12.74 to 22.7)	7.78 (5.68 to 11.9)	27.91 (20.38 to 42.6)
Liaoning	Other neoplasms	8.25 (6.1 to 10.2)	20.22 (14.95 to 24.95)	16.1 (11.8 to 26.4)	36.16 (26.46 to 59.04)
Ningxia	Other neoplasms	0.992 (0.538 to 1.43)	20.09 (10.9 to 28.95)	1.43 (1.11 to 1.86)	20.37 (15.84 to 26.45)
Qinghai	Other neoplasms	0.985 (0.548 to 1.37)	20.8 (11.57 to 28.87)	1.45 (1.07 to 2.18)	23.06 (16.97 to 34.72)
Shaanxi	Other neoplasms	5.57 (2.76 to 8.63)	16.2 (8.03 to 25.08)	10.5 (8.05 to 13.7)	26.46 (20.38 to 34.56)
Shandong	Other neoplasms	14.2 (10.7 to 17.4)	16.59 (12.54 to 20.31)	29.4 (20.4 to 50.2)	29.63 (20.56 to 50.46)
Shanghai	Other neoplasms	3.67 (2.48 to 4.88)	26.3 (17.78 to 34.93)	8.25 (6.28 to 11.9)	29.53 (22.48 to 42.62)
Shanxi	Other neoplasms	5.04 (3.49 to 6.19)	16.64 (11.53 to 20.44)	8.96 (6.79 to 13.3)	23.19 (17.58 to 34.54)
Sichuan	Other neoplasms	18.1 (10.5 to 24.8)	16.24 (9.41 to 22.24)	21.7 (17.1 to 30.7)	25.96 (20.53 to 36.81)
Tianjin	Other neoplasms	1.73 (1.37 to 2.58)	18.83 (14.84 to 28)	4.67 (2.78 to 9.23)	30.61 (18.2 to 60.5)
Tibet	Other neoplasms	0.499 (0.188 to 0.807)	21.42 (8.07 to 34.62)	0.691 (0.375 to 0.961)	20 (10.86 to 27.82)
Xinjiang	Other neoplasms	3.51 (2 to 4.85)	22.25 (12.67 to 30.73)	7.46 (5.39 to 12.3)	30.62 (22.12 to 50.3)
Yunnan	Other neoplasms	7.58 (4 to 10.9)	19.44 (10.27 to 27.92)	11.5 (8.7 to 15.1)	23.47 (17.69 to 30.76)
Zhejiang	Other neoplasms	7.01 (4.62 to 8.79)	15.89 (10.49 to 19.92)	15.1 (11.6 to 22.4)	24.72 (18.91 to 36.55)

China	Cardiovascular diseases	54200 (52400 to 56500)	4527.91 (4380.66 to 4720.81)	85000 (81500 to 89000)	6020.67 (5767.56 to 6297.61)
Taiwan	Cardiovascular diseases	754 (729 to 782)	3693.53 (3572.16 to 3834.3)	818 (759 to 880)	3470.5 (3216.9 to 3729.89)
Hong Kong Special Administrative Region of China	Cardiovascular diseases	175 (161 to 190)	3030.87 (2795.37 to 3295.55)	241 (210 to 276)	3215.15 (2799.64 to 3673.8)
Macau Special Administrative Region of China	Cardiovascular diseases	11.4 (10.7 to 12.1)	3207.28 (3023.83 to 3395.92)	16.3 (14.3 to 18.4)	2388.62 (2087.76 to 2687.1)
Anhui	Cardiovascular diseases	2490 (2260 to 2750)	4199.86 (3808.35 to 4640.63)	3710 (3250 to 4230)	6068.73 (5320.61 to 6917.72)
Beijing	Cardiovascular diseases	548 (486 to 613)	4884.38 (4337.02 to 5469.14)	829 (711 to 959)	3439.34 (2948.22 to 3980.22)
Chongqing	Cardiovascular diseases	648 (580 to 734)	4104.94 (3672.12 to 4648.8)	1450 (1220 to 1720)	5408.29 (4549.31 to 6437.05)
Fujian	Cardiovascular diseases	910 (837 to 1000)	2875.86 (2645.58 to 3172.46)	1360 (1180 to 1570)	3456.99 (2992.08 to 3972.05)
Gansu	Cardiovascular diseases	1190 (1100 to 1300)	5035.8 (4632.55 to 5500.04)	1700 (1480 to 1960)	6407.4 (5552.07 to 7374.66)
Guangdong	Cardiovascular diseases	2580 (2270 to 2920)	3921.99 (3449.76 to 4433.83)	4090 (3540 to 4700)	3498.01 (3032.86 to 4026.13)
Guangxi	Cardiovascular diseases	1840 (1630 to 2130)	4241.24 (3757.94 to 4887.86)	2920 (2530 to 3400)	6041.07 (5237.81 to 7027.98)
Guizhou	Cardiovascular diseases	1740 (1540 to 2000)	5250.43 (4654.61 to 6024.01)	2270 (1930 to 2610)	6711.74 (5700.17 to 7728.05)
Hainan	Cardiovascular diseases	271 (241 to 303)	3888.13 (3466.84 to 4355.91)	372 (313 to 440)	3874.92 (3265.38 to 4593.08)
Hebei	Cardiovascular diseases	3350 (3110 to 3590)	5269.17 (4889.27 to 5634.38)	6700 (5810 to 7660)	8761.93 (7600.06 to 10016.51)
Heilongjiang	Cardiovascular diseases	2140 (1960 to 2320)	5869.28 (5387.92 to 6365.28)	3710 (3160 to 4290)	9441.15 (8047.22 to 10940.77)
Henan	Cardiovascular diseases	4170 (3890 to 4460)	4729.17 (4409.92 to 5053.55)	7340 (6410 to 8410)	7625.16 (6652.84 to 8728.29)
Hubei	Cardiovascular diseases	2700 (2510 to 2890)	4864.89 (4519.65 to 5197.77)	3960 (3440 to 4530)	7168.87 (6222.95 to 8190.91)
Hunan	Cardiovascular diseases	3460 (3210 to 3790)	5566.82 (5159.58 to 6102.46)	5080 (4430 to 5810)	7569.66 (6593.79 to 8651.22)
Inner Mongolia	Cardiovascular diseases	1360 (1220 to 1520)	5981.46 (5349.77 to 6666.46)	1940 (1690 to 2230)	7502.19 (6523.03 to 8620.94)
Jiangsu	Cardiovascular diseases	2300 (2110 to 2520)	3341.3 (3060.14 to 3664.8)	3760 (3290 to 4300)	4607.63 (4023.39 to 5262.77)
Jiangxi	Cardiovascular diseases	2110 (1960 to 2330)	5347.81 (4954.81 to 5894.48)	2370 (2070 to 2700)	4978.22 (4364.29 to 5676.48)
Jilin	Cardiovascular diseases	1580 (1480 to 1690)	6095.04 (5701.94 to 6507.07)	2240 (1870 to 2590)	8040.2 (6714.84 to 9298.38)
Liaoning	Cardiovascular diseases	2230 (1990 to 2480)	5464.23 (4875.72 to 6086.08)	4010 (3450 to 4660)	8987.99 (7718.28 to 10432.84)
Ningxia	Cardiovascular diseases	189 (170 to 210)	3833.74 (3435.96 to 4254.65)	330 (277 to 392)	4695.85 (3950.68 to 5580.62)
Qinghai	Cardiovascular diseases	208 (186 to 236)	4400.17 (3924.93 to 4983.87)	370 (311 to 428)	5896.35 (4955.31 to 6812.57)
Shaanxi	Cardiovascular diseases	922 (849 to 1010)	2678.18 (2468.18 to 2921.33)	2550 (2140 to 3020)	6442.29 (5411.96 to 7653.47)
Shandong	Cardiovascular diseases	4400 (3970 to 4880)	5143.02 (4632.96 to 5700.7)	6600 (5690 to 7590)	6644.52 (5729.33 to 7634.87)
Shanghai	Cardiovascular diseases	451 (402 to 506)	3231.4 (2874.74 to 3618.97)	708 (608 to 816)	2535.21 (2175.25 to 2919.54)
Shanxi	Cardiovascular diseases	1690 (1530 to 1850)	5583.6 (5062.04 to 6122.81)	2190 (1820 to 2570)	5660.24 (4716.63 to 6653.68)
Sichuan	Cardiovascular diseases	3700 (3410 to 4120)	3315.65 (3054.44 to 3687.06)	4810 (4110 to 5510)	5766.03 (4926.56 to 6594.56)
Tianjin	Cardiovascular diseases	526 (469 to 581)	5714.06 (5095.28 to 6317.58)	938 (810 to 1080)	6148.17 (5308.05 to 7107.98)
Tibet	Cardiovascular diseases	227 (202 to 253)	9738.66 (8668.76 to 10868.64)	227 (194 to 266)	6572.45 (5612.79 to 7684.65)
Xinjiang	Cardiovascular diseases	712 (657 to 773)	4511.92 (4162.81 to 4899.96)	1550 (1340 to 1800)	6367.29 (5492.78 to 7385.1)

Yunnan	Cardiovascular diseases	1800 (1580 to 2130)	4627.25 (4057.87 to 5460.2)	2790 (2430 to 3250)	5681.11 (4945.79 to 6612.32)
Zhejiang	Cardiovascular diseases	1540 (1420 to 1720)	3490.6 (3212.34 to 3907.12)	1900 (1640 to 2180)	3099.64 (2685.13 to 3570.49)
China	Rheumatic heart disease	3980 (3740 to 4330)	332.89 (312.59 to 362.1)	1810 (1630 to 2020)	127.94 (115.6 to 143.13)
Taiwan	Rheumatic heart disease	17.6 (16.9 to 18.3)	86.28 (82.88 to 89.65)	6.34 (5.83 to 6.91)	26.87 (24.7 to 29.3)
Hong Kong Special Administrative Region of China	Rheumatic heart disease	4.85 (4.39 to 5.43)	84.15 (76.14 to 94.25)	3.68 (3.07 to 4.39)	48.98 (40.86 to 58.42)
Macau Special Administrative Region of China	Rheumatic heart disease	0.481 (0.432 to 0.537)	135.57 (121.81 to 151.35)	0.299 (0.236 to 0.377)	43.66 (34.46 to 55.14)
Anhui	Rheumatic heart disease	145 (128 to 162)	245.38 (215.49 to 272.81)	65 (54.5 to 77.5)	106.37 (89.11 to 126.72)
Beijing	Rheumatic heart disease	15.7 (13.6 to 18.1)	140.03 (120.93 to 161.75)	5.96 (4.97 to 6.97)	24.71 (20.64 to 28.93)
Chongqing	Rheumatic heart disease	98.3 (84.9 to 114)	622.97 (537.73 to 725.1)	60.3 (50.8 to 72)	225.75 (190.14 to 269.23)
Fujian	Rheumatic heart disease	63.5 (55.8 to 72.4)	200.61 (176.33 to 228.93)	20.6 (17.3 to 24.5)	52.21 (43.96 to 62.21)
Gansu	Rheumatic heart disease	97.7 (87.8 to 110)	413.25 (371.27 to 463.47)	41.8 (35.7 to 49.3)	157.12 (134.23 to 185.5)
Guangdong	Rheumatic heart disease	175 (147 to 203)	265.15 (224.02 to 309.07)	66.2 (56.5 to 78.3)	56.63 (48.39 to 67.03)
Guangxi	Rheumatic heart disease	209 (179 to 245)	479.55 (411.8 to 562.59)	84.2 (72.1 to 98.8)	174.32 (149.24 to 204.55)
Guizhou	Rheumatic heart disease	264 (226 to 310)	793.9 (682.07 to 935.17)	105 (88.8 to 125)	311.82 (262.54 to 368.26)
Hainan	Rheumatic heart disease	30.4 (26.2 to 36.1)	436.79 (375.93 to 518.54)	15.8 (13.1 to 18.8)	164.45 (136.11 to 195.83)
Hebei	Rheumatic heart disease	166 (149 to 184)	260.7 (234.43 to 289.22)	110 (93 to 129)	144.17 (121.74 to 168.73)
Heilongjiang	Rheumatic heart disease	82.2 (71.4 to 95)	225.61 (195.79 to 260.65)	45.6 (37.9 to 55.1)	116.11 (96.5 to 140.38)
Henan	Rheumatic heart disease	174 (156 to 195)	197.47 (176.75 to 220.62)	92.1 (75.8 to 111)	95.62 (78.73 to 114.89)
Hubei	Rheumatic heart disease	190 (171 to 215)	341.95 (307.79 to 387.23)	91 (76.8 to 106)	164.49 (138.79 to 191.41)
Hunan	Rheumatic heart disease	412 (364 to 481)	661.98 (585.82 to 772.75)	168 (145 to 195)	249.69 (215.29 to 290.8)
Inner Mongolia	Rheumatic heart disease	70 (61 to 79.7)	307.61 (268.24 to 350.38)	28.6 (23.6 to 34.3)	110.54 (91.24 to 132.61)
Jiangsu	Rheumatic heart disease	92.5 (82.8 to 103)	134.47 (120.37 to 150.15)	44.7 (37.4 to 52.4)	54.78 (45.78 to 64.17)
Jiangxi	Rheumatic heart disease	209 (185 to 240)	527.91 (468.24 to 608.34)	67.8 (57.9 to 79.4)	142.69 (121.88 to 166.97)
Jilin	Rheumatic heart disease	64.3 (56.7 to 73.2)	247.72 (218.33 to 282.14)	31.5 (26 to 37.6)	112.87 (93.26 to 134.87)
Liaoning	Rheumatic heart disease	70.9 (60.1 to 83.4)	173.72 (147.38 to 204.41)	45.6 (37 to 55.2)	102.08 (82.82 to 123.62)
Ningxia	Rheumatic heart disease	14.2 (12.3 to 16.6)	288.32 (249.38 to 335.65)	6.95 (5.67 to 8.41)	99.06 (80.73 to 119.76)
Qinghai	Rheumatic heart disease	20.7 (17.7 to 23.9)	437.17 (372.79 to 505.61)	9.93 (8.34 to 11.9)	158.02 (132.7 to 189.9)
Shaanxi	Rheumatic heart disease	61.5 (54.4 to 69.6)	178.76 (157.95 to 202.15)	48.6 (40.1 to 58)	123.04 (101.4 to 146.74)
Shandong	Rheumatic heart disease	194 (171 to 222)	226.99 (199.63 to 259.2)	91.9 (75.6 to 113)	92.52 (76.02 to 113.37)
Shanghai	Rheumatic heart disease	11.9 (10.2 to 13.8)	85.14 (73.01 to 98.91)	5.37 (4.41 to 6.47)	19.22 (15.78 to 23.14)
Shanxi	Rheumatic heart disease	92.8 (81.9 to 106)	306.55 (270.35 to 349.73)	41.5 (33.9 to 50.2)	107.38 (87.75 to 129.91)
Sichuan	Rheumatic heart disease	497 (445 to 566)	445.48 (398.66 to 507.16)	206 (175 to 239)	246.53 (209.7 to 286.67)
Tianjin	Rheumatic heart disease	18.9 (16.2 to 21.9)	205.65 (176.08 to 237.76)	12.4 (9.79 to 15.6)	81.31 (64.11 to 102.38)

Tibet	Rheumatic heart disease	20.7<br>(17.7 to 25.2)	886.32<br>(761.4 to 1081.82)	7.87<br>(6.53 to 9.65)	227.83<br>(189.06 to 279.29)
Xinjiang	Rheumatic heart disease	75.3<br>(66.7 to 85.6)	477.32<br>(422.69 to 542.71)	48.6<br>(41.2 to 57.7)	199.48<br>(168.88 to 236.68)
Yunnan	Rheumatic heart disease	268<br>(230 to 316)	687.16<br>(590.64 to 811.81)	113<br>(97.2 to 132)	229.85<br>(197.62 to 268.64)
Zhejiang	Rheumatic heart disease	75.9<br>(66.7 to 87.7)	172.15<br>(151.24 to 198.91)	21.1<br>(17.6 to 25.4)	34.54<br>(28.8 to 41.57)
China	Ischemic heart disease	13400<br>(12800 to 14300)	1116.45<br>(1071.82 to 1194.84)	30100<br>(29000 to 31200)	2131.45<br>(2054.18 to 2211.03)
Taiwan	Ischemic heart disease	222<br>(215 to 230)	1087.64<br>(1053.56 to 1125.89)	293<br>(277 to 311)	1241.57<br>(1175.1 to 1318.56)
Hong Kong Special Administrative Region of China	Ischemic heart disease	72.4<br>(66.1 to 82.1)	1256.35<br>(1147.03 to 1424.4)	99.9<br>(86.1 to 117)	1330.4<br>(1146.41 to 1559.17)
Macau Special Administrative Region of China	Ischemic heart disease	3.94<br>(3.63 to 4.34)	1110.75<br>(1023.32 to 1223.77)	5.4<br>(4.42 to 6.48)	789.67<br>(646.57 to 947.02)
Anhui	Ischemic heart disease	671<br>(592 to 771)	1132.79<br>(999.45 to 1300.94)	1180<br>(1020 to 1360)	1928.68<br>(1667.85 to 2221.56)
Beijing	Ischemic heart disease	160<br>(141 to 183)	1427.89<br>(1254.68 to 1632.6)	336<br>(281 to 395)	1395.66<br>(1167.1 to 1640.38)
Chongqing	Ischemic heart disease	138<br>(121 to 161)	875.91<br>(763.56 to 1021.87)	414<br>(345 to 494)	1547.5<br>(1291.71 to 1848.97)
Fujian	Ischemic heart disease	208<br>(188 to 236)	657.05<br>(594.23 to 747.23)	426<br>(363 to 502)	1080.96<br>(920.4 to 1272.77)
Gansu	Ischemic heart disease	278<br>(247 to 316)	1174.33<br>(1045.91 to 1335.74)	594<br>(502 to 689)	2233.58<br>(1888.23 to 2593.57)
Guangdong	Ischemic heart disease	673<br>(583 to 778)	1022.13<br>(885.1 to 1182.57)	1640<br>(1400 to 1910)	1399.92<br>(1199.97 to 1633.74)
Guangxi	Ischemic heart disease	429<br>(373 to 520)	986.1<br>(857.56 to 1195.03)	986<br>(838 to 1170)	2040.41<br>(1733.24 to 2420.09)
Guizhou	Ischemic heart disease	295<br>(256 to 350)	889.01<br>(771.01 to 1055.69)	538<br>(450 to 632)	1590.97<br>(1330.72 to 1869.14)
Hainan	Ischemic heart disease	67.5<br>(58.8 to 77.2)	968.41<br>(844.08 to 1108.13)	119<br>(97.9 to 145)	1238.75<br>(1020.75 to 1514.55)
Hebei	Ischemic heart disease	803<br>(724 to 897)	1261.17<br>(1137.72 to 1410.1)	2210<br>(1880 to 2560)	2890.56<br>(2458.94 to 3344.78)
Heilongjiang	Ischemic heart disease	700<br>(624 to 770)	1920.74<br>(1710.84 to 2112.03)	1590<br>(1340 to 1870)	4062.64<br>(3415.45 to 4768.74)
Henan	Ischemic heart disease	1000<br>(922 to 1100)	1136.06<br>(1044.07 to 1243.19)	2810<br>(2410 to 3280)	2914.73<br>(2504.29 to 3410.58)
Hubei	Ischemic heart disease	536<br>(481 to 622)	964.45<br>(864.9 to 1120.17)	1250<br>(1070 to 1450)	2257.58<br>(1935.49 to 2620.02)
Hunan	Ischemic heart disease	726<br>(650 to 866)	1168.27<br>(1045.33 to 1392.2)	1800<br>(1550 to 2120)	2687.25<br>(2310.6 to 3150)
Inner Mongolia	Ischemic heart disease	385<br>(337 to 436)	1692.5<br>(1480.54 to 1916.14)	847<br>(723 to 984)	3272.56<br>(2791.35 to 3799.48)
Jiangsu	Ischemic heart disease	550<br>(495 to 613)	799.63<br>(719.38 to 890.56)	1020<br>(873 to 1170)	1244.86<br>(1069.05 to 1436.9)
Jiangxi	Ischemic heart disease	482<br>(435 to 560)	1219.76<br>(1099.2 to 1415.94)	760<br>(653 to 894)	1599.74<br>(1373.72 to 1880.06)
Jilin	Ischemic heart disease	500<br>(457 to 545)	1927.92<br>(1760.59 to 2099.48)	948<br>(789 to 1110)	3398.74<br>(2827.38 to 3963.53)
Liaoning	Ischemic heart disease	673<br>(580 to 759)	1650.45<br>(1421.01 to 1861.08)	1660<br>(1400 to 1950)	3725.97<br>(3134.98 to 4370.82)
Ningxia	Ischemic heart disease	52.7<br>(46.1 to 59.7)	1067.68<br>(934.4 to 1208.1)	144<br>(119 to 174)	2053.3<br>(1692.76 to 2477.19)
Qinghai	Ischemic heart disease	47.1<br>(40.7 to 55.6)	994.21<br>(858.92 to 1174.24)	126<br>(104 to 148)	2008.63<br>(1659.93 to 2350)
Shaanxi	Ischemic heart disease	247<br>(222 to 281)	718.87<br>(644.68 to 815.57)	968<br>(804 to 1160)	2450.91<br>(2036.18 to 2929.5)
Shandong	Ischemic heart disease	1200<br>(1060 to 1360)	1401.25<br>(1237.62 to 1585.07)	2780<br>(2380 to 3240)	2797.21<br>(2390.65 to 3263.95)
Shanghai	Ischemic heart disease	125<br>(110 to 142)	895.74<br>(786.47 to 1016.56)	233<br>(199 to 272)	832.33<br>(710.7 to 974.01)
Shanxi	Ischemic heart disease	414<br>(367 to 464)	1367.72<br>(1210.78 to 1530.98)	840<br>(691 to 1000)	2175.69<br>(1789.07 to 2600.95)

Sichuan	Ischemic heart disease	843 (759 to 960)	755.6 (680.2 to 860.11)	1370 (1160 to 1570)	1637.72 (1394.88 to 1885.11)
Tianjin	Ischemic heart disease	168 (147 to 188)	1825.76 (1594.15 to 2049.47)	403 (345 to 475)	2639.58 (2262.29 to 3111.49)
Tibet	Ischemic heart disease	36.6 (31.2 to 47.3)	1569.11 (1338.78 to 2031)	55.4 (45.9 to 69.3)	1602.86 (1327.38 to 2005.44)
Xinjiang	Ischemic heart disease	206 (185 to 240)	1303.25 (1170.99 to 1518.45)	619 (520 to 732)	2541.09 (2135.3 to 3003.08)
Yunnan	Ischemic heart disease	352 (302 to 427)	903.68 (774.5 to 1094.57)	865 (733 to 1020)	1759.02 (1491.72 to 2077.32)
Zhejiang	Ischemic heart disease	318 (286 to 359)	720.21 (648.53 to 813.47)	473 (401 to 560)	772.6 (655.63 to 914.68)
China	Stroke	30200 (28900 to 32200)	2520.29 (2417.63 to 2688.61)	44300 (41900 to 46700)	3135.71 (2968.92 to 3308.33)
Taiwan	Stroke	387 (372 to 402)	1897.47 (1823.32 to 1970.73)	321 (290 to 350)	1360.76 (1227.82 to 1485.02)
Hong Kong Special Administrative Region of China	Stroke	67.8 (61.8 to 76.4)	1177.08 (1072.74 to 1325.52)	96.7 (82.9 to 112)	1288.01 (1103.58 to 1485.34)
Macau Special Administrative Region of China	Stroke	5 (4.63 to 5.44)	1408.3 (1304.27 to 1533.79)	7.2 (6.06 to 8.45)	1052.89 (886.43 to 1235.3)
Anhui	Stroke	1360 (1230 to 1500)	2296.48 (2080.09 to 2539.66)	2050 (1810 to 2330)	3360.69 (2954.2 to 3814.32)
Beijing	Stroke	333 (295 to 376)	2971.27 (2634.48 to 3352.47)	412 (351 to 478)	1711.31 (1457.14 to 1983.62)
Chongqing	Stroke	334 (296 to 383)	2119.09 (1878.39 to 2428.36)	792 (663 to 943)	2962.99 (2480.74 to 3529.55)
Fujian	Stroke	520 (477 to 580)	1643.12 (1507.62 to 1832.77)	734 (634 to 844)	1862.56 (1609.07 to 2142.33)
Gansu	Stroke	648 (592 to 719)	2738.37 (2503.28 to 3040.84)	857 (738 to 994)	3224.71 (2777.89 to 3741.18)
Guangdong	Stroke	1420 (1240 to 1610)	2151.16 (1887.84 to 2440.99)	1950 (1690 to 2250)	1666.73 (1445.2 to 1925.07)
Guangxi	Stroke	930 (817 to 1090)	2137.38 (1878.78 to 2499.58)	1490 (1290 to 1750)	3084.45 (2662.74 to 3621.16)
Guizhou	Stroke	993 (872 to 1150)	2991.37 (2628.47 to 3454.83)	1410 (1210 to 1610)	4159.12 (3565.78 to 4763.22)
Hainan	Stroke	150 (133 to 169)	2150.92 (1912.52 to 2422.86)	199 (168 to 235)	2078.8 (1751.71 to 2447.66)
Hebei	Stroke	1850 (1700 to 2030)	2907.21 (2677.5 to 3196.22)	3500 (3050 to 4000)	4578.56 (3996.32 to 5238.94)
Heilongjiang	Stroke	1240 (1120 to 1360)	3402.16 (3083.54 to 3721.55)	1870 (1590 to 2180)	4775.06 (4054.69 to 5545.59)
Henan	Stroke	2500 (2310 to 2680)	2828.21 (2617.11 to 3036.08)	3920 (3410 to 4470)	4066.68 (3538.47 to 4643.33)
Hubei	Stroke	1510 (1380 to 1710)	2718.21 (2482.57 to 3086.77)	2170 (1870 to 2490)	3920.97 (3389.18 to 4494.74)
Hunan	Stroke	1750 (1600 to 2070)	2815.71 (2573.22 to 3324.72)	2340 (2030 to 2700)	3490.85 (3016.78 to 4024.51)
Inner Mongolia	Stroke	789 (706 to 878)	3468.06 (3102.81 to 3861.61)	920 (803 to 1060)	3553.43 (3099.39 to 4084.57)
Jiangsu	Stroke	1380 (1250 to 1520)	2001.01 (1821.15 to 2204.87)	2280 (1990 to 2590)	2794.12 (2432.34 to 3176.06)
Jiangxi	Stroke	1080 (983 to 1240)	2727.37 (2487.13 to 3149.22)	1190 (1040 to 1370)	2513.17 (2186.66 to 2888.4)
Jilin	Stroke	901 (835 to 969)	3470.26 (3218.65 to 3735.36)	1090 (910 to 1260)	3906.43 (3261.88 to 4522.05)
Liaoning	Stroke	1360 (1210 to 1540)	3345.53 (2977.52 to 3780.48)	2090 (1800 to 2430)	4678.93 (4020.59 to 5431.56)
Ningxia	Stroke	102 (91.5 to 113)	2063.74 (1851.77 to 2292.47)	153 (130 to 181)	2184.66 (1854.15 to 2572.38)
Qinghai	Stroke	112 (98.9 to 128)	2357.53 (2088.35 to 2708.65)	197 (165 to 228)	3132.88 (2623.85 to 3622.8)
Shaanxi	Stroke	474 (430 to 525)	1376.68 (1249.91 to 1525.75)	1280 (1080 to 1520)	3246.62 (2731.97 to 3849.04)
Shandong	Stroke	2580 (2320 to 2840)	3009.84 (2710.35 to 3323.66)	3190 (2750 to 3680)	3211.58 (2766.27 to 3706.98)

Shanghai	Stroke	268 (238 to 299)	1920.47 (1702.31 to 2141.85)	377 (324 to 434)	1350.91 (1158.4 to 1553.6)
Shanxi	Stroke	1010 (919 to 1110)	3329.09 (3034.15 to 3657.16)	1110 (934 to 1300)	2885.2 (2418.07 to 3369.64)
Sichuan	Stroke	1870 (1710 to 2090)	1678.51 (1530.71 to 1876.64)	2740 (2320 to 3150)	3280.5 (2784.29 to 3772.75)
Tianjin	Stroke	311 (275 to 346)	3377.61 (2986.84 to 3760.15)	476 (409 to 549)	3119.77 (2678.05 to 3597.54)
Tibet	Stroke	120 (103 to 152)	5136.13 (4418.55 to 6521.33)	124 (104 to 153)	3573.91 (2998.21 to 4434.08)
Xinjiang	Stroke	301 (273 to 341)	1906.83 (1727.94 to 2160.13)	654 (561 to 760)	2682.14 (2303.27 to 3119.3)
Yunnan	Stroke	947 (830 to 1130)	2429.01 (2130.13 to 2894.71)	1500 (1300 to 1760)	3057.26 (2635.82 to 3587.28)
Zhejiang	Stroke	962 (880 to 1090)	2181.85 (1994.94 to 2464.91)	1100 (953 to 1270)	1803.98 (1556.73 to 2077.36)
China	Hypertensive heart disease	4100 (2710 to 4570)	342.78 (226.22 to 381.54)	4850 (3260 to 5370)	343.64 (230.9 to 380.2)
Taiwan	Hypertensive heart disease	42.1 (30.3 to 45.8)	206.3 (148.7 to 224.45)	57.9 (51 to 69.5)	245.43 (216.35 to 294.86)
Hong Kong Special Administrative Region of China	Hypertensive heart disease	14.8 (9.72 to 16.7)	255.97 (168.55 to 289.26)	14.8 (12.1 to 21.9)	197.65 (160.82 to 291.61)
Macau Special Administrative Region of China	Hypertensive heart disease	0.664 (0.548 to 0.759)	187.17 (154.34 to 213.8)	1 (0.77 to 1.35)	146.42 (112.54 to 197.79)
Anhui	Hypertensive heart disease	185 (121 to 219)	312.98 (204.24 to 368.96)	257 (145 to 307)	420.78 (237.77 to 502.68)
Beijing	Hypertensive heart disease	13.5 (10.2 to 27.6)	120.67 (91.14 to 246.44)	16.3 (11.8 to 35)	67.54 (48.77 to 145.17)
Chongqing	Hypertensive heart disease	47.1 (33.6 to 56.5)	298.14 (213.22 to 357.84)	102 (68 to 125)	381.54 (254.27 to 467.61)
Fujian	Hypertensive heart disease	72.2 (53 to 85)	228.23 (167.47 to 268.56)	97.6 (67.7 to 116)	247.72 (171.82 to 295.15)
Gansu	Hypertensive heart disease	89.4 (54 to 106)	377.83 (228.16 to 446.36)	128 (65.5 to 157)	481.77 (246.38 to 590.78)
Guangdong	Hypertensive heart disease	200 (148 to 239)	304.47 (225.49 to 362.43)	202 (152 to 241)	172.96 (130.35 to 206.22)
Guangxi	Hypertensive heart disease	160 (105 to 193)	369.02 (242.03 to 444.87)	178 (111 to 217)	367.6 (230.64 to 448.97)
Guizhou	Hypertensive heart disease	101 (84.8 to 121)	304.71 (255.45 to 363.96)	102 (83.6 to 126)	301.92 (247.12 to 372.51)
Hainan	Hypertensive heart disease	9.43 (6.53 to 14.4)	135.39 (93.81 to 206.14)	15.2 (12.1 to 19.8)	158.21 (125.77 to 206.77)
Hebei	Hypertensive heart disease	393 (150 to 466)	616.82 (235.7 to 731.93)	623 (233 to 784)	814.75 (304.39 to 1026.25)
Heilongjiang	Hypertensive heart disease	44.3 (32.3 to 81.3)	121.56 (88.5 to 222.94)	66.5 (52.7 to 110)	169.4 (134.22 to 281.27)
Henan	Hypertensive heart disease	327 (187 to 386)	370.16 (211.63 to 436.76)	293 (225 to 344)	304.47 (233.61 to 357.3)
Hubei	Hypertensive heart disease	359 (140 to 426)	645.72 (252.2 to 766.28)	299 (159 to 361)	541.06 (288.31 to 652.24)
Hunan	Hypertensive heart disease	443 (177 to 529)	712.32 (285.04 to 850.68)	541 (225 to 677)	805.49 (335.61 to 1008.12)
Inner Mongolia	Hypertensive heart disease	64.1 (44.4 to 75.7)	282 (195.32 to 333.05)	73.8 (53.9 to 88)	284.84 (207.98 to 339.83)
Jiangsu	Hypertensive heart disease	159 (136 to 185)	230.78 (197.61 to 268.98)	196 (166 to 239)	239.42 (203.4 to 292.44)
Jiangxi	Hypertensive heart disease	223 (118 to 263)	564.18 (299.68 to 666.28)	211 (101 to 257)	443.17 (213.3 to 539.96)
Jilin	Hypertensive heart disease	60.3 (45.6 to 70.5)	232.28 (175.6 to 271.66)	88.7 (62.9 to 105)	317.91 (225.5 to 377.03)
Liaoning	Hypertensive heart disease	42.7 (29.6 to 95.5)	104.78 (72.49 to 234.13)	68.1 (52.4 to 131)	152.56 (117.34 to 293.29)
Ningxia	Hypertensive heart disease	7.31 (6.19 to 9.18)	148.06 (125.27 to 185.87)	9.77 (7.97 to 13.1)	139.14 (113.6 to 187.1)
Qinghai	Hypertensive heart disease	12.9 (8.84 to 15.2)	272.19 (186.7 to 320.1)	17.6 (13.3 to 21)	280.58 (211.93 to 334.61)

Shaanxi	Hypertensive heart disease	62.2 (41 to 72.5)	180.72 (119.06 to 210.62)	124 (79.6 to 155)	314.72 (201.51 to 391.45)
Shandong	Hypertensive heart disease	267 (213 to 310)	311.36 (248.57 to 362.22)	267 (223 to 315)	268.9 (224.38 to 316.77)
Shanghai	Hypertensive heart disease	15.2 (11.7 to 29.6)	108.45 (83.67 to 211.81)	21.4 (16.2 to 42.5)	76.44 (57.85 to 152.17)
Shanxi	Hypertensive heart disease	110 (73.1 to 131)	364.21 (241.28 to 431.61)	96.4 (71.3 to 117)	249.58 (184.69 to 302.05)
Sichuan	Hypertensive heart disease	259 (215 to 297)	232.01 (192.41 to 266.08)	252 (207 to 295)	301.44 (248.43 to 353.82)
Tianjin	Hypertensive heart disease	9.65 (6.9 to 24.4)	104.9 (75.02 to 265.61)	11.7 (8.19 to 31.1)	76.58 (53.66 to 204.06)
Tibet	Hypertensive heart disease	38.7 (9.87 to 48.9)	1660.57 (423.55 to 2100.55)	25.8 (8.43 to 32.1)	746.26 (243.78 to 928.72)
Xinjiang	Hypertensive heart disease	79.4 (34.2 to 94.2)	503.58 (216.94 to 596.91)	145 (62.2 to 188)	593.35 (255.41 to 772.63)
Yunnan	Hypertensive heart disease	125 (99.1 to 148)	320.65 (254.23 to 379.4)	155 (116 to 183)	315.12 (236.49 to 372.25)
Zhejiang	Hypertensive heart disease	108 (88.1 to 126)	245.32 (199.74 to 286.82)	155 (103 to 185)	253.55 (168.76 to 301.88)
China	Cardiomyopathy and myocarditis	732 (672 to 815)	61.15 (56.1 to 68.1)	855 (797 to 923)	60.56 (56.39 to 65.34)
Taiwan	Cardiomyopathy and myocarditis	11.9 (11 to 12.9)	58.57 (53.96 to 63.06)	18 (15.9 to 19.8)	76.23 (67.45 to 83.96)
Hong Kong Special Administrative Region of China	Cardiomyopathy and myocarditis	2.18 (1.94 to 2.56)	37.85 (33.68 to 44.4)	3.53 (3 to 4.19)	46.97 (39.95 to 55.76)
Macau Special Administrative Region of China	Cardiomyopathy and myocarditis	0.119 (0.105 to 0.149)	33.44 (29.62 to 41.94)	0.168 (0.135 to 0.218)	24.63 (19.76 to 31.93)
Anhui	Cardiomyopathy and myocarditis	37.8 (30.5 to 47.6)	63.8 (51.44 to 80.33)	26.3 (22.8 to 30.8)	43.06 (37.29 to 50.38)
Beijing	Cardiomyopathy and myocarditis	4.59 (3.96 to 5.76)	40.93 (35.28 to 51.32)	8.19 (6.89 to 9.91)	33.98 (28.57 to 41.14)
Chongqing	Cardiomyopathy and myocarditis	8.83 (7.07 to 11.2)	55.94 (44.79 to 70.88)	15.2 (12.7 to 18.1)	56.94 (47.41 to 67.84)
Fujian	Cardiomyopathy and myocarditis	12.7 (10.9 to 15.6)	40.05 (34.33 to 49.44)	16.1 (13.9 to 19.2)	40.96 (35.19 to 48.74)
Gansu	Cardiomyopathy and myocarditis	28.1 (22.6 to 34.8)	118.78 (95.56 to 147.25)	24.1 (20 to 28.4)	90.67 (75.2 to 106.92)
Guangdong	Cardiomyopathy and myocarditis	26.5 (22.5 to 32)	40.26 (34.15 to 48.59)	55.6 (47.2 to 65.2)	47.62 (40.39 to 55.77)
Guangxi	Cardiomyopathy and myocarditis	62.5 (51.5 to 72.6)	143.62 (118.45 to 167.02)	87.5 (62.8 to 103)	181 (129.94 to 213.78)
Guizhou	Cardiomyopathy and myocarditis	41.2 (34 to 49.4)	124.17 (102.57 to 148.84)	43.6 (32.9 to 53.1)	128.89 (97.36 to 156.92)
Hainan	Cardiomyopathy and myocarditis	5.32 (4.17 to 6.76)	76.31 (59.84 to 97.08)	6.37 (5.22 to 7.54)	66.4 (54.46 to 78.62)
Hebei	Cardiomyopathy and myocarditis	25.7 (22 to 37.6)	40.42 (34.57 to 59.15)	35 (29 to 44.1)	45.84 (37.92 to 57.65)
Heilongjiang	Cardiomyopathy and myocarditis	16.9 (14 to 21.3)	46.24 (38.32 to 58.46)	16.9 (14.5 to 20.3)	43.1 (36.89 to 51.64)
Henan	Cardiomyopathy and myocarditis	40.6 (33.1 to 54.3)	46.05 (37.46 to 61.54)	36.3 (30.9 to 43.7)	37.66 (32.08 to 45.37)
Hubei	Cardiomyopathy and myocarditis	29.2 (24.6 to 35.9)	52.63 (44.23 to 64.61)	31.3 (27 to 36.5)	56.61 (48.83 to 66.08)
Hunan	Cardiomyopathy and myocarditis	31.6 (26.6 to 38.6)	50.78 (42.83 to 62.12)	46.8 (39.3 to 56.5)	69.63 (58.51 to 84.11)
Inner Mongolia	Cardiomyopathy and myocarditis	15.6 (12.7 to 19.6)	68.53 (56.03 to 86.04)	15.5 (13.4 to 18.2)	59.74 (51.66 to 70.23)
Jiangsu	Cardiomyopathy and myocarditis	23.4 (20.4 to 29.8)	33.93 (29.64 to 43.37)	31.5 (27 to 37.6)	38.52 (33.02 to 46.07)
Jiangxi	Cardiomyopathy and myocarditis	36.3 (29.7 to 45.5)	91.94 (75.17 to 115.2)	34.6 (29.9 to 40.6)	72.82 (62.83 to 85.46)
Jilin	Cardiomyopathy and myocarditis	14.2 (11.8 to 17.7)	54.76 (45.37 to 68.32)	12.4 (10.4 to 14.9)	44.52 (37.27 to 53.26)
Liaoning	Cardiomyopathy and myocarditis	14.5 (12 to 19.6)	35.52 (29.36 to 48.04)	16 (13.4 to 20.5)	35.94 (30.08 to 46.01)

Ningxia	Cardiomyopathy and myocarditis	3.67 (2.79 to 4.95)	74.31 (56.44 to 100.31)	3.28 (2.71 to 3.99)	46.68 (38.57 to 56.81)
Qinghai	Cardiomyopathy and myocarditis	4.35 (3.38 to 5.55)	91.8 (71.38 to 117.25)	5.3 (4.4 to 6.39)	84.31 (70.11 to 101.67)
Shaanxi	Cardiomyopathy and myocarditis	28.8 (23.6 to 35.1)	83.59 (68.62 to 102.13)	36.3 (29.8 to 43)	91.76 (75.41 to 108.73)
Shandong	Cardiomyopathy and myocarditis	37.7 (31.8 to 47.9)	44.08 (37.12 to 55.93)	42.3 (36 to 50.5)	42.57 (36.25 to 50.81)
Shanghai	Cardiomyopathy and myocarditis	4.98 (4.34 to 6.02)	35.68 (31.08 to 43.12)	9.84 (8.29 to 11.9)	35.23 (29.66 to 42.74)
Shanxi	Cardiomyopathy and myocarditis	14.7 (12.2 to 18.7)	48.46 (40.42 to 61.89)	17.2 (14.3 to 20.4)	44.44 (37.03 to 52.77)
Sichuan	Cardiomyopathy and myocarditis	74.7 (60.4 to 92.3)	66.95 (54.09 to 82.74)	63.2 (53.2 to 74.1)	75.74 (63.73 to 88.77)
Tianjin	Cardiomyopathy and myocarditis	3.35 (2.84 to 4.5)	36.47 (30.89 to 48.96)	4.52 (3.77 to 6.44)	29.6 (24.69 to 42.17)
Tibet	Cardiomyopathy and myocarditis	2.93 (2.42 to 3.6)	125.57 (103.82 to 154.41)	5.21 (4.21 to 6.6)	150.61 (121.89 to 190.93)
Xinjiang	Cardiomyopathy and myocarditis	15.9 (12.7 to 19.8)	100.79 (80.78 to 125.35)	20.8 (17.1 to 24.9)	85.2 (70.17 to 102.38)
Yunnan	Cardiomyopathy and myocarditis	43.3 (34.9 to 52.8)	111.19 (89.65 to 135.45)	56.9 (44.2 to 68.9)	115.78 (89.84 to 140.05)
Zhejiang	Cardiomyopathy and myocarditis	19.8 (16.8 to 23.6)	44.85 (38.02 to 53.46)	27.6 (23.3 to 32.4)	45.07 (38.06 to 52.99)
China	Atrial fibrillation and flutter	428 (348 to 520)	35.75 (29.1 to 43.42)	1150 (952 to 1380)	81.13 (67.42 to 97.57)
Taiwan	Atrial fibrillation and flutter	8.1 (6.63 to 9.97)	39.71 (32.47 to 48.88)	23.2 (19.3 to 27.8)	98.56 (81.94 to 117.96)
Hong Kong Special Administrative Region of China	Atrial fibrillation and flutter	2.78 (2.24 to 3.38)	48.19 (38.91 to 58.71)	8.71 (7.14 to 10.6)	115.92 (95.04 to 141.45)
Macau Special Administrative Region of China	Atrial fibrillation and flutter	0.161 (0.135 to 0.194)	45.43 (37.99 to 54.55)	0.464 (0.373 to 0.584)	67.89 (54.55 to 85.43)
Anhui	Atrial fibrillation and flutter	20.4 (16.3 to 25.4)	34.46 (27.44 to 42.84)	54.8 (45.1 to 67)	89.69 (73.79 to 109.63)
Beijing	Atrial fibrillation and flutter	5.72 (4.63 to 6.86)	50.96 (41.32 to 61.18)	18.5 (14.8 to 22.7)	76.66 (61.31 to 94.22)
Chongqing	Atrial fibrillation and flutter	5.41 (4.25 to 6.78)	34.29 (26.93 to 42.97)	28.5 (22.7 to 35)	106.56 (84.78 to 130.98)
Fujian	Atrial fibrillation and flutter	9.44 (7.43 to 11.8)	29.84 (23.48 to 37.39)	28.9 (23.7 to 35.3)	73.41 (60.16 to 89.49)
Gansu	Atrial fibrillation and flutter	6.09 (4.77 to 7.55)	25.75 (20.15 to 31.94)	18.7 (15.1 to 23.3)	70.31 (56.75 to 87.49)
Guangdong	Atrial fibrillation and flutter	21.4 (17 to 26.3)	32.47 (25.83 to 39.9)	60.2 (48.7 to 74.5)	51.54 (41.72 to 63.75)
Guangxi	Atrial fibrillation and flutter	15.2 (12.1 to 18.9)	34.98 (27.83 to 43.39)	38.1 (31.4 to 46.1)	78.92 (65.02 to 95.34)
Guizhou	Atrial fibrillation and flutter	10.6 (8.59 to 13.1)	32.08 (25.87 to 39.45)	25.7 (20.7 to 31.6)	76.01 (61.25 to 93.38)
Hainan	Atrial fibrillation and flutter	2.21 (1.76 to 2.72)	31.71 (25.31 to 39.06)	6.28 (5.01 to 7.79)	65.52 (52.22 to 81.26)
Hebei	Atrial fibrillation and flutter	23.7 (19 to 29.3)	37.25 (29.9 to 46)	59.3 (47.8 to 72.3)	77.63 (62.58 to 94.66)
Heilongjiang	Atrial fibrillation and flutter	10.4 (8.42 to 12.6)	28.4 (23.09 to 34.5)	33.7 (27.2 to 41)	85.73 (69.25 to 104.4)
Henan	Atrial fibrillation and flutter	32.9 (26.4 to 40.2)	37.32 (29.96 to 45.57)	72.9 (59.7 to 87.9)	75.71 (61.98 to 91.31)
Hubei	Atrial fibrillation and flutter	19.9 (16.1 to 24.2)	35.79 (28.9 to 43.51)	49.3 (39.8 to 60.4)	89.07 (71.93 to 109.2)
Hunan	Atrial fibrillation and flutter	21.9 (17.1 to 27.7)	35.18 (27.52 to 44.6)	59.8 (48.1 to 71.7)	89.05 (71.62 to 106.75)
Inner Mongolia	Atrial fibrillation and flutter	6.47 (5.18 to 7.86)	28.43 (22.78 to 34.57)	19.8 (16 to 24)	76.45 (61.95 to 92.86)
Jiangsu	Atrial fibrillation and flutter	28.9 (23.3 to 35.7)	42.07 (33.9 to 51.82)	83.9 (69.2 to 101)	102.75 (84.8 to 123.96)
Jiangxi	Atrial fibrillation and flutter	12.8 (9.98 to 15.9)	32.28 (25.24 to 40.31)	31.6 (25.5 to 38.5)	66.44 (53.67 to 81.06)

Jilin	Atrial fibrillation and flutter	8.34 (6.79 to 10.2)	32.12 (26.17 to 39.12)	23.3 (18.6 to 28.8)	83.52 (66.63 to 103.33)
Liaoning	Atrial fibrillation and flutter	19.5 (15.5 to 24)	47.71 (37.96 to 58.89)	54.9 (43.9 to 67.7)	122.93 (98.26 to 151.67)
Ningxia	Atrial fibrillation and flutter	1.17 (0.949 to 1.46)	23.69 (19.21 to 29.47)	3.85 (3.08 to 4.78)	54.85 (43.87 to 68.03)
Qinghai	Atrial fibrillation and flutter	1.02 (0.814 to 1.27)	21.61 (17.19 to 26.78)	3.27 (2.61 to 4.08)	52.1 (41.55 to 65.02)
Shaanxi	Atrial fibrillation and flutter	11.2 (8.2 to 14.2)	32.52 (23.83 to 41.3)	29.9 (24.2 to 36.8)	75.74 (61.24 to 93.15)
Shandong	Atrial fibrillation and flutter	35.1 (27.9 to 43.1)	40.99 (32.55 to 50.3)	92.7 (74.7 to 113)	93.23 (75.13 to 113.86)
Shanghai	Atrial fibrillation and flutter	7.9 (6.26 to 9.73)	56.53 (44.82 to 69.62)	23.7 (19 to 29)	84.96 (68.14 to 103.84)
Shanxi	Atrial fibrillation and flutter	11.4 (9.32 to 14)	37.66 (30.79 to 46.12)	27.4 (21.8 to 34.1)	71.05 (56.45 to 88.23)
Sichuan	Atrial fibrillation and flutter	38 (30.3 to 47.7)	34.08 (27.19 to 42.75)	76.4 (61.4 to 93.7)	91.49 (73.55 to 112.18)
Tianjin	Atrial fibrillation and flutter	3.87 (3.06 to 4.76)	42.07 (33.28 to 51.77)	10.8 (8.52 to 13.4)	70.62 (55.83 to 87.67)
Tibet	Atrial fibrillation and flutter	0.711 (0.557 to 0.902)	30.5 (23.92 to 38.72)	1.39 (1.1 to 1.7)	40.18 (31.91 to 49.26)
Xinjiang	Atrial fibrillation and flutter	4.12 (3.3 to 5.11)	26.1 (20.93 to 32.39)	14.8 (12 to 17.9)	60.86 (49.41 to 73.52)
Yunnan	Atrial fibrillation and flutter	12 (9.6 to 14.9)	30.89 (24.63 to 38.29)	32.7 (26.6 to 39.9)	66.57 (54.16 to 81.11)
Zhejiang	Atrial fibrillation and flutter	17.1 (13.2 to 21.9)	38.87 (29.9 to 49.61)	51.6 (41.6 to 62.8)	84.39 (67.98 to 102.69)
China	Aortic aneurysm	191 (157 to 245)	15.97 (13.15 to 20.48)	331 (296 to 368)	23.42 (20.93 to 26.04)
Taiwan	Aortic aneurysm	4.45 (4.23 to 4.67)	21.79 (20.73 to 22.89)	19.2 (17.7 to 20.8)	81.47 (75.15 to 88.18)
Hong Kong Special Administrative Region of China	Aortic aneurysm	4.21 (2.25 to 5.23)	73.03 (38.96 to 90.69)	2.44 (1.75 to 3.08)	32.49 (23.36 to 41.03)
Macau Special Administrative Region of China	Aortic aneurysm	0.311 (0.158 to 0.385)	87.55 (44.4 to 108.5)	0.469 (0.199 to 0.754)	68.55 (29.06 to 110.2)
Anhui	Aortic aneurysm	8.58 (5.99 to 13.2)	14.48 (10.11 to 22.2)	12.7 (10.3 to 15.3)	20.75 (16.9 to 25.07)
Beijing	Aortic aneurysm	2.67 (2.08 to 3.31)	23.82 (18.54 to 29.49)	4.68 (3 to 6.02)	19.43 (12.44 to 24.96)
Chongqing	Aortic aneurysm	2.4 (1.79 to 3.42)	15.21 (11.36 to 21.65)	6.28 (4.96 to 7.99)	23.49 (18.57 to 29.88)
Fujian	Aortic aneurysm	3.48 (2.57 to 4.64)	11.01 (8.13 to 14.66)	7.28 (5.82 to 9.28)	18.47 (14.76 to 23.54)
Gansu	Aortic aneurysm	4.01 (3.11 to 5.41)	16.97 (13.14 to 22.86)	5.84 (4.51 to 7.12)	21.97 (16.95 to 26.79)
Guangdong	Aortic aneurysm	13.2 (10.4 to 16.3)	20.04 (15.8 to 24.79)	27.4 (17.3 to 35.1)	23.45 (14.79 to 30.08)
Guangxi	Aortic aneurysm	6.55 (4.93 to 10)	15.07 (11.35 to 23.05)	11.1 (8.76 to 15.1)	22.87 (18.14 to 31.23)
Guizhou	Aortic aneurysm	4.31 (3.13 to 7.5)	13 (9.42 to 22.6)	7.45 (5.6 to 11.4)	22.03 (16.57 to 33.82)
Hainan	Aortic aneurysm	0.894 (0.695 to 1.19)	12.84 (9.98 to 17.07)	1.68 (1.29 to 2.15)	17.48 (13.43 to 22.45)
Hebei	Aortic aneurysm	9.6 (6.97 to 13.4)	15.08 (10.96 to 21.05)	18.9 (14.9 to 28.2)	24.69 (19.45 to 36.93)
Heilongjiang	Aortic aneurysm	5.39 (4.27 to 7.66)	14.78 (11.71 to 21.02)	9.52 (7.59 to 12.3)	24.25 (19.34 to 31.35)
Henan	Aortic aneurysm	11.4 (8.45 to 14.9)	12.91 (9.58 to 16.86)	19 (15.5 to 23.6)	19.69 (16.09 to 24.52)
Hubei	Aortic aneurysm	8.92 (6.99 to 11.4)	16.06 (12.59 to 20.49)	15.6 (12.1 to 19.1)	28.19 (21.85 to 34.54)
Hunan	Aortic aneurysm	11.4 (8.72 to 16.1)	18.39 (14.03 to 25.82)	21.6 (17.6 to 27.7)	32.14 (26.22 to 41.26)
Inner Mongolia	Aortic aneurysm	3.5 (2.71 to 4.75)	15.4 (11.9 to 20.87)	6.39 (5.14 to 8.14)	24.68 (19.87 to 31.43)

Jiangsu	Aortic aneurysm	9.91 (7.7 to 12.2)	14.41 (11.19 to 17.76)	18.3 (14.2 to 22.5)	22.4 (17.33 to 27.61)
Jiangxi	Aortic aneurysm	7.44 (5.2 to 10.7)	18.82 (13.16 to 27.18)	9.48 (7.63 to 11.4)	19.93 (16.06 to 23.98)
Jilin	Aortic aneurysm	4.36 (3.43 to 6.38)	16.81 (13.22 to 24.57)	6.39 (5.11 to 8.01)	22.9 (18.32 to 28.72)
Liaoning	Aortic aneurysm	7.2 (5.56 to 9.35)	17.64 (13.64 to 22.92)	12.9 (10.4 to 16.3)	28.89 (23.23 to 36.48)
Ningxia	Aortic aneurysm	0.762 (0.561 to 0.938)	15.43 (11.37 to 18.99)	1.48 (0.919 to 1.95)	21.07 (13.09 to 27.8)
Qinghai	Aortic aneurysm	0.718 (0.566 to 0.97)	15.15 (11.96 to 20.48)	1.46 (1.17 to 1.78)	23.24 (18.58 to 28.39)
Shaanxi	Aortic aneurysm	3.51 (2.34 to 5.37)	10.21 (6.8 to 15.61)	8.85 (6.18 to 11.4)	22.41 (15.65 to 28.87)
Shandong	Aortic aneurysm	15.4 (11.8 to 22.3)	17.95 (13.76 to 26.09)	22.4 (18.1 to 28.1)	22.58 (18.24 to 28.24)
Shanghai	Aortic aneurysm	2.76 (2.18 to 3.39)	19.77 (15.61 to 24.26)	5.41 (3.52 to 7.06)	19.36 (12.61 to 25.26)
Shanxi	Aortic aneurysm	5.31 (4.23 to 6.7)	17.53 (13.97 to 22.14)	6.71 (5.22 to 8.26)	17.37 (13.52 to 21.38)
Sichuan	Aortic aneurysm	13.4 (9.74 to 19.3)	12.02 (8.72 to 17.26)	19.9 (15.4 to 26.5)	23.78 (18.41 to 31.7)
Tianjin	Aortic aneurysm	1.99 (1.62 to 2.51)	21.67 (17.61 to 27.24)	3.22 (2.57 to 4.09)	21.13 (16.86 to 26.8)
Tibet	Aortic aneurysm	0.958 (0.648 to 1.48)	41.11 (27.8 to 63.4)	1.29 (1.05 to 1.57)	37.3 (30.51 to 45.28)
Xinjiang	Aortic aneurysm	3.63 (2.76 to 4.58)	23.01 (17.48 to 29.06)	10.8 (6.58 to 14.1)	44.14 (27 to 57.77)
Yunnan	Aortic aneurysm	6.72 (5.19 to 9.98)	17.25 (13.31 to 25.61)	13 (10.8 to 16.2)	26.52 (22.03 to 32.99)
Zhejiang	Aortic aneurysm	6.24 (4.88 to 7.86)	14.16 (11.06 to 17.82)	11 (8.57 to 13.6)	18 (14.01 to 22.3)
China	Peripheral artery disease	72.8 (38.2 to 124)	6.09 (3.19 to 10.38)	164 (93.8 to 266)	11.64 (6.64 to 18.84)
Taiwan	Peripheral artery disease	1.91 (0.971 to 3.25)	9.34 (4.76 to 15.93)	4.41 (2.45 to 7.27)	18.72 (10.4 to 30.83)
Hong Kong Special Administrative Region of China	Peripheral artery disease	0.442 (0.232 to 0.759)	7.68 (4.02 to 13.17)	1.09 (0.606 to 1.81)	14.46 (8.07 to 24.06)
Macau Special Administrative Region of China	Peripheral artery disease	0.0228 (0.0118 to 0.0388)	6.43 (3.31 to 10.94)	0.0671 (0.0378 to 0.112)	9.82 (5.53 to 16.31)
Anhui	Peripheral artery disease	3.23 (1.6 to 5.62)	5.46 (2.71 to 9.48)	6.93 (3.59 to 11.8)	11.34 (5.86 to 19.25)
Beijing	Peripheral artery disease	0.843 (0.465 to 1.4)	7.51 (4.14 to 12.45)	2.89 (1.68 to 4.74)	12.01 (6.98 to 19.67)
Chongqing	Peripheral artery disease	0.984 (0.485 to 1.72)	6.24 (3.07 to 10.92)	3.85 (1.97 to 6.55)	14.39 (7.37 to 24.51)
Fujian	Peripheral artery disease	1.42 (0.72 to 2.46)	4.49 (2.28 to 7.78)	3.45 (1.96 to 5.77)	8.76 (4.98 to 14.65)
Gansu	Peripheral artery disease	1.09 (0.572 to 1.9)	4.62 (2.42 to 8.03)	2.79 (1.55 to 4.7)	10.51 (5.85 to 17.69)
Guangdong	Peripheral artery disease	4.33 (2.3 to 7.34)	6.58 (3.49 to 11.16)	10.2 (5.86 to 16.7)	8.73 (5.02 to 14.26)
Guangxi	Peripheral artery disease	2.5 (1.28 to 4.28)	5.75 (2.95 to 9.85)	5.1 (2.81 to 8.51)	10.56 (5.81 to 17.6)
Guizhou	Peripheral artery disease	2.15 (1.11 to 3.69)	6.48 (3.36 to 11.11)	4.95 (2.66 to 8)	14.63 (7.86 to 23.64)
Hainan	Peripheral artery disease	0.422 (0.223 to 0.706)	6.06 (3.2 to 10.13)	1.03 (0.571 to 1.67)	10.7 (5.95 to 17.38)
Hebei	Peripheral artery disease	4.4 (2.37 to 7.48)	6.91 (3.73 to 11.75)	10 (6.15 to 15.7)	13.09 (8.04 to 20.59)
Heilongjiang	Peripheral artery disease	1.65 (0.869 to 2.9)	4.53 (2.38 to 7.96)	4.78 (2.59 to 8.13)	12.18 (6.6 to 20.71)
Henan	Peripheral artery disease	5.41 (2.73 to 9.41)	6.13 (3.1 to 10.66)	10.1 (5.48 to 17.3)	10.52 (5.69 to 18)
Hubei	Peripheral artery disease	3.02 (1.52 to 5.26)	5.44 (2.74 to 9.46)	6.32 (3.25 to 11.1)	11.43 (5.87 to 19.99)

Hunan	Peripheral artery disease	3.83<br>(2.01 to 6.61)	6.15<br>(3.24 to 10.62)	8.88<br>(5.11 to 14.4)	13.22<br>(7.61 to 21.4)
Inner Mongolia	Peripheral artery disease	1.08<br>(0.579 to 1.85)	4.74<br>(2.54 to 8.14)	2.86<br>(1.66 to 4.71)	11.04<br>(6.42 to 18.18)
Jiangsu	Peripheral artery disease	4.68<br>(2.28 to 8.29)	6.81<br>(3.31 to 12.04)	10.4<br>(5.36 to 18)	12.77<br>(6.57 to 22.09)
Jiangxi	Peripheral artery disease	2.15<br>(1.15 to 3.63)	5.43<br>(2.91 to 9.19)	4.57<br>(2.61 to 7.51)	9.62<br>(5.5 to 15.81)
Jilin	Peripheral artery disease	1.42<br>(0.753 to 2.38)	5.47<br>(2.9 to 9.17)	3.67<br>(2.12 to 5.96)	13.14<br>(7.58 to 21.38)
Liaoning	Peripheral artery disease	2.7<br>(1.45 to 4.6)	6.63<br>(3.55 to 11.29)	6.8<br>(3.86 to 11.1)	15.24<br>(8.64 to 24.79)
Ningxia	Peripheral artery disease	0.183<br>(0.0926 to 0.319)	3.71<br>(1.88 to 6.45)	0.547<br>(0.301 to 0.938)	7.8<br>(4.28 to 13.36)
Qinghai	Peripheral artery disease	0.226<br>(0.111 to 0.37)	4.77<br>(2.34 to 7.82)	0.754<br>(0.343 to 1.21)	12<br>(5.46 to 19.22)
Shaanxi	Peripheral artery disease	2.02<br>(0.997 to 3.56)	5.88<br>(2.9 to 10.35)	4.48<br>(2.39 to 7.49)	11.33<br>(6.06 to 18.95)
Shandong	Peripheral artery disease	5.71<br>(2.95 to 9.99)	6.67<br>(3.45 to 11.67)	12.9<br>(7.15 to 21.2)	12.93<br>(7.19 to 21.31)
Shanghai	Peripheral artery disease	1.31<br>(0.662 to 2.26)	9.35<br>(4.74 to 16.18)	3.21<br>(1.65 to 5.46)	11.49<br>(5.91 to 19.54)
Shanxi	Peripheral artery disease	1.99<br>(1.05 to 3.34)	6.56<br>(3.48 to 11.03)	4.27<br>(2.47 to 7.11)	11.06<br>(6.38 to 18.4)
Sichuan	Peripheral artery disease	6.74<br>(3.38 to 12.1)	6.04<br>(3.02 to 10.8)	11<br>(5.74 to 18.9)	13.21<br>(6.87 to 22.63)
Tianjin	Peripheral artery disease	0.691<br>(0.36 to 1.2)	7.52<br>(3.92 to 13.05)	1.71<br>(0.935 to 2.9)	11.23<br>(6.13 to 19.02)
Tibet	Peripheral artery disease	0.11<br>(0.0548 to 0.194)	4.71<br>(2.35 to 8.32)	0.177<br>(0.089 to 0.31)	5.12<br>(2.58 to 8.96)
Xinjiang	Peripheral artery disease	0.687<br>(0.356 to 1.19)	4.35<br>(2.26 to 7.54)	2.07<br>(1.15 to 3.5)	8.49<br>(4.73 to 14.37)
Yunnan	Peripheral artery disease	2.19<br>(1.16 to 3.74)	5.62<br>(2.98 to 9.6)	4.93<br>(2.83 to 8.04)	10.02<br>(5.75 to 16.35)
Zhejiang	Peripheral artery disease	3.19<br>(1.68 to 5.4)	7.24<br>(3.8 to 12.24)	7.64<br>(4.43 to 12.4)	12.49<br>(7.24 to 20.3)
China	Endocarditis	226<br>(206 to 250)	18.9<br>(17.23 to 20.86)	158<br>(142 to 169)	11.17<br>(10.02 to 11.99)
Taiwan	Endocarditis	2.45<br>(2.19 to 3.1)	12.02<br>(10.74 to 15.18)	12.7<br>(9.16 to 15)	53.72<br>(38.83 to 63.39)
Hong Kong Special Administrative Region of China	Endocarditis	0.521<br>(0.453 to 0.605)	9.04<br>(7.87 to 10.49)	0.89<br>(0.742 to 1.06)	11.85<br>(9.89 to 14.09)
Macau Special Administrative Region of China	Endocarditis	0.0283<br>(0.0247 to 0.0336)	7.96<br>(6.96 to 9.46)	0.0361<br>(0.0271 to 0.0458)	5.28<br>(3.96 to 6.7)
Anhui	Endocarditis	12.8<br>(9.7 to 16.7)	21.56<br>(16.37 to 28.26)	5.81<br>(4.9 to 6.8)	9.5<br>(8.02 to 11.12)
Beijing	Endocarditis	1.12<br>(0.963 to 1.3)	9.95<br>(8.59 to 11.59)	2.01<br>(1.67 to 2.4)	8.35<br>(6.92 to 9.94)
Chongqing	Endocarditis	2.21<br>(1.67 to 2.91)	14<br>(10.59 to 18.41)	2.37<br>(1.9 to 2.89)	8.86<br>(7.12 to 10.81)
Fujian	Endocarditis	3.05<br>(2.53 to 3.68)	9.65<br>(7.99 to 11.64)	3.01<br>(2.47 to 3.61)	7.64<br>(6.26 to 9.16)
Gansu	Endocarditis	11.4<br>(8.75 to 14.9)	48.36<br>(37.01 to 62.89)	3.41<br>(2.87 to 4.14)	12.84<br>(10.79 to 15.58)
Guangdong	Endocarditis	11.9<br>(10.1 to 14.1)	18.12<br>(15.38 to 21.38)	11.5<br>(9.72 to 13.6)	9.84<br>(8.32 to 11.6)
Guangxi	Endocarditis	6.35<br>(5.29 to 7.56)	14.6<br>(12.17 to 17.37)	5.4<br>(4.5 to 6.51)	11.18<br>(9.31 to 13.48)
Guizhou	Endocarditis	8.44<br>(6.7 to 10.9)	25.43<br>(20.18 to 32.7)	4.55<br>(3.7 to 5.5)	13.45<br>(10.94 to 16.27)
Hainan	Endocarditis	1.07<br>(0.82 to 1.38)	15.39<br>(11.78 to 19.82)	1.09<br>(0.858 to 1.32)	11.39<br>(8.95 to 13.75)
Hebei	Endocarditis	13.2<br>(10.9 to 16.2)	20.7<br>(17.07 to 25.4)	16.6<br>(12.6 to 20)	21.68<br>(16.45 to 26.18)
Heilongjiang	Endocarditis	5.37<br>(4.41 to 6.71)	14.72<br>(12.1 to 18.41)	4.54<br>(3.83 to 5.38)	11.58<br>(9.75 to 13.7)

Henan	Endocarditis	15.3<br>(11.9 to 19.8)	17.33<br>(13.48 to 22.48)	8.37<br>(7.14 to 9.81)	8.69<br>(7.42 to 10.19)
Hubei	Endocarditis	8.43<br>(6.75 to 10.6)	15.18<br>(12.15 to 19.15)	5.45<br>(4.59 to 6.5)	9.85<br>(8.31 to 11.75)
Hunan	Endocarditis	11.9<br>(9.77 to 14.9)	19.18<br>(15.72 to 23.96)	10.3<br>(8.18 to 12.4)	15.38<br>(12.17 to 18.53)
Inner Mongolia	Endocarditis	5.49<br>(4.35 to 7.02)	24.14<br>(19.13 to 30.85)	2.68<br>(2.28 to 3.13)	10.35<br>(8.82 to 12.08)
Jiangsu	Endocarditis	7.05<br>(5.94 to 8.47)	10.24<br>(8.63 to 12.31)	7.56<br>(6.34 to 8.95)	9.26<br>(7.76 to 10.96)
Jiangxi	Endocarditis	15.6<br>(12 to 20.1)	39.56<br>(30.41 to 50.72)	6.02<br>(5.02 to 7.35)	12.67<br>(10.55 to 15.45)
Jilin	Endocarditis	4.34<br>(3.52 to 5.43)	16.74<br>(13.58 to 20.93)	3.08<br>(2.55 to 3.67)	11.05<br>(9.15 to 13.15)
Liaoning	Endocarditis	3.66<br>(3.05 to 4.44)	8.96<br>(7.48 to 10.88)	4.39<br>(3.65 to 5.22)	9.84<br>(8.16 to 11.7)
Ningxia	Endocarditis	2.26<br>(1.61 to 3.13)	45.73<br>(32.67 to 63.29)	0.734<br>(0.597 to 0.881)	10.46<br>(8.51 to 12.55)
Qinghai	Endocarditis	3.53<br>(2.67 to 4.68)	74.62<br>(56.3 to 98.85)	1.41<br>(1.15 to 1.7)	22.37<br>(18.37 to 27.07)
Shaanxi	Endocarditis	6.29<br>(4.88 to 8.12)	18.29<br>(14.18 to 23.61)	3.82<br>(3.15 to 4.57)	9.66<br>(7.97 to 11.58)
Shandong	Endocarditis	9.5<br>(7.94 to 11.4)	11.1<br>(9.27 to 13.31)	8.92<br>(7.53 to 10.5)	8.97<br>(7.58 to 10.59)
Shanghai	Endocarditis	1.03<br>(0.897 to 1.17)	7.36<br>(6.42 to 8.4)	2.26<br>(1.84 to 2.77)	8.07<br>(6.59 to 9.92)
Shanxi	Endocarditis	5.4<br>(4.36 to 6.73)	17.82<br>(14.41 to 22.23)	3.49<br>(2.88 to 4.13)	9.04<br>(7.46 to 10.68)
Sichuan	Endocarditis	22.9<br>(17.2 to 31.1)	20.56<br>(15.38 to 27.87)	8.55<br>(7.07 to 10.1)	10.24<br>(8.47 to 12.06)
Tianjin	Endocarditis	0.715<br>(0.6 to 0.839)	7.78<br>(6.53 to 9.13)	1.37<br>(1.14 to 1.65)	8.95<br>(7.46 to 10.79)
Tibet	Endocarditis	1.99<br>(1.57 to 2.53)	85.5<br>(67.17 to 108.53)	1.13<br>(0.885 to 1.52)	32.68<br>(25.61 to 44.09)
Xinjiang	Endocarditis	6.16<br>(4.7 to 7.93)	39.06<br>(29.81 to 50.25)	6.05<br>(4.5 to 7.67)	24.83<br>(18.48 to 31.49)
Yunnan	Endocarditis	14.5<br>(11.3 to 18.3)	37.09<br>(29.09 to 47.06)	6.65<br>(5.62 to 8.12)	13.53<br>(11.42 to 16.52)
Zhejiang	Endocarditis	2.64<br>(2.26 to 3.12)	5.98<br>(5.12 to 7.07)	4.35<br>(3.5 to 5.34)	7.11<br>(5.72 to 8.73)
China	Non-rheumatic valvular heart disease	160<br>(136 to 202)	13.41<br>(11.4 to 16.84)	228<br>(207 to 283)	16.13<br>(14.65 to 20.01)
Taiwan	Non-rheumatic valvular heart disease	5.07<br>(4.57 to 5.8)	24.86<br>(22.39 to 28.42)	8.77<br>(7.52 to 11.1)	37.18<br>(31.88 to 47.16)
Hong Kong Special Administrative Region of China	Non-rheumatic valvular heart disease	0.698<br>(0.592 to 0.916)	12.11<br>(10.27 to 15.89)	1.49<br>(1.22 to 1.9)	19.9<br>(16.24 to 25.26)
Macau Special Administrative Region of China	Non-rheumatic valvular heart disease	0.0408<br>(0.0352 to 0.0531)	11.49<br>(9.93 to 14.97)	0.0715<br>(0.0547 to 0.0938)	10.45<br>(7.99 to 13.72)
Anhui	Non-rheumatic valvular heart disease	7.46<br>(5.53 to 11.2)	12.59<br>(9.33 to 18.9)	9.31<br>(7.8 to 12.1)	15.23<br>(12.76 to 19.77)
Beijing	Non-rheumatic valvular heart disease	1.7<br>(1.22 to 2.23)	15.2<br>(10.87 to 19.9)	2.63<br>(2.08 to 3.69)	10.91<br>(8.62 to 15.32)
Chongqing	Non-rheumatic valvular heart disease	1.64<br>(1.11 to 2.56)	10.42<br>(7.04 to 16.22)	4.2<br>(3.28 to 6.31)	15.7<br>(12.28 to 23.59)
Fujian	Non-rheumatic valvular heart disease	2.49<br>(1.89 to 4.02)	7.87<br>(5.96 to 12.7)	4.16<br>(3.33 to 6.5)	10.55<br>(8.46 to 16.48)
Gansu	Non-rheumatic valvular heart disease	4.38<br>(3.39 to 5.63)	18.51<br>(14.31 to 23.8)	5.7<br>(4.66 to 6.71)	21.45<br>(17.53 to 25.24)
Guangdong	Non-rheumatic valvular heart disease	7.87<br>(5.85 to 10.6)	11.96<br>(8.89 to 16.07)	12.9<br>(10.5 to 17.1)	11.02<br>(8.96 to 14.65)
Guangxi	Non-rheumatic valvular heart disease	5.05<br>(3.85 to 7.49)	11.61<br>(8.84 to 17.23)	6.7<br>(5.46 to 10)	13.86<br>(11.3 to 20.69)
Guizhou	Non-rheumatic valvular heart disease	6.56<br>(5.03 to 8.2)	19.76<br>(15.15 to 24.7)	10<br>(6.99 to 11.9)	29.65<br>(20.68 to 35.23)
Hainan	Non-rheumatic valvular heart disease	0.791<br>(0.591 to 1.02)	11.36<br>(8.49 to 14.6)	1.53<br>(1.22 to 1.87)	15.95<br>(12.76 to 19.54)

Hebei	Non-rheumatic valvular heart disease	10<br>(8.43 to 11.9)	15.75<br>(13.24 to 18.65)	15.9<br>(13.4 to 19.2)	20.81<br>(17.56 to 25.12)
Heilongjiang	Non-rheumatic valvular heart disease	5.28<br>(4.35 to 6.51)	14.48<br>(11.93 to 17.87)	7.63<br>(6.41 to 9.18)	19.45<br>(16.33 to 23.4)
Henan	Non-rheumatic valvular heart disease	8.6<br>(7.11 to 12.8)	9.74<br>(8.05 to 14.53)	11.7<br>(9.51 to 18)	12.16<br>(9.88 to 18.66)
Hubei	Non-rheumatic valvular heart disease	8.52<br>(7.07 to 10.3)	15.34<br>(12.72 to 18.51)	10.7<br>(8.9 to 13.1)	19.37<br>(16.09 to 23.7)
Hunan	Non-rheumatic valvular heart disease	8.04<br>(6.24 to 12.6)	12.93<br>(10.03 to 20.28)	15.3<br>(12.8 to 18.9)	22.71<br>(19.06 to 28.08)
Inner Mongolia	Non-rheumatic valvular heart disease	3.02<br>(2.35 to 3.92)	13.28<br>(10.34 to 17.25)	4.15<br>(3.42 to 5.15)	16.01<br>(13.19 to 19.87)
Jiangsu	Non-rheumatic valvular heart disease	8.88<br>(7.43 to 11.1)	12.9<br>(10.79 to 16.06)	12.3<br>(10.1 to 17)	15.07<br>(12.33 to 20.79)
Jiangxi	Non-rheumatic valvular heart disease	9.38<br>(6.22 to 13)	23.74<br>(15.72 to 32.84)	9.56<br>(7.78 to 11.3)	20.11<br>(16.37 to 23.76)
Jilin	Non-rheumatic valvular heart disease	3.37<br>(2.66 to 4.26)	12.99<br>(10.26 to 16.41)	4.43<br>(3.63 to 5.69)	15.87<br>(13.01 to 20.4)
Liaoning	Non-rheumatic valvular heart disease	4.63<br>(3.71 to 6.43)	11.35<br>(9.11 to 15.76)	6.75<br>(5.44 to 10.3)	15.12<br>(12.18 to 23.04)
Ningxia	Non-rheumatic valvular heart disease	0.544<br>(0.438 to 0.695)	11.02<br>(8.86 to 14.06)	0.856<br>(0.697 to 1.05)	12.19<br>(9.93 to 15)
Qinghai	Non-rheumatic valvular heart disease	1.57<br>(0.674 to 2.07)	33.05<br>(14.22 to 43.75)	2.4<br>(1.04 to 2.93)	38.21<br>(16.47 to 46.71)
Shaanxi	Non-rheumatic valvular heart disease	3.04<br>(2.18 to 4.01)	8.84<br>(6.34 to 11.65)	5.6<br>(4.57 to 7.18)	14.17<br>(11.56 to 18.17)
Shandong	Non-rheumatic valvular heart disease	10.2<br>(7.17 to 16)	11.92<br>(8.37 to 18.69)	13.3<br>(10.7 to 20.4)	13.43<br>(10.75 to 20.49)
Shanghai	Non-rheumatic valvular heart disease	1.86<br>(1.29 to 2.48)	13.29<br>(9.25 to 17.74)	2.92<br>(2.31 to 3.99)	10.46<br>(8.27 to 14.29)
Shanxi	Non-rheumatic valvular heart disease	4.32<br>(3.28 to 5.73)	14.26<br>(10.84 to 18.93)	5.14<br>(4.23 to 6.57)	13.32<br>(10.95 to 17.01)
Sichuan	Non-rheumatic valvular heart disease	13.2<br>(11.1 to 16.6)	11.85<br>(9.92 to 14.87)	14.9<br>(12.3 to 19.6)	17.84<br>(14.74 to 23.47)
Tianjin	Non-rheumatic valvular heart disease	1.29<br>(0.968 to 1.7)	13.99<br>(10.53 to 18.52)	1.85<br>(1.51 to 2.66)	12.12<br>(9.88 to 17.44)
Tibet	Non-rheumatic valvular heart disease	1.24<br>(0.55 to 1.72)	53.24<br>(23.59 to 73.9)	1.26<br>(0.572 to 1.53)	36.31<br>(16.56 to 44.23)
Xinjiang	Non-rheumatic valvular heart disease	2.94<br>(2.27 to 3.44)	18.64<br>(14.37 to 21.79)	5.62<br>(4.52 to 6.67)	23.07<br>(18.57 to 27.38)
Yunnan	Non-rheumatic valvular heart disease	7.63<br>(5.72 to 9.84)	19.58<br>(14.67 to 25.25)	10.5<br>(8.74 to 12.4)	21.42<br>(17.78 to 25.24)
Zhejiang	Non-rheumatic valvular heart disease	4.18<br>(2.82 to 6.86)	9.49<br>(6.4 to 15.56)	6.33<br>(4.93 to 10.2)	10.35<br>(8.06 to 16.61)
China	Other cardiovascular and circulatory diseases	769<br>(632 to 1000)	64.24<br>(52.78 to 83.64)	1100<br>(935 to 1300)	77.88<br>(66.2 to 91.81)
Taiwan	Other cardiovascular and circulatory diseases	50.9<br>(43.8 to 59.1)	249.53<br>(214.65 to 289.68)	54.2<br>(42 to 68.7)	229.98<br>(178.11 to 291.44)
Hong Kong Special Administrative Region of China	Other cardiovascular and circulatory diseases	4<br>(3.2 to 5.01)	69.43<br>(55.51 to 86.92)	8.15<br>(6.29 to 10.4)	108.52<br>(83.74 to 138.68)
Macau Special Administrative Region of China	Other cardiovascular and circulatory diseases	0.615<br>(0.316 to 0.778)	173.18<br>(89.17 to 219.23)	1.16<br>(0.699 to 1.56)	169.36<br>(102.25 to 228.13)
Anhui	Other cardiovascular and circulatory diseases	35.5<br>(25.5 to 55.6)	59.89<br>(43.05 to 93.85)	38.3<br>(30.9 to 47.9)	62.65<br>(50.46 to 78.3)
Beijing	Other cardiovascular and circulatory diseases	8.54<br>(6.8 to 10.5)	76.15<br>(60.62 to 93.52)	19<br>(14.5 to 25)	78.77<br>(59.98 to 103.64)
Chongqing	Other cardiovascular and circulatory diseases	8.32<br>(6.13 to 12.7)	52.71<br>(38.85 to 80.33)	17.3<br>(14.1 to 21.1)	64.58<br>(52.62 to 78.87)
Fujian	Other cardiovascular and circulatory diseases	13.9<br>(10.4 to 20.5)	43.93<br>(32.86 to 64.75)	21.2<br>(16.8 to 26.9)	53.77<br>(42.63 to 68.32)
Gansu	Other cardiovascular and circulatory diseases	23.4<br>(17.9 to 30.6)	99.03<br>(75.79 to 129.53)	21.9<br>(16 to 26.7)	82.47<br>(60.18 to 100.42)
Guangdong	Other cardiovascular and circulatory diseases	32.7<br>(25.5 to 47.3)	49.65<br>(38.73 to 71.93)	57.9<br>(46.7 to 72.2)	49.59<br>(39.99 to 61.78)
Guangxi	Other cardiovascular and circulatory diseases	18.9<br>(14.5 to 29.7)	43.57<br>(33.44 to 68.21)	27<br>(22.1 to 34.5)	55.89<br>(45.83 to 71.31)

Guizhou	Other cardiovascular and circulatory diseases	16.8 (12.4 to 27.9)	50.52 (37.29 to 83.94)	21.4 (17.7 to 25.9)	63.23 (52.24 to 76.52)
Hainan	Other cardiovascular and circulatory diseases	2.99 (2.28 to 4.47)	42.96 (32.71 to 64.16)	4.53 (3.51 to 5.95)	47.29 (36.64 to 62.08)
Hebei	Other cardiovascular and circulatory diseases	55.5 (47.2 to 65.2)	87.16 (74.12 to 102.49)	99.5 (65.9 to 120)	130.16 (86.25 to 157.5)
Heilongjiang	Other cardiovascular and circulatory diseases	27.7 (23.2 to 32.8)	76.05 (63.52 to 90.1)	47.8 (35.5 to 59.3)	121.66 (90.5 to 151.1)
Henan	Other cardiovascular and circulatory diseases	59.8 (47 to 81.5)	67.79 (53.22 to 92.37)	76.3 (62.7 to 91.1)	79.23 (65.08 to 94.64)
Hubei	Other cardiovascular and circulatory diseases	30.1 (22.8 to 45.8)	54.13 (40.97 to 82.43)	38.9 (32 to 47.8)	70.26 (57.78 to 86.41)
Hunan	Other cardiovascular and circulatory diseases	41 (31.7 to 54.8)	65.92 (50.9 to 88.18)	63.3 (51.7 to 74.8)	94.25 (77.03 to 111.43)
Inner Mongolia	Other cardiovascular and circulatory diseases	17.5 (13.4 to 23.7)	76.75 (58.8 to 104.19)	21.4 (16.9 to 25.8)	82.56 (65.37 to 99.62)
Jiangsu	Other cardiovascular and circulatory diseases	37.9 (30.3 to 47.7)	55.06 (44.07 to 69.34)	60.2 (48.6 to 75.1)	73.67 (59.52 to 92.03)
Jiangxi	Other cardiovascular and circulatory diseases	38.3 (29.4 to 48.5)	96.82 (74.4 to 122.74)	37 (27 to 45.2)	77.85 (56.89 to 95.18)
Jilin	Other cardiovascular and circulatory diseases	20.2 (16.6 to 23.9)	77.95 (64.1 to 92.18)	31.6 (22.3 to 39.6)	113.25 (80.04 to 141.99)
Liaoning	Other cardiovascular and circulatory diseases	25.3 (19.8 to 32.6)	61.93 (48.53 to 79.82)	44.9 (35.9 to 55.6)	100.5 (80.31 to 124.52)
Ningxia	Other cardiovascular and circulatory diseases	4.55 (3.22 to 6.54)	92.05 (65.12 to 132.35)	4.68 (3.76 to 5.73)	66.63 (53.51 to 81.61)
Qinghai	Other cardiovascular and circulatory diseases	4.65 (3.61 to 5.94)	98.07 (76.32 to 125.4)	5.28 (4.05 to 6.4)	84 (64.46 to 101.81)
Shaanxi	Other cardiovascular and circulatory diseases	22 (17.9 to 27.2)	63.82 (52.02 to 79.03)	32.4 (25.3 to 40.3)	81.92 (64.08 to 102.07)
Shandong	Other cardiovascular and circulatory diseases	52.1 (40.2 to 71.3)	60.86 (46.92 to 83.34)	80.1 (65.7 to 96.8)	80.6 (66.07 to 97.45)
Shanghai	Other cardiovascular and circulatory diseases	11.1 (8.82 to 14)	79.63 (63.15 to 100.32)	24.2 (18 to 32.1)	86.75 (64.54 to 114.96)
Shanxi	Other cardiovascular and circulatory diseases	22.3 (17.9 to 28.6)	73.73 (59.15 to 94.43)	29.4 (23 to 35.9)	76.1 (59.46 to 92.82)
Sichuan	Other cardiovascular and circulatory diseases	58.6 (44.8 to 88.1)	52.55 (40.13 to 78.92)	56.4 (47.1 to 68.4)	67.54 (56.42 to 81.94)
Tianjin	Other cardiovascular and circulatory diseases	6.5 (5.1 to 8.67)	70.64 (55.5 to 94.25)	11.8 (9.02 to 15.1)	77.28 (59.12 to 98.76)
Tibet	Other cardiovascular and circulatory diseases	3.4 (2.54 to 4.49)	145.9 (109.09 to 192.55)	4.13 (2.32 to 5.18)	119.39 (67.05 to 149.82)
Xinjiang	Other cardiovascular and circulatory diseases	17.2 (13.9 to 21.2)	109.01 (88.35 to 134.62)	25.5 (18.3 to 30.4)	104.63 (75.2 to 124.58)
Yunnan	Other cardiovascular and circulatory diseases	25.4 (18.8 to 38.8)	65.12 (48.3 to 99.59)	32.5 (27 to 37.8)	66.01 (55.01 to 76.79)
Zhejiang	Other cardiovascular and circulatory diseases	22.3 (16.4 to 34.1)	50.51 (37.16 to 77.4)	35.2 (27.7 to 44.5)	57.56 (45.35 to 72.67)
China	Chronic respiratory diseases	30100 (27200 to 31600)	2512 (2273.86 to 2643)	23100 (21600 to 24900)	1637.85 (1526.75 to 1761.77)
Taiwan	Chronic respiratory diseases	220 (197 to 244)	1080.12 (965.81 to 1193.49)	403 (353 to 448)	1707.69 (1495.52 to 1898.73)
Hong Kong Special Administrative Region of China	Chronic respiratory diseases	83.7 (74.9 to 96.7)	1451.38 (1298.58 to 1677.39)	98.4 (85.3 to 120)	1310.42 (1136.09 to 1603.6)
Macau Special Administrative Region of China	Chronic respiratory diseases	5.47 (5.02 to 5.87)	1541 (1414.73 to 1655.1)	7.67 (6.83 to 8.54)	1121.45 (998.39 to 1248.16)
Anhui	Chronic respiratory diseases	1380 (1240 to 1500)	2325.74 (2085.94 to 2530.76)	923 (817 to 1070)	1509.86 (1335.71 to 1751.39)
Beijing	Chronic respiratory diseases	175 (158 to 194)	1560.26 (1406.67 to 1729.41)	205 (180 to 231)	851.04 (745.09 to 958.95)
Chongqing	Chronic respiratory diseases	617 (518 to 687)	3907.08 (3284.96 to 4351.33)	768 (661 to 890)	2872.76 (2472.78 to 3330.55)
Fujian	Chronic respiratory diseases	671 (580 to 734)	2121.19 (1833.06 to 2319.29)	451 (398 to 508)	1144.93 (1008.92 to 1289.3)
Gansu	Chronic respiratory diseases	899 (782 to 975)	3800.89 (3304.5 to 4122.01)	633 (553 to 712)	2382.18 (2082.29 to 2678.48)

Guangdong	Chronic respiratory diseases	1610<br>(1410 to 1800)	2449.51<br>(2136.95 to 2731.91)	1350<br>(1210 to 1510)	1156.32<br>(1035.28 to 1295.63)
Guangxi	Chronic respiratory diseases	1070<br>(929 to 1190)	2465.32<br>(2136.65 to 2741.96)	785<br>(698 to 878)	1625.49<br>(1443.86 to 1816.65)
Guizhou	Chronic respiratory diseases	1030<br>(916 to 1150)	3097.42<br>(2760.96 to 3449.89)	834<br>(732 to 927)	2466.04<br>(2164.86 to 2740.38)
Hainan	Chronic respiratory diseases	126<br>(110 to 140)	1801.75<br>(1585.23 to 2004.05)	115<br>(99.8 to 133)	1198.81<br>(1040.4 to 1382.28)
Hebei	Chronic respiratory diseases	926<br>(853 to 1000)	1455.6<br>(1339.9 to 1577.08)	945<br>(822 to 1120)	1236.81<br>(1075.58 to 1463)
Heilongjiang	Chronic respiratory diseases	607<br>(543 to 682)	1666.19<br>(1488.69 to 1870.6)	529<br>(460 to 673)	1347.72<br>(1172.85 to 1715.37)
Henan	Chronic respiratory diseases	1630<br>(1490 to 1760)	1846.36<br>(1690.61 to 1992.73)	1280<br>(1120 to 1540)	1325.57<br>(1160.8 to 1600.66)
Hubei	Chronic respiratory diseases	1160<br>(1050 to 1250)	2079.18<br>(1884.37 to 2241.16)	1090<br>(964 to 1220)	1963.16<br>(1743.88 to 2204.25)
Hunan	Chronic respiratory diseases	1930<br>(1610 to 2120)	3108.27<br>(2584.92 to 3407.39)	1540<br>(1370 to 1720)	2292.72<br>(2043.31 to 2564.01)
Inner Mongolia	Chronic respiratory diseases	488<br>(438 to 534)	2143.86<br>(1927.33 to 2349.21)	365<br>(323 to 409)	1408.82<br>(1248.73 to 1581.11)
Jiangsu	Chronic respiratory diseases	1940<br>(1730 to 2100)	2812.61<br>(2507.06 to 3052.03)	1400<br>(1250 to 1570)	1720.18<br>(1531.58 to 1922.29)
Jiangxi	Chronic respiratory diseases	1430<br>(1240 to 1560)	3621<br>(3141.62 to 3951.15)	782<br>(702 to 875)	1644.92<br>(1477.01 to 1839.78)
Jilin	Chronic respiratory diseases	312<br>(278 to 367)	1203.34<br>(1071.95 to 1412.82)	271<br>(233 to 352)	972.87<br>(833.66 to 1263.09)
Liaoning	Chronic respiratory diseases	669<br>(588 to 752)	1640.51<br>(1440.82 to 1843.68)	575<br>(492 to 725)	1287.07<br>(1102.48 to 1624.47)
Ningxia	Chronic respiratory diseases	87<br>(76.9 to 97)	1760.65<br>(1556.91 to 1963.35)	87.1<br>(75.5 to 98.9)	1241.66<br>(1075.65 to 1409.66)
Qinghai	Chronic respiratory diseases	134<br>(113 to 150)	2833.79<br>(2376.45 to 3164.72)	129<br>(113 to 145)	2053.08<br>(1791.41 to 2305.18)
Shaanxi	Chronic respiratory diseases	462<br>(423 to 500)	1341.67<br>(1229.57 to 1453.39)	490<br>(428 to 573)	1239.23<br>(1083.01 to 1450.16)
Shandong	Chronic respiratory diseases	2610<br>(2300 to 2880)	3044.83<br>(2681.24 to 3366.28)	1540<br>(1350 to 1770)	1544.62<br>(1357.42 to 1781.91)
Shanghai	Chronic respiratory diseases	313<br>(277 to 348)	2237.6<br>(1983.29 to 2490.65)	282<br>(249 to 317)	1009.17<br>(889.59 to 1134.2)
Shanxi	Chronic respiratory diseases	615<br>(557 to 671)	2029.58<br>(1838.6 to 2214.61)	408<br>(350 to 488)	1055.81<br>(905.59 to 1263.25)
Sichuan	Chronic respiratory diseases	3590<br>(3090 to 3890)	3220.04<br>(2771.49 to 3488.76)	2660<br>(2310 to 2990)	3185.77<br>(2766.11 to 3583.18)
Tianjin	Chronic respiratory diseases	177<br>(159 to 195)	1923.6<br>(1730.35 to 2122.35)	151<br>(131 to 174)	986.32<br>(856.13 to 1139.13)
Tibet	Chronic respiratory diseases	96.1<br>(79 to 109)	4125.21<br>(3392.12 to 4683.24)	67.3<br>(57.7 to 77.2)	1948.42<br>(1669.14 to 2234.77)
Xinjiang	Chronic respiratory diseases	384<br>(316 to 421)	2434.87<br>(2000.49 to 2667.76)	486<br>(413 to 554)	1994.68<br>(1696.57 to 2274.9)
Yunnan	Chronic respiratory diseases	1470<br>(1270 to 1640)	3783.68<br>(3251.84 to 4219.15)	1150<br>(1020 to 1300)	2345.41<br>(2069.55 to 2643.68)
Zhejiang	Chronic respiratory diseases	1370<br>(1140 to 1500)	3110.92<br>(2583.39 to 3392.15)	741<br>(656 to 828)	1211.16<br>(1072.62 to 1353.91)
China	Chronic obstructive pulmonary disease	26900<br>(24300 to 28300)	2250.82<br>(2030.25 to 2361.38)	20400<br>(19200 to 21800)	1445.53<br>(1356.18 to 1546.89)
Taiwan	Chronic obstructive pulmonary disease	144<br>(128 to 158)	704.07<br>(629.42 to 773.72)	328<br>(288 to 366)	1392.54<br>(1221.36 to 1552.73)
Hong Kong Special Administrative Region of China	Chronic obstructive pulmonary disease	70.7<br>(63.4 to 82.7)	1226.76<br>(1099.53 to 1435.1)	83.6<br>(72.5 to 104)	1113.1<br>(965.34 to 1378.57)
Macau Special Administrative Region of China	Chronic obstructive pulmonary disease	4.74<br>(4.39 to 5.08)	1334.28<br>(1236 to 1432.4)	6.55<br>(5.87 to 7.26)	958.42<br>(857.8 to 1061.31)
Anhui	Chronic obstructive pulmonary disease	1220<br>(1090 to 1330)	2063.64<br>(1840.88 to 2251.17)	807<br>(714 to 944)	1318.96<br>(1167.06 to 1544.25)
Beijing	Chronic obstructive pulmonary disease	145<br>(131 to 160)	1290.69<br>(1167.2 to 1425.88)	154<br>(134 to 179)	640.3<br>(553.97 to 741.35)
Chongqing	Chronic obstructive pulmonary disease	571<br>(477 to 637)	3616.36<br>(3020.53 to 4037.42)	712<br>(612 to 829)	2662.15<br>(2291.46 to 3099.95)

Fujian	Chronic obstructive pulmonary disease	583 (496 to 637)	1842.54 (1567.68 to 2012.27)	373 (329 to 420)	945.46 (834.15 to 1065.54)
Gansu	Chronic obstructive pulmonary disease	827 (712 to 898)	3496.47 (3009.18 to 3798.71)	579 (505 to 651)	2177.61 (1900.26 to 2450.54)
Guangdong	Chronic obstructive pulmonary disease	1450 (1250 to 1620)	2203.53 (1902.23 to 2461.88)	1170 (1050 to 1320)	1003.65 (897.85 to 1125.88)
Guangxi	Chronic obstructive pulmonary disease	929 (795 to 1040)	2135.07 (1827.06 to 2394.53)	684 (609 to 768)	1415.69 (1260.28 to 1590.04)
Guizhou	Chronic obstructive pulmonary disease	878 (780 to 977)	2644.97 (2351.29 to 2943.76)	746 (659 to 833)	2205.06 (1948.27 to 2461.48)
Hainan	Chronic obstructive pulmonary disease	106 (93.4 to 118)	1526.5 (1340.47 to 1691.85)	99.2 (85.8 to 115)	1034.36 (894.54 to 1201.16)
Hebei	Chronic obstructive pulmonary disease	800 (739 to 862)	1256.4 (1161.15 to 1354.32)	811 (708 to 971)	1060.89 (926.5 to 1270.68)
Heilongjiang	Chronic obstructive pulmonary disease	546 (486 to 613)	1496.57 (1332.27 to 1682.06)	465 (403 to 604)	1185.23 (1026.83 to 1538.41)
Henan	Chronic obstructive pulmonary disease	1470 (1350 to 1580)	1660.45 (1528.76 to 1792.65)	1110 (973 to 1370)	1148.76 (1010.35 to 1420.88)
Hubei	Chronic obstructive pulmonary disease	942 (855 to 1030)	1696.27 (1538.96 to 1858.26)	959 (846 to 1080)	1733.77 (1529.56 to 1947.52)
Hunan	Chronic obstructive pulmonary disease	1760 (1450 to 1940)	2828.92 (2337.46 to 3112.33)	1380 (1230 to 1560)	2061.94 (1835.78 to 2323.72)
Inner Mongolia	Chronic obstructive pulmonary disease	439 (395 to 483)	1929.84 (1739.12 to 2126.1)	323 (286 to 367)	1249.18 (1103.15 to 1415.49)
Jiangsu	Chronic obstructive pulmonary disease	1760 (1550 to 1920)	2553.47 (2256.03 to 2783.6)	1240 (1100 to 1390)	1523.61 (1347.48 to 1707.97)
Jiangxi	Chronic obstructive pulmonary disease	1290 (1100 to 1410)	3260.68 (2791.87 to 3557.94)	680 (609 to 758)	1429.64 (1281.28 to 1594.34)
Jilin	Chronic obstructive pulmonary disease	263 (233 to 313)	1011.83 (898.57 to 1204.35)	225 (193 to 300)	806.07 (690.95 to 1075.56)
Liaoning	Chronic obstructive pulmonary disease	576 (507 to 652)	1412.86 (1242.29 to 1598.37)	481 (410 to 626)	1077.75 (918.12 to 1402.65)
Ningxia	Chronic obstructive pulmonary disease	73.9 (64.8 to 82.3)	1495.41 (1311.81 to 1666.46)	73.7 (64.2 to 83.6)	1050.16 (914.07 to 1191.46)
Qinghai	Chronic obstructive pulmonary disease	120 (98.9 to 135)	2540.27 (2088.26 to 2845.01)	116 (102 to 131)	1853.91 (1617.8 to 2084.28)
Shaanxi	Chronic obstructive pulmonary disease	394 (361 to 426)	1145.49 (1047.89 to 1237.67)	413 (358 to 486)	1044.68 (905 to 1230.96)
Shandong	Chronic obstructive pulmonary disease	2430 (2120 to 2690)	2836.93 (2481.97 to 3141.98)	1360 (1200 to 1570)	1372.8 (1204.67 to 1578.51)
Shanghai	Chronic obstructive pulmonary disease	285 (252 to 317)	2036.82 (1802.64 to 2267.37)	239 (212 to 271)	855.4 (759.19 to 970.68)
Shanxi	Chronic obstructive pulmonary disease	550 (502 to 596)	1817.15 (1658.09 to 1969.52)	344 (294 to 420)	890 (760.03 to 1086.48)
Sichuan	Chronic obstructive pulmonary disease	3320 (2820 to 3600)	2973.63 (2525.71 to 3224.04)	2470 (2140 to 2790)	2960.45 (2558.43 to 3340.52)
Tianjin	Chronic obstructive pulmonary disease	150 (135 to 166)	1631.38 (1467.4 to 1800.68)	120 (104 to 142)	785.43 (679.74 to 931.71)
Tibet	Chronic obstructive pulmonary disease	85.5 (68.3 to 97.9)	3667.67 (2930.69 to 4201.32)	54.6 (47 to 62.9)	1579.09 (1359.28 to 1820.95)
Xinjiang	Chronic obstructive pulmonary disease	334 (269 to 368)	2120.59 (1707.72 to 2331.78)	433 (367 to 495)	1776.78 (1506.77 to 2033.21)
Yunnan	Chronic obstructive pulmonary disease	1290 (1100 to 1440)	3319.4 (2824.24 to 3706.61)	1040 (916 to 1170)	2120.01 (1863.31 to 2385.49)
Zhejiang	Chronic obstructive pulmonary disease	1280 (1050 to 1400)	2913.67 (2383.84 to 3179.5)	652 (579 to 729)	1065.22 (945.86 to 1191.84)
China	Pneumoconiosis	251 (200 to 310)	20.93 (16.67 to 25.88)	248 (225 to 273)	17.53 (15.95 to 19.31)
Taiwan	Pneumoconiosis	2.76 (2.48 to 3.06)	13.51 (12.16 to 15.01)	4.35 (3.84 to 4.91)	18.43 (16.27 to 20.83)
Hong Kong Special Administrative Region of China	Pneumoconiosis	1.32 (1.07 to 1.6)	22.87 (18.61 to 27.76)	1.07 (0.848 to 1.35)	14.2 (11.29 to 17.95)
Macau Special Administrative Region of China	Pneumoconiosis	0.0687 (0.0569 to 0.0831)	19.36 (16.03 to 23.41)	0.0689 (0.0526 to 0.0899)	10.08 (7.69 to 13.15)
Anhui	Pneumoconiosis	6.5 (4.34 to 12)	10.96 (7.32 to 20.28)	5.16 (4.17 to 6.52)	8.45 (6.81 to 10.66)

Beijing	Pneumoconiosis	5.12 (4.09 to 6.45)	45.65 (36.5 to 57.48)	7.47 (5.96 to 9.25)	31.01 (24.74 to 38.37)
Chongqing	Pneumoconiosis	7.98 (5.91 to 10.8)	50.57 (37.46 to 68.31)	12.9 (10.1 to 16.5)	48.12 (37.77 to 61.8)
Fujian	Pneumoconiosis	5.78 (4.08 to 8.2)	18.26 (12.9 to 25.91)	8.58 (6.64 to 10.9)	21.77 (16.84 to 27.72)
Gansu	Pneumoconiosis	7.68 (5.88 to 9.7)	32.46 (24.85 to 41.03)	5.29 (4.29 to 6.44)	19.91 (16.13 to 24.24)
Guangdong	Pneumoconiosis	6.66 (4.91 to 8.94)	10.12 (7.46 to 13.59)	5.99 (4.72 to 7.32)	5.13 (4.04 to 6.26)
Guangxi	Pneumoconiosis	4.45 (3.32 to 5.72)	10.23 (7.64 to 13.15)	4.56 (3.68 to 5.61)	9.43 (7.62 to 11.6)
Guizhou	Pneumoconiosis	7.86 (5.9 to 10.2)	23.68 (17.77 to 30.66)	6.64 (5.29 to 8.04)	19.62 (15.63 to 23.78)
Hainan	Pneumoconiosis	0.702 (0.531 to 0.882)	10.07 (7.63 to 12.66)	0.584 (0.452 to 0.723)	6.09 (4.71 to 7.54)
Hebei	Pneumoconiosis	11.6 (8.75 to 15.6)	18.2 (13.75 to 24.51)	10.2 (8.11 to 12.8)	13.29 (10.61 to 16.71)
Heilongjiang	Pneumoconiosis	6.05 (4.61 to 7.79)	16.59 (12.64 to 21.38)	6.11 (4.99 to 7.54)	15.58 (12.71 to 19.21)
Henan	Pneumoconiosis	10.4 (7.7 to 14)	11.74 (8.72 to 15.87)	10.2 (8.24 to 12.4)	10.55 (8.56 to 12.83)
Hubei	Pneumoconiosis	8.73 (6.46 to 11.1)	15.71 (11.64 to 19.97)	10.9 (8.72 to 13.5)	19.66 (15.78 to 24.46)
Hunan	Pneumoconiosis	17.6 (12.9 to 25.2)	28.27 (20.79 to 40.57)	21.6 (16.8 to 27)	32.21 (24.95 to 40.2)
Inner Mongolia	Pneumoconiosis	3.18 (2.37 to 4.27)	14 (10.4 to 18.79)	2.84 (2.32 to 3.45)	10.98 (8.95 to 13.33)
Jiangsu	Pneumoconiosis	9.55 (7.2 to 12.7)	13.88 (10.46 to 18.39)	7.54 (6.12 to 9.18)	9.23 (7.49 to 11.24)
Jiangxi	Pneumoconiosis	19.7 (14.9 to 24.9)	49.81 (37.78 to 63.05)	16.9 (13.8 to 20.8)	35.57 (29.09 to 43.84)
Jilin	Pneumoconiosis	5.3 (4.15 to 6.73)	20.42 (16.01 to 25.92)	3.32 (2.66 to 4.04)	11.91 (9.55 to 14.47)
Liaoning	Pneumoconiosis	14.3 (11.1 to 18.6)	35.1 (27.23 to 45.54)	13.6 (11.1 to 16.4)	30.51 (24.75 to 36.79)
Ningxia	Pneumoconiosis	1.23 (0.873 to 1.64)	24.85 (17.68 to 33.17)	1.27 (0.963 to 1.63)	18.03 (13.72 to 23.19)
Qinghai	Pneumoconiosis	1.04 (0.776 to 1.4)	22.01 (16.38 to 29.56)	1.12 (0.852 to 1.43)	17.83 (13.56 to 22.79)
Shaanxi	Pneumoconiosis	9.87 (7.24 to 16.8)	28.69 (21.05 to 48.89)	10.2 (7.93 to 12.9)	25.83 (20.06 to 32.61)
Shandong	Pneumoconiosis	11.5 (8.66 to 15.5)	13.48 (10.12 to 18.09)	8.91 (7.21 to 10.9)	8.97 (7.25 to 10.93)
Shanghai	Pneumoconiosis	1.75 (1.35 to 2.2)	12.54 (9.69 to 15.71)	1.81 (1.44 to 2.21)	6.46 (5.17 to 7.91)
Shanxi	Pneumoconiosis	4.7 (3.51 to 6.01)	15.52 (11.59 to 19.84)	4.1 (3.24 to 5.13)	10.61 (8.4 to 13.27)
Sichuan	Pneumoconiosis	37.9 (28 to 54.6)	33.92 (25.12 to 48.94)	35.8 (27.5 to 44.7)	42.91 (32.97 to 53.55)
Tianjin	Pneumoconiosis	1.59 (1.24 to 1.99)	17.32 (13.44 to 21.66)	1.77 (1.42 to 2.15)	11.58 (9.33 to 14.08)
Tibet	Pneumoconiosis	0.41 (0.299 to 0.533)	17.59 (12.82 to 22.86)	0.355 (0.283 to 0.45)	10.26 (8.19 to 13.03)
Xinjiang	Pneumoconiosis	4.84 (3.67 to 6.88)	30.71 (23.27 to 43.65)	5.08 (3.97 to 6.37)	20.86 (16.31 to 26.14)
Yunnan	Pneumoconiosis	9.72 (7.07 to 13.1)	24.94 (18.14 to 33.65)	10.2 (8.23 to 12.7)	20.72 (16.75 to 25.83)
Zhejiang	Pneumoconiosis	5.51 (3.98 to 7.6)	12.49 (9.03 to 17.22)	5.54 (4.4 to 6.93)	9.06 (7.2 to 11.32)
China	Asthma	2290 (1740 to 2900)	191.52 (145.63 to 242.5)	1600 (1170 to 2120)	113.06 (82.96 to 149.91)
Taiwan	Asthma	63.7 (52.3 to 77.2)	312.36 (256.37 to 378.43)	44.3 (32.9 to 58.5)	187.95 (139.6 to 248.21)
Hong Kong Special Administrative Region of China	Asthma	8.42 (6.27 to 11.2)	146.12 (108.72 to 193.61)	8.31 (5.92 to 11.2)	110.68 (78.84 to 149.08)

Macau Special Administrative Region of China	Asthma	0.486 (0.361 to 0.644)	136.81 (101.6 to 181.49)	0.653 (0.45 to 0.906)	95.54 (65.78 to 132.46)
Anhui	Asthma	123 (91.5 to 166)	207.25 (154.44 to 280.13)	77.8 (58 to 103)	127.28 (94.87 to 168.92)
Beijing	Asthma	18.7 (13.9 to 24)	166.74 (124.28 to 213.86)	21 (15.2 to 28.3)	87.12 (63.17 to 117.55)
Chongqing	Asthma	27.6 (20.3 to 35.6)	174.66 (128.46 to 225.78)	27.2 (20 to 36.3)	101.77 (74.74 to 135.8)
Fujian	Asthma	70.6 (53.7 to 89.4)	223.02 (169.67 to 282.69)	51.8 (38.4 to 69)	131.52 (97.46 to 175.06)
Gansu	Asthma	47.7 (35.7 to 62.9)	201.85 (150.74 to 265.87)	30.5 (21.6 to 41.4)	114.59 (81.38 to 155.84)
Guangdong	Asthma	130 (95.2 to 168)	197.61 (144.67 to 255.11)	108 (77 to 147)	92.75 (65.92 to 125.97)
Guangxi	Asthma	120 (89.8 to 150)	276.03 (206.42 to 344.33)	69.4 (52.3 to 89.2)	143.57 (108.28 to 184.69)
Guizhou	Asthma	122 (93.3 to 149)	368.51 (280.97 to 450.31)	58.7 (44.8 to 74.6)	173.53 (132.52 to 220.57)
Hainan	Asthma	15.7 (12 to 19.6)	225.76 (172.01 to 280.66)	10.9 (7.99 to 14.6)	113.83 (83.29 to 151.83)
Hebei	Asthma	94.7 (70.4 to 126)	148.85 (110.56 to 198.61)	86.6 (61.1 to 120)	113.29 (79.97 to 156.87)
Heilongjiang	Asthma	38 (26.5 to 54.4)	104.12 (72.67 to 149.29)	31 (21.1 to 43.8)	79.07 (53.71 to 111.56)
Henan	Asthma	118 (85.5 to 161)	133.96 (96.87 to 182.09)	105 (73 to 145)	109.11 (75.83 to 150.57)
Hubei	Asthma	181 (124 to 235)	326.4 (222.56 to 422.39)	82 (63.4 to 103)	148.33 (114.65 to 186.41)
Hunan	Asthma	116 (85.4 to 151)	187.12 (137.32 to 242.21)	79.2 (57 to 105)	117.94 (84.9 to 157.07)
Inner Mongolia	Asthma	33.1 (24.3 to 44.3)	145.56 (106.74 to 195.03)	22.6 (15.6 to 31.4)	87.24 (60.42 to 121.18)
Jiangsu	Asthma	137 (103 to 177)	199.78 (149.35 to 257.82)	104 (77.1 to 138)	127.7 (94.45 to 169.34)
Jiangxi	Asthma	99 (73.2 to 126)	250.3 (185.2 to 318.36)	56.7 (40.8 to 76.8)	119.21 (85.88 to 161.64)
Jilin	Asthma	31.8 (23 to 43.4)	122.56 (88.72 to 167.21)	25.1 (17.4 to 34.6)	89.95 (62.27 to 124)
Liaoning	Asthma	58.6 (42.6 to 78.4)	143.78 (104.4 to 192.31)	47.2 (33.3 to 64.3)	105.79 (74.61 to 144)
Ningxia	Asthma	9.56 (7.01 to 12.5)	193.55 (142 to 253.65)	8.09 (5.66 to 11.1)	115.21 (80.68 to 157.8)
Qinghai	Asthma	9.49 (6.92 to 12.7)	200.4 (146.12 to 268.44)	6.97 (4.92 to 9.56)	110.97 (78.28 to 152.14)
Shaanxi	Asthma	44.9 (32 to 61.7)	130.55 (92.97 to 179.43)	41.8 (29.4 to 57.3)	105.86 (74.34 to 145.15)
Shandong	Asthma	125 (93.6 to 162)	146.07 (109.31 to 189.59)	99.7 (72.6 to 135)	100.36 (73.04 to 135.89)
Shanghai	Asthma	19.5 (14.5 to 25.2)	139.42 (103.77 to 180.61)	23.9 (17.2 to 32.6)	85.56 (61.42 to 116.67)
Shanxi	Asthma	46.8 (34.4 to 62.2)	154.68 (113.65 to 205.5)	39.7 (27.8 to 54.5)	102.74 (71.94 to 141.19)
Sichuan	Asthma	174 (129 to 234)	155.9 (115.24 to 209.93)	97.8 (69.9 to 133)	117.19 (83.74 to 158.95)
Tianjin	Asthma	21.1 (16 to 26.4)	229.38 (173.77 to 287.1)	17.9 (13.2 to 23.7)	117.47 (86.29 to 154.97)
Tibet	Asthma	6.05 (4.45 to 8.03)	259.84 (191.13 to 344.71)	4.68 (3.34 to 6.53)	135.31 (96.69 to 189.01)
Xinjiang	Asthma	35.2 (26.2 to 46.1)	223.07 (166.01 to 292.17)	30.9 (22.4 to 42.4)	126.82 (91.76 to 173.87)
Yunnan	Asthma	148 (110 to 186)	379.14 (282.31 to 476.29)	69.9 (54.1 to 89)	142.14 (110.07 to 181.05)
Zhejiang	Asthma	60 (44.7 to 78.6)	136.15 (101.26 to 178.22)	51.1 (36.2 to 70.9)	83.45 (59.14 to 115.8)
China	Interstitial lung disease and pulmonary sarcoidosis	110 (81.6 to 153)	9.15 (6.82 to 12.79)	284 (232 to 349)	20.07 (16.41 to 24.73)
Taiwan	Interstitial lung disease and pulmonary sarcoidosis	1.79 (1.41 to 2.38)	8.79 (6.91 to 11.65)	6.53 (4.21 to 8.17)	27.68 (17.84 to 34.62)

Hong Kong Special Administrative Region of China	Interstitial lung disease and pulmonary sarcoidosis	1.19<br>(0.955 to 1.44)	20.7<br>(16.57 to 25.05)	2.14<br>(1.63 to 2.96)	28.54<br>(21.66 to 39.44)
Macau Special Administrative Region of China	Interstitial lung disease and pulmonary sarcoidosis	0.0563<br>(0.0427 to 0.0684)	15.87<br>(12.02 to 19.28)	0.14<br>(0.098 to 0.184)	20.49<br>(14.32 to 26.95)
Anhui	Interstitial lung disease and pulmonary sarcoidosis	4.66<br>(3.07 to 7.09)	7.87<br>(5.19 to 11.96)	9.49<br>(7.31 to 12.8)	15.53<br>(11.95 to 20.9)
Beijing	Interstitial lung disease and pulmonary sarcoidosis	3.47<br>(1.94 to 4.67)	30.98<br>(17.27 to 41.66)	14.7<br>(7.23 to 19.9)	61.05<br>(30 to 82.51)
Chongqing	Interstitial lung disease and pulmonary sarcoidosis	1.28<br>(0.914 to 2.07)	8.13<br>(5.79 to 13.09)	4.49<br>(3.39 to 5.96)	16.81<br>(12.68 to 22.29)
Fujian	Interstitial lung disease and pulmonary sarcoidosis	1.91<br>(1.31 to 2.79)	6.04<br>(4.15 to 8.82)	5.56<br>(4.27 to 7.11)	14.11<br>(10.84 to 18.04)
Gansu	Interstitial lung disease and pulmonary sarcoidosis	2.65<br>(1.88 to 4.06)	11.21<br>(7.94 to 17.16)	5.83<br>(4.21 to 7.47)	21.95<br>(15.85 to 28.09)
Guangdong	Interstitial lung disease and pulmonary sarcoidosis	4.89<br>(3.58 to 6.89)	7.43<br>(5.44 to 10.46)	15<br>(11.5 to 19.4)	12.84<br>(9.84 to 16.61)
Guangxi	Interstitial lung disease and pulmonary sarcoidosis	3.25<br>(2.33 to 4.77)	7.48<br>(5.36 to 10.97)	7.97<br>(6.25 to 9.98)	16.5<br>(12.94 to 20.66)
Guizhou	Interstitial lung disease and pulmonary sarcoidosis	2.76<br>(1.96 to 4.29)	8.31<br>(5.92 to 12.92)	5.64<br>(4.21 to 7.28)	16.67<br>(12.45 to 21.52)
Hainan	Interstitial lung disease and pulmonary sarcoidosis	0.469<br>(0.344 to 0.644)	6.73<br>(4.93 to 9.24)	1.19<br>(0.911 to 1.53)	12.44<br>(9.5 to 15.95)
Hebei	Interstitial lung disease and pulmonary sarcoidosis	5.32<br>(3.95 to 8.21)	8.35<br>(6.21 to 12.9)	14<br>(10.9 to 18.6)	18.35<br>(14.23 to 24.39)
Heilongjiang	Interstitial lung disease and pulmonary sarcoidosis	4.28<br>(3.22 to 5.94)	11.75<br>(8.82 to 16.28)	11.5<br>(9.14 to 14.5)	29.31<br>(23.29 to 36.84)
Henan	Interstitial lung disease and pulmonary sarcoidosis	7.28<br>(5.23 to 11.1)	8.24<br>(5.92 to 12.59)	16.8<br>(12.9 to 23.6)	17.4<br>(13.4 to 24.53)
Hubei	Interstitial lung disease and pulmonary sarcoidosis	4.69<br>(3.42 to 6.39)	8.44<br>(6.16 to 11.5)	12.3<br>(9.35 to 16)	22.22<br>(16.9 to 28.98)
Hunan	Interstitial lung disease and pulmonary sarcoidosis	4.6<br>(3.19 to 6.96)	7.39<br>(5.14 to 11.19)	10.5<br>(7.8 to 14.7)	15.64<br>(11.62 to 21.94)
Inner Mongolia	Interstitial lung disease and pulmonary sarcoidosis	1.92<br>(1.4 to 2.94)	8.42<br>(6.14 to 12.92)	4.75<br>(3.78 to 5.99)	18.35<br>(14.6 to 23.13)
Jiangsu	Interstitial lung disease and pulmonary sarcoidosis	6.47<br>(4.85 to 8.59)	9.4<br>(7.05 to 12.49)	18.2<br>(14.5 to 22.8)	22.29<br>(17.76 to 27.88)
Jiangxi	Interstitial lung disease and pulmonary sarcoidosis	3.06<br>(2.17 to 5.1)	7.75<br>(5.49 to 12.9)	6.41<br>(5.06 to 8.03)	13.49<br>(10.63 to 16.9)
Jilin	Interstitial lung disease and pulmonary sarcoidosis	2.53<br>(1.93 to 3.53)	9.73<br>(7.44 to 13.59)	6.41<br>(5.02 to 8.29)	22.99<br>(18 to 29.72)
Liaoning	Interstitial lung disease and pulmonary sarcoidosis	5.54<br>(4.24 to 7.06)	13.58<br>(10.39 to 17.3)	16<br>(12.2 to 20.2)	35.94<br>(27.4 to 45.22)
Ningxia	Interstitial lung disease and pulmonary sarcoidosis	0.375<br>(0.27 to 0.56)	7.6<br>(5.48 to 11.34)	0.978<br>(0.767 to 1.23)	13.93<br>(10.93 to 17.54)
Qinghai	Interstitial lung disease and pulmonary sarcoidosis	0.455<br>(0.309 to 0.767)	9.6<br>(6.53 to 16.2)	1.05<br>(0.802 to 1.33)	16.72<br>(12.76 to 21.21)
Shaanxi	Interstitial lung disease and pulmonary sarcoidosis	2.76<br>(1.94 to 3.67)	8.02<br>(5.64 to 10.66)	8.33<br>(6.31 to 10.4)	21.08<br>(15.98 to 26.22)
Shandong	Interstitial lung disease and pulmonary sarcoidosis	8.59<br>(6.27 to 12.3)	10.04<br>(7.33 to 14.42)	23.6<br>(18.8 to 29.5)	23.73<br>(18.88 to 29.69)
Shanghai	Interstitial lung disease and pulmonary sarcoidosis	2.64<br>(1.94 to 3.38)	18.88<br>(13.88 to 24.21)	8.71<br>(6.04 to 11.3)	31.17<br>(21.62 to 40.31)
Shanxi	Interstitial lung disease and pulmonary sarcoidosis	2.66<br>(1.98 to 3.9)	8.77<br>(6.54 to 12.89)	6.17<br>(4.77 to 8.19)	15.98<br>(12.34 to 21.21)
Sichuan	Interstitial lung disease and pulmonary sarcoidosis	8.89<br>(6.47 to 13.8)	7.97<br>(5.8 to 12.39)	15<br>(11.4 to 19.5)	17.93<br>(13.7 to 23.35)
Tianjin	Interstitial lung disease and pulmonary sarcoidosis	1.58<br>(1.14 to 2.09)	17.23<br>(12.4 to 22.76)	6.12<br>(3.57 to 8.15)	40.1<br>(23.4 to 53.4)
Tibet	Interstitial lung disease and pulmonary sarcoidosis	0.305<br>(0.19 to 0.66)	13.09<br>(8.17 to 28.33)	0.656<br>(0.459 to 0.843)	18.99<br>(13.27 to 24.4)
Xinjiang	Interstitial lung disease and pulmonary sarcoidosis	1.57<br>(1.08 to 2.22)	9.93<br>(6.83 to 14.09)	5.06<br>(3.48 to 6.41)	20.77<br>(14.3 to 26.33)
Yunnan	Interstitial lung disease and pulmonary sarcoidosis	3.02<br>(2.13 to 4.95)	7.76<br>(5.47 to 12.71)	6.95<br>(5.32 to 9.01)	14.14<br>(10.82 to 18.32)
Zhejiang	Interstitial lung disease and pulmonary sarcoidosis	4.39<br>(3.07 to 6.44)	9.97<br>(6.95 to 14.61)	11.8<br>(9.03 to 15)	19.36<br>(14.75 to 24.47)

China	Other chronic respiratory diseases	474<br>(402 to 550)	39.57<br>(33.6 to 45.91)	588<br>(503 to 672)	41.65<br>(35.6 to 47.57)
Taiwan	Other chronic respiratory diseases	8.44<br>(7.29 to 9.75)	41.39<br>(35.75 to 47.78)	19.1<br>(16.1 to 22.3)	81.1<br>(68.17 to 94.72)
Hong Kong Special Administrative Region of China	Other chronic respiratory diseases	2.01<br>(1.67 to 2.34)	34.94<br>(29.04 to 40.68)	3.3<br>(2.76 to 3.86)	43.89<br>(36.81 to 51.44)
Macau Special Administrative Region of China	Other chronic respiratory diseases	0.123<br>(0.105 to 0.141)	34.69<br>(29.47 to 39.73)	0.252<br>(0.208 to 0.298)	36.91<br>(30.42 to 43.52)
Anhui	Other chronic respiratory diseases	21.3<br>(17.9 to 26.1)	36.02<br>(30.18 to 44.1)	24.2<br>(20.5 to 28.1)	39.65<br>(33.45 to 45.92)
Beijing	Other chronic respiratory diseases	2.94<br>(2.39 to 3.52)	26.2<br>(21.29 to 31.35)	7.61<br>(6.1 to 9.1)	31.57<br>(25.32 to 37.76)
Chongqing	Other chronic respiratory diseases	9.05<br>(7.56 to 10.5)	57.36<br>(47.92 to 66.33)	11.7<br>(9.89 to 13.5)	43.9<br>(36.99 to 50.46)
Fujian	Other chronic respiratory diseases	9.91<br>(8.2 to 11.9)	31.33<br>(25.92 to 37.53)	12.6<br>(10.5 to 14.8)	32.07<br>(26.52 to 37.56)
Gansu	Other chronic respiratory diseases	13.9<br>(11.9 to 16.4)	58.9<br>(50.28 to 69.21)	12.8<br>(10.7 to 14.7)	48.14<br>(40.31 to 55.22)
Guangdong	Other chronic respiratory diseases	20.3<br>(16.9 to 24)	30.81<br>(25.75 to 36.45)	49<br>(40.7 to 57.4)	41.96<br>(34.87 to 49.15)
Guangxi	Other chronic respiratory diseases	15.9<br>(13.3 to 18.7)	36.52<br>(30.51 to 42.92)	19.5<br>(16.5 to 22.3)	40.31<br>(34.23 to 46.18)
Guizhou	Other chronic respiratory diseases	17.2<br>(14.8 to 19.8)	51.95<br>(44.48 to 59.71)	17.3<br>(14.5 to 20.3)	51.15<br>(42.75 to 59.89)
Hainan	Other chronic respiratory diseases	2.28<br>(1.89 to 2.67)	32.68<br>(27.19 to 38.26)	3.08<br>(2.56 to 3.55)	32.08<br>(26.66 to 37.04)
Hebei	Other chronic respiratory diseases	15.1<br>(12.6 to 18.6)	23.79<br>(19.73 to 29.22)	23.7<br>(19.7 to 27.8)	31<br>(25.71 to 36.35)
Heilongjiang	Other chronic respiratory diseases	13.5<br>(11.1 to 17.4)	37.16<br>(30.34 to 47.7)	15.1<br>(12.4 to 17.9)	38.54<br>(31.56 to 45.56)
Henan	Other chronic respiratory diseases	28.2<br>(23.3 to 35.1)	31.97<br>(26.41 to 39.75)	38.3<br>(31.9 to 44.7)	39.75<br>(33.12 to 46.44)
Hubei	Other chronic respiratory diseases	18<br>(14.6 to 21.7)	32.35<br>(26.35 to 39.15)	21.7<br>(18.1 to 25.1)	39.18<br>(32.81 to 45.47)
Hunan	Other chronic respiratory diseases	35.2<br>(30.1 to 39.8)	56.57<br>(48.44 to 64.04)	43.6<br>(32.4 to 50.6)	64.99<br>(48.32 to 75.3)
Inner Mongolia	Other chronic respiratory diseases	10.5<br>(8.74 to 12.3)	46.04<br>(38.45 to 54.01)	11.1<br>(9.44 to 12.9)	43.06<br>(36.46 to 49.68)
Jiangsu	Other chronic respiratory diseases	24.8<br>(20.4 to 29.4)	36.08<br>(29.61 to 42.68)	30.5<br>(25.4 to 35.7)	37.34<br>(31.09 to 43.77)
Jiangxi	Other chronic respiratory diseases	20.7<br>(17.7 to 24)	52.45<br>(44.84 to 60.71)	22.3<br>(18.4 to 25.7)	47<br>(38.71 to 53.98)
Jilin	Other chronic respiratory diseases	10.1<br>(8.49 to 11.8)	38.8<br>(32.72 to 45.39)	11.7<br>(9.85 to 13.8)	41.95<br>(35.32 to 49.62)
Liaoning	Other chronic respiratory diseases	14.4<br>(11.4 to 17.8)	35.2<br>(27.92 to 43.65)	16.6<br>(13.5 to 19.6)	37.09<br>(30.21 to 43.84)
Ningxia	Other chronic respiratory diseases	1.94<br>(1.56 to 2.42)	39.24<br>(31.56 to 49.03)	3.11<br>(2.57 to 3.68)	44.33<br>(36.6 to 52.43)
Qinghai	Other chronic respiratory diseases	2.91<br>(2.41 to 3.5)	61.5<br>(50.98 to 73.97)	3.37<br>(2.86 to 3.89)	53.65<br>(45.52 to 61.92)
Shaanxi	Other chronic respiratory diseases	9.95<br>(8.25 to 12)	28.92<br>(23.99 to 34.81)	16.5<br>(13.8 to 19.1)	41.78<br>(35.05 to 48.38)
Shandong	Other chronic respiratory diseases	32.8<br>(27 to 39.4)	38.31<br>(31.54 to 46.08)	38.5<br>(32 to 45.3)	38.76<br>(32.18 to 45.58)
Shanghai	Other chronic respiratory diseases	4.18<br>(3.44 to 4.9)	29.94<br>(24.62 to 35.06)	8.54<br>(7.02 to 10.1)	30.57<br>(25.14 to 36.32)
Shanxi	Other chronic respiratory diseases	10.1<br>(8.5 to 12.1)	33.46<br>(28.07 to 39.96)	14.1<br>(11.8 to 16.4)	36.48<br>(30.48 to 42.51)
Sichuan	Other chronic respiratory diseases	54.3<br>(45.2 to 63.7)	48.62<br>(40.53 to 57.06)	39.5<br>(33.3 to 45.3)	47.29<br>(39.93 to 54.27)
Tianjin	Other chronic respiratory diseases	2.6<br>(2.1 to 3.19)	28.28<br>(22.87 to 34.66)	4.85<br>(3.88 to 5.85)	31.75<br>(25.4 to 38.31)
Tibet	Other chronic respiratory diseases	3.89<br>(2.6 to 4.79)	167.01<br>(111.47 to 205.77)	7.08<br>(2.7 to 8.6)	204.76<br>(78 to 248.85)
Xinjiang	Other chronic respiratory diseases	7.98<br>(6.78 to 9.22)	50.57<br>(42.97 to 58.42)	12.1<br>(10.1 to 14.1)	49.46<br>(41.43 to 57.7)

Yunnan	Other chronic respiratory diseases	20.4 (17.2 to 24.3)	52.43 (44.22 to 62.39)	23.8 (20.1 to 27.4)	48.39 (40.94 to 55.72)
Zhejiang	Other chronic respiratory diseases	17 (14 to 20.5)	38.64 (31.75 to 46.44)	20.8 (17.2 to 24.3)	34.07 (28.15 to 39.68)
China	Cirrhosis and other chronic liver diseases	5260 (4220 to 5680)	439.18 (352.88 to 474.93)	4600 (4210 to 5700)	325.6 (298.09 to 403.82)
Taiwan	Cirrhosis and other chronic liver diseases	159 (154 to 164)	777.03 (753.13 to 802.77)	180 (168 to 193)	763.04 (713.05 to 819.41)
Hong Kong Special Administrative Region of China	Cirrhosis and other chronic liver diseases	16.4 (13.7 to 18.1)	284.21 (238.47 to 314.84)	19.1 (16 to 22.8)	254.12 (212.61 to 303.28)
Macau Special Administrative Region of China	Cirrhosis and other chronic liver diseases	0.782 (0.691 to 0.881)	220.28 (194.8 to 248.16)	1.39 (1.04 to 1.79)	203.46 (151.37 to 261.91)
Anhui	Cirrhosis and other chronic liver diseases	215 (180 to 254)	362.77 (303.14 to 428.4)	139 (108 to 242)	228.02 (176.72 to 395.12)
Beijing	Cirrhosis and other chronic liver diseases	43.2 (35.9 to 51.1)	385.44 (320.39 to 455.91)	51.5 (42.1 to 64)	213.82 (174.87 to 265.52)
Chongqing	Cirrhosis and other chronic liver diseases	85 (65.8 to 102)	538.33 (417.05 to 644.8)	95.3 (75.8 to 119)	356.58 (283.66 to 445.35)
Fujian	Cirrhosis and other chronic liver diseases	95.7 (78.3 to 109)	302.48 (247.59 to 344.83)	97.2 (77.4 to 138)	246.55 (196.42 to 349.68)
Gansu	Cirrhosis and other chronic liver diseases	169 (110 to 200)	712.64 (464.2 to 843.88)	115 (94.2 to 138)	432.69 (354.56 to 517.87)
Guangdong	Cirrhosis and other chronic liver diseases	287 (215 to 346)	436.13 (326.97 to 526.41)	279 (226 to 362)	238.99 (193.29 to 309.42)
Guangxi	Cirrhosis and other chronic liver diseases	283 (161 to 352)	651 (369.14 to 808.51)	224 (182 to 277)	464.18 (375.73 to 572.89)
Guizhou	Cirrhosis and other chronic liver diseases	283 (127 to 354)	853.84 (381.5 to 1067.69)	228 (135 to 288)	672.65 (399.49 to 850.09)
Hainan	Cirrhosis and other chronic liver diseases	27.4 (21.1 to 35.2)	392.9 (302.72 to 505.15)	34.6 (25.7 to 43.6)	361.25 (267.55 to 454.87)
Hebei	Cirrhosis and other chronic liver diseases	169 (141 to 217)	264.89 (221.67 to 341.05)	203 (152 to 365)	266.02 (199.15 to 476.99)
Heilongjiang	Cirrhosis and other chronic liver diseases	146 (120 to 186)	401.54 (327.95 to 510.84)	156 (124 to 228)	397.84 (315.85 to 581.63)
Henan	Cirrhosis and other chronic liver diseases	253 (217 to 291)	287.1 (245.39 to 329.12)	222 (168 to 401)	230.83 (174.76 to 416.37)
Hubei	Cirrhosis and other chronic liver diseases	214 (185 to 245)	385.52 (332.43 to 440.26)	187 (150 to 281)	337.65 (271.15 to 507.26)
Hunan	Cirrhosis and other chronic liver diseases	332 (238 to 380)	533.5 (383.04 to 611.68)	313 (257 to 386)	465.6 (383.34 to 574.55)
Inner Mongolia	Cirrhosis and other chronic liver diseases	108 (90 to 126)	472.85 (395.73 to 553.63)	89.7 (72.3 to 122)	346.49 (279.41 to 470.72)
Jiangsu	Cirrhosis and other chronic liver diseases	286 (241 to 328)	415.99 (349.76 to 476.05)	193 (154 to 297)	236.92 (188.09 to 363.85)
Jiangxi	Cirrhosis and other chronic liver diseases	266 (192 to 309)	671.7 (485.72 to 780.95)	171 (143 to 204)	359.8 (301.6 to 429.98)
Jilin	Cirrhosis and other chronic liver diseases	122 (102 to 145)	471.3 (392.57 to 557.58)	107 (86.9 to 133)	384.82 (311.61 to 476.82)
Liaoning	Cirrhosis and other chronic liver diseases	162 (133 to 203)	397.38 (325.24 to 497.25)	191 (154 to 260)	427.05 (345.21 to 582.49)
Ningxia	Cirrhosis and other chronic liver diseases	22.4 (15.6 to 26.9)	453.02 (316.12 to 543.97)	21.9 (17.7 to 27.4)	312.45 (252.81 to 390.32)
Qinghai	Cirrhosis and other chronic liver diseases	39.5 (17.2 to 49.1)	833.04 (362.37 to 1036.62)	36.9 (24.6 to 45.8)	587.01 (391.81 to 728.66)
Shaanxi	Cirrhosis and other chronic liver diseases	94.5 (62.4 to 112)	274.68 (181.21 to 326.83)	122 (98.4 to 151)	309.33 (249.06 to 381.63)
Shandong	Cirrhosis and other chronic liver diseases	294 (242 to 394)	343.33 (283.28 to 459.71)	223 (166 to 405)	224.06 (167.26 to 407.53)
Shanghai	Cirrhosis and other chronic liver diseases	55.2 (35.6 to 65.7)	395.37 (254.77 to 470.55)	52.4 (43.3 to 64.9)	187.59 (154.9 to 232.17)
Shanxi	Cirrhosis and other chronic liver diseases	105 (88.5 to 124)	345.73 (292.26 to 410.14)	75.1 (57.7 to 116)	194.35 (149.46 to 300.41)
Sichuan	Cirrhosis and other chronic liver diseases	548 (352 to 641)	490.62 (315.3 to 574.54)	407 (332 to 493)	487.92 (397.74 to 590.22)
Tianjin	Cirrhosis and other chronic liver diseases	32.9 (27.7 to 40.9)	357.61 (301.67 to 444.84)	36.6 (28.8 to 58.6)	239.9 (188.91 to 383.85)

Tibet	Cirrhosis and other chronic liver diseases	23.4<br>(15.4 to 29.4)	1005.15<br>(659.14 to 1263.16)	32.4<br>(14.8 to 42.6)	937.21<br>(428.4 to 1232.91)
Xinjiang	Cirrhosis and other chronic liver diseases	55.4<br>(45 to 64.1)	351.51<br>(285.2 to 406.24)	77.7<br>(60.8 to 106)	318.92<br>(249.6 to 433.71)
Yunnan	Cirrhosis and other chronic liver diseases	259<br>(130 to 318)	664.03<br>(332.79 to 817.11)	259<br>(178 to 323)	526.02<br>(361.11 to 656.3)
Zhejiang	Cirrhosis and other chronic liver diseases	165<br>(139 to 187)	374.44<br>(314.59 to 423.48)	137<br>(110 to 189)	224.19<br>(179.29 to 308.91)
China	Digestive diseases	12900<br>(11300 to 14400)	1080.89<br>(940.68 to 1200.75)	11500<br>(9830 to 13800)	815.39<br>(696.22 to 974.64)
Taiwan	Digestive diseases	246<br>(233 to 263)	1204.55<br>(1140.48 to 1286.69)	296<br>(271 to 328)	1255.65<br>(1149.4 to 1389.21)
Hong Kong Special Administrative Region of China	Digestive diseases	38.6<br>(32.5 to 44.4)	669.87<br>(564.34 to 770.98)	52.4<br>(43.4 to 63.5)	697.76<br>(578.3 to 846.07)
Macau Special Administrative Region of China	Digestive diseases	2.12<br>(1.85 to 2.44)	598.37<br>(520.19 to 687.12)	4.01<br>(3.09 to 5.04)	586.59<br>(452.08 to 737.58)
Anhui	Digestive diseases	547<br>(468 to 649)	923.15<br>(790.52 to 1094.81)	409<br>(324 to 576)	669.3<br>(529.9 to 942.01)
Beijing	Digestive diseases	91.3<br>(78.1 to 106)	814.05<br>(696.45 to 943.57)	132<br>(107 to 161)	546.27<br>(443.49 to 666.59)
Chongqing	Digestive diseases	197<br>(168 to 227)	1247.62<br>(1062.04 to 1437.38)	254<br>(208 to 309)	949.11<br>(776.94 to 1156.56)
Fujian	Digestive diseases	266<br>(233 to 302)	842.04<br>(737.54 to 953.63)	255<br>(210 to 321)	646.64<br>(532.43 to 814.51)
Gansu	Digestive diseases	418<br>(319 to 481)	1765.38<br>(1349.65 to 2034.79)	277<br>(232 to 326)	1040.57<br>(872.13 to 1228.44)
Guangdong	Digestive diseases	668<br>(544 to 777)	1014.32<br>(827.25 to 1180.95)	740<br>(605 to 918)	633.32<br>(517.93 to 785.42)
Guangxi	Digestive diseases	619<br>(420 to 733)	1423.31<br>(965.76 to 1685.45)	503<br>(420 to 601)	1040<br>(869.92 to 1243.62)
Guizhou	Digestive diseases	721<br>(430 to 858)	2173.18<br>(1296.98 to 2584.11)	538<br>(374 to 651)	1589.99<br>(1106.06 to 1925.42)
Hainan	Digestive diseases	63.6<br>(53.4 to 76)	913.57<br>(766.74 to 1091.64)	72.5<br>(57.9 to 87.1)	756.13<br>(603.7 to 908.51)
Hebei	Digestive diseases	473<br>(402 to 585)	743.65<br>(631.61 to 919.31)	525<br>(417 to 766)	687.01<br>(545.38 to 1002.31)
Heilongjiang	Digestive diseases	273<br>(226 to 348)	749.99<br>(619.37 to 955.47)	304<br>(244 to 412)	773.91<br>(622.84 to 1050.22)
Henan	Digestive diseases	679<br>(586 to 813)	769.14<br>(663.38 to 921.05)	606<br>(475 to 890)	629.18<br>(493.5 to 923.79)
Hubei	Digestive diseases	557<br>(484 to 639)	1003.4<br>(870.87 to 1149.59)	538<br>(422 to 692)	973.67<br>(762.49 to 1251.79)
Hunan	Digestive diseases	769<br>(631 to 865)	1236.86<br>(1014.25 to 1391.73)	721<br>(608 to 844)	1074.14<br>(905.13 to 1256.78)
Inner Mongolia	Digestive diseases	242<br>(208 to 274)	1064.09<br>(916.66 to 1202.72)	204<br>(166 to 250)	789.05<br>(642.19 to 966.54)
Jiangsu	Digestive diseases	630<br>(553 to 712)	915.37<br>(803.46 to 1033.96)	539<br>(438 to 711)	659.57<br>(536.35 to 870.3)
Jiangxi	Digestive diseases	589<br>(492 to 664)	1489.35<br>(1243.96 to 1678.44)	404<br>(338 to 482)	850.21<br>(711.87 to 1013.08)
Jilin	Digestive diseases	246<br>(214 to 283)	949.57<br>(824.4 to 1092.11)	224<br>(184 to 270)	803.16<br>(660.34 to 969.36)
Liaoning	Digestive diseases	330<br>(275 to 400)	807.96<br>(673.93 to 980.37)	391<br>(320 to 494)	876.64<br>(715.77 to 1107.06)
Ningxia	Digestive diseases	55<br>(46.8 to 63.6)	1114.58<br>(947.36 to 1288.39)	49<br>(40.1 to 58.3)	697.82<br>(570.96 to 831.07)
Qinghai	Digestive diseases	97.3<br>(57.2 to 117)	2054.93<br>(1208.59 to 2467.42)	79.1<br>(57.8 to 95.7)	1258.62<br>(920.71 to 1523.98)
Shaanxi	Digestive diseases	279<br>(223 to 322)	809.6<br>(648.5 to 936.32)	289<br>(239 to 349)	730.62<br>(604.78 to 882.44)
Shandong	Digestive diseases	702<br>(599 to 882)	820.11<br>(700.19 to 1030.06)	644<br>(496 to 941)	647.5<br>(498.72 to 946.47)
Shanghai	Digestive diseases	132<br>(97.3 to 154)	947.36<br>(696.26 to 1102.64)	165<br>(135 to 199)	589.6<br>(484.71 to 711.35)
Shanxi	Digestive diseases	285<br>(250 to 328)	941.79<br>(825.32 to 1081.94)	228<br>(183 to 304)	591.29<br>(474.52 to 787.22)

Sichuan	Digestive diseases	1410<br>(1100 to 1640)	1263.73<br>(982.02 to 1466.18)	937<br>(781 to 1100)	1121.72<br>(935.1 to 1316.82)
Tianjin	Digestive diseases	69.6<br>(59.5 to 83.9)	757.19<br>(647.41 to 912.4)	87.7<br>(69.3 to 120)	574.86<br>(453.8 to 786.31)
Tibet	Digestive diseases	46<br>(36.8 to 53.9)	1973.73<br>(1579.24 to 2312.82)	55.6<br>(31.2 to 70.3)	1607.2<br>(903.51 to 2032.47)
Xinjiang	Digestive diseases	179<br>(156 to 203)	1134.62<br>(987.11 to 1285.5)	203<br>(168 to 245)	831.61<br>(687.69 to 1006.43)
Yunnan	Digestive diseases	833<br>(509 to 982)	2138.76<br>(1305.41 to 2520.39)	671<br>(494 to 800)	1363.72<br>(1005.61 to 1626.67)
Zhejiang	Digestive diseases	429<br>(376 to 489)	972.11<br>(852.65 to 1109.47)	419<br>(334 to 525)	684.22<br>(546.04 to 858.03)
China	Appendicitis	184<br>(148 to 205)	15.4<br>(12.32 to 17.12)	58.1<br>(51.5 to 66.3)	4.11<br>(3.65 to 4.69)
Taiwan	Appendicitis	1.67<br>(1.36 to 2.03)	8.17<br>(6.68 to 9.93)	1.71<br>(1.42 to 2.06)	7.24<br>(6.03 to 8.71)
Hong Kong Special Administrative Region of China	Appendicitis	0.412<br>(0.322 to 0.473)	7.15<br>(5.58 to 8.2)	0.322<br>(0.26 to 0.389)	4.29<br>(3.47 to 5.18)
Macau Special Administrative Region of China	Appendicitis	0.0239<br>(0.0202 to 0.0275)	6.73<br>(5.7 to 7.75)	0.0267<br>(0.0203 to 0.0335)	3.91<br>(2.97 to 4.9)
Anhui	Appendicitis	7.33<br>(6.16 to 9.1)	12.38<br>(10.4 to 15.35)	2.07<br>(1.65 to 3.21)	3.38<br>(2.69 to 5.24)
Beijing	Appendicitis	0.815<br>(0.677 to 0.975)	7.26<br>(6.03 to 8.69)	0.482<br>(0.383 to 0.589)	2<br>(1.59 to 2.44)
Chongqing	Appendicitis	2.52<br>(2.01 to 3.03)	15.95<br>(12.75 to 19.22)	1.1<br>(0.903 to 1.32)	4.1<br>(3.38 to 4.94)
Fujian	Appendicitis	3.92<br>(3.29 to 4.55)	12.38<br>(10.39 to 14.38)	1.18<br>(0.982 to 1.44)	3<br>(2.49 to 3.65)
Gansu	Appendicitis	4.65<br>(3.39 to 5.56)	19.66<br>(14.32 to 23.51)	1.23<br>(1.04 to 1.46)	4.62<br>(3.92 to 5.48)
Guangdong	Appendicitis	7.38<br>(6.01 to 8.72)	11.21<br>(9.14 to 13.24)	2.97<br>(2.44 to 3.61)	2.55<br>(2.09 to 3.09)
Guangxi	Appendicitis	8.92<br>(5.87 to 11)	20.51<br>(13.49 to 25.34)	2.37<br>(2 to 2.8)	4.9<br>(4.14 to 5.8)
Guizhou	Appendicitis	17.3<br>(7.98 to 21.8)	52.07<br>(24.04 to 65.82)	4.77<br>(2.69 to 5.98)	14.09<br>(7.96 to 17.68)
Hainan	Appendicitis	1.01<br>(0.776 to 1.28)	14.5<br>(11.14 to 18.43)	0.498<br>(0.382 to 0.603)	5.2<br>(3.99 to 6.29)
Hebei	Appendicitis	3.54<br>(2.89 to 5.75)	5.56<br>(4.55 to 9.03)	1.9<br>(1.4 to 3.5)	2.49<br>(1.83 to 4.58)
Heilongjiang	Appendicitis	2.13<br>(1.63 to 3.74)	5.84<br>(4.47 to 10.25)	1.02<br>(0.793 to 1.81)	2.61<br>(2.02 to 4.61)
Henan	Appendicitis	6.56<br>(5.54 to 8.29)	7.43<br>(6.28 to 9.39)	2.56<br>(1.99 to 4.38)	2.66<br>(2.06 to 4.55)
Hubei	Appendicitis	10.5<br>(8.68 to 12.4)	18.93<br>(15.62 to 22.26)	3.24<br>(2.68 to 4.17)	5.86<br>(4.85 to 7.54)
Hunan	Appendicitis	10.1<br>(8.28 to 12)	16.29<br>(13.32 to 19.34)	3.4<br>(2.76 to 4.05)	5.06<br>(4.11 to 6.03)
Inner Mongolia	Appendicitis	1.94<br>(1.61 to 2.42)	8.54<br>(7.06 to 10.64)	0.706<br>(0.553 to 1.02)	2.73<br>(2.14 to 3.95)
Jiangsu	Appendicitis	5.75<br>(4.91 to 6.73)	8.35<br>(7.13 to 9.79)	2.13<br>(1.72 to 3.01)	2.61<br>(2.1 to 3.69)
Jiangxi	Appendicitis	8.08<br>(6.71 to 9.73)	20.45<br>(16.97 to 24.61)	1.84<br>(1.55 to 2.21)	3.87<br>(3.26 to 4.65)
Jilin	Appendicitis	2.39<br>(1.9 to 3.03)	9.22<br>(7.33 to 11.67)	0.795<br>(0.64 to 1.05)	2.85<br>(2.3 to 3.77)
Liaoning	Appendicitis	3.27<br>(2.54 to 4.36)	8.01<br>(6.22 to 10.68)	1.49<br>(1.19 to 1.99)	3.33<br>(2.67 to 4.45)
Ningxia	Appendicitis	0.658<br>(0.534 to 0.815)	13.33<br>(10.81 to 16.51)	0.186<br>(0.149 to 0.239)	2.65<br>(2.12 to 3.4)
Qinghai	Appendicitis	1.94<br>(1.11 to 2.56)	40.98<br>(23.51 to 53.95)	0.504<br>(0.372 to 0.608)	8.03<br>(5.92 to 9.68)
Shaanxi	Appendicitis	3.02<br>(2.31 to 3.59)	8.78<br>(6.71 to 10.43)	1.18<br>(0.977 to 1.49)	3<br>(2.47 to 3.76)
Shandong	Appendicitis	6.79<br>(5.71 to 9.13)	7.94<br>(6.67 to 10.67)	2.46<br>(1.92 to 4.15)	2.48<br>(1.93 to 4.18)

Shanghai	Appendicitis	1.32<br>(0.992 to 1.6)	9.48<br>(7.1 to 11.48)	0.711<br>(0.586 to 0.854)	2.54<br>(2.1 to 3.06)
Shanxi	Appendicitis	2.99<br>(2.51 to 3.52)	9.87<br>(8.3 to 11.64)	0.991<br>(0.784 to 1.42)	2.57<br>(2.03 to 3.68)
Sichuan	Appendicitis	23.1<br>(15 to 27.9)	20.73<br>(13.4 to 25)	5.23<br>(4.33 to 6.12)	6.26<br>(5.19 to 7.33)
Tianjin	Appendicitis	0.562<br>(0.462 to 0.721)	6.12<br>(5.02 to 7.84)	0.323<br>(0.251 to 0.495)	2.12<br>(1.65 to 3.24)
Tibet	Appendicitis	0.915<br>(0.678 to 1.34)	39.26<br>(29.09 to 57.61)	0.48<br>(0.338 to 0.611)	13.9<br>(9.78 to 17.66)
Xinjiang	Appendicitis	5.05<br>(2.97 to 6.05)	32.02<br>(18.83 to 38.33)	2.21<br>(1.54 to 2.8)	9.06<br>(6.31 to 11.49)
Yunnan	Appendicitis	22.5<br>(10 to 28.9)	57.68<br>(25.72 to 74.04)	5.41<br>(3.3 to 6.52)	11<br>(6.72 to 13.27)
Zhejiang	Appendicitis	6.95<br>(5.94 to 8.25)	15.75<br>(13.47 to 18.7)	2.32<br>(1.92 to 2.82)	3.8<br>(3.14 to 4.61)
China	Paralytic ileus and intestinal obstruction	1270<br>(1140 to 1480)	105.72<br>(95.42 to 123.7)	453<br>(403 to 535)	32.07<br>(28.53 to 37.91)
Taiwan	Paralytic ileus and intestinal obstruction	7.26<br>(5.8 to 8)	35.57<br>(28.42 to 39.21)	9.77<br>(8.45 to 12.4)	41.41<br>(35.82 to 52.39)
Hong Kong Special Administrative Region of China	Paralytic ileus and intestinal obstruction	1.77<br>(1.25 to 2.04)	30.73<br>(21.65 to 35.37)	2.23<br>(1.76 to 3.32)	29.72<br>(23.49 to 44.26)
Macau Special Administrative Region of China	Paralytic ileus and intestinal obstruction	0.096<br>(0.0751 to 0.122)	27.05<br>(21.15 to 34.31)	0.113<br>(0.0779 to 0.149)	16.58<br>(11.39 to 21.84)
Anhui	Paralytic ileus and intestinal obstruction	74.2<br>(51.2 to 120)	125.15<br>(86.35 to 202.58)	18.4<br>(14 to 32.1)	30.03<br>(22.88 to 52.42)
Beijing	Paralytic ileus and intestinal obstruction	5.51<br>(3.76 to 6.8)	49.14<br>(33.5 to 60.65)	5.32<br>(4.06 to 6.56)	22.09<br>(16.84 to 27.21)
Chongqing	Paralytic ileus and intestinal obstruction	20.7<br>(14.3 to 29.4)	131.14<br>(90.55 to 186.43)	8.62<br>(6.95 to 10.8)	32.25<br>(25.99 to 40.38)
Fujian	Paralytic ileus and intestinal obstruction	25.4<br>(19.4 to 32.5)	80.2<br>(61.34 to 102.68)	9.4<br>(7.67 to 12.5)	23.85<br>(19.47 to 31.77)
Gansu	Paralytic ileus and intestinal obstruction	71.7<br>(52.8 to 96.5)	302.93<br>(223.05 to 407.79)	15.1<br>(10.7 to 19.6)	56.84<br>(40.39 to 73.71)
Guangdong	Paralytic ileus and intestinal obstruction	42.2<br>(34 to 52.7)	64.14<br>(51.6 to 80.09)	28.8<br>(23.7 to 35.4)	24.66<br>(20.3 to 30.29)
Guangxi	Paralytic ileus and intestinal obstruction	34.3<br>(26.4 to 43.9)	78.78<br>(60.61 to 101)	15<br>(11.8 to 18.6)	31.04<br>(24.51 to 38.47)
Guizhou	Paralytic ileus and intestinal obstruction	53.1<br>(39.7 to 69.3)	159.95<br>(119.74 to 208.76)	16.1<br>(7.23 to 21.8)	47.45<br>(21.38 to 64.48)
Hainan	Paralytic ileus and intestinal obstruction	5.47<br>(3.7 to 8.11)	78.52<br>(53.06 to 116.37)	3.68<br>(2.75 to 4.87)	38.43<br>(28.64 to 50.8)
Hebei	Paralytic ileus and intestinal obstruction	43.7<br>(31.9 to 65.7)	68.59<br>(50.14 to 103.2)	25.5<br>(19.1 to 35.4)	33.32<br>(24.93 to 46.26)
Heilongjiang	Paralytic ileus and intestinal obstruction	13.5<br>(9.61 to 27.2)	37.02<br>(26.36 to 74.72)	9.83<br>(7.68 to 15.6)	25.04<br>(19.57 to 39.73)
Henan	Paralytic ileus and intestinal obstruction	67.5<br>(44.2 to 126)	76.42<br>(50.04 to 142.58)	22<br>(16.5 to 41.1)	22.89<br>(17.18 to 42.69)
Hubei	Paralytic ileus and intestinal obstruction	49.7<br>(36.8 to 68.2)	89.41<br>(66.17 to 122.77)	15.5<br>(12.5 to 21.8)	28.06<br>(22.69 to 39.47)
Hunan	Paralytic ileus and intestinal obstruction	44.5<br>(34.3 to 60.3)	71.64<br>(55.13 to 96.99)	15.9<br>(13.1 to 20.9)	23.71<br>(19.5 to 31.09)
Inner Mongolia	Paralytic ileus and intestinal obstruction	31.1<br>(23.5 to 40.7)	136.86<br>(103.15 to 179.1)	8.63<br>(6.95 to 11.3)	33.33<br>(26.84 to 43.54)
Jiangsu	Paralytic ileus and intestinal obstruction	42.9<br>(34.1 to 52.7)	62.35<br>(49.48 to 76.55)	21.1<br>(16.5 to 29.7)	25.89<br>(20.17 to 36.31)
Jiangxi	Paralytic ileus and intestinal obstruction	70.9<br>(51.3 to 97.2)	179.39<br>(129.87 to 245.74)	19.3<br>(15.4 to 24.3)	40.61<br>(32.3 to 51.1)
Jilin	Paralytic ileus and intestinal obstruction	21.6<br>(16.5 to 27.7)	83.1<br>(63.73 to 106.59)	7.83<br>(6.32 to 9.72)	28.08<br>(22.66 to 34.86)
Liaoning	Paralytic ileus and intestinal obstruction	17.3<br>(13.6 to 22.3)	42.37<br>(33.29 to 54.75)	12.4<br>(9.27 to 15.4)	27.73<br>(20.77 to 34.4)
Ningxia	Paralytic ileus and intestinal obstruction	10.9<br>(7.52 to 15.5)	220.04<br>(152.33 to 313.68)	2.89<br>(2.26 to 3.78)	41.25<br>(32.27 to 53.91)
Qinghai	Paralytic ileus and intestinal obstruction	15.7<br>(9.62 to 22.5)	330.82<br>(203.1 to 475.64)	4.68<br>(2.29 to 6.55)	74.42<br>(36.46 to 104.28)

Shaanxi	Paralytic ileus and intestinal obstruction	50 (37.5 to 65.2)	145.27 (108.91 to 189.51)	15.3 (11.9 to 19.6)	38.81 (30.2 to 49.59)
Shandong	Paralytic ileus and intestinal obstruction	39.2 (28.5 to 64.8)	45.74 (33.27 to 75.71)	21 (16 to 39.6)	21.16 (16.08 to 39.84)
Shanghai	Paralytic ileus and intestinal obstruction	9.21 (4.72 to 12)	65.95 (33.76 to 85.65)	7.91 (5.23 to 10)	28.32 (18.73 to 35.95)
Shanxi	Paralytic ileus and intestinal obstruction	28.9 (21.8 to 38.7)	95.54 (71.9 to 127.8)	11.5 (8.76 to 16.6)	29.65 (22.68 to 43.08)
Sichuan	Paralytic ileus and intestinal obstruction	184 (132 to 253)	164.93 (118.13 to 226.38)	30.8 (23.9 to 38.2)	36.92 (28.63 to 45.7)
Tianjin	Paralytic ileus and intestinal obstruction	4.31 (3.06 to 5.36)	46.88 (33.27 to 58.3)	3.74 (2.86 to 5.09)	24.53 (18.75 to 33.32)
Tibet	Paralytic ileus and intestinal obstruction	5.52 (4.04 to 7.36)	236.97 (173.52 to 315.68)	4.53 (2.17 to 6.4)	131.18 (62.88 to 185.09)
Xinjiang	Paralytic ileus and intestinal obstruction	30.9 (22.1 to 40)	196.2 (140.33 to 253.43)	17 (11.9 to 23.1)	69.74 (48.97 to 94.95)
Yunnan	Paralytic ileus and intestinal obstruction	110 (63.6 to 154)	283.54 (163.23 to 396.43)	37.1 (15.7 to 52.1)	75.55 (31.9 to 106)
Zhejiang	Paralytic ileus and intestinal obstruction	39.3 (31.3 to 49.1)	89.21 (71.05 to 111.32)	15.7 (13.1 to 19.5)	25.6 (21.37 to 31.82)
China	Inguinal, femoral, and abdominal hernia	537 (369 to 734)	44.85 (30.82 to 61.31)	629 (433 to 858)	44.56 (30.67 to 60.77)
Taiwan	Inguinal, femoral, and abdominal hernia	6.23 (4.54 to 8.29)	30.53 (22.23 to 40.63)	7.31 (5.3 to 9.72)	31.01 (22.48 to 41.24)
Hong Kong Special Administrative Region of China	Inguinal, femoral, and abdominal hernia	1.6 (1.02 to 2.11)	27.69 (17.71 to 36.68)	2.14 (1.51 to 2.92)	28.54 (20.09 to 38.88)
Macau Special Administrative Region of China	Inguinal, femoral, and abdominal hernia	0.0831 (0.0554 to 0.11)	23.43 (15.61 to 30.99)	0.17 (0.118 to 0.227)	24.85 (17.29 to 33.18)
Anhui	Inguinal, femoral, and abdominal hernia	31.3 (21.1 to 43.1)	52.8 (35.59 to 72.77)	33.8 (23.6 to 46.4)	55.33 (38.61 to 75.86)
Beijing	Inguinal, femoral, and abdominal hernia	3 (2.09 to 4.02)	26.78 (18.65 to 35.82)	6.46 (4.45 to 9.04)	26.79 (18.48 to 37.5)
Chongqing	Inguinal, femoral, and abdominal hernia	8.77 (5.95 to 11.9)	55.57 (37.73 to 75.71)	17.4 (11.8 to 23.7)	64.94 (44.25 to 88.85)
Fujian	Inguinal, femoral, and abdominal hernia	19 (13 to 26)	60.01 (40.96 to 82.05)	22 (15.4 to 30.4)	55.87 (38.98 to 77.17)
Gansu	Inguinal, femoral, and abdominal hernia	10.1 (6.78 to 14)	42.65 (28.65 to 59.24)	12.2 (8.22 to 16.6)	45.85 (30.94 to 62.37)
Guangdong	Inguinal, femoral, and abdominal hernia	27.7 (18.9 to 38.3)	42.13 (28.73 to 58.16)	45.1 (30.8 to 62)	38.62 (26.4 to 53.06)
Guangxi	Inguinal, femoral, and abdominal hernia	23.4 (15.7 to 32.2)	53.8 (36.09 to 73.98)	26.4 (18.1 to 36.2)	54.69 (37.37 to 74.93)
Guizhou	Inguinal, femoral, and abdominal hernia	21.9 (15.4 to 29.3)	65.92 (46.48 to 88.33)	22.1 (15.3 to 30.1)	65.35 (45.24 to 89.1)
Hainan	Inguinal, femoral, and abdominal hernia	2.59 (1.71 to 3.62)	37.19 (24.53 to 51.91)	3.4 (2.31 to 4.71)	35.44 (24.07 to 49.07)
Hebei	Inguinal, femoral, and abdominal hernia	24.5 (16.5 to 33.9)	38.48 (25.88 to 53.3)	30 (20.7 to 40.8)	39.32 (27.02 to 53.37)
Heilongjiang	Inguinal, femoral, and abdominal hernia	10.4 (6.9 to 14.8)	28.59 (18.94 to 40.66)	10.1 (7 to 13.7)	25.67 (17.83 to 34.95)
Henan	Inguinal, femoral, and abdominal hernia	40.3 (27.2 to 56.4)	45.67 (30.83 to 63.85)	45.7 (30.9 to 62.4)	47.49 (32.12 to 64.79)
Hubei	Inguinal, femoral, and abdominal hernia	25.5 (17.3 to 35.2)	45.88 (31.11 to 63.28)	27.3 (18.3 to 37.8)	49.41 (33.14 to 68.44)
Hunan	Inguinal, femoral, and abdominal hernia	29 (19.7 to 40.4)	46.68 (31.6 to 65.04)	33.7 (23.5 to 46.3)	50.19 (35.01 to 68.97)
Inner Mongolia	Inguinal, femoral, and abdominal hernia	8.19 (5.51 to 11.4)	36.03 (24.23 to 50.22)	8.42 (5.69 to 11.6)	32.52 (21.96 to 44.62)
Jiangsu	Inguinal, femoral, and abdominal hernia	26.2 (17.7 to 36.8)	38.06 (25.77 to 53.52)	31.2 (21.7 to 42.5)	38.22 (26.58 to 52.08)
Jiangxi	Inguinal, femoral, and abdominal hernia	22.3 (15.5 to 30)	56.31 (39.26 to 75.99)	26.3 (17.9 to 36.1)	55.43 (37.63 to 76)
Jilin	Inguinal, femoral, and abdominal hernia	8.57 (5.76 to 12.1)	33.04 (22.2 to 46.73)	8.09 (5.56 to 11)	28.99 (19.94 to 39.47)
Liaoning	Inguinal, femoral, and abdominal hernia	11.6 (8.01 to 16.2)	28.5 (19.65 to 39.66)	13.7 (9.45 to 18.6)	30.62 (21.17 to 41.76)

Ningxia	Inguinal, femoral, and abdominal hernia	2.18 (1.47 to 3.04)	44.16 (29.72 to 61.48)	2.94 (2.01 to 4.02)	41.88 (28.58 to 57.26)
Qinghai	Inguinal, femoral, and abdominal hernia	1.94 (1.33 to 2.69)	41.03 (28.04 to 56.89)	2.86 (1.95 to 3.95)	45.53 (31.07 to 62.87)
Shaanxi	Inguinal, femoral, and abdominal hernia	14.8 (10.1 to 20.1)	42.96 (29.4 to 58.52)	16.7 (11.3 to 23.3)	42.16 (28.65 to 58.96)
Shandong	Inguinal, femoral, and abdominal hernia	33.1 (22.5 to 45.4)	38.65 (26.32 to 53.02)	39.7 (27.7 to 54.6)	39.95 (27.85 to 54.89)
Shanghai	Inguinal, femoral, and abdominal hernia	3.97 (2.81 to 5.29)	28.44 (20.09 to 37.88)	8.29 (5.82 to 11.3)	29.66 (20.85 to 40.47)
Shanxi	Inguinal, femoral, and abdominal hernia	10.5 (7.05 to 14.8)	34.78 (23.28 to 48.72)	13.3 (9.02 to 18.2)	34.46 (23.36 to 47.1)
Sichuan	Inguinal, femoral, and abdominal hernia	63.4 (43.5 to 86.2)	56.78 (38.98 to 77.27)	51.3 (34.9 to 69.6)	61.41 (41.77 to 83.3)
Tianjin	Inguinal, femoral, and abdominal hernia	2.2 (1.51 to 3.01)	23.88 (16.41 to 32.68)	3.94 (2.68 to 5.4)	25.85 (17.57 to 35.35)
Tibet	Inguinal, femoral, and abdominal hernia	1.17 (0.749 to 1.68)	50.1 (32.14 to 71.92)	1.75 (1.16 to 2.45)	50.53 (33.69 to 70.78)
Xinjiang	Inguinal, femoral, and abdominal hernia	8.31 (5.68 to 11.5)	52.68 (36 to 72.75)	12.4 (8.38 to 17.2)	50.93 (34.41 to 70.45)
Yunnan	Inguinal, femoral, and abdominal hernia	21.1 (14.7 to 29.1)	54.24 (37.59 to 74.62)	25.8 (17.7 to 35.7)	52.54 (36.08 to 72.6)
Zhejiang	Inguinal, femoral, and abdominal hernia	18.1 (12.6 to 24.8)	41.15 (28.64 to 56.23)	24.5 (16.7 to 33.6)	40.12 (27.24 to 54.9)
China	Inflammatory bowel disease	346 (254 to 441)	28.94 (21.21 to 36.82)	502 (380 to 647)	35.56 (26.91 to 45.78)
Taiwan	Inflammatory bowel disease	4.8 (3.73 to 5.97)	23.55 (18.27 to 29.26)	7.81 (5.98 to 10)	33.11 (25.35 to 42.59)
Hong Kong Special Administrative Region of China	Inflammatory bowel disease	0.905 (0.68 to 1.17)	15.71 (11.8 to 20.38)	2.93 (2.06 to 4.05)	39.05 (27.4 to 53.87)
Macau Special Administrative Region of China	Inflammatory bowel disease	0.0494 (0.0382 to 0.0635)	13.91 (10.76 to 17.9)	0.203 (0.14 to 0.279)	29.68 (20.43 to 40.79)
Anhui	Inflammatory bowel disease	15 (11.4 to 20.5)	25.31 (19.21 to 34.58)	18 (12.8 to 23.9)	29.41 (20.97 to 39.03)
Beijing	Inflammatory bowel disease	3.52 (2.53 to 4.75)	31.36 (22.51 to 42.32)	9.11 (6.28 to 12.6)	37.81 (26.06 to 52.44)
Chongqing	Inflammatory bowel disease	3.72 (2.64 to 5.15)	23.6 (16.73 to 32.65)	8.54 (6.1 to 11.5)	31.95 (22.83 to 42.89)
Fujian	Inflammatory bowel disease	6.78 (4.69 to 8.61)	21.42 (14.82 to 27.21)	10.4 (7.7 to 13.9)	26.44 (19.53 to 35.24)
Gansu	Inflammatory bowel disease	6.92 (4.55 to 9.35)	29.26 (19.23 to 39.52)	8.73 (6.29 to 11.8)	32.86 (23.66 to 44.41)
Guangdong	Inflammatory bowel disease	19.6 (14.1 to 25)	29.79 (21.45 to 37.99)	37.7 (26.9 to 51.9)	32.28 (23.01 to 44.39)
Guangxi	Inflammatory bowel disease	14.6 (7.45 to 19.9)	33.65 (17.13 to 45.7)	15.5 (11.9 to 20)	32.1 (24.57 to 41.42)
Guizhou	Inflammatory bowel disease	14.4 (3.91 to 25.5)	43.5 (11.78 to 76.97)	11.9 (6.91 to 15.1)	35.18 (20.42 to 44.63)
Hainan	Inflammatory bowel disease	1.37 (0.938 to 1.9)	19.74 (13.47 to 27.22)	2.55 (1.92 to 3.29)	26.62 (20.03 to 34.3)
Hebei	Inflammatory bowel disease	15.7 (12.1 to 19.6)	24.72 (19.08 to 30.77)	27.2 (19.5 to 36.8)	35.58 (25.46 to 48.21)
Heilongjiang	Inflammatory bowel disease	4.78 (3.47 to 7.44)	13.12 (9.51 to 20.41)	9.66 (6.65 to 13.3)	24.62 (16.95 to 33.81)
Henan	Inflammatory bowel disease	20.2 (15.1 to 26.7)	22.92 (17.1 to 30.28)	31.1 (22 to 42.6)	32.26 (22.84 to 44.23)
Hubei	Inflammatory bowel disease	14 (10.3 to 18.3)	25.13 (18.46 to 32.94)	19.4 (14.2 to 25.7)	35.14 (25.66 to 46.42)
Hunan	Inflammatory bowel disease	24.6 (11.9 to 33.1)	39.57 (19.14 to 53.18)	29.8 (19.8 to 38.4)	44.34 (29.49 to 57.24)
Inner Mongolia	Inflammatory bowel disease	4.74 (3.44 to 6.28)	20.83 (15.13 to 27.6)	7.9 (5.58 to 10.6)	30.5 (21.55 to 41.04)
Jiangsu	Inflammatory bowel disease	24.6 (18.3 to 31.6)	35.75 (26.56 to 45.85)	43.2 (29.9 to 58.7)	52.89 (36.57 to 71.86)
Jiangxi	Inflammatory bowel disease	12.2 (7.22 to 19.2)	30.83 (18.27 to 48.48)	14 (10.4 to 19)	29.5 (21.84 to 40.04)

Jilin	Inflammatory bowel disease	5.39 (4.21 to 6.58)	20.77 (16.22 to 25.35)	9.04 (6.44 to 12.3)	32.43 (23.11 to 44.04)
Liaoning	Inflammatory bowel disease	8.42 (6.2 to 11.1)	20.63 (15.2 to 27.32)	16.8 (11.4 to 24)	37.6 (25.63 to 53.71)
Ningxia	Inflammatory bowel disease	1.21 (0.809 to 1.91)	24.55 (16.38 to 38.66)	2.06 (1.42 to 2.83)	29.35 (20.26 to 40.38)
Qinghai	Inflammatory bowel disease	1.43 (0.669 to 2.36)	30.24 (14.13 to 49.81)	1.63 (1.21 to 2.17)	25.96 (19.22 to 34.51)
Shaanxi	Inflammatory bowel disease	8.48 (5.24 to 11.1)	24.64 (15.22 to 32.35)	10.8 (7.97 to 14.1)	27.29 (20.16 to 35.68)
Shandong	Inflammatory bowel disease	19.1 (14 to 24.8)	22.34 (16.4 to 29.02)	37.1 (25.4 to 50.9)	37.35 (25.57 to 51.17)
Shanghai	Inflammatory bowel disease	7.56 (5.6 to 9.64)	54.12 (40.1 to 68.99)	17.9 (12.5 to 25.6)	63.97 (44.61 to 91.48)
Shanxi	Inflammatory bowel disease	7.22 (5.49 to 8.88)	23.85 (18.14 to 29.33)	12.3 (8.87 to 16.5)	31.92 (22.97 to 42.7)
Sichuan	Inflammatory bowel disease	33.5 (23 to 45.4)	29.97 (20.62 to 40.67)	28.9 (21.4 to 37.3)	34.6 (25.6 to 44.65)
Tianjin	Inflammatory bowel disease	1.69 (1.26 to 2.18)	18.35 (13.73 to 23.74)	4.55 (3.13 to 6.21)	29.79 (20.49 to 40.67)
Tibet	Inflammatory bowel disease	0.606 (0.211 to 0.983)	26.02 (9.07 to 42.19)	1.07 (0.589 to 1.4)	30.97 (17.03 to 40.46)
Xinjiang	Inflammatory bowel disease	4.83 (3.4 to 6.18)	30.64 (21.54 to 39.2)	8.85 (6.32 to 12.4)	36.33 (25.92 to 50.97)
Yunnan	Inflammatory bowel disease	27.9 (4.73 to 46.4)	71.51 (12.14 to 118.97)	22.1 (10.8 to 27.3)	44.96 (21.93 to 55.44)
Zhejiang	Inflammatory bowel disease	11.3 (8.3 to 14.8)	25.71 (18.83 to 33.51)	21.3 (14.9 to 29)	34.86 (24.41 to 47.43)
China	Vascular intestinal disorders	67.8 (51.8 to 80.2)	5.67 (4.33 to 6.7)	111 (101 to 154)	7.86 (7.15 to 10.91)
Taiwan	Vascular intestinal disorders	1.1 (0.994 to 1.74)	5.38 (4.87 to 8.54)	6.39 (4.14 to 7.11)	27.09 (17.53 to 30.13)
Hong Kong Special Administrative Region of China	Vascular intestinal disorders	0.672 (0.326 to 0.796)	11.66 (5.65 to 13.81)	1.08 (0.675 to 1.35)	14.41 (8.99 to 17.94)
Macau Special Administrative Region of China	Vascular intestinal disorders	0.0425 (0.0167 to 0.0498)	11.97 (4.72 to 14.04)	0.0696 (0.0252 to 0.106)	10.17 (3.68 to 15.43)
Anhui	Vascular intestinal disorders	3.87 (2.67 to 5.17)	6.53 (4.5 to 8.72)	5.47 (4.3 to 9.02)	8.95 (7.03 to 14.75)
Beijing	Vascular intestinal disorders	1.01 (0.595 to 1.31)	9 (5.3 to 11.64)	1.71 (1.31 to 2.19)	7.08 (5.42 to 9.09)
Chongqing	Vascular intestinal disorders	0.834 (0.589 to 1.06)	5.29 (3.73 to 6.73)	2.52 (2.02 to 3.18)	9.43 (7.57 to 11.91)
Fujian	Vascular intestinal disorders	1.53 (1 to 2.01)	4.82 (3.17 to 6.34)	2.72 (2.22 to 3.61)	6.91 (5.64 to 9.16)
Gansu	Vascular intestinal disorders	1.16 (0.772 to 1.43)	4.9 (3.26 to 6.06)	1.84 (1.5 to 2.26)	6.91 (5.64 to 8.51)
Guangdong	Vascular intestinal disorders	4.47 (2.98 to 5.83)	6.79 (4.53 to 8.86)	7.15 (5.79 to 9.12)	6.12 (4.96 to 7.81)
Guangxi	Vascular intestinal disorders	2.56 (1.35 to 3.46)	5.88 (3.12 to 7.96)	3.58 (2.92 to 4.32)	7.42 (6.03 to 8.94)
Guizhou	Vascular intestinal disorders	1.6 (0.767 to 2.02)	4.81 (2.31 to 6.08)	2.4 (1.48 to 3)	7.08 (4.37 to 8.88)
Hainan	Vascular intestinal disorders	0.386 (0.268 to 0.487)	5.54 (3.84 to 6.99)	0.564 (0.42 to 0.726)	5.88 (4.38 to 7.57)
Hebei	Vascular intestinal disorders	2.79 (2.27 to 4.06)	4.39 (3.56 to 6.37)	5.11 (3.79 to 9.74)	6.68 (4.96 to 12.74)
Heilongjiang	Vascular intestinal disorders	1.86 (1.32 to 3.04)	5.1 (3.61 to 8.34)	3.65 (2.73 to 6.66)	9.3 (6.96 to 16.96)
Henan	Vascular intestinal disorders	4.2 (3.38 to 5.29)	4.76 (3.83 to 5.99)	7.45 (5.69 to 12.8)	7.74 (5.91 to 13.27)
Hubei	Vascular intestinal disorders	2.95 (2.25 to 3.71)	5.31 (4.04 to 6.67)	5.03 (4.04 to 7.3)	9.1 (7.31 to 13.2)
Hunan	Vascular intestinal disorders	3.55 (2.2 to 4.9)	5.71 (3.54 to 7.89)	5.75 (4.74 to 7.67)	8.57 (7.05 to 11.43)
Inner Mongolia	Vascular intestinal disorders	1.12 (0.812 to 1.41)	4.9 (3.57 to 6.2)	2 (1.56 to 2.99)	7.71 (6.03 to 11.54)

Jiangsu	Vascular intestinal disorders	4.49<br>(3.58 to 5.41)	6.52<br>(5.2 to 7.87)	8.08<br>(6.48 to 12.2)	9.9<br>(7.93 to 14.9)
Jiangxi	Vascular intestinal disorders	2.86<br>(1.67 to 3.83)	7.24<br>(4.22 to 9.69)	3.1<br>(2.59 to 3.88)	6.53<br>(5.44 to 8.17)
Jilin	Vascular intestinal disorders	1.36<br>(1.02 to 1.68)	5.25<br>(3.94 to 6.47)	2.14<br>(1.69 to 3.2)	7.67<br>(6.06 to 11.46)
Liaoning	Vascular intestinal disorders	2.47<br>(1.87 to 3.22)	6.05<br>(4.58 to 7.9)	4.56<br>(3.6 to 6.81)	10.21<br>(8.06 to 15.25)
Ningxia	Vascular intestinal disorders	0.243<br>(0.154 to 0.32)	4.93<br>(3.13 to 6.49)	0.374<br>(0.299 to 0.479)	5.33<br>(4.26 to 6.83)
Qinghai	Vascular intestinal disorders	0.232<br>(0.119 to 0.302)	4.89<br>(2.51 to 6.37)	0.336<br>(0.253 to 0.412)	5.35<br>(4.03 to 6.56)
Shaanxi	Vascular intestinal disorders	1.27<br>(0.861 to 1.68)	3.7<br>(2.5 to 4.88)	2.75<br>(2.18 to 3.52)	6.96<br>(5.51 to 8.91)
Shandong	Vascular intestinal disorders	5.84<br>(3.94 to 7.51)	6.82<br>(4.61 to 8.78)	9.55<br>(7.39 to 15.1)	9.61<br>(7.44 to 15.19)
Shanghai	Vascular intestinal disorders	1.28<br>(0.699 to 1.72)	9.15<br>(5.01 to 12.31)	1.93<br>(1.43 to 2.44)	6.9<br>(5.12 to 8.73)
Shanxi	Vascular intestinal disorders	1.67<br>(1.26 to 2.11)	5.51<br>(4.17 to 6.95)	2.53<br>(1.94 to 3.7)	6.56<br>(5.03 to 9.57)
Sichuan	Vascular intestinal disorders	5.16<br>(3.7 to 6.25)	4.62<br>(3.31 to 5.6)	7.13<br>(5.74 to 8.81)	8.54<br>(6.88 to 10.56)
Tianjin	Vascular intestinal disorders	0.579<br>(0.425 to 0.742)	6.29<br>(4.62 to 8.07)	1.1<br>(0.849 to 1.82)	7.18<br>(5.56 to 11.92)
Tibet	Vascular intestinal disorders	0.0742<br>(0.0496 to 0.142)	3.19<br>(2.13 to 6.08)	0.148<br>(0.0958 to 0.192)	4.27<br>(2.77 to 5.56)
Xinjiang	Vascular intestinal disorders	0.719<br>(0.511 to 0.905)	4.56<br>(3.24 to 5.74)	1.39<br>(1.09 to 2.1)	5.72<br>(4.47 to 8.62)
Yunnan	Vascular intestinal disorders	1.91<br>(0.888 to 2.47)	4.91<br>(2.28 to 6.34)	3.18<br>(2.16 to 3.83)	6.46<br>(4.39 to 7.79)
Zhejiang	Vascular intestinal disorders	3.07<br>(1.89 to 4.14)	6.96<br>(4.28 to 9.39)	4.63<br>(3.69 to 6.26)	7.57<br>(6.03 to 10.23)
China	Gallbladder and biliary diseases	457<br>(357 to 491)	38.2<br>(29.86 to 40.98)	287<br>(267 to 358)	20.29<br>(18.89 to 25.34)
Taiwan	Gallbladder and biliary diseases	7.6<br>(6.79 to 8.05)	37.25<br>(33.26 to 39.48)	9.91<br>(9.09 to 11.2)	42.01<br>(38.54 to 47.38)
Hong Kong Special Administrative Region of China	Gallbladder and biliary diseases	1.63<br>(1.18 to 1.85)	28.31<br>(20.45 to 32.15)	2.18<br>(1.69 to 2.58)	28.98<br>(22.46 to 34.37)
Macau Special Administrative Region of China	Gallbladder and biliary diseases	0.0953<br>(0.0668 to 0.109)	26.85<br>(18.81 to 30.76)	0.153<br>(0.102 to 0.214)	22.42<br>(14.95 to 31.26)
Anhui	Gallbladder and biliary diseases	18.8<br>(15.9 to 22.9)	31.67<br>(26.91 to 38.71)	9.22<br>(7.19 to 16.8)	15.09<br>(11.76 to 27.42)
Beijing	Gallbladder and biliary diseases	5.25<br>(3.57 to 6.23)	46.8<br>(31.82 to 55.52)	4.22<br>(3.44 to 5.21)	17.52<br>(14.28 to 21.61)
Chongqing	Gallbladder and biliary diseases	3.28<br>(2.71 to 3.95)	20.8<br>(17.19 to 25.05)	3.16<br>(2.46 to 5.46)	11.81<br>(9.2 to 20.42)
Fujian	Gallbladder and biliary diseases	17.1<br>(9.01 to 19.9)	54.16<br>(28.48 to 62.99)	10.4<br>(8.49 to 12.7)	26.3<br>(21.53 to 32.2)
Gansu	Gallbladder and biliary diseases	11.5<br>(7.02 to 13.4)	48.49<br>(29.69 to 56.63)	6.31<br>(5.3 to 7.5)	23.73<br>(19.93 to 28.21)
Guangdong	Gallbladder and biliary diseases	28<br>(20 to 33.4)	42.47<br>(30.46 to 50.67)	16.6<br>(13.8 to 20.5)	14.21<br>(11.8 to 17.56)
Guangxi	Gallbladder and biliary diseases	20.5<br>(11.9 to 24.7)	47.05<br>(27.28 to 56.86)	11.1<br>(9.2 to 13.3)	23<br>(19.04 to 27.56)
Guizhou	Gallbladder and biliary diseases	17.9<br>(8.33 to 22.2)	53.94<br>(25.09 to 66.87)	11.4<br>(6.84 to 14.2)	33.57<br>(20.23 to 42)
Hainan	Gallbladder and biliary diseases	2.45<br>(1.84 to 3.11)	35.11<br>(26.37 to 44.7)	2.54<br>(1.8 to 3.2)	26.47<br>(18.74 to 33.38)
Hebei	Gallbladder and biliary diseases	10.8<br>(9.07 to 14.2)	16.96<br>(14.25 to 22.25)	12.1<br>(9.04 to 23.3)	15.87<br>(11.83 to 30.45)
Heilongjiang	Gallbladder and biliary diseases	5.92<br>(4.75 to 9.69)	16.24<br>(13.04 to 26.58)	5.51<br>(4.1 to 12.1)	14.03<br>(10.45 to 30.72)
Henan	Gallbladder and biliary diseases	15.1<br>(12.8 to 18.8)	17.15<br>(14.55 to 21.33)	10.7<br>(7.88 to 23.2)	11.08<br>(8.19 to 24.04)
Hubei	Gallbladder and biliary diseases	19.9<br>(16.3 to 22.8)	35.78<br>(29.32 to 41.13)	13.8<br>(11.2 to 19)	24.93<br>(20.18 to 34.43)

Hunan	Gallbladder and biliary diseases	30.6 (21.4 to 35.9)	49.21 (34.42 to 57.75)	19.8 (16.4 to 24.2)	29.49 (24.35 to 36.09)
Inner Mongolia	Gallbladder and biliary diseases	5.82 (5.02 to 6.76)	25.6 (22.05 to 29.75)	3.49 (2.76 to 6.11)	13.47 (10.67 to 23.61)
Jiangsu	Gallbladder and biliary diseases	29.8 (21 to 34.5)	43.37 (30.48 to 50.08)	17.1 (13.8 to 24.3)	20.97 (16.86 to 29.73)
Jiangxi	Gallbladder and biliary diseases	24.4 (17.8 to 28.4)	61.67 (45.12 to 71.9)	10.1 (8.5 to 12)	21.19 (17.87 to 25.3)
Jilin	Gallbladder and biliary diseases	5.53 (4.68 to 6.63)	21.32 (18.05 to 25.53)	4.12 (3.25 to 6.64)	14.78 (11.64 to 23.8)
Liaoning	Gallbladder and biliary diseases	12.4 (10.2 to 15.8)	30.41 (24.9 to 38.62)	11.7 (9.37 to 16.7)	26.12 (20.98 to 37.3)
Ningxia	Gallbladder and biliary diseases	1.76 (1.36 to 2.13)	35.71 (27.55 to 43.04)	1.04 (0.847 to 1.35)	14.86 (12.07 to 19.22)
Qinghai	Gallbladder and biliary diseases	3.79 (1.5 to 4.68)	79.97 (31.58 to 98.9)	2.38 (1.63 to 2.93)	37.88 (25.88 to 46.56)
Shaanxi	Gallbladder and biliary diseases	7.42 (4.99 to 8.97)	21.58 (14.51 to 26.06)	6.71 (5.4 to 8.68)	16.99 (13.68 to 21.98)
Shandong	Gallbladder and biliary diseases	27.5 (23.1 to 34.6)	32.13 (26.93 to 40.48)	15.8 (12.2 to 29.8)	15.88 (12.26 to 29.94)
Shanghai	Gallbladder and biliary diseases	15.4 (6.35 to 18.6)	110.49 (45.44 to 132.99)	11.1 (7.6 to 13.5)	39.62 (27.18 to 48.43)
Shanxi	Gallbladder and biliary diseases	8.05 (6.76 to 9.38)	26.59 (22.33 to 30.99)	4.54 (3.46 to 7.93)	11.74 (8.96 to 20.52)
Sichuan	Gallbladder and biliary diseases	35.9 (25.8 to 41.5)	32.2 (23.13 to 37.21)	18 (14.8 to 22.4)	21.56 (17.72 to 26.86)
Tianjin	Gallbladder and biliary diseases	3.12 (2.61 to 3.83)	33.88 (28.33 to 41.67)	2.44 (1.89 to 4)	15.96 (12.38 to 26.2)
Tibet	Gallbladder and biliary diseases	1.89 (1.24 to 2.39)	80.92 (53.03 to 102.74)	2.67 (1.22 to 4.1)	77.19 (35.18 to 118.65)
Xinjiang	Gallbladder and biliary diseases	5.28 (4.23 to 6.05)	33.45 (26.83 to 38.33)	6.14 (4.97 to 7.93)	25.21 (20.39 to 32.54)
Yunnan	Gallbladder and biliary diseases	28.8 (11.2 to 35.5)	73.97 (28.62 to 91.08)	15.2 (9.62 to 18.4)	30.87 (19.57 to 37.49)
Zhejiang	Gallbladder and biliary diseases	31.5 (16.3 to 37.3)	71.49 (37.05 to 84.67)	15.1 (12.1 to 18.7)	24.64 (19.8 to 30.63)
China	Pancreatitis	257 (212 to 310)	21.49 (17.68 to 25.92)	348 (301 to 448)	24.64 (21.3 to 31.69)
Taiwan	Pancreatitis	5.45 (5.06 to 5.94)	26.69 (24.82 to 29.09)	9.07 (8.1 to 10.3)	38.48 (34.33 to 43.69)
Hong Kong Special Administrative Region of China	Pancreatitis	1.4 (0.896 to 1.71)	24.33 (15.54 to 29.68)	1.85 (1.47 to 2.47)	24.69 (19.55 to 32.83)
Macau Special Administrative Region of China	Pancreatitis	0.0758 (0.0499 to 0.0923)	21.36 (14.05 to 25.99)	0.176 (0.0869 to 0.284)	25.77 (12.71 to 41.58)
Anhui	Pancreatitis	12 (8.66 to 17.1)	20.24 (14.62 to 28.81)	12.8 (9.9 to 21.4)	20.99 (16.19 to 35.07)
Beijing	Pancreatitis	2.38 (1.69 to 3.17)	21.23 (15.11 to 28.23)	4.83 (3.65 to 6.62)	20.03 (15.13 to 27.47)
Chongqing	Pancreatitis	4.03 (2.71 to 5.85)	25.56 (17.18 to 37.05)	8.26 (5.45 to 10.7)	30.9 (20.37 to 39.89)
Fujian	Pancreatitis	4.4 (3.46 to 6.08)	13.91 (10.92 to 19.22)	6.82 (5.22 to 9.9)	17.3 (13.25 to 25.12)
Gansu	Pancreatitis	7.68 (4.38 to 10.3)	32.49 (18.53 to 43.45)	9.22 (5.83 to 11.9)	34.68 (21.93 to 44.78)
Guangdong	Pancreatitis	10.9 (8.01 to 14.6)	16.62 (12.18 to 22.23)	17.9 (13.9 to 25.6)	15.33 (11.9 to 21.87)
Guangxi	Pancreatitis	8.51 (6.22 to 11.1)	19.58 (14.31 to 25.41)	10.6 (8.41 to 13.5)	22.03 (17.41 to 27.9)
Guizhou	Pancreatitis	12.5 (4.47 to 18.3)	37.72 (13.47 to 55)	14.7 (6.36 to 19.8)	43.45 (18.81 to 58.5)
Hainan	Pancreatitis	0.746 (0.438 to 1.27)	10.72 (6.29 to 18.16)	1.31 (0.968 to 1.79)	13.66 (10.09 to 18.72)
Hebei	Pancreatitis	10.7 (8.03 to 17.4)	16.88 (12.62 to 27.37)	16.3 (11.1 to 31.7)	21.3 (14.56 to 41.51)
Heilongjiang	Pancreatitis	7.26 (5.07 to 13.2)	19.92 (13.91 to 36.33)	10.8 (8.1 to 17.6)	27.46 (20.63 to 44.73)

Henan	Pancreatitis	12.4 (9.28 to 21)	14.04 (10.51 to 23.75)	18.7 (12.7 to 39.5)	19.38 (13.2 to 41.02)
Hubei	Pancreatitis	9.51 (7.29 to 13.2)	17.12 (13.13 to 23.79)	13.6 (10.3 to 21.4)	24.65 (18.69 to 38.61)
Hunan	Pancreatitis	20.5 (10 to 29.1)	32.89 (16.13 to 46.86)	27.3 (16.4 to 34.8)	40.65 (24.48 to 51.87)
Inner Mongolia	Pancreatitis	6.02 (4.42 to 7.82)	26.47 (19.43 to 34.4)	8.65 (6.66 to 10.8)	33.41 (25.73 to 41.81)
Jiangsu	Pancreatitis	14.1 (11.4 to 18)	20.49 (16.51 to 26.13)	18.1 (13.9 to 27.8)	22.19 (16.98 to 34.05)
Jiangxi	Pancreatitis	10.6 (6.74 to 15.5)	26.72 (17.05 to 39.21)	11.6 (8.56 to 14.3)	24.43 (18.01 to 30.12)
Jilin	Pancreatitis	6.34 (4.79 to 8.09)	24.44 (18.46 to 31.17)	7.97 (6.36 to 9.87)	28.59 (22.79 to 35.38)
Liaoning	Pancreatitis	8.5 (6.04 to 13.5)	20.83 (14.81 to 33.06)	12.2 (9.15 to 18.7)	27.36 (20.48 to 41.81)
Ningxia	Pancreatitis	0.722 (0.532 to 1.07)	14.61 (10.76 to 21.64)	1.13 (0.87 to 1.6)	16.03 (12.4 to 22.83)
Qinghai	Pancreatitis	1.73 (0.611 to 2.47)	36.42 (12.91 to 52.19)	2.52 (1.17 to 3.42)	40.14 (18.68 to 54.43)
Shaanxi	Pancreatitis	4.64 (3.38 to 5.84)	13.5 (9.82 to 16.98)	7.8 (6.09 to 10.7)	19.74 (15.41 to 27.2)
Shandong	Pancreatitis	13.7 (8.11 to 29.1)	15.98 (9.47 to 34.04)	18.8 (12.1 to 42.7)	18.9 (12.13 to 42.95)
Shanghai	Pancreatitis	3.76 (2.26 to 5.41)	26.89 (16.17 to 38.74)	6.49 (4.38 to 8.26)	23.22 (15.67 to 29.55)
Shanxi	Pancreatitis	5.76 (3.89 to 8.65)	19.01 (12.85 to 28.58)	7.33 (5.45 to 11.9)	18.99 (14.11 to 30.91)
Sichuan	Pancreatitis	31.3 (16.6 to 40.5)	28.06 (14.86 to 36.31)	31.9 (19.5 to 41.1)	38.17 (23.38 to 49.17)
Tianjin	Pancreatitis	1.72 (1.15 to 2.93)	18.71 (12.46 to 31.83)	3.06 (2.17 to 6.01)	20.08 (14.2 to 39.4)
Tibet	Pancreatitis	0.521 (0.223 to 0.771)	22.37 (9.58 to 33.08)	1.21 (0.455 to 1.7)	35.15 (13.18 to 49.33)
Xinjiang	Pancreatitis	3.43 (2.61 to 4.19)	21.72 (16.54 to 26.57)	6.24 (4.91 to 8.15)	25.62 (20.14 to 33.46)
Yunnan	Pancreatitis	11.6 (4.8 to 16.2)	29.69 (12.32 to 41.46)	16.4 (8.86 to 21.1)	33.38 (18.02 to 42.97)
Zhejiang	Pancreatitis	7.83 (5.66 to 11.7)	17.75 (12.83 to 26.52)	11.4 (8.85 to 15.3)	18.55 (14.46 to 24.93)
China	Other digestive diseases	876 (766 to 1000)	73.18 (63.95 to 83.73)	426 (349 to 511)	30.16 (24.73 to 36.21)
Taiwan	Other digestive diseases	5.25 (4.69 to 5.82)	25.71 (23.01 to 28.54)	9.6 (8.55 to 10.8)	40.7 (36.25 to 45.73)
Hong Kong Special Administrative Region of China	Other digestive diseases	1.28 (1.05 to 1.54)	22.26 (18.26 to 26.63)	2.16 (1.72 to 2.67)	28.78 (22.94 to 35.56)
Macau Special Administrative Region of China	Other digestive diseases	0.123 (0.105 to 0.142)	34.79 (29.72 to 39.97)	0.244 (0.175 to 0.335)	35.66 (25.55 to 48.97)
Anhui	Other digestive diseases	28.5 (22.1 to 36.9)	48.18 (37.31 to 62.33)	16.4 (12.8 to 20.8)	26.83 (20.93 to 34.02)
Beijing	Other digestive diseases	4.01 (3.35 to 4.69)	35.71 (29.83 to 41.8)	4.59 (3.57 to 5.73)	19.06 (14.83 to 23.77)
Chongqing	Other digestive diseases	10.5 (8.47 to 12.8)	66.68 (53.68 to 81.1)	9.27 (7.01 to 11.7)	34.7 (26.22 to 43.69)
Fujian	Other digestive diseases	23.1 (19.7 to 27.1)	73.1 (62.13 to 85.63)	9.92 (7.94 to 12.1)	25.17 (20.15 to 30.82)
Gansu	Other digestive diseases	33.2 (25.8 to 41.1)	140.37 (109.08 to 173.87)	10.3 (8.44 to 12.5)	38.79 (31.74 to 47.1)
Guangdong	Other digestive diseases	36.6 (30.4 to 43.6)	55.57 (46.12 to 66.19)	26.7 (20.7 to 33.6)	22.87 (17.71 to 28.72)
Guangxi	Other digestive diseases	33.5 (26.4 to 40.2)	77.08 (60.64 to 92.53)	15.5 (12.2 to 19.3)	31.98 (25.25 to 39.85)
Guizhou	Other digestive diseases	64 (41.5 to 81.9)	192.83 (125.13 to 246.79)	20.1 (14.8 to 25.2)	59.56 (43.82 to 74.4)
Hainan	Other digestive diseases	2.32 (1.66 to 3.24)	33.36 (23.86 to 46.48)	2.04 (1.61 to 2.53)	21.28 (16.76 to 26.4)

Hebei	Other digestive diseases	38<br>(31.4 to 46.5)	59.74<br>(49.36 to 73.1)	26.1<br>(20.9 to 32.6)	34.18<br>(27.38 to 42.65)
Heilongjiang	Other digestive diseases	11.6<br>(8.36 to 17.5)	31.82<br>(22.95 to 48.08)	9.15<br>(7.34 to 11.7)	23.31<br>(18.71 to 29.74)
Henan	Other digestive diseases	53.1<br>(44.2 to 67.6)	60.13<br>(50.05 to 76.53)	25.4<br>(20 to 33.1)	26.37<br>(20.78 to 34.36)
Hubei	Other digestive diseases	30<br>(24.6 to 35.6)	53.98<br>(44.36 to 64.1)	19.6<br>(14.6 to 25.2)	35.42<br>(26.46 to 45.48)
Hunan	Other digestive diseases	34.9<br>(29.8 to 40.6)	56.19<br>(47.85 to 65.27)	20.5<br>(15.8 to 25.2)	30.5<br>(23.59 to 37.56)
Inner Mongolia	Other digestive diseases	18.5<br>(15.1 to 22.4)	81.48<br>(66.27 to 98.35)	7.24<br>(5.8 to 8.85)	27.95<br>(22.38 to 34.16)
Jiangsu	Other digestive diseases	31.4<br>(26.1 to 36.9)	45.61<br>(37.9 to 53.61)	20.5<br>(16.3 to 25.7)	25.15<br>(19.99 to 31.5)
Jiangxi	Other digestive diseases	36.1<br>(29.2 to 45.1)	91.23<br>(73.91 to 113.98)	13.1<br>(10.2 to 16.3)	27.63<br>(21.46 to 34.19)
Jilin	Other digestive diseases	11.9<br>(8.77 to 15.2)	45.96<br>(33.81 to 58.68)	7.36<br>(5.98 to 9.05)	26.39<br>(21.44 to 32.45)
Liaoning	Other digestive diseases	12.8<br>(9.37 to 17.6)	31.39<br>(22.98 to 43.06)	12<br>(9.46 to 15.1)	26.93<br>(21.19 to 33.78)
Ningxia	Other digestive diseases	4.22<br>(3.03 to 5.69)	85.54<br>(61.28 to 115.12)	1.57<br>(1.24 to 1.95)	22.39<br>(17.68 to 27.78)
Qinghai	Other digestive diseases	8.95<br>(6.09 to 12.9)	189.02<br>(128.48 to 272.27)	2.41<br>(1.89 to 2.9)	38.31<br>(30.09 to 46.2)
Shaanxi	Other digestive diseases	22.2<br>(17.8 to 27.3)	64.63<br>(51.85 to 79.23)	9.29<br>(7.51 to 11.2)	23.52<br>(19.02 to 28.29)
Shandong	Other digestive diseases	40.9<br>(33.9 to 51.4)	47.81<br>(39.55 to 60.09)	26.4<br>(20.4 to 33.8)	26.54<br>(20.56 to 34.05)
Shanghai	Other digestive diseases	5.14<br>(4.23 to 6.1)	36.8<br>(30.29 to 43.64)	6.08<br>(4.78 to 7.61)	21.76<br>(17.11 to 27.25)
Shanxi	Other digestive diseases	19.6<br>(16.4 to 23.1)	64.65<br>(54.2 to 76.29)	8.76<br>(6.97 to 10.9)	22.67<br>(18.05 to 28.25)
Sichuan	Other digestive diseases	107<br>(87.8 to 132)	95.43<br>(78.68 to 118.67)	32.4<br>(25.8 to 39.9)	38.85<br>(30.9 to 47.84)
Tianjin	Other digestive diseases	3.38<br>(2.77 to 4.29)	36.73<br>(30.07 to 46.64)	2.85<br>(2.21 to 3.63)	18.68<br>(14.46 to 23.79)
Tibet	Other digestive diseases	4.94<br>(3.95 to 6.88)	212.08<br>(169.43 to 295.33)	2.31<br>(1.65 to 2.95)	66.98<br>(47.87 to 85.21)
Xinjiang	Other digestive diseases	22.3<br>(18.2 to 27.1)	141.08<br>(115.48 to 171.58)	13.1<br>(10.8 to 15.8)	53.77<br>(44.3 to 64.96)
Yunnan	Other digestive diseases	104<br>(62.9 to 136)	266.74<br>(161.4 to 348.06)	27.7<br>(21.2 to 33.6)	56.43<br>(43.08 to 68.29)
Zhejiang	Other digestive diseases	18.3<br>(15 to 22)	41.42<br>(33.95 to 49.78)	14.8<br>(11.4 to 18.6)	24.19<br>(18.61 to 30.41)
China	Neurological disorders	10900<br>(8740 to 13500)	908.84<br>(729.76 to 1125.45)	17000<br>(14000 to 20600)	1202.91<br>(990.04 to 1458.41)
Taiwan	Neurological disorders	212<br>(160 to 278)	1039.88<br>(786.37 to 1362.22)	380<br>(307 to 468)	1611.26<br>(1301.82 to 1985.15)
Hong Kong Special Administrative Region of China	Neurological disorders	48.1<br>(38.2 to 60.4)	834.14<br>(663.46 to 1048.49)	112<br>(93.9 to 135)	1493.93<br>(1249.69 to 1792.38)
Macau Special Administrative Region of China	Neurological disorders	3.16<br>(2.5 to 3.97)	889.12<br>(704.09 to 1117.9)	6.99<br>(5.36 to 8.85)	1021.49<br>(784.02 to 1293.89)
Anhui	Neurological disorders	530<br>(426 to 655)	894.14<br>(718.96 to 1105.1)	777<br>(635 to 946)	1271.25<br>(1038.01 to 1547.65)
Beijing	Neurological disorders	109<br>(86.5 to 137)	975.46<br>(771.58 to 1219.27)	257<br>(203 to 326)	1064.97<br>(841.98 to 1352.64)
Chongqing	Neurological disorders	133<br>(103 to 171)	844.86<br>(650.59 to 1080.69)	338<br>(269 to 416)	1263.66<br>(1006.63 to 1555.51)
Fujian	Neurological disorders	270<br>(212 to 342)	854.78<br>(670.72 to 1081.7)	467<br>(380 to 581)	1185.13<br>(963.54 to 1473.45)
Gansu	Neurological disorders	224<br>(176 to 279)	945.92<br>(744.32 to 1179.18)	303<br>(240 to 372)	1139.98<br>(904.03 to 1399.86)
Guangdong	Neurological disorders	582<br>(461 to 738)	884.26<br>(701.08 to 1120.6)	1220<br>(955 to 1530)	1040.85<br>(817.38 to 1313.44)
Guangxi	Neurological disorders	381<br>(304 to 472)	876.66<br>(699.79 to 1085.53)	585<br>(477 to 713)	1211.08<br>(986.24 to 1475.98)

Guizhou	Neurological disorders	320<br>(257 to 403)	965.26<br>(774 to 1212.82)	382<br>(310 to 469)	1129.83<br>(915.33 to 1387.21)
Hainan	Neurological disorders	58.9<br>(45.5 to 74.1)	845.52<br>(652.58 to 1063.56)	105<br>(82.9 to 132)	1096.44<br>(864.56 to 1381.63)
Hebei	Neurological disorders	552<br>(426 to 710)	866.89<br>(669.97 to 1115.29)	896<br>(705 to 1110)	1171.85<br>(922.34 to 1457.96)
Heilongjiang	Neurological disorders	358<br>(267 to 465)	982.52<br>(731.43 to 1276.92)	548<br>(424 to 696)	1396.67<br>(1079.7 to 1774.43)
Henan	Neurological disorders	750<br>(586 to 950)	849.27<br>(663.85 to 1076.41)	1090<br>(878 to 1350)	1126.74<br>(911.74 to 1399.77)
Hubei	Neurological disorders	470<br>(370 to 593)	846.87<br>(665.7 to 1068.03)	694<br>(561 to 849)	1254.69<br>(1015.04 to 1535.48)
Hunan	Neurological disorders	565<br>(444 to 709)	908.47<br>(713.99 to 1140.35)	855<br>(694 to 1030)	1273.56<br>(1032.83 to 1541.28)
Inner Mongolia	Neurological disorders	190<br>(148 to 238)	834.62<br>(651.23 to 1046.31)	298<br>(240 to 366)	1150.9<br>(925.75 to 1412.48)
Jiangsu	Neurological disorders	674<br>(534 to 841)	979.55<br>(776.36 to 1222.07)	1140<br>(940 to 1390)	1399.26<br>(1151.55 to 1698.26)
Jiangxi	Neurological disorders	386<br>(312 to 477)	975.61<br>(788.02 to 1205.39)	499<br>(397 to 617)	1050.28<br>(834.54 to 1298.73)
Jilin	Neurological disorders	222<br>(172 to 281)	855.25<br>(663.7 to 1084.64)	325<br>(261 to 406)	1166.61<br>(936.74 to 1454.02)
Liaoning	Neurological disorders	381<br>(300 to 478)	935.05<br>(735.19 to 1170.69)	632<br>(521 to 763)	1414.77<br>(1167.27 to 1709.95)
Ningxia	Neurological disorders	44.9<br>(33.9 to 58.5)	908.28<br>(685.44 to 1184.41)	71.6<br>(54.2 to 92)	1019.64<br>(771.91 to 1310.44)
Qinghai	Neurological disorders	44.2<br>(34.6 to 55.2)	932.82<br>(730.1 to 1164.38)	62.3<br>(49.5 to 78.3)	992.33<br>(787.55 to 1246.98)
Shaanxi	Neurological disorders	269<br>(208 to 340)	781.08<br>(605.04 to 987.35)	446<br>(353 to 550)	1128.63<br>(893.24 to 1392.05)
Shandong	Neurological disorders	812<br>(647 to 1020)	948.88<br>(755.96 to 1192.98)	1260<br>(1020 to 1530)	1267.47<br>(1024.09 to 1543.37)
Shanghai	Neurological disorders	154<br>(121 to 197)	1101.13<br>(863.57 to 1409.62)	341<br>(263 to 432)	1220.43<br>(939.74 to 1546.38)
Shanxi	Neurological disorders	276<br>(217 to 346)	909.87<br>(715.17 to 1141.08)	429<br>(340 to 533)	1111.57<br>(879.07 to 1378.83)
Sichuan	Neurological disorders	997<br>(773 to 1270)	893.53<br>(692.79 to 1138.94)	1060<br>(848 to 1300)	1268.91<br>(1015.62 to 1554.16)
Tianjin	Neurological disorders	86.4<br>(69.3 to 108)	939.56<br>(752.93 to 1172.3)	175<br>(140 to 217)	1148.7<br>(915.02 to 1424.84)
Tibet	Neurological disorders	33.7<br>(24.3 to 42.5)	1447.12<br>(1041.67 to 1825.41)	38.6<br>(29.1 to 49.4)	1116.45<br>(842.19 to 1430.53)
Xinjiang	Neurological disorders	134<br>(105 to 168)	848.16<br>(662.65 to 1063.75)	252<br>(198 to 315)	1034.12<br>(813.31 to 1290.67)
Yunnan	Neurological disorders	399<br>(319 to 493)	1024.76<br>(818.59 to 1266.11)	578<br>(466 to 705)	1175.28<br>(948.42 to 1434.73)
Zhejiang	Neurological disorders	420<br>(334 to 520)	953.22<br>(757.46 to 1178.5)	753<br>(612 to 918)	1231.36<br>(1000.19 to 1500.22)
China	Alzheimer's disease and other dementias	2710<br>(2510 to 2950)	226.51<br>(209.54 to 246.28)	6970<br>(6430 to 7570)	493.25<br>(455.35 to 535.61)
Taiwan	Alzheimer's disease and other dementias	45.4<br>(42.4 to 48.7)	222.63<br>(207.82 to 238.86)	149<br>(138 to 160)	630.84<br>(583.86 to 680.52)
Hong Kong Special Administrative Region of China	Alzheimer's disease and other dementias	16.3<br>(14.9 to 18.1)	283.34<br>(257.81 to 313.32)	62.7<br>(54.6 to 72.6)	834.89<br>(726.93 to 966.76)
Macau Special Administrative Region of China	Alzheimer's disease and other dementias	0.899<br>(0.817 to 0.992)	253.35<br>(230.1 to 279.45)	2.28<br>(1.93 to 2.64)	333.74<br>(281.79 to 386.56)
Anhui	Alzheimer's disease and other dementias	133<br>(120 to 148)	225<br>(202.01 to 249.15)	348<br>(305 to 397)	569.31<br>(498.6 to 649.52)
Beijing	Alzheimer's disease and other dementias	29.5<br>(26.6 to 32.5)	263.37<br>(236.83 to 289.61)	89.3<br>(76.6 to 103)	370.47<br>(317.62 to 426.77)
Chongqing	Alzheimer's disease and other dementias	28.3<br>(24.6 to 32.8)	179.04<br>(156.04 to 207.67)	155<br>(131 to 181)	579.92<br>(491.85 to 677.19)
Fujian	Alzheimer's disease and other dementias	69.7<br>(61.5 to 81.1)	220.43<br>(194.34 to 256.27)	195<br>(171 to 224)	495.45<br>(433.61 to 569.16)
Gansu	Alzheimer's disease and other dementias	39<br>(34.6 to 45.4)	165.07<br>(146.3 to 192.07)	116<br>(101 to 132)	435.27<br>(378.35 to 498.52)

Guangdong	Alzheimer's disease and other dementias	179 (156 to 208)	272.08 (237.7 to 315.38)	451 (393 to 516)	386.34 (336.19 to 441.3)
Guangxi	Alzheimer's disease and other dementias	104 (89.7 to 122)	238.81 (206.36 to 280.5)	257 (223 to 293)	531.74 (461.82 to 606.27)
Guizhou	Alzheimer's disease and other dementias	65.9 (58.1 to 76.2)	198.44 (174.99 to 229.49)	143 (123 to 165)	424.2 (364.53 to 488.2)
Hainan	Alzheimer's disease and other dementias	16.5 (13.9 to 19.5)	237.28 (200.14 to 279.7)	42.3 (35.7 to 49.2)	440.94 (372.24 to 513.37)
Hebei	Alzheimer's disease and other dementias	143 (129 to 160)	225.15 (202.25 to 251.2)	348 (300 to 401)	454.98 (393.1 to 525.01)
Heilongjiang	Alzheimer's disease and other dementias	61.2 (55.2 to 67.6)	167.81 (151.44 to 185.57)	187 (161 to 216)	476.83 (410.54 to 550.3)
Henan	Alzheimer's disease and other dementias	193 (175 to 212)	218.31 (197.75 to 240.67)	440 (385 to 499)	456.83 (399.4 to 518.39)
Hubei	Alzheimer's disease and other dementias	114 (103 to 125)	204.36 (184.95 to 224.91)	295 (255 to 337)	534.15 (461.78 to 609.66)
Hunan	Alzheimer's disease and other dementias	147 (131 to 171)	236.32 (210.58 to 275.55)	379 (333 to 428)	564.3 (495.42 to 637.01)
Inner Mongolia	Alzheimer's disease and other dementias	39.6 (35.2 to 44)	174.02 (154.96 to 193.35)	116 (99.4 to 132)	446.66 (384.04 to 511.67)
Jiangsu	Alzheimer's disease and other dementias	198 (179 to 219)	287.15 (259.96 to 318.59)	533 (465 to 606)	653.07 (569.44 to 741.82)
Jiangxi	Alzheimer's disease and other dementias	82 (72.2 to 98)	207.54 (182.64 to 248.01)	176 (154 to 201)	370.22 (323.16 to 422.67)
Jilin	Alzheimer's disease and other dementias	49.4 (44.7 to 54.7)	190.52 (172.29 to 210.76)	124 (105 to 142)	443.49 (375.99 to 508.79)
Liaoning	Alzheimer's disease and other dementias	101 (89.7 to 114)	247.69 (219.88 to 280.06)	288 (250 to 331)	645.34 (559.01 to 741.05)
Ningxia	Alzheimer's disease and other dementias	6.22 (5.58 to 6.93)	125.89 (113.05 to 140.32)	20.4 (17.3 to 23.8)	291.09 (246.05 to 338.91)
Qinghai	Alzheimer's disease and other dementias	5.47 (4.82 to 6.24)	115.4 (101.85 to 131.76)	18.2 (15.7 to 20.9)	290.23 (250.35 to 332.42)
Shaanxi	Alzheimer's disease and other dementias	56.1 (48.9 to 63.8)	163.09 (142.25 to 185.27)	172 (147 to 200)	436.53 (372.94 to 505.51)
Shandong	Alzheimer's disease and other dementias	234 (209 to 261)	273.05 (244.26 to 305.21)	559 (478 to 643)	562.41 (480.97 to 647.47)
Shanghai	Alzheimer's disease and other dementias	46.3 (41 to 52.5)	331.11 (293.5 to 375.55)	125 (107 to 146)	448.97 (384.52 to 521.26)
Shanxi	Alzheimer's disease and other dementias	71.7 (65.2 to 78.7)	236.87 (215.22 to 259.92)	164 (137 to 192)	423.75 (355.38 to 497.85)
Sichuan	Alzheimer's disease and other dementias	222 (199 to 246)	198.78 (178.08 to 220.4)	450 (387 to 510)	538.87 (463.62 to 610.82)
Tianjin	Alzheimer's disease and other dementias	25.5 (22.5 to 28.9)	277.44 (244.66 to 313.77)	72.1 (62 to 83.4)	472.2 (405.92 to 546.49)
Tibet	Alzheimer's disease and other dementias	4.98 (4.29 to 5.8)	213.52 (184.03 to 248.88)	8.22 (7.11 to 9.48)	237.8 (205.8 to 274.33)
Xinjiang	Alzheimer's disease and other dementias	22.3 (20.2 to 24.7)	141.14 (127.94 to 156.34)	77.5 (67.6 to 88.5)	318.1 (277.6 to 363.21)
Yunnan	Alzheimer's disease and other dementias	83.4 (72.2 to 100)	213.97 (185.37 to 257.62)	225 (196 to 258)	457.92 (398.69 to 525.51)
Zhejiang	Alzheimer's disease and other dementias	124 (110 to 147)	281.14 (248.43 to 333.31)	327 (285 to 373)	533.92 (465.54 to 610.33)
China	Parkinson's disease	619 (566 to 688)	51.71 (47.28 to 57.47)	1400 (1260 to 1570)	99.19 (89.09 to 111.15)
Taiwan	Parkinson's disease	10.9 (9.89 to 12.2)	53.5 (48.47 to 59.98)	30.8 (27.7 to 33.6)	130.6 (117.45 to 142.6)
Hong Kong Special Administrative Region of China	Parkinson's disease	3.74 (3.32 to 4.25)	64.87 (57.67 to 73.7)	10.3 (8.63 to 12.2)	136.86 (114.91 to 162.68)
Macau Special Administrative Region of China	Parkinson's disease	0.191 (0.17 to 0.224)	53.86 (47.8 to 63.05)	0.408 (0.322 to 0.533)	59.6 (47.14 to 78.01)
Anhui	Parkinson's disease	30.6 (26.6 to 34.9)	51.67 (44.85 to 58.87)	68.1 (58 to 79.3)	111.4 (94.79 to 129.67)
Beijing	Parkinson's disease	6.91 (6.01 to 7.88)	61.65 (53.59 to 70.25)	18.4 (15.2 to 21.8)	76.27 (63.17 to 90.62)
Chongqing	Parkinson's disease	6.92 (5.99 to 8.04)	43.86 (37.96 to 50.92)	31.6 (26.1 to 38.1)	118.21 (97.58 to 142.59)

Fujian	Parkinson's disease	15.3<br>(13.3 to 17.2)	48.46<br>(42.16 to 54.44)	36.3<br>(30.1 to 42.4)	92.17<br>(76.43 to 107.5)
Gansu	Parkinson's disease	10.7<br>(9.29 to 12.4)	45.18<br>(39.28 to 52.57)	25.1<br>(21.5 to 29.2)	94.6<br>(80.94 to 109.97)
Guangdong	Parkinson's disease	40.2<br>(34.9 to 45.6)	61.09<br>(52.97 to 69.29)	87.6<br>(73 to 103)	74.98<br>(62.51 to 87.96)
Guangxi	Parkinson's disease	23.4<br>(20.5 to 27)	53.76<br>(47.22 to 62.13)	49.8<br>(42.4 to 57.4)	102.98<br>(87.79 to 118.8)
Guizhou	Parkinson's disease	16.5<br>(14.4 to 19.7)	49.66<br>(43.49 to 59.38)	34.1<br>(28.9 to 39.6)	100.79<br>(85.34 to 116.98)
Hainan	Parkinson's disease	3.64<br>(3.06 to 4.31)	52.31<br>(43.98 to 61.93)	8.06<br>(6.56 to 9.64)	84.06<br>(68.41 to 100.55)
Hebei	Parkinson's disease	28.3<br>(24.5 to 34.3)	44.41<br>(38.46 to 53.89)	69.9<br>(58 to 84.4)	91.43<br>(75.88 to 110.5)
Heilongjiang	Parkinson's disease	15.1<br>(13.4 to 17.7)	41.56<br>(36.78 to 48.47)	39.4<br>(33.5 to 46.7)	100.46<br>(85.47 to 118.86)
Henan	Parkinson's disease	40.8<br>(36.1 to 46.7)	46.17<br>(40.88 to 52.91)	91.4<br>(78.1 to 106)	94.88<br>(81.09 to 110.39)
Hubei	Parkinson's disease	26.5<br>(23.4 to 31.1)	47.64<br>(42.2 to 55.9)	61.7<br>(52 to 72.9)	111.63<br>(94.12 to 131.9)
Hunan	Parkinson's disease	34.7<br>(30.8 to 41.2)	55.84<br>(49.5 to 66.23)	77.2<br>(66.1 to 89.6)	114.95<br>(98.45 to 133.45)
Inner Mongolia	Parkinson's disease	9.93<br>(8.66 to 11.3)	43.65<br>(38.1 to 49.84)	24.1<br>(20.6 to 28.1)	93.24<br>(79.4 to 108.66)
Jiangsu	Parkinson's disease	43.5<br>(38.6 to 49.2)	63.23<br>(56.15 to 71.49)	101<br>(85.6 to 117)	123.97<br>(104.81 to 143.85)
Jiangxi	Parkinson's disease	22.7<br>(20 to 25.7)	57.48<br>(50.54 to 64.93)	41.1<br>(35 to 47.4)	86.37<br>(73.7 to 99.7)
Jilin	Parkinson's disease	12.3<br>(10.8 to 14.1)	47.26<br>(41.72 to 54.32)	27<br>(22.6 to 31.9)	96.79<br>(80.98 to 114.4)
Liaoning	Parkinson's disease	23.3<br>(20.2 to 26.9)	57.16<br>(49.47 to 65.89)	56.7<br>(47.8 to 65.8)	126.99<br>(107.16 to 147.35)
Ningxia	Parkinson's disease	1.55<br>(1.36 to 1.86)	31.46<br>(27.46 to 37.68)	4.57<br>(3.79 to 5.47)	65.08<br>(53.96 to 77.95)
Qinghai	Parkinson's disease	1.46<br>(1.25 to 1.9)	30.82<br>(26.43 to 40.04)	4.34<br>(3.61 to 5.22)	69.05<br>(57.53 to 83.08)
Shaanxi	Parkinson's disease	11.8<br>(9.86 to 15.9)	34.37<br>(28.66 to 46.12)	35.2<br>(29.3 to 42)	89.06<br>(74.26 to 106.4)
Shandong	Parkinson's disease	52.7<br>(46.5 to 61)	61.58<br>(54.28 to 71.29)	105<br>(88.9 to 124)	105.22<br>(89.41 to 125.02)
Shanghai	Parkinson's disease	10.3<br>(8.92 to 11.7)	73.65<br>(63.86 to 83.51)	22.8<br>(18.6 to 27.3)	81.59<br>(66.66 to 97.86)
Shanxi	Parkinson's disease	15.9<br>(14.1 to 17.9)	52.67<br>(46.53 to 59.06)	32.2<br>(26.5 to 37.8)	83.38<br>(68.6 to 97.98)
Sichuan	Parkinson's disease	47.5<br>(41.1 to 59.9)	42.55<br>(36.83 to 53.69)	96.2<br>(80.8 to 112)	115.17<br>(96.79 to 133.61)
Tianjin	Parkinson's disease	5.61<br>(4.85 to 6.41)	60.96<br>(52.71 to 69.69)	13.4<br>(11.1 to 15.8)	87.6<br>(72.99 to 103.35)
Tibet	Parkinson's disease	1.16<br>(0.991 to 1.52)	49.98<br>(42.52 to 65.06)	1.8<br>(1.52 to 2.24)	52.02<br>(44 to 64.68)
Xinjiang	Parkinson's disease	6.63<br>(5.86 to 7.55)	42.01<br>(37.16 to 47.86)	21.6<br>(18 to 25.3)	88.49<br>(73.86 to 103.72)
Yunnan	Parkinson's disease	19.6<br>(17 to 22.4)	50.21<br>(43.56 to 57.53)	44<br>(37.9 to 51.2)	89.52<br>(76.99 to 104.05)
Zhejiang	Parkinson's disease	29.4<br>(24.7 to 33.3)	66.78<br>(56.03 to 75.46)	60.9<br>(50.8 to 72.2)	99.57<br>(82.98 to 117.93)
China	Idiopathic epilepsy	2120<br>(1700 to 2580)	176.72<br>(141.93 to 215.21)	1410<br>(1020 to 1910)	99.72<br>(72.51 to 135.46)
Taiwan	Idiopathic epilepsy	24<br>(11.6 to 40)	117.69<br>(56.68 to 195.84)	26.8<br>(13.4 to 47.7)	113.5<br>(56.93 to 202.38)
Hong Kong Special Administrative Region of China	Idiopathic epilepsy	4.59<br>(2.1 to 8.28)	79.66<br>(36.47 to 143.65)	5.6<br>(2.28 to 10.3)	74.54<br>(30.42 to 137.3)
Macau Special Administrative Region of China	Idiopathic epilepsy	0.361<br>(0.19 to 0.598)	101.79<br>(53.52 to 168.45)	0.546<br>(0.217 to 1.04)	79.85<br>(31.72 to 152.36)
Anhui	Idiopathic epilepsy	102<br>(69.4 to 144)	172.71<br>(117.05 to 243.87)	56.2<br>(29.4 to 97.3)	91.84<br>(48.15 to 159.12)

Beijing	Idiopathic epilepsy	16.5<br>(9.88 to 24.9)	146.91<br>(88.06 to 222.1)	19.1<br>(8.12 to 36.4)	79.18<br>(33.7 to 151.19)
Chongqing	Idiopathic epilepsy	25.9<br>(16.5 to 38.5)	164.23<br>(104.45 to 243.76)	22.4<br>(10 to 40)	83.63<br>(37.48 to 149.75)
Fujian	Idiopathic epilepsy	50.8<br>(33.6 to 74.5)	160.41<br>(106.12 to 235.58)	35.9<br>(18.8 to 63.4)	91.1<br>(47.67 to 160.94)
Gansu	Idiopathic epilepsy	65.6<br>(45.8 to 88.8)	277.4<br>(193.54 to 375.64)	29.5<br>(17.1 to 47.5)	111.12<br>(64.42 to 178.63)
Guangdong	Idiopathic epilepsy	83.3<br>(49.6 to 127)	126.5<br>(75.43 to 193.28)	95.6<br>(41.3 to 183)	81.8<br>(35.34 to 156.85)
Guangxi	Idiopathic epilepsy	73.5<br>(49.7 to 107)	169<br>(114.31 to 246.88)	49.8<br>(26.4 to 83)	102.97<br>(54.67 to 171.86)
Guizhou	Idiopathic epilepsy	87.2<br>(58.8 to 120)	262.66<br>(177.07 to 362.37)	37.5<br>(22.2 to 57.9)	110.98<br>(65.7 to 171.28)
Hainan	Idiopathic epilepsy	10.7<br>(6.47 to 15.7)	154.21<br>(92.86 to 225.34)	9<br>(4.18 to 15.3)	93.88<br>(43.55 to 159.78)
Hebei	Idiopathic epilepsy	100<br>(53.4 to 166)	157.81<br>(83.83 to 260.65)	97.2<br>(41.5 to 176)	127.18<br>(54.26 to 230.69)
Heilongjiang	Idiopathic epilepsy	63.1<br>(35.6 to 99.5)	173.2<br>(97.54 to 272.84)	43.8<br>(18.1 to 79.6)	111.51<br>(46.03 to 202.71)
Henan	Idiopathic epilepsy	133<br>(83.6 to 201)	150.89<br>(94.74 to 227.44)	91<br>(45.3 to 165)	94.47<br>(47 to 171.42)
Hubei	Idiopathic epilepsy	85.6<br>(56.4 to 123)	154.14<br>(101.58 to 221.01)	52.9<br>(27.9 to 90.5)	95.69<br>(50.39 to 163.61)
Hunan	Idiopathic epilepsy	108<br>(68.3 to 158)	173.78<br>(109.83 to 254.71)	67.2<br>(34.7 to 115)	100.09<br>(51.61 to 171.06)
Inner Mongolia	Idiopathic epilepsy	39.2<br>(26.2 to 57.5)	172.38<br>(115.4 to 252.74)	22.6<br>(10.8 to 38.5)	87.44<br>(41.65 to 148.71)
Jiangsu	Idiopathic epilepsy	109<br>(66.8 to 168)	157.82<br>(97.01 to 243.64)	81.7<br>(35.6 to 147)	100.07<br>(43.63 to 180.13)
Jiangxi	Idiopathic epilepsy	94.8<br>(66.4 to 130)	239.75<br>(167.93 to 329.62)	46.3<br>(24.3 to 77.9)	97.3<br>(51.12 to 163.9)
Jilin	Idiopathic epilepsy	41.2<br>(25.2 to 63.5)	158.58<br>(96.96 to 244.58)	25.6<br>(11.5 to 45.8)	91.73<br>(41.26 to 164.3)
Liaoning	Idiopathic epilepsy	61<br>(39.6 to 89.5)	149.63<br>(96.97 to 219.49)	42.5<br>(21.5 to 73.6)	95.28<br>(48.2 to 164.8)
Ningxia	Idiopathic epilepsy	15.2<br>(9.51 to 22.9)	308.46<br>(192.61 to 463.27)	11.2<br>(5.24 to 20.1)	159.87<br>(74.66 to 285.92)
Qinghai	Idiopathic epilepsy	16.4<br>(9.81 to 21.9)	345.87<br>(207.07 to 462.11)	8.48<br>(5.22 to 12)	135<br>(83.02 to 190.64)
Shaanxi	Idiopathic epilepsy	50.8<br>(34.5 to 71)	147.57<br>(100.17 to 206.36)	37.8<br>(22 to 60.2)	95.61<br>(55.65 to 152.33)
Shandong	Idiopathic epilepsy	133<br>(86.6 to 195)	155.31<br>(101.2 to 228.27)	92.4<br>(46.2 to 163)	92.99<br>(46.5 to 163.81)
Shanghai	Idiopathic epilepsy	17.9<br>(10.7 to 28.3)	128.05<br>(76.4 to 202.43)	22<br>(9.39 to 41.9)	78.76<br>(33.6 to 149.82)
Shanxi	Idiopathic epilepsy	54.8<br>(35.5 to 80.9)	180.96<br>(117.09 to 267.29)	38.1<br>(19.1 to 66.9)	98.52<br>(49.53 to 173.13)
Sichuan	Idiopathic epilepsy	214<br>(138 to 309)	191.64<br>(124.07 to 277)	93.9<br>(47.4 to 162)	112.45<br>(56.81 to 194.23)
Tianjin	Idiopathic epilepsy	14.3<br>(8.93 to 20.9)	155.94<br>(97.12 to 226.89)	14.2<br>(7.35 to 24.5)	93.31<br>(48.14 to 160.57)
Tibet	Idiopathic epilepsy	17.1<br>(7.63 to 23.6)	731.92<br>(327.32 to 1011.78)	11.5<br>(4.96 to 16.8)	333.21<br>(143.58 to 486.99)
Xinjiang	Idiopathic epilepsy	36<br>(24.8 to 49.5)	228.23<br>(157.28 to 314.09)	30<br>(17.7 to 47)	123.15<br>(72.61 to 192.91)
Yunnan	Idiopathic epilepsy	127<br>(82.1 to 168)	325.06<br>(210.79 to 430.55)	67.5<br>(40.4 to 100)	137.34<br>(82.12 to 203.29)
Zhejiang	Idiopathic epilepsy	63.4<br>(40.9 to 93.8)	143.71<br>(92.68 to 212.64)	49.5<br>(21.6 to 88.6)	80.94<br>(35.23 to 144.81)
China	Multiple sclerosis	52.5<br>(40.5 to 67.9)	4.39<br>(3.38 to 5.67)	72.9<br>(62.1 to 84.9)	5.16<br>(4.4 to 6.01)
Taiwan	Multiple sclerosis	0.826<br>(0.711 to 0.918)	4.05<br>(3.48 to 4.5)	1.44<br>(1.24 to 1.68)	6.11<br>(5.25 to 7.11)
Hong Kong Special Administrative Region of China	Multiple sclerosis	0.229<br>(0.173 to 0.262)	3.96<br>(3.01 to 4.55)	0.416<br>(0.225 to 0.519)	5.54<br>(3 to 6.91)

Macau Special Administrative Region of China	Multiple sclerosis	0.0135<br>(0.00931 to 0.0172)	3.8<br>(2.62 to 4.84)	0.0251<br>(0.0146 to 0.034)	3.67<br>(2.13 to 4.97)
Anhui	Multiple sclerosis	2.97<br>(1.82 to 3.58)	5.01<br>(3.07 to 6.04)	3.01<br>(2.43 to 3.61)	4.92<br>(3.97 to 5.91)
Beijing	Multiple sclerosis	1.17<br>(0.577 to 1.57)	10.46<br>(5.15 to 14.04)	1.3<br>(0.795 to 1.75)	5.39<br>(3.3 to 7.27)
Chongqing	Multiple sclerosis	0.641<br>(0.478 to 1.08)	4.06<br>(3.03 to 6.81)	1.23<br>(0.983 to 1.48)	4.6<br>(3.68 to 5.55)
Fujian	Multiple sclerosis	1.02<br>(0.758 to 1.39)	3.21<br>(2.39 to 4.41)	1.78<br>(1.29 to 2.16)	4.51<br>(3.28 to 5.48)
Gansu	Multiple sclerosis	1.15<br>(0.831 to 1.89)	4.87<br>(3.51 to 7.99)	1.36<br>(1.1 to 1.69)	5.12<br>(4.13 to 6.34)
Guangdong	Multiple sclerosis	2.66<br>(1.58 to 3.5)	4.04<br>(2.4 to 5.32)	5.07<br>(3.32 to 6.2)	4.34<br>(2.84 to 5.31)
Guangxi	Multiple sclerosis	1.81<br>(1.11 to 2.49)	4.16<br>(2.55 to 5.71)	2.16<br>(1.74 to 2.93)	4.47<br>(3.61 to 6.07)
Guizhou	Multiple sclerosis	1.21<br>(0.83 to 2.42)	3.65<br>(2.5 to 7.29)	1.52<br>(1.17 to 1.9)	4.51<br>(3.46 to 5.62)
Hainan	Multiple sclerosis	0.197<br>(0.124 to 0.297)	2.82<br>(1.79 to 4.27)	0.325<br>(0.253 to 0.416)	3.39<br>(2.63 to 4.34)
Hebei	Multiple sclerosis	2.64<br>(2.13 to 3.22)	4.15<br>(3.34 to 5.07)	4.46<br>(3.47 to 5.62)	5.83<br>(4.54 to 7.35)
Heilongjiang	Multiple sclerosis	2.09<br>(1.69 to 3.04)	5.74<br>(4.64 to 8.33)	2.76<br>(2.19 to 3.77)	7.02<br>(5.59 to 9.6)
Henan	Multiple sclerosis	3.07<br>(2.41 to 3.75)	3.48<br>(2.74 to 4.25)	4.64<br>(3.87 to 5.74)	4.81<br>(4.02 to 5.97)
Hubei	Multiple sclerosis	2.02<br>(1.67 to 2.64)	3.63<br>(3 to 4.76)	2.99<br>(2.41 to 3.75)	5.4<br>(4.37 to 6.78)
Hunan	Multiple sclerosis	2.53<br>(1.89 to 4.41)	4.07<br>(3.05 to 7.09)	3.4<br>(2.75 to 4.57)	5.07<br>(4.1 to 6.8)
Inner Mongolia	Multiple sclerosis	1.27<br>(0.898 to 1.75)	5.61<br>(3.95 to 7.7)	1.61<br>(1.28 to 2.17)	6.24<br>(4.94 to 8.37)
Jiangsu	Multiple sclerosis	3.77<br>(2.57 to 4.53)	5.47<br>(3.74 to 6.58)	4.46<br>(3.21 to 5.51)	5.46<br>(3.93 to 6.74)
Jiangxi	Multiple sclerosis	1.63<br>(0.995 to 2.71)	4.13<br>(2.52 to 6.86)	2.03<br>(1.66 to 2.4)	4.28<br>(3.5 to 5.05)
Jilin	Multiple sclerosis	1.28<br>(1.04 to 1.86)	4.95<br>(4.02 to 7.18)	1.76<br>(1.38 to 2.35)	6.31<br>(4.96 to 8.42)
Liaoning	Multiple sclerosis	2.3<br>(1.86 to 3.13)	5.64<br>(4.56 to 7.68)	2.97<br>(2.38 to 3.92)	6.65<br>(5.33 to 8.77)
Ningxia	Multiple sclerosis	0.166<br>(0.132 to 0.259)	3.36<br>(2.68 to 5.25)	0.322<br>(0.259 to 0.401)	4.59<br>(3.69 to 5.72)
Qinghai	Multiple sclerosis	0.219<br>(0.131 to 0.374)	4.63<br>(2.76 to 7.89)	0.312<br>(0.244 to 0.452)	4.97<br>(3.89 to 7.2)
Shaanxi	Multiple sclerosis	1.21<br>(0.703 to 1.41)	3.51<br>(2.04 to 4.1)	2.02<br>(1.62 to 2.51)	5.12<br>(4.11 to 6.35)
Shandong	Multiple sclerosis	4.06<br>(3.25 to 5.22)	4.75<br>(3.79 to 6.1)	5.48<br>(4.29 to 6.94)	5.51<br>(4.31 to 6.98)
Shanghai	Multiple sclerosis	0.953<br>(0.664 to 1.17)	6.82<br>(4.75 to 8.39)	1.69<br>(1.05 to 2.09)	6.05<br>(3.78 to 7.49)
Shanxi	Multiple sclerosis	1.28<br>(1.03 to 1.9)	4.23<br>(3.41 to 6.29)	1.96<br>(1.55 to 2.48)	5.08<br>(4.01 to 6.42)
Sichuan	Multiple sclerosis	3.79<br>(3.15 to 5.61)	3.4<br>(2.83 to 5.02)	4.15<br>(3.37 to 5.2)	4.97<br>(4.03 to 6.23)
Tianjin	Multiple sclerosis	0.482<br>(0.405 to 0.603)	5.25<br>(4.4 to 6.56)	0.89<br>(0.677 to 1.17)	5.83<br>(4.44 to 7.69)
Tibet	Multiple sclerosis	0.108<br>(0.0515 to 0.28)	4.61<br>(2.21 to 12.01)	0.14<br>(0.0963 to 0.234)	4.04<br>(2.79 to 6.78)
Xinjiang	Multiple sclerosis	0.658<br>(0.535 to 0.989)	4.17<br>(3.39 to 6.27)	1.57<br>(1.13 to 1.97)	6.46<br>(4.64 to 8.07)
Yunnan	Multiple sclerosis	1.58<br>(0.908 to 2.57)	4.05<br>(2.33 to 6.6)	2.14<br>(1.74 to 2.69)	4.36<br>(3.54 to 5.47)
Zhejiang	Multiple sclerosis	2.31<br>(1.31 to 3.09)	5.23<br>(2.97 to 7.01)	2.92<br>(1.94 to 3.57)	4.77<br>(3.17 to 5.84)
China	Motor neuron disease	57.3<br>(45.8 to 91.6)	4.79<br>(3.83 to 7.65)	95.2<br>(82.8 to 105)	6.74<br>(5.86 to 7.43)
Taiwan	Motor neuron disease	1.96<br>(1.83 to 2.09)	9.59<br>(8.99 to 10.26)	3.9<br>(3.56 to 4.26)	16.52<br>(15.11 to 18.04)

Hong Kong Special Administrative Region of China	Motor neuron disease	0.602 (0.263 to 0.724)	10.44 (4.57 to 12.57)	0.459 (0.378 to 0.608)	6.11 (5.03 to 8.09)
Macau Special Administrative Region of China	Motor neuron disease	0.048 (0.0158 to 0.0574)	13.52 (4.44 to 16.17)	0.108 (0.0357 to 0.15)	15.85 (5.22 to 21.98)
Anhui	Motor neuron disease	3.9 (2.81 to 5.58)	6.58 (4.74 to 9.42)	3.75 (2.94 to 4.47)	6.14 (4.81 to 7.32)
Beijing	Motor neuron disease	1.2 (0.625 to 1.9)	10.73 (5.58 to 16.96)	3.11 (1.42 to 3.8)	12.92 (5.88 to 15.77)
Chongqing	Motor neuron disease	0.542 (0.355 to 1.04)	3.43 (2.25 to 6.57)	1.01 (0.801 to 1.73)	3.77 (3 to 6.46)
Fujian	Motor neuron disease	1.45 (1.06 to 2.08)	4.59 (3.37 to 6.57)	2.65 (1.96 to 3.16)	6.72 (4.97 to 8.01)
Gansu	Motor neuron disease	1.57 (1.03 to 2.67)	6.63 (4.35 to 11.3)	1.4 (1.16 to 1.81)	5.27 (4.35 to 6.83)
Guangdong	Motor neuron disease	2.8 (2.05 to 4.47)	4.26 (3.12 to 6.79)	7.63 (6.19 to 9.25)	6.53 (5.29 to 7.92)
Guangxi	Motor neuron disease	1.31 (0.937 to 2.25)	3 (2.16 to 5.18)	2.36 (1.9 to 3.59)	4.88 (3.94 to 7.43)
Guizhou	Motor neuron disease	0.847 (0.595 to 1.85)	2.55 (1.79 to 5.56)	1.05 (0.819 to 1.87)	3.09 (2.42 to 5.53)
Hainan	Motor neuron disease	0.2 (0.14 to 0.36)	2.88 (2.01 to 5.17)	0.373 (0.295 to 0.613)	3.89 (3.08 to 6.4)
Hebei	Motor neuron disease	2.08 (1.59 to 3.46)	3.27 (2.5 to 5.44)	4.31 (3.53 to 6.57)	5.64 (4.62 to 8.6)
Heilongjiang	Motor neuron disease	1.51 (1.22 to 2.31)	4.13 (3.34 to 6.34)	2.77 (2.28 to 3.5)	7.06 (5.81 to 8.92)
Henan	Motor neuron disease	2.84 (2.04 to 5.53)	3.22 (2.31 to 6.26)	4.44 (3.65 to 7.17)	4.61 (3.79 to 7.45)
Hubei	Motor neuron disease	2.31 (1.57 to 3.93)	4.15 (2.83 to 7.07)	3.63 (2.95 to 4.63)	6.57 (5.33 to 8.38)
Hunan	Motor neuron disease	1.81 (1.27 to 3.71)	2.91 (2.05 to 5.97)	2.93 (2.28 to 5.71)	4.36 (3.39 to 8.51)
Inner Mongolia	Motor neuron disease	1.2 (0.832 to 2.08)	5.26 (3.66 to 9.15)	1.66 (1.37 to 2.09)	6.43 (5.31 to 8.07)
Jiangsu	Motor neuron disease	4.23 (2.96 to 6.37)	6.15 (4.3 to 9.26)	8.5 (4.75 to 10.3)	10.41 (5.82 to 12.56)
Jiangxi	Motor neuron disease	2.58 (1.51 to 4.47)	6.53 (3.82 to 11.32)	2.9 (2.33 to 3.47)	6.1 (4.9 to 7.3)
Jilin	Motor neuron disease	1.13 (0.871 to 1.72)	4.34 (3.36 to 6.63)	1.81 (1.45 to 2.33)	6.48 (5.2 to 8.34)
Liaoning	Motor neuron disease	2.63 (1.87 to 3.55)	6.44 (4.58 to 8.69)	5.74 (3.14 to 6.97)	12.87 (7.04 to 15.6)
Ningxia	Motor neuron disease	0.462 (0.245 to 0.777)	9.36 (4.95 to 15.72)	0.535 (0.347 to 0.658)	7.62 (4.94 to 9.38)
Qinghai	Motor neuron disease	0.321 (0.209 to 0.513)	6.77 (4.42 to 10.83)	0.402 (0.315 to 0.506)	6.4 (5.01 to 8.06)
Shaanxi	Motor neuron disease	1.74 (1.24 to 2.62)	5.07 (3.61 to 7.62)	2.65 (2.1 to 3.28)	6.71 (5.31 to 8.3)
Shandong	Motor neuron disease	3.47 (2.61 to 5.72)	4.06 (3.05 to 6.69)	6.86 (5.7 to 8.36)	6.9 (5.74 to 8.41)
Shanghai	Motor neuron disease	1.61 (0.716 to 2.5)	11.5 (5.12 to 17.92)	3.95 (1.53 to 4.91)	14.15 (5.49 to 17.56)
Shanxi	Motor neuron disease	1.39 (0.948 to 2.27)	4.58 (3.13 to 7.5)	2.19 (1.77 to 2.89)	5.67 (4.59 to 7.49)
Sichuan	Motor neuron disease	4.91 (3.34 to 8.18)	4.4 (2.99 to 7.33)	4.53 (3.69 to 6)	5.42 (4.42 to 7.18)
Tianjin	Motor neuron disease	0.636 (0.433 to 0.913)	6.91 (4.71 to 9.92)	1.77 (1.03 to 2.13)	11.62 (6.75 to 13.98)
Tibet	Motor neuron disease	0.0805 (0.0521 to 0.132)	3.45 (2.23 to 5.67)	0.12 (0.092 to 0.173)	3.46 (2.66 to 5.02)
Xinjiang	Motor neuron disease	0.875 (0.614 to 1.41)	5.55 (3.89 to 8.95)	1.68 (1.33 to 2.38)	6.91 (5.45 to 9.77)
Yunnan	Motor neuron disease	1.8 (1.2 to 3.05)	4.61 (3.08 to 7.84)	2.25 (1.84 to 3.08)	4.58 (3.75 to 6.27)
Zhejiang	Motor neuron disease	3.26 (1.72 to 5.15)	7.39 (3.91 to 11.68)	5.62 (3.02 to 6.79)	9.19 (4.94 to 11.09)

China	Other neurological disorders	471<br>(333 to 578)	39.31<br>(27.8 to 48.29)	437<br>(357 to 541)	30.97<br>(25.29 to 38.27)
Taiwan	Other neurological disorders	6.84<br>(4.94 to 9.39)	33.53<br>(24.2 to 46.02)	7.46<br>(5.21 to 10.9)	31.65<br>(22.08 to 46.23)
Hong Kong Special Administrative Region of China	Other neurological disorders	1.56<br>(1.18 to 2.09)	27.05<br>(20.4 to 36.34)	1.84<br>(1.3 to 2.61)	24.53<br>(17.36 to 34.71)
Macau Special Administrative Region of China	Other neurological disorders	0.137<br>(0.0954 to 0.176)	38.47<br>(26.89 to 49.57)	0.27<br>(0.183 to 0.369)	39.45<br>(26.82 to 53.9)
Anhui	Other neurological disorders	25.9<br>(18.4 to 34.8)	43.7<br>(31.04 to 58.67)	18.1<br>(13.2 to 25.6)	29.6<br>(21.54 to 41.83)
Beijing	Other neurological disorders	5.01<br>(3.06 to 6.79)	44.63<br>(27.27 to 60.52)	7.48<br>(5.37 to 10.6)	31.05<br>(22.3 to 44.06)
Chongqing	Other neurological disorders	5.38<br>(3.09 to 7.78)	34.08<br>(19.57 to 49.32)	6.73<br>(4.43 to 9.99)	25.17<br>(16.57 to 37.38)
Fujian	Other neurological disorders	11.5<br>(7.72 to 15.9)	36.45<br>(24.41 to 50.25)	11.4<br>(7.97 to 16.3)	29.04<br>(20.23 to 41.36)
Gansu	Other neurological disorders	13.4<br>(8.62 to 18.8)	56.77<br>(36.46 to 79.45)	8.35<br>(5.84 to 11.7)	31.42<br>(21.99 to 44.06)
Guangdong	Other neurological disorders	20.7<br>(12.6 to 29)	31.52<br>(19.08 to 44.03)	30.8<br>(20.5 to 47.4)	26.33<br>(17.55 to 40.55)
Guangxi	Other neurological disorders	13.9<br>(8.43 to 20.1)	31.87<br>(19.39 to 46.27)	13.4<br>(8.96 to 20)	27.8<br>(18.54 to 41.32)
Guizhou	Other neurological disorders	22.2<br>(12.3 to 30.8)	66.76<br>(37.19 to 92.66)	12.9<br>(9.24 to 17.3)	38.17<br>(27.31 to 51.17)
Hainan	Other neurological disorders	2.14<br>(1.23 to 3.1)	30.77<br>(17.7 to 44.44)	2.45<br>(1.53 to 3.68)	25.52<br>(15.91 to 38.34)
Hebei	Other neurological disorders	22.7<br>(14 to 34.2)	35.64<br>(22 to 53.75)	30<br>(20 to 45.3)	39.29<br>(26.21 to 59.25)
Heilongjiang	Other neurological disorders	15.4<br>(10.2 to 22.1)	42.25<br>(27.96 to 60.64)	14.2<br>(9.77 to 20.7)	36.3<br>(24.88 to 52.61)
Henan	Other neurological disorders	27.8<br>(17 to 41.7)	31.55<br>(19.27 to 47.25)	25.9<br>(17.1 to 40.5)	26.91<br>(17.73 to 42.1)
Hubei	Other neurological disorders	18.8<br>(11.1 to 26.7)	33.82<br>(19.89 to 48.1)	15.7<br>(11.2 to 22.4)	28.39<br>(20.23 to 40.49)
Hunan	Other neurological disorders	21.5<br>(12.3 to 30.9)	34.55<br>(19.74 to 49.66)	19.6<br>(13.3 to 28.3)	29.14<br>(19.87 to 42.1)
Inner Mongolia	Other neurological disorders	9.47<br>(5.83 to 13.1)	41.64<br>(25.63 to 57.42)	7.98<br>(5.85 to 10.9)	30.81<br>(22.58 to 41.94)
Jiangsu	Other neurological disorders	23.4<br>(15.3 to 33.7)	34<br>(22.28 to 48.94)	25.6<br>(17.7 to 37)	31.36<br>(21.7 to 45.28)
Jiangxi	Other neurological disorders	32.1<br>(16.4 to 43.9)	81.27<br>(41.36 to 111.13)	19.3<br>(13.9 to 25.9)	40.54<br>(29.21 to 54.56)
Jilin	Other neurological disorders	10.4<br>(6.89 to 14.3)	40.06<br>(26.57 to 55.25)	8.9<br>(6.36 to 12.5)	31.9<br>(22.8 to 44.72)
Liaoning	Other neurological disorders	16.1<br>(11.1 to 21.4)	39.55<br>(27.1 to 52.55)	17.1<br>(13 to 22.7)	38.25<br>(29.03 to 50.83)
Ningxia	Other neurological disorders	2.99<br>(1.66 to 4.56)	60.59<br>(33.54 to 92.26)	2.79<br>(1.56 to 4.52)	39.69<br>(22.27 to 64.41)
Qinghai	Other neurological disorders	2.33<br>(1.48 to 3.26)	49.19<br>(31.15 to 68.81)	1.87<br>(1.35 to 2.51)	29.69<br>(21.5 to 39.99)
Shaanxi	Other neurological disorders	11.5<br>(7.98 to 15.4)	33.34<br>(23.18 to 44.81)	11.5<br>(8.42 to 15.5)	29.07<br>(21.32 to 39.2)
Shandong	Other neurological disorders	27.9<br>(17.6 to 38.8)	32.55<br>(20.62 to 45.35)	28.3<br>(19.5 to 40.4)	28.48<br>(19.63 to 40.67)
Shanghai	Other neurological disorders	5.39<br>(3.27 to 7.17)	38.58<br>(23.38 to 51.29)	8.74<br>(6.06 to 12.2)	31.29<br>(21.68 to 43.59)
Shanxi	Other neurological disorders	11.7<br>(7.26 to 17.1)	38.8<br>(23.97 to 56.44)	11.6<br>(7.98 to 16.9)	29.97<br>(20.66 to 43.82)
Sichuan	Other neurological disorders	40.4<br>(23.7 to 58.4)	36.17<br>(21.23 to 52.29)	25.2<br>(17.1 to 37.5)	30.21<br>(20.49 to 44.96)
Tianjin	Other neurological disorders	3.67<br>(2.44 to 4.9)	39.92<br>(26.5 to 53.3)	5.34<br>(4.04 to 7.23)	35.02<br>(26.5 to 47.36)
Tibet	Other neurological disorders	1.9<br>(1.02 to 2.84)	81.66<br>(43.9 to 121.82)	2.05<br>(1.28 to 3.01)	59.21<br>(36.95 to 87.03)
Xinjiang	Other neurological disorders	8.05<br>(5.35 to 10.9)	51.03<br>(33.94 to 69.29)	9.65<br>(7.3 to 13.2)	39.62<br>(29.94 to 54.07)

Yunnan	Other neurological disorders	18.9 (11 to 27.4)	48.54 (28.12 to 70.26)	15.3 (10.3 to 21.4)	31.09 (20.92 to 43.54)
Zhejiang	Other neurological disorders	16.2 (9.6 to 22.6)	36.84 (21.77 to 51.28)	17.1 (12 to 24.2)	27.9 (19.68 to 39.62)
China	Mental disorders	16600 (12400 to 21600)	1387.83 (1033.63 to 1800.54)	22200 (16500 to 28700)	1573.81 (1169.59 to 2030.92)
Taiwan	Mental disorders	258 (192 to 336)	1265.94 (939.74 to 1647.62)	357 (265 to 460)	1511.96 (1125.42 to 1951.78)
Hong Kong Special Administrative Region of China	Mental disorders	87.3 (64.3 to 113)	1514.5 (1116.23 to 1966.44)	133 (97.6 to 170)	1765.07 (1299.89 to 2270.11)
Macau Special Administrative Region of China	Mental disorders	5 (3.75 to 6.51)	1410.09 (1055.4 to 1835.44)	11.2 (8.37 to 14.4)	1633.1 (1223.35 to 2107.34)
Anhui	Mental disorders	820 (602 to 1060)	1384.12 (1016.22 to 1794.29)	951 (708 to 1230)	1556.06 (1157.27 to 2015.01)
Beijing	Mental disorders	160 (119 to 206)	1423.83 (1065.04 to 1832.63)	383 (289 to 491)	1590.21 (1197.35 to 2039.07)
Chongqing	Mental disorders	219 (162 to 285)	1386.86 (1026.12 to 1803.22)	410 (304 to 532)	1533.63 (1136.31 to 1989.66)
Fujian	Mental disorders	418 (309 to 541)	1320.5 (977.22 to 1708.75)	598 (447 to 765)	1516.11 (1133.79 to 1941.22)
Gansu	Mental disorders	367 (271 to 472)	1551.27 (1144.19 to 1994.76)	477 (354 to 615)	1795.56 (1331.91 to 2315.45)
Guangdong	Mental disorders	901 (677 to 1160)	1369.34 (1028.16 to 1762.95)	1840 (1380 to 2370)	1573.15 (1183.58 to 2024.46)
Guangxi	Mental disorders	589 (439 to 756)	1355.29 (1009.8 to 1737.23)	754 (567 to 965)	1561.24 (1173.18 to 1997.77)
Guizhou	Mental disorders	431 (317 to 556)	1298.32 (956.5 to 1676.04)	504 (371 to 651)	1488.49 (1097 to 1925.4)
Hainan	Mental disorders	88.9 (65.9 to 116)	1275.82 (945.32 to 1666.55)	141 (104 to 183)	1468.73 (1089.59 to 1906.38)
Hebei	Mental disorders	894 (666 to 1160)	1404.63 (1045.74 to 1818.1)	1210 (910 to 1570)	1588.97 (1191.29 to 2053.57)
Heilongjiang	Mental disorders	495 (366 to 643)	1358.56 (1002.92 to 1762.92)	642 (477 to 827)	1635.12 (1214.01 to 2107.84)
Henan	Mental disorders	1230 (905 to 1600)	1393.39 (1025.16 to 1810.74)	1460 (1090 to 1880)	1513.18 (1129.79 to 1950.15)
Hubei	Mental disorders	810 (595 to 1050)	1458.29 (1071.32 to 1895.22)	925 (683 to 1190)	1672.87 (1235.78 to 2156.25)
Hunan	Mental disorders	866 (637 to 1120)	1392.23 (1024.98 to 1805.26)	1040 (773 to 1350)	1551.71 (1151.58 to 2009.77)
Inner Mongolia	Mental disorders	296 (220 to 385)	1301.98 (967.4 to 1693.33)	405 (302 to 522)	1565.38 (1166.19 to 2014.19)
Jiangsu	Mental disorders	948 (700 to 1230)	1377.88 (1016.85 to 1792.82)	1260 (934 to 1630)	1542.9 (1143.38 to 1992.03)
Jiangxi	Mental disorders	535 (395 to 689)	1352.29 (999.39 to 1743.7)	725 (541 to 936)	1525.72 (1137.47 to 1968.78)
Jilin	Mental disorders	340 (252 to 443)	1309.86 (970.77 to 1705.51)	436 (325 to 563)	1561.56 (1164.89 to 2020.21)
Liaoning	Mental disorders	530 (395 to 681)	1299.34 (969.14 to 1668.26)	683 (508 to 880)	1529.28 (1137.96 to 1970.94)
Ningxia	Mental disorders	60.3 (44.4 to 78.3)	1220.87 (898.79 to 1585.29)	101 (75.3 to 131)	1443.26 (1072.94 to 1864.96)
Qinghai	Mental disorders	62.6 (45.8 to 81)	1321.41 (967.68 to 1709.84)	96.9 (71 to 126)	1542.07 (1129.76 to 2001.62)
Shaanxi	Mental disorders	468 (347 to 606)	1360.01 (1009.43 to 1762.23)	611 (459 to 792)	1546.63 (1161.29 to 2004.91)
Shandong	Mental disorders	1390 (1020 to 1800)	1621.94 (1191.69 to 2101.53)	1830 (1360 to 2360)	1841.41 (1371.22 to 2369.75)
Shanghai	Mental disorders	197 (147 to 253)	1406.89 (1050.49 to 1813.69)	427 (318 to 552)	1528.81 (1139.16 to 1976.12)
Shanxi	Mental disorders	387 (288 to 506)	1278.82 (950.3 to 1672.56)	571 (426 to 738)	1478.63 (1103.32 to 1911.8)
Sichuan	Mental disorders	1520 (1130 to 1970)	1365.9 (1013.49 to 1766.06)	1260 (936 to 1620)	1503.82 (1121.65 to 1945.56)
Tianjin	Mental disorders	124 (92.6 to 161)	1344.88 (1006.96 to 1749.11)	235 (176 to 304)	1537.69 (1151.63 to 1989.78)

Tibet	Mental disorders	27.8 (20.6 to 36.1)	1194.21 (883.66 to 1549.01)	45 (33.3 to 58.2)	1302.96 (964.53 to 1684.6)
Xinjiang	Mental disorders	192 (141 to 250)	1215.31 (895.94 to 1584.36)	336 (249 to 436)	1379.03 (1022.49 to 1790.8)
Yunnan	Mental disorders	522 (386 to 674)	1338.88 (990.16 to 1729.36)	749 (559 to 965)	1522.77 (1136.3 to 1962.01)
Zhejiang	Mental disorders	629 (467 to 814)	1427.5 (1058.63 to 1846.5)	983 (723 to 1280)	1606.56 (1182.29 to 2085.33)
China	Schizophrenia	2350 (1760 to 2890)	196.42 (147.38 to 241.82)	3670 (2750 to 4500)	259.72 (194.79 to 318.7)
Taiwan	Schizophrenia	31.1 (23.2 to 38.8)	152.6 (113.93 to 190.12)	47.6 (35.4 to 59.3)	202.03 (149.97 to 251.64)
Hong Kong Special Administrative Region of China	Schizophrenia	13 (9.63 to 16.6)	225.96 (167.06 to 288.19)	19.3 (14.5 to 24.1)	256.96 (193.11 to 320.81)
Macau Special Administrative Region of China	Schizophrenia	0.82 (0.608 to 1.03)	231.05 (171.38 to 291.41)	1.97 (1.45 to 2.46)	288.5 (212.73 to 359.21)
Anhui	Schizophrenia	102 (76.1 to 126)	172.12 (128.35 to 212.62)	138 (104 to 169)	225.61 (169.83 to 276.71)
Beijing	Schizophrenia	30.8 (22.8 to 38.5)	274.46 (203.6 to 342.96)	81.4 (60.5 to 103)	337.64 (250.88 to 425.76)
Chongqing	Schizophrenia	29.8 (22.2 to 37.1)	189.12 (140.54 to 234.98)	59.4 (44.4 to 74.1)	222.14 (166.04 to 277.3)
Fujian	Schizophrenia	60.9 (45.5 to 75.3)	192.47 (143.76 to 238)	104 (76.7 to 129)	263.01 (194.74 to 326.47)
Gansu	Schizophrenia	65.7 (48.3 to 82.8)	277.9 (204.22 to 350.25)	95.8 (70.5 to 121)	360.61 (265.39 to 453.87)
Guangdong	Schizophrenia	145 (110 to 179)	220.45 (166.85 to 272.07)	359 (271 to 440)	307.33 (231.98 to 376.55)
Guangxi	Schizophrenia	113 (83.5 to 141)	259.56 (192.07 to 324.98)	164 (122 to 205)	338.78 (252.6 to 423.81)
Guizhou	Schizophrenia	50 (37.2 to 62.4)	150.67 (112.07 to 187.88)	68.1 (50.1 to 84.6)	201.31 (147.98 to 250.23)
Hainan	Schizophrenia	11.2 (8.33 to 14.2)	160.96 (119.55 to 203.22)	21.7 (15.9 to 27.1)	226.55 (166.26 to 283.13)
Hebei	Schizophrenia	135 (100 to 167)	212.35 (157.2 to 263.05)	200 (150 to 248)	261.5 (196.62 to 323.95)
Heilongjiang	Schizophrenia	77.8 (58.6 to 96.9)	213.35 (160.85 to 265.75)	113 (85.2 to 139)	288.91 (217.06 to 354.61)
Henan	Schizophrenia	155 (114 to 193)	175.4 (129.59 to 218.71)	212 (157 to 266)	220.05 (163.11 to 276.55)
Hubei	Schizophrenia	103 (76.4 to 128)	185.25 (137.45 to 230)	133 (99 to 164)	241.3 (178.98 to 296.7)
Hunan	Schizophrenia	87.8 (64 to 110)	141.19 (102.86 to 177.33)	121 (88.9 to 154)	180.83 (132.44 to 229.29)
Inner Mongolia	Schizophrenia	39.6 (29.1 to 49.8)	173.94 (127.85 to 218.98)	66 (48.7 to 82.9)	254.87 (188.2 to 319.99)
Jiangsu	Schizophrenia	122 (90.5 to 149)	176.7 (131.46 to 215.92)	184 (139 to 229)	225.42 (169.9 to 280.38)
Jiangxi	Schizophrenia	86.7 (64.8 to 107)	219.43 (164.02 to 270.77)	138 (103 to 169)	289.83 (216.93 to 355.48)
Jilin	Schizophrenia	41.7 (30.6 to 53.4)	160.86 (117.92 to 205.64)	61.2 (44.9 to 77)	219.6 (161.16 to 276.01)
Liaoning	Schizophrenia	83.1 (61.7 to 105)	203.73 (151.24 to 256.51)	114 (84.5 to 142)	254.67 (189.32 to 317.17)
Ningxia	Schizophrenia	7.7 (5.72 to 9.72)	155.89 (115.86 to 196.8)	15.7 (11.6 to 19.6)	223.03 (165.46 to 279.36)
Qinghai	Schizophrenia	7.4 (5.42 to 9.27)	156.25 (114.39 to 195.62)	13.9 (10.3 to 17.4)	221.62 (164.66 to 277.26)
Shaanxi	Schizophrenia	63.1 (46.6 to 79.1)	183.36 (135.54 to 229.89)	97.9 (72.5 to 123)	247.81 (183.42 to 310.33)
Shandong	Schizophrenia	201 (152 to 248)	235.02 (177.06 to 290.28)	329 (245 to 402)	331.25 (246.08 to 404.05)
Shanghai	Schizophrenia	40.1 (29.9 to 49.8)	286.79 (214.04 to 356.43)	90.6 (67.4 to 112)	324.15 (241.41 to 400.8)
Shanxi	Schizophrenia	53.5 (39.5 to 67.6)	176.81 (130.3 to 223.34)	90.7 (67.3 to 112)	234.71 (174.16 to 290.72)

Sichuan	Schizophrenia	219<br>(164 to 272)	195.9<br>(146.6 to 243.97)	201<br>(150 to 248)	241.14<br>(179.58 to 296.51)
Tianjin	Schizophrenia	20.2<br>(14.9 to 25.5)	219.27<br>(162.44 to 277.28)	42.6<br>(31.6 to 53.5)	278.94<br>(207.25 to 350.83)
Tibet	Schizophrenia	3.23<br>(2.41 to 4.03)	138.56<br>(103.39 to 173.08)	6.2<br>(4.53 to 7.75)	179.24<br>(131.16 to 224.35)
Xinjiang	Schizophrenia	21.3<br>(15.8 to 26.5)	135.1<br>(100.25 to 167.81)	45.5<br>(34.1 to 55.8)	186.86<br>(140.06 to 228.94)
Yunnan	Schizophrenia	74<br>(55 to 91.2)	189.77<br>(141.12 to 234.05)	126<br>(95.5 to 155)	257.2<br>(194.3 to 314.77)
Zhejiang	Schizophrenia	87.3<br>(65.4 to 107)	197.9<br>(148.42 to 242.54)	154<br>(115 to 187)	250.96<br>(187.26 to 305.79)
China	Alcohol use disorders	1510<br>(1120 to 1960)	126.28<br>(93.97 to 163.84)	2760<br>(2080 to 3570)	195.7<br>(147.59 to 252.8)
Taiwan	Alcohol use disorders	42.1<br>(30.5 to 56.5)	206.35<br>(149.56 to 276.91)	74.6<br>(56.9 to 96.4)	316.54<br>(241.21 to 408.82)
Hong Kong Special Administrative Region of China	Alcohol use disorders	5.22<br>(3.62 to 7.29)	90.64<br>(62.82 to 126.41)	8.07<br>(6 to 10.9)	107.48<br>(79.86 to 145.27)
Macau Special Administrative Region of China	Alcohol use disorders	0.418<br>(0.304 to 0.554)	117.72<br>(85.69 to 156.14)	0.999<br>(0.72 to 1.34)	146.06<br>(105.28 to 196.5)
Anhui	Alcohol use disorders	65.3<br>(47.4 to 87.8)	110.2<br>(80.05 to 148.25)	108<br>(80.5 to 140)	176.11<br>(131.61 to 229.14)
Beijing	Alcohol use disorders	15.1<br>(10.6 to 20.7)	134.45<br>(94.11 to 184.49)	40.4<br>(28.4 to 55.6)	167.63<br>(117.7 to 230.88)
Chongqing	Alcohol use disorders	19.1<br>(13.9 to 25.3)	121.14<br>(87.94 to 160.1)	39.8<br>(29.5 to 52)	148.91<br>(110.21 to 194.5)
Fujian	Alcohol use disorders	34.5<br>(26 to 45)	109.2<br>(82.13 to 142.39)	78<br>(57.2 to 102)	198.01<br>(145.09 to 259.01)
Gansu	Alcohol use disorders	45.1<br>(33.2 to 58.7)	190.64<br>(140.57 to 248.27)	66.1<br>(48.3 to 86.5)	248.65<br>(181.82 to 325.44)
Guangdong	Alcohol use disorders	111<br>(77.3 to 152)	169.24<br>(117.4 to 230.48)	290<br>(204 to 396)	248.12<br>(174.88 to 338.87)
Guangxi	Alcohol use disorders	48.9<br>(36.9 to 63.2)	112.39<br>(84.89 to 145.23)	99.4<br>(73.4 to 129)	205.68<br>(151.92 to 267.08)
Guizhou	Alcohol use disorders	71.9<br>(44.9 to 89.8)	216.55<br>(135.31 to 270.57)	124<br>(61.8 to 161)	366.96<br>(182.81 to 474.75)
Hainan	Alcohol use disorders	8.42<br>(6.16 to 11.2)	120.91<br>(88.43 to 160.91)	15.2<br>(10.9 to 20.2)	158.03<br>(114.17 to 210.19)
Hebei	Alcohol use disorders	59.5<br>(41.8 to 80.7)	93.52<br>(65.71 to 126.8)	102<br>(75.1 to 134)	133.05<br>(98.32 to 175.01)
Heilongjiang	Alcohol use disorders	43.2<br>(29.9 to 59)	118.39<br>(82.01 to 161.84)	63.4<br>(45.5 to 85.8)	161.65<br>(115.81 to 218.69)
Henan	Alcohol use disorders	95.5<br>(70.9 to 125)	108.25<br>(80.34 to 141.14)	200<br>(141 to 263)	207.82<br>(146.62 to 272.73)
Hubei	Alcohol use disorders	58.7<br>(41.8 to 78.9)	105.71<br>(75.27 to 141.95)	93.7<br>(70.3 to 121)	169.4<br>(127.11 to 218.16)
Hunan	Alcohol use disorders	67.1<br>(49.7 to 87.6)	107.93<br>(79.92 to 140.82)	117<br>(86.6 to 150)	173.96<br>(128.97 to 223.81)
Inner Mongolia	Alcohol use disorders	38.3<br>(27 to 51.6)	168.6<br>(118.72 to 226.98)	66.1<br>(48.8 to 88.4)	255.24<br>(188.42 to 341.49)
Jiangsu	Alcohol use disorders	79.7<br>(57.3 to 107)	115.77<br>(83.21 to 155.5)	124<br>(92.6 to 163)	151.57<br>(113.34 to 200.15)
Jiangxi	Alcohol use disorders	40.1<br>(30.1 to 54.3)	101.41<br>(76.03 to 137.36)	63.7<br>(47.5 to 83.4)	134.08<br>(99.82 to 175.47)
Jilin	Alcohol use disorders	31.9<br>(22.6 to 43.2)	123.09<br>(87.2 to 166.57)	45.3<br>(32.3 to 61.3)	162.57<br>(115.66 to 219.68)
Liaoning	Alcohol use disorders	57.7<br>(40.9 to 78.4)	141.42<br>(100.25 to 192.12)	72.8<br>(52.6 to 97.6)	163.12<br>(117.82 to 218.58)
Ningxia	Alcohol use disorders	5.21<br>(3.81 to 6.85)	105.47<br>(77.06 to 138.8)	12.3<br>(9.09 to 16)	175.13<br>(129.53 to 228.05)
Qinghai	Alcohol use disorders	9.29<br>(6.55 to 11.7)	196.13<br>(138.38 to 246.38)	22.8<br>(11.8 to 29.8)	362.43<br>(187.38 to 474.89)
Shaanxi	Alcohol use disorders	31.6<br>(22.2 to 42.6)	91.84<br>(64.64 to 123.66)	65.3<br>(49.3 to 86)	165.38<br>(124.82 to 217.55)
Shandong	Alcohol use disorders	99.8<br>(72 to 132)	116.57<br>(84.08 to 154.24)	177<br>(132 to 234)	178.24<br>(133.09 to 235.92)

Shanghai	Alcohol use disorders	15.2 (10.6 to 21.2)	109.14 (75.6 to 151.51)	40.4 (28.3 to 56.6)	144.53 (101.33 to 202.62)
Shanxi	Alcohol use disorders	31.3 (22.6 to 41.4)	103.33 (74.78 to 136.84)	58.5 (43 to 76.7)	151.46 (111.44 to 198.45)
Sichuan	Alcohol use disorders	131 (97.4 to 172)	117.33 (87.23 to 154.43)	157 (116 to 201)	188.48 (139 to 240.66)
Tianjin	Alcohol use disorders	9.38 (6.46 to 12.9)	101.98 (70.22 to 140.68)	23.5 (16.2 to 32.1)	153.69 (106.44 to 210.16)
Tibet	Alcohol use disorders	4.34 (3.25 to 5.68)	186.34 (139.62 to 243.77)	7.97 (6.02 to 10.4)	230.65 (174.05 to 299.67)
Xinjiang	Alcohol use disorders	18.3 (13.6 to 23.4)	115.95 (86.08 to 148.61)	47.3 (35.5 to 61.4)	194.04 (145.49 to 252.03)
Yunnan	Alcohol use disorders	85.7 (54.9 to 109)	219.92 (140.83 to 280.33)	203 (99.4 to 260)	412.6 (202.15 to 529.47)
Zhejiang	Alcohol use disorders	73.4 (53.6 to 96.4)	166.36 (121.56 to 218.53)	132 (93.8 to 176)	215.11 (153.33 to 288.31)
China	Drug use disorders	5900 (4600 to 7330)	492.77 (384.29 to 612.61)	5600 (4060 to 7230)	396.63 (287.55 to 512.11)
Taiwan	Drug use disorders	55.9 (42.3 to 71.5)	273.76 (207.18 to 350.3)	76.4 (58.5 to 95.3)	324.02 (248.23 to 404.04)
Hong Kong Special Administrative Region of China	Drug use disorders	25.3 (18.5 to 32.5)	438.88 (321.56 to 564.18)	26.1 (19 to 34.3)	348.03 (252.4 to 456.67)
Macau Special Administrative Region of China	Drug use disorders	1.66 (1.25 to 2.11)	468.41 (351.26 to 593.3)	3.1 (2.29 to 3.99)	453.77 (335.11 to 583.94)
Anhui	Drug use disorders	227 (171 to 287)	383.26 (288.78 to 485.2)	205 (147 to 266)	335 (240.1 to 435.55)
Beijing	Drug use disorders	43.5 (30.4 to 57.5)	387.59 (270.81 to 512.94)	102 (70.4 to 138)	423.51 (292.09 to 571.72)
Chongqing	Drug use disorders	93.2 (72.9 to 115)	590.76 (462.16 to 728.77)	88.8 (64.1 to 114)	332.43 (239.69 to 426.43)
Fujian	Drug use disorders	118 (89 to 152)	374.35 (281.22 to 480.06)	145 (103 to 192)	368.69 (260.92 to 488.41)
Gansu	Drug use disorders	162 (129 to 197)	684.89 (544.57 to 833.87)	105 (76.4 to 138)	395.02 (287.58 to 518.46)
Guangdong	Drug use disorders	291 (222 to 365)	442.64 (336.78 to 554.29)	487 (342 to 648)	417.22 (292.94 to 554.91)
Guangxi	Drug use disorders	280 (223 to 343)	643.88 (513.36 to 789.32)	201 (150 to 255)	416.15 (309.76 to 527.34)
Guizhou	Drug use disorders	286 (227 to 354)	861.04 (682.65 to 1065.63)	204 (152 to 261)	604.14 (448.24 to 772.54)
Hainan	Drug use disorders	25.3 (18.6 to 32.3)	362.82 (266.51 to 463.7)	38.5 (28.4 to 49.5)	401.89 (296.35 to 516.54)
Hebei	Drug use disorders	295 (226 to 366)	463.33 (354.92 to 575.32)	345 (262 to 437)	451.76 (343.07 to 571.41)
Heilongjiang	Drug use disorders	134 (97 to 175)	368.76 (266.09 to 480.83)	141 (100 to 185)	358.91 (255.81 to 470.96)
Henan	Drug use disorders	331 (249 to 423)	375.47 (282.12 to 479.33)	328 (234 to 427)	340.83 (243.29 to 443.19)
Hubei	Drug use disorders	243 (184 to 304)	437.5 (331.48 to 547.97)	204 (148 to 263)	369.02 (267.31 to 476.31)
Hunan	Drug use disorders	409 (326 to 504)	657.66 (523.88 to 810.97)	287 (216 to 356)	427.31 (321.46 to 530.65)
Inner Mongolia	Drug use disorders	105 (80 to 132)	460.97 (351.91 to 578.31)	97 (69.9 to 125)	374.49 (269.89 to 481.81)
Jiangsu	Drug use disorders	263 (194 to 341)	382.57 (281.32 to 496.09)	299 (208 to 399)	366.75 (254.59 to 488.78)
Jiangxi	Drug use disorders	258 (206 to 316)	653.3 (520.48 to 798.47)	175 (127 to 226)	368.09 (267.19 to 475.96)
Jilin	Drug use disorders	111 (83.5 to 141)	427 (321.77 to 544.43)	104 (76.4 to 133)	373.86 (274.01 to 478.43)
Liaoning	Drug use disorders	182 (127 to 242)	447.11 (311.13 to 594.31)	171 (121 to 225)	383.17 (271.75 to 502.9)
Ningxia	Drug use disorders	20.1 (14.7 to 26.3)	407.7 (297.71 to 532.59)	26.7 (18.7 to 35.6)	380.71 (266.82 to 507.43)
Qinghai	Drug use disorders	23.9 (18.7 to 30)	505.64 (394.41 to 632.57)	23.9 (17.4 to 31.2)	381.01 (277.4 to 496.81)

Shaanxi	Drug use disorders	143<br>(108 to 182)	415.23<br>(314.45 to 528.78)	160<br>(117 to 206)	404.01<br>(296.11 to 520.63)
Shandong	Drug use disorders	334<br>(250 to 427)	389.8<br>(291.5 to 498.44)	345<br>(242 to 454)	346.67<br>(243.52 to 456.44)
Shanghai	Drug use disorders	52.4<br>(37.4 to 68.7)	375.33<br>(267.67 to 491.46)	114<br>(79.5 to 152)	406.71<br>(284.48 to 545.14)
Shanxi	Drug use disorders	149<br>(116 to 185)	490.84<br>(383.52 to 609.64)	146<br>(104 to 190)	378.39<br>(270.22 to 492.87)
Sichuan	Drug use disorders	624<br>(482 to 774)	559.13<br>(432.06 to 693.22)	321<br>(241 to 411)	385.01<br>(288.79 to 492.32)
Tianjin	Drug use disorders	36.1<br>(26.3 to 46.5)	392.95<br>(285.82 to 505.36)	66.5<br>(46.5 to 89)	435.93<br>(304.63 to 583.37)
Tibet	Drug use disorders	17<br>(13.7 to 20.7)	729.53<br>(585.82 to 886.55)	14.1<br>(10.4 to 18)	407.96<br>(301.46 to 520.21)
Xinjiang	Drug use disorders	89.1<br>(70.3 to 110)	565<br>(445.75 to 696.66)	143<br>(112 to 177)	588.12<br>(461.2 to 728.05)
Yunnan	Drug use disorders	357<br>(290 to 433)	917.21<br>(743.97 to 1110.47)	255<br>(198 to 320)	518.79<br>(402.96 to 651.6)
Zhejiang	Drug use disorders	167<br>(122 to 216)	378.67<br>(277.74 to 489.47)	228<br>(160 to 303)	373.16<br>(261.24 to 495.01)
China	Depressive disorders	6290<br>(4470 to 8610)	525.14<br>(373.51 to 719)	8580<br>(6040 to 11600)	607.35<br>(427.71 to 820.18)
Taiwan	Depressive disorders	77.3<br>(54.9 to 106)	378.96<br>(269.3 to 520)	128<br>(89.9 to 173)	541.44<br>(381.26 to 733.03)
Hong Kong Special Administrative Region of China	Depressive disorders	34.7<br>(24.3 to 47.4)	602.64<br>(421.66 to 823.06)	61.3<br>(42.8 to 82.7)	816.05<br>(569.4 to 1101.12)
Macau Special Administrative Region of China	Depressive disorders	1.78<br>(1.26 to 2.44)	501.4<br>(354.54 to 687.87)	4.15<br>(2.92 to 5.68)	607.43<br>(427.29 to 830.35)
Anhui	Depressive disorders	332<br>(231 to 456)	559.53<br>(390.42 to 769.78)	392<br>(275 to 532)	641.31<br>(449.12 to 870.33)
Beijing	Depressive disorders	49<br>(34.8 to 66.9)	436.82<br>(310.22 to 596.41)	122<br>(86.2 to 167)	506.11<br>(357.67 to 691.74)
Chongqing	Depressive disorders	84.5<br>(59.5 to 116)	535.45<br>(377.14 to 738.03)	166<br>(116 to 224)	620.01<br>(432.58 to 838.48)
Fujian	Depressive disorders	158<br>(112 to 215)	498.92<br>(352.91 to 681.06)	231<br>(161 to 313)	585.5<br>(409.62 to 795.03)
Gansu	Depressive disorders	134<br>(93.9 to 185)	565.87<br>(396.8 to 781.93)	179<br>(125 to 244)	672.73<br>(471.17 to 917.03)
Guangdong	Depressive disorders	323<br>(228 to 441)	490.57<br>(346.67 to 670.58)	650<br>(455 to 887)	556.08<br>(389.45 to 759.05)
Guangxi	Depressive disorders	191<br>(136 to 260)	438.4<br>(311.75 to 598.25)	259<br>(181 to 353)	534.98<br>(374.29 to 731.03)
Guizhou	Depressive disorders	164<br>(116 to 223)	493.04<br>(349.5 to 671.42)	202<br>(141 to 276)	595.97<br>(417.1 to 815.63)
Hainan	Depressive disorders	33.8<br>(24.1 to 46.7)	485.84<br>(345.45 to 670.83)	53.3<br>(37.4 to 72.6)	556.02<br>(389.57 to 756.98)
Hebei	Depressive disorders	318<br>(226 to 436)	500.07<br>(355.58 to 684.78)	457<br>(320 to 614)	597.62<br>(419.03 to 802.9)
Heilongjiang	Depressive disorders	175<br>(123 to 239)	480.28<br>(337.78 to 655.74)	247<br>(175 to 335)	629.86<br>(444.9 to 853.4)
Henan	Depressive disorders	496<br>(352 to 680)	561.69<br>(398.7 to 770.3)	580<br>(407 to 790)	602.2<br>(423.08 to 820.25)
Hubei	Depressive disorders	347<br>(244 to 474)	624.4<br>(438.91 to 852.6)	407<br>(289 to 558)	736.35<br>(521.91 to 1008.14)
Hunan	Depressive disorders	331<br>(232 to 452)	533.02<br>(372.77 to 726.4)	399<br>(281 to 543)	594.89<br>(419.17 to 808.2)
Inner Mongolia	Depressive disorders	109<br>(77.5 to 151)	479.81<br>(341.01 to 662.25)	157<br>(110 to 214)	605.63<br>(423.92 to 824.77)
Jiangsu	Depressive disorders	358<br>(254 to 488)	520.68<br>(369.58 to 709.51)	491<br>(345 to 662)	601.89<br>(421.93 to 810.89)
Jiangxi	Depressive disorders	190<br>(133 to 261)	480.55<br>(337.11 to 659.6)	261<br>(182 to 356)	548.9<br>(383.41 to 748.53)
Jilin	Depressive disorders	124<br>(87.9 to 171)	478.93<br>(338.65 to 658.7)	174<br>(122 to 238)	622.24<br>(436.64 to 851.57)
Liaoning	Depressive disorders	184<br>(131 to 256)	451.63<br>(321.34 to 627.27)	262<br>(188 to 354)	586.49<br>(420.11 to 793.68)

Ningxia	Depressive disorders	21.2 (14.9 to 28.9)	428.26 (301.1 to 584.33)	37.2 (26 to 50.2)	529.59 (370.25 to 715.55)
Qinghai	Depressive disorders	25.8 (18.1 to 35.6)	545.66 (382.29 to 752.06)	41.5 (28.9 to 55.6)	660.08 (460.48 to 885.76)
Shaanxi	Depressive disorders	181 (127 to 249)	526.17 (370.39 to 722.51)	237 (166 to 323)	599.49 (418.94 to 817.98)
Shandong	Depressive disorders	571 (401 to 782)	667.04 (468.83 to 913.27)	743 (523 to 1010)	747.14 (526.17 to 1017.62)
Shanghai	Depressive disorders	64.5 (45.9 to 88.1)	461.62 (328.78 to 630.37)	143 (101 to 195)	510.67 (361.68 to 698.43)
Shanxi	Depressive disorders	137 (97.4 to 190)	453.71 (321.56 to 626.27)	211 (148 to 285)	545.71 (383.98 to 738.78)
Sichuan	Depressive disorders	575 (408 to 791)	515.57 (365.32 to 708.9)	507 (356 to 689)	606.92 (426.2 to 824.84)
Tianjin	Depressive disorders	42.7 (30.6 to 58.3)	464.08 (332.31 to 633.95)	83.8 (59.8 to 115)	549.18 (391.54 to 750.72)
Tibet	Depressive disorders	9.99 (7.11 to 13.7)	428.79 (305.09 to 588.81)	15.8 (11.1 to 21.5)	455.74 (320.72 to 622.74)
Xinjiang	Depressive disorders	69.4 (49 to 95)	439.97 (310.79 to 602.39)	124 (87.7 to 170)	510.6 (360.11 to 695.9)
Yunnan	Depressive disorders	195 (138 to 268)	499.62 (353.69 to 688.85)	285 (199 to 389)	580.1 (405.03 to 790.8)
Zhejiang	Depressive disorders	255 (179 to 350)	578.25 (405.24 to 792.74)	398 (280 to 545)	650.6 (457.81 to 890.27)
China	Bipolar disorder	744 (469 to 1100)	62.17 (39.22 to 91.9)	1040 (654 to 1550)	73.51 (46.31 to 109.49)
Taiwan	Bipolar disorder	14.1 (8.74 to 21.3)	68.93 (42.84 to 104.42)	19.2 (12.1 to 28.7)	81.31 (51.16 to 121.57)
Hong Kong Special Administrative Region of China	Bipolar disorder	4.12 (2.56 to 6.21)	71.46 (44.46 to 107.66)	6.02 (3.78 to 9.04)	80.17 (50.39 to 120.31)
Macau Special Administrative Region of China	Bipolar disorder	0.232 (0.145 to 0.35)	65.35 (40.89 to 98.51)	0.535 (0.338 to 0.792)	78.18 (49.43 to 115.8)
Anhui	Bipolar disorder	34.6 (22.1 to 52.3)	58.42 (37.27 to 88.2)	42.8 (27 to 63.8)	70.05 (44.22 to 104.34)
Beijing	Bipolar disorder	7.76 (4.84 to 11.7)	69.15 (43.19 to 104.25)	18.9 (11.9 to 28.3)	78.24 (49.34 to 117.45)
Chongqing	Bipolar disorder	9.91 (6.21 to 14.6)	62.78 (39.38 to 92.27)	18.8 (11.7 to 28.3)	70.19 (43.62 to 105.78)
Fujian	Bipolar disorder	18 (11.2 to 26.9)	56.78 (35.5 to 85.02)	27.8 (17.3 to 41.8)	70.42 (43.96 to 105.97)
Gansu	Bipolar disorder	11.6 (7.14 to 17.4)	49.07 (30.17 to 73.37)	15.8 (9.81 to 23.7)	59.62 (36.93 to 89.2)
Guangdong	Bipolar disorder	38.5 (24 to 57.7)	58.48 (36.42 to 87.63)	83.4 (52.3 to 126)	71.42 (44.76 to 107.95)
Guangxi	Bipolar disorder	24 (15 to 35.9)	55.24 (34.42 to 82.65)	31.6 (19.7 to 47.5)	65.44 (40.67 to 98.2)
Guizhou	Bipolar disorder	18.3 (11.5 to 27.6)	55.05 (34.69 to 83.02)	22.5 (14.2 to 33.7)	66.38 (42.07 to 99.59)
Hainan	Bipolar disorder	3.81 (2.37 to 5.67)	54.68 (34.06 to 81.39)	6.54 (4.1 to 9.75)	68.18 (42.73 to 101.72)
Hebei	Bipolar disorder	78 (48.2 to 116)	122.48 (75.77 to 182.54)	105 (66.2 to 157)	137.95 (86.61 to 205.41)
Heilongjiang	Bipolar disorder	22.1 (13.7 to 32.9)	60.67 (37.51 to 90.38)	29.9 (18.9 to 44.6)	76.09 (48.03 to 113.75)
Henan	Bipolar disorder	53 (33.3 to 80.6)	60.04 (37.71 to 91.34)	65.9 (41.2 to 98.8)	68.43 (42.77 to 102.59)
Hubei	Bipolar disorder	33.2 (20.6 to 50.3)	59.84 (37.12 to 90.49)	40.6 (25.6 to 60.1)	73.38 (46.33 to 108.73)
Hunan	Bipolar disorder	36.8 (23.1 to 55.3)	59.14 (37.09 to 88.93)	46.1 (28.8 to 69.5)	68.64 (42.86 to 103.55)
Inner Mongolia	Bipolar disorder	13.1 (8.07 to 19.7)	57.5 (35.49 to 86.65)	18.9 (11.7 to 28.4)	72.9 (45.23 to 109.86)
Jiangsu	Bipolar disorder	45.3 (28.2 to 67.3)	65.88 (41 to 97.84)	61.8 (38.8 to 91.9)	75.63 (47.55 to 112.52)
Jiangxi	Bipolar disorder	21.8 (13.4 to 33)	55.16 (33.92 to 83.38)	31.2 (19.4 to 47)	65.74 (40.83 to 98.84)

Jilin	Bipolar disorder	15.6<br>(9.72 to 23.2)	60.01<br>(37.44 to 89.54)	21<br>(12.9 to 31.4)	75.15<br>(46.37 to 112.55)
Liaoning	Bipolar disorder	26.2<br>(16.4 to 39.4)	64.19<br>(40.2 to 96.54)	34.6<br>(21.6 to 51.9)	77.41<br>(48.47 to 116.19)
Ningxia	Bipolar disorder	2.66<br>(1.65 to 4.03)	53.84<br>(33.46 to 81.51)	4.74<br>(2.94 to 7.03)	67.48<br>(41.89 to 100.17)
Qinghai	Bipolar disorder	2.25<br>(1.36 to 3.36)	47.56<br>(28.79 to 70.94)	3.66<br>(2.27 to 5.64)	58.3<br>(36.19 to 89.69)
Shaanxi	Bipolar disorder	20.9<br>(12.9 to 31.2)	60.81<br>(37.62 to 90.78)	29.5<br>(18.2 to 43.9)	74.6<br>(46.15 to 111.02)
Shandong	Bipolar disorder	43.4<br>(27.2 to 65.3)	50.74<br>(31.81 to 76.32)	59.3<br>(36.7 to 89.6)	59.66<br>(36.97 to 90.16)
Shanghai	Bipolar disorder	9.89<br>(6.18 to 14.7)	70.77<br>(44.23 to 104.98)	22.3<br>(14 to 33.4)	79.67<br>(50.03 to 119.57)
Shanxi	Bipolar disorder	17.7<br>(11.1 to 26.3)	58.52<br>(36.6 to 86.81)	27.5<br>(17 to 40.7)	71.16<br>(44.02 to 105.27)
Sichuan	Bipolar disorder	71.9<br>(45.4 to 108)	64.38<br>(40.72 to 96.47)	61.6<br>(38.5 to 91.8)	73.84<br>(46.09 to 109.94)
Tianjin	Bipolar disorder	5.9<br>(3.68 to 8.87)	64.19<br>(39.96 to 96.42)	11.6<br>(7.22 to 17.3)	75.95<br>(47.33 to 113.19)
Tibet	Bipolar disorder	1.15<br>(0.716 to 1.72)	49.44<br>(30.71 to 73.92)	2.01<br>(1.27 to 3)	58.01<br>(36.61 to 86.76)
Xinjiang	Bipolar disorder	8.48<br>(5.3 to 12.7)	53.76<br>(33.63 to 80.27)	15.8<br>(9.92 to 23.8)	64.94<br>(40.71 to 97.57)
Yunnan	Bipolar disorder	19.4<br>(12 to 29.4)	49.84<br>(30.71 to 75.52)	30<br>(18.4 to 45.2)	60.98<br>(37.45 to 91.91)
Zhejiang	Bipolar disorder	24.6<br>(15.5 to 37.3)	55.73<br>(35.1 to 84.52)	40.4<br>(25.5 to 60.5)	66.1<br>(41.74 to 98.93)
China	Anxiety disorders	3420<br>(2420 to 4640)	286.02<br>(202.12 to 387.75)	4280<br>(3020 to 5680)	303.15<br>(214.11 to 402.28)
Taiwan	Anxiety disorders	63.7<br>(45.2 to 85.9)	312.29<br>(221.39 to 420.8)	82<br>(58.2 to 109)	347.8<br>(246.67 to 462.41)
Hong Kong Special Administrative Region of China	Anxiety disorders	16<br>(11.2 to 21.6)	277.84<br>(194.81 to 374.89)	21.4<br>(14.9 to 28.9)	285.39<br>(198.3 to 384.32)
Macau Special Administrative Region of China	Anxiety disorders	1.02<br>(0.717 to 1.39)	286.94<br>(202.17 to 390.92)	2.1<br>(1.49 to 2.8)	307.74<br>(217.33 to 410.15)
Anhui	Anxiety disorders	166<br>(117 to 224)	280.06<br>(197.45 to 378.88)	184<br>(129 to 248)	301.61<br>(211.7 to 405.67)
Beijing	Anxiety disorders	30.9<br>(21.8 to 41.7)	275.44<br>(194.33 to 372.03)	68.5<br>(48.4 to 92.4)	284.35<br>(200.75 to 383.27)
Chongqing	Anxiety disorders	45.2<br>(32 to 61.1)	286.43<br>(202.94 to 387.24)	81.9<br>(57.6 to 109)	306.33<br>(215.6 to 407.29)
Fujian	Anxiety disorders	86.7<br>(61.2 to 118)	274.1<br>(193.44 to 373.01)	116<br>(81.5 to 155)	294.63<br>(206.77 to 392.26)
Gansu	Anxiety disorders	73.3<br>(51.3 to 98.7)	310.07<br>(216.99 to 417.3)	91.6<br>(64.8 to 123)	344.71<br>(243.75 to 463.54)
Guangdong	Anxiety disorders	167<br>(117 to 225)	253.71<br>(177.42 to 342.56)	312<br>(219 to 419)	267.38<br>(187.64 to 358.69)
Guangxi	Anxiety disorders	120<br>(84.4 to 163)	276.39<br>(194.17 to 375.29)	141<br>(99.1 to 188)	292.55<br>(205.17 to 388.62)
Guizhou	Anxiety disorders	93.3<br>(65.9 to 127)	281.01<br>(198.61 to 381.36)	103<br>(72.5 to 137)	303.09<br>(214.35 to 404.15)
Hainan	Anxiety disorders	18.6<br>(13.1 to 25.4)	267.56<br>(188.28 to 365.05)	27.7<br>(19.4 to 36.8)	289.09<br>(202.52 to 383.51)
Hebei	Anxiety disorders	172<br>(121 to 230)	269.91<br>(189.47 to 360.82)	216<br>(151 to 288)	282.04<br>(198.01 to 376.82)
Heilongjiang	Anxiety disorders	104<br>(73.6 to 142)	285.91<br>(201.92 to 389.44)	125<br>(87.5 to 166)	317.84<br>(222.82 to 423.73)
Henan	Anxiety disorders	250<br>(175 to 340)	283.05<br>(198.77 to 384.94)	286<br>(201 to 380)	297.11<br>(208.32 to 395.1)
Hubei	Anxiety disorders	156<br>(110 to 213)	281.14<br>(197.31 to 382.92)	170<br>(119 to 227)	307.41<br>(215.7 to 411.21)
Hunan	Anxiety disorders	205<br>(143 to 279)	328.99<br>(230.52 to 449.43)	240<br>(168 to 320)	356.79<br>(250.59 to 476.72)
Inner Mongolia	Anxiety disorders	62.8<br>(44 to 85.9)	276.31<br>(193.65 to 377.75)	79.1<br>(55.6 to 106)	305.43<br>(214.75 to 407.64)

Jiangsu	Anxiety disorders	203<br>(143 to 275)	294.4<br>(208.24 to 399.14)	252<br>(177 to 337)	309.19<br>(217.28 to 412.24)
Jiangxi	Anxiety disorders	109<br>(77.2 to 150)	276.98<br>(195.29 to 378.61)	140<br>(97.9 to 186)	293.65<br>(205.94 to 391.95)
Jilin	Anxiety disorders	73.6<br>(51.7 to 100)	283.45<br>(199.26 to 386.99)	87.9<br>(61.8 to 117)	315.12<br>(221.42 to 417.83)
Liaoning	Anxiety disorders	112<br>(78.3 to 150)	275.25<br>(191.91 to 368.86)	134<br>(93.8 to 178)	299.59<br>(210.09 to 398)
Ningxia	Anxiety disorders	13.3<br>(9.36 to 18.1)	268.82<br>(189.42 to 366.26)	20.6<br>(14.4 to 27.4)	292.88<br>(204.68 to 390.56)
Qinghai	Anxiety disorders	11.9<br>(8.34 to 16.3)	251.38<br>(176.01 to 343.97)	16.9<br>(11.8 to 22.9)	268.84<br>(187.54 to 364.45)
Shaanxi	Anxiety disorders	95.4<br>(66.6 to 130)	277.25<br>(193.45 to 376.71)	119<br>(84.4 to 158)	300.56<br>(213.71 to 400.59)
Shandong	Anxiety disorders	282<br>(199 to 378)	329.47<br>(232.88 to 442.16)	349<br>(245 to 465)	351.37<br>(246.07 to 467.88)
Shanghai	Anxiety disorders	38.9<br>(27.5 to 52.5)	278.51<br>(196.86 to 375.86)	79.9<br>(56.3 to 107)	285.81<br>(201.63 to 382.65)
Shanxi	Anxiety disorders	84<br>(59.7 to 113)	277.37<br>(197.28 to 374.64)	116<br>(81.7 to 155)	300.52<br>(211.55 to 400.51)
Sichuan	Anxiety disorders	316<br>(223 to 426)	283.28<br>(200.02 to 382.11)	233<br>(167 to 312)	278.93<br>(199.52 to 373.78)
Tianjin	Anxiety disorders	26.6<br>(18.7 to 36.2)	289.51<br>(203.75 to 393.13)	45.8<br>(32.3 to 61)	299.92<br>(211.78 to 399.45)
Tibet	Anxiety disorders	6.2<br>(4.35 to 8.42)	266.24<br>(186.59 to 361.3)	9.52<br>(6.65 to 12.7)	275.33<br>(192.53 to 368.22)
Xinjiang	Anxiety disorders	42.9<br>(30.4 to 58.7)	272.2<br>(192.59 to 371.84)	70.3<br>(49.6 to 93.6)	288.35<br>(203.58 to 384.24)
Yunnan	Anxiety disorders	108<br>(75.1 to 148)	277.08<br>(192.79 to 378.78)	144<br>(102 to 193)	293.86<br>(206.74 to 393.12)
Zhejiang	Anxiety disorders	133<br>(92.7 to 179)	300.56<br>(210.19 to 405.63)	199<br>(140 to 269)	324.83<br>(228.87 to 438.94)
China	Eating disorders	245<br>(156 to 360)	20.47<br>(13 to 30.1)	454<br>(286 to 668)	32.12<br>(20.23 to 47.29)
Taiwan	Eating disorders	8.05<br>(5.11 to 11.8)	39.48<br>(25.03 to 57.99)	10.9<br>(6.85 to 16)	46.27<br>(29.03 to 67.76)
Hong Kong Special Administrative Region of China	Eating disorders	2.74<br>(1.69 to 4.09)	47.6<br>(29.26 to 70.93)	3.78<br>(2.38 to 5.6)	50.39<br>(31.65 to 74.58)
Macau Special Administrative Region of China	Eating disorders	0.194<br>(0.122 to 0.288)	54.59<br>(34.32 to 81.16)	0.492<br>(0.308 to 0.732)	72.02<br>(45.02 to 107.05)
Anhui	Eating disorders	10.9<br>(6.77 to 16)	18.36<br>(11.43 to 27.07)	15.9<br>(10 to 23.3)	26.07<br>(16.37 to 38.18)
Beijing	Eating disorders	3.25<br>(2.03 to 4.79)	28.97<br>(18.06 to 42.73)	11.8<br>(7.38 to 17.7)	49.01<br>(30.61 to 73.5)
Chongqing	Eating disorders	3.12<br>(1.93 to 4.69)	19.8<br>(12.2 to 29.74)	7.2<br>(4.52 to 10.7)	26.95<br>(16.93 to 40.2)
Fujian	Eating disorders	5.37<br>(3.08 to 8.85)	16.96<br>(9.72 to 27.97)	10.6<br>(5.91 to 17.7)	26.94<br>(15 to 44.95)
Gansu	Eating disorders	4.51<br>(2.8 to 6.62)	19.05<br>(11.82 to 28.01)	6.81<br>(4.23 to 10.1)	25.63<br>(15.9 to 38.07)
Guangdong	Eating disorders	13.8<br>(8.66 to 20.2)	20.92<br>(13.15 to 30.73)	49.8<br>(30.8 to 74.7)	42.61<br>(26.36 to 63.9)
Guangxi	Eating disorders	7.55<br>(4.68 to 11.4)	17.36<br>(10.77 to 26.1)	13.5<br>(8.48 to 20)	27.88<br>(17.56 to 41.4)
Guizhou	Eating disorders	5.42<br>(3.35 to 8.01)	16.33<br>(10.09 to 24.15)	8.01<br>(5.02 to 12)	23.69<br>(14.84 to 35.33)
Hainan	Eating disorders	1.31<br>(0.81 to 1.97)	18.85<br>(11.63 to 28.32)	2.95<br>(1.85 to 4.36)	30.75<br>(19.26 to 45.47)
Hebei	Eating disorders	9.69<br>(5.58 to 15.8)	15.22<br>(8.77 to 24.82)	16.7<br>(9.23 to 27.9)	21.87<br>(12.07 to 36.5)
Heilongjiang	Eating disorders	9.02<br>(5.76 to 13.3)	24.76<br>(15.79 to 36.36)	11.6<br>(7.34 to 17.3)	29.54<br>(18.69 to 43.97)
Henan	Eating disorders	16.3<br>(9.95 to 24.5)	18.5<br>(11.27 to 27.75)	28.2<br>(17.7 to 41.9)	29.29<br>(18.38 to 43.5)
Hubei	Eating disorders	11.4<br>(7.18 to 16.9)	20.59<br>(12.93 to 30.42)	16.1<br>(10.3 to 23.3)	29.06<br>(18.62 to 42.05)

Hunan	Eating disorders	13.8 (8.6 to 20.4)	22.12 (13.83 to 32.75)	20.8 (13.1 to 30.3)	31.01 (19.51 to 45.17)
Inner Mongolia	Eating disorders	4.79 (2.94 to 7.12)	21.04 (12.91 to 31.3)	8.54 (5.4 to 12.7)	32.97 (20.87 to 49.05)
Jiangsu	Eating disorders	15.9 (10.1 to 23.6)	23.09 (14.7 to 34.3)	31.5 (19.9 to 47)	38.56 (24.34 to 57.52)
Jiangxi	Eating disorders	7.42 (4.67 to 11)	18.78 (11.82 to 27.83)	13.4 (8.51 to 19.9)	28.16 (17.91 to 41.94)
Jilin	Eating disorders	5.9 (3.68 to 8.62)	22.74 (14.2 to 33.22)	8.33 (5.18 to 12.2)	29.86 (18.59 to 43.87)
Liaoning	Eating disorders	10.8 (6.73 to 15.9)	26.55 (16.49 to 38.92)	14.3 (9.15 to 21)	31.92 (20.49 to 47.13)
Ningxia	Eating disorders	1.01 (0.62 to 1.53)	20.49 (12.55 to 30.92)	2.19 (1.38 to 3.22)	31.14 (19.61 to 45.81)
Qinghai	Eating disorders	0.98 (0.604 to 1.45)	20.7 (12.75 to 30.66)	1.91 (1.21 to 2.8)	30.34 (19.22 to 44.64)
Shaanxi	Eating disorders	6.55 (4.08 to 9.72)	19.03 (11.87 to 28.24)	11.9 (7.35 to 17.6)	30.04 (18.59 to 44.64)
Shandong	Eating disorders	18.5 (11.6 to 27.6)	21.64 (13.53 to 32.22)	34.9 (22 to 51.6)	35.11 (22.15 to 51.89)
Shanghai	Eating disorders	4.21 (2.65 to 6.32)	30.12 (19 to 45.25)	12.4 (7.73 to 18.5)	44.39 (27.67 to 66.16)
Shanxi	Eating disorders	6.13 (3.82 to 9.08)	20.24 (12.62 to 29.99)	11.9 (7.44 to 17.6)	30.91 (19.26 to 45.57)
Sichuan	Eating disorders	21.5 (13.6 to 32.1)	19.26 (12.15 to 28.78)	22.5 (14.2 to 33.9)	26.95 (16.99 to 40.59)
Tianjin	Eating disorders	2.59 (1.61 to 3.84)	28.15 (17.5 to 41.73)	6.76 (4.22 to 9.85)	44.29 (27.66 to 64.57)
Tibet	Eating disorders	0.415 (0.263 to 0.611)	17.81 (11.27 to 26.23)	1.03 (0.651 to 1.51)	29.71 (18.84 to 43.7)
Xinjiang	Eating disorders	3.16 (1.95 to 4.65)	20.02 (12.34 to 29.49)	7.96 (4.93 to 11.7)	32.69 (20.22 to 47.95)
Yunnan	Eating disorders	6.9 (4.24 to 10.5)	17.69 (10.88 to 26.84)	14.2 (9.14 to 20.9)	28.8 (18.58 to 42.51)
Zhejiang	Eating disorders	9.87 (6.13 to 14.3)	22.39 (13.9 to 32.51)	25.8 (16.2 to 38.5)	42.14 (26.5 to 62.94)
China	Autism spectrum disorders	679 (462 to 940)	56.72 (38.56 to 78.51)	775 (529 to 1070)	54.86 (37.42 to 75.62)
Taiwan	Autism spectrum disorders	9.18 (6.18 to 12.8)	45 (30.3 to 62.58)	9.72 (6.56 to 13.3)	41.22 (27.81 to 56.58)
Hong Kong Special Administrative Region of China	Autism spectrum disorders	3.14 (2.12 to 4.38)	54.49 (36.72 to 75.92)	3.66 (2.51 to 5.05)	48.69 (33.4 to 67.25)
Macau Special Administrative Region of China	Autism spectrum disorders	0.189 (0.128 to 0.261)	53.34 (36.05 to 73.64)	0.348 (0.236 to 0.482)	50.85 (34.5 to 70.45)
Anhui	Autism spectrum disorders	31.4 (21.2 to 43.5)	53.01 (35.75 to 73.42)	29.9 (20.4 to 41.4)	48.84 (33.37 to 67.76)
Beijing	Autism spectrum disorders	11.8 (7.93 to 16.4)	105.14 (70.67 to 146.18)	24.2 (16.3 to 33.2)	100.29 (67.47 to 137.95)
Chongqing	Autism spectrum disorders	8.27 (5.6 to 11.5)	52.39 (35.46 to 73.09)	12.9 (8.73 to 17.9)	48.28 (32.67 to 67.14)
Fujian	Autism spectrum disorders	11.8 (7.52 to 17)	37.34 (23.77 to 53.58)	13.8 (8.79 to 19.8)	35.03 (22.29 to 50.16)
Gansu	Autism spectrum disorders	22.3 (15.3 to 30.8)	94.34 (64.55 to 130.02)	23.4 (16 to 32.5)	88.1 (60.03 to 122.21)
Guangdong	Autism spectrum disorders	55.2 (37.3 to 76.2)	83.93 (56.74 to 115.81)	95.9 (65.5 to 132)	82.06 (56.04 to 113.19)
Guangxi	Autism spectrum disorders	23.2 (15.8 to 32.3)	53.35 (36.31 to 74.19)	24.1 (16.4 to 33.7)	49.95 (33.88 to 69.71)
Guizhou	Autism spectrum disorders	13.1 (8.79 to 18.5)	39.46 (26.48 to 55.83)	12.3 (8.17 to 17.3)	36.46 (24.15 to 51.07)
Hainan	Autism spectrum disorders	3.77 (2.54 to 5.27)	54.06 (36.4 to 75.7)	4.94 (3.33 to 6.87)	51.57 (34.69 to 71.63)
Hebei	Autism spectrum disorders	33.3 (22.4 to 46.3)	52.38 (35.24 to 72.81)	37.9 (25.7 to 52.8)	49.63 (33.57 to 69.11)
Heilongjiang	Autism spectrum disorders	18.6 (12.5 to 25.9)	51.02 (34.31 to 71.01)	18.2 (12.3 to 25.3)	46.3 (31.33 to 64.45)

Henan	Autism spectrum disorders	46.7 (31.9 to 64.9)	52.94 (36.19 to 73.49)	48.2 (32.6 to 66.9)	50.06 (33.89 to 69.49)
Hubei	Autism spectrum disorders	29.3 (19.8 to 40.3)	52.82 (35.58 to 72.57)	27 (18.2 to 36.9)	48.75 (32.97 to 66.76)
Hunan	Autism spectrum disorders	48.5 (33 to 67.5)	78.05 (53.11 to 108.47)	49.2 (33.5 to 68.4)	73.29 (49.92 to 101.83)
Inner Mongolia	Autism spectrum disorders	12.2 (8.29 to 16.8)	53.53 (36.47 to 73.94)	12.8 (8.63 to 17.8)	49.48 (33.34 to 68.73)
Jiangsu	Autism spectrum disorders	41.8 (28.3 to 58)	60.7 (41.16 to 84.29)	46.7 (31.5 to 64.4)	57.17 (38.63 to 78.84)
Jiangxi	Autism spectrum disorders	21 (14.4 to 29.4)	53.11 (36.38 to 74.27)	23.7 (16.1 to 32.9)	49.96 (33.94 to 69.24)
Jilin	Autism spectrum disorders	16.1 (11 to 22.5)	62.1 (42.54 to 86.88)	15.8 (10.8 to 21.9)	56.6 (38.67 to 78.35)
Liaoning	Autism spectrum disorders	21.4 (14.5 to 29.6)	52.43 (35.5 to 72.66)	21.6 (14.7 to 30)	48.32 (33.03 to 67.1)
Ningxia	Autism spectrum disorders	2.65 (1.78 to 3.7)	53.7 (36.1 to 74.83)	3.54 (2.4 to 4.95)	50.46 (34.21 to 70.51)
Qinghai	Autism spectrum disorders	2.53 (1.71 to 3.51)	53.51 (36.2 to 74.12)	3.16 (2.15 to 4.34)	50.37 (34.26 to 69.15)
Shaanxi	Autism spectrum disorders	18.4 (12.6 to 25.4)	53.56 (36.47 to 73.84)	19.9 (13.5 to 27.5)	50.28 (34.12 to 69.56)
Shandong	Autism spectrum disorders	68.7 (45.6 to 95.9)	80.26 (53.28 to 112.02)	75.4 (51.2 to 104)	75.92 (51.47 to 105)
Shanghai	Autism spectrum disorders	6.55 (4.38 to 9.09)	46.89 (31.37 to 65.07)	12.7 (8.62 to 17.7)	45.6 (30.85 to 63.27)
Shanxi	Autism spectrum disorders	16.2 (11 to 22.6)	53.53 (36.43 to 74.66)	19.3 (13.1 to 27)	50.02 (33.96 to 69.95)
Sichuan	Autism spectrum disorders	40.3 (25.4 to 59.2)	36.14 (22.73 to 53.04)	27.7 (17.7 to 40.3)	33.22 (21.2 to 48.22)
Tianjin	Autism spectrum disorders	4.75 (3.17 to 6.59)	51.7 (34.43 to 71.7)	7.85 (5.24 to 10.9)	51.44 (34.31 to 71.6)
Tibet	Autism spectrum disorders	1.21 (0.82 to 1.67)	52.05 (35.18 to 71.81)	1.76 (1.18 to 2.45)	51.06 (34.26 to 70.89)
Xinjiang	Autism spectrum disorders	8.38 (5.65 to 11.6)	53.12 (35.83 to 73.4)	12.2 (8.17 to 16.8)	50.15 (33.53 to 69.1)
Yunnan	Autism spectrum disorders	20.4 (13.9 to 28.5)	52.43 (35.54 to 73.08)	24.4 (16.4 to 33.7)	49.53 (33.42 to 68.6)
Zhejiang	Autism spectrum disorders	15.5 (10.1 to 22.3)	35.19 (22.87 to 50.51)	20.4 (13.1 to 29.5)	33.32 (21.35 to 48.13)
China	Attention-deficit/hyperactivity disorder	216 (130 to 344)	18.04 (10.84 to 28.71)	207 (128 to 328)	14.68 (9.06 to 23.19)
Taiwan	Attention-deficit/hyperactivity disorder	3.66 (2.21 to 5.78)	17.92 (10.81 to 28.33)	3.12 (1.89 to 4.91)	13.25 (8 to 20.84)
Hong Kong Special Administrative Region of China	Attention-deficit/hyperactivity disorder	1.1 (0.655 to 1.75)	19 (11.36 to 30.37)	0.998 (0.612 to 1.6)	13.29 (8.15 to 21.31)
Macau Special Administrative Region of China	Attention-deficit/hyperactivity disorder	0.0556 (0.0332 to 0.0883)	15.67 (9.35 to 24.89)	0.0925 (0.0569 to 0.146)	13.52 (8.32 to 21.32)
Anhui	Attention-deficit/hyperactivity disorder	10.3 (6.08 to 16.4)	17.38 (10.26 to 27.76)	8.31 (5.01 to 13.3)	13.6 (8.19 to 21.68)
Beijing	Attention-deficit/hyperactivity disorder	2.39 (1.45 to 3.84)	21.27 (12.93 to 34.21)	4.08 (2.46 to 6.43)	16.95 (10.2 to 26.69)
Chongqing	Attention-deficit/hyperactivity disorder	2.54 (1.49 to 4.01)	16.07 (9.47 to 25.42)	3.56 (2.16 to 5.65)	13.32 (8.07 to 21.16)
Fujian	Attention-deficit/hyperactivity disorder	6.5 (3.83 to 10.3)	20.56 (12.1 to 32.56)	6.32 (3.79 to 10)	16.05 (9.62 to 25.47)
Gansu	Attention-deficit/hyperactivity disorder	4.11 (2.43 to 6.58)	17.39 (10.28 to 27.8)	3.82 (2.33 to 6.09)	14.39 (8.77 to 22.91)
Guangdong	Attention-deficit/hyperactivity disorder	17.7 (10.6 to 28.1)	26.89 (16.15 to 42.63)	25.9 (15.7 to 40.9)	22.18 (13.46 to 35.01)

Guangxi	Attention-deficit/hyperactivity disorder	9.46 (5.74 to 15)	21.74 (13.21 to 34.52)	8.32 (5.02 to 13.2)	17.21 (10.39 to 27.35)
Guizhou	Attention-deficit/hyperactivity disorder	6.18 (3.68 to 9.81)	18.62 (11.09 to 29.54)	5.27 (3.22 to 8.41)	15.57 (9.5 to 24.85)
Hainan	Attention-deficit/hyperactivity disorder	1.23 (0.737 to 1.95)	17.66 (10.59 to 28.05)	1.46 (0.881 to 2.31)	15.22 (9.19 to 24.05)
Hebei	Attention-deficit/hyperactivity disorder	10.5 (6.32 to 16.6)	16.48 (9.93 to 26.04)	10.9 (6.61 to 17.2)	14.21 (8.65 to 22.46)
Heilongjiang	Attention-deficit/hyperactivity disorder	6.52 (3.87 to 10.4)	17.9 (10.63 to 28.63)	5.02 (3.09 to 7.84)	12.8 (7.86 to 19.98)
Henan	Attention-deficit/hyperactivity disorder	15.2 (9.19 to 24.3)	17.27 (10.41 to 27.49)	14.6 (8.87 to 23.4)	15.21 (9.21 to 24.28)
Hubei	Attention-deficit/hyperactivity disorder	6.66 (3.94 to 10.7)	11.98 (7.09 to 19.31)	4.79 (2.76 to 7.79)	8.66 (4.99 to 14.08)
Hunan	Attention-deficit/hyperactivity disorder	12.7 (7.5 to 20.4)	20.41 (12.06 to 32.87)	11 (6.66 to 17.4)	16.31 (9.91 to 25.94)
Inner Mongolia	Attention-deficit/hyperactivity disorder	4.03 (2.4 to 6.36)	17.7 (10.56 to 27.96)	3.49 (2.13 to 5.49)	13.49 (8.22 to 21.22)
Jiangsu	Attention-deficit/hyperactivity disorder	10.6 (6.2 to 16.8)	15.37 (9.01 to 24.46)	9.45 (5.67 to 15.3)	11.57 (6.94 to 18.71)
Jiangxi	Attention-deficit/hyperactivity disorder	7.16 (4.22 to 11.4)	18.12 (10.67 to 28.82)	7.32 (4.42 to 11.6)	15.4 (9.3 to 24.45)
Jilin	Attention-deficit/hyperactivity disorder	4.48 (2.63 to 7.06)	17.25 (10.15 to 27.2)	3.52 (2.14 to 5.54)	12.63 (7.66 to 19.85)
Liaoning	Attention-deficit/hyperactivity disorder	3.61 (2.01 to 6.18)	8.84 (4.93 to 15.14)	2.72 (1.49 to 4.58)	6.08 (3.33 to 10.25)
Ningxia	Attention-deficit/hyperactivity disorder	0.904 (0.533 to 1.44)	18.31 (10.8 to 29.21)	1.09 (0.669 to 1.71)	15.47 (9.54 to 24.31)
Qinghai	Attention-deficit/hyperactivity disorder	0.877 (0.524 to 1.39)	18.52 (11.07 to 29.32)	0.979 (0.592 to 1.56)	15.59 (9.42 to 24.81)
Shaanxi	Attention-deficit/hyperactivity disorder	5.84 (3.49 to 9.26)	16.98 (10.13 to 26.92)	5.46 (3.3 to 8.64)	13.83 (8.36 to 21.87)
Shandong	Attention-deficit/hyperactivity disorder	14.5 (8.41 to 23)	16.95 (9.83 to 26.89)	13.7 (8.44 to 21.7)	13.83 (8.49 to 21.88)
Shanghai	Attention-deficit/hyperactivity disorder	2.55 (1.52 to 4.04)	18.27 (10.9 to 28.92)	4.25 (2.54 to 6.75)	15.2 (9.11 to 24.17)
Shanxi	Attention-deficit/hyperactivity disorder	5.16 (3.03 to 8.13)	17.05 (10.02 to 26.86)	5.56 (3.36 to 8.78)	14.4 (8.7 to 22.72)
Sichuan	Attention-deficit/hyperactivity disorder	24.3 (14.6 to 39.5)	21.79 (13.12 to 35.41)	13.4 (7.97 to 21.1)	16.02 (9.55 to 25.25)
Tianjin	Attention-deficit/hyperactivity disorder	1.23 (0.719 to 1.96)	13.39 (7.82 to 21.28)	1.72 (1.03 to 2.77)	11.28 (6.72 to 18.14)
Tibet	Attention-deficit/hyperactivity disorder	0.407 (0.246 to 0.642)	17.46 (10.54 to 27.57)	0.583 (0.356 to 0.922)	16.87 (10.3 to 26.69)
Xinjiang	Attention-deficit/hyperactivity disorder	2.86 (1.69 to 4.49)	18.13 (10.69 to 28.46)	3.7 (2.26 to 5.81)	15.17 (9.27 to 23.84)

Yunnan	Attention-deficit/hyperactivity disorder	7.01 (4.11 to 11.2)	17.99 (10.54 to 28.64)	7.61 (4.6 to 12.1)	15.49 (9.36 to 24.64)
Zhejiang	Attention-deficit/hyperactivity disorder	7.21 (4.25 to 11.5)	16.34 (9.64 to 26.16)	8.29 (5.11 to 13)	13.55 (8.35 to 21.3)
China	Conduct disorder	853 (507 to 1370)	71.25 (42.32 to 114.6)	615 (364 to 995)	43.51 (25.78 to 70.47)
Taiwan	Conduct disorder	17.3 (10.3 to 27.9)	84.56 (50.28 to 136.52)	10.9 (6.55 to 17.6)	46.35 (27.78 to 74.79)
Hong Kong Special Administrative Region of China	Conduct disorder	3.24 (1.9 to 5.38)	56.19 (32.91 to 93.3)	2.28 (1.33 to 3.76)	30.4 (17.66 to 50.13)
Macau Special Administrative Region of China	Conduct disorder	0.189 (0.111 to 0.306)	53.19 (31.22 to 86.1)	0.224 (0.132 to 0.364)	32.79 (19.32 to 53.23)
Anhui	Conduct disorder	45.8 (27.1 to 72.7)	77.27 (45.81 to 122.77)	26.6 (15.7 to 43.1)	43.43 (25.61 to 70.49)
Beijing	Conduct disorder	5.7 (3.38 to 9.23)	50.86 (30.17 to 82.28)	6.05 (3.62 to 9.88)	25.12 (15.04 to 40.99)
Chongqing	Conduct disorder	9.69 (5.69 to 15.7)	61.42 (36.08 to 99.59)	12.3 (7.31 to 20.2)	46.13 (27.36 to 75.62)
Fujian	Conduct disorder	25.1 (15 to 41.5)	79.26 (47.29 to 131.14)	15.6 (9.22 to 25.3)	39.52 (23.4 to 64.27)
Gansu	Conduct disorder	17.5 (10.5 to 28)	74.07 (44.21 to 118.51)	12.5 (7.37 to 20.4)	47.02 (27.72 to 76.67)
Guangdong	Conduct disorder	48.2 (28.7 to 77.8)	73.16 (43.58 to 118.28)	52.6 (31.4 to 84.6)	45.03 (26.88 to 72.41)
Guangxi	Conduct disorder	36.9 (21.7 to 59.2)	84.75 (49.86 to 136.07)	28 (16.5 to 45.7)	57.86 (34.21 to 94.57)
Guizhou	Conduct disorder	30.1 (17.8 to 48.9)	90.74 (53.69 to 147.44)	21.6 (12.5 to 35.4)	63.88 (37.05 to 104.57)
Hainan	Conduct disorder	5.54 (3.28 to 9.05)	79.55 (47.05 to 129.85)	4.82 (2.85 to 7.79)	50.26 (29.71 to 81.29)
Hebei	Conduct disorder	42 (25.2 to 67.2)	66 (39.55 to 105.57)	35.1 (20.4 to 57.4)	45.88 (26.73 to 75.05)
Heilongjiang	Conduct disorder	27.7 (16 to 44.7)	76.06 (43.96 to 122.51)	12.8 (7.47 to 20.7)	32.56 (19.04 to 52.65)
Henan	Conduct disorder	66.4 (38.8 to 107)	75.22 (43.96 to 121.58)	55.6 (32.6 to 91)	57.69 (33.81 to 94.47)
Hubei	Conduct disorder	39.5 (23.4 to 62.7)	71.11 (42.09 to 112.86)	20.1 (11.9 to 32.5)	36.37 (21.57 to 58.81)
Hunan	Conduct disorder	35.4 (21.5 to 56.5)	56.86 (34.52 to 90.92)	32.1 (18.7 to 52.6)	47.81 (27.88 to 78.37)
Inner Mongolia	Conduct disorder	17.3 (10.3 to 28.1)	76.05 (45.47 to 123.47)	9.42 (5.49 to 15.2)	36.37 (21.19 to 58.53)
Jiangsu	Conduct disorder	43.2 (26.2 to 69.8)	62.85 (38.08 to 101.38)	28.4 (17.1 to 46.6)	34.82 (20.94 to 57.09)
Jiangxi	Conduct disorder	34.2 (20 to 55.5)	86.59 (50.67 to 140.47)	27 (15.5 to 43.5)	56.88 (32.64 to 91.5)
Jilin	Conduct disorder	18.4 (10.9 to 30.2)	70.88 (41.98 to 116.29)	8.99 (5.25 to 14.8)	32.25 (18.84 to 52.91)
Liaoning	Conduct disorder	23.4 (13.2 to 39.4)	57.34 (32.45 to 96.47)	12.1 (6.9 to 20.7)	27.12 (15.46 to 46.4)
Ningxia	Conduct disorder	4.29 (2.51 to 6.97)	86.93 (50.87 to 141.09)	3.81 (2.22 to 6.24)	54.32 (31.67 to 88.92)
Qinghai	Conduct disorder	4.13 (2.45 to 6.69)	87.25 (51.68 to 141.35)	3.33 (1.97 to 5.38)	52.94 (31.36 to 85.63)
Shaanxi	Conduct disorder	24.2 (14.3 to 39)	70.44 (41.61 to 113.43)	15.1 (8.93 to 24.2)	38.21 (22.59 to 61.29)
Shandong	Conduct disorder	59.5 (35.2 to 97.3)	69.47 (41.1 to 113.62)	44.6 (26.1 to 73.4)	44.85 (26.3 to 73.89)
Shanghai	Conduct disorder	5.99 (3.55 to 9.6)	42.89 (25.41 to 68.68)	7.09 (4.18 to 11.5)	25.36 (14.96 to 41.02)
Shanxi	Conduct disorder	21.7 (13.1 to 34.6)	71.52 (43.26 to 114.32)	17.1 (10.1 to 27.9)	44.31 (26.2 to 72.13)
Sichuan	Conduct disorder	75.9 (43.6 to 121)	67.98 (39.09 to 108.56)	32 (18.8 to 52.4)	38.3 (22.54 to 62.71)

Tianjin	Conduct disorder	5.05<br>(2.96 to 8.15)	54.95<br>(32.13 to 88.6)	4.21<br>(2.49 to 6.89)	27.61<br>(16.34 to 45.16)
Tibet	Conduct disorder	1.87<br>(1.11 to 3.04)	80.05<br>(47.47 to 130.39)	2.1<br>(1.25 to 3.44)	60.8<br>(36.28 to 99.6)
Xinjiang	Conduct disorder	13.4<br>(8.04 to 21.7)	84.77<br>(50.95 to 137.48)	12.1<br>(7.25 to 19.4)	49.64<br>(29.75 to 79.77)
Yunnan	Conduct disorder	33.2<br>(19.9 to 53.5)	85.22<br>(51.03 to 137.26)	26.9<br>(15.7 to 43.4)	54.76<br>(31.92 to 88.27)
Zhejiang	Conduct disorder	28.2<br>(16.9 to 45.7)	64.01<br>(38.28 to 103.58)	22.1<br>(13.2 to 36.2)	36.17<br>(21.61 to 59.19)
China	Idiopathic developmental intellectual disability	236<br>(71.8 to 451)	19.68<br>(6 to 37.68)	224<br>(71 to 421)	15.84<br>(5.03 to 29.8)
Taiwan	Idiopathic developmental intellectual disability	4.04<br>(1.53 to 7.42)	19.81<br>(7.51 to 36.38)	2.78<br>(0.857 to 5.23)	11.79<br>(3.63 to 22.16)
Hong Kong Special Administrative Region of China	Idiopathic developmental intellectual disability	0.311<br>(0.028 to 0.676)	5.39<br>(0.49 to 11.73)	0.56<br>(0.102 to 1.17)	7.46<br>(1.36 to 15.53)
Macau Special Administrative Region of China	Idiopathic developmental intellectual disability	0.0167<br>(0.00148 to 0.0381)	4.71<br>(0.42 to 10.72)	0.0421<br>(0.00655 to 0.089)	6.15<br>(0.96 to 13.01)
Anhui	Idiopathic developmental intellectual disability	13.1<br>(4.07 to 24.8)	22.07<br>(6.86 to 41.92)	11.4<br>(3.7 to 21.3)	18.66<br>(6.05 to 34.81)
Beijing	Idiopathic developmental intellectual disability	0.952<br>(0.131 to 2.11)	8.49<br>(1.17 to 18.83)	1.83<br>(0.261 to 4.06)	7.59<br>(1.08 to 16.83)
Chongqing	Idiopathic developmental intellectual disability	3.55<br>(1.26 to 6.51)	22.48<br>(8 to 41.25)	4.35<br>(1.41 to 8.15)	16.27<br>(5.29 to 30.51)
Fujian	Idiopathic developmental intellectual disability	7.03<br>(2.56 to 12.9)	22.23<br>(8.09 to 40.89)	5.94<br>(1.98 to 11)	15.06<br>(5.04 to 27.88)
Gansu	Idiopathic developmental intellectual disability	4.22<br>(0.8 to 8.56)	17.83<br>(3.38 to 36.2)	5.01<br>(1.43 to 9.69)	18.85<br>(5.37 to 36.46)
Guangdong	Idiopathic developmental intellectual disability	10.2<br>(2.34 to 20.5)	15.55<br>(3.55 to 31.11)	13.2<br>(3.2 to 26.7)	11.33<br>(2.74 to 22.87)
Guangxi	Idiopathic developmental intellectual disability	11.8<br>(4.32 to 21.8)	27.24<br>(9.93 to 50.13)	9.72<br>(3.32 to 17.7)	20.12<br>(6.87 to 36.67)
Guizhou	Idiopathic developmental intellectual disability	11.1<br>(4.44 to 19.8)	33.5<br>(13.37 to 59.64)	8.56<br>(3.38 to 15.6)	25.32<br>(10 to 46.09)
Hainan	Idiopathic developmental intellectual disability	1.28<br>(0.351 to 2.48)	18.32<br>(5.04 to 35.57)	1.78<br>(0.585 to 3.31)	18.61<br>(6.1 to 34.52)
Hebei	Idiopathic developmental intellectual disability	10.9<br>(2.55 to 21.9)	17.2<br>(4.01 to 34.46)	11.7<br>(3.42 to 22.2)	15.3<br>(4.47 to 29.08)
Heilongjiang	Idiopathic developmental intellectual disability	5.6<br>(1.29 to 11.2)	15.36<br>(3.55 to 30.67)	6.31<br>(1.97 to 11.9)	16.07<br>(5.01 to 30.34)
Henan	Idiopathic developmental intellectual disability	18.2<br>(5.46 to 35.2)	20.57<br>(6.18 to 39.92)	16.7<br>(5.25 to 31.6)	17.38<br>(5.45 to 32.76)
Hubei	Idiopathic developmental intellectual disability	10.7<br>(3.23 to 20.3)	19.26<br>(5.81 to 36.53)	9.03<br>(2.97 to 16.9)	16.32<br>(5.37 to 30.53)
Hunan	Idiopathic developmental intellectual disability	12.4<br>(3.71 to 23.8)	20.01<br>(5.97 to 38.31)	11.3<br>(3.31 to 21.5)	16.79<br>(4.93 to 31.99)
Inner Mongolia	Idiopathic developmental intellectual disability	4.25<br>(1.19 to 8.16)	18.71<br>(5.22 to 35.88)	3.97<br>(1.27 to 7.58)	15.32<br>(4.9 to 29.26)
Jiangsu	Idiopathic developmental intellectual disability	9.89<br>(2.29 to 19.5)	14.38<br>(3.33 to 28.4)	9.56<br>(2.57 to 18.4)	11.71<br>(3.15 to 22.49)
Jiangxi	Idiopathic developmental intellectual disability	9.32<br>(3.14 to 17.7)	23.56<br>(7.95 to 44.82)	9.34<br>(3.06 to 17.4)	19.65<br>(6.45 to 36.61)
Jilin	Idiopathic developmental intellectual disability	5<br>(1.64 to 9.43)	19.26<br>(6.31 to 36.34)	4.48<br>(1.52 to 8.23)	16.05<br>(5.45 to 29.52)
Liaoning	Idiopathic developmental intellectual disability	6.03<br>(1.65 to 11.5)	14.77<br>(4.05 to 28.3)	6.21<br>(1.98 to 11.6)	13.91<br>(4.42 to 25.92)
Ningxia	Idiopathic developmental intellectual disability	0.984<br>(0.305 to 1.89)	19.92<br>(6.17 to 38.24)	1.35<br>(0.464 to 2.49)	19.18<br>(6.6 to 35.41)
Qinghai	Idiopathic developmental intellectual disability	1.04<br>(0.346 to 1.94)	22<br>(7.3 to 40.98)	1.33<br>(0.495 to 2.41)	21.14<br>(7.87 to 38.33)
Shaanxi	Idiopathic developmental intellectual disability	7.59<br>(2.65 to 14.2)	22.07<br>(7.7 to 41.31)	7.49<br>(2.74 to 13.8)	18.95<br>(6.93 to 34.88)
Shandong	Idiopathic developmental intellectual disability	11.1<br>(1.59 to 23.2)	12.95<br>(1.86 to 27.07)	10.9<br>(2.23 to 22.4)	11<br>(2.24 to 22.5)
Shanghai	Idiopathic developmental intellectual disability	1.39<br>(0.309 to 2.79)	9.97<br>(2.21 to 19.96)	3.21<br>(0.945 to 6.04)	11.49<br>(3.38 to 21.63)

Shanxi	Idiopathic developmental intellectual disability	6.06 (1.83 to 11.4)	20.01 (6.04 to 37.64)	7.06 (2.43 to 12.9)	18.27 (6.28 to 33.36)
Sichuan	Idiopathic developmental intellectual disability	27.3 (9.46 to 50.6)	24.43 (8.48 to 45.31)	15.9 (5.64 to 29)	19.02 (6.75 to 34.75)
Tianjin	Idiopathic developmental intellectual disability	1.13 (0.242 to 2.23)	12.26 (2.63 to 24.22)	1.81 (0.519 to 3.5)	11.88 (3.4 to 22.94)
Tibet	Idiopathic developmental intellectual disability	0.698 (0.284 to 1.26)	29.96 (12.18 to 54.16)	0.973 (0.389 to 1.73)	28.15 (11.25 to 49.93)
Xinjiang	Idiopathic developmental intellectual disability	3.18 (0.971 to 6.07)	20.19 (6.16 to 38.51)	4.62 (1.65 to 8.44)	18.96 (6.79 to 34.64)
Yunnan	Idiopathic developmental intellectual disability	11.5 (4.53 to 20.9)	29.62 (11.62 to 53.61)	10.6 (3.76 to 19.6)	21.48 (7.64 to 39.86)
Zhejiang	Idiopathic developmental intellectual disability	7.61 (2.27 to 14.4)	17.25 (5.15 to 32.64)	7.43 (2.22 to 14.2)	12.14 (3.62 to 23.22)
China	Other mental disorders	1580 (1040 to 2190)	131.94 (86.73 to 182.77)	2390 (1570 to 3300)	169.08 (110.82 to 233.28)
Taiwan	Other mental disorders	29.9 (19.6 to 41.8)	146.38 (95.86 to 204.8)	42.6 (28.2 to 58.7)	180.49 (119.66 to 248.85)
Hong Kong Special Administrative Region of China	Other mental disorders	8.87 (5.78 to 12.2)	153.95 (100.21 to 211.89)	13.2 (8.77 to 18.2)	176.28 (116.73 to 242.57)
Macau Special Administrative Region of China	Other mental disorders	0.511 (0.334 to 0.713)	143.86 (94.19 to 200.8)	1.2 (0.799 to 1.67)	175.91 (116.81 to 244.1)
Anhui	Other mental disorders	74.6 (48.5 to 103)	125.91 (81.92 to 173.97)	102 (67 to 141)	166.88 (109.5 to 230.98)
Beijing	Other mental disorders	17.2 (11.2 to 24.1)	153.22 (99.94 to 214.77)	44.6 (29.1 to 61.8)	184.92 (120.84 to 256.35)
Chongqing	Other mental disorders	22.2 (14.5 to 31)	140.92 (91.72 to 196.65)	43.8 (28.7 to 60.5)	164 (107.42 to 226.28)
Fujian	Other mental disorders	38.6 (25 to 53.4)	121.89 (78.99 to 168.91)	67 (43.9 to 93)	169.95 (111.4 to 236.06)
Gansu	Other mental disorders	29.7 (19.4 to 41.7)	125.67 (81.84 to 176.19)	43.6 (28.8 to 60.6)	163.89 (108.27 to 228.02)
Guangdong	Other mental disorders	82.7 (54.8 to 115)	125.7 (83.26 to 174.08)	196 (128 to 272)	167.74 (109.63 to 232.51)
Guangxi	Other mental disorders	52.7 (34.6 to 73.5)	121.25 (79.61 to 169.09)	75.6 (49.7 to 104)	156.46 (102.78 to 216.05)
Guizhou	Other mental disorders	39.8 (26.1 to 55.4)	119.9 (78.54 to 166.77)	53.1 (34.7 to 73.5)	156.83 (102.6 to 217.29)
Hainan	Other mental disorders	8.24 (5.4 to 11.4)	118.35 (77.54 to 163.72)	15.6 (10.3 to 21.5)	162.5 (107.16 to 224.76)
Hebei	Other mental disorders	84.3 (55.6 to 118)	132.53 (87.35 to 184.84)	125 (81.6 to 172)	162.97 (106.78 to 225.49)
Heilongjiang	Other mental disorders	48.6 (31.9 to 67.4)	133.26 (87.58 to 184.88)	72.7 (48.1 to 101)	185.16 (122.48 to 256.25)
Henan	Other mental disorders	114 (74.3 to 159)	128.71 (84.21 to 179.72)	150 (98.8 to 207)	155.77 (102.59 to 215.01)
Hubei	Other mental disorders	73.3 (47.7 to 102)	131.91 (85.88 to 183.51)	96.9 (63.8 to 134)	175.27 (115.44 to 242.17)
Hunan	Other mental disorders	82.4 (53.8 to 115)	132.44 (86.47 to 184.84)	111 (73.3 to 154)	165.36 (109.17 to 228.74)
Inner Mongolia	Other mental disorders	29 (18.9 to 40.5)	127.4 (83.19 to 178)	46.3 (30.5 to 64.1)	178.92 (117.95 to 247.51)
Jiangsu	Other mental disorders	99 (64.5 to 138)	143.83 (93.73 to 200.15)	144 (94.9 to 201)	176.94 (116.21 to 246.17)
Jiangxi	Other mental disorders	47.4 (30.8 to 66)	120.01 (77.86 to 167.02)	74.9 (48.7 to 104)	157.55 (102.35 to 218.57)
Jilin	Other mental disorders	34.9 (22.8 to 48.9)	134.38 (87.75 to 188.52)	50.8 (33.2 to 70.3)	182.06 (119.21 to 252)
Liaoning	Other mental disorders	59 (38.8 to 82.4)	144.6 (95.1 to 202.12)	82.1 (54 to 114)	183.77 (120.85 to 255.85)
Ningxia	Other mental disorders	5.67 (3.73 to 7.93)	114.71 (75.5 to 160.61)	11.2 (7.29 to 15.7)	159.73 (103.89 to 223.55)
Qinghai	Other mental disorders	5.62 (3.69 to 7.85)	118.58 (77.9 to 165.78)	10.2 (6.72 to 14.2)	162.86 (106.98 to 225.45)
Shaanxi	Other mental disorders	44.9 (29.3 to 62.3)	130.35 (85.27 to 181.1)	68.3 (45.2 to 94.7)	172.87 (114.37 to 239.72)

Shandong	Other mental disorders	118<br>(77 to 165)	138.38<br>(89.94 to 192.48)	170<br>(112 to 236)	171.28<br>(113.08 to 237.39)
Shanghai	Other mental disorders	22.5<br>(14.7 to 31.2)	161.06<br>(105.31 to 223.56)	52.1<br>(34.5 to 71.9)	186.48<br>(123.38 to 257.26)
Shanxi	Other mental disorders	39.4<br>(25.7 to 54.9)	130.06<br>(84.74 to 181.18)	65.1<br>(42.4 to 90.3)	168.61<br>(109.73 to 233.75)
Sichuan	Other mental disorders	153<br>(101 to 214)	137.17<br>(90.1 to 191.86)	142<br>(92.4 to 194)	169.49<br>(110.7 to 232.94)
Tianjin	Other mental disorders	13.6<br>(8.74 to 18.9)	147.37<br>(95.08 to 205.42)	28.6<br>(18.8 to 39.8)	187.19<br>(123.23 to 260.98)
Tibet	Other mental disorders	2.65<br>(1.73 to 3.7)	113.86<br>(74.32 to 158.83)	5.12<br>(3.34 to 7.13)	148.05<br>(96.69 to 206.29)
Xinjiang	Other mental disorders	18.6<br>(12.1 to 26)	118.05<br>(76.88 to 165.04)	39.4<br>(25.7 to 54.7)	161.66<br>(105.52 to 224.62)
Yunnan	Other mental disorders	46.6<br>(30.6 to 65)	119.61<br>(78.57 to 166.76)	79<br>(51.6 to 109)	160.57<br>(104.85 to 220.86)
Zhejiang	Other mental disorders	61.7<br>(40.7 to 85.2)	139.88<br>(92.2 to 193.17)	108<br>(71 to 149)	176.75<br>(116.08 to 244.01)
China	Diabetes mellitus	4950<br>(3880 to 6280)	413.53<br>(324.23 to 524.4)	10000<br>(7720 to 12800)	709.68<br>(546.81 to 906.74)
Taiwan	Diabetes mellitus	193<br>(161 to 231)	944.07<br>(789.79 to 1133.11)	344<br>(285 to 418)	1457.54<br>(1210.1 to 1771.72)
Hong Kong Special Administrative Region of China	Diabetes mellitus	19.7<br>(15 to 25.3)	341.95<br>(260.5 to 439.41)	37.2<br>(27.3 to 49.2)	495.2<br>(364 to 654.84)
Macau Special Administrative Region of China	Diabetes mellitus	1.12<br>(0.873 to 1.44)	316.11<br>(245.86 to 406.92)	3.51<br>(2.54 to 4.68)	512.7<br>(371.67 to 685.05)
Anhui	Diabetes mellitus	244<br>(192 to 307)	411.26<br>(323.81 to 518.88)	437<br>(338 to 555)	714.46<br>(552.07 to 907.2)
Beijing	Diabetes mellitus	58.8<br>(45.3 to 76)	524.26<br>(403.81 to 677.2)	182<br>(133 to 243)	753.51<br>(552.01 to 1008.76)
Chongqing	Diabetes mellitus	64.2<br>(50.1 to 81.5)	406.49<br>(317.23 to 516.29)	185<br>(141 to 236)	692.22<br>(526.3 to 882.58)
Fujian	Diabetes mellitus	127<br>(101 to 159)	401.15<br>(318.67 to 502.63)	261<br>(202 to 338)	662.53<br>(511.44 to 857.93)
Gansu	Diabetes mellitus	104<br>(83 to 131)	440.93<br>(350.79 to 554.98)	170<br>(134 to 218)	641.46<br>(503.25 to 819.1)
Guangdong	Diabetes mellitus	318<br>(242 to 413)	483<br>(368.02 to 627.73)	756<br>(557 to 996)	647.47<br>(477.01 to 852.46)
Guangxi	Diabetes mellitus	159<br>(125 to 202)	365.99<br>(288.4 to 463.53)	302<br>(234 to 381)	625.66<br>(485.2 to 788.19)
Guizhou	Diabetes mellitus	136<br>(108 to 170)	409.79<br>(325.44 to 512.03)	219<br>(171 to 276)	647.15<br>(505.03 to 814.52)
Hainan	Diabetes mellitus	35.8<br>(28.4 to 45.6)	514.43<br>(407.02 to 654.26)	73.7<br>(55.2 to 95.3)	768.21<br>(575.36 to 994.11)
Hebei	Diabetes mellitus	247<br>(188 to 320)	387.83<br>(295.06 to 502.95)	602<br>(463 to 770)	787.09<br>(606.2 to 1008.15)
Heilongjiang	Diabetes mellitus	168<br>(131 to 216)	461.56<br>(359.21 to 592.06)	349<br>(265 to 451)	889.79<br>(674.6 to 1148.78)
Henan	Diabetes mellitus	317<br>(246 to 404)	359.58<br>(278.4 to 457.98)	660<br>(519 to 830)	685.46<br>(538.42 to 861.57)
Hubei	Diabetes mellitus	178<br>(138 to 226)	320.07<br>(248.46 to 407.24)	353<br>(277 to 451)	637.96<br>(500.33 to 815.58)
Hunan	Diabetes mellitus	285<br>(229 to 354)	457.74<br>(367.64 to 569.26)	519<br>(403 to 652)	773.44<br>(599.54 to 971.47)
Inner Mongolia	Diabetes mellitus	90.3<br>(69.1 to 118)	397.31<br>(303.94 to 518.7)	185<br>(136 to 244)	715.36<br>(523.55 to 940.96)
Jiangsu	Diabetes mellitus	302<br>(236 to 390)	439.13<br>(342.33 to 566.65)	645<br>(496 to 823)	789.51<br>(606.97 to 1008.21)
Jiangxi	Diabetes mellitus	165<br>(133 to 204)	417.45<br>(337.06 to 515.94)	260<br>(199 to 331)	547.45<br>(418.48 to 695.28)
Jilin	Diabetes mellitus	135<br>(104 to 173)	518.99<br>(399.87 to 666.81)	240<br>(180 to 310)	859.27<br>(645.74 to 1112.72)
Liaoning	Diabetes mellitus	252<br>(201 to 321)	618.49<br>(492.38 to 787.49)	494<br>(388 to 617)	1106.65<br>(869.58 to 1381.27)
Ningxia	Diabetes mellitus	14.2<br>(11 to 18.2)	287.73<br>(223.4 to 368.08)	35<br>(26.7 to 45.7)	498.91<br>(380.33 to 650.95)

Qinghai	Diabetes mellitus	16.6<br>(13.4 to 20.5)	350.43<br>(283.44 to 433.25)	38.6<br>(30.5 to 48.7)	613.94<br>(485.1 to 775.7)
Shaanxi	Diabetes mellitus	87.5<br>(65.5 to 113)	254.22<br>(190.28 to 329.39)	238<br>(183 to 304)	601.81<br>(461.99 to 769.85)
Shandong	Diabetes mellitus	343<br>(264 to 439)	400.21<br>(308.6 to 513.09)	650<br>(491 to 844)	653.77<br>(494.15 to 849.63)
Shanghai	Diabetes mellitus	81.4<br>(64 to 103)	582.88<br>(457.95 to 734)	211<br>(160 to 274)	755.74<br>(571.9 to 980.05)
Shanxi	Diabetes mellitus	131<br>(100 to 169)	432.18<br>(330.29 to 559.62)	271<br>(198 to 358)	700.42<br>(513.89 to 927.34)
Sichuan	Diabetes mellitus	383<br>(293 to 493)	343.51<br>(262.46 to 442.14)	603<br>(473 to 756)	722.65<br>(566.07 to 905.75)
Tianjin	Diabetes mellitus	59<br>(46.2 to 75.1)	641.43<br>(502.84 to 816.55)	146<br>(110 to 189)	955.5<br>(720.67 to 1241.18)
Tibet	Diabetes mellitus	8.24<br>(6.57 to 10.2)	353.41<br>(282.04 to 437.32)	13.6<br>(10.4 to 17.6)	393.84<br>(300.18 to 509.06)
Xinjiang	Diabetes mellitus	75.4<br>(59.9 to 94.6)	478.23<br>(379.56 to 600.05)	217<br>(168 to 273)	890.89<br>(689.62 to 1121.79)
Yunnan	Diabetes mellitus	156<br>(125 to 195)	400.92<br>(320.16 to 501.11)	303<br>(237 to 379)	615.36<br>(482.28 to 770.09)
Zhejiang	Diabetes mellitus	187<br>(147 to 239)	424.84<br>(333.89 to 541.34)	365<br>(273 to 474)	596.05<br>(445.9 to 773.97)
China	Acute glomerulonephritis	377<br>(346 to 414)	31.5<br>(28.94 to 34.61)	187<br>(176 to 200)	13.24<br>(12.44 to 14.19)
Taiwan	Acute glomerulonephritis	0.571<br>(0.517 to 0.629)	2.8<br>(2.53 to 3.08)	0.0807<br>(0.0696 to 0.0927)	0.34<br>(0.3 to 0.39)
Hong Kong Special Administrative Region of China	Acute glomerulonephritis	0.681<br>(0.562 to 0.821)	11.81<br>(9.76 to 14.25)	0.619<br>(0.49 to 0.78)	8.24<br>(6.53 to 10.39)
Macau Special Administrative Region of China	Acute glomerulonephritis	0.0433<br>(0.0356 to 0.0519)	12.2<br>(10.02 to 14.61)	0.0296<br>(0.0205 to 0.0467)	4.33<br>(3 to 6.83)
Anhui	Acute glomerulonephritis	7.33<br>(6.03 to 8.63)	12.36<br>(10.17 to 14.56)	3.26<br>(2.69 to 4)	5.34<br>(4.39 to 6.54)
Beijing	Acute glomerulonephritis	0.467<br>(0.379 to 0.565)	4.17<br>(3.38 to 5.04)	0.369<br>(0.29 to 0.466)	1.53<br>(1.2 to 1.93)
Chongqing	Acute glomerulonephritis	5.15<br>(4.2 to 6.32)	32.62<br>(26.58 to 40.06)	3.48<br>(2.74 to 4.35)	13.02<br>(10.24 to 16.29)
Fujian	Acute glomerulonephritis	4.76<br>(3.91 to 5.65)	15.04<br>(12.37 to 17.85)	2.25<br>(1.83 to 2.77)	5.72<br>(4.64 to 7.02)
Gansu	Acute glomerulonephritis	16.6<br>(13.6 to 20)	70.09<br>(57.48 to 84.51)	6.54<br>(5.38 to 7.83)	24.61<br>(20.25 to 29.48)
Guangdong	Acute glomerulonephritis	14.6<br>(12.1 to 17.8)	22.2<br>(18.32 to 27.09)	8.93<br>(7.22 to 10.8)	7.64<br>(6.18 to 9.25)
Guangxi	Acute glomerulonephritis	16.5<br>(13.4 to 20.3)	37.86<br>(30.92 to 46.77)	8.67<br>(6.96 to 10.6)	17.95<br>(14.4 to 21.99)
Guizhou	Acute glomerulonephritis	24.8<br>(20.2 to 31)	74.72<br>(60.99 to 93.43)	8.73<br>(7.04 to 10.7)	25.82<br>(20.81 to 31.7)
Hainan	Acute glomerulonephritis	2.91<br>(2.28 to 3.61)	41.74<br>(32.76 to 51.86)	1.22<br>(0.958 to 1.53)	12.72<br>(10 to 15.97)
Hebei	Acute glomerulonephritis	12.7<br>(10.6 to 15.2)	19.95<br>(16.59 to 23.92)	8.56<br>(6.87 to 10.7)	11.2<br>(8.98 to 13.95)
Heilongjiang	Acute glomerulonephritis	16<br>(13.2 to 18.7)	43.94<br>(36.25 to 51.33)	8.03<br>(6.45 to 9.81)	20.47<br>(16.43 to 24.99)
Henan	Acute glomerulonephritis	14.4<br>(12.1 to 17.2)	16.37<br>(13.67 to 19.45)	9.05<br>(7.34 to 11)	9.4<br>(7.62 to 11.39)
Hubei	Acute glomerulonephritis	17.5<br>(14.8 to 20.4)	31.44<br>(26.56 to 36.71)	10.6<br>(8.61 to 12.8)	19.19<br>(15.57 to 23.14)
Hunan	Acute glomerulonephritis	36.9<br>(30.3 to 43.6)	59.34<br>(48.72 to 70.18)	17.6<br>(14.1 to 21.1)	26.19<br>(20.98 to 31.37)
Inner Mongolia	Acute glomerulonephritis	6.29<br>(5.18 to 7.61)	27.64<br>(22.76 to 33.47)	2.53<br>(2.03 to 3.09)	9.79<br>(7.85 to 11.93)
Jiangsu	Acute glomerulonephritis	9<br>(7.57 to 10.6)	13.08<br>(11 to 15.36)	5.45<br>(4.33 to 6.73)	6.67<br>(5.3 to 8.24)
Jiangxi	Acute glomerulonephritis	24.8<br>(20.7 to 29.5)	62.68<br>(52.37 to 74.52)	9.58<br>(7.9 to 11.5)	20.14<br>(16.62 to 24.13)
Jilin	Acute glomerulonephritis	10.1<br>(8.32 to 12.2)	38.89<br>(32.04 to 46.88)	3.92<br>(3.08 to 4.82)	14.06<br>(11.04 to 17.28)

Liaoning	Acute glomerulonephritis	5.16<br>(4.17 to 6.33)	12.64<br>(10.22 to 15.52)	2.88<br>(2.32 to 3.47)	6.44<br>(5.2 to 7.77)
Ningxia	Acute glomerulonephritis	2.66<br>(2.16 to 3.37)	53.88<br>(43.84 to 68.33)	1.31<br>(1.04 to 1.62)	18.64<br>(14.82 to 23.14)
Qinghai	Acute glomerulonephritis	3.36<br>(2.72 to 4.13)	70.96<br>(57.34 to 87.27)	1.77<br>(1.39 to 2.21)	28.17<br>(22.11 to 35.18)
Shaanxi	Acute glomerulonephritis	7.14<br>(5.88 to 8.58)	20.75<br>(17.1 to 24.94)	5.62<br>(4.5 to 6.92)	14.22<br>(11.39 to 17.52)
Shandong	Acute glomerulonephritis	16.9<br>(14.2 to 20.2)	19.76<br>(16.62 to 23.65)	9.18<br>(7.51 to 11.2)	9.23<br>(7.56 to 11.23)
Shanghai	Acute glomerulonephritis	0.223<br>(0.176 to 0.258)	1.59<br>(1.26 to 1.85)	0.185<br>(0.145 to 0.227)	0.66<br>(0.52 to 0.81)
Shanxi	Acute glomerulonephritis	7.92<br>(6.53 to 9.66)	26.14<br>(21.58 to 31.9)	3.96<br>(3.1 to 4.93)	10.26<br>(8.02 to 12.77)
Sichuan	Acute glomerulonephritis	34.1<br>(28.6 to 41)	30.53<br>(25.59 to 36.73)	14.7<br>(11.8 to 18.1)	17.59<br>(14.13 to 21.64)
Tianjin	Acute glomerulonephritis	0.838<br>(0.691 to 1.01)	9.11<br>(7.51 to 11.02)	0.679<br>(0.549 to 0.831)	4.45<br>(3.6 to 5.45)
Tibet	Acute glomerulonephritis	3.1<br>(2.54 to 3.83)	133.04<br>(109.15 to 164.49)	1.63<br>(1.34 to 1.96)	47.28<br>(38.7 to 56.84)
Xinjiang	Acute glomerulonephritis	6.88<br>(5.55 to 8.41)	43.59<br>(35.19 to 53.34)	3.68<br>(2.87 to 4.67)	15.12<br>(11.79 to 19.15)
Yunnan	Acute glomerulonephritis	43.3<br>(35.1 to 53.7)	111.21<br>(89.97 to 137.75)	20<br>(16.6 to 24.3)	40.72<br>(33.84 to 49.44)
Zhejiang	Acute glomerulonephritis	3.93<br>(3.26 to 4.69)	8.9<br>(7.38 to 10.63)	1.93<br>(1.53 to 2.43)	3.15<br>(2.51 to 3.98)
China	Chronic kidney disease	4230<br>(3950 to 4540)	353.39<br>(330.01 to 379.6)	4890<br>(4500 to 5250)	345.98<br>(318.77 to 371.81)
Taiwan	Chronic kidney disease	84.4<br>(78.3 to 90.4)	413.69<br>(383.95 to 443.11)	156<br>(141 to 171)	661.86<br>(598.12 to 723.93)
Hong Kong Special Administrative Region of China	Chronic kidney disease	25.5<br>(19.9 to 28.1)	442.65<br>(345.53 to 488.37)	31.5<br>(25 to 36.7)	419.71<br>(333.54 to 489.21)
Macau Special Administrative Region of China	Chronic kidney disease	0.991<br>(0.878 to 1.1)	279.25<br>(247.47 to 308.62)	1.7<br>(1.44 to 1.95)	248.35<br>(210.44 to 285.77)
Anhui	Chronic kidney disease	171<br>(151 to 200)	288.84<br>(255.34 to 337.1)	183<br>(159 to 210)	299.37<br>(259.65 to 343.25)
Beijing	Chronic kidney disease	32.7<br>(28.7 to 37.2)	291.1<br>(256.3 to 331.76)	50.1<br>(42.4 to 57.8)	207.73<br>(175.82 to 239.72)
Chongqing	Chronic kidney disease	59.5<br>(52.7 to 67.6)	376.73<br>(333.72 to 428.47)	94.2<br>(78.8 to 111)	352.38<br>(294.76 to 414.05)
Fujian	Chronic kidney disease	89.2<br>(80.5 to 98.7)	282.09<br>(254.38 to 311.82)	108<br>(92.1 to 125)	273.53<br>(233.68 to 318.31)
Gansu	Chronic kidney disease	118<br>(104 to 131)	500.41<br>(437.78 to 555.96)	98.4<br>(84.8 to 114)	370.07<br>(319.16 to 427.69)
Guangdong	Chronic kidney disease	237<br>(207 to 269)	359.76<br>(314.97 to 408.11)	309<br>(262 to 362)	264.84<br>(224.65 to 309.77)
Guangxi	Chronic kidney disease	193<br>(166 to 218)	443.82<br>(381.36 to 502.09)	215<br>(173 to 251)	445.26<br>(357.93 to 520.16)
Guizhou	Chronic kidney disease	181<br>(158 to 205)	544.98<br>(476.16 to 618.57)	154<br>(129 to 181)	456.66<br>(380.53 to 535.01)
Hainan	Chronic kidney disease	25<br>(21.8 to 28.7)	358.25<br>(312.87 to 412.28)	26<br>(21.8 to 30.8)	271.54<br>(227.53 to 320.78)
Hebei	Chronic kidney disease	195<br>(176 to 217)	306.89<br>(276.26 to 340.4)	284<br>(244 to 324)	371.78<br>(319.64 to 424.05)
Heilongjiang	Chronic kidney disease	119<br>(106 to 137)	326.79<br>(290.26 to 376.46)	129<br>(110 to 155)	328.16<br>(281.15 to 395.53)
Henan	Chronic kidney disease	227<br>(205 to 256)	256.98<br>(232.34 to 290.57)	301<br>(262 to 344)	312.68<br>(272.46 to 357.03)
Hubei	Chronic kidney disease	186<br>(167 to 204)	334.51<br>(300.56 to 367.58)	244<br>(204 to 281)	441.43<br>(368.18 to 507.38)
Hunan	Chronic kidney disease	371<br>(321 to 412)	597.36<br>(516.64 to 661.89)	428<br>(320 to 504)	637.3<br>(476.29 to 750.46)
Inner Mongolia	Chronic kidney disease	77<br>(68.4 to 88.5)	338.71<br>(300.83 to 389.31)	79.6<br>(68.7 to 92.8)	307.3<br>(265.13 to 358.34)
Jiangsu	Chronic kidney disease	176<br>(155 to 210)	255.6<br>(225.24 to 304.82)	231<br>(198 to 272)	282.83<br>(242.73 to 333.32)

Jiangxi	Chronic kidney disease	231<br>(204 to 255)	583.83<br>(516.32 to 645.36)	180<br>(147 to 207)	377.76<br>(308.97 to 435.5)
Jilin	Chronic kidney disease	101<br>(91 to 113)	390.86<br>(350.82 to 435.7)	100<br>(84.2 to 117)	359.29<br>(301.84 to 421.29)
Liaoning	Chronic kidney disease	137<br>(120 to 157)	337<br>(294.35 to 385.48)	172<br>(147 to 200)	385.93<br>(329.39 to 448.64)
Ningxia	Chronic kidney disease	15.1<br>(13.2 to 17.1)	304.79<br>(266.9 to 345.9)	17.7<br>(14.9 to 20.7)	251.78<br>(212.96 to 295.16)
Qinghai	Chronic kidney disease	22.7<br>(19.8 to 25.9)	480.25<br>(418.18 to 547.5)	25.3<br>(21.3 to 29.8)	402.66<br>(339.05 to 474.41)
Shaanxi	Chronic kidney disease	77.7<br>(69.1 to 86.5)	225.84<br>(200.9 to 251.39)	134<br>(110 to 157)	339.31<br>(279.1 to 396.75)
Shandong	Chronic kidney disease	239<br>(209 to 285)	278.76<br>(244.52 to 333.1)	276<br>(234 to 327)	277.36<br>(235.83 to 329.39)
Shanghai	Chronic kidney disease	40.6<br>(35.7 to 45.8)	290.64<br>(255.65 to 327.66)	66.2<br>(56 to 76.6)	236.82<br>(200.5 to 274.18)
Shanxi	Chronic kidney disease	103<br>(90.8 to 121)	338.97<br>(299.7 to 399.94)	106<br>(89.9 to 125)	274.11<br>(232.64 to 324.52)
Sichuan	Chronic kidney disease	322<br>(287 to 363)	288.4<br>(257.54 to 325.37)	319<br>(274 to 370)	382.16<br>(328.19 to 443.37)
Tianjin	Chronic kidney disease	27.8<br>(24.2 to 32.7)	301.78<br>(263.41 to 355.1)	43.3<br>(37 to 51.4)	283.71<br>(242.5 to 336.95)
Tibet	Chronic kidney disease	20.9<br>(16.6 to 23.9)	897.22<br>(713.19 to 1027.62)	15.8<br>(12.9 to 18.4)	458.18<br>(372.42 to 533.19)
Xinjiang	Chronic kidney disease	61.4<br>(54.8 to 67.7)	389.51<br>(347.15 to 429.01)	93.2<br>(80.9 to 108)	382.46<br>(332.15 to 444.77)
Yunnan	Chronic kidney disease	208<br>(181 to 239)	533.75<br>(464.19 to 612.77)	215<br>(179 to 248)	436.38<br>(364.95 to 505.22)
Zhejiang	Chronic kidney disease	138<br>(124 to 157)	313.84<br>(280.99 to 355.39)	156<br>(134 to 181)	254.43<br>(218.22 to 295.38)
China	Urinary diseases and male infertility	817<br>(702 to 965)	68.24<br>(58.62 to 80.58)	780<br>(602 to 1000)	55.2<br>(42.61 to 70.83)
Taiwan	Urinary diseases and male infertility	17.8<br>(15.1 to 21.1)	87<br>(73.86 to 103.53)	49.2<br>(42.7 to 56.6)	208.56<br>(181.16 to 239.81)
Hong Kong Special Administrative Region of China	Urinary diseases and male infertility	3.83<br>(3.09 to 4.75)	66.49<br>(53.55 to 82.42)	4.86<br>(3.71 to 6.31)	64.78<br>(49.45 to 84.03)
Macau Special Administrative Region of China	Urinary diseases and male infertility	0.197<br>(0.162 to 0.238)	55.48<br>(45.62 to 66.99)	0.404<br>(0.319 to 0.511)	59.02<br>(46.66 to 74.66)
Anhui	Urinary diseases and male infertility	27.9<br>(22.9 to 34.1)	47.16<br>(38.59 to 57.52)	25.4<br>(18.5 to 34.1)	41.49<br>(30.22 to 55.7)
Beijing	Urinary diseases and male infertility	6.38<br>(5.11 to 7.97)	56.84<br>(45.57 to 71.03)	10.1<br>(7.27 to 13.6)	41.86<br>(30.15 to 56.55)
Chongqing	Urinary diseases and male infertility	11<br>(8.96 to 13.3)	69.59<br>(56.8 to 84.3)	18<br>(13 to 24.1)	67.34<br>(48.73 to 90.15)
Fujian	Urinary diseases and male infertility	19.6<br>(16.8 to 23)	62.1<br>(53.05 to 72.67)	18.4<br>(14.2 to 23.9)	46.64<br>(35.93 to 60.73)
Gansu	Urinary diseases and male infertility	21.4<br>(18.4 to 25.1)	90.52<br>(77.76 to 106.08)	15.6<br>(11.9 to 20.2)	58.59<br>(44.77 to 76.17)
Guangdong	Urinary diseases and male infertility	48.6<br>(41.2 to 57.3)	73.79<br>(62.55 to 87.03)	58<br>(44.3 to 74.3)	49.65<br>(37.92 to 63.6)
Guangxi	Urinary diseases and male infertility	35.2<br>(30.2 to 41.1)	81.05<br>(69.37 to 94.56)	30.5<br>(24 to 38.4)	63.13<br>(49.76 to 79.43)
Guizhou	Urinary diseases and male infertility	38.7<br>(33.3 to 45.1)	116.71<br>(100.28 to 135.78)	29.4<br>(24 to 35.7)	86.79<br>(70.81 to 105.67)
Hainan	Urinary diseases and male infertility	5.03<br>(4.24 to 6.06)	72.24<br>(60.86 to 86.94)	5.46<br>(4.35 to 6.76)	57<br>(45.33 to 70.49)
Hebei	Urinary diseases and male infertility	27<br>(21 to 34)	42.43<br>(33.05 to 53.5)	32.9<br>(24.8 to 42.7)	43<br>(32.48 to 55.94)
Heilongjiang	Urinary diseases and male infertility	19.3<br>(15.9 to 23.6)	52.98<br>(43.7 to 64.61)	20.8<br>(15.7 to 27)	52.87<br>(40.08 to 68.78)
Henan	Urinary diseases and male infertility	38.9<br>(30.9 to 49)	44.08<br>(35.06 to 55.49)	37.9<br>(28 to 50.9)	39.35<br>(29.08 to 52.81)
Hubei	Urinary diseases and male infertility	43.6<br>(36.1 to 52.2)	78.49<br>(64.99 to 93.88)	45.8<br>(34.5 to 59.6)	82.89<br>(62.44 to 107.83)
Hunan	Urinary diseases and male infertility	60.5<br>(52.5 to 69.6)	97.21<br>(84.38 to 111.9)	58.2<br>(46.8 to 71)	86.67<br>(69.74 to 105.7)

Inner Mongolia	Urinary diseases and male infertility	12.8<br>(10.6 to 15.4)	56.37<br>(46.61 to 67.84)	12.8<br>(9.39 to 17.1)	49.44<br>(36.27 to 65.92)
Jiangsu	Urinary diseases and male infertility	33.3<br>(26.7 to 41.3)	48.38<br>(38.77 to 60.05)	37.4<br>(27.4 to 50.4)	45.78<br>(33.6 to 61.73)
Jiangxi	Urinary diseases and male infertility	43.4<br>(37.8 to 50)	109.81<br>(95.59 to 126.39)	27.1<br>(21.2 to 34.5)	56.93<br>(44.57 to 72.63)
Jilin	Urinary diseases and male infertility	10.7<br>(8.46 to 13.6)	41.27<br>(32.6 to 52.27)	12.9<br>(9.18 to 17.4)	46.29<br>(32.91 to 62.43)
Liaoning	Urinary diseases and male infertility	19.6<br>(15.5 to 24.7)	47.94<br>(38.09 to 60.56)	23.1<br>(17 to 30.7)	51.73<br>(38.13 to 68.76)
Ningxia	Urinary diseases and male infertility	3.42<br>(2.83 to 4.08)	69.33<br>(57.28 to 82.67)	2.97<br>(2.25 to 3.85)	42.32<br>(32.11 to 54.88)
Qinghai	Urinary diseases and male infertility	4.04<br>(3.41 to 4.72)	85.23<br>(72.09 to 99.58)	3.19<br>(2.54 to 3.91)	50.7<br>(40.41 to 62.31)
Shaanxi	Urinary diseases and male infertility	17.5<br>(14.3 to 21.3)	50.72<br>(41.46 to 61.78)	18.5<br>(13.9 to 24)	46.85<br>(35.09 to 60.75)
Shandong	Urinary diseases and male infertility	43.7<br>(35.3 to 53.5)	51.03<br>(41.24 to 62.51)	44.2<br>(32.1 to 59.5)	44.48<br>(32.26 to 59.83)
Shanghai	Urinary diseases and male infertility	9.4<br>(7.66 to 11.5)	67.3<br>(54.84 to 82.25)	13.6<br>(9.99 to 17.9)	48.57<br>(35.74 to 64.23)
Shanxi	Urinary diseases and male infertility	16.7<br>(13.8 to 20.4)	55.23<br>(45.49 to 67.34)	16.9<br>(12.6 to 22.4)	43.78<br>(32.67 to 57.99)
Sichuan	Urinary diseases and male infertility	78.3<br>(65.2 to 93.7)	70.16<br>(58.45 to 83.96)	55.5<br>(41.8 to 71.9)	66.48<br>(50.03 to 86.15)
Tianjin	Urinary diseases and male infertility	3.95<br>(3.05 to 5.17)	42.98<br>(33.13 to 56.17)	5.63<br>(4.02 to 7.82)	36.89<br>(26.34 to 51.24)
Tibet	Urinary diseases and male infertility	5.88<br>(5.16 to 6.7)	252.54<br>(221.6 to 287.36)	3.1<br>(2.64 to 3.64)	89.78<br>(76.29 to 105.18)
Xinjiang	Urinary diseases and male infertility	16.3<br>(14.1 to 19)	103.3<br>(89.44 to 120.66)	18.9<br>(15.4 to 22.6)	77.43<br>(63.04 to 92.9)
Yunnan	Urinary diseases and male infertility	61.1<br>(53.1 to 70.5)	156.87<br>(136.17 to 180.82)	39.1<br>(32.1 to 47.2)	79.47<br>(65.27 to 95.95)
Zhejiang	Urinary diseases and male infertility	29.5<br>(24.4 to 36)	66.81<br>(55.24 to 81.73)	33.2<br>(24.1 to 44.7)	54.34<br>(39.36 to 73.01)
China	Gynecological diseases	2100<br>(1460 to 2920)	175.79<br>(121.64 to 244)	2790<br>(1920 to 3860)	197.49<br>(136.02 to 273.56)
Taiwan	Gynecological diseases	51.4<br>(35.6 to 71.2)	251.9<br>(174.38 to 348.81)	78.1<br>(53.7 to 108)	331.3<br>(227.65 to 458.89)
Hong Kong Special Administrative Region of China	Gynecological diseases	10.3<br>(7.07 to 14.3)	177.85<br>(122.63 to 248.75)	14.9<br>(10.3 to 21.6)	198.98<br>(137.38 to 287.4)
Macau Special Administrative Region of China	Gynecological diseases	0.636<br>(0.436 to 0.893)	179.34<br>(122.75 to 251.72)	1.35<br>(0.951 to 1.93)	197.83<br>(139.02 to 282.53)
Anhui	Gynecological diseases	103<br>(71.5 to 142)	174.33<br>(120.69 to 239.98)	132<br>(89.7 to 187)	215.72<br>(146.69 to 305.56)
Beijing	Gynecological diseases	33.6<br>(23.2 to 46.3)	299.26<br>(206.89 to 412.63)	80.7<br>(55.7 to 113)	334.72<br>(231.07 to 470.59)
Chongqing	Gynecological diseases	32.1<br>(22.4 to 44.2)	203.29<br>(141.69 to 280.16)	54.4<br>(37.6 to 75.5)	203.37<br>(140.68 to 282.52)
Fujian	Gynecological diseases	43.6<br>(30.1 to 61.5)	137.73<br>(95.04 to 194.4)	65.3<br>(44.8 to 92.7)	165.76<br>(113.71 to 235.16)
Gansu	Gynecological diseases	34.9<br>(23.9 to 49)	147.34<br>(101.08 to 207.25)	42.5<br>(28.9 to 61)	159.85<br>(108.62 to 229.56)
Guangdong	Gynecological diseases	115<br>(79.2 to 160)	174.61<br>(120.32 to 243)	244<br>(167 to 338)	208.7<br>(143.29 to 289.22)
Guangxi	Gynecological diseases	65.7<br>(46.1 to 91.6)	151.18<br>(105.97 to 210.6)	83.8<br>(57.4 to 118)	173.52<br>(118.76 to 245.09)
Guizhou	Gynecological diseases	51.8<br>(36.2 to 71.9)	156.2<br>(108.98 to 216.63)	61.2<br>(42.4 to 84.8)	181.06<br>(125.22 to 250.72)
Hainan	Gynecological diseases	8.55<br>(5.86 to 11.9)	122.75<br>(84.16 to 171.51)	13.8<br>(9.39 to 19.6)	143.97<br>(97.98 to 204.27)
Hebei	Gynecological diseases	114<br>(79 to 159)	179.62<br>(124.09 to 249.42)	142<br>(98.7 to 200)	186.44<br>(129.14 to 261.29)
Heilongjiang	Gynecological diseases	79.8<br>(55.1 to 112)	218.85<br>(151.21 to 307.9)	103<br>(71.1 to 145)	261.85<br>(181.02 to 370.23)
Henan	Gynecological diseases	143<br>(99.3 to 198)	162.02<br>(112.51 to 223.97)	167<br>(115 to 234)	172.92<br>(119.26 to 242.7)

Hubei	Gynecological diseases	107<br>(73.9 to 148)	192.24<br>(132.95 to 266.57)	124<br>(84.9 to 171)	223.83<br>(153.43 to 308.74)
Hunan	Gynecological diseases	110<br>(76.5 to 153)	177.13<br>(122.98 to 245.47)	132<br>(91.9 to 186)	196.63<br>(136.86 to 277.01)
Inner Mongolia	Gynecological diseases	37.6<br>(25.9 to 52.9)	165.31<br>(113.88 to 232.47)	49.5<br>(33.5 to 71.2)	191.16<br>(129.3 to 275.09)
Jiangsu	Gynecological diseases	128<br>(88.4 to 176)	185.34<br>(128.42 to 255.84)	162<br>(111 to 229)	197.82<br>(135.65 to 280.12)
Jiangxi	Gynecological diseases	53.5<br>(36.6 to 75.5)	135.42<br>(92.7 to 190.9)	75.2<br>(52.2 to 107)	158.11<br>(109.76 to 224.96)
Jilin	Gynecological diseases	49.7<br>(33.8 to 69.2)	191.39<br>(130.1 to 266.79)	61.2<br>(41.6 to 86.5)	219.48<br>(149.16 to 310.09)
Liaoning	Gynecological diseases	87.5<br>(60 to 121)	214.58<br>(147.06 to 297.78)	102<br>(69.6 to 146)	229.38<br>(155.81 to 327.28)
Ningxia	Gynecological diseases	8.17<br>(5.67 to 11.3)	165.5<br>(114.71 to 228.67)	13.9<br>(9.64 to 19.5)	197.47<br>(137.41 to 277.13)
Qinghai	Gynecological diseases	6.62<br>(4.54 to 9.25)	139.81<br>(95.74 to 195.36)	10.3<br>(7.13 to 14.7)	164.13<br>(113.41 to 234.13)
Shaanxi	Gynecological diseases	48<br>(32.8 to 67.4)	139.52<br>(95.24 to 195.98)	56.5<br>(38.4 to 82.1)	143.09<br>(97.07 to 207.79)
Shandong	Gynecological diseases	137<br>(95.4 to 192)	160.57<br>(111.48 to 223.95)	163<br>(111 to 235)	164.38<br>(112.05 to 236.01)
Shanghai	Gynecological diseases	32<br>(21.9 to 44.2)	229.29<br>(156.99 to 316.33)	69.1<br>(47.4 to 97.3)	247.47<br>(169.75 to 348.29)
Shanxi	Gynecological diseases	45.8<br>(31.7 to 63.6)	151.12<br>(104.69 to 209.89)	66.6<br>(45.7 to 94.8)	172.33<br>(118.4 to 245.34)
Sichuan	Gynecological diseases	213<br>(148 to 298)	190.84<br>(132.19 to 266.78)	167<br>(114 to 232)	199.54<br>(137.08 to 277.36)
Tianjin	Gynecological diseases	22.4<br>(15.3 to 31.2)	243.43<br>(166.48 to 338.94)	36.3<br>(24.9 to 50.7)	238.08<br>(163.18 to 332.32)
Tibet	Gynecological diseases	3.3<br>(2.26 to 4.71)	141.68<br>(96.79 to 202.26)	5.44<br>(3.77 to 7.85)	157.41<br>(109.14 to 227)
Xinjiang	Gynecological diseases	25<br>(17.2 to 34.5)	158.43<br>(109.2 to 219.03)	47.8<br>(32.9 to 67.9)	196.28<br>(134.82 to 278.76)
Yunnan	Gynecological diseases	60.8<br>(42.6 to 84.1)	156.13<br>(109.24 to 215.85)	93.7<br>(65.2 to 130)	190.65<br>(132.63 to 265.14)
Zhejiang	Gynecological diseases	92.3<br>(64.2 to 128)	209.29<br>(145.48 to 290.35)	149<br>(102 to 208)	242.82<br>(166.89 to 339.31)
China	Hemoglobinopathies and hemolytic anemias	2310<br>(1700 to 3020)	192.9<br>(142.02 to 252.25)	1230<br>(903 to 1690)	87.19<br>(63.9 to 119.66)
Taiwan	Hemoglobinopathies and hemolytic anemias	9.03<br>(6.94 to 12.1)	44.25<br>(34 to 59.38)	6.68<br>(5.09 to 9.35)	28.34<br>(21.6 to 39.63)
Hong Kong Special Administrative Region of China	Hemoglobinopathies and hemolytic anemias	3.04<br>(2.09 to 4.38)	52.66<br>(36.19 to 76.02)	2.95<br>(2 to 4.33)	39.22<br>(26.65 to 57.72)
Macau Special Administrative Region of China	Hemoglobinopathies and hemolytic anemias	0.313<br>(0.179 to 0.533)	88.32<br>(50.52 to 150.27)	0.317<br>(0.167 to 0.548)	46.32<br>(24.36 to 80.14)
Anhui	Hemoglobinopathies and hemolytic anemias	125<br>(87.1 to 171)	210.64<br>(147.01 to 288.12)	67.1<br>(41.6 to 102)	109.69<br>(68.08 to 167.44)
Beijing	Hemoglobinopathies and hemolytic anemias	14.5<br>(10 to 20.6)	129.32<br>(89.39 to 184.03)	14.2<br>(8.35 to 23.1)	59.01<br>(34.63 to 95.9)
Chongqing	Hemoglobinopathies and hemolytic anemias	14.9<br>(10.1 to 21.7)	94.72<br>(63.96 to 137.59)	9.74<br>(7.02 to 13.7)	36.46<br>(26.27 to 51.31)
Fujian	Hemoglobinopathies and hemolytic anemias	80.7<br>(55.6 to 114)	254.93<br>(175.76 to 360.12)	43.5<br>(26.6 to 71.5)	110.27<br>(67.44 to 181.44)
Gansu	Hemoglobinopathies and hemolytic anemias	61.3<br>(45 to 80.2)	258.98<br>(190.22 to 338.9)	33<br>(21.7 to 49.3)	124.19<br>(81.67 to 185.46)
Guangdong	Hemoglobinopathies and hemolytic anemias	139<br>(96.8 to 195)	211.84<br>(147.14 to 296.96)	92<br>(57.6 to 148)	78.77<br>(49.28 to 126.74)
Guangxi	Hemoglobinopathies and hemolytic anemias	126<br>(89.8 to 167)	288.95<br>(206.55 to 383.35)	65.8<br>(43.8 to 98.6)	136.23<br>(90.66 to 204.07)
Guizhou	Hemoglobinopathies and hemolytic anemias	105<br>(69.9 to 151)	317.08<br>(210.47 to 455.28)	51.5<br>(33 to 77.5)	152.12<br>(97.51 to 229.2)
Hainan	Hemoglobinopathies and hemolytic anemias	13.4<br>(9.67 to 17.8)	192.17<br>(138.84 to 256.15)	9.37<br>(6.1 to 14.8)	97.77<br>(63.57 to 153.84)
Hebei	Hemoglobinopathies and hemolytic anemias	105<br>(80.4 to 138)	165.13<br>(126.24 to 216.19)	76.7<br>(52.3 to 113)	100.39<br>(68.48 to 148.34)

Heilongjiang	Hemoglobinopathies and hemolytic anemias	66.4 (45.3 to 88.8)	182.14 (124.15 to 243.7)	41.4 (26.2 to 62.9)	105.43 (66.72 to 160.2)
Henan	Hemoglobinopathies and hemolytic anemias	167 (118 to 227)	189.58 (134.14 to 257.45)	88.5 (57.4 to 131)	91.86 (59.55 to 135.81)
Hubei	Hemoglobinopathies and hemolytic anemias	69.3 (47.2 to 93.2)	124.74 (84.9 to 167.76)	33.7 (22.2 to 49.7)	60.93 (40.23 to 89.89)
Hunan	Hemoglobinopathies and hemolytic anemias	121 (85 to 160)	193.97 (136.65 to 256.51)	60.8 (42.4 to 87.9)	90.57 (63.21 to 130.94)
Inner Mongolia	Hemoglobinopathies and hemolytic anemias	48.6 (34.4 to 65.8)	213.72 (151.07 to 289.26)	26 (16.4 to 40.3)	100.26 (63.45 to 155.58)
Jiangsu	Hemoglobinopathies and hemolytic anemias	81.6 (58 to 110)	118.55 (84.28 to 159.58)	47.2 (31.3 to 72.4)	57.76 (38.3 to 88.65)
Jiangxi	Hemoglobinopathies and hemolytic anemias	102 (73.8 to 134)	258.74 (186.64 to 338.69)	49.7 (32.9 to 72.1)	104.63 (69.2 to 151.77)
Jilin	Hemoglobinopathies and hemolytic anemias	56.6 (36.5 to 75.2)	218.23 (140.65 to 289.69)	31.6 (19.5 to 48.3)	113.41 (69.83 to 173.1)
Liaoning	Hemoglobinopathies and hemolytic anemias	31.9 (24.2 to 39.8)	78.13 (59.34 to 97.46)	18.3 (13 to 24.4)	40.94 (29.19 to 54.59)
Ningxia	Hemoglobinopathies and hemolytic anemias	11.9 (8.36 to 15.7)	240.25 (169.26 to 318.11)	7.52 (4.75 to 11.9)	107.19 (67.73 to 169.93)
Qinghai	Hemoglobinopathies and hemolytic anemias	11.5 (8.09 to 15.9)	241.82 (170.85 to 335.45)	7.12 (4.65 to 10.4)	113.32 (73.99 to 165.98)
Shaanxi	Hemoglobinopathies and hemolytic anemias	72 (51.6 to 96.6)	209.32 (149.84 to 280.63)	34.6 (22.5 to 53.8)	87.66 (56.9 to 136.21)
Shandong	Hemoglobinopathies and hemolytic anemias	70.2 (53.3 to 88.1)	81.99 (62.29 to 102.97)	36.5 (27.6 to 49)	36.74 (27.79 to 49.33)
Shanghai	Hemoglobinopathies and hemolytic anemias	10.4 (7.5 to 14)	74.19 (53.71 to 100.22)	7.83 (5.29 to 11.6)	28.03 (18.94 to 41.54)
Shanxi	Hemoglobinopathies and hemolytic anemias	29.4 (22.9 to 37.4)	96.94 (75.59 to 123.35)	15.2 (11.4 to 21.1)	39.33 (29.6 to 54.6)
Sichuan	Hemoglobinopathies and hemolytic anemias	333 (230 to 467)	298.36 (205.97 to 418.51)	107 (68.3 to 162)	128.25 (81.84 to 194.11)
Tianjin	Hemoglobinopathies and hemolytic anemias	13.5 (9.74 to 18.1)	146.36 (105.93 to 196.83)	12.6 (7.85 to 19.9)	82.46 (51.45 to 130.11)
Tibet	Hemoglobinopathies and hemolytic anemias	6.75 (4.66 to 9.49)	289.82 (199.83 to 407.27)	5.17 (3.54 to 7.71)	149.59 (102.41 to 222.97)
Xinjiang	Hemoglobinopathies and hemolytic anemias	19.2 (13.4 to 29.9)	121.83 (85.01 to 189.87)	15 (10 to 21.7)	61.56 (41.03 to 88.96)
Yunnan	Hemoglobinopathies and hemolytic anemias	140 (95.5 to 198)	358.99 (245.04 to 507.63)	83.2 (54.8 to 126)	169.2 (111.43 to 255.62)
Zhejiang	Hemoglobinopathies and hemolytic anemias	58.4 (42.8 to 77.8)	132.5 (97.09 to 176.37)	36.4 (23.5 to 55.5)	59.45 (38.37 to 90.78)
China	Endocrine, metabolic, blood, and immune disorders	1120 (876 to 1350)	93.46 (73.19 to 113.09)	778 (625 to 925)	55.07 (44.26 to 65.48)
Taiwan	Endocrine, metabolic, blood, and immune disorders	16 (12.7 to 18.7)	78.37 (62.22 to 91.78)	19.1 (16.4 to 23.2)	80.8 (69.58 to 98.49)
Hong Kong Special Administrative Region of China	Endocrine, metabolic, blood, and immune disorders	2.52 (1.96 to 3.33)	43.77 (33.96 to 57.85)	3.42 (2.68 to 4.58)	45.56 (35.62 to 60.93)
Macau Special Administrative Region of China	Endocrine, metabolic, blood, and immune disorders	0.187 (0.141 to 0.24)	52.62 (39.68 to 67.75)	0.309 (0.233 to 0.386)	45.14 (34.09 to 56.39)
Anhui	Endocrine, metabolic, blood, and immune disorders	57.5 (44.1 to 78.9)	97.1 (74.41 to 133.13)	31 (24.8 to 39)	50.62 (40.57 to 63.83)
Beijing	Endocrine, metabolic, blood, and immune disorders	6.87 (5.41 to 9.06)	61.23 (48.24 to 80.76)	10.4 (8.12 to 13.5)	43.03 (33.7 to 56.02)
Chongqing	Endocrine, metabolic, blood, and immune disorders	26.8 (12.3 to 35.7)	169.85 (78.19 to 226.04)	26 (12.4 to 34.6)	97.21 (46.26 to 129.61)
Fujian	Endocrine, metabolic, blood, and immune disorders	21.2 (17.3 to 25.9)	67.03 (54.67 to 81.75)	17 (13.3 to 21.3)	43.07 (33.74 to 54)
Gansu	Endocrine, metabolic, blood, and immune disorders	27.9 (18.4 to 40.2)	117.75 (77.71 to 169.78)	15.2 (12 to 18.8)	57.32 (45.12 to 70.66)

Guangdong	Endocrine, metabolic, blood, and immune disorders	47<br>(37 to 56.9)	71.45<br>(56.2 to 86.39)	54.2<br>(36.6 to 67.1)	46.38<br>(31.3 to 57.4)
Guangxi	Endocrine, metabolic, blood, and immune disorders	38.2<br>(28 to 46.8)	87.92<br>(64.39 to 107.53)	29<br>(20.3 to 36.1)	60.01<br>(42.1 to 74.62)
Guizhou	Endocrine, metabolic, blood, and immune disorders	52.1<br>(26 to 67.6)	157.08<br>(78.47 to 203.59)	24.6<br>(15.2 to 31.6)	72.81<br>(45.04 to 93.53)
Hainan	Endocrine, metabolic, blood, and immune disorders	7.4<br>(4.8 to 9.61)	106.17<br>(68.84 to 137.89)	5.33<br>(3.25 to 6.96)	55.63<br>(33.88 to 72.58)
Hebei	Endocrine, metabolic, blood, and immune disorders	53.9<br>(43.8 to 66)	84.75<br>(68.82 to 103.64)	49.3<br>(36.8 to 59.9)	64.52<br>(48.09 to 78.43)
Heilongjiang	Endocrine, metabolic, blood, and immune disorders	24.6<br>(17.2 to 38.2)	67.44<br>(47.16 to 104.87)	19<br>(13.9 to 27)	48.45<br>(35.38 to 68.74)
Henan	Endocrine, metabolic, blood, and immune disorders	71.7<br>(55 to 101)	81.18<br>(62.31 to 114.09)	47.2<br>(38.4 to 58.4)	48.97<br>(39.83 to 60.68)
Hubei	Endocrine, metabolic, blood, and immune disorders	50.3<br>(37.5 to 68.7)	90.57<br>(67.53 to 123.67)	27.8<br>(22.1 to 35.6)	50.23<br>(39.88 to 64.41)
Hunan	Endocrine, metabolic, blood, and immune disorders	60.9<br>(41.8 to 74.2)	97.98<br>(67.25 to 119.39)	40.5<br>(28.4 to 49.5)	60.31<br>(42.24 to 73.66)
Inner Mongolia	Endocrine, metabolic, blood, and immune disorders	19.9<br>(13.8 to 24.9)	87.72<br>(60.85 to 109.49)	14.9<br>(11.5 to 18.4)	57.64<br>(44.51 to 71.11)
Jiangsu	Endocrine, metabolic, blood, and immune disorders	56.5<br>(41.4 to 77.4)	82.08<br>(60.16 to 112.41)	35.2<br>(26.9 to 48.4)	43.16<br>(32.96 to 59.31)
Jiangxi	Endocrine, metabolic, blood, and immune disorders	54.3<br>(28.9 to 68.4)	137.23<br>(73.2 to 173.09)	33.9<br>(21.8 to 42.8)	71.41<br>(45.94 to 89.95)
Jilin	Endocrine, metabolic, blood, and immune disorders	19.4<br>(14.8 to 26.5)	74.7<br>(57.06 to 101.97)	14<br>(11.3 to 17.6)	50.24<br>(40.47 to 63.01)
Liaoning	Endocrine, metabolic, blood, and immune disorders	25.7<br>(18.9 to 35.9)	62.99<br>(46.36 to 88.1)	22.6<br>(17.4 to 29.7)	50.56<br>(38.99 to 66.55)
Ningxia	Endocrine, metabolic, blood, and immune disorders	4.39<br>(3.23 to 7.91)	88.89<br>(65.32 to 160.2)	3.2<br>(2.48 to 4.26)	45.55<br>(35.39 to 60.66)
Qinghai	Endocrine, metabolic, blood, and immune disorders	5.47<br>(3.45 to 7.6)	115.59<br>(72.77 to 160.48)	4.02<br>(2.91 to 4.99)	63.91<br>(46.31 to 79.47)
Shaanxi	Endocrine, metabolic, blood, and immune disorders	37<br>(27.9 to 45.5)	107.61<br>(81.17 to 132.29)	25.6<br>(18 to 32.5)	64.79<br>(45.68 to 82.18)
Shandong	Endocrine, metabolic, blood, and immune disorders	60.6<br>(43.6 to 89.3)	70.77<br>(50.89 to 104.34)	44.8<br>(34.5 to 60.3)	45.04<br>(34.68 to 60.67)
Shanghai	Endocrine, metabolic, blood, and immune disorders	11.8<br>(8.85 to 15.5)	84.55<br>(63.32 to 110.75)	12.5<br>(9.48 to 16.5)	44.91<br>(33.93 to 59.12)
Shanxi	Endocrine, metabolic, blood, and immune disorders	28.7<br>(22.6 to 38.6)	94.75<br>(74.58 to 127.47)	20.9<br>(16.8 to 26.2)	54.03<br>(43.4 to 67.9)
Sichuan	Endocrine, metabolic, blood, and immune disorders	118<br>(78.4 to 149)	105.98<br>(70.22 to 133.35)	50.4<br>(36.6 to 62.2)	60.36<br>(43.87 to 74.55)
Tianjin	Endocrine, metabolic, blood, and immune disorders	5.17<br>(3.66 to 8.1)	56.21<br>(39.79 to 88.05)	6.16<br>(4.5 to 9.06)	40.39<br>(29.45 to 59.36)
Tibet	Endocrine, metabolic, blood, and immune disorders	4.43<br>(1.62 to 5.94)	190.04<br>(69.43 to 255)	4.36<br>(1.9 to 5.94)	126.02<br>(55.11 to 171.99)

Xinjiang	Endocrine, metabolic, blood, and immune disorders	19<br>(11.7 to 26.4)	120.34<br>(74.19 to 167.08)	17.9<br>(12.8 to 23)	73.32<br>(52.66 to 94.53)
Yunnan	Endocrine, metabolic, blood, and immune disorders	60.8<br>(23.2 to 80.9)	155.96<br>(59.41 to 207.49)	40.5<br>(22 to 52.1)	82.4<br>(44.72 to 105.87)
Zhejiang	Endocrine, metabolic, blood, and immune disorders	38.1<br>(29.2 to 49.8)	86.34<br>(66.32 to 112.86)	26.7<br>(20.9 to 35)	43.65<br>(34.09 to 57.24)
China	Musculoskeletal disorders	16100<br>(11800 to 21300)	1344.6<br>(983.02 to 1781.4)	25500<br>(18600 to 33800)	1805.35<br>(1317.76 to 2395.03)
Taiwan	Musculoskeletal disorders	302<br>(222 to 398)	1479.63<br>(1087.14 to 1952.65)	502<br>(372 to 661)	2130.1<br>(1575.38 to 2802.29)
Hong Kong Special Administrative Region of China	Musculoskeletal disorders	89.5<br>(64.9 to 120)	1552.38<br>(1126.36 to 2075.69)	153<br>(112 to 203)	2033.23<br>(1486.88 to 2705.77)
Macau Special Administrative Region of China	Musculoskeletal disorders	5.09<br>(3.69 to 6.75)	1433.57<br>(1040.68 to 1902.65)	13<br>(9.47 to 17.3)	1898.13<br>(1384.15 to 2528.71)
Anhui	Musculoskeletal disorders	779<br>(573 to 1030)	1315.24<br>(966.38 to 1745.56)	1160<br>(847 to 1550)	1899.18<br>(1384.58 to 2542.51)
Beijing	Musculoskeletal disorders	191<br>(138 to 255)	1700.13<br>(1234.6 to 2272.02)	490<br>(358 to 650)	2033.4<br>(1483.9 to 2697.16)
Chongqing	Musculoskeletal disorders	235<br>(173 to 313)	1488.57<br>(1094.53 to 1982.18)	535<br>(391 to 708)	2002.06<br>(1464.46 to 2647.34)
Fujian	Musculoskeletal disorders	392<br>(288 to 518)	1237.98<br>(909.34 to 1637.69)	683<br>(498 to 916)	1734.14<br>(1262.76 to 2325.43)
Gansu	Musculoskeletal disorders	310<br>(226 to 410)	1308.76<br>(955.72 to 1733.26)	498<br>(361 to 663)	1873.96<br>(1359.36 to 2493.45)
Guangdong	Musculoskeletal disorders	803<br>(589 to 1070)	1220.23<br>(894.24 to 1625.04)	1850<br>(1340 to 2460)	1583.62<br>(1150.22 to 2104.63)
Guangxi	Musculoskeletal disorders	580<br>(428 to 766)	1334.03<br>(984.36 to 1761.27)	864<br>(635 to 1140)	1787.81<br>(1314.58 to 2360.82)
Guizhou	Musculoskeletal disorders	435<br>(321 to 571)	1310.27<br>(966.52 to 1719.92)	613<br>(448 to 810)	1811.56<br>(1325.82 to 2393.94)
Hainan	Musculoskeletal disorders	86.2<br>(63.4 to 114)	1237.91<br>(910.76 to 1633.13)	161<br>(118 to 215)	1676.94<br>(1228 to 2238.53)
Hebei	Musculoskeletal disorders	867<br>(633 to 1150)	1361.74<br>(993.97 to 1811.65)	1340<br>(979 to 1800)	1757.79<br>(1281.52 to 2358.99)
Heilongjiang	Musculoskeletal disorders	494<br>(362 to 657)	1354.75<br>(992.76 to 1801.71)	844<br>(614 to 1120)	2149.83<br>(1563.41 to 2863.25)
Henan	Musculoskeletal disorders	1190<br>(866 to 1580)	1351.01<br>(980.81 to 1795.11)	1690<br>(1240 to 2250)	1758.36<br>(1285.08 to 2336.15)
Hubei	Musculoskeletal disorders	683<br>(496 to 909)	1228.93<br>(892.6 to 1635.95)	1050<br>(764 to 1380)	1893.63<br>(1381.1 to 2500.49)
Hunan	Musculoskeletal disorders	873<br>(638 to 1150)	1403.46<br>(1025.36 to 1854.84)	1270<br>(931 to 1680)	1890.26<br>(1385.9 to 2499.68)
Inner Mongolia	Musculoskeletal disorders	292<br>(213 to 388)	1284.57<br>(935.87 to 1707.45)	507<br>(370 to 677)	1957.24<br>(1427.88 to 2613.85)
Jiangsu	Musculoskeletal disorders	1040<br>(763 to 1380)	1516.04<br>(1108.2 to 2010.88)	1580<br>(1150 to 2090)	1932.94<br>(1405.09 to 2562.27)
Jiangxi	Musculoskeletal disorders	500<br>(366 to 659)	1265.77<br>(925.52 to 1666.59)	795<br>(580 to 1060)	1672.28<br>(1219.21 to 2227.75)
Jilin	Musculoskeletal disorders	320<br>(234 to 426)	1233.51<br>(900.79 to 1639.85)	547<br>(398 to 732)	1960.09<br>(1427.62 to 2625.77)
Liaoning	Musculoskeletal disorders	591<br>(432 to 791)	1449.02<br>(1059.52 to 1939.31)	939<br>(687 to 1250)	2101.97<br>(1538.76 to 2798.05)
Ningxia	Musculoskeletal disorders	57.2<br>(41.6 to 75.9)	1159.03<br>(842.53 to 1537.27)	120<br>(87.4 to 160)	1711.59<br>(1244.77 to 2285.16)
Qinghai	Musculoskeletal disorders	58<br>(42.3 to 76.6)	1224.82<br>(892.8 to 1617.23)	111<br>(81.6 to 147)	1761.26<br>(1298.56 to 2339.34)
Shaanxi	Musculoskeletal disorders	420<br>(304 to 561)	1219.38<br>(882.92 to 1629.51)	699<br>(509 to 927)	1769.03<br>(1288.61 to 2346.39)
Shandong	Musculoskeletal disorders	1110<br>(804 to 1480)	1302.38<br>(939.1 to 1732.29)	1760<br>(1280 to 2340)	1773.18<br>(1287.81 to 2355.43)
Shanghai	Musculoskeletal disorders	236<br>(169 to 314)	1688.2<br>(1207.99 to 2249.82)	470<br>(339 to 634)	1681.43<br>(1214.83 to 2267.58)
Shanxi	Musculoskeletal disorders	406<br>(292 to 539)	1341.28<br>(963.84 to 1779.51)	696<br>(507 to 928)	1802.64<br>(1313.71 to 2403.32)

Sichuan	Musculoskeletal disorders	1610 (1170 to 2140)	1438.35 (1047.95 to 1913.97)	1620 (1190 to 2140)	1946.12 (1423.31 to 2567.64)
Tianjin	Musculoskeletal disorders	138 (100 to 185)	1504.82 (1089.79 to 2010.35)	288 (210 to 385)	1885.77 (1376.81 to 2519.47)
Tibet	Musculoskeletal disorders	29 (21.1 to 37.9)	1243.4 (904.75 to 1626.42)	51.3 (37.5 to 67.7)	1483.53 (1084.2 to 1958.93)
Xinjiang	Musculoskeletal disorders	191 (140 to 256)	1213.26 (887.34 to 1622.63)	417 (303 to 555)	1709.84 (1242.78 to 2276.97)
Yunnan	Musculoskeletal disorders	474 (349 to 631)	1217.45 (895.42 to 1619.19)	791 (577 to 1050)	1608.31 (1174.34 to 2144.56)
Zhejiang	Musculoskeletal disorders	604 (418 to 825)	1369.08 (948.98 to 1870.72)	887 (644 to 1190)	1449.72 (1052.14 to 1943.48)
China	Rheumatoid arthritis	334 (262 to 415)	27.93 (21.92 to 34.64)	679 (509 to 861)	48.05 (36.04 to 60.94)
Taiwan	Rheumatoid arthritis	5.6 (4.38 to 7.06)	27.44 (21.45 to 34.58)	9.62 (7.16 to 12.4)	40.8 (30.35 to 52.44)
Hong Kong Special Administrative Region of China	Rheumatoid arthritis	2.08 (1.49 to 2.75)	36.14 (25.77 to 47.68)	4.63 (3.34 to 6.15)	61.7 (44.49 to 81.93)
Macau Special Administrative Region of China	Rheumatoid arthritis	0.0889 (0.0646 to 0.116)	25.06 (18.21 to 32.7)	0.301 (0.21 to 0.406)	44 (30.65 to 59.4)
Anhui	Rheumatoid arthritis	13.9 (10.4 to 18.8)	23.44 (17.49 to 31.77)	27 (20 to 34.7)	44.23 (32.65 to 56.8)
Beijing	Rheumatoid arthritis	3.58 (2.68 to 4.58)	31.88 (23.89 to 40.8)	10.3 (7.29 to 13.6)	42.56 (30.25 to 56.49)
Chongqing	Rheumatoid arthritis	4.89 (3.86 to 6.07)	30.98 (24.47 to 38.49)	14.4 (10.8 to 18.3)	53.83 (40.53 to 68.51)
Fujian	Rheumatoid arthritis	8.36 (6.54 to 10.5)	26.41 (20.68 to 33.09)	18.1 (13.4 to 23.2)	46.05 (34.04 to 58.99)
Gansu	Rheumatoid arthritis	6.53 (5.19 to 8.11)	27.6 (21.95 to 34.27)	12.9 (9.82 to 16.5)	48.37 (36.96 to 62.03)
Guangdong	Rheumatoid arthritis	14.6 (11.3 to 18.4)	22.22 (17.17 to 27.92)	39 (28.5 to 50.9)	33.38 (24.36 to 43.58)
Guangxi	Rheumatoid arthritis	18.8 (13.6 to 22.7)	43.15 (31.17 to 52.11)	31.5 (21.9 to 39.1)	65.26 (45.31 to 80.98)
Guizhou	Rheumatoid arthritis	11 (8.77 to 13.6)	33.22 (26.42 to 40.92)	19.2 (14.6 to 23.8)	56.89 (43.23 to 70.4)
Hainan	Rheumatoid arthritis	3.3 (2.17 to 4.24)	47.43 (31.21 to 60.92)	5.44 (3.79 to 6.76)	56.72 (39.51 to 70.56)
Hebei	Rheumatoid arthritis	11.8 (8.35 to 16.1)	18.57 (13.12 to 25.26)	27 (18.6 to 35.6)	35.27 (24.4 to 46.52)
Heilongjiang	Rheumatoid arthritis	6.86 (4.95 to 9.17)	18.82 (13.59 to 25.16)	16.4 (11.6 to 22)	41.83 (29.65 to 56.09)
Henan	Rheumatoid arthritis	19.9 (14.1 to 26.5)	22.51 (16.02 to 29.98)	38.6 (27.4 to 50.5)	40.13 (28.45 to 52.4)
Hubei	Rheumatoid arthritis	15.1 (11.5 to 18.9)	27.15 (20.78 to 34.08)	32.1 (24.1 to 40.6)	58.13 (43.58 to 73.33)
Hunan	Rheumatoid arthritis	23.4 (18.5 to 28.6)	37.59 (29.8 to 46.05)	40.5 (30.8 to 50.1)	60.31 (45.84 to 74.61)
Inner Mongolia	Rheumatoid arthritis	5.36 (4.1 to 6.74)	23.58 (18.05 to 29.66)	12.4 (9.09 to 16.1)	47.83 (35.12 to 62.1)
Jiangsu	Rheumatoid arthritis	22.8 (17.7 to 28.4)	33.16 (25.67 to 41.34)	51.2 (38.4 to 64.7)	62.68 (47.02 to 79.2)
Jiangxi	Rheumatoid arthritis	13.7 (10.6 to 16.8)	34.56 (26.93 to 42.58)	22.9 (17.1 to 28.9)	48.21 (36.02 to 60.83)
Jilin	Rheumatoid arthritis	5.21 (3.86 to 6.99)	20.09 (14.89 to 26.95)	11.9 (8.39 to 15.8)	42.8 (30.07 to 56.58)
Liaoning	Rheumatoid arthritis	10.1 (7.21 to 13.4)	24.65 (17.68 to 32.78)	22.1 (15.8 to 28.9)	49.43 (35.35 to 64.81)
Ningxia	Rheumatoid arthritis	1.03 (0.778 to 1.35)	20.93 (15.76 to 27.3)	2.67 (1.94 to 3.47)	38.07 (27.58 to 49.4)
Qinghai	Rheumatoid arthritis	1.53 (1.19 to 1.88)	32.29 (25.2 to 39.77)	3.25 (2.46 to 4.17)	51.79 (39.19 to 66.35)
Shaanxi	Rheumatoid arthritis	6.15 (4.4 to 8.33)	17.88 (12.78 to 24.2)	16.3 (11.9 to 21)	41.34 (30.12 to 53.23)
Shandong	Rheumatoid arthritis	19.5 (14.2 to 25.7)	22.77 (16.6 to 30.01)	42.3 (29.8 to 55.9)	42.53 (29.98 to 56.23)

Shanghai	Rheumatoid arthritis	5.47<br>(4.09 to 6.98)	39.14<br>(29.3 to 49.94)	13.3<br>(9.6 to 17.6)	47.66<br>(34.37 to 63.14)
Shanxi	Rheumatoid arthritis	7.1<br>(5.27 to 9.17)	23.44<br>(17.41 to 30.29)	15.2<br>(10.9 to 20)	39.25<br>(28.09 to 51.71)
Sichuan	Rheumatoid arthritis	27.7<br>(20.7 to 35.7)	24.81<br>(18.57 to 32.01)	45<br>(34.1 to 55.9)	53.9<br>(40.87 to 67)
Tianjin	Rheumatoid arthritis	2.17<br>(1.56 to 2.87)	23.55<br>(16.92 to 31.16)	5.77<br>(4.03 to 7.69)	37.81<br>(26.38 to 50.36)
Tibet	Rheumatoid arthritis	0.884<br>(0.557 to 1.23)	37.96<br>(23.9 to 52.87)	1.32<br>(0.979 to 1.7)	38.1<br>(28.33 to 49.12)
Xinjiang	Rheumatoid arthritis	3.35<br>(2.58 to 4.32)	21.24<br>(16.33 to 27.41)	8.7<br>(6.23 to 11.4)	35.72<br>(25.59 to 46.91)
Yunnan	Rheumatoid arthritis	22.3<br>(15 to 27.5)	57.26<br>(38.58 to 70.52)	35.3<br>(24.1 to 43.9)	71.76<br>(49.01 to 89.35)
Zhejiang	Rheumatoid arthritis	15.9<br>(12.5 to 19.5)	35.97<br>(28.28 to 44.21)	31.7<br>(23.7 to 40.5)	51.77<br>(38.74 to 66.2)
China	Osteoarthritis	841<br>(415 to 1690)	70.26<br>(34.67 to 141.1)	1970<br>(984 to 3940)	139.6<br>(69.66 to 278.63)
Taiwan	Osteoarthritis	11.4<br>(5.63 to 22.6)	55.7<br>(27.6 to 110.97)	25.3<br>(12.7 to 50.7)	107.14<br>(54.03 to 215.14)
Hong Kong Special Administrative Region of China	Osteoarthritis	5.15<br>(2.55 to 10.2)	89.42<br>(44.22 to 177.76)	13.5<br>(6.74 to 26.8)	180.05<br>(89.77 to 357.14)
Macau Special Administrative Region of China	Osteoarthritis	0.245<br>(0.121 to 0.489)	68.99<br>(34.08 to 137.83)	1.03<br>(0.515 to 2.05)	150.95<br>(75.31 to 299.7)
Anhui	Osteoarthritis	31.8<br>(15.8 to 63.1)	53.7<br>(26.74 to 106.42)	72.1<br>(35.3 to 144)	117.86<br>(57.74 to 236.24)
Beijing	Osteoarthritis	8.49<br>(4.2 to 16.9)	75.67<br>(37.48 to 150.68)	29.1<br>(14.5 to 58.1)	120.54<br>(60.25 to 241.01)
Chongqing	Osteoarthritis	15<br>(7.51 to 29.7)	95.25<br>(47.56 to 188.39)	54.3<br>(27.3 to 109)	203.05<br>(102.14 to 409.07)
Fujian	Osteoarthritis	15.6<br>(7.63 to 31)	49.4<br>(24.12 to 97.91)	41<br>(20.3 to 82)	103.92<br>(51.47 to 208.06)
Gansu	Osteoarthritis	15.5<br>(7.56 to 31)	65.42<br>(31.95 to 130.94)	38.6<br>(19.4 to 77.1)	145.26<br>(73.04 to 290.27)
Guangdong	Osteoarthritis	56.2<br>(27.7 to 114)	85.46<br>(42.15 to 172.89)	166<br>(82.2 to 329)	141.76<br>(70.36 to 281.47)
Guangxi	Osteoarthritis	26.1<br>(13 to 52)	60.1<br>(29.83 to 119.47)	55.6<br>(27.5 to 111)	114.98<br>(56.86 to 230.29)
Guizhou	Osteoarthritis	25.8<br>(12.8 to 51.3)	77.69<br>(38.53 to 154.49)	56<br>(28.2 to 111)	165.6<br>(83.37 to 327.34)
Hainan	Osteoarthritis	3.77<br>(1.87 to 7.47)	54.18<br>(26.79 to 107.3)	10.2<br>(5.08 to 20.4)	106.42<br>(52.94 to 213.07)
Hebei	Osteoarthritis	38<br>(18.8 to 76.1)	59.71<br>(29.6 to 119.62)	86.2<br>(42.2 to 170)	112.82<br>(55.19 to 222.35)
Heilongjiang	Osteoarthritis	25.9<br>(12.8 to 52.4)	71.13<br>(35.16 to 143.86)	73.7<br>(36.8 to 145)	187.77<br>(93.87 to 370.11)
Henan	Osteoarthritis	57.7<br>(28.6 to 115)	65.38<br>(32.42 to 130.44)	117<br>(57.6 to 234)	121.07<br>(59.85 to 243.43)
Hubei	Osteoarthritis	35.3<br>(17.5 to 70.5)	63.47<br>(31.57 to 126.84)	78.5<br>(39.4 to 154)	141.88<br>(71.33 to 278.77)
Hunan	Osteoarthritis	40<br>(19.8 to 79.4)	64.38<br>(31.77 to 127.66)	87.2<br>(44 to 174)	129.92<br>(65.5 to 259.03)
Inner Mongolia	Osteoarthritis	12.8<br>(6.2 to 25.2)	56.08<br>(27.28 to 110.86)	36.1<br>(17.9 to 71.6)	139.38<br>(69.06 to 276.45)
Jiangsu	Osteoarthritis	67.4<br>(33.7 to 136)	97.94<br>(49.03 to 197.16)	147<br>(71.8 to 295)	179.59<br>(87.88 to 361.1)
Jiangxi	Osteoarthritis	19<br>(9.48 to 38)	48.17<br>(23.98 to 96.07)	46.2<br>(23 to 91.9)	97.23<br>(48.42 to 193.38)
Jilin	Osteoarthritis	14.6<br>(7.29 to 28.9)	56.26<br>(28.09 to 111.51)	40.1<br>(19.9 to 80.1)	143.78<br>(71.35 to 287.15)
Liaoning	Osteoarthritis	27.1<br>(13.5 to 54.7)	66.38<br>(33.04 to 134.17)	68.5<br>(34.3 to 136)	153.45<br>(76.72 to 305.09)
Ningxia	Osteoarthritis	2.24<br>(1.1 to 4.46)	45.3<br>(22.37 to 90.4)	7.67<br>(3.83 to 15.4)	109.24<br>(54.6 to 219.75)
Qinghai	Osteoarthritis	2.73<br>(1.36 to 5.44)	57.71<br>(28.65 to 114.86)	7.8<br>(3.82 to 15.7)	124.1<br>(60.81 to 250.16)

Shaanxi	Osteoarthritis	26<br>(13 to 52.1)	75.64<br>(37.64 to 151.43)	61.3<br>(31 to 124)	155.22<br>(78.44 to 313.3)
Shandong	Osteoarthritis	53.8<br>(26.4 to 108)	62.91<br>(30.8 to 125.94)	125<br>(61.7 to 250)	125.38<br>(62.1 to 251.58)
Shanghai	Osteoarthritis	11.8<br>(5.82 to 23.3)	84.11<br>(41.66 to 166.72)	33.8<br>(16.7 to 67.2)	120.81<br>(59.78 to 240.65)
Shanxi	Osteoarthritis	25.3<br>(12.6 to 50)	83.4<br>(41.46 to 164.98)	60.2<br>(29.9 to 119)	155.79<br>(77.29 to 308.96)
Sichuan	Osteoarthritis	103<br>(51.2 to 205)	92.27<br>(45.91 to 183.47)	164<br>(83.2 to 326)	196.8<br>(99.69 to 390.78)
Tianjin	Osteoarthritis	6.26<br>(3.09 to 12.4)	68.1<br>(33.57 to 134.64)	17.8<br>(8.83 to 35.2)	116.9<br>(57.83 to 230.76)
Tibet	Osteoarthritis	1.2<br>(0.596 to 2.4)	51.62<br>(25.57 to 103.15)	2.61<br>(1.3 to 5.22)	75.62<br>(37.54 to 150.89)
Xinjiang	Osteoarthritis	9.73<br>(4.84 to 19.4)	61.66<br>(30.67 to 123.23)	30.7<br>(15.3 to 62)	125.87<br>(62.9 to 254.43)
Yunnan	Osteoarthritis	30.2<br>(15.1 to 60.6)	77.53<br>(38.72 to 155.4)	73.6<br>(37.1 to 148)	149.77<br>(75.39 to 300.63)
Zhejiang	Osteoarthritis	27.2<br>(13.5 to 54)	61.59<br>(30.58 to 122.36)	70.7<br>(34.8 to 140)	115.48<br>(56.85 to 228.88)
China	Low back pain	5830<br>(4120 to 7900)	487.12<br>(344.61 to 659.64)	7180<br>(5080 to 9740)	508.65<br>(359.73 to 689.23)
Taiwan	Low back pain	140<br>(99.5 to 190)	683.97<br>(487.65 to 931.2)	216<br>(154 to 291)	914.8<br>(652.39 to 1235.93)
Hong Kong Special Administrative Region of China	Low back pain	37.3<br>(26.3 to 50.7)	647<br>(456.4 to 880.44)	56.1<br>(40.2 to 76.4)	747.05<br>(535.28 to 1016.79)
Macau Special Administrative Region of China	Low back pain	1.92<br>(1.36 to 2.65)	541.21<br>(381.9 to 746.87)	4.07<br>(2.88 to 5.56)	595.11<br>(421.25 to 813.32)
Anhui	Low back pain	301<br>(214 to 407)	507.65<br>(360.48 to 686.57)	349<br>(249 to 475)	569.93<br>(406.54 to 777.25)
Beijing	Low back pain	79.4<br>(56.6 to 107)	708.07<br>(504.44 to 957.72)	191<br>(138 to 259)	792.86<br>(571.25 to 1073.6)
Chongqing	Low back pain	86.9<br>(61.8 to 119)	550.49<br>(391.38 to 753.93)	158<br>(113 to 215)	589.99<br>(420.93 to 804.17)
Fujian	Low back pain	150<br>(107 to 205)	474.57<br>(339.42 to 648.51)	208<br>(149 to 283)	527.87<br>(377.59 to 718.28)
Gansu	Low back pain	116<br>(82.7 to 158)	490.61<br>(349.79 to 670.06)	146<br>(104 to 200)	549.21<br>(391.16 to 753.5)
Guangdong	Low back pain	253<br>(171 to 355)	383.8<br>(259.75 to 539.12)	477<br>(340 to 654)	408.01<br>(290.73 to 559.71)
Guangxi	Low back pain	212<br>(151 to 288)	488.3<br>(347.15 to 662.31)	250<br>(178 to 339)	516.56<br>(368.18 to 700.8)
Guizhou	Low back pain	163<br>(115 to 222)	491.95<br>(345.79 to 669.12)	186<br>(132 to 250)	548.56<br>(390.61 to 740.48)
Hainan	Low back pain	31.6<br>(22.5 to 43.3)	454.03<br>(322.71 to 620.95)	47.4<br>(33.8 to 64.2)	494.54<br>(352.4 to 669.16)
Hebei	Low back pain	334<br>(237 to 453)	524.69<br>(371.9 to 711.82)	409<br>(291 to 553)	534.68<br>(381.24 to 722.98)
Heilongjiang	Low back pain	184<br>(131 to 252)	505.14<br>(360.31 to 691.18)	241<br>(171 to 329)	615.1<br>(436.52 to 837.36)
Henan	Low back pain	462<br>(326 to 629)	522.85<br>(369.72 to 712.31)	509<br>(359 to 692)	528.04<br>(373.22 to 718.41)
Hubei	Low back pain	215<br>(140 to 307)	387.34<br>(252.37 to 553.24)	268<br>(188 to 364)	484.67<br>(340.11 to 658.96)
Hunan	Low back pain	327<br>(232 to 438)	525.35<br>(373.53 to 703.87)	374<br>(267 to 507)	557.22<br>(397.03 to 754.46)
Inner Mongolia	Low back pain	110<br>(78.3 to 150)	484.91<br>(344.11 to 660.58)	149<br>(104 to 201)	574.63<br>(403.21 to 777.04)
Jiangsu	Low back pain	388<br>(274 to 526)	564.2<br>(398.62 to 764.22)	478<br>(342 to 652)	585.38<br>(418.35 to 798.11)
Jiangxi	Low back pain	189<br>(133 to 257)	478.23<br>(337.3 to 649.58)	245<br>(175 to 334)	516.21<br>(367.83 to 702.74)
Jilin	Low back pain	102<br>(67.5 to 146)	392.79<br>(260.01 to 561.82)	139<br>(98.6 to 189)	499.89<br>(353.5 to 676.69)
Liaoning	Low back pain	224<br>(158 to 305)	548.82<br>(388.53 to 747.85)	280<br>(198 to 382)	627.04<br>(444.52 to 856.38)

Ningxia	Low back pain	22 (15.6 to 30.2)	445.8 (315.19 to 612.05)	35.6 (25.5 to 48.4)	506.56 (363.3 to 689.45)
Qinghai	Low back pain	21.6 (15.2 to 29.3)	457.03 (321.83 to 618.19)	31.8 (22.8 to 44.2)	506.69 (362.84 to 703.35)
Shaanxi	Low back pain	136 (88.6 to 194)	394.96 (257.38 to 562.61)	175 (123 to 237)	442.29 (310.66 to 598.99)
Shandong	Low back pain	357 (230 to 523)	417.64 (268.41 to 610.65)	436 (309 to 604)	438.5 (311.24 to 607.54)
Shanghai	Low back pain	67.1 (41.6 to 101)	480.49 (297.41 to 721.82)	94.5 (64.1 to 133)	338.23 (229.54 to 475.38)
Shanxi	Low back pain	155 (110 to 210)	510.63 (362.46 to 693.88)	212 (151 to 287)	547.86 (392.13 to 743.06)
Sichuan	Low back pain	612 (439 to 830)	548.25 (393.39 to 743.75)	491 (352 to 661)	588.43 (421.63 to 792.08)
Tianjin	Low back pain	50.7 (36.2 to 68.3)	550.78 (393.39 to 742.81)	83.6 (59.5 to 114)	547.84 (390.06 to 744.74)
Tibet	Low back pain	10.7 (7.62 to 14.6)	460.85 (327.16 to 627.54)	15.3 (10.9 to 20.9)	442.35 (314.81 to 603.3)
Xinjiang	Low back pain	72.3 (51.5 to 99.5)	458.37 (326.81 to 630.89)	121 (86.4 to 165)	496.74 (354.72 to 678.47)
Yunnan	Low back pain	141 (87.6 to 210)	362.12 (224.85 to 537.69)	172 (118 to 240)	350.65 (240.77 to 487.18)
Zhejiang	Low back pain	218 (120 to 353)	494.31 (271.02 to 800.22)	154 (98.6 to 224)	251.3 (161.08 to 366.08)
China	Neck pain	4840 (3370 to 6750)	404.1 (281.57 to 563.94)	8760 (6070 to 12300)	620.04 (429.52 to 870.27)
Taiwan	Neck pain	61.7 (42.7 to 86.6)	302.41 (209.27 to 424.21)	94.7 (66.5 to 130)	401.76 (282.04 to 551.52)
Hong Kong Special Administrative Region of China	Neck pain	22.7 (15.3 to 32.6)	394.35 (265.64 to 565.65)	43.1 (29.4 to 62)	574.38 (391.01 to 825.57)
Macau Special Administrative Region of China	Neck pain	1.51 (1.05 to 2.11)	426.71 (295.55 to 593.76)	4.29 (2.98 to 6.05)	626.84 (436.31 to 884.02)
Anhui	Neck pain	229 (159 to 322)	386.51 (268.99 to 542.99)	387 (267 to 543)	633.62 (436.31 to 887.27)
Beijing	Neck pain	54 (37.4 to 76.3)	481 (333.13 to 680.63)	153 (106 to 217)	636.51 (441.29 to 899.06)
Chongqing	Neck pain	68.2 (47.4 to 96)	431.97 (300.38 to 608.29)	172 (119 to 240)	643.3 (446.65 to 899.72)
Fujian	Neck pain	116 (80.4 to 162)	366.03 (254.11 to 510.56)	237 (165 to 333)	601.74 (418.92 to 846.16)
Gansu	Neck pain	88.1 (61 to 124)	372.48 (257.97 to 523.23)	160 (110 to 225)	600.28 (414.47 to 847.15)
Guangdong	Neck pain	254 (177 to 358)	385.84 (268.18 to 543.2)	649 (452 to 915)	555.89 (386.46 to 783.25)
Guangxi	Neck pain	162 (112 to 226)	372.42 (258.36 to 518.56)	273 (189 to 383)	565.57 (391.86 to 792.83)
Guizhou	Neck pain	122 (84.9 to 170)	366.96 (255.7 to 513.3)	197 (137 to 276)	581.96 (404.57 to 814.9)
Hainan	Neck pain	25.1 (17.5 to 35.3)	360.18 (251.07 to 506.73)	53.9 (37.2 to 75.6)	562.2 (388.17 to 788.44)
Hebei	Neck pain	260 (181 to 363)	408.15 (283.61 to 569.76)	455 (315 to 639)	594.92 (412.49 to 836.77)
Heilongjiang	Neck pain	140 (97.6 to 197)	384.97 (267.67 to 540.05)	275 (190 to 390)	699.76 (484.99 to 993.15)
Henan	Neck pain	349 (243 to 488)	394.94 (274.85 to 552.98)	554 (385 to 782)	575.07 (400.26 to 811.86)
Hubei	Neck pain	221 (154 to 309)	398.11 (277.01 to 556.62)	366 (253 to 511)	660.99 (457.89 to 924.22)
Hunan	Neck pain	250 (174 to 350)	402.59 (279.46 to 562.83)	415 (285 to 583)	617.62 (424.99 to 867.75)
Inner Mongolia	Neck pain	84 (58.2 to 118)	369.19 (255.95 to 517.66)	169 (117 to 239)	651.9 (450.16 to 922.53)
Jiangsu	Neck pain	309 (214 to 435)	449.74 (310.65 to 631.84)	541 (375 to 759)	662.28 (459.15 to 929.39)
Jiangxi	Neck pain	142 (99.3 to 197)	360.38 (251.25 to 498.84)	269 (188 to 379)	566.84 (395.87 to 797.75)

Jilin	Neck pain	102<br>(70.9 to 143)	392.96<br>(273.18 to 550.04)	193<br>(134 to 273)	692.21<br>(480.19 to 979.86)
Liaoning	Neck pain	178<br>(123 to 249)	435.62<br>(302.01 to 610)	316<br>(218 to 445)	706.67<br>(489.12 to 996.34)
Ningxia	Neck pain	16.5<br>(11.4 to 23)	333.9<br>(230.96 to 466.38)	39.1<br>(27.2 to 55.4)	557.44<br>(387.01 to 789.3)
Qinghai	Neck pain	16.3<br>(11.3 to 23)	345.16<br>(238.83 to 484.53)	35.4<br>(24.5 to 50.4)	562.9<br>(389.65 to 801.74)
Shaanxi	Neck pain	136<br>(94.3 to 190)	394.48<br>(274.09 to 552.06)	245<br>(171 to 346)	620.05<br>(431.76 to 874.69)
Shandong	Neck pain	366<br>(255 to 512)	428.16<br>(297.71 to 597.62)	639<br>(442 to 901)	643.33<br>(445.18 to 907)
Shanghai	Neck pain	97.5<br>(67.8 to 137)	697.73<br>(484.93 to 980.67)	227<br>(158 to 318)	814<br>(565.21 to 1138.29)
Shanxi	Neck pain	118<br>(82.7 to 166)	391.19<br>(273.09 to 547.68)	232<br>(162 to 326)	601.71<br>(420.06 to 844.84)
Sichuan	Neck pain	471<br>(328 to 661)	421.74<br>(293.7 to 592.6)	541<br>(375 to 754)	648.15<br>(448.93 to 903.39)
Tianjin	Neck pain	42.1<br>(29.4 to 59.1)	457.7<br>(319.22 to 642.5)	96.4<br>(67 to 135)	631.73<br>(439.15 to 886.59)
Tibet	Neck pain	8.25<br>(5.75 to 11.6)	354.2<br>(246.65 to 497.68)	16.4<br>(11.3 to 23.1)	473.38<br>(328.08 to 668.39)
Xinjiang	Neck pain	55.4<br>(38.4 to 77.8)	351.35<br>(243.77 to 493.02)	136<br>(94.3 to 193)	557.23<br>(387.01 to 790.25)
Yunnan	Neck pain	142<br>(98.5 to 200)	364.67<br>(252.64 to 513.75)	277<br>(192 to 391)	563.06<br>(390.8 to 794.82)
Zhejiang	Neck pain	189<br>(132 to 263)	429.67<br>(300.11 to 597.34)	391<br>(270 to 553)	639.51<br>(440.89 to 903.73)
China	Gout	122<br>(80.9 to 168)	10.18<br>(6.76 to 14.06)	265<br>(178 to 363)	18.74<br>(12.62 to 25.71)
Taiwan	Gout	2.59<br>(1.68 to 3.63)	12.72<br>(8.24 to 17.79)	5.76<br>(3.83 to 8.07)	24.43<br>(16.25 to 34.21)
Hong Kong Special Administrative Region of China	Gout	0.794<br>(0.526 to 1.09)	13.77<br>(9.12 to 18.99)	1.73<br>(1.14 to 2.43)	23.09<br>(15.2 to 32.32)
Macau Special Administrative Region of China	Gout	0.039<br>(0.0257 to 0.0541)	11<br>(7.24 to 15.23)	0.135<br>(0.0892 to 0.189)	19.8<br>(13.04 to 27.63)
Anhui	Gout	5.54<br>(3.65 to 7.75)	9.36<br>(6.15 to 13.08)	11.5<br>(7.57 to 16.1)	18.86<br>(12.38 to 26.39)
Beijing	Gout	1.38<br>(0.903 to 1.91)	12.28<br>(8.05 to 17.07)	4.5<br>(2.95 to 6.22)	18.66<br>(12.26 to 25.83)
Chongqing	Gout	1.7<br>(1.12 to 2.38)	10.77<br>(7.13 to 15.08)	5.43<br>(3.63 to 7.62)	20.31<br>(13.57 to 28.53)
Fujian	Gout	2.76<br>(1.8 to 3.86)	8.73<br>(5.7 to 12.22)	6.73<br>(4.45 to 9.35)	17.07<br>(11.28 to 23.71)
Gansu	Gout	2.02<br>(1.32 to 2.81)	8.55<br>(5.57 to 11.88)	4.52<br>(2.97 to 6.3)	16.99<br>(11.18 to 23.71)
Guangdong	Gout	5.6<br>(3.62 to 7.94)	8.51<br>(5.5 to 12.06)	16.3<br>(10.5 to 22.8)	13.92<br>(9.01 to 19.53)
Guangxi	Gout	3.94<br>(2.63 to 5.52)	9.07<br>(6.06 to 12.7)	7.85<br>(5.31 to 11)	16.25<br>(10.98 to 22.78)
Guizhou	Gout	2.85<br>(1.85 to 4)	8.59<br>(5.56 to 12.06)	5.62<br>(3.65 to 7.85)	16.62<br>(10.79 to 23.2)
Hainan	Gout	0.604<br>(0.39 to 0.845)	8.67<br>(5.6 to 12.13)	1.51<br>(0.994 to 2.12)	15.72<br>(10.37 to 22.13)
Hebei	Gout	6.61<br>(4.34 to 9.09)	10.39<br>(6.82 to 14.28)	14.1<br>(9.35 to 19.6)	18.49<br>(12.23 to 25.61)
Heilongjiang	Gout	3.32<br>(2.2 to 4.68)	9.1<br>(6.03 to 12.85)	8.3<br>(5.51 to 11.6)	21.14<br>(14.05 to 29.57)
Henan	Gout	8.7<br>(5.72 to 12)	9.86<br>(6.48 to 13.65)	16.5<br>(11.2 to 23.1)	17.16<br>(11.64 to 23.95)
Hubei	Gout	5.45<br>(3.57 to 7.6)	9.81<br>(6.42 to 13.69)	11<br>(7.32 to 15.4)	19.9<br>(13.24 to 27.91)
Hunan	Gout	6.22<br>(4.07 to 8.74)	9.99<br>(6.55 to 14.06)	12.4<br>(8.08 to 17.4)	18.41<br>(12.03 to 25.87)
Inner Mongolia	Gout	2<br>(1.3 to 2.83)	8.77<br>(5.72 to 12.43)	5.07<br>(3.3 to 7.12)	19.6<br>(12.76 to 27.48)

Jiangsu	Gout	7.83 (5.11 to 11)	11.37 (7.43 to 16.01)	16.9 (11.5 to 23.6)	20.72 (14.03 to 28.84)
Jiangxi	Gout	3.37 (2.24 to 4.76)	8.53 (5.68 to 12.03)	7.47 (4.96 to 10.4)	15.71 (10.43 to 21.86)
Jilin	Gout	2.46 (1.61 to 3.45)	9.5 (6.19 to 13.3)	5.88 (3.8 to 8.2)	21.1 (13.63 to 29.4)
Liaoning	Gout	4.42 (2.88 to 6.26)	10.84 (7.06 to 15.34)	10 (6.59 to 13.8)	22.39 (14.77 to 30.97)
Ningxia	Gout	0.375 (0.247 to 0.524)	7.6 (4.99 to 10.61)	1.08 (0.712 to 1.49)	15.41 (10.14 to 21.27)
Qinghai	Gout	0.359 (0.235 to 0.509)	7.59 (4.97 to 10.75)	0.947 (0.616 to 1.35)	15.07 (9.81 to 21.45)
Shaanxi	Gout	3.37 (2.23 to 4.73)	9.8 (6.49 to 13.75)	7.34 (4.83 to 10.4)	18.57 (12.23 to 26.32)
Shandong	Gout	13 (8.54 to 18.2)	15.23 (9.97 to 21.22)	26.9 (18.1 to 37.7)	27.03 (18.23 to 37.97)
Shanghai	Gout	1.79 (1.17 to 2.52)	12.84 (8.41 to 18.06)	5.1 (3.28 to 7.17)	18.26 (11.75 to 25.66)
Shanxi	Gout	3.03 (1.99 to 4.2)	10 (6.56 to 13.86)	6.88 (4.6 to 9.61)	17.82 (11.9 to 24.88)
Sichuan	Gout	11.5 (7.38 to 16.1)	10.3 (6.61 to 14.42)	16.5 (10.9 to 22.9)	19.78 (13.01 to 27.4)
Tianjin	Gout	1.1 (0.728 to 1.56)	11.99 (7.92 to 16.94)	3.09 (2.08 to 4.35)	20.27 (13.61 to 28.53)
Tibet	Gout	0.186 (0.12 to 0.256)	7.99 (5.16 to 10.98)	0.391 (0.255 to 0.549)	11.31 (7.38 to 15.87)
Xinjiang	Gout	1.33 (0.887 to 1.88)	8.45 (5.63 to 11.89)	3.96 (2.67 to 5.55)	16.27 (10.95 to 22.77)
Yunnan	Gout	3.31 (2.15 to 4.6)	8.48 (5.52 to 11.79)	7.51 (4.96 to 10.4)	15.27 (10.09 to 21.16)
Zhejiang	Gout	4.81 (3.19 to 6.72)	10.9 (7.24 to 15.25)	11.6 (7.62 to 15.9)	18.9 (12.45 to 25.94)
China	Other musculoskeletal disorders	4130 (2870 to 5710)	345.02 (239.4 to 476.96)	6640 (4560 to 9250)	470.26 (322.56 to 655.12)
Taiwan	Other musculoskeletal disorders	81.1 (56.1 to 112)	397.39 (274.73 to 547.44)	151 (106 to 206)	641.18 (450.37 to 873.14)
Hong Kong Special Administrative Region of China	Other musculoskeletal disorders	21.4 (14.6 to 29.7)	371.71 (253.92 to 515.06)	33.6 (22.4 to 47.5)	446.96 (297.78 to 632.82)
Macau Special Administrative Region of China	Other musculoskeletal disorders	1.28 (0.883 to 1.77)	360.59 (248.9 to 498.47)	3.16 (2.13 to 4.33)	461.44 (311.26 to 633.03)
Anhui	Other musculoskeletal disorders	198 (138 to 273)	334.59 (233.31 to 460.48)	315 (214 to 437)	514.68 (349.71 to 715.03)
Beijing	Other musculoskeletal disorders	43.9 (30.5 to 61.2)	391.24 (271.62 to 545.53)	102 (67.9 to 143)	422.29 (281.67 to 591.43)
Chongqing	Other musculoskeletal disorders	58.3 (39.9 to 80.1)	369.11 (252.94 to 507.56)	131 (89.4 to 183)	491.58 (334.55 to 684.92)
Fujian	Other musculoskeletal disorders	99 (69.1 to 137)	312.84 (218.49 to 432.77)	172 (117 to 244)	437.49 (296.27 to 618.41)
Gansu	Other musculoskeletal disorders	81.4 (57.2 to 112)	344.09 (241.78 to 475.33)	137 (92.7 to 189)	513.85 (348.89 to 712.52)
Guangdong	Other musculoskeletal disorders	220 (152 to 300)	334.39 (231.25 to 455.97)	503 (343 to 705)	430.66 (293.83 to 603.75)
Guangxi	Other musculoskeletal disorders	157 (112 to 215)	360.98 (256.53 to 495.28)	246 (170 to 338)	509.18 (352.51 to 700.04)
Guizhou	Other musculoskeletal disorders	110 (77.1 to 151)	331.85 (232.4 to 455.07)	149 (102 to 207)	441.93 (302.54 to 612.58)
Hainan	Other musculoskeletal disorders	21.8 (15.3 to 30)	313.43 (218.96 to 430.3)	42.3 (29 to 58.4)	441.34 (302.5 to 608.76)
Hebei	Other musculoskeletal disorders	217 (148 to 303)	340.23 (232.54 to 476.24)	353 (238 to 493)	461.62 (310.95 to 644.5)
Heilongjiang	Other musculoskeletal disorders	133 (91.8 to 182)	365.59 (251.95 to 499.25)	229 (157 to 315)	584.24 (399.26 to 801.76)
Henan	Other musculoskeletal disorders	296 (200 to 410)	335.47 (226.37 to 464.56)	459 (315 to 637)	476.88 (327.34 to 661.16)
Hubei	Other musculoskeletal disorders	191 (131 to 261)	343.05 (235.44 to 470.56)	292 (197 to 405)	528.07 (355.59 to 732.32)

Hunan	Other musculoskeletal disorders	226<br>(157 to 311)	363.56<br>(252.16 to 499.83)	340<br>(232 to 473)	506.77<br>(346.2 to 704.05)
Inner Mongolia	Other musculoskeletal disorders	77.8<br>(53.6 to 108)	342.03<br>(235.68 to 474.19)	136<br>(91.6 to 192)	523.91<br>(353.56 to 739.75)
Jiangsu	Other musculoskeletal disorders	247<br>(170 to 344)	359.64<br>(246.81 to 499.18)	345<br>(231 to 489)	422.28<br>(282.41 to 599.05)
Jiangxi	Other musculoskeletal disorders	133<br>(94 to 179)	335.88<br>(237.86 to 452.4)	203<br>(138 to 285)	428.07<br>(289.29 to 600.41)
Jilin	Other musculoskeletal disorders	93.9<br>(65.1 to 129)	361.91<br>(250.85 to 497.19)	156<br>(107 to 216)	560.3<br>(382.22 to 775.68)
Liaoning	Other musculoskeletal disorders	148<br>(101 to 206)	362.7<br>(248.17 to 506.1)	242<br>(165 to 343)	542.99<br>(370.28 to 768.4)
Ningxia	Other musculoskeletal disorders	15.1<br>(10.3 to 20.7)	305.5<br>(208.18 to 420.02)	34<br>(23.3 to 47.7)	484.88<br>(331.43 to 680)
Qinghai	Other musculoskeletal disorders	15.4<br>(10.7 to 21.2)	325.04<br>(226.06 to 447.17)	31.5<br>(21.7 to 43.5)	500.7<br>(346.18 to 692.43)
Shaanxi	Other musculoskeletal disorders	112<br>(76.6 to 159)	326.62<br>(222.68 to 461.02)	194<br>(129 to 272)	491.56<br>(327.47 to 687.34)
Shandong	Other musculoskeletal disorders	304<br>(209 to 426)	355.68<br>(243.82 to 497.45)	493<br>(336 to 686)	496.41<br>(337.65 to 690.26)
Shanghai	Other musculoskeletal disorders	52.2<br>(35.5 to 73.2)	373.89<br>(253.99 to 524.2)	95.7<br>(60.7 to 137)	342.47<br>(217.32 to 489.67)
Shanxi	Other musculoskeletal disorders	97.7<br>(67.2 to 136)	322.61<br>(222.06 to 450.39)	170<br>(115 to 239)	440.21<br>(297.2 to 619.81)
Sichuan	Other musculoskeletal disorders	381<br>(263 to 533)	340.99<br>(235.69 to 477.5)	367<br>(248 to 509)	439.05<br>(296.45 to 609.91)
Tianjin	Other musculoskeletal disorders	36.1<br>(25.2 to 50.6)	392.7<br>(274.19 to 550.04)	81.1<br>(54.8 to 113)	531.21<br>(358.76 to 742.3)
Tibet	Other musculoskeletal disorders	7.71<br>(5.35 to 10.6)	330.78<br>(229.58 to 456.11)	15.3<br>(10.5 to 21)	442.77<br>(302.4 to 608.06)
Xinjiang	Other musculoskeletal disorders	49.2<br>(34.1 to 68.6)	312.19<br>(216.08 to 434.68)	116<br>(79.8 to 161)	478.01<br>(327.46 to 660.29)
Yunnan	Other musculoskeletal disorders	135<br>(95.5 to 184)	347.38<br>(245.07 to 471.97)	225<br>(154 to 312)	457.8<br>(313.38 to 634.09)
Zhejiang	Other musculoskeletal disorders	148<br>(102 to 203)	336.65<br>(231.99 to 461.36)	228<br>(150 to 326)	372.76<br>(245.94 to 532.21)
China	Other non-communicable diseases	23000<br>(20200 to 26500)	1918.08<br>(1691.73 to 2211)	14500<br>(11400 to 18600)	1025.18<br>(806.5 to 1315.62)
Taiwan	Other non-communicable diseases	239<br>(198 to 287)	1173.24<br>(969.99 to 1408.76)	264<br>(207 to 335)	1117.61<br>(876.34 to 1418.85)
Hong Kong Special Administrative Region of China	Other non-communicable diseases	56<br>(43.1 to 73.2)	970.8<br>(748.53 to 1269.66)	66.2<br>(45.8 to 94.5)	882.11<br>(609.72 to 1257.86)
Macau Special Administrative Region of China	Other non-communicable diseases	3.17<br>(2.5 to 4.08)	893.36<br>(704.26 to 1148.71)	4.79<br>(3.48 to 6.58)	701.12<br>(509.32 to 962.06)
Anhui	Other non-communicable diseases	1330<br>(1090 to 1620)	2245.86<br>(1835.16 to 2729.18)	686<br>(525 to 889)	1121.2<br>(858.67 to 1454.06)
Beijing	Other non-communicable diseases	158<br>(128 to 198)	1408.07<br>(1138.95 to 1764.51)	260<br>(194 to 342)	1077.78<br>(805.75 to 1418.84)
Chongqing	Other non-communicable diseases	321<br>(254 to 402)	2036.51<br>(1611.37 to 2547.36)	260<br>(198 to 342)	972.56<br>(741.07 to 1280.62)
Fujian	Other non-communicable diseases	576<br>(486 to 683)	1821.41<br>(1535.95 to 2158.75)	372<br>(286 to 484)	944.9<br>(726.01 to 1228.08)
Gansu	Other non-communicable diseases	880<br>(720 to 1060)	3719.41<br>(3045.31 to 4498.73)	362<br>(288 to 453)	1363.74<br>(1083.47 to 1705.24)
Guangdong	Other non-communicable diseases	915<br>(767 to 1100)	1390.23<br>(1164.77 to 1674.84)	1040<br>(806 to 1330)	885.9<br>(690.05 to 1138.04)
Guangxi	Other non-communicable diseases	630<br>(517 to 764)	1448.12<br>(1189.44 to 1757.82)	467<br>(361 to 607)	967.18<br>(747.26 to 1255.77)
Guizhou	Other non-communicable diseases	557<br>(456 to 678)	1679.26<br>(1373.71 to 2043.13)	328<br>(248 to 434)	970.47<br>(734.04 to 1282.02)
Hainan	Other non-communicable diseases	117<br>(94.6 to 144)	1673.1<br>(1358.51 to 2069.46)	115<br>(94.6 to 144)	1203.49<br>(986.31 to 1506.69)
Hebei	Other non-communicable diseases	1270<br>(1050 to 1510)	1991.37<br>(1655.26 to 2374.37)	1010<br>(819 to 1250)	1326.03<br>(1071.16 to 1633.42)
Heilongjiang	Other non-communicable diseases	642<br>(530 to 771)	1762.03<br>(1455.06 to 2115.67)	423<br>(314 to 568)	1078.54<br>(800.48 to 1446.42)

Henan	Other non-communicable diseases	1890<br>(1570 to 2260)	2137.48<br>(1773.07 to 2559.61)	926<br>(709 to 1200)	961.37<br>(736.64 to 1242.83)
Hubei	Other non-communicable diseases	959<br>(794 to 1140)	1726.26<br>(1429.52 to 2056.25)	529<br>(400 to 688)	957.08<br>(724.16 to 1244.86)
Hunan	Other non-communicable diseases	999<br>(824 to 1190)	1606.84<br>(1324.31 to 1920.06)	641<br>(488 to 847)	954.82<br>(726.44 to 1261.29)
Inner Mongolia	Other non-communicable diseases	635<br>(525 to 766)	2792.24<br>(2310.72 to 3366.41)	320<br>(251 to 409)	1235.53<br>(971.26 to 1579.42)
Jiangsu	Other non-communicable diseases	942<br>(774 to 1150)	1369.58<br>(1124.51 to 1670.27)	664<br>(486 to 892)	812.93<br>(595.33 to 1092.22)
Jiangxi	Other non-communicable diseases	948<br>(772 to 1150)	2398.75<br>(1952.6 to 2903.06)	534<br>(419 to 671)	1123.48<br>(881.97 to 1411.65)
Jilin	Other non-communicable diseases	478<br>(394 to 577)	1841.45<br>(1516.82 to 2223.45)	299<br>(228 to 396)	1072.66<br>(818.44 to 1421.2)
Liaoning	Other non-communicable diseases	563<br>(465 to 688)	1381.36<br>(1139.63 to 1685.86)	410<br>(301 to 554)	917.21<br>(673.45 to 1239.94)
Ningxia	Other non-communicable diseases	215<br>(166 to 269)	4349.72<br>(3354.07 to 5449.57)	94<br>(74.2 to 118)	1339.13<br>(1057.05 to 1685.2)
Qinghai	Other non-communicable diseases	115<br>(91.8 to 145)	2436.75<br>(1938.69 to 3060.77)	70.8<br>(55.1 to 90.3)	1127.51<br>(877.16 to 1436.95)
Shaanxi	Other non-communicable diseases	804<br>(674 to 957)	2337.42<br>(1957.89 to 2780.87)	455<br>(349 to 575)	1150.65<br>(882.17 to 1455.65)
Shandong	Other non-communicable diseases	1260<br>(1040 to 1510)	1475.46<br>(1217.06 to 1768.34)	814<br>(610 to 1080)	818.99<br>(613.83 to 1087.11)
Shanghai	Other non-communicable diseases	190<br>(156 to 234)	1362.7<br>(1115.89 to 1675.21)	293<br>(222 to 377)	1047.23<br>(795.95 to 1348.25)
Shanxi	Other non-communicable diseases	647<br>(543 to 765)	2136.75<br>(1792.06 to 2525.43)	440<br>(351 to 551)	1138.46<br>(907.68 to 1426.48)
Sichuan	Other non-communicable diseases	2470<br>(2000 to 3030)	2214.62<br>(1795.11 to 2717.83)	918<br>(688 to 1210)	1099.13<br>(824.2 to 1448.06)
Tianjin	Other non-communicable diseases	149<br>(123 to 180)	1621.18<br>(1338.93 to 1957.45)	168<br>(130 to 217)	1100.96<br>(853.84 to 1418.73)
Tibet	Other non-communicable diseases	79.2<br>(63.5 to 104)	3397.68<br>(2723.13 to 4444.27)	69.9<br>(57.1 to 85.6)	2022.06<br>(1651.6 to 2476.18)
Xinjiang	Other non-communicable diseases	285<br>(233 to 341)	1804.72<br>(1477.33 to 2162.52)	276<br>(222 to 341)	1134.19<br>(912.56 to 1397.77)
Yunnan	Other non-communicable diseases	936<br>(772 to 1140)	2403.08<br>(1982.12 to 2928.15)	569<br>(447 to 723)	1156.94<br>(909.77 to 1471.42)
Zhejiang	Other non-communicable diseases	937<br>(785 to 1110)	2125.44<br>(1780.81 to 2526.33)	597<br>(458 to 792)	975.88<br>(747.87 to 1295.02)
China	Congenital birth defects	14300<br>(13100 to 15800)	1195.77<br>(1095.49 to 1317.02)	5240<br>(4660 to 5840)	370.91<br>(329.83 to 413.49)
Taiwan	Congenital birth defects	98.4<br>(86.6 to 109)	482.44<br>(424.33 to 534.83)	54.2<br>(44.5 to 64.4)	229.78<br>(188.77 to 273.15)
Hong Kong Special Administrative Region of China	Congenital birth defects	20.5<br>(17.7 to 23.2)	355.63<br>(307.7 to 402.6)	10.5<br>(8.12 to 13.1)	139.43<br>(108.08 to 174.41)
Macau Special Administrative Region of China	Congenital birth defects	1.22<br>(1.03 to 1.42)	343.86<br>(290.04 to 400.16)	0.916<br>(0.701 to 1.15)	133.93<br>(102.48 to 168.09)
Anhui	Congenital birth defects	898<br>(717 to 1140)	1516.28<br>(1210.26 to 1917.85)	261<br>(214 to 313)	426.73<br>(349.74 to 511.23)
Beijing	Congenital birth defects	71.4<br>(60.1 to 84.8)	636.6<br>(536.24 to 755.87)	76.5<br>(62.7 to 91.2)	317.38<br>(260.16 to 378.49)
Chongqing	Congenital birth defects	206<br>(153 to 278)	1307.96<br>(969.83 to 1763.8)	77.2<br>(63.4 to 93.3)	288.88<br>(237.2 to 349.2)
Fujian	Congenital birth defects	357<br>(298 to 428)	1128.21<br>(940.9 to 1352.12)	139<br>(115 to 165)	352.15<br>(290.97 to 419.19)
Gansu	Congenital birth defects	685<br>(549 to 857)	2895.04<br>(2320.93 to 3622.53)	185<br>(149 to 230)	696.55<br>(561.22 to 864.2)
Guangdong	Congenital birth defects	467<br>(389 to 557)	709.06<br>(591.68 to 846.28)	377<br>(310 to 450)	322.95<br>(265.57 to 385.12)
Guangxi	Congenital birth defects	293<br>(240 to 351)	672.8<br>(551.49 to 807.76)	147<br>(123 to 176)	304.34<br>(254.24 to 363.67)
Guizhou	Congenital birth defects	248<br>(197 to 310)	748.29<br>(592.21 to 934.38)	80.7<br>(65 to 97.9)	238.61<br>(192.21 to 289.33)
Hainan	Congenital birth defects	70.7<br>(54.3 to 93.5)	1015.33<br>(779.67 to 1341.71)	60.1<br>(48.4 to 73.5)	627.33<br>(505.2 to 766.14)

Hebei	Congenital birth defects	844<br>(688 to 1010)	1326.42<br>(1081.38 to 1586.12)	504<br>(413 to 606)	659.93<br>(540.31 to 793.26)
Heilongjiang	Congenital birth defects	389<br>(310 to 480)	1066.73<br>(850.23 to 1315.9)	119<br>(98 to 144)	303.58<br>(249.7 to 366.43)
Henan	Congenital birth defects	1290<br>(1040 to 1610)	1462.39<br>(1173.89 to 1818.37)	335<br>(273 to 407)	347.84<br>(283.02 to 422.42)
Hubei	Congenital birth defects	593<br>(478 to 737)	1066.53<br>(860.89 to 1327.34)	159<br>(131 to 188)	286.8<br>(237.73 to 339.07)
Hunan	Congenital birth defects	534<br>(423 to 668)	859.19<br>(680.54 to 1074.01)	169<br>(141 to 203)	252.14<br>(210.01 to 301.58)
Inner Mongolia	Congenital birth defects	474<br>(380 to 586)	2086.22<br>(1670.01 to 2575.28)	144<br>(118 to 173)	557.36<br>(456.06 to 667.74)
Jiangsu	Congenital birth defects	506<br>(412 to 619)	735.78<br>(598.14 to 899.24)	165<br>(136 to 201)	202.63<br>(166.68 to 246.15)
Jiangxi	Congenital birth defects	623<br>(479 to 797)	1576.53<br>(1212.65 to 2016.77)	239<br>(195 to 291)	502.88<br>(409.22 to 612.78)
Jilin	Congenital birth defects	295<br>(235 to 362)	1135.47<br>(905.69 to 1394.26)	93.4<br>(77.8 to 112)	334.74<br>(279.05 to 400.18)
Liaoning	Congenital birth defects	319<br>(256 to 384)	781.91<br>(628.67 to 940.79)	101<br>(83 to 121)	226.31<br>(185.83 to 270.85)
Ningxia	Congenital birth defects	178<br>(132 to 231)	3600.12<br>(2680.7 to 4682.2)	50.1<br>(39.6 to 63.1)	714.01<br>(564.4 to 899.38)
Qinghai	Congenital birth defects	79.1<br>(58.5 to 105)	1670.08<br>(1235.98 to 2218.17)	32<br>(25.6 to 39.5)	508.75<br>(407.9 to 628.82)
Shaanxi	Congenital birth defects	566<br>(457 to 702)	1643.67<br>(1327.98 to 2040.17)	213<br>(169 to 267)	539.02<br>(426.75 to 675.86)
Shandong	Congenital birth defects	784<br>(634 to 959)	916.2<br>(740.78 to 1120.71)	244<br>(204 to 292)	245.99<br>(204.98 to 293.62)
Shanghai	Congenital birth defects	96.7<br>(80.7 to 115)	691.88<br>(577.88 to 819.64)	120<br>(96.9 to 145)	428.16<br>(346.98 to 517.42)
Shanxi	Congenital birth defects	468<br>(379 to 565)	1545.33<br>(1250.85 to 1866.71)	219<br>(182 to 261)	566.82<br>(470.78 to 677.01)
Sichuan	Congenital birth defects	1470<br>(1130 to 1890)	1314.58<br>(1014.78 to 1694.03)	275<br>(219 to 336)	329.54<br>(262.78 to 402.31)
Tianjin	Congenital birth defects	84.4<br>(69.9 to 100)	917.64<br>(759.97 to 1087.29)	66.5<br>(55.6 to 79.2)	435.58<br>(364.02 to 518.82)
Tibet	Congenital birth defects	54.5<br>(41.1 to 78.7)	2340.44<br>(1762.17 to 3375.59)	45.3<br>(34.9 to 58.2)	1310.66<br>(1009.53 to 1683.02)
Xinjiang	Congenital birth defects	181<br>(143 to 230)	1148.63<br>(905.66 to 1461.03)	131<br>(104 to 164)	537.1<br>(424.97 to 671.03)
Yunnan	Congenital birth defects	543<br>(421 to 695)	1394.15<br>(1081.14 to 1782.72)	203<br>(163 to 253)	412.51<br>(331.75 to 515.3)
Zhejiang	Congenital birth defects	625<br>(520 to 747)	1416.89<br>(1178.98 to 1694.95)	196<br>(160 to 236)	319.59<br>(262.08 to 385.69)
China	Skin and subcutaneous diseases	6380<br>(4180 to 9500)	532.69<br>(349.3 to 793.67)	7170<br>(4730 to 10700)	507.92<br>(334.92 to 758.15)
Taiwan	Skin and subcutaneous diseases	107<br>(72.3 to 156)	524.64<br>(354.54 to 763.88)	129<br>(87 to 186)	545.48<br>(368.88 to 788.71)
Hong Kong Special Administrative Region of China	Skin and subcutaneous diseases	29.1<br>(19.1 to 43.6)	505.27<br>(331.37 to 756)	39.1<br>(25.7 to 58.2)	520.85<br>(342.66 to 774.73)
Macau Special Administrative Region of China	Skin and subcutaneous diseases	1.89<br>(1.25 to 2.83)	533.58<br>(351.27 to 796.09)	3.62<br>(2.36 to 5.42)	529.2<br>(344.75 to 791.93)
Anhui	Skin and subcutaneous diseases	320<br>(210 to 473)	539.79<br>(354.32 to 797.69)	308<br>(203 to 460)	503.78<br>(332.11 to 752.68)
Beijing	Skin and subcutaneous diseases	54.1<br>(35.6 to 81.7)	482.24<br>(317.56 to 728.41)	115<br>(75.9 to 173)	477.67<br>(315.12 to 716.39)
Chongqing	Skin and subcutaneous diseases	85.5<br>(56.7 to 126)	541.57<br>(359.32 to 801.02)	147<br>(98.5 to 217)	550.77<br>(368.69 to 812.18)
Fujian	Skin and subcutaneous diseases	176<br>(116 to 262)	556.09<br>(366.76 to 828.89)	202<br>(134 to 301)	511.62<br>(338.89 to 762.66)
Gansu	Skin and subcutaneous diseases	127<br>(83.7 to 189)	535.38<br>(353.8 to 799.58)	132<br>(87.1 to 198)	497.89<br>(327.57 to 744.05)
Guangdong	Skin and subcutaneous diseases	339<br>(220 to 505)	515.27<br>(334.32 to 767.4)	578<br>(382 to 868)	494.99<br>(326.8 to 743.07)
Guangxi	Skin and subcutaneous diseases	236<br>(154 to 354)	542.47<br>(354.65 to 814.78)	248<br>(165 to 370)	513.98<br>(341.27 to 765.18)

Guizhou	Skin and subcutaneous diseases	192 (127 to 284)	577.41 (381.33 to 854.58)	178 (118 to 267)	526.59 (349.4 to 788.78)
Hainan	Skin and subcutaneous diseases	37.6 (24.6 to 56)	539.71 (352.55 to 804.4)	49.1 (32.8 to 72.8)	511.73 (341.66 to 759.71)
Hebei	Skin and subcutaneous diseases	336 (220 to 502)	527.45 (345.17 to 788.32)	390 (257 to 578)	510.67 (336.6 to 755.82)
Heilongjiang	Skin and subcutaneous diseases	196 (128 to 292)	538.2 (350.66 to 800.13)	199 (131 to 299)	506.36 (334.55 to 761.39)
Henan	Skin and subcutaneous diseases	474 (310 to 711)	537.31 (351.21 to 805.6)	496 (325 to 744)	515 (337.56 to 772.83)
Hubei	Skin and subcutaneous diseases	306 (198 to 454)	550.88 (356.99 to 817.23)	287 (189 to 426)	519.09 (340.95 to 770.48)
Hunan	Skin and subcutaneous diseases	324 (211 to 485)	520.74 (339.49 to 779.91)	340 (225 to 508)	506.2 (334.35 to 757.1)
Inner Mongolia	Skin and subcutaneous diseases	126 (81.5 to 187)	553.11 (358.47 to 822.26)	131 (85.5 to 196)	507.01 (330.36 to 755.78)
Jiangsu	Skin and subcutaneous diseases	356 (234 to 533)	517.1 (339.79 to 774.03)	418 (275 to 620)	512.23 (337.28 to 759.62)
Jiangxi	Skin and subcutaneous diseases	218 (144 to 327)	551.51 (364.06 to 827.04)	241 (158 to 359)	507.67 (333.26 to 755.68)
Jilin	Skin and subcutaneous diseases	134 (86.9 to 200)	514.93 (334.69 to 772.07)	139 (91.1 to 207)	496.74 (326.5 to 742.25)
Liaoning	Skin and subcutaneous diseases	208 (137 to 312)	510.86 (335.31 to 765.84)	225 (149 to 336)	504.77 (332.98 to 753.49)
Ningxia	Skin and subcutaneous diseases	26.9 (17.7 to 40)	544.74 (359.27 to 810.43)	35.3 (23.1 to 52.6)	502.47 (328.44 to 748.8)
Qinghai	Skin and subcutaneous diseases	26.2 (17.1 to 39.1)	553.59 (362.03 to 825.06)	31.4 (20.7 to 47.4)	499.84 (329.16 to 754.11)
Shaanxi	Skin and subcutaneous diseases	183 (119 to 273)	532.4 (344.41 to 793.56)	198 (130 to 295)	500.5 (328.11 to 745.8)
Shandong	Skin and subcutaneous diseases	447 (295 to 673)	522.55 (344.75 to 785.76)	505 (334 to 761)	508.57 (336.31 to 765.98)
Shanghai	Skin and subcutaneous diseases	77.1 (50.7 to 114)	551.65 (362.86 to 812.74)	151 (100 to 224)	541.75 (358.95 to 801.79)
Shanxi	Skin and subcutaneous diseases	161 (105 to 239)	530.28 (346.37 to 790.06)	194 (128 to 293)	502.81 (330.96 to 757.85)
Sichuan	Skin and subcutaneous diseases	580 (379 to 872)	519.78 (339.77 to 780.89)	415 (271 to 620)	496.64 (325.17 to 742.89)
Tianjin	Skin and subcutaneous diseases	46.7 (30.5 to 69.9)	508.11 (331.84 to 760.5)	76.7 (50.8 to 114)	502.71 (332.81 to 750.15)
Tibet	Skin and subcutaneous diseases	13.2 (8.57 to 19.8)	564.89 (367.77 to 848.9)	17.6 (11.4 to 26.1)	507.85 (330.5 to 756.11)
Xinjiang	Skin and subcutaneous diseases	84.6 (54.9 to 126)	536.07 (348.25 to 796.02)	121 (81 to 180)	495.86 (332.23 to 739.23)
Yunnan	Skin and subcutaneous diseases	222 (147 to 330)	569.75 (378.34 to 846.52)	249 (164 to 372)	505.42 (334.01 to 756.82)
Zhejiang	Skin and subcutaneous diseases	233 (153 to 347)	528.97 (347.62 to 786.08)	313 (206 to 467)	511.17 (337.41 to 762.53)
China	Dermatitis	1320 (781 to 2070)	110.15 (65.29 to 173.13)	1390 (840 to 2120)	98.06 (59.49 to 149.82)
Taiwan	Dermatitis	24.9 (14.5 to 38.6)	121.89 (70.84 to 189.43)	25.9 (15.5 to 40.7)	109.98 (65.78 to 172.5)
Hong Kong Special Administrative Region of China	Dermatitis	5.93 (3.56 to 9.2)	102.92 (61.73 to 159.61)	7.2 (4.41 to 11.1)	95.86 (58.66 to 148.29)
Macau Special Administrative Region of China	Dermatitis	0.381 (0.226 to 0.594)	107.39 (63.66 to 167.39)	0.655 (0.4 to 1)	95.74 (58.51 to 146.85)
Anhui	Dermatitis	64.7 (38.2 to 101)	109.22 (64.44 to 170.64)	60.2 (36.3 to 93.1)	98.41 (59.34 to 152.23)
Beijing	Dermatitis	9.85 (5.88 to 15.2)	87.81 (52.38 to 135.47)	19.2 (11.9 to 29)	79.74 (49.21 to 120.34)
Chongqing	Dermatitis	16.5 (9.89 to 25.7)	104.83 (62.67 to 162.67)	26.6 (16.3 to 40.6)	99.71 (60.87 to 151.99)
Fujian	Dermatitis	35.6 (21 to 56)	112.66 (66.44 to 176.97)	38.8 (23.5 to 59.5)	98.42 (59.75 to 151.03)
Gansu	Dermatitis	26 (15.2 to 40.9)	109.83 (64.38 to 173.09)	26.2 (15.8 to 40.8)	98.69 (59.58 to 153.51)

Guangdong	Dermatitis	61.9<br>(36.7 to 96.3)	94.04<br>(55.78 to 146.24)	96.4<br>(58.8 to 149)	82.55<br>(50.3 to 127.92)
Guangxi	Dermatitis	49<br>(28.7 to 76.7)	112.65<br>(65.9 to 176.44)	49.5<br>(29.7 to 76.5)	102.38<br>(61.46 to 158.33)
Guizhou	Dermatitis	36.6<br>(21.5 to 57.3)	110.2<br>(64.75 to 172.67)	33.5<br>(20.2 to 51.6)	98.96<br>(59.61 to 152.4)
Hainan	Dermatitis	8<br>(4.63 to 12.6)	114.9<br>(66.52 to 180.95)	9.58<br>(5.71 to 14.8)	99.9<br>(59.59 to 153.87)
Hebei	Dermatitis	70.3<br>(41.5 to 109)	110.53<br>(65.22 to 171.6)	76.7<br>(46.1 to 118)	100.41<br>(60.32 to 154.2)
Heilongjiang	Dermatitis	45.2<br>(26.2 to 71.7)	123.89<br>(71.76 to 196.67)	40.3<br>(24.6 to 61.9)	102.73<br>(62.58 to 157.81)
Henan	Dermatitis	96.7<br>(57.9 to 151)	109.56<br>(65.61 to 170.79)	98<br>(59.4 to 151)	101.74<br>(61.72 to 156.99)
Hubei	Dermatitis	76.4<br>(44.4 to 121)	137.44<br>(80 to 218.36)	64.3<br>(38.6 to 100)	116.22<br>(69.79 to 180.83)
Hunan	Dermatitis	67.4<br>(40 to 106)	108.33<br>(64.28 to 170.37)	66.6<br>(40.3 to 103)	99.11<br>(59.95 to 152.83)
Inner Mongolia	Dermatitis	32.7<br>(19 to 51.9)	143.88<br>(83.41 to 228.44)	30.4<br>(18.2 to 47.2)	117.59<br>(70.42 to 182.1)
Jiangsu	Dermatitis	71.5<br>(43.2 to 111)	103.91<br>(62.74 to 161.87)	77.7<br>(47.7 to 119)	95.16<br>(58.38 to 146.12)
Jiangxi	Dermatitis	43.9<br>(26.1 to 69.3)	111.16<br>(65.98 to 175.38)	48.1<br>(28.5 to 74)	101.09<br>(59.97 to 155.68)
Jilin	Dermatitis	27.9<br>(16.4 to 43.8)	107.46<br>(63.24 to 168.61)	26.1<br>(16 to 40)	93.6<br>(57.36 to 143.33)
Liaoning	Dermatitis	42.5<br>(25.3 to 65.8)	104.11<br>(61.99 to 161.42)	41.3<br>(25 to 62.8)	92.56<br>(55.96 to 140.71)
Ningxia	Dermatitis	5.63<br>(3.28 to 8.9)	114.04<br>(66.51 to 180.17)	7.02<br>(4.18 to 10.9)	100.02<br>(59.59 to 155.48)
Qinghai	Dermatitis	5.26<br>(3.08 to 8.28)	111.01<br>(64.98 to 174.79)	6.2<br>(3.75 to 9.59)	98.74<br>(59.74 to 152.71)
Shaanxi	Dermatitis	39.4<br>(23.1 to 61.6)	114.42<br>(67.09 to 178.94)	38.9<br>(23.6 to 59.5)	98.54<br>(59.76 to 150.69)
Shandong	Dermatitis	91.1<br>(53.9 to 143)	106.37<br>(63 to 166.71)	96.5<br>(58.2 to 147)	97.09<br>(58.6 to 148.3)
Shanghai	Dermatitis	19.8<br>(11.6 to 31.6)	141.65<br>(83.03 to 225.96)	34.9<br>(20.7 to 54.6)	125<br>(74.18 to 195.48)
Shanxi	Dermatitis	33.3<br>(19.5 to 52.3)	110.05<br>(64.41 to 172.67)	37.4<br>(22.8 to 57.3)	96.89<br>(58.95 to 148.37)
Sichuan	Dermatitis	117<br>(69.3 to 183)	105.09<br>(62.08 to 163.68)	80.6<br>(49.2 to 125)	96.59<br>(58.91 to 149.2)
Tianjin	Dermatitis	9.7<br>(5.71 to 15)	105.43<br>(62.05 to 163.6)	14.1<br>(8.66 to 21.4)	92.47<br>(56.72 to 140.43)
Tibet	Dermatitis	2.65<br>(1.54 to 4.16)	113.7<br>(66.2 to 178.68)	3.52<br>(2.07 to 5.53)	101.96<br>(59.97 to 159.89)
Xinjiang	Dermatitis	15.6<br>(9.15 to 24.4)	98.74<br>(58.03 to 154.42)	21.4<br>(13 to 33)	87.71<br>(53.54 to 135.58)
Yunnan	Dermatitis	43.4<br>(25.5 to 68.5)	111.33<br>(65.42 to 175.7)	48.8<br>(29 to 75.9)	99.32<br>(58.95 to 154.44)
Zhejiang	Dermatitis	46.5<br>(27.6 to 72.2)	105.35<br>(62.48 to 163.83)	58.1<br>(35.5 to 89.1)	95.03<br>(58 to 145.57)
China	Psoriasis	391<br>(277 to 520)	32.68<br>(23.1 to 43.48)	754<br>(534 to 998)	53.35<br>(37.81 to 70.67)
Taiwan	Psoriasis	3.2<br>(2.21 to 4.26)	15.69<br>(10.85 to 20.88)	6.23<br>(4.39 to 8.41)	26.43<br>(18.63 to 35.66)
Hong Kong Special Administrative Region of China	Psoriasis	2.81<br>(1.98 to 3.76)	48.7<br>(34.3 to 65.24)	5.03<br>(3.51 to 6.72)	67<br>(46.8 to 89.53)
Macau Special Administrative Region of China	Psoriasis	0.16<br>(0.112 to 0.213)	45.06<br>(31.45 to 59.92)	0.435<br>(0.305 to 0.58)	63.64<br>(44.63 to 84.86)
Anhui	Psoriasis	16.9<br>(11.7 to 23.3)	28.45<br>(19.74 to 39.29)	30.3<br>(21.5 to 39.9)	49.61<br>(35.08 to 65.23)
Beijing	Psoriasis	3.56<br>(2.49 to 4.77)	31.78<br>(22.24 to 42.52)	12<br>(8.36 to 16)	49.6<br>(34.7 to 66.52)
Chongqing	Psoriasis	10.1<br>(7.02 to 13.5)	64.26<br>(44.51 to 85.3)	24<br>(17.1 to 31.7)	89.77<br>(64.04 to 118.79)

Fujian	Psoriasis	9.05<br>(6.35 to 12.1)	28.59<br>(20.06 to 38.21)	20.9<br>(15 to 27.9)	53.09<br>(38.07 to 70.87)
Gansu	Psoriasis	6.57<br>(4.57 to 8.75)	27.77<br>(19.32 to 36.99)	12.5<br>(8.84 to 16.6)	47.2<br>(33.24 to 62.41)
Guangdong	Psoriasis	20.5<br>(14.2 to 27.5)	31.22<br>(21.6 to 41.74)	63.3<br>(44.7 to 83.5)	54.16<br>(38.25 to 71.5)
Guangxi	Psoriasis	12.6<br>(8.61 to 17)	28.87<br>(19.8 to 39)	23.6<br>(16.7 to 31.2)	48.74<br>(34.6 to 64.65)
Guizhou	Psoriasis	8.58<br>(5.96 to 11.5)	25.84<br>(17.95 to 34.67)	14.8<br>(10.4 to 20)	43.68<br>(30.75 to 59.09)
Hainan	Psoriasis	1.98<br>(1.4 to 2.66)	28.43<br>(20.07 to 38.22)	4.69<br>(3.27 to 6.25)	48.87<br>(34.09 to 65.23)
Hebei	Psoriasis	23.2<br>(16.5 to 31.2)	36.49<br>(25.87 to 49.08)	38.9<br>(27.2 to 51.8)	50.95<br>(35.59 to 67.73)
Heilongjiang	Psoriasis	12.5<br>(8.62 to 16.8)	34.37<br>(23.64 to 46.03)	24.1<br>(17.2 to 32.5)	61.48<br>(43.86 to 82.85)
Henan	Psoriasis	30.6<br>(21.5 to 40.8)	34.66<br>(24.3 to 46.23)	45.9<br>(32.4 to 61.3)	47.71<br>(33.66 to 63.6)
Hubei	Psoriasis	17.5<br>(12.2 to 23.7)	31.49<br>(22.02 to 42.64)	30.8<br>(21.5 to 40.4)	55.65<br>(38.92 to 73.04)
Hunan	Psoriasis	19.4<br>(13.7 to 25.8)	31.23<br>(21.96 to 41.44)	35.1<br>(24.9 to 46.5)	52.25<br>(37.06 to 69.29)
Inner Mongolia	Psoriasis	5.79<br>(4.05 to 7.76)	25.47<br>(17.83 to 34.12)	12<br>(8.43 to 16.2)	46.47<br>(32.56 to 62.46)
Jiangsu	Psoriasis	24.5<br>(17.1 to 32.5)	35.59<br>(24.81 to 47.25)	48.6<br>(33.7 to 64.2)	59.53<br>(41.28 to 78.62)
Jiangxi	Psoriasis	10.9<br>(7.45 to 14.7)	27.46<br>(18.83 to 37.2)	22.2<br>(15.7 to 29.5)	46.68<br>(32.92 to 62.02)
Jilin	Psoriasis	8.67<br>(6.07 to 11.6)	33.42<br>(23.41 to 44.65)	17.1<br>(12 to 22.5)	61.14<br>(42.85 to 80.69)
Liaoning	Psoriasis	15.6<br>(10.9 to 20.9)	38.29<br>(26.79 to 51.32)	28.6<br>(20.3 to 37.8)	64.12<br>(45.41 to 84.73)
Ningxia	Psoriasis	1.31<br>(0.919 to 1.76)	26.58<br>(18.6 to 35.58)	3.31<br>(2.32 to 4.38)	47.09<br>(33.11 to 62.42)
Qinghai	Psoriasis	1.28<br>(0.91 to 1.73)	27.12<br>(19.21 to 36.49)	2.94<br>(2.05 to 3.94)	46.73<br>(32.66 to 62.66)
Shaanxi	Psoriasis	10.4<br>(7.35 to 14.1)	30.26<br>(21.35 to 40.86)	21.2<br>(14.8 to 28.1)	53.58<br>(37.52 to 71.14)
Shandong	Psoriasis	30.2<br>(21 to 40.8)	35.23<br>(24.48 to 47.7)	51.7<br>(36.5 to 68.7)	52.07<br>(36.69 to 69.1)
Shanghai	Psoriasis	6.5<br>(4.58 to 8.68)	46.54<br>(32.76 to 62.15)	18.6<br>(12.9 to 24.7)	66.52<br>(46.26 to 88.29)
Shanxi	Psoriasis	11.6<br>(8.1 to 15.4)	38.41<br>(26.74 to 50.89)	21.2<br>(15.1 to 28.4)	54.87<br>(39.15 to 73.56)
Sichuan	Psoriasis	34.6<br>(24.3 to 46.6)	30.97<br>(21.76 to 41.75)	38.5<br>(27 to 51.3)	46.15<br>(32.3 to 61.42)
Tianjin	Psoriasis	3.75<br>(2.66 to 5.04)	40.79<br>(28.88 to 54.77)	9.96<br>(7.02 to 13.3)	65.29<br>(46 to 86.88)
Tibet	Psoriasis	0.529<br>(0.369 to 0.714)	22.7<br>(15.82 to 30.63)	1.18<br>(0.828 to 1.58)	34.27<br>(23.94 to 45.57)
Xinjiang	Psoriasis	4.67<br>(3.29 to 6.26)	29.64<br>(20.88 to 39.66)	12.3<br>(8.63 to 16.5)	50.43<br>(35.4 to 67.52)
Yunnan	Psoriasis	10.2<br>(7.27 to 13.5)	26.2<br>(18.66 to 34.75)	22.2<br>(15.7 to 30)	45.25<br>(31.97 to 61.11)
Zhejiang	Psoriasis	14.6<br>(10.2 to 19.7)	33.09<br>(23.14 to 44.67)	35.6<br>(24.7 to 47.9)	58.12<br>(40.4 to 78.35)
China	Scabies	1440<br>(800 to 2360)	120.54<br>(66.83 to 197.29)	1390<br>(760 to 2290)	98.58<br>(53.84 to 161.83)
Taiwan	Scabies	20.5<br>(11.4 to 33.4)	100.28<br>(55.75 to 163.95)	20.9<br>(11.5 to 33.9)	88.5<br>(48.6 to 143.66)
Hong Kong Special Administrative Region of China	Scabies	6.11<br>(3.35 to 9.95)	106.03<br>(58.07 to 172.56)	7.04<br>(3.86 to 11.4)	93.73<br>(51.4 to 151.63)
Macau Special Administrative Region of China	Scabies	0.385<br>(0.209 to 0.627)	108.38<br>(59.03 to 176.76)	0.671<br>(0.372 to 1.1)	98.05<br>(54.36 to 161.49)
Anhui	Scabies	74.1<br>(41.2 to 122)	125.1<br>(69.47 to 206.72)	60.8<br>(33.5 to 99.4)	99.49<br>(54.86 to 162.5)

Beijing	Scabies	11.7<br>(6.4 to 19.1)	104.7<br>(57.05 to 169.94)	22.6<br>(12.5 to 37.3)	93.92<br>(51.93 to 154.81)
Chongqing	Scabies	19<br>(10.6 to 31)	120.51<br>(66.92 to 196.36)	26<br>(14.3 to 42.6)	97.27<br>(53.51 to 159.24)
Fujian	Scabies	38.7<br>(21.6 to 63.4)	122.3<br>(68.34 to 200.45)	38.5<br>(21.4 to 62.7)	97.65<br>(54.19 to 159.19)
Gansu	Scabies	30<br>(16.6 to 48.9)	126.81<br>(70.26 to 206.64)	27.4<br>(15.1 to 45)	103.1<br>(56.76 to 169.33)
Guangdong	Scabies	76.1<br>(42.1 to 124)	115.63<br>(64 to 188.8)	118<br>(64.3 to 194)	100.7<br>(55.01 to 166.43)
Guangxi	Scabies	53.6<br>(29.8 to 87.7)	123.16<br>(68.63 to 201.59)	48.1<br>(26.6 to 79.2)	99.63<br>(55.12 to 163.92)
Guizhou	Scabies	42.4<br>(23.2 to 68.8)	127.84<br>(69.99 to 207.16)	35.1<br>(19.2 to 57.6)	103.9<br>(56.76 to 170.3)
Hainan	Scabies	8.26<br>(4.63 to 13.5)	118.61<br>(66.44 to 193.23)	9.65<br>(5.3 to 15.8)	100.63<br>(55.31 to 164.59)
Hebei	Scabies	74.2<br>(41.1 to 120)	116.53<br>(64.62 to 188.21)	74.5<br>(41.1 to 121)	97.54<br>(53.78 to 158.65)
Heilongjiang	Scabies	45.8<br>(25.5 to 75.5)	125.52<br>(69.85 to 207)	38.8<br>(21.1 to 64)	98.89<br>(53.73 to 163.16)
Henan	Scabies	113<br>(62.9 to 187)	128.55<br>(71.27 to 211.69)	100<br>(55.8 to 166)	104.29<br>(57.96 to 172.04)
Hubei	Scabies	66.3<br>(36.9 to 108)	119.34<br>(66.45 to 194.89)	53.1<br>(29.1 to 86.7)	96<br>(52.67 to 156.87)
Hunan	Scabies	73.5<br>(40.9 to 119)	118.2<br>(65.78 to 191.51)	64.4<br>(35.3 to 105)	95.85<br>(52.53 to 156.69)
Inner Mongolia	Scabies	27.3<br>(15.2 to 44)	119.9<br>(66.69 to 193.67)	24.7<br>(13.7 to 40.7)	95.49<br>(52.91 to 157.35)
Jiangsu	Scabies	77.2<br>(42.5 to 125)	112.17<br>(61.8 to 181.83)	76.7<br>(42.3 to 125)	93.94<br>(51.75 to 153.2)
Jiangxi	Scabies	49.9<br>(27.5 to 82.1)	126.19<br>(69.57 to 207.64)	48.8<br>(27.1 to 79.1)	102.69<br>(57.05 to 166.32)
Jilin	Scabies	30.4<br>(16.8 to 49.8)	116.99<br>(64.68 to 191.81)	26.2<br>(14.5 to 43.1)	93.97<br>(52.05 to 154.51)
Liaoning	Scabies	47<br>(25.7 to 76.5)	115.2<br>(63.06 to 187.57)	41.9<br>(23 to 69)	93.93<br>(51.61 to 154.43)
Ningxia	Scabies	6.22<br>(3.46 to 10.2)	125.95<br>(70.04 to 207.14)	7.25<br>(4.02 to 11.8)	103.29<br>(57.32 to 168.81)
Qinghai	Scabies	6.04<br>(3.34 to 9.97)	127.52<br>(70.56 to 210.55)	6.55<br>(3.63 to 10.7)	104.19<br>(57.74 to 170.29)
Shaanxi	Scabies	41.6<br>(23.1 to 68.6)	121.01<br>(67.2 to 199.28)	39<br>(21.4 to 64.2)	98.74<br>(54.16 to 162.52)
Shandong	Scabies	103<br>(57 to 170)	120.9<br>(66.6 to 198.06)	97<br>(53.2 to 161)	97.57<br>(53.5 to 162.04)
Shanghai	Scabies	13.9<br>(7.56 to 22.8)	99.46<br>(54.07 to 163.36)	25.9<br>(14.2 to 41.9)	92.75<br>(50.97 to 149.92)
Shanxi	Scabies	35.8<br>(19.8 to 58)	118.19<br>(65.51 to 191.51)	38.6<br>(21.3 to 63.3)	99.97<br>(55.16 to 163.97)
Sichuan	Scabies	137<br>(75.7 to 226)	123.07<br>(67.81 to 202.46)	82<br>(45.5 to 134)	98.23<br>(54.54 to 160.08)
Tianjin	Scabies	9.71<br>(5.34 to 15.8)	105.62<br>(58.05 to 172.25)	14.4<br>(7.97 to 23.6)	94.6<br>(52.2 to 154.89)
Tibet	Scabies	2.98<br>(1.66 to 4.9)	127.72<br>(71.37 to 210.4)	3.88<br>(2.14 to 6.39)	112.18<br>(61.88 to 184.77)
Xinjiang	Scabies	19.4<br>(10.7 to 31.7)	122.92<br>(68.01 to 201.28)	24.5<br>(13.5 to 39.8)	100.7<br>(55.6 to 163.53)
Yunnan	Scabies	50.2<br>(27.9 to 82)	128.94<br>(71.68 to 210.45)	51.3<br>(28.3 to 84.5)	104.24<br>(57.52 to 171.9)
Zhejiang	Scabies	50.7<br>(28.2 to 82)	115.02<br>(63.86 to 186.02)	58.7<br>(32.3 to 95.9)	95.91<br>(52.84 to 156.78)
China	Fungal skin diseases	526<br>(209 to 1090)	43.97<br>(17.43 to 90.94)	663<br>(267 to 1390)	46.97<br>(18.93 to 98.2)
Taiwan	Fungal skin diseases	6.02<br>(2.39 to 12.7)	29.49<br>(11.71 to 62.15)	8.63<br>(3.45 to 17.8)	36.57<br>(14.64 to 75.37)
Hong Kong Special Administrative Region of China	Fungal skin diseases	2.11<br>(0.813 to 4.36)	36.55<br>(14.1 to 75.69)	3.6<br>(1.44 to 7.53)	47.89<br>(19.12 to 100.29)

Macau Special Administrative Region of China	Fungal skin diseases	0.143 (0.0565 to 0.298)	40.33 (15.92 to 83.97)	0.31 (0.124 to 0.651)	45.29 (18.09 to 95.18)
Anhui	Fungal skin diseases	25 (9.94 to 51.9)	42.13 (16.77 to 87.52)	29.8 (12 to 62.5)	48.66 (19.63 to 102.18)
Beijing	Fungal skin diseases	4.69 (1.86 to 9.82)	41.77 (16.58 to 87.58)	11.2 (4.5 to 23.3)	46.64 (18.68 to 96.73)
Chongqing	Fungal skin diseases	7.01 (2.78 to 14.5)	44.44 (17.61 to 92.1)	14 (5.65 to 29.3)	52.42 (21.15 to 109.67)
Fujian	Fungal skin diseases	13.4 (5.31 to 27.6)	42.27 (16.77 to 87.27)	17.9 (7.21 to 37.5)	45.36 (18.29 to 95.02)
Gansu	Fungal skin diseases	10.5 (4.16 to 21.7)	44.2 (17.57 to 91.83)	12.2 (4.89 to 25.5)	45.77 (18.41 to 95.98)
Guangdong	Fungal skin diseases	28.4 (11.3 to 59.6)	43.08 (17.18 to 90.52)	49.7 (19.9 to 105)	42.55 (17.06 to 89.82)
Guangxi	Fungal skin diseases	20.4 (8.1 to 42.5)	46.9 (18.61 to 97.82)	22.4 (9.01 to 46.9)	46.29 (18.64 to 97.03)
Guizhou	Fungal skin diseases	17.1 (6.7 to 35.9)	51.41 (20.17 to 108.09)	16.2 (6.57 to 34.2)	48 (19.43 to 100.97)
Hainan	Fungal skin diseases	3.16 (1.24 to 6.56)	45.41 (17.86 to 94.18)	4.36 (1.76 to 9.15)	45.44 (18.32 to 95.4)
Hebei	Fungal skin diseases	27.2 (10.8 to 56.8)	42.7 (16.91 to 89.24)	34.5 (13.8 to 72.5)	45.19 (18.09 to 94.92)
Heilongjiang	Fungal skin diseases	14.6 (5.76 to 30.5)	40.07 (15.81 to 83.8)	19 (7.64 to 39.1)	48.35 (19.46 to 99.68)
Henan	Fungal skin diseases	38.6 (15.3 to 80.1)	43.72 (17.38 to 90.75)	43.3 (17.4 to 91.3)	44.98 (18.09 to 94.77)
Hubei	Fungal skin diseases	24.2 (9.63 to 49.9)	43.52 (17.34 to 89.82)	26.8 (10.7 to 55.5)	48.38 (19.36 to 100.29)
Hunan	Fungal skin diseases	27.9 (11.1 to 58.1)	44.84 (17.86 to 93.37)	31.9 (12.8 to 66.5)	47.46 (19.09 to 99.08)
Inner Mongolia	Fungal skin diseases	9.52 (3.76 to 19.7)	41.84 (16.55 to 86.49)	11.9 (4.79 to 24.7)	45.84 (18.48 to 95.42)
Jiangsu	Fungal skin diseases	29.3 (11.6 to 61.6)	42.6 (16.89 to 89.45)	40.9 (16.5 to 86.1)	50.03 (20.16 to 105.39)
Jiangxi	Fungal skin diseases	17.9 (7.07 to 37.3)	45.27 (17.89 to 94.33)	21.1 (8.48 to 44.4)	44.43 (17.83 to 93.37)
Jilin	Fungal skin diseases	10.8 (4.3 to 22.4)	41.7 (16.56 to 86.23)	13.6 (5.47 to 28.3)	48.81 (19.62 to 101.49)
Liaoning	Fungal skin diseases	16.8 (6.65 to 35.3)	41.26 (16.29 to 86.45)	22.7 (9.15 to 46.9)	50.84 (20.49 to 105.06)
Ningxia	Fungal skin diseases	2.06 (0.818 to 4.3)	41.78 (16.56 to 87.12)	2.99 (1.2 to 6.28)	42.62 (17.15 to 89.43)
Qinghai	Fungal skin diseases	2.33 (0.908 to 4.89)	49.18 (19.18 to 103.34)	2.76 (1.1 to 5.79)	43.86 (17.55 to 92.18)
Shaanxi	Fungal skin diseases	15.9 (6.31 to 33)	46.24 (18.32 to 95.9)	18.7 (7.49 to 39.2)	47.29 (18.96 to 99.16)
Shandong	Fungal skin diseases	36 (14.2 to 75.2)	42.02 (16.63 to 87.88)	48.1 (19.3 to 99.9)	48.37 (19.43 to 100.47)
Shanghai	Fungal skin diseases	6.28 (2.5 to 13.2)	44.94 (17.88 to 94.63)	13.8 (5.54 to 28.5)	49.55 (19.81 to 102.08)
Shanxi	Fungal skin diseases	12.8 (5.11 to 26.7)	42.43 (16.88 to 88.13)	17.6 (7.06 to 36.7)	45.44 (18.28 to 95.1)
Sichuan	Fungal skin diseases	51.5 (20.4 to 108)	46.13 (18.3 to 96.33)	42 (16.8 to 88.3)	50.25 (20.17 to 105.78)
Tianjin	Fungal skin diseases	3.8 (1.51 to 7.99)	41.32 (16.37 to 86.89)	7.08 (2.84 to 14.6)	46.38 (18.59 to 95.96)
Tibet	Fungal skin diseases	1.29 (0.502 to 2.66)	55.18 (21.56 to 114.14)	1.55 (0.619 to 3.32)	44.9 (17.91 to 95.91)
Xinjiang	Fungal skin diseases	8.03 (3.15 to 16.6)	50.93 (19.97 to 105.14)	10.5 (4.25 to 22)	43.29 (17.45 to 90.48)
Yunnan	Fungal skin diseases	19.3 (7.57 to 40.2)	49.46 (19.41 to 103.16)	22.1 (8.82 to 46.2)	44.96 (17.93 to 93.86)
Zhejiang	Fungal skin diseases	18.5 (7.33 to 38.6)	41.89 (16.62 to 87.55)	29 (11.7 to 60.4)	47.43 (19.1 to 98.73)
China	Viral skin diseases	951 (609 to 1410)	79.45 (50.91 to 117.69)	831 (534 to 1230)	58.81 (37.79 to 86.96)
Taiwan	Viral skin diseases	13.1 (8.42 to 19.7)	64.2 (41.29 to 96.73)	10.4 (6.64 to 15.6)	44 (28.17 to 66.23)

Hong Kong Special Administrative Region of China	Viral skin diseases	3.98<br>(2.55 to 5.9)	69.11<br>(44.23 to 102.39)	4.03<br>(2.59 to 6)	53.61<br>(34.47 to 79.83)
Macau Special Administrative Region of China	Viral skin diseases	0.267<br>(0.17 to 0.397)	75.37<br>(47.91 to 111.74)	0.38<br>(0.244 to 0.564)	55.58<br>(35.73 to 82.53)
Anhui	Viral skin diseases	47.7<br>(30.7 to 71.2)	80.49<br>(51.77 to 120.2)	36.1<br>(23.4 to 53.3)	58.96<br>(38.3 to 87.18)
Beijing	Viral skin diseases	7.7<br>(4.95 to 11.5)	68.68<br>(44.11 to 102.2)	12.2<br>(7.84 to 17.9)	50.57<br>(32.53 to 74.25)
Chongqing	Viral skin diseases	11.3<br>(7.23 to 16.7)	71.84<br>(45.79 to 106.04)	16.1<br>(10.2 to 23.8)	60.08<br>(38.33 to 88.88)
Fujian	Viral skin diseases	26.9<br>(17 to 40.1)	84.96<br>(53.8 to 126.66)	23.2<br>(14.9 to 35)	58.94<br>(37.83 to 88.71)
Gansu	Viral skin diseases	19.1<br>(12.3 to 28.3)	80.95<br>(52.18 to 119.83)	16.1<br>(10.4 to 24)	60.59<br>(39.08 to 90.17)
Guangdong	Viral skin diseases	54.6<br>(35.1 to 81)	82.94<br>(53.3 to 123.07)	68.6<br>(43.9 to 102)	58.7<br>(37.6 to 87.11)
Guangxi	Viral skin diseases	37.5<br>(24 to 55.8)	86.32<br>(55.27 to 128.41)	32<br>(20.4 to 47.4)	66.26<br>(42.28 to 98.18)
Guizhou	Viral skin diseases	34.9<br>(22.3 to 51.4)	105.19<br>(67.1 to 154.91)	27.6<br>(17.9 to 40.5)	81.6<br>(52.94 to 119.77)
Hainan	Viral skin diseases	6.09<br>(3.88 to 9.11)	87.41<br>(55.7 to 130.74)	5.99<br>(3.83 to 8.94)	62.49<br>(39.92 to 93.2)
Hebei	Viral skin diseases	51.1<br>(32.8 to 75.7)	80.29<br>(51.58 to 118.92)	47.6<br>(30.3 to 70.8)	62.22<br>(39.64 to 92.6)
Heilongjiang	Viral skin diseases	28.7<br>(18.3 to 42.5)	78.76<br>(50.08 to 116.64)	19.5<br>(12.6 to 29.1)	49.78<br>(32.06 to 74.19)
Henan	Viral skin diseases	71.1<br>(45.7 to 106)	80.59<br>(51.75 to 119.77)	63.1<br>(40.5 to 93.7)	65.57<br>(42.04 to 97.25)
Hubei	Viral skin diseases	43.9<br>(28.1 to 64.8)	79.03<br>(50.62 to 116.69)	30.1<br>(19.3 to 44.4)	54.39<br>(34.84 to 80.34)
Hunan	Viral skin diseases	49.2<br>(31.2 to 73.3)	79.12<br>(50.19 to 117.81)	41.1<br>(26.4 to 60.9)	61.15<br>(39.25 to 90.68)
Inner Mongolia	Viral skin diseases	18.7<br>(12 to 27.9)	82.32<br>(52.98 to 122.62)	13.9<br>(8.93 to 20.7)	53.8<br>(34.5 to 80.01)
Jiangsu	Viral skin diseases	49.8<br>(31.9 to 73.4)	72.41<br>(46.31 to 106.7)	43.9<br>(27.9 to 64.7)	53.78<br>(34.16 to 79.29)
Jiangxi	Viral skin diseases	33.4<br>(21.2 to 49.6)	84.37<br>(53.74 to 125.4)	30.8<br>(19.6 to 45.7)	64.74<br>(41.24 to 96.24)
Jilin	Viral skin diseases	20.2<br>(12.9 to 30)	77.74<br>(49.74 to 115.78)	14.4<br>(9.31 to 21.4)	51.63<br>(33.36 to 76.57)
Liaoning	Viral skin diseases	29.7<br>(19.1 to 44.3)	72.85<br>(46.88 to 108.51)	22.3<br>(14.2 to 33.1)	50.01<br>(31.79 to 74.15)
Ningxia	Viral skin diseases	4.37<br>(2.8 to 6.48)	88.57<br>(56.68 to 131.18)	4.5<br>(2.89 to 6.68)	64.13<br>(41.11 to 95.19)
Qinghai	Viral skin diseases	4.02<br>(2.54 to 5.97)	84.92<br>(53.56 to 126.09)	3.93<br>(2.51 to 5.87)	62.59<br>(39.93 to 93.5)
Shaanxi	Viral skin diseases	27.6<br>(17.5 to 40.9)	80.1<br>(50.91 to 118.95)	22.3<br>(14.2 to 32.9)	56.4<br>(35.96 to 83.21)
Shandong	Viral skin diseases	65.5<br>(41.8 to 97)	76.54<br>(48.78 to 113.29)	57.2<br>(36.6 to 84.5)	57.51<br>(36.86 to 84.99)
Shanghai	Viral skin diseases	9.02<br>(5.78 to 13.3)	64.57<br>(41.4 to 95.1)	13.9<br>(8.87 to 20.6)	49.76<br>(31.73 to 73.65)
Shanxi	Viral skin diseases	24.3<br>(15.5 to 36.2)	80.41<br>(51.22 to 119.54)	22.6<br>(14.8 to 33.6)	58.45<br>(38.2 to 87)
Sichuan	Viral skin diseases	82.1<br>(53 to 123)	73.61<br>(47.49 to 109.76)	47.4<br>(30.5 to 70.2)	56.77<br>(36.55 to 84.1)
Tianjin	Viral skin diseases	6.64<br>(4.24 to 9.83)	72.14<br>(46.07 to 106.92)	7.68<br>(4.89 to 11.3)	50.3<br>(32.04 to 74.17)
Tibet	Viral skin diseases	2.1<br>(1.33 to 3.13)	90.12<br>(57.19 to 134.13)	2.47<br>(1.56 to 3.66)	71.42<br>(45.1 to 105.82)
Xinjiang	Viral skin diseases	13.7<br>(8.73 to 20.6)	87.17<br>(55.32 to 130.66)	15.5<br>(9.95 to 22.8)	63.69<br>(40.83 to 93.65)
Yunnan	Viral skin diseases	33<br>(21 to 49)	84.69<br>(53.99 to 125.81)	31.1<br>(20 to 46.4)	63.19<br>(40.68 to 94.42)
Zhejiang	Viral skin diseases	32.6<br>(20.7 to 48.7)	73.83<br>(46.87 to 110.33)	33.3<br>(21.3 to 49.3)	54.39<br>(34.86 to 80.53)

China	Acne vulgaris	217<br>(128 to 348)	18.15<br>(10.73 to 29.11)	306<br>(183 to 494)	21.68<br>(12.95 to 35)
Taiwan	Acne vulgaris	4.1<br>(2.39 to 6.8)	20.1<br>(11.72 to 33.32)	6.41<br>(3.85 to 10.4)	27.19<br>(16.31 to 44.06)
Hong Kong Special Administrative Region of China	Acne vulgaris	0.96<br>(0.554 to 1.59)	16.66<br>(9.61 to 27.62)	1.36<br>(0.802 to 2.24)	18.17<br>(10.68 to 29.78)
Macau Special Administrative Region of China	Acne vulgaris	0.0744<br>(0.0439 to 0.12)	20.96<br>(12.36 to 33.78)	0.164<br>(0.0974 to 0.267)	23.91<br>(14.24 to 39.01)
Anhui	Acne vulgaris	10.7<br>(6.38 to 17.1)	18.09<br>(10.76 to 28.93)	11.8<br>(7.05 to 18.9)	19.38<br>(11.54 to 30.95)
Beijing	Acne vulgaris	2.35<br>(1.42 to 3.81)	20.96<br>(12.64 to 33.95)	5.72<br>(3.4 to 9.12)	23.72<br>(14.12 to 37.84)
Chongqing	Acne vulgaris	2.66<br>(1.57 to 4.25)	16.85<br>(9.95 to 26.95)	5.34<br>(3.19 to 8.61)	19.97<br>(11.94 to 32.23)
Fujian	Acne vulgaris	5.79<br>(3.38 to 9.36)	18.3<br>(10.67 to 29.58)	8.37<br>(5 to 13.4)	21.23<br>(12.69 to 34.06)
Gansu	Acne vulgaris	4<br>(2.3 to 6.56)	16.89<br>(9.74 to 27.72)	5.27<br>(3.13 to 8.46)	19.83<br>(11.76 to 31.82)
Guangdong	Acne vulgaris	12.2<br>(7.19 to 19.5)	18.51<br>(10.93 to 29.68)	31.6<br>(18.8 to 50.1)	27.09<br>(16.07 to 42.85)
Guangxi	Acne vulgaris	7.4<br>(4.29 to 12.1)	17.01<br>(9.86 to 27.85)	10.5<br>(6.17 to 17)	21.82<br>(12.77 to 35.1)
Guizhou	Acne vulgaris	5.51<br>(3.21 to 8.9)	16.59<br>(9.66 to 26.81)	7.26<br>(4.36 to 11.5)	21.45<br>(12.89 to 34.11)
Hainan	Acne vulgaris	1.17<br>(0.69 to 1.92)	16.87<br>(9.91 to 27.49)	2.06<br>(1.22 to 3.31)	21.47<br>(12.7 to 34.51)
Hebei	Acne vulgaris	11.1<br>(6.66 to 18)	17.44<br>(10.46 to 28.21)	16.3<br>(9.71 to 26.3)	21.37<br>(12.71 to 34.37)
Heilongjiang	Acne vulgaris	7.61<br>(4.53 to 12.3)	20.86<br>(12.42 to 33.81)	7.44<br>(4.42 to 12)	18.97<br>(11.25 to 30.51)
Henan	Acne vulgaris	15.9<br>(9.35 to 25.4)	18.01<br>(10.59 to 28.77)	22.5<br>(13.3 to 36.2)	23.36<br>(13.84 to 37.63)
Hubei	Acne vulgaris	9.91<br>(5.78 to 15.9)	17.83<br>(10.41 to 28.54)	10.7<br>(6.45 to 17.2)	19.4<br>(11.66 to 31.13)
Hunan	Acne vulgaris	10.9<br>(6.35 to 17.6)	17.53<br>(10.22 to 28.37)	13.9<br>(8.36 to 22.4)	20.77<br>(12.45 to 33.3)
Inner Mongolia	Acne vulgaris	4.23<br>(2.52 to 6.82)	18.62<br>(11.07 to 29.98)	5.27<br>(3.1 to 8.47)	20.36<br>(11.99 to 32.72)
Jiangsu	Acne vulgaris	13.1<br>(7.93 to 21.1)	19.06<br>(11.53 to 30.68)	17.7<br>(10.5 to 28.8)	21.74<br>(12.9 to 35.24)
Jiangxi	Acne vulgaris	6.98<br>(4.12 to 11.2)	17.65<br>(10.41 to 28.4)	10.4<br>(6.28 to 16.7)	21.79<br>(13.21 to 35.07)
Jilin	Acne vulgaris	4.98<br>(2.95 to 8.01)	19.17<br>(11.36 to 30.86)	5.24<br>(3.14 to 8.43)	18.8<br>(11.26 to 30.22)
Liaoning	Acne vulgaris	8.34<br>(5.01 to 13.3)	20.44<br>(12.27 to 32.65)	8.93<br>(5.28 to 14.3)	20.01<br>(11.83 to 32.05)
Ningxia	Acne vulgaris	0.904<br>(0.535 to 1.47)	18.3<br>(10.84 to 29.7)	1.6<br>(0.942 to 2.58)	22.85<br>(13.43 to 36.82)
Qinghai	Acne vulgaris	0.816<br>(0.473 to 1.32)	17.22<br>(9.99 to 27.89)	1.29<br>(0.77 to 2.1)	20.58<br>(12.25 to 33.47)
Shaanxi	Acne vulgaris	5.64<br>(3.32 to 9.21)	16.38<br>(9.64 to 26.75)	7.8<br>(4.6 to 12.5)	19.73<br>(11.65 to 31.76)
Shandong	Acne vulgaris	16.9<br>(9.98 to 27.1)	19.75<br>(11.66 to 31.67)	22.6<br>(13.5 to 36)	22.74<br>(13.59 to 36.27)
Shanghai	Acne vulgaris	2.54<br>(1.54 to 4.04)	18.15<br>(11.03 to 28.88)	6.13<br>(3.61 to 9.77)	21.92<br>(12.93 to 34.95)
Shanxi	Acne vulgaris	5.57<br>(3.34 to 9.05)	18.4<br>(11.02 to 29.88)	8.84<br>(5.22 to 14.3)	22.89<br>(13.52 to 36.99)
Sichuan	Acne vulgaris	19.5<br>(11.4 to 31.9)	17.51<br>(10.22 to 28.57)	16.4<br>(9.94 to 26.7)	19.7<br>(11.91 to 31.98)
Tianjin	Acne vulgaris	1.81<br>(1.09 to 2.93)	19.72<br>(11.83 to 31.82)	3.49<br>(2.06 to 5.62)	22.88<br>(13.5 to 36.85)
Tibet	Acne vulgaris	0.326<br>(0.193 to 0.528)	13.99<br>(8.3 to 22.64)	0.712<br>(0.419 to 1.15)	20.61<br>(12.14 to 33.38)
Xinjiang	Acne vulgaris	2.68<br>(1.58 to 4.35)	17<br>(9.99 to 27.56)	5<br>(2.97 to 7.91)	20.52<br>(12.18 to 32.45)

Yunnan	Acne vulgaris	6.38 (3.76 to 10.5)	16.37 (9.65 to 26.9)	10.4 (6.1 to 16.8)	21.08 (12.41 to 34.09)
Zhejiang	Acne vulgaris	8.32 (4.94 to 13.3)	18.86 (11.2 to 30.06)	13.9 (8.29 to 22.1)	22.64 (13.55 to 36.04)
China	Alopecia areata	75.4 (48.4 to 112)	6.3 (4.04 to 9.39)	96.7 (62.3 to 145)	6.85 (4.41 to 10.24)
Taiwan	Alopecia areata	1.33 (0.829 to 2)	6.5 (4.06 to 9.82)	1.6 (1.02 to 2.37)	6.79 (4.32 to 10.04)
Hong Kong Special Administrative Region of China	Alopecia areata	0.397 (0.252 to 0.595)	6.89 (4.38 to 10.33)	0.512 (0.325 to 0.755)	6.82 (4.33 to 10.05)
Macau Special Administrative Region of China	Alopecia areata	0.0243 (0.0155 to 0.0369)	6.84 (4.38 to 10.41)	0.0498 (0.031 to 0.0759)	7.28 (4.54 to 11.09)
Anhui	Alopecia areata	3.6 (2.28 to 5.37)	6.08 (3.85 to 9.07)	4.06 (2.6 to 6.11)	6.63 (4.25 to 9.99)
Beijing	Alopecia areata	0.779 (0.502 to 1.18)	6.95 (4.48 to 10.5)	1.83 (1.17 to 2.71)	7.57 (4.84 to 11.26)
Chongqing	Alopecia areata	1.03 (0.65 to 1.57)	6.51 (4.12 to 9.95)	1.7 (1.1 to 2.58)	6.37 (4.12 to 9.66)
Fujian	Alopecia areata	1.9 (1.21 to 2.89)	6.01 (3.83 to 9.13)	2.75 (1.73 to 4.17)	6.97 (4.38 to 10.59)
Gansu	Alopecia areata	1.45 (0.936 to 2.18)	6.15 (3.96 to 9.2)	1.79 (1.14 to 2.65)	6.72 (4.3 to 9.95)
Guangdong	Alopecia areata	4.02 (2.54 to 5.98)	6.11 (3.86 to 9.09)	8.32 (5.34 to 12.5)	7.12 (4.57 to 10.68)
Guangxi	Alopecia areata	2.59 (1.64 to 3.94)	5.95 (3.76 to 9.07)	3.15 (2.02 to 4.75)	6.51 (4.19 to 9.83)
Guizhou	Alopecia areata	1.97 (1.25 to 3)	5.92 (3.75 to 9.05)	2.19 (1.41 to 3.25)	6.48 (4.17 to 9.62)
Hainan	Alopecia areata	0.407 (0.257 to 0.62)	5.85 (3.69 to 8.9)	0.65 (0.408 to 0.987)	6.78 (4.25 to 10.3)
Hebei	Alopecia areata	4 (2.54 to 5.97)	6.28 (3.98 to 9.39)	5.12 (3.28 to 7.57)	6.7 (4.29 to 9.9)
Heilongjiang	Alopecia areata	2.38 (1.49 to 3.61)	6.54 (4.09 to 9.9)	2.81 (1.8 to 4.22)	7.17 (4.58 to 10.76)
Henan	Alopecia areata	5.46 (3.49 to 8.26)	6.19 (3.96 to 9.35)	6.3 (4.02 to 9.41)	6.54 (4.17 to 9.77)
Hubei	Alopecia areata	3.52 (2.2 to 5.31)	6.33 (3.96 to 9.56)	3.81 (2.43 to 5.78)	6.89 (4.4 to 10.45)
Hunan	Alopecia areata	3.92 (2.53 to 5.85)	6.3 (4.06 to 9.41)	4.48 (2.82 to 6.77)	6.67 (4.21 to 10.07)
Inner Mongolia	Alopecia areata	1.43 (0.912 to 2.16)	6.28 (4.01 to 9.51)	1.84 (1.16 to 2.79)	7.09 (4.5 to 10.76)
Jiangsu	Alopecia areata	4.56 (2.86 to 6.91)	6.62 (4.16 to 10.04)	5.69 (3.63 to 8.56)	6.96 (4.44 to 10.48)
Jiangxi	Alopecia areata	2.35 (1.46 to 3.54)	5.95 (3.69 to 8.95)	3.15 (1.96 to 4.81)	6.62 (4.12 to 10.11)
Jilin	Alopecia areata	1.69 (1.08 to 2.57)	6.5 (4.15 to 9.91)	1.96 (1.24 to 2.96)	7.05 (4.46 to 10.6)
Liaoning	Alopecia areata	2.76 (1.74 to 4.18)	6.77 (4.28 to 10.24)	3.12 (2.01 to 4.68)	7 (4.5 to 10.48)
Ningxia	Alopecia areata	0.29 (0.184 to 0.442)	5.87 (3.73 to 8.95)	0.475 (0.298 to 0.724)	6.77 (4.25 to 10.31)
Qinghai	Alopecia areata	0.283 (0.179 to 0.436)	5.98 (3.77 to 9.21)	0.432 (0.278 to 0.662)	6.88 (4.43 to 10.53)
Shaanxi	Alopecia areata	2.15 (1.35 to 3.29)	6.25 (3.91 to 9.56)	2.76 (1.79 to 4.16)	6.99 (4.53 to 10.52)
Shandong	Alopecia areata	5.57 (3.52 to 8.26)	6.51 (4.11 to 9.65)	6.76 (4.33 to 10.3)	6.81 (4.35 to 10.36)
Shanghai	Alopecia areata	0.978 (0.617 to 1.47)	7 (4.41 to 10.49)	2.08 (1.33 to 3.11)	7.44 (4.76 to 11.15)
Shanxi	Alopecia areata	1.89 (1.2 to 2.84)	6.24 (3.97 to 9.39)	2.67 (1.7 to 4.02)	6.92 (4.39 to 10.42)
Sichuan	Alopecia areata	7.17 (4.52 to 10.8)	6.42 (4.05 to 9.71)	5.55 (3.5 to 8.36)	6.65 (4.19 to 10.01)
Tianjin	Alopecia areata	0.624 (0.395 to 0.945)	6.78 (4.3 to 10.28)	1.15 (0.717 to 1.72)	7.51 (4.69 to 11.24)

Tibet	Alopecia areata	0.134<br>(0.0869 to 0.202)	5.73<br>(3.73 to 8.68)	0.232<br>(0.152 to 0.356)	6.72<br>(4.39 to 10.3)
Xinjiang	Alopecia areata	0.932<br>(0.588 to 1.41)	5.91<br>(3.73 to 8.94)	1.67<br>(1.06 to 2.51)	6.84<br>(4.35 to 10.31)
Yunnan	Alopecia areata	2.3<br>(1.47 to 3.45)	5.91<br>(3.76 to 8.85)	3.33<br>(2.13 to 5.02)	6.77<br>(4.34 to 10.2)
Zhejiang	Alopecia areata	2.86<br>(1.85 to 4.32)	6.49<br>(4.19 to 9.81)	4.31<br>(2.73 to 6.53)	7.05<br>(4.45 to 10.68)
China	Pruritus	107<br>(50 to 204)	8.94<br>(4.18 to 17.08)	164<br>(78.2 to 306)	11.63<br>(5.53 to 21.64)
Taiwan	Pruritus	1.93<br>(0.904 to 3.64)	9.46<br>(4.43 to 17.84)	2.85<br>(1.35 to 5.33)	12.08<br>(5.74 to 22.6)
Hong Kong Special Administrative Region of China	Pruritus	0.638<br>(0.296 to 1.2)	11.06<br>(5.14 to 20.85)	1.02<br>(0.488 to 1.9)	13.6<br>(6.5 to 25.31)
Macau Special Administrative Region of China	Pruritus	0.0376<br>(0.0178 to 0.0714)	10.58<br>(5.03 to 20.11)	0.0882<br>(0.0413 to 0.164)	12.9<br>(6.04 to 23.97)
Anhui	Pruritus	5.12<br>(2.44 to 9.6)	8.63<br>(4.12 to 16.21)	7.05<br>(3.36 to 13.2)	11.54<br>(5.5 to 21.57)
Beijing	Pruritus	1.13<br>(0.534 to 2.17)	10.11<br>(4.76 to 19.34)	2.97<br>(1.42 to 5.59)	12.32<br>(5.91 to 23.21)
Chongqing	Pruritus	1.43<br>(0.679 to 2.78)	9.08<br>(4.3 to 17.63)	3.21<br>(1.54 to 5.99)	12.02<br>(5.75 to 22.41)
Fujian	Pruritus	2.73<br>(1.27 to 5.16)	8.62<br>(4.03 to 16.3)	4.58<br>(2.18 to 8.66)	11.62<br>(5.53 to 21.97)
Gansu	Pruritus	2<br>(0.942 to 3.77)	8.46<br>(3.98 to 15.95)	2.91<br>(1.37 to 5.36)	10.97<br>(5.17 to 20.16)
Guangdong	Pruritus	5.89<br>(2.75 to 11.1)	8.95<br>(4.18 to 16.83)	13.1<br>(6.15 to 24.4)	11.21<br>(5.26 to 20.91)
Guangxi	Pruritus	3.71<br>(1.74 to 7.07)	8.54<br>(3.99 to 16.26)	5.3<br>(2.53 to 9.97)	10.97<br>(5.23 to 20.63)
Guizhou	Pruritus	2.77<br>(1.31 to 5.25)	8.34<br>(3.94 to 15.83)	3.66<br>(1.77 to 6.81)	10.82<br>(5.23 to 20.12)
Hainan	Pruritus	0.596<br>(0.279 to 1.12)	8.56<br>(4 to 16.07)	1.05<br>(0.496 to 1.94)	10.94<br>(5.18 to 20.25)
Hebei	Pruritus	5.74<br>(2.71 to 11)	9.02<br>(4.26 to 17.21)	8.7<br>(4.15 to 16.1)	11.39<br>(5.43 to 21.08)
Heilongjiang	Pruritus	3.23<br>(1.53 to 6.18)	8.86<br>(4.2 to 16.97)	4.78<br>(2.25 to 8.85)	12.17<br>(5.74 to 22.54)
Henan	Pruritus	7.75<br>(3.63 to 14.9)	8.78<br>(4.11 to 16.87)	10.7<br>(5.04 to 19.7)	11.11<br>(5.23 to 20.43)
Hubei	Pruritus	4.93<br>(2.31 to 9.35)	8.88<br>(4.16 to 16.83)	6.61<br>(3.12 to 12.2)	11.96<br>(5.64 to 22.09)
Hunan	Pruritus	5.52<br>(2.6 to 10.6)	8.87<br>(4.18 to 17.03)	7.71<br>(3.65 to 14.5)	11.48<br>(5.44 to 21.63)
Inner Mongolia	Pruritus	1.95<br>(0.912 to 3.71)	8.58<br>(4.01 to 16.33)	3.07<br>(1.47 to 5.78)	11.86<br>(5.67 to 22.32)
Jiangsu	Pruritus	6.57<br>(3.12 to 12.7)	9.54<br>(4.53 to 18.4)	10.2<br>(4.86 to 18.7)	12.5<br>(5.95 to 22.93)
Jiangxi	Pruritus	3.34<br>(1.57 to 6.48)	8.45<br>(3.97 to 16.39)	5.15<br>(2.42 to 9.77)	10.83<br>(5.09 to 20.55)
Jilin	Pruritus	2.3<br>(1.08 to 4.43)	8.87<br>(4.17 to 17.09)	3.41<br>(1.63 to 6.34)	12.22<br>(5.83 to 22.74)
Liaoning	Pruritus	3.86<br>(1.81 to 7.33)	9.47<br>(4.43 to 17.97)	5.65<br>(2.69 to 10.5)	12.66<br>(6.01 to 23.61)
Ningxia	Pruritus	0.406<br>(0.19 to 0.788)	8.21<br>(3.85 to 15.96)	0.753<br>(0.36 to 1.42)	10.73<br>(5.13 to 20.28)
Qinghai	Pruritus	0.394<br>(0.185 to 0.751)	8.32<br>(3.91 to 15.86)	0.67<br>(0.317 to 1.26)	10.66<br>(5.04 to 20.06)
Shaanxi	Pruritus	3.02<br>(1.41 to 5.75)	8.77<br>(4.09 to 16.72)	4.55<br>(2.17 to 8.54)	11.51<br>(5.5 to 21.61)
Shandong	Pruritus	7.92<br>(3.72 to 14.9)	9.26<br>(4.35 to 17.46)	12.1<br>(5.83 to 22.4)	12.16<br>(5.87 to 22.59)
Shanghai	Pruritus	1.52<br>(0.717 to 2.8)	10.86<br>(5.14 to 20.05)	3.52<br>(1.67 to 6.6)	12.61<br>(5.98 to 23.61)
Shanxi	Pruritus	2.67<br>(1.25 to 5.1)	8.82<br>(4.14 to 16.83)	4.35<br>(2.09 to 8.13)	11.27<br>(5.4 to 21.04)

Sichuan	Pruritus	9.97 (4.64 to 18.8)	8.93 (4.16 to 16.82)	9.87 (4.72 to 18.1)	11.82 (5.65 to 21.71)
Tianjin	Pruritus	0.908 (0.429 to 1.73)	9.87 (4.66 to 18.82)	1.86 (0.886 to 3.51)	12.19 (5.8 to 22.99)
Tibet	Pruritus	0.196 (0.0914 to 0.375)	8.4 (3.92 to 16.08)	0.337 (0.159 to 0.642)	9.74 (4.59 to 18.58)
Xinjiang	Pruritus	1.32 (0.624 to 2.49)	8.39 (3.96 to 15.79)	2.62 (1.24 to 4.88)	10.73 (5.09 to 20.04)
Yunnan	Pruritus	3.28 (1.53 to 6.14)	8.42 (3.92 to 15.75)	5.28 (2.49 to 9.94)	10.75 (5.07 to 20.21)
Zhejiang	Pruritus	4.12 (1.96 to 7.74)	9.35 (4.45 to 17.56)	7.48 (3.55 to 14)	12.22 (5.8 to 22.84)
China	Urticaria	802 (524 to 1140)	67.02 (43.81 to 95.36)	811 (528 to 1150)	57.42 (37.38 to 81.45)
Taiwan	Urticaria	12.6 (8.46 to 18.2)	61.76 (41.48 to 89.38)	12.4 (8.24 to 17.5)	52.78 (34.94 to 74.1)
Hong Kong Special Administrative Region of China	Urticaria	3.28 (2.13 to 4.75)	56.91 (36.95 to 82.44)	3.8 (2.53 to 5.36)	50.58 (33.7 to 71.34)
Macau Special Administrative Region of China	Urticaria	0.237 (0.156 to 0.341)	66.9 (44 to 96.1)	0.39 (0.256 to 0.545)	57.02 (37.5 to 79.67)
Anhui	Urticaria	40.3 (26.2 to 58.2)	68.03 (44.29 to 98.15)	35.3 (23.3 to 50)	57.8 (38.11 to 81.82)
Beijing	Urticaria	7.08 (4.64 to 10.1)	63.12 (41.36 to 89.81)	13.3 (8.84 to 18.9)	55.2 (36.67 to 78.37)
Chongqing	Urticaria	10.3 (6.84 to 14.6)	65.37 (43.34 to 92.44)	15.4 (10.1 to 21.6)	57.59 (37.69 to 80.98)
Fujian	Urticaria	22.2 (14.5 to 31.9)	70.02 (45.8 to 100.83)	23.2 (15.3 to 32.9)	58.84 (38.9 to 83.55)
Gansu	Urticaria	16.3 (10.7 to 23.4)	68.92 (45.25 to 98.91)	15.6 (10.3 to 22)	58.68 (38.58 to 82.66)
Guangdong	Urticaria	45.5 (29.5 to 65.8)	69.11 (44.87 to 99.96)	68.3 (45.2 to 97.7)	58.44 (38.65 to 83.59)
Guangxi	Urticaria	30.1 (19.7 to 43.2)	69.16 (45.39 to 99.38)	29.2 (19.2 to 41.5)	60.48 (39.76 to 85.88)
Guizhou	Urticaria	22.6 (14.7 to 32.3)	68.11 (44.26 to 97.45)	19.8 (12.9 to 28)	58.44 (38.1 to 82.82)
Hainan	Urticaria	4.94 (3.19 to 7.11)	70.98 (45.8 to 102.13)	5.73 (3.75 to 8.13)	59.71 (39.1 to 84.77)
Hebei	Urticaria	43.4 (28.2 to 62.2)	68.2 (44.34 to 97.77)	45.4 (30.2 to 63.9)	59.37 (39.47 to 83.67)
Heilongjiang	Urticaria	24.3 (15.8 to 34.2)	66.56 (43.44 to 93.84)	20.8 (13.7 to 29.7)	53.03 (34.84 to 75.55)
Henan	Urticaria	59.8 (39 to 86)	67.78 (44.2 to 97.48)	58.1 (38.2 to 81.6)	60.37 (39.69 to 84.73)
Hubei	Urticaria	37.3 (24.8 to 53.2)	67.13 (44.58 to 95.81)	30.7 (20.3 to 43.7)	55.51 (36.71 to 78.96)
Hunan	Urticaria	41.5 (27.3 to 59.3)	66.75 (43.97 to 95.34)	38.9 (25.7 to 55.5)	57.87 (38.31 to 82.7)
Inner Mongolia	Urticaria	15.7 (10.3 to 22.4)	68.84 (45.48 to 98.44)	14.4 (9.33 to 20.4)	55.68 (36.05 to 78.89)
Jiangsu	Urticaria	44 (28.7 to 62.7)	63.92 (41.73 to 91.12)	45.1 (30 to 64.2)	55.28 (36.8 to 78.57)
Jiangxi	Urticaria	27.4 (18 to 39.2)	69.33 (45.63 to 99.06)	28.7 (18.8 to 40)	60.28 (39.64 to 84.2)
Jilin	Urticaria	17.4 (11.5 to 24.9)	67.23 (44.13 to 95.87)	15.2 (9.87 to 21.5)	54.46 (35.4 to 77.1)
Liaoning	Urticaria	26.3 (17.3 to 37.2)	64.45 (42.46 to 91.21)	23.7 (15.7 to 33.3)	53.15 (35.22 to 74.54)
Ningxia	Urticaria	3.54 (2.31 to 5.03)	71.6 (46.77 to 101.91)	4.23 (2.76 to 6.03)	60.28 (39.37 to 85.96)
Qinghai	Urticaria	3.31 (2.17 to 4.74)	69.9 (45.73 to 100.08)	3.74 (2.45 to 5.37)	59.55 (38.99 to 85.48)
Shaanxi	Urticaria	23.6 (15.4 to 33.5)	68.47 (44.79 to 97.38)	22.6 (14.8 to 31.7)	57.16 (37.57 to 80.28)
Shandong	Urticaria	56 (36.8 to 80.1)	65.38 (42.94 to 93.52)	56.2 (36.6 to 79.9)	56.52 (36.88 to 80.43)

Shanghai	Urticaria	8.56 (5.63 to 12.1)	61.24 (40.29 to 86.88)	15.2 (9.97 to 21.7)	54.26 (35.68 to 77.78)
Shanxi	Urticaria	20.7 (13.5 to 29.4)	68.24 (44.57 to 97.08)	22.3 (14.6 to 31.3)	57.68 (37.71 to 80.98)
Sichuan	Urticaria	72.2 (47.9 to 103)	64.65 (42.94 to 92.32)	47 (31.3 to 66.4)	56.29 (37.51 to 79.55)
Tianjin	Urticaria	5.98 (3.92 to 8.6)	65.04 (42.66 to 93.48)	8.34 (5.47 to 11.8)	54.64 (35.87 to 77.28)
Tibet	Urticaria	1.68 (1.09 to 2.41)	72.19 (46.72 to 103.35)	2.22 (1.46 to 3.15)	64.3 (42.28 to 91.07)
Xinjiang	Urticaria	11.1 (7.24 to 15.9)	70.63 (45.93 to 101.11)	14.8 (9.69 to 20.7)	60.68 (39.75 to 85.09)
Yunnan	Urticaria	27.1 (17.6 to 38.3)	69.46 (45.18 to 98.36)	29.3 (19.3 to 41.1)	59.51 (39.22 to 83.58)
Zhejiang	Urticaria	28.7 (18.9 to 40.6)	65.18 (42.85 to 92.18)	34.3 (22.3 to 48.4)	56.06 (36.5 to 79.03)
China	Decubitus ulcer	38.5 (21.1 to 50.8)	3.21 (1.76 to 4.24)	50.9 (37.6 to 72.8)	3.6 (2.66 to 5.15)
Taiwan	Decubitus ulcer	4.42 (2.27 to 6.59)	21.65 (11.15 to 32.28)	5.56 (3.78 to 12.8)	23.59 (16.02 to 54.26)
Hong Kong Special Administrative Region of China	Decubitus ulcer	0.122 (0.0888 to 0.191)	2.11 (1.54 to 3.32)	0.42 (0.312 to 0.658)	5.59 (4.16 to 8.76)
Macau Special Administrative Region of China	Decubitus ulcer	0.00589 (0.00429 to 0.00973)	1.66 (1.21 to 2.74)	0.0129 (0.00926 to 0.0216)	1.88 (1.35 to 3.15)
Anhui	Decubitus ulcer	2.8 (0.923 to 3.83)	4.73 (1.56 to 6.46)	2.99 (1.93 to 3.95)	4.9 (3.16 to 6.46)
Beijing	Decubitus ulcer	0.3 (0.224 to 0.444)	2.67 (2 to 3.96)	0.68 (0.478 to 1.04)	2.82 (1.98 to 4.31)
Chongqing	Decubitus ulcer	0.237 (0.171 to 0.378)	1.5 (1.08 to 2.4)	0.7 (0.486 to 1.07)	2.62 (1.82 to 4)
Fujian	Decubitus ulcer	2.35 (0.585 to 3.55)	7.43 (1.85 to 11.22)	2.93 (1.43 to 3.98)	7.43 (3.63 to 10.1)
Gansu	Decubitus ulcer	0.413 (0.289 to 0.73)	1.74 (1.22 to 3.09)	0.482 (0.348 to 0.774)	1.81 (1.31 to 2.91)
Guangdong	Decubitus ulcer	2.16 (1.39 to 3.36)	3.28 (2.12 to 5.11)	3.65 (2.76 to 5.42)	3.12 (2.36 to 4.64)
Guangxi	Decubitus ulcer	1.45 (0.92 to 2.37)	3.34 (2.11 to 5.45)	1.99 (1.48 to 2.89)	4.11 (3.05 to 5.99)
Guizhou	Decubitus ulcer	1.27 (0.623 to 2.41)	3.82 (1.88 to 7.27)	1.65 (0.95 to 2.28)	4.87 (2.81 to 6.75)
Hainan	Decubitus ulcer	0.219 (0.129 to 0.306)	3.14 (1.85 to 4.39)	0.392 (0.254 to 0.54)	4.09 (2.65 to 5.63)
Hebei	Decubitus ulcer	1.91 (1.11 to 2.62)	3.01 (1.74 to 4.12)	2.8 (1.85 to 4.15)	3.66 (2.43 to 5.43)
Heilongjiang	Decubitus ulcer	0.429 (0.3 to 0.736)	1.18 (0.82 to 2.02)	0.684 (0.46 to 1.2)	1.74 (1.17 to 3.06)
Henan	Decubitus ulcer	2.23 (1.3 to 3.03)	2.52 (1.48 to 3.44)	2.79 (2.04 to 4.52)	2.89 (2.12 to 4.69)
Hubei	Decubitus ulcer	1.12 (0.815 to 1.73)	2.02 (1.47 to 3.12)	1.63 (1.18 to 2.67)	2.95 (2.14 to 4.82)
Hunan	Decubitus ulcer	1.16 (0.803 to 2.16)	1.86 (1.29 to 3.47)	2.07 (1.5 to 3.45)	3.09 (2.23 to 5.13)
Inner Mongolia	Decubitus ulcer	0.393 (0.286 to 0.628)	1.73 (1.26 to 2.76)	0.532 (0.378 to 0.92)	2.06 (1.46 to 3.55)
Jiangsu	Decubitus ulcer	4.77 (1.76 to 6.75)	6.93 (2.55 to 9.81)	5.21 (3.37 to 7.09)	6.38 (4.13 to 8.68)
Jiangxi	Decubitus ulcer	1.64 (0.865 to 2.55)	4.14 (2.19 to 6.44)	1.72 (1.22 to 2.34)	3.63 (2.58 to 4.92)
Jilin	Decubitus ulcer	0.409 (0.297 to 0.657)	1.57 (1.15 to 2.53)	0.59 (0.415 to 0.947)	2.12 (1.49 to 3.39)
Liaoning	Decubitus ulcer	0.913 (0.674 to 1.39)	2.24 (1.65 to 3.41)	1.25 (0.89 to 2.2)	2.81 (1.99 to 4.93)
Ningxia	Decubitus ulcer	0.0737 (0.0505 to 0.111)	1.49 (1.02 to 2.25)	0.113 (0.081 to 0.177)	1.61 (1.15 to 2.52)
Qinghai	Decubitus ulcer	0.105 (0.0684 to 0.199)	2.22 (1.44 to 4.19)	0.147 (0.108 to 0.225)	2.34 (1.71 to 3.58)

Shaanxi	Decubitus ulcer	0.602<br>(0.351 to 0.961)	1.75<br>(1.02 to 2.79)	0.992<br>(0.72 to 1.56)	2.51<br>(1.82 to 3.94)
Shandong	Decubitus ulcer	2.87<br>(1.74 to 3.86)	3.35<br>(2.03 to 4.51)	3.38<br>(2.45 to 5.78)	3.4<br>(2.46 to 5.81)
Shanghai	Decubitus ulcer	0.598<br>(0.4 to 0.858)	4.28<br>(2.86 to 6.14)	1.09<br>(0.824 to 1.58)	3.91<br>(2.95 to 5.66)
Shanxi	Decubitus ulcer	0.689<br>(0.482 to 1.09)	2.28<br>(1.59 to 3.59)	0.849<br>(0.618 to 1.34)	2.2<br>(1.6 to 3.47)
Sichuan	Decubitus ulcer	2.15<br>(1.53 to 3.4)	1.92<br>(1.37 to 3.05)	2.45<br>(1.82 to 3.81)	2.93<br>(2.18 to 4.56)
Tianjin	Decubitus ulcer	0.149<br>(0.106 to 0.252)	1.62<br>(1.15 to 2.74)	0.29<br>(0.196 to 0.538)	1.9<br>(1.29 to 3.53)
Tibet	Decubitus ulcer	0.0869<br>(0.0446 to 0.149)	3.73<br>(1.91 to 6.4)	0.088<br>(0.062 to 0.113)	2.54<br>(1.8 to 3.26)
Xinjiang	Decubitus ulcer	0.204<br>(0.144 to 0.351)	1.3<br>(0.92 to 2.23)	0.407<br>(0.284 to 0.705)	1.67<br>(1.17 to 2.89)
Yunnan	Decubitus ulcer	1.64<br>(0.732 to 2.96)	4.2<br>(1.88 to 7.6)	1.96<br>(1.19 to 2.62)	3.99<br>(2.43 to 5.33)
Zhejiang	Decubitus ulcer	3.01<br>(1.05 to 4.59)	6.82<br>(2.39 to 10.4)	3.96<br>(2.44 to 5.06)	6.48<br>(3.99 to 8.28)
China	Other skin and subcutaneous diseases	324<br>(162 to 588)	27.07<br>(13.56 to 49.13)	634<br>(313 to 1160)	44.87<br>(22.13 to 82.05)
Taiwan	Other skin and subcutaneous diseases	10.7<br>(6.5 to 17.2)	52.57<br>(31.87 to 84.42)	16.1<br>(8.43 to 27.5)	68.21<br>(35.75 to 116.47)
Hong Kong Special Administrative Region of China	Other skin and subcutaneous diseases	2.5<br>(1.25 to 4.5)	43.34<br>(21.62 to 78.04)	4.59<br>(2.25 to 8.35)	61.15<br>(30 to 111.2)
Macau Special Administrative Region of China	Other skin and subcutaneous diseases	0.149<br>(0.0751 to 0.268)	41.93<br>(21.16 to 75.43)	0.411<br>(0.204 to 0.74)	60.08<br>(29.82 to 108.19)
Anhui	Other skin and subcutaneous diseases	15.6<br>(7.89 to 27.9)	26.29<br>(13.32 to 47.09)	26.4<br>(12.9 to 48.4)	43.11<br>(21.13 to 79.09)
Beijing	Other skin and subcutaneous diseases	3.89<br>(1.96 to 7.04)	34.64<br>(17.5 to 62.8)	12.3<br>(6.14 to 22.4)	51.06<br>(25.46 to 93.06)
Chongqing	Other skin and subcutaneous diseases	4.23<br>(2.1 to 7.7)	26.79<br>(13.29 to 48.81)	12.6<br>(6.17 to 23.1)	47.26<br>(23.09 to 86.41)
Fujian	Other skin and subcutaneous diseases	8.3<br>(4.13 to 15)	26.25<br>(13.04 to 47.55)	18.1<br>(8.76 to 32.9)	45.85<br>(22.22 to 83.49)
Gansu	Other skin and subcutaneous diseases	5.97<br>(2.99 to 10.7)	25.23<br>(12.66 to 45.19)	10.4<br>(5.07 to 19)	39.05<br>(19.09 to 71.48)
Guangdong	Other skin and subcutaneous diseases	18.3<br>(9.25 to 33.5)	27.85<br>(14.06 to 50.87)	51.1<br>(24.9 to 93.1)	43.72<br>(21.29 to 79.69)
Guangxi	Other skin and subcutaneous diseases	10.9<br>(5.4 to 19.9)	25.01<br>(12.42 to 45.72)	19.6<br>(9.58 to 35.7)	40.54<br>(19.82 to 73.84)
Guizhou	Other skin and subcutaneous diseases	7.82<br>(3.92 to 14.2)	23.55<br>(11.81 to 42.92)	12.9<br>(6.3 to 23.4)	38.08<br>(18.62 to 69.31)
Hainan	Other skin and subcutaneous diseases	1.8<br>(0.9 to 3.27)	25.82<br>(12.92 to 46.89)	3.85<br>(1.89 to 7.09)	40.2<br>(19.73 to 73.92)
Hebei	Other skin and subcutaneous diseases	17.3<br>(8.61 to 31.4)	27.22<br>(13.53 to 49.33)	33.6<br>(16.4 to 61.2)	43.94<br>(21.44 to 80.13)
Heilongjiang	Other skin and subcutaneous diseases	9.63<br>(4.81 to 17.6)	26.42<br>(13.2 to 48.35)	18.2<br>(8.95 to 33.3)	46.38<br>(22.81 to 84.92)
Henan	Other skin and subcutaneous diseases	22.6<br>(11.3 to 41.1)	25.55<br>(12.79 to 46.57)	39.8<br>(19.5 to 72.9)	41.3<br>(20.26 to 75.68)
Hubei	Other skin and subcutaneous diseases	14.6<br>(7.24 to 26.7)	26.22<br>(13.04 to 48)	25.3<br>(12.4 to 46.5)	45.7<br>(22.38 to 84.13)
Hunan	Other skin and subcutaneous diseases	16.2<br>(8.07 to 29.4)	26.07<br>(12.98 to 47.25)	29.1<br>(14.3 to 53.5)	43.26<br>(21.34 to 79.67)
Inner Mongolia	Other skin and subcutaneous diseases	5.8<br>(2.91 to 10.5)	25.51<br>(12.81 to 45.97)	11.8<br>(5.83 to 21.6)	45.66<br>(22.52 to 83.47)
Jiangsu	Other skin and subcutaneous diseases	20.6<br>(10.1 to 37.4)	29.87<br>(14.63 to 54.28)	42.1<br>(20.6 to 76.7)	51.59<br>(25.21 to 93.93)
Jiangxi	Other skin and subcutaneous diseases	9.97<br>(5.05 to 18.1)	25.22<br>(12.77 to 45.68)	18.6<br>(9.08 to 34)	39.05<br>(19.1 to 71.62)
Jilin	Other skin and subcutaneous diseases	6.83<br>(3.41 to 12.4)	26.3<br>(13.13 to 47.69)	13.2<br>(6.48 to 24.2)	47.32<br>(23.25 to 86.64)
Liaoning	Other skin and subcutaneous diseases	12.1<br>(6.01 to 22)	29.77<br>(14.74 to 54.05)	23<br>(11.4 to 41.9)	51.53<br>(25.58 to 93.78)

Ningxia	Other skin and subcutaneous diseases	1.27 (0.67 to 2.22)	25.68 (13.56 to 44.87)	2.72 (1.34 to 4.95)	38.71 (19.06 to 70.53)
Qinghai	Other skin and subcutaneous diseases	1.14 (0.572 to 2.09)	24.1 (12.07 to 44.14)	2.35 (1.16 to 4.29)	37.34 (18.44 to 68.31)
Shaanxi	Other skin and subcutaneous diseases	8.9 (4.44 to 16.3)	25.87 (12.89 to 47.23)	16.9 (8.33 to 30.7)	42.89 (21.08 to 77.72)
Shandong	Other skin and subcutaneous diseases	23.9 (11.8 to 43.6)	27.93 (13.77 to 50.92)	48.6 (23.8 to 88.7)	48.92 (23.95 to 89.24)
Shanghai	Other skin and subcutaneous diseases	5.5 (2.76 to 9.9)	39.39 (19.74 to 70.86)	14.8 (7.35 to 26.9)	52.94 (26.29 to 96.44)
Shanxi	Other skin and subcutaneous diseases	7.98 (3.99 to 14.5)	26.37 (13.18 to 48.02)	16.1 (7.84 to 29.3)	41.56 (20.29 to 75.86)
Sichuan	Other skin and subcutaneous diseases	29.6 (14.9 to 53.4)	26.5 (13.37 to 47.88)	37.7 (18.4 to 69.2)	45.16 (22.03 to 82.86)
Tianjin	Other skin and subcutaneous diseases	2.99 (1.49 to 5.44)	32.51 (16.21 to 59.12)	7.55 (3.73 to 13.7)	49.49 (24.42 to 89.69)
Tibet	Other skin and subcutaneous diseases	0.652 (0.344 to 1.14)	27.97 (14.75 to 49.03)	1.16 (0.584 to 2.09)	33.55 (16.91 to 60.39)
Xinjiang	Other skin and subcutaneous diseases	4.06 (2.08 to 7.29)	25.73 (13.21 to 46.24)	9.56 (4.76 to 17.4)	39.24 (19.54 to 71.61)
Yunnan	Other skin and subcutaneous diseases	10.3 (5.28 to 18.2)	26.38 (13.54 to 46.71)	19 (9.39 to 34.5)	38.7 (19.09 to 70.27)
Zhejiang	Other skin and subcutaneous diseases	12.7 (6.16 to 23.3)	28.9 (13.97 to 52.77)	30.5 (14.9 to 55.8)	49.83 (24.42 to 91.19)
China	Sense organ diseases	7950 (5300 to 11600)	664.39 (442.49 to 970.41)	14200 (9470 to 20700)	1007.04 (670.51 to 1462.16)
Taiwan	Sense organ diseases	122 (78.9 to 183)	596.75 (386.51 to 896.02)	218 (142 to 324)	925.53 (603.2 to 1375.06)
Hong Kong Special Administrative Region of China	Sense organ diseases	44.6 (29.7 to 65.1)	773.04 (515.7 to 1129.76)	87.8 (58.5 to 127)	1169.42 (779.57 to 1694.72)
Macau Special Administrative Region of China	Sense organ diseases	2.33 (1.55 to 3.39)	656.26 (435.58 to 954.89)	5.93 (3.9 to 8.74)	867.84 (570.9 to 1278.26)
Anhui	Sense organ diseases	386 (259 to 562)	651.69 (437.66 to 948.2)	658 (440 to 956)	1075.76 (720.18 to 1563.82)
Beijing	Sense organ diseases	76.6 (50.9 to 112)	682.89 (453.73 to 1000.85)	216 (143 to 317)	898.25 (594.89 to 1313.57)
Chongqing	Sense organ diseases	118 (78.4 to 170)	745.06 (496.81 to 1078.86)	331 (224 to 472)	1237.57 (836.91 to 1765.93)
Fujian	Sense organ diseases	188 (126 to 273)	594.85 (398.05 to 863.91)	359 (239 to 525)	910.71 (607.43 to 1330.84)
Gansu	Sense organ diseases	142 (94.1 to 208)	600.35 (397.74 to 880.73)	257 (172 to 375)	968.46 (647.35 to 1412.16)
Guangdong	Sense organ diseases	437 (292 to 635)	664.11 (443.77 to 965.38)	1010 (673 to 1470)	865.04 (576.02 to 1258.21)
Guangxi	Sense organ diseases	287 (194 to 414)	660.83 (445.13 to 952.39)	473 (317 to 678)	978.48 (656.02 to 1403.27)
Guizhou	Sense organ diseases	204 (136 to 296)	614.19 (410.25 to 893.27)	330 (221 to 476)	974.67 (654.05 to 1406.08)
Hainan	Sense organ diseases	47.4 (32 to 67.7)	680.25 (458.97 to 971.24)	94.2 (63.4 to 135)	982.29 (661.58 to 1404.56)
Hebei	Sense organ diseases	423 (283 to 618)	664.09 (444.3 to 970.33)	707 (470 to 1040)	924.66 (615.11 to 1355.46)
Heilongjiang	Sense organ diseases	215 (144 to 315)	591 (394.99 to 864.95)	428 (285 to 621)	1089.49 (726.93 to 1583.17)
Henan	Sense organ diseases	612 (409 to 890)	693.29 (462.82 to 1008.02)	925 (616 to 1350)	960.85 (640.16 to 1399.13)
Hubei	Sense organ diseases	444 (297 to 652)	799.71 (535.35 to 1174.26)	734 (488 to 1070)	1327.16 (882.73 to 1935.2)
Hunan	Sense organ diseases	424 (282 to 618)	682.4 (453.96 to 994.39)	697 (467 to 1010)	1038.68 (695.31 to 1501.94)
Inner Mongolia	Sense organ diseases	130 (86.5 to 191)	572.37 (380.29 to 841.46)	250 (166 to 364)	964.62 (641.79 to 1407.19)
Jiangsu	Sense organ diseases	504 (337 to 729)	732.66 (489.78 to 1059.18)	888 (592 to 1290)	1086.93 (724.51 to 1580.61)
Jiangxi	Sense organ diseases	243 (161 to 355)	613.97 (408.19 to 898.67)	431 (288 to 629)	907.43 (605.27 to 1323.92)

Jilin	Sense organ diseases	153 (102 to 220)	587.83 (391.88 to 848.2)	293 (196 to 426)	1049.78 (703.01 to 1525.69)
Liaoning	Sense organ diseases	271 (181 to 395)	665.54 (443.02 to 968.55)	506 (339 to 733)	1132.82 (759.52 to 1642.53)
Ningxia	Sense organ diseases	26.2 (17.5 to 38.2)	529.73 (355.18 to 772.68)	58.7 (39.2 to 85.8)	836.98 (558.68 to 1222.62)
Qinghai	Sense organ diseases	25.3 (16.8 to 37.2)	534.38 (354.39 to 786.06)	51.2 (34 to 74.9)	815.17 (540.87 to 1191.6)
Shaanxi	Sense organ diseases	212 (142 to 307)	615.99 (412.84 to 893.26)	385 (256 to 559)	974.2 (648.47 to 1415.96)
Shandong	Sense organ diseases	600 (394 to 892)	700.88 (460.8 to 1041.58)	1090 (709 to 1620)	1098.8 (713.77 to 1626.48)
Shanghai	Sense organ diseases	130 (85.3 to 193)	932.29 (610.17 to 1378.78)	322 (210 to 478)	1152.52 (753.13 to 1712.36)
Shanxi	Sense organ diseases	197 (132 to 287)	650.08 (434.46 to 949.18)	359 (239 to 523)	928.39 (618.64 to 1353.5)
Sichuan	Sense organ diseases	690 (459 to 1020)	618.4 (410.98 to 912.62)	851 (567 to 1250)	1019.65 (679.03 to 1494.76)
Tianjin	Sense organ diseases	61.8 (41.2 to 91.2)	672.45 (448.2 to 991.88)	141 (94.2 to 206)	925.55 (617.51 to 1346.65)
Tibet	Sense organ diseases	15.7 (10.7 to 22.3)	672.92 (457.9 to 957.66)	26.4 (17.6 to 38.1)	762.58 (508.12 to 1101.8)
Xinjiang	Sense organ diseases	86.4 (57.5 to 127)	547.56 (364.6 to 802.22)	194 (129 to 284)	795.03 (529.58 to 1164.95)
Yunnan	Sense organ diseases	256 (173 to 368)	658.12 (442.81 to 944.51)	466 (311 to 674)	947.9 (633.41 to 1371.35)
Zhejiang	Sense organ diseases	299 (200 to 431)	677.09 (453.28 to 977.1)	597 (399 to 867)	976 (652.88 to 1416.84)
China	Age-related and other hearing loss	4500 (3030 to 6450)	375.73 (253.06 to 539.03)	8150 (5470 to 11700)	577.21 (387.14 to 826.67)
Taiwan	Age-related and other hearing loss	82.3 (54.9 to 119)	403.62 (269.18 to 583.74)	152 (102 to 220)	646.57 (430.54 to 931.14)
Hong Kong Special Administrative Region of China	Age-related and other hearing loss	26.2 (17.6 to 37.9)	455.04 (304.48 to 656.93)	52.2 (35.1 to 74.8)	694.74 (467.59 to 995.74)
Macau Special Administrative Region of China	Age-related and other hearing loss	1.37 (0.917 to 1.98)	385.9 (258.34 to 559.31)	3.5 (2.29 to 5.16)	511.69 (335.48 to 755.22)
Anhui	Age-related and other hearing loss	216 (145 to 310)	363.79 (244.91 to 522.71)	378 (255 to 541)	617.69 (416.39 to 885.02)
Beijing	Age-related and other hearing loss	47.7 (31.8 to 68.7)	425 (283.13 to 612.88)	136 (90.2 to 196)	562.62 (374.24 to 812.01)
Chongqing	Age-related and other hearing loss	63 (42.3 to 90.4)	399.46 (267.94 to 573.1)	182 (123 to 258)	679.25 (460.76 to 964.94)
Fujian	Age-related and other hearing loss	111 (74.8 to 159)	350.46 (236.3 to 501.95)	215 (143 to 310)	544.92 (361.63 to 787.76)
Gansu	Age-related and other hearing loss	79 (53.3 to 114)	334.01 (225.24 to 481.35)	147 (98.4 to 212)	553.93 (370.17 to 796.19)
Guangdong	Age-related and other hearing loss	245 (164 to 350)	372.11 (249.72 to 531.96)	568 (379 to 819)	486.37 (324.63 to 701.37)
Guangxi	Age-related and other hearing loss	157 (106 to 224)	361.03 (243.33 to 515.37)	265 (178 to 378)	548.8 (369.24 to 783.2)
Guizhou	Age-related and other hearing loss	116 (78.7 to 166)	350.15 (237.2 to 500.47)	192 (129 to 272)	566.37 (382.66 to 805.28)
Hainan	Age-related and other hearing loss	24.4 (16.5 to 34.9)	350.55 (237.19 to 501.34)	51.1 (34.6 to 73.1)	532.63 (360.8 to 762.26)
Hebei	Age-related and other hearing loss	248 (168 to 355)	388.92 (263.33 to 557.94)	422 (283 to 610)	551.9 (369.66 to 798.25)
Heilongjiang	Age-related and other hearing loss	122 (81.3 to 178)	335.76 (223.13 to 488.27)	246 (163 to 355)	625.49 (415.77 to 904.41)
Henan	Age-related and other hearing loss	334 (225 to 478)	378.09 (255.2 to 541.5)	520 (349 to 746)	540.48 (362.33 to 774.79)
Hubei	Age-related and other hearing loss	223 (153 to 315)	401.02 (274.76 to 567.35)	379 (257 to 537)	686.11 (464.36 to 971.54)
Hunan	Age-related and other hearing loss	235 (157 to 334)	377.24 (253.07 to 537.69)	396 (266 to 569)	589.36 (396.35 to 847.15)
Inner Mongolia	Age-related and other hearing loss	75.6 (50.3 to 109)	332.52 (221.2 to 477.21)	148 (98.3 to 214)	570.86 (379.79 to 827)

Jiangsu	Age-related and other hearing loss	289<br>(193 to 414)	419.28<br>(280.58 to 601)	520<br>(350 to 745)	637.04<br>(428.22 to 912.7)
Jiangxi	Age-related and other hearing loss	135<br>(91.3 to 195)	342.67<br>(230.98 to 493.99)	250<br>(168 to 359)	525.62<br>(352.54 to 755.51)
Jilin	Age-related and other hearing loss	91.5<br>(61.4 to 131)	352.61<br>(236.8 to 506.44)	176<br>(117 to 254)	630.96<br>(419.9 to 911.06)
Liaoning	Age-related and other hearing loss	160<br>(107 to 231)	392.25<br>(262.99 to 565.1)	299<br>(200 to 430)	669.78<br>(447.44 to 962.12)
Ningxia	Age-related and other hearing loss	15.2<br>(10.2 to 21.9)	307.94<br>(207.39 to 443.93)	34.6<br>(23 to 49.7)	492.41<br>(327.6 to 707.44)
Qinghai	Age-related and other hearing loss	14.6<br>(9.78 to 21.1)	308.67<br>(206.43 to 445.01)	30.1<br>(20 to 43.5)	479.88<br>(318.56 to 692.88)
Shaanxi	Age-related and other hearing loss	120<br>(81.9 to 170)	349.2<br>(238.03 to 494.76)	223<br>(150 to 317)	563.76<br>(379.72 to 802.22)
Shandong	Age-related and other hearing loss	317<br>(212 to 459)	370.56<br>(247.78 to 536.13)	568<br>(379 to 820)	571.94<br>(381.25 to 825.05)
Shanghai	Age-related and other hearing loss	69.7<br>(46.7 to 99.6)	498.96<br>(334.53 to 712.92)	173<br>(116 to 247)	617.41<br>(415.1 to 885.47)
Shanxi	Age-related and other hearing loss	112<br>(75.4 to 159)	369.39<br>(248.93 to 526.34)	214<br>(144 to 307)	553.59<br>(372.86 to 793.62)
Sichuan	Age-related and other hearing loss	437<br>(294 to 627)	391.36<br>(263.24 to 562.24)	536<br>(361 to 767)	642.4<br>(432.01 to 918.64)
Tianjin	Age-related and other hearing loss	38.1<br>(25.4 to 55.1)	414.12<br>(275.77 to 598.68)	87<br>(57.9 to 126)	569.98<br>(379.28 to 827.92)
Tibet	Age-related and other hearing loss	7.88<br>(5.32 to 11.3)	338.33<br>(228.46 to 484.7)	14<br>(9.36 to 20.3)	406.41<br>(270.93 to 586.28)
Xinjiang	Age-related and other hearing loss	50.5<br>(33.9 to 72.7)	320<br>(214.69 to 461.08)	116<br>(77 to 167)	475.57<br>(315.98 to 685.46)
Yunnan	Age-related and other hearing loss	135<br>(91.2 to 193)	347.27<br>(233.98 to 495.08)	253<br>(170 to 363)	515.49<br>(345.25 to 737.28)
Zhejiang	Age-related and other hearing loss	182<br>(123 to 260)	411.88<br>(278.16 to 589.37)	360<br>(241 to 517)	587.71<br>(394.38 to 844.61)
China	Other sense organ diseases	303<br>(188 to 446)	25.33<br>(15.67 to 37.26)	560<br>(346 to 815)	39.62<br>(24.48 to 57.7)
Taiwan	Other sense organ diseases	5.69<br>(3.52 to 8.19)	27.89<br>(17.27 to 40.16)	10.7<br>(6.56 to 15.5)	45.27<br>(27.81 to 65.68)
Hong Kong Special Administrative Region of China	Other sense organ diseases	1.87<br>(1.15 to 2.71)	32.51<br>(19.98 to 46.99)	3.7<br>(2.29 to 5.38)	49.23<br>(30.51 to 71.6)
Macau Special Administrative Region of China	Other sense organ diseases	0.101<br>(0.0618 to 0.148)	28.34<br>(17.43 to 41.7)	0.269<br>(0.166 to 0.39)	39.36<br>(24.21 to 57.03)
Anhui	Other sense organ diseases	14.4<br>(8.79 to 21.1)	24.32<br>(14.84 to 35.54)	25<br>(15.3 to 36.6)	40.86<br>(25.07 to 59.86)
Beijing	Other sense organ diseases	3.33<br>(2.03 to 4.85)	29.65<br>(18.07 to 43.23)	9.67<br>(5.93 to 14.3)	40.14<br>(24.59 to 59.21)
Chongqing	Other sense organ diseases	4.18<br>(2.56 to 6.12)	26.46<br>(16.25 to 38.75)	12.1<br>(7.45 to 17.6)	45.13<br>(27.86 to 65.93)
Fujian	Other sense organ diseases	7.52<br>(4.61 to 11)	23.78<br>(14.58 to 34.62)	14.8<br>(9.22 to 21.4)	37.51<br>(23.4 to 54.29)
Gansu	Other sense organ diseases	5.27<br>(3.23 to 7.83)	22.29<br>(13.65 to 33.1)	9.83<br>(6.08 to 14.3)	36.99<br>(22.87 to 53.67)
Guangdong	Other sense organ diseases	16.7<br>(10.3 to 24.3)	25.38<br>(15.64 to 36.94)	39.5<br>(24.3 to 57.7)	33.82<br>(20.83 to 49.36)
Guangxi	Other sense organ diseases	10.5<br>(6.51 to 15.4)	24.21<br>(14.98 to 35.46)	17.9<br>(11 to 26.1)	37.09<br>(22.79 to 53.99)
Guizhou	Other sense organ diseases	7.58<br>(4.65 to 11.2)	22.84<br>(14.01 to 33.72)	12.6<br>(7.72 to 18.4)	37.18<br>(22.84 to 54.52)
Hainan	Other sense organ diseases	1.67<br>(1.02 to 2.45)	24<br>(14.67 to 35.16)	3.42<br>(2.13 to 5)	35.7<br>(22.18 to 52.19)
Hebei	Other sense organ diseases	16.6<br>(10.2 to 24.4)	26.12<br>(16.1 to 38.3)	29.6<br>(18.2 to 43)	38.7<br>(23.8 to 56.3)
Heilongjiang	Other sense organ diseases	8.35<br>(5.05 to 12.3)	22.92<br>(13.86 to 33.66)	17<br>(10.5 to 24.9)	43.23<br>(26.76 to 63.35)
Henan	Other sense organ diseases	22.4<br>(13.9 to 33)	25.37<br>(15.78 to 37.44)	36<br>(22.3 to 53.4)	37.37<br>(23.18 to 55.47)
Hubei	Other sense organ diseases	13.9<br>(8.66 to 20.2)	25.01<br>(15.59 to 36.41)	23.6<br>(14.6 to 34.1)	42.59<br>(26.35 to 61.62)

Hunan	Other sense organ diseases	15.8<br>(9.72 to 23.2)	25.35<br>(15.63 to 37.28)	27<br>(16.6 to 39.5)	40.16<br>(24.75 to 58.77)
Inner Mongolia	Other sense organ diseases	5.15<br>(3.16 to 7.53)	22.63<br>(13.9 to 33.11)	10.3<br>(6.21 to 14.8)	39.7<br>(24 to 57.21)
Jiangsu	Other sense organ diseases	19.3<br>(12 to 28.2)	28.09<br>(17.4 to 40.94)	36<br>(22.2 to 52.3)	44.03<br>(27.16 to 64.06)
Jiangxi	Other sense organ diseases	9.14<br>(5.61 to 13.3)	23.11<br>(14.19 to 33.6)	16.9<br>(10.3 to 24.7)	35.59<br>(21.72 to 51.92)
Jilin	Other sense organ diseases	6.21<br>(3.85 to 9.09)	23.93<br>(14.83 to 35.03)	12.2<br>(7.49 to 17.7)	43.71<br>(26.87 to 63.38)
Liaoning	Other sense organ diseases	10.9<br>(6.68 to 15.7)	26.67<br>(16.39 to 38.43)	20.8<br>(12.8 to 30.5)	46.6<br>(28.76 to 68.2)
Ningxia	Other sense organ diseases	1.03<br>(0.632 to 1.49)	20.82<br>(12.79 to 30.2)	2.34<br>(1.44 to 3.44)	33.36<br>(20.47 to 48.97)
Qinghai	Other sense organ diseases	0.985<br>(0.609 to 1.46)	20.8<br>(12.86 to 30.81)	2.03<br>(1.25 to 2.99)	32.39<br>(19.91 to 47.59)
Shaanxi	Other sense organ diseases	8.53<br>(5.28 to 12.5)	24.79<br>(15.35 to 36.43)	15.7<br>(9.64 to 22.7)	39.63<br>(24.39 to 57.55)
Shandong	Other sense organ diseases	23<br>(14.1 to 34)	26.91<br>(16.46 to 39.77)	42.2<br>(26.2 to 60.7)	42.42<br>(26.39 to 61.07)
Shanghai	Other sense organ diseases	4.81<br>(2.96 to 7.05)	34.39<br>(21.22 to 50.44)	12.1<br>(7.46 to 17.4)	43.23<br>(26.7 to 62.1)
Shanxi	Other sense organ diseases	7.56<br>(4.62 to 11)	24.96<br>(15.25 to 36.44)	14.6<br>(9 to 20.9)	37.79<br>(23.31 to 54.17)
Sichuan	Other sense organ diseases	28.7<br>(17.5 to 42.1)	25.75<br>(15.72 to 37.77)	36<br>(22.1 to 52.6)	43.17<br>(26.41 to 62.97)
Tianjin	Other sense organ diseases	2.64<br>(1.62 to 3.92)	28.66<br>(17.64 to 42.61)	6.17<br>(3.74 to 8.98)	40.41<br>(24.51 to 58.85)
Tibet	Other sense organ diseases	0.519<br>(0.321 to 0.759)	22.29<br>(13.76 to 32.56)	0.917<br>(0.563 to 1.35)	26.54<br>(16.29 to 38.93)
Xinjiang	Other sense organ diseases	3.44<br>(2.13 to 5.09)	21.84<br>(13.49 to 32.26)	7.99<br>(4.87 to 11.7)	32.78<br>(20.01 to 48.16)
Yunnan	Other sense organ diseases	9.01<br>(5.51 to 13.1)	23.12<br>(14.13 to 33.69)	16.9<br>(10.3 to 24.6)	34.28<br>(21.02 to 49.97)
Zhejiang	Other sense organ diseases	12<br>(7.4 to 17.6)	27.31<br>(16.78 to 39.89)	24.8<br>(15.4 to 36.3)	40.6<br>(25.1 to 59.39)
China	Oral disorders	1970<br>(1160 to 3090)	164.3<br>(96.56 to 258.1)	3590<br>(2110 to 5710)	254.02<br>(149.22 to 404.21)
Taiwan	Oral disorders	34.4<br>(20.6 to 53.6)	168.44<br>(100.85 to 262.52)	53.6<br>(32.8 to 82.2)	227.14<br>(139.25 to 348.56)
Hong Kong Special Administrative Region of China	Oral disorders	15.5<br>(8.73 to 25.8)	269.27<br>(151.51 to 448.01)	29.5<br>(16.9 to 48.3)	392.72<br>(224.62 to 643.41)
Macau Special Administrative Region of China	Oral disorders	0.593<br>(0.354 to 0.916)	167.12<br>(99.8 to 258.02)	1.49<br>(0.877 to 2.35)	217.31<br>(128.28 to 343.13)
Anhui	Oral disorders	93.6<br>(54.5 to 147)	158.01<br>(92.05 to 248.54)	166<br>(98.3 to 264)	270.95<br>(160.71 to 431.79)
Beijing	Oral disorders	24.3<br>(14.7 to 37.3)	216.46<br>(131.2 to 332.56)	66.9<br>(40.6 to 103)	277.71<br>(168.63 to 428.15)
Chongqing	Oral disorders	26.4<br>(15.4 to 41.5)	167.11<br>(97.5 to 263.26)	73.8<br>(43.5 to 117)	276.3<br>(162.83 to 439.13)
Fujian	Oral disorders	47.1<br>(27.7 to 73.5)	148.98<br>(87.64 to 232.44)	87.8<br>(51.8 to 140)	222.88<br>(131.41 to 354.77)
Gansu	Oral disorders	36.2<br>(21 to 57.1)	152.93<br>(88.6 to 241.58)	68.5<br>(39.9 to 111)	257.86<br>(150.06 to 417.36)
Guangdong	Oral disorders	87.4<br>(52.8 to 136)	132.77<br>(80.28 to 207.06)	202<br>(123 to 319)	172.88<br>(105.07 to 272.63)
Guangxi	Oral disorders	63.1<br>(37.1 to 98.3)	144.99<br>(85.2 to 226.11)	108<br>(63.6 to 172)	223.47<br>(131.66 to 355.21)
Guizhou	Oral disorders	45.6<br>(26.6 to 70.8)	137.45<br>(80.28 to 213.42)	78.9<br>(46.6 to 125)	233.11<br>(137.62 to 368.86)
Hainan	Oral disorders	9.84<br>(5.81 to 15.3)	141.32<br>(83.34 to 219.79)	20.4<br>(12 to 32.3)	212.72<br>(125.66 to 336.71)
Hebei	Oral disorders	109<br>(63.9 to 170)	170.69<br>(100.37 to 267.89)	202<br>(119 to 324)	264.4<br>(155.09 to 423.67)
Heilongjiang	Oral disorders	56.3<br>(32.8 to 89.1)	154.51<br>(90.08 to 244.29)	119<br>(69.4 to 194)	302.44<br>(176.69 to 494.27)

Henan	Oral disorders	148<br>(86.8 to 234)	167.33<br>(98.33 to 264.99)	245<br>(144 to 393)	254.92<br>(149.78 to 407.97)
Hubei	Oral disorders	80.3<br>(48.9 to 127)	144.62<br>(88.02 to 228.38)	137<br>(82.1 to 216)	247.13<br>(148.4 to 390.04)
Hunan	Oral disorders	97.4<br>(57.2 to 153)	156.67<br>(92 to 245.65)	177<br>(105 to 286)	264.05<br>(155.82 to 425.44)
Inner Mongolia	Oral disorders	34.2<br>(19.8 to 54.1)	150.2<br>(87.22 to 237.99)	71.1<br>(41.2 to 115)	274.58<br>(159.23 to 444.64)
Jiangsu	Oral disorders	125<br>(73.4 to 195)	181.05<br>(106.67 to 283.32)	214<br>(127 to 337)	262.42<br>(155.62 to 413.09)
Jiangxi	Oral disorders	54.6<br>(32.3 to 85.3)	138.05<br>(81.73 to 215.72)	106<br>(61.9 to 168)	222.47<br>(130.2 to 354.1)
Jilin	Oral disorders	41.8<br>(24.3 to 66.2)	160.95<br>(93.69 to 254.98)	85<br>(50.1 to 138)	304.77<br>(179.68 to 493.1)
Liaoning	Oral disorders	74.4<br>(43.5 to 118)	182.41<br>(106.76 to 289.89)	141<br>(82.5 to 225)	315.73<br>(184.69 to 504.98)
Ningxia	Oral disorders	6.42<br>(3.74 to 10.1)	129.93<br>(75.64 to 205.29)	15.8<br>(9.2 to 25.6)	225.14<br>(131.01 to 364.77)
Qinghai	Oral disorders	6.01<br>(3.51 to 9.44)	126.87<br>(74.14 to 199.26)	13.7<br>(7.99 to 22.3)	218.51<br>(127.1 to 354.3)
Shaanxi	Oral disorders	53.4<br>(31.1 to 84.2)	155.12<br>(90.49 to 244.72)	104<br>(61.4 to 166)	263.48<br>(155.32 to 420.78)
Shandong	Oral disorders	151<br>(89 to 237)	175.96<br>(103.95 to 276.71)	276<br>(163 to 442)	277.95<br>(164.45 to 444.54)
Shanghai	Oral disorders	29<br>(17.4 to 45.3)	207.84<br>(124.46 to 324.01)	68.9<br>(40.9 to 108)	246.61<br>(146.39 to 388.19)
Shanxi	Oral disorders	50<br>(29.4 to 79.3)	165.13<br>(96.97 to 261.75)	98.9<br>(57.8 to 159)	256.03<br>(149.72 to 411.95)
Sichuan	Oral disorders	224<br>(125 to 367)	200.8<br>(111.64 to 328.5)	260<br>(149 to 429)	311.17<br>(178.73 to 513.64)
Tianjin	Oral disorders	18.8<br>(11 to 29.9)	203.99<br>(119.91 to 325.22)	40.1<br>(23.5 to 64.1)	262.8<br>(154.28 to 419.8)
Tibet	Oral disorders	3.13<br>(1.83 to 4.92)	134.51<br>(78.55 to 210.98)	5.99<br>(3.5 to 9.54)	173.23<br>(101.33 to 275.94)
Xinjiang	Oral disorders	18.1<br>(11 to 28.2)	114.54<br>(69.56 to 178.93)	43.8<br>(26.8 to 68.6)	179.76<br>(109.9 to 281.69)
Yunnan	Oral disorders	52.9<br>(31 to 82.8)	135.83<br>(79.65 to 212.41)	106<br>(62.1 to 168)	214.95<br>(126.31 to 341.28)
Zhejiang	Oral disorders	84.6<br>(49.6 to 134)	191.88<br>(112.51 to 302.99)	155<br>(91.1 to 246)	252.51<br>(148.89 to 402.12)
China	Sudden infant death syndrome	331<br>(172 to 602)	27.63<br>(14.39 to 50.31)	74.9<br>(48.6 to 103)	5.3<br>(3.44 to 7.26)
Taiwan	Sudden infant death syndrome	12.4<br>(10.1 to 17.1)	60.84<br>(49.68 to 83.61)	2.76<br>(1.66 to 3.77)	11.7<br>(7.05 to 15.98)
Hong Kong Special Administrative Region of China	Sudden infant death syndrome	0.295<br>(0.125 to 0.502)	5.12<br>(2.17 to 8.72)	0.107<br>(0.02 to 0.183)	1.42<br>(0.27 to 2.44)
Macau Special Administrative Region of China	Sudden infant death syndrome	0.0235<br>(0.00823 to 0.0452)	6.62<br>(2.32 to 12.73)	0.0107<br>(0.00118 to 0.0215)	1.57<br>(0.17 to 3.14)
Anhui	Sudden infant death syndrome	25.1<br>(9.28 to 55.1)	42.33<br>(15.66 to 93.04)	3.66<br>(1.91 to 5.92)	5.99<br>(3.12 to 9.68)
Beijing	Sudden infant death syndrome	0.939<br>(0.479 to 1.59)	8.37<br>(4.27 to 14.17)	0.983<br>(0.401 to 2.01)	4.08<br>(1.66 to 8.33)
Chongqing	Sudden infant death syndrome	3.79<br>(1.63 to 8.34)	24<br>(10.31 to 52.87)	0.8<br>(0.517 to 1.28)	2.99<br>(1.93 to 4.79)
Fujian	Sudden infant death syndrome	7.09<br>(3.25 to 12.8)	22.42<br>(10.29 to 40.39)	1.63<br>(0.873 to 2.75)	4.13<br>(2.22 to 6.99)
Gansu	Sudden infant death syndrome	13.4<br>(6.13 to 28.3)	56.85<br>(25.91 to 119.47)	2.49<br>(1.27 to 3.97)	9.37<br>(4.77 to 14.92)
Guangdong	Sudden infant death syndrome	11<br>(6.31 to 18.1)	16.71<br>(9.59 to 27.53)	7.67<br>(2.71 to 14.3)	6.57<br>(2.32 to 12.26)
Guangxi	Sudden infant death syndrome	9.24<br>(4.55 to 16.5)	21.24<br>(10.46 to 37.92)	3.13<br>(1.21 to 5.81)	6.48<br>(2.51 to 12.02)
Guizhou	Sudden infant death syndrome	15.4<br>(6.02 to 31.2)	46.46<br>(18.14 to 93.85)	2.02<br>(0.763 to 3.66)	5.97<br>(2.26 to 10.82)
Hainan	Sudden infant death syndrome	1.61<br>(0.651 to 3.77)	23.13<br>(9.35 to 54.19)	0.87<br>(0.495 to 1.51)	9.07<br>(5.16 to 15.75)

Hebei	Sudden infant death syndrome	14.2<br>(6.52 to 28.1)	22.33<br>(10.25 to 44.12)	5.62<br>(3.35 to 9.21)	7.35<br>(4.39 to 12.05)
Heilongjiang	Sudden infant death syndrome	7.06<br>(3.99 to 12.3)	19.38<br>(10.95 to 33.81)	1.55<br>(0.571 to 2.92)	3.94<br>(1.46 to 7.43)
Henan	Sudden infant death syndrome	27.3<br>(11.8 to 59.2)	30.91<br>(13.34 to 67.11)	5.3<br>(2.36 to 9.67)	5.51<br>(2.45 to 10.04)
Hubei	Sudden infant death syndrome	16.1<br>(7.65 to 31.2)	29.07<br>(13.77 to 56.24)	2.9<br>(1.02 to 5.4)	5.25<br>(1.84 to 9.77)
Hunan	Sudden infant death syndrome	15.3<br>(7.9 to 26.4)	24.68<br>(12.71 to 42.41)	2.99<br>(0.831 to 5.71)	4.46<br>(1.24 to 8.5)
Inner Mongolia	Sudden infant death syndrome	7.43<br>(3.13 to 18.9)	32.69<br>(13.75 to 83.25)	1.32<br>(0.716 to 2.28)	5.08<br>(2.77 to 8.79)
Jiangsu	Sudden infant death syndrome	12.7<br>(6.49 to 22)	18.4<br>(9.43 to 31.98)	2.74<br>(0.665 to 5.51)	3.36<br>(0.81 to 6.74)
Jiangxi	Sudden infant death syndrome	17<br>(5.19 to 42.3)	42.97<br>(13.13 to 106.89)	3.35<br>(1.95 to 5.33)	7.05<br>(4.1 to 11.22)
Jilin	Sudden infant death syndrome	5.04<br>(2.62 to 8.66)	19.44<br>(10.09 to 33.37)	1.04<br>(0.576 to 1.77)	3.73<br>(2.07 to 6.36)
Liaoning	Sudden infant death syndrome	5.46<br>(2.9 to 9.52)	13.4<br>(7.11 to 23.33)	1.14<br>(0.379 to 2.16)	2.56<br>(0.85 to 4.84)
Ningxia	Sudden infant death syndrome	2.75<br>(0.924 to 7.46)	55.69<br>(18.7 to 151.01)	0.523<br>(0.288 to 0.921)	7.46<br>(4.11 to 13.12)
Qinghai	Sudden infant death syndrome	2.72<br>(0.871 to 6.94)	57.35<br>(18.38 to 146.54)	0.515<br>(0.275 to 0.966)	8.19<br>(4.37 to 15.38)
Shaanxi	Sudden infant death syndrome	10.8<br>(4.12 to 28.5)	31.45<br>(11.96 to 82.84)	2.27<br>(1.26 to 3.73)	5.75<br>(3.18 to 9.44)
Shandong	Sudden infant death syndrome	16.2<br>(8.39 to 29)	18.94<br>(9.8 to 33.88)	4.38<br>(1.23 to 8.26)	4.41<br>(1.24 to 8.31)
Shanghai	Sudden infant death syndrome	1.07<br>(0.565 to 1.82)	7.64<br>(4.05 to 13.02)	0.971<br>(0.482 to 1.85)	3.47<br>(1.73 to 6.63)
Shanxi	Sudden infant death syndrome	8.56<br>(4.09 to 17.9)	28.26<br>(13.52 to 59.04)	2.37<br>(1.31 to 4.02)	6.13<br>(3.4 to 10.4)
Sichuan	Sudden infant death syndrome	37.8<br>(12.5 to 111)	33.9<br>(11.21 to 99.31)	3.17<br>(1.63 to 5.59)	3.8<br>(1.95 to 6.7)
Tianjin	Sudden infant death syndrome	0.973<br>(0.522 to 1.63)	10.57<br>(5.67 to 17.7)	0.728<br>(0.347 to 1.41)	4.77<br>(2.28 to 9.21)
Tibet	Sudden infant death syndrome	1.13<br>(0.247 to 3.16)	48.65<br>(10.6 to 135.64)	0.531<br>(0.171 to 1.27)	15.37<br>(4.96 to 36.6)
Xinjiang	Sudden infant death syndrome	5.94<br>(1.85 to 18)	37.65<br>(11.74 to 113.85)	2.13<br>(0.873 to 5.34)	8.75<br>(3.58 to 21.93)
Yunnan	Sudden infant death syndrome	17.6<br>(5.93 to 45.1)	45.16<br>(15.22 to 115.85)	3.81<br>(2.17 to 6.44)	7.75<br>(4.42 to 13.1)
Zhejiang	Sudden infant death syndrome	9.58<br>(3.61 to 19.4)	21.73<br>(8.19 to 44.05)	2.15<br>(1.17 to 3.63)	3.52<br>(1.91 to 5.93)
China	Injuries	57800<br>(55400 to 60400)	4831.85<br>(4625.27 to 5045.99)	37200<br>(34200 to 40800)	2635.09<br>(2418.44 to 2888.88)
Taiwan	Injuries	912<br>(883 to 947)	4467.94<br>(4327.87 to 4640.17)	582<br>(535 to 636)	2467.98<br>(2268.93 to 2697.21)
Hong Kong Special Administrative Region of China	Injuries	102<br>(92.1 to 112)	1767.49<br>(1597.13 to 1950.48)	130<br>(108 to 157)	1736.52<br>(1438.1 to 2085.64)
Macau Special Administrative Region of China	Injuries	7.3<br>(6.81 to 7.81)	2056.32<br>(1917.63 to 2199.86)	10.7<br>(9.15 to 12.6)	1569.36<br>(1338.24 to 1841.99)
Anhui	Injuries	3900<br>(3470 to 4320)	6574.71<br>(5861.02 to 7291.67)	1680<br>(1460 to 1910)	2743.07<br>(2379.96 to 3128.34)
Beijing	Injuries	233<br>(206 to 269)	2077.46<br>(1833.04 to 2397.75)	420<br>(342 to 513)	1744.22<br>(1418.83 to 2127.97)
Chongqing	Injuries	882<br>(765 to 1000)	5587.62<br>(4849.13 to 6357.99)	617<br>(525 to 727)	2307.49<br>(1964.37 to 2719.32)
Fujian	Injuries	1410<br>(1300 to 1530)	4469.44<br>(4102.55 to 4836.41)	994<br>(851 to 1160)	2520.95<br>(2158.44 to 2939.51)
Gansu	Injuries	1770<br>(1590 to 1960)	7486.7<br>(6726.51 to 8270.66)	777<br>(675 to 886)	2921.8<br>(2540.99 to 3334.34)
Guangdong	Injuries	2100<br>(1880 to 2380)	3198.05<br>(2852.6 to 3617.79)	2310<br>(1990 to 2680)	1974.19<br>(1700.48 to 2294.95)
Guangxi	Injuries	2090<br>(1880 to 2340)	4804.76<br>(4322.57 to 5381.39)	1290<br>(1110 to 1510)	2669.34<br>(2297.29 to 3117.9)

Guizhou	Injuries	2130<br>(1870 to 2420)	6406.51<br>(5633.64 to 7303.25)	1030<br>(876 to 1200)	3034.7<br>(2590.05 to 3553.05)
Hainan	Injuries	340<br>(298 to 387)	4880.42<br>(4282.94 to 5549.93)	229<br>(197 to 264)	2384.84<br>(2058.03 to 2758.04)
Hebei	Injuries	2430<br>(2210 to 2660)	3812.27<br>(3474.38 to 4177.46)	2180<br>(1910 to 2460)	2850.6<br>(2493.73 to 3223.08)
Heilongjiang	Injuries	1230<br>(1110 to 1360)	3371.4<br>(3048.98 to 3744.02)	757<br>(646 to 884)	1928.17<br>(1645.52 to 2252.92)
Henan	Injuries	3820<br>(3460 to 4150)	4326.86<br>(3921.68 to 4698.97)	2480<br>(2150 to 2860)	2578.63<br>(2228.46 to 2972.86)
Hubei	Injuries	2970<br>(2650 to 3230)	5339.39<br>(4773.96 to 5816.96)	1760<br>(1470 to 2040)	3181.62<br>(2659.14 to 3680.17)
Hunan	Injuries	3330<br>(3080 to 3600)	5357.11<br>(4956.34 to 5785.56)	2000<br>(1710 to 2310)	2977.96<br>(2552.05 to 3432.82)
Inner Mongolia	Injuries	1100<br>(980 to 1230)	4827.15<br>(4307.31 to 5409.14)	618<br>(532 to 709)	2386.06<br>(2053.45 to 2739)
Jiangsu	Injuries	2660<br>(2440 to 2910)	3869.67<br>(3545.86 to 4226.85)	2180<br>(1890 to 2550)	2667.27<br>(2310.99 to 3125.31)
Jiangxi	Injuries	3200<br>(2840 to 3580)	8084.36<br>(7175.06 to 9043.33)	1480<br>(1300 to 1690)	3119.94<br>(2735.97 to 3564.91)
Jilin	Injuries	848<br>(757 to 973)	3267.56<br>(2917.39 to 3751.05)	493<br>(410 to 583)	1767.69<br>(1470.05 to 2088.68)
Liaoning	Injuries	1280<br>(1140 to 1440)	3143.58<br>(2784.16 to 3541.13)	947<br>(807 to 1110)	2122.03<br>(1806.96 to 2476.14)
Ningxia	Injuries	363<br>(309 to 426)	7357.14<br>(6265.15 to 8631.73)	207<br>(178 to 241)	2949.29<br>(2537.86 to 3430.98)
Qinghai	Injuries	342<br>(293 to 396)	7229.13<br>(6191.4 to 8355.41)	215<br>(185 to 255)	3429.24<br>(2951.73 to 4059.64)
Shaanxi	Injuries	1250<br>(1130 to 1380)	3636.2<br>(3275.8 to 4013.52)	1070<br>(901 to 1250)	2710.92<br>(2279.49 to 3154.89)
Shandong	Injuries	3810<br>(3410 to 4230)	4455.27<br>(3987.36 to 4939.77)	2430<br>(2090 to 2810)	2441.25<br>(2103.18 to 2829.86)
Shanghai	Injuries	351<br>(315 to 392)	2515.17<br>(2256.34 to 2804.18)	663<br>(541 to 801)	2372.14<br>(1937.22 to 2867.3)
Shanxi	Injuries	1220<br>(1080 to 1410)	4021.87<br>(3566.12 to 4649.57)	975<br>(828 to 1130)	2525.5<br>(2142.44 to 2922.65)
Sichuan	Injuries	6080<br>(5380 to 6840)	5451.52<br>(4822.06 to 6127.18)	2600<br>(2210 to 3040)	3115.37<br>(2649.4 to 3635.31)
Tianjin	Injuries	258<br>(232 to 290)	2804.27<br>(2520.02 to 3148.54)	301<br>(258 to 347)	1972.45<br>(1692.94 to 2275.07)
Tibet	Injuries	163<br>(142 to 189)	7011.56<br>(6092.49 to 8109.87)	111<br>(95.7 to 129)	3209.83<br>(2767.87 to 3718.13)
Xinjiang	Injuries	852<br>(713 to 977)	5403.04<br>(4521.74 to 6196.76)	724<br>(633 to 826)	2969.19<br>(2598.3 to 3389.46)
Yunnan	Injuries	3180<br>(2760 to 3600)	8167.32<br>(7088.85 to 9232.99)	1870<br>(1630 to 2140)	3806.12<br>(3314.6 to 4360.88)
Zhejiang	Injuries	2130<br>(1950 to 2340)	4832.66<br>(4430.34 to 5298.46)	1680<br>(1420 to 1980)	2745.07<br>(2324.78 to 3235.48)
China	Transport injuries	15500<br>(14500 to 16900)	1293.87<br>(1210.84 to 1415.72)	14600<br>(13400 to 15900)	1031.7<br>(948.2 to 1126.18)
Taiwan	Transport injuries	420<br>(408 to 436)	2059.43<br>(2000.14 to 2134.61)	206<br>(186 to 230)	874.27<br>(788.04 to 975.07)
Hong Kong Special Administrative Region of China	Transport injuries	28.6<br>(25.7 to 32)	495.32<br>(445.18 to 555.28)	33.1<br>(26.7 to 41)	440.5<br>(355.26 to 546.49)
Macau Special Administrative Region of China	Transport injuries	2.07<br>(1.87 to 2.29)	583.13<br>(526.34 to 644.82)	3.37<br>(2.76 to 4.1)	492.95<br>(403.44 to 599.09)
Anhui	Transport injuries	874<br>(763 to 1010)	1474.58<br>(1287.43 to 1707.45)	669<br>(579 to 774)	1093.74<br>(947.27 to 1266.26)
Beijing	Transport injuries	76.6<br>(66.7 to 89.9)	682.9<br>(594.31 to 801.19)	123<br>(101 to 148)	509.6<br>(418.29 to 614.42)
Chongqing	Transport injuries	217<br>(183 to 259)	1375.38<br>(1158.31 to 1640.74)	211<br>(177 to 250)	790.16<br>(663.51 to 935.7)
Fujian	Transport injuries	313<br>(279 to 355)	989.71<br>(880.63 to 1122.5)	334<br>(281 to 394)	847.16<br>(713.65 to 1000.17)
Gansu	Transport injuries	608<br>(532 to 696)	2571.38<br>(2251.34 to 2942.94)	358<br>(305 to 417)	1345.18<br>(1147.89 to 1570.42)

Guangdong	Transport injuries	552<br>(481 to 653)	838.42<br>(731.55 to 991.71)	801<br>(679 to 948)	685.82<br>(580.89 to 811.56)
Guangxi	Transport injuries	525<br>(453 to 620)	1207.32<br>(1040.99 to 1425.08)	501<br>(425 to 600)	1036.59<br>(878.91 to 1242.35)
Guizhou	Transport injuries	491<br>(416 to 597)	1479.33<br>(1253.97 to 1798.73)	365<br>(304 to 441)	1077.81<br>(897.3 to 1304.47)
Hainan	Transport injuries	84.7<br>(70.4 to 102)	1216.53<br>(1011.01 to 1467.8)	72.2<br>(60.4 to 85.5)	753.31<br>(629.61 to 892.07)
Hebei	Transport injuries	966<br>(862 to 1090)	1517.05<br>(1355.13 to 1712.15)	1040<br>(889 to 1210)	1366.35<br>(1163.53 to 1581.93)
Heilongjiang	Transport injuries	472<br>(414 to 556)	1296.06<br>(1136.19 to 1523.94)	334<br>(284 to 395)	851.72<br>(724.02 to 1006.76)
Henan	Transport injuries	1040<br>(920 to 1160)	1175.9<br>(1041.92 to 1319.74)	1050<br>(896 to 1230)	1090.91<br>(930.29 to 1273.12)
Hubei	Transport injuries	600<br>(533 to 686)	1080.47<br>(959.64 to 1234.92)	625<br>(529 to 729)	1130.19<br>(956.76 to 1318.96)
Hunan	Transport injuries	767<br>(686 to 879)	1233.37<br>(1102.93 to 1413.55)	767<br>(647 to 901)	1142.63<br>(963.46 to 1342.1)
Inner Mongolia	Transport injuries	386<br>(337 to 446)	1696.49<br>(1480.57 to 1961.6)	305<br>(258 to 356)	1177.22<br>(995.18 to 1374.04)
Jiangsu	Transport injuries	732<br>(654 to 824)	1063.94<br>(951.04 to 1197.83)	872<br>(753 to 1010)	1067.75<br>(922.49 to 1242.02)
Jiangxi	Transport injuries	770<br>(675 to 879)	1947.1<br>(1708.12 to 2223.46)	568<br>(491 to 656)	1195.11<br>(1033.07 to 1379.3)
Jilin	Transport injuries	351<br>(302 to 411)	1353.41<br>(1163.34 to 1584.11)	222<br>(182 to 263)	795.04<br>(653.2 to 944.41)
Liaoning	Transport injuries	510<br>(432 to 604)	1249.33<br>(1058.33 to 1479.7)	393<br>(331 to 463)	880.98<br>(741.23 to 1037.56)
Ningxia	Transport injuries	116<br>(97.9 to 138)	2339.24<br>(1982.75 to 2789.77)	101<br>(86 to 120)	1443.56<br>(1225.78 to 1713.11)
Qinghai	Transport injuries	98<br>(83.8 to 116)	2068.72<br>(1769.66 to 2453.5)	97.6<br>(82.9 to 118)	1553.85<br>(1319.99 to 1875.05)
Shaanxi	Transport injuries	351<br>(308 to 399)	1020.75<br>(896.35 to 1158.64)	453<br>(385 to 530)	1147.04<br>(974.92 to 1341.97)
Shandong	Transport injuries	1120<br>(984 to 1290)	1313.92<br>(1150.03 to 1504.63)	1050<br>(884 to 1230)	1056.3<br>(889.94 to 1239.73)
Shanghai	Transport injuries	112<br>(99.2 to 127)	800.71<br>(710.27 to 912.45)	225<br>(185 to 272)	806.35<br>(661.01 to 973.59)
Shanxi	Transport injuries	459<br>(398 to 545)	1515.51<br>(1314.13 to 1800.51)	446<br>(377 to 520)	1154.66<br>(977.09 to 1346.61)
Sichuan	Transport injuries	1310<br>(1140 to 1520)	1170.43<br>(1022.08 to 1361.91)	872<br>(734 to 1020)	1044.87<br>(879.04 to 1226.35)
Tianjin	Transport injuries	91.2<br>(79 to 105)	991.79<br>(859.07 to 1145.99)	133<br>(113 to 153)	869.15<br>(743.59 to 1005.03)
Tibet	Transport injuries	27.1<br>(22.4 to 33.3)	1161.69<br>(962.84 to 1430.67)	31.3<br>(26.5 to 37.2)	906.01<br>(766.89 to 1076.66)
Xinjiang	Transport injuries	252<br>(207 to 290)	1595.4<br>(1313.48 to 1836.09)	310<br>(264 to 364)	1271.87<br>(1085.23 to 1495.79)
Yunnan	Transport injuries	598<br>(500 to 740)	1534.55<br>(1283.71 to 1897.71)	616<br>(524 to 727)	1252.02<br>(1066.72 to 1479.36)
Zhejiang	Transport injuries	589<br>(525 to 657)	1335.99<br>(1190.29 to 1490.76)	586<br>(491 to 691)	958.49<br>(803.03 to 1128.57)
China	Road injuries	14000<br>(13100 to 15400)	1170.1<br>(1096.63 to 1286.39)	13500<br>(12500 to 14600)	953.47<br>(881.87 to 1031.44)
Taiwan	Road injuries	396<br>(386 to 409)	1942.65<br>(1892.42 to 2005.41)	188<br>(170 to 208)	795.08<br>(722.33 to 880.37)
Hong Kong Special Administrative Region of China	Road injuries	24.3<br>(22 to 27.1)	422.33<br>(382.28 to 469.61)	27<br>(21.9 to 33.3)	358.92<br>(292.13 to 443.88)
Macau Special Administrative Region of China	Road injuries	1.82<br>(1.66 to 2)	513.59<br>(466.9 to 564.3)	2.89<br>(2.38 to 3.5)	421.91<br>(347.66 to 511.4)
Anhui	Road injuries	783<br>(673 to 918)	1321.96<br>(1136.3 to 1548.48)	618<br>(536 to 717)	1011.13<br>(876.31 to 1172.9)
Beijing	Road injuries	67.1<br>(58.4 to 79.8)	597.91<br>(520.28 to 711.27)	106<br>(88 to 127)	440.49<br>(364.94 to 527.12)
Chongqing	Road injuries	195<br>(164 to 234)	1238.52<br>(1040.35 to 1485.01)	192<br>(161 to 227)	716.84<br>(601.44 to 848.96)

Fujian	Road injuries	276<br>(245 to 315)	873.35<br>(774.08 to 996.67)	305<br>(258 to 359)	772.84<br>(654.31 to 910.28)
Gansu	Road injuries	556<br>(485 to 639)	2351.37<br>(2050.48 to 2703.52)	333<br>(284 to 389)	1254.09<br>(1069.35 to 1463.87)
Guangdong	Road injuries	498<br>(433 to 592)	756.78<br>(657.12 to 898.74)	732<br>(621 to 862)	626.3<br>(531.41 to 737.41)
Guangxi	Road injuries	476<br>(408 to 562)	1095.46<br>(937.62 to 1292.63)	467<br>(393 to 561)	965.49<br>(814.22 to 1160.51)
Guizhou	Road injuries	430<br>(362 to 523)	1294.47<br>(1089.18 to 1574.97)	333<br>(277 to 406)	984.46<br>(817.65 to 1199.22)
Hainan	Road injuries	78.1<br>(64.4 to 94.8)	1120.62<br>(924.52 to 1361.1)	65.6<br>(55.1 to 77.8)	683.82<br>(574.42 to 811.3)
Hebei	Road injuries	871<br>(771 to 988)	1368.84<br>(1212.11 to 1551.65)	973<br>(829 to 1130)	1273.74<br>(1084.81 to 1475.89)
Heilongjiang	Road injuries	431<br>(377 to 509)	1182.05<br>(1033.26 to 1395.17)	303<br>(255 to 359)	771.74<br>(649.31 to 914.53)
Henan	Road injuries	943<br>(837 to 1060)	1068<br>(947.78 to 1206.09)	982<br>(839 to 1140)	1019.89<br>(871.51 to 1186.67)
Hubei	Road injuries	545<br>(482 to 624)	980.49<br>(868.12 to 1122.42)	584<br>(493 to 683)	1056.61<br>(891.4 to 1234.72)
Hunan	Road injuries	693<br>(618 to 802)	1114.57<br>(994.09 to 1289.18)	714<br>(602 to 841)	1063.93<br>(896.53 to 1252.46)
Inner Mongolia	Road injuries	353<br>(306 to 410)	1553.83<br>(1346.12 to 1804.79)	283<br>(239 to 331)	1092.52<br>(921.92 to 1279.1)
Jiangsu	Road injuries	660<br>(591 to 743)	959.25<br>(858.29 to 1080.35)	811<br>(701 to 942)	993.04<br>(858.72 to 1154.01)
Jiangxi	Road injuries	703<br>(615 to 807)	1779.03<br>(1554.89 to 2040.71)	531<br>(459 to 615)	1117.73<br>(966.47 to 1294.42)
Jilin	Road injuries	314<br>(269 to 372)	1210.82<br>(1037.36 to 1433.11)	197<br>(162 to 234)	705.47<br>(579.49 to 838.21)
Liaoning	Road injuries	469<br>(395 to 559)	1149.01<br>(969.21 to 1369.91)	357<br>(300 to 420)	799.96<br>(671.44 to 941.75)
Ningxia	Road injuries	105<br>(88.6 to 126)	2136.09<br>(1793.69 to 2560.53)	94.2<br>(80.1 to 112)	1342.36<br>(1140.86 to 1600.34)
Qinghai	Road injuries	88.5<br>(75.2 to 106)	1868.62<br>(1588.21 to 2237.97)	91<br>(77 to 111)	1448.2<br>(1225.44 to 1759.22)
Shaanxi	Road injuries	315<br>(276 to 360)	915.33<br>(802.73 to 1047.25)	420<br>(356 to 493)	1062.77<br>(900.13 to 1247.95)
Shandong	Road injuries	1030<br>(893 to 1180)	1199.09<br>(1043.43 to 1379.21)	971<br>(818 to 1140)	976.96<br>(823.43 to 1151.3)
Shanghai	Road injuries	99.7<br>(88.3 to 113)	713.76<br>(631.84 to 811.2)	205<br>(168 to 247)	734.78<br>(602.21 to 884.51)
Shanxi	Road injuries	417<br>(360 to 496)	1378.46<br>(1188.19 to 1639.27)	414<br>(350 to 484)	1071.69<br>(905.44 to 1252.15)
Sichuan	Road injuries	1170<br>(1010 to 1360)	1043.95<br>(904.23 to 1221.04)	805<br>(673 to 950)	963.67<br>(806.64 to 1137.98)
Tianjin	Road injuries	81.7<br>(70.2 to 94.6)	887.96<br>(763.65 to 1028.78)	120<br>(103 to 140)	789.33<br>(673.75 to 917.41)
Tibet	Road injuries	23.9<br>(19.8 to 29.8)	1027.54<br>(851.65 to 1280.36)	28.3<br>(23.8 to 33.8)	818.5<br>(688.22 to 976.91)
Xinjiang	Road injuries	229<br>(187 to 267)	1454.95<br>(1183.98 to 1693.71)	288<br>(244 to 339)	1181.26<br>(1000.95 to 1391.88)
Yunnan	Road injuries	533<br>(443 to 667)	1368.12<br>(1137.01 to 1710.83)	572<br>(489 to 678)	1164.1<br>(995.14 to 1379.43)
Zhejiang	Road injuries	551<br>(492 to 614)	1249.24<br>(1116.58 to 1393.45)	542<br>(455 to 637)	885.43<br>(744.37 to 1040.58)
China	Other transport injuries	1480<br>(1230 to 1760)	123.77<br>(102.59 to 147.17)	1100<br>(891 to 1360)	78.23<br>(63.09 to 96.24)
Taiwan	Other transport injuries	23.8<br>(21.2 to 27)	116.77<br>(103.68 to 132.45)	18.7<br>(15 to 23)	79.2<br>(63.81 to 97.73)
Hong Kong Special Administrative Region of China	Other transport injuries	4.21<br>(3.36 to 5.25)	72.99<br>(58.31 to 91.04)	6.13<br>(4.65 to 7.92)	81.58<br>(61.87 to 105.45)
Macau Special Administrative Region of China	Other transport injuries	0.247<br>(0.195 to 0.311)	69.53<br>(55.04 to 87.57)	0.486<br>(0.36 to 0.638)	71.04<br>(52.65 to 93.29)
Anhui	Other transport injuries	90.4<br>(74.4 to 124)	152.62<br>(125.55 to 209.62)	50.5<br>(41.1 to 61.4)	82.6<br>(67.26 to 100.4)

Beijing	Other transport injuries	9.53<br>(7.56 to 12)	84.98<br>(67.43 to 106.6)	16.7<br>(12.5 to 21.6)	69.12<br>(52.04 to 89.77)
Chongqing	Other transport injuries	21.6<br>(16.8 to 26.2)	136.86<br>(106.17 to 165.84)	19.6<br>(15.2 to 24.7)	73.32<br>(56.82 to 92.33)
Fujian	Other transport injuries	36.8<br>(30.5 to 44.4)	116.36<br>(96.51 to 140.45)	29.3<br>(23 to 36.4)	74.31<br>(58.46 to 92.48)
Gansu	Other transport injuries	52<br>(40.6 to 62.7)	220.02<br>(171.49 to 264.98)	24.2<br>(19.7 to 29.3)	91.09<br>(74.28 to 110.36)
Guangdong	Other transport injuries	53.7<br>(42.7 to 66.7)	81.64<br>(64.83 to 101.38)	69.5<br>(53.3 to 90.2)	59.52<br>(45.64 to 77.2)
Guangxi	Other transport injuries	48.6<br>(38.2 to 59.6)	111.86<br>(87.82 to 136.98)	34.4<br>(27.2 to 43.1)	71.1<br>(56.2 to 89.18)
Guizhou	Other transport injuries	61.4<br>(48.5 to 75.7)	184.85<br>(146.04 to 228.19)	31.6<br>(25.3 to 38.5)	93.35<br>(74.85 to 113.9)
Hainan	Other transport injuries	6.68<br>(5.07 to 8.62)	95.92<br>(72.74 to 123.77)	6.66<br>(5.25 to 8.32)	69.49<br>(54.72 to 86.81)
Hebei	Other transport injuries	94.3<br>(76.8 to 113)	148.21<br>(120.73 to 176.84)	70.8<br>(58.7 to 84.5)	92.61<br>(76.75 to 110.62)
Heilongjiang	Other transport injuries	41.6<br>(32.4 to 51.8)	114.01<br>(88.93 to 142.21)	31.4<br>(25.3 to 39)	79.98<br>(64.47 to 99.28)
Henan	Other transport injuries	95.2<br>(76.8 to 115)	107.9<br>(86.98 to 130.41)	68.4<br>(54.2 to 85.2)	71.02<br>(56.25 to 88.49)
Hubei	Other transport injuries	55.5<br>(44.1 to 67.3)	99.98<br>(79.35 to 121.09)	40.7<br>(32.1 to 51.3)	73.57<br>(57.96 to 92.79)
Hunan	Other transport injuries	73.9<br>(61.2 to 89)	118.81<br>(98.38 to 143.13)	52.8<br>(41.6 to 65.6)	78.7<br>(62.01 to 97.67)
Inner Mongolia	Other transport injuries	32.4<br>(25.8 to 39.6)	142.65<br>(113.3 to 174.08)	21.9<br>(17.7 to 26.5)	84.71<br>(68.35 to 102.35)
Jiangsu	Other transport injuries	72<br>(57.5 to 86.7)	104.69<br>(83.52 to 126.01)	61<br>(47.7 to 76.6)	74.71<br>(58.44 to 93.79)
Jiangxi	Other transport injuries	66.4<br>(52.9 to 79.9)	168.07<br>(133.7 to 202.1)	36.8<br>(29.5 to 46)	77.38<br>(62.06 to 96.87)
Jilin	Other transport injuries	37<br>(28.6 to 45.7)	142.59<br>(110.35 to 176.06)	25<br>(19.8 to 31.1)	89.57<br>(70.88 to 111.37)
Liaoning	Other transport injuries	40.9<br>(31.5 to 51.9)	100.32<br>(77.33 to 127.32)	36.2<br>(28.3 to 46.3)	81.02<br>(63.3 to 103.63)
Ningxia	Other transport injuries	10<br>(7.98 to 12.8)	203.16<br>(161.5 to 259.05)	7.1<br>(5.79 to 8.63)	101.2<br>(82.44 to 122.92)
Qinghai	Other transport injuries	9.48<br>(7.57 to 11.5)	200.09<br>(159.73 to 242.62)	6.64<br>(5.43 to 8.04)	105.65<br>(86.41 to 127.95)
Shaanxi	Other transport injuries	36.3<br>(29.2 to 44.5)	105.43<br>(84.82 to 129.18)	33.3<br>(26.7 to 41.2)	84.27<br>(67.52 to 104.27)
Shandong	Other transport injuries	98.3<br>(79.9 to 119)	114.82<br>(93.38 to 138.98)	78.9<br>(62.1 to 99.8)	79.34<br>(62.44 to 100.46)
Shanghai	Other transport injuries	12.1<br>(9.68 to 15.1)	86.95<br>(69.31 to 108.07)	20<br>(15.3 to 25.6)	71.57<br>(54.93 to 91.74)
Shanxi	Other transport injuries	41.5<br>(32.4 to 50.2)	137.05<br>(107.14 to 165.84)	32<br>(25.6 to 39.7)	82.97<br>(66.33 to 102.77)
Sichuan	Other transport injuries	141<br>(114 to 172)	126.48<br>(102.12 to 153.83)	67.8<br>(53.3 to 84.8)	81.2<br>(63.81 to 101.63)
Tianjin	Other transport injuries	9.55<br>(7.66 to 11.7)	103.84<br>(83.32 to 127.62)	12.2<br>(9.49 to 15.4)	79.82<br>(62.18 to 100.84)
Tibet	Other transport injuries	3.13<br>(2.4 to 3.92)	134.14<br>(103.16 to 168.3)	3.02<br>(2.41 to 3.75)	87.51<br>(69.7 to 108.63)
Xinjiang	Other transport injuries	22.2<br>(18 to 27.1)	140.44<br>(113.85 to 171.6)	22.1<br>(18.1 to 27.1)	90.61<br>(74.28 to 111.24)
Yunnan	Other transport injuries	64.9<br>(50.4 to 80.2)	166.42<br>(129.43 to 205.75)	43.2<br>(34.8 to 52.9)	87.92<br>(70.81 to 107.57)
Zhejiang	Other transport injuries	38.3<br>(30.3 to 47.2)	86.75<br>(68.61 to 106.99)	44.7<br>(33.9 to 57.3)	73.05<br>(55.38 to 93.67)
China	Unintentional injuries	27400<br>(25700 to 29300)	2289.87<br>(2149.41 to 2449.2)	16100<br>(13800 to 18300)	1139.58<br>(980.26 to 1295.5)
Taiwan	Unintentional injuries	362<br>(346 to 381)	1773.06<br>(1698.11 to 1868.33)	176<br>(155 to 200)	745.81<br>(656.56 to 848.77)
Hong Kong Special Administrative Region of China	Unintentional injuries	35.6<br>(30.3 to 42.6)	617.26<br>(525.42 to 739.07)	62.7<br>(49.1 to 79)	834.26<br>(653.97 to 1051.79)

Macau Special Administrative Region of China	Unintentional injuries	2.59 (2.33 to 2.95)	729.5 (655.67 to 831.11)	4.32 (3.58 to 5.29)	632.34 (523.28 to 773.44)
Anhui	Unintentional injuries	1930 (1670 to 2210)	3253.02 (2818.28 to 3735.74)	672 (567 to 769)	1099.38 (928 to 1258.18)
Beijing	Unintentional injuries	92.1 (77.8 to 111)	821.28 (693.4 to 993.59)	216 (166 to 277)	897.07 (688.65 to 1147.37)
Chongqing	Unintentional injuries	443 (371 to 523)	2807.94 (2349.57 to 3313.82)	303 (242 to 362)	1132.72 (907.03 to 1356.2)
Fujian	Unintentional injuries	665 (592 to 741)	2102.92 (1871.74 to 2343.25)	491 (399 to 589)	1245.6 (1013.27 to 1495.05)
Gansu	Unintentional injuries	711 (610 to 861)	3005.11 (2579.65 to 3638.85)	280 (240 to 320)	1055.25 (903.89 to 1205.58)
Guangdong	Unintentional injuries	1050 (913 to 1210)	1589.25 (1386.82 to 1834.23)	1110 (941 to 1310)	947.27 (805.46 to 1117.93)
Guangxi	Unintentional injuries	1100 (968 to 1250)	2536.85 (2226.23 to 2880.19)	598 (510 to 695)	1236.52 (1054.64 to 1438.68)
Guizhou	Unintentional injuries	1090 (930 to 1280)	3298.57 (2803.31 to 3861.04)	468 (373 to 559)	1382.68 (1102.31 to 1653.46)
Hainan	Unintentional injuries	180 (154 to 208)	2581.07 (2214.78 to 2982.06)	111 (95.6 to 129)	1156.14 (996.88 to 1341.32)
Hebei	Unintentional injuries	978 (868 to 1100)	1536.1 (1363.28 to 1735.72)	774 (667 to 888)	1012.61 (872.45 to 1161.47)
Heilongjiang	Unintentional injuries	430 (369 to 539)	1179.59 (1011.3 to 1479.87)	253 (212 to 301)	644.87 (540.43 to 765.95)
Henan	Unintentional injuries	1810 (1570 to 2030)	2045.49 (1778.8 to 2301.61)	968 (778 to 1140)	1005.26 (808.33 to 1182.56)
Hubei	Unintentional injuries	1380 (1220 to 1540)	2475.39 (2195.78 to 2771.29)	704 (551 to 836)	1273.71 (996.29 to 1511.39)
Hunan	Unintentional injuries	1560 (1390 to 1720)	2503.55 (2239.14 to 2763.42)	878 (725 to 1020)	1307.52 (1079.5 to 1525.7)
Inner Mongolia	Unintentional injuries	397 (341 to 496)	1745.55 (1500.44 to 2181.13)	197 (165 to 232)	760.92 (639 to 894.27)
Jiangsu	Unintentional injuries	1350 (1200 to 1520)	1965.2 (1742.03 to 2210.68)	985 (815 to 1180)	1205.98 (997.76 to 1440.95)
Jiangxi	Unintentional injuries	1770 (1500 to 2090)	4488.13 (3801.94 to 5279.26)	727 (609 to 845)	1529.3 (1281.32 to 1777.01)
Jilin	Unintentional injuries	272 (231 to 361)	1047.67 (890.01 to 1390.89)	166 (137 to 199)	594.92 (490.01 to 714.93)
Liaoning	Unintentional injuries	438 (379 to 522)	1074.29 (928.73 to 1279.47)	355 (295 to 422)	794.38 (661.77 to 944.75)
Ningxia	Unintentional injuries	189 (154 to 232)	3834.58 (3121.53 to 4702.1)	77.6 (64.3 to 90.9)	1105.89 (915.84 to 1294.48)
Qinghai	Unintentional injuries	169 (137 to 209)	3568.6 (2901.52 to 4416.24)	83.2 (70.6 to 97.1)	1323.57 (1124.37 to 1544.91)
Shaanxi	Unintentional injuries	649 (559 to 740)	1885.15 (1624.9 to 2149.29)	441 (336 to 527)	1114.96 (850.67 to 1333.49)
Shandong	Unintentional injuries	1180 (1040 to 1370)	1381.02 (1212.97 to 1604.92)	885 (743 to 1050)	890.34 (747.81 to 1056.04)
Shanghai	Unintentional injuries	162 (142 to 185)	1160.45 (1018.72 to 1321.54)	338 (268 to 422)	1210.07 (959.28 to 1508.67)
Shanxi	Unintentional injuries	491 (431 to 563)	1620.51 (1424.39 to 1858.94)	390 (313 to 462)	1010.48 (810.25 to 1194.94)
Sichuan	Unintentional injuries	3520 (2970 to 4120)	3152.64 (2661.36 to 3693.39)	1320 (1050 to 1570)	1575.15 (1256.17 to 1877.66)
Tianjin	Unintentional injuries	97.1 (85.3 to 114)	1055.92 (927.13 to 1242.54)	114 (97.2 to 132)	747.75 (636.85 to 867.55)
Tibet	Unintentional injuries	103 (84.6 to 123)	4440.46 (3632.34 to 5266.3)	63.8 (53.4 to 75.6)	1847.2 (1545.59 to 2187.2)
Xinjiang	Unintentional injuries	467 (381 to 552)	2958.5 (2412.53 to 3501.69)	304 (263 to 355)	1247.39 (1079.94 to 1455.19)
Yunnan	Unintentional injuries	1660 (1380 to 1970)	4267.73 (3549.26 to 5046.92)	895 (725 to 1050)	1820.34 (1475.39 to 2128.01)
Zhejiang	Unintentional injuries	1040 (903 to 1170)	2354.55 (2048.29 to 2664.17)	871 (707 to 1050)	1422.75 (1155.69 to 1715.78)
China	Falls	3910 (3410 to 4770)	326.49 (285.11 to 398.53)	5930 (4620 to 7120)	420.13 (327.36 to 504.18)
Taiwan	Falls	66.8 (61.8 to 73.3)	327.39 (303.09 to 359.04)	81.6 (71 to 94.4)	345.85 (301.12 to 400.09)

Hong Kong Special Administrative Region of China	Falls	14.6 (11.6 to 18.3)	253.97 (201.79 to 316.98)	37.1 (27.8 to 48)	493.5 (370.24 to 639.44)
Macau Special Administrative Region of China	Falls	0.652 (0.533 to 0.846)	183.8 (150.15 to 238.34)	1.71 (1.3 to 2.22)	250.58 (189.83 to 324.54)
Anhui	Falls	173 (143 to 222)	292.62 (241.97 to 374.43)	206 (155 to 253)	337.54 (253.51 to 413.04)
Beijing	Falls	28.8 (23 to 37)	257.09 (205.12 to 329.9)	103 (77.6 to 133)	426.51 (322.04 to 550.76)
Chongqing	Falls	67.7 (49.6 to 82.8)	429.15 (314.61 to 524.94)	116 (79 to 147)	433.62 (295.57 to 549.79)
Fujian	Falls	153 (122 to 178)	484.87 (385.83 to 562.1)	244 (173 to 304)	619.77 (438.11 to 772.16)
Gansu	Falls	84.6 (63.2 to 133)	357.74 (267.4 to 563.25)	78.7 (64.7 to 93.9)	295.97 (243.57 to 353.14)
Guangdong	Falls	197 (164 to 237)	299.37 (249.8 to 360.08)	479 (380 to 594)	410.11 (325.1 to 507.99)
Guangxi	Falls	151 (119 to 191)	347.82 (273.12 to 439.4)	194 (143 to 239)	402.41 (295.06 to 493.78)
Guizhou	Falls	179 (127 to 219)	540.13 (383.86 to 658.44)	191 (128 to 240)	565.59 (379.5 to 709.28)
Hainan	Falls	27.9 (21.9 to 34)	399.91 (314.81 to 488.4)	37.6 (30.6 to 45.3)	392.42 (319.3 to 472.9)
Hebei	Falls	120 (89.7 to 198)	187.87 (140.94 to 310.36)	207 (162 to 263)	270.61 (211.95 to 343.59)
Heilongjiang	Falls	59.5 (41.5 to 106)	163.3 (113.71 to 291.57)	79.9 (61.3 to 109)	203.62 (156.11 to 277.83)
Henan	Falls	205 (172 to 274)	231.88 (195.3 to 310.39)	277 (205 to 339)	287.84 (212.96 to 352.3)
Hubei	Falls	189 (155 to 217)	339.65 (279.78 to 390.66)	267 (170 to 339)	482.72 (307.87 to 613.75)
Hunan	Falls	178 (152 to 220)	285.61 (244.74 to 353.78)	258 (189 to 316)	384.79 (280.72 to 470.14)
Inner Mongolia	Falls	45.9 (32.8 to 84.7)	201.95 (144.08 to 372.58)	63.4 (49 to 81.8)	244.71 (189.34 to 315.92)
Jiangsu	Falls	204 (177 to 237)	296.67 (256.57 to 344.17)	429 (326 to 534)	524.81 (398.88 to 653.45)
Jiangxi	Falls	199 (151 to 241)	504.14 (381.83 to 608.66)	248 (179 to 308)	522.55 (376.62 to 648.78)
Jilin	Falls	44.1 (30.2 to 80)	169.82 (116.21 to 308.16)	53.3 (39.8 to 73.8)	191.2 (142.62 to 264.61)
Liaoning	Falls	101 (75.8 to 141)	246.45 (185.9 to 344.47)	137 (110 to 169)	306.02 (247.33 to 379.24)
Ningxia	Falls	16.8 (13.2 to 23.1)	340.87 (267.77 to 467.04)	19.9 (16.1 to 23.7)	283.18 (229.62 to 337.42)
Qinghai	Falls	19.1 (14.3 to 28)	403.1 (302.81 to 591.97)	22.3 (17.9 to 26.5)	355.49 (284.62 to 422.08)
Shaanxi	Falls	106 (87.5 to 125)	309.23 (254.35 to 364.31)	150 (105 to 186)	379.61 (266.08 to 470.34)
Shandong	Falls	182 (142 to 272)	212.59 (166.41 to 317.8)	298 (240 to 362)	299.52 (241.35 to 363.91)
Shanghai	Falls	52.7 (44.2 to 62.7)	376.86 (316.36 to 448.81)	183 (140 to 234)	653.82 (499.54 to 835.73)
Shanxi	Falls	73.9 (60.1 to 104)	243.97 (198.56 to 343.61)	123 (98.4 to 150)	318.1 (254.83 to 387.73)
Sichuan	Falls	458 (368 to 542)	410.81 (329.73 to 485.99)	518 (356 to 652)	620.13 (426.88 to 780.58)
Tianjin	Falls	17.8 (13.6 to 26)	193.26 (148.3 to 282.47)	39.6 (31.9 to 48.5)	259.51 (209.25 to 318)
Tibet	Falls	21 (13.3 to 25.5)	901.24 (572.33 to 1095.86)	18.7 (14 to 23.4)	541.7 (404.81 to 677.55)
Xinjiang	Falls	42 (32.3 to 71.7)	265.98 (204.51 to 454.27)	70.7 (57 to 88.3)	290.21 (233.79 to 362.56)
Yunnan	Falls	270 (187 to 330)	692.16 (480.59 to 847)	347 (229 to 428)	705.56 (466.48 to 871.36)
Zhejiang	Falls	225 (182 to 263)	511.2 (413.13 to 595.59)	436 (317 to 544)	713.05 (517.55 to 888.86)

China	Drowning	12600 (11700 to 13500)	1053.47 (978.24 to 1128.91)	3120 (2980 to 3260)	221.11 (210.69 to 230.97)
Taiwan	Drowning	99.1 (95.1 to 103)	485.63 (465.96 to 506.83)	22.8 (21.2 to 24.5)	96.58 (90.02 to 103.9)
Hong Kong Special Administrative Region of China	Drowning	5.51 (4.83 to 6.82)	95.52 (83.74 to 118.36)	3.76 (3.15 to 4.5)	50.06 (42.01 to 59.91)
Macau Special Administrative Region of China	Drowning	0.702 (0.628 to 0.787)	197.88 (177.06 to 221.72)	0.404 (0.344 to 0.481)	59.04 (50.3 to 70.3)
Anhui	Drowning	1150 (951 to 1360)	1941.06 (1604.76 to 2290.58)	194 (166 to 222)	317.56 (271.34 to 363.76)
Beijing	Drowning	12.4 (10.3 to 16.7)	110.34 (92.01 to 148.95)	9.02 (7.61 to 11.1)	37.41 (31.58 to 46.18)
Chongqing	Drowning	203 (160 to 248)	1286.06 (1012.64 to 1573.73)	63.8 (54.1 to 75)	238.72 (202.27 to 280.67)
Fujian	Drowning	268 (227 to 317)	846.61 (716.68 to 1000.61)	65.8 (56.5 to 77)	167 (143.32 to 195.5)
Gansu	Drowning	252 (209 to 303)	1067.28 (885.64 to 1281.37)	45.1 (39.4 to 51.7)	169.82 (148.34 to 194.46)
Guangdong	Drowning	490 (417 to 577)	744.29 (633.3 to 876.93)	195 (167 to 228)	166.96 (142.85 to 194.97)
Guangxi	Drowning	587 (501 to 689)	1350.49 (1151.61 to 1583.66)	172 (149 to 197)	356.31 (309.35 to 406.7)
Guizhou	Drowning	444 (358 to 548)	1337.06 (1077.44 to 1651.85)	75.8 (62.6 to 92.2)	224.01 (185.1 to 272.64)
Hainan	Drowning	74.1 (61.8 to 87.7)	1063.64 (887.32 to 1259.06)	25.5 (21.7 to 30.1)	265.94 (226.22 to 313.81)
Hebei	Drowning	308 (256 to 363)	484.53 (402.14 to 571.11)	114 (98.1 to 135)	149.72 (128.34 to 176.79)
Heilongjiang	Drowning	162 (137 to 189)	443.41 (376.24 to 518.85)	41.4 (34.7 to 49.8)	105.48 (88.33 to 126.97)
Henan	Drowning	794 (669 to 924)	899.65 (758.18 to 1046.69)	201 (171 to 233)	208.5 (177.29 to 241.77)
Hubei	Drowning	724 (607 to 831)	1303.97 (1092.99 to 1495.08)	185 (155 to 212)	334.68 (280.9 to 384.17)
Hunan	Drowning	813 (709 to 922)	1307.22 (1140.93 to 1482.55)	252 (215 to 287)	374.75 (320.39 to 426.77)
Inner Mongolia	Drowning	146 (121 to 177)	640.21 (533.63 to 778.74)	27.8 (23.9 to 33.1)	107.41 (92.28 to 127.95)
Jiangsu	Drowning	712 (594 to 834)	1034.68 (863.31 to 1211.35)	183 (151 to 211)	224.11 (185.3 to 258.33)
Jiangxi	Drowning	980 (809 to 1170)	2479.28 (2045.37 to 2963.64)	202 (176 to 229)	425 (370.31 to 482.44)
Jilin	Drowning	69.8 (57.7 to 92.4)	268.94 (222.2 to 356.21)	18.2 (14.7 to 23.8)	65.1 (52.63 to 85.18)
Liaoning	Drowning	120 (103 to 141)	293.15 (251.36 to 344.54)	43.7 (36.5 to 51.6)	97.84 (81.74 to 115.47)
Ningxia	Drowning	95.2 (73.2 to 120)	1927.42 (1483.15 to 2436.67)	18.6 (15.4 to 22.2)	264.35 (219.38 to 316.98)
Qinghai	Drowning	60.1 (46.1 to 79.7)	1269.46 (972.45 to 1681.87)	13.4 (11 to 16.3)	213.8 (174.67 to 260)
Shaanxi	Drowning	211 (169 to 259)	611.86 (492.01 to 753.67)	55.3 (46.4 to 66.9)	140.09 (117.47 to 169.27)
Shandong	Drowning	493 (424 to 563)	575.6 (495.48 to 657.74)	150 (129 to 173)	150.6 (129.55 to 174.58)
Shanghai	Drowning	49.3 (42.5 to 56.3)	352.83 (304.51 to 402.68)	28.7 (24.5 to 33.6)	102.75 (87.58 to 120.1)
Shanxi	Drowning	120 (100 to 146)	395.94 (330.83 to 482.8)	34.9 (29.6 to 41.7)	90.41 (76.53 to 108)
Sichuan	Drowning	1820 (1430 to 2210)	1627.91 (1278.05 to 1980.24)	272 (228 to 320)	326.21 (273.56 to 383.43)
Tianjin	Drowning	40 (35 to 46.4)	435.04 (380.64 to 503.96)	21 (18.1 to 24.2)	137.86 (118.64 to 158.51)
Tibet	Drowning	28.9 (22.2 to 38.6)	1240.55 (952.03 to 1655.44)	10.1 (7.95 to 13.3)	292.62 (230.07 to 384.97)
Xinjiang	Drowning	246 (189 to 298)	1561.8 (1199.99 to 1889.75)	101 (83.5 to 120)	413.27 (342.63 to 492.44)

Yunnan	Drowning	730 (573 to 901)	1874.24 (1469.51 to 2312.82)	181 (154 to 214)	367.16 (312.82 to 434.25)
Zhejiang	Drowning	404 (344 to 470)	915.81 (779.62 to 1066.26)	119 (101 to 139)	194.6 (165.88 to 227.88)
China	Fire, heat, and hot substances	1430 (1030 to 1670)	119.84 (86.22 to 139.47)	745 (566 to 1000)	52.75 (40.07 to 71.05)
Taiwan	Fire, heat, and hot substances	32.6 (30.1 to 35.5)	159.55 (147.57 to 174.2)	10.7 (8.83 to 13.1)	45.28 (37.43 to 55.38)
Hong Kong Special Administrative Region of China	Fire, heat, and hot substances	2.69 (1.83 to 3.5)	46.72 (31.7 to 60.74)	3.15 (2.23 to 4.37)	41.97 (29.75 to 58.23)
Macau Special Administrative Region of China	Fire, heat, and hot substances	0.175 (0.135 to 0.231)	49.42 (38.01 to 65.16)	0.275 (0.19 to 0.385)	40.17 (27.84 to 56.32)
Anhui	Fire, heat, and hot substances	83 (51.6 to 108)	140.07 (87.02 to 182.31)	23.5 (17.9 to 29.5)	38.35 (29.26 to 48.21)
Beijing	Fire, heat, and hot substances	10.3 (8.02 to 12.9)	91.67 (71.47 to 114.61)	18.3 (12.5 to 26.6)	76.14 (51.82 to 110.17)
Chongqing	Fire, heat, and hot substances	21.2 (15.6 to 25.6)	134.35 (98.88 to 162.49)	12.6 (9.33 to 17)	47.02 (34.91 to 63.61)
Fujian	Fire, heat, and hot substances	33.4 (26.3 to 40.7)	105.42 (83.15 to 128.72)	15.6 (12 to 21)	39.57 (30.5 to 53.39)
Gansu	Fire, heat, and hot substances	39.3 (24.7 to 53.5)	166.17 (104.25 to 225.99)	16.1 (11.8 to 21.6)	60.58 (44.29 to 81.12)
Guangdong	Fire, heat, and hot substances	58.7 (47.6 to 69.5)	89.18 (72.33 to 105.59)	48.7 (35.8 to 67.2)	41.68 (30.66 to 57.49)
Guangxi	Fire, heat, and hot substances	57.4 (35.4 to 72.7)	131.92 (81.49 to 167.14)	20.8 (15.7 to 26.1)	43 (32.5 to 54.08)
Guizhou	Fire, heat, and hot substances	66.2 (41.8 to 82.4)	199.46 (125.96 to 248.35)	25 (19.7 to 31.5)	73.87 (58.16 to 93)
Hainan	Fire, heat, and hot substances	7.41 (5.66 to 9.42)	106.42 (81.3 to 135.22)	5.26 (3.82 to 7.23)	54.86 (39.89 to 75.35)
Hebei	Fire, heat, and hot substances	62.8 (44.5 to 77.7)	98.59 (69.87 to 122.14)	32.5 (25.8 to 40.1)	42.59 (33.71 to 52.45)
Heilongjiang	Fire, heat, and hot substances	25.8 (19.3 to 35.9)	70.88 (52.96 to 98.41)	14.4 (10.6 to 19.4)	36.71 (27.03 to 49.42)
Henan	Fire, heat, and hot substances	107 (74.8 to 134)	121.78 (84.71 to 152.31)	37.7 (29.8 to 47.7)	39.15 (30.93 to 49.53)
Hubei	Fire, heat, and hot substances	69 (45.1 to 89.5)	124.16 (81.18 to 161.05)	23.6 (17 to 30.3)	42.72 (30.82 to 54.72)
Hunan	Fire, heat, and hot substances	88.5 (56.2 to 114)	142.38 (90.35 to 183.09)	34 (23.7 to 43.6)	50.61 (35.32 to 64.92)
Inner Mongolia	Fire, heat, and hot substances	27.9 (20.5 to 35.5)	122.59 (90.35 to 156.19)	11.7 (8.62 to 15.5)	45.01 (33.3 to 59.79)
Jiangsu	Fire, heat, and hot substances	77.9 (58.5 to 96.2)	113.23 (85.03 to 139.75)	51.9 (37.4 to 72)	63.5 (45.85 to 88.18)
Jiangxi	Fire, heat, and hot substances	72.7 (33.1 to 98.6)	184 (83.69 to 249.38)	32.3 (23.2 to 45.2)	67.85 (48.77 to 95.11)
Jilin	Fire, heat, and hot substances	18 (13.7 to 23.9)	69.28 (52.97 to 92.16)	11.3 (8.01 to 15.9)	40.44 (28.74 to 56.99)
Liaoning	Fire, heat, and hot substances	27.4 (22 to 34.6)	67.06 (53.96 to 84.72)	21 (15.2 to 29.2)	46.93 (34.01 to 65.3)
Ningxia	Fire, heat, and hot substances	8.26 (4.28 to 11.2)	167.24 (86.66 to 226.34)	3.34 (2.48 to 4.5)	47.59 (35.27 to 64.15)
Qinghai	Fire, heat, and hot substances	6.71 (3.78 to 11.9)	141.68 (79.89 to 250.7)	2.56 (1.75 to 3.74)	40.75 (27.78 to 59.54)
Shaanxi	Fire, heat, and hot substances	42.6 (28.1 to 56)	123.8 (81.62 to 162.87)	21 (15 to 29.2)	53.07 (37.98 to 73.99)
Shandong	Fire, heat, and hot substances	83.2 (56.5 to 110)	97.18 (66.03 to 128.27)	64.3 (43.9 to 93.3)	64.75 (44.16 to 93.83)
Shanghai	Fire, heat, and hot substances	12.1 (8.91 to 16.7)	86.88 (63.74 to 119.84)	27.1 (16.7 to 42.5)	97.16 (59.76 to 152.1)
Shanxi	Fire, heat, and hot substances	27 (19.9 to 33.1)	89.06 (65.66 to 109.47)	20.4 (14.4 to 29.2)	52.84 (37.17 to 75.63)
Sichuan	Fire, heat, and hot substances	140 (93.1 to 177)	125.8 (83.45 to 158.56)	49.7 (35.8 to 68.6)	59.47 (42.91 to 82.15)
Tianjin	Fire, heat, and hot substances	7.54 (5.24 to 9.86)	81.97 (56.94 to 107.17)	8.49 (6.04 to 11.8)	55.65 (39.6 to 77.43)

Tibet	Fire, heat, and hot substances	3.21<br>(1.53 to 7.24)	137.92<br>(65.57 to 310.76)	1.61<br>(0.864 to 3.16)	46.5<br>(25.01 to 91.52)
Xinjiang	Fire, heat, and hot substances	19.2<br>(9.88 to 26.5)	122.03<br>(62.66 to 167.82)	10.4<br>(7.86 to 13.9)	42.88<br>(32.24 to 57.17)
Yunnan	Fire, heat, and hot substances	73.5<br>(38.7 to 96.8)	188.48<br>(99.18 to 248.29)	30.8<br>(23.5 to 41.5)	62.7<br>(47.81 to 84.46)
Zhejiang	Fire, heat, and hot substances	53.6<br>(33 to 72.7)	121.47<br>(74.83 to 164.92)	45.8<br>(30.3 to 69.2)	74.91<br>(49.45 to 113.13)
China	Poisonings	1440<br>(1130 to 2170)	119.97<br>(94.82 to 181.01)	1050<br>(603 to 1250)	74.44<br>(42.72 to 88.63)
Taiwan	Poisonings	27.3<br>(26.1 to 28.5)	133.93<br>(127.98 to 139.9)	4.13<br>(3.73 to 4.6)	17.53<br>(15.84 to 19.51)
Hong Kong Special Administrative Region of China	Poisonings	1.51<br>(1.12 to 3.07)	26.27<br>(19.43 to 53.21)	2.23<br>(1.77 to 3.35)	29.65<br>(23.63 to 44.66)
Macau Special Administrative Region of China	Poisonings	0.108<br>(0.0808 to 0.212)	30.4<br>(22.76 to 59.86)	0.131<br>(0.0915 to 0.241)	19.18<br>(13.38 to 35.18)
Anhui	Poisonings	62.7<br>(43.4 to 124)	105.78<br>(73.23 to 208.85)	36.2<br>(26.2 to 44.4)	59.16<br>(42.9 to 72.66)
Beijing	Poisonings	5.05<br>(3.74 to 9.6)	45.01<br>(33.36 to 85.57)	7<br>(5.13 to 10.2)	29.03<br>(21.29 to 42.39)
Chongqing	Poisonings	18<br>(12.5 to 33.3)	113.83<br>(79.4 to 210.75)	15.9<br>(10.5 to 19.7)	59.56<br>(39.36 to 73.52)
Fujian	Poisonings	19.8<br>(12.8 to 47.8)	62.68<br>(40.45 to 151.04)	14.5<br>(11.7 to 20.5)	36.84<br>(29.7 to 52.04)
Gansu	Poisonings	51.8<br>(29.8 to 78.4)	218.94<br>(125.79 to 331.65)	30.7<br>(14.3 to 40.1)	115.6<br>(53.63 to 150.78)
Guangdong	Poisonings	43.4<br>(30.7 to 90.9)	65.93<br>(46.58 to 138.07)	50.8<br>(38.3 to 64.1)	43.48<br>(32.74 to 54.86)
Guangxi	Poisonings	47.8<br>(34.6 to 90.7)	109.85<br>(79.46 to 208.61)	39.6<br>(25.1 to 49.9)	81.94<br>(51.9 to 103.3)
Guizhou	Poisonings	68.8<br>(46 to 110)	207.24<br>(138.69 to 331.2)	34.4<br>(20.2 to 46.2)	101.63<br>(59.81 to 136.61)
Hainan	Poisonings	8.65<br>(6.25 to 11.9)	124.21<br>(89.76 to 170.27)	4.43<br>(3.57 to 6.22)	46.16<br>(37.28 to 64.85)
Hebei	Poisonings	105<br>(64.6 to 129)	164.71<br>(101.49 to 203.08)	92<br>(40.1 to 128)	120.42<br>(52.44 to 167.75)
Heilongjiang	Poisonings	40.7<br>(29.8 to 55.6)	111.68<br>(81.73 to 152.58)	23.6<br>(16.4 to 28.9)	60<br>(41.86 to 73.67)
Henan	Poisonings	123<br>(89.5 to 154)	139.56<br>(101.37 to 174.3)	97.5<br>(40.6 to 125)	101.22<br>(42.13 to 130.17)
Hubei	Poisonings	47.3<br>(34.9 to 87.3)	85.19<br>(62.86 to 157.22)	33.7<br>(22.3 to 41.2)	60.88<br>(40.35 to 74.5)
Hunan	Poisonings	68.9<br>(53.2 to 120)	110.88<br>(85.49 to 193.29)	50.4<br>(33.1 to 62.2)	75.07<br>(49.31 to 92.64)
Inner Mongolia	Poisonings	29.8<br>(21.8 to 47.9)	130.95<br>(96.05 to 210.5)	15.6<br>(10.7 to 19.3)	60.27<br>(41.34 to 74.57)
Jiangsu	Poisonings	34.2<br>(22.1 to 83.8)	49.73<br>(32.07 to 121.72)	29<br>(23.7 to 40.5)	35.49<br>(29.05 to 49.63)
Jiangxi	Poisonings	62.6<br>(43.9 to 120)	158.34<br>(110.95 to 303.77)	32.8<br>(23 to 39.7)	69.03<br>(48.37 to 83.44)
Jilin	Poisonings	26.2<br>(17.7 to 43.6)	101.04<br>(68.35 to 168.06)	18.7<br>(11 to 23.8)	66.91<br>(39.34 to 85.16)
Liaoning	Poisonings	43.2<br>(32.3 to 52.9)	105.89<br>(79.06 to 129.72)	32.1<br>(16.3 to 40.8)	71.86<br>(36.51 to 91.41)
Ningxia	Poisonings	12.6<br>(7.07 to 17.5)	254.3<br>(143.22 to 354.29)	7.57<br>(3.14 to 10)	107.92<br>(44.8 to 142.68)
Qinghai	Poisonings	13<br>(7.49 to 18.8)	274.57<br>(158.04 to 396.09)	9.46<br>(4.1 to 12.5)	150.62<br>(65.23 to 198.84)
Shaanxi	Poisonings	50.5<br>(37.3 to 62.5)	146.72<br>(108.5 to 181.65)	46.9<br>(17.3 to 61.9)	118.63<br>(43.87 to 156.68)
Shandong	Poisonings	92.9<br>(73.5 to 125)	108.53<br>(85.92 to 146.45)	84.3<br>(34.6 to 109)	84.83<br>(34.77 to 110.09)
Shanghai	Poisonings	3.54<br>(1.93 to 10.5)	25.31<br>(13.83 to 74.83)	4.88<br>(3.36 to 9.97)	17.46<br>(12.03 to 35.67)
Shanxi	Poisonings	63<br>(34.8 to 79.1)	208.13<br>(114.88 to 261.26)	53.7<br>(14.7 to 72.8)	139.09<br>(38.04 to 188.5)

Sichuan	Poisonings	133<br>(90.7 to 238)	118.89<br>(81.26 to 213.11)	66.5<br>(39.9 to 84.1)	79.65<br>(47.78 to 100.78)
Tianjin	Poisonings	7.5<br>(6.11 to 10.2)	81.53<br>(66.46 to 111.09)	9.81<br>(4.86 to 12.7)	64.27<br>(31.85 to 83.05)
Tibet	Poisonings	3.79<br>(1.28 to 12.7)	162.52<br>(54.79 to 543.18)	1.9<br>(1.08 to 5.02)	54.98<br>(31.1 to 145.24)
Xinjiang	Poisonings	30.7<br>(17.8 to 43.2)	194.47<br>(112.85 to 273.68)	24.1<br>(13.7 to 34.8)	98.78<br>(56.17 to 142.76)
Yunnan	Poisonings	94.3<br>(52.5 to 149)	241.9<br>(134.74 to 381.62)	63.1<br>(31.6 to 81.4)	128.4<br>(64.26 to 165.48)
Zhejiang	Poisonings	23.1<br>(14.7 to 56.7)	52.3<br>(33.24 to 128.6)	18.1<br>(14.4 to 28.2)	29.53<br>(23.59 to 46.05)
China	Exposure to mechanical forces	2070<br>(1860 to 2880)	172.54<br>(155.77 to 240.34)	2160<br>(1730 to 2560)	152.97<br>(122.66 to 181.4)
Taiwan	Exposure to mechanical forces	19.6<br>(17.8 to 22)	96.29<br>(87.19 to 107.82)	18.3<br>(14.7 to 23.3)	77.71<br>(62.49 to 98.65)
Hong Kong Special Administrative Region of China	Exposure to mechanical forces	3.44<br>(2.65 to 5.03)	59.67<br>(46.04 to 87.34)	7.16<br>(5.42 to 9.46)	95.29<br>(72.14 to 126.02)
Macau Special Administrative Region of China	Exposure to mechanical forces	0.319<br>(0.267 to 0.381)	89.87<br>(75.35 to 107.34)	0.609<br>(0.456 to 0.811)	89<br>(66.66 to 118.6)
Anhui	Exposure to mechanical forces	94.7<br>(78.8 to 146)	159.83<br>(132.92 to 245.81)	77.5<br>(62.3 to 92.3)	126.72<br>(101.95 to 150.95)
Beijing	Exposure to mechanical forces	10.5<br>(8 to 15)	94.06<br>(71.3 to 133.98)	37.2<br>(26.7 to 51.4)	154.39<br>(110.58 to 213.37)
Chongqing	Exposure to mechanical forces	40.8<br>(34 to 48)	258.32<br>(215.62 to 303.94)	41.3<br>(25.3 to 51.9)	154.61<br>(94.56 to 194.05)
Fujian	Exposure to mechanical forces	42.2<br>(36.1 to 57.6)	133.41<br>(114.07 to 182.04)	60.1<br>(46.9 to 76)	152.57<br>(118.95 to 192.71)
Gansu	Exposure to mechanical forces	65.3<br>(54.2 to 113)	275.97<br>(229.33 to 475.83)	40.4<br>(31.9 to 47.9)	152.07<br>(119.89 to 180.31)
Guangdong	Exposure to mechanical forces	69.6<br>(55 to 120)	105.8<br>(83.59 to 182.4)	142<br>(111 to 183)	121.9<br>(95.19 to 156.66)
Guangxi	Exposure to mechanical forces	61.8<br>(48.8 to 122)	142.07<br>(112.17 to 279.74)	57.7<br>(48.1 to 69.8)	119.5<br>(99.57 to 144.39)
Guizhou	Exposure to mechanical forces	81.2<br>(66.7 to 137)	244.54<br>(201.03 to 411.62)	60.1<br>(49.1 to 73)	177.71<br>(145.23 to 215.71)
Hainan	Exposure to mechanical forces	19.4<br>(15.6 to 23.6)	277.85<br>(224.41 to 339.16)	12.5<br>(10.2 to 15.2)	130.76<br>(106.25 to 158.4)
Hebei	Exposure to mechanical forces	127<br>(111 to 146)	199.8<br>(174.71 to 229.17)	144<br>(87.3 to 177)	188.87<br>(114.29 to 231.57)
Heilongjiang	Exposure to mechanical forces	60.3<br>(50.7 to 79.7)	165.33<br>(139.14 to 218.59)	41.3<br>(34.1 to 49.8)	105.32<br>(86.97 to 126.77)
Henan	Exposure to mechanical forces	141<br>(120 to 181)	159.76<br>(135.8 to 204.75)	142<br>(93.4 to 173)	147.8<br>(96.95 to 179.37)
Hubei	Exposure to mechanical forces	69.8<br>(57.3 to 113)	125.55<br>(103.19 to 202.93)	62.9<br>(50 to 76)	113.79<br>(90.35 to 137.51)
Hunan	Exposure to mechanical forces	130<br>(109 to 149)	209.23<br>(174.74 to 238.97)	141<br>(81.1 to 176)	209.7<br>(120.84 to 261.83)
Inner Mongolia	Exposure to mechanical forces	34.5<br>(27.7 to 65.1)	151.77<br>(121.98 to 286.11)	30.3<br>(25 to 36.6)	117.07<br>(96.37 to 141.27)
Jiangsu	Exposure to mechanical forces	94.7<br>(79.4 to 125)	137.59<br>(115.45 to 181.46)	148<br>(114 to 189)	180.82<br>(139.6 to 230.86)
Jiangxi	Exposure to mechanical forces	93.5<br>(78.2 to 135)	236.5<br>(197.69 to 342.29)	68.9<br>(53.4 to 82.9)	144.97<br>(112.33 to 174.4)
Jilin	Exposure to mechanical forces	43.6<br>(36.7 to 59.2)	168.14<br>(141.33 to 228.03)	26.9<br>(21.7 to 32.9)	96.55<br>(77.83 to 117.82)
Liaoning	Exposure to mechanical forces	65.9<br>(54.2 to 83.7)	161.48<br>(132.76 to 205.15)	60.1<br>(47.4 to 76.9)	134.49<br>(106.2 to 172.3)
Ningxia	Exposure to mechanical forces	11.2<br>(9 to 15.5)	226.16<br>(182.18 to 314.28)	9.57<br>(6.66 to 11.7)	136.39<br>(94.94 to 166.96)
Qinghai	Exposure to mechanical forces	14.2<br>(11.4 to 22.4)	299.63<br>(240.12 to 472.78)	13.3<br>(9.89 to 16.6)	212.38<br>(157.45 to 264.1)
Shaanxi	Exposure to mechanical forces	48<br>(40.5 to 64.9)	139.56<br>(117.69 to 188.62)	56.9<br>(37.9 to 69.5)	144.1<br>(95.8 to 175.99)
Shandong	Exposure to mechanical forces	108<br>(87.5 to 169)	126.71<br>(102.27 to 197.3)	140<br>(111 to 174)	140.75<br>(111.54 to 174.67)

Shanghai	Exposure to mechanical forces	14.2 (10.9 to 19)	101.37 (78.35 to 136.26)	49.7 (35.5 to 70)	178.06 (126.99 to 250.61)
Shanxi	Exposure to mechanical forces	64.5 (55.3 to 77.7)	212.96 (182.66 to 256.57)	67.6 (41.6 to 83.6)	174.93 (107.82 to 216.56)
Sichuan	Exposure to mechanical forces	229 (195 to 286)	205.03 (175.14 to 256.24)	155 (112 to 194)	185.81 (134.71 to 231.79)
Tianjin	Exposure to mechanical forces	7.62 (5.99 to 12.3)	82.83 (65.15 to 133.63)	13 (10.5 to 15.9)	84.97 (68.97 to 103.93)
Tibet	Exposure to mechanical forces	21.1 (16.4 to 25.5)	903.38 (705.58 to 1094.13)	13.9 (9.95 to 17.4)	402.81 (287.79 to 503.25)
Xinjiang	Exposure to mechanical forces	25.1 (19.2 to 51.8)	159.3 (121.94 to 328.43)	35.5 (28.1 to 44.2)	145.78 (115.43 to 181.24)
Yunnan	Exposure to mechanical forces	116 (94.9 to 194)	297.6 (243.41 to 497.05)	105 (84.3 to 127)	214.06 (171.42 to 257.43)
Zhejiang	Exposure to mechanical forces	56.4 (48.5 to 67.7)	127.95 (109.94 to 153.61)	98 (73.6 to 130)	160.18 (120.32 to 213.18)
China	Adverse effects of medical treatment	826 (532 to 983)	69.04 (44.45 to 82.12)	322 (271 to 401)	22.77 (19.16 to 28.41)
Taiwan	Adverse effects of medical treatment	2.68 (2.37 to 3.29)	13.13 (11.63 to 16.11)	7.15 (4.71 to 8.22)	30.33 (19.96 to 34.84)
Hong Kong Special Administrative Region of China	Adverse effects of medical treatment	1.52 (0.942 to 1.84)	26.42 (16.34 to 31.89)	1.36 (1.04 to 1.79)	18.15 (13.84 to 23.79)
Macau Special Administrative Region of China	Adverse effects of medical treatment	0.087 (0.0643 to 0.112)	24.52 (18.13 to 31.6)	0.114 (0.0816 to 0.15)	16.71 (11.94 to 21.9)
Anhui	Adverse effects of medical treatment	48.6 (31.6 to 63.5)	82.09 (53.33 to 107.24)	13.4 (10.7 to 17.3)	21.93 (17.55 to 28.25)
Beijing	Adverse effects of medical treatment	4.51 (3.2 to 5.53)	40.19 (28.51 to 49.27)	6.33 (4.6 to 8.49)	26.28 (19.07 to 35.21)
Chongqing	Adverse effects of medical treatment	9.84 (6.6 to 13.5)	62.36 (41.83 to 85.39)	4.95 (3.59 to 7.01)	18.51 (13.42 to 26.22)
Fujian	Adverse effects of medical treatment	24.9 (12.3 to 36)	78.64 (38.88 to 113.89)	9.84 (7.67 to 12.3)	24.98 (19.45 to 31.32)
Gansu	Adverse effects of medical treatment	37.2 (14.5 to 55.1)	157.29 (61.41 to 232.93)	7.42 (5.47 to 9.08)	27.94 (20.59 to 34.15)
Guangdong	Adverse effects of medical treatment	32.8 (23.2 to 40.8)	49.86 (35.25 to 62.04)	23.1 (17.8 to 31.5)	19.8 (15.26 to 26.96)
Guangxi	Adverse effects of medical treatment	30.4 (19.3 to 40)	69.88 (44.44 to 92.08)	12.1 (9.92 to 15.2)	24.98 (20.54 to 31.42)
Guizhou	Adverse effects of medical treatment	41.2 (20.6 to 60.4)	124.13 (61.99 to 181.83)	9.68 (7.2 to 11.8)	28.63 (21.27 to 34.97)
Hainan	Adverse effects of medical treatment	4.78 (2.58 to 6.8)	68.57 (37.03 to 97.56)	3.06 (2.18 to 3.87)	31.89 (22.71 to 40.38)
Hebei	Adverse effects of medical treatment	32.2 (23.8 to 39.6)	50.56 (37.37 to 62.19)	19.5 (15.3 to 26.2)	25.58 (20.02 to 34.34)
Heilongjiang	Adverse effects of medical treatment	13.5 (9.73 to 20.7)	36.91 (26.7 to 56.87)	8.66 (5.92 to 12.6)	22.05 (15.09 to 31.99)
Henan	Adverse effects of medical treatment	57.2 (39.5 to 73.4)	64.84 (44.72 to 83.15)	19.9 (15.7 to 26.7)	20.68 (16.32 to 27.77)
Hubei	Adverse effects of medical treatment	34.4 (23.6 to 43.3)	61.86 (42.54 to 78.02)	11.5 (8.76 to 15.4)	20.71 (15.84 to 27.91)
Hunan	Adverse effects of medical treatment	36.4 (27.2 to 44)	58.61 (43.76 to 70.71)	14.8 (11.4 to 19.8)	22.06 (17.04 to 29.48)
Inner Mongolia	Adverse effects of medical treatment	21.6 (10.5 to 30.9)	94.97 (46.14 to 135.69)	6.18 (4.95 to 7.6)	23.87 (19.11 to 29.36)
Jiangsu	Adverse effects of medical treatment	26.7 (21.9 to 32.6)	38.86 (31.77 to 47.39)	15.9 (11.7 to 21.9)	19.5 (14.3 to 26.87)
Jiangxi	Adverse effects of medical treatment	47.9 (20.7 to 70.2)	121.12 (52.27 to 177.63)	12.8 (10.1 to 15.8)	27 (21.29 to 33.2)
Jilin	Adverse effects of medical treatment	13.5 (10.6 to 16.4)	51.96 (41.01 to 63.24)	6.02 (4.65 to 8.06)	21.57 (16.67 to 28.91)
Liaoning	Adverse effects of medical treatment	12.4 (9.25 to 18.4)	30.41 (22.67 to 45.22)	9.44 (6.56 to 13.6)	21.15 (14.69 to 30.55)
Ningxia	Adverse effects of medical treatment	7.57 (2.58 to 12.1)	153.32 (52.16 to 244.76)	1.98 (1.38 to 2.54)	28.14 (19.64 to 36.23)
Qinghai	Adverse effects of medical treatment	8.65 (3.28 to 13.6)	182.54 (69.22 to 288.14)	2.26 (1.49 to 2.89)	35.91 (23.66 to 45.93)

Shaanxi	Adverse effects of medical treatment	28.3<br>(15.7 to 39.4)	82.29<br>(45.65 to 114.49)	9.42<br>(7.44 to 11.7)	23.83<br>(18.84 to 29.63)
Shandong	Adverse effects of medical treatment	33.3<br>(27.5 to 42.2)	38.85<br>(32.18 to 49.36)	16.9<br>(12.1 to 24.9)	17.01<br>(12.2 to 25.05)
Shanghai	Adverse effects of medical treatment	4.89<br>(3.58 to 5.9)	34.99<br>(25.62 to 42.26)	5.99<br>(4.49 to 7.9)	21.42<br>(16.08 to 28.27)
Shanxi	Adverse effects of medical treatment	18<br>(12.7 to 22.4)	59.6<br>(41.8 to 73.85)	8.53<br>(6.53 to 11.3)	22.09<br>(16.9 to 29.16)
Sichuan	Adverse effects of medical treatment	79.6<br>(46.4 to 109)	71.3<br>(41.57 to 97.98)	17.4<br>(13.4 to 24.6)	20.84<br>(16.02 to 29.42)
Tianjin	Adverse effects of medical treatment	2.73<br>(2.16 to 3.88)	29.71<br>(23.52 to 42.19)	2.8<br>(1.92 to 4.15)	18.38<br>(12.61 to 27.17)
Tibet	Adverse effects of medical treatment	7.14<br>(1.42 to 11.8)	306.57<br>(61.01 to 508.21)	2.78<br>(1.09 to 4.34)	80.55<br>(31.52 to 125.48)
Xinjiang	Adverse effects of medical treatment	18.3<br>(10 to 25.5)	115.87<br>(63.62 to 161.87)	9.29<br>(6.08 to 12.2)	38.13<br>(24.94 to 49.88)
Yunnan	Adverse effects of medical treatment	59.2<br>(21.8 to 90)	151.99<br>(55.83 to 230.92)	15.4<br>(11.2 to 19.1)	31.31<br>(22.69 to 38.79)
Zhejiang	Adverse effects of medical treatment	27.1<br>(14.2 to 36.9)	61.4<br>(32.26 to 83.76)	12.8<br>(10.1 to 16.1)	20.89<br>(16.58 to 26.29)
China	Animal contact	534<br>(246 to 634)	44.59<br>(20.52 to 53)	173<br>(140 to 218)	12.27<br>(9.89 to 15.43)
Taiwan	Animal contact	1.09<br>(0.903 to 1.32)	5.33<br>(4.43 to 6.48)	0.999<br>(0.802 to 1.25)	4.24<br>(3.4 to 5.3)
Hong Kong Special Administrative Region of China	Animal contact	0.487<br>(0.393 to 0.641)	8.44<br>(6.81 to 11.13)	0.485<br>(0.381 to 0.645)	6.46<br>(5.08 to 8.59)
Macau Special Administrative Region of China	Animal contact	0.0677<br>(0.0288 to 0.084)	19.08<br>(8.12 to 23.66)	0.0725<br>(0.0321 to 0.0965)	10.6<br>(4.69 to 14.12)
Anhui	Animal contact	17.5<br>(10.1 to 21.6)	29.58<br>(17.06 to 36.47)	3.8<br>(2.97 to 6.09)	6.22<br>(4.85 to 9.96)
Beijing	Animal contact	2.21<br>(1.67 to 2.92)	19.68<br>(14.92 to 26.05)	3.7<br>(2.48 to 5.4)	15.34<br>(10.28 to 22.4)
Chongqing	Animal contact	8.9<br>(3.11 to 11.7)	56.4<br>(19.74 to 73.94)	3.27<br>(2.51 to 4.09)	12.22<br>(9.39 to 15.3)
Fujian	Animal contact	13.5<br>(5.11 to 17.6)	42.73<br>(16.14 to 55.77)	4.37<br>(3.41 to 5.51)	11.09<br>(8.65 to 13.99)
Gansu	Animal contact	11.9<br>(5.65 to 14.8)	50.43<br>(23.9 to 62.66)	2.16<br>(1.72 to 3.4)	8.12<br>(6.46 to 12.79)
Guangdong	Animal contact	24.4<br>(12.1 to 30.9)	37.11<br>(18.36 to 46.92)	15.8<br>(11.7 to 21.3)	13.5<br>(10.03 to 18.22)
Guangxi	Animal contact	37.8<br>(9.94 to 49.5)	87.03<br>(22.85 to 113.82)	7.85<br>(4.35 to 9.92)	16.24<br>(9.01 to 20.53)
Guizhou	Animal contact	38.8<br>(9.38 to 50.9)	117.04<br>(28.25 to 153.5)	8.62<br>(3.67 to 11.2)	25.49<br>(10.84 to 33.24)
Hainan	Animal contact	4.81<br>(1.48 to 6.44)	68.99<br>(21.18 to 92.48)	2.45<br>(1.17 to 3.14)	25.57<br>(12.22 to 32.71)
Hebei	Animal contact	11<br>(9.02 to 13.9)	17.35<br>(14.17 to 21.87)	4.93<br>(3.55 to 8.32)	6.45<br>(4.65 to 10.89)
Heilongjiang	Animal contact	4.64<br>(3.39 to 7.95)	12.73<br>(9.3 to 21.81)	2.04<br>(1.32 to 4.36)	5.19<br>(3.37 to 11.11)
Henan	Animal contact	19.2<br>(14.3 to 22.8)	21.75<br>(16.2 to 25.83)	5.4<br>(3.96 to 9.52)	5.6<br>(4.12 to 9.88)
Hubei	Animal contact	27.9<br>(9.88 to 36.2)	50.25<br>(17.79 to 65.22)	5.84<br>(3.96 to 7.09)	10.57<br>(7.16 to 12.81)
Hunan	Animal contact	26.1<br>(11.6 to 32.4)	41.92<br>(18.59 to 52.16)	7.89<br>(5.46 to 9.81)	11.74<br>(8.13 to 14.61)
Inner Mongolia	Animal contact	5.31<br>(4.31 to 6.66)	23.34<br>(18.94 to 29.3)	2.16<br>(1.52 to 3.74)	8.33<br>(5.88 to 14.46)
Jiangsu	Animal contact	11.8<br>(9.32 to 15.3)	17.12<br>(13.54 to 22.17)	9.32<br>(6.26 to 13.6)	11.41<br>(7.66 to 16.6)
Jiangxi	Animal contact	32<br>(10 to 41.2)	80.85<br>(25.35 to 104.32)	7<br>(5.28 to 8.74)	14.73<br>(11.12 to 18.39)
Jilin	Animal contact	4.5<br>(3.37 to 6.49)	17.33<br>(12.98 to 25.03)	1.81<br>(1.22 to 3.3)	6.49<br>(4.36 to 11.82)
Liaoning	Animal contact	7.09<br>(5.47 to 9.46)	17.37<br>(13.41 to 23.19)	3.15<br>(2.22 to 5.03)	7.06<br>(4.97 to 11.27)

Ningxia	Animal contact	2.23 (1.26 to 2.93)	45.06 (25.47 to 59.24)	0.566 (0.432 to 0.838)	8.06 (6.16 to 11.94)
Qinghai	Animal contact	3.16 (1.4 to 4.09)	66.71 (29.64 to 86.36)	0.789 (0.635 to 1.1)	12.57 (10.11 to 17.51)
Shaanxi	Animal contact	18.2 (7.33 to 23.6)	52.79 (21.3 to 68.46)	4.65 (3.45 to 5.74)	11.77 (8.72 to 14.52)
Shandong	Animal contact	14.3 (11.3 to 18.8)	16.68 (13.16 to 21.96)	5.48 (3.58 to 10.3)	5.51 (3.61 to 10.32)
Shanghai	Animal contact	1.8 (1.41 to 2.31)	12.86 (10.11 to 16.53)	1.39 (0.958 to 2.13)	4.96 (3.43 to 7.63)
Shanxi	Animal contact	10.5 (6.02 to 13.1)	34.55 (19.86 to 43.34)	4.83 (3.58 to 6.55)	12.49 (9.26 to 16.96)
Sichuan	Animal contact	77.7 (30.6 to 100)	69.58 (27.39 to 89.73)	21.3 (15.7 to 27.8)	25.56 (18.84 to 33.3)
Tianjin	Animal contact	1.2 (0.868 to 1.76)	13.08 (9.44 to 19.14)	0.854 (0.546 to 1.51)	5.6 (3.58 to 9.87)
Tibet	Animal contact	2.87 (1.01 to 3.7)	123.3 (43.46 to 158.82)	0.762 (0.6 to 1.03)	22.05 (17.35 to 29.89)
Xinjiang	Animal contact	8.82 (3.83 to 11.3)	55.89 (24.31 to 71.52)	4 (2.55 to 5.07)	16.43 (10.48 to 20.8)
Yunnan	Animal contact	65.2 (14.2 to 87.1)	167.26 (36.41 to 223.55)	14 (8.33 to 17.4)	28.41 (16.95 to 35.32)
Zhejiang	Animal contact	17.9 (9.87 to 22.9)	40.57 (22.37 to 52.02)	12.6 (8.97 to 17.4)	20.53 (14.67 to 28.46)
China	Foreign body	2770 (2490 to 3320)	231.16 (207.76 to 277.57)	1080 (946 to 1180)	76.33 (66.95 to 83.49)
Taiwan	Foreign body	28.3 (25.8 to 30.9)	138.87 (126.63 to 151.56)	13.6 (12.4 to 14.8)	57.48 (52.6 to 62.9)
Hong Kong Special Administrative Region of China	Foreign body	2.1 (1.83 to 2.47)	36.46 (31.75 to 42.83)	2.36 (1.95 to 2.84)	31.42 (26.02 to 37.88)
Macau Special Administrative Region of China	Foreign body	0.149 (0.125 to 0.182)	42.05 (35.31 to 51.23)	0.16 (0.129 to 0.201)	23.43 (18.86 to 29.34)
Anhui	Foreign body	186 (140 to 245)	313.42 (235.47 to 413.57)	46.6 (35.9 to 58.4)	76.17 (58.79 to 95.44)
Beijing	Foreign body	10.6 (8.65 to 13.1)	94.29 (77.13 to 116.5)	16.6 (13.2 to 20.3)	69.04 (54.91 to 84.19)
Chongqing	Foreign body	46.3 (31.3 to 63.4)	293.2 (198.1 to 401.58)	19.4 (15.1 to 24.2)	72.46 (56.58 to 90.52)
Fujian	Foreign body	62.1 (49.2 to 78)	196.2 (155.48 to 246.6)	35.4 (28.4 to 42.5)	89.7 (72.06 to 107.83)
Gansu	Foreign body	117 (86.3 to 160)	494.63 (364.85 to 675.35)	26.8 (21 to 34.4)	100.97 (78.83 to 129.43)
Guangdong	Foreign body	72.5 (58.1 to 97)	110.22 (88.23 to 147.45)	72.1 (58.3 to 86.7)	61.72 (49.89 to 74.24)
Guangxi	Foreign body	56.6 (42.5 to 80.4)	130.12 (97.63 to 184.93)	28.5 (23.2 to 34.9)	58.93 (47.92 to 72.21)
Guizhou	Foreign body	108 (81.7 to 142)	326.81 (246.21 to 428.9)	23.7 (19.5 to 29)	70.2 (57.58 to 85.59)
Hainan	Foreign body	12.2 (8.81 to 19.3)	174.7 (126.43 to 276.43)	8.25 (6.4 to 11.7)	86.02 (66.76 to 121.74)
Hebei	Foreign body	114 (87.7 to 148)	178.98 (137.73 to 232.64)	69.8 (55.9 to 86.4)	91.32 (73.11 to 113.03)
Heilongjiang	Foreign body	26.8 (19.5 to 47.9)	73.44 (53.61 to 131.5)	13.7 (11.4 to 17.6)	34.89 (28.98 to 44.92)
Henan	Foreign body	225 (167 to 294)	254.87 (189.73 to 333.5)	67.4 (51.1 to 84.9)	69.98 (53.08 to 88.18)
Hubei	Foreign body	118 (88 to 156)	211.5 (158.34 to 280.79)	35.5 (28.4 to 42.8)	64.18 (51.44 to 77.45)
Hunan	Foreign body	106 (80.1 to 137)	170.01 (128.73 to 220.78)	35.4 (29.3 to 42)	52.7 (43.59 to 62.5)
Inner Mongolia	Foreign body	51.7 (38.2 to 76.8)	227.34 (167.84 to 337.51)	16.9 (13.5 to 21.1)	65.16 (52.31 to 81.55)
Jiangsu	Foreign body	101 (80.2 to 129)	147.4 (116.52 to 187.25)	49.8 (40.4 to 60)	60.95 (49.54 to 73.5)
Jiangxi	Foreign body	190 (140 to 251)	480.29 (354.35 to 635.93)	68.1 (50 to 86.3)	143.3 (105.26 to 181.63)

Jilin	Foreign body	27.2 (19.8 to 44.6)	104.93 (76.25 to 171.74)	11.7 (9.74 to 15.1)	41.94 (34.94 to 54.01)
Liaoning	Foreign body	24.4 (19.1 to 38.2)	59.92 (46.92 to 93.64)	14 (11.7 to 17.5)	31.36 (26.25 to 39.12)
Ningxia	Foreign body	25.5 (18 to 35.8)	516.59 (364.88 to 724.58)	7.9 (5.87 to 10.4)	112.55 (83.6 to 147.61)
Qinghai	Foreign body	25.5 (18.4 to 35)	537.8 (387.46 to 739.78)	8.81 (6.39 to 11.5)	140.3 (101.71 to 182.36)
Shaanxi	Foreign body	98.9 (74.3 to 130)	287.34 (215.97 to 379.24)	41.8 (32.2 to 53)	105.88 (81.48 to 134.27)
Shandong	Foreign body	75.5 (58.2 to 110)	88.16 (68.03 to 129)	37.3 (31.4 to 44.3)	37.51 (31.63 to 44.56)
Shanghai	Foreign body	13.6 (11.1 to 16.7)	97.32 (79.52 to 119.81)	20.2 (16.3 to 24.6)	72.43 (58.52 to 87.97)
Shanxi	Foreign body	70.4 (53.1 to 94.1)	232.36 (175.26 to 310.66)	37.1 (29.8 to 45)	95.95 (77.24 to 116.48)
Sichuan	Foreign body	409 (296 to 547)	366.3 (265.39 to 490.35)	90.4 (71.7 to 113)	108.27 (85.83 to 135.26)
Tianjin	Foreign body	5.59 (4.41 to 9.36)	60.79 (47.9 to 101.73)	6.72 (5.54 to 8.65)	44.01 (36.33 to 56.7)
Tibet	Foreign body	9.4 (6.86 to 12.4)	403.58 (294.38 to 532.13)	7.99 (5.07 to 10.8)	231.1 (146.59 to 313.23)
Xinjiang	Foreign body	44.9 (31.9 to 73.3)	284.77 (202.22 to 464.98)	26.1 (19.5 to 38.9)	107.09 (79.83 to 159.47)
Yunnan	Foreign body	171 (128 to 230)	437.56 (328.15 to 590.17)	64.1 (49.4 to 82.5)	130.28 (100.54 to 167.83)
Zhejiang	Foreign body	161 (119 to 207)	365.65 (269.09 to 468.71)	67.6 (49.6 to 81.5)	110.51 (81.12 to 133.22)
China	Other unintentional injuries	970 (899 to 1040)	81.02 (75.13 to 87.18)	1070 (981 to 1170)	75.57 (69.44 to 82.58)
Taiwan	Other unintentional injuries	80 (75.6 to 84.7)	392.01 (370.52 to 415.38)	9.82 (8.05 to 12.2)	41.63 (34.13 to 51.55)
Hong Kong Special Administrative Region of China	Other unintentional injuries	2.05 (1.69 to 2.54)	35.53 (29.38 to 44.05)	2.77 (2.17 to 3.49)	36.89 (28.89 to 46.42)
Macau Special Administrative Region of China	Other unintentional injuries	0.189 (0.161 to 0.225)	53.38 (45.27 to 63.45)	0.244 (0.185 to 0.31)	35.64 (27.04 to 45.29)
Anhui	Other unintentional injuries	49.9 (42.3 to 58.5)	84.14 (71.45 to 98.73)	50.9 (41.5 to 61.8)	83.2 (67.8 to 101.11)
Beijing	Other unintentional injuries	3.99 (3.14 to 5.12)	35.57 (27.96 to 45.69)	9.35 (7.01 to 12.4)	38.8 (29.09 to 51.52)
Chongqing	Other unintentional injuries	16.7 (13.9 to 20.1)	105.8 (88.04 to 127.5)	18.2 (14.7 to 22.4)	68.11 (54.93 to 83.68)
Fujian	Other unintentional injuries	21.6 (18.7 to 24.9)	68.12 (59.23 to 78.55)	30.1 (24.4 to 36.7)	76.31 (61.94 to 93.11)
Gansu	Other unintentional injuries	35 (30.1 to 40.5)	147.85 (127.13 to 171.21)	26.4 (21.4 to 31.8)	99.45 (80.68 to 119.63)
Guangdong	Other unintentional injuries	29.4 (24.9 to 34.4)	44.63 (37.8 to 52.19)	50.4 (41.8 to 60.7)	43.11 (35.77 to 51.96)
Guangxi	Other unintentional injuries	41.2 (35 to 48.6)	94.8 (80.53 to 111.68)	50 (40.5 to 60.6)	103.46 (83.79 to 125.43)
Guizhou	Other unintentional injuries	40.1 (33.6 to 48.3)	120.68 (101.09 to 145.41)	28.7 (22.9 to 36.4)	84.72 (67.57 to 107.52)
Hainan	Other unintentional injuries	17.6 (14.5 to 21.9)	253.24 (208.76 to 313.77)	9.24 (7.54 to 11.1)	96.35 (78.64 to 115.88)
Hebei	Other unintentional injuries	65.5 (57.8 to 74.1)	102.97 (90.8 to 116.49)	69.6 (57.3 to 83.4)	91.04 (74.93 to 109.1)
Heilongjiang	Other unintentional injuries	22 (18.6 to 25.9)	60.45 (50.92 to 71.17)	18.6 (15.3 to 22.5)	47.34 (38.87 to 57.28)
Henan	Other unintentional injuries	72.8 (63.9 to 83.1)	82.52 (72.35 to 94.18)	93.3 (75 to 113)	96.83 (77.89 to 116.94)
Hubei	Other unintentional injuries	51.8 (45.1 to 60.1)	93.25 (81.11 to 108.14)	61.1 (49 to 74.9)	110.41 (88.66 to 135.41)
Hunan	Other unintentional injuries	48.5 (42.7 to 55.6)	78.07 (68.71 to 89.41)	64.3 (52.2 to 77.6)	95.7 (77.79 to 115.52)
Inner Mongolia	Other unintentional injuries	15.8 (13.5 to 18.3)	69.28 (59.25 to 80.59)	15.8 (12.8 to 18.9)	61.06 (49.4 to 72.89)

Jiangsu	Other unintentional injuries	31.2<br>(26.5 to 35.9)	45.29<br>(38.54 to 52.21)	39.6<br>(32.9 to 48.1)	48.51<br>(40.33 to 58.93)
Jiangxi	Other unintentional injuries	47.4<br>(41.4 to 55.3)	119.99<br>(104.69 to 139.87)	38.9<br>(32.3 to 46.2)	81.83<br>(67.98 to 97.12)
Jilin	Other unintentional injuries	15.5<br>(13.1 to 18.7)	59.9<br>(50.39 to 72.17)	11<br>(8.79 to 13.6)	39.5<br>(31.51 to 48.6)
Liaoning	Other unintentional injuries	22.6<br>(18.7 to 27)	55.38<br>(45.87 to 66.1)	21.6<br>(17.8 to 25.9)	48.35<br>(39.89 to 58.05)
Ningxia	Other unintentional injuries	6<br>(5.01 to 7.23)	121.47<br>(101.49 to 146.49)	6.31<br>(5.09 to 7.67)	89.84<br>(72.5 to 109.32)
Qinghai	Other unintentional injuries	6.61<br>(5.53 to 7.88)	139.55<br>(116.71 to 166.35)	7.43<br>(6.09 to 9.32)	118.21<br>(96.93 to 148.37)
Shaanxi	Other unintentional injuries	27.4<br>(23.5 to 31.6)	79.59<br>(68.38 to 91.69)	43.3<br>(35.1 to 53.2)	109.7<br>(88.95 to 134.57)
Shandong	Other unintentional injuries	50.3<br>(42.8 to 59)	58.73<br>(50.03 to 68.93)	59.6<br>(48.6 to 71.8)	59.97<br>(48.88 to 72.26)
Shanghai	Other unintentional injuries	4.22<br>(3.53 to 5.02)	30.24<br>(25.27 to 35.92)	9.09<br>(7.36 to 11.3)	32.54<br>(26.34 to 40.29)
Shanxi	Other unintentional injuries	28.8<br>(24 to 35.5)	95.23<br>(79.3 to 117.27)	30.4<br>(24.2 to 37)	78.8<br>(62.71 to 95.79)
Sichuan	Other unintentional injuries	92.1<br>(79.2 to 107)	82.53<br>(70.94 to 95.52)	75.9<br>(61.6 to 92.9)	90.96<br>(73.74 to 111.3)
Tianjin	Other unintentional injuries	3.61<br>(3.03 to 4.3)	39.29<br>(32.92 to 46.8)	7.55<br>(6.19 to 9.1)	49.45<br>(40.57 to 59.65)
Tibet	Other unintentional injuries	4.19<br>(3.48 to 5.03)	179.61<br>(149.49 to 215.84)	5.05<br>(4.17 to 6.15)	146.1<br>(120.51 to 177.85)
Xinjiang	Other unintentional injuries	12.8<br>(10.6 to 14.9)	81.26<br>(67.47 to 94.28)	13.8<br>(11.2 to 16.6)	56.54<br>(46.09 to 67.97)
Yunnan	Other unintentional injuries	55.2<br>(46 to 66.4)	141.53<br>(118.01 to 170.26)	60.7<br>(49.5 to 73.9)	123.36<br>(100.61 to 150.27)
Zhejiang	Other unintentional injuries	27.7<br>(24.2 to 31.8)	62.74<br>(54.9 to 72.11)	38.4<br>(31.6 to 46.3)	62.79<br>(51.63 to 75.71)
China	Self-harm and interpersonal violence	14900<br>(12800 to 15900)	1248.12<br>(1069.98 to 1326.35)	6550<br>(6040 to 7260)	463.82<br>(427.56 to 513.72)
Taiwan	Self-harm and interpersonal violence	130<br>(124 to 136)	635.45<br>(605.72 to 667.54)	200<br>(188 to 212)	847.9<br>(797.58 to 900.35)
Hong Kong Special Administrative Region of China	Self-harm and interpersonal violence	37.7<br>(30.2 to 41.8)	654.91<br>(524.44 to 725.99)	34.7<br>(28.6 to 40.9)	461.76<br>(380.65 to 544.33)
Macau Special Administrative Region of China	Self-harm and interpersonal violence	2.64<br>(2.16 to 2.91)	743.69<br>(609.71 to 818.99)	3.04<br>(2.53 to 3.56)	444.07<br>(369.37 to 520.91)
Anhui	Self-harm and interpersonal violence	1090<br>(842 to 1250)	1847.11<br>(1420.42 to 2116.12)	336<br>(283 to 392)	549.96<br>(463.48 to 641.22)
Beijing	Self-harm and interpersonal violence	64.3<br>(55.7 to 76.3)	573.28<br>(496.49 to 680.69)	81.4<br>(68.1 to 95.3)	337.55<br>(282.65 to 395.59)
Chongqing	Self-harm and interpersonal violence	222<br>(189 to 256)	1404.29<br>(1195.67 to 1619.1)	103<br>(87 to 123)	384.61<br>(325.59 to 461.01)
Fujian	Self-harm and interpersonal violence	436<br>(382 to 484)	1376.8<br>(1206.76 to 1528.34)	169<br>(144 to 199)	428.19<br>(366.36 to 505.6)
Gansu	Self-harm and interpersonal violence	452<br>(341 to 514)	1910.21<br>(1443.49 to 2172.92)	139<br>(117 to 160)	521.38<br>(440.66 to 603.22)
Guangdong	Self-harm and interpersonal violence	507<br>(438 to 583)	770.39<br>(665.09 to 885.3)	399<br>(339 to 471)	341.1<br>(289.89 to 403.38)
Guangxi	Self-harm and interpersonal violence	461<br>(393 to 527)	1060.59<br>(903.75 to 1211.58)	191<br>(155 to 277)	396.23<br>(320.13 to 572.51)
Guizhou	Self-harm and interpersonal violence	541<br>(450 to 620)	1628.62<br>(1355.34 to 1868.48)	194<br>(164 to 232)	574.21<br>(485.8 to 685.27)
Hainan	Self-harm and interpersonal violence	75.4<br>(61.3 to 90.9)	1082.82<br>(880.7 to 1305.01)	45.6<br>(38.7 to 53.4)	475.39<br>(403.47 to 556.59)
Hebei	Self-harm and interpersonal violence	483<br>(428 to 550)	759.13<br>(672.89 to 864.33)	360<br>(301 to 438)	471.64<br>(394.28 to 572.53)
Heilongjiang	Self-harm and interpersonal violence	327<br>(285 to 374)	895.75<br>(782.44 to 1026.89)	169<br>(143 to 214)	431.59<br>(364.51 to 544.52)
Henan	Self-harm and interpersonal violence	976<br>(787 to 1100)	1105.47<br>(891.84 to 1245.9)	465<br>(398 to 535)	482.46<br>(413.08 to 555.9)
Hubei	Self-harm and interpersonal violence	991<br>(723 to 1120)	1783.53<br>(1301.14 to 2023.12)	430<br>(338 to 508)	777.73<br>(611.2 to 919.23)

Hunan	Self-harm and interpersonal violence	1010<br>(892 to 1120)	1620.19<br>(1434.54 to 1804.58)	354<br>(301 to 427)	527.81<br>(447.54 to 636.41)
Inner Mongolia	Self-harm and interpersonal violence	315<br>(242 to 368)	1385.12<br>(1063.99 to 1620.12)	116<br>(98.9 to 136)	447.92<br>(382.12 to 526.52)
Jiangsu	Self-harm and interpersonal violence	578<br>(518 to 643)	840.53<br>(752.31 to 934.81)	321<br>(273 to 375)	393.55<br>(334.92 to 458.85)
Jiangxi	Self-harm and interpersonal violence	652<br>(565 to 727)	1649.13<br>(1429.59 to 1839.97)	188<br>(159 to 232)	395.53<br>(335.21 to 487.05)
Jilin	Self-harm and interpersonal violence	225<br>(194 to 265)	866.48<br>(747.84 to 1019.79)	105<br>(86.1 to 133)	377.72<br>(308.62 to 477.95)
Liaoning	Self-harm and interpersonal violence	334<br>(288 to 386)	819.96<br>(705.59 to 946.68)	199<br>(170 to 237)	446.68<br>(381.12 to 531.4)
Ningxia	Self-harm and interpersonal violence	58.4<br>(47.3 to 68.5)	1183.32<br>(956.92 to 1386.31)	28.1<br>(23.9 to 33.4)	399.84<br>(340.69 to 475.91)
Qinghai	Self-harm and interpersonal violence	75.4<br>(62.7 to 86.6)	1591.81<br>(1324.21 to 1829.17)	34.7<br>(29.3 to 42.7)	551.82<br>(465.72 to 679.22)
Shaanxi	Self-harm and interpersonal violence	251<br>(205 to 282)	730.3<br>(596.66 to 819.57)	177<br>(151 to 210)	448.92<br>(382.18 to 531.33)
Shandong	Self-harm and interpersonal violence	1510<br>(1110 to 1760)	1760.33<br>(1300.71 to 2054.33)	492<br>(420 to 573)	494.61<br>(422.7 to 577.04)
Shanghai	Self-harm and interpersonal violence	77.4<br>(68.1 to 88.4)	554<br>(487.16 to 632.35)	99.4<br>(81.3 to 119)	355.72<br>(290.98 to 425.23)
Shanxi	Self-harm and interpersonal violence	268<br>(232 to 313)	885.85<br>(765.16 to 1034.39)	139<br>(116 to 178)	360.36<br>(300.45 to 459.96)
Sichuan	Self-harm and interpersonal violence	1260<br>(1080 to 1420)	1128.45<br>(970.55 to 1268.23)	414<br>(353 to 490)	495.35<br>(422.73 to 586.59)
Tianjin	Self-harm and interpersonal violence	69.6<br>(61.1 to 80.6)	756.56<br>(664.38 to 876.25)	54.3<br>(45.2 to 67.7)	355.55<br>(295.85 to 443.41)
Tibet	Self-harm and interpersonal violence	32.8<br>(27.7 to 39.1)	1409.41<br>(1188.68 to 1677.92)	15.8<br>(12.8 to 22.6)	456.61<br>(370.06 to 653.06)
Xinjiang	Self-harm and interpersonal violence	134<br>(115 to 165)	849.15<br>(725.93 to 1043.26)	110<br>(90.5 to 144)	449.93<br>(371.42 to 589.18)
Yunnan	Self-harm and interpersonal violence	922<br>(690 to 1060)	2365.04<br>(1771.27 to 2719.84)	361<br>(303 to 425)	733.76<br>(616.82 to 864.62)
Zhejiang	Self-harm and interpersonal violence	504<br>(435 to 566)	1142.11<br>(986.09 to 1283.99)	223<br>(190 to 261)	363.83<br>(311.06 to 426.26)
China	Self-harm	10800<br>(9060 to 11500)	906.43<br>(757.3 to 958.94)	4210<br>(3970 to 4690)	298.04<br>(281.24 to 331.99)
Taiwan	Self-harm	79.3<br>(76.4 to 81.9)	388.61<br>(374.56 to 401.62)	159<br>(150 to 169)	674.72<br>(634.63 to 717.39)
Hong Kong Special Administrative Region of China	Self-harm	27<br>(20.3 to 30.2)	468.48<br>(352.98 to 523.88)	23.9<br>(17.5 to 29.5)	318.01<br>(232.62 to 393.28)
Macau Special Administrative Region of China	Self-harm	1.84<br>(1.45 to 2.04)	518.94<br>(408.87 to 573.78)	1.86<br>(1.48 to 2.29)	271.29<br>(215.78 to 334.6)
Anhui	Self-harm	912<br>(663 to 1060)	1538.93<br>(1118.84 to 1787.66)	250<br>(197 to 299)	408.96<br>(321.45 to 489.77)
Beijing	Self-harm	37.7<br>(31.8 to 46.8)	336.56<br>(283.21 to 417.19)	32.7<br>(25.2 to 41.6)	135.66<br>(104.67 to 172.57)
Chongqing	Self-harm	161<br>(133 to 190)	1021.39<br>(839.73 to 1202.51)	65.5<br>(53 to 81.9)	245.21<br>(198.36 to 306.48)
Fujian	Self-harm	338<br>(295 to 380)	1068.95<br>(933.38 to 1201.94)	102<br>(82.9 to 125)	259.12<br>(210.35 to 317.06)
Gansu	Self-harm	324<br>(241 to 377)	1369.83<br>(1018.29 to 1593.88)	94.2<br>(77.5 to 112)	354.36<br>(291.66 to 421.75)
Guangdong	Self-harm	319<br>(266 to 376)	485.32<br>(404.02 to 570.9)	211<br>(172 to 264)	180.28<br>(147.63 to 226.31)
Guangxi	Self-harm	277<br>(230 to 348)	637.79<br>(528.4 to 800.74)	110<br>(82.9 to 189)	226.72<br>(171.46 to 390.32)
Guizhou	Self-harm	352<br>(294 to 415)	1061.79<br>(884.68 to 1250.47)	120<br>(97.1 to 154)	353.49<br>(286.94 to 455.56)
Hainan	Self-harm	49.9<br>(40.9 to 63)	716.85<br>(587.13 to 903.69)	27.4<br>(22 to 33.7)	285.53<br>(228.96 to 351.99)
Hebei	Self-harm	310<br>(267 to 354)	487.11<br>(419.62 to 556.1)	227<br>(182 to 290)	297.53<br>(238.54 to 378.93)
Heilongjiang	Self-harm	204<br>(173 to 244)	559.69<br>(474.28 to 670.65)	97.4<br>(79.1 to 133)	248.2<br>(201.57 to 338.28)

Henan	Self-harm	719 (552 to 825)	814.86 (625.65 to 934.86)	324 (273 to 388)	336.44 (283.39 to 402.43)
Hubei	Self-harm	815 (564 to 943)	1466.52 (1015.98 to 1697.5)	350 (255 to 423)	633.66 (461.92 to 765.57)
Hunan	Self-harm	806 (703 to 905)	1295.59 (1130.2 to 1455.27)	246 (203 to 302)	365.71 (302.56 to 449.46)
Inner Mongolia	Self-harm	209 (162 to 249)	918.51 (711.12 to 1096.21)	67.9 (56.3 to 84.6)	262.07 (217.38 to 326.54)
Jiangsu	Self-harm	416 (362 to 470)	604.35 (526.08 to 682.74)	189 (156 to 228)	231.7 (190.94 to 279.83)
Jiangxi	Self-harm	481 (413 to 549)	1217.87 (1044.66 to 1388.76)	114 (93.6 to 146)	240.62 (196.82 to 307.83)
Jilin	Self-harm	149 (124 to 180)	572.75 (477.34 to 692.49)	65.7 (51.1 to 86.1)	235.72 (183.08 to 308.57)
Liaoning	Self-harm	205 (170 to 249)	503.01 (417.91 to 609.61)	115 (92.1 to 144)	256.71 (206.34 to 321.93)
Ningxia	Self-harm	32.3 (26.7 to 38.6)	654.03 (540.98 to 782.17)	15.4 (12.4 to 20.1)	219.95 (176.26 to 286.8)
Qinghai	Self-harm	50.3 (41.2 to 59)	1061.08 (870.42 to 1245.7)	22.6 (18.2 to 28.5)	359.1 (289.51 to 454.17)
Shaanxi	Self-harm	146 (121 to 167)	423.43 (352.18 to 484.74)	112 (91.7 to 141)	283.66 (232.1 to 355.93)
Shandong	Self-harm	1280 (887 to 1500)	1490.5 (1036.22 to 1756.72)	350 (285 to 420)	351.89 (286.72 to 422.66)
Shanghai	Self-harm	43.3 (37 to 52.6)	309.93 (264.57 to 376.24)	47.7 (32.4 to 61.8)	170.88 (115.88 to 221.26)
Shanxi	Self-harm	177 (148 to 210)	582.88 (488.59 to 693.27)	79 (63.4 to 107)	204.66 (164.05 to 275.82)
Sichuan	Self-harm	852 (726 to 974)	763.67 (650.8 to 872.46)	274 (227 to 339)	328.63 (271.91 to 406.06)
Tianjin	Self-harm	44.3 (37.4 to 53.4)	481.66 (406.6 to 580.77)	31.3 (25.1 to 41)	204.8 (164.71 to 268.77)
Tibet	Self-harm	19.7 (16 to 25.7)	845.98 (687.77 to 1104.01)	8.42 (6.38 to 14.1)	243.53 (184.65 to 406.53)
Xinjiang	Self-harm	76.1 (63.1 to 97.3)	482.42 (400.36 to 616.68)	62.1 (48 to 91.4)	254.93 (196.94 to 374.92)
Yunnan	Self-harm	659 (505 to 774)	1692.27 (1295.91 to 1985.24)	256 (207 to 309)	520.23 (420.08 to 629.28)
Zhejiang	Self-harm	359 (314 to 417)	814.66 (712.73 to 944.86)	117 (95.4 to 146)	191.41 (155.99 to 238.08)
China	Interpersonal violence	3790 (3020 to 4310)	316.57 (252.41 to 360.18)	2260 (1850 to 2790)	159.94 (131.1 to 197.69)
Taiwan	Interpersonal violence	47.7 (42.8 to 53.8)	234 (209.76 to 263.5)	40.5 (34.2 to 47.9)	171.56 (145.03 to 203.1)
Hong Kong Special Administrative Region of China	Interpersonal violence	10.5 (8.64 to 12.6)	182.62 (149.93 to 217.94)	10.6 (8.35 to 13.4)	141.36 (111.22 to 178.48)
Macau Special Administrative Region of China	Interpersonal violence	0.739 (0.592 to 0.888)	208.24 (166.81 to 250.19)	1.08 (0.858 to 1.34)	157.4 (125.47 to 195.31)
Anhui	Interpersonal violence	169 (142 to 204)	285.92 (239.43 to 343.74)	83.4 (66 to 106)	136.43 (107.94 to 172.72)
Beijing	Interpersonal violence	24.3 (20 to 30.4)	216.5 (178.09 to 270.76)	46.7 (36.5 to 58.5)	193.88 (151.33 to 242.86)
Chongqing	Interpersonal violence	56.5 (44.1 to 66.9)	357.96 (279.13 to 423.94)	36.2 (28.7 to 45.5)	135.35 (107.33 to 170.16)
Fujian	Interpersonal violence	88.6 (72 to 103)	280.03 (227.67 to 327.02)	63.9 (51.7 to 78.8)	162.24 (131.24 to 200.04)
Gansu	Interpersonal violence	116 (84.1 to 136)	490.83 (355.63 to 576.84)	42.1 (33.9 to 52.1)	158.39 (127.71 to 196.15)
Guangdong	Interpersonal violence	180 (143 to 214)	273.38 (217.59 to 325.8)	185 (148 to 230)	158.2 (126.52 to 197)
Guangxi	Interpersonal violence	178 (118 to 214)	409.58 (271.07 to 491.42)	80.3 (65.5 to 97.5)	166.21 (135.46 to 201.85)
Guizhou	Interpersonal violence	173 (116 to 203)	520.59 (350.49 to 613.1)	70.9 (57.3 to 85.2)	209.64 (169.38 to 251.78)
Hainan	Interpersonal violence	24.5 (16.5 to 29.9)	351.83 (236.39 to 428.97)	17.7 (14.2 to 21.3)	184.77 (148.53 to 222.37)

Hebei	Interpersonal violence	159<br>(133 to 197)	249.59<br>(208.75 to 309.8)	128<br>(102 to 158)	167.52<br>(133.43 to 206.6)
Heilongjiang	Interpersonal violence	111<br>(93.1 to 131)	303.66<br>(255.32 to 358.86)	68.5<br>(55.8 to 83.6)	174.51<br>(142.26 to 212.91)
Henan	Interpersonal violence	238<br>(197 to 279)	269.12<br>(222.72 to 316.42)	136<br>(109 to 172)	141.31<br>(112.71 to 179.05)
Hubei	Interpersonal violence	161<br>(129 to 189)	289.62<br>(232.84 to 339.78)	76.1<br>(60.6 to 95.6)	137.64<br>(109.61 to 172.83)
Hunan	Interpersonal violence	188<br>(153 to 225)	302.8<br>(246.02 to 361.65)	105<br>(84.1 to 129)	155.91<br>(125.3 to 191.87)
Inner Mongolia	Interpersonal violence	99<br>(67.3 to 119)	435.28<br>(295.98 to 522.47)	46.5<br>(38.1 to 56.1)	179.5<br>(147.02 to 216.54)
Jiangsu	Interpersonal violence	152<br>(128 to 187)	220.47<br>(186.23 to 271.07)	129<br>(102 to 162)	158.52<br>(125.04 to 198.34)
Jiangxi	Interpersonal violence	158<br>(125 to 185)	399.57<br>(317.04 to 469.19)	70.9<br>(57 to 89.2)	149.19<br>(119.95 to 187.59)
Jilin	Interpersonal violence	70.8<br>(58.6 to 88.5)	272.83<br>(225.78 to 340.9)	38.1<br>(30 to 48.7)	136.56<br>(107.44 to 174.66)
Liaoning	Interpersonal violence	120<br>(97.7 to 142)	295.35<br>(239.63 to 348.87)	82.2<br>(67.1 to 99.4)	183.99<br>(150.3 to 222.56)
Ningxia	Interpersonal violence	23.7<br>(14.6 to 29.8)	479.31<br>(294.75 to 603.31)	11.9<br>(9.71 to 14.4)	169.63<br>(138.39 to 204.91)
Qinghai	Interpersonal violence	23.9<br>(17.8 to 28.4)	504.09<br>(376.39 to 600.45)	11.7<br>(9.67 to 14.6)	186.48<br>(153.9 to 231.81)
Shaanxi	Interpersonal violence	101<br>(73.2 to 120)	294.74<br>(212.79 to 349.98)	63.9<br>(52.7 to 78.6)	161.74<br>(133.42 to 198.99)
Shandong	Interpersonal violence	210<br>(175 to 256)	245.55<br>(204.53 to 299.23)	137<br>(108 to 174)	137.72<br>(108.43 to 175.25)
Shanghai	Interpersonal violence	30.7<br>(25.6 to 36.6)	220.06<br>(183.49 to 261.66)	50.3<br>(39.8 to 62.6)	180.14<br>(142.29 to 224.09)
Shanxi	Interpersonal violence	85.5<br>(70.9 to 102)	282.28<br>(234.01 to 338.34)	58.6<br>(46.5 to 74.2)	151.64<br>(120.5 to 192.1)
Sichuan	Interpersonal violence	377<br>(290 to 442)	337.66<br>(260.05 to 395.86)	134<br>(108 to 166)	160.67<br>(129.37 to 199.41)
Tianjin	Interpersonal violence	23.1<br>(19 to 27.5)	251.19<br>(206.59 to 298.88)	22.2<br>(17.5 to 28)	145.18<br>(114.66 to 183.74)
Tibet	Interpersonal violence	13.1<br>(10.1 to 15.6)	561.87<br>(433.78 to 671.57)	7.31<br>(5.99 to 9.67)	211.63<br>(173.16 to 279.65)
Xinjiang	Interpersonal violence	52.5<br>(43.4 to 65.5)	333<br>(275.23 to 415.26)	44.1<br>(35.7 to 54.7)	181.11<br>(146.48 to 224.54)
Yunnan	Interpersonal violence	235<br>(140 to 286)	603.46<br>(359.21 to 733.75)	96.9<br>(78.2 to 116)	197.13<br>(159.07 to 235.5)
Zhejiang	Interpersonal violence	135<br>(97.3 to 158)	307.19<br>(220.69 to 357.94)	103<br>(82.5 to 126)	167.96<br>(134.86 to 205.89)
China	Exposure to forces of nature	83.1<br>(78.8 to 87.6)	6.94<br>(6.58 to 7.32)	63.2<br>(54.3 to 74.2)	4.48<br>(3.85 to 5.26)
Taiwan	Exposure to forces of nature	0.664<br>(0.565 to 0.793)	3.26<br>(2.77 to 3.89)	1.37<br>(1.09 to 1.71)	5.8<br>(4.64 to 7.24)
Hong Kong Special Administrative Region of China	Exposure to forces of nature	0.202<br>(0.18 to 0.227)	3.5<br>(3.12 to 3.95)	0.266<br>(0.227 to 0.31)	3.54<br>(3.02 to 4.12)
Macau Special Administrative Region of China	Exposure to forces of nature	0.0168<br>(0.0152 to 0.0189)	4.74<br>(4.27 to 5.32)	0.422<br>(0.379 to 0.469)	61.73<br>(55.4 to 68.58)
Anhui	Exposure to forces of nature	1.61<br>(1.43 to 1.81)	2.72<br>(2.41 to 3.05)	1.51<br>(1.34 to 1.7)	2.46<br>(2.19 to 2.78)
Beijing	Exposure to forces of nature	0.609<br>(0.54 to 0.681)	5.43<br>(4.82 to 6.07)	0.826<br>(0.702 to 0.978)	3.43<br>(2.91 to 4.06)
Chongqing	Exposure to forces of nature	0.763<br>(0.674 to 0.864)	4.83<br>(4.27 to 5.48)	0.764<br>(0.67 to 0.882)	2.86<br>(2.51 to 3.3)
Fujian	Exposure to forces of nature	9.07<br>(8.03 to 10.2)	28.67<br>(25.39 to 32.39)	1.08<br>(0.947 to 1.24)	2.74<br>(2.4 to 3.15)
Gansu	Exposure to forces of nature	0.77<br>(0.686 to 0.865)	3.25<br>(2.9 to 3.66)	0.809<br>(0.706 to 0.927)	3.04<br>(2.66 to 3.49)
Guangdong	Exposure to forces of nature	3.33<br>(2.94 to 3.79)	5.06<br>(4.47 to 5.76)	4.24<br>(3.73 to 4.88)	3.63<br>(3.19 to 4.18)
Guangxi	Exposure to forces of nature	1.74<br>(1.47 to 2.04)	4<br>(3.39 to 4.69)	1.37<br>(1.21 to 1.57)	2.83<br>(2.5 to 3.25)

Guizhou	Exposure to forces of nature	0.914 (0.802 to 1.04)	2.75 (2.42 to 3.13)	0.896 (0.786 to 1.02)	2.65 (2.32 to 3.01)
Hainan	Exposure to forces of nature	0.258 (0.231 to 0.289)	3.7 (3.31 to 4.15)	0.255 (0.226 to 0.289)	2.66 (2.35 to 3.02)
Hebei	Exposure to forces of nature	3.77 (3.38 to 4.24)	5.92 (5.3 to 6.66)	1.91 (1.69 to 2.16)	2.5 (2.22 to 2.82)
Heilongjiang	Exposure to forces of nature	0.996 (0.883 to 1.12)	2.73 (2.42 to 3.07)	0.982 (0.87 to 1.1)	2.5 (2.22 to 2.82)
Henan	Exposure to forces of nature	2.47 (2.2 to 2.82)	2.8 (2.49 to 3.19)	2.28 (2.04 to 2.56)	2.37 (2.11 to 2.66)
Hubei	Exposure to forces of nature	1.41 (1.26 to 1.59)	2.55 (2.26 to 2.86)	1.36 (1.21 to 1.52)	2.46 (2.18 to 2.76)
Hunan	Exposure to forces of nature	22.1 (19.5 to 24.9)	35.58 (31.35 to 40.08)	2.53 (2.27 to 2.84)	3.76 (3.38 to 4.24)
Inner Mongolia	Exposure to forces of nature	0.659 (0.589 to 0.742)	2.9 (2.59 to 3.26)	0.653 (0.583 to 0.743)	2.52 (2.25 to 2.87)
Jiangsu	Exposure to forces of nature	2.05 (1.85 to 2.28)	2.98 (2.68 to 3.32)	2.08 (1.84 to 2.35)	2.55 (2.25 to 2.87)
Jiangxi	Exposure to forces of nature	1.24 (1.1 to 1.4)	3.14 (2.78 to 3.55)	1.25 (1.11 to 1.43)	2.64 (2.34 to 3.01)
Jilin	Exposure to forces of nature	0.705 (0.63 to 0.795)	2.72 (2.43 to 3.06)	0.691 (0.61 to 0.78)	2.48 (2.19 to 2.8)
Liaoning	Exposure to forces of nature	1.11 (0.991 to 1.24)	2.73 (2.43 to 3.03)	1.12 (0.994 to 1.26)	2.5 (2.23 to 2.82)
Ningxia	Exposure to forces of nature	0.181 (0.161 to 0.203)	3.66 (3.27 to 4.11)	0.181 (0.16 to 0.205)	2.58 (2.28 to 2.92)
Qinghai	Exposure to forces of nature	7.2 (6.38 to 8.16)	152.09 (134.65 to 172.19)	1.08 (0.848 to 1.37)	17.19 (13.5 to 21.82)
Shaanxi	Exposure to forces of nature	1.44 (1.23 to 1.73)	4.18 (3.59 to 5.03)	1.22 (1.06 to 1.42)	3.08 (2.68 to 3.6)
Shandong	Exposure to forces of nature	5.86 (5.21 to 6.53)	6.85 (6.09 to 7.63)	2.56 (2.26 to 2.9)	2.57 (2.27 to 2.92)
Shanghai	Exposure to forces of nature	0.685 (0.61 to 0.765)	4.9 (4.37 to 5.48)	0.945 (0.809 to 1.11)	3.38 (2.89 to 3.97)
Shanxi	Exposure to forces of nature	1.06 (0.946 to 1.2)	3.52 (3.12 to 3.96)	1.05 (0.921 to 1.19)	2.71 (2.39 to 3.09)
Sichuan	Exposure to forces of nature	2.59 (2.28 to 3.01)	2.32 (2.04 to 2.7)	23.9 (18.9 to 30.2)	28.58 (22.65 to 36.11)
Tianjin	Exposure to forces of nature	0.377 (0.337 to 0.418)	4.1 (3.66 to 4.55)	0.453 (0.396 to 0.524)	2.97 (2.6 to 3.43)
Tibet	Exposure to forces of nature	0.0973 (0.0857 to 0.112)	4.18 (3.68 to 4.83)	0.13 (0.109 to 0.158)	3.76 (3.16 to 4.58)
Xinjiang	Exposure to forces of nature	0.683 (0.602 to 0.778)	4.33 (3.81 to 4.94)	0.825 (0.709 to 0.975)	3.38 (2.91 to 4)
Yunnan	Exposure to forces of nature	5.44 (4.83 to 6.06)	13.97 (12.4 to 15.54)	1.92 (1.63 to 2.27)	3.9 (3.32 to 4.62)
Zhejiang	Exposure to forces of nature	1.6 (1.43 to 1.8)	3.62 (3.24 to 4.09)	1.71 (1.5 to 1.96)	2.8 (2.46 to 3.2)
China	Environmental heat and cold exposure	776 (492 to 939)	64.81 (41.09 to 78.43)	378 (281 to 483)	26.76 (19.91 to 34.22)
Taiwan	Environmental heat and cold exposure	3.61 (2.75 to 4.62)	17.67 (13.49 to 22.62)	5.51 (4.21 to 7.12)	23.38 (17.85 to 30.2)
Hong Kong Special Administrative Region of China	Environmental heat and cold exposure	1.43 (1.07 to 1.83)	24.74 (18.53 to 31.82)	2.05 (1.53 to 2.64)	27.33 (20.34 to 35.17)
Macau Special Administrative Region of China	Environmental heat and cold exposure	0.122 (0.0785 to 0.155)	34.36 (22.13 to 43.57)	0.18 (0.124 to 0.231)	26.27 (18.18 to 33.78)
Anhui	Environmental heat and cold exposure	60.3 (30.4 to 78.6)	101.72 (51.25 to 132.62)	18.4 (12.6 to 23.2)	30.06 (20.69 to 38.02)
Beijing	Environmental heat and cold exposure	3.13 (2.4 to 4.18)	27.94 (21.38 to 37.22)	4.99 (3.59 to 6.58)	20.7 (14.88 to 27.28)
Chongqing	Environmental heat and cold exposure	10 (6.61 to 12.9)	63.64 (41.89 to 81.44)	6.69 (5.05 to 8.62)	25.02 (18.89 to 32.24)
Fujian	Environmental heat and cold exposure	17.6 (11.5 to 22.5)	55.57 (36.44 to 71.11)	9.87 (7.37 to 12.6)	25.03 (18.69 to 32.01)
Gansu	Environmental heat and cold exposure	15.5 (12 to 22.4)	65.54 (50.83 to 94.8)	5.76 (4.14 to 8.2)	21.68 (15.58 to 30.84)

Guangdong	Environmental heat and cold exposure	24.9 (19.7 to 31.5)	37.8 (29.99 to 47.79)	25 (18.3 to 32.7)	21.39 (15.64 to 27.96)
Guangxi	Environmental heat and cold exposure	30 (18.8 to 38.1)	68.89 (43.32 to 87.6)	13 (9.7 to 16.4)	26.91 (20.07 to 34)
Guizhou	Environmental heat and cold exposure	26.1 (20.3 to 33.3)	78.72 (61.19 to 100.46)	9.54 (7.07 to 12.9)	28.19 (20.91 to 38.28)
Hainan	Environmental heat and cold exposure	2.78 (2.19 to 3.51)	39.85 (31.4 to 50.43)	2.26 (1.66 to 2.94)	23.52 (17.3 to 30.63)
Hebei	Environmental heat and cold exposure	28.5 (20.7 to 35)	44.8 (32.58 to 55.06)	18 (13.3 to 23.5)	23.53 (17.34 to 30.7)
Heilongjiang	Environmental heat and cold exposure	14.1 (11 to 19.4)	38.73 (30.29 to 53.1)	8.54 (6.37 to 11.2)	21.75 (16.23 to 28.62)
Henan	Environmental heat and cold exposure	58.3 (36.4 to 72.3)	66.07 (41.24 to 81.89)	24.3 (18.2 to 31.1)	25.28 (18.91 to 32.32)
Hubei	Environmental heat and cold exposure	43 (23.3 to 56.6)	77.46 (41.94 to 101.86)	16.9 (11.7 to 21.5)	30.6 (21.12 to 38.95)
Hunan	Environmental heat and cold exposure	39.8 (26.1 to 48.8)	64.04 (41.99 to 78.42)	17.9 (13.3 to 22.7)	26.62 (19.75 to 33.79)
Inner Mongolia	Environmental heat and cold exposure	18.2 (9.48 to 25.1)	80.24 (41.7 to 110.43)	6.6 (4.78 to 8.31)	25.51 (18.45 to 32.08)
Jiangsu	Environmental heat and cold exposure	56.2 (25.7 to 75.8)	81.63 (37.4 to 110.22)	28 (17 to 36.6)	34.33 (20.79 to 44.88)
Jiangxi	Environmental heat and cold exposure	47.6 (18.8 to 68.2)	120.48 (47.53 to 172.46)	14.4 (9.72 to 19)	30.39 (20.45 to 39.88)
Jilin	Environmental heat and cold exposure	8.73 (6.31 to 13.3)	33.63 (24.33 to 51.13)	6.35 (4.33 to 8.8)	22.75 (15.51 to 31.54)
Liaoning	Environmental heat and cold exposure	14.1 (10.6 to 19.6)	34.46 (25.91 to 48.1)	12 (8.48 to 16.4)	26.8 (18.98 to 36.64)
Ningxia	Environmental heat and cold exposure	3.88 (2.09 to 5.3)	78.5 (42.23 to 107.28)	1.77 (1.29 to 2.35)	25.27 (18.44 to 33.47)
Qinghai	Environmental heat and cold exposure	4.81 (2.55 to 6.75)	101.47 (53.87 to 142.43)	1.66 (1.25 to 2.1)	26.35 (19.83 to 33.42)
Shaanxi	Environmental heat and cold exposure	16.4 (12.4 to 21.8)	47.78 (36.17 to 63.36)	9.95 (7.11 to 13.5)	25.19 (17.99 to 34.26)
Shandong	Environmental heat and cold exposure	43.8 (28.1 to 55.6)	51.14 (32.83 to 64.95)	27.2 (19.7 to 36)	27.33 (19.82 to 36.21)
Shanghai	Environmental heat and cold exposure	5.15 (3.42 to 6.65)	36.88 (24.47 to 47.59)	7.29 (5.13 to 10.1)	26.09 (18.37 to 36.15)
Shanxi	Environmental heat and cold exposure	13.7 (9.81 to 17.3)	45.2 (32.39 to 57.1)	8.91 (6.32 to 12.2)	23.07 (16.36 to 31.61)
Sichuan	Environmental heat and cold exposure	80.6 (47.9 to 106)	72.17 (42.94 to 94.66)	24.8 (18 to 32.4)	29.67 (21.6 to 38.81)
Tianjin	Environmental heat and cold exposure	3.16 (2.24 to 3.98)	34.32 (24.4 to 43.29)	3.83 (2.7 to 5.18)	25.08 (17.7 to 33.93)
Tibet	Environmental heat and cold exposure	1.81 (1.29 to 2.7)	77.61 (55.46 to 115.93)	0.865 (0.578 to 1.58)	25.03 (16.72 to 45.68)
Xinjiang	Environmental heat and cold exposure	17.8 (8.4 to 25.2)	112.81 (53.23 to 159.5)	8.5 (5.28 to 11.8)	34.9 (21.68 to 48.25)
Yunnan	Environmental heat and cold exposure	23.8 (18.3 to 31.7)	61.05 (46.98 to 81.42)	12.4 (8.63 to 17.3)	25.2 (17.55 to 35.27)
Zhejiang	Environmental heat and cold exposure	40.5 (15.2 to 56.7)	91.84 (34.49 to 128.59)	20.2 (12.4 to 27)	32.96 (20.29 to 44.12)
China	Ebola	0 (0 to 0)	0 (0 to 0)	0 (0 to 0)	0 (0 to 0)
Taiwan	Ebola	0 (0 to 0)	0 (0 to 0)	0 (0 to 0)	0 (0 to 0)
Hong Kong Special Administrative Region of China	Ebola	0 (0 to 0)	0 (0 to 0)	0 (0 to 0)	0 (0 to 0)
Macau Special Administrative Region of China	Ebola	0 (0 to 0)	0 (0 to 0)	0 (0 to 0)	0 (0 to 0)
Anhui	Ebola	0 (0 to 0)	0 (0 to 0)	0 (0 to 0)	0 (0 to 0)
Beijing	Ebola	0 (0 to 0)	0 (0 to 0)	0 (0 to 0)	0 (0 to 0)
Chongqing	Ebola	0 (0 to 0)	0 (0 to 0)	0 (0 to 0)	0 (0 to 0)

Fujian	Ebola	0<br>(0 to 0)	0<br>(0 to 0)	0<br>(0 to 0)	0<br>(0 to 0)
Gansu	Ebola	0<br>(0 to 0)	0<br>(0 to 0)	0<br>(0 to 0)	0<br>(0 to 0)
Guangdong	Ebola	0<br>(0 to 0)	0<br>(0 to 0)	0<br>(0 to 0)	0<br>(0 to 0)
Guangxi	Ebola	0<br>(0 to 0)	0<br>(0 to 0)	0<br>(0 to 0)	0<br>(0 to 0)
Guizhou	Ebola	0<br>(0 to 0)	0<br>(0 to 0)	0<br>(0 to 0)	0<br>(0 to 0)
Hainan	Ebola	0<br>(0 to 0)	0<br>(0 to 0)	0<br>(0 to 0)	0<br>(0 to 0)
Hebei	Ebola	0<br>(0 to 0)	0<br>(0 to 0)	0<br>(0 to 0)	0<br>(0 to 0)
Heilongjiang	Ebola	0<br>(0 to 0)	0<br>(0 to 0)	0<br>(0 to 0)	0<br>(0 to 0)
Henan	Ebola	0<br>(0 to 0)	0<br>(0 to 0)	0<br>(0 to 0)	0<br>(0 to 0)
Hubei	Ebola	0<br>(0 to 0)	0<br>(0 to 0)	0<br>(0 to 0)	0<br>(0 to 0)
Hunan	Ebola	0<br>(0 to 0)	0<br>(0 to 0)	0<br>(0 to 0)	0<br>(0 to 0)
Inner Mongolia	Ebola	0<br>(0 to 0)	0<br>(0 to 0)	0<br>(0 to 0)	0<br>(0 to 0)
Jiangsu	Ebola	0<br>(0 to 0)	0<br>(0 to 0)	0<br>(0 to 0)	0<br>(0 to 0)
Jiangxi	Ebola	0<br>(0 to 0)	0<br>(0 to 0)	0<br>(0 to 0)	0<br>(0 to 0)
Jilin	Ebola	0<br>(0 to 0)	0<br>(0 to 0)	0<br>(0 to 0)	0<br>(0 to 0)
Liaoning	Ebola	0<br>(0 to 0)	0<br>(0 to 0)	0<br>(0 to 0)	0<br>(0 to 0)
Ningxia	Ebola	0<br>(0 to 0)	0<br>(0 to 0)	0<br>(0 to 0)	0<br>(0 to 0)
Qinghai	Ebola	0<br>(0 to 0)	0<br>(0 to 0)	0<br>(0 to 0)	0<br>(0 to 0)
Shaanxi	Ebola	0<br>(0 to 0)	0<br>(0 to 0)	0<br>(0 to 0)	0<br>(0 to 0)
Shandong	Ebola	0<br>(0 to 0)	0<br>(0 to 0)	0<br>(0 to 0)	0<br>(0 to 0)
Shanghai	Ebola	0<br>(0 to 0)	0<br>(0 to 0)	0<br>(0 to 0)	0<br>(0 to 0)
Shanxi	Ebola	0<br>(0 to 0)	0<br>(0 to 0)	0<br>(0 to 0)	0<br>(0 to 0)
Sichuan	Ebola	0<br>(0 to 0)	0<br>(0 to 0)	0<br>(0 to 0)	0<br>(0 to 0)
Tianjin	Ebola	0<br>(0 to 0)	0<br>(0 to 0)	0<br>(0 to 0)	0<br>(0 to 0)
Tibet	Ebola	0<br>(0 to 0)	0<br>(0 to 0)	0<br>(0 to 0)	0<br>(0 to 0)
Xinjiang	Ebola	0<br>(0 to 0)	0<br>(0 to 0)	0<br>(0 to 0)	0<br>(0 to 0)
Yunnan	Ebola	0<br>(0 to 0)	0<br>(0 to 0)	0<br>(0 to 0)	0<br>(0 to 0)
Zhejiang	Ebola	0<br>(0 to 0)	0<br>(0 to 0)	0<br>(0 to 0)	0<br>(0 to 0)
China	Executions and police conflict	297<br>(102 to 344)	24.8<br>(8.55 to 28.76)	79.7<br>(65.2 to 101)	5.65<br>(4.62 to 7.17)
Taiwan	Executions and police conflict	2.62<br>(2.08 to 3.28)	12.84<br>(10.21 to 16.07)	0.368<br>(0.297 to 0.466)	1.56<br>(1.26 to 1.98)
Hong Kong Special Administrative Region of China	Executions and police conflict	0.212<br>(0.141 to 0.354)	3.67<br>(2.45 to 6.14)	0.171<br>(0.122 to 0.274)	2.28<br>(1.63 to 3.65)
Macau Special Administrative Region of China	Executions and police conflict	0.0577<br>(0.0303 to 0.0945)	16.27<br>(8.55 to 26.64)	0.104<br>(0.0473 to 0.165)	15.14<br>(6.92 to 24.13)
Anhui	Executions and police conflict	13.1<br>(5.01 to 20.6)	22.1<br>(8.45 to 34.75)	2.74<br>(2.02 to 3.81)	4.49<br>(3.3 to 6.22)

Beijing	Executions and police conflict	2.24 (1.69 to 3.02)	19.93 (15.05 to 26.96)	1.88 (1.37 to 2.76)	7.78 (5.68 to 11.45)
Chongqing	Executions and police conflict	3.88 (1.77 to 6.2)	24.57 (11.2 to 39.29)	1.04 (0.741 to 1.51)	3.88 (2.77 to 5.65)
Fujian	Executions and police conflict	8.74 (1.98 to 14)	27.63 (6.27 to 44.18)	2.66 (1.86 to 3.63)	6.74 (4.72 to 9.22)
Gansu	Executions and police conflict	11.7 (2.86 to 18.3)	49.37 (12.09 to 77.44)	2.27 (1.52 to 3.12)	8.55 (5.72 to 11.73)
Guangdong	Executions and police conflict	7.52 (4.46 to 12)	11.42 (6.77 to 18.23)	2.91 (2.16 to 4.79)	2.49 (1.85 to 4.1)
Guangxi	Executions and police conflict	5.67 (3.31 to 9.31)	13.03 (7.61 to 21.42)	1.56 (1.12 to 2.75)	3.22 (2.31 to 5.69)
Guizhou	Executions and police conflict	15.3 (3.16 to 24.6)	46.06 (9.53 to 74.02)	3.72 (2.39 to 5.23)	10.98 (7.06 to 15.46)
Hainan	Executions and police conflict	0.97 (0.499 to 1.63)	13.93 (7.16 to 23.46)	0.478 (0.338 to 0.67)	4.99 (3.53 to 6.98)
Hebei	Executions and police conflict	14.2 (5.27 to 21.3)	22.26 (8.29 to 33.51)	4.96 (3.6 to 6.91)	6.49 (4.71 to 9.04)
Heilongjiang	Executions and police conflict	11.8 (3.65 to 18.4)	32.25 (10.01 to 50.43)	3.46 (2.39 to 4.78)	8.8 (6.08 to 12.18)
Henan	Executions and police conflict	18.8 (6.31 to 29.8)	21.32 (7.15 to 33.81)	4.46 (3.35 to 6.08)	4.64 (3.47 to 6.32)
Hubei	Executions and police conflict	15.1 (4.86 to 23.2)	27.23 (8.75 to 41.69)	3.49 (2.57 to 4.76)	6.32 (4.64 to 8.62)
Hunan	Executions and police conflict	13.4 (5.35 to 20.4)	21.62 (8.61 to 32.83)	4.11 (2.95 to 5.73)	6.12 (4.39 to 8.53)
Inner Mongolia	Executions and police conflict	7.08 (2.28 to 11.7)	31.15 (10.04 to 51.25)	1.62 (1.16 to 2.25)	6.26 (4.48 to 8.7)
Jiangsu	Executions and police conflict	10.7 (4.97 to 15.6)	15.54 (7.22 to 22.73)	2.64 (1.97 to 3.73)	3.24 (2.42 to 4.57)
Jiangxi	Executions and police conflict	12.4 (3.78 to 19.1)	31.49 (9.57 to 48.25)	2.68 (1.93 to 3.7)	5.63 (4.07 to 7.78)
Jilin	Executions and police conflict	5.38 (2.62 to 9.07)	20.73 (10.11 to 34.95)	1.49 (1.04 to 2.1)	5.34 (3.72 to 7.54)
Liaoning	Executions and police conflict	8.74 (4.55 to 14.3)	21.44 (11.14 to 34.99)	2.63 (1.93 to 3.63)	5.88 (4.31 to 8.13)
Ningxia	Executions and police conflict	2.46 (0.341 to 4.09)	49.74 (6.91 to 82.77)	0.711 (0.354 to 1)	10.13 (5.05 to 14.29)
Qinghai	Executions and police conflict	1.25 (0.514 to 1.96)	26.4 (10.84 to 41.41)	0.385 (0.281 to 0.543)	6.12 (4.48 to 8.64)
Shaanxi	Executions and police conflict	4.11 (2.33 to 6.31)	11.94 (6.76 to 18.33)	1.35 (0.998 to 1.9)	3.41 (2.53 to 4.82)
Shandong	Executions and police conflict	20.6 (7.65 to 32.4)	24.11 (8.94 to 37.88)	4.87 (3.51 to 6.58)	4.9 (3.53 to 6.62)
Shanghai	Executions and police conflict	3.31 (1.54 to 5.32)	23.68 (11.01 to 38.05)	1.24 (0.853 to 1.78)	4.43 (3.05 to 6.37)
Shanxi	Executions and police conflict	6.21 (2.76 to 9.89)	20.5 (9.13 to 32.67)	1.53 (1.11 to 2.15)	3.96 (2.87 to 5.56)
Sichuan	Executions and police conflict	30 (9.36 to 48.2)	26.89 (8.38 to 43.14)	4.99 (3.64 to 6.93)	5.97 (4.36 to 8.3)
Tianjin	Executions and police conflict	2.16 (0.941 to 3.43)	23.48 (10.24 to 37.35)	0.828 (0.583 to 1.18)	5.43 (3.82 to 7.74)
Tibet	Executions and police conflict	0.0302 (0.0189 to 0.105)	1.29 (0.81 to 4.5)	0.0344 (0.024 to 0.135)	1 (0.69 to 3.91)
Xinjiang	Executions and police conflict	3.93 (1.86 to 6.14)	24.94 (11.82 to 38.9)	2.09 (1.44 to 2.87)	8.58 (5.92 to 11.79)
Yunnan	Executions and police conflict	26.9 (4.09 to 41.6)	69.08 (10.5 to 106.78)	7.99 (4.27 to 11.1)	16.26 (8.68 to 22.58)
Zhejiang	Executions and police conflict	8.85 (2.4 to 14.3)	20.06 (5.45 to 32.38)	2.65 (1.9 to 3.61)	4.33 (3.1 to 5.89)
China	Zika virus	0 (0 to 0)	0 (0 to 0)	0 (0 to 0)	0 (0 to 0)
Taiwan	Zika virus	0 (0 to 0)	0 (0 to 0)	0 (0 to 0)	0 (0 to 0)
Hong Kong Special Administrative Region of China	Zika virus	0 (0 to 0)	0 (0 to 0)	0 (0 to 0)	0 (0 to 0)

Macau Special Administrative Region of China	Zika virus	0 (0 to 0)	0 (0 to 0)	0 (0 to 0)	0 (0 to 0)
Anhui	Zika virus	0 (0 to 0)	0 (0 to 0)	0 (0 to 0)	0 (0 to 0)
Beijing	Zika virus	0 (0 to 0)	0 (0 to 0)	0 (0 to 0)	0 (0 to 0)
Chongqing	Zika virus	0 (0 to 0)	0 (0 to 0)	0 (0 to 0)	0 (0 to 0)
Fujian	Zika virus	0 (0 to 0)	0 (0 to 0)	0 (0 to 0)	0 (0 to 0)
Gansu	Zika virus	0 (0 to 0)	0 (0 to 0)	0 (0 to 0)	0 (0 to 0)
Guangdong	Zika virus	0 (0 to 0)	0 (0 to 0)	0 (0 to 0)	0 (0 to 0)
Guangxi	Zika virus	0 (0 to 0)	0 (0 to 0)	0 (0 to 0)	0 (0 to 0)
Guizhou	Zika virus	0 (0 to 0)	0 (0 to 0)	0 (0 to 0)	0 (0 to 0)
Hainan	Zika virus	0 (0 to 0)	0 (0 to 0)	0 (0 to 0)	0 (0 to 0)
Hebei	Zika virus	0 (0 to 0)	0 (0 to 0)	0 (0 to 0)	0 (0 to 0)
Heilongjiang	Zika virus	0 (0 to 0)	0 (0 to 0)	0 (0 to 0)	0 (0 to 0)
Henan	Zika virus	0 (0 to 0)	0 (0 to 0)	0 (0 to 0)	0 (0 to 0)
Hubei	Zika virus	0 (0 to 0)	0 (0 to 0)	0 (0 to 0)	0 (0 to 0)
Hunan	Zika virus	0 (0 to 0)	0 (0 to 0)	0 (0 to 0)	0 (0 to 0)
Inner Mongolia	Zika virus	0 (0 to 0)	0 (0 to 0)	0 (0 to 0)	0 (0 to 0)
Jiangsu	Zika virus	0 (0 to 0)	0 (0 to 0)	0 (0 to 0)	0 (0 to 0)
Jiangxi	Zika virus	0 (0 to 0)	0 (0 to 0)	0 (0 to 0)	0 (0 to 0)
Jilin	Zika virus	0 (0 to 0)	0 (0 to 0)	0 (0 to 0)	0 (0 to 0)
Liaoning	Zika virus	0 (0 to 0)	0 (0 to 0)	0 (0 to 0)	0 (0 to 0)
Ningxia	Zika virus	0 (0 to 0)	0 (0 to 0)	0 (0 to 0)	0 (0 to 0)
Qinghai	Zika virus	0 (0 to 0)	0 (0 to 0)	0 (0 to 0)	0 (0 to 0)
Shaanxi	Zika virus	0 (0 to 0)	0 (0 to 0)	0 (0 to 0)	0 (0 to 0)
Shandong	Zika virus	0 (0 to 0)	0 (0 to 0)	0 (0 to 0)	0 (0 to 0)
Shanghai	Zika virus	0 (0 to 0)	0 (0 to 0)	0 (0 to 0)	0 (0 to 0)
Shanxi	Zika virus	0 (0 to 0)	0 (0 to 0)	0 (0 to 0)	0 (0 to 0)
Sichuan	Zika virus	0 (0 to 0)	0 (0 to 0)	0 (0 to 0)	0 (0 to 0)
Tianjin	Zika virus	0 (0 to 0)	0 (0 to 0)	0 (0 to 0)	0 (0 to 0)
Tibet	Zika virus	0 (0 to 0)	0 (0 to 0)	0 (0 to 0)	0 (0 to 0)
Xinjiang	Zika virus	0 (0 to 0)	0 (0 to 0)	0 (0 to 0)	0 (0 to 0)
Yunnan	Zika virus	0 (0 to 0)	0 (0 to 0)	0 (0 to 0)	0 (0 to 0)
Zhejiang	Zika virus	0 (0 to 0)	0 (0 to 0)	0 (0 to 0)	0 (0 to 0)
China	Guinea worm disease	0 (0 to 0)	0 (0 to 0)	0 (0 to 0)	0 (0 to 0)
Taiwan	Guinea worm disease	0 (0 to 0)	0 (0 to 0)	0 (0 to 0)	0 (0 to 0)

Hong Kong Special Administrative Region of China	Guinea worm disease	0<br>(0 to 0)	0<br>(0 to 0)	0<br>(0 to 0)	0<br>(0 to 0)
Macau Special Administrative Region of China	Guinea worm disease	0<br>(0 to 0)	0<br>(0 to 0)	0<br>(0 to 0)	0<br>(0 to 0)
Anhui	Guinea worm disease	0<br>(0 to 0)	0<br>(0 to 0)	0<br>(0 to 0)	0<br>(0 to 0)
Beijing	Guinea worm disease	0<br>(0 to 0)	0<br>(0 to 0)	0<br>(0 to 0)	0<br>(0 to 0)
Chongqing	Guinea worm disease	0<br>(0 to 0)	0<br>(0 to 0)	0<br>(0 to 0)	0<br>(0 to 0)
Fujian	Guinea worm disease	0<br>(0 to 0)	0<br>(0 to 0)	0<br>(0 to 0)	0<br>(0 to 0)
Gansu	Guinea worm disease	0<br>(0 to 0)	0<br>(0 to 0)	0<br>(0 to 0)	0<br>(0 to 0)
Guangdong	Guinea worm disease	0<br>(0 to 0)	0<br>(0 to 0)	0<br>(0 to 0)	0<br>(0 to 0)
Guangxi	Guinea worm disease	0<br>(0 to 0)	0<br>(0 to 0)	0<br>(0 to 0)	0<br>(0 to 0)
Guizhou	Guinea worm disease	0<br>(0 to 0)	0<br>(0 to 0)	0<br>(0 to 0)	0<br>(0 to 0)
Hainan	Guinea worm disease	0<br>(0 to 0)	0<br>(0 to 0)	0<br>(0 to 0)	0<br>(0 to 0)
Hebei	Guinea worm disease	0<br>(0 to 0)	0<br>(0 to 0)	0<br>(0 to 0)	0<br>(0 to 0)
Heilongjiang	Guinea worm disease	0<br>(0 to 0)	0<br>(0 to 0)	0<br>(0 to 0)	0<br>(0 to 0)
Henan	Guinea worm disease	0<br>(0 to 0)	0<br>(0 to 0)	0<br>(0 to 0)	0<br>(0 to 0)
Hubei	Guinea worm disease	0<br>(0 to 0)	0<br>(0 to 0)	0<br>(0 to 0)	0<br>(0 to 0)
Hunan	Guinea worm disease	0<br>(0 to 0)	0<br>(0 to 0)	0<br>(0 to 0)	0<br>(0 to 0)
Inner Mongolia	Guinea worm disease	0<br>(0 to 0)	0<br>(0 to 0)	0<br>(0 to 0)	0<br>(0 to 0)
Jiangsu	Guinea worm disease	0<br>(0 to 0)	0<br>(0 to 0)	0<br>(0 to 0)	0<br>(0 to 0)
Jiangxi	Guinea worm disease	0<br>(0 to 0)	0<br>(0 to 0)	0<br>(0 to 0)	0<br>(0 to 0)
Jilin	Guinea worm disease	0<br>(0 to 0)	0<br>(0 to 0)	0<br>(0 to 0)	0<br>(0 to 0)
Liaoning	Guinea worm disease	0<br>(0 to 0)	0<br>(0 to 0)	0<br>(0 to 0)	0<br>(0 to 0)
Ningxia	Guinea worm disease	0<br>(0 to 0)	0<br>(0 to 0)	0<br>(0 to 0)	0<br>(0 to 0)
Qinghai	Guinea worm disease	0<br>(0 to 0)	0<br>(0 to 0)	0<br>(0 to 0)	0<br>(0 to 0)
Shaanxi	Guinea worm disease	0<br>(0 to 0)	0<br>(0 to 0)	0<br>(0 to 0)	0<br>(0 to 0)
Shandong	Guinea worm disease	0<br>(0 to 0)	0<br>(0 to 0)	0<br>(0 to 0)	0<br>(0 to 0)
Shanghai	Guinea worm disease	0<br>(0 to 0)	0<br>(0 to 0)	0<br>(0 to 0)	0<br>(0 to 0)
Shanxi	Guinea worm disease	0<br>(0 to 0)	0<br>(0 to 0)	0<br>(0 to 0)	0<br>(0 to 0)
Sichuan	Guinea worm disease	0<br>(0 to 0)	0<br>(0 to 0)	0<br>(0 to 0)	0<br>(0 to 0)
Tianjin	Guinea worm disease	0<br>(0 to 0)	0<br>(0 to 0)	0<br>(0 to 0)	0<br>(0 to 0)
Tibet	Guinea worm disease	0<br>(0 to 0)	0<br>(0 to 0)	0<br>(0 to 0)	0<br>(0 to 0)
Xinjiang	Guinea worm disease	0<br>(0 to 0)	0<br>(0 to 0)	0<br>(0 to 0)	0<br>(0 to 0)
Yunnan	Guinea worm disease	0<br>(0 to 0)	0<br>(0 to 0)	0<br>(0 to 0)	0<br>(0 to 0)
Zhejiang	Guinea worm disease	0<br>(0 to 0)	0<br>(0 to 0)	0<br>(0 to 0)	0<br>(0 to 0)

China	Conflict and terrorism	3.67<br>(2.88 to 4.95)	0.31<br>(0.24 to 0.41)	2.74<br>(1.82 to 4.44)	0.19<br>(0.13 to 0.31)
Taiwan	Conflict and terrorism	0<br>(0 to 0)	0<br>(0 to 0)	0.0154<br>(0.0102 to 0.0244)	0.07<br>(0.04 to 0.1)
Hong Kong Special Administrative Region of China	Conflict and terrorism	0.00832<br>(0.00527 to 0.014)	0.14<br>(0.09 to 0.24)	0.00887<br>(0.00576 to 0.0145)	0.12<br>(0.08 to 0.19)
Macau Special Administrative Region of China	Conflict and terrorism	0.000889<br>(0.000569 to 0.00146)	0.25<br>(0.16 to 0.41)	0.0016<br>(0.00103 to 0.00269)	0.23<br>(0.15 to 0.39)
Anhui	Conflict and terrorism	0.0928<br>(0.0602 to 0.144)	0.16<br>(0.1 to 0.24)	0.0476<br>(0.032 to 0.073)	0.08<br>(0.05 to 0.12)
Beijing	Conflict and terrorism	0.0324<br>(0.0211 to 0.0528)	0.29<br>(0.19 to 0.47)	0.0574<br>(0.0371 to 0.0934)	0.24<br>(0.15 to 0.39)
Chongqing	Conflict and terrorism	0.0582<br>(0.0374 to 0.0893)	0.37<br>(0.24 to 0.57)	0.0459<br>(0.0308 to 0.0698)	0.17<br>(0.12 to 0.26)
Fujian	Conflict and terrorism	0.0601<br>(0.0385 to 0.0926)	0.19<br>(0.12 to 0.29)	0.0355<br>(0.0237 to 0.054)	0.09<br>(0.06 to 0.14)
Gansu	Conflict and terrorism	0.043<br>(0.0274 to 0.0665)	0.18<br>(0.12 to 0.28)	0.0208<br>(0.014 to 0.031)	0.08<br>(0.05 to 0.12)
Guangdong	Conflict and terrorism	0.178<br>(0.116 to 0.277)	0.27<br>(0.18 to 0.42)	0.147<br>(0.0964 to 0.228)	0.13<br>(0.08 to 0.2)
Guangxi	Conflict and terrorism	0.0793<br>(0.0507 to 0.122)	0.18<br>(0.12 to 0.28)	0.0364<br>(0.0245 to 0.0545)	0.08<br>(0.05 to 0.11)
Guizhou	Conflict and terrorism	0.0615<br>(0.0385 to 0.0945)	0.19<br>(0.12 to 0.28)	0.0309<br>(0.0213 to 0.0458)	0.09<br>(0.06 to 0.14)
Hainan	Conflict and terrorism	0.0147<br>(0.00938 to 0.0224)	0.21<br>(0.13 to 0.32)	0.00918<br>(0.00618 to 0.0139)	0.1<br>(0.06 to 0.15)
Hebei	Conflict and terrorism	0.112<br>(0.0723 to 0.173)	0.18<br>(0.11 to 0.27)	0.0688<br>(0.0461 to 0.106)	0.09<br>(0.06 to 0.14)
Heilongjiang	Conflict and terrorism	0.0559<br>(0.0361 to 0.0872)	0.15<br>(0.1 to 0.24)	0.0315<br>(0.021 to 0.0485)	0.08<br>(0.05 to 0.12)
Henan	Conflict and terrorism	0.151<br>(0.0963 to 0.234)	0.17<br>(0.11 to 0.27)	0.0707<br>(0.0476 to 0.109)	0.07<br>(0.05 to 0.11)
Hubei	Conflict and terrorism	0.0858<br>(0.0552 to 0.133)	0.15<br>(0.1 to 0.24)	0.0576<br>(0.0386 to 0.087)	0.1<br>(0.07 to 0.16)
Hunan	Conflict and terrorism	0.107<br>(0.069 to 0.167)	0.17<br>(0.11 to 0.27)	0.0523<br>(0.0351 to 0.0777)	0.08<br>(0.05 to 0.12)
Inner Mongolia	Conflict and terrorism	0.0391<br>(0.0254 to 0.0596)	0.17<br>(0.11 to 0.26)	0.022<br>(0.0147 to 0.0327)	0.09<br>(0.06 to 0.13)
Jiangsu	Conflict and terrorism	0.117<br>(0.0758 to 0.184)	0.17<br>(0.11 to 0.27)	0.0736<br>(0.0488 to 0.116)	0.09<br>(0.06 to 0.14)
Jiangxi	Conflict and terrorism	0.0779<br>(0.048 to 0.128)	0.2<br>(0.12 to 0.32)	0.0411<br>(0.0266 to 0.0658)	0.09<br>(0.06 to 0.14)
Jilin	Conflict and terrorism	0.0423<br>(0.0261 to 0.0701)	0.16<br>(0.1 to 0.27)	0.0266<br>(0.0172 to 0.0432)	0.1<br>(0.06 to 0.15)
Liaoning	Conflict and terrorism	0.0642<br>(0.0404 to 0.104)	0.16<br>(0.1 to 0.26)	0.0435<br>(0.0281 to 0.0719)	0.1<br>(0.06 to 0.16)
Ningxia	Conflict and terrorism	0.0114<br>(0.007 to 0.0189)	0.23<br>(0.14 to 0.38)	0.00953<br>(0.00614 to 0.0154)	0.14<br>(0.09 to 0.22)
Qinghai	Conflict and terrorism	0.0113<br>(0.00675 to 0.0184)	0.24<br>(0.14 to 0.39)	0.00683<br>(0.00443 to 0.0108)	0.11<br>(0.07 to 0.17)
Shaanxi	Conflict and terrorism	0.0653<br>(0.0399 to 0.107)	0.19<br>(0.12 to 0.31)	0.0436<br>(0.0276 to 0.0703)	0.11<br>(0.07 to 0.18)
Shandong	Conflict and terrorism	0.148<br>(0.0918 to 0.243)	0.17<br>(0.11 to 0.28)	0.0969<br>(0.0626 to 0.157)	0.1<br>(0.06 to 0.16)
Shanghai	Conflict and terrorism	0.0472<br>(0.0296 to 0.0782)	0.34<br>(0.21 to 0.56)	0.0758<br>(0.0465 to 0.129)	0.27<br>(0.17 to 0.46)
Shanxi	Conflict and terrorism	0.0588<br>(0.0366 to 0.0984)	0.19<br>(0.12 to 0.33)	0.039<br>(0.025 to 0.0638)	0.1<br>(0.06 to 0.17)
Sichuan	Conflict and terrorism	0.265<br>(0.205 to 0.356)	0.24<br>(0.18 to 0.32)	0.0658<br>(0.0428 to 0.105)	0.08<br>(0.05 to 0.13)
Tianjin	Conflict and terrorism	0.0208<br>(0.013 to 0.0342)	0.23<br>(0.14 to 0.37)	0.0215<br>(0.0136 to 0.0359)	0.14<br>(0.09 to 0.24)
Tibet	Conflict and terrorism	0.00641<br>(0.00379 to 0.0103)	0.28<br>(0.16 to 0.44)	0.0156<br>(0.00998 to 0.0249)	0.45<br>(0.29 to 0.72)
Xinjiang	Conflict and terrorism	1.39<br>(1.24 to 1.54)	8.78<br>(7.86 to 9.78)	1.29<br>(0.839 to 2.09)	5.31<br>(3.44 to 8.6)

Yunnan	Conflict and terrorism	0.0868 (0.0525 to 0.144)	0.22 (0.13 to 0.37)	0.0678 (0.0444 to 0.107)	0.14 (0.09 to 0.22)
Zhejiang	Conflict and terrorism	0.0882 (0.0548 to 0.146)	0.2 (0.12 to 0.33)	0.0722 (0.0459 to 0.118)	0.12 (0.07 to 0.19)
China	HIV/AIDS and sexually transmitted infections	1160 (664 to 1940)	96.99 (55.49 to 162.43)	2000 (1750 to 2350)	141.83 (123.89 to 166.49)
Taiwan	HIV/AIDS and sexually transmitted infections	7.44 (4.78 to 11.4)	36.46 (23.41 to 55.79)	14.9 (12.5 to 19.1)	63.35 (52.86 to 81.01)
Hong Kong Special Administrative Region of China	HIV/AIDS and sexually transmitted infections	3.35 (2.08 to 5.19)	58.03 (36.16 to 90.12)	2.89 (2.03 to 4.12)	38.44 (27.06 to 54.84)
Macau Special Administrative Region of China	HIV/AIDS and sexually transmitted infections	0.275 (0.153 to 0.45)	77.55 (43.14 to 126.88)	0.315 (0.198 to 0.463)	46.13 (29.01 to 67.64)
Anhui	HIV/AIDS and sexually transmitted infections	61 (29.3 to 114)	102.96 (49.38 to 192.25)	38.1 (28.2 to 52.2)	62.24 (46.17 to 85.32)
Beijing	HIV/AIDS and sexually transmitted infections	7.07 (4.02 to 11.6)	63 (35.86 to 103.7)	15.5 (10.4 to 23.5)	64.23 (43.17 to 97.34)
Chongqing	HIV/AIDS and sexually transmitted infections	37.4 (29.9 to 49.2)	236.82 (189.47 to 311.92)	68.3 (62.9 to 75.6)	255.42 (235.45 to 282.76)
Fujian	HIV/AIDS and sexually transmitted infections	25.1 (13.7 to 43.1)	79.42 (43.18 to 136.14)	28.3 (22 to 38.3)	71.91 (55.7 to 97.23)
Gansu	HIV/AIDS and sexually transmitted infections	29 (14.2 to 55)	122.47 (60.11 to 232.54)	13.3 (9.06 to 20.1)	50.03 (34.1 to 75.63)
Guangdong	HIV/AIDS and sexually transmitted infections	60 (34.2 to 102)	91.13 (52.03 to 154.97)	149 (121 to 187)	127.25 (103.57 to 160.37)
Guangxi	HIV/AIDS and sexually transmitted infections	54.1 (28.2 to 77.6)	124.42 (64.84 to 178.33)	296 (244 to 341)	612.62 (505.71 to 706.19)
Guizhou	HIV/AIDS and sexually transmitted infections	37.9 (21.8 to 57.3)	114.12 (65.54 to 172.7)	82.5 (68.8 to 94.7)	244 (203.36 to 280.02)
Hainan	HIV/AIDS and sexually transmitted infections	6.09 (3.23 to 10.9)	87.49 (46.32 to 156.53)	9.77 (7.6 to 13.1)	101.94 (79.3 to 136.36)
Hebei	HIV/AIDS and sexually transmitted infections	49 (23.6 to 91.9)	76.93 (37.07 to 144.36)	36.3 (22.6 to 59.1)	47.51 (29.63 to 77.28)
Heilongjiang	HIV/AIDS and sexually transmitted infections	22.8 (11.9 to 40.1)	62.66 (32.57 to 109.96)	18.1 (13.5 to 24.7)	46.03 (34.49 to 62.97)
Henan	HIV/AIDS and sexually transmitted infections	82 (42.9 to 138)	92.85 (48.64 to 156.82)	114 (93.5 to 138)	118.39 (97.06 to 143.09)
Hubei	HIV/AIDS and sexually transmitted infections	42.3 (21.7 to 77.7)	76.13 (39.1 to 139.91)	40.6 (33.5 to 50.6)	73.48 (60.56 to 91.41)
Hunan	HIV/AIDS and sexually transmitted infections	57.3 (35.4 to 90.7)	92.09 (56.92 to 145.85)	95 (79.6 to 111)	141.53 (118.6 to 165.51)
Inner Mongolia	HIV/AIDS and sexually transmitted infections	19.6 (9.43 to 36)	86.11 (41.49 to 158.22)	11.6 (7.6 to 17.9)	44.99 (29.33 to 69.21)
Jiangsu	HIV/AIDS and sexually transmitted infections	44.3 (24 to 76.2)	64.35 (34.94 to 110.72)	44.3 (34 to 58.7)	54.21 (41.66 to 71.85)
Jiangxi	HIV/AIDS and sexually transmitted infections	39.3 (21 to 69.9)	99.52 (53.23 to 176.91)	46.1 (35.9 to 60.1)	96.97 (75.45 to 126.33)
Jilin	HIV/AIDS and sexually transmitted infections	21.7 (11.2 to 38.9)	83.74 (43.2 to 150)	18.8 (14.4 to 25.6)	67.25 (51.59 to 91.7)
Liaoning	HIV/AIDS and sexually transmitted infections	23.3 (12.7 to 39.8)	57.08 (31.01 to 97.58)	25.7 (20.6 to 33.1)	57.54 (46.08 to 74.22)
Ningxia	HIV/AIDS and sexually transmitted infections	5.14 (2.29 to 10.2)	103.99 (46.4 to 206.03)	4.64 (3.33 to 6.74)	66.06 (47.51 to 95.96)
Qinghai	HIV/AIDS and sexually transmitted infections	5.57 (2.67 to 10.2)	117.66 (56.46 to 215.5)	5.48 (4.22 to 7.36)	87.2 (67.21 to 117.08)
Shaanxi	HIV/AIDS and sexually transmitted infections	27.7 (13.3 to 50)	80.49 (38.57 to 145.37)	24.9 (17.9 to 35.7)	63.02 (45.2 to 90.43)
Shandong	HIV/AIDS and sexually transmitted infections	51.1 (28.8 to 85.7)	59.67 (33.66 to 100.15)	40.7 (29.2 to 57.2)	41 (29.42 to 57.58)
Shanghai	HIV/AIDS and sexually transmitted infections	25.7 (12.2 to 45.4)	184.02 (87.57 to 325.08)	32.8 (18.8 to 56.7)	117.43 (67.25 to 202.76)
Shanxi	HIV/AIDS and sexually transmitted infections	26.1 (13.4 to 45.9)	86.09 (44.15 to 151.65)	24.6 (18 to 34.9)	63.77 (46.57 to 90.4)
Sichuan	HIV/AIDS and sexually transmitted infections	150 (77.9 to 252)	134.18 (69.78 to 226.14)	319 (265 to 368)	381.96 (317.97 to 440.74)
Tianjin	HIV/AIDS and sexually transmitted infections	6.13 (3.15 to 10.7)	66.64 (34.29 to 116.54)	9.15 (6.31 to 13.5)	59.94 (41.36 to 88.23)

Tibet	HIV/AIDS and sexually transmitted infections	2.53 (1.38 to 4.47)	108.55 (59.3 to 191.98)	3.21 (2.17 to 5.05)	92.74 (62.92 to 146.19)
Xinjiang	HIV/AIDS and sexually transmitted infections	24.5 (13.2 to 42)	155.21 (83.63 to 266.28)	101 (84.2 to 121)	415.91 (345.66 to 495.24)
Yunnan	HIV/AIDS and sexually transmitted infections	71.8 (36.8 to 119)	184.13 (94.33 to 305.12)	248 (208 to 290)	503.4 (423.51 to 590.44)
Zhejiang	HIV/AIDS and sexually transmitted infections	42.2 (23.9 to 73.1)	95.74 (54.29 to 165.75)	35.8 (26 to 51.2)	58.43 (42.57 to 83.66)
China	Respiratory infections and tuberculosis	46700 (43400 to 49800)	3903.32 (3621.69 to 4159.29)	7360 (6630 to 8380)	521.41 (469.61 to 593.12)
Taiwan	Respiratory infections and tuberculosis	172 (162 to 185)	844.27 (794.63 to 908.38)	213 (199 to 229)	902.43 (843.35 to 970.73)
Hong Kong Special Administrative Region of China	Respiratory infections and tuberculosis	48.1 (29.5 to 55.8)	833.85 (511.75 to 967.32)	50.3 (25.3 to 62.7)	669.76 (336.69 to 834.49)
Macau Special Administrative Region of China	Respiratory infections and tuberculosis	3.83 (3.06 to 4.31)	1079.21 (861.05 to 1215.73)	2.52 (1.77 to 3.06)	368.23 (259.53 to 446.87)
Anhui	Respiratory infections and tuberculosis	2580 (2140 to 3050)	4347.9 (3605.52 to 5146.63)	257 (212 to 330)	420.12 (346.79 to 539.01)
Beijing	Respiratory infections and tuberculosis	105 (90.4 to 120)	940.2 (806.04 to 1073.2)	65.6 (54.5 to 79.9)	272.23 (226.31 to 331.4)
Chongqing	Respiratory infections and tuberculosis	589 (459 to 753)	3733.73 (2906.71 to 4771.37)	123 (103 to 154)	458.59 (383.99 to 576.63)
Fujian	Respiratory infections and tuberculosis	891 (759 to 1030)	2814.71 (2397.49 to 3246.95)	165 (140 to 195)	417.52 (355.81 to 495.94)
Gansu	Respiratory infections and tuberculosis	1810 (1520 to 2130)	7667.16 (6441.74 to 9000.88)	163 (135 to 200)	613.5 (506.81 to 751.45)
Guangdong	Respiratory infections and tuberculosis	1860 (1590 to 2140)	2832.31 (2420.74 to 3244.98)	565 (482 to 659)	483.39 (412.86 to 564)
Guangxi	Respiratory infections and tuberculosis	1870 (1570 to 2200)	4299.31 (3608.72 to 5063.77)	393 (332 to 457)	813.8 (687.96 to 945.72)
Guizhou	Respiratory infections and tuberculosis	3650 (2900 to 4400)	11003.85 (8745.61 to 13258.62)	328 (262 to 382)	969.05 (773.55 to 1128.01)
Hainan	Respiratory infections and tuberculosis	292 (245 to 346)	4186.77 (3522.78 to 4961.81)	79.5 (68 to 92.4)	828.87 (709.04 to 963.61)
Hebei	Respiratory infections and tuberculosis	1200 (1010 to 1410)	1881.8 (1580.05 to 2215.64)	364 (310 to 437)	476.58 (405.3 to 571.42)
Heilongjiang	Respiratory infections and tuberculosis	622 (530 to 722)	1706.23 (1453.69 to 1981.07)	171 (144 to 208)	436.35 (365.77 to 529.72)
Henan	Respiratory infections and tuberculosis	2600 (2160 to 3080)	2947.9 (2450.14 to 3487)	364 (297 to 494)	378.08 (308.62 to 512.93)
Hubei	Respiratory infections and tuberculosis	1570 (1330 to 1850)	2828.83 (2390.36 to 3322.97)	240 (199 to 314)	434.05 (359.11 to 567.98)
Hunan	Respiratory infections and tuberculosis	2200 (1850 to 2620)	3545.33 (2982.44 to 4209.08)	374 (322 to 432)	557.25 (480.19 to 644.08)
Inner Mongolia	Respiratory infections and tuberculosis	763 (651 to 897)	3353.19 (2861.05 to 3944.18)	122 (101 to 148)	470.91 (391.98 to 570.68)
Jiangsu	Respiratory infections and tuberculosis	948 (809 to 1090)	1377.18 (1175.37 to 1589.47)	253 (211 to 334)	309.76 (257.81 to 409.07)
Jiangxi	Respiratory infections and tuberculosis	3110 (2490 to 3750)	7859.86 (6306.71 to 9484.45)	345 (295 to 401)	725.69 (620.93 to 843.76)
Jilin	Respiratory infections and tuberculosis	603 (525 to 685)	2325.24 (2021.48 to 2638.74)	143 (122 to 166)	511.54 (437.38 to 596.05)
Liaoning	Respiratory infections and tuberculosis	500 (437 to 566)	1226.75 (1071.01 to 1387.39)	200 (172 to 235)	449.03 (384.11 to 527.02)
Ningxia	Respiratory infections and tuberculosis	389 (300 to 493)	7877.82 (6064.4 to 9990.9)	42.3 (34.9 to 51.2)	602.49 (497.35 to 728.78)
Qinghai	Respiratory infections and tuberculosis	465 (362 to 587)	9826.01 (7640.98 to 12398.94)	59.7 (49.4 to 73.1)	949.85 (786.55 to 1164.05)
Shaanxi	Respiratory infections and tuberculosis	1420 (1180 to 1680)	4129 (3423.51 to 4892.08)	181 (148 to 226)	457.66 (374.07 to 573.17)
Shandong	Respiratory infections and tuberculosis	988 (838 to 1150)	1154.39 (978.59 to 1343.06)	282 (223 to 402)	284.12 (224.82 to 404.33)
Shanghai	Respiratory infections and tuberculosis	106 (93.7 to 121)	759.68 (670.42 to 867.44)	70.3 (56.5 to 89.5)	251.45 (202.2 to 320.44)
Shanxi	Respiratory infections and tuberculosis	855 (731 to 1000)	2824.93 (2415.19 to 3308.36)	168 (141 to 204)	435.76 (363.8 to 529.16)

Sichuan	Respiratory infections and tuberculosis	7160 (5650 to 8860)	6414.04 (5058.24 to 7937.08)	506 (427 to 595)	605.49 (511.36 to 712.41)
Tianjin	Respiratory infections and tuberculosis	125 (99.3 to 144)	1357.36 (1079.63 to 1568.79)	63.1 (50.8 to 74.7)	413.6 (332.71 to 489.21)
Tibet	Respiratory infections and tuberculosis	382 (325 to 446)	16398.34 (13959 to 19129.6)	84.6 (72.1 to 100)	2447.16 (2086.21 to 2892.95)
Xinjiang	Respiratory infections and tuberculosis	1760 (1480 to 2070)	11184.27 (9357.02 to 13140.94)	434 (372 to 510)	1780.81 (1525.58 to 2092.27)
Yunnan	Respiratory infections and tuberculosis	4300 (3430 to 5220)	11021.31 (8806.58 to 13392.33)	491 (419 to 569)	997.58 (853.14 to 1157.62)
Zhejiang	Respiratory infections and tuberculosis	948 (817 to 1100)	2150.09 (1853.31 to 2488.7)	216 (184 to 256)	352.66 (299.93 to 417.62)
China	Enteric infections	9070 (7920 to 10500)	757.51 (661.68 to 876.82)	1240 (979 to 1560)	87.97 (69.29 to 110.14)
Taiwan	Enteric infections	20 (15.6 to 24.9)	98.11 (76.5 to 122.11)	26.2 (19.3 to 34.3)	111.27 (81.65 to 145.62)
Hong Kong Special Administrative Region of China	Enteric infections	4.98 (3.81 to 6.43)	86.47 (66.11 to 111.5)	4.13 (3.05 to 5.42)	55.05 (40.6 to 72.12)
Macau Special Administrative Region of China	Enteric infections	0.576 (0.441 to 0.72)	162.36 (124.35 to 202.98)	0.386 (0.285 to 0.502)	56.38 (41.7 to 73.46)
Anhui	Enteric infections	453 (326 to 642)	764.53 (551.02 to 1083.37)	40 (29.9 to 51.1)	65.42 (48.89 to 83.5)
Beijing	Enteric infections	16.8 (12.9 to 21.3)	150.01 (115.12 to 189.57)	21.9 (15.6 to 29.3)	90.75 (64.68 to 121.4)
Chongqing	Enteric infections	134 (90.3 to 192)	850.6 (572.35 to 1214.86)	24.6 (18.2 to 32.3)	92.03 (68.04 to 120.87)
Fujian	Enteric infections	168 (127 to 220)	532.1 (400.67 to 693.82)	23 (17.6 to 29.7)	58.45 (44.7 to 75.37)
Gansu	Enteric infections	575 (421 to 786)	2429.91 (1780.06 to 3322.73)	23.3 (18.1 to 28.9)	87.51 (68.16 to 108.88)
Guangdong	Enteric infections	280 (213 to 362)	425.89 (324.29 to 549.4)	86.4 (66.6 to 111)	73.99 (57 to 94.95)
Guangxi	Enteric infections	314 (231 to 412)	721.1 (532.29 to 948.1)	52 (39.2 to 69.4)	107.68 (81.19 to 143.7)
Guizhou	Enteric infections	446 (319 to 587)	1343.95 (962.39 to 1768.92)	64.1 (46.6 to 87)	189.5 (137.73 to 257.1)
Hainan	Enteric infections	64.6 (45.1 to 91.8)	927.27 (647.52 to 1318.31)	10.8 (8.37 to 13.5)	112.29 (87.35 to 140.35)
Hebei	Enteric infections	256 (191 to 336)	402.37 (299.57 to 527.35)	50 (38.8 to 61.9)	65.42 (50.81 to 81.01)
Heilongjiang	Enteric infections	57.4 (44.1 to 75.3)	157.47 (121.1 to 206.56)	21.7 (15.4 to 29.2)	55.36 (39.26 to 74.29)
Henan	Enteric infections	633 (462 to 861)	717.48 (523.26 to 975.45)	70.5 (52 to 91.8)	73.23 (53.95 to 95.31)
Hubei	Enteric infections	246 (178 to 330)	442.13 (320.15 to 594.12)	42 (31.3 to 54.8)	75.88 (56.56 to 99.04)
Hunan	Enteric infections	381 (274 to 499)	613.14 (440.79 to 802.31)	63.3 (48.4 to 82.3)	94.24 (72.07 to 122.63)
Inner Mongolia	Enteric infections	138 (100 to 186)	608.66 (440.72 to 817.16)	12.2 (9.3 to 15.5)	46.93 (35.91 to 59.67)
Jiangsu	Enteric infections	156 (120 to 204)	226.98 (173.66 to 296.57)	51.3 (37.8 to 67.4)	62.77 (46.33 to 82.48)
Jiangxi	Enteric infections	604 (426 to 829)	1526.79 (1077.2 to 2097.57)	46.6 (36.2 to 59)	98.1 (76.08 to 124.19)
Jilin	Enteric infections	52.3 (38.3 to 70.3)	201.37 (147.66 to 270.89)	11.8 (8.93 to 15.3)	42.43 (32.02 to 54.72)
Liaoning	Enteric infections	62.2 (47.8 to 79.6)	152.4 (117.13 to 195.09)	29.3 (21.1 to 39.2)	65.63 (47.33 to 87.88)
Ningxia	Enteric infections	66.2 (44.9 to 93.8)	1339.5 (908.21 to 1898.51)	4.51 (3.39 to 5.73)	64.25 (48.36 to 81.65)
Qinghai	Enteric infections	143 (99.9 to 203)	3018.78 (2110.03 to 4278.66)	6.91 (5.45 to 8.77)	109.94 (86.75 to 139.58)
Shaanxi	Enteric infections	411 (304 to 545)	1193.05 (882.09 to 1585.26)	26.4 (20.5 to 33.7)	66.87 (51.92 to 85.22)
Shandong	Enteric infections	196 (152 to 251)	228.75 (177.67 to 293.59)	59.8 (43 to 80.2)	60.2 (43.3 to 80.75)

Shanghai	Enteric infections	12.9 (10.1 to 16.2)	92.51 (72.16 to 115.66)	10.7 (8.01 to 13.8)	38.46 (28.67 to 49.52)
Shanxi	Enteric infections	149 (110 to 199)	492.28 (362.23 to 658.58)	23.4 (17.8 to 30.2)	60.62 (46.07 to 78.25)
Sichuan	Enteric infections	1060 (761 to 1480)	948.07 (681.46 to 1322.85)	62.6 (46.9 to 80.9)	75.03 (56.2 to 96.85)
Tianjin	Enteric infections	8.52 (6.73 to 10.8)	92.68 (73.17 to 117.24)	5.52 (4.01 to 7.3)	36.17 (26.28 to 47.8)
Tibet	Enteric infections	120 (90.1 to 163)	5165.77 (3866.3 to 6993.1)	19.1 (14.3 to 25.5)	551.87 (414.9 to 738.99)
Xinjiang	Enteric infections	480 (349 to 629)	3042.87 (2213.88 to 3989.97)	62.2 (46.8 to 82.3)	255.25 (192.04 to 337.6)
Yunnan	Enteric infections	1220 (900 to 1590)	3119.19 (2310.36 to 4080.29)	147 (108 to 199)	298.31 (219.12 to 405.37)
Zhejiang	Enteric infections	164 (120 to 214)	371.09 (272.94 to 486.02)	65.3 (48.3 to 86.4)	106.69 (78.94 to 141.16)
China	Typhoid and paratyphoid	398 (198 to 713)	33.26 (16.51 to 59.55)	234 (122 to 404)	16.56 (8.63 to 28.64)
Taiwan	Typhoid and paratyphoid	0.136 (0.0941 to 0.183)	0.67 (0.46 to 0.9)	0.026 (0.0182 to 0.0376)	0.11 (0.08 to 0.16)
Hong Kong Special Administrative Region of China	Typhoid and paratyphoid	1.17 (0.587 to 2.07)	20.31 (10.19 to 35.99)	0.339 (0.165 to 0.606)	4.51 (2.2 to 8.07)
Macau Special Administrative Region of China	Typhoid and paratyphoid	0.0748 (0.0376 to 0.134)	21.08 (10.59 to 37.8)	0.0274 (0.0133 to 0.0486)	4 (1.95 to 7.11)
Anhui	Typhoid and paratyphoid	5.88 (2.51 to 12)	9.92 (4.24 to 20.32)	2.81 (1.37 to 5.06)	4.59 (2.24 to 8.27)
Beijing	Typhoid and paratyphoid	0.414 (0.155 to 0.934)	3.69 (1.38 to 8.33)	0.243 (0.111 to 0.457)	1.01 (0.46 to 1.9)
Chongqing	Typhoid and paratyphoid	1.94 (0.891 to 3.62)	12.29 (5.64 to 22.97)	1.67 (0.825 to 3.02)	6.23 (3.09 to 11.29)
Fujian	Typhoid and paratyphoid	8.25 (4.01 to 15.1)	26.08 (12.67 to 47.67)	4.83 (2.39 to 8.54)	12.25 (6.05 to 21.67)
Gansu	Typhoid and paratyphoid	2.25 (0.873 to 4.84)	9.52 (3.69 to 20.45)	1.01 (0.473 to 1.87)	3.8 (1.78 to 7.03)
Guangdong	Typhoid and paratyphoid	24.3 (11.7 to 43.3)	36.99 (17.71 to 65.76)	20.9 (10.5 to 37.8)	17.87 (8.96 to 32.38)
Guangxi	Typhoid and paratyphoid	30.5 (15.2 to 53.6)	70.05 (34.9 to 123.17)	18.8 (9.44 to 33.9)	38.85 (19.53 to 70.1)
Guizhou	Typhoid and paratyphoid	56.2 (27.2 to 99.6)	169.4 (81.9 to 300.02)	28.6 (14.8 to 49.1)	84.61 (43.87 to 145.15)
Hainan	Typhoid and paratyphoid	0.498 (0.201 to 1.05)	7.15 (2.88 to 15.06)	0.302 (0.148 to 0.574)	3.15 (1.54 to 5.98)
Hebei	Typhoid and paratyphoid	6.86 (2.97 to 13.5)	10.77 (4.67 to 21.19)	3.74 (1.83 to 6.76)	4.9 (2.39 to 8.84)
Heilongjiang	Typhoid and paratyphoid	2.97 (0.987 to 7.54)	8.16 (2.71 to 20.69)	0.563 (0.257 to 1.05)	1.43 (0.65 to 2.68)
Henan	Typhoid and paratyphoid	7.47 (2.67 to 17.8)	8.47 (3.03 to 20.12)	2.69 (1.25 to 5.07)	2.79 (1.29 to 5.26)
Hubei	Typhoid and paratyphoid	10.2 (4.82 to 18.4)	18.28 (8.68 to 33.1)	4.46 (2.27 to 8.2)	8.06 (4.1 to 14.82)
Hunan	Typhoid and paratyphoid	28.8 (14.6 to 51.4)	46.38 (23.48 to 82.61)	16.4 (8.25 to 29.3)	24.5 (12.28 to 43.69)
Inner Mongolia	Typhoid and paratyphoid	1.75 (0.68 to 3.9)	7.71 (2.99 to 17.14)	0.545 (0.27 to 1)	2.11 (1.04 to 3.88)
Jiangsu	Typhoid and paratyphoid	9.3 (4.51 to 17)	13.51 (6.56 to 24.76)	5.14 (2.56 to 9.24)	6.29 (3.13 to 11.32)
Jiangxi	Typhoid and paratyphoid	14.2 (7.08 to 26.8)	35.97 (17.9 to 67.71)	9.04 (4.54 to 15.8)	19.01 (9.56 to 33.14)
Jilin	Typhoid and paratyphoid	1.69 (0.571 to 4.15)	6.53 (2.2 to 15.98)	0.358 (0.16 to 0.699)	1.28 (0.58 to 2.51)
Liaoning	Typhoid and paratyphoid	5.95 (2.81 to 11.2)	14.59 (6.88 to 27.38)	2.51 (1.24 to 4.36)	5.61 (2.78 to 9.76)
Ningxia	Typhoid and paratyphoid	0.373 (0.135 to 0.933)	7.55 (2.73 to 18.9)	0.178 (0.0825 to 0.327)	2.54 (1.18 to 4.66)
Qinghai	Typhoid and paratyphoid	0.401 (0.141 to 0.976)	8.46 (2.98 to 20.6)	0.167 (0.076 to 0.312)	2.65 (1.21 to 4.97)

Shaanxi	Typhoid and paratyphoid	1.9<br>(0.623 to 5.12)	5.52<br>(1.81 to 14.88)	0.468<br>(0.206 to 0.932)	1.18<br>(0.52 to 2.36)
Shandong	Typhoid and paratyphoid	5.6<br>(1.98 to 13.6)	6.54<br>(2.32 to 15.84)	1.89<br>(0.902 to 3.49)	1.9<br>(0.91 to 3.51)
Shanghai	Typhoid and paratyphoid	0.889<br>(0.417 to 1.63)	6.36<br>(2.98 to 11.7)	0.791<br>(0.39 to 1.4)	2.83<br>(1.4 to 5)
Shanxi	Typhoid and paratyphoid	9.23<br>(4.42 to 16.7)	30.48<br>(14.6 to 55.06)	5.16<br>(2.56 to 9.03)	13.36<br>(6.63 to 23.37)
Sichuan	Typhoid and paratyphoid	16<br>(7.52 to 29.3)	14.3<br>(6.74 to 26.3)	5.72<br>(2.8 to 10.3)	6.85<br>(3.35 to 12.35)
Tianjin	Typhoid and paratyphoid	0.412<br>(0.169 to 0.877)	4.48<br>(1.84 to 9.54)	0.234<br>(0.116 to 0.434)	1.54<br>(0.76 to 2.84)
Tibet	Typhoid and paratyphoid	0.193<br>(0.065 to 0.482)	8.28<br>(2.79 to 20.68)	0.107<br>(0.0476 to 0.212)	3.08<br>(1.38 to 6.13)
Xinjiang	Typhoid and paratyphoid	11.6<br>(5.65 to 20.4)	73.83<br>(35.84 to 129.4)	10.2<br>(5.04 to 17.9)	41.78<br>(20.68 to 73.45)
Yunnan	Typhoid and paratyphoid	100<br>(49.6 to 177)	257.64<br>(127.34 to 455.1)	63.4<br>(32.7 to 110)	128.88<br>(66.44 to 223.31)
Zhejiang	Typhoid and paratyphoid	30.4<br>(15.4 to 53.1)	68.94<br>(34.82 to 120.36)	20.7<br>(10.5 to 36.4)	33.79<br>(17.21 to 59.43)
China	Invasive Non-typhoidal Salmonella (iNTS)	66.3<br>(34.8 to 117)	5.54<br>(2.91 to 9.77)	26.9<br>(13.7 to 47.2)	1.9<br>(0.97 to 3.34)
Taiwan	Invasive Non-typhoidal Salmonella (iNTS)	4.05<br>(2.21 to 6.88)	19.86<br>(10.84 to 33.71)	1.75<br>(0.897 to 3.06)	7.43<br>(3.8 to 12.96)
Hong Kong Special Administrative Region of China	Invasive Non-typhoidal Salmonella (iNTS)	0.099<br>(0.0469 to 0.183)	1.72<br>(0.81 to 3.17)	0.0997<br>(0.0445 to 0.179)	1.33<br>(0.59 to 2.38)
Macau Special Administrative Region of China	Invasive Non-typhoidal Salmonella (iNTS)	0.00643<br>(0.00307 to 0.0116)	1.81<br>(0.86 to 3.27)	0.00783<br>(0.00346 to 0.0148)	1.14<br>(0.51 to 2.16)
Anhui	Invasive Non-typhoidal Salmonella (iNTS)	4.86<br>(2.41 to 9.06)	8.21<br>(4.07 to 15.29)	1.48<br>(0.748 to 2.68)	2.42<br>(1.22 to 4.39)
Beijing	Invasive Non-typhoidal Salmonella (iNTS)	0.241<br>(0.11 to 0.456)	2.15<br>(0.98 to 4.06)	0.239<br>(0.109 to 0.446)	0.99<br>(0.45 to 1.85)
Chongqing	Invasive Non-typhoidal Salmonella (iNTS)	0.788<br>(0.393 to 1.38)	4.99<br>(2.49 to 8.72)	0.471<br>(0.234 to 0.827)	1.76<br>(0.88 to 3.09)
Fujian	Invasive Non-typhoidal Salmonella (iNTS)	1.74<br>(0.883 to 3.16)	5.5<br>(2.79 to 9.98)	0.653<br>(0.311 to 1.2)	1.66<br>(0.79 to 3.05)
Gansu	Invasive Non-typhoidal Salmonella (iNTS)	2.41<br>(1.22 to 4.35)	10.18<br>(5.17 to 18.39)	0.73<br>(0.367 to 1.3)	2.75<br>(1.38 to 4.89)
Guangdong	Invasive Non-typhoidal Salmonella (iNTS)	2.72<br>(1.36 to 4.88)	4.14<br>(2.06 to 7.41)	1.95<br>(0.937 to 3.65)	1.67<br>(0.8 to 3.12)
Guangxi	Invasive Non-typhoidal Salmonella (iNTS)	2.05<br>(1.01 to 3.88)	4.71<br>(2.32 to 8.92)	0.833<br>(0.383 to 1.68)	1.72<br>(0.79 to 3.47)
Guizhou	Invasive Non-typhoidal Salmonella (iNTS)	2.61<br>(1.31 to 4.66)	7.85<br>(3.94 to 14.03)	0.897<br>(0.44 to 1.6)	2.65<br>(1.3 to 4.74)
Hainan	Invasive Non-typhoidal Salmonella (iNTS)	0.274<br>(0.139 to 0.498)	3.94<br>(2 to 7.15)	0.183<br>(0.0898 to 0.333)	1.9<br>(0.94 to 3.47)
Hebei	Invasive Non-typhoidal Salmonella (iNTS)	3.33<br>(1.67 to 5.86)	5.23<br>(2.62 to 9.21)	1.57<br>(0.777 to 2.82)	2.05<br>(1.02 to 3.7)
Heilongjiang	Invasive Non-typhoidal Salmonella (iNTS)	2.2<br>(1.05 to 3.9)	6.04<br>(2.88 to 10.7)	0.828<br>(0.403 to 1.45)	2.11<br>(1.03 to 3.7)
Henan	Invasive Non-typhoidal Salmonella (iNTS)	6.77<br>(3.54 to 11.9)	7.67<br>(4.01 to 13.45)	2.27<br>(1.12 to 4.09)	2.35<br>(1.16 to 4.25)
Hubei	Invasive Non-typhoidal Salmonella (iNTS)	2.55<br>(1.31 to 4.53)	4.6<br>(2.36 to 8.16)	1<br>(0.482 to 1.82)	1.82<br>(0.87 to 3.29)
Hunan	Invasive Non-typhoidal Salmonella (iNTS)	3.1<br>(1.53 to 5.37)	4.98<br>(2.46 to 8.63)	1.22<br>(0.603 to 2.28)	1.82<br>(0.9 to 3.39)
Inner Mongolia	Invasive Non-typhoidal Salmonella (iNTS)	1.29<br>(0.625 to 2.29)	5.68<br>(2.75 to 10.08)	0.476<br>(0.237 to 0.853)	1.84<br>(0.91 to 3.3)
Jiangsu	Invasive Non-typhoidal Salmonella (iNTS)	2.46<br>(1.2 to 4.38)	3.57<br>(1.75 to 6.36)	1.22<br>(0.584 to 2.2)	1.49<br>(0.71 to 2.7)
Jiangxi	Invasive Non-typhoidal Salmonella (iNTS)	2.79<br>(1.47 to 4.86)	7.07<br>(3.71 to 12.3)	1.14<br>(0.576 to 2.01)	2.39<br>(1.21 to 4.22)
Jilin	Invasive Non-typhoidal Salmonella (iNTS)	1.29<br>(0.627 to 2.36)	4.95<br>(2.42 to 9.1)	0.482<br>(0.213 to 0.871)	1.73<br>(0.76 to 3.12)
Liaoning	Invasive Non-typhoidal Salmonella (iNTS)	1.47<br>(0.687 to 2.76)	3.59<br>(1.68 to 6.76)	0.706<br>(0.337 to 1.28)	1.58<br>(0.75 to 2.87)

Ningxia	Invasive Non-typhoidal Salmonella (iNTS)	0.367 (0.185 to 0.672)	7.44 (3.75 to 13.61)	0.156 (0.076 to 0.285)	2.22 (1.08 to 4.06)
Qinghai	Invasive Non-typhoidal Salmonella (iNTS)	0.383 (0.197 to 0.693)	8.09 (4.17 to 14.63)	0.158 (0.0794 to 0.291)	2.52 (1.26 to 4.64)
Shaanxi	Invasive Non-typhoidal Salmonella (iNTS)	1.85 (0.937 to 3.31)	5.37 (2.72 to 9.62)	0.724 (0.348 to 1.33)	1.83 (0.88 to 3.37)
Shandong	Invasive Non-typhoidal Salmonella (iNTS)	4.51 (2.32 to 7.84)	5.27 (2.71 to 9.16)	1.79 (0.884 to 3.27)	1.81 (0.89 to 3.29)
Shanghai	Invasive Non-typhoidal Salmonella (iNTS)	0.268 (0.122 to 0.498)	1.92 (0.87 to 3.57)	0.313 (0.142 to 0.581)	1.12 (0.51 to 2.08)
Shanxi	Invasive Non-typhoidal Salmonella (iNTS)	1.56 (0.764 to 2.81)	5.16 (2.52 to 9.27)	0.702 (0.335 to 1.26)	1.82 (0.87 to 3.26)
Sichuan	Invasive Non-typhoidal Salmonella (iNTS)	5.76 (2.87 to 10.2)	5.16 (2.57 to 9.13)	1.57 (0.792 to 2.74)	1.88 (0.95 to 3.29)
Tianjin	Invasive Non-typhoidal Salmonella (iNTS)	0.245 (0.112 to 0.454)	2.66 (1.22 to 4.94)	0.198 (0.0918 to 0.368)	1.3 (0.6 to 2.41)
Tibet	Invasive Non-typhoidal Salmonella (iNTS)	0.277 (0.142 to 0.487)	11.89 (6.1 to 20.9)	0.177 (0.0903 to 0.325)	5.12 (2.61 to 9.41)
Xinjiang	Invasive Non-typhoidal Salmonella (iNTS)	0.964 (0.481 to 1.81)	6.11 (3.05 to 11.49)	0.487 (0.233 to 0.899)	2 (0.95 to 3.69)
Yunnan	Invasive Non-typhoidal Salmonella (iNTS)	3.42 (1.72 to 6.28)	8.77 (4.41 to 16.11)	1.22 (0.602 to 2.17)	2.48 (1.22 to 4.42)
Zhejiang	Invasive Non-typhoidal Salmonella (iNTS)	1.68 (0.811 to 3.12)	3.81 (1.84 to 7.08)	0.89 (0.414 to 1.64)	1.46 (0.68 to 2.69)
China	Other infectious diseases	13000 (9400 to 19200)	1087.72 (785.72 to 1599.87)	1610 (1410 to 1860)	114.15 (99.49 to 131.67)
Taiwan	Other infectious diseases	26.8 (23.9 to 30.4)	131.45 (117 to 148.82)	25.9 (20.7 to 30.1)	110 (87.95 to 127.77)
Hong Kong Special Administrative Region of China	Other infectious diseases	6.97 (5.57 to 10.2)	121.01 (96.58 to 176.69)	4.67 (3.87 to 5.91)	62.25 (51.54 to 78.65)
Macau Special Administrative Region of China	Other infectious diseases	0.734 (0.465 to 1.06)	206.87 (130.96 to 299.81)	0.498 (0.325 to 0.637)	72.79 (47.58 to 93.15)
Anhui	Other infectious diseases	729 (465 to 1310)	1231.02 (785.58 to 2211.5)	69.7 (55.3 to 90.4)	113.98 (90.51 to 147.86)
Beijing	Other infectious diseases	22.7 (17 to 34.9)	202.8 (151.82 to 310.79)	13.3 (10.6 to 17.2)	55.22 (44.12 to 71.22)
Chongqing	Other infectious diseases	183 (112 to 347)	1158.9 (706.56 to 2196.83)	24 (19.3 to 31)	89.71 (72.36 to 115.8)
Fujian	Other infectious diseases	252 (160 to 448)	797.28 (506.49 to 1414.78)	32 (25.9 to 40.9)	81.29 (65.63 to 103.87)
Gansu	Other infectious diseases	572 (382 to 1000)	2417.16 (1616.19 to 4231.59)	46 (36.7 to 59.8)	172.89 (138.25 to 225.07)
Guangdong	Other infectious diseases	459 (331 to 719)	697.25 (502.17 to 1092.1)	116 (95.2 to 142)	98.97 (81.51 to 121.6)
Guangxi	Other infectious diseases	585 (398 to 968)	1345.01 (914.24 to 2225.16)	74.2 (61.1 to 91.4)	153.65 (126.41 to 189.09)
Guizhou	Other infectious diseases	850 (530 to 1480)	2560.33 (1596.92 to 4448.73)	60.4 (46.2 to 79.5)	178.46 (136.6 to 235.13)
Hainan	Other infectious diseases	77.9 (53.8 to 127)	1118.55 (772.56 to 1816.15)	17.2 (13.1 to 21.7)	179.5 (137.04 to 225.86)
Hebei	Other infectious diseases	554 (361 to 898)	869.71 (567.67 to 1410.22)	114 (91.2 to 137)	149.3 (119.28 to 179.02)
Heilongjiang	Other infectious diseases	236 (157 to 432)	647.3 (431.57 to 1184.66)	40.9 (34 to 50.2)	104.32 (86.62 to 127.81)
Henan	Other infectious diseases	1020 (659 to 1780)	1155.83 (746.85 to 2014.06)	114 (94.5 to 138)	118.66 (98.16 to 143.75)
Hubei	Other infectious diseases	497 (313 to 884)	895.33 (563.49 to 1591.52)	51.3 (41 to 67)	92.72 (74.18 to 121.16)
Hunan	Other infectious diseases	604 (392 to 1060)	971.17 (630.59 to 1706.75)	81 (66.5 to 99.7)	120.64 (99.02 to 148.47)
Inner Mongolia	Other infectious diseases	222 (143 to 396)	978.17 (627.55 to 1739.83)	25.8 (21.1 to 31.2)	99.79 (81.65 to 120.44)
Jiangsu	Other infectious diseases	346 (235 to 596)	502.76 (341.01 to 866.55)	56.3 (45.8 to 71.4)	68.92 (56.11 to 87.47)
Jiangxi	Other infectious diseases	850 (567 to 1440)	2150.03 (1435.43 to 3636.58)	84.3 (67.6 to 103)	177.29 (142.28 to 217.21)

Jilin	Other infectious diseases	204<br>(135 to 351)	786.93<br>(521.7 to 1353.85)	30.9<br>(25.1 to 36.8)	110.67<br>(89.89 to 131.88)
Liaoning	Other infectious diseases	149<br>(106 to 250)	364.82<br>(259.34 to 613.86)	33.2<br>(27 to 42.1)	74.28<br>(60.48 to 94.32)
Ningxia	Other infectious diseases	82<br>(49.8 to 143)	1660.16<br>(1007.48 to 2905.55)	8.2<br>(6.41 to 11.1)	116.84<br>(91.32 to 158.31)
Qinghai	Other infectious diseases	129<br>(80 to 217)	2722.11<br>(1689.34 to 4578.65)	13.8<br>(10.8 to 18.3)	219.59<br>(172.31 to 290.97)
Shaanxi	Other infectious diseases	445<br>(291 to 823)	1294.64<br>(846.03 to 2393.12)	52.8<br>(42.3 to 65.6)	133.75<br>(107.15 to 166.15)
Shandong	Other infectious diseases	492<br>(328 to 919)	574.69<br>(383.36 to 1073.14)	80.7<br>(66 to 102)	81.23<br>(66.45 to 103.13)
Shanghai	Other infectious diseases	33<br>(26.4 to 44.1)	236.53<br>(188.95 to 315.45)	18.8<br>(15.3 to 22.8)	67.3<br>(54.79 to 81.71)
Shanxi	Other infectious diseases	254<br>(167 to 455)	837.6<br>(551.14 to 1504.11)	40.5<br>(33.2 to 50)	104.77<br>(85.85 to 129.5)
Sichuan	Other infectious diseases	1520<br>(1010 to 2530)	1359.21<br>(908.29 to 2268.95)	92.8<br>(76.2 to 113)	111.15<br>(91.32 to 135.71)
Tianjin	Other infectious diseases	24.2<br>(17.3 to 40.3)	262.9<br>(187.8 to 438.62)	9.92<br>(7.84 to 13.1)	65.02<br>(51.36 to 85.7)
Tibet	Other infectious diseases	76.4<br>(48.5 to 125)	3279.72<br>(2080.44 to 5367.58)	17.7<br>(12.1 to 27.6)	512.48<br>(349.51 to 798.59)
Xinjiang	Other infectious diseases	325<br>(215 to 548)	2059.93<br>(1363.05 to 3477.35)	49.1<br>(36.1 to 62.6)	201.3<br>(148.07 to 256.8)
Yunnan	Other infectious diseases	945<br>(613 to 1570)	2423.67<br>(1572.47 to 4020.84)	90.9<br>(73.1 to 117)	184.96<br>(148.67 to 237.55)
Zhejiang	Other infectious diseases	277<br>(200 to 447)	627.89<br>(454.56 to 1013.7)	47.5<br>(39.8 to 57.2)	77.56<br>(65 to 93.46)
China	Maternal and neonatal disorders	33200<br>(31000 to 35600)	2775.44<br>(2593.75 to 2970.24)	11400<br>(9520 to 13500)	803.75<br>(673.85 to 955.35)
Taiwan	Maternal and neonatal disorders	80.4<br>(70.5 to 91.5)	393.88<br>(345.77 to 448.41)	70.1<br>(56.7 to 87.5)	297.4<br>(240.28 to 371.15)
Hong Kong Special Administrative Region of China	Maternal and neonatal disorders	29.3<br>(25.5 to 33.3)	507.51<br>(443 to 577.39)	22.5<br>(16.6 to 30.2)	299.86<br>(220.59 to 401.81)
Macau Special Administrative Region of China	Maternal and neonatal disorders	3.28<br>(2.84 to 3.75)	925.33<br>(801.45 to 1058.02)	2.73<br>(2.1 to 3.58)	399.01<br>(306.77 to 524.21)
Anhui	Maternal and neonatal disorders	2360<br>(1950 to 2800)	3979.66<br>(3289.02 to 4726.99)	622<br>(464 to 850)	1017.76<br>(758.33 to 1390.33)
Beijing	Maternal and neonatal disorders	105<br>(88.4 to 122)	934.11<br>(787.75 to 1086.31)	150<br>(121 to 186)	623.68<br>(503.54 to 772.56)
Chongqing	Maternal and neonatal disorders	484<br>(356 to 631)	3065.55<br>(2256.69 to 3999.52)	149<br>(122 to 178)	558.59<br>(456.63 to 666.66)
Fujian	Maternal and neonatal disorders	801<br>(677 to 929)	2531.19<br>(2139.8 to 2937.41)	268<br>(219 to 327)	681.16<br>(554.82 to 830.06)
Gansu	Maternal and neonatal disorders	1710<br>(1450 to 1980)	7237.1<br>(6127.93 to 8379.31)	332<br>(266 to 397)	1248.21<br>(1002.12 to 1492.78)
Guangdong	Maternal and neonatal disorders	1140<br>(974 to 1340)	1733.62<br>(1479.49 to 2034.65)	830<br>(675 to 1010)	710.07<br>(577.53 to 867.92)
Guangxi	Maternal and neonatal disorders	859<br>(722 to 1020)	1975.25<br>(1660.31 to 2349.63)	329<br>(267 to 402)	680.41<br>(553.1 to 831.59)
Guizhou	Maternal and neonatal disorders	1190<br>(947 to 1490)	3583.21<br>(2852.26 to 4503.65)	199<br>(160 to 245)	588.2<br>(473.54 to 723.45)
Hainan	Maternal and neonatal disorders	211<br>(169 to 259)	3035.22<br>(2426.14 to 3711.44)	114<br>(94.5 to 136)	1189.59<br>(985.41 to 1422.64)
Hebei	Maternal and neonatal disorders	1580<br>(1360 to 1840)	2483.94<br>(2134.8 to 2889.62)	1200<br>(877 to 1590)	1565.27<br>(1147.98 to 2086)
Heilongjiang	Maternal and neonatal disorders	558<br>(459 to 670)	1529.46<br>(1260.3 to 1838.61)	214<br>(166 to 267)	545.88<br>(423.05 to 681.42)
Henan	Maternal and neonatal disorders	2390<br>(2030 to 2810)	2705.71<br>(2300.2 to 3186.12)	685<br>(543 to 853)	711.6<br>(563.52 to 885.62)
Hubei	Maternal and neonatal disorders	1260<br>(1060 to 1500)	2274.03<br>(1902.54 to 2691.85)	313<br>(247 to 386)	566.55<br>(446.34 to 697.63)
Hunan	Maternal and neonatal disorders	1280<br>(1070 to 1540)	2062.73<br>(1721.87 to 2476.85)	355<br>(280 to 450)	528.7<br>(417.51 to 669.54)
Inner Mongolia	Maternal and neonatal disorders	716<br>(602 to 841)	3147.52<br>(2646.02 to 3698.4)	197<br>(159 to 240)	761.29<br>(614.41 to 926.97)

Jiangsu	Maternal and neonatal disorders	1030<br>(842 to 1220)	1490.63<br>(1224.08 to 1775.27)	445<br>(348 to 564)	544.98<br>(426.64 to 691.26)
Jiangxi	Maternal and neonatal disorders	1820<br>(1450 to 2250)	4601.23<br>(3656.83 to 5686.66)	467<br>(386 to 555)	981.64<br>(812.87 to 1168.14)
Jilin	Maternal and neonatal disorders	537<br>(453 to 628)	2070.07<br>(1746.38 to 2418.29)	155<br>(127 to 187)	555.03<br>(454.33 to 670.46)
Liaoning	Maternal and neonatal disorders	393<br>(330 to 469)	962.48<br>(808.31 to 1148.67)	179<br>(142 to 222)	400.9<br>(316.96 to 496.63)
Ningxia	Maternal and neonatal disorders	287<br>(219 to 360)	5801.24<br>(4443.87 to 7295.09)	81.3<br>(65.5 to 99.9)	1158.88<br>(933.31 to 1423.26)
Qinghai	Maternal and neonatal disorders	294<br>(223 to 367)	6201.9<br>(4709.24 to 7755.88)	81.8<br>(64.9 to 103)	1302.21<br>(1032.3 to 1634.52)
Shaanxi	Maternal and neonatal disorders	1250<br>(1070 to 1450)	3640.77<br>(3105.79 to 4223.03)	595<br>(419 to 814)	1505.58<br>(1060.99 to 2059.6)
Shandong	Maternal and neonatal disorders	1190<br>(1010 to 1410)	1387.17<br>(1175.12 to 1646.34)	520<br>(412 to 648)	523.22<br>(414.61 to 651.81)
Shanghai	Maternal and neonatal disorders	117<br>(97.5 to 138)	834.86<br>(697.67 to 986.38)	166<br>(134 to 204)	592.71<br>(479.58 to 728.56)
Shanxi	Maternal and neonatal disorders	945<br>(819 to 1080)	3121.47<br>(2703.52 to 3577.56)	320<br>(263 to 381)	828.89<br>(681.71 to 985.48)
Sichuan	Maternal and neonatal disorders	4550<br>(3570 to 5650)	4079.02<br>(3199.67 to 5064.66)	854<br>(631 to 1160)	1022.83<br>(755.86 to 1388.86)
Tianjin	Maternal and neonatal disorders	99.5<br>(83.9 to 118)	1081.61<br>(911.74 to 1281.19)	90.1<br>(73.2 to 108)	590.11<br>(479.73 to 708.87)
Tibet	Maternal and neonatal disorders	148<br>(117 to 182)	6367.38<br>(5013.17 to 7795.97)	80<br>(63.5 to 100)	2314.57<br>(1836.41 to 2897.02)
Xinjiang	Maternal and neonatal disorders	821<br>(665 to 993)	5202.2<br>(4216.53 to 6298.19)	398<br>(317 to 494)	1633.48<br>(1300.41 to 2027.01)
Yunnan	Maternal and neonatal disorders	2050<br>(1590 to 2580)	5256.61<br>(4073.71 to 6620.79)	553<br>(443 to 675)	1123.95<br>(900.74 to 1373.45)
Zhejiang	Maternal and neonatal disorders	1010<br>(864 to 1190)	2299.29<br>(1959.22 to 2690.42)	389<br>(310 to 486)	635.46<br>(506.81 to 794.37)
China	Headache disorders	4850<br>(3120 to 6990)	405.42<br>(260.55 to 584.15)	6610<br>(4270 to 9560)	467.89<br>(302.65 to 676.79)
Taiwan	Headache disorders	122<br>(78.1 to 180)	598.89<br>(382.55 to 883.13)	161<br>(103 to 236)	682.04<br>(438.86 to 1001.38)
Hong Kong Special Administrative Region of China	Headache disorders	21<br>(13.7 to 30.9)	364.82<br>(237.18 to 535.96)	30.9<br>(20.2 to 45.9)	411.47<br>(268.7 to 611.4)
Macau Special Administrative Region of China	Headache disorders	1.51<br>(0.965 to 2.19)	424.33<br>(271.93 to 616.1)	3.35<br>(2.18 to 4.84)	489.33<br>(318.45 to 707.39)
Anhui	Headache disorders	231<br>(150 to 333)	389.47<br>(252.49 to 561.67)	280<br>(183 to 404)	458.03<br>(299.67 to 659.91)
Beijing	Headache disorders	49.1<br>(31.5 to 70.5)	437.71<br>(280.63 to 628.11)	118<br>(76.5 to 171)	489.69<br>(317.3 to 709.35)
Chongqing	Headache disorders	65.7<br>(42.5 to 94.1)	416.15<br>(269.55 to 596.06)	120<br>(78.5 to 173)	448.37<br>(293.62 to 646.11)
Fujian	Headache disorders	121<br>(78 to 175)	381.23<br>(246.63 to 553.3)	184<br>(120 to 264)	466.15<br>(304.32 to 669.94)
Gansu	Headache disorders	92.2<br>(60 to 133)	390<br>(253.72 to 563.89)	122<br>(78.8 to 175)	457.17<br>(296.5 to 658.98)
Guangdong	Headache disorders	253<br>(164 to 366)	384.78<br>(249.24 to 556.08)	538<br>(353 to 780)	460.53<br>(302 to 667.4)
Guangxi	Headache disorders	164<br>(105 to 235)	376.06<br>(241.37 to 540.4)	211<br>(136 to 306)	436.23<br>(281.52 to 633.66)
Guizhou	Headache disorders	127<br>(81.6 to 182)	381.53<br>(245.84 to 548.21)	152<br>(98.7 to 220)	448.1<br>(291.83 to 650.43)
Hainan	Headache disorders	25.4<br>(16.6 to 36.9)	365.25<br>(237.78 to 529.9)	42.6<br>(27.6 to 61.7)	444.78<br>(288.26 to 643.96)
Hebei	Headache disorders	252<br>(163 to 368)	396.47<br>(255.7 to 577.98)	342<br>(223 to 494)	447.5<br>(291.88 to 645.96)
Heilongjiang	Headache disorders	200<br>(130 to 289)	547.82<br>(355.32 to 792.68)	258<br>(168 to 372)	657.49<br>(428.19 to 946.55)
Henan	Headache disorders	349<br>(225 to 504)	395.65<br>(255.2 to 570.85)	428<br>(277 to 617)	444.23<br>(288.15 to 640.41)
Hubei	Headache disorders	222<br>(142 to 321)	399.13<br>(256.23 to 578.6)	261<br>(170 to 378)	472.86<br>(308.16 to 683.3)

Hunan	Headache disorders	249<br>(162 to 360)	401<br>(260.8 to 578.74)	306<br>(199 to 441)	455.65<br>(296.73 to 657.26)
Inner Mongolia	Headache disorders	89.2<br>(57.2 to 130)	392.06<br>(251.48 to 569.49)	124<br>(80.8 to 179)	480.08<br>(312.21 to 691.64)
Jiangsu	Headache disorders	293<br>(189 to 423)	425.73<br>(275.17 to 614.56)	388<br>(255 to 564)	474.92<br>(311.77 to 690.19)
Jiangxi	Headache disorders	150<br>(96.6 to 218)	378.91<br>(244.43 to 551.16)	212<br>(137 to 304)	445.47<br>(289.2 to 640.03)
Jilin	Headache disorders	106<br>(68 to 152)	409.53<br>(262.21 to 586.32)	137<br>(89.7 to 198)	489.9<br>(321.52 to 710.85)
Liaoning	Headache disorders	175<br>(113 to 254)	428.95<br>(277.37 to 623.77)	219<br>(144 to 317)	489.4<br>(321.6 to 709.84)
Ningxia	Headache disorders	18.2<br>(11.8 to 26.4)	369.17<br>(238.15 to 534.81)	31.7<br>(20.5 to 45.8)	451.69<br>(292.7 to 652.82)
Qinghai	Headache disorders	18<br>(11.7 to 26.2)	380.14<br>(246.97 to 552.78)	28.7<br>(18.8 to 41.6)	456.99<br>(298.84 to 661.62)
Shaanxi	Headache disorders	136<br>(87.2 to 196)	394.13<br>(253.39 to 569.64)	184<br>(120 to 268)	466.53<br>(304.18 to 677.33)
Shandong	Headache disorders	357<br>(233 to 513)	417.57<br>(272.7 to 599.53)	463<br>(302 to 668)	465.96<br>(303.77 to 672.09)
Shanghai	Headache disorders	71.5<br>(45.4 to 107)	511.43<br>(325.07 to 767.03)	156<br>(100 to 234)	559.61<br>(358.44 to 837.11)
Shanxi	Headache disorders	119<br>(76.8 to 171)	391.77<br>(253.62 to 566.07)	180<br>(117 to 261)	465.2<br>(302.41 to 675.89)
Sichuan	Headache disorders	465<br>(301 to 670)	416.6<br>(269.86 to 600.56)	386<br>(251 to 557)	461.81<br>(301.21 to 666.78)
Tianjin	Headache disorders	36.2<br>(23.4 to 52.5)	393.14<br>(254.56 to 570.8)	67.6<br>(44.4 to 98.5)	443.12<br>(291.12 to 645.66)
Tibet	Headache disorders	8.43<br>(5.48 to 12.1)	361.97<br>(235.3 to 520.51)	14.7<br>(9.56 to 21.4)	426.71<br>(276.71 to 619.18)
Xinjiang	Headache disorders	59.3<br>(37.9 to 85.3)	376.03<br>(240.32 to 540.72)	110<br>(71.4 to 159)	451.39<br>(293.17 to 652.53)
Yunnan	Headache disorders	147<br>(95 to 212)	378.32<br>(243.79 to 544.65)	221<br>(143 to 320)	450.48<br>(291.83 to 650.69)
Zhejiang	Headache disorders	182<br>(117 to 260)	412.13<br>(266.37 to 590.33)	291<br>(190 to 418)	475.07<br>(311.14 to 682.68)
China	Substance use disorders	7410<br>(5760 to 9150)	619.05<br>(480.92 to 764.51)	8370<br>(6340 to 10600)	592.32<br>(449.04 to 747.41)
Taiwan	Substance use disorders	98<br>(74.2 to 125)	480.11<br>(363.44 to 610.72)	151<br>(119 to 188)	640.56<br>(503.25 to 796.06)
Hong Kong Special Ad	Substance use disorders	30.5<br>(22.7 to 39.1)	529.52<br>(394.68 to 677.9)	34.2<br>(25.2 to 43.5)	455.51<br>(335.32 to 579.32)
Macau Special Admini	Substance use disorders	2.08<br>(1.59 to 2.61)	586.13<br>(449.12 to 735.32)	4.1<br>(3.07 to 5.22)	599.83<br>(449.11 to 763.8)
Anhui	Substance use disorders	292<br>(223 to 363)	493.45<br>(375.59 to 612.54)	313<br>(237 to 398)	511.11<br>(387.52 to 650.07)
Beijing	Substance use disorders	58.6<br>(42 to 75.8)	522.04<br>(374.76 to 675.74)	142<br>(102 to 186)	591.14<br>(422.89 to 771.15)
Chongqing	Substance use disorders	112<br>(88.9 to 137)	711.9<br>(563.24 to 870.95)	129<br>(95.3 to 163)	481.33<br>(356.6 to 610.23)
Fujian	Substance use disorders	153<br>(116 to 193)	483.55<br>(367.12 to 610.95)	223<br>(169 to 284)	566.7<br>(427.7 to 721.76)
Gansu	Substance use disorders	207<br>(165 to 252)	875.53<br>(697.68 to 1065.56)	171<br>(129 to 216)	643.67<br>(483.7 to 814.48)
Guangdong	Substance use disorders	403<br>(308 to 505)	611.89<br>(467.92 to 767.29)	777<br>(568 to 1000)	665.33<br>(486.27 to 855.9)
Guangxi	Substance use disorders	329<br>(263 to 402)	756.27<br>(605.47 to 924.85)	300<br>(233 to 371)	621.83<br>(482.57 to 768.58)
Guizhou	Substance use disorders	358<br>(287 to 437)	1077.59<br>(865.45 to 1318.04)	328<br>(246 to 412)	971.1<br>(726.92 to 1216.86)
Hainan	Substance use disorders	33.7<br>(25.6 to 42.3)	483.73<br>(367.7 to 607.85)	53.7<br>(40.4 to 67.6)	559.92<br>(421.15 to 704.79)
Hebei	Substance use disorders	354<br>(273 to 440)	556.85<br>(429.29 to 691.8)	447<br>(345 to 556)	584.81<br>(450.97 to 727.66)
Heilongjiang	Substance use disorders	178<br>(132 to 228)	487.15<br>(361.65 to 625.38)	204<br>(149 to 262)	520.56<br>(380.81 to 668.37)
Henan	Substance use disorders	427<br>(323 to 532)	483.72<br>(366.24 to 602.85)	528<br>(401 to 666)	548.65<br>(415.9 to 691.65)

Hubei	Substance use disorders	302 (230 to 379)	543.21 (413.91 to 681.6)	298 (227 to 374)	538.42 (410.01 to 675.76)
Hunan	Substance use disorders	476 (381 to 585)	765.59 (612.52 to 941.22)	404 (312 to 497)	601.27 (464.92 to 739.47)
Inner Mongolia	Substance use disorders	143 (111 to 179)	629.57 (485.95 to 787.36)	163 (122 to 209)	629.74 (469.81 to 807.97)
Jiangsu	Substance use disorders	343 (257 to 441)	498.34 (372.9 to 640.42)	423 (306 to 543)	518.32 (375.18 to 665.21)
Jiangxi	Substance use disorders	298 (237 to 363)	754.71 (598.63 to 918.54)	239 (177 to 305)	502.17 (372.75 to 642.42)
Jilin	Substance use disorders	143 (108 to 181)	550.09 (417.59 to 696.81)	150 (112 to 190)	536.43 (402.48 to 679.85)
Liaoning	Substance use disorders	240 (175 to 308)	588.53 (427.91 to 755.94)	244 (178 to 309)	546.28 (397.61 to 691.24)
Ningxia	Substance use disorders	25.3 (18.9 to 32.4)	513.18 (382.85 to 655.42)	39 (28.3 to 50)	555.84 (403.68 to 712.68)
Qinghai	Substance use disorders	33.2 (26.7 to 40.8)	701.76 (563.3 to 862.18)	46.7 (33.9 to 59.2)	743.44 (539.43 to 941.73)
Shaanxi	Substance use disorders	174 (135 to 219)	507.07 (390.91 to 637.49)	225 (169 to 286)	569.39 (428.79 to 723.77)
Shandong	Substance use disorders	433 (327 to 549)	506.38 (381.82 to 641.77)	522 (384 to 670)	524.92 (386.37 to 674.23)
Shanghai	Substance use disorders	67.7 (49.6 to 87.4)	484.47 (354.87 to 625.44)	154 (110 to 201)	551.24 (392.22 to 718.85)
Shanxi	Substance use disorders	180 (140 to 224)	594.17 (463.93 to 738.16)	205 (152 to 261)	529.85 (393.82 to 675.14)
Sichuan	Substance use disorders	755 (590 to 933)	676.46 (528.91 to 835.71)	479 (367 to 592)	573.49 (439.08 to 708.84)
Tianjin	Substance use disorders	45.5 (33.5 to 57.7)	494.92 (364.49 to 627.52)	90 (64.6 to 117)	589.62 (423.26 to 767.96)
Tibet	Substance use disorders	21.3 (17.2 to 25.9)	915.87 (737.26 to 1109.42)	22.1 (16.9 to 27.7)	638.61 (489.61 to 800.54)
Xinjiang	Substance use disorders	107 (86.5 to 132)	680.94 (548.33 to 834.05)	191 (151 to 237)	782.16 (619.68 to 971.22)
Yunnan	Substance use disorders	443 (364 to 533)	1137.13 (933.48 to 1367.83)	458 (341 to 564)	931.39 (692.73 to 1147.23)
Zhejiang	Substance use disorders	240 (178 to 305)	545.03 (403.92 to 692.07)	360 (263 to 463)	588.27 (429.05 to 756.87)
China	Diabetes and kidney disease	9560 (8320 to 11100)	798.42 (695.23 to 924.9)	15100 (12600 to 18100)	1068.9 (891.43 to 1281.19)
Taiwan	Diabetes and kidney disease	278 (243 to 320)	1360.56 (1191.82 to 1567.93)	500 (432 to 583)	2119.74 (1833.17 to 2472.65)
Hong Kong Special Ad	Diabetes and kidney disease	45.9 (38.7 to 53.1)	796.41 (671.5 to 920.55)	69.3 (56.7 to 85.1)	923.16 (755.53 to 1132.9)
Macau Special Admini	Diabetes and kidney disease	2.16 (1.84 to 2.54)	607.56 (518.62 to 714.28)	5.23 (4.12 to 6.6)	765.39 (601.98 to 965.38)
Anhui	Diabetes and kidney disease	422 (357 to 499)	712.47 (601.69 to 842.12)	623 (505 to 760)	1019.16 (826.29 to 1242.19)
Beijing	Diabetes and kidney disease	91.9 (76 to 112)	819.53 (677.51 to 998.29)	232 (179 to 298)	962.77 (741.89 to 1238.4)
Chongqing	Diabetes and kidney disease	129 (109 to 150)	815.85 (691.48 to 950.63)	283 (227 to 343)	1057.62 (850.45 to 1283.31)
Fujian	Diabetes and kidney disease	221 (189 to 259)	698.27 (596.52 to 818.31)	371 (303 to 458)	941.78 (767.71 to 1163.06)
Gansu	Diabetes and kidney disease	239 (210 to 275)	1011.43 (886.97 to 1164.38)	275 (230 to 333)	1036.13 (864.03 to 1253.74)
Guangdong	Diabetes and kidney disease	569 (480 to 683)	864.96 (728.94 to 1037.53)	1070 (847 to 1340)	919.95 (725.27 to 1145.73)
Guangxi	Diabetes and kidney disease	369 (318 to 427)	847.67 (731.14 to 981)	526 (441 to 628)	1088.86 (912.22 to 1299.81)
Guizhou	Diabetes and kidney disease	342 (294 to 392)	1029.49 (886.7 to 1182.27)	382 (316 to 452)	1129.63 (935.32 to 1337.05)
Hainan	Diabetes and kidney disease	63.7 (53.9 to 76)	914.41 (774.06 to 1090.48)	101 (79.3 to 125)	1052.48 (827.38 to 1301.53)
Hebei	Diabetes and kidney disease	455 (384 to 538)	714.68 (602.8 to 845.47)	894 (731 to 1080)	1170.07 (957.16 to 1419.3)
Heilongjiang	Diabetes and kidney disease	303 (258 to 360)	832.28 (707.39 to 987.79)	486 (391 to 593)	1238.41 (996.98 to 1511.3)

Henan	Diabetes and kidney disease	559<br>(471 to 660)	632.92<br>(533.29 to 748.03)	970<br>(805 to 1170)	1007.54<br>(836.25 to 1216)
Hubei	Diabetes and kidney disease	381<br>(330 to 440)	686.03<br>(593.88 to 792.74)	608<br>(504 to 723)	1098.58<br>(910.61 to 1307.68)
Hunan	Diabetes and kidney disease	693<br>(614 to 787)	1114.43<br>(987.19 to 1266.25)	965<br>(798 to 1130)	1436.93<br>(1188.89 to 1684.75)
Inner Mongolia	Diabetes and kidney disease	174<br>(147 to 206)	763.66<br>(644.96 to 907.36)	267<br>(214 to 333)	1032.44<br>(824.93 to 1286.47)
Jiangsu	Diabetes and kidney disease	487<br>(406 to 585)	707.81<br>(589.27 to 850.47)	881<br>(717 to 1070)	1079.02<br>(878.58 to 1312.31)
Jiangxi	Diabetes and kidney disease	421<br>(372 to 474)	1063.97<br>(941.64 to 1198.48)	449<br>(375 to 538)	945.36<br>(788.97 to 1131.88)
Jilin	Diabetes and kidney disease	246<br>(209 to 289)	948.74<br>(804.3 to 1113.5)	344<br>(276 to 420)	1232.62<br>(989.11 to 1505.23)
Liaoning	Diabetes and kidney disease	395<br>(331 to 474)	968.12<br>(811.06 to 1162.38)	669<br>(550 to 804)	1499.03<br>(1232.41 to 1801.44)
Ningxia	Diabetes and kidney disease	31.9<br>(27.5 to 37.1)	646.39<br>(557.52 to 751.25)	54<br>(43.7 to 66.4)	769.33<br>(623.06 to 946.33)
Qinghai	Diabetes and kidney disease	42.7<br>(37.3 to 48.3)	901.65<br>(788.12 to 1020.04)	65.6<br>(55.3 to 78.3)	1044.77<br>(880.25 to 1246.72)
Shaanxi	Diabetes and kidney disease	172<br>(145 to 203)	500.81<br>(421.58 to 588.57)	377<br>(310 to 461)	955.34<br>(784.05 to 1167.59)
Shandong	Diabetes and kidney disease	598<br>(501 to 710)	698.73<br>(585.85 to 829.32)	935<br>(749 to 1150)	940.37<br>(753.15 to 1161.94)
Shanghai	Diabetes and kidney disease	122<br>(102 to 147)	875.11<br>(729.69 to 1048.97)	278<br>(221 to 348)	993.23<br>(791.79 to 1245.29)
Shanxi	Diabetes and kidney disease	241<br>(202 to 289)	797.29<br>(666.9 to 953.59)	380<br>(301 to 474)	984.79<br>(778.77 to 1226.82)
Sichuan	Diabetes and kidney disease	739<br>(626 to 877)	662.44<br>(560.82 to 785.48)	937<br>(773 to 1120)	1122.4<br>(925.32 to 1336.14)
Tianjin	Diabetes and kidney disease	87.6<br>(72.1 to 106)	952.32<br>(783.71 to 1156.2)	190<br>(150 to 237)	1243.67<br>(982.76 to 1550.51)
Tibet	Diabetes and kidney disease	32.2<br>(27.8 to 36.5)	1383.67<br>(1194.9 to 1568.52)	31.1<br>(26.3 to 36.7)	899.3<br>(760.72 to 1062.61)
Xinjiang	Diabetes and kidney disease	144<br>(124 to 167)	911.34<br>(787.15 to 1056.15)	314<br>(258 to 377)	1288.47<br>(1060.15 to 1547.88)
Yunnan	Diabetes and kidney disease	408<br>(360 to 466)	1045.88<br>(922.5 to 1195)	537<br>(452 to 630)	1092.46<br>(919.78 to 1280.48)
Zhejiang	Diabetes and kidney disease	330<br>(279 to 389)	747.59<br>(631.94 to 881.55)	522<br>(416 to 641)	853.63<br>(680.45 to 1047.37)
China	Bacterial skin diseases	182<br>(57.3 to 264)	15.21<br>(4.79 to 22.04)	86.1<br>(64.2 to 173)	6.1<br>(4.55 to 12.24)
Taiwan	Bacterial skin diseases	4.29<br>(2.54 to 6.53)	21.05<br>(12.43 to 32.01)	11.6<br>(6.26 to 21)	49.35<br>(26.56 to 88.98)
Hong Kong Special Ad	Bacterial skin diseases	0.288<br>(0.218 to 0.538)	4.99<br>(3.78 to 9.34)	0.517<br>(0.306 to 1.15)	6.88<br>(4.08 to 15.27)
Macau Special Admini	Bacterial skin diseases	0.029<br>(0.015 to 0.0365)	8.17<br>(4.23 to 10.3)	0.0535<br>(0.0279 to 0.0707)	7.82<br>(4.07 to 10.34)
Anhui	Bacterial skin diseases	13.4<br>(3.41 to 22.3)	22.55<br>(5.75 to 37.7)	3.24<br>(2.35 to 5.82)	5.3<br>(3.84 to 9.52)
Beijing	Bacterial skin diseases	1.01<br>(0.483 to 1.38)	9.04<br>(4.31 to 12.28)	1.08<br>(0.577 to 2.76)	4.49<br>(2.39 to 11.44)
Chongqing	Bacterial skin diseases	1.51<br>(0.842 to 2.03)	9.58<br>(5.34 to 12.89)	1.53<br>(0.822 to 3.64)	5.71<br>(3.08 to 13.63)
Fujian	Bacterial skin diseases	9.07<br>(1.03 to 16.5)	28.68<br>(3.27 to 52.23)	2.46<br>(1.39 to 3.25)	6.24<br>(3.53 to 8.25)
Gansu	Bacterial skin diseases	4.36<br>(1.36 to 6.84)	18.42<br>(5.75 to 28.92)	1.46<br>(0.93 to 3.08)	5.48<br>(3.5 to 11.59)
Guangdong	Bacterial skin diseases	9.58<br>(2.48 to 15.5)	14.55<br>(3.76 to 23.48)	6.58<br>(4.61 to 12.9)	5.63<br>(3.94 to 11.05)
Guangxi	Bacterial skin diseases	6.77<br>(1.7 to 11)	15.57<br>(3.91 to 25.37)	3.03<br>(2.18 to 5.65)	6.26<br>(4.51 to 11.7)
Guizhou	Bacterial skin diseases	10.2<br>(1.52 to 18)	30.59<br>(4.58 to 54.19)	3.48<br>(1.45 to 4.89)	10.3<br>(4.29 to 14.47)
Hainan	Bacterial skin diseases	0.956<br>(0.254 to 1.57)	13.72<br>(3.65 to 22.52)	1.07<br>(0.436 to 1.5)	11.21<br>(4.55 to 15.69)
Hebei	Bacterial skin diseases	6.19<br>(2.58 to 9.02)	9.73<br>(4.05 to 14.17)	6.07<br>(3.77 to 9.75)	7.94<br>(4.94 to 12.75)

Heilongjiang	Bacterial skin diseases	1.89 (1.2 to 4.09)	5.19 (3.3 to 11.22)	2.22 (1.02 to 6.18)	5.66 (2.59 to 15.75)
Henan	Bacterial skin diseases	10.1 (4.47 to 14.3)	11.41 (5.06 to 16.25)	4.96 (2.72 to 11.8)	5.15 (2.82 to 12.3)
Hubei	Bacterial skin diseases	6.47 (2.84 to 9.24)	11.65 (5.11 to 16.63)	3.34 (1.96 to 7.82)	6.04 (3.54 to 14.15)
Hunan	Bacterial skin diseases	7.22 (2.99 to 10.2)	11.61 (4.81 to 16.38)	4.85 (3.44 to 8.94)	7.23 (5.12 to 13.32)
Inner Mongolia	Bacterial skin diseases	2.31 (1.16 to 3.19)	10.14 (5.08 to 14.03)	1.33 (0.704 to 3.4)	5.14 (2.72 to 13.13)
Jiangsu	Bacterial skin diseases	9.96 (2.56 to 16.1)	14.47 (3.72 to 23.37)	4.36 (2.97 to 8.63)	5.34 (3.64 to 10.57)
Jiangxi	Bacterial skin diseases	10.4 (1.76 to 18.2)	26.33 (4.45 to 46.05)	2.78 (2.11 to 4.96)	5.84 (4.44 to 10.43)
Jilin	Bacterial skin diseases	2.07 (1.27 to 2.76)	7.97 (4.88 to 10.63)	1.57 (0.92 to 3.57)	5.64 (3.3 to 12.8)
Liaoning	Bacterial skin diseases	2.45 (1.83 to 4)	6.02 (4.47 to 9.8)	2.75 (1.4 to 7.06)	6.15 (3.14 to 15.82)
Ningxia	Bacterial skin diseases	0.823 (0.239 to 1.38)	16.67 (4.84 to 27.98)	0.307 (0.183 to 0.667)	4.38 (2.61 to 9.5)
Qinghai	Bacterial skin diseases	1.24 (0.274 to 2.16)	26.1 (5.78 to 45.51)	0.401 (0.292 to 0.745)	6.38 (4.65 to 11.86)
Shaanxi	Bacterial skin diseases	4.43 (1.65 to 6.85)	12.88 (4.81 to 19.91)	2.04 (1.28 to 4.5)	5.17 (3.25 to 11.4)
Shandong	Bacterial skin diseases	7.98 (3.95 to 10.6)	9.32 (4.61 to 12.43)	5.38 (2.76 to 13.7)	5.41 (2.77 to 13.83)
Shanghai	Bacterial skin diseases	1.9 (0.521 to 2.95)	13.58 (3.73 to 21.14)	1.42 (0.96 to 2.91)	5.08 (3.44 to 10.42)
Shanxi	Bacterial skin diseases	3.17 (1.47 to 4.44)	10.46 (4.85 to 14.65)	1.81 (0.994 to 4.34)	4.67 (2.57 to 11.23)
Sichuan	Bacterial skin diseases	16.7 (5.23 to 26.8)	14.97 (4.69 to 24)	5.1 (3.36 to 10.7)	6.11 (4.03 to 12.86)
Tianjin	Bacterial skin diseases	0.667 (0.447 to 0.998)	7.25 (4.86 to 10.85)	0.774 (0.351 to 2.22)	5.07 (2.3 to 14.53)
Tibet	Bacterial skin diseases	0.547 (0.0795 to 0.973)	23.46 (3.41 to 41.74)	0.195 (0.134 to 0.291)	5.65 (3.88 to 8.41)
Xinjiang	Bacterial skin diseases	2.79 (0.832 to 4.5)	17.71 (5.27 to 28.56)	2.53 (1.34 to 3.6)	10.37 (5.5 to 14.78)
Yunnan	Bacterial skin diseases	15 (1.44 to 27.8)	38.39 (3.7 to 71.4)	3.77 (2.13 to 5.31)	7.67 (4.33 to 10.8)
Zhejiang	Bacterial skin diseases	10.7 (1.44 to 18.8)	24.16 (3.27 to 42.74)	3.68 (2.5 to 5.26)	6.01 (4.09 to 8.6)
China	Blindness and vision impair	3150 (2060 to 4730)	263.32 (171.72 to 395.06)	5510 (3650 to 8250)	390.21 (258.17 to 583.98)
Taiwan	Blindness and vision impair	33.7 (19.9 to 56)	165.24 (97.43 to 274.43)	55.1 (31.6 to 91.1)	233.69 (134.11 to 386.4)
Hong Kong Special Ad	Blindness and vision impair	16.5 (10.9 to 24.6)	285.49 (189 to 427.29)	32 (21.2 to 47.1)	425.45 (282.15 to 627.41)
Macau Special Admini	Blindness and vision impair	0.859 (0.564 to 1.28)	242.01 (158.89 to 360.58)	2.17 (1.42 to 3.26)	316.79 (206.94 to 476.57)
Anhui	Blindness and vision impair	156 (102 to 234)	263.58 (171.31 to 395.39)	255 (171 to 379)	417.21 (279.05 to 620.58)
Beijing	Blindness and vision impair	25.6 (16.5 to 39.3)	228.24 (147.53 to 350.8)	71.2 (46.4 to 109)	295.49 (192.38 to 452.11)
Chongqing	Blindness and vision impair	50.4 (33.1 to 75.3)	319.14 (209.95 to 477.39)	137 (92 to 201)	513.19 (344.15 to 751.59)
Fujian	Blindness and vision impair	69.8 (44.7 to 106)	220.61 (141.42 to 334.78)	129 (85.2 to 195)	328.28 (216.17 to 493.92)
Gansu	Blindness and vision impair	57.7 (37.4 to 87.9)	244.05 (158.22 to 371.64)	100 (65.5 to 150)	377.54 (246.44 to 564.07)
Guangdong	Blindness and vision impair	175 (115 to 263)	266.62 (175.33 to 399.72)	403 (263 to 607)	344.85 (225.19 to 519.23)
Guangxi	Blindness and vision impair	120 (78.8 to 177)	275.59 (181.1 to 407.73)	190 (128 to 279)	392.59 (265.29 to 577.41)
Guizhou	Blindness and vision impair	80.1 (52.1 to 121)	241.2 (156.94 to 363.19)	126 (82.8 to 189)	371.12 (244.77 to 558.37)
Hainan	Blindness and vision impair	21.3 (14.1 to 30.8)	305.7 (201.93 to 442.78)	39.7 (26.8 to 57.5)	413.96 (279.09 to 599.98)

Hebei	Blindness and vision impair	159<br>(102 to 241)	249.06<br>(161.03 to 377.99)	255<br>(169 to 387)	334.06<br>(220.81 to 505.86)
Heilongjiang	Blindness and vision impair	84.7<br>(55.3 to 127)	232.32<br>(151.71 to 348.35)	165<br>(109 to 245)	420.77<br>(276.87 to 623.26)
Henan	Blindness and vision impair	256<br>(170 to 384)	289.83<br>(192.46 to 434.5)	369<br>(244 to 547)	383<br>(253.86 to 567.81)
Hubei	Blindness and vision impair	208<br>(135 to 312)	373.68<br>(243.85 to 562.01)	331<br>(216 to 505)	598.47<br>(389.98 to 914)
Hunan	Blindness and vision impair	174<br>(116 to 259)	279.81<br>(185.91 to 416.82)	275<br>(182 to 405)	409.16<br>(270.67 to 603.7)
Inner Mongolia	Blindness and vision impair	49.4<br>(31.8 to 74.5)	217.22<br>(139.96 to 327.5)	91.7<br>(60.5 to 137)	354.06<br>(233.62 to 528.13)
Jiangsu	Blindness and vision impair	196<br>(128 to 295)	285.28<br>(185.84 to 428.04)	331<br>(221 to 494)	405.86<br>(270.29 to 604.56)
Jiangxi	Blindness and vision impair	98.1<br>(64.2 to 148)	248.19<br>(162.4 to 374.32)	165<br>(109 to 245)	346.22<br>(229.28 to 515.48)
Jilin	Blindness and vision impair	54.8<br>(36.4 to 80.5)	211.3<br>(140.4 to 310.1)	105<br>(70.9 to 153)	375.11<br>(254.03 to 549.67)
Liaoning	Blindness and vision impair	101<br>(65.5 to 150)	246.62<br>(160.51 to 366.7)	186<br>(124 to 276)	416.44<br>(277.62 to 619.01)
Ningxia	Blindness and vision impair	9.93<br>(6.44 to 15.1)	200.98<br>(130.37 to 304.94)	21.8<br>(14.4 to 32.9)	311.21<br>(205.84 to 468.4)
Qinghai	Blindness and vision impair	9.71<br>(6.22 to 14.7)	204.91<br>(131.26 to 310.07)	19<br>(12.4 to 28.8)	302.9<br>(196.87 to 457.84)
Shaanxi	Blindness and vision impair	83.3<br>(55.5 to 125)	242<br>(161.36 to 363.14)	147<br>(96.6 to 219)	370.82<br>(244.42 to 555.32)
Shandong	Blindness and vision impair	260<br>(162 to 408)	303.41<br>(188.86 to 477.13)	481<br>(303 to 745)	484.44<br>(304.8 to 750.03)
Shanghai	Blindness and vision impair	55.7<br>(34.5 to 88.8)	398.94<br>(247.04 to 635.38)	137<br>(84.5 to 217)	491.88<br>(302.55 to 775.64)
Shanxi	Blindness and vision impair	77.4<br>(50.3 to 116)	255.73<br>(166.15 to 381.98)	130<br>(85.1 to 195)	337.01<br>(220.2 to 504.06)
Sichuan	Blindness and vision impair	225<br>(141 to 352)	201.29<br>(126.08 to 315.37)	279<br>(180 to 434)	334.08<br>(215.89 to 519.44)
Tianjin	Blindness and vision impair	21.1<br>(13.6 to 32.1)	229.67<br>(147.87 to 349.19)	48.1<br>(31.7 to 72.1)	315.17<br>(207.5 to 472.7)
Tibet	Blindness and vision impair	7.28<br>(4.9 to 10.5)	312.3<br>(210.36 to 451.65)	11.4<br>(7.59 to 16.6)	329.63<br>(219.47 to 481.11)
Xinjiang	Blindness and vision impair	32.4<br>(20.9 to 49.3)	205.72<br>(132.28 to 312.69)	69.9<br>(45.4 to 106)	286.68<br>(186.37 to 435.27)
Yunnan	Blindness and vision impair	112<br>(75.1 to 165)	287.72<br>(192.72 to 422.51)	196<br>(130 to 288)	398.14<br>(264.94 to 586.41)
Zhejiang	Blindness and vision impair	105<br>(69.6 to 156)	237.91<br>(157.92 to 353.1)	213<br>(142 to 316)	347.69<br>(232.77 to 516.93)
China	Upper digestive system dise	3690<br>(2940 to 4670)	308.26<br>(245.75 to 390.14)	4100<br>(3040 to 5500)	290.54<br>(215.18 to 389.11)
Taiwan	Upper digestive system dise	47.9<br>(38.5 to 61.1)	234.67<br>(188.89 to 299.23)	54.6<br>(38.9 to 76.2)	231.56<br>(164.98 to 323.32)
Hong Kong Special Ad	Upper digestive system dise	12.6<br>(9.56 to 16.8)	217.82<br>(165.81 to 291.23)	18.4<br>(13.2 to 25.7)	245.17<br>(175.53 to 341.92)
Macau Special Admini	Upper digestive system dise	0.752<br>(0.573 to 0.988)	211.99<br>(161.5 to 278.46)	1.46<br>(1.01 to 2.11)	214.08<br>(147.5 to 308.43)
Anhui	Upper digestive system dise	141<br>(108 to 183)	238.13<br>(182.39 to 308.19)	154<br>(109 to 210)	251.28<br>(178.64 to 344.22)
Beijing	Upper digestive system dise	22.6<br>(16.8 to 30.7)	201.32<br>(149.54 to 273.47)	43.4<br>(29 to 63)	180.06<br>(120.48 to 261.37)
Chongqing	Upper digestive system dise	57.6<br>(45.9 to 72.6)	364.71<br>(290.76 to 459.85)	99.5<br>(74.6 to 133)	372.45<br>(279.17 to 496.32)
Fujian	Upper digestive system dise	69.5<br>(54.2 to 90.7)	219.56<br>(171.41 to 286.6)	84.8<br>(60.7 to 120)	215.25<br>(153.99 to 305.5)
Gansu	Upper digestive system dise	102<br>(81 to 125)	432<br>(342.48 to 529.07)	96.6<br>(73.9 to 126)	363.6<br>(278.23 to 472.62)
Guangdong	Upper digestive system dise	204<br>(161 to 255)	309.47<br>(244.89 to 387.02)	278<br>(199 to 385)	237.69<br>(170.12 to 329.14)
Guangxi	Upper digestive system dise	190<br>(136 to 235)	435.99<br>(312.63 to 541.05)	178<br>(138 to 232)	368.67<br>(285.29 to 479.81)
Guizhou	Upper digestive system dise	235<br>(158 to 288)	708.59<br>(476.45 to 867.97)	207<br>(155 to 263)	611.6<br>(458.37 to 777.7)

Hainan	Upper digestive system dise	19.9<br>(15.7 to 25.5)	285.99<br>(225.08 to 365.47)	21.3<br>(15.7 to 28.8)	221.9<br>(164.2 to 300.57)
Hebei	Upper digestive system dise	155<br>(120 to 203)	243.46<br>(187.85 to 319.56)	177<br>(128 to 247)	232.25<br>(166.96 to 323.37)
Heilongjiang	Upper digestive system dise	69.5<br>(51.5 to 92.7)	190.79<br>(141.21 to 254.22)	87.9<br>(62.1 to 122)	224.02<br>(158.3 to 311.49)
Henan	Upper digestive system dise	206<br>(160 to 271)	233.52<br>(181.72 to 306.52)	220<br>(158 to 304)	228.49<br>(164.56 to 315.19)
Hubei	Upper digestive system dise	181<br>(138 to 241)	326.34<br>(248.27 to 433.1)	234<br>(167 to 323)	423.43<br>(301.78 to 584.5)
Hunan	Upper digestive system dise	240<br>(194 to 293)	385.19<br>(312.23 to 470.67)	252<br>(192 to 329)	376.01<br>(285.36 to 490.54)
Inner Mongolia	Upper digestive system dise	57<br>(43.8 to 73.6)	250.52<br>(192.48 to 323.58)	67.6<br>(48 to 93.9)	260.95<br>(185.4 to 362.48)
Jiangsu	Upper digestive system dise	164<br>(128 to 215)	238.87<br>(185.82 to 311.82)	184<br>(131 to 253)	224.85<br>(160.91 to 309.83)
Jiangxi	Upper digestive system dise	136<br>(110 to 168)	343.82<br>(279.13 to 424.23)	134<br>(98.8 to 179)	281.23<br>(207.73 to 376.84)
Jilin	Upper digestive system dise	61<br>(47.4 to 78.8)	235.17<br>(182.7 to 303.84)	69.3<br>(49.6 to 96.2)	248.55<br>(178.01 to 344.89)
Liaoning	Upper digestive system dise	90.7<br>(67 to 123)	222.38<br>(164.26 to 302.11)	116<br>(81.3 to 162)	259.67<br>(182.15 to 362.26)
Ningxia	Upper digestive system dise	10.8<br>(8.32 to 14.1)	218.68<br>(168.47 to 285.63)	14.9<br>(10.5 to 20.8)	211.63<br>(149.25 to 295.98)
Qinghai	Upper digestive system dise	22.2<br>(15.7 to 26.9)	468.49<br>(331.69 to 567.1)	24.9<br>(19.2 to 31.7)	395.98<br>(305.91 to 504.33)
Shaanxi	Upper digestive system dise	72.2<br>(54.7 to 96.2)	209.85<br>(158.9 to 279.48)	95.9<br>(71.5 to 130)	242.81<br>(180.89 to 329.66)
Shandong	Upper digestive system dise	222<br>(172 to 289)	259.38<br>(201.51 to 337.85)	250<br>(177 to 351)	251.58<br>(177.72 to 353.3)
Shanghai	Upper digestive system dise	29.4<br>(21.9 to 39.8)	210.66<br>(156.87 to 284.99)	52<br>(35.2 to 75.7)	186.02<br>(126.16 to 271.07)
Shanxi	Upper digestive system dise	95.8<br>(78.3 to 119)	316.27<br>(258.43 to 394.41)	92.1<br>(68.8 to 125)	238.38<br>(178.2 to 324.41)
Sichuan	Upper digestive system dise	380<br>(289 to 486)	340.38<br>(259.15 to 435.76)	324<br>(241 to 425)	387.49<br>(288.49 to 509.53)
Tianjin	Upper digestive system dise	19.2<br>(14.5 to 25.4)	208.73<br>(157.43 to 275.89)	29.1<br>(20.2 to 41.8)	190.78<br>(132.1 to 273.62)
Tibet	Upper digestive system dise	6.94<br>(5.18 to 8.8)	297.68<br>(222.3 to 377.78)	8.98<br>(6.46 to 11.9)	259.82<br>(187.03 to 344.06)
Xinjiang	Upper digestive system dise	42.7<br>(34.3 to 53.6)	270.77<br>(217.65 to 339.55)	57.6<br>(42.3 to 78.7)	236.32<br>(173.66 to 323.07)
Yunnan	Upper digestive system dise	246<br>(167 to 301)	632.44<br>(428.03 to 772.22)	259<br>(197 to 325)	526.51<br>(400.88 to 660.53)
Zhejiang	Upper digestive system dise	127<br>(97.8 to 171)	288.22<br>(221.68 to 387.3)	172<br>(119 to 250)	280.7<br>(193.67 to 407.79)

China Cause of Death data sources and data quality rating from 0 to 5 stars		
China ★★★	Citation	Data type
	Centre for Research on the Epidemiology of Disasters (CRED). EM-DAT: The OFDA/CRED International Disaster Database. Brussels, Belgium: Catholic University of Leuven	Estimate
	Centre for Research on the Epidemiology of Disasters (CRED). EM-DAT: The OFDA/CRED International Disaster Database. Brussels, Belgium: Catholic University of Leuven	Vital registration
	Centre for Research on the Epidemiology of Disasters (CRED). EM-DAT: The OFDA/CRED International Disaster Database. Brussels, Belgium: Catholic University of Leuven	Vital registration
	Centre for Research on the Epidemiology of Disasters (CRED). EM-DAT: The OFDA/CRED International Disaster Database. Brussels, Belgium: Catholic University of Leuven	Vital registration
	Centre for Research on the Epidemiology of Disasters (CRED). EM-DAT: The OFDA/CRED International Disaster Database. Brussels, Belgium: Catholic University of Leuven	Vital registration
	Centre for Research on the Epidemiology of Disasters (CRED). EM-DAT: The OFDA/CRED International Disaster Database. Brussels, Belgium: Catholic University of Leuven	Vital registration
	Centre for Research on the Epidemiology of Disasters (CRED). EM-DAT: The OFDA/CRED International Disaster Database. Brussels, Belgium: Catholic University of Leuven	Vital registration
	Centre for Research on the Epidemiology of Disasters (CRED). EM-DAT: The OFDA/CRED International Disaster Database. Brussels, Belgium: Catholic University of Leuven	Vital registration
	Centre for Research on the Epidemiology of Disasters (CRED). EM-DAT: The OFDA/CRED International Disaster Database. Brussels, Belgium: Catholic University of Leuven	Vital registration
	Centre for Research on the Epidemiology of Disasters (CRED). EM-DAT: The OFDA/CRED International Disaster Database. Brussels, Belgium: Catholic University of Leuven	Vital registration
	Centre for Research on the Epidemiology of Disasters (CRED). EM-DAT: The OFDA/CRED International Disaster Database. Brussels, Belgium: Catholic University of Leuven	Vital registration
	Centre for Research on the Epidemiology of Disasters (CRED). EM-DAT: The OFDA/CRED International Disaster Database. Brussels, Belgium: Catholic University of Leuven	Vital registration
	Centre for Research on the Epidemiology of Disasters (CRED). EM-DAT: The OFDA/CRED International Disaster Database. Brussels, Belgium: Catholic University of Leuven	Vital registration
	Centre for Research on the Epidemiology of Disasters (CRED). EM-DAT: The OFDA/CRED International Disaster Database. Brussels, Belgium: Catholic University of Leuven	Vital registration
	Centre for Research on the Epidemiology of Disasters (CRED). EM-DAT: The OFDA/CRED International Disaster Database. Brussels, Belgium: Catholic University of Leuven	Vital registration
	Centre for Research on the Epidemiology of Disasters (CRED). EM-DAT: The OFDA/CRED International Disaster Database. Brussels, Belgium: Catholic University of Leuven	Vital registration
	United Nations Children's Fund (UNICEF), World Health Organization (WHO). WHO and UNICEF Reported Disease Incidence Time Series. Geneva, Switzerland: World Health Organization (WHO)	Epi surveillance
	International Institute for Strategic Studies. International Institute for Strategic Studies Armed Conflict Database. London, United Kingdom: International Institute for Strategic Studies	Estimate
	International Institute for Strategic Studies. International Institute for Strategic Studies Armed Conflict Database. London, United Kingdom: International Institute for Strategic Studies	Vital registration
	International Institute for Strategic Studies. International Institute for Strategic Studies Armed Conflict Database. London, United Kingdom: International Institute for Strategic Studies	Vital registration
	Cao B, Song S-F, Bai L, Yin YD, Zhang Y-Y, Liu Y-M, Guo P, Wang C, Ren L-L, Wang J-W, Zhao F, Zhang J-Z, Gonzalez R. Viral and Mycoplasma pneumoniae community-acquired pneumonia and novel clinical outcome evaluation in ambulatory adult patients in China. Eur J Clin Microbiol Infect Dis. 2010; 29(11): 1443-8	Scientific literature
	Sung RYT, Cheng AFB, Chan RCK, Tam JS, Oppenheimer SJ. Epidemiology and Etiology of Pneumonia in Children in Hong Kong. Clin Infect Dis. 1993; 17(5): 894-6	Scientific literature
	Zhang Q, MacDonald NE, Guo Z. Vaccine preventable community-acquired pneumonia in hospitalized children in Northwest China. Pediatr Infect Dis J. 2011; 30(1): 7-10	Scientific literature
	United Nations Office on Drugs and Crime (UNODC). United Nations Office on Drugs and Crime Global Study on Homicide 2011. Vienna, Austria: United Nations Office on Drugs and Crime (UNODC), 2011	Report
	Chinese Center for Disease Control and Prevention (CCDC). China Notifiable Infectious Diseases 2004	Epi surveillance
	Huang W, Yu H, Wang F, Li G. Infant mortality among various nationalities in the middle part of Guizhou, China. Soc Sci Med. 1997; 45(7): 1031-40	Scientific literature
	Chinese Center for Disease Control and Prevention (CCDC). China Notifiable Infectious Diseases 2012	Epi surveillance
	Chinese Center for Disease Control and Prevention (CCDC). China Notifiable Infectious Diseases 2005	Epi surveillance
	Chinese Center for Disease Control and Prevention (CCDC). China Notifiable Infectious Diseases 2006	Epi surveillance
	Chinese Center for Disease Control and Prevention (CCDC). China Notifiable Infectious Diseases 2007	Epi surveillance
	Chinese Center for Disease Control and Prevention (CCDC). China Notifiable Infectious Diseases 2008	Epi surveillance
	Chinese Center for Disease Control and Prevention (CCDC). China Notifiable Infectious Diseases 2009	Epi surveillance
	Chinese Center for Disease Control and Prevention (CCDC). China Notifiable Infectious Diseases 2010	Epi surveillance
	Chinese Center for Disease Control and Prevention (CCDC). China Notifiable Infectious Diseases 2011	Epi surveillance
	National Central Cancer Registry (China). China Cancer Registry Annual Report 2003-2007. Beijing, China: Military Medical Sciences Press (China)	Disease registry

	Dai S, Shen Z, Zha Z, Leng R, Qin W, Wang C, Chen L, Tian M, Huang Z, Chen G, Cen H, Xue L, Wang J, Lu Y, Cao B, Ye D. Seroprevalence of HIV, syphilis, and hepatitis C virus in the general population of the Liangshan Prefecture, Sichuan Province, China. J Med Virol. 2012; 84(1): 1-5	Scientific literature
	Forman D, Bray F, Brewster DH, Gombe Mbalawa C, Kohler B, Piñeros M, Steliarova-Foucher E, Swaminathan R and Ferlay J, eds (2013). Cancer Incidence in Five Continents, Vol. X Summary Database (electronic version). Lyon, IARC. http://ci5.iarc.fr	Disease registry
	Hong Kong Cancer Registry 2003-2007 - CI5 as it appears in Forman D, Bray F, Brewster DH, Gombe Mbalawa C, Kohler B, Piñeros M, Steliarova-Foucher E, Swaminathan R and Ferlay J, eds (2013). Cancer Incidence in Five Continents, Vol. X Summary Database (electronic version). Lyon, IARC. http://ci5.iarc.fr	Disease registry
	Macao Cancer Registry 2003-2007 - CI5 as it appears in Forman D, Bray F, Brewster DH, Gombe Mbalawa C, Kohler B, Piñeros M, Steliarova-Foucher E, Swaminathan R and Ferlay J, eds (2013). Cancer Incidence in Five Continents, Vol. X Summary Database (electronic version). Lyon, IARC. http://ci5.iarc.fr	Disease registry
	China - Cixian Cancer Registry 2003-2007 - CI5 as it appears in Forman D, Bray F, Brewster DH, Gombe Mbalawa C, Kohler B, Piñeros M, Steliarova-Foucher E, Swaminathan R and Ferlay J, eds (2013). Cancer Incidence in Five Continents, Vol. X Summary Database (electronic version). Lyon, IARC. http://ci5.iarc.fr	Disease registry
	China - Nangang District Harbin City Cancer Registry 2003-2007 - CI5 as it appears in Forman D, Bray F, Brewster DH, Gombe Mbalawa C, Kohler B, Piñeros M, Steliarova-Foucher E, Swaminathan R and Ferlay J, eds (2013). Cancer Incidence in Five Continents, Vol. X Summary Database (electronic version). Lyon, IARC. http://ci5.iarc.fr	Disease registry
	China - Qidong County Cancer Registry 2003-2007 - CI5 as it appears in Forman D, Bray F, Brewster DH, Gombe Mbalawa C, Kohler B, Piñeros M, Steliarova-Foucher E, Swaminathan R and Ferlay J, eds (2013). Cancer Incidence in Five Continents, Vol. X Summary Database (electronic version). Lyon, IARC. http://ci5.iarc.fr	Disease registry
	China - Yangcheng County Cancer Registry 2003-2007 - CI5 as it appears in Forman D, Bray F, Brewster DH, Gombe Mbalawa C, Kohler B, Piñeros M, Steliarova-Foucher E, Swaminathan R and Ferlay J, eds (2013). Cancer Incidence in Five Continents, Vol. X Summary Database (electronic version). Lyon, IARC. http://ci5.iarc.fr	Disease registry
	China - Yanting County Cancer Registry 2003-2007 - CI5 as it appears in Forman D, Bray F, Brewster DH, Gombe Mbalawa C, Kohler B, Piñeros M, Steliarova-Foucher E, Swaminathan R and Ferlay J, eds (2013). Cancer Incidence in Five Continents, Vol. X Summary Database (electronic version). Lyon, IARC. http://ci5.iarc.fr	Disease registry
	China - Haining County Cancer Registry 2003-2007 - CI5 as it appears in Forman D, Bray F, Brewster DH, Gombe Mbalawa C, Kohler B, Piñeros M, Steliarova-Foucher E, Swaminathan R and Ferlay J, eds (2013). Cancer Incidence in Five Continents, Vol. X Summary Database (electronic version). Lyon, IARC. http://ci5.iarc.fr	Disease registry
	China - Jiashan Cancer Registry 2003-2007 - CI5 as it appears in Forman D, Bray F, Brewster DH, Gombe Mbalawa C, Kohler B, Piñeros M, Steliarova-Foucher E, Swaminathan R and Ferlay J, eds (2013). Cancer Incidence in Five Continents, Vol. X Summary Database (electronic version). Lyon, IARC. http://ci5.iarc.fr	Disease registry
	China - Jiaxing City Cancer Registry 2005-2007 - CI5 as it appears in Forman D, Bray F, Brewster DH, Gombe Mbalawa C, Kohler B, Piñeros M, Steliarova-Foucher E, Swaminathan R and Ferlay J, eds (2013). Cancer Incidence in Five Continents, Vol. X Summary Database (electronic version). Lyon, IARC. http://ci5.iarc.fr	Disease registry
	Prince M, Acosta D, Ferri CP, Guerra M, Huang Y, Llibre Rodriguez JJ, Salas A, Sosa AL, Williams JD, Dewey ME, Acosta I, Jotheeswaran AT, Liu Z. Dementia incidence and mortality in middle-income countries, and associations with indicators of cognitive reserve: a 10/66 Dementia Research Group population-based cohort study. Lancet. 2012; 380(9836): 50-8	Scientific literature
	Zhao Q, Zhou B, Ding D, Guo Q, Hong Z. Prevalence, mortality, and predictive factors on survival of dementia in Shanghai, China. Alzheimer Dis Assoc Disord. 2010; 24(2): 151-8	Scientific literature
	By Gao ZX, Xie B, Fang YS, Pan JX. The Prevalence of Dementia Among Elderly in Shanghai Area, China- A Survey in City, Town and Countryside. Hong Kong J Psychiatry. 1999; 9(1): 12-4	Scientific literature
	Li G, Shen YC, Chen CH, Zhao YW, Li SR, Lu M. An epidemiological survey of age-related dementia in an urban area of Beijing. Acta Psychiatr Scand. 1989; 79(6): 557-63	Scientific literature
	Li S, Yan F, Li G, Chen C, Zhang W, Liu J, Jia X, Shen Y. Is the dementia rate increasing in Beijing? Prevalence and incidence of dementia 10 years later in an urban elderly population. Acta Psychiatr Scand. 2007; 115(1): 73-9	Scientific literature
	Shen YC, Li G, Li YT, Chen CH, Li SR, Zhao YW, Zhang WX. Epidemiology of age-related dementia in China. Chin Med J (Engl). 1994; 107(1): 60-4	Scientific literature
	Zhang MY, Katzman R, Salmon D, Jin H, Cai GJ, Wang ZY, Qu GY, Grant I, Yu E, Levy P. The prevalence of dementia and Alzheimer's disease in Shanghai, China: impact of age, gender, and education. Ann Neurol. 1990; 27(4): 428-37	Scientific literature
	Wang W, Wu S, Cheng X, Dai H, Ross K, Du X, Yin W. Prevalence of Alzheimer's disease and other dementing disorders in an urban community of Beijing, China. Neuroepidemiology. 2000; 19(4): 194-200	Scientific literature
	National Central Cancer Registry (China). China Cancer Registry Incidence and Mortality 1990-2009	Disease registry
	Ministry of Health (China). China Maternal and Child Mortality Data 1996-2012 - China CDC	Vital registration
	Yongjun T, Samuelson J, Qingsheng D, Ali MM, Li X, Yanjian M, Xiaoqing C, Jun L, Jian C, Bi L. The prevalence of sexually transmitted and other lower reproductive tract infections among rural women in Sichuan Province, China. Southeast Asian J Trop Med Public Health. 2009; 40(5): 1038-47	Scientific literature
	He N, Detels R, Zhu J, Jiang Q, Chen Z, Fang Y, Zhang X, Wu M, Zhao Q. Characteristics and sexually transmitted diseases of male rural migrants in a metropolitan area of Eastern China. Sex Transm Dis. 2005; 32(5): 286-92	Scientific literature
	Wu Z, Rotheram-Borus MJ, Li L, Guan J, Detels R, Yin Y, Wu S, Liu Z, Lin C, Hsieh J, NIMH Collaborative HIV/STD Prevention Trial Group. Sexually transmitted diseases and risk behaviors among market vendors in China. Sex Transm Dis. 2007; 34(12): 1030-4	Scientific literature
	Chen P, Yu ES, Zhang M, Liu WT, Hill R, Katzman R. ADL dependence and medical conditions in Chinese older persons: a population-based survey in Shanghai, China. J Am Geriatr Soc. 1995; 43(4): 378-83	Scientific literature

	Shi YM. [Study on the prevalence of Parkinson's disease in Hongkou District, Shanghai]. Chin J Epidemiol. 1987; 8(4): 205-7	Scientific literature
	Chinese Center for Disease Control and Prevention (CCDC). China Notifiable Infectious Diseases 2004-2012	Epi surveillance
	Zhang S, Lu Z, Liu H, Xiao X, Zhao Z, Bao G, Han J, Jing T, Chen G. Incidence of Japanese encephalitis, visceral leishmaniasis and malaria before and after the Wenchuan earthquake, in China. Addict Behav Rep. 2013; 128(1): 85-9	Scientific literature
	Hon KL, Leung E, Hung E, Tang J, Chow CM, Leung TF, Cheung KL, Ng PC. Premorbid factors and outcome associated with respiratory virus infections in a pediatric intensive care unit. Pediatr Pulmonol. 2008; 43(3): 275-80	Scientific literature
	Sung RYT, Chan PKS, Tsen T, Li AM, Lam WY, Yeung ACM, Nelson EAS. Identification of viral and atypical bacterial pathogens in children hospitalized with acute respiratory infections in Hong Kong by multiplex PCR assays. J Med Virol. 2009; 81(1): 153-9	Scientific literature
	Wen H, Zhang Z, Huang J, Duan L, Wang Q. Mortality of dementia and its major subtypes in urban and rural communities of Beijing. Biomed Environ Sci. 2011; 24(5): 483-90	Scientific literature
	Feng XL, Zhu J, Zhang L, Song L, Hipgrave D, Guo S, Ronsmans C, Guo Y, Yang Q. Socio-economic disparities in maternal mortality in China between 1996 and 2006. BJOG. 2010; 117(12): 1527-36	Scientific literature
	Zhu L, Qin M, Du L, Jia W, Yang Q, Walker MC, Wen SW. Comparison of maternal mortality between migrating population and permanent residents in Shanghai, China, 1996-2005. BJOG. 2009; 116(3): 401-7	Scientific literature
	Li LL, Ma Y, Chen R, Liu ZM, Qin X et al. Prevalence of dementia among the elderly in rural community of Anhui. Chin J Dis Control Prev. 2011; 15(4): 292-4	Scientific literature
	Zhe, Tang, Chen, Meng, Donghui Qing. [Beijing urban and rural senile dementia prevalence study 1]. Chin J Gerontol. 2002; 22(04): 244-8	Scientific literature
	Zhenxin Zhang, Jing Wei, Xia Hong. Prevalence of dementia and major subtypes in urban and rural communities of Beijing. Chin J Neurol. 2001; 34(4): 199-203	Scientific literature
	Shen Y, Tang M. The epidemiologic survey of mild cognitive impairment and dementia among residents aged 65 years and over in the urban and the rural areas in Guangzhou [dissertation]. [Guangzhou, China]: Guangzhou Medical University; 2011. 72 p	Other publication
	Huang W, Yang X, Yang JY. Investigation on prevalence of dementia among elderly in urban communities of Guiyang city. Chin J Public Health. 2007; 23(8): 983-6	Scientific literature
	Kang M, Gao YM, Huo HQ. Prevalence of Alzheimer's disease and its related factors among elderly people in Hebei province. Chin J Public Health. 2011; 27(9): 1123-5	Scientific literature
	Li Keqing, Jiang Qinpu, Cui Lijun. Epidemiological Investigation of Alzheimer Disease and Rural Areas in Hebei Province. China J Health Psychol. 2008; 16(11): 1251-3	Scientific literature
	Xiu-Shan He. Analysis of the Status and Influence of Alzheimer's Disease in Rural Communities. J Jiujiang Univ Natural Sci. 2012; 1: 29-32	Scientific literature
	Wang Feng, Xu Pin, Zhu Sihui. Survey on the awareness rate and prevalence of Alzheimer's disease in the urban area of Zaozhuang City. Chin J Practical Nerv Dis. 2010; 13(23): 9-11	Scientific literature
	Qing-Hua Fan. Study and Analysis of Impact Factors and Health Service of Dementia in Shanxi Province [dissertation]. Shanxi, China: Shanxi Medical University; 2012. 115 p	Report
	Guo-ying Ye, Jian-er Chen, Kai-Zhong Shen. Dementia in people over 60 years old and related risk factors. Chin Rural Health Serv Adm. 2011; 31(2): 152	Scientific literature
	Guo-Chuan Zhang, Bao-Long Z, Suzhen X. [A survey of prevalence of senile dementia and Parkinson disease]. J Clin Psychol Med. 2001; 11(Pt 3): 143-5	Scientific literature
	Chen Y-D, Liu M-Y, Yu W-L, Li J-Q, Peng M, Dai Q, Liu X, Zhou Z-Q. Hepatitis C virus infections and genotypes in China. Hepatobiliary Pancreat Dis Int. 2002; 1(2): 194-201	Scientific literature
	Wang B-E, Ma W-M, Sulaiman A, Noer S, Sumoharjo S, Sumarsidi D, Tandon BN, Nakao K, Mishiro S, Miyakawa Y, Akahane Y, Suzuki H. Demographic, clinical, and virological characteristics of hepatocellular carcinoma in Asia: survey of 414 patients from four countries. J Med Virol. 2002; 67(3): 394-400	Scientific literature
	World Health Organization (WHO). WHO Tuberculosis Case Notifications. Geneva, Switzerland: World Health Organization (WHO)	Epi surveillance
	Du L, Qin M, Zhang L, Xu H, Zhu L. Trends in maternal mortality in resident vs. migrant women in Shanghai, China, 2000-2009: a register-based analysis. Reprod Health Matters. 2012; 20(39): 73-80	Scientific literature
	Liang J, Zhu J, Dai L, Li X, Li M, Wang Y. Maternal mortality in China, 1996-2005. Int J Gynaecol Obstet. 2010; 110(2): 93-6	Scientific literature
	You F, Huo K, Wang R, Xu D, Deng J, Wei Y, Shi F, Liu H, Cheng G, Zhang Z, Yang P, Sun T, Wang X, Jacobsson B, Zhu C. Maternal mortality in Henan Province, China: changes between 1996 and 2009. PLoS One. 2012; 7(10): e47153	Scientific literature
	Chinese Center for Disease Control and Prevention (CCDC). China Disease Surveillance Points 2004 - China CDC	Vital registration
	Chinese Center for Disease Control and Prevention (CCDC). China Disease Surveillance Points 2005 - China CDC	Vital registration
	Chinese Center for Disease Control and Prevention (CCDC). China Disease Surveillance Points 2006 - China CDC	Vital registration
	Chinese Center for Disease Control and Prevention (CCDC). China Disease Surveillance Points 2007 - China CDC	Vital registration
	Chinese Center for Disease Control and Prevention (CCDC). China Mortality Registration and Reporting System 2008	Vital registration
	Chinese Center for Disease Control and Prevention (CCDC). China Mortality Registration and Reporting System 2009	Vital registration
	Chinese Center for Disease Control and Prevention (CCDC). China Mortality Registration and Reporting System 2010	Vital registration
	Chinese Center for Disease Control and Prevention (CCDC). China Mortality Registration and Reporting System 2012	Vital registration
	Chinese Center for Disease Control and Prevention (CCDC). China Mortality Registration and Reporting System 2011	Vital registration
	Chinese Center for Disease Control and Prevention (CCDC). China Disease Surveillance Points 1991 - China CDC	Vital registration
	Chinese Center for Disease Control and Prevention (CCDC). China Disease Surveillance Points 1992 - China CDC	Vital registration

	Chinese Center for Disease Control and Prevention (CCDC). China Disease Surveillance Points 1993 - China CDC	Vital registration
	Chinese Center for Disease Control and Prevention (CCDC). China Disease Surveillance Points 1994 - China CDC	Vital registration
	Chinese Center for Disease Control and Prevention (CCDC). China Disease Surveillance Points 1995 - China CDC	Vital registration
	Chinese Center for Disease Control and Prevention (CCDC). China Disease Surveillance Points 1996 - China CDC	Vital registration
	Chinese Center for Disease Control and Prevention (CCDC). China Disease Surveillance Points 1997 - China CDC	Vital registration
	Chinese Center for Disease Control and Prevention (CCDC). China Disease Surveillance Points 1998 - China CDC	Vital registration
	Chinese Center for Disease Control and Prevention (CCDC). China Disease Surveillance Points 1999 - China CDC	Vital registration
	Chinese Center for Disease Control and Prevention (CCDC). China Disease Surveillance Points 2001 - China CDC	Vital registration
	Chinese Center for Disease Control and Prevention (CCDC). China Disease Surveillance Points 2002 - China CDC	Vital registration
	Chinese Center for Disease Control and Prevention (CCDC). China Disease Surveillance Points 2000 - China CDC	Vital registration
	Hong Kong Cancer Registry. Hong Kong Cancer Registry Annual Tables. Kowloon, Hong Kong: Hong Kong Cancer Registry	Disease registry
	Hong Kong Cancer Registry 1998-2002 - CI5 as it appears in Curado MP, Edwards B, Shin HR, Storm H, Ferlay J, Heanue M and Boyle P, eds (2007). Cancer Incidence in Five Continents, Vol. IX Periodic Data (electronic version). Lyon, IARC. http://ci5.iarc.fr	Disease registry
	China - Nangang District Harbin City Cancer Registry 1998-2002 - CI5 as it appears in Curado MP, Edwards B, Shin HR, Storm H, Ferlay J, Heanue M and Boyle P, eds (2007). Cancer Incidence in Five Continents, Vol. IX Periodic Data (electronic version). Lyon, IARC. http://ci5.iarc.fr	Disease registry
	China - Zhongshan Cancer Registry 1998-2002 - CI5 as it appears in Curado MP, Edwards B, Shin HR, Storm H, Ferlay J, Heanue M and Boyle P, eds (2007). Cancer Incidence in Five Continents, Vol. IX Periodic Data (electronic version). Lyon, IARC. http://ci5.iarc.fr	Disease registry
	China - Jiashan Cancer Registry 1998-2002 - CI5 as it appears in Curado MP, Edwards B, Shin HR, Storm H, Ferlay J, Heanue M and Boyle P, eds (2007). Cancer Incidence in Five Continents, Vol. IX Periodic Data (electronic version). Lyon, IARC. http://ci5.iarc.fr	Disease registry
	China - Beijing Cancer Registry 1993-1997 - CI5 as it appears in Parkin DM, International Agency for Research on Cancer, International Association of Cancer Registries. Cancer Incidence in Five Continents. Vol. I to VIII. Lyon, France, IARCPress, 2005	Disease registry
	Hong Kong Cancer Registry 1978-1982 - CI5 as it appears in Parkin DM, International Agency for Research on Cancer, International Association of Cancer Registries. Cancer Incidence in Five Continents. Vol. I to VIII. Lyon, France, IARCPress, 2005	Disease registry
	Hong Kong Cancer Registry 1983-1987 - CI5 as it appears in Parkin DM, International Agency for Research on Cancer, International Association of Cancer Registries. Cancer Incidence in Five Continents. Vol. I to VIII. Lyon, France, IARCPress, 2005	Disease registry
	Hong Kong Cancer Registry 1974-1977 - CI5 as it appears in Parkin DM, International Agency for Research on Cancer, International Association of Cancer Registries. Cancer Incidence in Five Continents. Vol. I to VIII. Lyon, France, IARCPress, 2005	Disease registry
	Hong Kong Cancer Registry 1988-1992 - CI5 as it appears in Parkin DM, International Agency for Research on Cancer, International Association of Cancer Registries. Cancer Incidence in Five Continents. Vol. I to VIII. Lyon, France, IARCPress, 2005	Disease registry
	Hong Kong Cancer Registry 1993-1997 - CI5 as it appears in Parkin DM, International Agency for Research on Cancer, International Association of Cancer Registries. Cancer Incidence in Five Continents. Vol. I to VIII. Lyon, France, IARCPress, 2005	Disease registry
	China - Jiashan Cancer Registry 1993-1997 - CI5 as it appears in Parkin DM, International Agency for Research on Cancer, International Association of Cancer Registries. Cancer Incidence in Five Continents. Vol. I to VIII. Lyon, France, IARCPress, 2005	Disease registry
	China - Qidong County Cancer Registry 1983-1987 - CI5 as it appears in Parkin DM, International Agency for Research on Cancer, International Association of Cancer Registries. Cancer Incidence in Five Continents. Vol. I to VIII. Lyon, France, IARCPress, 2005	Disease registry
	China - Qidong County Cancer Registry 1988-1992 - CI5 as it appears in Parkin DM, International Agency for Research on Cancer, International Association of Cancer Registries. Cancer Incidence in Five Continents. Vol. I to VIII. Lyon, France, IARCPress, 2005	Disease registry
	China - Qidong County Cancer Registry 1993-1997 - CI5 as it appears in Parkin DM, International Agency for Research on Cancer, International Association of Cancer Registries. Cancer Incidence in Five Continents. Vol. I to VIII. Lyon, France, IARCPress, 2005	Disease registry
	China - Shanghai Cancer Registry 1978-1982 - CI5 as it appears in Parkin DM, International Agency for Research on Cancer, International Association of Cancer Registries. Cancer Incidence in Five Continents. Vol. I to VIII. Lyon, France, IARCPress, 2005	Disease registry
	China - Shanghai Cancer Registry 1975 - CI5 as it appears in Parkin DM, International Agency for Research on Cancer, International Association of Cancer Registries. Cancer Incidence in Five Continents. Vol. I to VIII. Lyon, France, IARCPress, 2005	Disease registry
	China - Shanghai Cancer Registry 1983-1987 - CI5 as it appears in Parkin DM, International Agency for Research on Cancer, International Association of Cancer Registries. Cancer Incidence in Five Continents. Vol. I to VIII. Lyon, France, IARCPress, 2005	Disease registry
	China - Tianjin Cancer Registry 1981-1982 - CI5 as it appears in Parkin DM, International Agency for Research on Cancer, International Association of Cancer Registries. Cancer Incidence in Five Continents. Vol. I to VIII. Lyon, France, IARCPress, 2005	Disease registry

	China - Tianjin Cancer Registry 1983-1987 - CI5 as it appears in Parkin DM, International Agency for Research on Cancer, International Association of Cancer Registries. Cancer Incidence in Five Continents. Vol. I to VIII. Lyon, France, IARCPress, 2005	Disease registry
	Ochiai RL, Wang X, von Seidlein L, Yang J, Bhutta ZA, Bhattacharya SK, Agtini M, Deen JL, Wain J, Kim DR, Ali M, Acosta CJ, Jodar L, Clemens JD. Salmonella Paratyphi A Rates, Asia. Emerg Infect Dis. 2005; 11(11): 1764-6	Scientific literature
	Ministry of Health (China). China National Maternal and Child Health Surveillance System Maternal Mortality By Cause 1996-2005 - MCHS	Demographic surveillar
	Ministry of Health (China). China National Maternal and Child Health Surveillance System Maternal Mortality By Cause 2006-2012 - MCHS	Demographic surveillar
	Ministry of Health (China). China National Maternal and Child Health Surveillance System Child Mortality Data By Cause 1996-2012 - MCHS	Demographic surveillar
	Bin Z, Zhen H, Mao-Sheng H. Prevalence of Parkinson's Disease in Shanghai urban and rural area. J Brain Nerv Dis. 2001; 9(6): 330-2	Scientific literature
	Jin Q, Qiu-Min Q, Jian-Feng H. The epidemiology of Parkinson Disease among Elderly people in Xi'an, China. Chin J Neuroimmunol Neurol. 2001; 2: 79-83	Scientific literature
	Jian-Long Z, Yu-Ling W, Ya-Ni Y. Analysis of prevalence and related factors in different national Parkinson's disease in Yili of Xinjiang area. J Xinjiang Med Univ. 2013; 36(3): 278-281	Scientific literature
	Lu-Ning W, Tai-Fang Z, Yu-Ling W. Study on the prevalence and risk factors of the Parkinson's disease in residents aged 35 years or older in Urumqi city. J Xinjiang Med Univ. 2013; 3	Scientific literature
	Yan L, Xiao-Ying Z, Ying HE. Investigation on prevalence rate of Parkinson's disease in population aged 55 years old and above in Kashi, Xinjiang between 2008 and 2009. Chin J Neurol. 2010; 12(43): 863-5	Scientific literature
	Ochiai RL, Acosta CJ, Danovaro-Holliday MC, Baiqing D, Bhattacharya SK, Agtini MD, Bhutta ZA, Canh DG, Ali M, Shin S, Wain J, Page A-L, Albert MJ, Farrar J, Abu-Elyazeed R, Pang T, Galindo CM, von Seidlein L, Clemens JD, Domi Typhoid Study Group. A study of typhoid fever in five Asian countries: disease burden and implications for controls. Bull World Health Organ. 2008; 86(4): 260-8	Scientific literature
	Hong Kong Cancer Registry 1986 - CI5 as it appears in Ferlay J, Parkin DM, Curado MP, Bray F, Edwards B, Shin HR and Forman D. Cancer Incidence in Five Continents Time Trends, Volumes I to X and Volume XI: IARC CancerBase No. 9 [Internet]. Lyon, France: International Agency for Research on Cancer; 2010. Available from: http://ci5.iarc.fr	Disease registry
	Hong Kong Cancer Registry 1989 - CI5 as it appears in Ferlay J, Parkin DM, Curado MP, Bray F, Edwards B, Shin HR and Forman D. Cancer Incidence in Five Continents Time Trends, Volumes I to X and Volume XI: IARC CancerBase No. 9 [Internet]. Lyon, France: International Agency for Research on Cancer; 2010. Available from: http://ci5.iarc.fr	Disease registry
	Hong Kong Cancer Registry 1992 - CI5 as it appears in Ferlay J, Parkin DM, Curado MP, Bray F, Edwards B, Shin HR and Forman D. Cancer Incidence in Five Continents Time Trends, Volumes I to X and Volume XI: IARC CancerBase No. 9 [Internet]. Lyon, France: International Agency for Research on Cancer; 2010. Available from: http://ci5.iarc.fr	Disease registry
	Hong Kong Cancer Registry 1996 - CI5 as it appears in Ferlay J, Parkin DM, Curado MP, Bray F, Edwards B, Shin HR and Forman D. Cancer Incidence in Five Continents Time Trends, Volumes I to X and Volume XI: IARC CancerBase No. 9 [Internet]. Lyon, France: International Agency for Research on Cancer; 2010. Available from: http://ci5.iarc.fr	Disease registry
	Hong Kong Cancer Registry 1999 - CI5 as it appears in Ferlay J, Parkin DM, Curado MP, Bray F, Edwards B, Shin HR and Forman D. Cancer Incidence in Five Continents Time Trends, Volumes I to X and Volume XI: IARC CancerBase No. 9 [Internet]. Lyon, France: International Agency for Research on Cancer; 2010. Available from: http://ci5.iarc.fr	Disease registry
	Hong Kong Cancer Registry 2002 - CI5 as it appears in Ferlay J, Parkin DM, Curado MP, Bray F, Edwards B, Shin HR and Forman D. Cancer Incidence in Five Continents Time Trends, Volumes I to X and Volume XI: IARC CancerBase No. 9 [Internet]. Lyon, France: International Agency for Research on Cancer; 2010. Available from: http://ci5.iarc.fr	Disease registry
	Hong Kong Cancer Registry 1983 - CI5 as it appears in Ferlay J, Parkin DM, Curado MP, Bray F, Edwards B, Shin HR and Forman D. Cancer Incidence in Five Continents Time Trends, Volumes I to X and Volume XI: IARC CancerBase No. 9 [Internet]. Lyon, France: International Agency for Research on Cancer; 2010. Available from: http://ci5.iarc.fr	Disease registry
	Hong Kong Cancer Registry 1987 - CI5 as it appears in Ferlay J, Parkin DM, Curado MP, Bray F, Edwards B, Shin HR and Forman D. Cancer Incidence in Five Continents Time Trends, Volumes I to X and Volume XI: IARC CancerBase No. 9 [Internet]. Lyon, France: International Agency for Research on Cancer; 2010. Available from: http://ci5.iarc.fr	Disease registry
	Hong Kong Cancer Registry 1988 - CI5 as it appears in Ferlay J, Parkin DM, Curado MP, Bray F, Edwards B, Shin HR and Forman D. Cancer Incidence in Five Continents Time Trends, Volumes I to X and Volume XI: IARC CancerBase No. 9 [Internet]. Lyon, France: International Agency for Research on Cancer; 2010. Available from: http://ci5.iarc.fr	Disease registry
	Hong Kong Cancer Registry 1990 - CI5 as it appears in Ferlay J, Parkin DM, Curado MP, Bray F, Edwards B, Shin HR and Forman D. Cancer Incidence in Five Continents Time Trends, Volumes I to X and Volume XI: IARC CancerBase No. 9 [Internet]. Lyon, France: International Agency for Research on Cancer; 2010. Available from: http://ci5.iarc.fr	Disease registry
	Hong Kong Cancer Registry 1991 - CI5 as it appears in Ferlay J, Parkin DM, Curado MP, Bray F, Edwards B, Shin HR and Forman D. Cancer Incidence in Five Continents Time Trends, Volumes I to X and Volume XI: IARC CancerBase No. 9 [Internet]. Lyon, France: International Agency for Research on Cancer; 2010. Available from: http://ci5.iarc.fr	Disease registry
	Hong Kong Cancer Registry 1993 - CI5 as it appears in Ferlay J, Parkin DM, Curado MP, Bray F, Edwards B, Shin HR and Forman D. Cancer Incidence in Five Continents Time Trends, Volumes I to X and Volume XI: IARC CancerBase No. 9 [Internet]. Lyon, France: International Agency for Research on Cancer; 2010. Available from: http://ci5.iarc.fr	Disease registry
	Hong Kong Cancer Registry 1994 - CI5 as it appears in Ferlay J, Parkin DM, Curado MP, Bray F, Edwards B, Shin HR and Forman D. Cancer Incidence in Five Continents Time Trends, Volumes I to X and Volume XI: IARC CancerBase No. 9 [Internet]. Lyon, France: International Agency for Research on Cancer; 2010. Available from: http://ci5.iarc.fr	Disease registry

	Hong Kong Cancer Registry 1995 - CI5 as it appears in Ferlay J, Parkin DM, Curado MP, Bray F, Edwards B, Shin HR and Forman D. Cancer Incidence in Five Continents Time Trends, Volumes I to X and Volume XI: IARC CancerBase No. 9 [Internet]. Lyon, France: International Agency for Research on Cancer; 2010. Available from: http://ci5.iarc.fr	Disease registry
	Hong Kong Cancer Registry 1997 - CI5 as it appears in Ferlay J, Parkin DM, Curado MP, Bray F, Edwards B, Shin HR and Forman D. Cancer Incidence in Five Continents Time Trends, Volumes I to X and Volume XI: IARC CancerBase No. 9 [Internet]. Lyon, France: International Agency for Research on Cancer; 2010. Available from: http://ci5.iarc.fr	Disease registry
	Hong Kong Cancer Registry 1998 - CI5 as it appears in Ferlay J, Parkin DM, Curado MP, Bray F, Edwards B, Shin HR and Forman D. Cancer Incidence in Five Continents Time Trends, Volumes I to X and Volume XI: IARC CancerBase No. 9 [Internet]. Lyon, France: International Agency for Research on Cancer; 2010. Available from: http://ci5.iarc.fr	Disease registry
	Hong Kong Cancer Registry 2000 - CI5 as it appears in Ferlay J, Parkin DM, Curado MP, Bray F, Edwards B, Shin HR and Forman D. Cancer Incidence in Five Continents Time Trends, Volumes I to X and Volume XI: IARC CancerBase No. 9 [Internet]. Lyon, France: International Agency for Research on Cancer; 2010. Available from: http://ci5.iarc.fr	Disease registry
	Hong Kong Cancer Registry 2001 - CI5 as it appears in Ferlay J, Parkin DM, Curado MP, Bray F, Edwards B, Shin HR and Forman D. Cancer Incidence in Five Continents Time Trends, Volumes I to X and Volume XI: IARC CancerBase No. 9 [Internet]. Lyon, France: International Agency for Research on Cancer; 2010. Available from: http://ci5.iarc.fr	Disease registry
	China - Shanghai Cancer Registry 1988 - CI5 as it appears in Ferlay J, Parkin DM, Curado MP, Bray F, Edwards B, Shin HR and Forman D. Cancer Incidence in Five Continents Time Trends, Volumes I to X and Volume XI: IARC CancerBase No. 9 [Internet]. Lyon, France: International Agency for Research on Cancer; 2010. Available from: http://ci5.iarc.fr	Disease registry
	China - Shanghai Cancer Registry 1989 - CI5 as it appears in Ferlay J, Parkin DM, Curado MP, Bray F, Edwards B, Shin HR and Forman D. Cancer Incidence in Five Continents Time Trends, Volumes I to X and Volume XI: IARC CancerBase No. 9 [Internet]. Lyon, France: International Agency for Research on Cancer; 2010. Available from: http://ci5.iarc.fr	Disease registry
	Hong Kong Cancer Registry 1984 - CI5 as it appears in Ferlay J, Parkin DM, Curado MP, Bray F, Edwards B, Shin HR and Forman D. Cancer Incidence in Five Continents Time Trends, Volumes I to X and Volume XI: IARC CancerBase No. 9 [Internet]. Lyon, France: International Agency for Research on Cancer; 2010. Available from: http://ci5.iarc.fr	Disease registry
	Hong Kong Cancer Registry 1985 - CI5 as it appears in Ferlay J, Parkin DM, Curado MP, Bray F, Edwards B, Shin HR and Forman D. Cancer Incidence in Five Continents Time Trends, Volumes I to X and Volume XI: IARC CancerBase No. 9 [Internet]. Lyon, France: International Agency for Research on Cancer; 2010. Available from: http://ci5.iarc.fr	Disease registry
	Llibre Rodriguez JJ, Ferri CP, Acosta D, Guerra M, Huang Y, Jacob KS, Krishnamoorthy ES, Salas A, Sosa AL, Acosta I, Dewey ME, Gaona C, Jotheeswaran AT, Li S, Rodriguez D, Rodriguez G, Kumar PS, Valhuerdi A, Prince M, 10/66 Dementia Research Group. Prevalence of dementia in Latin America, India, and China: a population-based cross-sectional survey. Lancet. 2008; 372(9637): 464-74	Scientific literature
	Li G, Shen YC, Chen CH, Zhau YW, Li SR, Lu M. A three-year follow-up study of age-related dementia in an urban area of Beijing. Acta Psychiatr Scand. 1991; 83(2): 99-104	Scientific literature
	Lu G, Li J, Xie Z, Liu C, Guo L, Vernet G, Shen K, Wang J. Human metapneumovirus associated with community-acquired pneumonia in children in Beijing, China. J Med Virol. 2013; 85(1): 138-43	Scientific literature
	Jia J, Wang F, Wei C, Zhou A, Jia X, Li F, Tang M, Chu L, Zhou Y, Zhou C, Cui Y, Wang Q, Wang W, Yin P, Hu N, Zuo X, Song H, Qin W, Wu L, Li D, Jia L, Song J, Han Y, Xing Y, Yang P, Li Y, Qiao Y, Tang Y, Lv J, Dong X. The prevalence of dementia in urban and rural areas of China. Alzheimers Dement. 2014; 10(1): 1-9	Scientific literature
	Zhou DF, Wu CS, Qi H, Fan JH, Sun XD, Como P, Qiao YL, Zhang L, Kieburtz K. Prevalence of dementia in rural China: impact of age, gender and education. Acta Neurol Scand. 2006; 114(4): 273-80	Scientific literature
	Wang YS, Shi YM, Wu ZY, He YX, Zhang BZ. Parkinson's disease in China. Coordinational Group of Neuroepidemiology, PLA. Chin Med J (Engl). 1991; 104(11): 960-4	Scientific literature
	Li SC, Schoenberg BS, Wang CC, Cheng XM, Rui DY, Bolis CL, Schoenberg DG. A prevalence survey of Parkinson's disease and other movement disorders in the People's Republic of China. Arch Neurol. 1985; 42(7): 655-7	Scientific literature
	Dong C, Dai X, Liang J, Dong M, Meng J. Seroprevalence of Hepatitis E virus varies considerably among Chinese provinces. Hepat Mon. 2012; 12(6): 386-90	Scientific literature
	Lu W-P, Lin G-X, Shi S, Dong J-H. Simultaneously high prevalences of hepatitis B and C virus infections in a population in Putian County, China. J Clin Microbiol. 2012; 50(6): 2142-4	Scientific literature
	Li D, Long Y, Wang T, Xiao D, Zhang J, Guo Z, Wang B, Yan Y. Epidemiology of Hepatitis C Virus Infection in Highly Endemic HBV Areas in China. PLoS One. 2013; 8(1): e54815	Scientific literature
	Zhu L, Qin M, Du L, Xie RH, Wong T, Wen SW. Maternal and congenital syphilis in Shanghai, China, 2002 to 2006. Int J Infect Dis. 2010; Suppl. 3: e45-8	Scientific literature
	Liang X, Bi S, Yang W, Wang L, Cui G, Cui F, Zhang Y, Liu J, Gong X, Chen Y, Wang F, Zheng H, Wang F, Guo J, Jia Z, Ma J, Wang H, Luo H, Li L, Jin S, Hadler SC, Wang Y. Reprint of: Epidemiological serosurvey of Hepatitis B in China--declining HBV prevalence due to Hepatitis B vaccination. Vaccine. 2013; J21-28	Scientific literature
	Aiqiang X, Zijian F, Wenbo X, Lixia W, Wanshen G, Qing X, Haijun S, Lee LA, Xiaofeng L. Active case-based surveillance for measles in China: lessons learned from Shandong and Henan provinces. J Infect Dis. 2003; 187(Suppl 1): S258-63	Scientific literature
	Yu X, Wang S, Guan J, Mahemuti, Purhati, Gou A, Liu Q, Jin X, Ghildyal R. Analysis of the cause of increased measles incidence in Xinjiang, China in 2004. Pediatr Infect Dis J. 2007; 26(6): 513-8	Scientific literature
	World Health Organization (WHO). Expanded programme on immunization. Measles outbreak. Wkly Epidemiol Rec. 1990; 65(49): 379-81	Epi surveillance
	Zhu Y, Yuan Z, Zhao Q, Chen G, Xu B. Seroprevalence of hepatitis A virus antibody in a population aged 0-30 years in Shanghai, China: implications for hepatitis A immunization. Epidemiol Infect. 2013; 141(3): 556-62	Scientific literature
	Chitsulo L, Engels D, Montresor A, Savioli L. The global status of schistosomiasis and its control. Addict Behav Rep. 2000; 77(1): 41-51	Scientific literature
	Sun YD. [A sero-epidemiological study of viral hepatitis A in a village]. Chin J Prev Med. 1985; 19(3): 140-2	Scientific literature

	Hu M, Schenzle D, Deinhardt F, Scheid R. Prevalence of markers of hepatitis A and B in the Shanghai area. J Infect Dis. 1983; 147(2): 360	Scientific literature
	Hu MD, Schenzle D, Deinhardt F, Scheid R. Epidemiology of hepatitis A and B in the Shanghai area: prevalence of serum markers. Am J Epidemiol. 1984; 120(3): 404-13	Scientific literature
	Xu DZ. [Epidemiologic study of viral hepatitis A in kindergartens and nurseries]. Nat Med J Chin. 1985; 65(3): 148-50	Scientific literature
	Wang HT. [An epidemiological study of viral hepatitis A in Beijing suburb]. Chin J Prev Med. 1985; 19(1): 8-10	Scientific literature
	Zou QH. [A seroepidemiological study of hepatitis A viral infection]. Chin J Epidemiol. 1984; 5(6): 329-31	Scientific literature
	Li L. [The relationship between changing of anti-HAV in population and prevalence of hepatitis A]. Chin J Prev Med. 1989; 23(3): 139-40	Scientific literature
	Song Y. [The investigation of anti-hepatitis A virus antibody rate in Guangzhou population]. Chin J Epidemiol. 1991; 12(5): 265-8	Scientific literature
	Geng J, Xu D, Gong J, Li W. Assessing hepatitis A virus epidemic stochastic process in eight cities in China in 1990. Int J Epidemiol. 1998; 27(2): 320-2	Scientific literature
	Li L, He J, Zhao L. [Epidemiologic features of viral hepatitis in Fujian]. Chin J Epidemiol. 1998; 19(2): 89-92	Scientific literature
	Hazell SL, Mitchell HM, Hedges M, Shi X, Hu PJ, Li YY, Lee A, Reiss-Levy E. Hepatitis A and evidence against the community dissemination of Helicobacter pylori via feces. J Infect Dis. 1994; 170(3): 686-9	Scientific literature
	Chen G, Lin W, Shen F, Iloeje UH, London WT, Evans AA. Chronic hepatitis B virus infection and mortality from non-liver causes: results from the Haimen City cohort study. Int J Epidemiol. 2005; 34(1): 132-7	Scientific literature
	Chen H-F, Li C-Y, Chen P, See T-T, Lee H-Y. Seroprevalence of hepatitis B and C in type 2 diabetic patients. J Chin Med Assoc. 2006; 69(4): 146-52	Scientific literature
	Clift A, Morgan C, Anderson D, Toole M. Alarming levels of hepatitis B virus detected among rural Tibetans. Trop Doct. 2004; 34(3): 156-7	Scientific literature
	Ito S, Yao DF, Nii C, Hibino S, Kamamura M, Nisikado T, Honda H, Shimizu I, Meng XY. Epidemiological characteristics of the incidence of hepatitis C virus (C100-3) antibodies in patients with liver diseases in the inshore area of the Yangtze River. J Gastroenterol Hepatol. 1993; 8(3): 232-7	Scientific literature
	Jiang Y, Lu J, Zhang L, Tian R, Liu Q, Bi S. [Antibody detection and sequencing analysis of hepatitis E virus in human population, swine and chicken in Sichuan region in China]. Chin J Exper Clin Virol. 2008; 22(6): 468-71	Scientific literature
	Liu P, Shi ZX, Zhang YC, Xu ZC, Shu HS, Zhang XY. A prospective study of a serum-pooling strategy in screening blood donors for antibody to hepatitis C virus. Transfusion. 1997; 37(7): 732-6	Scientific literature
	Lu J, Li H, Tian R, Jiang Y, Xu Y, Yu D, Jiang J, Bi S. [Hepatitis viruses infection situation in human population of the Gansu province]. Chin J Exper Clin Virol. 2009; 23(5): 349-51	Scientific literature
	Ma Z, Feng R, Zhao C, Harrison TJ, Li M, Qiao Z, Feng Y, Wang Y. Seroprevalence and distribution of hepatitis E virus in various ethnic groups in Gansu province, China. Infect Genet Evol. 2010; 10(5): 614-9	Scientific literature
	Seiji K, Inoue O, Liu SJ, Xu XP, Jin C, Cai SX, Nakatsuka H, Watanabe T, Uchida Y, Ikeda M. Prevalence of hepatitis B virus infection markers among factory workers in Beijing, China. Asia Pac J Public Health. 1991; 5(4): 345-9	Scientific literature
	Shimbo S, Zhang ZW, Qu JB, Wang JJ, Zhang CL, Song LH, Watanabe T, Higashikawa K, Ikeda M. Urban-rural comparison of HBV and HCV infection prevalence among adult women in Shandong Province, China. Southeast Asian J Trop Med Public Health. 1997; 28(3): 500-6	Scientific literature
	Taniguchi M, Kim SR, Mishiro S, Takahashi K, Shin MH, Yun H, Park MS, Li ZM, Kim MK, Fang J, Hayashi Y. Epidemiology of hepatitis E in Northeastern China, South Korea and Japan. J Infect. 2009; 58(3): 232-7	Scientific literature
	Tao QM, Wang Y, Wang H, Chen WR, Sun Y, Meng Q, Watanabe J, Nishioka K. Seroepidemiology of HCV and HBV infection in northern China. Gastroenterol Jpn. 1991; 156-8	Scientific literature
	Tian R-G, Lu J, Zhang B-C, Jiang Y-Z, Bi S-L. [Antibody detection of hepatitis E virus in some human population, swine and chicken in Beijing in China]. Chin J Exper Clin Virol. 2009; 23(1): 14-6	Scientific literature
	Tong D-Y, Wang X-H, Xu C-F, Yang Y-Z, Xiong S-D. Hepatitis B virus infection and coronary atherosclerosis: results from a population with relatively high prevalence of hepatitis B virus. World J Gastroenterol. 2005; 11(9): 1292-6	Scientific literature
	Wang Y, Tao QM, Zhao HY, Tsuda F, Nagayama R, Yamamoto K, Tanaka T, Tokita H, Okamoto H, Miyakawa Y. Hepatitis C virus RNA and antibodies among blood donors in Beijing. J Hepatol. 1994; 21(4): 634-40	Scientific literature
	Zhang M, Sun X-D, Mark SD, Chen W, Wong L, Dawsey SM, Qiao Y-L, Fraumeni JF Jr, Taylor PR, O'Brien TR. Hepatitis C virus infection, Linxian, China. Emerg Infect Dis. 2005; 11(1): 17-21	Scientific literature
	Zhang ZW, Shimbo S, Qu JB, Liu ZM, Cai XC, Wang LQ, Watanabe T, Nakatsuka H, Matsuda-Inoguchi N, Higashikawa K, Ikeda M. Hepatitis B and C virus infection among adult women in Jilin Province, China: an urban-rural comparison in prevalence of infection markers. Southeast Asian J Trop Med Public Health. 2000; 31(3): 530-6	Scientific literature
	Zhao SM, Jiang TL, Gao FX, Lu L, Zheng HQ, Hu J, Fan YH, Li B, Xiao RR, Yury K. Analysis of true voluntary blood donors with anti-HCV prevalence and implications for donor management in Chongqing, China. Transfus Med. 2007; 17(3): 210-1	Scientific literature
	Zhu G, Qu Y, Jin N, Sun Z, Liu T, Lee H, Tian M, Wang T. Seroepidemiology and molecular characterization of hepatitis E virus in Jilin, China. Infection. 2008; 36(2): 140-6	Scientific literature
	Zhuo J, Tao G, Ebrahim SH, Wang S, Luo Z, Wang H. The relationship of hepatitis B virus infection between adults and their children in Guangxi Province, China. J Hepatol. 2000; 33(4): 628-31	Scientific literature
	Chiu HFK, Lam LCW, Chi I, Leung T, Li SW, Law WT, Chung DWS, Fung HHL, Kan PS, Lum CM, Ng J, Lau J. Prevalence of dementia in Chinese elderly in Hong Kong. Neurology. 1998; 50(4): 1002-9	Scientific literature
	Malaria Atlas Project. Malaria Atlas Project Plasmodium Falciparum Parasite Rate Database. Oxford, United Kingdom: Malaria Atlas Project	Survey
	Malaria Atlas Project. Malaria Atlas Project Plasmodium Falciparum Parasite Rate Database. Oxford, United Kingdom: Malaria Atlas Project	Vital registration

	Malaria Atlas Project. Malaria Atlas Project Plasmodium Falciparum Parasite Rate Database. Oxford, United Kingdom: Malaria Atlas Project	Vital registration
	Zhang, T, Yang, Y, Yu, F, Zhao, Y, Lin, F, Minhas, V, Wood, C, He, N. Kaposi`s sarcoma associated herpesvirus infection among female sex workers and general population women in Shanghai, China: a cross-sectional study. BMC Infect Dis. 2014; 58	Scientific literature
	Yang, LG, Tucker, JD, Liu, FY, Ren, XQ, Hong, X, Wang, C, McLaughlin, MM, Bien, CH, Chen, XS, Yang, B. Syphilis screening among 27,150 pregnant women in South Chinese rural areas using point-of-care tests. PLoS One. 2013; 8(8): e72149	Scientific literature
	Cheng Q, Sun H-X, Ye F-L, Wang G, Ling H-W, Chen S-D, Jiang G-X. Dementia among elderly in Shanghai suburb: a rural community survey. J Alzheimers Dis. 2014; 39(4): 883–9	Scientific literature
	Cheng W, Wu Y, Wen Y, Ma Y, Zhao D, Dou Z, Zhang W, Bulterys M, Zhang F. Cotrimoxazole prophylaxis and antiretroviral therapy: an observational cohort study in China. Bull World Health Organ. 2015; 93(3): 152–60	Scientific literature
	Chen J, Yu B, Wang Y, Tang M, Hu Y, Cai T, Zhang F, Zinkernagel D von, Harwell JI, Huang ZJ. Expansion of HIV care and treatment in Yunnan Province, China: Treatment outcomes with scale up of combination antiretroviral therapy. AIDS Care. 2014; 26(5): 633–41	Scientific literature
	Zhu Q, Wang L, Lin W, Bulterys M, Yang W, Sun D, Cui Z, Kaplan J, Kleinman N, Wei X, Chung J, Wang Z. Improved survival with co-trimoxazole prophylaxis among people living with HIV/AIDS who initiated antiretroviral treatment in Henan Province, China. Curr HIV Res. 2014; 12(5): 359–65	Scientific literature
	Feng L, Lai S, Li F, Ye X, Li S, Ren X, Zhang H, Li Z, Yu H, Yang W. [Viral etiologies of hospitalized pneumonia patients aged less than five years in six provinces, 2009-2012]. Chin J Epidemiol. 2014; 35(6): 646–9	Scientific literature
	Fung KT, Fung J, Lai CL, Yuen MF. Etiologies of chronic liver diseases in Hong Kong. Eur J Gastroenterol Hepatol. 2007; 19(8): 659-64	Scientific literature
	Ji Y, Shi Z, Zhang Y, Liu S, Liu S, Yue W, Liu M, Huo YR, Wang J, Wisniewski T. Prevalence of dementia and main subtypes in rural northern China. Dement Geriatr Cogn Disord. 2015; 39(5-6): 294–302	Scientific literature
	Zhu H, Napravnik S, Eron JJ, Cole SR, Ma Y, Wohl DA, Dou Z, Zhang Y, Liu Z, Zhao D, Yu L, Liu X, Cohen MS, Zhang F. Decreasing excess mortality of HIV-infected patients initiating antiretroviral therapy: comparison with mortality in general population in China, 2003-2009. J Acquir Immune Defic Syndr. 2013; 63(5): e150-157	Scientific literature
	Liao L, Xing H, Su B, Wang Z, Ruan Y, Wang X, Liu Z, Lu Y, Yang S, Zhao Q, Vermund SH, Chen RY, Shao Y. Impact of HIV drug resistance on virologic and immunologic failure and mortality in a cohort of patients on antiretroviral therapy in China. AIDS. 2013; 27(11): 1815-24	Scientific literature
	Yang S, Zhang B, Zhao J, Wang J, Flick L, Qian Z, Zhang D, Mei H. Progress on the maternal mortality ratio reduction in Wuhan, China in 2001-2012. PLoS One. 2014; 9(2): e89510	Scientific literature
	Zhang T, Zhao W, Yang D, Piao D, Huang S, Mi Y, Zhao X, Cao J, Shen Y, Zhang W, Liu A. Human cystic echinococcosis in Heilongjiang Province, China: a retrospective study. BMC Gastroenterol. 2015; 15(1): 29	Scientific literature
	Chen XS, Yin YP, Chen LP, Thuy NT, Zhang GY, Shi MQ, Hu LH, Yu YH. Sexually transmitted infections among pregnant women attending an antenatal clinic in Fuzhou, China. Sex Transm Dis. 2006; 33(5): 296-301	Scientific literature
	Liu J, Huang Y, Wang J, Guo N, Li J, Dong X, Ma H, Tiemuer M, Huang M, Wright DJ, Ness, P, Shan, H. The increasing prevalence of serologic markers for syphilis among Chinese blood donors in 2008 through 2010 during a syphilis epidemic. Transfusion. 2012; 52(8): 1741-9	Scientific literature
	Zhang X, Liu L, Shi P, Jiang G, Jia P, Wang C, Wang L, Qian L. [Risk factors for acute respiratory syncytial virus infection of lower respiratory tract in hospitalized infants]. Chin J Pediatr. 2014; 52(5): 373-7	Scientific literature
	He Y, Lin GY, Wang Q, Cai XY, Zhang YH, Lin CX, Lu CD, Lu XD. A 3-year prospective study of the epidemiology of acute respiratory viral infections in hospitalized children in Shenzhen, China. Influenza Other Respir Viruses. 2014; 8(4): 443-51	Scientific literature
	Liu WK, Liu Q, Chen de H, Liang HX, Chen XK, Chen MX, Qiu SY, Yang ZY, Zhou R. Epidemiology of acute respiratory infections in children in Guangzhou: a three-year study. PLoS One. 2014; 9(5): e96674	Scientific literature
	Ju X, Fang Q, Zhang J, Xu A, Liang L, Ke C. Viral etiology of influenza-like illnesses in Huizhou, China, from 2011 to 2013. Arch Virol. 2014; 159(8): 2003-10	Scientific literature
	Zhao S, Li Z, Zhou S, Zheng C, Ma H. Epidemiological Feature of Visceral Leishmaniasis in China, 2004-2012. Iran J Public Health. 2015; 44(1): 51-9	Scientific literature
	Fu Q, Li SZ, Wu WP, Hou YY, Zhang S, Feng Y, Zhang LP, Tang LH. Endemic characteristics of infantile visceral leishmaniasis in the People's Republic of China. Parasites Vectors. 2013; 143	Scientific literature
	Cheng JQ, Zhou H, Hong FC, Zhang D, Zhang YJ, Pan P, Cai YM. Syphilis screening and intervention in 500,000 pregnant women in Shenzhen, the People's Republic of China. Sex Transm Infect. 2007; 83(5): 347–50	Scientific literature
	Xiao X, Zhou Y, Sun W, Chen D. [Prevalence of syphilis during pregnancy and risk factors for maternal and perinatal infections: a 2009-2013 survey]. J South Med Univ. 2014; 34(1): 144–6	Scientific literature
	Yang X, Yao Y, Chen M, Yang X, Xie Y, Liu Y, Zhao X, Gao Y, Wei L. Etiology and clinical characteristics of influenza-like illness (ILI) in outpatients in Beijing, June 2010 to May 2011. PLoS One. 2012; 7(1): e28786	Scientific literature
	Peng J, Kong W, Guo D, Liu M, Wang Y, Zhu H, Pang B, Miao X, Yu B, Luo T, Hu Q, Zhou D. The epidemiology and etiology of influenza-like illness in Chinese children from 2008 to 2010. J Med Virol. 2012; 84(4): 672-8	Scientific literature
	Yu X, Lu R, Wang Z, Zhu N, Wang W, Julian D, Chris B, Lu J, Lv J, Tan W. Etiology and clinical characterization of respiratory virus infections in adult patients attending an emergency department in Beijing. PLoS One. 2012; 7(2): e32174	Scientific literature
	Leung TF, Lam DS, Miu TY, Hon KL, Chau CS, Ku SW, Lee RS, Chow PY, Chiu WK, Ng DK. Epidemiology and risk factors for severe respiratory syncytial virus infections requiring pediatric intensive care admission in Hong Kong children. Infection. 2014; 42(2): 343-50	Scientific literature

	Zhang Q, Guo Z, Langley JM, Bai Z. Respiratory syncytial virus-associated intensive care unit admission in children in Southern China. BMC Res Notes. 2013; 447	Scientific literature
	Zhang C, Zhu N, Xie Z, Lu R, He B, Liu C, Ma X, Tan W. Viral etiology and clinical profiles of children with severe acute respiratory infections in China. PLoS One. 2013; 8(8): e72606	Scientific literature
	Liu YF, Gao Y, Chen MF, Cao B, Yang XH, Wei L. Etiological analysis and predictive diagnostic model building of community-acquired pneumonia in adult outpatients in Beijing, China. BMC Infect Dis. 2013; 309	Scientific literature
	Zhang PJ, Cao B, Li XL, Liang LR, Yang SG, Gu L, Xu Z, Hu K, Zhang HY, Yan XX, Huang WB, Chen W, Zhang JX, Li LJ, Wang C. Risk factors for adult death due to 2009 pandemic influenza A (H1N1) virus infection: a 2151 severe and critical cases analysis. Chin Med J (Engl). 2013; 126(12): 2222-8	Scientific literature
	Huang G, Yu D, Mao N, Zhu Z, Zhang H, Jiang Z, Li H, Zhang Y, Shi J, Zhang S, Wang X, Xu W. Viral etiology of acute respiratory infection in Gansu Province, China, 2011. PLoS One. 2013; 8(5): e64254	Scientific literature
	Li H, Wei Q, Tan A, Wang L. Epidemiological analysis of respiratory viral etiology for influenza-like illness during 2010 in Zhuhai, China. Virol J. 2013; 143	Scientific literature
	Bao Z, Yuan X, Wang L, Sun Y, Dong X. The incidence and etiology of community-acquired pneumonia in fever outpatients. Exp Biol Med (Maywood). 2012; 237(11): 1256-61	Scientific literature
	Huo X, Qin Y, Qi X, Zu R, Tang F, Li L, Hu Z, Zhu F. Surveillance of 16 respiratory viruses in patients with influenza-like illness in Nanjing, China. J Med Virol. 2012; 84(12): 1980-4	Scientific literature
	Guo RN, Zheng HZ, Huang LQ, Zhou Y, Zhang X, Liang CK, Lin JY, He JF, Zhang JQ. Epidemiologic and economic burden of influenza in the outpatient setting: a prospective study in a subtropical area of China. PLoS One. 2012; 7(7): e41403	Scientific literature
	Zhang Q, Guo Z, Bai Z, MacDonald NE. A 4 year prospective study to determine risk factors for severe community acquired pneumonia in children in southern China. Pediatr Pulmonol. 2013; 48(4): 390-7	Scientific literature
	National Central Cancer Registry (China). China Cancer Registry Incidence and Mortality 2011	Disease registry
	Chinese Center for Disease Control and Prevention (CCDC). China Disease Surveillance Points and Information System for Disease Control and Prevention Deaths 2013	Vital registration
	Chinese Center for Disease Control and Prevention (CCDC). China Disease Surveillance Points and Information System for Disease Control and Prevention Deaths 2014	Vital registration
	Department of Peace and Conflict Research, Uppsala University. UCDP/PRIO Armed Conflict Database Version 4, 2015 - UCDP. Uppsala, Sweden: Department of Peace and Conflict Research, Uppsala University, 2014	Estimate
	Ministry of Health (China). China National Maternal and Child Health Surveillance System Maternal Mortality By Cause 2013 - MCHS	Demographic surveillar
	Ministry of Health (China). China National Maternal and Child Health Surveillance System Child Mortality By Cause 2013 - MCHS	Demographic surveillar
	Department of Peace and Conflict Research, Uppsala University. UCDP One-Sided Violence Dataset, Version 1.4, 2015. Uppsala, Sweden: Department of Peace and Conflict Research, Uppsala University, 2015	Estimate
	Chen K, Jia R, Li L, Yang C, Shi Y. The aetiology of community associated pneumonia in children in Nanjing, China and aetiological patterns associated with age and season. BMC Public Health. 2015; 113	Scientific literature
	Qu J-X, Gu L, Pu Z-H, Yu X-M, Liu Y-M, Li R, Wang Y-M, Cao B, Wang C, Beijing Network for Adult Community-Acquired Pneumonia (BNACAP). Viral etiology of community-acquired pneumonia among adolescents and adults with mild or moderate severity and its relation to age and severity. BMC Infect Dis. 2015; 89	Scientific literature
	Feng L, Li Z, Zhao S, Nair H, Lai S, Xu W, Li M, Wu J, Ren L, Liu W, Yuan Z, Chen Y, Wang X, Zhao Z, Zhang H, Li F, Ye X, Li S, Feikin D, Yu H, Yang W. Viral etiologies of hospitalized acute lower respiratory infection patients in China, 2009-2013. PLoS One. 2014; 9(6): e99419	Scientific literature
	World Health Organization (WHO). WHO Mortality Database Version November 2015. Geneva, Switzerland: World Health Organization (WHO), 2015	Vital registration
	Hong Kong Vital Registration - Deaths 1994 ICD9 as it appears in World Health Organization (WHO). WHO Mortality Database Version November 2015. Geneva, Switzerland: World Health Organization (WHO), 2015	Vital registration
	Hong Kong Vital Registration - Deaths 1997 ICD9 as it appears in World Health Organization (WHO). WHO Mortality Database Version November 2015. Geneva, Switzerland: World Health Organization (WHO), 2015	Vital registration
	Macau Vital Registration - Deaths 1994 ICD9 as it appears in World Health Organization (WHO). WHO Mortality Database Version November 2015. Geneva, Switzerland: World Health Organization (WHO), 2015	Vital registration
	Chinese Center for Disease Control and Prevention (CCDC). China Notifiable Infectious Diseases 2013 - China CDC	Epi surveillance
	Chinese Center for Disease Control and Prevention (CCDC). China Notifiable Infectious Diseases 2014 - China CDC	Epi surveillance
	Chen Y, Zhang X-J, Zhao Y-L, Zhang Y-H, Wang S-M, Hao Z-Y, Zhang Z-Y, Ma J-C, Fang Y, Wang X-Y. Waning of anti-HAV immunity in Shijiazhuang prefecture, Hebei province, China: a comparison of seroprevalence between 1992 and 2011. Vaccine. 2014; 32(47): 6227–32	Scientific literature
	Agence France-Presse (AFP). China Landslide Leaves 91 Missing, Sparks Gas Explosion. AsiaOne [Internet]. 2015 Dec 21. Asia	Other publication
	Xia GL, Liu CB, Caoa HL, BP SL, Zhan MY, Sub CA, Nan JH, Qi XQ. Prevalence of hepatitis B and C virus infections in the general Chinese population: Results from a nationwide cross-sectional seroepidemiologic study of hepatitis A, B, C, D, and E virus infections in China, 1992. Int Hepatol Commun. 1996; 5: 62-73	Scientific literature
	Yang Y, Gao J, Li HL, Zheng W, Yang G, Zhang W, Ma X, Tan YT, Rothman N, Gao YT, Chow WH, Shu XO, Xiang YB. Dose-response association between hepatitis B surface antigen levels and liver cancer risk in Chinese men and women. Int J Cancer. 2016. [Epub ahead of print]	Scientific literature

	Ming L, Thorgeirsson SS, Gail MH, Lu P, Harris CC, Wang N, Shao Y, Wu Z, Liu G, Wang X, Sun Z. Dominant role of hepatitis B virus and cofactor role of aflatoxin in hepatocarcinogenesis in Qidong, China. Hepatology. 2002; 36(5): 1214-20	Scientific literature
	Zhang JY, Dai M, Wang X, Lu WQ, Li DS, Zhang MX, Wang KJ, Dai LP, Han SG, Zhou YF, Zhuang H. A case-control study of hepatitis B and C virus infection as risk factors for hepatocellular carcinoma in Henan, China. Int J Epidemiol. 1998; 27(4): 574-8	Scientific literature
	Gao JD, Shao YF, Xu Y, Ming LH, Wu ZY, Liu GT, Wang XH, Gao WH, Sun YT, Feng XL, Liang LM, Zhang YH, Sun ZT. Tight association of hepatocellular carcinoma with HBV infection in North China. Hepatobiliary Pancreat Dis Int. 2005; 4(1): 46-9	Scientific literature
	Liang X1, Bi S, Yang W, Wang L, Cui G, Cui F, Zhang Y, Liu J, Gong X, Chen Y, Wang F, Zheng H, Wang F, Guo J, Jia Z, Ma J, Wang H, Luo H, Li L, Jin S, Hadler SC, Wang Y. Epidemiological serosurvey of hepatitis B in China--declining HBV prevalence due to hepatitis B vaccination. Vaccine. 2009; 5(27): 6550-7	Scientific literature
	Wang ZJ, Zhou YP, Cheng B, Liang ZN, Peng WW. An epidemiologic study on the aetiological factors of primary liver cancer in Shunde City of Guangdong province. Chin J Epidemiol. 1996; 17(3): 141-4	Scientific literature
	Ding BG, Fan DM, Mu LN, Yu SZ. A case–control study of hepatitis B virus, hepatitis C virus and liver cancer. Chin J Epidemiol. 2004; 25(22)	Scientific literature
	Zeng JH, Dai YS, Cao MQ, Hu XK. Serological markers of HCV and HBV and primary hepatocellular carcinoma. Modern Prev Med. 2003; 30: 324-5	Scientific literature
	Wu JZ, Su MH, Chen MW, Luo GH, Liang RX, Wei ZL, Jiang JN, Huang LY. A matching case–control study on the relationship between HBV, HCV infection and HCC in endemic area of HCC in Guangxi, China. J Guangxi Med Univ. 2003; 20: 313-5	Scientific literature
	Wang ZF. A study of the relationship between the primary carcinoma of liver and hepatitis B, C and TTV. Henan Med Res. 2003; 12: 43-44	Scientific literature
	Su MH, Wu JZ, Luo GH, Huang LY, Chen MW. A case–control study of relationship in HBV, HCV infection and HCC in areas with low incidences in Guangxi. Clin Foc. 2002; 17: 209-10	Scientific literature
	Zhao JF, Yang P, Su W, Cui Z, Wang SM. Hepatitis C virus infection and hepatocellular carcinoma. J Bengbu Med Coll. 2000; 25: 169-170	Scientific literature
	Li XJ, Yang HX, Wang XQ, Tang NH, Xu CS. Detection of HBV and HCV in patients with primary liver cancer. J Fujian Med Univ. 2002; 36: 380-1	Scientific literature
	Li D, Cui LH, Jin DZ, Piao XX. Analysis of serum hepatitis B virus and hepatitis C virus infection markers in patients with primary carcinoma of liver. J Med Sci Yanbian Univ. 1999; 22: 185-8	Scientific literature
	Cui LH, Fang JN, Jin HZ, Quan ZY, Jin CJ. An analysis of the interaction of Hepatitis virus in primary hepatocellular carcinoma in Korean of Yanbian. Chin J Prev Control Chronic Dis. 1999; 7: 28-30	Scientific literature
	Gao XH, Zhang CL, Li XL, Mei H, Qi F, Zhu XY. Analysis of the infection hepatitis B and C from sera of the patients suffered from primary hepatoma. J Dalian Med Univ. 1998; 20: 19-21	Scientific literature
	Dai M, Shi Y, Zhang JY, Zhou YF, Zhang MX, He Z. A study of the interaction of HBV and HCV in PHC. Henan J Oncol. 1998; 11: 81-3	Scientific literature
	Zuo Q, Liu YJ, Liang WT. A case–control study of the synergistic action of HBV and HCV in the development of primary hepatocellular carcinoma. Chin J Public Health. 1997; 16: 173-275	Scientific literature
	Yu SZ, Zi XL, Chen G, Li J. The relationship between viral hepatitis and primary liver cancer in four areas of China. Chin J Epidemiol. 1997; 18: 214-6	Scientific literature
	Shen J, Xu YC, Gao Z, Niu JY, Shen HB, Ye BF. Epidemiological statocellulaudy on the etiologic synergistic interaction of HCV and HBV in the development of hepatocacinoma. Chin Natl J New Gastroenterol. 1997; 14: 72-74	Scientific literature
	Lu CH, Hou SJ. The relationship in HBV, HCV infection and cirrhosis and primary liver cancer. Clin Med. 1997; 17: 17-18	Scientific literature
	Yang JT, Zhao HG, Zhao SF, Li PZ. Prevalence of HCV and HBV infection in patients with primary hepatocellular carcinoma in Shanxi Province. Chin J Epidemiol. 1999; 20: 215-7	Scientific literature
	Wang SS, Jiang PL, Pang HX, Peng GF. An epidemiological study on the etiology of primary hepatocellular carcinoma in Guangzhou, Guangdong Province. Chin J Epidemiol. 1999; 18: 33-6	Scientific literature
	Gong J, Yang JY, Li RC, Wang SS, Li YP, Chen KL. Hepatitis C virus and primary liver cancer in a hyperendemic area of liver cancer. Chin J Public Health. 1993; 12: 328-330	Scientific literature
	Xu Z, Shen FM, Xu ZY, Huang QS. HCV infection and primary liver cell cancer. Tumor. 1990; 10: 115	Scientific literature
	Zhang ZQ, Huang TR, He ZF, Yu JH, Xu QF, Huang ZD. The relationship between HCV and HBV in the etiology of primary liver cancer. Guangxi Med J. 1993; 15: 171-4	Scientific literature
	Ye BF, Shen J, Xu YC, Niu JY, Chen JG, Zhang BC, Liu B, Jiang YH. Etiology study on the relationship between HBV, HCV and HCC. Chin J Epidemiol. 1994; 15: 131-4	Scientific literature
	Zhou YP, Peng WW, Yao JL, Lu L, Li GH, Huang JF, Peng BG, Chen Q. A case control study on relationship between hepatocellular carcinoma and infection of HCV and HCV. Acad J SUMS. 1994; 15: 45-49	Scientific literature
	Fan XL, Peng WW, Yao JL, Zhou YP, Lu L, Zhen XC, Chen Q. Infection of HBV, HCV and HDV in Hepatocellular Carcinoma. Chin J Infect Dis. 1995; 13: 129-32	Scientific literature
	Fan XL, Zhou YP, Peng WW, Yao JL, Li GH, Guo RP. A case control study on relationship between hepatocellular carcinoma and infection of HCV and HBV in Guangzhou. Chin J Cancer. 1995; 14: 328-30	Scientific literature
	Pan WS, Tian X, Tang SZ. A case–control study of HCV infection and primary liver cancer. Chin J Public Health. 1995; 11: 291 4	Scientific literature
	Wang CX, Feng XH, Yyuan RZ, Li DS, Wang HJ, Cheng SQ. A study of the relationship in HBV, HCV infection and primary hepatocellular carcinoma. Chin J Health Lab Tech. 1996; 6: 184-5	Scientific literature

	World Health Organization (WHO). WHO Mortality Database Version September 2016. Geneva, Switzerland: World Health Organization (WHO), 2016	Vital registration
	Hong Kong Vital Registration - Deaths 1980 ICD9 as it appears in World Health Organization (WHO). WHO Mortality Database Version September 2016. Geneva, Switzerland: World Health Organization (WHO), 2016	Vital registration
	Hong Kong Vital Registration - Deaths 1981 ICD9 as it appears in World Health Organization (WHO). WHO Mortality Database Version September 2016. Geneva, Switzerland: World Health Organization (WHO), 2016	Vital registration
	Hong Kong Vital Registration - Deaths 1982 ICD9 as it appears in World Health Organization (WHO). WHO Mortality Database Version September 2016. Geneva, Switzerland: World Health Organization (WHO), 2016	Vital registration
	Hong Kong Vital Registration - Deaths 1983 ICD9 as it appears in World Health Organization (WHO). WHO Mortality Database Version September 2016. Geneva, Switzerland: World Health Organization (WHO), 2016	Vital registration
	Hong Kong Vital Registration - Deaths 1984 ICD9 as it appears in World Health Organization (WHO). WHO Mortality Database Version September 2016. Geneva, Switzerland: World Health Organization (WHO), 2016	Vital registration
	Hong Kong Vital Registration - Deaths 1985 ICD9 as it appears in World Health Organization (WHO). WHO Mortality Database Version September 2016. Geneva, Switzerland: World Health Organization (WHO), 2016	Vital registration
	Hong Kong Vital Registration - Deaths 1986 ICD9 as it appears in World Health Organization (WHO). WHO Mortality Database Version September 2016. Geneva, Switzerland: World Health Organization (WHO), 2016	Vital registration
	Hong Kong Vital Registration - Deaths 1987 ICD9 as it appears in World Health Organization (WHO). WHO Mortality Database Version September 2016. Geneva, Switzerland: World Health Organization (WHO), 2016	Vital registration
	Hong Kong Vital Registration - Deaths 1988 ICD9 as it appears in World Health Organization (WHO). WHO Mortality Database Version September 2016. Geneva, Switzerland: World Health Organization (WHO), 2016	Vital registration
	Hong Kong Vital Registration - Deaths 1989 ICD9 as it appears in World Health Organization (WHO). WHO Mortality Database Version September 2016. Geneva, Switzerland: World Health Organization (WHO), 2016	Vital registration
	Hong Kong Vital Registration - Deaths 1991 ICD9 as it appears in World Health Organization (WHO). WHO Mortality Database Version September 2016. Geneva, Switzerland: World Health Organization (WHO), 2016	Vital registration
	Hong Kong Vital Registration - Deaths 1992 ICD9 as it appears in World Health Organization (WHO). WHO Mortality Database Version September 2016. Geneva, Switzerland: World Health Organization (WHO), 2016	Vital registration
	Hong Kong Vital Registration - Deaths 1993 ICD9 as it appears in World Health Organization (WHO). WHO Mortality Database Version September 2016. Geneva, Switzerland: World Health Organization (WHO), 2016	Vital registration
	Li QH, Gao WJ, Li JY, Shi LA, Hao XJ, Ge SW, An SH. [Detection of respiratory viruses in children with acute lower respiratory tract infection: an analysis of 5,150 children]. Chin J Contemp Pediatr. 2016; 18(1): 51-4	Scientific literature
	Shu LH, Xu JJ, Wang S, Zhong HQ, Dong XY, Jiang K, Zhang HY, Xiong Q, Wang C, Sun T, Sun C, Lu Q. [Distribution of pathogenic microorganisms and its relationship with clinical features in children with community-acquired pneumonia]. Chin J Contemp Pediatr. 2015; 17(10): 1056-61	Scientific literature
	Liu C, Xiao Y, Zhang J, Ren L, Li J, Xie Z, Xu B, Yang Y, Qian S, Wang J, Shen K. Adenovirus infection in children with acute lower respiratory tract infections in Beijing, China, 2007 to 2012. BMC Infect Dis. 2015; 15: 408	Scientific literature
	Lu L, Yan Y, Yang B, Xiao Z, Feng X, Wang Y, Ji W, Mize M, Hao C, Chen Z. Epidemiological and clinical profiles of respiratory syncytial virus infection in hospitalized neonates in Suzhou, China. BMC Infect Dis. 2015; 15: 431	Scientific literature
	Yu X, Kou Y, Xia D, Li J, Yang X, Zhou Y, He X. Human respiratory syncytial virus in children with lower respiratory tract infections or influenza-like illness and its co-infection characteristics with viruses and atypical bacteria in Hangzhou, China. J Clin Virol. 2015; 69: 1-6	Scientific literature
	Guan WD, Gong XY, Mok CK, Chen TT, Wu SG, Pan SH, Cowling BJ, Yang ZF, Chen de H. Surveillance for seasonal influenza virus prevalence in hospitalized children with lower respiratory tract infection in Guangzhou, China during the post-pandemic era. PLoS One. 2015; 10(4): e0120983	Scientific literature
	Ren L, Xiao Q, Zhou L, Xia Q, Liu E. Molecular characterization of human respiratory syncytial virus subtype B: a novel genotype of subtype B circulating in China. J Med Virol. 2015; 87(1): 1-9	Scientific literature
	Peng Y, Shu C, Fu Z, Li QB, Liu Z, Yan L. [Pathogen detection of 1 613 cases of hospitalized children with community acquired pneumonia]. Chin J Contemp Pediatr. 2015; 17.0(11): 1193-9	Scientific literature
	Lin Y, Fu Y, Xu M, Su L, Cao L, Xu J, Cheng X. Evaluation of a PCR/ESI-MS platform to identify respiratory viruses from nasopharyngeal aspirates. J Med Virol. 2015; 87.0(11): 1867-71	Scientific literature
	Wu S, Qiu Y, Yan P, Wang W, Xie M, Xia P, Wang Z, Yan Y. Effectiveness of first-line antiretroviral therapy in HIV/AIDS patients: A 5-year longitudinal evaluation in Fujian Province, Southeast China. Arch Virol. 2015; 160(11): 2693–701	Scientific literature
	Joint United Nations Program on HIV/AIDS (UNAIDS), United Nations Children's Fund (UNICEF), World Health Organization (WHO). China Global AIDS Response Progress Reporting (GARPR) System - Antenatal Care Attendees Positive for Syphilis	Epi surveillance
	Department of Peace and Conflict Research, Uppsala University. UCDP Battle-Related Deaths Database, Version 5, 2016. Uppsala, Sweden: Department of Peace and Conflict Research, Uppsala University, 2015	Estimate
	Department of Peace and Conflict Research, Uppsala University. UCDP Nonstate Conflict Dataset, Version 2.5, 2016. Uppsala, Sweden: Department of Peace and Conflict Research, Uppsala University, 2013	Estimate
	Chinese Center for Disease Control and Prevention (CCDC). China Disease Surveillance Points and Death Registration 2008 - China CDC	Vital registration
	Chinese Center for Disease Control and Prevention (CCDC). China Disease Surveillance Points and Death Registration 2009 - China CDC	Vital registration
	Chinese Center for Disease Control and Prevention (CCDC). China Disease Surveillance Points and Death Registration 2010 - China CDC	Vital registration
	Chinese Center for Disease Control and Prevention (CCDC). China Disease Surveillance Points and Death Registration 2011 - China CDC	Vital registration

	Chinese Center for Disease Control and Prevention (CCDC). China Disease Surveillance Points and Death Registration 2012 - China CDC	Vital registration
	Chinese Center for Disease Control and Prevention (CCDC). China Disease Surveillance Points 2013 - China CDC	Vital registration
	Chinese Center for Disease Control and Prevention (CCDC). China Disease Surveillance Points 2014 - China CDC	Vital registration
	Chinese Center for Disease Control and Prevention (CCDC). China Disease Surveillance Points 2015 - China CDC	Vital registration
	London School of Hygiene and Tropical Medicine. Global Atlas of Helminth Infections - Soil Transmitted Helminths. London, United Kingdom: London School of Hygiene and Tropical Medicine	Estimate
	Yang L, Jin X, Yan J, Jin Y, Yu W, Wu H, Xu S. Prevalence of dementia, cognitive status and associated risk factors among elderly of Zhejiang province, China in 2014. Age Ageing. 2016; 45(5): 708-12	Scientific literature
	Ban CX, Zhong L, Wang T, Zhu MJ, Wang JH, Zhang ZL, Wang Z, Su N, Liu YY, Shi YC, Xiao SF, Li X. Enhanced Diabetes Susceptibility in Community Dwelling Han Elders Carrying the Apolipoprotein E 3/3 Genotype. PLoS One. 2016; 11(3): e0151336	Scientific literature
	Xu X, Xiao S, Rahardjo TB, Hogervorst E. Risk Factors for Possible Dementia Using the Hopkins Verbal Learning Test and the Mini-Mental State Examination in Shanghai. Diagnostics (Basel). 2015; 5(4): 487-96	Scientific literature
	Yuan J, Zhang Z, Wen H, Hong X, Hong Z, Qu Q, Tang M, Wu J, Xu Q, Li H, Cummings JL. Incidence of dementia and subtypes: A cohort study in four regions in China. Alzheimers Dement. 2016; 12(3): 262-71	Scientific literature
	Xiao S, Lewis M, Mellor D, McCabe M, Byrne L, Wang T, Wang J, Zhu M, Cheng Y, Yang C, Dong S. The China longitudinal ageing study: overview of the demographic, psychosocial and cognitive data of the Shanghai sample. J Ment Health. 2016; 25(2): 131-6	Scientific literature
	Ellis MK, Raso G, Li Y-S, Rong Z, Chen H-G, McManus DP. Familial aggregation of human susceptibility to co- and multiple helminth infections in a population from the Poyang Lake region, China. Int J Parasitol. 2007; 37(10): 1153–61	Scientific literature
	Xu Z-G, He Z-W, Wang Y-B, Tu Z-W, Xiong B. [Schistosomiasis endemic situation at a national surveillance site in Gong'an County, Hubei Province from 2004 to 2013]. Chin J Schistosomiasis Control. 2014; 26(5): 588–90	Scientific literature
	Ross A, Li Y, Williams G, Li Y, Sleigh A, Zhang X, Zhou D, McManus D. An examination of current control strategies for Asian schistosomiasis in the Dongting lake region of China .2. A five year follow-up survey on Qingshan island. Addict Behav Rep. 1997; 68(1): 93–104	Scientific literature
	Zhang Y, Feng X, Wu M, Xiong M, Shen M, Sun J. [Evaluation of effectiveness of comprehensive schistosomiasis control project in Yunnan Province from 2004 to 2013]. Chin J Schistosomiasis Control. 2015; 27(2): 115–8, 133	Scientific literature
	Ai Z-Y. [Effect of schistosomiasis comprehensive control in Linxiang City, 2004-2012]. Chin J Schistosomiasis Control. 2013; 25(3): 296–8, 306	Scientific literature
	Hu Y, Li R, Ward MP, Chen Y, Lynn H, Wang D, Chen G, He Z, Sun L, Xiong C, Zhang Z, Jiang Q. Human infections and co-infections with helminths in a rural population in Guichi, Anhui Province, China. Geospat Health. 2015; 10(2): 374	Scientific literature
	Chen B-J, Li L-S, Lin M-X, Xu M-C, Chen Z-L, Fang Y-E, Yang H. [Surveillance of schistosomiasis in national surveillance sites of Fuqing City and Xiapu County, 2006 -2009]. Chin J Schistosomiasis Control. 2011; 23(1): 89–92	Scientific literature
	Xie C, Yang P, Wang Y. [Impact of implementation of Three Gorges Project on schistosomiasis endemic situation and control in Nanjing City]. Chin J Schistosomiasis Control. 2015; 27(6): 583–5, 607	Scientific literature
	Tu Z-W, Huang X-B, Cai S-X, Fan H-P, Wang K-Y, Liu X-G. [Surveillance of schistosomiasis japonica in potential endemic areas in Hubei Province from 2008 to 2012]. Chin J Schistosomiasis Control. 2014; 26(1): 56–8	Scientific literature
	Liu L, Yang G-J, Zhu H-R, Yang K, Ai L. Knowledge of, attitudes towards, and practice relating to schistosomiasis in two subtypes of a mountainous region of the People's Republic of China. Infect Dis Poverty. 2014; 3(1)	Scientific literature
	Xiang R, Zhang Z, Yu B, Shan X, Deng F, Xu X, Fang R, Liu Zhi-Shuang null, Zhang H, Wang H. [Effect of comprehensive schistosomiasis control strategy based on infection source control in Hanchuan City]. Chin J Schistosomiasis Control. 2014; 26(6): 658–61	Scientific literature
	Lu D-B, Wang T-P, Rudge JW, Donnelly CA, Fang G-R, Webster JP. Contrasting reservoirs for Schistosoma japonicum between marshland and hilly regions in Anhui, China--a two-year longitudinal parasitological survey. Parasitology. 2010; 137(1): 99–110	Scientific literature
	Cao Z, Wang T, Zhang S, Sha J, Huang H, Zhu L. [Risk evaluation of schistosomiasis japonica input to potential endemic areas in Anhui province]. Chin J Prev Med. 2014; 48(1): 58–61	Scientific literature
	Liu J-M, Liao X-C. [Effect of integrated control of schistosomiasis in Shashi District from 2009 to 2013]. Chin J Schistosomiasis Control. 2014; 26(5): 581–2, 585	Scientific literature
	Xu J, Chen N, Feng T, Wang E, Wu X, Chen H, Wang T, Zhou X, Zheng J. [Effectiveness of routinely used assays for the diagnosis of schistosomiasis japonica in the field]. Chin J Parasitol Parasit Dis. 2007; 25(3): 175–9	Scientific literature
	Gray DJ, Williams GM, Li Y, Chen H, Forsyth SJ, Li RS, Barnett AG, Guo J, Ross AG, Feng Z, McManus DP. A cluster-randomised intervention trial against Schistosoma japonicum in the Peoples' Republic of China: bovine and human transmission. PLoS One. 2009; 4(6): e5900	Scientific literature
	Shen M, Huang N, Feng X, Zhang Y, Wang L, Xiong M, Li T, Yang W, Yang W, Chen S. [Analysis of schistosomiasis endemic status of national surveillance sites in Yunnan Province, 2014]. Chin J Schistosomiasis Control. 2015; 27(5): 463–6	Scientific literature
	Yu JM, de Vlas SJ, Yuan HC, Gryseels B. Variations in fecal Schistosoma japonicum egg counts. Am J Trop Med Hyg. 1998; 59(3): 370–5	Scientific literature
	Booth M, Li Y, Tanner M. Helminth infections, morbidity indicators and schistosomiasis treatment history in three villages, Dongting Lake region, PR China. Trop Med Int Health. 1996; 1(4): 464–74	Scientific literature
	Wu Z, Shaoji Z, Pan B, Hu L, Wei R, Gao Z, Li J, Uwe B. Reinfection with Schistosoma japonicum after treatment with praziquantel in Poyang lake region, China. Southeast Asian J Trop Med Public Health. 1994; 25(1): 163–9	Scientific literature

	Zhao G, Jiang Q, Wasley A, Zhang S, ljWu Z, Liu Z, Yuan H. Changes in prevalence, intensity of infection and morbidity due to Schistosoma japonicum infection in a community following a single treatment with praziquantel. Trop Geogr Med. 1995; 47(6): 289–92	Scientific literature
	Jiang Q, Zhang S, Yuan H, Liu Z, Zhao G, Brinkmann U. The effect of a combined approach to schistosomiasis control on the transmission of Schistosoma japonicum in Xingzi of Poyang Lake area, China. Southeast Asian J Trop Med Public Health. 1996; 27(3): 535–41	Scientific literature
	Hu G-H, Jia H, Song K-Y, Lin D-D, Ju Z, Cao C-L, Jing X, Dong L, Jiang W-S. The role of health education and health promotion in the control of schistosomiasis: Experiences from a 12-year intervention study in the Poyang Lake area. Addict Behav Rep. 2005; 96(2-3): 232–41	Scientific literature
	Yu Q, Wan X-X, Liu Q, Cao C-L, Bao Z-P, Zhu H-Q, Zhong B, Guo J-G. [Cost-effectiveness evaluation and investigation of control measure changes in areas of schistosomiasis transmission control in hilly regions of mountain areas I epidemiological investigation and analysis of prevalence factors of schistosomiasis]. Chin J Schistosomiasis Control. 2012; 24(3): 250–4, 365	Scientific literature
	Li S-Z, Zheng H, Gao J, Zhang L-J, Zhu R, Xu J, Guo J-G, Xiao N, Zhou X-N. [Endemic status of schistosomiasis in People's Republic of China in 2012]. Chin J Schistosomiasis Control. 2013; 25(6): 557–63	Scientific literature
	Li Y, Ross A, Yu D, Li Y, Williams G, McManus D. An evaluation of Schistosoma japonicum infections in three villages in the Dongting lake region of China .1. Prevalence, intensity and morbidity before the implementation of adequate control strategies. Addict Behav Rep. 1997; 68(1): 77–91	Scientific literature
	Ministry of Public Health (China). China Schistosomiasis Prevalence National Sample Survey 1989	Survey
	Pigott DM, Bhatt S, Golding N, Duda KA, Battle KE, Brady OJ, Messina JP, Balard Y, Bastien P, Pratlong F, Brownstein JS, Freifeld CC, Mekaru SR, Gething PW, George DB, Myers MF, Reithinger R, Hay SI. Global distribution maps of the leishmaniases. Elife. 2014; 3: e02851	Scientific literature
	Cheung T-K, Lai C-L, Wong BC-Y, Fung J, Yuen M-F. Clinical features, biochemical parameters, and virological profiles of patients with hepatocellular carcinoma in Hong Kong. Aliment Pharmacol Ther. 2006; 24(4): 573-83	Scientific literature
	Ding X, Park YN, Taltavull TC, Thung SN, Jin X, Jin Y, Trung NS, Edamoto Y, Sata T, Abe K. Geographic characterization of hepatitis virus infections, genotyping of hepatitis B virus, and p53 mutation in hepatocellular carcinoma analyzed by in situ detection of viral genomes from carcinoma tissues: comparison among six different countries. Jpn J Infect Dis. 2003; 56(1): 12-8	Scientific literature
	World Health Organization (WHO). WHO Global Health Observatory Interactive Graph - Number of Cases of Visceral Leishmaniasis Reported 2013. Geneva, Switzerland: World Health Organization (WHO)	Epi surveillance
	London School of Hygiene and Tropical Medicine. Global Atlas of Helminth Infections - Schistosomiasis. London, United Kingdom: London School of Hygiene and Tropical Medicine	Estimate
	World Health Organization (WHO). WHO Global Project on Anti-Tuberculosis Drug Resistance Surveillance Data 1988-2015	Survey
	Wang Q, Tan Y, Ren Y, Dong L, Xie Z, Tang L, Cao D, Zhang W, Hu H, Wang H. Zinc finger protein ZBTB20 expression is increased in hepatocellular carcinoma and associated with poor prognosis . BMC Cancer. 2011; 11(1): 271	Scientific literature
	Xuan S-Y, Xin Y-N, Chen H, Shi G-J, Guan H-S, Li Y. Significance of hepatitis B virus surface antigen, hepatitis C virus expression in hepatocellular carcinoma and pericarcinomatous tissues . World J Gastroenterol. 2007; 13(12): 1870	Scientific literature
	Yuan J-M, Fan Y, Ognjanovic S, Wang R, Van Den Berg D, Govindarajan S, Mimi CY. Genetic polymorphisms of epidermal growth factor in relation to risk of hepatocellular carcinoma: two case-control studies . BMC Gastroenterol. 2013; 13(1): 32	Scientific literature
	Yuan J-M, Ross RK, Stanczyk FZ, Govindarajan S, Gao Y-T, Henderson BE, Yu MC. A cohort study of serum testosterone and hepatocellular carcinoma in Shanghai, China . Int J Cancer. 1995; 63(4): 491-3	Scientific literature
	Li Q, Li H, Qin Y, Wang PP, Hao X. Comparison of surgical outcomes for small hepatocellular carcinoma in patients with hepatitis B versus hepatitis C: A Chinese experience . J Gastroenterol Hepatol. 2007; 22(11): 1936-41	Scientific literature
	Liu C, Xiao G-Q, Yan L-N, Li B, Li J, Wen T-F, Wang W-T, Xu M-Q, Yang J-Y. Value of ?-fetoprotein in association with clinicopathological features of hepatocellular carcinoma . World J Gastroenterol. 2013; 19(11): 1811	Scientific literature
	Long J, Wang Y, Li M, Tong W-M, Jia J-D, Huang J. Correlation of TP53 mutations with HCV positivity in hepatocarcinogenesis: identification of a novel TP53 microindel in hepatocellular carcinoma with HCV infection . Oncol Rep. 2013; 30(1): 119-24	Scientific literature
	Mao Y-M, Luo Z-Y, Li B, Hu T-Y. Prospective Study on the Survival of HCC Patients Treated with Transcatheter Arterial Lipiodol Chemoembolization . Asian Pac J Cancer Prev. 2012; 13(3): 1039-42	Scientific literature
	Okuno H, Xie Z-C, Lu B-Y, Qin X, Takasu M, Kano H, Shiozaki Y, Inoue K. A low prevalence of anti-hepatitis C virus antibody in patients with hepatocellular carcinoma in guangxi province, southern china . Cancer. 1994; 73(1): 58-62	Scientific literature
	Qiao C-X. Health-related quality of life evaluated by tumor node metastasis staging system in patients with hepatocellular carcinoma . World J Gastroenterol. 2012; 18(21): 2689	Scientific literature
	World Health Organization (WHO). WHO Global Health Observatory - Cholera: Number of Reported Deaths by Country. Geneva, Switzerland: World Health Organization (WHO)	Epi surveillance
	Aon Benfield. Global Catastrophe Recap October 2016. London, United Kingdom: Aon Benfield, 2016	Report
	Shen X, DeRiemer K, Yuan Z 'an, Shen M, Xia Z, Gui X, Wang L, Mei J. Deaths among tuberculosis cases in Shanghai, China: who is at risk?. BMC Infect Dis. 2009; 9(1): 95	Scientific literature
	Sun Y, Harley D, Vally H, Sleigh A. Comparison of characteristics and mortality in multidrug resistant (MDR) and non-MDR tuberculosis patients in China. BMC Public Health. 2015; 15(1): 1027	Scientific literature
	Malaria Atlas Project. Malaria Atlas Project Annual Parasite Incidence Database	Epi surveillance

	Zhou H, Zhao M, Li X, Zhang D, Zhou S, Chen C, Feng Z, Ma X. Clinical evaluation of the isothermal amplification assays for the detection of four common respiratory viruses in children with pneumonia. Arch Virol. 2017; 162(5): 1311-1318	Scientific literature
	World Health Organization (WHO). WHO Mortality Database Version October 2017. Geneva, Switzerland: World Health Organization (WHO), 2017	Vital registration
	Khan MI, Ochiai RL, Von Seidlein L, Dong B, Bhattacharya SK, Agtini MD, Bhutta ZA, Do GC, Ali M, Kim DR, Favorov M, Clemens JD. Non-typhoidal Salmonella rates in febrile children at sites in five Asian countries: Non-typhoidal Salmonella in Asia. Trop Med Int Health. 2010; 15(8): 960–3	Scientific literature
	Bray F, Colombet M, Mery L, Piñeros M, Znaor A, Zanetti R and Ferlay J, editors (2017). Cancer Incidence in Five Continents, Vol. XI (electronic version). Lyon: International Agency for Research on Cancer. Available from: http://ci5.iarc.fr	Disease registry
	China - Anshan City Cancer Registry 2008-2012 - CI5 as it appears in Bray F, Colombet M, Mery L, Piñeros M, Znaor A, Zanetti R and Ferlay J, editors (2017). Cancer Incidence in Five Continents, Vol. XI (electronic version). Lyon: International Agency for Research on Cancer. Available from: http://ci5.iarc.fr	Disease registry
	China - Beijing Cancer Registry 2008-2012 - CI5 as it appears in Bray F, Colombet M, Mery L, Piñeros M, Znaor A, Zanetti R and Ferlay J, editors (2017). Cancer Incidence in Five Continents, Vol. XI (electronic version). Lyon: International Agency for Research on Cancer. Available from: http://ci5.iarc.fr	Disease registry
	China - Benxi Cancer Registry 2008-2011 - CI5 as it appears in Bray F, Colombet M, Mery L, Piñeros M, Znaor A, Zanetti R and Ferlay J, editors (2017). Cancer Incidence in Five Continents, Vol. XI (electronic version). Lyon: International Agency for Research on Cancer. Available from: http://ci5.iarc.fr	Disease registry
	China - Guangzhou Cancer Registry 2010-2012 - CI5 as it appears in Bray F, Colombet M, Mery L, Piñeros M, Znaor A, Zanetti R and Ferlay J, editors (2017). Cancer Incidence in Five Continents, Vol. XI (electronic version). Lyon: International Agency for Research on Cancer. Available from: http://ci5.iarc.fr	Disease registry
	China - Guanyun Cancer Registry 2008-2012 - CI5 as it appears in Bray F, Colombet M, Mery L, Piñeros M, Znaor A, Zanetti R and Ferlay J, editors (2017). Cancer Incidence in Five Continents, Vol. XI (electronic version). Lyon: International Agency for Research on Cancer. Available from: http://ci5.iarc.fr	Disease registry
	China - Haimen County Cancer Registry 2008-2012 - CI5 as it appears in Bray F, Colombet M, Mery L, Piñeros M, Znaor A, Zanetti R and Ferlay J, editors (2017). Cancer Incidence in Five Continents, Vol. XI (electronic version). Lyon: International Agency for Research on Cancer. Available from: http://ci5.iarc.fr	Disease registry
	China - Harbin City, Nangang District Cancer Registry 2008-2012 - CI5 as it appears in Bray F, Colombet M, Mery L, Piñeros M, Znaor A, Zanetti R and Ferlay J, editors (2017). Cancer Incidence in Five Continents, Vol. XI (electronic version). Lyon: International Agency for Research on Cancer. Available from: http://ci5.iarc.fr	Disease registry
	China - Hefei Cancer Registry 2010-2012 - CI5 as it appears in Bray F, Colombet M, Mery L, Piñeros M, Znaor A, Zanetti R and Ferlay J, editors (2017). Cancer Incidence in Five Continents, Vol. XI (electronic version). Lyon: International Agency for Research on Cancer. Available from: http://ci5.iarc.fr	Disease registry
	China - Hengdong Cancer Registry 2009-2012 - CI5 as it appears in Bray F, Colombet M, Mery L, Piñeros M, Znaor A, Zanetti R and Ferlay J, editors (2017). Cancer Incidence in Five Continents, Vol. XI (electronic version). Lyon: International Agency for Research on Cancer. Available from: http://ci5.iarc.fr	Disease registry
	China - Hong Kong Cancer Registry 2008-2012 - CI5 as it appears in Bray F, Colombet M, Mery L, Piñeros M, Znaor A, Zanetti R and Ferlay J, editors (2017). Cancer Incidence in Five Continents, Vol. XI (electronic version). Lyon: International Agency for Research on Cancer. Available from: http://ci5.iarc.fr	Disease registry
	China - Huaiyin District, Huai'an Cancer Registry 2009-2012 - CI5 as it appears in Bray F, Colombet M, Mery L, Piñeros M, Znaor A, Zanetti R and Ferlay J, editors (2017). Cancer Incidence in Five Continents, Vol. XI (electronic version). Lyon: International Agency for Research on Cancer. Available from: http://ci5.iarc.fr	Disease registry
	China - Jiangmen Cancer Registry 2010-2012 - CI5 as it appears in Bray F, Colombet M, Mery L, Piñeros M, Znaor A, Zanetti R and Ferlay J, editors (2017). Cancer Incidence in Five Continents, Vol. XI (electronic version). Lyon: International Agency for Research on Cancer. Available from: http://ci5.iarc.fr	Disease registry
	China - Jianhu County Cancer Registry 2010-2012 - CI5 as it appears in Bray F, Colombet M, Mery L, Piñeros M, Znaor A, Zanetti R and Ferlay J, editors (2017). Cancer Incidence in Five Continents, Vol. XI (electronic version). Lyon: International Agency for Research on Cancer. Available from: http://ci5.iarc.fr	Disease registry
	China - Jiashan County Cancer Registry 2008-2012 - CI5 as it appears in Bray F, Colombet M, Mery L, Piñeros M, Znaor A, Zanetti R and Ferlay J, editors (2017). Cancer Incidence in Five Continents, Vol. XI (electronic version). Lyon: International Agency for Research on Cancer. Available from: http://ci5.iarc.fr	Disease registry
	China - Jiaxing City Cancer Registry 2008-2012 - CI5 as it appears in Bray F, Colombet M, Mery L, Piñeros M, Znaor A, Zanetti R and Ferlay J, editors (2017). Cancer Incidence in Five Continents, Vol. XI (electronic version). Lyon: International Agency for Research on Cancer. Available from: http://ci5.iarc.fr	Disease registry
	China - Lianyungang Cancer Registry 2008-2012 - CI5 as it appears in Bray F, Colombet M, Mery L, Piñeros M, Znaor A, Zanetti R and Ferlay J, editors (2017). Cancer Incidence in Five Continents, Vol. XI (electronic version). Lyon: International Agency for Research on Cancer. Available from: http://ci5.iarc.fr	Disease registry
	China - Linzhou County Cancer Registry 2008-2012 - CI5 as it appears in Bray F, Colombet M, Mery L, Piñeros M, Znaor A, Zanetti R and Ferlay J, editors (2017). Cancer Incidence in Five Continents, Vol. XI (electronic version). Lyon: International Agency for Research on Cancer. Available from: http://ci5.iarc.fr	Disease registry
	China - Liuzhou Cancer Registry 2009-2012 - CI5 as it appears in Bray F, Colombet M, Mery L, Piñeros M, Znaor A, Zanetti R and Ferlay J, editors (2017). Cancer Incidence in Five Continents, Vol. XI (electronic version). Lyon: International Agency for Research on Cancer. Available from: http://ci5.iarc.fr	Disease registry

	China - Maanshan Cancer Registry 2008-2012 - CI5 as it appears in Bray F, Colombet M, Mery L, Piñeros M, Znaor A, Zanetti R and Ferlay J, editors (2017). Cancer Incidence in Five Continents, Vol. XI (electronic version). Lyon: International Agency for Research on Cancer. Available from: http://ci5.iarc.fr	Disease registry
	China - Qidong County Cancer Registry 2008-2012 - CI5 as it appears in Bray F, Colombet M, Mery L, Piñeros M, Znaor A, Zanetti R and Ferlay J, editors (2017). Cancer Incidence in Five Continents, Vol. XI (electronic version). Lyon: International Agency for Research on Cancer. Available from: http://ci5.iarc.fr	Disease registry
	China - Shanghai City Cancer Registry 2008-2012 - CI5 as it appears in Bray F, Colombet M, Mery L, Piñeros M, Znaor A, Zanetti R and Ferlay J, editors (2017). Cancer Incidence in Five Continents, Vol. XI (electronic version). Lyon: International Agency for Research on Cancer. Available from: http://ci5.iarc.fr	Disease registry
	China - Shenyang Cancer Registry 2008-2012 - CI5 as it appears in Bray F, Colombet M, Mery L, Piñeros M, Znaor A, Zanetti R and Ferlay J, editors (2017). Cancer Incidence in Five Continents, Vol. XI (electronic version). Lyon: International Agency for Research on Cancer. Available from: http://ci5.iarc.fr	Disease registry
	China - Shexian County Cancer Registry 2008-2012 - CI5 as it appears in Bray F, Colombet M, Mery L, Piñeros M, Znaor A, Zanetti R and Ferlay J, editors (2017). Cancer Incidence in Five Continents, Vol. XI (electronic version). Lyon: International Agency for Research on Cancer. Available from: http://ci5.iarc.fr	Disease registry
	China - Sheyang Cancer Registry 2008-2012 - CI5 as it appears in Bray F, Colombet M, Mery L, Piñeros M, Znaor A, Zanetti R and Ferlay J, editors (2017). Cancer Incidence in Five Continents, Vol. XI (electronic version). Lyon: International Agency for Research on Cancer. Available from: http://ci5.iarc.fr	Disease registry
	China - Tongling City Cancer Registry 2008-2012 - CI5 as it appears in Bray F, Colombet M, Mery L, Piñeros M, Znaor A, Zanetti R and Ferlay J, editors (2017). Cancer Incidence in Five Continents, Vol. XI (electronic version). Lyon: International Agency for Research on Cancer. Available from: http://ci5.iarc.fr	Disease registry
	China - Wuhan City Cancer Registry 2008-2012 - CI5 as it appears in Bray F, Colombet M, Mery L, Piñeros M, Znaor A, Zanetti R and Ferlay J, editors (2017). Cancer Incidence in Five Continents, Vol. XI (electronic version). Lyon: International Agency for Research on Cancer. Available from: http://ci5.iarc.fr	Disease registry
	China - Wuxi Cancer Registry 2010-2012 - CI5 as it appears in Bray F, Colombet M, Mery L, Piñeros M, Znaor A, Zanetti R and Ferlay J, editors (2017). Cancer Incidence in Five Continents, Vol. XI (electronic version). Lyon: International Agency for Research on Cancer. Available from: http://ci5.iarc.fr	Disease registry
	China - Xianju Cancer Registry 2010-2012 - CI5 as it appears in Bray F, Colombet M, Mery L, Piñeros M, Znaor A, Zanetti R and Ferlay J, editors (2017). Cancer Incidence in Five Continents, Vol. XI (electronic version). Lyon: International Agency for Research on Cancer. Available from: http://ci5.iarc.fr	Disease registry
	China - Xiping Cancer Registry 2010-2012 - CI5 as it appears in Bray F, Colombet M, Mery L, Piñeros M, Znaor A, Zanetti R and Ferlay J, editors (2017). Cancer Incidence in Five Continents, Vol. XI (electronic version). Lyon: International Agency for Research on Cancer. Available from: http://ci5.iarc.fr	Disease registry
	China - Yanshi Cancer Registry 2010-2012 - CI5 as it appears in Bray F, Colombet M, Mery L, Piñeros M, Znaor A, Zanetti R and Ferlay J, editors (2017). Cancer Incidence in Five Continents, Vol. XI (electronic version). Lyon: International Agency for Research on Cancer. Available from: http://ci5.iarc.fr	Disease registry
	China - Yanting County Cancer Registry 2008-2012 - CI5 as it appears in Bray F, Colombet M, Mery L, Piñeros M, Znaor A, Zanetti R and Ferlay J, editors (2017). Cancer Incidence in Five Continents, Vol. XI (electronic version). Lyon: International Agency for Research on Cancer. Available from: http://ci5.iarc.fr	Disease registry
	China - Yueyanglou Cancer Registry 2009-2012 - CI5 as it appears in Bray F, Colombet M, Mery L, Piñeros M, Znaor A, Zanetti R and Ferlay J, editors (2017). Cancer Incidence in Five Continents, Vol. XI (electronic version). Lyon: International Agency for Research on Cancer. Available from: http://ci5.iarc.fr	Disease registry
	China - Zhongshan City Cancer Registry 2010-2012 - CI5 as it appears in Bray F, Colombet M, Mery L, Piñeros M, Znaor A, Zanetti R and Ferlay J, editors (2017). Cancer Incidence in Five Continents, Vol. XI (electronic version). Lyon: International Agency for Research on Cancer. Available from: http://ci5.iarc.fr	Disease registry
	China - Zhuhai City Cancer Registry 2010-2012 - CI5 as it appears in Bray F, Colombet M, Mery L, Piñeros M, Znaor A, Zanetti R and Ferlay J, editors (2017). Cancer Incidence in Five Continents, Vol. XI (electronic version). Lyon: International Agency for Research on Cancer. Available from: http://ci5.iarc.fr	Disease registry
	Department of Peace and Conflict Research, Uppsala University. UCDP Georeferenced Event Dataset, Version 17.1, 2016. Uppsala, Sweden: Department of Peace and Conflict Research, Uppsala University, 2017	Estimate
	Peace Research Institute Oslo (PRIO). Battle Deaths Dataset Version 3.1, 2009. Oslo, Norway: Peace Research Institute Oslo (PRIO), 2009	Estimate
	National Consortium for the Study of Terrorism and Responses to Terrorism (START). University of Maryland. Global Terrorism Database. 2017	Estimate
	Department of Political Science, University of Chicago, University of Chicago. Chicago Project on Security and Terrorism Suicide Attack Database	Vital registration
	British Broadcasting Corporation (BBC). China bus fire kills dozens in Xiamen in Fujian province. British Broadcasting Corporation (BBC) [Internet]. 2013 Jun 7; Asia-China	Other publication
	Center for Health Statistics and Information, National Health and Family Planning Commission (China), Shanghai Municipal Center for Disease Control and Prevention (Shanghai CDC), Shanghai Health Information Center. China Hospital Inpatient Data 2013-2016	Administrative record
	Chinese Center for Disease Control and Prevention (CCDC). China Disease Surveillance Points 2016 - China CDC	Vital registration
	World Health Organization (WHO). WHO Mortality Database Version April 2018. Geneva, Switzerland: World Health Organization (WHO), 2018	Vital registration
	Hong Kong Vital Registration - Deaths 2014 ICD10 as it appears in World Health Organization (WHO). WHO Mortality Database Version April 2018. Geneva, Switzerland: World Health Organization (WHO), 2018	Vital registration

	Hong Kong Vital Registration - Deaths 2015 ICD10 as it appears in World Health Organization (WHO). WHO Mortality Database Version April 2018. Geneva, Switzerland: World Health Organization (WHO), 2018	Vital registration
	Dananché C, Sánchez Picot V, Bénet T, Messaoudi M, Chou M, Wang J, Pape J-W, Awasthi S, Bavdekar A, Rakoto-Andrianarivelo M, Sylla M, Nymadawa P, Russomando G, Komurian-Pradel F, Endtz H, Paranhos-Baccalà G, Vanhems P, For The Gabriel Network null. Burden of Influenza in Less Than 5-Year-Old Children Admitted to Hospital with Pneumonia in Developing and Emerging Countries: A Descriptive, Multicenter Study. Am J Trop Med Hyg. 2018; 98(6): 1805–10	Scientific literature

5q0 (per 1,000) for China and provinces from 1990 to 2017

	Years																											
**Location name**	**1990**	**1991**	**1992**	**1993**	**1994**	**1995**	**1996**	**1997**	**1998**	**1999**	**2000**	**2001**	**2002**	**2003**	**2004**	**2005**	**2006**	**2007**	**2008**	**2009**	**2010**	**2011**	**2012**	**2013**	**2014**	**2015**	**2016**	**2017**
Anhui	51.0	50.0	47.0	45.0	43.0	41.0	39.0	38.0	36.0	34.0	32.0	30.0	29.0	27.0	26.0	25.0	23.0	22.0	20.0	19.0	18.0	17.0	16.0	15.0	14.0	14.0	13.0	12.0
Beijing	14.0	14.0	13.0	13.0	13.0	13.0	12.0	12.0	12.0	11.0	11.0	10.0	10.0	9.0	9.0	8.0	8.0	8.0	7.0	7.0	7.0	6.0	6.0	6.0	6.0	6.0	6.0	5.0
China	50.0	49.0	47.0	46.0	44.0	43.0	41.0	39.0	38.0	36.0	34.0	32.0	30.0	28.0	26.0	24.0	23.0	21.0	20.0	18.0	17.0	16.0	15.0	15.0	14.0	14.0	13.0	12.0
Chongqing	59.0	57.0	55.0	52.0	50.0	48.0	46.0	44.0	42.0	40.0	38.0	36.0	33.0	31.0	29.0	27.0	25.0	23.0	22.0	20.0	19.0	18.0	17.0	16.0	16.0	15.0	14.0	13.0
Fujian	28.0	28.0	27.0	26.0	25.0	25.0	24.0	23.0	22.0	21.0	20.0	19.0	18.0	17.0	16.0	15.0	14.0	13.0	12.0	11.0	11.0	10.0	10.0	9.0	9.0	8.0	8.0	7.0
Gansu	80.0	79.0	77.0	74.0	72.0	70.0	68.0	66.0	63.0	60.0	57.0	54.0	51.0	47.0	45.0	43.0	40.0	38.0	36.0	33.0	31.0	29.0	28.0	26.0	25.0	24.0	22.0	20.0
Guangdong	25.0	25.0	24.0	24.0	23.0	23.0	22.0	22.0	21.0	20.0	19.0	18.0	17.0	16.0	15.0	14.0	13.0	12.0	11.0	11.0	10.0	9.0	9.0	9.0	8.0	8.0	8.0	7.0
Guangxi	48.0	48.0	47.0	45.0	44.0	44.0	43.0	42.0	40.0	38.0	36.0	34.0	32.0	30.0	28.0	26.0	24.0	22.0	21.0	19.0	17.0	16.0	15.0	15.0	14.0	14.0	13.0	12.0
Guizhou	120.0	116.0	111.0	105.0	100.0	96.0	91.0	86.0	81.0	76.0	71.0	66.0	60.0	55.0	50.0	46.0	42.0	39.0	35.0	32.0	29.0	27.0	25.0	23.0	22.0	21.0	20.0	18.0
Hainan	45.0	45.0	44.0	42.0	41.0	40.0	40.0	39.0	37.0	36.0	36.0	34.0	33.0	32.0	31.0	30.0	28.0	27.0	25.0	24.0	22.0	21.0	20.0	19.0	19.0	18.0	17.0	16.0
Hebei	30.0	30.0	29.0	29.0	29.0	29.0	28.0	28.0	27.0	27.0	26.0	26.0	25.0	24.0	23.0	22.0	22.0	20.0	20.0	18.0	17.0	17.0	16.0	15.0	15.0	14.0	14.0	13.0
Heilongjiang	30.0	29.0	28.0	27.0	26.0	25.0	24.0	24.0	23.0	22.0	22.0	21.0	21.0	21.0	20.0	20.0	19.0	19.0	18.0	17.0	16.0	16.0	15.0	15.0	14.0	14.0	13.0	12.0
Henan	55.0	55.0	53.0	52.0	49.0	47.0	45.0	42.0	40.0	38.0	35.0	33.0	31.0	28.0	26.0	25.0	23.0	21.0	20.0	18.0	17.0	16.0	16.0	15.0	15.0	14.0	13.0	13.0
Hong Kong Special A	7.0	7.0	6.0	6.0	6.0	5.0	5.0	5.0	4.0	4.0	4.0	3.0	3.0	3.0	3.0	3.0	3.0	3.0	3.0	3.0	3.0	2.0	2.0	2.0	2.0	2.0	2.0	1.0
Hubei	48.0	47.0	44.0	42.0	39.0	38.0	36.0	34.0	33.0	31.0	29.0	28.0	26.0	24.0	23.0	22.0	21.0	20.0	19.0	18.0	17.0	16.0	15.0	15.0	14.0	14.0	13.0	12.0
Hunan	49.0	48.0	46.0	43.0	41.0	40.0	38.0	36.0	35.0	33.0	30.0	28.0	26.0	23.0	22.0	20.0	18.0	17.0	16.0	14.0	13.0	12.0	12.0	11.0	11.0	10.0	10.0	9.0
Inner Mongolia	44.0	43.0	42.0	40.0	39.0	38.0	37.0	36.0	34.0	33.0	31.0	30.0	28.0	26.0	25.0	24.0	23.0	22.0	20.0	19.0	18.0	17.0	17.0	16.0	16.0	15.0	14.0	13.0
Jiangsu	30.0	30.0	28.0	27.0	26.0	25.0	24.0	23.0	22.0	21.0	20.0	18.0	17.0	16.0	15.0	14.0	13.0	12.0	11.0	10.0	9.0	9.0	8.0	8.0	8.0	8.0	7.0	7.0
Jiangxi	74.0	72.0	69.0	66.0	63.0	60.0	58.0	55.0	52.0	49.0	46.0	43.0	40.0	37.0	35.0	33.0	30.0	28.0	27.0	25.0	23.0	22.0	21.0	20.0	19.0	18.0	17.0	16.0
Jilin	25.0	24.0	23.0	22.0	21.0	21.0	20.0	19.0	18.0	18.0	17.0	16.0	16.0	15.0	15.0	14.0	13.0	13.0	12.0	12.0	11.0	11.0	10.0	10.0	10.0	10.0	9.0	9.0
Liaoning	17.0	17.0	17.0	16.0	16.0	16.0	16.0	16.0	16.0	15.0	15.0	15.0	14.0	14.0	13.0	13.0	12.0	11.0	11.0	10.0	9.0	9.0	8.0	8.0	8.0	8.0	7.0	7.0
Macau Special Admin	9.0	9.0	8.0	8.0	7.0	7.0	6.0	6.0	6.0	5.0	5.0	5.0	4.0	4.0	4.0	4.0	4.0	4.0	3.0	3.0	3.0	3.0	3.0	2.0	2.0	2.0	2.0	2.0
Ningxia	77.0	75.0	71.0	68.0	65.0	62.0	59.0	57.0	54.0	51.0	49.0	46.0	43.0	40.0	38.0	36.0	33.0	31.0	29.0	27.0	25.0	23.0	22.0	20.0	19.0	18.0	17.0	16.0
Qinghai	94.0	91.0	87.0	83.0	80.0	76.0	73.0	69.0	66.0	62.0	59.0	56.0	52.0	49.0	46.0	43.0	40.0	38.0	35.0	33.0	32.0	29.0	28.0	26.0	26.0	25.0	23.0	21.0
Shaanxi	55.0	54.0	52.0	50.0	49.0	48.0	46.0	45.0	43.0	42.0	41.0	39.0	37.0	36.0	34.0	32.0	30.0	28.0	26.0	24.0	22.0	20.0	19.0	18.0	17.0	17.0	16.0	15.0
Shandong	30.0	30.0	29.0	28.0	28.0	27.0	27.0	26.0	26.0	25.0	24.0	23.0	22.0	21.0	20.0	19.0	17.0	16.0	15.0	14.0	13.0	13.0	12.0	12.0	11.0	11.0	10.0	10.0
Shanghai	16.0	16.0	15.0	14.0	14.0	13.0	13.0	13.0	12.0	12.0	11.0	11.0	10.0	10.0	10.0	9.0	9.0	9.0	9.0	9.0	9.0	8.0	8.0	8.0	8.0	7.0	7.0	7.0
Shanxi	35.0	35.0	34.0	33.0	32.0	32.0	32.0	31.0	30.0	29.0	27.0	26.0	24.0	23.0	22.0	21.0	20.0	19.0	18.0	17.0	16.0	15.0	15.0	14.0	14.0	13.0	13.0	12.0
Sichuan	74.0	71.0	67.0	64.0	61.0	58.0	55.0	52.0	50.0	47.0	45.0	43.0	40.0	38.0	35.0	33.0	31.0	29.0	31.0	25.0	23.0	22.0	20.0	19.0	19.0	18.0	17.0	15.0
Tianjin	17.0	17.0	16.0	16.0	16.0	16.0	16.0	15.0	15.0	15.0	14.0	14.0	13.0	13.0	12.0	12.0	11.0	11.0	11.0	10.0	10.0	9.0	9.0	9.0	9.0	9.0	8.0	8.0
Tibet	119.0	117.0	113.0	110.0	106.0	103.0	99.0	95.0	92.0	87.0	83.0	79.0	75.0	70.0	67.0	64.0	61.0	57.0	54.0	51.0	48.0	46.0	45.0	43.0	42.0	41.0	38.0	36.0
Xinjiang	93.0	91.0	87.0	83.0	80.0	77.0	75.0	72.0	69.0	66.0	63.0	60.0	57.0	54.0	51.0	49.0	47.0	45.0	43.0	41.0	39.0	37.0	36.0	34.0	33.0	32.0	30.0	28.0
Yunnan	93.0	90.0	86.0	82.0	78.0	75.0	71.0	67.0	63.0	59.0	55.0	50.0	46.0	43.0	40.0	37.0	34.0	31.0	29.0	27.0	26.0	24.0	23.0	21.0	21.0	19.0	18.0	17.0
Zhejiang	30.0	30.0	28.0	27.0	27.0	26.0	26.0	25.0	24.0	23.0	21.0	20.0	19.0	18.0	17.0	16.0	14.0	13.0	13.0	12.0	11.0	10.0	10.0	9.0	9.0	9.0	8.0	7.0

45q15 for China and provinces from 1990 to 2017

	Years																											
**Location name**	**1990**	**1991**	**1992**	**1993**	**1994**	**1995**	**1996**	**1997**	**1998**	**1999**	**2000**	**2001**	**2002**	**2003**	**2004**	**2005**	**2006**	**2007**	**2008**	**2009**	**2010**	**2011**	**2012**	**2013**	**2014**	**2015**	**2016**	**2017**
Anhui	0.167	0.166	0.165	0.160	0.156	0.151	0.147	0.141	0.136	0.131	0.127	0.122	0.118	0.114	0.111	0.106	0.100	0.095	0.092	0.089	0.087	0.086	0.084	0.083	0.082	0.082	0.081	0.080
Beijing	0.107	0.107	0.106	0.103	0.102	0.098	0.096	0.093	0.090	0.087	0.083	0.080	0.076	0.071	0.067	0.063	0.059	0.056	0.053	0.050	0.048	0.047	0.046	0.045	0.044	0.044	0.043	0.043
China	0.155	0.154	0.152	0.149	0.146	0.143	0.140	0.136	0.132	0.129	0.126	0.122	0.119	0.116	0.114	0.110	0.105	0.101	0.101	0.095	0.093	0.092	0.091	0.089	0.088	0.087	0.086	0.085
Chongqing	0.184	0.180	0.175	0.168	0.160	0.153	0.147	0.139	0.133	0.127	0.122	0.116	0.111	0.106	0.102	0.097	0.093	0.090	0.086	0.084	0.082	0.081	0.080	0.079	0.078	0.077	0.076	0.076
Fujian	0.137	0.132	0.126	0.128	0.127	0.126	0.126	0.123	0.121	0.120	0.118	0.116	0.113	0.108	0.105	0.098	0.095	0.092	0.089	0.086	0.083	0.081	0.079	0.074	0.072	0.071	0.070	0.069
Gansu	0.223	0.222	0.222	0.207	0.195	0.185	0.174	0.165	0.160	0.155	0.147	0.141	0.136	0.131	0.129	0.124	0.115	0.114	0.112	0.109	0.107	0.105	0.103	0.099	0.097	0.096	0.095	0.093
Guangdong	0.147	0.146	0.145	0.142	0.138	0.135	0.131	0.126	0.122	0.118	0.115	0.112	0.110	0.107	0.105	0.101	0.097	0.093	0.089	0.084	0.081	0.078	0.076	0.073	0.072	0.071	0.069	0.068
Guangxi	0.177	0.175	0.174	0.170	0.165	0.162	0.157	0.151	0.147	0.144	0.138	0.132	0.127	0.124	0.122	0.119	0.113	0.113	0.112	0.112	0.113	0.113	0.114	0.115	0.116	0.116	0.116	0.115
Guizhou	0.225	0.225	0.224	0.220	0.215	0.210	0.205	0.197	0.191	0.185	0.180	0.173	0.168	0.162	0.158	0.153	0.147	0.141	0.136	0.132	0.130	0.128	0.126	0.124	0.123	0.121	0.120	0.118
Hainan	0.167	0.165	0.162	0.156	0.150	0.145	0.140	0.134	0.129	0.124	0.119	0.114	0.109	0.105	0.101	0.097	0.093	0.089	0.085	0.083	0.081	0.080	0.079	0.078	0.078	0.077	0.076	0.076
Hebei	0.134	0.133	0.131	0.132	0.131	0.130	0.129	0.127	0.125	0.123	0.121	0.120	0.120	0.119	0.119	0.116	0.111	0.110	0.109	0.108	0.107	0.107	0.108	0.107	0.107	0.106	0.105	0.104
Heilongjiang	0.176	0.172	0.167	0.164	0.160	0.156	0.153	0.148	0.145	0.142	0.140	0.137	0.135	0.133	0.131	0.128	0.122	0.117	0.113	0.108	0.106	0.104	0.103	0.101	0.099	0.098	0.097	0.096
Henan	0.129	0.126	0.122	0.123	0.123	0.122	0.122	0.121	0.120	0.120	0.119	0.118	0.118	0.117	0.117	0.114	0.110	0.107	0.104	0.100	0.099	0.098	0.097	0.095	0.095	0.094	0.093	0.091
Hong Kong Special Adm	0.094	0.091	0.089	0.086	0.083	0.080	0.077	0.077	0.073	0.071	0.068	0.066	0.065	0.064	0.062	0.059	0.057	0.056	0.056	0.056	0.056	0.053	0.051	0.052	0.051	0.051	0.050	0.049
Hubei	0.144	0.138	0.133	0.135	0.135	0.136	0.139	0.135	0.135	0.131	0.128	0.121	0.118	0.116	0.114	0.110	0.105	0.101	0.099	0.095	0.093	0.092	0.091	0.089	0.088	0.087	0.086	0.085
Hunan	0.184	0.180	0.177	0.172	0.166	0.161	0.156	0.151	0.146	0.142	0.138	0.134	0.131	0.128	0.126	0.122	0.119	0.116	0.113	0.111	0.110	0.109	0.110	0.109	0.110	0.110	0.109	0.107
Inner Mongolia	0.185	0.183	0.180	0.176	0.171	0.166	0.162	0.154	0.149	0.145	0.143	0.136	0.134	0.131	0.130	0.126	0.122	0.117	0.112	0.107	0.103	0.100	0.097	0.094	0.091	0.089	0.087	0.085
Jiangsu	0.125	0.126	0.125	0.126	0.124	0.123	0.121	0.120	0.118	0.115	0.113	0.110	0.106	0.102	0.099	0.095	0.089	0.085	0.081	0.076	0.074	0.071	0.069	0.066	0.064	0.064	0.063	0.061
Jiangxi	0.223	0.223	0.223	0.212	0.202	0.192	0.182	0.172	0.163	0.155	0.147	0.139	0.133	0.126	0.122	0.116	0.110	0.106	0.103	0.099	0.096	0.094	0.093	0.090	0.088	0.087	0.085	0.084
Jilin	0.173	0.172	0.171	0.167	0.163	0.158	0.154	0.148	0.143	0.138	0.134	0.129	0.125	0.121	0.118	0.114	0.109	0.104	0.100	0.096	0.093	0.092	0.090	0.089	0.088	0.087	0.086	0.086
Liaoning	0.140	0.139	0.138	0.135	0.132	0.130	0.126	0.122	0.120	0.117	0.114	0.111	0.109	0.107	0.106	0.104	0.101	0.099	0.096	0.093	0.092	0.091	0.090	0.089	0.088	0.088	0.087	0.086
Macau Special Administ	0.104	0.099	0.094	0.091	0.087	0.083	0.080	0.077	0.074	0.071	0.069	0.067	0.065	0.063	0.061	0.059	0.058	0.056	0.054	0.053	0.051	0.050	0.048	0.047	0.046	0.046	0.046	0.046
Ningxia	0.165	0.164	0.162	0.158	0.154	0.150	0.145	0.140	0.135	0.131	0.127	0.123	0.120	0.116	0.114	0.111	0.107	0.103	0.100	0.097	0.096	0.094	0.093	0.092	0.092	0.091	0.089	0.089
Qinghai	0.221	0.222	0.222	0.220	0.216	0.213	0.209	0.203	0.197	0.192	0.188	0.181	0.177	0.172	0.168	0.163	0.157	0.152	0.148	0.144	0.162	0.140	0.138	0.137	0.135	0.133	0.131	0.130
Shaanxi	0.084	0.081	0.079	0.080	0.081	0.082	0.084	0.085	0.086	0.088	0.090	0.091	0.092	0.092	0.093	0.093	0.092	0.090	0.089	0.088	0.088	0.088	0.088	0.088	0.087	0.087	0.086	0.086
Shandong	0.155	0.154	0.151	0.148	0.144	0.140	0.135	0.131	0.126	0.122	0.118	0.114	0.111	0.106	0.104	0.099	0.095	0.091	0.088	0.086	0.084	0.083	0.082	0.081	0.081	0.080	0.079	0.078
Shanghai	0.087	0.087	0.087	0.086	0.085	0.083	0.082	0.079	0.076	0.072	0.068	0.064	0.060	0.057	0.054	0.051	0.048	0.046	0.044	0.042	0.041	0.040	0.040	0.039	0.039	0.038	0.038	0.038
Shanxi	0.171	0.169	0.166	0.161	0.155	0.150	0.145	0.139	0.134	0.129	0.125	0.119	0.115	0.111	0.107	0.103	0.099	0.095	0.091	0.088	0.087	0.085	0.085	0.084	0.083	0.082	0.081	0.080
Sichuan	0.140	0.138	0.136	0.136	0.135	0.135	0.135	0.133	0.133	0.132	0.132	0.131	0.130	0.129	0.128	0.126	0.122	0.118	0.157	0.112	0.111	0.110	0.108	0.107	0.106	0.105	0.104	0.103
Tianjin	0.129	0.126	0.124	0.120	0.116	0.112	0.107	0.106	0.103	0.101	0.098	0.095	0.094	0.092	0.091	0.088	0.085	0.083	0.081	0.078	0.076	0.076	0.075	0.074	0.073	0.072	0.071	0.071
Tibet	0.341	0.344	0.347	0.346	0.343	0.340	0.335	0.328	0.317	0.299	0.297	0.280	0.272	0.252	0.239	0.225	0.210	0.198	0.188	0.176	0.170	0.166	0.162	0.159	0.159	0.156	0.154	0.153
Xinjiang	0.190	0.187	0.183	0.184	0.183	0.182	0.181	0.178	0.175	0.174	0.173	0.169	0.166	0.163	0.162	0.158	0.151	0.147	0.144	0.141	0.137	0.134	0.133	0.129	0.128	0.127	0.125	0.124
Yunnan	0.208	0.207	0.207	0.203	0.199	0.195	0.190	0.184	0.178	0.173	0.168	0.162	0.157	0.151	0.148	0.143	0.137	0.132	0.129	0.126	0.125	0.124	0.123	0.122	0.123	0.122	0.121	0.119
Zhejiang	0.135	0.132	0.130	0.124	0.119	0.114	0.108	0.105	0.103	0.101	0.100	0.096	0.093	0.089	0.086	0.081	0.077	0.073	0.069	0.065	0.063	0.061	0.059	0.058	0.056	0.055	0.054	0.053

Province	County	Code	Urbanicity
Anhui	Bagongshan Qi	340405	Rural
Anhui	Bangshan District	340406	Urban
Anhui	Baohe District	340407	Urban
Anhui	Bowang District	340408	Urban
Anhui	Changfeng County	340409	Rural
Anhui	Chizhou City	340410	Rural
Anhui	Daguan District	340411	Urban
Anhui	Dangshan County	340412	Rural
Anhui	Dangtu County	340413	Rural
Anhui	Datong District	340414	Urban
Anhui	Dingyuan County	340415	Rural
Anhui	Dongzhi County	340416	Rural
Anhui	Duji District	340417	Urban
Anhui	Fangchang County	340418	Rural
Anhui	Feidong County	340419	Rural
Anhui	Feixi County	340420	Rural
Anhui	Fengtai County	340421	Rural
Anhui	Fengyang County	340422	Rural
Anhui	Funan County	340423	Rural
Anhui	Gejiang District	340424	Urban
Anhui	Guangde County	340425	Rural
Anhui	Guoyang County	340426	Rural
Anhui	Guzhen County	340427	Rural
Anhui	Hanshan County	340428	Rural
Anhui	He County	340429	Rural
Anhui	Huaining County	340430	Rural
Anhui	Huaishang District	340431	Urban
Anhui	Huaiyuan County	340432	Rural
Anhui	Huangshan District	340433	Urban
Anhui	Huashan District	340434	Urban
Anhui	Huizhou District	340435	Urban
Anhui	Huoqiu County	340436	Rural
Anhui	Huoshan County	340437	Rural
Anhui	Jiao District	340438	Urban
Anhui	Jieshou City	340439	Rural
Anhui	Jin'an District	340440	Urban
Anhui	Jing County	340441	Rural
Anhui	Jingde County	340442	Rural
Anhui	Jinghu District	340443	Urban
Anhui	Jinzhai County	340444	Rural
Anhui	Jiujiang District	340445	Urban
Anhui	Jixi County	340446	Rural
Anhui	Juchao District	340447	Urban
Anhui	Lai'an County	340448	Rural
Anhui	Langxi County	340449	Rural
Anhui	Langya District	340450	Urban
Anhui	Lieshan District	340451	Urban
Anhui	Lingbi County	340452	Rural
Anhui	Linquan County	340453	Rural

Anhui	Lixin County	340454	Rural
Anhui	Longzihu District	340455	Urban
Anhui	Lujiang County	340456	Rural
Anhui	Luyang District	340457	Urban
Anhui	Mengcheng County	340458	Rural
Anhui	Mingguang City	340459	Rural
Anhui	Nanling County	340460	Rural
Anhui	Nanqiao District	340461	Urban
Anhui	Ningguo City	340462	Rural
Anhui	Panji District	340463	Urban
Anhui	Qianshan County	340464	Rural
Anhui	Qiaocheng District	340465	Urban
Anhui	Qimen County	340466	Rural
Anhui	Qingyang County	340467	Rural
Anhui	Quanjiao County	340468	Rural
Anhui	Samshan District	340469	Urban
Anhui	She County	340470	Rural
Anhui	Shitai County	340471	Rural
Anhui	Shizishan District	340472	Urban
Anhui	Shou County	340473	Rural
Anhui	Shucheng County	340474	Rural
Anhui	Shushan District	340475	Urban
Anhui	Si County	340476	Rural
Anhui	Suixi County	340477	Rural
Anhui	Susong County	340478	Rural
Anhui	Taihe County	340479	Rural
Anhui	Taihu County	340480	Rural
Anhui	Tianchang City	340481	Rural
Anhui	Tianjia'an District	340482	Urban
Anhui	Tongcheng City	340483	Rural
Anhui	Tongguanshan District	340484	Urban
Anhui	Tongling County	340485	Rural
Anhui	Tunxi District	340486	Urban
Anhui	Wangjiang County	340487	Rural
Anhui	Wuhe County	340488	Rural
Anhui	Wuhu County	340489	Rural
Anhui	Wuwei County	340490	Rural
Anhui	Xiangshan District	340491	Urban
Anhui	Xiao County	340492	Rural
Anhui	Xiejiaji qu	340493	Urban
Anhui	Xiuning County	340494	Rural
Anhui	Xuanzhou District	340495	Urban
Anhui	Yaohai District	340496	Urban
Anhui	Yi County	340497	Rural
Anhui	Yingdong District	340498	Urban
Anhui	Yingjiang District	340499	Urban
Anhui	Yingquan District	340500	Urban
Anhui	Yingshang County	340501	Rural
Anhui	Yingzhou District	340502	Urban
Anhui	Yixiu District	340503	Urban

Anhui	Yongqiao District	340504	Urban
Anhui	Yu'an District	340505	Urban
Anhui	Yuexi County	340506	Rural
Anhui	Yuhui District	340507	Urban
Anhui	Yushan District	340508	Urban
Anhui	Zongyang County	340509	Rural
Beijing	Changping District	340510	Urban
Beijing	Chaoyang District	340511	Urban
Beijing	Daxing District	340512	Urban
Beijing	Dongcheng District	340513	Urban
Beijing	Fangshan District	340514	Urban
Beijing	Fengtai District	340515	Urban
Beijing	Haidian District	340516	Urban
Beijing	Huairou District	340517	Urban
Beijing	Mentougou District	340518	Urban
Beijing	Miyun County	340519	Rural
Beijing	Pinggu District	340520	Urban
Beijing	Shijingshan District	340521	Urban
Beijing	Shunyi District	340522	Urban
Beijing	Tongzhou District	340523	Urban
Beijing	Xicheng District	340524	Urban
Beijing	Yanqing County	340525	Rural
Chongqing	Banan District	340526	Urban
Chongqing	Beibei District	340527	Urban
Chongqing	Bishan County	340528	Rural
Chongqing	Changshou District	340529	Urban
Chongqing	Chengkou County	340530	Rural
Chongqing	Dadukou District	340531	Urban
Chongqing	Dianjiang County	340532	Rural
Chongqing	Fengdu County	340533	Rural
Chongqing	Fengjie County	340534	Rural
Chongqing	Fuling District	340535	Urban
Chongqing	Hechuan District	340536	Urban
Chongqing	Jiangbei District	340537	Urban
Chongqing	Jiangjin District	340538	Urban
Chongqing	Jiulongpo District	340539	Urban
Chongqing	Kai County	340540	Rural
Chongqing	Liangping County	340541	Rural
Chongqing	Nan'an District	340542	Urban
Chongqing	Nanchuan District	340543	Urban
Chongqing	Pengshui Miaozu Tujiazu Zizhixian	340544	Rural
Chongqing	Qianjiang District	340545	Urban
Chongqing	Rongchang County	340546	Rural
Chongqing	Shapingba District	340547	Urban
Chongqing	Shizhu Tujiazu Zizhixian	340548	Rural
Chongqing	Shuangqiao District	340549	Urban
Chongqing	Tongliang County	340550	Rural
Chongqing	Tongnan County	340551	Rural
Chongqing	Wansheng District	340552	Urban

Chongqing	Wanzhou District	340553	Urban
Chongqing	Wulong County	340554	Rural
Chongqing	Wushan County	340555	Rural
Chongqing	Wuxi County	340556	Rural
Chongqing	Xiushan Tujiazu Miaozu Zizhixian	340557	Rural
Chongqing	Yongchuan District	340558	Urban
Chongqing	Youyang Tujiazu Miaozu Zizhixian	340559	Rural
Chongqing	Yubei District	340560	Urban
Chongqing	Yunyang County	340561	Rural
Chongqing	Yuzhong District	340562	Urban
Chongqing	Zhong County	340563	Rural
Fujian	Anxi County	340564	Rural
Fujian	Cangshan District	340565	Urban
Fujian	Changle City	340566	Rural
Fujian	Changtai County	340567	Rural
Fujian	Changting County	340568	Rural
Fujian	Chengxiang District	340569	Urban
Fujian	Datian County	340570	Rural
Fujian	Dehua County	340571	Rural
Fujian	Dongshan County	340572	Rural
Fujian	Fengze District	340573	Urban
Fujian	Fu'an City	340574	Rural
Fujian	Fuding City	340575	Rural
Fujian	Fuqing City	340576	Rural
Fujian	Guangze County	340577	Rural
Fujian	Gulou District	340578	Urban
Fujian	Gutian County	340579	Rural
Fujian	Haicang District	340580	Urban
Fujian	Hanjiang District	340581	Urban
Fujian	Hua'an County	340582	Rural
Fujian	Hui'an County	340583	Rural
Fujian	Huli District	340584	Urban
Fujian	Jian'ou City	340585	Rural
Fujian	Jiangle County	340586	Rural
Fujian	Jianning County	340587	Rural
Fujian	Jianyang City	340588	Rural
Fujian	Jiaocheng District	340589	Urban
Fujian	Jimei District	340590	Urban
Fujian	Jin'an District	340591	Urban
Fujian	Jinjiang City	340592	Rural
Fujian	Jinmen County	340593	Rural
Fujian	Liancheng County	340594	Rural
Fujian	Lianjiang County	340595	Rural
Fujian	Licheng District	340596	Urban
Fujian	Licheng District	340597	Urban
Fujian	Longhai City	340598	Rural
Fujian	Longwen District	340599	Urban
Fujian	Luojiang District	340600	Urban

Fujian	Luoyuan County	340601	Rural
Fujian	Mawei District	340602	Urban
Fujian	Meilie District	340603	Urban
Fujian	Mingxi County	340604	Rural
Fujian	Minhou County	340605	Rural
Fujian	Minqing County	340606	Rural
Fujian	Nan'an City	340607	Rural
Fujian	Nanjing County	340608	Rural
Fujian	Ninghua County	340609	Rural
Fujian	Pinghe County	340610	Rural
Fujian	Pingnan County	340611	Rural
Fujian	Pingtan County	340612	Rural
Fujian	Pucheng County	340613	Rural
Fujian	Qingliu County	340614	Rural
Fujian	Quangang District	340615	Urban
Fujian	Sanyuan District	340616	Urban
Fujian	Sha County	340617	Rural
Fujian	Shanghang County	340618	Rural
Fujian	Shaowu City	340619	Rural
Fujian	Shishi City	340620	Rural
Fujian	Shouning County	340621	Rural
Fujian	Shunchang County	340622	Rural
Fujian	Siming District	340623	Urban
Fujian	Songxi County	340624	Rural
Fujian	Taijiang District	340625	Urban
Fujian	Taining County	340626	Rural
Fujian	Tongan District	340627	Urban
Fujian	Wiyishan City	340628	Rural
Fujian	Wuping County	340629	Rural
Fujian	Xiang'an District	340630	Urban
Fujian	Xiangcheng District	340631	Urban
Fujian	Xianyou County	340632	Rural
Fujian	Xiapu County	340633	Rural
Fujian	Xinluo District	340634	Urban
Fujian	Xiuyu District	340635	Urban
Fujian	Yanping District	340636	Urban
Fujian	Yong'an City	340637	Rural
Fujian	Yongchun County	340638	Rural
Fujian	Yongding County	340639	Rural
Fujian	Yongtai County	340640	Rural
Fujian	Youxi County	340641	Rural
Fujian	Yunxiao County	340642	Rural
Fujian	Zhangping City	340643	Rural
Fujian	Zhangpu County	340644	Rural
Fujian	Zhao'an County	340645	Rural
Fujian	Zhenghe County	340646	Rural
Fujian	Zherong County	340647	Rural
Fujian	Zhouning County	340648	Rural
Gansu	Aksay Kazakzu Zizhixian	340649	Rural
Gansu	Anding District	340650	Urban

Gansu	Anning District	340651	Urban
Gansu	Baiyin District	340652	Urban
Gansu	Cheng County	340653	Rural
Gansu	Chengguan District	620102	Urban
Gansu	Chongxin County	620823	Rural
Gansu	Dangchang County	621223	Rural
Gansu	Dongxiangzu Zizhixian	622926	Rural
Gansu	Dunhuang City	620982	Rural
Gansu	Gan'gu County	620523	Rural
Gansu	Ganzhou District	620702	Urban
Gansu	Gaolan County	620122	Rural
Gansu	Gaotai County	620724	Rural
Gansu	Guanghe County	622924	Rural
Gansu	Guazhou County	620922	Rural
Gansu	Gulang County	620622	Rural
Gansu	Heshui County	621024	Rural
Gansu	Hezheng County	622925	Rural
Gansu	Hezuo City	623001	Rural
Gansu	Honggu District	620111	Urban
Gansu	Huachi County	621023	Rural
Gansu	Huan County	621022	Rural
Gansu	Huating County	620824	Rural
Gansu	Hui County	621227	Rural
Gansu	Huining County	620422	Rural
Gansu	Jinchuan District	620302	Urban
Gansu	Jingchuan County	620821	Rural
Gansu	Jingtai County	620423	Rural
Gansu	Jingyuan County	620421	Rural
Gansu	Jinta County	620921	Rural
Gansu	Jishishan Bonanzu Dongxiangzu Salarzu Zizhixian	622927	Rural
Gansu	Jonee County	623022	Rural
Gansu	Kang County	621224	Rural
Gansu	Kangle County	622922	Rural
Gansu	Kongtong District	620802	Urban
Gansu	Li County	621226	Rural
Gansu	Liangdang County	621228	Rural
Gansu	Liangzhou District	620602	Urban
Gansu	Lingtai County	620822	Rural
Gansu	Lintan County	623021	Rural
Gansu	Lintao County	621124	Rural
Gansu	Linxia City	622901	Rural
Gansu	Linxia County	622921	Rural
Gansu	Linze County	620723	Rural
Gansu	Longxi County	621122	Rural
Gansu	Luqu County	623026	Rural
Gansu	Maiji District	620503	Urban
Gansu	Maqu County	623025	Rural
Gansu	Min County	621126	Rural

Gansu	Minle County	620722	Rural
Gansu	Minqin County	620621	Rural
Gansu	Ning County	621026	Rural
Gansu	Pingchuan District	620403	Urban
Gansu	Qilihe District	620103	Urban
Gansu	Qin'an County	620522	Rural
Gansu	Qingcheng County	621021	Rural
Gansu	Qingshui County	620521	Rural
Gansu	Qinzhou District	620502	Urban
Gansu	Shandan County	620725	Rural
Gansu	Shixia District	620201	Urban
Gansu	Subei Mongolzu Zizhixian	620923	Rural
Gansu	Sunan Yugurzu Zizhixian	620721	Rural
Gansu	Suzhou District	620902	Urban
Gansu	Teewo County	623024	Rural
Gansu	Tianzhu Zangzu Zizhixian	620623	Rural
Gansu	Tongwei County	621121	Rural
Gansu	Weiyuan County	621123	Rural
Gansu	Wen County	621222	Rural
Gansu	Wudu District	621202	Urban
Gansu	Wushan County	620524	Rural
Gansu	Xiahe County	623027	Rural
Gansu	Xifeng District	621002	Urban
Gansu	Xigu District	620104	Urban
Gansu	Xihe County	621225	Rural
Gansu	Yongchang County	620321	Rural
Gansu	Yongdeng County	620121	Rural
Gansu	Yongjing County	622923	Rural
Gansu	Yumen City	620981	Rural
Gansu	Yuzhong County	620123	Rural
Gansu	Zhang County	621125	Rural
Gansu	Zhangjiachuan Huizu Zizhixian	620525	Rural
Gansu	Zhengning County	621025	Rural
Gansu	Zhenyuan County	621027	Rural
Gansu	Zhuanglang County	620825	Rural
Gansu	Zhugqu County	623023	Rural
Gansu	jingning County	620826	Rural
Guangdong	Baiyun District	440111	Urban
Guangdong	Baoan District	440306	Urban
Guangdong	Boluo County	441322	Rural
Guangdong	Chancheng District	440604	Urban
Guangdong	Chaoan County	445121	Rural
Guangdong	Chaonan District	440514	Urban
Guangdong	Chaoyang District	440513	Urban
Guangdong	Chenghai District	440515	Urban
Guangdong	Chengqu	441502	Urban
Guangdong	Chikan District	440802	Urban
Guangdong	Conghua City	440184	Rural
Guangdong	Dapu County	441422	Rural

Guangdong	Deqing County	441226	Rural
Guangdong	Dianbai County	440923	Rural
Guangdong	Dinghu District	441203	Urban
Guangdong	Dongguan City	441900	Rural
Guangdong	Dongyuan County	441625	Rural
Guangdong	Doumen District	440403	Urban
Guangdong	Duanzhou District	441202	Urban
Guangdong	Enping City	440785	Rural
Guangdong	Fengkai County	441225	Rural
Guangdong	Fengshun County	441423	Rural
Guangdong	Fogang County	441821	Rural
Guangdong	Futian District	440304	Urban
Guangdong	Gaoming District	440608	Urban
Guangdong	Gaoyao City	441283	Rural
Guangdong	Gaozhou City	440981	Rural
Guangdong	Guangning County	441223	Rural
Guangdong	Haifeng County	441521	Rural
Guangdong	Haizhu District	440105	Urban
Guangdong	Haojiang District	440512	Urban
Guangdong	Heping County	441624	Rural
Guangdong	Heshan City	440784	Rural
Guangdong	Huadu District	440114	Urban
Guangdong	Huaiji County	441224	Rural
Guangdong	Huangpu District	440112	Urban
Guangdong	Huazhou City	440982	Rural
Guangdong	Huicheng District	441302	Urban
Guangdong	Huidong County	441323	Rural
Guangdong	Huilai County	445224	Rural
Guangdong	Huiyang District	441303	Urban
Guangdong	Jiangcheng District	441702	Urban
Guangdong	Jianghai District	440704	Urban
Guangdong	Jiaoling County	441427	Rural
Guangdong	Jiedong County	445221	Rural
Guangdong	Jiexi County	445222	Rural
Guangdong	Jinping District	440511	Urban
Guangdong	Jinwan District	440404	Urban
Guangdong	Kaiping City	440783	Rural
Guangdong	Lechang City	440281	Rural
Guangdong	Leizhou City	440882	Rural
Guangdong	Lianjiang City	440881	Rural
Guangdong	Liannan Yaozu Zizhixian	441826	Rural
Guangdong	Lianping County	441623	Rural
Guangdong	Lianshan Zhuangzu Yaozu Zizhixian	441825	Rural
Guangdong	Lianzhou City	441882	Rural
Guangdong	Liwan District	440103	Urban
Guangdong	Longchuan County	441622	Rural
Guangdong	Longgang District	440307	Urban
Guangdong	Longhu District	440507	Urban
Guangdong	Longmen County	441324	Rural

Guangdong	Lufeng City	441581	Rural
Guangdong	Luhe County	441523	Rural
Guangdong	Luoding City	445381	Rural
Guangdong	Luogang District	440116	Urban
Guangdong	Luohu District	440303	Urban
Guangdong	Maogang District	440903	Urban
Guangdong	Maonan District	440902	Urban
Guangdong	Mazhang District	440811	Urban
Guangdong	Mei County	441421	Rural
Guangdong	Meijiang District	441402	Urban
Guangdong	Nan'ao County	440523	Rural
Guangdong	Nanhai District	440605	Urban
Guangdong	Nansha District	440115	Urban
Guangdong	Nanshan District	440305	Urban
Guangdong	Nanxiong City	440282	Rural
Guangdong	Panyu District	440113	Urban
Guangdong	Pengjiang District	440703	Urban
Guangdong	Pingyuan County	441426	Rural
Guangdong	Potou District	440804	Urban
Guangdong	Puning City	445281	Rural
Guangdong	Qingcheng District	441802	Urban
Guangdong	Qingxin County	441827	Rural
Guangdong	Qujiang District	440205	Urban
Guangdong	Raoping County	445122	Rural
Guangdong	Renhua County	440224	Rural
Guangdong	Rongcheng District	445202	Urban
Guangdong	Ruyuan Yaozu Zizhixian	440232	Rural
Guangdong	Sanshui District	440607	Urban
Guangdong	Shixing County	440222	Rural
Guangdong	Shunde District	440606	Urban
Guangdong	Sihui City	441284	Rural
Guangdong	Suixi County	440823	Rural
Guangdong	Taishan City	440781	Rural
Guangdong	Tianhe District	440106	Urban
Guangdong	Wengyuan County	440229	Rural
Guangdong	Wuchuan City	440883	Rural
Guangdong	Wuhua County	441424	Rural
Guangdong	Wujiang District	440203	Urban
Guangdong	Xiangqiao District	445102	Urban
Guangdong	Xiangzhou District	440402	Urban
Guangdong	Xiashan District	440803	Urban
Guangdong	Xinfeng County	440233	Rural
Guangdong	Xingning City	441481	Rural
Guangdong	Xinhui District	440705	Urban
Guangdong	Xinxing County	445321	Rural
Guangdong	Xinyi City	440983	Rural
Guangdong	Xuwen County	440825	Rural
Guangdong	Yangchun City	441781	Rural
Guangdong	Yangdong County	441723	Rural
Guangdong	Yangshan County	441823	Rural

Guangdong	Yangxi County	441721	Rural
Guangdong	Yantian District	440308	Urban
Guangdong	Yingde City	441881	Rural
Guangdong	Yu'nan County	445322	Rural
Guangdong	Yuancheng District	441602	Urban
Guangdong	Yuexiu District	440104	Urban
Guangdong	Yun'an County	445323	Rural
Guangdong	Yuncheng District	445302	Urban
Guangdong	Zengcheng City	440183	Rural
Guangdong	Zhenjiang District	440204	Urban
Guangdong	Zhongshan City	442000	Rural
Guangdong	Zijin County	441621	Rural
Guangxi	Babu District	451102	Urban
Guangxi	Bama Yaozu Zizhixian	451227	Rural
Guangxi	Beiliu City	450981	Rural
Guangxi	Binyang County	450126	Rural
Guangxi	Bobai County	450923	Rural
Guangxi	Cangwu County	450421	Rural
Guangxi	Cenxi City	450481	Rural
Guangxi	Changzhou District	450405	Urban
Guangxi	Chengzhong District	450202	Urban
Guangxi	Dahua Yaozu Zizhixian	451229	Rural
Guangxi	Daxin County	451424	Rural
Guangxi	Debao County	451024	Rural
Guangxi	Diecai District	450303	Urban
Guangxi	Dieshan District	450404	Urban
Guangxi	Donglan County	451224	Rural
Guangxi	Dongxing City	450681	Rural
Guangxi	Du'an Yaozu Zizhixian	451228	Rural
Guangxi	Fangcheng District	450603	Urban
Guangxi	Fengshan County	451223	Rural
Guangxi	Fuchuan Yaozu Zizhixian	451123	Rural
Guangxi	Fusui County	451421	Rural
Guangxi	Gangbei District	450802	Urban
Guangxi	Gangkou District	450602	Urban
Guangxi	Gangnan District	450803	Urban
Guangxi	Gongcheng Yaozu Zizhixian	450332	Rural
Guangxi	Guanyang County	450327	Rural
Guangxi	Guiping City	450881	Rural
Guangxi	Haicheng District	450502	Urban
Guangxi	Heng County	450127	Rural
Guangxi	Hepu County	450521	Rural
Guangxi	Heshan City	451381	Rural
Guangxi	Huanjiang Maonanzu Zizhixian	451226	Rural
Guangxi	Jiangnan District	450105	Urban
Guangxi	Jiangzhou District	451402	Urban
Guangxi	Jinchengjiang District	451202	Urban
Guangxi	Jingxi County	451025	Rural
Guangxi	Jinxiu Yaozu Zizhixian	451324	Rural

Guangxi	Laibin District	451302	Urban
Guangxi	Leye County	451028	Rural
Guangxi	Liangqing District	450108	Urban
Guangxi	Lin'gui County	450322	Rural
Guangxi	Lingchuan County	450323	Rural
Guangxi	Lingshan County	450721	Rural
Guangxi	Lingyun County	451027	Rural
Guangxi	Lipu County	450331	Rural
Guangxi	Liubei District	450205	Urban
Guangxi	Liucheng County	450222	Rural
Guangxi	Liujiang County	450221	Rural
Guangxi	Liunan District	450204	Urban
Guangxi	Long'an County	450123	Rural
Guangxi	Longlin Gezu Zizhixian	451031	Rural
Guangxi	Longsheng Gezu Zizhixian	450328	Rural
Guangxi	Longzhou County	451423	Rural
Guangxi	Luchuan County	450922	Rural
Guangxi	Luocheng Mulaozu Zizhixian	451225	Rural
Guangxi	Luzhai County	450223	Rural
Guangxi	Mashan County	450124	Rural
Guangxi	Meng shan County	450423	Rural
Guangxi	Nandan County	451221	Rural
Guangxi	Napo County	451026	Rural
Guangxi	Ningming County	451422	Rural
Guangxi	Pingguo County	451023	Rural
Guangxi	Pingle County	450330	Rural
Guangxi	Pingnan County	450821	Rural
Guangxi	Pingxiang City	451481	Rural
Guangxi	Pubei County	450722	Rural
Guangxi	Qinbei District	450703	Urban
Guangxi	Qingxiu District	450103	Urban
Guangxi	Qinnan District	450702	Urban
Guangxi	Qixing District	450305	Urban
Guangxi	Quanzhou County	450324	Rural
Guangxi	Rong County	450921	Rural
Guangxi	Rong'an County	450224	Rural
Guangxi	Rongshui Miaozu Zizhixian	450225	Rural
Guangxi	Sanjiang Tongzu Zizhixian	450226	Rural
Guangxi	Shanglin County	450125	Rural
Guangxi	Shangsi County	450621	Rural
Guangxi	Tantang District	450804	Urban
Guangxi	Teng County	450422	Rural
Guangxi	Tian'e County	451222	Rural
Guangxi	Tiandeng County	451425	Rural
Guangxi	Tiandong County	451022	Rural
Guangxi	Tianlin County	451029	Rural
Guangxi	Tianyang County	451021	Rural
Guangxi	Tieshangang District	450512	Urban
Guangxi	Wanxiu District	450403	Urban
Guangxi	Wuming County	450122	Rural

Guangxi	Wuxuan County	451323	Rural
Guangxi	Xiangshan District	450304	Urban
Guangxi	Xiangzhou County	451322	Rural
Guangxi	Xilin County	451030	Rural
Guangxi	Xincheng County	451321	Rural
Guangxi	Xing'an County	450325	Rural
Guangxi	Xingning District	450102	Urban
Guangxi	Xingye County	450924	Rural
Guangxi	Xiufeng District	450302	Urban
Guangxi	Xixiangtang District	450107	Urban
Guangxi	Yangshuo County	450321	Rural
Guangxi	Yanshan District	450311	Urban
Guangxi	Yinhai District	450503	Urban
Guangxi	Yizhou City	451281	Rural
Guangxi	Yongfu County	450326	Rural
Guangxi	Yongning District	450109	Urban
Guangxi	Youjiang District	451002	Urban
Guangxi	Yufeng District	450203	Urban
Guangxi	Yuzhou District	450902	Urban
Guangxi	Zhaoping County	451121	Rural
Guangxi	Zhongshan County	451122	Rural
Guangxi	Ziyuan County	450329	Rural
Guizhou	Anlong County	522328	Rural
Guizhou	Baiyun District	520113	Urban
Guizhou	Bijie City	520502	Rural
Guizhou	Ceheng County	522327	Rural
Guizhou	Cen'gong County	522626	Rural
Guizhou	Changshun County	522729	Rural
Guizhou	Chishui City	520381	Rural
Guizhou	Congjiang County	522633	Rural
Guizhou	Dafang County	520521	Rural
Guizhou	Danzhai County	522636	Rural
Guizhou	Daozhen Gelaozu Miaozu Zizhixian	520325	Rural
Guizhou	Dejiang County	520626	Rural
Guizhou	Dushan County	522726	Rural
Guizhou	Duyun City	522701	Rural
Guizhou	Fenggang County	520327	Rural
Guizhou	Fuquan City	522702	Rural
Guizhou	Guanling Buyizu Miaozu Zizhixian	520424	Rural
Guizhou	Guiding County	522723	Rural
Guizhou	Hezhang County	520527	Rural
Guizhou	Honghuagang District	520302	Urban
Guizhou	Huangping County	522622	Rural
Guizhou	Huaxi District	520111	Urban
Guizhou	Huichuan District	520303	Urban
Guizhou	Huishui County	522731	Rural
Guizhou	Jiangkou County	520621	Rural
Guizhou	Jianhe County	522629	Rural

Guizhou	Jinping County	522628	Rural
Guizhou	Jinsha County	520523	Rural
Guizhou	Kaili City	522601	Rural
Guizhou	Kaiyang County	520121	Rural
Guizhou	Leishan County	522634	Rural
Guizhou	Libo County	522722	Rural
Guizhou	Liping County	522631	Rural
Guizhou	Longli County	522730	Rural
Guizhou	Luodian County	522728	Rural
Guizhou	Luzhi Tequ	520203	Urban
Guizhou	Majiang County	522635	Rural
Guizhou	Meitan County	520328	Rural
Guizhou	Nanming District	520102	Urban
Guizhou	Nayong County	520525	Rural
Guizhou	Pan County	520222	Rural
Guizhou	Pingba County	520421	Rural
Guizhou	Pingtang County	522727	Rural
Guizhou	Pu'an County	522323	Rural
Guizhou	Puding County	520422	Rural
Guizhou	Qianxi County	520522	Rural
Guizhou	Qinglong County	522324	Rural
Guizhou	Qingzhen City	520181	Rural
Guizhou	Renhuai City	520382	Rural
Guizhou	Rongjiang County	522632	Rural
Guizhou	Sandu Shuizu Zizhixian	522732	Rural
Guizhou	Sansui County	522624	Rural
Guizhou	Shibing County	522623	Rural
Guizhou	Shiqian County	520623	Rural
Guizhou	Shuicheng County	520221	Rural
Guizhou	Sinan County	520624	Rural
Guizhou	Songtao Miaozu Zizhixian	520628	Rural
Guizhou	Suiyang County	520323	Rural
Guizhou	Taijiang County	522630	Rural
Guizhou	Tianzhu County	522627	Rural
Guizhou	Tongren City	520602	Rural
Guizhou	Tongzi County	520322	Rural
Guizhou	Wangmo County	522326	Rural
Guizhou	Wanshan Tequ	520603	Urban
Guizhou	Weining Yizu Huizu Miaozu Zizhixian	520526	Rural
Guizhou	Weng'an County	522725	Rural
Guizhou	Wuchuan Gelaozu Miaozu Zizhixian	520326	Rural
Guizhou	Wudang District	520112	Urban
Guizhou	Xiaohe District	520114	Urban
Guizhou	Xifeng County	520122	Rural
Guizhou	Xingren County	522322	Rural
Guizhou	Xingyi City	522301	Rural
Guizhou	Xishui County	520330	Rural
Guizhou	Xiuwen County	520123	Rural

Guizhou	Xixiu District	520402	Urban
Guizhou	Yanhe Tujiazu Zizhixian	520627	Rural
Guizhou	Yinjiang Tujia Miaozu Zizhixian	520625	Rural
Guizhou	Yunyan District	520103	Urban
Guizhou	Yuping Dongzu Zizhixian	520622	Rural
Guizhou	Yuqing County	520329	Rural
Guizhou	Zhenfeng County	522325	Rural
Guizhou	Zheng'an County	520324	Rural
Guizhou	Zhenning Buyizu Miaozu Zizhixian	520423	Rural
Guizhou	Zhenyuan County	522625	Rural
Guizhou	Zhijin County	520524	Rural
Guizhou	Zhongshan District	520201	Urban
Guizhou	Ziyun Miaozu Buyizu Zizhixian	520425	Rural
Guizhou	Zunyi County	520321	Rural
Hainan	Baisha Lizu Zizhixian	469025	Rural
Hainan	Baoting LiMiaozu Zizhixian	469029	Rural
Hainan	Changjiang Lizu Zizhixian	469026	Rural
Hainan	Chengmai County	469023	Rural
Hainan	Danzhou City	469003	Rural
Hainan	Ding'an County	469021	Rural
Hainan	Dongfang City	469007	Rural
Hainan	Ledong Lizu Zizhixian	469027	Rural
Hainan	Lingao County	469024	Rural
Hainan	Lingshui Lizu Zizhixian	469028	Rural
Hainan	Longhua District	460106	Urban
Hainan	Meilan District	460108	Urban
Hainan	Qionghai City	469002	Rural
Hainan	Qiongshan District	460107	Urban
Hainan	Qiongzhong LiMiaozu Zizhixian	469030	Rural
Hainan	Tunchang County	469022	Rural
Hainan	Wanning City	469006	Rural
Hainan	Wenchang City	469005	Rural
Hainan	Wuzhishan City	469001	Rural
Hainan	Xiuying District	460105	Urban
Hainan	sanya shi	460201	Urban
Hebei	Anci District	131002	Urban
Hebei	Anguo City	130683	Rural
Hebei	Anping County	131125	Rural
Hebei	Anxin County	130632	Rural
Hebei	Baixiang County	130524	Rural
Hebei	Bazhou City	131081	Rural
Hebei	Beidaihe District	130304	Urban
Hebei	Beishi District	130603	Urban
Hebei	Botou City	130981	Rural
Hebei	Boye County	130637	Rural
Hebei	Cang County	130921	Rural

Hebei	Caofeidian District	130209	Urban
Hebei	Changan District	130102	Urban
Hebei	Changli County	130322	Rural
Hebei	Cheng'an County	130424	Rural
Hebei	Chengde County	130821	Rural
Hebei	Chicheng County	130732	Rural
Hebei	Chongli County	130733	Rural
Hebei	Ci County	130427	Rural
Hebei	Congtai District	130403	Urban
Hebei	Dachang Huizu Zizhixian	131028	Rural
Hebei	Dacheng County	131025	Rural
Hebei	Daming County	130425	Rural
Hebei	Dingxing County	130626	Rural
Hebei	Dingzhou City	130682	Rural
Hebei	Dongguang County	130923	Rural
Hebei	Dongkuang District	130204	Urban
Hebei	Feixiang County	130428	Rural
Hebei	Fengfengkuang District	130406	Urban
Hebei	Fengnan District	130207	Urban
Hebei	Fengning Manzu Zizhixian	130826	Rural
Hebei	Fengrun District	130208	Urban
Hebei	Fucheng County	131128	Rural
Hebei	Funing County	130323	Rural
Hebei	Fuping County	130624	Rural
Hebei	Fuxing District	130404	Urban
Hebei	Gaobeidian City	130684	Rural
Hebei	Gaocheng City	130182	Rural
Hebei	Gaoyang County	130628	Rural
Hebei	Gaoyi County	130127	Rural
Hebei	Gu'an County	131022	Rural
Hebei	Guangping County	130432	Rural
Hebei	Guangyang District	131003	Urban
Hebei	Guangzong County	130531	Rural
Hebei	Guantao County	130433	Rural
Hebei	Gucheng County	131126	Rural
Hebei	Guyuan County	130724	Rural
Hebei	Haigang District	130302	Urban
Hebei	Haixing County	130924	Rural
Hebei	Handan County	130421	Rural
Hebei	Hanshan District	130402	Urban
Hebei	Hejian City	130984	Rural
Hebei	Huai'an County	130728	Rural
Hebei	Huailai County	130730	Rural
Hebei	Huanghua City	130983	Rural
Hebei	Jing County	131127	Rural
Hebei	Jingxing County	130121	Rural
Hebei	Jingxingkuang District	130107	Urban
Hebei	Jinzhou City	130183	Rural
Hebei	Jize County	130431	Rural
Hebei	Jizhou City	131181	Rural

Hebei	Julu County	130529	Rural
Hebei	Kaiping District	130205	Urban
Hebei	Kangbao County	130723	Rural
Hebei	Kuancheng Manzu Zizhixian	130827	Rural
Hebei	Laishui County	130623	Rural
Hebei	Laiyuan County	130630	Rural
Hebei	Leting County	130225	Rural
Hebei	Li County	130635	Rural
Hebei	Lincheng County	130522	Rural
Hebei	Lingshou County	130126	Rural
Hebei	Linxi County	130535	Rural
Hebei	Linzhang County	130423	Rural
Hebei	Longhua County	130825	Rural
Hebei	Longyao County	130525	Rural
Hebei	Luan County	130223	Rural
Hebei	Luancheng County	130124	Rural
Hebei	Luannan County	130224	Rural
Hebei	Luanping County	130824	Rural
Hebei	Lubei District	130203	Urban
Hebei	Lulong County	130324	Rural
Hebei	Lunan District	130202	Urban
Hebei	Luquan City	130185	Rural
Hebei	Mancheng County	130621	Rural
Hebei	Mengcun Huizu Zizhixian	130930	Rural
Hebei	Nangong City	130581	Rural
Hebei	Nanhe County	130527	Rural
Hebei	Nanpi County	130927	Rural
Hebei	Nanshi District	130604	Urban
Hebei	Neiqiu County	130523	Rural
Hebei	Ningjin County	130528	Rural
Hebei	Pingquan County	130823	Rural
Hebei	Pingshan County	130131	Rural
Hebei	Pingxiang County	130532	Rural
Hebei	Qian'an City	130283	Rural
Hebei	Qianxi County	130227	Rural
Hebei	Qiaodong District	130103	Urban
Hebei	Qiaodong District	130502	Urban
Hebei	Qiaodong District	130702	Urban
Hebei	Qiaoxi District	130104	Urban
Hebei	Qiaoxi District	130503	Urban
Hebei	Qiaoxi District	130703	Urban
Hebei	Qing County	130922	Rural
Hebei	Qinghe County	130534	Rural
Hebei	Qinglong Manzu Zizhixian	130321	Rural
Hebei	Qingyuan County	130622	Rural
Hebei	Qiu County	130430	Rural
Hebei	Quyang County	130634	Rural
Hebei	Quzhou County	130435	Rural
Hebei	Raoyang County	131124	Rural
Hebei	Ren County	130526	Rural

Hebei	Renqiu City	130982	Rural
Hebei	Rongcheng County	130629	Rural
Hebei	Sanhe City	131082	Rural
Hebei	Shahe City	130582	Rural
Hebei	Shangyi County	130725	Rural
Hebei	Shanhaiguan District	130303	Urban
Hebei	She County	130426	Rural
Hebei	Shenze County	130128	Rural
Hebei	Shenzhou City	131182	Rural
Hebei	Shuangluan District	130803	Urban
Hebei	Shuangqiao District	130802	Urban
Hebei	Shunping County	130636	Rural
Hebei	Suning County	130926	Rural
Hebei	Tang County	130627	Rural
Hebei	Taocheng District	131102	Urban
Hebei	Wangdu County	130631	Rural
Hebei	Wanquan County	130729	Rural
Hebei	Wei County	130434	Rural
Hebei	Wei County	130533	Rural
Hebei	Weichang Manzu Mongolzu Zizhixian	130828	Rural
Hebei	Wen'an County	131026	Rural
Hebei	Wu'an City	130481	Rural
Hebei	Wuji County	130130	Rural
Hebei	Wuqiang County	131123	Rural
Hebei	Wuqiao County	130928	Rural
Hebei	Wuyi County	131122	Rural
Hebei	Xiahuayuan District	130706	Urban
Hebei	Xian County	130929	Rural
Hebei	Xianghe County	131024	Rural
Hebei	Xinglong County	130822	Rural
Hebei	Xingtai County	130521	Rural
Hebei	Xingtang County	130125	Rural
Hebei	Xinhe County	130530	Rural
Hebei	Xinhua District	130105	Urban
Hebei	Xinhua District	130902	Urban
Hebei	Xinji City	130181	Rural
Hebei	Xinle City	130184	Rural
Hebei	Xinshi District	130602	Urban
Hebei	Xiong County	130638	Rural
Hebei	Xuanhua District	130705	Urban
Hebei	Xuanhua County	130721	Rural
Hebei	Xushui County	130625	Rural
Hebei	Yangyuan County	130727	Rural
Hebei	Yanshan County	130925	Rural
Hebei	Yi County	130633	Rural
Hebei	Yingshouyingzikuang District	130804	Urban
Hebei	Yongnian County	130429	Rural
Hebei	Yongqing County	131023	Rural
Hebei	Yu County	130726	Rural

Hebei	Yuanshi County	130132	Rural
Hebei	Yuhua District	130108	Urban
Hebei	Yunhe District	130903	Urban
Hebei	Yutian County	130229	Rural
Hebei	Zanhuang County	130129	Rural
Hebei	Zaoqiang County	131121	Rural
Hebei	Zhangbei County	130722	Rural
Hebei	Zhao County	130133	Rural
Hebei	Zhengding County	130123	Rural
Hebei	Zhuolu County	130731	Rural
Hebei	Zhuozhou City	130681	Rural
Hebei	Zunhua City	130281	Rural
Heilongjiar	Acheng District	230112	Urban
Heilongjiar	Aihui District	231102	Urban
Heilongjiar	Aimin District	231004	Urban
Heilongjiar	Anda City	231281	Rural
Heilongjiar	Ang'angxi District	230205	Urban
Heilongjiar	Baiquan County	230231	Rural
Heilongjiar	Baoqing County	230523	Rural
Heilongjiar	Baoshan District	230506	Urban
Heilongjiar	Bayan County	230126	Rural
Heilongjiar	Bei'an City	231181	Rural
Heilongjiar	Beilin District	231202	Urban
Heilongjiar	Bin County	230125	Rural
Heilongjiar	Boli County	230921	Rural
Heilongjiar	Chengzihe District	230306	Urban
Heilongjiar	Cuiluan District	230706	Urban
Heilongjiar	Dailing District	230713	Urban
Heilongjiar	Daoli District	230102	Urban
Heilongjiar	Daowai District	230104	Urban
Heilongjiar	Datong District	230606	Urban
Heilongjiar	Didao District	230304	Urban
Heilongjiar	Dong'an District	231002	Urban
Heilongjiar	Dongfeng District	230805	Urban
Heilongjiar	Dongfeng District	230811	Urban
Heilongjiar	Dongning County	231024	Rural
Heilongjiar	Dongshan District	230406	Urban
Heilongjiar	Dorbod Mongolzu Zizhixian	230624	Rural
Heilongjiar	Fangzheng County	230124	Rural
Heilongjiar	Fujin City	230882	Rural
Heilongjiar	Fuyu County	230227	Rural
Heilongjiar	Fuyuan County	230833	Rural
Heilongjiar	Gannan County	230225	Rural
Heilongjiar	Gongnong District	230403	Urban
Heilongjiar	Hailin City	231083	Rural
Heilongjiar	Hailun City	231283	Rural
Heilongjiar	Hengshan District	230303	Urban
Heilongjiar	Honggang District	230605	Urban
Heilongjiar	Hongxing District	230715	Urban
Heilongjiar	Huachuan County	230826	Rural

Heilongjiar	Huanan County	230822	Rural
Heilongjiar	Hulan Ergi District	230206	Urban
Heilongjiar	Hulan District	230111	Urban
Heilongjiar	Hulin City	230381	Rural
Heilongjiar	Huma County	232721	Rural
Heilongjiar	Jianhua District	230203	Urban
Heilongjiar	Jianshan District	230502	Urban
Heilongjiar	Jiayin County	230722	Rural
Heilongjiar	Jidong County	230321	Rural
Heilongjiar	Jiguan District	230302	Urban
Heilongjiar	Jinshantun District	230709	Urban
Heilongjiar	Jixian County	230521	Rural
Heilongjiar	Kedong County	230230	Rural
Heilongjiar	Keshan County	230229	Rural
Heilongjiar	Lanxi County	231222	Rural
Heilongjiar	Lindian County	230623	Rural
Heilongjiar	Lingdong District	230503	Urban
Heilongjiar	Linkou County	231025	Rural
Heilongjiar	Lishu District	230305	Urban
Heilongjiar	Longfeng District	230603	Urban
Heilongjiar	Longjiang County	230221	Rural
Heilongjiar	Longsha District	230202	Urban
Heilongjiar	Luobei County	230421	Rural
Heilongjiar	Mashan District	230307	Urban
Heilongjiar	Meilisi Daurzu District	230208	Urban
Heilongjiar	Meixi District	230708	Urban
Heilongjiar	Mingshui County	231225	Rural
Heilongjiar	Mishan City	230382	Rural
Heilongjiar	Mohe County	232723	Rural
Heilongjiar	Mulan County	230127	Rural
Heilongjiar	Muleng City	231085	Rural
Heilongjiar	Nancha District	230703	Urban
Heilongjiar	Nangang District	230103	Urban
Heilongjiar	Nanshan District	230404	Urban
Heilongjiar	Nehe City	230281	Rural
Heilongjiar	Nenjiang County	231121	Rural
Heilongjiar	Nianzishan District	230207	Urban
Heilongjiar	Ning'an City	231084	Rural
Heilongjiar	Pingfang District	230108	Urban
Heilongjiar	Qianjin District	230804	Urban
Heilongjiar	Qiezihe District	230904	Urban
Heilongjiar	Qing'an County	231224	Rural
Heilongjiar	Qinggang County	231223	Rural
Heilongjiar	Ranghulu District	230604	Urban
Heilongjiar	Raohe County	230524	Rural
Heilongjiar	Sairt District	230602	Urban
Heilongjiar	Shangganling District	230716	Urban
Heilongjiar	Shangzhi City	230183	Rural
Heilongjiar	Shuangcheng City	230182	Rural
Heilongjiar	Sifangtai District	230505	Urban

Heilongjiar	Songbei District	230109	Urban
Heilongjiar	Suibin County	230422	Rural
Heilongjiar	Suifenhe City	231081	Rural
Heilongjiar	Suileng County	231226	Rural
Heilongjiar	Sunwu County	231124	Rural
Heilongjiar	Tahe County	232722	Rural
Heilongjiar	Tailai County	230224	Rural
Heilongjiar	Tangwanghe District	230712	Urban
Heilongjiar	Tangyuan County	230828	Rural
Heilongjiar	Taoshan District	230903	Urban
Heilongjiar	Tiefeng District	230204	Urban
Heilongjiar	Tieli City	230781	Rural
Heilongjiar	Tonghe County	230128	Rural
Heilongjiar	Tongjiang City	230881	Rural
Heilongjiar	Wangkui County	231221	Rural
Heilongjiar	Wuchang City	230184	Rural
Heilongjiar	Wudalianchi City	231182	Rural
Heilongjiar	Wumahe District	230711	Urban
Heilongjiar	Wuyiling District	230714	Urban
Heilongjiar	Wuying District	230710	Urban
Heilongjiar	Xi'an District	231005	Urban
Heilongjiar	Xiangfang District	230110	Urban
Heilongjiar	Xiangyang District	230402	Urban
Heilongjiar	Xiangyang District	230803	Urban
Heilongjiar	Xilin District	230705	Urban
Heilongjiar	Xingan District	230405	Urban
Heilongjiar	Xingshan District	230407	Urban
Heilongjiar	Xinqing District	230707	Urban
Heilongjiar	Xinxing District	230902	Urban
Heilongjiar	Xunke County	231123	Rural
Heilongjiar	Yangming District	231003	Urban
Heilongjiar	Yanshou County	230129	Rural
Heilongjiar	Yi'an County	230223	Rural
Heilongjiar	Yichun District	230702	Urban
Heilongjiar	Yilan County	230123	Rural
Heilongjiar	Youhao District	230704	Urban
Heilongjiar	Youyi County	230522	Rural
Heilongjiar	Zhaodong City	231282	Rural
Heilongjiar	Zhaoyuan County	230622	Rural
Heilongjiar	Zhaozhou County	230621	Rural
Henan	Anyang County	410522	Rural
Henan	Baofeng County	410421	Rural
Henan	Beiguan District	410503	Urban
Henan	Biyang County	411726	Rural
Henan	Bo'ai County	410822	Rural
Henan	Changge City	411082	Rural
Henan	Changyuan County	410728	Rural
Henan	Chanhe Huizu District	410304	Urban
Henan	Chuanhui District	411602	Urban
Henan	Dancheng County	411625	Rural

Henan	Dengfeng City	410185	Rural
Henan	Dengzhou City	411381	Rural
Henan	Erqi District	410103	Urban
Henan	Fan County	410926	Rural
Henan	Fangcheng County	411322	Rural
Henan	Fengqiu County	410727	Rural
Henan	Fengquan District	410704	Urban
Henan	Fugou County	411621	Rural
Henan	Gongyi City	410181	Rural
Henan	Guancheng Huizu District	410104	Urban
Henan	Guangshan County	411522	Rural
Henan	Gulou District	410204	Urban
Henan	Gushi County	411525	Rural
Henan	Heshan District	410602	Urban
Henan	Hongqi District	410702	Urban
Henan	Hua County	410526	Rural
Henan	Huaibin County	411527	Rural
Henan	Huaiyang County	411626	Rural
Henan	Hualong District	410902	Urban
Henan	Huangchuan County	411526	Rural
Henan	Hubin District	411202	Urban
Henan	Huiji District	410108	Urban
Henan	Huixian City	410782	Rural
Henan	Huojia County	410724	Rural
Henan	Jia County	410425	Rural
Henan	Jianxi District	410305	Urban
Henan	Jiefang District	410802	Urban
Henan	Jili District	410306	Urban
Henan	Jinming District	410211	Urban
Henan	Jinshui District	410105	Urban
Henan	Jiyuan City(XianJi)	419001	Rural
Henan	Kaifeng County	410224	Rural
Henan	Lankao County	410225	Rural
Henan	Laocheng District	410302	Urban
Henan	Liangyuan District	411402	Urban
Henan	Lingbao City	411282	Rural
Henan	Linying County	411122	Rural
Henan	Linzhou City	410581	Rural
Henan	Liwangtai District	410205	Urban
Henan	Long'an District	410506	Urban
Henan	Longting District	410202	Urban
Henan	Luanchuan County	410324	Rural
Henan	Luolong District	410311	Urban
Henan	Luoning County	410328	Rural
Henan	Luoshan County	411521	Rural
Henan	Lushan County	410423	Rural
Henan	Lushi County	411224	Rural
Henan	Luyi County	411628	Rural
Henan	Macun District	410804	Urban
Henan	Mengjin County	410322	Rural

Henan	Mengzhou City	410883	Rural
Henan	Mianchi County	411221	Rural
Henan	Minquan County	411421	Rural
Henan	Muye District	410711	Urban
Henan	Nanle County	410923	Rural
Henan	Nanzhao County	411321	Rural
Henan	Neihuang County	410527	Rural
Henan	Neixiang County	411325	Rural
Henan	Ningling County	411423	Rural
Henan	Pingqiao District	411503	Urban
Henan	Pingyu County	411723	Rural
Henan	Puyang County	410928	Rural
Henan	Qi County	410221	Rural
Henan	Qi County	410622	Rural
Henan	Qibin District	410611	Urban
Henan	Qingfeng County	410922	Rural
Henan	Qinyang City	410882	Rural
Henan	Queshan County	411725	Rural
Henan	Runan County	411727	Rural
Henan	Ruyang County	410326	Rural
Henan	Ruzhou City	410482	Rural
Henan	Shan County	411222	Rural
Henan	Shancheng District	410603	Urban
Henan	Shangcai County	411722	Rural
Henan	Shangcheng County	411524	Rural
Henan	Shangjie District	410106	Urban
Henan	Shangshui County	411623	Rural
Henan	Shanyang District	410811	Urban
Henan	Shenqiu County	411624	Rural
Henan	Sheqi County	411327	Rural
Henan	Shihe District	411502	Urban
Henan	Shilong District	410404	Urban
Henan	Shunhe Huizu District	410203	Urban
Henan	Song County	410325	Rural
Henan	Sui County	411422	Rural
Henan	Suiping County	411728	Rural
Henan	Suiyang District	411403	Urban
Henan	Taikang County	411627	Rural
Henan	Taiqian County	410927	Rural
Henan	Tanghe County	411328	Rural
Henan	Tangyin County	410523	Rural
Henan	Tongbai County	411330	Rural
Henan	Tongxu County	410222	Rural
Henan	Weibin District	410703	Urban
Henan	Weidong District	410403	Urban
Henan	Weidu District	411002	Urban
Henan	Weihui City	410781	Rural
Henan	Weishi County	410223	Rural
Henan	Wen County	410825	Rural
Henan	Wenfeng District	410502	Urban

Henan	Wolong District	411303	Urban
Henan	Wugang City	410481	Rural
Henan	Wuyang County	411121	Rural
Henan	Wuzhi County	410823	Rural
Henan	Xi County	411528	Rural
Henan	Xiangcheng City	411681	Rural
Henan	Xiangcheng County	411025	Rural
Henan	Xiayi County	411426	Rural
Henan	Xichuan County	411326	Rural
Henan	Xigong District	410303	Urban
Henan	Xihua County	411622	Rural
Henan	Xin County	411523	Rural
Henan	Xin'an County	410323	Rural
Henan	Xincai County	411729	Rural
Henan	Xingyang City	410182	Rural
Henan	Xinhua District	410402	Urban
Henan	Xinmi City	410183	Rural
Henan	Xinxiang County	410721	Rural
Henan	Xinye County	411329	Rural
Henan	Xinzheng City	410184	Rural
Henan	Xiping County	411721	Rural
Henan	Xiuwu County	410821	Rural
Henan	Xixia County	411323	Rural
Henan	Xuchang County	411023	Rural
Henan	Xun County	410621	Rural
Henan	Yancheng District	411103	Urban
Henan	Yanjin County	410726	Rural
Henan	Yanling County	411024	Rural
Henan	Yanshi City	410381	Rural
Henan	Ye County	410422	Rural
Henan	Yicheng District	411702	Urban
Henan	Yichuan County	410329	Rural
Henan	Yima City	411281	Rural
Henan	Yindu District	410505	Urban
Henan	Yiyang County	410327	Rural
Henan	Yongcheng City	411481	Rural
Henan	Yuancheng District	411302	Urban
Henan	Yuanhui District	411102	Urban
Henan	Yuanyang County	410725	Rural
Henan	Yucheng County	411425	Rural
Henan	Yuzhou City	411081	Rural
Henan	Zhaoling District	411104	Urban
Henan	Zhecheng County	411424	Rural
Henan	Zhengyang County	411724	Rural
Henan	Zhenhe District	410411	Urban
Henan	Zhenping County	411324	Rural
Henan	Zhongmu County	410122	Rural
Henan	Zhongyuan District	410102	Urban
Henan	Zhongzhan District	410803	Urban
Hubei	Anlu City	420982	Rural

Hubei	Badong County	422823	Rural
Hubei	Baokang County	420626	Rural
Hubei	Caidian District	420114	Urban
Hubei	Cengdu District	421303	Urban
Hubei	Changyang Tujiazu Zizhixian	420528	Rural
Hubei	Chibi City	421281	Rural
Hubei	Chongyang County	421223	Rural
Hubei	Dangyang City	420582	Rural
Hubei	Danjiangkou City	420381	Rural
Hubei	Dawu County	420922	Rural
Hubei	Daye City	420281	Rural
Hubei	Dianjun District	420504	Urban
Hubei	Dongbao District	420802	Urban
Hubei	Dongxihu District	420112	Urban
Hubei	Duodao District	420804	Urban
Hubei	Echeng District	420704	Urban
Hubei	Enshi City	422801	Rural
Hubei	Fancheng District	420606	Urban
Hubei	Fang County	420325	Rural
Hubei	Gong'an County	421022	Rural
Hubei	Guangshui City	421381	Rural
Hubei	Gucheng County	420625	Rural
Hubei	Hanchuan City	420984	Rural
Hubei	Hannan District	420113	Urban
Hubei	Hanyang District	420105	Urban
Hubei	Hefeng County	422828	Rural
Hubei	Hong'an County	421122	Rural
Hubei	Honghu City	421083	Rural
Hubei	Hongshan District	420111	Urban
Hubei	Huangmei County	421127	Rural
Hubei	Huangpi District	420116	Urban
Hubei	Huangshigang District	420202	Urban
Hubei	Huangzhou District	421102	Urban
Hubei	Huarong District	420703	Urban
Hubei	Jiang'an District	420102	Urban
Hubei	Jianghan District	420103	Urban
Hubei	Jiangling County	421024	Rural
Hubei	Jiangxia District	420115	Urban
Hubei	Jianli County	421023	Rural
Hubei	Jianshi County	422822	Rural
Hubei	Jiayu County	421221	Rural
Hubei	Jingshan County	420821	Rural
Hubei	Jingzhou District	421003	Urban
Hubei	Laifeng County	422827	Rural
Hubei	Laohekou City	420682	Rural
Hubei	Liangzihu District	420702	Urban
Hubei	Lichuan City	422802	Rural
Hubei	Luotian County	421123	Rural
Hubei	Macheng City	421181	Rural
Hubei	Maojian District	420302	Urban

Hubei	Nanzhang County	420624	Rural
Hubei	Qianjiang City	429005	Rural
Hubei	Qiaokou District	420104	Urban
Hubei	Qichun County	421126	Rural
Hubei	Qingshan District	420107	Urban
Hubei	Shashi District	421002	Urban
Hubei	Shayang County	420822	Rural
Hubei	Shennongjia Linqu	429021	Urban
Hubei	Shishou City	421081	Rural
Hubei	Songzi City	421087	Rural
Hubei	Sui County	421321	Rural
Hubei	Tianmen City	429006	Rural
Hubei	Tieshan District	420205	Urban
Hubei	Tongcheng County	421222	Rural
Hubei	Tongshan County	421224	Rural
Hubei	Tuanfeng County	421121	Rural
Hubei	Wuchang District	420106	Urban
Hubei	Wufeng Tujiazu Zizhixian	420529	Rural
Hubei	Wujiagang District	420503	Urban
Hubei	Wuxue City	421182	Rural
Hubei	Xialu District	420204	Urban
Hubei	Xian'an District	421202	Urban
Hubei	Xianfeng County	422826	Rural
Hubei	Xiangcheng District	420602	Urban
Hubei	Xiangyang District	420607	Urban
Hubei	Xiantao City	429004	Rural
Hubei	Xiaochang City	420921	Rural
Hubei	Xiaonan City	420902	Rural
Hubei	Xiaoting District	420505	Urban
Hubei	Xiling District	420502	Urban
Hubei	Xingshan County	420526	Rural
Hubei	Xinzhou District	420117	Urban
Hubei	Xiseshan District	420203	Urban
Hubei	Xishui County	421125	Rural
Hubei	Xuan'en County	422825	Rural
Hubei	Yangxin County	420222	Rural
Hubei	Yicheng City	420684	Rural
Hubei	Yidu City	420581	Rural
Hubei	Yiling District	420506	Urban
Hubei	Yingcheng City	420981	Rural
Hubei	Yingshan County	421124	Rural
Hubei	Yuan'an County	420525	Rural
Hubei	Yun County	420321	Rural
Hubei	Yunmeng County	420923	Rural
Hubei	Yunxi County	420322	Rural
Hubei	Zaoyang City	420683	Rural
Hubei	Zhangwan District	420303	Urban
Hubei	Zhijiang City	420583	Rural
Hubei	Zhongxiang City	420881	Rural
Hubei	Zhushan County	420323	Rural

Hubei	Zhuxi County	420324	Rural
Hubei	Zigui County	420527	Rural
Hunan	Anhua County	430923	Rural
Hunan	Anren County	431028	Rural
Hunan	Anxiang County	430721	Rural
Hunan	Baojing County	433125	Rural
Hunan	Beihu District	431002	Urban
Hunan	Beita District	430511	Urban
Hunan	Chaling County	430224	Rural
Hunan	Changning City	430482	Rural
Hunan	Changsha County	430121	Rural
Hunan	Chengbu Miaozu Zizhixian	430529	Rural
Hunan	Chenxi County	431223	Rural
Hunan	Cili County	430821	Rural
Hunan	Dao County	431124	Rural
Hunan	Daxiang District	430503	Urban
Hunan	Dingcheng District	430703	Urban
Hunan	Dong'an County	431122	Rural
Hunan	Dongkou County	430525	Rural
Hunan	Fenghuang County	433123	Rural
Hunan	Furong District	430102	Urban
Hunan	Guidong County	431027	Rural
Hunan	Guiyang County	431021	Rural
Hunan	Guzhang County	433126	Rural
Hunan	Hanshou County	430722	Rural
Hunan	Hecheng District	431202	Urban
Hunan	Hengdong County	430424	Rural
Hunan	Hengnan County	430422	Rural
Hunan	Hengshan County	430423	Rural
Hunan	Hengyang County	430421	Rural
Hunan	Heshan District	430903	Urban
Hunan	Hetang District	430202	Urban
Hunan	Hongjiang City	431281	Rural
Hunan	Huarong County	430623	Rural
Hunan	Huayuan County	433124	Rural
Hunan	Huitong County	431225	Rural
Hunan	Jiahe County	431024	Rural
Hunan	Jianghua Yaozu Zizhixian	431129	Rural
Hunan	Jiangyong County	431125	Rural
Hunan	Jingzhou Miaozu Dongzu Zizhixian	431229	Rural
Hunan	Jinshi City	430781	Rural
Hunan	Jishou City	433101	Rural
Hunan	Junshan District	430611	Urban
Hunan	Kaifu District	430105	Urban
Hunan	Lanshan County	431127	Rural
Hunan	Leiyang City	430481	Rural
Hunan	Lengshuijiang City	431381	Rural
Hunan	Lengshuitan District	431103	Urban
Hunan	Li County	430723	Rural

Hunan	Lianyuan City	431382	Rural
Hunan	Liling City	430281	Rural
Hunan	LinXiang City	430682	Rural
Hunan	Lingling District	431102	Urban
Hunan	Linli County	430724	Rural
Hunan	Linwu County	431025	Rural
Hunan	Liuyang City	430181	Rural
Hunan	Longhui County	430524	Rural
Hunan	Longshan County	433130	Rural
Hunan	Louxing District	431302	Urban
Hunan	Lusong District	430203	Urban
Hunan	Luxi County	433122	Rural
Hunan	Mayang Miaozu Zizhixian	431226	Rural
Hunan	Miluo City	430681	Rural
Hunan	Nan County	430921	Rural
Hunan	Nanyue District	430412	Urban
Hunan	Ningxiang County	430124	Rural
Hunan	Ningyuan County	431126	Rural
Hunan	Pingjiang County	430626	Rural
Hunan	Qidong County	430426	Rural
Hunan	Qiyang County	431121	Rural
Hunan	Rucheng County	431026	Rural
Hunan	Sangzhi County	430822	Rural
Hunan	Shaodong County	430521	Rural
Hunan	Shaoshan City	430382	Rural
Hunan	Shaoyang County	430523	Rural
Hunan	Shifeng District	430204	Urban
Hunan	Shigu District	430407	Urban
Hunan	Shimen County	430726	Rural
Hunan	Shuangfeng County	431321	Rural
Hunan	Shuangpai County	431123	Rural
Hunan	Shuangqing District	430502	Urban
Hunan	Suining County	430527	Rural
Hunan	Suxian District	431003	Urban
Hunan	Taojiang County	430922	Rural
Hunan	Taoyuan County	430725	Rural
Hunan	Tianxin District	430103	Urban
Hunan	Tianyuan District	430211	Urban
Hunan	Tongdao Dongzu Zizhixian	431230	Rural
Hunan	Wangcheng County	430112	Rural
Hunan	Wugang City	430581	Rural
Hunan	Wuling District	430702	Urban
Hunan	Wulingyuan District	430811	Urban
Hunan	Xiangtan County	430321	Rural
Hunan	Xiangxiang City	430381	Rural
Hunan	Xiangyin County	430624	Rural
Hunan	Xinhua County	431322	Rural
Hunan	Xinhuang Dongzu Zizhixian	431227	Rural
Hunan	Xinning County	430528	Rural
Hunan	Xinshao County	430522	Rural

Hunan	Xintian County	431128	Rural
Hunan	Xupu County	431224	Rural
Hunan	Yanfeng District	430406	Urban
Hunan	Yizhang County	431022	Rural
Hunan	Yongding District	430802	Urban
Hunan	Yongshun County	433127	Rural
Hunan	Yongxing County	431023	Rural
Hunan	You County	430223	Rural
Hunan	Yuanjiang City	430981	Rural
Hunan	Yuanling County	431222	Rural
Hunan	Yuelu District	430104	Urban
Hunan	Yuetang District	430304	Urban
Hunan	Yueyang County	430621	Rural
Hunan	Yueyanglou District	430602	Urban
Hunan	Yuhu District	430302	Urban
Hunan	Yuhua District	430111	Urban
Hunan	Yunxi District	430603	Urban
Hunan	Zhengxiang District	430408	Urban
Hunan	Zhijiang Dongzu Zizhixian	431228	Rural
Hunan	Zhongfang County	431221	Rural
Hunan	Zhuhui District	430405	Urban
Hunan	Zhuzhou County	430221	Rural
Hunan	Zixing City	431081	Rural
Hunan	Ziyang District	430902	Urban
Hunan	yanling County	430225	Rural
Inner Mon	Abag Qi	152522	Rural
Inner Mon	Alxa Youqi	152922	Rural
Inner Mon	Alxa Zuoqi	152921	Rural
Inner Mon	Aohan Qi	150430	Rural
Inner Mon	Ar Horqin Qi	150421	Rural
Inner Mon	Arun Qi	150721	Rural
Inner Mon	Arxan City	152202	Rural
Inner Mon	Bairin Youqi	150423	Rural
Inner Mon	Bairin Zuoqi	150422	Rural
Inner Mon	Baiyun Kuangqu	150206	Urban
Inner Mon	Chen Barag Qi	150725	Rural
Inner Mon	Dalad Qi	150621	Rural
Inner Mon	Darhan Muminggan Lianheqi	150223	Rural
Inner Mon	Dengkou County	150822	Rural
Inner Mon	Dong Ujimqin Qi	152525	Rural
Inner Mon	Donghe District	150202	Urban
Inner Mon	Dongsheng District	150602	Urban
Inner Mon	Dorbod Qi	150929	Rural
Inner Mon	Duolun County	152531	Rural
Inner Mon	Ejin Horo Qi	150627	Rural
Inner Mon	Ejin Qi	152923	Rural
Inner Mon	Erenhot City	152501	Rural
Inner Mon	Ergun City	150784	Rural
Inner Mon	Ewenkizu Zizhiqi	150724	Rural
Inner Mon	Fengzhen District	150981	Urban

Inner Mon	Genhe City	150785	Rural
Inner Mon	Guyang County	150222	Rural
Inner Mon	Haibowan District	150302	Urban
Inner Mon	Hailar District	150702	Urban
Inner Mon	Hainan District	150303	Urban
Inner Mon	Hanggin Houqi	150826	Rural
Inner Mon	Hanggin Qi	150625	Rural
Inner Mon	Harqin Qi	150428	Rural
Inner Mon	Hexigten Qi	150425	Rural
Inner Mon	Holingol City	150581	Rural
Inner Mon	Hongshan District	150402	Urban
Inner Mon	Horinger County	150123	Rural
Inner Mon	Horqin District	150502	Urban
Inner Mon	Horqin Youyi Qianqi	152221	Rural
Inner Mon	Horqin Youyi Zhongqi	152222	Rural
Inner Mon	Horqin Zuoyi Houqi	150522	Rural
Inner Mon	Horqin Zuoyi Zhongqi	150521	Rural
Inner Mon	Huade County	150922	Rural
Inner Mon	Huimin District	150103	Urban
Inner Mon	Hure Qi	150524	Rural
Inner Mon	Jalaid Qi	152223	Rural
Inner Mon	Jarud Qi	150526	Rural
Inner Mon	Jining District	150902	Rural
Inner Mon	Jiuyuan District	150207	Urban
Inner Mon	Jungar Qi	150622	Rural
Inner Mon	Kailu County	150523	Rural
Inner Mon	Kundulun District	150203	Urban
Inner Mon	Liangcheng County	150925	Rural
Inner Mon	Linhe District	150802	Urban
Inner Mon	Linxi County	150424	Rural
Inner Mon	Manzhouli City	150781	Rural
Inner Mon	Morin Dawa Daurzu Zizhiqi	150722	Rural
Inner Mon	Naiman Qi	150525	Rural
Inner Mon	Ningcheng County	150429	Rural
Inner Mon	Ongniud Qi	150426	Rural
Inner Mon	Oroqen Zizhiqi	150723	Rural
Inner Mon	Otog Qi	150624	Rural
Inner Mon	Otog Qianqi	150623	Rural
Inner Mon	Qahar Youyi Houqi	150928	Rural
Inner Mon	Qahar Youyi Qianqi	150926	Rural
Inner Mon	Qahar Youyi Zhongqi	150927	Rural
Inner Mon	Qingshan District	150204	Urban
Inner Mon	Qingshuihe County	150124	Rural
Inner Mon	Saihan District	150105	Urban
Inner Mon	Shangdu County	150923	Rural
Inner Mon	Shiguai District	150205	Urban
Inner Mon	Songshan District	150404	Urban
Inner Mon	Sonid Youqi	152524	Rural
Inner Mon	Sonid Zuoqi	152523	Rural
Inner Mon	Taibus Qi	152527	Rural

Inner Mon	Togtoh County	150122	Rural
Inner Mon	Tumd Youqi	150221	Rural
Inner Mon	Tumd Zuoqi	150121	Rural
Inner Mon	Tuquan County	152224	Rural
Inner Mon	Ud District	150304	Urban
Inner Mon	Ulan Hot City	152201	Rural
Inner Mon	Urad Houqi	150825	Rural
Inner Mon	Urad Qianqi	150823	Rural
Inner Mon	Urad Zhongqi	150824	Rural
Inner Mon	Uxin Qi	150626	Rural
Inner Mon	Wuchuan County	150125	Rural
Inner Mon	Wuyuan County	150821	Rural
Inner Mon	Xi Ujimqin Qi	152526	Rural
Inner Mon	Xianghuang Qi	152528	Rural
Inner Mon	Xilinhot City	152502	Rural
Inner Mon	Xin Barag Youqi	150727	Rural
Inner Mon	Xin Barag Zuoqi	150726	Rural
Inner Mon	Xincheng District	150102	Urban
Inner Mon	Xinghe County	150924	Rural
Inner Mon	Yakeshi City	150782	Rural
Inner Mon	Yuanbaoshan District	150403	Urban
Inner Mon	Yuquan District	150104	Urban
Inner Mon	Zalantun City	150783	Rural
Inner Mon	Zhenglan Qi	152530	Rural
Inner Mon	Zhengxiangbai Qi	152529	Rural
Inner Mon	Zhuozi County	150921	Rural
Jiangsu	Baixia District	320103	Urban
Jiangsu	Baoying County	321023	Rural
Jiangsu	Beitang District	320204	Urban
Jiangsu	Binhai County	320922	Rural
Jiangsu	Binhu District	320211	Urban
Jiangsu	Changshu City	320581	Rural
Jiangsu	Chong'an District	320202	Urban
Jiangsu	Chongchuan District	320602	Urban
Jiangsu	Chuzhou District	320803	Urban
Jiangsu	Dafeng City	320982	Rural
Jiangsu	Dantu District	321112	Urban
Jiangsu	Danyang City	321181	Rural
Jiangsu	Donghai County	320722	Rural
Jiangsu	Dongtai City	320981	Rural
Jiangsu	Feng County	320321	Rural
Jiangsu	Funing County	320923	Rural
Jiangsu	Gangzha District	320611	Urban
Jiangsu	Ganyu County	320721	Rural
Jiangsu	Gaochun County	320125	Rural
Jiangsu	Gaogang District	321203	Urban
Jiangsu	Gaoyou City	321084	Rural
Jiangsu	Guangling District	321002	Urban
Jiangsu	Guannan County	320724	Rural
Jiangsu	Guanyun County	320723	Rural

Jiangsu	Gulou District	320302	Urban
Jiangsu	Gusu District	320508	Urban
Jiangsu	Hai'an County	320621	Rural
Jiangsu	Hailing District	321202	Urban
Jiangsu	Haimen City	320684	Rural
Jiangsu	Haizhou District	320706	Urban
Jiangsu	Hanjiang District	321003	Urban
Jiangsu	Hongze County	320829	Rural
Jiangsu	Huaiyin District	320804	Urban
Jiangsu	Huishan District	320206	Urban
Jiangsu	Huqiu District	320505	Urban
Jiangsu	Jiangdu District	321012	Urban
Jiangsu	Jiangning District	320115	Urban
Jiangsu	Jiangyan City	321284	Rural
Jiangsu	Jiangyin City	320281	Rural
Jiangsu	Jianhu County	320925	Rural
Jiangsu	Jianye District	320105	Urban
Jiangsu	Jiawang District	320305	Urban
Jiangsu	Jingjiang City	321282	Rural
Jiangsu	Jingkou District	321102	Urban
Jiangsu	Jinhu County	320831	Rural
Jiangsu	Jintan City	320482	Rural
Jiangsu	Jurong City	321183	Rural
Jiangsu	Kunshan City	320583	Rural
Jiangsu	Lianshui County	320826	Rural
Jiangsu	Lianyun District	320703	Urban
Jiangsu	Lishui County	320124	Rural
Jiangsu	Liuhe District	320116	Urban
Jiangsu	Liyang City	320481	Rural
Jiangsu	Nanchang District	320203	Urban
Jiangsu	Nanjinggulou District	320106	Urban
Jiangsu	Pei County	320322	Rural
Jiangsu	Pizhou City	320382	Rural
Jiangsu	Pukou District	320111	Urban
Jiangsu	Qidong City	320681	Rural
Jiangsu	Qinghe District	320802	Urban
Jiangsu	Qingpu District	320811	Urban
Jiangsu	Qinhuai District	320104	Urban
Jiangsu	Qishuyan District	320405	Urban
Jiangsu	Qixia District	320113	Urban
Jiangsu	Quanshan District	320311	Urban
Jiangsu	Rudong County	320623	Rural
Jiangsu	Rugao City	320682	Rural
Jiangsu	Runzhou District	321111	Urban
Jiangsu	Sheyang County	320924	Rural
Jiangsu	Shuyang County	321322	Rural
Jiangsu	Sihong County	321324	Rural
Jiangsu	Siyang County	321323	Rural
Jiangsu	Sucheng District	321302	Urban
Jiangsu	Suining County	320324	Rural

Jiangsu	Suyu District	321311	Urban
Jiangsu	Taicang City	320585	Rural
Jiangsu	Taixing City	321283	Rural
Jiangsu	Tianning District	320402	Urban
Jiangsu	Tinghu District	320902	Urban
Jiangsu	Tongshan District	320312	Urban
Jiangsu	Tongzhou District	320612	Urban
Jiangsu	Wujiang District	320509	Urban
Jiangsu	Wujin District	320412	Urban
Jiangsu	Wuzhong District	320506	Urban
Jiangsu	Xiaguan District	320107	Urban
Jiangsu	Xiangcheng District	320507	Urban
Jiangsu	Xiangshui County	320921	Rural
Jiangsu	Xinbei District	320411	Urban
Jiangsu	Xinghua City	321281	Rural
Jiangsu	Xinpu District	320705	Urban
Jiangsu	Xinyi City	320381	Rural
Jiangsu	Xishan District	320205	Urban
Jiangsu	Xuanwu District	320102	Urban
Jiangsu	Xuyi County	320830	Rural
Jiangsu	Yandu District	320903	Urban
Jiangsu	Yangzhong City	321182	Rural
Jiangsu	Yixing City	320282	Rural
Jiangsu	Yizheng City	321081	Rural
Jiangsu	Yuhuatai District	320114	Urban
Jiangsu	Yunlong District	320303	Urban
Jiangsu	Zhangjiagang City	320582	Rural
Jiangsu	Zhonglou District	320404	Urban
Jiangxi	Anfu County	360829	Rural
Jiangxi	Anyi County	360123	Rural
Jiangxi	Anyuan District	360302	Urban
Jiangxi	Anyuan County	360726	Rural
Jiangxi	Changjiang District	360202	Urban
Jiangxi	Chongren County	361024	Rural
Jiangxi	Chongyi County	360725	Rural
Jiangxi	Dayu County	360723	Rural
Jiangxi	De'an County	360426	Rural
Jiangxi	Dexing City	361181	Rural
Jiangxi	Dingnan County	360728	Rural
Jiangxi	Donghu District	360102	Urban
Jiangxi	Dongxiang County	361029	Rural
Jiangxi	Duchang County	360428	Rural
Jiangxi	Fengcheng City	360981	Rural
Jiangxi	Fengxin County	360921	Rural
Jiangxi	Fenyi County	360521	Rural
Jiangxi	Fuliang County	360222	Rural
Jiangxi	Gan County	360721	Rural
Jiangxi	Gao'an City	360983	Rural
Jiangxi	Gongqingcheng City	360482	Rural
Jiangxi	Guangchang County	361030	Rural

Jiangxi	Guangfeng County	361122	Rural
Jiangxi	Guixi City	360681	Rural
Jiangxi	Hengfeng County	361125	Rural
Jiangxi	Huichang County	360733	Rural
Jiangxi	Hukou County	360429	Rural
Jiangxi	Ji'an County	360821	Rural
Jiangxi	Jing'an County	360925	Rural
Jiangxi	Jinggangshan City	360881	Rural
Jiangxi	Jinxi County	361027	Rural
Jiangxi	Jinxian County	360124	Rural
Jiangxi	Jishui County	360822	Rural
Jiangxi	Jiujiang County	360421	Rural
Jiangxi	Jizhou District	360802	Urban
Jiangxi	Le'an County	361025	Rural
Jiangxi	Leping City	360281	Rural
Jiangxi	Lianhua County	360321	Rural
Jiangxi	Lichuan County	361022	Rural
Jiangxi	Linchuan District	361002	Urban
Jiangxi	Longnan County	360727	Rural
Jiangxi	Lushan District	360402	Urban
Jiangxi	Luxi County	360323	Rural
Jiangxi	Nanchang County	360121	Rural
Jiangxi	Nancheng County	361021	Rural
Jiangxi	Nanfeng County	361023	Rural
Jiangxi	Nankang City	360782	Rural
Jiangxi	Ningdu County	360730	Rural
Jiangxi	Pengze County	360430	Rural
Jiangxi	Poyang County	361128	Rural
Jiangxi	Qingshanhu District	360111	Urban
Jiangxi	Qingyuan District	360803	Urban
Jiangxi	Qingyunpu District	360104	Urban
Jiangxi	Quannan County	360729	Rural
Jiangxi	Ruichang City	360481	Rural
Jiangxi	Ruijin City	360781	Rural
Jiangxi	Shanggao County	360923	Rural
Jiangxi	Shangli County	360322	Rural
Jiangxi	Shangrao County	361121	Rural
Jiangxi	Shangyou County	360724	Rural
Jiangxi	Shicheng County	360735	Rural
Jiangxi	Suichuan County	360827	Rural
Jiangxi	Taihe County	360826	Rural
Jiangxi	Tonggu County	360926	Rural
Jiangxi	Wan'an County	360828	Rural
Jiangxi	Wanli District	360105	Urban
Jiangxi	Wannian County	361129	Rural
Jiangxi	Wanzai County	360922	Rural
Jiangxi	Wuning County	360423	Rural
Jiangxi	Wuyuan Xia	361130	Rural
Jiangxi	Xiajiang County	360823	Rural
Jiangxi	Xiangdong District	360313	Urban

Jiangxi	Xihu District	360103	Urban
Jiangxi	Xinfeng County	360722	Rural
Jiangxi	Xingan County	360824	Rural
Jiangxi	Xingguo County	360732	Rural
Jiangxi	Xingzi County	360427	Rural
Jiangxi	Xinjian County	360122	Rural
Jiangxi	Xiushui County	360424	Rural
Jiangxi	Xunwu County	360734	Rural
Jiangxi	Xunyang District	360403	Urban
Jiangxi	Yanshan County	361124	Rural
Jiangxi	Yifeng County	360924	Rural
Jiangxi	Yihuang County	361026	Rural
Jiangxi	Yiyang County	361126	Rural
Jiangxi	Yongfeng County	360825	Rural
Jiangxi	Yongxin County	360830	Rural
Jiangxi	Yongxiu County	360425	Rural
Jiangxi	Yuanzhou District	360902	Urban
Jiangxi	Yudu County	360731	Rural
Jiangxi	Yuehu District	360602	Urban
Jiangxi	Yugan County	361127	Rural
Jiangxi	Yujiang County	360622	Rural
Jiangxi	Yushan County	361123	Rural
Jiangxi	Yushui District	360502	Urban
Jiangxi	ZhangGong District	360702	Urban
Jiangxi	Zhangshu City	360982	Rural
Jiangxi	Zhushan District	360203	Urban
Jiangxi	Zixi County	361028	Rural
Jiangxi	xinzhou District	361102	Urban
Jilin	Antu County	222426	Rural
Jilin	Badaojiang District	220602	Urban
Jilin	Changbai Chosenzu Zizhixian	220623	Rural
Jilin	Changling County	220722	Rural
Jilin	Changyi District	220202	Urban
Jilin	Chaoyang District	220104	Urban
Jilin	Chuanying District	220204	Urban
Jilin	Da'an City	220882	Rural
Jilin	Dehui City	220183	Rural
Jilin	Dongchang District	220502	Urban
Jilin	Dongfeng County	220421	Rural
Jilin	Dongliao County	220422	Rural
Jilin	Dunhua City	222403	Rural
Jilin	Erdao District	220105	Urban
Jilin	Erdaojiang District	220503	Urban
Jilin	Fengman District	220211	Urban
Jilin	Fusong County	220621	Rural
Jilin	Fuyu County	220724	Rural
Jilin	Gongzhuling City	220381	Rural
Jilin	Helong City	222406	Rural
Jilin	Huadian City	220282	Rural
Jilin	Huichun City	222404	Rural

Jilin	Huinan County	220523	Rural
Jilin	Ji'an City	220582	Rural
Jilin	Jiangyuan County	220605	Rural
Jilin	Jiaohe City	220281	Rural
Jilin	Jingyu County	220622	Rural
Jilin	Jiutai City	220181	Rural
Jilin	Kuancheng District	220103	Urban
Jilin	Linjiang City	220681	Rural
Jilin	Lishu County	220322	Rural
Jilin	Liuhe County	220524	Rural
Jilin	Longjing City	222405	Rural
Jilin	Longshan District	220402	Urban
Jilin	Longtan District	220203	Urban
Jilin	Luyuan District	220106	Urban
Jilin	Meihekou City	220581	Rural
Jilin	Nanguan District	220102	Urban
Jilin	Ningjiang District	220702	Urban
Jilin	Nong'an County	220122	Rural
Jilin	Panshi City	220284	Rural
Jilin	Qian Gorlos Mongolzu Zizhixian	220721	Rural
Jilin	Qian'an County	220723	Rural
Jilin	Shuangliao City	220382	Rural
Jilin	Shuangyang District	220112	Urban
Jilin	Shulan City	220283	Rural
Jilin	Taobei District	220802	Urban
Jilin	Taonan City	220881	Rural
Jilin	Tiedong District	220303	Urban
Jilin	Tiexi District	220302	Urban
Jilin	Tonghua County	220521	Rural
Jilin	Tongyu County	220822	Rural
Jilin	Tumen City	222402	Rural
Jilin	Wangqing County	222424	Rural
Jilin	Xi'an District	220403	Urban
Jilin	Yanji City	222401	Rural
Jilin	Yitong Manzu Zizhixian	220323	Rural
Jilin	Yongji County	220221	Rural
Jilin	Yushu City	220182	Rural
Jilin	Zhenlai County	220821	Rural
Liaoning	Baita District	211002	Urban
Liaoning	Bayuquan District	210804	Urban
Liaoning	Beipiao City	211381	Rural
Liaoning	Beizhen City	210782	Rural
Liaoning	Benxi Manzu Zizhixian	210521	Rural
Liaoning	Changhai County	210224	Rural
Liaoning	Changtu County	211224	Rural
Liaoning	Chaoyang County	211321	Rural
Liaoning	Dadong District	210104	Urban
Liaoning	Dashiqiao City	210882	Rural
Liaoning	Dawa County	211121	Rural

Liaoning	Dengta City	211081	Rural
Liaoning	Diaobingshan City	211281	Rural
Liaoning	Donggang City	210681	Rural
Liaoning	Dongling District?Hunnanxin District?	210112	Urban
Liaoning	Dongzhou District	210403	Urban
Liaoning	Faku County	210124	Rural
Liaoning	Fengcheng City	210682	Rural
Liaoning	Fushun County	210421	Rural
Liaoning	Fuxin Mongolzu Zizhixian	210921	Rural
Liaoning	Gaizhou City	210881	Rural
Liaoning	Ganjingzi District	210211	Urban
Liaoning	Gongchangling District	211005	Urban
Liaoning	Guta District	210702	Urban
Liaoning	Haicheng City	210381	Rural
Liaoning	Haizhou District	210902	Urban
Liaoning	Harqin Zuoyi Mongolzu Zizhixian	211324	Rural
Liaoning	Heishan County	210726	Rural
Liaoning	Heping District	210102	Urban
Liaoning	Hongwei District	211004	Urban
Liaoning	Huanggu District	210105	Urban
Liaoning	Huanren Manzu Zizhixian	210522	Rural
Liaoning	Jianchang County	211422	Rural
Liaoning	Jianping County	211322	Rural
Liaoning	Jinzhouxin District	210213	Urban
Liaoning	Kaiyuan City	211282	Rural
Liaoning	Kangping County	210123	Rural
Liaoning	Kuandian Manzu Zizhixian	210624	Rural
Liaoning	Laobian District	210811	Urban
Liaoning	Lianshan District	211402	Urban
Liaoning	Liaoyang County	211021	Rural
Liaoning	Liaozhong County	210122	Rural
Liaoning	Linghai City	210781	Rural
Liaoning	Linghe District	210703	Urban
Liaoning	Lingyuan City	211382	Rural
Liaoning	Lishan District	210304	Urban
Liaoning	Longcheng District	211303	Urban
Liaoning	Longgang District	211403	Urban
Liaoning	Lushunkou District	210212	Urban
Liaoning	Mingshan District	210504	Urban
Liaoning	Nanfen District	210505	Urban
Liaoning	Nanpiao District	211404	Urban
Liaoning	Panshan County	211122	Rural
Liaoning	Pingshan District	210502	Urban
Liaoning	Pulandian City	210282	Rural
Liaoning	Qianshan District	210311	Urban
Liaoning	Qinghe District	211204	Urban
Liaoning	Qinghemen District	210905	Urban
Liaoning	Qingyuan Manzu Zizhixian	210423	Rural

Liaoning	Shahekou District	210204	Urban
Liaoning	Shenbeixin District	210113	Urban
Liaoning	Shenhe District	210103	Urban
Liaoning	Shuangta District	211302	Urban
Liaoning	Shuangtaizi District	211102	Urban
Liaoning	Shuncheng District	210411	Urban
Liaoning	Suizhong County	211421	Rural
Liaoning	Sujiatun District	210111	Urban
Liaoning	Tai'an County	210321	Rural
Liaoning	Taihe District	210711	Urban
Liaoning	Taiping District	210904	Urban
Liaoning	Taizihe District	211011	Urban
Liaoning	Tiedong District	210302	Urban
Liaoning	Tieling County	211221	Rural
Liaoning	Tiexi District	210106	Urban
Liaoning	Tiexi District	210303	Urban
Liaoning	Wafangdian City	210281	Rural
Liaoning	Wanghua District	210404	Urban
Liaoning	Wensheng District	211003	Urban
Liaoning	Xifeng County	211223	Rural
Liaoning	Xigang District	210203	Urban
Liaoning	Xihe District	210911	Urban
Liaoning	Xihu District	210503	Urban
Liaoning	Xinbin Manzu Zizhixian	210422	Rural
Liaoning	Xinfu District	210402	Urban
Liaoning	Xingcheng City	211481	Rural
Liaoning	Xinglongtai District	211103	Urban
Liaoning	Xinmin City	210181	Rural
Liaoning	Xinqiu District	210903	Urban
Liaoning	Xishi District	210803	Urban
Liaoning	Xiuyan Manzu Zizhixian	210323	Rural
Liaoning	Yi County	210727	Rural
Liaoning	Yinzhou District	211202	Urban
Liaoning	Yuanbao District	210602	Urban
Liaoning	Yuhong District	210114	Urban
Liaoning	Zhangwu County	210922	Rural
Liaoning	Zhanqian District	210802	Urban
Liaoning	Zhen'an District	210604	Urban
Liaoning	Zhenxing District	210603	Urban
Liaoning	Zhongshan District	210202	Urban
Liaoning	Zhuanghe City	210283	Rural
Ningxia	Dawukou District	640202	Urban
Ningxia	Haiyuan County	640522	Rural
Ningxia	Helan County	640122	Rural
Ningxia	Hongsibao District	640303	Urban
Ningxia	Huinong District	640205	Urban
Ningxia	Jinfeng District	640106	Urban
Ningxia	Jingyuan County	640424	Rural
Ningxia	Lingwu City	640181	Rural
Ningxia	Litong District	640302	Urban

Ningxia	Longde County	640423	Rural
Ningxia	Pengyang County	640425	Rural
Ningxia	Pingluo County	640221	Rural
Ningxia	Qingtongxia City	640381	Rural
Ningxia	Tongxin County	640324	Rural
Ningxia	Xiji County	640422	Rural
Ningxia	Xingqing District	640104	Urban
Ningxia	Xixia District	640105	Urban
Ningxia	Yanchi County	640323	Rural
Ningxia	Yongning County	640121	Rural
Ningxia	Yuanzhou District	640402	Urban
Ningxia	Zhongning County	640521	Rural
Ningxia	Zhongwei City Chengqu	640502	Rural
Qinghai	Baima County	632622	Rural
Qinghai	Chengbei District	630105	Urban
Qinghai	Chengdong District	630102	Urban
Qinghai	Chengxi District	630104	Urban
Qinghai	Chengzhong District	630103	Urban
Qinghai	Chindu County	632723	Rural
Qinghai	Datong Huizu Tuzu Zizhixian	630121	Rural
Qinghai	Delhi City	632802	Rural
Qinghai	Dulan County	632822	Rural
Qinghai	Gadee County	632623	Rural
Qinghai	Gangcha County	632224	Rural
Qinghai	Golmud City	632801	Rural
Qinghai	Gonghe County	632521	Rural
Qinghai	Guide County	632523	Rural
Qinghai	Guinan County	632525	Rural
Qinghai	Haiyan County	632223	Rural
Qinghai	Henan Mongolzu Zizhixian	632324	Rural
Qinghai	Hualong Huizu Zizhixian	632127	Rural
Qinghai	Huangyuan County	630123	Rural
Qinghai	Huangzhong County	630122	Rural
Qinghai	Huzhu Tuzu Zizhixian	632126	Rural
Qinghai	Jainca County	632322	Rural
Qinghai	Jigzhi County	632625	Rural
Qinghai	Ledu County	632123	Rural
Qinghai	Madoi County	632626	Rural
Qinghai	Maqeen County	632621	Rural
Qinghai	Menyuan Huizu Zizhixian	632221	Rural
Qinghai	Minhe Huizu Tuzu Zizhixian	632122	Rural
Qinghai	Nangqeen County	632725	Rural
Qinghai	Ping'an County	632121	Rural
Qinghai	Qilian County	632222	Rural
Qinghai	Qumarleeb County	632726	Rural
Qinghai	Tarlag County	632624	Rural
Qinghai	Tianjun County	632823	Rural
Qinghai	Tongde County	632522	Rural
Qinghai	Tongren County	632321	Rural
Qinghai	Ulan County	632821	Rural

Qinghai	Xinghai County	632524	Rural
Qinghai	Xunhua Salarzu Zizhixian	632128	Rural
Qinghai	Yushu County	632721	Rural
Qinghai	Zadoi County	632722	Rural
Qinghai	Zeekog County	632323	Rural
Qinghai	Zhidoi County	632724	Rural
Shaanxi	Ansai County	610624	Rural
Shaanxi	Baihe County	610929	Rural
Shaanxi	Baishui County	610527	Rural
Shaanxi	Baota District	610602	Urban
Shaanxi	Baqiao District	610111	Urban
Shaanxi	Beilin District	610103	Urban
Shaanxi	Bin County	610427	Rural
Shaanxi	Chang'an District	610116	Urban
Shaanxi	Changwu County	610428	Rural
Shaanxi	Chencang District	610304	Urban
Shaanxi	Chengcheng County	610525	Rural
Shaanxi	Chenggu County	610722	Rural
Shaanxi	Chunhua County	610430	Rural
Shaanxi	Dali County	610523	Rural
Shaanxi	Danfeng County	611022	Rural
Shaanxi	Dingbian County	610825	Rural
Shaanxi	Feng County	610330	Rural
Shaanxi	Fengxiang County	610322	Rural
Shaanxi	Foping County	610730	Rural
Shaanxi	Fu County	610628	Rural
Shaanxi	Fufeng County	610324	Rural
Shaanxi	Fugu County	610822	Rural
Shaanxi	Fuping County	610528	Rural
Shaanxi	Ganquan County	610627	Rural
Shaanxi	Gaoling County	610126	Rural
Shaanxi	Hanbin District	610902	Urban
Shaanxi	Hancheng City	610581	Rural
Shaanxi	Hantai District	610702	Urban
Shaanxi	Hanyin County	610921	Rural
Shaanxi	Hengshan County	610823	Rural
Shaanxi	Heyang County	610524	Rural
Shaanxi	Hu County	610125	Rural
Shaanxi	Hua County	610521	Rural
Shaanxi	Huangling County	610632	Rural
Shaanxi	Huanglong County	610631	Rural
Shaanxi	Huayin City	610582	Rural
Shaanxi	Jia County	610828	Rural
Shaanxi	Jingbian County	610824	Rural
Shaanxi	Jingyang County	610423	Rural
Shaanxi	Jintai District	610303	Urban
Shaanxi	Lan'gao County	610925	Rural
Shaanxi	Lantian County	610122	Rural
Shaanxi	Lianhu District	610104	Urban
Shaanxi	Lintong District	610115	Urban

Shaanxi	Linwei District	610502	Urban
Shaanxi	Linyou County	610329	Rural
Shaanxi	Liquan County	610425	Rural
Shaanxi	Liuba County	610729	Rural
Shaanxi	Long County	610327	Rural
Shaanxi	Lueyang County	610727	Rural
Shaanxi	Luochuan County	610629	Rural
Shaanxi	Luonan County	611021	Rural
Shaanxi	Mei County	610326	Rural
Shaanxi	Mian County	610725	Rural
Shaanxi	Mizhi County	610827	Rural
Shaanxi	Nanzheng County	610721	Rural
Shaanxi	Ningqiang County	610726	Rural
Shaanxi	Ningshan County	610923	Rural
Shaanxi	Pingli County	610926	Rural
Shaanxi	Pucheng County	610526	Rural
Shaanxi	Qian County	610424	Rural
Shaanxi	Qianyang County	610328	Rural
Shaanxi	Qindu District	610402	Urban
Shaanxi	Qingjian County	610830	Rural
Shaanxi	Qishan County	610323	Rural
Shaanxi	Sanyuan County	610422	Rural
Shaanxi	Shangnan County	611023	Rural
Shaanxi	Shangzhou District	611002	Urban
Shaanxi	Shanyang County	611024	Rural
Shaanxi	Shenmu County	610821	Rural
Shaanxi	Shiquan County	610922	Rural
Shaanxi	Suide County	610826	Rural
Shaanxi	Taibai County	610331	Rural
Shaanxi	Tongguan County	610522	Rural
Shaanxi	Wangyi District	610202	Urban
Shaanxi	Weibin District	610302	Urban
Shaanxi	Weicheng District	610404	Urban
Shaanxi	Weiyang District	610112	Urban
Shaanxi	Wubu County	610829	Rural
Shaanxi	Wugong County	610431	Rural
Shaanxi	Wuqi County	610626	Rural
Shaanxi	Xincheng District	610102	Urban
Shaanxi	Xingping City	610481	Rural
Shaanxi	Xixiang County	610724	Rural
Shaanxi	Xunyang County	610928	Rural
Shaanxi	Xunyi County	610429	Rural
Shaanxi	Yanchang County	610621	Rural
Shaanxi	Yanchuan County	610622	Rural
Shaanxi	Yang County	610723	Rural
Shaanxi	Yangling District	610403	Urban
Shaanxi	Yanliang District	610114	Urban
Shaanxi	Yanta District	610113	Urban
Shaanxi	Yaozhou District	610204	Urban
Shaanxi	Yichuan County	610630	Rural

Shaanxi	Yijun County	610222	Rural
Shaanxi	Yintai District	610203	Urban
Shaanxi	Yongshou County	610426	Rural
Shaanxi	Yuyang District	610802	Urban
Shaanxi	Zhashui County	611026	Rural
Shaanxi	Zhen'an County	611025	Rural
Shaanxi	Zhenba County	610728	Rural
Shaanxi	Zhenping County	610927	Rural
Shaanxi	Zhidan County	610625	Rural
Shaanxi	Zhouzhi County	610124	Rural
Shaanxi	Zichang County	610623	Rural
Shaanxi	Ziyang County	610924	Rural
Shaanxi	Zizhou County	610831	Rural
Shandong	Anqiu City	370784	Rural
Shandong	BinCheng District	371602	Urban
Shandong	Boshan District	370304	Urban
Shandong	Boxing County	371625	Rural
Shandong	Cangshan County	371324	Rural
Shandong	Cao County	371721	Rural
Shandong	Changdao County	370634	Rural
Shandong	Changle County	370725	Rural
Shandong	Changqing District	370113	Urban
Shandong	Changyi City	370786	Rural
Shandong	Chengwu County	371723	Rural
Shandong	Chengyang District	370214	Urban
Shandong	Chiping County	371523	Rural
Shandong	Daiyue District	370911	Urban
Shandong	DeCheng District	371402	Urban
Shandong	Dingtao County	371727	Rural
Shandong	Dong'e County	371524	Rural
Shandong	Dongchangfu District	371502	Urban
Shandong	Donggang District	371102	Urban
Shandong	Dongming County	371728	Rural
Shandong	Dongping County	370923	Rural
Shandong	Dongying District	370502	Urban
Shandong	Fangzi District	370704	Urban
Shandong	Fei County	371325	Rural
Shandong	Feicheng City	370983	Rural
Shandong	Fushan District	370611	Urban
Shandong	Gangcheng District	371203	Urban
Shandong	Gaomi City	370785	Rural
Shandong	Gaoqing County	370322	Rural
Shandong	Gaotang County	371526	Rural
Shandong	Guan County	371525	Rural
Shandong	Guangrao County	370523	Rural
Shandong	Haiyang City	370687	Rural
Shandong	Hanting District	370703	Urban
Shandong	Hedong District	371312	Urban
Shandong	Hekou District	370503	Urban
Shandong	Huaiyin District	370104	Urban

Shandong	Huancui District	371002	Urban
Shandong	Huangdao District	370211	Urban
Shandong	Huantai County	370321	Rural
Shandong	Huimin County	371621	Rural
Shandong	Jiaonan City	370284	Rural
Shandong	Jiaozhou City	370281	Rural
Shandong	Jiaxiang County	370829	Rural
Shandong	Jimo City	370282	Rural
Shandong	Jinxiang County	370828	Rural
Shandong	Jiyang County	370125	Rural
Shandong	Ju County	371122	Rural
Shandong	Juancheng County	371726	Rural
Shandong	Junan County	371327	Rural
Shandong	Juye County	371724	Rural
Shandong	Kenli County	370521	Rural
Shandong	Kuiwen District	370705	Urban
Shandong	Laicheng District	371202	Urban
Shandong	Laishan District	370613	Urban
Shandong	Laixi City	370285	Rural
Shandong	Laiyang City	370682	Rural
Shandong	Laizhou City	370683	Rural
Shandong	Lanshan District	371103	Urban
Shandong	Lanshan District	371302	Urban
Shandong	Laoshan District	370212	Urban
Shandong	Leling City	371481	Rural
Shandong	Liangshan County	370832	Rural
Shandong	Licang District	370213	Urban
Shandong	Licheng District	370112	Urban
Shandong	Lijin County	370522	Rural
Shandong	Ling County	371421	Rural
Shandong	Linqing City	371581	Rural
Shandong	Linqu County	370724	Rural
Shandong	Linshu County	371329	Rural
Shandong	Linyi County	371424	Rural
Shandong	Linzi District	370305	Urban
Shandong	Lixia District	370102	Urban
Shandong	Longkou City	370681	Rural
Shandong	Luozhuang District	371311	Urban
Shandong	Mengyin County	371328	Rural
Shandong	Mudan District	371702	Urban
Shandong	Muping District	370612	Urban
Shandong	Ningjin County	371422	Rural
Shandong	Ningyang County	370921	Rural
Shandong	Penglai City	370684	Rural
Shandong	Pingdu City	370283	Rural
Shandong	Pingyi County	371326	Rural
Shandong	Pingyin County	370124	Rural
Shandong	Pingyuan County	371426	Rural
Shandong	Qihe County	371425	Rural
Shandong	Qingyun County	371423	Rural

Shandong	Qingzhou City	370781	Rural
Shandong	Qixia City	370686	Rural
Shandong	Qufu City	370881	Rural
Shandong	Rencheng District	370811	Urban
Shandong	Rongcheng City	371082	Rural
Shandong	Rushan City	371083	Rural
Shandong	Shan County	371722	Rural
Shandong	Shanghe County	370126	Rural
Shandong	Shanting District	370406	Urban
Shandong	Shen County	371522	Rural
Shandong	Shibei District	370203	Urban
Shandong	Shinan District	370202	Urban
Shandong	Shizhong District	370103	Urban
Shandong	Shizhong District	370402	Urban
Shandong	Shizhong District	370802	Urban
Shandong	Shouguang City	370783	Rural
Shandong	Sifang District	370205	Urban
Shandong	Sishui County	370831	Rural
Shandong	Tai'erzhuang District	370405	Urban
Shandong	Taishan District	370902	Urban
Shandong	Tancheng County	371322	Rural
Shandong	Tengzhou City	370481	Rural
Shandong	Tianqiao District	370105	Urban
Shandong	Weicheng District	370702	Urban
Shandong	Weishan County	370826	Rural
Shandong	Wendeng City	371081	Rural
Shandong	Wenshang County	370830	Rural
Shandong	Wucheng County	371428	Rural
Shandong	Wudi County	371623	Rural
Shandong	Wulian County	371121	Rural
Shandong	Xiajin County	371427	Rural
Shandong	Xintai City	370982	Rural
Shandong	Xuecheng District	370403	Urban
Shandong	Yanggu County	371521	Rural
Shandong	Yangxin County	371622	Rural
Shandong	Yanzhou City	370882	Rural
Shandong	Yicheng District	370404	Urban
Shandong	Yinan County	371321	Rural
Shandong	Yishui County	371323	Rural
Shandong	Yiyuan County	370323	Rural
Shandong	Yucheng City	371482	Rural
Shandong	Yuncheng County	371725	Rural
Shandong	Yutai County	370827	Rural
Shandong	Zhangdian District	370303	Urban
Shandong	Zhangqiu City	370181	Rural
Shandong	Zhanhua County	371624	Rural
Shandong	Zhaoyuan City	370685	Rural
Shandong	Zhifu District	370602	Urban
Shandong	Zhoucun District	370306	Urban
Shandong	Zhucheng City	370782	Rural

Shandong	Zichuan District	370302	Urban
Shandong	Zoucheng City	370883	Rural
Shandong	Zouping County	371626	Rural
Shanghai	Baoshan District	310113	Urban
Shanghai	Changning District	310105	Urban
Shanghai	Chongming County	310230	Rural
Shanghai	Fengxian District	310120	Urban
Shanghai	Hongkou District	310109	Urban
Shanghai	Huangpu District	310101	Urban
Shanghai	Jiading District	310114	Urban
Shanghai	Jing'an District	310106	Urban
Shanghai	Jinshan District	310116	Urban
Shanghai	Minxing District	310112	Urban
Shanghai	Pudongxin District	310115	Urban
Shanghai	Putuo District	310107	Urban
Shanghai	Qingpu District	310118	Urban
Shanghai	Songjiang District	310117	Urban
Shanghai	Xuhui District	310104	Urban
Shanghai	Yangpu District	310110	Urban
Shanghai	Zhabei District	310108	Urban
Shanxi	Anze County	141026	Rural
Shanxi	Baode County	140931	Rural
Shanxi	Changzhi County	140421	Rural
Shanxi	Cheng District	140202	Urban
Shanxi	Cheng District	140302	Urban
Shanxi	Cheng District	140402	Urban
Shanxi	Cheng District	140502	Urban
Shanxi	Dai County	140923	Rural
Shanxi	Daning County	141030	Rural
Shanxi	Datong County	140227	Rural
Shanxi	Dingxiang County	140921	Rural
Shanxi	Fangshan County	141128	Rural
Shanxi	Fanzhi County	140924	Rural
Shanxi	Fenxi County	141034	Rural
Shanxi	Fenyang City	141182	Rural
Shanxi	Fushan County	141027	Rural
Shanxi	Gaoping City	140581	Rural
Shanxi	Gu County	141025	Rural
Shanxi	Guangling County	140223	Rural
Shanxi	Gujiao City	140181	Rural
Shanxi	Hejin City	140882	Rural
Shanxi	Hequ County	140930	Rural
Shanxi	Heshun County	140723	Rural
Shanxi	Hongdong County	141024	Rural
Shanxi	Houma City	141081	Rural
Shanxi	Huairen County	140624	Rural
Shanxi	Huguan County	140427	Rural
Shanxi	Hunyuan County	140225	Rural
Shanxi	Huozhou City	141082	Rural
Shanxi	Ji County	141028	Rural

Shanxi	Jiancaoping District	140108	Urban
Shanxi	Jiang County	140826	Rural
Shanxi	Jiao District	140311	Urban
Shanxi	Jiao District	140411	Urban
Shanxi	Jiaocheng County	141122	Rural
Shanxi	Jiaokou County	141130	Rural
Shanxi	Jiexiu City	140781	Rural
Shanxi	Jingle County	140926	Rural
Shanxi	Jinyuan District	140110	Urban
Shanxi	Jishan County	140824	Rural
Shanxi	Kelan County	140929	Rural
Shanxi	Kuang District	140203	Urban
Shanxi	Kuang District	140303	Urban
Shanxi	Lan County	141127	Rural
Shanxi	Licheng County	140426	Rural
Shanxi	Lin County	141124	Rural
Shanxi	Lingchuan County	140524	Rural
Shanxi	Lingqiu County	140224	Rural
Shanxi	Lingshi County	140729	Rural
Shanxi	Linyi County	140821	Rural
Shanxi	Lishi District	141102	Urban
Shanxi	Liulin County	141125	Rural
Shanxi	Loufan County	140123	Rural
Shanxi	Lucheng City	140481	Rural
Shanxi	Nanjiao District	140211	Urban
Shanxi	Ningwu County	140925	Rural
Shanxi	Pianguan County	140932	Rural
Shanxi	Pingding County	140321	Rural
Shanxi	Pinglu District	140603	Urban
Shanxi	Pinglu County	140829	Rural
Shanxi	Pingshun County	140425	Rural
Shanxi	Pingyao County	140728	Rural
Shanxi	Pu County	141033	Rural
Shanxi	Qi County	140727	Rural
Shanxi	Qin County	140430	Rural
Shanxi	Qingxu County	140121	Rural
Shanxi	Qinshui County	140521	Rural
Shanxi	Qinyuan County	140431	Rural
Shanxi	Quwo County	141021	Rural
Shanxi	Ruicheng County	140830	Rural
Shanxi	Shanyin County	140621	Rural
Shanxi	Shenchi County	140927	Rural
Shanxi	Shilou County	141126	Rural
Shanxi	Shouyang County	140725	Rural
Shanxi	Shuocheng District	140602	Urban
Shanxi	Taigu County	140726	Rural
Shanxi	Tianzhen County	140222	Rural
Shanxi	Tunliu County	140424	Rural
Shanxi	Wanbailin District	140109	Urban
Shanxi	Wanrong County	140822	Rural

Shanxi	Wenshui County	141121	Rural
Shanxi	Wenxi County	140823	Rural
Shanxi	Wutai County	140922	Rural
Shanxi	Wuxiang County	140429	Rural
Shanxi	Wuzhai County	140928	Rural
Shanxi	Xi County	141031	Rural
Shanxi	Xia County	140828	Rural
Shanxi	Xiangfen County	141023	Rural
Shanxi	Xiangning County	141029	Rural
Shanxi	Xiangyuan County	140423	Rural
Shanxi	Xiaodian District	140105	Urban
Shanxi	Xiaoyi City	141181	Rural
Shanxi	Xinfu District	140902	Urban
Shanxi	Xing County	141123	Rural
Shanxi	Xinghualing District	140107	Urban
Shanxi	Xinjiang County	140825	Rural
Shanxi	Xinrong District	140212	Urban
Shanxi	Xiyang County	140724	Rural
Shanxi	Yangcheng County	140522	Rural
Shanxi	Yanggao County	140221	Rural
Shanxi	Yangqu County	140122	Rural
Shanxi	Yanhu District	140802	Urban
Shanxi	Yaodu District	141002	Urban
Shanxi	Yicheng County	141022	Rural
Shanxi	Ying County	140622	Rural
Shanxi	Yingze District	140106	Urban
Shanxi	Yonghe County	141032	Rural
Shanxi	Yongji City	140881	Rural
Shanxi	Youyu County	140623	Rural
Shanxi	Yu County	140322	Rural
Shanxi	Yuanping City	140981	Rural
Shanxi	Yuanqu County	140827	Rural
Shanxi	Yuci District	140702	Urban
Shanxi	Yushe County	140721	Rural
Shanxi	Zezhou County	140525	Rural
Shanxi	Zhangzi County	140428	Rural
Shanxi	Zhongyang County	141129	Rural
Shanxi	Zuoquan County	140722	Rural
Shanxi	Zuoyun County	140226	Rural
Sichuan	Aba(Ngawa) County	513231	Rural
Sichuan	An County	510724	Rural
Sichuan	Anju District	510904	Urban
Sichuan	Anyue County	512021	Rural
Sichuan	Baiyu County	513331	Rural
Sichuan	Baoxing County	511827	Rural
Sichuan	Barkam County	513229	Rural
Sichuan	Batang County	513335	Rural
Sichuan	Bazhou District	511902	Urban
Sichuan	Beichuan Qiangzu Zizhixian	510726	Rural
Sichuan	Butuo County	513429	Rural

Sichuan	Cangxi County	510824	Rural
Sichuan	Changning County	511524	Rural
Sichuan	Chaotian District	510812	Urban
Sichuan	Chenghua District	510108	Urban
Sichuan	Chongzhou City	510184	Rural
Sichuan	Chuanshan District	510903	Urban
Sichuan	Cuiping District	511502	Urban
Sichuan	Da County	511721	Rural
Sichuan	Daan District	510304	Urban
Sichuan	Danba(Rongzhag) County	513323	Rural
Sichuan	Danling County	511424	Rural
Sichuan	Daocheng(Dabba) County	513337	Rural
Sichuan	Dawu County	513326	Rural
Sichuan	Dayi County	510129	Rural
Sichuan	Daying County	510923	Rural
Sichuan	Dazhu County	511724	Rural
Sichuan	Dechang County	513424	Rural
Sichuan	Deegee County	513330	Rural
Sichuan	Deerong County	513338	Rural
Sichuan	Dong District	510402	Urban
Sichuan	Dongpo District	511402	Urban
Sichuan	Dongxing District	511011	Urban
Sichuan	Dujiangyan City	510181	Rural
Sichuan	Ebian Yizu Zizhixian	511132	Rural
Sichuan	Emeishan City	511181	Rural
Sichuan	Fucheng District	510703	Urban
Sichuan	Fushun County	510322	Rural
Sichuan	Ganluo County	513435	Rural
Sichuan	Gao County	511525	Rural
Sichuan	Gaoping District	511303	Urban
Sichuan	Garzee County	513328	Rural
Sichuan	Gong County	511526	Rural
Sichuan	Gongjing District	510303	Urban
Sichuan	Guang'an District	511602	Urban
Sichuan	Guanghan City	510681	Rural
Sichuan	Gulin County	510525	Rural
Sichuan	Hanyuan County	511823	Rural
Sichuan	Heishui County	513228	Rural
Sichuan	Hejiang County	510522	Rural
Sichuan	Hongya County	511423	Rural
Sichuan	Hongyuan County	513233	Rural
Sichuan	Huaying City	511681	Rural
Sichuan	Huidong County	513426	Rural
Sichuan	Huili County	513425	Rural
Sichuan	Jiajiang County	511126	Rural
Sichuan	Jialing District	511304	Urban
Sichuan	Jian'ge County	510823	Rural
Sichuan	Jiang'an County	511523	Rural
Sichuan	Jiangyang District	510502	Urban
Sichuan	Jiangyou City	510781	Rural

Sichuan	Jianyang City	512081	Rural
Sichuan	Jinchuan(Quqeen) County	513226	Rural
Sichuan	Jingyan County	511124	Rural
Sichuan	Jingyang District	510603	Urban
Sichuan	Jinjiang District	510104	Urban
Sichuan	Jinkouhe District	511113	Urban
Sichuan	Jinniu District	510106	Urban
Sichuan	Jintang County	510121	Rural
Sichuan	Jinyang County	513430	Rural
Sichuan	Jiulong(Gyaisi) County	513324	Rural
Sichuan	Jiuzhaigou County	513225	Rural
Sichuan	Junlian County	511527	Rural
Sichuan	Kaijiang County	511723	Rural
Sichuan	Kangding(Dardo) County	513321	Rural
Sichuan	Langzhong City	511381	Rural
Sichuan	Leibo County	513437	Rural
Sichuan	Lezhi County	512022	Rural
Sichuan	Li County	513222	Rural
Sichuan	Linshui County	511623	Rural
Sichuan	Litang County	513334	Rural
Sichuan	Lizhou District	510802	Urban
Sichuan	Longchang County	511028	Rural
Sichuan	Longmatan District	510504	Urban
Sichuan	Longquanyi District	510112	Urban
Sichuan	Lu County	510521	Rural
Sichuan	Luding County	513322	Rural
Sichuan	Luhuo(Zhaggo) County	513327	Rural
Sichuan	Luojiang County	510626	Rural
Sichuan	Lushan County	511826	Rural
Sichuan	Mabian Yizu Zizhixian	511133	Rural
Sichuan	Mao County	513223	Rural
Sichuan	Meigu County	513436	Rural
Sichuan	Mianning County	513433	Rural
Sichuan	Mianzhu City	510683	Rural
Sichuan	Mingshan District	511803	Urban
Sichuan	Miyi County	510421	Rural
Sichuan	Muchuan County	511129	Rural
Sichuan	Muli Zangzu Zizhixian	513422	Rural
Sichuan	Nanbu County	511321	Rural
Sichuan	Nanjiang County	511922	Rural
Sichuan	Nanxi District	511503	Urban
Sichuan	Naxi District	510503	Urban
Sichuan	Ningnan County	513427	Rural
Sichuan	Peng'an County	511323	Rural
Sichuan	Pengshan County	511422	Rural
Sichuan	Pengxi County	510921	Rural
Sichuan	Pengzhou City	510182	Rural
Sichuan	Pi County	510124	Rural
Sichuan	Pingchang County	511923	Rural
Sichuan	Pingshan County	511529	Rural

Sichuan	Pingwu County	510727	Rural
Sichuan	Puge County	513428	Rural
Sichuan	Pujiang County	510131	Rural
Sichuan	Qianweixian	511123	Rural
Sichuan	Qingbaijiang District	510113	Urban
Sichuan	Qingchuan County	510822	Rural
Sichuan	Qingshen County	511425	Rural
Sichuan	Qingyang District	510105	Urban
Sichuan	Qionglai City	510183	Rural
Sichuan	Qu County	511725	Rural
Sichuan	Renhe District	510411	Urban
Sichuan	Renshou County	511421	Rural
Sichuan	Rong County	510321	Rural
Sichuan	Santai County	510722	Rural
Sichuan	Seertar County	513333	Rural
Sichuan	SeerxuuXian	513332	Rural
Sichuan	Shawan District	511111	Urban
Sichuan	Shehong County	510922	Rural
Sichuan	Shifang City	510682	Rural
Sichuan	Shimian County	511824	Rural
Sichuan	Shizhong District	511002	Urban
Sichuan	Shizhong District	511102	Urban
Sichuan	Shuangliu County	510122	Rural
Sichuan	Shunqing District	511302	Urban
Sichuan	Songpan County	513224	Rural
Sichuan	Tianquan County	511825	Rural
Sichuan	Tongchuan District	511702	Urban
Sichuan	Tongjiang County	511921	Rural
Sichuan	Wangcang County	510821	Rural
Sichuan	Wanyuan City	511781	Rural
Sichuan	Weiyuan County	511024	Rural
Sichuan	Wenchuan County	513221	Rural
Sichuan	Wenjiang District	510115	Urban
Sichuan	Wuhou District	510107	Urban
Sichuan	Wusheng County	511622	Rural
Sichuan	Wutongqiao District	511112	Urban
Sichuan	Xi District	510403	Urban
Sichuan	Xiangcheng(Qagcheeng) County	513336	Rural
Sichuan	Xiaojin County	513227	Rural
Sichuan	Xichang City	513401	Rural
Sichuan	Xichong County	511325	Rural
Sichuan	Xide County	513432	Rural
Sichuan	Xindu District	510114	Urban
Sichuan	Xingwen County	511528	Rural
Sichuan	Xinjin County	510132	Rural
Sichuan	Xinlong(Nyagrong) County	513329	Rural
Sichuan	Xuanhan County	511722	Rural
Sichuan	Xuyong County	510524	Rural
Sichuan	Yajiang(Nyagquka) County	513325	Rural

Sichuan	Yanbian County	510422	Rural
Sichuan	Yanjiang District	512002	Urban
Sichuan	Yantan District	510311	Urban
Sichuan	Yanting County	510723	Rural
Sichuan	Yanyuan County	513423	Rural
Sichuan	Yibin County	511521	Rural
Sichuan	Yilong County	511324	Rural
Sichuan	Yingjing County	511822	Rural
Sichuan	Yingshan County	511322	Rural
Sichuan	Youxian District	510704	Urban
Sichuan	Yuanba District	510811	Urban
Sichuan	Yucheng District	511802	Urban
Sichuan	Yuechi County	511621	Rural
Sichuan	Yuexi County	513434	Rural
Sichuan	Zamtang County	513230	Rural
Sichuan	Zhaojue County	513431	Rural
Sichuan	Zhongjiang County	510623	Rural
Sichuan	Ziliujing District	510302	Urban
Sichuan	Zitong County	510725	Rural
Sichuan	Zizhong County	511025	Rural
Sichuan	Zoigee County	513232	Rural
Tianjin	Baodi District	120115	Urban
Tianjin	Beichen District	120113	Urban
Tianjin	Binhaixin District	120116	Urban
Tianjin	Dongli District	120110	Urban
Tianjin	Hebei District	120105	Urban
Tianjin	Hedong District	120102	Urban
Tianjin	Heping District	120101	Urban
Tianjin	Hexi District	120103	Urban
Tianjin	Hongqiao District	120106	Urban
Tianjin	Ji County	120225	Rural
Tianjin	Jinghai County	120223	Rural
Tianjin	Jinnan District	120112	Urban
Tianjin	Nankai District	120104	Urban
Tianjin	Ninghe County	120221	Rural
Tianjin	Wuqing District	120114	Urban
Tianjin	Xiqing District	120111	Urban
Tibet	Amdo County	542425	Rural
Tibet	Bainang County	542329	Rural
Tibet	Banbar County	542133	Rural
Tibet	Bangoin County	542428	Rural
Tibet	Baqeen County	542429	Rural
Tibet	Baxoi County	542127	Rural
Tibet	Biru County	542423	Rural
Tibet	Bomi(Bowo) County	542625	Rural
Tibet	Burang County	542521	Rural
Tibet	Chagyab County	542126	Rural
Tibet	Chanang County	542222	Rural
Tibet	Chengguan District	540102	Urban
Tibet	Comai County	542227	Rural

Tibet	Cona County	542232	Rural
Tibet	Coqeen County	542527	Rural
Tibet	Dagzee County	540126	Rural
Tibet	Damxung County	540122	Rural
Tibet	Deengqeen County	542125	Rural
Tibet	Dinggyee County	542332	Rural
Tibet	Doilungdeeqeen County	540125	Rural
Tibet	Gamba County	542338	Rural
Tibet	Gar County	542523	Rural
Tibet	Gee'gyai County	542525	Rural
Tibet	Geerzee County	542526	Rural
Tibet	Gongbo'gyamda County	542622	Rural
Tibet	Gonggar County	542223	Rural
Tibet	Gyaca County	542229	Rural
Tibet	Gyangzee County	542323	Rural
Tibet	Gyirong County	542335	Rural
Tibet	Jiali(Lhari) County	542422	Rural
Tibet	Jomda County	542122	Rural
Tibet	Kangmar County	542331	Rural
Tibet	Konjo County	542123	Rural
Tibet	Lhazee County	542326	Rural
Tibet	Lhorong County	542132	Rural
Tibet	Lhozhag County	542228	Rural
Tibet	Lhuunzee County	542231	Rural
Tibet	Lhuunzhub County	540121	Rural
Tibet	Mainling County	542623	Rural
Tibet	Maizhokunggar County	540127	Rural
Tibet	Mangkam County	542129	Rural
Tibet	Metog County	542624	Rural
Tibet	Nagarzee County	542233	Rural
Tibet	Nagqu County	542421	Rural
Tibet	Namling County	542322	Rural
Tibet	Nang County	542627	Rural
Tibet	Needong County	542221	Rural
Tibet	Ngamring County	542327	Rural
Tibet	Nyainrong County	542424	Rural
Tibet	Nyalam County	542336	Rural
Tibet	Nyeemo County	540123	Rural
Tibet	Nyima County	542430	Rural
Tibet	Nyingchi County	542621	Rural
Tibet	Qamdo County	542121	Rural
Tibet	Qonggyai County	542225	Rural
Tibet	Qusum County	542226	Rural
Tibet	Quuxuu County	540124	Rural
Tibet	Rinbung County	542330	Rural
Tibet	Riwoqee County	542124	Rural
Tibet	Rutog County	542524	Rural
Tibet	Sa'gya County	542325	Rural
Tibet	Saga County	542337	Rural
Tibet	Sangri County	542224	Rural

Tibet	Sog County	542427	Rural
Tibet	Tingri County	542324	Rural
Tibet	Xainza County	542426	Rural
Tibet	Xaitongmoin County	542328	Rural
Tibet	Xigazee City	542301	Rural
Tibet	Yadong(Chomo) County	542334	Rural
Tibet	Zanda County	542522	Rural
Tibet	Zayuu County	542626	Rural
Tibet	Zhongba County	542333	Rural
Tibet	Zogang County	542128	Rural
Xinjiang	Akqi County	653023	Rural
Xinjiang	Aksu City	652901	Rural
Xinjiang	Akto County	653022	Rural
Xinjiang	Altay City	654301	Rural
Xinjiang	Artux City	653001	Rural
Xinjiang	Awat County	652928	Rural
Xinjiang	Bachu County	653130	Rural
Xinjiang	Baicheng County	652926	Rural
Xinjiang	Baijiantan District	650204	Urban
Xinjiang	Barkol Kazak Zizhixian	652222	Rural
Xinjiang	Bohu(Bagrax) County	652829	Rural
Xinjiang	Bole City	652701	Rural
Xinjiang	Burqin County	654321	Rural
Xinjiang	Changji City	652301	Rural
Xinjiang	Dabancheng District	650107	Urban
Xinjiang	Dushanzi District	650202	Urban
Xinjiang	Emin County	654221	Rural
Xinjiang	Fuhai(Burultokay) County	654323	Rural
Xinjiang	Fukang City	652302	Rural
Xinjiang	Fuyun County	654322	Rural
Xinjiang	Gongliu(Tokkuztara) County	654024	Rural
Xinjiang	Habahe(Kaba) County	654324	Rural
Xinjiang	Hami(Kumul) City	652201	Rural
Xinjiang	Hejing County	652827	Rural
Xinjiang	Hoboksar Mongol Zizhixian	654226	Rural
Xinjiang	Hotan City	653201	Rural
Xinjiang	Hotan County	653221	Rural
Xinjiang	Hoxud County	652828	Rural
Xinjiang	Huocheng County	654023	Rural
Xinjiang	Hutubi County	652323	Rural
Xinjiang	Jeminay County	654326	Rural
Xinjiang	Jiashi County	653129	Rural
Xinjiang	Jimsar County	652327	Rural
Xinjiang	Jinghe County	652722	Rural
Xinjiang	Kalpin County	652929	Rural
Xinjiang	Kashi(Kaxgar) City	653101	Rural
Xinjiang	Korla City	652801	Rural
Xinjiang	Kuqa County	652923	Rural
Xinjiang	Kuytun City	654003	Rural
Xinjiang	Lop County	653224	Rural

Xinjiang	Luntai(Buuguur) County	652822	Rural
Xinjiang	Manas County	652324	Rural
Xinjiang	Markit County	653127	Rural
Xinjiang	Midong District	650109	Urban
Xinjiang	Minfeng(Niya) County	653227	Rural
Xinjiang	Mori Kazak Zizhixian	652328	Rural
Xinjiang	Moyu(Karakax) County	653222	Rural
Xinjiang	Nilka County	654028	Rural
Xinjiang	Orku District	650205	Urban
Xinjiang	Pishan(Guma) County	653223	Rural
Xinjiang	Qapqal Xibe Zizhixian	654022	Rural
Xinjiang	Qiemo(Qarqan) County	652825	Rural
Xinjiang	Qinghe(Qinggil) County	654325	Rural
Xinjiang	Qira County	653225	Rural
Xinjiang	Qitai County	652325	Rural
Xinjiang	Ruoqiang(Qakilik) County	652824	Rural
Xinjiang	Sayar County	652924	Rural
Xinjiang	Saybag District	650103	Urban
Xinjiang	Shache(Yarkant) County	653125	Rural
Xinjiang	Shanshan(Piqan) County	652122	Rural
Xinjiang	Shawan County	654223	Rural
Xinjiang	Shihezi City	659001	Rural
Xinjiang	Shufu County	653121	Rural
Xinjiang	Shuimogou District	650105	Urban
Xinjiang	Shule County	653122	Rural
Xinjiang	Tacheng City	654201	Rural
Xinjiang	Taxkorgan Tajik Zizhixian	653131	Rural
Xinjiang	Tekes County	654027	Rural
Xinjiang	Tianshan District	650102	Urban
Xinjiang	Toksun County	652123	Rural
Xinjiang	Toli County	654224	Rural
Xinjiang	Toutunhe District	650106	Urban
Xinjiang	Turpan City	652101	Rural
Xinjiang	Urumqi County	650121	Rural
Xinjiang	Usu City	654202	Rural
Xinjiang	Wenquan County	652723	Rural
Xinjiang	Wensu County	652922	Rural
Xinjiang	Wuqia(Ulugqat) County	653024	Rural
Xinjiang	Wushi(Uqturpan) County	652927	Rural
Xinjiang	Xinhe County	652925	Rural
Xinjiang	Xinshi District	650104	Urban
Xinjiang	Xinyuan County	654025	Rural
Xinjiang	Yanqi Huizu Zizhixian	652826	Rural
Xinjiang	Yecheng County	653126	Rural
Xinjiang	Yengisar County	653123	Rural
Xinjiang	Yining City	654002	Rural
Xinjiang	Yining County	654021	Rural
Xinjiang	Yiwu(Aratuuruk) County	652223	Rural
Xinjiang	Yopurga County	653128	Rural
Xinjiang	Yuli(Lopnur) County	652823	Rural

Xinjiang	Yumin County	654225	Rural
Xinjiang	Yutian(Keriya) County	653226	Rural
Xinjiang	Zepu(Poskam) County	653124	Rural
Xinjiang	Zhaosu County	654026	Rural
Xinjiang	karamay District	650203	Urban
Yunnan	Anning City	530181	Rural
Yunnan	Binchuan County	532924	Rural
Yunnan	Cangyuan Vazu Zizhixian	530927	Rural
Yunnan	Changning County	530524	Rural
Yunnan	Chenggong County	530114	Rural
Yunnan	Chengjiang County	530422	Rural
Yunnan	Chuxiong City	532301	Rural
Yunnan	Daguan County	530624	Rural
Yunnan	Dali City	532901	Rural
Yunnan	Dayao County	532326	Rural
Yunnan	Deeqeen County	533422	Rural
Yunnan	Dongchuan District	530113	Urban
Yunnan	Eryuan County	532930	Rural
Yunnan	Eshan Yizu Zizhixian	530426	Rural
Yunnan	Fengqing County	530921	Rural
Yunnan	Fugong County	533323	Rural
Yunnan	Fumin County	530124	Rural
Yunnan	Funing County	532628	Rural
Yunnan	Fuyuan County	530325	Rural
Yunnan	Gejiu City	532501	Rural
Yunnan	Gengma Daizu Vazu Zizhixian	530926	Rural
Yunnan	Gongshan Derungzu Nuzu Zizhixian	533324	Rural
Yunnan	Guandu District	530111	Urban
Yunnan	Guangnan County	532627	Rural
Yunnan	Gucheng District	530702	Urban
Yunnan	Hekou Yaozu Zizhixian	532532	Rural
Yunnan	Heqing County	532932	Rural
Yunnan	Honghe County	532529	Rural
Yunnan	Hongta District	530402	Urban
Yunnan	Huaning County	530424	Rural
Yunnan	Huaping County	530723	Rural
Yunnan	Huize County	530326	Rural
Yunnan	Jianchuan County	532931	Rural
Yunnan	Jiangcheng Hanizu Yizu Zizhixian	530826	Rural
Yunnan	Jiangchuan County	530421	Rural
Yunnan	Jianshui County	532524	Rural
Yunnan	Jingdong Yizu Zizhixian	530823	Rural
Yunnan	Jinggu Daizu Yizu Zizhixian	530824	Rural
Yunnan	Jinghong City	532801	Rural
Yunnan	Jinning County	530122	Rural
Yunnan	Jinping Miaozu Yaozu Daizu Zizhixian	532530	Rural

Yunnan	Kaiyuan City	532502	Rural
Yunnan	Lancang Lahuzu Zizhixian	530828	Rural
Yunnan	Lanping Baizu Pumizu Zizhixian	533325	Rural
Yunnan	Lianghe County	533122	Rural
Yunnan	Linxiang District	530902	Urban
Yunnan	Longchuan County	533124	Rural
Yunnan	Longling County	530523	Rural
Yunnan	Longyang District	530502	Urban
Yunnan	Luchuan Yizu Miaozu Zizhixian	530128	Rural
Yunnan	Luchun County	532531	Rural
Yunnan	Ludian County	530621	Rural
Yunnan	Lufeng County	532331	Rural
Yunnan	Luliang County	530322	Rural
Yunnan	Luoping County	530324	Rural
Yunnan	Lushui County	533321	Rural
Yunnan	Luxi County	532527	Rural
Yunnan	Maguan County	532625	Rural
Yunnan	Malipo County	532624	Rural
Yunnan	Malong County	530321	Rural
Yunnan	Mang City	533103	Rural
Yunnan	Menghai County	532822	Rural
Yunnan	Mengla County	532823	Rural
Yunnan	Menglian Daizu Lahuzu Vazu Zizhixian	530827	Rural
Yunnan	Mengzi County	532503	Rural
Yunnan	Midu County	532925	Rural
Yunnan	Mile County	532526	Rural
Yunnan	Mojiang Hanizu Zizhixian	530822	Rural
Yunnan	Mouding County	532323	Rural
Yunnan	Nanhua County	532324	Rural
Yunnan	Nanjian Yizu Zizhixian	532926	Rural
Yunnan	Ninger Hanizu Yizu Zizhixian	530821	Rural
Yunnan	Ninglang Yizu Zizhixian	530724	Rural
Yunnan	Panlong District	530103	Urban
Yunnan	Pingbian Miaozu Zizhixian	532523	Rural
Yunnan	Qiaojia County	530622	Rural
Yunnan	Qiubei County	532626	Rural
Yunnan	Qulin City	530302	Rural
Yunnan	Ruili City	533102	Rural
Yunnan	Shanggelila County	533421	Rural
Yunnan	Shidian County	530521	Rural
Yunnan	Shilin Yizu Zizhixian	530126	Rural
Yunnan	Shiping County	532525	Rural
Yunnan	Shizong County	530323	Rural
Yunnan	Shuangbai County	532322	Rural
Yunnan	Shuangjiang Lahuzu Vazu Blangzu Daizu Zizhixian	530925	Rural
Yunnan	Shuifu County	530630	Rural

Yunnan	Simao District	530802	Urban
Yunnan	Songming County	530127	Rural
Yunnan	Suijiang County	530626	Rural
Yunnan	Tengchong County	530522	Rural
Yunnan	Tonghai County	530423	Rural
Yunnan	Weishan Yizu Huizu Zizhixian	532927	Rural
Yunnan	Weixi Lisuzu ZizhiXian	533423	Rural
Yunnan	Weixin County	530629	Rural
Yunnan	Wenshan County	532601	Rural
Yunnan	Wuding County	532329	Rural
Yunnan	Wuhua District	530102	Urban
Yunnan	Xiangyun County	532923	Rural
Yunnan	Xichou County	532623	Rural
Yunnan	Ximeng Vazu Zizhixian	530829	Rural
Yunnan	Xinping Yizu Daizu Zizhixian	530427	Rural
Yunnan	Xishan District	530112	Urban
Yunnan	Xuanwei City	530381	Rural
Yunnan	Xundian Huizu Yizu Zizhixian	530129	Rural
Yunnan	Yangbi Yizu Zizhixian	532922	Rural
Yunnan	Yanjin County	530623	Rural
Yunnan	Yanshan County	532622	Rural
Yunnan	Yao'an County	532325	Rural
Yunnan	Yiliang County	530125	Rural
Yunnan	Yiliang County	530628	Rural
Yunnan	Yimen County	530425	Rural
Yunnan	Yingjiang County	533123	Rural
Yunnan	Yongde County	530923	Rural
Yunnan	Yongping County	532928	Rural
Yunnan	Yongren County	532327	Rural
Yunnan	Yongshan County	530625	Rural
Yunnan	Yongsheng County	530722	Rural
Yunnan	Yuanjiang Hanizu Yizu Daizu Zizhixian	530428	Rural
Yunnan	Yuanmou County	532328	Rural
Yunnan	Yuanyang County	532528	Rural
Yunnan	Yulong Naxizu Zizhixian	530721	Rural
Yunnan	Yun County	530922	Rural
Yunnan	Yunlong County	532929	Rural
Yunnan	Zhanyi County	530328	Rural
Yunnan	Zhaoyang District	530602	Urban
Yunnan	Zhenkang County	530924	Rural
Yunnan	Zhenxiong County	530627	Rural
Yunnan	Zhenyuan Yizu Hanizu Lahuzu Zizhixian	530825	Rural
Zhejiang	Anji County	330523	Rural
Zhejiang	Beilun District	330206	Urban
Zhejiang	Binjiang District	330108	Urban
Zhejiang	Cangnan County	330327	Rural
Zhejiang	Changshan County	330822	Rural
Zhejiang	Changxing County	330522	Rural

Zhejiang	Chun'an County	330127	Rural
Zhejiang	Cixi City	330282	Rural
Zhejiang	Daishan County	330921	Rural
Zhejiang	Deqing County	330521	Rural
Zhejiang	Dinghai District	330902	Urban
Zhejiang	Dongtou County	330322	Rural
Zhejiang	Dongyang City	330783	Rural
Zhejiang	Fenghua City	330283	Rural
Zhejiang	Fuyang City	330183	Rural
Zhejiang	Gongshu District	330105	Urban
Zhejiang	Haining City	330481	Rural
Zhejiang	Haishu District	330203	Urban
Zhejiang	Haiyan County	330424	Rural
Zhejiang	Huangyan District	331003	Urban
Zhejiang	Jiande City	330182	Rural
Zhejiang	Jiangan District	330104	Urban
Zhejiang	Jiangbei District	330205	Urban
Zhejiang	Jiangdong District	330204	Urban
Zhejiang	Jiangshan City	330881	Rural
Zhejiang	Jiaojiang District	331002	Urban
Zhejiang	Jiashan County	330421	Rural
Zhejiang	Jindong District	330703	Urban
Zhejiang	Jingning Shezu Zizhixian	331127	Rural
Zhejiang	Jinyun County	331122	Rural
Zhejiang	Kaihua County	330824	Rural
Zhejiang	Kecheng District	330802	Urban
Zhejiang	Lanxi City	330781	Rural
Zhejiang	Liandu District	331102	Urban
Zhejiang	Lin'an City	330185	Rural
Zhejiang	Linhai City	331082	Rural
Zhejiang	Longquan City	331181	Rural
Zhejiang	Longwan District	330303	Urban
Zhejiang	Longyou County	330825	Rural
Zhejiang	Lucheng District	330302	Urban
Zhejiang	Luqiao District	331004	Urban
Zhejiang	Nanhu District	330402	Urban
Zhejiang	Nanxue District	330503	Urban
Zhejiang	Ninghai County	330226	Rural
Zhejiang	Ouhai District	330304	Urban
Zhejiang	Pan'an County	330727	Rural
Zhejiang	Pinghu City	330482	Rural
Zhejiang	Pingyang County	330326	Rural
Zhejiang	Pujiang County	330726	Rural
Zhejiang	Putuo District	330903	Urban
Zhejiang	Qingtian County	331121	Rural
Zhejiang	Qingyuan County	331126	Rural
Zhejiang	Qujiang District	330803	Urban
Zhejiang	Rui'an City	330381	Rural
Zhejiang	Sanmen County	331022	Rural
Zhejiang	Shangcheng District	330102	Urban

Zhejiang	Shangyu City	330682	Rural
Zhejiang	Shaoxing County	330621	Rural
Zhejiang	Shengsi County	330922	Rural
Zhejiang	Shengzhou City	330683	Rural
Zhejiang	Songyang County	331124	Rural
Zhejiang	Suichang County	331123	Rural
Zhejiang	Taishun County	330329	Rural
Zhejiang	Tiantai County	331023	Rural
Zhejiang	Tonglu County	330122	Rural
Zhejiang	Tongxiang City	330483	Rural
Zhejiang	Wencheng County	330328	Rural
Zhejiang	Wenling City	331081	Rural
Zhejiang	Wucheng District	330702	Urban
Zhejiang	Wuxing District	330502	Urban
Zhejiang	Wuyi County	330723	Rural
Zhejiang	Xiacheng District	330103	Urban
Zhejiang	Xiangshan County	330225	Rural
Zhejiang	Xianju County	331024	Rural
Zhejiang	Xiaoshan District	330109	Urban
Zhejiang	Xihu District	330106	Urban
Zhejiang	Xinchang County	330624	Rural
Zhejiang	Xiuzhou District	330411	Urban
Zhejiang	Yinzhou District	330212	Urban
Zhejiang	Yiwu City	330782	Rural
Zhejiang	Yongjia County	330324	Rural
Zhejiang	Yongkang City	330784	Rural
Zhejiang	Yuecheng District	330602	Urban
Zhejiang	Yueqing City	330382	Rural
Zhejiang	Yuhang District	330110	Urban
Zhejiang	Yuhuan County	331021	Rural
Zhejiang	Yunhe County	331125	Rural
Zhejiang	Yuyao City	330281	Rural
Zhejiang	Zhenhai District	330211	Urban
Zhejiang	Zhuji City	330681	Rural

Citation	Component	Cause
Xu X-Z, Jin X-L, Cao H-J, Qian Y-X, Shen M-X, Jiang W-C, Sun F-H. [Process for lymphatic filariasis elimination in Jiangsu Province, China]. Chin J Schistosomiasis Control. 2011; 23(2): 207-10	Nonfatal Health Outcomes	Lymphatic filariasis
Ministry of Public Health (China). China National Tuberculosis Prevalence Survey 1990	Nonfatal Health Outcomes	Drug-susceptible tuberculosis
Ministry of Public Health (China). China National Tuberculosis Survey 1984-1985	Nonfatal Health Outcomes	Multidrug-resistant tuberculosis without extensive drug resistance
National Central Cancer Registry (China). China Cancer Registry Incidence and Mortality 1990-2009	Nonfatal Health Outcomes	Breast cancer
World Health Organization (WHO). WHO Mortality Database Version September 2016. Geneva, Switzerland: World Health Organization (WHO), 2016	Nonfatal Health Outcomes	HIV/AIDS
Liu X, Guo Q, Zhang Y, Su W, Li Z, Yu Y. Epidemiologic survey of epilepsy in 160,000 population in Shanxi province. J Fourth Mil Med Uni. 1990; 11: 338-41	Nonfatal Health Outcomes	Epilepsy
National Central Cancer Registry (China). China Cancer Registry Incidence and Mortality 1990-2009	Nonfatal Health Outcomes	Non-melanoma skin cancer (basal-cell carcinoma)
Organization for Economic Co-operation and Development (OECD). OECD Health Statistics. Paris, France: Organization for Economic Co-operation and Development (OECD)	Nonfatal Health Outcomes	Intracerebral hemorrhage
Deng SS, Hueng PP, Yan XL, Shen HY, Shen ZL, Song CC. [Serological epidemiologic surveillance on filariasis by ELISA]. Chin J Parasitol Parasit Dis. 1992; 10(4): 271-4	Nonfatal Health Outcomes	Lymphatic filariasis
Organization for Economic Co-operation and Development (OECD). OECD Health Statistics. Paris, France: Organization for Economic Co-operation and Development (OECD)	Nonfatal Health Outcomes	Syphilis
Liu XH. [An epidemiological survey of epilepsy in 42 thousand population in Sichuan Province (author's transl)]. Chin J Neurol Psychiatry. 1981; 14(3): 135-8	Nonfatal Health Outcomes	Epilepsy
Lam BC, Yeung CY. Perinatal features of birth asphyxia and neurologic outcome. Acta Paediatr Jpn. 1992; 34(1): 17-22	Nonfatal Health Outcomes	Neonatal encephalopathy due to birth asphyxia and trauma
Ministry of Public Health (China). China Schistosomiasis Prevalence National Sample Survey 1989	Nonfatal Health Outcomes	Schistosomiasis
Organization for Economic Co-operation and Development (OECD). OECD Health Statistics. Paris, France: Organization for Economic Co-operation and Development (OECD)	Nonfatal Health Outcomes	Klinefelter syndrome
Liu JY, Liu XJ, Chen Z, Tu ZP, Zheng GB, Chen YN, Zhang YZ, Weng SP, Huang XH, Yang FZ. Filariasis and its control in Fujian, China. Southeast Asian J Trop Med Public Health. 1991; 22(2): 147-54	Nonfatal Health Outcomes	Lymphatic filariasis
United Nations Population Division. Abortion Policies: A Global Review. New York, United States: United Nations (UN), 2002	Nonfatal Health Outcomes	Ectopic pregnancy
United Nations Population Division. Abortion Policies: A Global Review. New York, United States: United Nations (UN), 2002	Nonfatal Health Outcomes	Maternal abortion and miscarriage
Ministry of Public Health (China). China National Tuberculosis Survey 1984-1985	Nonfatal Health Outcomes	Extensively drug-resistant tuberculosis
Ministry of Public Health (China). China National Tuberculosis Survey 1984-1985	Nonfatal Health Outcomes	HIV/AIDS - Extensively drug-resistant Tuberculosis
Ministry of Public Health (China). China National Tuberculosis Prevalence Survey 1990	Nonfatal Health Outcomes	Multidrug-resistant tuberculosis without extensive drug resistance
Ministry of Public Health (China). China National Tuberculosis Prevalence Survey 1990	Nonfatal Health Outcomes	HIV/AIDS - Multidrug-resistant Tuberculosis without extensive drug resistance
Chen H, Wu X, Wei M, Eichner JE, Fan Y, Zhang Z, Lei C, Stone DU, Yang J. Changes in the prevalence of visual impairment due to blinding trachoma in Sichuan province, China: a comparative study between 1987 and 2006. Ophthalmic Epidemiol. 2012; 19(1): 29-37	Nonfatal Health Outcomes	Glaucoma
Organization for Economic Co-operation and Development (OECD). OECD Health Statistics. Paris, France: Organization for Economic Co-operation and Development (OECD)	Nonfatal Health Outcomes	Animal contact
Association S of CCM, Cheng K. Retrospective investigation of hospitalized patients with heart failure in some parts of China, in 1980, 1990 and 2000. Chin J Cardiol. 2002; 30(8): 450-4	Nonfatal Health Outcomes	Heart failure
Ministry of Public Health (China). China National Tuberculosis Survey 1984-1985	Nonfatal Health Outcomes	Drug-susceptible tuberculosis
Wang W, Chen X, Liu D. Hematologic response to iron and ascorbic acid administration in preschool children with anemia. Nutr Res. 1986; 241-8	Nonfatal Health Outcomes	Dietary iron deficiency
Organization for Economic Co-operation and Development (OECD). OECD Health Statistics. Paris, France: Organization for Economic Co-operation and Development (OECD)	Nonfatal Health Outcomes	Foreign body in eyes

Ministry of Public Health (China). China National Tuberculosis Prevalence Survey 1990	Nonfatal Health Outcomes	Extensively drug-resistant tuberculosis
Ministry of Public Health (China). China National Tuberculosis Prevalence Survey 1990	Nonfatal Health Outcomes	HIV/AIDS - Extensively drug-resistant Tuberculosis
Zhang SY, Zou LH, Gao YQ, Di Y, Wang XD. National epidemiological survey of blindness and low vision in China. Chin Med J (Engl). 1992; 105(7): 603-8	Nonfatal Health Outcomes	Vision loss
Karvonen M, Viik-Kajander M, Moltchanova E, Libman I, LaPorte R, Tuomilehto J. Incidence of childhood type 1 diabetes worldwide. Diabetes Mondiale (DiaMond) Project Group. Diabetes Care. 2000; 23(10): 1516Â–26	Nonfatal Health Outcomes	Diabetes mellitus type 1
National Central Cancer Registry (China). China Cancer Registry Incidence and Mortality 1990-2009	Nonfatal Health Outcomes	Bladder cancer
Organization for Economic Co-operation and Development (OECD). OECD Health Statistics. Paris, France: Organization for Economic Co-operation and Development (OECD)	Nonfatal Health Outcomes	Self-harm by firearm
Ministry of Public Health (China). China National Tuberculosis Survey 1984-1985	Nonfatal Health Outcomes	Latent tuberculosis infection
Ministry of Public Health (China). China National Tuberculosis Survey 1984-1985	Nonfatal Health Outcomes	HIV/AIDS - Multidrug-resistant Tuberculosis without extensive drug resistance
Organization for Economic Co-operation and Development (OECD). OECD Health Statistics. Paris, France: Organization for Economic Co-operation and Development (OECD)	Nonfatal Health Outcomes	Maternal hypertensive disorders
Pigott DM, Bhatt S, Golding N, Duda KA, Battle KE, Brady OJ, Messina JP, Balard Y, Bastien P, Pratlong F, Brownstein JS, Freifeld CC, Mekaru SR, Gething PW, George DB, Myers MF, Reithinger R, Hay SI. Global distribution maps of the leishmaniases. Elife. 2014; 3: e02851	Nonfatal Health Outcomes	Visceral leishmaniasis
Organization for Economic Co-operation and Development (OECD). OECD Health Statistics. Paris, France: Organization for Economic Co-operation and Development (OECD)	Nonfatal Health Outcomes	Silicosis
Ministry of Public Health (China). China National Tuberculosis Survey 1984-1985	Nonfatal Health Outcomes	HIV/AIDS - Drug-susceptible Tuberculosis
Chitsulo L, Engels D, Montresor A, Savioli L. The global status of schistosomiasis and its control. Addict Behav Rep. 2000; 77(1): 41-51	Nonfatal Health Outcomes	Schistosomiasis
Organization for Economic Co-operation and Development (OECD). OECD Health Statistics. Paris, France: Organization for Economic Co-operation and Development (OECD)	Nonfatal Health Outcomes	Appendicitis
United Nations Population Division. Abortion Policies: A Global Review. New York, United States: United Nations (UN), 2002	Nonfatal Health Outcomes	None
Ministry of Public Health (China). China National Tuberculosis Prevalence Survey 1990	Nonfatal Health Outcomes	Latent tuberculosis infection
Organization for Economic Co-operation and Development (OECD). OECD Health Statistics. Paris, France: Organization for Economic Co-operation and Development (OECD)	Nonfatal Health Outcomes	Cellulitis
Ministry of Public Health (China). China National Tuberculosis Prevalence Survey 1990	Nonfatal Health Outcomes	HIV/AIDS - Drug-susceptible Tuberculosis
Organization for Economic Co-operation and Development (OECD). OECD Health Statistics. Paris, France: Organization for Economic Co-operation and Development (OECD)	Nonfatal Health Outcomes	Neural tube defects
Liu J, Chen Z, Huang X, Tu Z. Mass treatment of filariasis using DEC-medicated salt. J Trop Med Hyg. 1992; 95(2): 132-5	Nonfatal Health Outcomes	Lymphatic filariasis
Organization for Economic Co-operation and Development (OECD). OECD Health Statistics. Paris, France: Organization for Economic Co-operation and Development (OECD)	Nonfatal Health Outcomes	Environmental heat and cold exposure
Organization for Economic Co-operation and Development (OECD). OECD Health Statistics. Paris, France: Organization for Economic Co-operation and Development (OECD)	Nonfatal Health Outcomes	Orofacial clefts
Ministry of Public Health (China). China National Tuberculosis Prevalence Survey 1990	Nonfatal Health Outcomes	Tuberculosis
Carolina Population Center, University of North Carolina at Chapel Hill, Chinese Center for Disease Control and Prevention (CCDC). China Health and Nutrition Survey 1989-2011. Chapel Hill, United States: Carolina Population Center, University of North Carolina at Chapel Hill	Nonfatal Health Outcomes	Diarrheal diseases
Zhang SY, Zou LH, Gao YQ, Di Y, Wang XD. National epidemiological survey of blindness and low vision in China. Chin Med J (Engl). 1992; 105(7): 603-8	Nonfatal Health Outcomes	
Organization for Economic Co-operation and Development (OECD). OECD Health Statistics. Paris, France: Organization for Economic Co-operation and Development (OECD)	Nonfatal Health Outcomes	Cirrhosis and other chronic liver diseases

Yang TY, Yang XY, Ch'en WC, Qi SL, Jin YJ, Gan WJ, Qu Q. Thalassemia in China. Ann N Y Acad Sci. 1985; 445: 92-105	Nonfatal Health Outcomes	Thalassemias
Jiang J, Li B, Cao W, Jiang X, Jia X, Chen Q, Wu J. Screening and prevention of neonatal glucose 6-phosphate dehydrogenase deficiency in Guangzhou, China. Genet Mol Res. 2014; 13(2): 4272Â–9	Nonfatal Health Outcomes	G6PD deficiency
Organization for Economic Co-operation and Development (OECD). OECD Health Statistics. Paris, France: Organization for Economic Co-operation and Development (OECD)	Nonfatal Health Outcomes	Polycystic ovarian syndrome
Cheng SY, Luk NM, Chong LY. Special features of non-melanoma skin cancer in Hong Kong Chinese patients: 10-year retrospective study. Hong Kong Med J. 2001; 7(1): 22-8	Nonfatal Health Outcomes	Non-melanoma skin cancer (squamous-cell carcinoma)
Organization for Economic Co-operation and Development (OECD). OECD Health Statistics. Paris, France: Organization for Economic Co-operation and Development (OECD)	Nonfatal Health Outcomes	Neonatal sepsis and other neonatal infections
Wigley RD, Zhang NZ, Zeng QY, Shi CS, Hu DW, Couchman K, Duff IF, Bennett PH. Rheumatic diseases in China: ILAR-China study comparing the prevalence of rheumatic symptoms in northern and southern rural populations. J Rheumatol. 1994; 21(8): 1484-90	Nonfatal Health Outcomes	Rheumatoid arthritis
Organization for Economic Co-operation and Development (OECD). OECD Health Statistics. Paris, France: Organization for Economic Co-operation and Development (OECD)	Nonfatal Health Outcomes	Physical violence by firearm
Da G-W. Tibetan farmers epidemiology of epilepsy of Lhasa. Tibet J Med. 1997; 18: 23-4	Nonfatal Health Outcomes	Epilepsy
Ministry of Public Health (China). China National Tuberculosis Survey 1984-1985	Nonfatal Health Outcomes	Tuberculosis
Zeng Q, Huang S, Chen R. 10-year epidemiological study on rheumatic diseases in Shantou area . Chin J Intern Med. 1997; 36(3): 193-7	Nonfatal Health Outcomes	Gout
Organization for Economic Co-operation and Development (OECD). OECD Health Statistics. Paris, France: Organization for Economic Co-operation and Development (OECD)	Nonfatal Health Outcomes	Lower respiratory infections
Sun YD. [A sero-epidemiological study of viral hepatitis A in a village]. Chin J Prev Med. 1985; 19(3): 140-2	Nonfatal Health Outcomes	Acute hepatitis A
Geng J, Xu D, Gong J, Li W. Assessing hepatitis A virus epidemic stochastic process in eight cities in China in 1990. Int J Epidemiol. 1998; 27(2): 320-2	Nonfatal Health Outcomes	Acute hepatitis A
Organization for Economic Co-operation and Development (OECD). OECD Health Statistics. Paris, France: Organization for Economic Co-operation and Development (OECD)	Nonfatal Health Outcomes	Other pneumoconiosis
Parkin DM, International Agency for Research on Cancer, International Association of Cancer Registries. Cancer Incidence in Five Continents. Vol. I to VIII. Lyon, France, IARCPress, 2005	Nonfatal Health Outcomes	Non-melanoma skin cancer (basal-cell carcinoma)
Wang W, Chen X, Liu D. Hematologic response to iron and ascorbic acid administration in preschool children with anemia. Nutr Res. 1986; 241-8	Nonfatal Health Outcomes	Anemia
Organization for Economic Co-operation and Development (OECD). OECD Health Statistics. Paris, France: Organization for Economic Co-operation and Development (OECD)	Nonfatal Health Outcomes	Inguinal, femoral, and abdominal hernia
Organization for Economic Co-operation and Development (OECD). OECD Health Statistics. Paris, France: Organization for Economic Co-operation and Development (OECD)	Nonfatal Health Outcomes	Gallbladder and biliary diseases
Organization for Economic Co-operation and Development (OECD). OECD Health Statistics. Paris, France: Organization for Economic Co-operation and Development (OECD)	Nonfatal Health Outcomes	Benign prostatic hyperplasia
Organization for Economic Co-operation and Development (OECD). OECD Health Statistics. Paris, France: Organization for Economic Co-operation and Development (OECD)	Nonfatal Health Outcomes	Atrial fibrillation and flutter
Organization for Economic Co-operation and Development (OECD). OECD Health Statistics. Paris, France: Organization for Economic Co-operation and Development (OECD)	Nonfatal Health Outcomes	Cutaneous and mucocutaneous leishmaniasis
Organization for Economic Co-operation and Development (OECD). OECD Health Statistics. Paris, France: Organization for Economic Co-operation and Development (OECD)	Nonfatal Health Outcomes	Cystic echinococcosis
Chen C-Z. The epidemiological investigation on mental disorders in Shandong province. Shandong Psychiatry. 1989; 2: 1-6	Nonfatal Health Outcomes	Schizophrenia
Organization for Economic Co-operation and Development (OECD). OECD Health Statistics. Paris, France: Organization for Economic Co-operation and Development (OECD)	Nonfatal Health Outcomes	Endocrine, metabolic, blood, and immune disorders

Organization for Economic Co-operation and Development (OECD). OECD Health Statistics. Paris, France: Organization for Economic Co-operation and Development (OECD)	Nonfatal Health Outcomes	Congenital heart anomalies
Organization for Economic Co-operation and Development (OECD). OECD Health Statistics. Paris, France: Organization for Economic Co-operation and Development (OECD)	Nonfatal Health Outcomes	Self-harm
Organization for Economic Co-operation and Development (OECD). OECD Health Statistics. Paris, France: Organization for Economic Co-operation and Development (OECD)	Nonfatal Health Outcomes	Unintentional firearm injuries
Organization for Economic Co-operation and Development (OECD). OECD Health Statistics. Paris, France: Organization for Economic Co-operation and Development (OECD)	Nonfatal Health Outcomes	Venomous animal contact
Organization for Economic Co-operation and Development (OECD). OECD Health Statistics. Paris, France: Organization for Economic Co-operation and Development (OECD)	Nonfatal Health Outcomes	Unintentional suffocation
Organization for Economic Co-operation and Development (OECD). OECD Health Statistics. Paris, France: Organization for Economic Co-operation and Development (OECD)	Nonfatal Health Outcomes	Other unintentional injuries
Organization for Economic Co-operation and Development (OECD). OECD Health Statistics. Paris, France: Organization for Economic Co-operation and Development (OECD)	Nonfatal Health Outcomes	Rheumatic heart disease
Organization for Economic Co-operation and Development (OECD). OECD Health Statistics. Paris, France: Organization for Economic Co-operation and Development (OECD)	Nonfatal Health Outcomes	Maternal sepsis and other maternal infections
Organization for Economic Co-operation and Development (OECD). OECD Health Statistics. Paris, France: Organization for Economic Co-operation and Development (OECD)	Nonfatal Health Outcomes	Congenital musculoskeletal and limb anomalies
Bian JY, Li RY, Wang WJ. Feasibility of milk fluoridation and trends in dental caries of children in China. Adv Dent Res. 1995; 9(2): 112-5	Nonfatal Health Outcomes	Caries of deciduous teeth
Organization for Economic Co-operation and Development (OECD). OECD Health Statistics. Paris, France: Organization for Economic Co-operation and Development (OECD)	Nonfatal Health Outcomes	Meningitis
Organization for Economic Co-operation and Development (OECD). OECD Health Statistics. Paris, France: Organization for Economic Co-operation and Development (OECD)	Nonfatal Health Outcomes	Ischemic heart disease
Organization for Economic Co-operation and Development (OECD). OECD Health Statistics. Paris, France: Organization for Economic Co-operation and Development (OECD)	Nonfatal Health Outcomes	Interstitial lung disease and pulmonary sarcoidosis
Organization for Economic Co-operation and Development (OECD). OECD Health Statistics. Paris, France: Organization for Economic Co-operation and Development (OECD)	Nonfatal Health Outcomes	Other road injuries
Organization for Economic Co-operation and Development (OECD). OECD Health Statistics. Paris, France: Organization for Economic Co-operation and Development (OECD)	Nonfatal Health Outcomes	Vascular intestinal disorders
Organization for Economic Co-operation and Development (OECD). OECD Health Statistics. Paris, France: Organization for Economic Co-operation and Development (OECD)	Nonfatal Health Outcomes	Pancreatitis
Organization for Economic Co-operation and Development (OECD). OECD Health Statistics. Paris, France: Organization for Economic Co-operation and Development (OECD)	Nonfatal Health Outcomes	Endometriosis
Organization for Economic Co-operation and Development (OECD). OECD Health Statistics. Paris, France: Organization for Economic Co-operation and Development (OECD)	Nonfatal Health Outcomes	Other transport injuries
Organization for Economic Co-operation and Development (OECD). OECD Health Statistics. Paris, France: Organization for Economic Co-operation and Development (OECD)	Nonfatal Health Outcomes	Motorcyclist road injuries
Organization for Economic Co-operation and Development (OECD). OECD Health Statistics. Paris, France: Organization for Economic Co-operation and Development (OECD)	Nonfatal Health Outcomes	Pulmonary aspiration and foreign body in airway
Organization for Economic Co-operation and Development (OECD). OECD Health Statistics. Paris, France: Organization for Economic Co-operation and Development (OECD)	Nonfatal Health Outcomes	Acute glomerulonephritis
Organization for Economic Co-operation and Development (OECD). OECD Health Statistics. Paris, France: Organization for Economic Co-operation and Development (OECD)	Nonfatal Health Outcomes	Physical violence by other means

Li FB, Gao CL, Qi MW. [Epidemiological study of epilepsy among 100,000 population in Qinghai Province]. Qinghai Med J. 1997; 27: 1-3	Nonfatal Health Outcomes	Epilepsy
Carolina Population Center, University of North Carolina at Chapel Hill, Chinese Center for Disease Control and Prevention (CCDC). China Health and Nutrition Survey. Chapel Hill, United States: Carolina Population Center, University of North Carolina at Chapel Hill	Nonfatal Health Outcomes	Pulmonary aspiration and foreign body in airway
Organization for Economic Co-operation and Development (OECD). OECD Health Statistics. Paris, France: Organization for Economic Co-operation and Development (OECD)	Nonfatal Health Outcomes	Ischemic stroke
Carolina Population Center, University of North Carolina at Chapel Hill, Chinese Center for Disease Control and Prevention (CCDC). China Health and Nutrition Survey. Chapel Hill, United States: Carolina Population Center, University of North Carolina at Chapel Hill	Nonfatal Health Outcomes	Motor vehicle road injuries
Carolina Population Center, University of North Carolina at Chapel Hill, Chinese Center for Disease Control and Prevention (CCDC). China Health and Nutrition Survey. Chapel Hill, United States: Carolina Population Center, University of North Carolina at Chapel Hill	Nonfatal Health Outcomes	Foreign body in other body part
Organization for Economic Co-operation and Development (OECD). OECD Health Statistics. Paris, France: Organization for Economic Co-operation and Development (OECD)	Nonfatal Health Outcomes	Down syndrome
Chen H, Wu X, Wei M, Eichner JE, Fan Y, Zhang Z, Lei C, Stone DU, Yang J. Changes in the prevalence of visual impairment due to blinding trachoma in Sichuan province, China: a comparative study between 1987 and 2006. Ophthalmic Epidemiol. 2012; 19(1): 29-37	Nonfatal Health Outcomes	Vision loss
Organization for Economic Co-operation and Development (OECD). OECD Health Statistics. Paris, France: Organization for Economic Co-operation and Development (OECD)	Nonfatal Health Outcomes	Drowning
Organization for Economic Co-operation and Development (OECD). OECD Health Statistics. Paris, France: Organization for Economic Co-operation and Development (OECD)	Nonfatal Health Outcomes	Coal workers pneumoconiosis
Organization for Economic Co-operation and Development (OECD). OECD Health Statistics. Paris, France: Organization for Economic Co-operation and Development (OECD)	Nonfatal Health Outcomes	Gastritis and duodenitis
International Clearinghouse for Birth Defects Monitoring Systems. Congenital Malformations Worldwide: A Report from the International Clearinghouse for Birth Defects Monitoring Systems. Amsterdam, Netherlands: Elsevier, 1991	Nonfatal Health Outcomes	Neural tube defects
Organization for Economic Co-operation and Development (OECD). OECD Health Statistics. Paris, France: Organization for Economic Co-operation and Development (OECD)	Nonfatal Health Outcomes	Maternal abortion, miscarriage, and ectopic pregnancy
Zhang S, Cheng F, Webber R. A successful control programme for lymphatic filariasis in Hubei, China. Trans R Soc Trop Med Hyg. 1994; 88(5): 510-2	Nonfatal Health Outcomes	Lymphatic filariasis
Shen SX, Wang HB, Chen ZW, Shen YE, Fu H, Wu CE, Ye TT, Wang JJ, Wang KA, Li TL, Yang Z, LaPorte RE, Dorman JS. The incidence of insulin-dependent diabetes mellitus in urban districts of Shanghai (1989-1993). J Pediatr Endocrinol Metab. 1996; 9(4): 469-73	Nonfatal Health Outcomes	Diabetes mellitus
Organization for Economic Co-operation and Development (OECD). OECD Health Statistics. Paris, France: Organization for Economic Co-operation and Development (OECD)	Nonfatal Health Outcomes	Endocarditis
Organization for Economic Co-operation and Development (OECD). OECD Health Statistics. Paris, France: Organization for Economic Co-operation and Development (OECD)	Nonfatal Health Outcomes	Fire, heat, and hot substances
Organization for Economic Co-operation and Development (OECD). OECD Health Statistics. Paris, France: Organization for Economic Co-operation and Development (OECD)	Nonfatal Health Outcomes	Interpersonal violence
National Central Cancer Registry (China). China Cancer Registry Incidence and Mortality 1990-2009	Nonfatal Health Outcomes	Nasopharynx cancer
Cai MQ, Yan WY. Study on iron nutritional status in adolescence. Biomed Environ Sci. 1990; 3(1): 113-9	Nonfatal Health Outcomes	Anemia
Cai MQ, Yan WY. Study on iron nutritional status in adolescence. Biomed Environ Sci. 1990; 3(1): 113-9	Nonfatal Health Outcomes	Dietary iron deficiency
Guo Y-S. A Survey of Main Hereditary Diseases in Shandong Province. J Shandong Univ Health Sci. 1993; 31(4): 271-4	Nonfatal Health Outcomes	Schizophrenia
Gao J, Zeng S, Sun BL, Fan HM, Han LH. Menstrual blood loss and hematologic indices in healthy Chinese women. J Reprod Med. 1987; 32(11): 822-6	Nonfatal Health Outcomes	Anemia
National Central Cancer Registry (China). China Cancer Registry Incidence and Mortality 1990-2009	Nonfatal Health Outcomes	Stomach cancer

National Central Cancer Registry (China). China Cancer Registry Incidence and Mortality 1990-2009	Nonfatal Health Outcomes	Colon and rectum cancer
National Central Cancer Registry (China). China Cancer Registry Incidence and Mortality 1990-2009	Nonfatal Health Outcomes	Liver cancer
National Central Cancer Registry (China). China Cancer Registry Incidence and Mortality 1990-2009	Nonfatal Health Outcomes	Tracheal, bronchus, and lung cancer
National Central Cancer Registry (China). China Cancer Registry Incidence and Mortality 1990-2009	Nonfatal Health Outcomes	Esophageal cancer
Drew JH, Beischer NA, Walstab JE, Liang ST, Stronge JM. Contribution of prematurity and low birth-weight to neonatal death in Chinese and Western populations. Aust N Z J Obstet Gynaecol. 1988; 28(3): 190-2	Nonfatal Health Outcomes	Neonatal preterm birth
Sung RY, So LY, Ng HK, Ho JK, Fok TF. Echocardiography as a tool for determining the incidence of congenital heart disease in newborn babies: a pilot study in Hong Kong. Int J Cardiol. 1991; 30(1): 43-7	Nonfatal Health Outcomes	Congenital birth defects
Pullan RL, Smith JL, Jasrasaria R, Brooker SJ. Global numbers of infection and disease burden of soil transmitted helminth infections in 2010 [Unpublished data]. Parasit Vectors. 2014; 7(37). [Unpublished data as provided by the Global Burden of Disease 2010 soil transmitted helminths expert group]	Nonfatal Health Outcomes	Ascariasis
Pullan RL, Smith JL, Jasrasaria R, Brooker SJ. Global numbers of infection and disease burden of soil transmitted helminth infections in 2010 [Unpublished data]. Parasit Vectors. 2014; 7(37). [Unpublished data as provided by the Global Burden of Disease 2010 soil transmitted helminths expert group]	Nonfatal Health Outcomes	Hookworm disease
Karvonen M, Viik-Kajander M, Moltchanova E, Libman I, LaPorte R, Tuomilehto J. Incidence of childhood type 1 diabetes worldwide. Diabetes Mondiale (DiaMond) Project Group. Diabetes Care. 2000; 23(10): 1516Â–26	Nonfatal Health Outcomes	Diabetes mellitus
Pullan RL, Smith JL, Jasrasaria R, Brooker SJ. Global numbers of infection and disease burden of soil transmitted helminth infections in 2010 [Unpublished data]. Parasit Vectors. 2014; 7(37). [Unpublished data as provided by the Global Burden of Disease 2010 soil transmitted helminths expert group]	Nonfatal Health Outcomes	Trichuriasis
National Central Cancer Registry (China). China Cancer Registry Incidence and Mortality 1990-2009	Nonfatal Health Outcomes	Uterine cancer
National Central Cancer Registry (China). China Cancer Registry Incidence and Mortality 1990-2009	Nonfatal Health Outcomes	Leukemia
Organization for Economic Co-operation and Development (OECD). OECD Health Statistics. Paris, France: Organization for Economic Co-operation and Development (OECD)	Nonfatal Health Outcomes	Encephalitis
National Central Cancer Registry (China). China Cancer Registry Incidence and Mortality 1990-2009	Nonfatal Health Outcomes	Pancreatic cancer
National Central Cancer Registry (China). China Cancer Registry Incidence and Mortality 1990-2009	Nonfatal Health Outcomes	Larynx cancer
National Central Cancer Registry (China). China Cancer Registry Incidence and Mortality 1990-2009	Nonfatal Health Outcomes	Cervical cancer
National Central Cancer Registry (China). China Cancer Registry Incidence and Mortality 1990-2009	Nonfatal Health Outcomes	Non-melanoma skin cancer (squamous-cell carcinoma)
National Central Cancer Registry (China). China Cancer Registry Incidence and Mortality 1990-2009	Nonfatal Health Outcomes	Prostate cancer
National Central Cancer Registry (China). China Cancer Registry Incidence and Mortality 1990-2009	Nonfatal Health Outcomes	Brain and central nervous system cancer
National Central Cancer Registry (China). China Cancer Registry Incidence and Mortality 1990-2009	Nonfatal Health Outcomes	Non-Hodgkin lymphoma
National Central Cancer Registry (China). China Cancer Registry Incidence and Mortality 1990-2009	Nonfatal Health Outcomes	Other leukemia
Organization for Economic Co-operation and Development (OECD). OECD Health Statistics. Paris, France: Organization for Economic Co-operation and Development (OECD)	Nonfatal Health Outcomes	Decubitus ulcer
National Central Cancer Registry (China). China Cancer Registry Incidence and Mortality 1990-2009	Nonfatal Health Outcomes	Testicular cancer
Carolina Population Center, University of North Carolina at Chapel Hill, Chinese Center for Disease Control and Prevention (CCDC). China Health and Nutrition Survey. Chapel Hill, United States: Carolina Population Center, University of North Carolina at Chapel Hill	Nonfatal Health Outcomes	None
Organization for Economic Co-operation and Development (OECD). OECD Health Statistics. Paris, France: Organization for Economic Co-operation and Development (OECD)	Nonfatal Health Outcomes	Road injuries

Organization for Economic Co-operation and Development (OECD). OECD Health Statistics. Paris, France: Organization for Economic Co-operation and Development (OECD)	Nonfatal Health Outcomes	Foreign body in other body part
Organization for Economic Co-operation and Development (OECD). OECD Health Statistics. Paris, France: Organization for Economic Co-operation and Development (OECD)	Nonfatal Health Outcomes	Self-harm by other specified means
Organization for Economic Co-operation and Development (OECD). OECD Health Statistics. Paris, France: Organization for Economic Co-operation and Development (OECD)	Nonfatal Health Outcomes	Paralytic ileus and intestinal obstruction
Organization for Economic Co-operation and Development (OECD). OECD Health Statistics. Paris, France: Organization for Economic Co-operation and Development (OECD)	Nonfatal Health Outcomes	Motor vehicle road injuries
Organization for Economic Co-operation and Development (OECD). OECD Health Statistics. Paris, France: Organization for Economic Co-operation and Development (OECD)	Nonfatal Health Outcomes	Diarrheal diseases
Organization for Economic Co-operation and Development (OECD). OECD Health Statistics. Paris, France: Organization for Economic Co-operation and Development (OECD)	Nonfatal Health Outcomes	Urolithiasis
Parkin DM, International Agency for Research on Cancer, International Association of Cancer Registries. Cancer Incidence in Five Continents. Vol. I to VIII. Lyon, France, IARCPress, 2005	Nonfatal Health Outcomes	Non-melanoma skin cancer (squamous-cell carcinoma)
Organization for Economic Co-operation and Development (OECD). OECD Health Statistics. Paris, France: Organization for Economic Co-operation and Development (OECD)	Nonfatal Health Outcomes	Neonatal preterm birth
Organization for Economic Co-operation and Development (OECD). OECD Health Statistics. Paris, France: Organization for Economic Co-operation and Development (OECD)	Nonfatal Health Outcomes	Asbestosis
Organization for Economic Co-operation and Development (OECD). OECD Health Statistics. Paris, France: Organization for Economic Co-operation and Development (OECD)	Nonfatal Health Outcomes	Pyoderma
Organization for Economic Co-operation and Development (OECD). OECD Health Statistics. Paris, France: Organization for Economic Co-operation and Development (OECD)	Nonfatal Health Outcomes	Exposure to mechanical forces
Organization for Economic Co-operation and Development (OECD). OECD Health Statistics. Paris, France: Organization for Economic Co-operation and Development (OECD)	Nonfatal Health Outcomes	Pedestrian road injuries
Jiang G, Li W, Wang D, Shen C, Ji Y, Zheng W. Epidemiological transition and distribution of stroke incidence in Tianjin, China, 1988-2010. Public Health. 2016; 131: 11-9	Nonfatal Health Outcomes	Subarachnoid hemorrhage
Organization for Economic Co-operation and Development (OECD). OECD Health Statistics. Paris, France: Organization for Economic Co-operation and Development (OECD)	Nonfatal Health Outcomes	Genital prolapse
Organization for Economic Co-operation and Development (OECD). OECD Health Statistics. Paris, France: Organization for Economic Co-operation and Development (OECD)	Nonfatal Health Outcomes	Physical violence by sharp object
Organization for Economic Co-operation and Development (OECD). OECD Health Statistics. Paris, France: Organization for Economic Co-operation and Development (OECD)	Nonfatal Health Outcomes	Inflammatory bowel disease
Organization for Economic Co-operation and Development (OECD). OECD Health Statistics. Paris, France: Organization for Economic Co-operation and Development (OECD)	Nonfatal Health Outcomes	Uterine fibroids
Organization for Economic Co-operation and Development (OECD). OECD Health Statistics. Paris, France: Organization for Economic Co-operation and Development (OECD)	Nonfatal Health Outcomes	Urinary tract infections
Organization for Economic Co-operation and Development (OECD). OECD Health Statistics. Paris, France: Organization for Economic Co-operation and Development (OECD)	Nonfatal Health Outcomes	None
Organization for Economic Co-operation and Development (OECD). OECD Health Statistics. Paris, France: Organization for Economic Co-operation and Development (OECD)	Nonfatal Health Outcomes	Poisonings
Organization for Economic Co-operation and Development (OECD). OECD Health Statistics. Paris, France: Organization for Economic Co-operation and Development (OECD)	Nonfatal Health Outcomes	Neonatal encephalopathy due to birth asphyxia and trauma
Organization for Economic Co-operation and Development (OECD). OECD Health Statistics. Paris, France: Organization for Economic Co-operation and Development (OECD)	Nonfatal Health Outcomes	Turner syndrome

Zhan FX, Zhang SQ, Wang LL, Yuan FY, Zhang HX, Dong XR, Li KJ, Yu PH. [Lymphatic filariasis in Hubei Province: from prevailing to elimination]. Chin J Parasitol Parasit Dis. 2012; 30(1): 65-70	Nonfatal Health Outcomes	Lymphatic filariasis
Shen SX, Wang HB, Chen ZW, Shen YE, Fu H, Wu CE, Ye TT, Wang JJ, Wang KA, Li TL, Yang Z, LaPorte RE, Dorman JS. The incidence of insulin-dependent diabetes mellitus in urban districts of Shanghai (1989-1993). J Pediatr Endocrinol Metab. 1996; 9(4): 469-73	Nonfatal Health Outcomes	Diabetes mellitus type 1
Organization for Economic Co-operation and Development (OECD). OECD Health Statistics. Paris, France: Organization for Economic Co-operation and Development (OECD)	Nonfatal Health Outcomes	Visceral leishmaniasis
Organization for Economic Co-operation and Development (OECD). OECD Health Statistics. Paris, France: Organization for Economic Co-operation and Development (OECD)	Nonfatal Health Outcomes	Myocarditis
Li QJ, Duan JH, Hu GL, Yu LR, Yang WQ, Li LZ, Ouyang HK. [Epidemiological characteristics and control of filariasis in Hunan Province]. Chin J Parasitol Parasit Dis. 1990; 8(2): 134-7	Nonfatal Health Outcomes	Lymphatic filariasis
Zhong CH, Zhen TM. Study on application of filarial specific IgG4 kit in disease surveillance of lymphatic filariasis. Chin Med J. 1991; 104(3): 179-85	Nonfatal Health Outcomes	Lymphatic filariasis
Organization for Economic Co-operation and Development (OECD). OECD Health Statistics. Paris, France: Organization for Economic Co-operation and Development (OECD)	Nonfatal Health Outcomes	Falls
Chow DKL, Leong RWL, Tsoi KKF, Ng SSM, Leung W, Wu JCY, Wong VWS, Chan FKL, Sung JJY. Long-term follow-up of ulcerative colitis in the Chinese population. Am J Gastroenterol. 2009; 104(3): 647-54	Nonfatal Health Outcomes	Inflammatory bowel disease
Leong RWL, Lau JY, Sung JJY. The epidemiology and phenotype of Crohn's disease in the Chinese population. Inflamm Bowel Dis. 2004; 10(5): 646-51	Nonfatal Health Outcomes	Inflammatory bowel disease
Ng SC, Leung WK, Shi HY, Li MK, Leung CM, Ng CK, Lo FH, Hui YT, Tsang SW, Chan YK, Loo CK, Chan KH, Hui AJ, Chow WH, Harbord M, Ching JY, Lee M, Chan V, Tang W, Hung IF, Ho J, Lao WC, Wong MT, Sze SF, Shan EH, Lam BC, Tong RW, Mak LY, Wong SH, Wu JC, Chan FK, Sung JJ. Epidemiology of Inflammatory Bowel Disease from 1981 to 2014: Results from a Territory-Wide Population-Based Registry in Hong Kong. Inflamm Bowel Dis. 2016; 22(8): 1954-60	Nonfatal Health Outcomes	Inflammatory bowel disease
Organization for Economic Co-operation and Development (OECD). OECD Health Statistics. Paris, France: Organization for Economic Co-operation and Development (OECD)	Nonfatal Health Outcomes	Adverse effects of medical treatment
Harrison G, Hopper K, Craig T, Laska E, Siegel C, Wanderling J, Dube KC, Ganev K, Giel R, an der Heiden W, Holmberg SK, Janca A, Lee PW, LeÃ³n CA, Malhotra S, Marsella AJ, Nakane Y, Sartorius N, Shen Y, Skoda C, Thara R, Tsirkin SJ, Varma VK, Walsh D, Wiersma D. Recovery from psychotic illness: a 15- and 25-year international follow-up study. Br J Psychiatry. 2001; 178: 506-17	Nonfatal Health Outcomes	Schizophrenia
Organization for Economic Co-operation and Development (OECD). OECD Health Statistics. Paris, France: Organization for Economic Co-operation and Development (OECD)	Nonfatal Health Outcomes	Peptic ulcer disease
Lee S, Chan YYL, Hsu LKG. The intermediate-term outcome of Chinese patients with anorexia nervosa in Hong Kong. Am J Psychiatry. 2003; 160(5): 967-72	Nonfatal Health Outcomes	Anorexia nervosa
Cockram CS, Woo J, Lau E, Chan JC, Chan AY, Lau J, Swaminathan R, Donnan SP. The prevalence of diabetes mellitus and impaired glucose tolerance among Hong Kong Chinese adults of working age. Diabetes Res Clin Pract. 1993; 21(1): 67-73	Nonfatal Health Outcomes	Diabetes mellitus
Li F, Gao C, Qi M. The epidemiologic survey of epilepsy of 100,000 population in Qinghai province. Qinghai Med J. 1997; 27: 1-3	Nonfatal Health Outcomes	Epilepsy
Organization for Economic Co-operation and Development (OECD). OECD Health Statistics. Paris, France: Organization for Economic Co-operation and Development (OECD)	Nonfatal Health Outcomes	Cyclist road injuries
Organization for Economic Co-operation and Development (OECD). OECD Health Statistics. Paris, France: Organization for Economic Co-operation and Development (OECD)	Nonfatal Health Outcomes	Non-venomous animal contact
Carolina Population Center, University of North Carolina at Chapel Hill, Chinese Center for Disease Control and Prevention (CCDC). China Health and Nutrition Survey. Chapel Hill, United States: Carolina Population Center, University of North Carolina at Chapel Hill	Nonfatal Health Outcomes	Interstitial lung disease and pulmonary sarcoidosis
Lam STS, Cheng ML. Neonatal screening in Hong Kong and Macau. Southeast Asian J Trop Med Public Health. 2003; 73-5	Nonfatal Health Outcomes	G6PD deficiency
Lau E, Symmons D, Bankhead C, MacGregor A, Donnan S, Silman A. Low prevalence of rheumatoid arthritis in the urbanized Chinese of Hong Kong. J Rheumatol. 1993; 20(7): 1133-7	Nonfatal Health Outcomes	Rheumatoid arthritis

Evans RW, Lo EC. Effects of School Dental Care Service in Hong Kong--primary teeth. Community Dent Oral Epidemiol. 1992; 20(4): 193-5	Nonfatal Health Outcomes	Caries of deciduous teeth
Hu CZ, Huang CR, Rong S, Zhang W, Wu J, Pilot T. Periodontal conditions in elderly people of Shanghai, People's Republic of China, in 1986. Community Dent Health. 1990; 7(1): 69-71	Nonfatal Health Outcomes	Periodontal diseases
Lo EC, Evans RW, Lind OP. Dental caries status and treatment needs of the permanent dentition of 6-12-year-olds in Hong Kong. Community Dent Oral Epidemiol. 1990; 1(18): 9-11	Nonfatal Health Outcomes	Caries of permanent teeth
Lind OP, Holmgren CJ, Evans RW, Corbet EF, Lim LP, Davies WI. Part 1: Clinical findings Community Dent Health. Community Dent Health. 1987; 4(4): 351-66	Nonfatal Health Outcomes	Caries of permanent teeth
King NM, Ling JY, Ng BV, Wei SH. The dental caries status and dental treatment patterns of 12-year-old children in Hong Kong. J Dent Res. 1986; 65(11): 1371-4	Nonfatal Health Outcomes	Caries of permanent teeth
Lo EC, Schwarz E. Tooth and root conditions in the middle-aged and the elderly in Hong Kong. Community Dent Oral Epidemiol. 1994; 22(5 Pt 2): 381-5	Nonfatal Health Outcomes	Edentulism and severe tooth loss
Carolina Population Center, University of North Carolina at Chapel Hill, Chinese Center for Disease Control and Prevention (CCDC). China Health and Nutrition Survey 1989-2011. Chapel Hill, United States: Carolina Population Center, University of North Carolina at Chapel Hill	Nonfatal Health Outcomes	Chronic kidney disease
World Health Organization (WHO). WHO Mortality Database Version September 2016. Geneva, Switzerland: World Health Organization (WHO), 2016	Nonfatal Health Outcomes	HIV/AIDS resulting in other diseases
Hu CZ, Huang CR, Rong S, Zhang W, Wu J, Pilot T. Periodontal conditions in elderly people of Shanghai, People's Republic of China, in 1986. Community Dent Health. 1990; 7(1): 69-71	Nonfatal Health Outcomes	Edentulism and severe tooth loss
Organization for Economic Co-operation and Development (OECD). OECD Health Statistics. Paris, France: Organization for Economic Co-operation and Development (OECD)	Nonfatal Health Outcomes	Other exposure to mechanical forces
Sun KO, Chan YW, Cheung RT, So PC, Yu YL, Li PC. Management of tetanus: a review of 18 cases. J R Soc Med. 1994; 87(3): 135-7	Nonfatal Health Outcomes	Tetanus
Carolina Population Center, University of North Carolina at Chapel Hill, Chinese Center for Disease Control and Prevention (CCDC). China Health and Nutrition Survey. Chapel Hill, United States: Carolina Population Center, University of North Carolina at Chapel Hill	Nonfatal Health Outcomes	Paralytic ileus and intestinal obstruction
Tu J, Cao H-G, Tang J-P, Liu L-Z, Li D. Prevalence Data Analysis of Two Epidemiological Surveys of Mental Illness in Hunan Province. Chin J Clin Psychol. 1995; 3(2): 101-4	Nonfatal Health Outcomes	Alcohol use disorders
Carolina Population Center, University of North Carolina at Chapel Hill, Chinese Center for Disease Control and Prevention (CCDC). China Health and Nutrition Survey. Chapel Hill, United States: Carolina Population Center, University of North Carolina at Chapel Hill	Nonfatal Health Outcomes	Other transport injuries
Carolina Population Center, University of North Carolina at Chapel Hill, Chinese Center for Disease Control and Prevention (CCDC). China Health and Nutrition Survey. Chapel Hill, United States: Carolina Population Center, University of North Carolina at Chapel Hill	Nonfatal Health Outcomes	Urolithiasis
Su L-Y, Luo X-R, Yang Z-W, Wan G-B, Li X-R. Investigation on prevalence of mental disorders and their influential factors in 13-16-year-old adolescents in Hunan province. Chin Ment Health J. 1993; 7(5): 221-224	Nonfatal Health Outcomes	Attention-deficit/hyperactivity disorder
Luo X-R, Wan G-B, Su L-Y, Yang Z-W. Epidemiological study of conduct disorder (CD) of 7-16-year-olds in Hunan province, China. Chin Ment Health J. 1994; 8(5): 227-228	Nonfatal Health Outcomes	Conduct disorder
Wei B-X, Hu X-L, Chen J-M. The epidemiological investigation on mental disorders in Mianyang city. Sichuan Ment Health. 1994; 7(1): 50-1	Nonfatal Health Outcomes	Schizophrenia
Carolina Population Center, University of North Carolina at Chapel Hill, Chinese Center for Disease Control and Prevention (CCDC). China Health and Nutrition Survey. Chapel Hill, United States: Carolina Population Center, University of North Carolina at Chapel Hill	Nonfatal Health Outcomes	Self-harm
Hu M, Schenzle D, Deinhardt F, Scheid R. Prevalence of markers of hepatitis A and B in the Shanghai area. J Infect Dis. 1983; 147(2): 360	Nonfatal Health Outcomes	Acute hepatitis A
Liu X-H, Guo Q-T, Zhang Y-D. Epidemiological survey of epilepsy among 160,000 in Shaanxi, China. J Fourth Mil Med Uni. 1990; 338-41	Nonfatal Health Outcomes	Epilepsy
Hu MD, Schenzle D, Deinhardt F, Scheid R. Epidemiology of hepatitis A and B in the Shanghai area: prevalence of serum markers. Am J Epidemiol. 1984; 120(3): 404-13	Nonfatal Health Outcomes	Acute hepatitis A
Sung RY, So LY, Ng HK, Ho JK, Fok TF. Echocardiography as a tool for determining the incidence of congenital heart disease in newborn babies: a pilot study in Hong Kong. Int J Cardiol. 1991; 30(1): 43-7	Nonfatal Health Outcomes	Congenital heart anomalies

Carolina Population Center, University of North Carolina at Chapel Hill, Chinese Center for Disease Control and Prevention (CCDC). China Health and Nutrition Survey. Chapel Hill, United States: Carolina Population Center, University of North Carolina at Chapel Hill	Nonfatal Health Outcomes	Lower respiratory infections
Carolina Population Center, University of North Carolina at Chapel Hill, Chinese Center for Disease Control and Prevention (CCDC). China Health and Nutrition Survey. Chapel Hill, United States: Carolina Population Center, University of North Carolina at Chapel Hill	Nonfatal Health Outcomes	Neonatal sepsis and other neonatal infections
Carolina Population Center, University of North Carolina at Chapel Hill, Chinese Center for Disease Control and Prevention (CCDC). China Health and Nutrition Survey. Chapel Hill, United States: Carolina Population Center, University of North Carolina at Chapel Hill	Nonfatal Health Outcomes	Cyclist road injuries
Carolina Population Center, University of North Carolina at Chapel Hill, Chinese Center for Disease Control and Prevention (CCDC). China Health and Nutrition Survey. Chapel Hill, United States: Carolina Population Center, University of North Carolina at Chapel Hill	Nonfatal Health Outcomes	Ischemic heart disease
Zhu X-X, Li C-L, Meng G-R, Zhang Y-F, Zhou G-F, Chen Q-M. Genetic investigation on schizophrenia. J Clin Psychol Med. 1992; 2(4): 193-195	Nonfatal Health Outcomes	Schizophrenia
Carolina Population Center, University of North Carolina at Chapel Hill, Chinese Center for Disease Control and Prevention (CCDC). China Health and Nutrition Survey. Chapel Hill, United States: Carolina Population Center, University of North Carolina at Chapel Hill	Nonfatal Health Outcomes	Venomous animal contact
Carolina Population Center, University of North Carolina at Chapel Hill, Chinese Center for Disease Control and Prevention (CCDC). China Health and Nutrition Survey. Chapel Hill, United States: Carolina Population Center, University of North Carolina at Chapel Hill	Nonfatal Health Outcomes	Myocarditis
Carolina Population Center, University of North Carolina at Chapel Hill, Chinese Center for Disease Control and Prevention (CCDC). China Health and Nutrition Survey. Chapel Hill, United States: Carolina Population Center, University of North Carolina at Chapel Hill	Nonfatal Health Outcomes	Coal workers pneumoconiosis
Carolina Population Center, University of North Carolina at Chapel Hill, Chinese Center for Disease Control and Prevention (CCDC). China Health and Nutrition Survey. Chapel Hill, United States: Carolina Population Center, University of North Carolina at Chapel Hill	Nonfatal Health Outcomes	Congenital musculoskeletal and limb anomalies
International Clearinghouse for Birth Defects Monitoring Systems. Congenital Malformations Worldwide: A Report from the International Clearinghouse for Birth Defects Monitoring Systems. Amsterdam, Netherlands: Elsevier, 1991	Nonfatal Health Outcomes	Urogenital congenital anomalies
Carolina Population Center, University of North Carolina at Chapel Hill, Chinese Center for Disease Control and Prevention (CCDC). China Health and Nutrition Survey. Chapel Hill, United States: Carolina Population Center, University of North Carolina at Chapel Hill	Nonfatal Health Outcomes	Asbestosis
Carolina Population Center, University of North Carolina at Chapel Hill, Chinese Center for Disease Control and Prevention (CCDC). China Health and Nutrition Survey. Chapel Hill, United States: Carolina Population Center, University of North Carolina at Chapel Hill	Nonfatal Health Outcomes	Intracerebral hemorrhage
Carolina Population Center, University of North Carolina at Chapel Hill, Chinese Center for Disease Control and Prevention (CCDC). China Health and Nutrition Survey. Chapel Hill, United States: Carolina Population Center, University of North Carolina at Chapel Hill	Nonfatal Health Outcomes	Other pneumoconiosis
Carolina Population Center, University of North Carolina at Chapel Hill, Chinese Center for Disease Control and Prevention (CCDC). China Health and Nutrition Survey. Chapel Hill, United States: Carolina Population Center, University of North Carolina at Chapel Hill	Nonfatal Health Outcomes	Adverse effects of medical treatment
Carolina Population Center, University of North Carolina at Chapel Hill, Chinese Center for Disease Control and Prevention (CCDC). China Health and Nutrition Survey. Chapel Hill, United States: Carolina Population Center, University of North Carolina at Chapel Hill	Nonfatal Health Outcomes	Encephalitis
Carolina Population Center, University of North Carolina at Chapel Hill, Chinese Center for Disease Control and Prevention (CCDC). China Health and Nutrition Survey. Chapel Hill, United States: Carolina Population Center, University of North Carolina at Chapel Hill	Nonfatal Health Outcomes	Cutaneous and mucocutaneous leishmaniasis
Carolina Population Center, University of North Carolina at Chapel Hill, Chinese Center for Disease Control and Prevention (CCDC). China Health and Nutrition Survey. Chapel Hill, United States: Carolina Population Center, University of North Carolina at Chapel Hill	Nonfatal Health Outcomes	Drowning

Carolina Population Center, University of North Carolina at Chapel Hill, Chinese Center for Disease Control and Prevention (CCDC). China Health and Nutrition Survey. Chapel Hill, United States: Carolina Population Center, University of North Carolina at Chapel Hill	Nonfatal Health Outcomes	Rheumatic heart disease
Jiang Q, Zhang S, Yuan H, Liu Z, Zhao G, Brinkmann U. The effect of a combined approach to schistosomiasis control on the transmission of Schistosoma japonicum in Xingzi of Poyang Lake area, China. Southeast Asian J Trop Med Public Health. 1996; 27(3): 535Â–41	Nonfatal Health Outcomes	Schistosomiasis
Wu Z, Shaoji Z, Pan B, Hu L, Wei R, Gao Z, Li J, Uwe B. Reinfection with Schistosoma japonicum after treatment with praziquantel in Poyang lake region, China. Southeast Asian J Trop Med Public Health. 1994; 25(1): 163Â–9	Nonfatal Health Outcomes	Schistosomiasis
Zhao G, Jiang Q, Wasley A, Zhang S, ljWu Z, Liu Z, Yuan H. Changes in prevalence, intensity of infection and morbidity due to Schistosoma japonicum infection in a community following a single treatment with praziquantel. Trop Geogr Med. 1995; 47(6): 289Â–92	Nonfatal Health Outcomes	Schistosomiasis
Tao QM, Wang Y, Wang H, Chen WR, Sun Y, Meng Q, Watanabe J, Nishioka K. Seroepidemiology of HCV and HBV infection in northern China. Gastroenterol Jpn. 1991; 156-8	Nonfatal Health Outcomes	Acute hepatitis B
Tao QM, Wang Y, Wang H, Chen WR, Sun Y, Meng Q, Watanabe J, Nishioka K. Seroepidemiology of HCV and HBV infection in northern China. Gastroenterol Jpn. 1991; 156-8	Nonfatal Health Outcomes	Acute hepatitis C
Li SL, He G, Wei MB, Tan YG, Zhu QY, Shang SM, Zhang LT, Huang KL, Zhu FZ. Epidemiological investigation of clonorchiasis in Guangxi province. Chin J Parasit Dis Cont. 2002; 15: 214-16	Nonfatal Health Outcomes	Food-borne trematodiases
Wang HT. [An epidemiological study of viral hepatitis A in Beijing suburb]. Chin J Prev Med. 1985; 19(1): 8-10	Nonfatal Health Outcomes	Acute hepatitis A
Seiji K, Inoue O, Liu SJ, Xu XP, Jin C, Cai SX, Nakatsuka H, Watanabe T, Uchida Y, Ikeda M. Prevalence of hepatitis B virus infection markers among factory workers in Beijing, China. Asia Pac J Public Health. 1991; 5(4): 345-9	Nonfatal Health Outcomes	Acute hepatitis B
Yang L, Li S, Wang W, Fang X. Epidemiologic survey of epilepsy in rural area and minority nationality in China. Chin J Neurosurg. 1989; 5: 22-7	Nonfatal Health Outcomes	Epilepsy
Zhang SY, Zou LH, Gao YQ, Di Y, Wang XD. National epidemiological survey of blindness and low vision in China. Chin Med J (Engl). 1992; 105(7): 603-8	Nonfatal Health Outcomes	Glaucoma
Carolina Population Center, University of North Carolina at Chapel Hill, Chinese Center for Disease Control and Prevention (CCDC). China Health and Nutrition Survey. Chapel Hill, United States: Carolina Population Center, University of North Carolina at Chapel Hill	Nonfatal Health Outcomes	Syphilis
Carolina Population Center, University of North Carolina at Chapel Hill, Chinese Center for Disease Control and Prevention (CCDC). China Health and Nutrition Survey. Chapel Hill, United States: Carolina Population Center, University of North Carolina at Chapel Hill	Nonfatal Health Outcomes	Motorcyclist road injuries
Carolina Population Center, University of North Carolina at Chapel Hill, Chinese Center for Disease Control and Prevention (CCDC). China Health and Nutrition Survey. Chapel Hill, United States: Carolina Population Center, University of North Carolina at Chapel Hill	Nonfatal Health Outcomes	Appendicitis
Carolina Population Center, University of North Carolina at Chapel Hill, Chinese Center for Disease Control and Prevention (CCDC). China Health and Nutrition Survey. Chapel Hill, United States: Carolina Population Center, University of North Carolina at Chapel Hill	Nonfatal Health Outcomes	Orofacial clefts
Carolina Population Center, University of North Carolina at Chapel Hill, Chinese Center for Disease Control and Prevention (CCDC). China Health and Nutrition Survey. Chapel Hill, United States: Carolina Population Center, University of North Carolina at Chapel Hill	Nonfatal Health Outcomes	Ischemic stroke
Zhang X. Investigate report of epilepsy incidence in 600,000 population. J Pract Internal Med. 1986; 6: 602-4	Nonfatal Health Outcomes	Epilepsy
Carolina Population Center, University of North Carolina at Chapel Hill, Chinese Center for Disease Control and Prevention (CCDC). China Health and Nutrition Survey. Chapel Hill, United States: Carolina Population Center, University of North Carolina at Chapel Hill	Nonfatal Health Outcomes	Cystic echinococcosis
Carolina Population Center, University of North Carolina at Chapel Hill, Chinese Center for Disease Control and Prevention (CCDC). China Health and Nutrition Survey. Chapel Hill, United States: Carolina Population Center, University of North Carolina at Chapel Hill	Nonfatal Health Outcomes	Interpersonal violence
National Central Cancer Registry (China). China Cancer Registry Incidence and Mortality 1990-2009	Nonfatal Health Outcomes	Multiple myeloma

Carolina Population Center, University of North Carolina at Chapel Hill, Chinese Center for Disease Control and Prevention (CCDC). China Health and Nutrition Survey. Chapel Hill, United States: Carolina Population Center, University of North Carolina at Chapel Hill	Nonfatal Health Outcomes	Road injuries
Zhang S-Q. Epidemiology Investigation of Mental Health in Hangzhou City. Zhejiang Med. 1990; 12(2): 9-10	Nonfatal Health Outcomes	Schizophrenia
Carolina Population Center, University of North Carolina at Chapel Hill, Chinese Center for Disease Control and Prevention (CCDC). China Health and Nutrition Survey. Chapel Hill, United States: Carolina Population Center, University of North Carolina at Chapel Hill	Nonfatal Health Outcomes	Endocrine, metabolic, blood, and immune disorders
Carolina Population Center, University of North Carolina at Chapel Hill, Chinese Center for Disease Control and Prevention (CCDC). China Health and Nutrition Survey. Chapel Hill, United States: Carolina Population Center, University of North Carolina at Chapel Hill	Nonfatal Health Outcomes	Neonatal preterm birth
Centre for Research on the Epidemiology of Disasters (CRED). EM-DAT: The OFDA/CRED International Disaster Database. Brussels, Belgium: Catholic University of Leuven	Nonfatal Health Outcomes	Injuries
Cystic Echinococcosis Endemicity Estimates identified through systematic review and personal communication, as provided by GBD 2015 expert. [Unpublished]	Nonfatal Health Outcomes	Cystic echinococcosis
Carolina Population Center, University of North Carolina at Chapel Hill, Chinese Center for Disease Control and Prevention (CCDC). China Health and Nutrition Survey. Chapel Hill, United States: Carolina Population Center, University of North Carolina at Chapel Hill	Nonfatal Health Outcomes	Gastritis and duodenitis
Carolina Population Center, University of North Carolina at Chapel Hill, Chinese Center for Disease Control and Prevention (CCDC). China Health and Nutrition Survey. Chapel Hill, United States: Carolina Population Center, University of North Carolina at Chapel Hill	Nonfatal Health Outcomes	Maternal sepsis and other maternal infections
Carolina Population Center, University of North Carolina at Chapel Hill, Chinese Center for Disease Control and Prevention (CCDC). China Health and Nutrition Survey. Chapel Hill, United States: Carolina Population Center, University of North Carolina at Chapel Hill	Nonfatal Health Outcomes	Polycystic ovarian syndrome
Carolina Population Center, University of North Carolina at Chapel Hill, Chinese Center for Disease Control and Prevention (CCDC). China Health and Nutrition Survey. Chapel Hill, United States: Carolina Population Center, University of North Carolina at Chapel Hill	Nonfatal Health Outcomes	Pancreatitis
Carolina Population Center, University of North Carolina at Chapel Hill, Chinese Center for Disease Control and Prevention (CCDC). China Health and Nutrition Survey. Chapel Hill, United States: Carolina Population Center, University of North Carolina at Chapel Hill	Nonfatal Health Outcomes	Genital prolapse
Jin T-A, Wan X-D, Cao Y-M, Zhu X-Y, Che H-L, Wu Y-P. Epidemiological investigation on alcohol dependence in Guiyang city. J Guiyand Med Coll. 1998; 23(2): 84-85	Nonfatal Health Outcomes	Alcohol use disorders
Carolina Population Center, University of North Carolina at Chapel Hill, Chinese Center for Disease Control and Prevention (CCDC). China Health and Nutrition Survey. Chapel Hill, United States: Carolina Population Center, University of North Carolina at Chapel Hill	Nonfatal Health Outcomes	Inflammatory bowel disease
Carolina Population Center, University of North Carolina at Chapel Hill, Chinese Center for Disease Control and Prevention (CCDC). China Health and Nutrition Survey. Chapel Hill, United States: Carolina Population Center, University of North Carolina at Chapel Hill	Nonfatal Health Outcomes	Uterine fibroids
Carolina Population Center, University of North Carolina at Chapel Hill, Chinese Center for Disease Control and Prevention (CCDC). China Health and Nutrition Survey. Chapel Hill, United States: Carolina Population Center, University of North Carolina at Chapel Hill	Nonfatal Health Outcomes	Neural tube defects
International Clearinghouse for Birth Defects Monitoring Systems. Congenital Malformations Worldwide: A Report from the International Clearinghouse for Birth Defects Monitoring Systems. Amsterdam, Netherlands: Elsevier, 1991	Nonfatal Health Outcomes	Congenital birth defects
Carolina Population Center, University of North Carolina at Chapel Hill, Chinese Center for Disease Control and Prevention (CCDC). China Health and Nutrition Survey. Chapel Hill, United States: Carolina Population Center, University of North Carolina at Chapel Hill	Nonfatal Health Outcomes	Decubitus ulcer
Carolina Population Center, University of North Carolina at Chapel Hill, Chinese Center for Disease Control and Prevention (CCDC). China Health and Nutrition Survey. Chapel Hill, United States: Carolina Population Center, University of North Carolina at Chapel Hill	Nonfatal Health Outcomes	Non-venomous animal contact

Carolina Population Center, University of North Carolina at Chapel Hill, Chinese Center for Disease Control and Prevention (CCDC). China Health and Nutrition Survey. Chapel Hill, United States: Carolina Population Center, University of North Carolina at Chapel Hill	Nonfatal Health Outcomes	Turner syndrome
Carolina Population Center, University of North Carolina at Chapel Hill, Chinese Center for Disease Control and Prevention (CCDC). China Health and Nutrition Survey. Chapel Hill, United States: Carolina Population Center, University of North Carolina at Chapel Hill	Nonfatal Health Outcomes	Maternal abortion, miscarriage, and ectopic pregnancy
Carolina Population Center, University of North Carolina at Chapel Hill, Chinese Center for Disease Control and Prevention (CCDC). China Health and Nutrition Survey. Chapel Hill, United States: Carolina Population Center, University of North Carolina at Chapel Hill	Nonfatal Health Outcomes	Endocarditis
Carolina Population Center, University of North Carolina at Chapel Hill, Chinese Center for Disease Control and Prevention (CCDC). China Health and Nutrition Survey. Chapel Hill, United States: Carolina Population Center, University of North Carolina at Chapel Hill	Nonfatal Health Outcomes	Acute glomerulonephritis
Carolina Population Center, University of North Carolina at Chapel Hill, Chinese Center for Disease Control and Prevention (CCDC). China Health and Nutrition Survey. Chapel Hill, United States: Carolina Population Center, University of North Carolina at Chapel Hill	Nonfatal Health Outcomes	Fire, heat, and hot substances
Carolina Population Center, University of North Carolina at Chapel Hill, Chinese Center for Disease Control and Prevention (CCDC). China Health and Nutrition Survey. Chapel Hill, United States: Carolina Population Center, University of North Carolina at Chapel Hill	Nonfatal Health Outcomes	Unintentional suffocation
Carolina Population Center, University of North Carolina at Chapel Hill, Chinese Center for Disease Control and Prevention (CCDC). China Health and Nutrition Survey. Chapel Hill, United States: Carolina Population Center, University of North Carolina at Chapel Hill	Nonfatal Health Outcomes	Vascular intestinal disorders
Carolina Population Center, University of North Carolina at Chapel Hill, Chinese Center for Disease Control and Prevention (CCDC). China Health and Nutrition Survey. Chapel Hill, United States: Carolina Population Center, University of North Carolina at Chapel Hill	Nonfatal Health Outcomes	Diarrheal diseases
Carolina Population Center, University of North Carolina at Chapel Hill, Chinese Center for Disease Control and Prevention (CCDC). China Health and Nutrition Survey. Chapel Hill, United States: Carolina Population Center, University of North Carolina at Chapel Hill	Nonfatal Health Outcomes	Pyoderma
Carolina Population Center, University of North Carolina at Chapel Hill, Chinese Center for Disease Control and Prevention (CCDC). China Health and Nutrition Survey. Chapel Hill, United States: Carolina Population Center, University of North Carolina at Chapel Hill	Nonfatal Health Outcomes	Exposure to mechanical forces
Carolina Population Center, University of North Carolina at Chapel Hill, Chinese Center for Disease Control and Prevention (CCDC). China Health and Nutrition Survey. Chapel Hill, United States: Carolina Population Center, University of North Carolina at Chapel Hill	Nonfatal Health Outcomes	Pedestrian road injuries
Carolina Population Center, University of North Carolina at Chapel Hill, Chinese Center for Disease Control and Prevention (CCDC). China Health and Nutrition Survey. Chapel Hill, United States: Carolina Population Center, University of North Carolina at Chapel Hill	Nonfatal Health Outcomes	Down syndrome
Carolina Population Center, University of North Carolina at Chapel Hill, Chinese Center for Disease Control and Prevention (CCDC). China Health and Nutrition Survey. Chapel Hill, United States: Carolina Population Center, University of North Carolina at Chapel Hill	Nonfatal Health Outcomes	Congenital heart anomalies
Zhao L-Z, Zhang L-P, Shen Y-R, Feng M. Epidemiological Investigation on Alcohol Dependence in Changchun Area. Chin J Drug Abuse Prev Treat. 1996; 4(7): 17-20	Nonfatal Health Outcomes	Alcohol use disorders
International Clearinghouse for Birth Defects Monitoring Systems. Congenital Malformations Worldwide: A Report from the International Clearinghouse for Birth Defects Monitoring Systems. Amsterdam, Netherlands: Elsevier, 1991	Nonfatal Health Outcomes	Orofacial clefts
Carolina Population Center, University of North Carolina at Chapel Hill, Chinese Center for Disease Control and Prevention (CCDC). China Health and Nutrition Survey. Chapel Hill, United States: Carolina Population Center, University of North Carolina at Chapel Hill	Nonfatal Health Outcomes	Meningitis
Carolina Population Center, University of North Carolina at Chapel Hill, Chinese Center for Disease Control and Prevention (CCDC). China Health and Nutrition Survey. Chapel Hill, United States: Carolina Population Center, University of North Carolina at Chapel Hill	Nonfatal Health Outcomes	Cirrhosis and other chronic liver diseases

Gong J, Yang JY, Li RC, Wang SS, Li YP, Chen KL. Hepatitis C virus and primary liver cancer in a hyperendemic area of liver cancer. Chin J Public Health. 1993; 12: 328-330	Nonfatal Health Outcomes	Liver cancer due to hepatitis B
Zhang ZQ, Huang TR, He ZF, Yu JH, Xu QF, Huang ZD. The relationship between HCV and HBV in the etiology of primary liver cancer. Guangxi Med J. 1993; 15: 171-4	Nonfatal Health Outcomes	Liver cancer due to hepatitis B
Gong J, Yang JY, Li RC, Wang SS, Li YP, Chen KL. Hepatitis C virus and primary liver cancer in a hyperendemic area of liver cancer. Chin J Public Health. 1993; 12: 328-330	Nonfatal Health Outcomes	Liver cancer due to hepatitis C
International Clearinghouse for Birth Defects Monitoring Systems. Congenital Malformations Worldwide: A Report from the International Clearinghouse for Birth Defects Monitoring Systems. Amsterdam, Netherlands: Elsevier, 1991	Nonfatal Health Outcomes	Down syndrome
Carolina Population Center, University of North Carolina at Chapel Hill, Chinese Center for Disease Control and Prevention (CCDC). China Health and Nutrition Survey. Chapel Hill, United States: Carolina Population Center, University of North Carolina at Chapel Hill	Nonfatal Health Outcomes	Environmental heat and cold exposure
Carolina Population Center, University of North Carolina at Chapel Hill, Chinese Center for Disease Control and Prevention (CCDC). China Health and Nutrition Survey. Chapel Hill, United States: Carolina Population Center, University of North Carolina at Chapel Hill	Nonfatal Health Outcomes	Foreign body in eyes
Carolina Population Center, University of North Carolina at Chapel Hill, Chinese Center for Disease Control and Prevention (CCDC). China Health and Nutrition Survey. Chapel Hill, United States: Carolina Population Center, University of North Carolina at Chapel Hill	Nonfatal Health Outcomes	Klinefelter syndrome
Carolina Population Center, University of North Carolina at Chapel Hill, Chinese Center for Disease Control and Prevention (CCDC). China Health and Nutrition Survey. Chapel Hill, United States: Carolina Population Center, University of North Carolina at Chapel Hill	Nonfatal Health Outcomes	Maternal hypertensive disorders
International Clearinghouse for Birth Defects Monitoring Systems. Congenital Malformations Worldwide: A Report from the International Clearinghouse for Birth Defects Monitoring Systems. Amsterdam, Netherlands: Elsevier, 1991	Nonfatal Health Outcomes	Digestive congenital anomalies
Cheng SY, Luk NM, Chong LY. Special features of non-melanoma skin cancer in Hong Kong Chinese patients: 10-year retrospective study. Hong Kong Med J. 2001; 7(1): 22-8	Nonfatal Health Outcomes	Non-melanoma skin cancer (basal-cell carcinoma)
Carolina Population Center, University of North Carolina at Chapel Hill, Chinese Center for Disease Control and Prevention (CCDC). China Health and Nutrition Survey. Chapel Hill, United States: Carolina Population Center, University of North Carolina at Chapel Hill	Nonfatal Health Outcomes	Peptic ulcer disease
Carolina Population Center, University of North Carolina at Chapel Hill, Chinese Center for Disease Control and Prevention (CCDC). China Health and Nutrition Survey. Chapel Hill, United States: Carolina Population Center, University of North Carolina at Chapel Hill	Nonfatal Health Outcomes	Unintentional firearm injuries
Carolina Population Center, University of North Carolina at Chapel Hill, Chinese Center for Disease Control and Prevention (CCDC). China Health and Nutrition Survey. Chapel Hill, United States: Carolina Population Center, University of North Carolina at Chapel Hill	Nonfatal Health Outcomes	Physical violence by sharp object
Carolina Population Center, University of North Carolina at Chapel Hill, Chinese Center for Disease Control and Prevention (CCDC). China Health and Nutrition Survey. Chapel Hill, United States: Carolina Population Center, University of North Carolina at Chapel Hill	Nonfatal Health Outcomes	Self-harm by firearm
Carolina Population Center, University of North Carolina at Chapel Hill, Chinese Center for Disease Control and Prevention (CCDC). China Health and Nutrition Survey. Chapel Hill, United States: Carolina Population Center, University of North Carolina at Chapel Hill	Nonfatal Health Outcomes	Silicosis
National Central Cancer Registry (China). China Cancer Registry Incidence and Mortality 1990-2009	Nonfatal Health Outcomes	Gallbladder and biliary tract cancer
Zhang SY, Zou LH, Gao YQ, Di Y, Wang XD. National epidemiological survey of blindness and low vision in China. Chin Med J (Engl). 1992; 105(7): 603-8	Nonfatal Health Outcomes	Cataract
Zhu X-G, Wan C, Lan Z-S, Chen J-P. Epidemic investigation of the mental disorders in Yichun city, Jiangxi province. Chin J Epidemiol. 2004; 6: 526	Nonfatal Health Outcomes	Schizophrenia
Centre for Research on the Epidemiology of Disasters (CRED). EM-DAT: The OFDA/CRED International Disaster Database. Brussels, Belgium: Catholic University of Leuven	Nonfatal Health Outcomes	Protein-energy malnutrition
Zhang SY, Zou LH, Gao YQ, Di Y, Wang XD. National epidemiological survey of blindness and low vision in China. Chin Med J (Engl). 1992; 105(7): 603-8	Nonfatal Health Outcomes	Trachoma

Carolina Population Center, University of North Carolina at Chapel Hill, Chinese Center for Disease Control and Prevention (CCDC). China Health and Nutrition Survey. Chapel Hill, United States: Carolina Population Center, University of North Carolina at Chapel Hill	Nonfatal Health Outcomes	Falls
Carolina Population Center, University of North Carolina at Chapel Hill, Chinese Center for Disease Control and Prevention (CCDC). China Health and Nutrition Survey. Chapel Hill, United States: Carolina Population Center, University of North Carolina at Chapel Hill	Nonfatal Health Outcomes	Other unintentional injuries
Dong Y. Female simple gonorrhoea. J Pract Obstret Gynaecol. 1991; 172-4	Nonfatal Health Outcomes	Sexually transmitted infections excluding HIV
Zhang SY, Zou LH, Gao YQ, Di Y, Wang XD. National epidemiological survey of blindness and low vision in China. Chin Med J (Engl). 1992; 105(7): 603-8	Nonfatal Health Outcomes	Other vision loss
Li SZ. [Epidemiologic study of epilepsy in six cities in China]. Chin J Neurol Psychiatry. 1986; 19(4): 193-6	Nonfatal Health Outcomes	Epilepsy
Carolina Population Center, University of North Carolina at Chapel Hill, Chinese Center for Disease Control and Prevention (CCDC). China Health and Nutrition Survey. Chapel Hill, United States: Carolina Population Center, University of North Carolina at Chapel Hill	Nonfatal Health Outcomes	Cellulitis
Xue JT. [An investigation of the prevalence of epilepsy in three northwestern provinces of China]. Chin J Epidemiol. 1987; 8(3): 165-7	Nonfatal Health Outcomes	Epilepsy
Hao T, Min-Tian D, Liang C. Five provinces of East China adult epilepsy epidemiological survey. J Clin Neurol. 1991; 3: 139-42	Nonfatal Health Outcomes	Epilepsy
Li XH, Li TL, Yang Z, Liu ZY, Wei YD, Jin SX, Hong C, Qin RL, Li YQ, Dorman JS, Laporte RE, Wang KA. A nine-year prospective study on the incidence of childhood type 1 diabetes mellitus in China. Biomed Environ Sci. 2000; 13(4): 263-70	Nonfatal Health Outcomes	Diabetes mellitus type 1
Association S of CCM, Cheng K. Retrospective investigation of hospitalized patients with heart failure in some parts of China, in 1980, 1990 and 2000. Chin J Cardiol. 2002; 30(8): 450-4	Nonfatal Health Outcomes	Ischemic heart disease
Chen H, Wu X, Wei M, Eichner JE, Fan Y, Zhang Z, Lei C, Stone DU, Yang J. Changes in the prevalence of visual impairment due to blinding trachoma in Sichuan province, China: a comparative study between 1987 and 2006. Ophthalmic Epidemiol. 2012; 19(1): 29-37	Nonfatal Health Outcomes	Trachoma
Association S of CCM, Cheng K. Retrospective investigation of hospitalized patients with heart failure in some parts of China, in 1980, 1990 and 2000. Chin J Cardiol. 2002; 30(8): 450-4	Nonfatal Health Outcomes	Hypertensive heart disease
Carolina Population Center, University of North Carolina at Chapel Hill, Chinese Center for Disease Control and Prevention (CCDC). China Health and Nutrition Survey. Chapel Hill, United States: Carolina Population Center, University of North Carolina at Chapel Hill	Nonfatal Health Outcomes	Physical violence by other means
Lam BC, Yeung CY. Perinatal features of birth asphyxia and neurologic outcome. Acta Paediatr Jpn. 1992; 34(1): 17-22	Nonfatal Health Outcomes	Neonatal disorders
Carolina Population Center, University of North Carolina at Chapel Hill, Chinese Center for Disease Control and Prevention (CCDC). China Health and Nutrition Survey. Chapel Hill, United States: Carolina Population Center, University of North Carolina at Chapel Hill	Nonfatal Health Outcomes	Self-harm by other specified means
Tunstall-Pedoe H, Kuulasmaa K, MÃ¤hÃ¶nen M, Tolonen H, Ruokokoski E. Contribution of trends in survival and coronary-event rates to changes in coronary heart disease mortality: 10-year results from 37 WHO MONICA Project populations. Lancet. 1999; 353(9164): 1547-57	Nonfatal Health Outcomes	Ischemic heart disease
Li G, Shen YC, Chen CH, Zhau YW, Li SR, Lu M. A three-year follow-up study of age-related dementia in an urban area of Beijing. Acta Psychiatr Scand. 1991; 83(2): 99-104	Nonfatal Health Outcomes	Alzheimer's disease and other dementias
Li YS, Lin JX. [Epidemiological survey of paragonimiasis in Fujian Province]. Chin J Prev Med. 1987; 21(6): 331-4	Nonfatal Health Outcomes	Food-borne trematodiases
Association S of CCM, Cheng K. Retrospective investigation of hospitalized patients with heart failure in some parts of China, in 1980, 1990 and 2000. Chin J Cardiol. 2002; 30(8): 450-4	Nonfatal Health Outcomes	Rheumatic heart disease
Association S of CCM, Cheng K. Retrospective investigation of hospitalized patients with heart failure in some parts of China, in 1980, 1990 and 2000. Chin J Cardiol. 2002; 30(8): 450-4	Nonfatal Health Outcomes	Alcoholic cardiomyopathy
Association S of CCM, Cheng K. Retrospective investigation of hospitalized patients with heart failure in some parts of China, in 1980, 1990 and 2000. Chin J Cardiol. 2002; 30(8): 450-4	Nonfatal Health Outcomes	Myocarditis
Association S of CCM, Cheng K. Retrospective investigation of hospitalized patients with heart failure in some parts of China, in 1980, 1990 and 2000. Chin J Cardiol. 2002; 30(8): 450-4	Nonfatal Health Outcomes	Other cardiomyopathy

Huang L-S, Chen Y-F, Lin X-Z, Wu Y-T. A 10 year investigation on incidence of schizophrenia in community. Chin J Nerv Ment Dis. 1990; 16(2): 100-103	Nonfatal Health Outcomes	Schizophrenia
Zhang MY, Katzman R, Salmon D, Jin H, Cai GJ, Wang ZY, Qu GY, Grant I, Yu E, Levy P. The prevalence of dementia and Alzheimer's disease in Shanghai, China: impact of age, gender, and education. Ann Neurol. 1990; 27(4): 428-37	Nonfatal Health Outcomes	Alzheimer's disease and other dementias
By Gao ZX, Xie B, Fang YS, Pan JX. The Prevalence of Dementia Among Elderly in Shanghai Area, China- A Survey in City, Town and Countryside. Hong Kong J Psychiatry. 1999; 9(1): 12-4	Nonfatal Health Outcomes	Alzheimer's disease and other dementias
Li XH, Li TL, Yang Z, Liu ZY, Wei YD, Jin SX, Hong C, Qin RL, Li YQ, Dorman JS, Laporte RE, Wang KA. A nine-year prospective study on the incidence of childhood type 1 diabetes mellitus in China. Biomed Environ Sci. 2000; 13(4): 263-70	Nonfatal Health Outcomes	Diabetes mellitus
Song W-H. An epidemiologic investigation of mental disorders in the rural area of Meishan County. West China Med J. 1988; 3(1): 10-1	Nonfatal Health Outcomes	Schizophrenia
Duan C-F, Xu X-Q, Yuan H-H, Mo F, Zhang X-S. Epidemiological survey on mental disorders in 5 jurisdictional streets in central urban of Chongqing city. Sichuan Ment Health. 1989; 2(1): 1-5	Nonfatal Health Outcomes	Schizophrenia
Wei B-X, Hu X-L, Chen J-M. The epidemiological investigation on mental disorders in Mianyang city. Sichuan Ment Health. 1994; 7(1): 50-1	Nonfatal Health Outcomes	Alcohol use disorders
Song W-H. An epidemiologic investigation of mental disorders in the rural area of Meishan County. West China Med J. 1988; 3(1): 10-1	Nonfatal Health Outcomes	Alcohol use disorders
Deng J-L, He G-Y, Jiang X-M, Mao W-J, Ouyang X-W, Guo J, Wang Q-R, Song C-L. Epidemiological investigation on mental disorders among elderlies in Chengdu city. Chin J Gerontol. 1988; 8(5): 277-279	Nonfatal Health Outcomes	Alcohol use disorders
Lu X, Wu S, Gu X, Li L, Zhang G, Sun W, Yu J. Long-term results of surgical treatment of tetralogy of Fallot in adults. Thorac Cardiovasc Surg. 2006; 54(5): 295-9	Nonfatal Health Outcomes	Congenital birth defects
Chen W-H, Lu S-W, Ma Q, Zhuang Y-G, Lin J-G, Luo W-W. Mental disease epidemiology research in 5 areas of Fujian I, research methods and data analysis. Fujian Med J. 1988; 10(8): 45-46	Nonfatal Health Outcomes	Schizophrenia
Li L. [The relationship between changing of anti-HAV in population and prevalence of hepatitis A]. Chin J Prev Med. 1989; 23(3): 139-40	Nonfatal Health Outcomes	Acute hepatitis A
Ou Z-Y. [The general situation of clonorchiasis sinensis in Guangdong Province]. Ann Bull Soc Parasitol Guangdong Prov. 1997; 19: 52-62	Nonfatal Health Outcomes	Food-borne trematodiases
National Bureau of Statistics of China, China Population Information Center (CIPC). China In-depth Fertility Sample Survey 1985. Hong Kong, China: Chinese University of Hong Kong	Nonfatal Health Outcomes	Infertility
China Population Information and Research Center, National Bureau of Statistics of China. China In-Depth Fertility Sample Survey 1987	Nonfatal Health Outcomes	Infertility
World Health Organization (WHO). WHO Global Database on Vitamin A Deficiency. Geneva, Switzerland: World Health Organization (WHO)	Nonfatal Health Outcomes	Vitamin A deficiency
World Health Organization (WHO). WHO Global Database on Vitamin A Deficiency. Geneva, Switzerland: World Health Organization (WHO)	Nonfatal Health Outcomes	Nutritional deficiencies
Lu X, Wu S, Gu X, Li L, Zhang G, Sun W, Yu J. Long-term results of surgical treatment of tetralogy of Fallot in adults. Thorac Cardiovasc Surg. 2006; 54(5): 295-9	Nonfatal Health Outcomes	Congenital heart anomalies
Hou JB, Zhang ZX. Prevalence of multiple sclerosis: a door-to-door survey in Lan Cang La Hu Zu Autonomous County, Yunnan Province of China. Neuroepidemiology. 1992; 11(1): 52	Nonfatal Health Outcomes	Multiple sclerosis
Yang J, Kang C, Zeng Y, Li J, Li P, Wan W, Zhao X, Guo W, Xu X, Yang X. Prevalence and prognosis of schizophrenia in Jinuo people in China: A prospective 30-year follow-up study. Int J Soc Psychiatry. 2014; 60(5): 482Â–8	Nonfatal Health Outcomes	Schizophrenia
Hong Y, Bots ML, Pan X, Hofman A, Grobbee DE, Chen H. Stroke Incidence and Mortality in Rural and Urban Shanghai From 1984 Through 1991: Findings From a Community-Based Registry. Stroke. 1994; 25(6): 1165-9	Nonfatal Health Outcomes	Ischemic stroke
Hong Y, Bots ML, Pan X, Hofman A, Grobbee DE, Chen H. Stroke Incidence and Mortality in Rural and Urban Shanghai From 1984 Through 1991: Findings From a Community-Based Registry. Stroke. 1994; 25(6): 1165-9	Nonfatal Health Outcomes	Intracerebral hemorrhage
Lv Q-Y, Wang Y-F, Shen Y-C. Epidemiological investigation report on alcohol dependence in Yunnan, Simao and Xishuangbanna. Chin Ment Health J. 1987; 1(6): 253-256	Nonfatal Health Outcomes	Alcohol use disorders
Hong Y, Bots ML, Pan X, Hofman A, Grobbee DE, Chen H. Stroke Incidence and Mortality in Rural and Urban Shanghai From 1984 Through 1991: Findings From a Community-Based Registry. Stroke. 1994; 25(6): 1165-9	Nonfatal Health Outcomes	Subarachnoid hemorrhage
Shen YC, Li G, Li YT, Chen CH, Li SR, Zhao YW, Zhang WX. Epidemiology of age-related dementia in China. Chin Med J (Engl). 1994; 107(1): 60-4	Nonfatal Health Outcomes	Alzheimer's disease and other dementias
Jiang G, Li W, Wang D, Shen C, Ji Y, Zheng W. Epidemiological transition and distribution of stroke incidence in Tianjin, China, 1988-2010. Public Health. 2016; 131: 11-9	Nonfatal Health Outcomes	Ischemic stroke

Chen H, Wu X, Wei M, Eichner JE, Fan Y, Zhang Z, Lei C, Stone DU, Yang J. Changes in the prevalence of visual impairment due to blinding trachoma in Sichuan province, China: a comparative study between 1987 and 2006. Ophthalmic Epidemiol. 2012; 19(1): 29-37	Nonfatal Health Outcomes	
Jiang G, Li W, Wang D, Shen C, Ji Y, Zheng W. Epidemiological transition and distribution of stroke incidence in Tianjin, China, 1988-2010. Public Health. 2016; 131: 11-9	Nonfatal Health Outcomes	Intracerebral hemorrhage
Tao Tao, Si-Ming Chang, Shu-Huai Su. Epidemiological survey of Chengguan Townm Dushan District, Guizhou Province. Guizhou Med J. 1994; 120-1	Nonfatal Health Outcomes	Epilepsy
Carolina Population Center, University of North Carolina at Chapel Hill, Chinese Center for Disease Control and Prevention (CCDC). China Health and Nutrition Survey. Chapel Hill, United States: Carolina Population Center, University of North Carolina at Chapel Hill	Nonfatal Health Outcomes	Visceral leishmaniasis
Wu Y, Yao C, Wu Z, Zhang R, Zhang M, Wu G, Zhao D, Hong Z. Interim report of Sino-Monica-Beijing for the years 1985-1989. Chin Med Sci J. 1992; 7(3): 125-9	Nonfatal Health Outcomes	Ischemic heart disease
Li MG. [Epidemiological survey on paragonimiasis in Kaiyang, Wansan, Jiangkou Counties, Guizhou]. Chin J Parasitol Parasit Dis. 1984; 2(1): 55	Nonfatal Health Outcomes	Food-borne trematodiases
Zou QH. [A seroepidemiological study of hepatitis A viral infection]. Chin J Epidemiol. 1984; 5(6): 329-31	Nonfatal Health Outcomes	Acute hepatitis A
Carolina Population Center, University of North Carolina at Chapel Hill, Chinese Center for Disease Control and Prevention (CCDC). China Health and Nutrition Survey. Chapel Hill, United States: Carolina Population Center, University of North Carolina at Chapel Hill	Nonfatal Health Outcomes	Gallbladder and biliary diseases
Carolina Population Center, University of North Carolina at Chapel Hill, Chinese Center for Disease Control and Prevention (CCDC). China Health and Nutrition Survey. Chapel Hill, United States: Carolina Population Center, University of North Carolina at Chapel Hill	Nonfatal Health Outcomes	Endometriosis
Carolina Population Center, University of North Carolina at Chapel Hill, Chinese Center for Disease Control and Prevention (CCDC). China Health and Nutrition Survey. Chapel Hill, United States: Carolina Population Center, University of North Carolina at Chapel Hill	Nonfatal Health Outcomes	Other road injuries
Carolina Population Center, University of North Carolina at Chapel Hill, Chinese Center for Disease Control and Prevention (CCDC). China Health and Nutrition Survey. Chapel Hill, United States: Carolina Population Center, University of North Carolina at Chapel Hill	Nonfatal Health Outcomes	Physical violence by firearm
Carolina Population Center, University of North Carolina at Chapel Hill, Chinese Center for Disease Control and Prevention (CCDC). China Health and Nutrition Survey. Chapel Hill, United States: Carolina Population Center, University of North Carolina at Chapel Hill	Nonfatal Health Outcomes	Inguinal, femoral, and abdominal hernia
Carolina Population Center, University of North Carolina at Chapel Hill, Chinese Center for Disease Control and Prevention (CCDC). China Health and Nutrition Survey. Chapel Hill, United States: Carolina Population Center, University of North Carolina at Chapel Hill	Nonfatal Health Outcomes	Benign prostatic hyperplasia
Carolina Population Center, University of North Carolina at Chapel Hill, Chinese Center for Disease Control and Prevention (CCDC). China Health and Nutrition Survey. Chapel Hill, United States: Carolina Population Center, University of North Carolina at Chapel Hill	Nonfatal Health Outcomes	Atrial fibrillation and flutter
Carolina Population Center, University of North Carolina at Chapel Hill, Chinese Center for Disease Control and Prevention (CCDC). China Health and Nutrition Survey. Chapel Hill, United States: Carolina Population Center, University of North Carolina at Chapel Hill	Nonfatal Health Outcomes	Animal contact
Carolina Population Center, University of North Carolina at Chapel Hill, Chinese Center for Disease Control and Prevention (CCDC). China Health and Nutrition Survey. Chapel Hill, United States: Carolina Population Center, University of North Carolina at Chapel Hill	Nonfatal Health Outcomes	Urinary tract infections
Carolina Population Center, University of North Carolina at Chapel Hill, Chinese Center for Disease Control and Prevention (CCDC). China Health and Nutrition Survey. Chapel Hill, United States: Carolina Population Center, University of North Carolina at Chapel Hill	Nonfatal Health Outcomes	Poisonings
Carolina Population Center, University of North Carolina at Chapel Hill, Chinese Center for Disease Control and Prevention (CCDC). China Health and Nutrition Survey. Chapel Hill, United States: Carolina Population Center, University of North Carolina at Chapel Hill	Nonfatal Health Outcomes	Neonatal encephalopathy due to birth asphyxia and trauma

Carolina Population Center, University of North Carolina at Chapel Hill, Chinese Center for Disease Control and Prevention (CCDC). China Health and Nutrition Survey. Chapel Hill, United States: Carolina Population Center, University of North Carolina at Chapel Hill	Nonfatal Health Outcomes	Other exposure to mechanical forces
National Central Cancer Registry (China). China Cancer Registry Incidence and Mortality 1990-2009	Nonfatal Health Outcomes	Ovarian cancer
Xu B, Cui Z, Zhang Y, Chang J, Zhao Q, Huang Q, Lin X. Studies on the transmission potential of filariasis in controlled areas of Henan Province. Chin Med J (Engl). 1997; 110(10): 807-10	Nonfatal Health Outcomes	Lymphatic filariasis
Cao WC, Xu JF, Ren ZX. Epidemiological surveillance of filariasis after its control in Shandong Province, China. Southeast Asian J Trop Med Public Health. 1994; 25(4): 714-8	Nonfatal Health Outcomes	Lymphatic filariasis
Zhong CH, Zhen TM. Control and surveillance of filariasis in Shandong. Chin Med J (Engl). 1991; 104(3): 179-85	Nonfatal Health Outcomes	Lymphatic filariasis
International Clearinghouse for Birth Defects Monitoring Systems. Congenital Malformations Worldwide: A Report from the International Clearinghouse for Birth Defects Monitoring Systems. Amsterdam, Netherlands: Elsevier, 1991	Nonfatal Health Outcomes	Congenital musculoskeletal and limb anomalies
Song Y. [The investigation of anti-hepatitis A virus antibody rate in Guangzhou population]. Chin J Epidemiol. 1991; 12(5): 265-8	Nonfatal Health Outcomes	Acute hepatitis A
Hui ACF, Chow KM, Tang ASY, Fu M, Kay R, Wong KS. Electrophysiological, clinical and epidemiological study of Guillain-Barre Syndrome in Hong Kong Chinese. J Clin Neurosci. 2005; 12(2): 134-6	Nonfatal Health Outcomes	Guillain-BarrÃ© syndrome
Gao J, Zeng S, Sun BL, Fan HM, Han LH. Menstrual blood loss and hematologic indices in healthy Chinese women. J Reprod Med. 1987; 32(11): 822-6	Nonfatal Health Outcomes	Dietary iron deficiency
Shen YC, Wang YF, Yang XL. An epidemiological investigation of minimal brain dysfunction in six elementary schools in Beijing. J Child Psychol Psychiatry. 1985; 26(5): 777-87	Nonfatal Health Outcomes	Attention-deficit/hyperactivity disorder
Zhang ZJ, Gao LM, Wang ZL, Cao YP, Wu GC, Zhu ZH. Acute respiratory infections in Beijing children Epidemiological studies at Dongguan Brigade. Chin Med J (Engl). 1986; 7(99): 561-8	Nonfatal Health Outcomes	Lower respiratory infections
Zeng DL, Zhang ZJ, Wang ZL, Zhang JN, Yang ML. Surveillance of acute respiratory infections in three kindergartens in Beijing. Chin Med J (Engl). 1988; 11(101): 787-92	Nonfatal Health Outcomes	Lower respiratory infections
Zeng DL, Zhang ZJ, Wang ZL, Zhang JN, Yang ML. Surveillance of acute respiratory infections in three kindergartens in Beijing. Chin Med J (Engl). 1988; 11(101): 787-92	Nonfatal Health Outcomes	Upper respiratory infections
Zhang ZJ, Gao LM, Wang ZL, Cao YP, Wu GC, Zhu ZH. Epidemiological studies at Dongguan Brigade. Chin Med J (Engl). 1986; 99(7): 561-8	Nonfatal Health Outcomes	Upper respiratory infections
Shandong Province Mental Illness Coordination Group. 10 years comparison of alcohol dependence in Shandong Province. Shandong Psychiatry. 1998; 11(2): 43-47	Nonfatal Health Outcomes	Alcohol use disorders
World Health Organization (WHO). WHO DengueNet. Geneva, Switzerland: World Health Organization (WHO)	Nonfatal Health Outcomes	Dengue
World Health Organization (WHO). Leprosy control in China: trends in detection of new cases, 1987-2008. Wkly Epidemiol Rec. 2010: 85(17): 149-56	Nonfatal Health Outcomes	Leprosy
Dong Y. Female simple gonorrhoea. J Pract Obstret Gynaecol. 1991; 172-4	Nonfatal Health Outcomes	Gonococcal infection
Sung RYT, Cheng AFB, Chan RCK, Tam JS, Oppenheimer SJ. Epidemiology and Etiology of Pneumonia in Children in Hong Kong. Clin Infect Dis. 1993; 17(5): 894-6	Nonfatal Health Outcomes	Influenza
Sung RYT, Cheng AFB, Chan RCK, Tam JS, Oppenheimer SJ. Epidemiology and Etiology of Pneumonia in Children in Hong Kong. Clin Infect Dis. 1993; 17(5): 894-6	Nonfatal Health Outcomes	Respiratory syncytial virus pneumonia
Sung RY, Senok AC, Ho A, Oppenheimer SJ, Davies DP. Meningitis in Hong Kong children, with special reference to the infrequency of haemophilus and meningococcal infection. J Paediatr Child Health. 1997; 33(4): 296-9	Nonfatal Health Outcomes	Meningitis
Sung RY, Senok AC, Ho A, Oppenheimer SJ, Davies DP. Meningitis in Hong Kong children, with special reference to the infrequency of haemophilus and meningococcal infection. J Paediatr Child Health. 1997; 33(4): 296-9	Nonfatal Health Outcomes	Pneumococcal meningitis
Sung RY, Senok AC, Ho A, Oppenheimer SJ, Davies DP. Meningitis in Hong Kong children, with special reference to the infrequency of haemophilus and meningococcal infection. J Paediatr Child Health. 1997; 33(4): 296-9	Nonfatal Health Outcomes	H influenzae type B meningitis
Sung RY, Senok AC, Ho A, Oppenheimer SJ, Davies DP. Meningitis in Hong Kong children, with special reference to the infrequency of haemophilus and meningococcal infection. J Paediatr Child Health. 1997; 33(4): 296-9	Nonfatal Health Outcomes	Other meningitis
Wang YS,Â Shi YM,Â Wu ZY,Â He YX,Â Zhang BZ. Parkinson'sÂ diseaseÂ in China. Coordinational Group of Neuroepidemiology, PLA. Chin Med J (Engl). 1991; 104(11): 960-4	Nonfatal Health Outcomes	Parkinson's disease

Li SC, Schoenberg BS, Wang CC, Cheng XM, Rui DY, Bolis CL, Schoenberg DG. A prevalence survey of Parkinson's disease and other movement disorders in the People's Republic of China. Arch Neurol. 1985; 42(7): 655-7	Nonfatal Health Outcomes	Parkinson's disease
[A mass survey of diabetes mellitus in a population of 300,000 in 14 provinces and municipalities in China]	Nonfatal Health Outcomes	Diabetes mellitus
Pan LX. [Recovery of paragonimiasis in an endemic area in Meixian District, Guangdong Province]. Chin J Epidemiol. 1986; 7(1): 42-44	Nonfatal Health Outcomes	Food-borne trematodiases
Xu Y. Investigation of RBC-G6PD deficiency gene frequency in 9 nationality populations in 7 provinces (autonomous regions). Heredity Dis. 1985; 2: 67-71	Nonfatal Health Outcomes	G6PD deficiency
Baelum V, Wen-Min L, Fejerskov O, Xia C. Tooth mortality and periodontal conditions in 60-80-year-old Chinese. Scand J Dent Res. 1988; 96(2): 99-107	Nonfatal Health Outcomes	Edentulism and severe tooth loss
Ding X, Park YN, Taltavull TC, Thung SN, Jin X, Jin Y, Trung NS, Edamoto Y, Sata T, Abe K. Geographic characterization of hepatitis virus infections, genotyping of hepatitis B virus, and p53 mutation in hepatocellular carcinoma analyzed by in situ detection of viral genomes from carcinoma tissues: comparison among six different countries. Jpn J Infect Dis. 2003; 56(1): 12-8	Nonfatal Health Outcomes	Liver cancer due to hepatitis B
Ding X, Park YN, Taltavull TC, Thung SN, Jin X, Jin Y, Trung NS, Edamoto Y, Sata T, Abe K. Geographic characterization of hepatitis virus infections, genotyping of hepatitis B virus, and p53 mutation in hepatocellular carcinoma analyzed by in situ detection of viral genomes from carcinoma tissues: comparison among six different countries. Jpn J Infect Dis. 2003; 56(1): 12-8	Nonfatal Health Outcomes	Liver cancer due to hepatitis C
World Health Organization (WHO). WHO Global Database on Vitamin A Deficiency. Geneva, Switzerland: World Health Organization (WHO)	Nonfatal Health Outcomes	
Zhao Y, Zhang R, Hu Y, Li R, Liang L, Gang Y. [An epidemiological survey of gallstones with gray-scale ultrasound]. J West Chin Univ Med Sci. 1990; 21(2): 217-20	Nonfatal Health Outcomes	Gallbladder and biliary diseases
Song CC. [A survey of paragonimiasis control in the Lanting People's Commune of Shaoxing County, Zhejiang Province, China]. Chin J Prev Med. 1982; 16(4): 213-15	Nonfatal Health Outcomes	Food-borne trematodiases
Malaria Atlas Project. Malaria Atlas Project Annual Parasite Incidence Database	Nonfatal Health Outcomes	Malaria
Hodgson JE, Shi YF, Gao YL, Wu KJ, Jiang BY, Chen YL. Chlamydial infection in a Chinese gynecologic outpatient clinic. Obstet Gynecol. 1988; 71(1): 96-100	Nonfatal Health Outcomes	Chlamydial infection
Kang M, Lu B, Xie J, Li J, Cai Z-H. Epidemiological survey on alcohol dependence and related problems in Huaiyin City, Jiangshu Province. Chin Ment Health J. 1992; 6(3): 118-119	Nonfatal Health Outcomes	Alcohol use disorders
Tang W-B, Yan P-Q, Ding Z-P, Zhang X, Hou J-Q, Mao X-W. The investigation of prevalence on ADHD among elementary school students in Hexi area in Tianjin Province. Chin J Public Health. 1993; 9(2): 90	Nonfatal Health Outcomes	Attention-deficit/hyperactivity disorder
Shan X, Chen R, Deng J. An epidemiological investigation on epilepsy among children 0-14 age in Jiangsu Province. Acta Univ Med Nanjing. 1992; 12: 170-2	Nonfatal Health Outcomes	Epilepsy
United Nations Children's Fund (UNICEF), World Health Organization (WHO). WHO and UNICEF Reported Disease Incidence Time Series. Geneva, Switzerland: World Health Organization (WHO)	Nonfatal Health Outcomes	Whooping cough
United Nations Population Division. Trends in International Migrant Stock: Migrants by Destination and Origin - 2013 Revision. New York City, United States: United Nations Population Division	Nonfatal Health Outcomes	Chagas disease
Pullan RL, Smith JL, Jasrasaria R, Brooker SJ. Global numbers of infection and disease burden of soil transmitted helminth infections in 2010 [Unpublished data]. Parasit Vectors. 2014; 7(37). [Unpublished data as provided by the Global Burden of Disease 2010 soil transmitted helminths expert group]	Nonfatal Health Outcomes	Intestinal nematode infections
Shyu W-C, Lin S-Z, Chiang M-F, Pang C-Y, Chen S-Y, Hsin Y-L, Thajeb P, Lee Y-J, Li H. Early-onset Parkinson's disease in a Chinese population: 99mTc-TRODAT-1 SPECT, Parkin gene analysis and clinical study. Parkinsonism Relat Disord. 2005; 11(3): 173-80	Nonfatal Health Outcomes	Parkinson's disease
Wang SJ, Fuh JL, Liu CY, Lin KP, Chang R, Yih JS, Chou P, Lin KN, Teng EL, Larson EB. Parkinson's disease in Kin-Hu, Kinmen: a community survey by neurologists. Neuroepidemiology. 1994; 13(1-2): 69-74	Nonfatal Health Outcomes	Parkinson's disease
Zhang Z-X, Roman GC, Hong Z, Wu C-B, Qu Q-M, Huang J-B, Zhou B, Geng Z-P, Wu J-X, Wen H-B, Zhao H, Zahner GEP. Parkinson's disease in China: prevalence in Beijing, Xian, and Shanghai. Lancet. 2005; 365(9459): 595-7	Nonfatal Health Outcomes	Parkinson's disease
Wang SJ, Fuh JL, Teng EL, Liu CY, Lin KP, Chen HM, Lin CH, Wang PN, Ting YC, Wang HC, Lin KN, Chou P, Larson EB, Liu HC. A door-to-door survey of Parkinson's disease in a Chinese population in Kinmen. Arch Neurol. 1996; 53(1): 66-71	Nonfatal Health Outcomes	Parkinson's disease
Ding X, Gao J, Xie C, Xiong B, Wu S, Cen Z, Lou Y, Lou D, Xie F, Luo W. Prevalence and clinical correlation of dysphagia in Parkinson disease: a study on Chinese patients. Eur J Clin Nutr. 2017	Nonfatal Health Outcomes	Parkinson's disease

Mao ZJ, Liu CC, Ji SQ, Yang QM, Ye HX, Han HY, Xue Z. Clinical characteristics of sleep disorders in patients with Parkinson's disease. J Huazhong Univ Sci Technolog Med Sci. 2017; 37(1): 100-104	Nonfatal Health Outcomes	Parkinson's disease
Cheng Q, Wang D-S, Jiang G-X, Han H, Zhang Y, Wang W-Z, Fredrikson S. Distinct pattern of age-specific incidence of Guillain-BarrÃ© syndrome in Harbin, China. J Neurol. 2002; 249(1): 25-32	Nonfatal Health Outcomes	Guillain-BarrÃ© syndrome
Zhang Y, Wang D, Han H, Li F, Sheng L, Link H. Epidemiological survey of the incidence of Guillain-Barre syndrome in Harbin from 1997 to 1999. Chin J Clin Rehab. 2004; 34: 7812-5	Nonfatal Health Outcomes	Guillain-BarrÃ© syndrome
Xu Z, Shen FM, Xu ZY, Huang QS. HCV infection and primary liver cell cancer. Tumor. 1990; 10: 115	Nonfatal Health Outcomes	Liver cancer due to hepatitis B
Xu Z, Shen FM, Xu ZY, Huang QS. HCV infection and primary liver cell cancer. Tumor. 1990; 10: 115	Nonfatal Health Outcomes	Liver cancer due to hepatitis C
Malaria Atlas Project. Malaria Atlas Project Plasmodium Falciparum Parasite Rate Database. Oxford, United Kingdom: Malaria Atlas Project	Nonfatal Health Outcomes	Malaria
Wang CQ. [Epidemiological study of paragonimiasis in Xingshan County, Hubei Province]. Chin J Epidemiol. 1986; 7(1): 45-7	Nonfatal Health Outcomes	Food-borne trematodiases
Xu DZ. [Epidemiologic study of viral hepatitis A in kindergartens and nurseries]. Nat Med J Chin. 1985; 65(3): 148-50	Nonfatal Health Outcomes	Acute hepatitis A
Guo-Chuan Zhang, Bao-Long Z, Suzhen X. [A survey of prevalence of senile dementia and Parkinson disease]. J Clin Psychol Med. 2001; 11(Pt 3): 143-5	Nonfatal Health Outcomes	Parkinson's disease
Chen P, Yu ES, Zhang M, Liu WT, Hill R, Katzman R. ADL dependence and medical conditions in Chinese older persons: a population-based survey in Shanghai, China. J Am Geriatr Soc. 1995; 43(4): 378-83	Nonfatal Health Outcomes	Parkinson's disease
Shi YM. [Study on the prevalence of Parkinson's disease in Hongkou District, Shanghai]. Chin J Epidemiol. 1987; 8(4): 205-7	Nonfatal Health Outcomes	Parkinson's disease
Li G, Shen YC, Chen CH, Zhao YW, Li SR, Lu M. An epidemiological survey of age-related dementia in an urban area of Beijing. Acta Psychiatr Scand. 1989; 79(6): 557-63	Nonfatal Health Outcomes	Alzheimer's disease and other dementias
Hodgson JE, Shi YF, Gao YL, Wu KJ, Jiang BY, Chen YL. Chlamydial infection in a Chinese gynecologic outpatient clinic. Obstet Gynecol. 1988; 71(1): 96-100	Nonfatal Health Outcomes	Sexually transmitted infections excluding HIV
Pilot T, Lu ZY, Lin ZQ, Yen WP, Cao GR. Periodontal conditions in 35-44-year-old factory workers in Shanghai. Community Dent Oral Epidemiol. 1989; 17(4): 216	Nonfatal Health Outcomes	Periodontal diseases
Lai HC, Lai MP, Leung KS. Glucose-6-phosphate dehydrogenase deficiency in Chinese. J Clin Pathol. 1968; 21(1): 44-7	Nonfatal Health Outcomes	G6PD deficiency
Yue PC, Strickland M. Glucose-6-phosphate-dehydrogenase deficiency and neonatal jaundice in Chinese male infants in Hong Kong. Lancet. 1965; 1(7381): 350-1	Nonfatal Health Outcomes	G6PD deficiency
Fung RH, Keung YK, Chung GS. Screening of pyruvate kinase deficiency and G6PD deficiency in Chinese newborn in Hong Kong. Arch Dis Child. 1969; 44(235): 373-6	Nonfatal Health Outcomes	G6PD deficiency
Baelum V, Luan WM, Chen X, Fejerskov O. Predictors of tooth loss over 10 years in adult and elderly Chinese. Community Dent Oral Epidemiol. 1997; 25(3): 204-10	Nonfatal Health Outcomes	Edentulism and severe tooth loss
Yuan J-M, Ross RK, Stanczyk FZ, Govindarajan S, Gao Y-T, Henderson BE, Yu MC. A cohort study of serum testosterone and hepatocellular carcinoma in Shanghai, China . Int J Cancer. 1995; 63(4): 491-3	Nonfatal Health Outcomes	Liver cancer due to hepatitis B
Yuan J-M, Ross RK, Stanczyk FZ, Govindarajan S, Gao Y-T, Henderson BE, Yu MC. A cohort study of serum testosterone and hepatocellular carcinoma in Shanghai, China . Int J Cancer. 1995; 63(4): 491-3	Nonfatal Health Outcomes	Liver cancer due to hepatitis C
Zhang ZQ, Huang TR, He ZF, Yu JH, Xu QF, Huang ZD. The relationship between HCV and HBV in the etiology of primary liver cancer. Guangxi Med J. 1993; 15: 171-4	Nonfatal Health Outcomes	Liver cancer due to hepatitis C
Sung RYT, Cheng AFB, Chan RCK, Tam JS, Oppenheimer SJ. Epidemiology and Etiology of Pneumonia in Children in Hong Kong. Clin Infect Dis. 1993; 17(5): 894-6	Nonfatal Health Outcomes	Lower respiratory infections
Sung RY, Senok AC, Ho A, Oppenheimer SJ, Davies DP. Meningitis in Hong Kong children, with special reference to the infrequency of haemophilus and meningococcal infection. J Paediatr Child Health. 1997; 33(4): 296-9	Nonfatal Health Outcomes	Hearing loss
Sung RY, Senok AC, Ho A, Oppenheimer SJ, Davies DP. Meningitis in Hong Kong children, with special reference to the infrequency of haemophilus and meningococcal infection. J Paediatr Child Health. 1997; 33(4): 296-9	Nonfatal Health Outcomes	Vision loss
Ministry of Public Health (China). China National Nutrition Survey 1992 - China CDC	Nonfatal Health Outcomes	Dietary iron deficiency
Ministry of Public Health (China). China National Nutrition Survey 1992 - China CDC	Nonfatal Health Outcomes	Anemia
Xia GL, Liu CB, Caoa HL, BP SL, Zhan MY, Sub CA, Nan JH, Qi XQ. Prevalence of hepatitis B and C virus infections in the general Chinese population: Results from a nationwide cross-sectional seroepidemiologic study of hepatitis A, B, C, D, and E virus infections in China, 1992. Int Hepatol Commun. 1996; 5: 62-73	Nonfatal Health Outcomes	Acute hepatitis B

Xia GL, Liu CB, Caoa HL, BP SL, Zhan MY, Sub CA, Nan JH, Qi XQ. Prevalence of hepatitis B and C virus infections in the general Chinese population: Results from a nationwide cross-sectional seroepidemiologic study of hepatitis A, B, C, D, and E virus infections in China, 1992. Int Hepatol Commun. 1996; 5: 62-73	Nonfatal Health Outcomes	Acute hepatitis C
Hui AC, Ng K, Tong P, Mok V, Chow K, Wu A, Wong LK. Bacterial meningitis in Hong Kong: 10-years experience. Clin Neurol Neurosurg. 2005; 107(5): 366-70	Nonfatal Health Outcomes	Meningitis
Hui AC, Ng K, Tong P, Mok V, Chow K, Wu A, Wong LK. Bacterial meningitis in Hong Kong: 10-years experience. Clin Neurol Neurosurg. 2005; 107(5): 366-70	Nonfatal Health Outcomes	Pneumococcal meningitis
Hui AC, Ng K, Tong P, Mok V, Chow K, Wu A, Wong LK. Bacterial meningitis in Hong Kong: 10-years experience. Clin Neurol Neurosurg. 2005; 107(5): 366-70	Nonfatal Health Outcomes	H influenzae type B meningitis
Hui AC, Ng K, Tong P, Mok V, Chow K, Wu A, Wong LK. Bacterial meningitis in Hong Kong: 10-years experience. Clin Neurol Neurosurg. 2005; 107(5): 366-70	Nonfatal Health Outcomes	Meningococcal meningitis
Hui AC, Ng K, Tong P, Mok V, Chow K, Wu A, Wong LK. Bacterial meningitis in Hong Kong: 10-years experience. Clin Neurol Neurosurg. 2005; 107(5): 366-70	Nonfatal Health Outcomes	Other meningitis
Wang Q, Chen R, Du L, Zeng Q. An epidemiological and clinical study of primary gout. Chin J Intern Med. 2001; 40(5): 313-5	Nonfatal Health Outcomes	Gout
World Health Organization (WHO). Progress towards eliminating leprosy as a public health problem. Part I. Wkly Epidemiol Rec. 1994: 69(20): 145-51.	Nonfatal Health Outcomes	Leprosy
University of Hong Kong. Hong Kong Drug Market 2000	Nonfatal Health Outcomes	Cocaine use disorders
Xu L, Jiang Z, Yu S, Xu S, Huang D, Yang S, Zhao G, Gan Y, Kang Q, Yu D. [Nationwide survey of the distribution of human parasites in China--infection with parasite species in human population]. Chin J Parasitol Parasit Dis. 1995; 13(1): 1-7	Nonfatal Health Outcomes	Food-borne trematodiases
Lau EM, Egger P, Coggon D, Cooper C, Valenti L, O'Connell D. Low back pain in Hong Kong: prevalence and characteristics compared with Britain. J Epidemiol Community Health. 1995; 49(5): 492-4	Nonfatal Health Outcomes	Low back pain
Woo J, Ho SC, Lau J, Leung PC. Musculoskeletal complaints and associated consequences in elderly Chinese aged 70 years and over. J Rheumatol. 1994; 21(10): 1927-31	Nonfatal Health Outcomes	Low back pain
Lau EM, Sham A, Wong KC. The prevalence of and risk factors for neck pain in Hong Kong Chinese. J Public Health Med. 1996; 18(4): 396-9	Nonfatal Health Outcomes	Neck pain
Holmgren CJ, Corbet EF, Lim LP. Periodontal conditions among the middle-aged and the elderly in Hong Kong. Community Dent Oral Epidemiol. 1994; 22(5 Pt 2): 396-402	Nonfatal Health Outcomes	Periodontal diseases
Sanderson JE, Chan SKW, Chan WWM, Hung YT, Woo KS. The aetiology of heart failure in the Chinese population of Hong Kong - a prospective study of 730 consecutive patients. Int J Cardiol. 1995; 51(1): 29-35	Nonfatal Health Outcomes	Heart failure
Sanderson JE, Chan SKW, Chan WWM, Hung YT, Woo KS. The aetiology of heart failure in the Chinese population of Hong Kong - a prospective study of 730 consecutive patients. Int J Cardiol. 1995; 51(1): 29-35	Nonfatal Health Outcomes	Coal workers pneumoconiosis
Franceschi S, Smith JS, van den Brule A, Herrero R, Arslan A, Anh P-T-H, Bosch FX, Hieu N-T, Matos E, Posso H, Qiao Y-L, Shin H-R, Sukvirach S, Thomas JO, Snijders PJF, MuÃ±oz N, Meijer CJLM. Cervical infection with Chlamydia trachomatis and Neisseria gonorrhoeae in women from ten areas in four continents. A cross-sectional study. Sex Transm Dis. 2007; 34(8): 563-9	Nonfatal Health Outcomes	Chlamydial infection
Franceschi S, Smith JS, van den Brule A, Herrero R, Arslan A, Anh P-T-H, Bosch FX, Hieu N-T, Matos E, Posso H, Qiao Y-L, Shin H-R, Sukvirach S, Thomas JO, Snijders PJF, MuÃ±oz N, Meijer CJLM. Cervical infection with Chlamydia trachomatis and Neisseria gonorrhoeae in women from ten areas in four continents. A cross-sectional study. Sex Transm Dis. 2007; 34(8): 563-9	Nonfatal Health Outcomes	Gonococcal infection
Sanderson JE, Chan SKW, Chan WWM, Hung YT, Woo KS. The aetiology of heart failure in the Chinese population of Hong Kong - a prospective study of 730 consecutive patients. Int J Cardiol. 1995; 51(1): 29-35	Nonfatal Health Outcomes	Alcoholic cardiomyopathy
Sanderson JE, Chan SKW, Chan WWM, Hung YT, Woo KS. The aetiology of heart failure in the Chinese population of Hong Kong - a prospective study of 730 consecutive patients. Int J Cardiol. 1995; 51(1): 29-35	Nonfatal Health Outcomes	Myocarditis
Sanderson JE, Chan SKW, Chan WWM, Hung YT, Woo KS. The aetiology of heart failure in the Chinese population of Hong Kong - a prospective study of 730 consecutive patients. Int J Cardiol. 1995; 51(1): 29-35	Nonfatal Health Outcomes	Other cardiomyopathy
Zhou Y. Seasonal analysis of the morbidity of pneumonia in children under 5 years of age in Guangzhou area. Chin J Child Health Care. 2000; 8: 35Â–6	Nonfatal Health Outcomes	Lower respiratory infections
Chinese Center for Disease Control and Prevention (CCDC). China Disease Surveillance Points 1991 - China CDC	Nonfatal Health Outcomes	Rabies
Chinese Center for Disease Control and Prevention (CCDC). China Disease Surveillance Points 1992 - China CDC	Nonfatal Health Outcomes	Rabies
Chinese Center for Disease Control and Prevention (CCDC). China Disease Surveillance Points 1993 - China CDC	Nonfatal Health Outcomes	Rabies

Chinese Center for Disease Control and Prevention (CCDC). China Disease Surveillance Points 1994 - China CDC	Nonfatal Health Outcomes	Rabies
Douglass JM, Wei Y, Zhang BX, Tinanoff N. Caries prevalence and patterns in 3-6-year-old Beijing children. Community Dent Oral Epidemiol. 1995; 23(6): 340-3	Nonfatal Health Outcomes	Caries of deciduous teeth
Sanderson JE, Chan SKW, Chan WWM, Hung YT, Woo KS. The aetiology of heart failure in the Chinese population of Hong Kong - a prospective study of 730 consecutive patients. Int J Cardiol. 1995; 51(1): 29-35	Nonfatal Health Outcomes	Silicosis
World Health Organization (WHO). WHO Mortality Database Version November 2015. Geneva, Switzerland: World Health Organization (WHO), 2015	Nonfatal Health Outcomes	HIV/AIDS resulting in other diseases
Lok N-S, Lau C-P. Prevalence of palpitations, cardiac arrhythmias and their associated risk factors in ambulant elderly. Int J Cardiol. 1996; 54(3): 231-6	Nonfatal Health Outcomes	Atrial fibrillation and flutter
Jacobs EG, Leung MP, Karlberg J. Distribution of symptomatic congenital heart disease in Hong Kong. Pediatr Cardiol. 2000; 21(2): 148-57	Nonfatal Health Outcomes	Congenital birth defects
Ran M-S, Xiang M-Z, Li S-X, Shan Y-H, Huang M-S, Li S-G, Liu Z-R, Chen EY-H, Chan CL-W. Prevalence and course of schizophrenia in a Chinese rural area. Aust N Z J Psychiatry. 2003; 37(4): 452-7	Nonfatal Health Outcomes	Schizophrenia
Sloan N, Durocher J, Aldrich T, Blum J, Winikoff B. What measured blood loss tells us about postpartum bleeding: a systematic review. BJOG. 2010; 117(7): 788-800	Nonfatal Health Outcomes	Maternal disorders
Wagner KS, Ronsmans C, Thomas SL, Calvert C, Adler A, Ganaba R, Goufodji S, Filippi V. Women who experience obstetric haemorrhage are at higher risk of anaemia, in both rich and poor countries. Trop Med Int Health. 2012; 17(1): 9-22	Nonfatal Health Outcomes	Dietary iron deficiency
Wagner KS, Ronsmans C, Thomas SL, Calvert C, Adler A, Ganaba R, Goufodji S, Filippi V. Women who experience obstetric haemorrhage are at higher risk of anaemia, in both rich and poor countries. Trop Med Int Health. 2012; 17(1): 9-22	Nonfatal Health Outcomes	Anemia
World Health Organization (WHO). Progress towards the elimination of leprosy as a public health problem. Part I. Wkly Epidemiol Rec. 1995: 70(25): 177-82.	Nonfatal Health Outcomes	Leprosy
Hu G-H, Jia H, Song K-Y, Lin D-D, Ju Z, Cao C-L, Jing X, Dong L, Jiang W-S. The role of health education and health promotion in the control of schistosomiasis: Experiences from a 12-year intervention study in the Poyang Lake area. Addict Behav Rep. 2005; 96(2-3): 232Â–41	Nonfatal Health Outcomes	Schistosomiasis
Wang Y, Tao QM, Zhao HY, Tsuda F, Nagayama R, Yamamoto K, Tanaka T, Tokita H, Okamoto H, Miyakawa Y. Hepatitis C virus RNA and antibodies among blood donors in Beijing. J Hepatol. 1994; 21(4): 634-40	Nonfatal Health Outcomes	Acute hepatitis B
Zhuo J, Tao G, Ebrahim SH, Wang S, Luo Z, Wang H. The relationship of hepatitis B virus infection between adults and their children in Guangxi Province, China. J Hepatol. 2000; 33(4): 628-31	Nonfatal Health Outcomes	Acute hepatitis B
Wong TW, Wong KS, Yu TS, Kay R. Prevalence of migraine and other headaches in Hong Kong. Neuroepidemiology. 1995; 14(2): 82-91	Nonfatal Health Outcomes	Migraine
Wong TW, Wong KS, Yu TS, Kay R. Prevalence of migraine and other headaches in Hong Kong. Neuroepidemiology. 1995; 14(2): 82-91	Nonfatal Health Outcomes	Tension-type headache
Yuen PM, Chan NS, Yim SF, Chang AM. A randomised double blind comparison of Syntometrine and Syntocinon in the management of the third stage of labour. Br J Obstet Gynaecol. 1995; 102(5): 377-80	Nonfatal Health Outcomes	Maternal disorders
Sanderson JE, Chan SKW, Chan WWM, Hung YT, Woo KS. The aetiology of heart failure in the Chinese population of Hong Kong - a prospective study of 730 consecutive patients. Int J Cardiol. 1995; 51(1): 29-35	Nonfatal Health Outcomes	Rheumatic heart disease
Fan XL, Peng WW, Yao JL, Zhou YP, Lu L, Zhen XC, Chen Q. Infection of HBV, HCV and HDV in Hepatocellular Carcinoma. Chin J Infect Dis. 1995; 13: 129-32	Nonfatal Health Outcomes	Liver cancer due to hepatitis B
Fan XL, Zhou YP, Peng WW, Yao JL, Li GH, Guo RP. A case control study on relationship between hepatocellular carcinoma and infection of HCV and HBV in Guangzhou. Chin J Cancer. 1995; 14: 328-30	Nonfatal Health Outcomes	Liver cancer due to hepatitis B
Wang ZJ, Zhou YP, Cheng B, Liang ZN, Peng WW. An epidemiologic study on the aetiological factors of primary liver cancer in Shunde City of Guangdong province. Chin J Epidemiol. 1996; 17(3): 141-4	Nonfatal Health Outcomes	Liver cancer due to hepatitis B
Fan XL, Peng WW, Yao JL, Zhou YP, Lu L, Zhen XC, Chen Q. Infection of HBV, HCV and HDV in Hepatocellular Carcinoma. Chin J Infect Dis. 1995; 13: 129-32	Nonfatal Health Outcomes	Liver cancer due to hepatitis C
Fan XL, Zhou YP, Peng WW, Yao JL, Li GH, Guo RP. A case control study on relationship between hepatocellular carcinoma and infection of HCV and HBV in Guangzhou. Chin J Cancer. 1995; 14: 328-30	Nonfatal Health Outcomes	Liver cancer due to hepatitis C
Wang ZJ, Zhou YP, Cheng B, Liang ZN, Peng WW. An epidemiologic study on the aetiological factors of primary liver cancer in Shunde City of Guangdong province. Chin J Epidemiol. 1996; 17(3): 141-4	Nonfatal Health Outcomes	Liver cancer due to hepatitis C
Gao CL, Cao WC, Chen XX. Changes in anti-filarial antibody after control of filariasis in Shandong Province. Chin Med J (Engl). 1994; 107(5): 360-3	Nonfatal Health Outcomes	Lymphatic filariasis
Chen C, Shen Y, Zhang W. Epidemiological survey on schizophrenia in 7 areas of China. Chin J Psychiatry. 1998; 31(2): 72-4	Nonfatal Health Outcomes	Schizophrenia

Okuno H, Xie Z-C, Lu B-Y, Qin X, Takasu M, Kano H, Shiozaki Y, Inoue K. A low prevalence of anti-hepatitis C virus antibody in patients with hepatocellular carcinoma in guangxi province, southern china . Cancer. 1994; 73(1): 58-62	Nonfatal Health Outcomes	Liver cancer due to hepatitis B
Okuno H, Xie Z-C, Lu B-Y, Qin X, Takasu M, Kano H, Shiozaki Y, Inoue K. A low prevalence of anti-hepatitis C virus antibody in patients with hepatocellular carcinoma in guangxi province, southern china . Cancer. 1994; 73(1): 58-62	Nonfatal Health Outcomes	Liver cancer due to hepatitis C
Li Z, Ye R, Zhang L, Li H, Liu J, Ren A. Folic acid supplementation during early pregnancy and the risk of gestational hypertension and preeclampsia. Hypertension. 2013; 61(4): 873-9	Nonfatal Health Outcomes	Maternal hypertensive disorders
Ning X, Sun J, Jiang R, Lu H, Bai L, Shi M, Tu J, Wu Y, Wang J, Zhang J. Increased Stroke Burdens Among the Low-Income Young and Middle Aged in Rural China. Stroke. 2017; 48(1): 77-83	Nonfatal Health Outcomes	Ischemic stroke
Ning X, Sun J, Jiang R, Lu H, Bai L, Shi M, Tu J, Wu Y, Wang J, Zhang J. Increased Stroke Burdens Among the Low-Income Young and Middle Aged in Rural China. Stroke. 2017; 48(1): 77-83	Nonfatal Health Outcomes	Intracerebral hemorrhage
Yuen PM, Chan NS, Yim SF, Chang AM. A randomised double blind comparison of Syntometrine and Syntocinon in the management of the third stage of labour. Br J Obstet Gynaecol. 1995; 102(5): 377-80	Nonfatal Health Outcomes	Maternal hemorrhage
Dong B-Q, Yang J, Wang X-Y, Gong J, von Seidlein L, Wang M-L, Lin M, Liao H-Z, Ochiai RL, Xu Z-Y, Jodar L, Clemens JD. Trends and disease burden of enteric fever in Guangxi province, China, 1994-2004. Bull World Health Organ. 2010; 88(9): 689-96	Nonfatal Health Outcomes	Intestinal infectious diseases
Jacobs EG, Leung MP, Karlberg J. Distribution of symptomatic congenital heart disease in Hong Kong. Pediatr Cardiol. 2000; 21(2): 148-57	Nonfatal Health Outcomes	Congenital heart anomalies
Jin L, Ye R, Zheng J, Hong S, Ren A. Secular trends of hypospadias prevalence and factors associated with it in southeast China during 1993-2005. Birth Defects Res A Clin Mol Teratol. 2010; 88(6): 458-65	Nonfatal Health Outcomes	Congenital birth defects
Booth M, Li Y, Tanner M. Helminth infections, morbidity indicators and schistosomiasis treatment history in three villages, Dongting Lake region, PR China. Trop Med Int Health. 1996; 1(4): 464Â–74	Nonfatal Health Outcomes	Schistosomiasis
Li Y, Ross A, Yu D, Li Y, Williams G, McManus D. An evaluation of Schistosoma japonicum infections in three villages in the Dongting lake region of China .1. Prevalence, intensity and morbidity before the implementation of adequate control strategies. Addict Behav Rep. 1997; 68(1): 77Â–91	Nonfatal Health Outcomes	Schistosomiasis
Li G, Wang C, Li J, Ai Y, Zhang Y. Dental caries in Yanan schoolchildren aged 12 years: China. Community Dent Oral Epidemiol. 1994; 22(3): 208	Nonfatal Health Outcomes	Caries of permanent teeth
Zhang L, Yang J, Hong Z, Yuan G, Zhou B, Zhao L, Huang Y, Chen J, Wu Y. Proportion of different subtypes of stroke in China. Stroke. 2003; 34(9): 2091-6	Nonfatal Health Outcomes	Subarachnoid hemorrhage
Sanderson JE, Chan SKW, Chan WWM, Hung YT, Woo KS. The aetiology of heart failure in the Chinese population of Hong Kong - a prospective study of 730 consecutive patients. Int J Cardiol. 1995; 51(1): 29-35	Nonfatal Health Outcomes	Other pneumoconiosis
Yan C, Zhang R-L, Wu M-X. Epidemiology of epilepsy in rural Panzhihua City. Sichuan Med J. 1999	Nonfatal Health Outcomes	Epilepsy
Jiang B, Wang W, Chen H, Hong Z, Yang Q, Wu S. Incidence and Trends of Stroke and Its Subtypes in China: Results From Three Large Cities. Stroke. 2006; 37(1): 63-5	Nonfatal Health Outcomes	Ischemic stroke
Jiang B, Wang W, Chen H, Hong Z, Yang Q, Wu S. Incidence and Trends of Stroke and Its Subtypes in China: Results From Three Large Cities. Stroke. 2006; 37(1): 63-5	Nonfatal Health Outcomes	Intracerebral hemorrhage
Li L, He J, Zhao L. [Epidemiologic features of viral hepatitis in Fujian]. Chin J Epidemiol. 1998; 19(2): 89-92	Nonfatal Health Outcomes	Acute hepatitis A
Jiang B, Wang W, Chen H, Hong Z, Yang Q, Wu S. Incidence and Trends of Stroke and Its Subtypes in China: Results From Three Large Cities. Stroke. 2006; 37(1): 63-5	Nonfatal Health Outcomes	Subarachnoid hemorrhage
Li L, He J, Zhao L. [Epidemiologic features of viral hepatitis in Fujian]. Chin J Epidemiol. 1998; 19(2): 89-92	Nonfatal Health Outcomes	Acute hepatitis E
Nokes C, McGarvey ST, Shiue L, Wu G, Wu H, Bundy DA, Olds GR. Evidence for an improvement in cognitive function following treatment of Schistosoma japonicum infection in Chinese primary schoolchildren. Am J Trop Med Hyg. 1999; 60(4): 556-65	Nonfatal Health Outcomes	Schistosomiasis
Williams H, Stewart A, Von Mutius E, Cookson W, Anderson HR. Is eczema really on the increase worldwide. J Allergy Clin Immunol. 2008; 121(4): 947-954	Nonfatal Health Outcomes	Dermatitis
Hou G-M, Wang S-R. An epidemiological study of mental disorders in Wuxi city of Jiangsu Province. J Clin Psychiatry. 1996; 6(4): 206-208	Nonfatal Health Outcomes	Schizophrenia
Petersen PE, Guang LX. Dental caries prevalence in a group of schoolchildren in Wuhan City, PR China, 1993. Community Dent Oral Epidemiol. 1994; 22(6): 465-6	Nonfatal Health Outcomes	Caries of permanent teeth

World Health Organization (WHO). WHO Mortality Database Version November 2015. Geneva, Switzerland: World Health Organization (WHO), 2015	Nonfatal Health Outcomes	HIV/AIDS
Li N, Li Z, Ye R, Zhu Y, Li S, Yang N, Zhang L, Li H, Liu J, Ren A. Preconception blood pressure and risk of preterm birth: a large cohort study in China. J Hypertens. 2016; 34(11): 2243-7	Nonfatal Health Outcomes	Neonatal preterm birth
Wang Y-H, Zhu Z-H, Li Z-B. An epidemiological research of alcohol dependence and its related factors. 1994; 3(1): 22-29	Nonfatal Health Outcomes	Alcohol use disorders
Li B, Lou Y, Gu H, Long X, Wang T, Wei J, Wang J, Tu J, Ning X. Trends in Incidence of Stroke and Transition of Stroke Subtypes in Rural Tianjin China: A Population-Based Study from 1992 to 2012. PLoS One. 2015; 10(10): e0139461	Nonfatal Health Outcomes	Ischemic stroke
Li B, Lou Y, Gu H, Long X, Wang T, Wei J, Wang J, Tu J, Ning X. Trends in Incidence of Stroke and Transition of Stroke Subtypes in Rural Tianjin China: A Population-Based Study from 1992 to 2012. PLoS One. 2015; 10(10): e0139461	Nonfatal Health Outcomes	Intracerebral hemorrhage
Sanderson JE, Chan SKW, Chan WWM, Hung YT, Woo KS. The aetiology of heart failure in the Chinese population of Hong Kong - a prospective study of 730 consecutive patients. Int J Cardiol. 1995; 51(1): 29-35	Nonfatal Health Outcomes	Ischemic heart disease
Sanderson JE, Chan SKW, Chan WWM, Hung YT, Woo KS. The aetiology of heart failure in the Chinese population of Hong Kong - a prospective study of 730 consecutive patients. Int J Cardiol. 1995; 51(1): 29-35	Nonfatal Health Outcomes	Asbestosis
National Central Cancer Registry (China). China Cancer Registry Incidence and Mortality 1990-2009	Nonfatal Health Outcomes	Kidney cancer
Sloan N, Durocher J, Aldrich T, Blum J, Winikoff B. What measured blood loss tells us about postpartum bleeding: a systematic review. BJOG. 2010; 117(7): 788-800	Nonfatal Health Outcomes	Maternal abortion, miscarriage, and ectopic pregnancy
Chen X, Zhang M, Huang D, Huang M, Xiong Y, Xie M, Shou Y, Li M, Wu C, Zeng H, Li X, Zheng M. An epidemiologic investigation of acute rheumatic fever and rheumatic heart disease among students aged 5-18 in west area of Sichuan Province. J Sichuan Univ Med Sci Ed. 2003; 34(3): 533-5	Nonfatal Health Outcomes	Rheumatic heart disease
Zhang D, Wang L,Cai Y. A survey of drug abuse between 3-year interval in the epidemic area of Anshun, Guizhou Province. Chin J Psychiatry. 1999; 32(1): 47-49	Nonfatal Health Outcomes	Opioid use disorders
Sanderson JE, Chan SKW, Chan WWM, Hung YT, Woo KS. The aetiology of heart failure in the Chinese population of Hong Kong - a prospective study of 730 consecutive patients. Int J Cardiol. 1995; 51(1): 29-35	Nonfatal Health Outcomes	Chronic obstructive pulmonary disease
Sanderson JE, Chan SKW, Chan WWM, Hung YT, Woo KS. The aetiology of heart failure in the Chinese population of Hong Kong - a prospective study of 730 consecutive patients. Int J Cardiol. 1995; 51(1): 29-35	Nonfatal Health Outcomes	Interstitial lung disease and pulmonary sarcoidosis
Wong Z, Zhang J-X, Ma D-D, Ma S-P, Li X-F, Jiang Y-Q, Xu L-Y, Chen D-C, Cao X-Y, Meng G-Y, He J-Y, Sun L-M, Zhang S-J, Zhu B-J, Cui W-C, Gong P-J, Hu B-W, Liu Z-X, Mo Z-P, Li X-Q. An epidemiological investigation of mental disorders in Shandong province in 1984 and 1994. Chin J Psychiatry. 1998; 31(4): 222-224	Nonfatal Health Outcomes	Schizophrenia
Worldwide variations in the prevalence of asthma symptoms: the International Study of Asthma and Allergies in Childhood (ISAAC). Eur Respir J. 1998; 12(2): 315-35	Nonfatal Health Outcomes	Asthma
Zhang W-X, Shen Y-C, Li S-R, Chen C-H, Huang Y-Q, Wang J-R, Wang D-P, Tu J, Ning Z-X, Fu L-M, Ji L-P, Liu Z-G, Wu H-M, Luo K-L, Zhai S-T, Yan H-J, Meng G-R. Epidemiological investigation on mental disorders in 7 areas of China. Chin J Psychiatry. 1998; 31(2): 69-71	Nonfatal Health Outcomes	Alcohol use disorders
Hao W, Young Derson, Xiao S-Y, Li L-J, Zhang Y-L. Alcohol consumption and alcohol-related problems: Chinese experience from six area samples, 1994. Addiction. 1999; 94(10): 1467-1476	Nonfatal Health Outcomes	Alcohol use disorders
Pan X, Yang W, Liu J. Prevalence of diabetes and its risk factors in China 1994. National Diabetes Prevention and Control Cooperative Group. Chin J Intern Med. 1997; 36(6): 384-9	Nonfatal Health Outcomes	Diabetes mellitus
Wang CX, Feng XH, Yyuan RZ, Li DS, Wang HJ, Cheng SQ. A study of the relationship in HBV, HCV infection and primary hepatocellular carcinoma. Chin J Health Lab Tech. 1996; 6: 184-5	Nonfatal Health Outcomes	Liver cancer due to hepatitis C
Chang AM, Holroyd E, Chau JP. Premenstrual syndrome in employed Chinese women in Hong Kong. Health Care Women Int. 1995; 16(6): 551-61	Nonfatal Health Outcomes	Premenstrual syndrome
Jiang W, Yu G, Liu P, Geng Q, Chen L, Lin Q, Ren X, Ye W, He Y, Guo Y, Duan S, Wen J, Li H, Qi Y, Jiang C, Zheng Y, Liu C, Si E, Zhang Q, Tian Q, Du C. Structure and function of glucose-6-phosphate dehydrogenase-deficient variants in Chinese population. Hum Genet. 2006; 119(5): 463-78	Nonfatal Health Outcomes	G6PD deficiency
Tang J, Yu Y, Ma Y. The epidemic tendency of dental caries prevalence of school students from 1991 to 2005 in China. J Huazhong Univ Sci Technolog Med Sci. 2010; 30(1): 132-7	Nonfatal Health Outcomes	Caries of deciduous teeth

Tang J, Yu Y, Ma Y. The epidemic tendency of dental caries prevalence of school students from 1991 to 2005 in China. J Huazhong Univ Sci Technolog Med Sci. 2010; 30(1): 132-7	Nonfatal Health Outcomes	Caries of permanent teeth
Chen G, Lin W, Shen F, Iloeje UH, London WT, Evans AA. Chronic hepatitis B virus infection and mortality from non-liver causes: results from the Haimen City cohort study. Int J Epidemiol. 2005; 34(1): 132-7	Nonfatal Health Outcomes	Acute hepatitis B
Ito S, Yao DF, Nii C, Hibino S, Kamamura M, Nisikado T, Honda H, Shimizu I, Meng XY. Epidemiological characteristics of the incidence of hepatitis C virus (C100-3) antibodies in patients with liver diseases in the inshore area of the Yangtze River. J Gastroenterol Hepatol. 1993; 8(3): 232-7	Nonfatal Health Outcomes	Acute hepatitis B
Wang SJ, Liu HC, Fuh JL, Liu CY, Lin KP, Chen HM, Lin CH, Wang PN, Hsu LC, Wang HC, Lin KN. Prevalence of headaches in a Chinese elderly population in Kinmen: age and gender effect and cross-cultural comparisons. Neurology. 1997; 49(1): 195-200	Nonfatal Health Outcomes	Migraine
Wang SJ, Liu HC, Fuh JL, Liu CY, Lin KP, Chen HM, Lin CH, Wang PN, Hsu LC, Wang HC, Lin KN. Prevalence of headaches in a Chinese elderly population in Kinmen: age and gender effect and cross-cultural comparisons. Neurology. 1997; 49(1): 195-200	Nonfatal Health Outcomes	Tension-type headache
Williams H, Stewart A, Von Mutius E, Cookson W, Anderson HR. Is eczema really on the increase worldwide. J Allergy Clin Immunol. 2008; 121(4): 947-954	Nonfatal Health Outcomes	Atopic dermatitis
Zhou YP, Peng WW, Yao JL, Lu L, Li GH, Huang JF, Peng BG, Chen Q. A case control study on relationship between hepatocellular carcinoma and infection of HCV and HCV. Acad J SUMS. 1994; 15: 45-49	Nonfatal Health Outcomes	Liver cancer due to hepatitis B
Zhou YP, Peng WW, Yao JL, Lu L, Li GH, Huang JF, Peng BG, Chen Q. A case control study on relationship between hepatocellular carcinoma and infection of HCV and HCV. Acad J SUMS. 1994; 15: 45-49	Nonfatal Health Outcomes	Liver cancer due to hepatitis C
Zhang J, Liu G, Arima H, Li Y, Cheng G, Shiue I, Lv L, Wang H, Zhang C, Zhao J, Anderson C. Incidence and risks of subarachnoid hemorrhage in China. Stroke. 2013; 44(10): 2891-3	Nonfatal Health Outcomes	Ischemic stroke
Hazell SL, Mitchell HM, Hedges M, Shi X, Hu PJ, Li YY, Lee A, Reiss-Levy E. Hepatitis A and evidence against the community dissemination of Helicobacter pylori via feces. J Infect Dis. 1994; 170(3): 686-9	Nonfatal Health Outcomes	Acute hepatitis A
Jin L, Ye R, Zheng J, Hong S, Ren A. Secular trends of hypospadias prevalence and factors associated with it in southeast China during 1993-2005. Birth Defects Res A Clin Mol Teratol. 2010; 88(6): 458-65	Nonfatal Health Outcomes	Urogenital congenital anomalies
Chen Y, Zhang X-J, Zhao Y-L, Zhang Y-H, Wang S-M, Hao Z-Y, Zhang Z-Y, Ma J-C, Fang Y, Wang X-Y. Waning of anti-HAV immunity in Shijiazhuang prefecture, Hebei province, China: a comparison of seroprevalence between 1992 and 2011. Vaccine. 2014; 32(47): 6227Â–32	Nonfatal Health Outcomes	Acute hepatitis A
Dong B-Q, Yang J, Wang X-Y, Gong J, von Seidlein L, Wang M-L, Lin M, Liao H-Z, Ochiai RL, Xu Z-Y, Jodar L, Clemens JD. Trends and disease burden of enteric fever in Guangxi province, China, 1994-2004. Bull World Health Organ. 2010; 88(9): 689-96	Nonfatal Health Outcomes	Typhoid and paratyphoid
Yu J-H, Chuang X-S, Liang G-X, Wan X-D. An epidemiological survey of psychoactive substance use in Zhenjiang City. J Clin Psychiatry. 1996; 6(2): 71-73	Nonfatal Health Outcomes	Alcohol use disorders
National Central Cancer Registry (China). China Cancer Registry Incidence and Mortality 1990-2009	Nonfatal Health Outcomes	Thyroid cancer
Ye BF, Shen J, Xu YC, Niu JY, Chen JG, Zhang BC, Liu B, Jiang YH. Etiology study on the relationship between HBV, HCV and HCC. Chin J Epidemiol. 1994; 15: 131-4	Nonfatal Health Outcomes	Liver cancer due to hepatitis B
Ye BF, Shen J, Xu YC, Niu JY, Chen JG, Zhang BC, Liu B, Jiang YH. Etiology study on the relationship between HBV, HCV and HCC. Chin J Epidemiol. 1994; 15: 131-4	Nonfatal Health Outcomes	Liver cancer due to hepatitis C
Ye T-W, Wu C-D, Chen W-W. Wang S-Y, Cai Y-D, Yang Z-Q, Yu M-H, Han T-M, Luo H-D, Li L-C, Huang S, Huang J-D, Wang G-L, Xie Y-N, Xu Z-X, Wu T-G, Guan S-M, Zhu G-Q, Wu C-Z. Epidemiological Investigation of Mental Disorders in Hainan Province. Hainan Med J. 1995; 6(4): 257-60	Nonfatal Health Outcomes	Alcohol use disorders
Li Y, Li Y, Yu D, Xia M, Hu S, Xiang Y, Zhong Z. A multivariate analysis of the relationship between work ability and S japonicum infection in Dongting Lake region, in China. Rev Inst Med Trop Sao Paulo. 1993; 35(4): 347-53	Nonfatal Health Outcomes	Schistosomiasis
Franceschi S, Smith JS, van den Brule A, Herrero R, Arslan A, Anh P-T-H, Bosch FX, Hieu N-T, Matos E, Posso H, Qiao Y-L, Shin H-R, Sukvirach S, Thomas JO, Snijders PJF, MuÃ±oz N, Meijer CJLM. Cervical infection with Chlamydia trachomatis and Neisseria gonorrhoeae in women from ten areas in four continents. A cross-sectional study. Sex Transm Dis. 2007; 34(8): 563-9	Nonfatal Health Outcomes	Sexually transmitted infections excluding HIV
Leung PW, Luk SL, Ho TP, Taylor E, Mak FL, Bacon-Shone J. The diagnosis and prevalence of hyperactivity in Chinese schoolboys. Br J Psychiatry. 1996; 168(4): 486-96	Nonfatal Health Outcomes	Attention-deficit/hyperactivity disorder
Tu Y, Zhou D-Y, Zheng W-M. Epidemiological investigation on mental disorders in Yichang area. Med J Chin Civ Adm. 1995; 7(6): 328-30	Nonfatal Health Outcomes	Schizophrenia

Yao WZ,Â Zhu H,Â Shen N,Â Han X,Â Liang YJ,Â Zhang LQ,Â Sun YC,Â Hao ZT,Â Zhao MW. Epidemiological data of chronic obstructive pulmonary disease in Yanqing County in Beijing. J Peking Univ Health Sci. 2005; 37(2): 121	Nonfatal Health Outcomes	Chronic obstructive pulmonary disease
Hui AC, Ng K, Tong P, Mok V, Chow K, Wu A, Wong LK. Bacterial meningitis in Hong Kong: 10-years experience. Clin Neurol Neurosurg. 2005; 107(5): 366-70	Nonfatal Health Outcomes	Hearing loss
World Health Organization (WHO). Mental Illness in General Health Care: An International Study. Geneva, Switzerland: World Health Organization (WHO), 1995	Nonfatal Health Outcomes	Alcohol use disorders
Petersen PE, Guang LX. Dental caries prevalence in a group of schoolchildren in Wuhan City, PR China, 1993. Community Dent Oral Epidemiol. 1994; 22(6): 465-6	Nonfatal Health Outcomes	Caries of deciduous teeth
Zhang JY, Dai M, Wang X, Lu WQ, Li DS, Zhang MX, Wang KJ, Dai LP, Han SG, Zhou YF, Zhuang H. A case-control study of hepatitis B and C virus infection as risk factors for hepatocellular carcinoma in Henan, China. Int J Epidemiol. 1998; 27(4): 574-8	Nonfatal Health Outcomes	Liver cancer due to hepatitis B
Wang CX, Feng XH, Yyuan RZ, Li DS, Wang HJ, Cheng SQ. A study of the relationship in HBV, HCV infection and primary hepatocellular carcinoma. Chin J Health Lab Tech. 1996; 6: 184-5	Nonfatal Health Outcomes	Liver cancer due to hepatitis B
Zhang JY, Dai M, Wang X, Lu WQ, Li DS, Zhang MX, Wang KJ, Dai LP, Han SG, Zhou YF, Zhuang H. A case-control study of hepatitis B and C virus infection as risk factors for hepatocellular carcinoma in Henan, China. Int J Epidemiol. 1998; 27(4): 574-8	Nonfatal Health Outcomes	Liver cancer due to hepatitis C
Wang W, Wu S, Cheng X, Dai H, Ross K, Du X, Yin W. Prevalence of Alzheimer's disease and other dementing disorders in an urban community of Beijing, China. Neuroepidemiology. 2000; 19(4): 194-200	Nonfatal Health Outcomes	Alzheimer's disease and other dementias
Lau EM, Egger P, Coggon D, Cooper C, Valenti L, O'Connell D. Low back pain in Hong Kong: prevalence and characteristics compared with Britain. J Epidemiol Community Health. 1995; 49(5): 492-4	Nonfatal Health Outcomes	Low back and neck pain
Woo J, Ho SC, Lau J, Leung PC. Musculoskeletal complaints and associated consequences in elderly Chinese aged 70 years and over. J Rheumatol. 1994; 21(10): 1927-31	Nonfatal Health Outcomes	Low back and neck pain
Lau EM, Sham A, Wong KC. The prevalence of and risk factors for neck pain in Hong Kong Chinese. J Public Health Med. 1996; 18(4): 396-9	Nonfatal Health Outcomes	Low back and neck pain
Sanderson JE, Chan SKW, Chan WWM, Hung YT, Woo KS. The aetiology of heart failure in the Chinese population of Hong Kong - a prospective study of 730 consecutive patients. Int J Cardiol. 1995; 51(1): 29-35	Nonfatal Health Outcomes	
Pan WS, Tian X, Tang SZ. A caseÂ–control study of HCV infection and primary liver cancer. Chin J Public Health. 1995; 11: 291-4	Nonfatal Health Outcomes	Liver cancer due to hepatitis B
Pan WS, Tian X, Tang SZ. A caseÂ–control study of HCV infection and primary liver cancer. Chin J Public Health. 1995; 11: 291-4	Nonfatal Health Outcomes	Liver cancer due to hepatitis C
Hui AC, Ng K, Tong P, Mok V, Chow K, Wu A, Wong LK. Bacterial meningitis in Hong Kong: 10-years experience. Clin Neurol Neurosurg. 2005; 107(5): 366-70	Nonfatal Health Outcomes	Vision loss
China National Maternal and Child Health Surveillance System Congenital Anomalies 1996-2012 - MCHS	Nonfatal Health Outcomes	Congenital birth defects
Chinese Center for Disease Control and Prevention (CCDC). China Disease Surveillance Points 1997 - China CDC	Nonfatal Health Outcomes	Rabies
China National Maternal and Child Health Surveillance System Congenital Anomalies 1996-2012 - MCHS	Nonfatal Health Outcomes	Neural tube defects
China National Maternal and Child Health Surveillance System Congenital Anomalies 1996-2012 - MCHS	Nonfatal Health Outcomes	Orofacial clefts
Chinese Center for Disease Control and Prevention (CCDC). China Disease Surveillance Points 1996 - China CDC	Nonfatal Health Outcomes	Rabies
China National Maternal and Child Health Surveillance System Congenital Anomalies 1996-2012 - MCHS	Nonfatal Health Outcomes	Congenital musculoskeletal and limb anomalies
China National Maternal and Child Health Surveillance System Congenital Anomalies 1996-2012 - MCHS	Nonfatal Health Outcomes	Down syndrome
European Surveillance of Congenital Anomalies (EUROCAT), International Centre on Birth Defects, World Health Organization (WHO). World Atlas of Birth Defects. 2nd ed. Geneva, Switzerland: World Health Organization (WHO), 2003	Nonfatal Health Outcomes	Digestive congenital anomalies
World Health Organization (WHO). Control of Foodborne Trematode Infections 1995. Geneva, Switzerland: World Health Organization (WHO), 1995	Nonfatal Health Outcomes	Food-borne trematodiases
Liu P, Shi ZX, Zhang YC, Xu ZC, Shu HS, Zhang XY. A prospective study of a serum-pooling strategy in screening blood donors for antibody to hepatitis C virus. Transfusion. 1997; 37(7): 732-6	Nonfatal Health Outcomes	Acute hepatitis C
Chinese Center for Disease Control and Prevention (CCDC). China Disease Surveillance Points 1995 - China CDC	Nonfatal Health Outcomes	Rabies

Li Z, Zhang K, Yan Y. [Evaluation on surveillance and intervention of lymphatic filariasis after interruption of the disease transmission in Jiangxi province]. Chin J Parasitol Parasit Dis. 2002; 20(4): 249-50	Nonfatal Health Outcomes	Lymphatic filariasis
Zhao J, Jia L, Sui R, Ellwein LB. Prevalence of blindness and cataract surgery in Shunyi County, China. Am J Ophthalmol. 1998; 126(4): 506-14	Nonfatal Health Outcomes	
Leung CB, Cheung WL, Li PKT. Renal registry in Hong Kong-the first 20 years. Kidney Int Suppl. 2015; 5(1): 33Â–8	Nonfatal Health Outcomes	Chronic kidney disease
China National Maternal and Child Health Surveillance System Congenital Anomalies 1996-2012 - MCHS	Nonfatal Health Outcomes	Digestive congenital anomalies
Zeng QY, Darmawan J, Xiao ZY, Chen SB, Chen R, Lin K, Wigley R, Chen SL, Zhang NZ. Risk factors associated with rheumatic complaints: a WHO-ILAR COPCORD study in Shantou, Southeast China. J Rheumatol. 2005; 32(5): 920-7	Nonfatal Health Outcomes	Rheumatoid arthritis
Zeng QY, Chen R, Xiao ZY, Huang S-B, Liu Y, Xu JC, Chen SL, Darmawan J, Couchman KG, Wigley RD, Muirden KD. Low prevalence of knee and back pain in southeast China; the Shantou COPCORD study. J Rheumatol. 2004; 31(12): 2439-43	Nonfatal Health Outcomes	Osteoarthritis
Nagel G, Weinmayr G, Flohr C, Kleiner A, Strachan DP. Association of pertussis and measles infections and immunizations with asthma and allergic sensitization in ISAAC Phase Two. Pediatr Allergy Immunol. 2012; 23(8): 737-46	Nonfatal Health Outcomes	Atopic dermatitis
Wang H-Y, Petersen PE, Bian J-Y, Zhang B-X. The second national survey of oral health status of children and adults in China. Int Dent J. 2002; 52(4): 283-90	Nonfatal Health Outcomes	Caries of deciduous teeth
Wang H-Y, Petersen PE, Bian J-Y, Zhang B-X. The second national survey of oral health status of children and adults in China. Int Dent J. 2002; 52(4): 283-90	Nonfatal Health Outcomes	Caries of permanent teeth
Li S, Xu J, He M, Wu K, Munoz SR, Ellwein LB. A survey of blindness and cataract surgery in Doumen County, China. Ophthalmology. 1999; 106(8): 1602-8	Nonfatal Health Outcomes	Refraction and accommodation disorders
China National Maternal and Child Health Surveillance System Congenital Anomalies 1996-2012 - MCHS	Nonfatal Health Outcomes	Urogenital congenital anomalies
Lin HC, Corbet EF, Lo EC, Zhang HG. Tooth loss, occluding pairs, and prosthetic status of Chinese adults. J Dent Res. 2001; 80(5): 1491-5	Nonfatal Health Outcomes	Edentulism and severe tooth loss
Cheng Z-R, Gao H, Zhang X, Tang Z-R, Lu Y-W, Yang W-Q, et al. Survey on mental disorder epidemiology and mental health service situation in Shenzhen city. Med J Chin Civ Adm. 1999; 11(1): 32-5	Nonfatal Health Outcomes	Schizophrenia
China National Maternal and Child Health Surveillance System Congenital Anomalies 1996-2012 - MCHS	Nonfatal Health Outcomes	Congenital heart anomalies
Peng T, Wang W-G, Zhao Y-Y, Liu A-S. Epidemiological investigation on mental disorders in Daowai District of Harbin. Chin Ment Health J. 1999; 13(3): 158-159	Nonfatal Health Outcomes	Schizophrenia
Peng T, Wang W-G, Zhao Y-Y, Liu A-S. Epidemiological investigation on mental disorders in Daowai District of Harbin. Chin Ment Health J. 1999; 13(3): 158-159	Nonfatal Health Outcomes	Alcohol use disorders
Li S, Xu J, He M, Wu K, Munoz SR, Ellwein LB. A survey of blindness and cataract surgery in Doumen County, China. Ophthalmology. 1999; 106(8): 1602-8	Nonfatal Health Outcomes	Vision loss
Wang SS, Jiang PL, Pang HX, Peng GF. An epidemiological study on the etiology of primary hepatocellular carcinoma in Guangzhou, Guangdong Province. Chin J Epidemiol. 1999; 18: 33-6	Nonfatal Health Outcomes	Liver cancer due to hepatitis B
Wang SS, Jiang PL, Pang HX, Peng GF. An epidemiological study on the etiology of primary hepatocellular carcinoma in Guangzhou, Guangdong Province. Chin J Epidemiol. 1999; 18: 33-6	Nonfatal Health Outcomes	Liver cancer due to hepatitis C
European Surveillance of Congenital Anomalies (EUROCAT), International Centre on Birth Defects, World Health Organization (WHO). World Atlas of Birth Defects. 2nd ed. Geneva, Switzerland: World Health Organization (WHO), 2003	Nonfatal Health Outcomes	Neural tube defects
Ho Y-W, Chau K-F, Choy B-Y, Fung K-S, Cheng Y-L, Kwan T-H, Wong P-N, Lai W-M, Yuen S-K, Hok-King L, Chan C-K, Leung C-B. Hong Kong renal registry report 2012. Hong Kong J Nephrol. 2013; 15(1): 28Â–43	Nonfatal Health Outcomes	Chronic kidney disease
Zhang L-X, Tu D-H, He G-X, Ma Z-Q, Nagelkerke NJ, Borgdorff MW, Enarson DA, Broekmans JF. Risk of tuberculosis infection and tuberculous meningitis after discontinuation of Bacillus CalmetteÂ–Guerin in Beijing. Am J Respir Crit Care Med. 2000; 162(4): 1314Â–1317	Nonfatal Health Outcomes	Tuberculosis
Liu Y, Zhang J, Li Z. Perinatal outcomes in native Chinese and Chinese-American women. Paediatr Perinat Epidemiol. 2011; 25(3): 202-9	Nonfatal Health Outcomes	Neonatal preterm birth
Ren A, Wang J, Ye RW, Li S, Liu JM, Li Z. Low first-trimester hemoglobin and low birth weight, preterm birth and small for gestational age newborns. Int J Gynaecol Obstet. 2007; 98(2): 124-8	Nonfatal Health Outcomes	Neonatal preterm birth
Yu JM, de Vlas SJ, Yuan HC, Gryseels B. Variations in fecal Schistosoma japonicum egg counts. Am J Trop Med Hyg. 1998; 59(3): 370Â–5	Nonfatal Health Outcomes	Schistosomiasis
Zuo Q, Liu YJ, Liang WT. A caseÂ–control study of the synergistic action of HBV and HCV in the development of primary hepatocellular carcinoma. Chin J Public Health. 1997; 16: 173-275	Nonfatal Health Outcomes	Liver cancer due to hepatitis B

Zuo Q, Liu YJ, Liang WT. A caseÂ–control study of the synergistic action of HBV and HCV in the development of primary hepatocellular carcinoma. Chin J Public Health. 1997; 16: 173-275	Nonfatal Health Outcomes	Liver cancer due to hepatitis C
Xiao X, Zhou Y, Sun W, Chen D. [Prevalence of syphilis during pregnancy and risk factors for maternal and perinatal infections: a 2009-2013 survey]. J South Med Univ. 2014; 34(1): 144Â–6	Nonfatal Health Outcomes	Syphilis
Li D, Cui LH, Jin DZ, Piao XX. Analysis of serum hepatitis B virus and hepatitis C virus infection markers in patients with primary carcinoma of liver. J Med Sci Yanbian Univ. 1999; 22: 185-8	Nonfatal Health Outcomes	Liver cancer due to hepatitis C
Tang M-N, Liu X-H, Han H-Y, Tang M-M, Wang Y-F, Zhang L-X. Prevalence of depressive disorders among residents aged 55 or above in Chengdu area. Chin Ment Health J. 2001; 15(2): 103-106	Nonfatal Health Outcomes	Major depressive disorder
Jin Q, Qiu-Min Q, Jian-Feng H. The epidemiology of Parkinson Disease among Elderly people in Xi'an, China. Chin J Neuroimmunol Neurol. 2001; 2: 79-83	Nonfatal Health Outcomes	Parkinson's disease
National Central Cancer Registry (China). China Cancer Registry Incidence and Mortality 1990-2009	Nonfatal Health Outcomes	Mesothelioma
Gong C, Meng X, Jiang Y, Wang X, Cui H, Chen X. Trends in childhood type 1 diabetes mellitus incidence in Beijing from 1995 to 2010: a retrospective multicenter study based on hospitalization data. Diabetes Technol Ther. 2015; 17(3): 159-65	Nonfatal Health Outcomes	Diabetes mellitus type 1
Zhou H, Ross AG, Hartel GF, Sleigh AC, Williams GM, McManus DP, Luo XS, He Y, Li YS. Diagnosis of schistosomiasis japonica in Chinese schoolchildren by administration of a questionnaire. Trans R Soc Trop Med Hyg. 1998; 92(3): 245-50	Nonfatal Health Outcomes	Schistosomiasis
Chiu HFK, Lam LCW, Chi I, Leung T, Li SW, Law WT, Chung DWS, Fung HHL, Kan PS, Lum CM, Ng J, Lau J. Prevalence of dementia in Chinese elderly in Hong Kong. Neurology. 1998; 50(4): 1002-9	Nonfatal Health Outcomes	Alzheimer's disease and other dementias
Ho SC, Chen YM, Woo JL, Leung SS, Lam TH, Janus ED. Association between simple anthropometric indices and cardiovascular risk factors. Int J Obes Relat Metab Disord. 2001; 25(11): 1689-97	Nonfatal Health Outcomes	Diabetes mellitus
Janus ED, Watt NM, Lam KS, Cockram CS, Siu ST, Liu LJ, Lam TH. The prevalence of diabetes, association with cardiovascular risk factors and implications of diagnostic criteria (ADA 1997 and WHO 1998) in a 1996 community-based population study in Hong Kong Chinese. Diabet Med. 2000; 17(10): 741-5	Nonfatal Health Outcomes	Diabetes mellitus
European Surveillance of Congenital Anomalies (EUROCAT), International Centre on Birth Defects, World Health Organization (WHO). World Atlas of Birth Defects. 2nd ed. Geneva, Switzerland: World Health Organization (WHO), 2003	Nonfatal Health Outcomes	Congenital birth defects
Weatherall D. Sickle Cell and Thalassemias Prevalence Data, Personal Correspondence with David Weatherall. [Unpublished]	Nonfatal Health Outcomes	Thalassemias
Fung WK, Lo KK. Prevalence of skin disease among school children and adolescents in a Student Health Service Center in Hong Kong. Pediatr Dermatol. 2000; 17(6): 440-6	Nonfatal Health Outcomes	Atopic dermatitis
Fung WK, Lo KK. Prevalence of skin disease among school children and adolescents in a Student Health Service Center in Hong Kong. Pediatr Dermatol. 2000; 17(6): 440-6	Nonfatal Health Outcomes	Urticaria
Chu CH, Fung DS, Lo EC. Dental caries status of preschool children in Hong Kong. Br Dent J. 1999; 187(11): 616-620	Nonfatal Health Outcomes	Caries of deciduous teeth
Kwong KL, Chak WK, Wong SN, So KT. Epidemiology of childhood epilepsy in a cohort of 309 Chinese children. Pediatr Neurol. 2001; 24(4): 276-82	Nonfatal Health Outcomes	Epilepsy
Wong V. Study of seizure and epilepsy in Chinese children in Hong Kong: period prevalence and patterns. J Child Neurol. 2004; 19(1): 19-25	Nonfatal Health Outcomes	Epilepsy
Hung YT, Cheung NT, Ip S, Fung H. Epidemiology of heart failure in Hong Kong, 1997. Hong Kong Med J. 2000; 6(2): 159-62	Nonfatal Health Outcomes	Heart failure
Fung WK, Lo KK. Prevalence of skin disease among school children and adolescents in a Student Health Service Center in Hong Kong. Pediatr Dermatol. 2000; 17(6): 440-6	Nonfatal Health Outcomes	Fungal skin diseases
Aoki K, Kihaile PE, Wenyuan Z, Xianghang Z, Castro M, Disla M, Nyambo TB, Misumi J. Comparison of prevalence of chronic atrophic gastritis in Japan, China, Tanzania, and the Dominican Republic. Ann Epidemiol. 2005; 15(8): 598-606	Nonfatal Health Outcomes	Gastritis and duodenitis
Leung TY, Leung TN, Sahota DS, Chan OK, Chan LW, Fung TY, Lau TK. Trends in maternal obesity and associated risks of adverse pregnancy outcomes in a population of Chinese women. BJOG. 2008; 115(12): 1529-37	Nonfatal Health Outcomes	Neonatal preterm birth
Leung TN, Roach VJ, Lau TK. Incidence of preterm delivery in Hong Kong Chinese. Aust N Z J Obstet Gynaecol. 1998; 38(2): 138-41	Nonfatal Health Outcomes	Neonatal preterm birth
Ran M-S, Chen EY-H, Conwell Y, Chan CL-W, Yip PS, Xiang M-Z, Caine ED. Mortality in people with schizophrenia in rural China 10-year cohort study. Br J Psychiatry. 2007; 190(3): 237Â–42	Nonfatal Health Outcomes	Schizophrenia

Lo JY, Lim WW, Ho DW, Field PR, Cunningham AL. Difference in seroprevalence of herpes simplex virus type 2 infection among antenatal women in Hong Kong and southern China. Sex Transm Infect. 1999; 75(2): 123	Nonfatal Health Outcomes	Genital herpes
Sheng J-L, Bai S-Z, Zhao L, Cai D-X, Wang Y-M, Wang A-J, Wang Y-Y. Epidemiological investigation on mental illness in Xinshi area, Xinjiang Province. Med J Chin Civ Adm. 2000; 12(1): 33-5	Nonfatal Health Outcomes	Schizophrenia
Yang JT, Zhao HG, Zhao SF, Li PZ. Prevalence of HCV and HBV infection in patients with primary hepatocellular carcinoma in Shanxi Province. Chin J Epidemiol. 1999; 20: 215-7	Nonfatal Health Outcomes	Liver cancer due to hepatitis B
Yuan J-M, Fan Y, Ognjanovic S, Wang R, Van Den Berg D, Govindarajan S, Mimi CY. Genetic polymorphisms of epidermal growth factor in relation to risk of hepatocellular carcinoma: two case-control studies . BMC Gastroenterol. 2013; 13(1): 32	Nonfatal Health Outcomes	Liver cancer due to hepatitis B
Lu CH, Hou SJ. The relationship in HBV, HCV infection and cirrhosis and primary liver cancer. Clin Med. 1997; 17: 17-18	Nonfatal Health Outcomes	Liver cancer due to hepatitis C
Yuan J-M, Fan Y, Ognjanovic S, Wang R, Van Den Berg D, Govindarajan S, Mimi CY. Genetic polymorphisms of epidermal growth factor in relation to risk of hepatocellular carcinoma: two case-control studies . BMC Gastroenterol. 2013; 13(1): 32	Nonfatal Health Outcomes	Liver cancer due to hepatitis C
Yuan J-M, Fan Y, Ognjanovic S, Wang R, Van Den Berg D, Govindarajan S, Mimi CY. Genetic polymorphisms of epidermal growth factor in relation to risk of hepatocellular carcinoma: two case-control studies . BMC Gastroenterol. 2013; 13(1): 32	Nonfatal Health Outcomes	Liver cancer due to alcohol use
Yuan J-M, Fan Y, Ognjanovic S, Wang R, Van Den Berg D, Govindarajan S, Mimi CY. Genetic polymorphisms of epidermal growth factor in relation to risk of hepatocellular carcinoma: two case-control studies . BMC Gastroenterol. 2013; 13(1): 32	Nonfatal Health Outcomes	Liver cancer due to other causes
Deng K, Qiu J, Dai L, Yi L, Deng C, Mu Y, Zhu J. Perinatal mortality in pregnancies with omphalocele: data from the Chinese national birth defects monitoring network, 1996-2006. BMC Pediatr. 2014; 14: 160	Nonfatal Health Outcomes	Digestive congenital anomalies
Ding D, Hong Z, Chen GS, Dai XY, Wu JZ, Wang WZ, De Boer HM, Sander JW, Prilipko L, Chisholm D. Primary care treatment of epilepsy with phenobarbital in rural China: Cost-outcome analysis from the WHO/ILAE/IBE global campaign against epilepsy demonstration project. Epilepsia. 2008; 49(3): 535-9	Nonfatal Health Outcomes	Epilepsy
Cui LH, Fang JN, Jin HZ, Quan ZY, Jin CJ. An analysis of the interaction of Hepatitis virus in primary hepatocellular carcinoma in Korean of Yanbian. Chin J Prev Control Chronic Dis. 1999; 7: 28-30	Nonfatal Health Outcomes	Liver cancer due to hepatitis B
Li D, Cui LH, Jin DZ, Piao XX. Analysis of serum hepatitis B virus and hepatitis C virus infection markers in patients with primary carcinoma of liver. J Med Sci Yanbian Univ. 1999; 22: 185-8	Nonfatal Health Outcomes	Liver cancer due to hepatitis B
Cui LH, Fang JN, Jin HZ, Quan ZY, Jin CJ. An analysis of the interaction of Hepatitis virus in primary hepatocellular carcinoma in Korean of Yanbian. Chin J Prev Control Chronic Dis. 1999; 7: 28-30	Nonfatal Health Outcomes	Liver cancer due to hepatitis C
Ross A, Li Y, Williams G, Li Y, Sleigh A, Zhang X, Zhou D, McManus D. An examination of current control strategies for Asian schistosomiasis in the Dongting lake region of China .2. A five year follow-up survey on Qingshan island. Addict Behav Rep. 1997; 68(1): 93Â–104	Nonfatal Health Outcomes	Schistosomiasis
Tsou K-I, Tsao P-N, Taiwan Infant Development Collaborative Study Group. The morbidity and survival of very-low-birth-weight infants in Taiwan. Acta Paediatr Taiwan. 2003; 44(6): 349Â–55	Nonfatal Health Outcomes	Neonatal preterm birth
Gao ZY, Li YL, Chai DF. Investigation of cervical Chlamydia trachomatis infection in gynecologic outpatients. Chin J Epidemiol. 1995; 16(4): 211-2	Nonfatal Health Outcomes	Sexually transmitted infections excluding HIV
European Surveillance of Congenital Anomalies (EUROCAT), International Centre on Birth Defects, World Health Organization (WHO). World Atlas of Birth Defects. 2nd ed. Geneva, Switzerland: World Health Organization (WHO), 2003	Nonfatal Health Outcomes	Congenital musculoskeletal and limb anomalies
Nagel G, Weinmayr G, Flohr C, Kleiner A, Strachan DP. Association of pertussis and measles infections and immunizations with asthma and allergic sensitization in ISAAC Phase Two. Pediatr Allergy Immunol. 2012; 23(8): 737-46	Nonfatal Health Outcomes	Dermatitis
Li S, Xu J, He M, Wu K, Munoz SR, Ellwein LB. A survey of blindness and cataract surgery in Doumen County, China. Ophthalmology. 1999; 106(8): 1602-8	Nonfatal Health Outcomes	Refraction disorders
Zhao Q, Zhou B, Ding D, Guo Q, Hong Z. Prevalence, mortality, and predictive factors on survival of dementia in Shanghai, China. Alzheimer Dis Assoc Disord. 2010; 24(2): 151-8	Nonfatal Health Outcomes	Alzheimer's disease and other dementias
Bin Z, Zhen H, Mao-Sheng H. Prevalence of Parkinson's Disease in Shanghai urban and rural area. J Brain Nerv Dis. 2001; 9(6): 330-2	Nonfatal Health Outcomes	Parkinson's disease

Yang JT, Zhao HG, Zhao SF, Li PZ. Prevalence of HCV and HBV infection in patients with primary hepatocellular carcinoma in Shanxi Province. Chin J Epidemiol. 1999; 20: 215-7	Nonfatal Health Outcomes	Liver cancer due to hepatitis C
World Health Organization (WHO). WHO Global Project on Anti-Tuberculosis Drug Resistance Surveillance Data 1988-2015	Nonfatal Health Outcomes	Multidrug-resistant tuberculosis without extensive drug resistance
World Health Organization (WHO). WHO Global Project on Anti-Tuberculosis Drug Resistance Surveillance Data 1988-2015	Nonfatal Health Outcomes	HIV/AIDS - Multidrug-resistant Tuberculosis without extensive drug resistance
Wang Y, Yang X-Z, Li J-R, Liang K-L. Epidemiological survey of male wine dependence in Urumqi, Inner Mongolia. Chin J Psychiatry. 1997; 11(6): 364-365	Nonfatal Health Outcomes	Alcohol use disorders
Wang Y-H, Heng K-L, Luo M, Li P-H. An Analysis of Relative Factors for Patients with Alcoholic Dependence in the Panzihua City. Sichuan Ment Health. 2001; 14(1): 36-8	Nonfatal Health Outcomes	Alcohol use disorders
Lin Y-C, Zhuang H-C, Deng Y-T. Epidemiological Investigation on ADHD among Pre-scholars in Putian Area. Fujian Med J. 1999; 21(4): 105-7	Nonfatal Health Outcomes	Attention-deficit/hyperactivity disorder
Zhao Z-S, Wu Y-T, Zhou J-Q, Zhao Y-L, Wang B-Z, Wang J-B, Li H-P, Li M-F, Zhao J-C, Shen J-X. Epidemiological Investigation on Schizophrenia in Dali Area in Yunnan Province. Mod Rehabil. 1997; 1(1): 78-9	Nonfatal Health Outcomes	Schizophrenia
Li S, Yan F, Li G, Chen C, Zhang W, Liu J, Jia X, Shen Y. Is the dementia rate increasing in Beijing? Prevalence and incidence of dementia 10 years later in an urban elderly population. Acta Psychiatr Scand. 2007; 115(1): 73-9	Nonfatal Health Outcomes	Alzheimer's disease and other dementias
Zhe, Tang, Chen, Meng, Donghui Qing. [Beijing urban and rural senile dementia prevalence study 1]. Chin J Gerontol. 2002; 22(04): 244-8	Nonfatal Health Outcomes	Alzheimer's disease and other dementias
Ran M-S, Mao W-J, Chan Cecilia L-W, Chen Eric Y-H, Conwell Y. Gender differences in outcomes in people with schizophrenia in rural China: 14-year follow-up study. Br J Psychiatry. 2015; 206(4): 283-288	Nonfatal Health Outcomes	Schizophrenia
Li S, Xu J, He M, Wu K, Munoz SR, Ellwein LB. A survey of blindness and cataract surgery in Doumen County, China. Ophthalmology. 1999; 106(8): 1602-8	Nonfatal Health Outcomes	
Xiao X, Zhou Y, Sun W, Chen D. [Prevalence of syphilis during pregnancy and risk factors for maternal and perinatal infections: a 2009-2013 survey]. J South Med Univ. 2014; 34(1): 144Â–6	Nonfatal Health Outcomes	Sexually transmitted infections excluding HIV
Tan VP, Wong BC, Wong WM, Leung WK, Tong D, Yuen MF, Fass R. Gastroesophageal Reflux Disease: Cross-Sectional Study Demonstrating Rising Prevalence in a Chinese Population. J Clin Gastroenterol. 2016; 50(1): e1-7	Nonfatal Health Outcomes	Gastroesophageal reflux disease
Xu JT, Li FH, Sun T et al. An investigation of Clonorchis sinensis in Liaoning. Chin J Parasit Dis Cont. 1998; 11: 75	Nonfatal Health Outcomes	Food-borne trematodiases
Gao J, Feng B, Li L. Establishing a surveillance network for children respiratory infection to reduce pneumonia mortality. Matern Child Health Care China. 2004; 19: 13Â–4	Nonfatal Health Outcomes	Lower respiratory infections
Dai S-M, Han X-H, Zhao D-B, Shi Y-Q, Liu Y, Meng J-M. Prevalence of rheumatic symptoms, rheumatoid arthritis, ankylosing spondylitis, and gout in Shanghai, China: a COPCORD study. J Rheumatol. 2003; 30(10): 2245-51	Nonfatal Health Outcomes	Gout
Shimbo S, Zhang ZW, Qu JB, Wang JJ, Zhang CL, Song LH, Watanabe T, Higashikawa K, Ikeda M. Urban-rural comparison of HBV and HCV infection prevalence among adult women in Shandong Province, China. Southeast Asian J Trop Med Public Health. 1997; 28(3): 500-6	Nonfatal Health Outcomes	Acute hepatitis B
Shimbo S, Zhang ZW, Qu JB, Wang JJ, Zhang CL, Song LH, Watanabe T, Higashikawa K, Ikeda M. Urban-rural comparison of HBV and HCV infection prevalence among adult women in Shandong Province, China. Southeast Asian J Trop Med Public Health. 1997; 28(3): 500-6	Nonfatal Health Outcomes	Acute hepatitis C
Ng KK, Ng PW, Tsang KL, Hong Kong Epilepsy Study Group. Clinical characteristics of adult epilepsy patients in the 1997 Hong Kong epilepsy registry. Chin Med J (Engl). 2001; 114(1): 84-7	Nonfatal Health Outcomes	Epilepsy
Zhang L-X, Tu D-H, He G-X, Ma Z-Q, Nagelkerke NJ, Borgdorff MW, Enarson DA, Broekmans JF. Risk of tuberculosis infection and tuberculous meningitis after discontinuation of Bacillus CalmetteÂ–Guerin in Beijing. Am J Respir Crit Care Med. 2000; 162(4): 1314Â–1317	Nonfatal Health Outcomes	HIV/AIDS - Extensively drug-resistant Tuberculosis
Zhang L-X, Tu D-H, He G-X, Ma Z-Q, Nagelkerke NJ, Borgdorff MW, Enarson DA, Broekmans JF. Risk of tuberculosis infection and tuberculous meningitis after discontinuation of Bacillus CalmetteÂ–Guerin in Beijing. Am J Respir Crit Care Med. 2000; 162(4): 1314Â–1317	Nonfatal Health Outcomes	Multidrug-resistant tuberculosis without extensive drug resistance
Zhang L-X, Tu D-H, He G-X, Ma Z-Q, Nagelkerke NJ, Borgdorff MW, Enarson DA, Broekmans JF. Risk of tuberculosis infection and tuberculous meningitis after discontinuation of Bacillus CalmetteÂ–Guerin in Beijing. Am J Respir Crit Care Med. 2000; 162(4): 1314Â–1317	Nonfatal Health Outcomes	HIV/AIDS - Multidrug-resistant Tuberculosis without extensive drug resistance

Zhang L-X, Tu D-H, He G-X, Ma Z-Q, Nagelkerke NJ, Borgdorff MW, Enarson DA, Broekmans JF. Risk of tuberculosis infection and tuberculous meningitis after discontinuation of Bacillus CalmetteÂ–Guerin in Beijing. Am J Respir Crit Care Med. 2000; 162(4): 1314Â–1317	Nonfatal Health Outcomes	Drug-susceptible tuberculosis
Zhang L-X, Tu D-H, He G-X, Ma Z-Q, Nagelkerke NJ, Borgdorff MW, Enarson DA, Broekmans JF. Risk of tuberculosis infection and tuberculous meningitis after discontinuation of Bacillus CalmetteÂ–Guerin in Beijing. Am J Respir Crit Care Med. 2000; 162(4): 1314Â–1317	Nonfatal Health Outcomes	HIV/AIDS - Drug-susceptible Tuberculosis
Zhang L-X, Tu D-H, He G-X, Ma Z-Q, Nagelkerke NJ, Borgdorff MW, Enarson DA, Broekmans JF. Risk of tuberculosis infection and tuberculous meningitis after discontinuation of Bacillus CalmetteÂ–Guerin in Beijing. Am J Respir Crit Care Med. 2000; 162(4): 1314Â–1317	Nonfatal Health Outcomes	Extensively drug-resistant tuberculosis
Zhang L-X, Tu D-H, He G-X, Ma Z-Q, Nagelkerke NJ, Borgdorff MW, Enarson DA, Broekmans JF. Risk of tuberculosis infection and tuberculous meningitis after discontinuation of Bacillus CalmetteÂ–Guerin in Beijing. Am J Respir Crit Care Med. 2000; 162(4): 1314Â–1317	Nonfatal Health Outcomes	Latent tuberculosis infection
Gao ZY, Li YL, Chai DF. Investigation of cervical Chlamydia trachomatis infection in gynecologic outpatients. Chin J Epidemiol. 1995; 16(4): 211-2	Nonfatal Health Outcomes	Chlamydial infection
World Health Organization (WHO). Progress towards the elimination of leprosy as a public health problem. Wkly Epidemiol Rec. 1996: 71(20): 149-56.	Nonfatal Health Outcomes	Leprosy
Wang LW, Li ZS, Li SD, Jin ZD, Zou DW, Chen F. Prevalence and clinical features of chronic pancreatitis in China: a retrospective multicenter analysis over 10 years. Pancreas. 2009; 38(3): 248Â–54	Nonfatal Health Outcomes	Pancreatitis
Wang W, Zhao D, Sun JY, Liu J, Qin LP, Wu ZS. Impact of new criterion of glucose level on the prevalence of impaired fasting glucose and risk of ischemic cardiovascular diseases. Chin J Intern Med. 2007; 46(1): 20-4	Nonfatal Health Outcomes	Diabetes mellitus
Dai M, Shi Y, Zhang JY, Zhou YF, Zhang MX, He Z. A study of the interaction of HBV and HCV in PHC. Henan J Oncol. 1998; 11: 81-3	Nonfatal Health Outcomes	Liver cancer due to hepatitis B
Dai M, Shi Y, Zhang JY, Zhou YF, Zhang MX, He Z. A study of the interaction of HBV and HCV in PHC. Henan J Oncol. 1998; 11: 81-3	Nonfatal Health Outcomes	Liver cancer due to hepatitis C
Chau JP, Chang AM, Chang AM. Relationship between premenstrual tension syndrome and anxiety in Chinese adolescents. J Adolesc Health. 1998; 22(3): 247-9	Nonfatal Health Outcomes	Premenstrual syndrome
Zhao G, Wang L, Qu C. Prevalence of premenstrual syndrome in reproductive women and its influential factors. Chin J Obstet Gynecol. 1998; 33(4): 222-4	Nonfatal Health Outcomes	Premenstrual syndrome
Wang H-Y, Petersen PE, Bian J-Y, Zhang B-X. The second national survey of oral health status of children and adults in China. Int Dent J. 2002; 52(4): 283-90	Nonfatal Health Outcomes	Edentulism and severe tooth loss
Hung YT, Cheung NT, Ip S, Fung H. Epidemiology of heart failure in Hong Kong, 1997. Hong Kong Med J. 2000; 6(2): 159-62	Nonfatal Health Outcomes	
Gong C, Meng X, Jiang Y, Wang X, Cui H, Chen X. Trends in childhood type 1 diabetes mellitus incidence in Beijing from 1995 to 2010: a retrospective multicenter study based on hospitalization data. Diabetes Technol Ther. 2015; 17(3): 159-65	Nonfatal Health Outcomes	Diabetes mellitus
Yu SZ, Zi XL, Chen G, Li J. The relationship between viral hepatitis and primary liver cancer in four areas of China. Chin J Epidemiol. 1997; 18: 214-6	Nonfatal Health Outcomes	Liver cancer due to hepatitis B
Yu SZ, Zi XL, Chen G, Li J. The relationship between viral hepatitis and primary liver cancer in four areas of China. Chin J Epidemiol. 1997; 18: 214-6	Nonfatal Health Outcomes	Liver cancer due to hepatitis C
Ming L, Thorgeirsson SS, Gail MH, Lu P, Harris CC, Wang N, Shao Y, Wu Z, Liu G, Wang X, Sun Z. Dominant role of hepatitis B virus and cofactor role of aflatoxin in hepatocarcinogenesis in Qidong, China. Hepatology. 2002; 36(5): 1214-20	Nonfatal Health Outcomes	Liver cancer due to hepatitis B
European Surveillance of Congenital Anomalies (EUROCAT), International Centre on Birth Defects, World Health Organization (WHO). World Atlas of Birth Defects. 2nd ed. Geneva, Switzerland: World Health Organization (WHO), 2003	Nonfatal Health Outcomes	Urogenital congenital anomalies
Deng K, Qiu J, Dai L, Yi L, Deng C, Mu Y, Zhu J. Perinatal mortality in pregnancies with omphalocele: data from the Chinese national birth defects monitoring network, 1996-2006. BMC Pediatr. 2014; 14: 160	Nonfatal Health Outcomes	Congenital birth defects
Zhao J, Jia L, Sui R, Ellwein LB. Prevalence of blindness and cataract surgery in Shunyi County, China. Am J Ophthalmol. 1998; 126(4): 506-14	Nonfatal Health Outcomes	Vision loss
Gao XH, Zhang CL, Li XL, Mei H, Qi F, Zhu XY. Analysis of the infection hepatitis B and C from sera of the patients suffered from primary hepatoma. J Dalian Med Univ. 1998; 20: 19-21	Nonfatal Health Outcomes	Liver cancer due to hepatitis C
European Surveillance of Congenital Anomalies (EUROCAT), International Centre on Birth Defects, World Health Organization (WHO). World Atlas of Birth Defects. 2nd ed. Geneva, Switzerland: World Health Organization (WHO), 2003	Nonfatal Health Outcomes	Down syndrome
United Nations Children's Fund (UNICEF), World Health Organization (WHO). WHO and UNICEF Reported Disease Incidence Time Series. Geneva, Switzerland: World Health Organization (WHO)	Nonfatal Health Outcomes	Measles

Â Shen J, Xu YC, Gao Z, Niu JY, Shen HB, Ye BF. Epidemiological statocellulaudy on the etiologic synergistic interaction of HCV and HBV in the development of hepatocacinoma. Chin Natl J New Gastroenterol. 1997; 14: 72-74	Nonfatal Health Outcomes	Liver cancer due to hepatitis B
Â Shen J, Xu YC, Gao Z, Niu JY, Shen HB, Ye BF. Epidemiological statocellulaudy on the etiologic synergistic interaction of HCV and HBV in the development of hepatocacinoma. Chin Natl J New Gastroenterol. 1997; 14: 72-74	Nonfatal Health Outcomes	Liver cancer due to hepatitis C
Ming L, Thorgeirsson SS, Gail MH, Lu P, Harris CC, Wang N, Shao Y, Wu Z, Liu G, Wang X, Sun Z. Dominant role of hepatitis B virus and cofactor role of aflatoxin in hepatocarcinogenesis in Qidong, China. Hepatology. 2002; 36(5): 1214-20	Nonfatal Health Outcomes	Liver cancer due to hepatitis C
Hu Y-Z, Wu H-R, Yu J-Q. Subtyping and Inquiring for Ethology of Children ADHD Aged 6 to 12 Years. Chin J Sch Dr. 1998; 12(5): 321-4	Nonfatal Health Outcomes	Attention-deficit/hyperactivity disorder
Petersen P-E, Peng B, Bao Jun Tai. Oral health status and oral health behaviour of middle-aged and elderly people in PR China. Int Dent J. 1997; 47(6): 305	Nonfatal Health Outcomes	Periodontal diseases
Fung WK, Lo KK. Prevalence of skin disease among school children and adolescents in a Student Health Service Center in Hong Kong. Pediatr Dermatol. 2000; 17(6): 440-6	Nonfatal Health Outcomes	Scabies
Fung WK, Lo KK. Prevalence of skin disease among school children and adolescents in a Student Health Service Center in Hong Kong. Pediatr Dermatol. 2000; 17(6): 440-6	Nonfatal Health Outcomes	Acne vulgaris
Dai S-M, Han X-H, Zhao D-B, Shi Y-Q, Liu Y, Meng J-M. Prevalence of rheumatic symptoms, rheumatoid arthritis, ankylosing spondylitis, and gout in Shanghai, China: a COPCORD study. J Rheumatol. 2003; 30(10): 2245-51	Nonfatal Health Outcomes	Rheumatoid arthritis
Chen S, Du H, Wang Y, Xu L. The epidemiology study of hyperuricemia and gout in a community population of Huangpu District in Shanghai. Chin Med J (Engl). 1998; 111(3): 228-30	Nonfatal Health Outcomes	Gout
Gao XH, Zhang CL, Li XL, Mei H, Qi F, Zhu XY. Analysis of the infection hepatitis B and C from sera of the patients suffered from primary hepatoma. J Dalian Med Univ. 1998; 20: 19-21	Nonfatal Health Outcomes	Liver cancer due to hepatitis B
Lu CH, Hou SJ. The relationship in HBV, HCV infection and cirrhosis and primary liver cancer. Clin Med. 1997; 17: 17-18	Nonfatal Health Outcomes	Liver cancer due to hepatitis B
Fung WK, Lo KK. Prevalence of skin disease among school children and adolescents in a Student Health Service Center in Hong Kong. Pediatr Dermatol. 2000; 17(6): 440-6	Nonfatal Health Outcomes	Dermatitis
Zhang X-G, Zhuang X-H, Chen J-F, Zhao D-Q. Epidemiological study of schizophrenia in metropolitan Shantou. J Clin Psychol Med. 1997; 7(2): 83-85	Nonfatal Health Outcomes	Schizophrenia
Chinese Center for Disease Control and Prevention (CCDC). China Disease Surveillance Points 1999 - China CDC	Nonfatal Health Outcomes	Rabies
Chinese Center for Disease Control and Prevention (CCDC). China Disease Surveillance Points 1998 - China CDC	Nonfatal Health Outcomes	Rabies
Chinese Center for Disease Control and Prevention (CCDC). China Disease Surveillance Points 2000 - China CDC	Nonfatal Health Outcomes	Rabies
Huon GF, Mingyi Q, Oliver K, Xiao G. A large-scale survey of eating disorder symptomatology among female adolescents in the People's Republic of China. Int J Eat Disord. 2002; 32(2): 192-205	Nonfatal Health Outcomes	Anorexia nervosa
Lin S, Cheng TO, Liu X, Mai J, Rao X, Gao X, Deng H, Shi M. Impact of dysglycemia, body mass index, and waist-to-hip ratio on the prevalence of systemic hypertension in a lean Chinese population. Am J Cardiol. 2006; 97(6): 839-42	Nonfatal Health Outcomes	Diabetes mellitus
Lao TT, Sahota DS, Cheng YKY, Law LW, Leung TY. Advanced maternal age and postpartum hemorrhage - risk factor or red herring?. J Matern Fetal Neonatal Med. 2014 Feb;27(3):243-6. doi: 10.3109/14767058.2013.807240. Epub 2013 Jul 23. PMID: 23713943	Nonfatal Health Outcomes	Maternal hemorrhage
Chinese Center for Disease Control and Prevention (CCDC). China National Tuberculosis Prevalence Survey 2000	Nonfatal Health Outcomes	Extensively drug-resistant tuberculosis
Chinese Center for Disease Control and Prevention (CCDC). China National Tuberculosis Prevalence Survey 2000	Nonfatal Health Outcomes	Tuberculosis
Chinese Center for Disease Control and Prevention (CCDC). China National Tuberculosis Prevalence Survey 2000	Nonfatal Health Outcomes	Multidrug-resistant tuberculosis without extensive drug resistance
Huang J-M, Lv J-J, Xu J-Q, Chen X-Q, Huo B-Q, Jiang L-Y, Zeng J-S, Liu L-H. A Cross-sectional Study and Quality of Life Evaluation for Mental Disorders in a Community. J Prev Med Inf. 2001; 17(6): 420-2	Nonfatal Health Outcomes	Schizophrenia
Luo W, Lin L, Chen R, Cheng W, Huang Y, Hu T, et al. Epidemiological investigation on autistic disorder in Fujian province. Shanghai Arch Psychiatry. 2000; 12(1): 3Â–5	Nonfatal Health Outcomes	Autism spectrum disorders
Chang W-C, Hui Christy L-M, Wong Gloria H-Y, Chan Sherry K-W, Lee Edwin H-M, Chen Eric Y-H. Symptomatic remission and cognitive impairment in first-episode schizophrenia: A prospective 3-year follow-up study. J Clin Psychiatry. 2013; 74(11): 1046-1053	Nonfatal Health Outcomes	Schizophrenia

Dunzhu S, Wang FS, Courtright P, Liu L, Tenzing C, Noertjojo K, Wilkie A, Santangelo M, Bassett KL. Blindness and eye diseases in Tibet: findings from a randomised, population based survey. Br J Ophthalmol. 2003; 87(12): 1443-8	Nonfatal Health Outcomes	Vision loss
Chinese Center for Disease Control and Prevention (CCDC). China National Tuberculosis Prevalence Survey 2000	Nonfatal Health Outcomes	Drug-susceptible tuberculosis
Chinese Center for Disease Control and Prevention (CCDC). China National Tuberculosis Prevalence Survey 2000	Nonfatal Health Outcomes	HIV/AIDS - Drug-susceptible Tuberculosis
Chen C-Y, Liu C-Y, Su W-C, Huang S-L, Lin K-M. Factors associated with the diagnosis of neurodevelopmental disorders: a population-based longitudinal study. Pediatrics. 2007; 119(2): e435-443	Nonfatal Health Outcomes	Developmental intellectual disability
Corbet EF, Wong MC, Lin HC. Periodontal conditions in adult Southern Chinese. J Dent Res. 2001; 80(5): 1480-5	Nonfatal Health Outcomes	Periodontal diseases
Rong WS, Bian JY, Wang WJ, Wang JD. Effectiveness of an oral health education and caries prevention program in kindergartens in China. Community Dent Oral Epidemiol. 2003; 31(6): 412-6	Nonfatal Health Outcomes	Caries of deciduous teeth
Chinese Center for Disease Control and Prevention (CCDC). China National Tuberculosis Prevalence Survey 2000	Nonfatal Health Outcomes	HIV/AIDS - Multidrug-resistant Tuberculosis without extensive drug resistance
Meng Q-Z, Yu Z-G, Liu J-P, He P-H, Zhang X-P, Liu J, Ma H-Z, He P-E. Epidemiological investigation on mental disorders among rural population. Med J Chin Civ Adm. 2001; 13(1): 31-2	Nonfatal Health Outcomes	Schizophrenia
Wei M, Chen H, Fan Y-C, Pathai S. Prevalence and causes of visual impairment and blindness in Sichuan province of China. Int J Ophthalmol. 2010; 3(1): 83-8	Nonfatal Health Outcomes	Vision loss
Mo X-M, Oyang W-P, Chen J-R. Survey on Prevalence of Psychopathy in Hualin Street, Liwan District, Guangzhou. South China J Prev Med. 2003; 29(1): 48-9	Nonfatal Health Outcomes	Schizophrenia
Zhang Y, Xu L, Nevitt MC, Aliabadi P, Yu W, Qin M, Lui LY, Felson DT. Comparison of the prevalence of knee osteoarthritis between the elderly Chinese population in Beijing and whites in the United States: The Beijing Osteoarthritis Study. Arthritis Rheum. 2001; 44(9): 2065-71	Nonfatal Health Outcomes	Osteoarthritis
World Health Organization (WHO). WHO Global Programme for the Prevention of Rheumatic Fever and Rheumatic Heart Disease: Report of a Consultation to Review Progress and Develop Future Activities. Geneva, Switzerland: World Health Organization (WHO), 2000	Nonfatal Health Outcomes	Rheumatic heart disease
Yan-hui D, Chen G-S, Xiu-Ying D. Epidemiological Survey on Epilepsy in Rural Residents of Ningxia and on Tentative Effectiveness with Single Antiepileptic Drug-Luminal. J Ningxia Med Coll. 2002; 5(24): 316-22	Nonfatal Health Outcomes	Epilepsy
Curado MP, Edwards B, Shin HR, Storm H, Ferlay J, Heanue M and Boyle P, eds (2007). Cancer Incidence in Five Continents, Vol. IX Periodic Data (electronic version). Lyon, IARC. http://ci5.iarc.fr	Nonfatal Health Outcomes	Non-melanoma skin cancer (squamous-cell carcinoma)
Wei M, Chen H, Fan Y-C, Pathai S. Prevalence and causes of visual impairment and blindness in Sichuan province of China. Int J Ophthalmol. 2010; 3(1): 83-8	Nonfatal Health Outcomes	
Hong Kong Cancer Registry. Hong Kong Cancer Registry Annual Tables. Kowloon, Hong Kong: Hong Kong Cancer Registry	Nonfatal Health Outcomes	Leukemia
Dang S, Yan H, Yamamoto S, Wang X, Zeng L. Poor nutritional status of younger Tibetan children living at high altitudes. Eur J Clin Nutr. 2004; 58(6): 938-46	Nonfatal Health Outcomes	Dietary iron deficiency
Dang S, Yan H, Yamamoto S, Wang X, Zeng L. Poor nutritional status of younger Tibetan children living at high altitudes. Eur J Clin Nutr. 2004; 58(6): 938-46	Nonfatal Health Outcomes	Anemia
Ying Tang, Guo-Zhong Li. Epidemiological survey of epilepsy in Dongning county, a rural area in Heilongjiang Province of China. Chin J Clin Rehab. 2004; 8(4): 770-1	Nonfatal Health Outcomes	Epilepsy
Lao TT, Sahota DS, Suen SS, Law LW. The impact of fetal gender on preterm birth in a southern Chinese population. J Matern Fetal Neonatal Med. 2011; 24(12): 1440-3	Nonfatal Health Outcomes	Neonatal preterm birth
Tai-Ping Wang, Mei-Hua Han, Fen-Xia Li. [Twice Epilepsy Epidemiology Survey Report of Shanxi]. Chin J Integr Med Cardio Cerebrovasc Dis. 2009; 7(1): 111-2	Nonfatal Health Outcomes	Epilepsy
Zhou Z, Hu D. An Epidemiological Study on the Prevalence of Atrial Fibrillation in the Chinese Population of Mainland China. J Epidemiol. 2008; 18(5): 209-16	Nonfatal Health Outcomes	Atrial fibrillation and flutter
Nevitt MC, Xu L, Zhang Y, Lui L-Y, Yu W, Lane NE, Qin M, Hochberg MC, Cummings SR, Felson DT. Very low prevalence of hip osteoarthritis among Chinese elderly in Beijing, China, compared with whites in the United States: the Beijing osteoarthritis study. Arthritis Rheum. 2002; 46(7): 1773-9	Nonfatal Health Outcomes	Osteoarthritis
Dunzhu S, Wang FS, Courtright P, Liu L, Tenzing C, Noertjojo K, Wilkie A, Santangelo M, Bassett KL. Blindness and eye diseases in Tibet: findings from a randomised, population based survey. Br J Ophthalmol. 2003; 87(12): 1443-8	Nonfatal Health Outcomes	
Zhenxin Zhang, Jing Wei, Xia Hong. Prevalence of dementia and major subtypes in urban and rural communities of Beijing. Chin J Neurol. 2001; 34(4): 199-203	Nonfatal Health Outcomes	Alzheimer's disease and other dementias
Huang M, Hong Z, Zeng J, Rong X, Sheng Y, Lu C. [The prevalence of epilepsy in rural Jinshan in Shanghai]. Chin J Epidemiol. 2002; 23(5): 345-6	Nonfatal Health Outcomes	Epilepsy

World Health Organization Regional Office for the Western Pacific (WPRO-WHO). Number of Reported Cases of Dengue Fever and Dengue Hemorrhagic Fever in the Western Pacific Region, by Country 2000. Manila, Philippines: World Health Organization Regional Office for the Western Pacific (WPRO-WHO)	Nonfatal Health Outcomes	Dengue
Zhao Z, Sun C, Wang C, Li P, Wang W, Ye J, Gu X, Wang X, Shen S, Zhi D, Lu Z, Ye R, Cheng R, Xi L, Li X, Zheng Z, Zhang M, Luo F. Rapidly rising incidence of childhood type 1 diabetes in Chinese population: epidemiology in Shanghai during 1997-2011. Acta Diabetol. 2014; 51(6): 947-53	Nonfatal Health Outcomes	Diabetes mellitus type 1
Zhao Z, Sun C, Wang C, Li P, Wang W, Ye J, Gu X, Wang X, Shen S, Zhi D, Lu Z, Ye R, Cheng R, Xi L, Li X, Zheng Z, Zhang M, Luo F. Rapidly rising incidence of childhood type 1 diabetes in Chinese population: epidemiology in Shanghai during 1997-2011. Acta Diabetol. 2014; 51(6): 947-53	Nonfatal Health Outcomes	Diabetes mellitus
Curado MP, Edwards B, Shin HR, Storm H, Ferlay J, Heanue M and Boyle P, eds (2007). Cancer Incidence in Five Continents, Vol. IX Periodic Data (electronic version). Lyon, IARC. http://ci5.iarc.fr	Nonfatal Health Outcomes	Non-melanoma skin cancer (basal-cell carcinoma)
Lau KK, Wong LKS, Li LSW, Chan YW, Li HL, Wong V. Epidemiological study of multiple sclerosis in Hong Kong Chinese: questionnaire survey. Hong Kong Med J. 2002; 8(2): 77Â–80	Nonfatal Health Outcomes	Multiple sclerosis
Zhou DF, Wu CS, Qi H, Fan JH, Sun XD, Como P, Qiao YL, Zhang L, Kieburtz K. Prevalence of dementia in rural China: impact of age, gender and education. Acta Neurol Scand. 2006; 114(4): 273-80	Nonfatal Health Outcomes	Alzheimer's disease and other dementias
London School of Hygiene and Tropical Medicine. Global Atlas of Helminth Infections - Soil Transmitted Helminths. London, United Kingdom: London School of Hygiene and Tropical Medicine	Nonfatal Health Outcomes	Hookworm disease
Xu Z-G, Wang T-P, Sun Y-P. Epidemiological survey on epilepsy in rural population of Zezhou Shanxi. Proc Clin Med. 2002; 11: 811-14	Nonfatal Health Outcomes	Epilepsy
Lao TT, Sahota DS, Cheng YKY, Law LW, Leung TY. Advanced maternal age and postpartum hemorrhage - risk factor or red herring?. J Matern Fetal Neonatal Med. 2014 Feb;27(3):243-6. doi: 10.3109/14767058.2013.807240. Epub 2013 Jul 23. PMID: 23713943	Nonfatal Health Outcomes	Maternal disorders
Hui AC, Wong A, Wong HC, Man BL, Au-Yeung KM, Wong KS. Refractory epilepsy in a Chinese population. Clin Neurol Neurosurg. 2007; 109(8): 672-5	Nonfatal Health Outcomes	Epilepsy
Leung CC, Yew WW, Au KF, Tam CM, Chang KC, Mak KY, Tam SY, Chan KF, Fong KL, Tam SW. A Strong Tuberculin Reaction in Primary School Children Predicts Tuberculosis in Adolescence:. Pediatr Infect Dis J. 2012; 31(2): 150Â–3	Nonfatal Health Outcomes	Drug-susceptible tuberculosis
Leung CC, Yew WW, Au KF, Tam CM, Chang KC, Mak KY, Tam SY, Chan KF, Fong KL, Tam SW. A Strong Tuberculin Reaction in Primary School Children Predicts Tuberculosis in Adolescence:. Pediatr Infect Dis J. 2012; 31(2): 150Â–3	Nonfatal Health Outcomes	Tuberculosis
Leung CC, Yew WW, Au KF, Tam CM, Chang KC, Mak KY, Tam SY, Chan KF, Fong KL, Tam SW. A Strong Tuberculin Reaction in Primary School Children Predicts Tuberculosis in Adolescence:. Pediatr Infect Dis J. 2012; 31(2): 150Â–3	Nonfatal Health Outcomes	HIV/AIDS - Drug-susceptible Tuberculosis
Leung CC, Yew WW, Au KF, Tam CM, Chang KC, Mak KY, Tam SY, Chan KF, Fong KL, Tam SW. A Strong Tuberculin Reaction in Primary School Children Predicts Tuberculosis in Adolescence:. Pediatr Infect Dis J. 2012; 31(2): 150Â–3	Nonfatal Health Outcomes	Extensively drug-resistant tuberculosis
Leung CC, Yew WW, Au KF, Tam CM, Chang KC, Mak KY, Tam SY, Chan KF, Fong KL, Tam SW. A Strong Tuberculin Reaction in Primary School Children Predicts Tuberculosis in Adolescence:. Pediatr Infect Dis J. 2012; 31(2): 150Â–3	Nonfatal Health Outcomes	HIV/AIDS - Extensively drug-resistant Tuberculosis
Leung CC, Yew WW, Au KF, Tam CM, Chang KC, Mak KY, Tam SY, Chan KF, Fong KL, Tam SW. A Strong Tuberculin Reaction in Primary School Children Predicts Tuberculosis in Adolescence:. Pediatr Infect Dis J. 2012; 31(2): 150Â–3	Nonfatal Health Outcomes	Multidrug-resistant tuberculosis without extensive drug resistance
Leung CC, Yew WW, Au KF, Tam CM, Chang KC, Mak KY, Tam SY, Chan KF, Fong KL, Tam SW. A Strong Tuberculin Reaction in Primary School Children Predicts Tuberculosis in Adolescence:. Pediatr Infect Dis J. 2012; 31(2): 150Â–3	Nonfatal Health Outcomes	HIV/AIDS - Multidrug-resistant Tuberculosis without extensive drug resistance
National Opinion Research Center, University of Chicago (NORC), Renmin University, Beijing, Peking Union Medical College, Beijing, University of North Carolina. China Health and Family Life Survey 1999-2000. Chicago, USA: Population Research Center, University of Chicago	Nonfatal Health Outcomes	Sexual violence
Ma GY, Li ZQ, Lu S, Wang LH, et al. Survey of etiological factors of epilepsy in Mudanjian rural population by randomly cluster sampling. Chin J Clin Rehab. 2004; 8(16): 3178-9	Nonfatal Health Outcomes	Epilepsy
World Health Organization Regional Office for the Western Pacific (WPRO-WHO). Review on the Epidemiological Profile of Helminthiases and their Control in the Western Pacific Region, 1997-2008. Manila, Philippines: World Health Organization Regional Office for the Western Pacific (WPRO-WHO), 2008	Nonfatal Health Outcomes	Food-borne trematodiases

Gao JD, Shao YF, Xu Y, Ming LH, Wu ZY, Liu GT, Wang XH, Gao WH, Sun YT, Feng XL, Liang LM, Zhang YH, Sun ZT. Tight association of hepatocellular carcinoma with HBV infection in North China. Hepatobiliary Pancreat Dis Int. 2005; 4(1): 46-9	Nonfatal Health Outcomes	Liver cancer due to hepatitis B
Kong CK, Cheng WW, Wong LY. Epidemiology of headache in Hong Kong primary-level schoolchildren: questionnaire study. Hong Kong Med J. 2001; 7(1): 29-33	Nonfatal Health Outcomes	Migraine
Wang W, Wu J, Wang D, Chen G, Wang T, Yuan C, Yang B, Zhao D. [Epidemiological survey on epilepsy among rural populations in five provinces in China]. Nat Med J Chin. 2002; 82(7): 449-52	Nonfatal Health Outcomes	Epilepsy
Sun G, Xu Z-M, Liang J-F, Li L, Tang D-X. Twelve-year prevalence of common neonatal congenital malformations in Zhejiang Province, China. World J Pediatr. 2011; 7(4): 331-6	Nonfatal Health Outcomes	Congenital birth defects
Zhang ZW, Shimbo S, Qu JB, Liu ZM, Cai XC, Wang LQ, Watanabe T, Nakatsuka H, Matsuda-Inoguchi N, Higashikawa K, Ikeda M. Hepatitis B and C virus infection among adult women in Jilin Province, China: an urban-rural comparison in prevalence of infection markers. Southeast Asian J Trop Med Public Health. 2000; 31(3): 530-6	Nonfatal Health Outcomes	Acute hepatitis B
Tu D, Liu Y, Zhang L. [Tuberculin reactivity criteria for chemoprophylaxis in college students]. J Chin Antituberc Assoc. 2006; 28(5): 265Â–8	Nonfatal Health Outcomes	HIV/AIDS - Extensively drug-resistant Tuberculosis
Su MH, Wu JZ, Luo GH, Huang LY, Chen MW. A caseÂ–control study of relationship in HBV, HCV infection and HCC in areas with low incidences in Guangxi. Clin Foc. 2002; 17: 209-10	Nonfatal Health Outcomes	Liver cancer due to hepatitis B
Su MH, Wu JZ, Luo GH, Huang LY, Chen MW. A caseÂ–control study of relationship in HBV, HCV infection and HCC in areas with low incidences in Guangxi. Clin Foc. 2002; 17: 209-10	Nonfatal Health Outcomes	Liver cancer due to hepatitis C
Yuan J, Zhang Z, Wen H, Hong X, Hong Z, Qu Q, Tang M, Wu J, Xu Q, Li H, Cummings JL. Incidence of dementia and subtypes: A cohort study in four regions in China. Alzheimers Dement. 2016; 12(3): 262-71	Nonfatal Health Outcomes	Alzheimer's disease and other dementias
Lee G. End-stage renal disease in the Asian-Pacific region. Semin Nephrol. 2003; 23(1): 107-14	Nonfatal Health Outcomes	Chronic kidney disease
Lao TT, Sahota DS, Cheng YKY, Law LW, Leung TY. Advanced maternal age and postpartum hemorrhage - risk factor or red herring?. J Matern Fetal Neonatal Med. 2014 Feb;27(3):243-6. doi: 10.3109/14767058.2013.807240. Epub 2013 Jul 23. PMID: 23713943	Nonfatal Health Outcomes	Maternal hypertensive disorders
Cheng C-A, Ai C-Q, Cheng J-L, He J-P, Ma G-Z, He Y-M. Epidemiological investigation on mental health in Danjiangkou city in 1998. Med J Chin Civ Adm. 2001; 13(6): 334-5	Nonfatal Health Outcomes	Schizophrenia
Tang Jennifer Y-M, Chang W-C, Hui Christy L-M, Wong Gloria H-Y, Chan Sherry K-W, Lee Edwin H-M, Yeung W-S, Wong C-K, Tang W-N, Chan W-F, Pang Edwin P-F, Tso Steve, Ng Roger M-K, Hung S-F, Dunn Eva L-W, Sham P-C, Chen Eric Y-H. Prospective relationship between duration of untreated psychosis and 13-year clinical outcome: A first-episode psychosis study. Schizophr Res. 2014; 153(1-3): 1-8	Nonfatal Health Outcomes	Schizophrenia
Wen H, Zhang Z, Huang J, Duan L, Wang Q. Mortality of dementia and its major subtypes in urban and rural communities of Beijing. Biomed Environ Sci. 2011; 24(5): 483-90	Nonfatal Health Outcomes	Alzheimer's disease and other dementias
Deng X-H, Yang M-H, Zou J-H, Peng H-Z, Du J-L, Zhou X-H, Gao H-J, Tang Z-H, Liu Y-Q, Zhao D-M, Gu W-J, Wu B, Zhu Y-Q, Ding Y-J. Epidemiological Survey of the Mental Illness in Leshan City of Sichuan Province. Med J Chin Peoples Health. 2003; 15(5): 265-8	Nonfatal Health Outcomes	Alcohol use disorders
Chen C-Y, Liu C-Y, Su W-C, Huang S-L, Lin K-M. Factors associated with the diagnosis of neurodevelopmental disorders: a population-based longitudinal study. Pediatrics. 2007; 119(2): e435-443	Nonfatal Health Outcomes	Idiopathic developmental intellectual disability
Li XJ, Yang HX, Wang XQ, Tang NH, Xu CS. Detection of HBV and HCV in patients with primary liver cancer. J Fujian Med Univ. 2002; 36: 380-1	Nonfatal Health Outcomes	Liver cancer due to hepatitis B
Li XJ, Yang HX, Wang XQ, Tang NH, Xu CS. Detection of HBV and HCV in patients with primary liver cancer. J Fujian Med Univ. 2002; 36: 380-1	Nonfatal Health Outcomes	Liver cancer due to hepatitis C
Gilbert CE, Ellwein LB. Prevalence and causes of functional low vision in school-age children: results from standardized population surveys in Asia, Africa, and Latin America. Invest Ophthalmol Vis Sci. 2008; 49(3): 877-81	Nonfatal Health Outcomes	
Sun G, Xu Z-M, Liang J-F, Li L, Tang D-X. Twelve-year prevalence of common neonatal congenital malformations in Zhejiang Province, China. World J Pediatr. 2011; 7(4): 331-6	Nonfatal Health Outcomes	Urogenital congenital anomalies
Sun G, Xu Z-M, Liang J-F, Li L, Tang D-X. Twelve-year prevalence of common neonatal congenital malformations in Zhejiang Province, China. World J Pediatr. 2011; 7(4): 331-6	Nonfatal Health Outcomes	Digestive congenital anomalies

Liu J, Zhang L, Li Z, Jin L, Zhang Y, Ye R, Liu J, Ren A. Prevalence and trend of neural tube defects in five counties in Shanxi province of Northern China, 2000 to 2014. Birth Defects Res A Clin Mol Teratol. 2016; 106(4): 267-74	Nonfatal Health Outcomes	Congenital birth defects
Leung CC, Yew WW, Au KF, Tam CM, Chang KC, Mak KY, Tam SY, Chan KF, Fong KL, Tam SW. A Strong Tuberculin Reaction in Primary School Children Predicts Tuberculosis in Adolescence:. Pediatr Infect Dis J. 2012; 31(2): 150Â–3	Nonfatal Health Outcomes	Latent tuberculosis infection
Sun G, Xu Z-M, Liang J-F, Li L, Tang D-X. Twelve-year prevalence of common neonatal congenital malformations in Zhejiang Province, China. World J Pediatr. 2011; 7(4): 331-6	Nonfatal Health Outcomes	Congenital heart anomalies
Wu X-H, Wang T-P, Lu D-B, Hu H-T, Gao Z-B, Zhu C-G, Fang G-R, He Y-C, Mei Q-J, Wu W-D, Ge J-H, Zheng J. Studies of impact on physical fitness and working capacity of patients with advanced Schistosomiasis japonica in Susong County, Anhui Province. Addict Behav Rep. 2002; 82(2): 247-52	Nonfatal Health Outcomes	Schistosomiasis
Xu X, Zhu F, OÂ'Campo P, Koenig MA, Mock V, Campbell J. Prevalence of and Risk Factors for Intimate Partner Violence in China. Am J Public Health. 2005; 95(1): 78Â–85	Nonfatal Health Outcomes	Sexual violence
Ma Z-W, Li M-X, Han Y, Wang Z-M, Niu J-L, Zhao L, Shi L, Wang X-Q, Li C-X, Wang W, Meng Q-W, Zhang X-D. Epidemiological investigation on mental disorders in Xinxiang city. Med J Chin Peoples Health. 2005; 17(9): 501-2	Nonfatal Health Outcomes	Alcohol use disorders
Zhang J-X, Weng Z, Qin Q-L, Chai X-S, Ma D-D. A comparison study of epidemiology between Type I and Type II schizophrenia in Shandong. Chin Ment Health J. 2001; 15(2): 100-102	Nonfatal Health Outcomes	Schizophrenia
Hong Kong Cancer Registry. Hong Kong Cancer Registry Annual Tables. Kowloon, Hong Kong: Hong Kong Cancer Registry	Nonfatal Health Outcomes	Multiple myeloma
Zhao JF, Yang P, Su W, Cui Z, Wang SM. Hepatitis C virus infection and hepatocellular carcinoma. J Bengbu Med Coll. 2000; 25: 169-170	Nonfatal Health Outcomes	Liver cancer due to hepatitis B
Zhao JF, Yang P, Su W, Cui Z, Wang SM. Hepatitis C virus infection and hepatocellular carcinoma. J Bengbu Med Coll. 2000; 25: 169-170	Nonfatal Health Outcomes	Liver cancer due to hepatitis C
Zhang M, Sun X-D, Mark SD, Chen W, Wong L, Dawsey SM, Qiao Y-L, Fraumeni JF Jr, Taylor PR, O'Brien TR. Hepatitis C virus infection, Linxian, China. Emerg Infect Dis. 2005; 11(1): 17-21	Nonfatal Health Outcomes	Acute hepatitis C
Sun G, Xu Z-M, Liang J-F, Li L, Tang D-X. Twelve-year prevalence of common neonatal congenital malformations in Zhejiang Province, China. World J Pediatr. 2011; 7(4): 331-6	Nonfatal Health Outcomes	Congenital musculoskeletal and limb anomalies
King CH, Dickman K, Tisch DJ. Reassessment of the cost of chronic helmintic infection: a meta-analysis of disability-related outcomes in endemic schistosomiasis. Lancet. 2005; 365(9470): 1561Â–9	Nonfatal Health Outcomes	Dietary iron deficiency
Li X, Zhu Z, Zhu J, Lu W, Zhu J. An Epidemiological Survey on schizophrenia in Yancheng of Jiangsu Province. Nerv Dis Mental Hyg. 2003; 3(5): 356-57	Nonfatal Health Outcomes	Schizophrenia
Zheng JS, Guan Y, Zhao Y, Zhao W, Tang X, Chen H, Xu M, Wu L, Zhu S, Liu H, Huang T, Li D. Pre-conceptional intake of folic acid supplements is inversely associated with risk of preterm birth and small-for-gestational-age birth: a prospective cohort study. Br J Nutr. 2016; 115(3): 509-16	Nonfatal Health Outcomes	Neonatal preterm birth
Zhou H, Guo Z, Yu L, Hu X, Xu B, Liu H, Wu M, Zhou Z. Evidence on the applicability of the ATPIII, IDF and CDS metabolic syndrome diagnostic criteria to identify CVD and T2DM in the Chinese population from a 6.3-year cohort study in mid-eastern China. Diabetes Res Clin Pract. 2010; 90(3): 319Â–25	Nonfatal Health Outcomes	Diabetes mellitus
Dai S, Shen Z, Zha Z, Leng R, Qin W, Wang C, Chen L, Tian M, Huang Z, Chen G, Cen H, Xue L, Wang J, Lu Y, Cao B, Ye D. Seroprevalence of HIV, syphilis, and hepatitis C virus in the general population of the Liangshan Prefecture, Sichuan Province, China. J Med Virol. 2012; 84(1): 1-5	Nonfatal Health Outcomes	Acute hepatitis C
Li Q, Li H, Qin Y, Wang PP, Hao X. Comparison of surgical outcomes for small hepatocellular carcinoma in patients with hepatitis B versus hepatitis C: A Chinese experience . J Gastroenterol Hepatol. 2007; 22(11): 1936-41	Nonfatal Health Outcomes	Liver cancer due to hepatitis B
Li Q, Li H, Qin Y, Wang PP, Hao X. Comparison of surgical outcomes for small hepatocellular carcinoma in patients with hepatitis B versus hepatitis C: A Chinese experience . J Gastroenterol Hepatol. 2007; 22(11): 1936-41	Nonfatal Health Outcomes	Liver cancer due to hepatitis C
Tu D, Liu Y, Zhang L. [Tuberculin reactivity criteria for chemoprophylaxis in college students]. J Chin Antituberc Assoc. 2006; 28(5): 265Â–8	Nonfatal Health Outcomes	Latent tuberculosis infection
Liu J, Zhang L, Li Z, Jin L, Zhang Y, Ye R, Liu J, Ren A. Prevalence and trend of neural tube defects in five counties in Shanxi province of Northern China, 2000 to 2014. Birth Defects Res A Clin Mol Teratol. 2016; 106(4): 267-74	Nonfatal Health Outcomes	Neural tube defects
Pan C, Yang W, Jia W, Weng J, Tian H. Management of Chinese patients with type 2 diabetes, 1998-2006: the Diabcare-China surveys. Curr Med Res Opin. 2009; 25(1): 39Â–45	Nonfatal Health Outcomes	Diabetes mellitus

Guo-Chuan Zhang, Bao-Long Z, Suzhen X. [A survey of prevalence of senile dementia and Parkinson disease]. J Clin Psychol Med. 2001; 11(Pt 3): 143-5	Nonfatal Health Outcomes	Alzheimer's disease and other dementias
Gilbert CE, Ellwein LB. Prevalence and causes of functional low vision in school-age children: results from standardized population surveys in Asia, Africa, and Latin America. Invest Ophthalmol Vis Sci. 2008; 49(3): 877-81	Nonfatal Health Outcomes	Refraction disorders
Gilbert CE, Ellwein LB. Prevalence and causes of functional low vision in school-age children: results from standardized population surveys in Asia, Africa, and Latin America. Invest Ophthalmol Vis Sci. 2008; 49(3): 877-81	Nonfatal Health Outcomes	Refraction and accommodation disorders
King CH, Dickman K, Tisch DJ. Reassessment of the cost of chronic helmintic infection: a meta-analysis of disability-related outcomes in endemic schistosomiasis. Lancet. 2005; 365(9470): 1561Â–9	Nonfatal Health Outcomes	Anemia
Tu D, Liu Y, Zhang L. [Tuberculin reactivity criteria for chemoprophylaxis in college students]. J Chin Antituberc Assoc. 2006; 28(5): 265Â–8	Nonfatal Health Outcomes	Drug-susceptible tuberculosis
Tu D, Liu Y, Zhang L. [Tuberculin reactivity criteria for chemoprophylaxis in college students]. J Chin Antituberc Assoc. 2006; 28(5): 265Â–8	Nonfatal Health Outcomes	Tuberculosis
Tu D, Liu Y, Zhang L. [Tuberculin reactivity criteria for chemoprophylaxis in college students]. J Chin Antituberc Assoc. 2006; 28(5): 265Â–8	Nonfatal Health Outcomes	HIV/AIDS - Drug-susceptible Tuberculosis
Tu D, Liu Y, Zhang L. [Tuberculin reactivity criteria for chemoprophylaxis in college students]. J Chin Antituberc Assoc. 2006; 28(5): 265Â–8	Nonfatal Health Outcomes	Extensively drug-resistant tuberculosis
Tu D, Liu Y, Zhang L. [Tuberculin reactivity criteria for chemoprophylaxis in college students]. J Chin Antituberc Assoc. 2006; 28(5): 265Â–8	Nonfatal Health Outcomes	Multidrug-resistant tuberculosis without extensive drug resistance
Tu D, Liu Y, Zhang L. [Tuberculin reactivity criteria for chemoprophylaxis in college students]. J Chin Antituberc Assoc. 2006; 28(5): 265Â–8	Nonfatal Health Outcomes	HIV/AIDS - Multidrug-resistant Tuberculosis without extensive drug resistance
Hu J-M, Li Z, Chen Y-H, Zhou X-M, M Y-H, Huang H-F, Yan H-R, Wang X-L, Guan L-Y, Wang W-B. Epidemiological investigation on mental disorders in Zhongshan city, Guangzhou Province. Chin J Nerv Ment Dis. 2002; 28(6): 456-458	Nonfatal Health Outcomes	Schizophrenia
Gao JD, Shao YF, Xu Y, Ming LH, Wu ZY, Liu GT, Wang XH, Gao WH, Sun YT, Feng XL, Liang LM, Zhang YH, Sun ZT. Tight association of hepatocellular carcinoma with HBV infection in North China. Hepatobiliary Pancreat Dis Int. 2005; 4(1): 46-9	Nonfatal Health Outcomes	Liver cancer due to hepatitis C
Yao Q, Zhang W, Qian J. Dialysis status in China: a report from the Shanghai Dialysis Registry (2000-2005). Ethn Dis. 2009; 19(1 Suppl 1): S23-6	Nonfatal Health Outcomes	Chronic kidney disease
Sun G, Xu Z-M, Liang J-F, Li L, Tang D-X. Twelve-year prevalence of common neonatal congenital malformations in Zhejiang Province, China. World J Pediatr. 2011; 7(4): 331-6	Nonfatal Health Outcomes	Orofacial clefts
Jiang H, Tai B, Du M, Peng B. Effect of professional application of APF foam on caries reduction in permanent first molars in 6-7-year-old children: 24-month clinical trial. J Dent. 2005; 33(6): 469-73	Nonfatal Health Outcomes	Caries of permanent teeth
Yang Y, Gao J, Li HL, Zheng W, Yang G, Zhang W, Ma X, Tan YT, Rothman N, Gao YT, Chow WH, Shu XO, Xiang YB. Dose-response association between hepatitis B surface antigen levels and liver cancer risk in Chinese men and women. Int J Cancer. 2016. [Epub ahead of print]	Nonfatal Health Outcomes	Liver cancer due to hepatitis B
Gilbert CE, Ellwein LB. Prevalence and causes of functional low vision in school-age children: results from standardized population surveys in Asia, Africa, and Latin America. Invest Ophthalmol Vis Sci. 2008; 49(3): 877-81	Nonfatal Health Outcomes	Vision loss
Huon GF, Mingyi Q, Oliver K, Xiao G. A large-scale survey of eating disorder symptomatology among female adolescents in the People's Republic of China. Int J Eat Disord. 2002; 32(2): 192-205	Nonfatal Health Outcomes	Bulimia nervosa
Clift A, Morgan C, Anderson D, Toole M. Alarming levels of hepatitis B virus detected among rural Tibetans. Trop Doct. 2004; 34(3): 156-7	Nonfatal Health Outcomes	Acute hepatitis B
London School of Hygiene and Tropical Medicine. Global Atlas of Helminth Infections - Soil Transmitted Helminths. London, United Kingdom: London School of Hygiene and Tropical Medicine	Nonfatal Health Outcomes	Ascariasis
Hu J-M, Li Z, Chen Y-H, Zhou X-M, M Y-H, Huang H-F, Yan H-R, Wang X-L, Guan L-Y, Wang W-B. Epidemiological investigation on mental disorders in Zhongshan city, Guangzhou Province. Chin J Nerv Ment Dis. 2002; 28(6): 456-458	Nonfatal Health Outcomes	Alcohol use disorders
London School of Hygiene and Tropical Medicine. Global Atlas of Helminth Infections - Soil Transmitted Helminths. London, United Kingdom: London School of Hygiene and Tropical Medicine	Nonfatal Health Outcomes	Trichuriasis
Ministry of Health (China), National Bureau of Statistics of China. China National Nutrition Survey 2002 - China CDC	Nonfatal Health Outcomes	Dietary iron deficiency
Chinese Center for Disease Control and Prevention (CCDC). China Disease Surveillance Points 2001 - China CDC	Nonfatal Health Outcomes	Rabies
Chinese Center for Disease Control and Prevention (CCDC). China Notifiable Infectious Diseases 2004-2012	Nonfatal Health Outcomes	Intestinal infectious diseases

Chinese Center for Disease Control and Prevention (CCDC). China Disease Surveillance Points 2002 - China CDC	Nonfatal Health Outcomes	Rabies
Du D-Y, Li M-W, Chen J-S. Epidemiological Investigation on ADHD among Children in Urban Area of Guangzhou. Guangdong Med J. 2003; 24(3): 299-300	Nonfatal Health Outcomes	Attention-deficit/hyperactivity disorder
World Health Organization (WHO). WHO Mortality Database Version October 2017. Geneva, Switzerland: World Health Organization (WHO), 2017	Nonfatal Health Outcomes	HIV/AIDS
Ministry of Health (China), National Bureau of Statistics of China. China National Nutrition Survey 2002 - China CDC	Nonfatal Health Outcomes	Anemia
Chinese Center for Disease Control and Prevention (CCDC). China Disease Surveillance Points 2004 - China CDC	Nonfatal Health Outcomes	Rabies
Chinese Center for Disease Control and Prevention (CCDC). China Notifiable Infectious Diseases 2004	Nonfatal Health Outcomes	Rabies
Chinese Center for Disease Control and Prevention (CCDC). China Notifiable Infectious Diseases 2004-2012	Nonfatal Health Outcomes	Paratyphoid fever
Chinese Center for Disease Control and Prevention (CCDC). China Notifiable Infectious Diseases 2004-2012	Nonfatal Health Outcomes	Typhoid fever
Chen XS, Yin YP, Chen LP, Thuy NT, Zhang GY, Shi MQ, Hu LH, Yu YH. Sexually transmitted infections among pregnant women attending an antenatal clinic in Fuzhou, China. Sex Transm Dis. 2006; 33(5): 296-301	Nonfatal Health Outcomes	Sexually transmitted infections excluding HIV
Wu Z, Rotheram-Borus MJ, Li L, Guan J, Detels R, Yin Y, Wu S, Liu Z, Lin C, Hsieh J, NIMH Collaborative HIV/STD Prevention Trial Group. Sexually transmitted diseases and risk behaviors among market vendors in China. Sex Transm Dis. 2007; 34(12): 1030-4	Nonfatal Health Outcomes	Sexually transmitted infections excluding HIV
Odhiambo JA, Williams HC, Clayton TO, Robertson CF, Asher MI, ISAAC Phase Three Study Group. Global variations in prevalence of eczema symptoms in children from ISAAC Phase Three. J Allergy Clin Immunol. 2009; 124(6): 1251-1258	Nonfatal Health Outcomes	Atopic dermatitis
World Health Organization (WHO). China World Health Survey 2002. Geneva, Switzerland: World Health Organization (WHO), 2005	Nonfatal Health Outcomes	Vascular intestinal disorders
Institute of Social Medicine and Health Policy, Shandong University, Shandong University School of Medicine, World Health Organization (WHO). China WHO Multi-country Survey Study on Health and Health System Responsiveness 2000-2001	Nonfatal Health Outcomes	Vision loss
Institute of Social Medicine and Health Policy, Shandong University, Shandong University School of Medicine, World Health Organization (WHO). China WHO Multi-country Survey Study on Health and Health System Responsiveness 2000-2001	Nonfatal Health Outcomes	Venomous animal contact
United Nations Population Division. World Abortion Policies 2007. New York, United States: United Nations (UN), 2007	Nonfatal Health Outcomes	Ectopic pregnancy
United Nations Population Division. World Abortion Policies 2007. New York, United States: United Nations (UN), 2007	Nonfatal Health Outcomes	Maternal abortion and miscarriage
Forman D, Bray F, Brewster DH, Gombe Mbalawa C, Kohler B, PiÃ±eros M, Steliarova-Foucher E, Swaminathan R and Ferlay J, eds (2013). Cancer Incidence in Five Continents, Vol. X Summary Database (electronic version). Lyon, IARC. http://ci5.iarc.fr	Nonfatal Health Outcomes	Non-melanoma skin cancer (squamous-cell carcinoma)
Institute of Social Medicine and Health Policy, Shandong University, Shandong University School of Medicine, World Health Organization (WHO). China WHO Multi-country Survey Study on Health and Health System Responsiveness 2000-2001	Nonfatal Health Outcomes	Inguinal, femoral, and abdominal hernia
Wang WZ, Wu JZ, Wang DS, Dai XY, Yang B, Wang TP, Yuan CL, Scott RA, Prilipko LL, de Boer HM, Sander JW. The prevalence and treatment gap in epilepsy in China: an ILAE/IBE/WHO study. Neurology. 2003; 60(9): 1544Â–5	Nonfatal Health Outcomes	Epilepsy
Institute of Social Medicine and Health Policy, Shandong University, Shandong University School of Medicine, World Health Organization (WHO). China WHO Multi-country Survey Study on Health and Health System Responsiveness 2000-2001	Nonfatal Health Outcomes	Interpersonal violence
Tian F, Yang D, Zhang X, Zhao Q, Zhan X, Li R, Mu L, Jiang X, Li J. Survey of Health Status and Healthcare Service Demand of Perimenopausal Women in Guangdong. J First Mil Med Univ. 2004; 24(8): 928-32	Nonfatal Health Outcomes	Uterine fibroids
Institute of Social Medicine and Health Policy, Shandong University, Shandong University School of Medicine, World Health Organization (WHO). China WHO Multi-country Survey Study on Health and Health System Responsiveness 2000-2001	Nonfatal Health Outcomes	Congenital musculoskeletal and limb anomalies
Institute of Social Medicine and Health Policy, Shandong University, Shandong University School of Medicine, World Health Organization (WHO). China WHO Multi-country Survey Study on Health and Health System Responsiveness 2000-2001	Nonfatal Health Outcomes	Drowning

Zhang J-X, Lu C-H, Tang J-S, Qiu H-M, Liu L-F, Wang S-B, Wang A-Z, Zhang T-L, Phillips M-R, Li X-Y, Zhang S-D, Jiang Y-H, Zhao C-Y, Hu B-W, Cao X-Y, Zhang Y, Gao C-N. Epidemiological survey of mental disorders in persons aged 18 years and older in Shandong Province. Chin Ment Health J. 2010; 24(3): 161-167	Nonfatal Health Outcomes	Anxiety disorders
Xiang R, Zhang Z, Yu B, Shan X, Deng F, Xu X, Fang R, Liu Zhi-Shuang null, Zhang H, Wang H. [Effect of comprehensive schistosomiasis control strategy based on infection source control in Hanchuan City]. Chin J Schistosomiasis Control. 2014; 26(6): 658Â–61	Nonfatal Health Outcomes	Schistosomiasis
Wei G. The Epidemiology Survey and Study of Preventative and Curative Strategies on Mental Disorders at Tibet in China [dissertation]. [Chengdu]: Sichuan University; 2004. 91 p	Nonfatal Health Outcomes	Epilepsy
Chinese Center for Disease Control and Prevention (CCDC). China Notifiable Infectious Diseases 2004	Nonfatal Health Outcomes	Syphilis
United Nations Population Division. World Abortion Policies 2007. New York, United States: United Nations (UN), 2007	Nonfatal Health Outcomes	None
He M, Zeng J, Liu Y, Xu J, Pokharel GP, Ellwein LB. Refractive error and visual impairment in urban children in southern China. Invest Ophthalmol Vis Sci. 2004; 45(3): 793-9	Nonfatal Health Outcomes	Refraction and accommodation disorders
Huang S, Zheng Y, Foster PJ, Huang W, He M. Prevalence and causes of visual impairment in Chinese adults in urban southern China. Arch Ophthalmol. 2009; 127(10): 1362-7	Nonfatal Health Outcomes	Refraction and accommodation disorders
Institute of Social Medicine and Health Policy, Shandong University, Shandong University School of Medicine, World Health Organization (WHO). China WHO Multi-country Survey Study on Health and Health System Responsiveness 2000-2001	Nonfatal Health Outcomes	Turner syndrome
Chinese Center for Disease Control and Prevention (CCDC). China National Tuberculosis Prevalence Survey 2000	Nonfatal Health Outcomes	HIV/AIDS - Extensively drug-resistant Tuberculosis
International Clearinghouse for Birth Defects Surveillance and Research. International Clearinghouse for Birth Defects Surveillance and Research Annual Report 2006. Rome, Italy: International Clearinghouse for Birth Defects Surveillance and Research, 2007	Nonfatal Health Outcomes	Congenital heart anomalies
Institute of Social Medicine and Health Policy, Shandong University, Shandong University School of Medicine, World Health Organization (WHO). China WHO Multi-country Survey Study on Health and Health System Responsiveness 2000-2001	Nonfatal Health Outcomes	Genital prolapse
International Clearinghouse for Birth Defects Monitoring Systems. International Clearinghouse for Birth Defects Monitoring Systems Annual Report 2003. Rome, Italy: International Centre on Birth Defects	Nonfatal Health Outcomes	Congenital birth defects
World Health Organization (WHO). WHO World Mental Health Survey Prevalence of Alcohol Use, Abuse, and Dependence Tabulations	Nonfatal Health Outcomes	Alcohol use disorders
World Health Organization Regional Office for the Western Pacific (WPRO-WHO). Number of Reported Cases of Dengue Fever and Dengue Hemorrhagic Fever in the Western Pacific Region, by Country 2004. Manila, Philippines: World Health Organization Regional Office for the Western Pacific (WPRO-WHO)	Nonfatal Health Outcomes	Dengue
World Health Organization (WHO). China World Health Survey 2002. Geneva, Switzerland: World Health Organization (WHO), 2005	Nonfatal Health Outcomes	Peptic ulcer disease
World Health Organization (WHO). China World Health Survey 2002. Geneva, Switzerland: World Health Organization (WHO), 2005	Nonfatal Health Outcomes	Pulmonary aspiration and foreign body in airway
Institute of Social Medicine and Health Policy, Shandong University, Shandong University School of Medicine, World Health Organization (WHO). China WHO Multi-country Survey Study on Health and Health System Responsiveness 2000-2001	Nonfatal Health Outcomes	Klinefelter syndrome
Jia L. ZD Luo H., Zhou C.,Wang L., Ouyang X. et al. An epidemiological investigation of alcohol dependence in Guiyang, Anshun and Bijie of Guizhou province. Chin J Mod Drug Appl. 2009; 209Â–10	Nonfatal Health Outcomes	Alcohol use disorders
Garcia-Marcos L, Robertson CF, Ross Anderson H, Ellwood P, Williams HC, Wong GW. Does migration affect asthma, rhinoconjunctivitis and eczema prevalence? Global findings from the international study of asthma and allergies in childhood. Int J Epidemiol. 2014; 43(6): 1846-54	Nonfatal Health Outcomes	Dermatitis
Buist AS,Â McBurnie MA,Â Vollmer WM,Â Gillespie S,Â Burney P,Â Mannino DM,Â Menezes AM,Â Sullivan SD,Â Lee TA,Â Weiss KB,Â Jensen RL,Â Marks GB,Â Gulsvik A,Nizankowska-Mogilnicka E, BOLD Collaborative Research Group. International variation in the prevalence of COPD (the BOLD Study): a population-based prevalence study. Lancet. 2007; 9589(9589): 741-50	Nonfatal Health Outcomes	Chronic obstructive pulmonary disease

Xu Z-G, He Z-W, Wang Y-B, Tu Z-W, Xiong B. [Schistosomiasis endemic situation at a national surveillance site in GongÂ'an County, Hubei Province from 2004 to 2013]. Chin J Schistosomiasis Control. 2014; 26(5): 588Â–90	Nonfatal Health Outcomes	Schistosomiasis
Zhimin W, Yubao Z, Lei S, Xianliang Z, Wei Z, Li S, Hao W, Jianjun L, Detrano R, Rutai H. Prevalence of chronic rheumatic heart disease in Chinese adults. Int J Cardiol. 2006; 107(3): 356-9	Nonfatal Health Outcomes	Rheumatic heart disease
Institute of Social Medicine and Health Policy, Shandong University, Shandong University School of Medicine, World Health Organization (WHO). China WHO Multi-country Survey Study on Health and Health System Responsiveness 2000-2001	Nonfatal Health Outcomes	Vascular intestinal disorders
Chinese Center for Disease Control and Prevention (CCDC). China Notifiable Infectious Diseases 2004-2012	Nonfatal Health Outcomes	Dengue
Huang S, Zheng Y, Foster PJ, Huang W, He M. Prevalence and causes of visual impairment in Chinese adults in urban southern China. Arch Ophthalmol. 2009; 127(10): 1362-7	Nonfatal Health Outcomes	Vision loss
He M, Huang W, Zheng Y, Huang L, Ellwein LB. Refractive error and visual impairment in school children in rural southern China. Ophthalmology. 2007; 114(2): 374-82	Nonfatal Health Outcomes	Vision loss
He M, Zeng J, Liu Y, Xu J, Pokharel GP, Ellwein LB. Refractive error and visual impairment in urban children in southern China. Invest Ophthalmol Vis Sci. 2004; 45(3): 793-9	Nonfatal Health Outcomes	Vision loss
Institute of Social Medicine and Health Policy, Shandong University, Shandong University School of Medicine, World Health Organization (WHO). China WHO Multi-country Survey Study on Health and Health System Responsiveness 2000-2001	Nonfatal Health Outcomes	Motorcyclist road injuries
Institute of Social Medicine and Health Policy, Shandong University, Shandong University School of Medicine, World Health Organization (WHO). China WHO Multi-country Survey Study on Health and Health System Responsiveness 2000-2001	Nonfatal Health Outcomes	Diarrheal diseases
Hong Kong Cancer Registry. Hong Kong Cancer Registry Annual Tables. Kowloon, Hong Kong: Hong Kong Cancer Registry	Nonfatal Health Outcomes	Colon and rectum cancer
Chen X-S, Yin Y-P, Chen L-P, Yu Y-H, Wei W-H, Thuy NTT, Smith JS. Herpes simplex virus 2 infection in women attending an antenatal clinic in Fuzhou, China. Sex Transm Infect. 2007; 83(5): 369Â–70	Nonfatal Health Outcomes	Genital herpes
National Central Cancer Registry (China). China Cancer Registry Annual Report 2003-2007. Beijing, China: Military Medical Sciences Press (China)	Nonfatal Health Outcomes	Non-melanoma skin cancer (squamous-cell carcinoma)
Huang L-C, Zheng S-M, Luo W-Y, Chen J-Y. Analysis on the Epidemic Condition about Psychosis Disease of the Local People in Huojie Town Dongguan City. Int Med Health Guid News. 2006; 12(18): 128-30	Nonfatal Health Outcomes	Schizophrenia
World Health Organization (WHO). China World Health Survey 2002. Geneva, Switzerland: World Health Organization (WHO), 2005	Nonfatal Health Outcomes	Vision loss
Hong Kong Cancer Registry. Hong Kong Cancer Registry Annual Tables. Kowloon, Hong Kong: Hong Kong Cancer Registry	Nonfatal Health Outcomes	Esophageal cancer
Hong Kong Cancer Registry. Hong Kong Cancer Registry Annual Tables. Kowloon, Hong Kong: Hong Kong Cancer Registry	Nonfatal Health Outcomes	Stomach cancer
Hong Kong Cancer Registry. Hong Kong Cancer Registry Annual Tables. Kowloon, Hong Kong: Hong Kong Cancer Registry	Nonfatal Health Outcomes	Liver cancer
Hong Kong Cancer Registry. Hong Kong Cancer Registry Annual Tables. Kowloon, Hong Kong: Hong Kong Cancer Registry	Nonfatal Health Outcomes	Tracheal, bronchus, and lung cancer
Hong Kong Cancer Registry. Hong Kong Cancer Registry Annual Tables. Kowloon, Hong Kong: Hong Kong Cancer Registry	Nonfatal Health Outcomes	Breast cancer
Hong Kong Cancer Registry. Hong Kong Cancer Registry Annual Tables. Kowloon, Hong Kong: Hong Kong Cancer Registry	Nonfatal Health Outcomes	Uterine cancer
Gao W, Liu X, Yan H. Prevalence of Diarrhea among Children Less Than 36 Months of Age in Rural Western China in 2001 and 2005. Am J Trop Med Hyg. 2014; 91(6): 1197	Nonfatal Health Outcomes	Diarrheal diseases
Institute of Social Medicine and Health Policy, Shandong University, Shandong University School of Medicine, World Health Organization (WHO). China WHO Multi-country Survey Study on Health and Health System Responsiveness 2000-2001	Nonfatal Health Outcomes	Pulmonary aspiration and foreign body in airway
Food and Agriculture Organization of the United Nations (FAO), World Health Organization (WHO). Joint WHO/FAO Workshop on Foodborne Trematode Infections in Asia. Manila, Philippines: World Health Organization Regional Office for the Western Pacific (WPRO-WHO), 2004	Nonfatal Health Outcomes	Food-borne trematodiases

Chinese Center for Disease Control and Prevention (CCDC). China National Survey on Current Status of the Important Parasitic Diseases in the Human Population 2001-2004	Nonfatal Health Outcomes	Food-borne trematodiases
Institute of Social Medicine and Health Policy, Shandong University, Shandong University School of Medicine, World Health Organization (WHO). China WHO Multi-country Survey Study on Health and Health System Responsiveness 2000-2001	Nonfatal Health Outcomes	Other pneumoconiosis
Lu Q, Zheng Y, Sun B, Cui T, Congdon N, Hu A, Chen J, Shi J. A population-based study of visual impairment among pre-school children in Beijing: the Beijing study of visual impairment in children. Am J Ophthalmol. 2009; 147(6): 1075-81	Nonfatal Health Outcomes	Refraction and accommodation disorders
London School of Hygiene and Tropical Medicine. Global Atlas of Helminth Infections - Schistosomiasis. London, United Kingdom: London School of Hygiene and Tropical Medicine	Nonfatal Health Outcomes	Schistosomiasis
World Health Organization (WHO). China World Health Survey 2002. Geneva, Switzerland: World Health Organization (WHO), 2005	Nonfatal Health Outcomes	Lower respiratory infections
World Health Organization (WHO). China World Health Survey 2002. Geneva, Switzerland: World Health Organization (WHO), 2005	Nonfatal Health Outcomes	Foreign body in other body part
Li ZY, Xu GB, Xia TA, Wang HY. Prevalence of chronic kidney disease in a middle and old-aged population of Beijing. Clin Chim Acta. 2006; 366(1-2): 209-15	Nonfatal Health Outcomes	Chronic kidney disease
World Health Organization (WHO). China World Health Survey 2002. Geneva, Switzerland: World Health Organization (WHO), 2005	Nonfatal Health Outcomes	Decubitus ulcer
Cui L, Li K, Cui Z, Jiang Q, Gao L, Zhang Y, Li J. Prevalence, demographic characteristics and function status of the schizophrenia in Hebei province. Chin J Nerv Ment Dis. 2007; 33(3)	Nonfatal Health Outcomes	Schizophrenia
Institute of Social Medicine and Health Policy, Shandong University, Shandong University School of Medicine, World Health Organization (WHO). China WHO Multi-country Survey Study on Health and Health System Responsiveness 2000-2001	Nonfatal Health Outcomes	Silicosis
World Health Organization (WHO). China World Health Survey 2002. Geneva, Switzerland: World Health Organization (WHO), 2005	Nonfatal Health Outcomes	None
Li L, Guan H, Xun P, Zhou J, Gu H. Prevalence and causes of visual impairment among the elderly in Nantong, China. Eye (Lond). 2008; 22(8): 1069-75	Nonfatal Health Outcomes	
World Health Organization (WHO). China World Health Survey 2002. Geneva, Switzerland: World Health Organization (WHO), 2005	Nonfatal Health Outcomes	Acute glomerulonephritis
World Health Organization (WHO). China World Health Survey 2002. Geneva, Switzerland: World Health Organization (WHO), 2005	Nonfatal Health Outcomes	Endocrine, metabolic, blood, and immune disorders
World Health Organization (WHO). China World Health Survey 2002. Geneva, Switzerland: World Health Organization (WHO), 2005	Nonfatal Health Outcomes	Interpersonal violence
World Health Organization (WHO). China World Health Survey 2002. Geneva, Switzerland: World Health Organization (WHO), 2005	Nonfatal Health Outcomes	Unintentional suffocation
World Health Organization (WHO). China World Health Survey 2002. Geneva, Switzerland: World Health Organization (WHO), 2005	Nonfatal Health Outcomes	Polycystic ovarian syndrome
World Health Organization (WHO). China World Health Survey 2002. Geneva, Switzerland: World Health Organization (WHO), 2005	Nonfatal Health Outcomes	Maternal sepsis and other maternal infections
World Health Organization (WHO). China World Health Survey 2002. Geneva, Switzerland: World Health Organization (WHO), 2005	Nonfatal Health Outcomes	Physical violence by sharp object
World Health Organization (WHO). China World Health Survey 2002. Geneva, Switzerland: World Health Organization (WHO), 2005	Nonfatal Health Outcomes	Congenital musculoskeletal and limb anomalies
Institute of Social Medicine and Health Policy, Shandong University, Shandong University School of Medicine, World Health Organization (WHO). China WHO Multi-country Survey Study on Health and Health System Responsiveness 2000-2001	Nonfatal Health Outcomes	Neural tube defects
World Health Organization (WHO). China World Health Survey 2002. Geneva, Switzerland: World Health Organization (WHO), 2005	Nonfatal Health Outcomes	Encephalitis
World Health Organization (WHO). China World Health Survey 2002. Geneva, Switzerland: World Health Organization (WHO), 2005	Nonfatal Health Outcomes	Interstitial lung disease and pulmonary sarcoidosis
Zhang S-J, Jiang C, Wang P, Liu M-J, Liu H, Yu C-Y, Hu Z-G, Yu F-Q, Yang X-L, N J, Li N, Yan T-M, Pan G-W. Epidemiological Survey of Mood Disorders Among Urban and Rural Residents in Liaoning Province. Chin J Prev Control Chronic Dis. 2008; 16(4): 378-81	Nonfatal Health Outcomes	Major depressive disorder
World Health Organization (WHO). China World Health Survey 2002. Geneva, Switzerland: World Health Organization (WHO), 2005	Nonfatal Health Outcomes	Other transport injuries
Ai Z-Y. [Effect of schistosomiasis comprehensive control in Linxiang City, 2004-2012]. Chin J Schistosomiasis Control. 2013; 25(3): 296Â–8, 306	Nonfatal Health Outcomes	Schistosomiasis

Chiu SS, Ho P-L, Khong P-L, Ooi C, So LY, Wong WHS, Chan ELY. Population-based incidence of community-acquired pneumonia hospitalization in Hong Kong children younger than 5 years before universal conjugate pneumococcal immunization. J Microbiol Immunol Infect. 2016; 49(2): 225Â–9	Nonfatal Health Outcomes	Lower respiratory infections
Hon KL, Leung E, Hung E, Tang J, Chow CM, Leung TF, Cheung KL, Ng PC. Premorbid factors and outcome associated with respiratory virus infections in a pediatric intensive care unit. Pediatr Pulmonol. 2008; 43(3): 275-80	Nonfatal Health Outcomes	Influenza
Hon KL, Leung E, Hung E, Tang J, Chow CM, Leung TF, Cheung KL, Ng PC. Premorbid factors and outcome associated with respiratory virus infections in a pediatric intensive care unit. Pediatr Pulmonol. 2008; 43(3): 275-80	Nonfatal Health Outcomes	Respiratory syncytial virus pneumonia
World Health Organization (WHO). China World Health Survey 2002. Geneva, Switzerland: World Health Organization (WHO), 2005	Nonfatal Health Outcomes	Motor vehicle road injuries
World Health Organization (WHO). China World Health Survey 2002. Geneva, Switzerland: World Health Organization (WHO), 2005	Nonfatal Health Outcomes	Diarrheal diseases
World Health Organization (WHO). China World Health Survey 2002. Geneva, Switzerland: World Health Organization (WHO), 2005	Nonfatal Health Outcomes	Paralytic ileus and intestinal obstruction
Institute of Social Medicine and Health Policy, Shandong University, Shandong University School of Medicine, World Health Organization (WHO). China WHO Multi-country Survey Study on Health and Health System Responsiveness 2000-2001	Nonfatal Health Outcomes	Pedestrian road injuries
World Health Organization (WHO). China World Health Survey 2002. Geneva, Switzerland: World Health Organization (WHO), 2005	Nonfatal Health Outcomes	Exposure to mechanical forces
Cheng JQ, Zhou H, Hong FC, Zhang D, Zhang YJ, Pan P, Cai YM. Syphilis screening and intervention in 500,000 pregnant women in Shenzhen, the PeopleÂ's Republic of China. Sex Transm Infect. 2007; 83(5): 347Â–50	Nonfatal Health Outcomes	Syphilis
World Health Organization Regional Office for the Western Pacific (WPRO-WHO). Number of Reported Cases of Dengue Fever and Dengue Hemorrhagic Fever in the Western Pacific Region, by Country 2001. Manila, Philippines: World Health Organization Regional Office for the Western Pacific (WPRO-WHO)	Nonfatal Health Outcomes	Dengue
World Health Organization Regional Office for the Western Pacific (WPRO-WHO). Number of Reported Cases of Dengue Fever and Dengue Hemorrhagic Fever in the Western Pacific Region, by Country 2002. Manila, Philippines: World Health Organization Regional Office for the Western Pacific (WPRO-WHO)	Nonfatal Health Outcomes	Dengue
World Health Organization Regional Office for the Western Pacific (WPRO-WHO). Number of Reported Cases of Dengue Fever and Dengue Hemorrhagic Fever in the Western Pacific Region, by Country 2003. Manila, Philippines: World Health Organization Regional Office for the Western Pacific (WPRO-WHO)	Nonfatal Health Outcomes	Dengue
World Health Organization Regional Office for the Western Pacific (WPRO-WHO). Number of Reported Cases of Dengue Fever and Dengue Hemorrhagic Fever in the Western Pacific Region, by Country 2005. Manila, Philippines: World Health Organization Regional Office for the Western Pacific (WPRO-WHO)	Nonfatal Health Outcomes	Dengue
Phillips MR, Zhang J, Shi Q, Song Z, Ding Z, Pang S, Li X, Zhang Y, Wang Z. Prevalence, treatment, and associated disability of mental disorders in four provinces in China during 2001-05: an epidemiological survey. Lancet. 2009; 373(9680): 2041-53	Nonfatal Health Outcomes	Major depressive disorder
World Health Organization (WHO). China World Health Survey 2002. Geneva, Switzerland: World Health Organization (WHO), 2005	Nonfatal Health Outcomes	Cutaneous and mucocutaneous leishmaniasis
International Clearinghouse for Birth Defects Surveillance and Research. International Clearinghouse for Birth Defects Surveillance and Research Annual Report 2005. Rome, Italy: International Clearinghouse for Birth Defects Surveillance and Research, 2007	Nonfatal Health Outcomes	Congenital birth defects
World Health Organization (WHO). China World Health Survey 2002. Geneva, Switzerland: World Health Organization (WHO), 2005	Nonfatal Health Outcomes	Pancreatitis
Institute of Social Medicine and Health Policy, Shandong University, Shandong University School of Medicine, World Health Organization (WHO). China WHO Multi-country Survey Study on Health and Health System Responsiveness 2000-2001	Nonfatal Health Outcomes	Ischemic stroke
Phillips MR, Zhang J, Shi Q, Song Z, Ding Z, Pang S, Li X, Zhang Y, Wang Z. Prevalence, treatment, and associated disability of mental disorders in four provinces in China during 2001-05: an epidemiological survey. Lancet. 2009; 373(9680): 2041-53	Nonfatal Health Outcomes	Dysthymia
Phillips MR, Zhang J, Shi Q, Song Z, Ding Z, Pang S, Li X, Zhang Y, Wang Z. Prevalence, treatment, and associated disability of mental disorders in four provinces in China during 2001-05: an epidemiological survey. Lancet. 2009; 373(9680): 2041-53	Nonfatal Health Outcomes	Bipolar disorder
World Health Organization (WHO). Global leprosy situation, 2004. Wkly Epidemiol Rec. 2005: 80(13): 118-24	Nonfatal Health Outcomes	Leprosy
World Health Organization (WHO). Global leprosy situation, 2005. Wkly Epidemiol Rec. 2005: 80(34): 289-95.	Nonfatal Health Outcomes	Leprosy

World Health Organization (WHO). China World Health Survey 2002. Geneva, Switzerland: World Health Organization (WHO), 2005	Nonfatal Health Outcomes	Road injuries
Kong F-Y, Tao W-D, Hao Z-L, Liu M. Predictors of one-year disability and death in Chinese hospitalized women after ischemic stroke. Cerebrovasc Dis. 2010; 29(3): 255Â–62	Nonfatal Health Outcomes	Ischemic stroke
World Health Organization (WHO). China World Health Survey 2002. Geneva, Switzerland: World Health Organization (WHO), 2005	Nonfatal Health Outcomes	Animal contact
World Health Organization (WHO). China World Health Survey 2002. Geneva, Switzerland: World Health Organization (WHO), 2005	Nonfatal Health Outcomes	Asbestosis
Institute of Social Medicine and Health Policy, Shandong University, Shandong University School of Medicine, World Health Organization (WHO). China WHO Multi-country Survey Study on Health and Health System Responsiveness 2000-2001	Nonfatal Health Outcomes	Self-harm by firearm
World Health Organization (WHO). China World Health Survey 2002. Geneva, Switzerland: World Health Organization (WHO), 2005	Nonfatal Health Outcomes	Klinefelter syndrome
World Health Organization (WHO). China World Health Survey 2002. Geneva, Switzerland: World Health Organization (WHO), 2005	Nonfatal Health Outcomes	Self-harm by firearm
World Health Organization (WHO). China World Health Survey 2002. Geneva, Switzerland: World Health Organization (WHO), 2005	Nonfatal Health Outcomes	Maternal hypertensive disorders
World Health Organization (WHO). China World Health Survey 2002. Geneva, Switzerland: World Health Organization (WHO), 2005	Nonfatal Health Outcomes	Neonatal encephalopathy due to birth asphyxia and trauma
Long MJ, Jiang CQ, Lam TH, Xu L, Zhang WS, Lin JM, Ou JP, Cheng KK. Atrial fibrillation and obesity among older Chinese: the Guangzhou Biobank Cohort Study. Int J Cardiol. 2011; 148(1): 48Â–52	Nonfatal Health Outcomes	Atrial fibrillation and flutter
World Health Organization (WHO). China World Health Survey 2002. Geneva, Switzerland: World Health Organization (WHO), 2005	Nonfatal Health Outcomes	Orofacial clefts
Institute of Social Medicine and Health Policy, Shandong University, Shandong University School of Medicine, World Health Organization (WHO). China WHO Multi-country Survey Study on Health and Health System Responsiveness 2000-2001	Nonfatal Health Outcomes	Ischemic heart disease
International Clearinghouse for Birth Defects Monitoring Systems. International Clearinghouse for Birth Defects Monitoring Systems Annual Report 2004. Rome, Italy: International Centre on Birth Defects, 2006	Nonfatal Health Outcomes	Neural tube defects
World Health Organization (WHO). China World Health Survey 2002. Geneva, Switzerland: World Health Organization (WHO), 2005	Nonfatal Health Outcomes	Other unintentional injuries
World Health Organization (WHO). China World Health Survey 2002. Geneva, Switzerland: World Health Organization (WHO), 2005	Nonfatal Health Outcomes	Physical violence by firearm
World Health Organization (WHO). China World Health Survey 2002. Geneva, Switzerland: World Health Organization (WHO), 2005	Nonfatal Health Outcomes	Rheumatic heart disease
Forman D, Bray F, Brewster DH, Gombe Mbalawa C, Kohler B, PiÃ±eros M, Steliarova-Foucher E, Swaminathan R and Ferlay J, eds (2013). Cancer Incidence in Five Continents, Vol. X Summary Database (electronic version). Lyon, IARC. http://ci5.iarc.fr	Nonfatal Health Outcomes	Non-melanoma skin cancer (basal-cell carcinoma)
World Health Organization (WHO). China World Health Survey 2002. Geneva, Switzerland: World Health Organization (WHO), 2005	Nonfatal Health Outcomes	Coal workers pneumoconiosis
Du M, Luo Y, Zeng X, Alkhatib N, Bedi R. Caries in preschool children and its risk factors in 2 provinces in China. Quintessence Int. 2007; 38(2): 143-51	Nonfatal Health Outcomes	Caries of deciduous teeth
Institute of Social Medicine and Health Policy, Shandong University, Shandong University School of Medicine, World Health Organization (WHO). China WHO Multi-country Survey Study on Health and Health System Responsiveness 2000-2001	Nonfatal Health Outcomes	Inflammatory bowel disease
Institute of Social Medicine and Health Policy, Shandong University, Shandong University School of Medicine, World Health Organization (WHO). China WHO Multi-country Survey Study on Health and Health System Responsiveness 2000-2001	Nonfatal Health Outcomes	Poisonings
World Health Organization (WHO). China World Health Survey 2002. Geneva, Switzerland: World Health Organization (WHO), 2005	Nonfatal Health Outcomes	Drowning
Chinese Center for Disease Control and Prevention (CCDC). China Notifiable Infectious Diseases 2004-2012	Nonfatal Health Outcomes	Visceral leishmaniasis
Zhou H, Ohtsuka R, He Y, Yuan L, Yamauchi T, Sleigh AC. Impact of Parasitic Infections and Dietary Intake on Child Growth in the Schistosomiasis-Endemic Dongting Lake Region, China. Am J Trop Med Hyg. 2005; 72(5): 534-9	Nonfatal Health Outcomes	Schistosomiasis
Institute of Social Medicine and Health Policy, Shandong University, Shandong University School of Medicine, World Health Organization (WHO). China WHO Multi-country Survey Study on Health and Health System Responsiveness 2000-2001	Nonfatal Health Outcomes	Neonatal sepsis and other neonatal infections

Ko FW,Â Woo J,Â Tam W,Â Lai CK,Â Ngai J,Â Kwok T,Â Hui DS. Prevalence and risk factors of airflow obstruction in an elderly Chinese population. Eur Respir J. 2008; 32(6): 1472-8	Nonfatal Health Outcomes	Chronic obstructive pulmonary disease
Lai CK, Beasley R, Crane J, Foliaki S, Shah J, Weiland S. Global variation in the prevalence and severity of asthma symptoms: phase three of the International Study of Asthma and Allergies in Childhood (ISAAC). Thorax. 2009; 64(6): 476-483	Nonfatal Health Outcomes	Asthma
Fung KT, Fung J, Lai CL, Yuen MF. Etiologies of chronic liver diseases in Hong Kong. Eur J Gastroenterol Hepatol. 2007; 19(8): 659-64	Nonfatal Health Outcomes	Cirrhosis and other chronic liver diseases due to hepatitis B
Fung KT, Fung J, Lai CL, Yuen MF. Etiologies of chronic liver diseases in Hong Kong. Eur J Gastroenterol Hepatol. 2007; 19(8): 659-64	Nonfatal Health Outcomes	Cirrhosis and other chronic liver diseases due to hepatitis C
Fung KT, Fung J, Lai CL, Yuen MF. Etiologies of chronic liver diseases in Hong Kong. Eur J Gastroenterol Hepatol. 2007; 19(8): 659-64	Nonfatal Health Outcomes	Cirrhosis and other chronic liver diseases due to alcohol use
Fung KT, Fung J, Lai CL, Yuen MF. Etiologies of chronic liver diseases in Hong Kong. Eur J Gastroenterol Hepatol. 2007; 19(8): 659-64	Nonfatal Health Outcomes	Cirrhosis and other chronic liver diseases due to other causes
Institute of Social Medicine and Health Policy, Shandong University, Shandong University School of Medicine, World Health Organization (WHO). China WHO Multi-country Survey Study on Health and Health System Responsiveness 2000-2001	Nonfatal Health Outcomes	Road injuries
Phillips MR, Zhang J, Shi Q, Song Z, Ding Z, Pang S, Li X, Zhang Y, Wang Z. Prevalence, treatment, and associated disability of mental disorders in four provinces in China during 2001-05: an epidemiological survey. Lancet. 2009; 373(9680): 2041-53	Nonfatal Health Outcomes	Schizophrenia
Zhang Y, Feng X, Wu M, Xiong M, Shen M, Sun J. [Evaluation of effectiveness of comprehensive schistosomiasis control project in Yunnan Province from 2004 to 2013]. Chin J Schistosomiasis Control. 2015; 27(2): 115Â–8, 133	Nonfatal Health Outcomes	Schistosomiasis
Leung P, Hung S, Ho T, Lee C, Liu W, Tang C, Kwong S. Prevalence of DSM-IV disorders in Chinese adolescents and the effects of an impairment criterion: a pilot community study in Hong Kong. Eur Child Adolesc Psychiatry. 2008; 17(7): 452-61	Nonfatal Health Outcomes	Anxiety disorders
Odhiambo JA, Williams HC, Clayton TO, Robertson CF, Asher MI, ISAAC Phase Three Study Group. Global variations in prevalence of eczema symptoms in children from ISAAC Phase Three. J Allergy Clin Immunol. 2009; 124(6): 1251-1258	Nonfatal Health Outcomes	Dermatitis
Cheung BMY, Ong KL, Tso AWK, Leung RYH, Cherny SS, Sham PC, Thomas GN, Lam TH, Lam KSL, Investigators of the Hong Kong Cardiovascular Risk Factor Prevalence Study. Relationship of plasma interleukin-6 and its genetic variants with hypertension in Hong Kong Chinese. Am J Hypertens. 2011; 24(12): 1331Â–7	Nonfatal Health Outcomes	Diabetes mellitus
National Central Cancer Registry (China). China Cancer Registry Annual Report 2003-2007. Beijing, China: Military Medical Sciences Press (China)	Nonfatal Health Outcomes	Non-melanoma skin cancer (basal-cell carcinoma)
Chinese Center for Disease Control and Prevention (CCDC). China Disease Surveillance Points 2005 - China CDC	Nonfatal Health Outcomes	Rabies
Ying Lei. Epidemiology and drug treatment of epilepsy research in Quingtongxia, Ningxia. Shaanxi Med J. 2008; 37(8): 1068-70	Nonfatal Health Outcomes	Epilepsy
Au W-Y, Ma ESK, Lam VMS, Chan JLC, Pang A, Kwong Y-L. Glucose 6-phosphate dehydrogenase (G6PD) deficiency in elderly Chinese women heterozygous for G6PD variants. Am J Med Genet A. 2004; 129A(2): 208-11	Nonfatal Health Outcomes	G6PD deficiency
Woo J, Leung J, Lau E. Prevalence and correlates of musculoskeletal pain in Chinese elderly and the impact on 4-year physical function and quality of life. Public Health. 2009; 123(8): 549-56	Nonfatal Health Outcomes	Low back pain
Chiu TTW, Leung ASL. Neck pain in Hong Kong: a telephone survey on prevalence, consequences, and risk groups. Spine. 2006; 31(16): E540-544	Nonfatal Health Outcomes	Neck pain
Woo J, Leung J, Lau E. Prevalence and correlates of musculoskeletal pain in Chinese elderly and the impact on 4-year physical function and quality of life. Public Health. 2009; 123(8): 549-56	Nonfatal Health Outcomes	Neck pain
Garcia-Marcos L, Robertson CF, Ross Anderson H, Ellwood P, Williams HC, Wong GW. Does migration affect asthma, rhinoconjunctivitis and eczema prevalence? Global findings from the international study of asthma and allergies in childhood. Int J Epidemiol. 2014; 43(6): 1846-54	Nonfatal Health Outcomes	Atopic dermatitis
Zhang Q, MacDonald NE, Guo Z. Vaccine preventable community-acquired pneumonia in hospitalized children in Northwest China. Pediatr Infect Dis J. 2011; 30(1): 7-10	Nonfatal Health Outcomes	Lower respiratory infections
Fong GCY, Kwan P, Hui ACF, Lui CHT, Fong JKY, Wong V. An epidemiological study of epilepsy in Hong Kong SAR, China. Seizure. 2008; 17(5): 457-64	Nonfatal Health Outcomes	Epilepsy
Fong GCY, Mak W, Cheng TS, Chan KH, Fong JKY, Ho SL. A prevalence study of epilepsy in Hong Kong. Hong Kong Med J. 2003; 9(4): 252-7	Nonfatal Health Outcomes	Epilepsy
Institute of Social Medicine and Health Policy, Shandong University, Shandong University School of Medicine, World Health Organization (WHO). China WHO Multi-country Survey Study on Health and Health System Responsiveness 2000-2001	Nonfatal Health Outcomes	Appendicitis

Chinese Center for Disease Control and Prevention (CCDC). China Notifiable Infectious Diseases 2004	Nonfatal Health Outcomes	Chlamydial infection
Cheung T-K, Lai C-L, Wong BC-Y, Fung J, Yuen M-F. Clinical features, biochemical parameters, and virological profiles of patients with hepatocellular carcinoma in Hong Kong. Aliment Pharmacol Ther. 2006; 24(4): 573-83	Nonfatal Health Outcomes	Liver cancer due to hepatitis B
Cheung T-K, Lai C-L, Wong BC-Y, Fung J, Yuen M-F. Clinical features, biochemical parameters, and virological profiles of patients with hepatocellular carcinoma in Hong Kong. Aliment Pharmacol Ther. 2006; 24(4): 573-83	Nonfatal Health Outcomes	Liver cancer due to hepatitis C
Cheung T-K, Lai C-L, Wong BC-Y, Fung J, Yuen M-F. Clinical features, biochemical parameters, and virological profiles of patients with hepatocellular carcinoma in Hong Kong. Aliment Pharmacol Ther. 2006; 24(4): 573-83	Nonfatal Health Outcomes	Liver cancer due to alcohol use
Institute of Social Medicine and Health Policy, Shandong University, Shandong University School of Medicine, World Health Organization (WHO). China WHO Multi-country Survey Study on Health and Health System Responsiveness 2000-2001	Nonfatal Health Outcomes	Cellulitis
Zhong N,Â Wang C,Â Yao W,Â Chen P,Â Kang J,Â Huang S,Â Chen B,Â Wang C,Â Ni D,Â Zhou Y,Â Liu S,Â Wang X,Â Wang D,Â Lu J,Â Zheng J,Â Ran P. Prevalence of chronic obstructive pulmonary disease in China A large, population-based survey. Am J Respir Crit Care Med. 2007; 176(8): 753-60	Nonfatal Health Outcomes	Chronic obstructive pulmonary disease
Hong Kong Cancer Registry. Hong Kong Cancer Registry Annual Tables. Kowloon, Hong Kong: Hong Kong Cancer Registry	Nonfatal Health Outcomes	Non-melanoma skin cancer (squamous-cell carcinoma)
World Health Organization (WHO). China World Health Survey 2002. Geneva, Switzerland: World Health Organization (WHO), 2005	Nonfatal Health Outcomes	Cirrhosis and other chronic liver diseases
Wong SYS, Kwok T, Woo J, Lynn H, Griffith JF, Leung J, Tang YYN, Leung PC. Bone mineral density and the risk of peripheral arterial disease in men and women: results from Mr. and Ms Os, Hong Kong. Osteoporos Int. 2005; 16(12): 1933-8	Nonfatal Health Outcomes	Peripheral artery disease
Institute of Social Medicine and Health Policy, Shandong University, Shandong University School of Medicine, World Health Organization (WHO). China WHO Multi-country Survey Study on Health and Health System Responsiveness 2000-2001	Nonfatal Health Outcomes	None
Institute of Social Medicine and Health Policy, Shandong University, Shandong University School of Medicine, World Health Organization (WHO). China WHO Multi-country Survey Study on Health and Health System Responsiveness 2000-2001	Nonfatal Health Outcomes	Rheumatic heart disease
Pan G-W, Jiang C, Yang C-L, Na J, Fu Z-G, Yu F-Q, Yu C-Y, Liu M-J, Liu H, Wang P, Li N, Yan T-H, Zhang S-J. Epidemiological survey of mental disorders in urban and rural areas of Liaoning province. Chin J Public Health. 2006; 22(12): 1505-1507	Nonfatal Health Outcomes	Major depressive disorder
International Clearinghouse for Birth Defects Surveillance and Research. International Clearinghouse for Birth Defects Surveillance and Research Annual Report 2006. Rome, Italy: International Clearinghouse for Birth Defects Surveillance and Research, 2007	Nonfatal Health Outcomes	Congenital birth defects
Institute of Social Medicine and Health Policy, Shandong University, Shandong University School of Medicine, World Health Organization (WHO). China WHO Multi-country Survey Study on Health and Health System Responsiveness 2000-2001	Nonfatal Health Outcomes	Maternal hypertensive disorders
Institute of Social Medicine and Health Policy, Shandong University, Shandong University School of Medicine, World Health Organization (WHO). China WHO Multi-country Survey Study on Health and Health System Responsiveness 2000-2001	Nonfatal Health Outcomes	Endocarditis
Institute of Social Medicine and Health Policy, Shandong University, Shandong University School of Medicine, World Health Organization (WHO). China WHO Multi-country Survey Study on Health and Health System Responsiveness 2000-2001	Nonfatal Health Outcomes	Myocarditis
Dong B, Tang Z, Lin M, Li C, Tan D, Liang D, Liao H, Liu X, Quan Y, Fang J, Wu X, Qin W, Kilgore PE, Kennedy WA, Xu Z, Clemens JD. [Epidemiologic surveillance for bacterial meningitis in 140 000 children under 5 years of age in Nanning district, Guangxi province]. Chin J Epidemiol. 2004; 25(5): 391-5	Nonfatal Health Outcomes	Meningitis
World Health Organization (WHO). WHO Mortality Database Version October 2017. Geneva, Switzerland: World Health Organization (WHO), 2017	Nonfatal Health Outcomes	HIV/AIDS resulting in other diseases
Phillips MR, Zhang J, Shi Q, Song Z, Ding Z, Pang S, Li X, Zhang Y, Wang Z. Prevalence, treatment, and associated disability of mental disorders in four provinces in China during 2001-05: an epidemiological survey. Lancet. 2009; 373(9680): 2041-53	Nonfatal Health Outcomes	Alcohol use disorders
International Clearinghouse for Birth Defects Monitoring Systems. International Clearinghouse for Birth Defects Monitoring Systems Annual Report 2004. Rome, Italy: International Centre on Birth Defects, 2006	Nonfatal Health Outcomes	Congenital birth defects

Institute of Social Medicine and Health Policy, Shandong University, Shandong University School of Medicine, World Health Organization (WHO). China WHO Multi-country Survey Study on Health and Health System Responsiveness 2000-2001	Nonfatal Health Outcomes	Pyoderma
Hong Kong Cancer Registry. Hong Kong Cancer Registry Annual Tables. Kowloon, Hong Kong: Hong Kong Cancer Registry	Nonfatal Health Outcomes	Pancreatic cancer
Institute of Social Medicine and Health Policy, Shandong University, Shandong University School of Medicine, World Health Organization (WHO). China WHO Multi-country Survey Study on Health and Health System Responsiveness 2000-2001	Nonfatal Health Outcomes	Polycystic ovarian syndrome
Pan G-W, Jiang C, Yang C-L, Na J, Fu Z-G, Yu F-Q, Yu C-Y, Liu M-J, Liu H, Wang P, Li N, Yan T-H, Zhang S-J. Epidemiological survey of mental disorders in urban and rural areas of Liaoning province. Chin J Public Health. 2006; 22(12): 1505-1507	Nonfatal Health Outcomes	Anxiety disorders
Institute of Social Medicine and Health Policy, Shandong University, Shandong University School of Medicine, World Health Organization (WHO). China WHO Multi-country Survey Study on Health and Health System Responsiveness 2000-2001	Nonfatal Health Outcomes	Cyclist road injuries
Institute of Social Medicine and Health Policy, Shandong University, Shandong University School of Medicine, World Health Organization (WHO). China WHO Multi-country Survey Study on Health and Health System Responsiveness 2000-2001	Nonfatal Health Outcomes	Decubitus ulcer
Kulibahan, Li D-M, Yeerken, Ruo M, Panggejiapu. Investigation of attention deficit hyperactivity disorder among the ethnic Han and Kazakh students from Kuntun City of Xinjiang. Chin J Contemp Pediatr. 2005; 7(4): 366-368	Nonfatal Health Outcomes	Attention-deficit/hyperactivity disorder
Institute of Social Medicine and Health Policy, Shandong University, Shandong University School of Medicine, World Health Organization (WHO). China WHO Multi-country Survey Study on Health and Health System Responsiveness 2000-2001	Nonfatal Health Outcomes	Endometriosis
Institute of Social Medicine and Health Policy, Shandong University, Shandong University School of Medicine, World Health Organization (WHO). China WHO Multi-country Survey Study on Health and Health System Responsiveness 2000-2001	Nonfatal Health Outcomes	Alcohol use disorders
Gray DJ, Williams GM, Li Y, Chen H, Forsyth SJ, Li RS, Barnett AG, Guo J, Ross AG, Feng Z, McManus DP. A cluster-randomised intervention trial against Schistosoma japonicum in the PeoplesÂ' Republic of China: bovine and human transmission. PLoS One. 2009; 4(6): e5900	Nonfatal Health Outcomes	Schistosomiasis
Shi Q-C, Zhang J-M, Xu F-Z et al. Epidemiological survey of mental illnesses in the people aged 15 and older in Zhejiang Province. Chin J Prev Med. 2005; 39(4): 229-236	Nonfatal Health Outcomes	Anxiety disorders
Institute of Social Medicine and Health Policy, Shandong University, Shandong University School of Medicine, World Health Organization (WHO). China WHO Multi-country Survey Study on Health and Health System Responsiveness 2000-2001	Nonfatal Health Outcomes	Non-venomous animal contact
Chen H-F, Li C-Y, Chen P, See T-T, Lee H-Y. Seroprevalence of hepatitis B and C in type 2 diabetic patients. J Chin Med Assoc. 2006; 69(4): 146-52	Nonfatal Health Outcomes	Acute hepatitis B
Institute of Social Medicine and Health Policy, Shandong University, Shandong University School of Medicine, World Health Organization (WHO). China WHO Multi-country Survey Study on Health and Health System Responsiveness 2000-2001	Nonfatal Health Outcomes	Orofacial clefts
Zhu H, Napravnik S, Eron JJ, Cole SR, Ma Y, Wohl DA, Dou Z, Zhang Y, Liu Z, Zhao D, Yu L, Liu X, Cohen MS, Zhang F. Decreasing excess mortality of HIV-infected patients initiating antiretroviral therapy: comparison with mortality in general population in China, 2003-2009. J Acquir Immune Defic Syndr. 2013; 63(5): e150-157	Nonfatal Health Outcomes	HIV/AIDS resulting in other diseases
Institute of Social Medicine and Health Policy, Shandong University, Shandong University School of Medicine, World Health Organization (WHO). China WHO Multi-country Survey Study on Health and Health System Responsiveness 2000-2001	Nonfatal Health Outcomes	Cystic echinococcosis
Itoh M, Wu W, Sun D, Yao L, Li Z, Islam MZ, Chen R, Zhang K, Wang F, Zhu S, Kimura E. Confirmation of elimination of lymphatic filariasis by an IgG4 enzyme-linked immunosorbent assay with urine samples in Yongjia, Zhejiang Province and Gaoan, Jiangxi Province, PeopleÃs Republic of China. Am J Trop Med Hyg. 2007; 77(2): 330-3	Nonfatal Health Outcomes	Lymphatic filariasis
Liao L, Xing H, Su B, Wang Z, Ruan Y, Wang X, Liu Z, Lu Y, Yang S, Zhao Q, Vermund SH, Chen RY, Shao Y. Impact of HIV drug resistance on virologic and immunologic failure and mortality in a cohort of patients on antiretroviral therapy in China. AIDS. 2013; 27(11): 1815-24	Nonfatal Health Outcomes	HIV/AIDS resulting in other diseases

Institute of Social Medicine and Health Policy, Shandong University, Shandong University School of Medicine, World Health Organization (WHO). China WHO Multi-country Survey Study on Health and Health System Responsiveness 2000-2001	Nonfatal Health Outcomes	Exposure to mechanical forces
Institute of Social Medicine and Health Policy, Shandong University, Shandong University School of Medicine, World Health Organization (WHO). China WHO Multi-country Survey Study on Health and Health System Responsiveness 2000-2001	Nonfatal Health Outcomes	Self-harm by other specified means
Chen M, Xiong L, Chen H, Xu A, He L, Hu P. Prevalence, risk factors and impact of gastroesophageal reflux disease symptoms: a population-based study in South China. Scand J Gastroenterol. 2005; 40(7): 759-67	Nonfatal Health Outcomes	Gastroesophageal reflux disease
Dong B, Tang Z, Lin M, Li C, Tan D, Liang D, Liao H, Liu X, Quan Y, Fang J, Wu X, Qin W, Kilgore PE, Kennedy WA, Xu Z, Clemens JD. [Epidemiologic surveillance for bacterial meningitis in 140 000 children under 5 years of age in Nanning district, Guangxi province]. Chin J Epidemiol. 2004; 25(5): 391-5	Nonfatal Health Outcomes	Hearing loss
Ochiai RL, Wang X, von Seidlein L, Yang J, Bhutta ZA, Bhattacharya SK, Agtini M, Deen JL, Wain J, Kim DR, Ali M, Acosta CJ, Jodar L, Clemens JD. Salmonella Paratyphi A Rates, Asia. Emerg Infect Dis. 2005; 11(11): 1764-6	Nonfatal Health Outcomes	Typhoid fever
Ochiai RL, Wang X, von Seidlein L, Yang J, Bhutta ZA, Bhattacharya SK, Agtini M, Deen JL, Wain J, Kim DR, Ali M, Acosta CJ, Jodar L, Clemens JD. Salmonella Paratyphi A Rates, Asia. Emerg Infect Dis. 2005; 11(11): 1764-6	Nonfatal Health Outcomes	Paratyphoid fever
Dong B, Tang Z, Lin M, Li C, Tan D, Liang D, Liao H, Liu X, Quan Y, Fang J, Wu X, Qin W, Kilgore PE, Kennedy WA, Xu Z, Clemens JD. [Epidemiologic surveillance for bacterial meningitis in 140 000 children under 5 years of age in Nanning district, Guangxi province]. Chin J Epidemiol. 2004; 25(5): 391-5	Nonfatal Health Outcomes	Vision loss
Detels R, Wu Z, Rotheram MJ, Li L, Guan J, Yin Y, Liang G, Lee M, Hu L, National Institute of Mental Health (NIMH) Collaborative HIV Prevention Trial Group. Sexually transmitted disease prevalence and characteristics of market vendors in eastern China. Sex Transm Dis. 2003; 30(11): 803-8	Nonfatal Health Outcomes	Genital herpes
Institute of Social Medicine and Health Policy, Shandong University, Shandong University School of Medicine, World Health Organization (WHO). China WHO Multi-country Survey Study on Health and Health System Responsiveness 2000-2001	Nonfatal Health Outcomes	Pancreatitis
Llibre Rodriguez JJ, Ferri CP, Acosta D, Guerra M, Huang Y, Jacob KS, Krishnamoorthy ES, Salas A, Sosa AL, Acosta I, Dewey ME, Gaona C, Jotheeswaran AT, Li S, Rodriguez D, Rodriguez G, Kumar PS, Valhuerdi A, Prince M, 10/66 Dementia Research Group. Prevalence of dementia in Latin America, India, and China: a population-based cross-sectional survey. Lancet. 2008; 372(9637): 464-74	Nonfatal Health Outcomes	Other chromosomal abnormalities
Chen J, Wildman RP, Gu D, Kusek JW, Spruill M, Reynolds K, Liu D, Hamm LL, Whelton PK, He J. Prevalence of decreased kidney function in Chinese adults aged 35 to 74 years. Kidney Int. 2005; 68(6): 2837-45	Nonfatal Health Outcomes	Chronic kidney disease
Institute of Social Medicine and Health Policy, Shandong University, Shandong University School of Medicine, World Health Organization (WHO). China WHO Multi-country Survey Study on Health and Health System Responsiveness 2000-2001	Nonfatal Health Outcomes	Neonatal encephalopathy due to birth asphyxia and trauma
Xu H, Dai Q, Xu Y, Gong Z, Dai G, Ding M, Duggan C, Hu Z, Hu FB. Time trends and risk factor associated with premature birth and infants deaths due to prematurity in Hubei Province, China from 2001 to 2012. BMC Pregnancy Childbirth. 2015; 15: 329	Nonfatal Health Outcomes	Neonatal preterm birth
Fung KT, Fung J, Lai CL, Yuen MF. Etiologies of chronic liver diseases in Hong Kong. Eur J Gastroenterol Hepatol. 2007; 19(8): 659-64	Nonfatal Health Outcomes	Cirrhosis due to NASH
Wong WM, Lai KC, Lam KF, Hui WM, Huang JQ, Xia HH, Hu WH, Lam CL, Chan CK, Lam SK, Wong BC. Onset and disappearance of reflux symptoms in a Chinese population: a 1-year follow-up study. Aliment Pharmacol Ther. 2004; 20(7): 803-12	Nonfatal Health Outcomes	Gastroesophageal reflux disease
Cheung T-K, Lai C-L, Wong BC-Y, Fung J, Yuen M-F. Clinical features, biochemical parameters, and virological profiles of patients with hepatocellular carcinoma in Hong Kong. Aliment Pharmacol Ther. 2006; 24(4): 573-83	Nonfatal Health Outcomes	Liver cancer due to NASH
World Health Organization (WHO). China World Health Survey 2002. Geneva, Switzerland: World Health Organization (WHO), 2005	Nonfatal Health Outcomes	Major depressive disorder
International Clearinghouse for Birth Defects Monitoring Systems. International Clearinghouse for Birth Defects Monitoring Systems Annual Report 2004. Rome, Italy: International Centre on Birth Defects, 2006	Nonfatal Health Outcomes	Congenital musculoskeletal and limb anomalies
International Clearinghouse for Birth Defects Surveillance and Research. International Clearinghouse for Birth Defects Surveillance and Research Annual Report 2005. Rome, Italy: International Clearinghouse for Birth Defects Surveillance and Research, 2007	Nonfatal Health Outcomes	Congenital musculoskeletal and limb anomalies

World Health Organization (WHO). China World Health Survey 2002. Geneva, Switzerland: World Health Organization (WHO), 2005	Nonfatal Health Outcomes	Low back and neck pain
Institute of Social Medicine and Health Policy, Shandong University, Shandong University School of Medicine, World Health Organization (WHO). China WHO Multi-country Survey Study on Health and Health System Responsiveness 2000-2001	Nonfatal Health Outcomes	Physical violence by sharp object
Chinese Center for Disease Control and Prevention (CCDC). China Notifiable Infectious Diseases 2004	Nonfatal Health Outcomes	Gonococcal infection
Institute of Social Medicine and Health Policy, Shandong University, Shandong University School of Medicine, World Health Organization (WHO). China WHO Multi-country Survey Study on Health and Health System Responsiveness 2000-2001	Nonfatal Health Outcomes	Meningitis
Hong Kong Cancer Registry. Hong Kong Cancer Registry Annual Tables. Kowloon, Hong Kong: Hong Kong Cancer Registry	Nonfatal Health Outcomes	Cervical cancer
Hong Kong Cancer Registry. Hong Kong Cancer Registry Annual Tables. Kowloon, Hong Kong: Hong Kong Cancer Registry	Nonfatal Health Outcomes	Non-Hodgkin lymphoma
Chinese Center for Disease Control and Prevention (CCDC). China Notifiable Infectious Diseases 2005	Nonfatal Health Outcomes	Rabies
Institute of Social Medicine and Health Policy, Shandong University, Shandong University School of Medicine, World Health Organization (WHO). China WHO Multi-country Survey Study on Health and Health System Responsiveness 2000-2001	Nonfatal Health Outcomes	Cirrhosis and other chronic liver diseases
Chinese Center for Disease Control and Prevention (CCDC). China Notifiable Infectious Diseases 2004	Nonfatal Health Outcomes	Sexually transmitted infections excluding HIV
Institute of Social Medicine and Health Policy, Shandong University, Shandong University School of Medicine, World Health Organization (WHO). China WHO Multi-country Survey Study on Health and Health System Responsiveness 2000-2001	Nonfatal Health Outcomes	Neonatal preterm birth
Chinese Center for Disease Control and Prevention (CCDC). China National Tuberculosis Prevalence Survey 2000	Nonfatal Health Outcomes	Latent tuberculosis infection
Guerra M, Prina AM, Ferri CP, Acosta D, Gallardo S, Huang Y, Jacob KS, Jimenez-Velazquez IZ, Llibre Rodriguez JJ, Liu Z, Salas A, Sosa AL, Williams JD, Uwakwe R, Prince M. A comparative cross-cultural study of the prevalence of late life depression in low and middle income countries. J Affect Disord. 2016; 190: 362Â–8	Nonfatal Health Outcomes	Major depressive disorder
Jiang RG,Â Luo DS,Â Huang CP,Â Li WM. Study on the prevalence rate and risk factors of chronic obstructive pulmonary disease in rural community population in Hubei province. Chin J Epidemiol. 2007; 28(10): 976-9	Nonfatal Health Outcomes	Chronic obstructive pulmonary disease
International Clearinghouse for Birth Defects Monitoring Systems. International Clearinghouse for Birth Defects Monitoring Systems Annual Report 2003. Rome, Italy: International Centre on Birth Defects	Nonfatal Health Outcomes	Neural tube defects
International Clearinghouse for Birth Defects Surveillance and Research. International Clearinghouse for Birth Defects Surveillance and Research Annual Report 2005. Rome, Italy: International Clearinghouse for Birth Defects Surveillance and Research, 2007	Nonfatal Health Outcomes	Neural tube defects
Jin W-L, Ayinuer W-M-E, Du Y-S, Zhong X-Y, David Coghill. Prevalence and infludential factors of ADHD in children aged 5-15 years old in Zhabei district in Shanghai. Shanghai Arch Psychiatry. 2010; 22(4): 211-216	Nonfatal Health Outcomes	Attention-deficit/hyperactivity disorder
Xiaoyong L, Helong C, Bin H. Epidemiological survey on prevalence of schizophrenia in Jiangxi province. Shanghai Arch Psychiatry. 2004; 16(4): 014	Nonfatal Health Outcomes	Schizophrenia
Institute of Social Medicine and Health Policy, Shandong University, Shandong University School of Medicine, World Health Organization (WHO). China WHO Multi-country Survey Study on Health and Health System Responsiveness 2000-2001	Nonfatal Health Outcomes	Down syndrome
Dongfeng G, Guangyong H, Jiang H, Xigui W, Xiufang D, Macmahon S, Whelton PK. Investigation of Prevalence and Distributing Feature of Chronic Heart Failure in Chinese Adult Population. Chin J Cardiol. 2003; 31(1): 3Â–6	Nonfatal Health Outcomes	
International Clearinghouse for Birth Defects Monitoring Systems. International Clearinghouse for Birth Defects Monitoring Systems Annual Report 2004. Rome, Italy: International Centre on Birth Defects, 2006	Nonfatal Health Outcomes	Orofacial clefts
Institute of Social Medicine and Health Policy, Shandong University, Shandong University School of Medicine, World Health Organization (WHO). China WHO Multi-country Survey Study on Health and Health System Responsiveness 2000-2001	Nonfatal Health Outcomes	Cutaneous and mucocutaneous leishmaniasis

Yan T, Cai R, Mo O, Zhu D, Ouyang H, Huang L, Zhao M, Huang F, Li L, Liang X, Xu X. Incidence and complete molecular characterization of glucose-6-phosphate dehydrogenase deficiency in the Guangxi Zhuang autonomous region of southern China: description of four novel mutations. Haematologica. 2006; 91(10): 1321-8	Nonfatal Health Outcomes	G6PD deficiency
Yongjun T, Samuelson J, Qingsheng D, Ali MM, Li X, Yanjian M, Xiaoqing C, Jun L, Jian C, Bi L. The prevalence of sexually transmitted and other lower reproductive tract infections among rural women in Sichuan Province, China. Southeast Asian J Trop Med Public Health. 2009; 40(5): 1038-47	Nonfatal Health Outcomes	Sexually transmitted infections excluding HIV
World Health Organization (WHO). China World Health Survey 2002. Geneva, Switzerland: World Health Organization (WHO), 2005	Nonfatal Health Outcomes	Unintentional firearm injuries
World Health Organization (WHO). China World Health Survey 2002. Geneva, Switzerland: World Health Organization (WHO), 2005	Nonfatal Health Outcomes	Meningitis
World Health Organization (WHO). China World Health Survey 2002. Geneva, Switzerland: World Health Organization (WHO), 2005	Nonfatal Health Outcomes	Silicosis
Liu L, Zheng C-X, Peng S-F, Zhou H-Y, Su Z-Y, He L, Ai T. Evaluation of urinary S100B protein level and lactate/creatinine ratio for early diagnosis and prognostic prediction of neonatal hypoxic-ischemic encephalopathy. Neonatology. 2010; 97(1): 41-4	Nonfatal Health Outcomes	Neonatal encephalopathy due to birth asphyxia and trauma
World Health Organization (WHO). China World Health Survey 2002. Geneva, Switzerland: World Health Organization (WHO), 2005	Nonfatal Health Outcomes	Physical violence by other means
Institute of Social Medicine and Health Policy, Shandong University, Shandong University School of Medicine, World Health Organization (WHO). China WHO Multi-country Survey Study on Health and Health System Responsiveness 2000-2001	Nonfatal Health Outcomes	Maternal abortion, miscarriage, and ectopic pregnancy
Wu JZ, Su MH, Chen MW, Luo GH, Liang RX, Wei ZL, Jiang JN, Huang LY. A matching caseÂ–control study on the relationship between HBV, HCV infection and HCC in endemic area of HCC in Guangxi, China. J Guangxi Med Univ. 2003; 20: 313-5	Nonfatal Health Outcomes	Liver cancer due to hepatitis B
Wu JZ, Su MH, Chen MW, Luo GH, Liang RX, Wei ZL, Jiang JN, Huang LY. A matching caseÂ–control study on the relationship between HBV, HCV infection and HCC in endemic area of HCC in Guangxi, China. J Guangxi Med Univ. 2003; 20: 313-5	Nonfatal Health Outcomes	Liver cancer due to hepatitis C
World Health Organization (WHO). China World Health Survey 2002. Geneva, Switzerland: World Health Organization (WHO), 2005	Nonfatal Health Outcomes	Neonatal sepsis and other neonatal infections
World Health Organization (WHO). China World Health Survey 2002. Geneva, Switzerland: World Health Organization (WHO), 2005	Nonfatal Health Outcomes	Pyoderma
Wang W-N, Li X-P, Cong X-S. Epidemiological survey of alcohol abuse and dependence in Baoding. Shanghai Arch Psychiatry. 2008; 20(4): 209-212	Nonfatal Health Outcomes	Alcohol use disorders
Keqing L, Ze C, Lijun C, Qinpu J, Guang S, Haoran W, Jing H, Wuwen Z, Jianguo X, Yanping Z, Ben Z, Jianxun J, Xueyi W, Jun T, Yufu Z, Haishan H, Jianping G, Enyi Z. Epidemiological survey of mental disorders in the people aged 18 and older in Hebei Province. Asian J Psychiatr. 2008; 1(2): 51-5	Nonfatal Health Outcomes	Major depressive disorder
World Health Organization (WHO). China World Health Survey 2002. Geneva, Switzerland: World Health Organization (WHO), 2005	Nonfatal Health Outcomes	Self-harm
Institute of Social Medicine and Health Policy, Shandong University, Shandong University School of Medicine, World Health Organization (WHO). China WHO Multi-country Survey Study on Health and Health System Responsiveness 2000-2001	Nonfatal Health Outcomes	Acute glomerulonephritis
Institute of Social Medicine and Health Policy, Shandong University, Shandong University School of Medicine, World Health Organization (WHO). China WHO Multi-country Survey Study on Health and Health System Responsiveness 2000-2001	Nonfatal Health Outcomes	Other unintentional injuries
International Clearinghouse for Birth Defects Monitoring Systems. International Clearinghouse for Birth Defects Monitoring Systems Annual Report 2003. Rome, Italy: International Centre on Birth Defects	Nonfatal Health Outcomes	Digestive congenital anomalies
International Clearinghouse for Birth Defects Monitoring Systems. International Clearinghouse for Birth Defects Monitoring Systems Annual Report 2004. Rome, Italy: International Centre on Birth Defects, 2006	Nonfatal Health Outcomes	Digestive congenital anomalies
International Clearinghouse for Birth Defects Surveillance and Research. International Clearinghouse for Birth Defects Surveillance and Research Annual Report 2005. Rome, Italy: International Clearinghouse for Birth Defects Surveillance and Research, 2007	Nonfatal Health Outcomes	Digestive congenital anomalies
International Clearinghouse for Birth Defects Surveillance and Research. International Clearinghouse for Birth Defects Surveillance and Research Annual Report 2006. Rome, Italy: International Clearinghouse for Birth Defects Surveillance and Research, 2007	Nonfatal Health Outcomes	Digestive congenital anomalies

Jia L, Zhang D, Zhou C, Wang L, Luo H, Wu A. [A Epidemiological Investigation of Alcohol Dependence in Partial Area of Guizhou Province]. Guizhou Med J. 2002; 1078Â–9	Nonfatal Health Outcomes	Alcohol use disorders
Institute of Social Medicine and Health Policy, Shandong University, Shandong University School of Medicine, World Health Organization (WHO). China WHO Multi-country Survey Study on Health and Health System Responsiveness 2000-2001	Nonfatal Health Outcomes	Encephalitis
Keqing L, Ze C, Lijun C, Qinpu J, Guang S, Haoran W, Jing H, Wuwen Z, Jianguo X, Yanping Z, Ben Z, Jianxun J, Xueyi W, Jun T, Yufu Z, Haishan H, Jianping G, Enyi Z. Epidemiological survey of mental disorders in the people aged 18 and older in Hebei Province. Asian J Psychiatr. 2008; 1(2): 51-5	Nonfatal Health Outcomes	Dysthymia
World Health Organization (WHO). China World Health Survey 2002. Geneva, Switzerland: World Health Organization (WHO), 2005	Nonfatal Health Outcomes	Foreign body in eyes
World Health Organization (WHO). China World Health Survey 2002. Geneva, Switzerland: World Health Organization (WHO), 2005	Nonfatal Health Outcomes	Other road injuries
Mao Y-M, Luo Z-Y, Li B, Hu T-Y. Prospective Study on the Survival of HCC Patients Treated with Transcatheter Arterial Lipiodol Chemoembolization . Asian Pac J Cancer Prev. 2012; 13(3): 1039-42	Nonfatal Health Outcomes	Liver cancer due to hepatitis B
Institute of Social Medicine and Health Policy, Shandong University, Shandong University School of Medicine, World Health Organization (WHO). China WHO Multi-country Survey Study on Health and Health System Responsiveness 2000-2001	Nonfatal Health Outcomes	Intracerebral hemorrhage
Sun X-L, Li K-Q, Cui L-J, Jiang Q-P, Gao L-H, Liu Y-Q, Han Y-C, Yang L-H, Li J-F, Yan B-P, Zhang Y, Lv H. Epidemiological Survey on Anxiety Disorder in of Hebei Province. Cap Med. 2009; 7: 37-9	Nonfatal Health Outcomes	Anxiety disorders
Hong S, Xin C, Qianhong Y, Yanan W, Wenyan X, Peeling RW, Mabey D. Pelvic inflammatory disease in the People's Republic of China: aetiology and management. Int J STD AIDS. 2002; 13(8): 568-72	Nonfatal Health Outcomes	Chlamydial infection
Institute of Social Medicine and Health Policy, Shandong University, Shandong University School of Medicine, World Health Organization (WHO). China WHO Multi-country Survey Study on Health and Health System Responsiveness 2000-2001	Nonfatal Health Outcomes	Unintentional suffocation
International Clearinghouse for Birth Defects Monitoring Systems. International Clearinghouse for Birth Defects Monitoring Systems Annual Report 2003. Rome, Italy: International Centre on Birth Defects	Nonfatal Health Outcomes	Congenital heart anomalies
World Health Organization (WHO). China World Health Survey 2002. Geneva, Switzerland: World Health Organization (WHO), 2005	Nonfatal Health Outcomes	Appendicitis
Institute of Social Medicine and Health Policy, Shandong University, Shandong University School of Medicine, World Health Organization (WHO). China WHO Multi-country Survey Study on Health and Health System Responsiveness 2000-2001	Nonfatal Health Outcomes	Gallbladder and biliary diseases
Hong S, Xin C, Qianhong Y, Yanan W, Wenyan X, Peeling RW, Mabey D. Pelvic inflammatory disease in the People's Republic of China: aetiology and management. Int J STD AIDS. 2002; 13(8): 568-72	Nonfatal Health Outcomes	Pelvic inflammatory disease
Institute of Social Medicine and Health Policy, Shandong University, Shandong University School of Medicine, World Health Organization (WHO). China WHO Multi-country Survey Study on Health and Health System Responsiveness 2000-2001	Nonfatal Health Outcomes	Interstitial lung disease and pulmonary sarcoidosis
Miao Z, Li C, Chen Y, Zhao S, Wang Y, Wang Z, Chen X, Xu F, Wang F, Sun R, Hu J, Song W, Yan S, Wang C-Y. Dietary and lifestyle changes associated with high prevalence of hyperuricemia and gout in the Shandong coastal cities of Eastern China. J Rheumatol. 2008; 35(9): 1859-64	Nonfatal Health Outcomes	Gout
Institute of Social Medicine and Health Policy, Shandong University, Shandong University School of Medicine, World Health Organization (WHO). China WHO Multi-country Survey Study on Health and Health System Responsiveness 2000-2001	Nonfatal Health Outcomes	Motor vehicle road injuries
Zhang M-L, Yuan G-Z, Mao J-Y, Yao J-J, Chu X, Qian Y-C, Ji Q, Zhang F-J, Qi S-G, Xu W-W, Zhang X, Tang R-C, Liu X-W, Huang Y-P, Zhou D-X, Jiang Q-Y, Bao Z-H. Epidemiologic Features of the Prevalence Rates of Various Mental Disorders and their Related Factors in Wuxi City: a Cross-sectional Survey. Chin J Clin Rehab. 2004; 8(24): 4950-2	Nonfatal Health Outcomes	Schizophrenia
Ochiai RL, Wang X, von Seidlein L, Yang J, Bhutta ZA, Bhattacharya SK, Agtini M, Deen JL, Wain J, Kim DR, Ali M, Acosta CJ, Jodar L, Clemens JD. Salmonella Paratyphi A Rates, Asia. Emerg Infect Dis. 2005; 11(11): 1764-6	Nonfatal Health Outcomes	Intestinal infectious diseases

Ochiai RL, Acosta CJ, Danovaro-Holliday MC, Baiqing D, Bhattacharya SK, Agtini MD, Bhutta ZA, Canh DG, Ali M, Shin S, Wain J, Page A-L, Albert MJ, Farrar J, Abu-Elyazeed R, Pang T, Galindo CM, von Seidlein L, Clemens JD, Domi Typhoid Study Group. A study of typhoid fever in five Asian countries: disease burden and implications for controls. Bull World Health Organ. 2008; 86(4): 260-8	Nonfatal Health Outcomes	Intestinal infectious diseases
Wang H, Wang F, Zhang L, Zuo L, Chronic Kidney Disease Prognosis Consortium (CKD-PC). China - Beijing Community-Based Screening for Chronic Kidney Disease Prevalence Data 2004 [Unpublished]	Nonfatal Health Outcomes	Chronic kidney disease
Zhang L, Zuo L, Xu G, Wang F, Wang M, Wang S, Lv J, Liu L, Wang H. Community-based screening for chronic kidney disease among populations older than 40 years in Beijing. Nephrol Dial Transplant. 2007; 22(4): 1093-9	Nonfatal Health Outcomes	Chronic kidney disease
Sun Y, Pei W, Wu Y, Wang G. [Association of herpes simplex virus type2 infection with dyslipidemia in Chinese]. Nat Med J Chin. 2003; 83(20): 1774-7	Nonfatal Health Outcomes	Genital herpes
Institute of Social Medicine and Health Policy, Shandong University, Shandong University School of Medicine, World Health Organization (WHO). China WHO Multi-country Survey Study on Health and Health System Responsiveness 2000-2001	Nonfatal Health Outcomes	Syphilis
Institute of Social Medicine and Health Policy, Shandong University, Shandong University School of Medicine, World Health Organization (WHO). China WHO Multi-country Survey Study on Health and Health System Responsiveness 2000-2001	Nonfatal Health Outcomes	Asbestosis
Tan Z, Zeng B-Q, Ma J-Q, Cao F-P, Wu Y-X. Current situation of intenstinal parasite infection and its prevention and treatment in Shunde district. J Trop Med. 2004; 4(4): 435-37	Nonfatal Health Outcomes	Food-borne trematodiases
Detels R, Wu Z, Rotheram MJ, Li L, Guan J, Yin Y, Liang G, Lee M, Hu L, National Institute of Mental Health (NIMH) Collaborative HIV Prevention Trial Group. Sexually transmitted disease prevalence and characteristics of market vendors in eastern China. Sex Transm Dis. 2003; 30(11): 803-8	Nonfatal Health Outcomes	Sexually transmitted infections excluding HIV
World Health Organization (WHO). China World Health Survey 2002. Geneva, Switzerland: World Health Organization (WHO), 2005	Nonfatal Health Outcomes	Falls
World Health Organization (WHO). China World Health Survey 2002. Geneva, Switzerland: World Health Organization (WHO), 2005	Nonfatal Health Outcomes	Poisonings
He M, Zeng J, Liu Y, Xu J, Pokharel GP, Ellwein LB. Refractive error and visual impairment in urban children in southern China. Invest Ophthalmol Vis Sci. 2004; 45(3): 793-9	Nonfatal Health Outcomes	Refraction disorders
Huang S, Zheng Y, Foster PJ, Huang W, He M. Prevalence and causes of visual impairment in Chinese adults in urban southern China. Arch Ophthalmol. 2009; 127(10): 1362-7	Nonfatal Health Outcomes	Refraction disorders
Institute of Social Medicine and Health Policy, Shandong University, Shandong University School of Medicine, World Health Organization (WHO). China WHO Multi-country Survey Study on Health and Health System Responsiveness 2000-2001	Nonfatal Health Outcomes	Paralytic ileus and intestinal obstruction
World Health Organization (WHO). China World Health Survey 2002. Geneva, Switzerland: World Health Organization (WHO), 2005	Nonfatal Health Outcomes	Ischemic stroke
Institute of Social Medicine and Health Policy, Shandong University, Shandong University School of Medicine, World Health Organization (WHO). China WHO Multi-country Survey Study on Health and Health System Responsiveness 2000-2001	Nonfatal Health Outcomes	Near vision loss
Hong Kong Cancer Registry. Hong Kong Cancer Registry Annual Tables. Kowloon, Hong Kong: Hong Kong Cancer Registry	Nonfatal Health Outcomes	Prostate cancer
Yu M, Ping Z, Zhang S, He Y, Dong R, Guo X. The survey of birth defects rate based on birth registration system. Chin Med J (Engl). 2015; 128(1): 7-14	Nonfatal Health Outcomes	Orofacial clefts
Institute of Social Medicine and Health Policy, Shandong University, Shandong University School of Medicine, World Health Organization (WHO). China WHO Multi-country Survey Study on Health and Health System Responsiveness 2000-2001	Nonfatal Health Outcomes	Uterine fibroids
World Health Organization (WHO). China World Health Survey 2002. Geneva, Switzerland: World Health Organization (WHO), 2005	Nonfatal Health Outcomes	Other exposure to mechanical forces
World Health Organization (WHO). China World Health Survey 2002. Geneva, Switzerland: World Health Organization (WHO), 2005	Nonfatal Health Outcomes	Cellulitis
Zhang M-L, Yuan G-Z, Mao J-Y, Yao J-J, Chu X, Qian Y-C, Ji Q, Zhang F-J, Qi S-G, Xu W-W, Zhang X, Tang R-C, Liu X-W, Huang Y-P, Zhou D-X, Jiang Q-Y, Bao Z-H. Epidemiologic Features of the Prevalence Rates of Various Mental Disorders and their Related Factors in Wuxi City: a Cross-sectional Survey. Chin J Clin Rehab. 2004; 8(24): 4950-2	Nonfatal Health Outcomes	Alcohol use disorders

Institute of Social Medicine and Health Policy, Shandong University, Shandong University School of Medicine, World Health Organization (WHO). China WHO Multi-country Survey Study on Health and Health System Responsiveness 2000-2001	Nonfatal Health Outcomes	Foreign body in eyes
World Health Organization (WHO). China World Health Survey 2002. Geneva, Switzerland: World Health Organization (WHO), 2005	Nonfatal Health Outcomes	Neural tube defects
Zeng L, Cheng Y, Dang S, Yan H, Dibley MJ, Chang S, Kong L. Impact of micronutrient supplementation during pregnancy on birth weight, duration of gestation, and perinatal mortality in rural western China: double blind cluster randomised controlled trial. BMJ. 2008; 337: a2001	Nonfatal Health Outcomes	Neonatal preterm birth
World Health Organization (WHO). China World Health Survey 2002. Geneva, Switzerland: World Health Organization (WHO), 2005	Nonfatal Health Outcomes	Visceral leishmaniasis
Llibre Rodriguez JJ, Ferri CP, Acosta D, Guerra M, Huang Y, Jacob KS, Krishnamoorthy ES, Salas A, Sosa AL, Acosta I, Dewey ME, Gaona C, Jotheeswaran AT, Li S, Rodriguez D, Rodriguez G, Kumar PS, Valhuerdi A, Prince M, 10/66 Dementia Research Group. Prevalence of dementia in Latin America, India, and China: a population-based cross-sectional survey. Lancet. 2008; 372(9637): 464-74	Nonfatal Health Outcomes	Congenital birth defects
Hong S, Xin C, Qianhong Y, Yanan W, Wenyan X, Peeling RW, Mabey D. Pelvic inflammatory disease in the People's Republic of China: aetiology and management. Int J STD AIDS. 2002; 13(8): 568-72	Nonfatal Health Outcomes	Other sexually transmitted infections
Institute of Social Medicine and Health Policy, Shandong University, Shandong University School of Medicine, World Health Organization (WHO). China WHO Multi-country Survey Study on Health and Health System Responsiveness 2000-2001	Nonfatal Health Outcomes	Falls
Shen YC ZM, Huang YQ, He YL, Liu ZR, Cheng H, Tsang A, Lee S, Kessler RC. Twelve-month prevalence, severity, and unmet need for treatment of mental disorders in metropolitan China. Psychol Med. 2006; 36(2): 257-67	Nonfatal Health Outcomes	Major depressive disorder
World Health Organization (WHO). China World Health Survey 2002. Geneva, Switzerland: World Health Organization (WHO), 2005	Nonfatal Health Outcomes	Adverse effects of medical treatment
World Health Organization (WHO). China World Health Survey 2002. Geneva, Switzerland: World Health Organization (WHO), 2005	Nonfatal Health Outcomes	Cyclist road injuries
Zhang S, Tong W, Xu T, Wu B, Zhang Y. Diabetes and impaired fasting glucose in Mongolian population, Inner Mongolia, China. Diabetes Res Clin Pract. 2009; 86(2): 124-9	Nonfatal Health Outcomes	Diabetes mellitus
World Health Organization (WHO). China World Health Survey 2002. Geneva, Switzerland: World Health Organization (WHO), 2005	Nonfatal Health Outcomes	Gastritis and duodenitis
Institute of Social Medicine and Health Policy, Shandong University, Shandong University School of Medicine, World Health Organization (WHO). China WHO Multi-country Survey Study on Health and Health System Responsiveness 2000-2001	Nonfatal Health Outcomes	Maternal sepsis and other maternal infections
Jiang L, Chang W-J, Su W, Liu W-H, Cao G-W. Epidemiological Survey of Attention-deficit Hyperactivity Disorder in Pupils of Urban Districts in Zhenjiang. Acad J Second Mil Med Univ. 2004; 25(11): 1238-40	Nonfatal Health Outcomes	Attention-deficit/hyperactivity disorder
Zhang S, Lu Z, Liu H, Xiao X, Zhao Z, Bao G, Han J, Jing T, Chen G. Incidence of Japanese encephalitis, visceral leishmaniasis and malaria before and after the Wenchuan earthquake, in China. Addict Behav Rep. 2013; 128(1): 85-9	Nonfatal Health Outcomes	Visceral leishmaniasis
Yongjun T, Samuelson J, Qingsheng D, Ali MM, Li X, Yanjian M, Xiaoqing C, Jun L, Jian C, Bi L. The prevalence of sexually transmitted and other lower reproductive tract infections among rural women in Sichuan Province, China. Southeast Asian J Trop Med Public Health. 2009; 40(5): 1038-47	Nonfatal Health Outcomes	Chlamydial infection
Shi Z, Yuan B, Zhang C, Zhou M, Holmboe-Ottesen G. Egg consumption and the risk of diabetes in adults, Jiangsu, China. Nutrition. 2011; 27(2): 194Â–8	Nonfatal Health Outcomes	Diabetes mellitus
Yongjun T, Samuelson J, Qingsheng D, Ali MM, Li X, Yanjian M, Xiaoqing C, Jun L, Jian C, Bi L. The prevalence of sexually transmitted and other lower reproductive tract infections among rural women in Sichuan Province, China. Southeast Asian J Trop Med Public Health. 2009; 40(5): 1038-47	Nonfatal Health Outcomes	Trichomoniasis
Institute of Social Medicine and Health Policy, Shandong University, Shandong University School of Medicine, World Health Organization (WHO). China WHO Multi-country Survey Study on Health and Health System Responsiveness 2000-2001	Nonfatal Health Outcomes	Foreign body in other body part
Liu H-Y, Zheng F-L, Shi S-T, Shi M-Z, Ni L-X, Zhou J-J, Li L-J. Epidemiological Investigation of the Association between Serum Elements and Children with ADHD in four Ethnic Groups in Yunnan Province. Chin J Sch Dr. 2006; 20(4): 349-51	Nonfatal Health Outcomes	Attention-deficit/hyperactivity disorder
Yu M, Ping Z, Zhang S, He Y, Dong R, Guo X. The survey of birth defects rate based on birth registration system. Chin Med J (Engl). 2015; 128(1): 7-14	Nonfatal Health Outcomes	Neural tube defects

Institute of Social Medicine and Health Policy, Shandong University, Shandong University School of Medicine, World Health Organization (WHO). China WHO Multi-country Survey Study on Health and Health System Responsiveness 2000-2001	Nonfatal Health Outcomes	Physical violence by firearm
Ma X, Xiang Y-T, Cai Z-J, Li S-R, Xiang Y-Q, Guo H-L, Hou Y-Z, Li Z-B, Li Z-J, Tao Y-F, Dang W-M, Wu X-M, Deng J, Wang C-Y, Lai Kelly Y-C, Ungvari Gabor S. Prevalence and Socio-demographic Correlates of Major Depressive Episode in Rural and Urban Areas of Beijing. J Affect Disord. 2009; 115(3): 323-33	Nonfatal Health Outcomes	Major depressive disorder
Tang W, Liu Q-J, Wen X-T, Zhang C-Y, Yan X-L, Zhang B-Q, Sha B, Ye T-M, Lin C-C, Tu X-D, Chen D-S, Chen G-D, Ye J-C, Sun H-B. Epidemiological survey on mental disorders in Wenzhou area. Shanghai Arch Psychiatry. 2005; 17(4): 197-9	Nonfatal Health Outcomes	Schizophrenia
Yu M, Ping Z, Zhang S, He Y, Dong R, Guo X. The survey of birth defects rate based on birth registration system. Chin Med J (Engl). 2015; 128(1): 7-14	Nonfatal Health Outcomes	Digestive congenital anomalies
World Health Organization (WHO). China World Health Survey 2002. Geneva, Switzerland: World Health Organization (WHO), 2005	Nonfatal Health Outcomes	Endometriosis
Yu M, Ping Z, Zhang S, He Y, Dong R, Guo X. The survey of birth defects rate based on birth registration system. Chin Med J (Engl). 2015; 128(1): 7-14	Nonfatal Health Outcomes	Congenital heart anomalies
World Health Organization (WHO). China World Health Survey 2002. Geneva, Switzerland: World Health Organization (WHO), 2005	Nonfatal Health Outcomes	Myocarditis
Yu M, Ping Z, Zhang S, He Y, Dong R, Guo X. The survey of birth defects rate based on birth registration system. Chin Med J (Engl). 2015; 128(1): 7-14	Nonfatal Health Outcomes	Congenital birth defects
World Health Organization (WHO). China World Health Survey 2002. Geneva, Switzerland: World Health Organization (WHO), 2005	Nonfatal Health Outcomes	Motorcyclist road injuries
Zhou Y, Wang C, Yao W, Chen P, Kang J, Huang S, Chen B, Wang C, Ni D, Wang X, Wang D, Liu S, Lu J, Zheng J, Zhong N, Ran P. COPD in Chinese nonsmokers. Eur Respir J. 2009; 33(3): 509Â–18	Nonfatal Health Outcomes	Chronic obstructive pulmonary disease
Ochiai RL, Acosta CJ, Danovaro-Holliday MC, Baiqing D, Bhattacharya SK, Agtini MD, Bhutta ZA, Canh DG, Ali M, Shin S, Wain J, Page A-L, Albert MJ, Farrar J, Abu-Elyazeed R, Pang T, Galindo CM, von Seidlein L, Clemens JD, Domi Typhoid Study Group. A study of typhoid fever in five Asian countries: disease burden and implications for controls. Bull World Health Organ. 2008; 86(4): 260-8	Nonfatal Health Outcomes	Typhoid fever
Ochiai RL, Acosta CJ, Danovaro-Holliday MC, Baiqing D, Bhattacharya SK, Agtini MD, Bhutta ZA, Canh DG, Ali M, Shin S, Wain J, Page A-L, Albert MJ, Farrar J, Abu-Elyazeed R, Pang T, Galindo CM, von Seidlein L, Clemens JD, Domi Typhoid Study Group. A study of typhoid fever in five Asian countries: disease burden and implications for controls. Bull World Health Organ. 2008; 86(4): 260-8	Nonfatal Health Outcomes	Paratyphoid fever
Institute of Social Medicine and Health Policy, Shandong University, Shandong University School of Medicine, World Health Organization (WHO). China WHO Multi-country Survey Study on Health and Health System Responsiveness 2000-2001	Nonfatal Health Outcomes	Adverse effects of medical treatment
Huang S, Zheng Y, Foster PJ, Huang W, He M. Prevalence and causes of visual impairment in Chinese adults in urban southern China. Arch Ophthalmol. 2009; 127(10): 1362-7	Nonfatal Health Outcomes	
He M, Huang W, Zheng Y, Huang L, Ellwein LB. Refractive error and visual impairment in school children in rural southern China. Ophthalmology. 2007; 114(2): 374-82	Nonfatal Health Outcomes	
He M, Zeng J, Liu Y, Xu J, Pokharel GP, Ellwein LB. Refractive error and visual impairment in urban children in southern China. Invest Ophthalmol Vis Sci. 2004; 45(3): 793-9	Nonfatal Health Outcomes	
Institute of Social Medicine and Health Policy, Shandong University, Shandong University School of Medicine, World Health Organization (WHO). China WHO Multi-country Survey Study on Health and Health System Responsiveness 2000-2001	Nonfatal Health Outcomes	Visceral leishmaniasis
Ding D, Wang W, Wu J, Ma G, Dai X, Yang B, Wang T, Yuan C, Hong Z, de Boer HM, Prilipko L, Sander JW. Premature mortality in people with epilepsy in rural China: a prospective study. Lancet Neurol. 2006; 5(10): 823-7	Nonfatal Health Outcomes	Epilepsy
Khan MI, Ochiai RL, Von Seidlein L, Dong B, Bhattacharya SK, Agtini MD, Bhutta ZA, Do GC, Ali M, Kim DR, Favorov M, Clemens JD. Non-typhoidal Salmonella rates in febrile children at sites in five Asian countries: Non-typhoidal Salmonella in Asia. Trop Med Int Health. 2010; 15(8): 960Â–3	Nonfatal Health Outcomes	Invasive Non-typhoidal Salmonella (iNTS)
Institute of Social Medicine and Health Policy, Shandong University, Shandong University School of Medicine, World Health Organization (WHO). China WHO Multi-country Survey Study on Health and Health System Responsiveness 2000-2001	Nonfatal Health Outcomes	Other road injuries
Yang S, Hu Y, Han Y. Prevalence investigation of autism in children. J Appl Clin Pediatr. 2007; 22(24): 1872Â–1873	Nonfatal Health Outcomes	Autism spectrum disorders

World Health Organization (WHO). China World Health Survey 2002. Geneva, Switzerland: World Health Organization (WHO), 2005	Nonfatal Health Outcomes	Low back pain
World Health Organization (WHO). China World Health Survey 2002. Geneva, Switzerland: World Health Organization (WHO), 2005	Nonfatal Health Outcomes	Self-harm by other specified means
Cheng JQ, Zhou H, Hong FC, Zhang D, Zhang YJ, Pan P, Cai YM. Syphilis screening and intervention in 500,000 pregnant women in Shenzhen, the PeopleÂ's Republic of China. Sex Transm Infect. 2007; 83(5): 347Â–50	Nonfatal Health Outcomes	Sexually transmitted infections excluding HIV
Institute of Social Medicine and Health Policy, Shandong University, Shandong University School of Medicine, World Health Organization (WHO). China WHO Multi-country Survey Study on Health and Health System Responsiveness 2000-2001	Nonfatal Health Outcomes	Gastritis and duodenitis
Institute of Social Medicine and Health Policy, Shandong University, Shandong University School of Medicine, World Health Organization (WHO). China WHO Multi-country Survey Study on Health and Health System Responsiveness 2000-2001	Nonfatal Health Outcomes	Coal workers pneumoconiosis
Institute of Social Medicine and Health Policy, Shandong University, Shandong University School of Medicine, World Health Organization (WHO). China WHO Multi-country Survey Study on Health and Health System Responsiveness 2000-2001	Nonfatal Health Outcomes	Fire, heat, and hot substances
Zhou Y, Wang D, Liu S, Lu J, Zheng J, Zhong N, Ran P. The association between BMI and COPD: the results of two population-based studies in Guangzhou, China. COPD. 2013; 10(5): 567-72	Nonfatal Health Outcomes	Chronic obstructive pulmonary disease
World Health Organization (WHO). China World Health Survey 2002. Geneva, Switzerland: World Health Organization (WHO), 2005	Nonfatal Health Outcomes	Gallbladder and biliary diseases
Zhang D-R, Lia L-C, Zhou C, Wang L-Q, Wu A-R, Wu G, Luo H-Y, Rong D-S, Huang X-C, Cao Y-m, Su H, Zhang W. A comparative study on the epidemiological investigation of mental disorders in some areas of Guizhou province. J Guiyand Med Coll. 2003; 28(6): 516-518	Nonfatal Health Outcomes	Alcohol use disorders
Wang JH, Luo JY, Dong L, Gong J, Tong M. Epidemiology of gastroesophageal reflux disease: a general population-based study in Xi'an of Northwest China. World J Gastroenterol. 2004; 10(11): 1647-51	Nonfatal Health Outcomes	Gastroesophageal reflux disease
Xueqiang Fang YZ. Prevalence and risk factors of trichomoniasis, bacterial vaginosis, and candidiasis for married women of child-bearing age in rural Shandong. Jpn J Infect Dis. 2007; 60(5): 257-61	Nonfatal Health Outcomes	Gonococcal infection
Shi Q-C, Zhang J-M, Xu F-Z et al. Epidemiological survey of mental illnesses in the people aged 15 and older in Zhejiang Province. Chin J Prev Med. 2005; 39(4): 229-236	Nonfatal Health Outcomes	Schizophrenia
Zhang J-H. Epidemiological investigation on mental disorders in Shaoxing city. Med J Chin Peoples Health. 2003; 15(5): 313-4	Nonfatal Health Outcomes	Schizophrenia
Dong Y, Gao W, Nan H, Yu H, Li F, Duan W, Wang Y, Sun B, Qian R, Tuomilehto J, Qiao Q. Prevalence of Type 2 diabetes in urban and rural Chinese populations in Qingdao, China. Diabet Med. 2005; 22(10): 1427-33	Nonfatal Health Outcomes	Diabetes mellitus
Wang S-J, Wei Z-Y, Niu F Zhang G, Wei X-B, Zhang K-M, Cheng X-F, Xi X-Y, Wu Q-Y, Liu Q, Ning N-Y, Yu S-J, Gao K, Liu H-F, Zhang X-G, Guo H, Guo C-L, Liu X-H, Cai H-Y, Peng X-F. An epidemiological survey on schizophrenia in Fuyang, Anhui. J Clin Psychol Med. 2002; 12(1): 3-4	Nonfatal Health Outcomes	Schizophrenia
Chen R, Hu Z, Qin X, Xu X, Copeland JRM. A community-based study of depression in older people in Hefei, China Â– the GMS-AGECAT prevalence, case validation and socio-economic correlates. Int J Geriatr Psychiatry. 2004; 19(5): 407-13	Nonfatal Health Outcomes	Major depressive disorder
World Health Organization (WHO). China World Health Survey 2002. Geneva, Switzerland: World Health Organization (WHO), 2005	Nonfatal Health Outcomes	Near vision loss
Xiao J, Shen F, Xue Q, Chen G, Zeng K, Stone P, Zhao M, Chen Q. Is ethnicity a risk factor for developing preeclampsia? An analysis of the prevalence of preeclampsia in China. J Hum Hypertens. 2014; 28(11): 694Â–8	Nonfatal Health Outcomes	Maternal hypertensive disorders
Li L, Guan H, Xun P, Zhou J, Gu H. Prevalence and causes of visual impairment among the elderly in Nantong, China. Eye (Lond). 2008; 22(8): 1069-75	Nonfatal Health Outcomes	Cataract
Shi Q-C, Zhang J-M, Xu F-Z et al. Epidemiological survey of mental illnesses in the people aged 15 and older in Zhejiang Province. Chin J Prev Med. 2005; 39(4): 229-236	Nonfatal Health Outcomes	Alcohol use disorders
Zeng JH, Dai YS, Cao MQ, Hu XK. Serological markers of HCV and HBV and primary hepatocellular carcinoma. Modern Prev Med. 2003; 30: 324-5	Nonfatal Health Outcomes	Liver cancer due to hepatitis B
Li L, Guan H, Xun P, Zhou J, Gu H. Prevalence and causes of visual impairment among the elderly in Nantong, China. Eye (Lond). 2008; 22(8): 1069-75	Nonfatal Health Outcomes	Vision loss

Institute of Social Medicine and Health Policy, Shandong University, Shandong University School of Medicine, World Health Organization (WHO). China WHO Multi-country Survey Study on Health and Health System Responsiveness 2000-2001	Nonfatal Health Outcomes	Congenital heart anomalies
Bai Q, Xu B, Sun T, Zhang T. [Inspection on the one-month attack rate of community acquired pneumonia among old persons in Shanghai, China]. J Hyg Res. 2007; 36(4): 510Â–2	Nonfatal Health Outcomes	Lower respiratory infections
Institute of Social Medicine and Health Policy, Shandong University, Shandong University School of Medicine, World Health Organization (WHO). China WHO Multi-country Survey Study on Health and Health System Responsiveness 2000-2001	Nonfatal Health Outcomes	Other transport injuries
Institute of Social Medicine and Health Policy, Shandong University, Shandong University School of Medicine, World Health Organization (WHO). China WHO Multi-country Survey Study on Health and Health System Responsiveness 2000-2001	Nonfatal Health Outcomes	Peptic ulcer disease
Institute of Social Medicine and Health Policy, Shandong University, Shandong University School of Medicine, World Health Organization (WHO). China WHO Multi-country Survey Study on Health and Health System Responsiveness 2000-2001	Nonfatal Health Outcomes	Urolithiasis
Wei G, Liu S-M, Zhang W, Xiang Y, Huang X-Q, Yang C, Huang W-J, Xie W-J, He X, Su X-F, Wang J, Ciren P-C, Baima Z-G, Ci-P, Zha S, Liu X-H. Epidemiological investigation on mental disorders at Tibet in China: I major psychiatric disorders. Chin J Nerv Ment Dis. 2008; 34(10): 601-604	Nonfatal Health Outcomes	Schizophrenia
Mao Y-M, Luo Z-Y, Li B, Hu T-Y. Prospective Study on the Survival of HCC Patients Treated with Transcatheter Arterial Lipiodol Chemoembolization . Asian Pac J Cancer Prev. 2012; 13(3): 1039-42	Nonfatal Health Outcomes	Liver cancer due to hepatitis C
Institute of Social Medicine and Health Policy, Shandong University, Shandong University School of Medicine, World Health Organization (WHO). China WHO Multi-country Survey Study on Health and Health System Responsiveness 2000-2001	Nonfatal Health Outcomes	Other exposure to mechanical forces
Institute of Social Medicine and Health Policy, Shandong University, Shandong University School of Medicine, World Health Organization (WHO). China WHO Multi-country Survey Study on Health and Health System Responsiveness 2000-2001	Nonfatal Health Outcomes	Atrial fibrillation and flutter
Zhang J-X, Lu C-H, Tang J-S, Qiu H-M, Liu L-F, Wang S-B, Wang A-Z, Zhang T-L, Phillips M-R, Li X-Y, Zhang S-D, Jiang Y-H, Zhao C-Y, Hu B-W, Cao X-Y, Zhang Y, Gao C-N. Epidemiological survey of mental disorders in persons aged 18 years and older in Shandong Province. Chin Ment Health J. 2010; 24(3): 161-167	Nonfatal Health Outcomes	Schizophrenia
Institute of Social Medicine and Health Policy, Shandong University, Shandong University School of Medicine, World Health Organization (WHO). China WHO Multi-country Survey Study on Health and Health System Responsiveness 2000-2001	Nonfatal Health Outcomes	Self-harm
He N, Detels R, Zhu J, Jiang Q, Chen Z, Fang Y, Zhang X, Wu M, Zhao Q. Characteristics and sexually transmitted diseases of male rural migrants in a metropolitan area of Eastern China. Sex Transm Dis. 2005; 32(5): 286-92	Nonfatal Health Outcomes	Chlamydial infection
He N, Detels R, Zhu J, Jiang Q, Chen Z, Fang Y, Zhang X, Wu M, Zhao Q. Characteristics and sexually transmitted diseases of male rural migrants in a metropolitan area of Eastern China. Sex Transm Dis. 2005; 32(5): 286-92	Nonfatal Health Outcomes	Gonococcal infection
Tong D-Y, Wang X-H, Xu C-F, Yang Y-Z, Xiong S-D. Hepatitis B virus infection and coronary atherosclerosis: results from a population with relatively high prevalence of hepatitis B virus. World J Gastroenterol. 2005; 11(9): 1292-6	Nonfatal Health Outcomes	Acute hepatitis B
Du H, Li L, Bennett D, Guo Y, Key T, Bian Z, Sherliker P, Gao H, Chen Y, Yang L, Chen J, Wang S, Du R, Su H, Collins R, Peto R, Chen Z. Fresh Fruit Consumption and Major Cardiovascular Disease in China. N Engl J Med. 2016; 374(14): 1332-43	Nonfatal Health Outcomes	Ischemic stroke
World Health Organization (WHO). China World Health Survey 2002. Geneva, Switzerland: World Health Organization (WHO), 2005	Nonfatal Health Outcomes	Pedestrian road injuries
He N, Detels R, Zhu J, Jiang Q, Chen Z, Fang Y, Zhang X, Wu M, Zhao Q. Characteristics and sexually transmitted diseases of male rural migrants in a metropolitan area of Eastern China. Sex Transm Dis. 2005; 32(5): 286-92	Nonfatal Health Outcomes	Sexually transmitted infections excluding HIV
Chen Y-Q, Shen D-Y. Epidemiological Investigation on Mental Disorders in Shaoxing County. Strait J Prev Med. 2004; 10(4): 7-9	Nonfatal Health Outcomes	Schizophrenia
Demyttenaere K, Bruffaerts R, Lee S, Posada-Villa J, Kovess V, Angermeyer MC, Levinson D, de Girolamo G, Nakane H, Mneimneh Z, Lara C, de Graaf R, Scott KM, Gureje O, Stein DJ, Haro JM, Bromet EJ, Kessler RC, Alonso J, Von Korff M. Mental disorders among persons with chronic back or neck pain: results from the World Mental Health Surveys. Pain. 2007; 129(3): 332-42	Nonfatal Health Outcomes	Low back and neck pain

Institute of Social Medicine and Health Policy, Shandong University, Shandong University School of Medicine, World Health Organization (WHO). China WHO Multi-country Survey Study on Health and Health System Responsiveness 2000-2001	Nonfatal Health Outcomes	Animal contact
Lu Q, Zheng Y, Sun B, Cui T, Congdon N, Hu A, Chen J, Shi J. A population-based study of visual impairment among pre-school children in Beijing: the Beijing study of visual impairment in children. Am J Ophthalmol. 2009; 147(6): 1075-81	Nonfatal Health Outcomes	Refraction disorders
He Y, Jiang Y, Wang J, Fan L, Li X, Hu FB. Prevalence of peripheral arterial disease and its association with smoking in a population-based study in Beijing, China. J Vasc Surg. 2006; 44(2): 333-8	Nonfatal Health Outcomes	Peripheral artery disease
World Health Organization (WHO). China World Health Survey 2002. Geneva, Switzerland: World Health Organization (WHO), 2005	Nonfatal Health Outcomes	Inflammatory bowel disease
Cheng Q, Miao L, Zhang J, Ding SJ, Liu ZG, Wang X, Sun XJ, Zhao ZX, Song YJ, Ding XY, Guo ZL, Yang Y, Chen SD, Jiang GX, Fredrikson S. A population-based survey of multiple sclerosis in Shanghai, China. Neurology. 2007; 68(18): 1495-500	Nonfatal Health Outcomes	Multiple sclerosis
Simon GE, Goldberg DP, Von Korff M, UstÃ¼n TB. Understanding cross-national differences in depression prevalence. Psychol Med. 2002; 32(4): 585-94	Nonfatal Health Outcomes	Major depressive disorder
Lee S, Tsang A, Huang Y-Q, He Y-L, Liu ZR, Zhang M-Y, Shen Y-C, Kessler RC. The epidemiology of depression in metropolitan China. Psychol Med. 2009; 39(5): 735-47	Nonfatal Health Outcomes	Major depressive disorder
International Clearinghouse for Birth Defects Surveillance and Research. International Clearinghouse for Birth Defects Surveillance and Research Annual Report 2006. Rome, Italy: International Clearinghouse for Birth Defects Surveillance and Research, 2007	Nonfatal Health Outcomes	Orofacial clefts
Shen YC ZM, Huang YQ, He YL, Liu ZR, Cheng H, Tsang A, Lee S, Kessler RC. Twelve-month prevalence, severity, and unmet need for treatment of mental disorders in metropolitan China. Psychol Med. 2006; 36(2): 257-67	Nonfatal Health Outcomes	Dysthymia
World Health Organization (WHO). WHO World Mental Health Surveys: Global Perspectives on the Epidemiology of Mental Disorders. Cambridge, United Kingdom: Cambridge University Press, 2008	Nonfatal Health Outcomes	Anxiety disorders
Llibre Rodriguez JJ, Ferri CP, Acosta D, Guerra M, Huang Y, Jacob KS, Krishnamoorthy ES, Salas A, Sosa AL, Acosta I, Dewey ME, Gaona C, Jotheeswaran AT, Li S, Rodriguez D, Rodriguez G, Kumar PS, Valhuerdi A, Prince M, 10/66 Dementia Research Group. Prevalence of dementia in Latin America, India, and China: a population-based cross-sectional survey. Lancet. 2008; 372(9637): 464-74	Nonfatal Health Outcomes	Alzheimer's disease and other dementias
He Y-H, Chen Y-C, Jiang G-X, Huang H-E, Li R, Li X-Y, Ning G, Cheng Q. Evaluation of anthropometric indices for metabolic syndrome in Chinese adults aged 40Â years and over. Eur J Nutr. 2012; 51(1): 81Â–7	Nonfatal Health Outcomes	Diabetes mellitus
He Y-H, Jiang G-X, Yang Y, Huang H-E, Li R, Li X-Y, Ning G, Cheng Q. Obesity and its associations with hypertension and type 2 diabetes among Chinese adults age 40 years and over. Nutrition. 2009; 25(11-12): 1143Â–9	Nonfatal Health Outcomes	Diabetes mellitus
Wang C, Hou X, Bao Y, Pan J, Zuo Y, Zhong W, Jia W, Xiang K. The metabolic syndrome increased risk of cardiovascular events in Chinese--a community based study. Int J Cardiol. 2010; 139(2): 159Â–65	Nonfatal Health Outcomes	Diabetes mellitus
Prina A-M, Ferri C-P, Guerra M, Brayne C, Prince M. Prevalence of anxiety and its correlates among older adults in Latin America, India and China: cross-cultural study. Br J Psychiatry. 2011; 199(5): 485-491	Nonfatal Health Outcomes	Anxiety disorders
Xie J, Guan F, Wang J-H, Hu D-Y. Reasons for the upsetting cholesterol level during the community investigation from residents, physicians, and social aspects: the China Cholesterol Education Program (CCEP). Chin Med J (Engl). 2011; 124(19): 3030Â–4	Nonfatal Health Outcomes	Diabetes mellitus
Institute of Social Medicine and Health Policy, Shandong University, Shandong University School of Medicine, World Health Organization (WHO). China WHO Multi-country Survey Study on Health and Health System Responsiveness 2000-2001	Nonfatal Health Outcomes	Urinary tract infections
Li L, Guan H, Xun P, Zhou J, Gu H. Prevalence and causes of visual impairment among the elderly in Nantong, China. Eye (Lond). 2008; 22(8): 1069-75	Nonfatal Health Outcomes	Glaucoma
Li L, Guan H, Xun P, Zhou J, Gu H. Prevalence and causes of visual impairment among the elderly in Nantong, China. Eye (Lond). 2008; 22(8): 1069-75	Nonfatal Health Outcomes	Age-related macular degeneration
Lee S-L, Wong W, Lau Y-L. Increasing prevalence of allergic rhinitis but not asthma among children in Hong Kong from 1995 to 2001 (Phase 3 International Study of Asthma and Allergies in Childhood). Pediatr Allergy Immunol. 2004; 15(1): 72Â–8	Nonfatal Health Outcomes	Atopic dermatitis
Du H, Chen S-L, Bao C-D, Wang X-D, Lu Y, Gu Y-Y, Xu J-R, Chai W-M, Chen J, Nakamura H, Nishioka K. Prevalence and risk factors of knee osteoarthritis in Huang-Pu District, Shanghai, China. Rheumatol Int. 2005; 25(8): 585-90	Nonfatal Health Outcomes	Osteoarthritis

Demyttenaere K, Bruffaerts R, Lee S, Posada-Villa J, Kovess V, Angermeyer MC, Levinson D, de Girolamo G, Nakane H, Mneimneh Z, Lara C, de Graaf R, Scott KM, Gureje O, Stein DJ, Haro JM, Bromet EJ, Kessler RC, Alonso J, Von Korff M. Mental disorders among persons with chronic back or neck pain: results from the World Mental Health Surveys. Pain. 2007; 129(3): 332-42	Nonfatal Health Outcomes	Low back pain
Shi F, Gu K, Lu W, Weng W, Zhu M, Peng Y, Fu D, Fu H. Study on the prevalence of arthritis and relevant factors in Shanghai. Chin J Epidemiol. 2003; 24(12): 1136-40	Nonfatal Health Outcomes	Gout
Chen C, Luo Y-N, Liu X-B, Deng L-H, Li C-Y, Feng W-X, Cai L-R, Gu H. An Analysis of Alcohol Consumption Status and Alcohol Related Factors in the Pearl River Delta Area in China. Chin J Behav Med Sci. 2005; 14(8): 730-2	Nonfatal Health Outcomes	Alcohol use disorders
Xie C, Yang P, Wang Y. [Impact of implementation of Three Gorges Project on schistosomiasis endemic situation and control in Nanjing City]. Chin J Schistosomiasis Control. 2015; 27(6): 583Â–5, 607	Nonfatal Health Outcomes	Schistosomiasis
Zhang QH, Jiang ZX, Sun Y, Xia AX, Lin H, Tian L. Investigation and analysis of disease prevalence among 20 500 urban middle-aged and elderly women in five communities of Beijing. Chin J Clin Rehab. 2005; 9(39): 20-22	Nonfatal Health Outcomes	Gout
Lu Q, Zheng Y, Sun B, Cui T, Congdon N, Hu A, Chen J, Shi J. A population-based study of visual impairment among pre-school children in Beijing: the Beijing study of visual impairment in children. Am J Ophthalmol. 2009; 147(6): 1075-81	Nonfatal Health Outcomes	Other vision loss
United Nations Children's Fund (UNICEF). Child Mortality and Injury in Asia. New York, United States: United Nations Children's Fund (UNICEF), 2008. (Innocenti Working Papers: Special Series on Child Injuries Nos. 1-4)	Nonfatal Health Outcomes	Falls
World Health Organization (WHO). China World Health Survey 2002. Geneva, Switzerland: World Health Organization (WHO), 2005	Nonfatal Health Outcomes	Inguinal, femoral, and abdominal hernia
United Nations Children's Fund (UNICEF). Child Mortality and Injury in Asia. New York, United States: United Nations Children's Fund (UNICEF), 2008. (Innocenti Working Papers: Special Series on Child Injuries Nos. 1-4)	Nonfatal Health Outcomes	Drowning
United Nations Children's Fund (UNICEF). Child Mortality and Injury in Asia. New York, United States: United Nations Children's Fund (UNICEF), 2008. (Innocenti Working Papers: Special Series on Child Injuries Nos. 1-4)	Nonfatal Health Outcomes	Fire, heat, and hot substances
United Nations Children's Fund (UNICEF). Child Mortality and Injury in Asia. New York, United States: United Nations Children's Fund (UNICEF), 2008. (Innocenti Working Papers: Special Series on Child Injuries Nos. 1-4)	Nonfatal Health Outcomes	Poisonings
World Health Organization (WHO). China World Health Survey 2002. Geneva, Switzerland: World Health Organization (WHO), 2005	Nonfatal Health Outcomes	Neonatal preterm birth
World Health Organization (WHO). China World Health Survey 2002. Geneva, Switzerland: World Health Organization (WHO), 2005	Nonfatal Health Outcomes	Urolithiasis
World Health Organization (WHO). China World Health Survey 2002. Geneva, Switzerland: World Health Organization (WHO), 2005	Nonfatal Health Outcomes	Genital prolapse
United Nations Children's Fund (UNICEF). Child Mortality and Injury in Asia. New York, United States: United Nations Children's Fund (UNICEF), 2008. (Innocenti Working Papers: Special Series on Child Injuries Nos. 1-4)	Nonfatal Health Outcomes	Animal contact
International Clearinghouse for Birth Defects Monitoring Systems. International Clearinghouse for Birth Defects Monitoring Systems Annual Report 2004. Rome, Italy: International Centre on Birth Defects, 2006	Nonfatal Health Outcomes	Down syndrome
World Health Organization (WHO). China World Health Survey 2002. Geneva, Switzerland: World Health Organization (WHO), 2005	Nonfatal Health Outcomes	Benign prostatic hyperplasia
International Clearinghouse for Birth Defects Surveillance and Research. International Clearinghouse for Birth Defects Surveillance and Research Annual Report 2005. Rome, Italy: International Clearinghouse for Birth Defects Surveillance and Research, 2007	Nonfatal Health Outcomes	Down syndrome
Institute of Social Medicine and Health Policy, Shandong University, Shandong University School of Medicine, World Health Organization (WHO). China WHO Multi-country Survey Study on Health and Health System Responsiveness 2000-2001	Nonfatal Health Outcomes	Environmental heat and cold exposure
World Health Organization (WHO). China World Health Survey 2002. Geneva, Switzerland: World Health Organization (WHO), 2005	Nonfatal Health Outcomes	Cystic echinococcosis
World Health Organization (WHO). China World Health Survey 2002. Geneva, Switzerland: World Health Organization (WHO), 2005	Nonfatal Health Outcomes	Fire, heat, and hot substances
United Nations Children's Fund (UNICEF). Child Mortality and Injury in Asia. New York, United States: United Nations Children's Fund (UNICEF), 2008. (Innocenti Working Papers: Special Series on Child Injuries Nos. 1-4)	Nonfatal Health Outcomes	Interpersonal violence
Wang Q, Tan Y, Ren Y, Dong L, Xie Z, Tang L, Cao D, Zhang W, Hu H, Wang H. Zinc finger protein ZBTB20 expression is increased in hepatocellular carcinoma and associated with poor prognosis . BMC Cancer. 2011; 11(1): 271	Nonfatal Health Outcomes	Liver cancer due to hepatitis B

Wang Q, Tan Y, Ren Y, Dong L, Xie Z, Tang L, Cao D, Zhang W, Hu H, Wang H. Zinc finger protein ZBTB20 expression is increased in hepatocellular carcinoma and associated with poor prognosis . BMC Cancer. 2011; 11(1): 271	Nonfatal Health Outcomes	Liver cancer due to hepatitis C
Lu Q, Zheng Y, Sun B, Cui T, Congdon N, Hu A, Chen J, Shi J. A population-based study of visual impairment among pre-school children in Beijing: the Beijing study of visual impairment in children. Am J Ophthalmol. 2009; 147(6): 1075-81	Nonfatal Health Outcomes	Vision loss
Institute of Social Medicine and Health Policy, Shandong University, Shandong University School of Medicine, World Health Organization (WHO). China WHO Multi-country Survey Study on Health and Health System Responsiveness 2000-2001	Nonfatal Health Outcomes	Physical violence by other means
Wang B-E, Ma W-M, Sulaiman A, Noer S, Sumoharjo S, Sumarsidi D, Tandon BN, Nakao K, Mishiro S, Miyakawa Y, Akahane Y, Suzuki H. Demographic, clinical, and virological characteristics of hepatocellular carcinoma in Asia: survey of 414 patients from four countries. J Med Virol. 2002; 67(3): 394-400	Nonfatal Health Outcomes	Liver cancer due to hepatitis B
Wang B-E, Ma W-M, Sulaiman A, Noer S, Sumoharjo S, Sumarsidi D, Tandon BN, Nakao K, Mishiro S, Miyakawa Y, Akahane Y, Suzuki H. Demographic, clinical, and virological characteristics of hepatocellular carcinoma in Asia: survey of 414 patients from four countries. J Med Virol. 2002; 67(3): 394-400	Nonfatal Health Outcomes	Liver cancer due to hepatitis C
Institute of Social Medicine and Health Policy, Shandong University, Shandong University School of Medicine, World Health Organization (WHO). China WHO Multi-country Survey Study on Health and Health System Responsiveness 2000-2001	Nonfatal Health Outcomes	Lower respiratory infections
World Health Organization (WHO). China World Health Survey 2002. Geneva, Switzerland: World Health Organization (WHO), 2005	Nonfatal Health Outcomes	Uterine fibroids
World Health Organization (WHO). China World Health Survey 2002. Geneva, Switzerland: World Health Organization (WHO), 2005	Nonfatal Health Outcomes	Urinary tract infections
World Health Organization (WHO). China World Health Survey 2002. Geneva, Switzerland: World Health Organization (WHO), 2005	Nonfatal Health Outcomes	Endocarditis
World Health Organization (WHO). China World Health Survey 2002. Geneva, Switzerland: World Health Organization (WHO), 2005	Nonfatal Health Outcomes	Ischemic heart disease
Hon KL, Leung E, Hung E, Tang J, Chow CM, Leung TF, Cheung KL, Ng PC. Premorbid factors and outcome associated with respiratory virus infections in a pediatric intensive care unit. Pediatr Pulmonol. 2008; 43(3): 275-80	Nonfatal Health Outcomes	Lower respiratory infections
World Health Organization (WHO). China World Health Survey 2002. Geneva, Switzerland: World Health Organization (WHO), 2005	Nonfatal Health Outcomes	Venomous animal contact
Woo J, Leung J, Lau E. Prevalence and correlates of musculoskeletal pain in Chinese elderly and the impact on 4-year physical function and quality of life. Public Health. 2009; 123(8): 549-56	Nonfatal Health Outcomes	Low back and neck pain
Chiu TTW, Leung ASL. Neck pain in Hong Kong: a telephone survey on prevalence, consequences, and risk groups. Spine. 2006; 31(16): E540-544	Nonfatal Health Outcomes	Low back and neck pain
Lee S-L, Wong W, Lau Y-L. Increasing prevalence of allergic rhinitis but not asthma among children in Hong Kong from 1995 to 2001 (Phase 3 International Study of Asthma and Allergies in Childhood). Pediatr Allergy Immunol. 2004; 15(1): 72Â–8	Nonfatal Health Outcomes	Dermatitis
Wang W, Zhai L, Zheng L, Zhu J, Qu X, Feng Q, et al. An epidemiology survey of autism in Jiangsu province. Chin Behav Sci. 2003; 12(2): 173Â–174	Nonfatal Health Outcomes	Autism spectrum disorders
Lu Q, Zheng Y, Sun B, Cui T, Congdon N, Hu A, Chen J, Shi J. A population-based study of visual impairment among pre-school children in Beijing: the Beijing study of visual impairment in children. Am J Ophthalmol. 2009; 147(6): 1075-81	Nonfatal Health Outcomes	
Yongjun T, Samuelson J, Qingsheng D, Ali MM, Li X, Yanjian M, Xiaoqing C, Jun L, Jian C, Bi L. The prevalence of sexually transmitted and other lower reproductive tract infections among rural women in Sichuan Province, China. Southeast Asian J Trop Med Public Health. 2009; 40(5): 1038-47	Nonfatal Health Outcomes	Syphilis
Li YM, Du J, Zhang H, Yu CH. Epidemiological investigation in outpatients with symptomatic gastroesophageal reflux from the Department of Medicine in Zhejiang Province, east China. J Gastroenterol Hepatol. 2008; 23(2): 283-9	Nonfatal Health Outcomes	Gastroesophageal reflux disease
World Health Organization (WHO). China World Health Survey 2002. Geneva, Switzerland: World Health Organization (WHO), 2005	Nonfatal Health Outcomes	Environmental heat and cold exposure
Wang X-Y, Xu Z-Y, Seidlein L von, Zhang Y-L, Zhao S-J, Hao Z-Y, Han OP, Kilgore P, Xing Z-C, Han C-Q, Ma J-C, Chen J-C, Clemens J. Incidence of Diarrhea Caused by Rotavirus Infections in Rural Zhengding, China: Prospective, Population-Based Surveillance. J Infect Dis. 2005; 192(Supplement 1): S100Â–5	Nonfatal Health Outcomes	Diarrheal diseases
World Health Organization (WHO). China World Health Survey 2002. Geneva, Switzerland: World Health Organization (WHO), 2005	Nonfatal Health Outcomes	Down syndrome
World Health Organization (WHO). China World Health Survey 2002. Geneva, Switzerland: World Health Organization (WHO), 2005	Nonfatal Health Outcomes	Congenital heart anomalies

World Health Organization (WHO). China World Health Survey 2002. Geneva, Switzerland: World Health Organization (WHO), 2005	Nonfatal Health Outcomes	Intracerebral hemorrhage
World Health Organization (WHO). China World Health Survey 2002. Geneva, Switzerland: World Health Organization (WHO), 2005	Nonfatal Health Outcomes	Syphilis
World Health Organization (WHO). China World Health Survey 2002. Geneva, Switzerland: World Health Organization (WHO), 2005	Nonfatal Health Outcomes	Other pneumoconiosis
He N, Detels R, Zhu J, Jiang Q, Chen Z, Fang Y, Zhang X, Wu M, Zhao Q. Characteristics and sexually transmitted diseases of male rural migrants in a metropolitan area of Eastern China. Sex Transm Dis. 2005; 32(5): 286-92	Nonfatal Health Outcomes	Syphilis
International Clearinghouse for Birth Defects Monitoring Systems. International Clearinghouse for Birth Defects Monitoring Systems Annual Report 2003. Rome, Italy: International Centre on Birth Defects	Nonfatal Health Outcomes	Orofacial clefts
International Clearinghouse for Birth Defects Surveillance and Research. International Clearinghouse for Birth Defects Surveillance and Research Annual Report 2005. Rome, Italy: International Clearinghouse for Birth Defects Surveillance and Research, 2007	Nonfatal Health Outcomes	Orofacial clefts
Chuang S-Y, Chen C-H, Cheng C-M, Chou P. Combined use of brachial-ankle pulse wave velocity and ankle-brachial index for fast assessment of arteriosclerosis and atherosclerosis in a community. Int J Cardiol. 2005; 98(1): 99-105	Nonfatal Health Outcomes	Peripheral artery disease
International Clearinghouse for Birth Defects Monitoring Systems. International Clearinghouse for Birth Defects Monitoring Systems Annual Report 2003. Rome, Italy: International Centre on Birth Defects	Nonfatal Health Outcomes	Down syndrome
International Clearinghouse for Birth Defects Surveillance and Research. International Clearinghouse for Birth Defects Surveillance and Research Annual Report 2006. Rome, Italy: International Clearinghouse for Birth Defects Surveillance and Research, 2007	Nonfatal Health Outcomes	Down syndrome
World Health Organization (WHO). China World Health Survey 2002. Geneva, Switzerland: World Health Organization (WHO), 2005	Nonfatal Health Outcomes	Atrial fibrillation and flutter
Liu S,Â Zhou Y,Â Wang X,Â Wang D,Â Lu J,Â Zheng J,Â Zhong N,Â Ran P. Biomass fuels are the probable risk factor for chronic obstructive pulmonary disease in rural South China. Thorax. 2007; 62(10): 889-97	Nonfatal Health Outcomes	Chronic obstructive pulmonary disease
International Clearinghouse for Birth Defects Monitoring Systems. International Clearinghouse for Birth Defects Monitoring Systems Annual Report 2003. Rome, Italy: International Centre on Birth Defects	Nonfatal Health Outcomes	Congenital musculoskeletal and limb anomalies
International Clearinghouse for Birth Defects Surveillance and Research. International Clearinghouse for Birth Defects Surveillance and Research Annual Report 2006. Rome, Italy: International Clearinghouse for Birth Defects Surveillance and Research, 2007	Nonfatal Health Outcomes	Congenital musculoskeletal and limb anomalies
International Clearinghouse for Birth Defects Surveillance and Research. International Clearinghouse for Birth Defects Surveillance and Research Annual Report 2006. Rome, Italy: International Clearinghouse for Birth Defects Surveillance and Research, 2007	Nonfatal Health Outcomes	Neural tube defects
International Clearinghouse for Birth Defects Monitoring Systems. International Clearinghouse for Birth Defects Monitoring Systems Annual Report 2004. Rome, Italy: International Centre on Birth Defects, 2006	Nonfatal Health Outcomes	Urogenital congenital anomalies
International Clearinghouse for Birth Defects Surveillance and Research. International Clearinghouse for Birth Defects Surveillance and Research Annual Report 2005. Rome, Italy: International Clearinghouse for Birth Defects Surveillance and Research, 2007	Nonfatal Health Outcomes	Urogenital congenital anomalies
Hong Kong Cancer Registry. Hong Kong Cancer Registry Annual Tables. Kowloon, Hong Kong: Hong Kong Cancer Registry	Nonfatal Health Outcomes	Lip and oral cavity cancer
Hong Kong Cancer Registry. Hong Kong Cancer Registry Annual Tables. Kowloon, Hong Kong: Hong Kong Cancer Registry	Nonfatal Health Outcomes	Larynx cancer
Hong Kong Cancer Registry. Hong Kong Cancer Registry Annual Tables. Kowloon, Hong Kong: Hong Kong Cancer Registry	Nonfatal Health Outcomes	Kidney cancer
Keqing L, Ze C, Lijun C, Qinpu J, Guang S, Haoran W, Jing H, Wuwen Z, Jianguo X, Yanping Z, Ben Z, Jianxun J, Xueyi W, Jun T, Yufu Z, Haishan H, Jianping G, Enyi Z. Epidemiological survey of mental disorders in the people aged 18 and older in Hebei Province. Asian J Psychiatr. 2008; 1(2): 51-5	Nonfatal Health Outcomes	Bipolar disorder
Keqing L, Ze C, Lijun C, Qinpu J, Guang S, Haoran W, Jing H, Wuwen Z, Jianguo X, Yanping Z, Ben Z, Jianxun J, Xueyi W, Jun T, Yufu Z, Haishan H, Jianping G, Enyi Z. Epidemiological survey of mental disorders in the people aged 18 and older in Hebei Province. Asian J Psychiatr. 2008; 1(2): 51-5	Nonfatal Health Outcomes	Anorexia nervosa

Keqing L, Ze C, Lijun C, Qinpu J, Guang S, Haoran W, Jing H, Wuwen Z, Jianguo X, Yanping Z, Ben Z, Jianxun J, Xueyi W, Jun T, Yufu Z, Haishan H, Jianping G, Enyi Z. Epidemiological survey of mental disorders in the people aged 18 and older in Hebei Province. Asian J Psychiatr. 2008; 1(2): 51-5	Nonfatal Health Outcomes	Bulimia nervosa
International Clearinghouse for Birth Defects Surveillance and Research. International Clearinghouse for Birth Defects Surveillance and Research Annual Report 2005. Rome, Italy: International Clearinghouse for Birth Defects Surveillance and Research, 2007	Nonfatal Health Outcomes	Congenital heart anomalies
Institute of Social Medicine and Health Policy, Shandong University, Shandong University School of Medicine, World Health Organization (WHO). China WHO Multi-country Survey Study on Health and Health System Responsiveness 2000-2001	Nonfatal Health Outcomes	Benign prostatic hyperplasia
Institute of Social Medicine and Health Policy, Shandong University, Shandong University School of Medicine, World Health Organization (WHO). China WHO Multi-country Survey Study on Health and Health System Responsiveness 2000-2001	Nonfatal Health Outcomes	Unintentional firearm injuries
Liu J, Yang X, Jia M, Qu C, Shi J, Liu G, et al. Survey on pervasive developmental disorder in 2-6 year-old children in Beijing. Chin Ment Health J. 2007; 21(5): 290Â–293	Nonfatal Health Outcomes	Autism spectrum disorders
World Health Organization (WHO). China World Health Survey 2002. Geneva, Switzerland: World Health Organization (WHO), 2005	Nonfatal Health Outcomes	Turner syndrome
Ding BG, Fan DM, Mu LN, Yu SZ. A caseÂ–control study of hepatitis B virus, hepatitis C virus and liver cancer. Chin J Epidemiol. 2004; 25(22)	Nonfatal Health Outcomes	Liver cancer due to hepatitis B
Lu M, Yao W, Zhong N, Zhou Y, Wang C, Chen P, Kang J, Huang S, Chen B, Wang C, Ni D, Wang X, Wang D, Liu S, LÃ¼ J, Shen N, Ding Y, Ran P. Asymptomatic patients of chronic obstructive pulmonary disease in China. Chin Med J (Engl). 2010; 123(12): 1494-9	Nonfatal Health Outcomes	Chronic obstructive pulmonary disease
Wang X-L, Yang M-F, Yang H, Jiang R-F, Liu Y-D, Bai S-F, Lin Y-Z, Wu L-Z, Liu A-C, Lv M-Y. The Prevalence of ADHD Among Pupils in Xiamen. Chin J Sch Health. 2007; 28(11): 995-6	Nonfatal Health Outcomes	Attention-deficit/hyperactivity disorder
Xu J, Chen N, Feng T, Wang E, Wu X, Chen H, Wang T, Zhou X, Zheng J. [Effectiveness of routinely used assays for the diagnosis of schistosomiasis japonica in the field]. Chin J Parasitol Parasit Dis. 2007; 25(3): 175Â–9	Nonfatal Health Outcomes	Schistosomiasis
World Health Organization (WHO). China World Health Survey 2002. Geneva, Switzerland: World Health Organization (WHO), 2005	Nonfatal Health Outcomes	Non-venomous animal contact
Shi Q-C, Zhang J-M, Xu F-Z et al. Epidemiological survey of mental illnesses in the people aged 15 and older in Zhejiang Province. Chin J Prev Med. 2005; 39(4): 229-236	Nonfatal Health Outcomes	Bipolar disorder
World Health Organization (WHO). China World Health Survey 2002. Geneva, Switzerland: World Health Organization (WHO), 2005	Nonfatal Health Outcomes	Maternal abortion, miscarriage, and ectopic pregnancy
Zhu G, Qu Y, Jin N, Sun Z, Liu T, Lee H, Tian M, Wang T. Seroepidemiology and molecular characterization of hepatitis E virus in Jilin, China. Infection. 2008; 36(2): 140-6	Nonfatal Health Outcomes	Acute hepatitis E
Tang W, Liu Q-J, Wen X-T, Zhang C-Y, Yan X-L, Zhang B-Q, Sha B, Ye T-M, Lin C-C, Tu X-D, Chen D-S, Chen G-D, Ye J-C, Sun H-B. Epidemiological survey on mental disorders in Wenzhou area. Shanghai Arch Psychiatry. 2005; 17(4): 197-9	Nonfatal Health Outcomes	Alcohol use disorders
Hong S, Xin C, Qianhong Y, Yanan W, Wenyan X, Peeling RW, Mabey D. Pelvic inflammatory disease in the People's Republic of China: aetiology and management. Int J STD AIDS. 2002; 13(8): 568-72	Nonfatal Health Outcomes	Gonococcal infection
Lu L, Shi Q-J, Zhong Y-F, Wang Z-M, Chen Z-Y. Attention deficit hyperactivity disorder and the related factors in children in Wuhan city: an analysis of 2199 questionnaires coming from 12 grades. Chin J Clin Rehab. 2005; 9(12): 116-118	Nonfatal Health Outcomes	Attention-deficit/hyperactivity disorder
Fan S, Dong Y. Screening report for STD in 1000 pregnant women. Beijing Med J. 1991; 76	Nonfatal Health Outcomes	Sexually transmitted infections excluding HIV
Xu G, Huang Y, Chen Y, Du F. Treatment gap of epilepsy in a rural Chinese community: A population based study. Epilepsia. 2003; 44(Suppl 8): 194	Nonfatal Health Outcomes	Epilepsy
Lin CX, Li YS, Zhang RY, Cheng Y, Li L. [Investigation on Paragonimiasis infection in 6 counties of Fujian Province]. Chin J Parasitol Parasit Dis. 2003; 23(3): 191	Nonfatal Health Outcomes	Food-borne trematodiases
Wu Z, Rotheram-Borus MJ, Li L, Guan J, Detels R, Yin Y, Wu S, Liu Z, Lin C, Hsieh J, NIMH Collaborative HIV/STD Prevention Trial Group. Sexually transmitted diseases and risk behaviors among market vendors in China. Sex Transm Dis. 2007; 34(12): 1030-4	Nonfatal Health Outcomes	Gonococcal infection
Wu Z, Rotheram-Borus MJ, Li L, Guan J, Detels R, Yin Y, Wu S, Liu Z, Lin C, Hsieh J, NIMH Collaborative HIV/STD Prevention Trial Group. Sexually transmitted diseases and risk behaviors among market vendors in China. Sex Transm Dis. 2007; 34(12): 1030-4	Nonfatal Health Outcomes	Trichomoniasis

Liu J, Jia MX, Yang XL, Shao CX, Li Y, Shi JL, Qu CY, Zhang ZX, Zhang XL, Liang AM. A 3-year follow-up study of 2-6 years children with diagnosis of pervasive developmental disorder. Chin Ment Health J. 2012; 26(6): 460Â–465	Nonfatal Health Outcomes	Autism spectrum disorders
Yongjun T, Samuelson J, Qingsheng D, Ali MM, Li X, Yanjian M, Xiaoqing C, Jun L, Jian C, Bi L. The prevalence of sexually transmitted and other lower reproductive tract infections among rural women in Sichuan Province, China. Southeast Asian J Trop Med Public Health. 2009; 40(5): 1038-47	Nonfatal Health Outcomes	Gonococcal infection
Zhang T, Zhao W, Yang D, Piao D, Huang S, Mi Y, Zhao X, Cao J, Shen Y, Zhang W, Liu A. Human cystic echinococcosis in Heilongjiang Province, China: a retrospective study. BMC Gastroenterol. 2015; 15(1): 29	Nonfatal Health Outcomes	Cystic echinococcosis
Llibre Rodriguez JJ, Ferri CP, Acosta D, Guerra M, Huang Y, Jacob KS, Krishnamoorthy ES, Salas A, Sosa AL, Acosta I, Dewey ME, Gaona C, Jotheeswaran AT, Li S, Rodriguez D, Rodriguez G, Kumar PS, Valhuerdi A, Prince M, 10/66 Dementia Research Group. Prevalence of dementia in Latin America, India, and China: a population-based cross-sectional survey. Lancet. 2008; 372(9637): 464-74	Nonfatal Health Outcomes	Down syndrome
Zhang L, Ruan Y, Jiang Z, Yang Z, Liu S, Zhou F, He Y, Yin L, Qin G, Shao Y. [An 1-year prospective cohort study on mortality of injecting drug users]. Chin J Epidemiol. 2005; 26(3): 190-3	Nonfatal Health Outcomes	Opioid use disorders
Zhang W, Tang M-N, Qiu C-J. Han H-Y, Dai L. Lu J, Wu S, Wang S-H, Chen J-M, Guo L-J. Ding Y-Q, Li S-X, Liu X-H. The Prevalence of Depressive Disorder among Residents aged 55 or over in Chengdu Area. Chin J Geriatr. 2004; 23(12): 883-5	Nonfatal Health Outcomes	Dysthymia
Kong F-Y, Tao W-D, Hao Z-L, Liu M. Predictors of one-year disability and death in Chinese hospitalized women after ischemic stroke. Cerebrovasc Dis. 2010; 29(3): 255Â–62	Nonfatal Health Outcomes	Intracerebral hemorrhage
Xueqiang Fang YZ. Prevalence and risk factors of trichomoniasis, bacterial vaginosis, and candidiasis for married women of child-bearing age in rural Shandong. Jpn J Infect Dis. 2007; 60(5): 257-61	Nonfatal Health Outcomes	Trichomoniasis
Pan G-W, Jiang C, Yang C-L, Na J, Fu Z-G, Yu F-Q, Yu C-Y, Liu M-J, Liu H, Wang P, Li N, Yan T-H, Zhang S-J. Epidemiological survey of mental disorders in urban and rural areas of Liaoning province. Chin J Public Health. 2006; 22(12): 1505-1507	Nonfatal Health Outcomes	Alcohol use disorders
International Clearinghouse for Birth Defects Surveillance and Research. International Clearinghouse for Birth Defects Surveillance and Research Annual Report 2006. Rome, Italy: International Clearinghouse for Birth Defects Surveillance and Research, 2007	Nonfatal Health Outcomes	Urogenital congenital anomalies
Chen XS, Yin YP, Chen LP, Thuy NT, Zhang GY, Shi MQ, Hu LH, Yu YH. Sexually transmitted infections among pregnant women attending an antenatal clinic in Fuzhou, China. Sex Transm Dis. 2006; 33(5): 296-301	Nonfatal Health Outcomes	Syphilis
Wu Z, Rotheram-Borus MJ, Li L, Guan J, Detels R, Yin Y, Wu S, Liu Z, Lin C, Hsieh J, NIMH Collaborative HIV/STD Prevention Trial Group. Sexually transmitted diseases and risk behaviors among market vendors in China. Sex Transm Dis. 2007; 34(12): 1030-4	Nonfatal Health Outcomes	Syphilis
Zhao S, Li Z, Zhou S, Zheng C, Ma H. Epidemiological Feature of Visceral Leishmaniasis in China, 2004-2012. Iran J Public Health. 2015; 44(1): 51-9	Nonfatal Health Outcomes	Visceral leishmaniasis
Zhu C, Kang W, Xu F, Cheng X, Zhang Z, Jia L, Ji L, Guo X, Xiong H, Simbruner G, Blomgren K, Wang X. Erythropoietin Improved Neurologic Outcomes in Newborns With Hypoxic-Ischemic Encephalopathy. Pediatrics. 2009; 124(2): e218-e226	Nonfatal Health Outcomes	Neonatal encephalopathy due to birth asphyxia and trauma
Yang X, Jiang C, Fu Z, Yu Q, Yu C, Yan T. [Alcohol Use Disorder and its Influence Factors Among Residents in Liaoning Province]. Chin J Public Health. 2011; 27(7): 935Â–7	Nonfatal Health Outcomes	Alcohol use disorders
International Clearinghouse for Birth Defects Monitoring Systems. International Clearinghouse for Birth Defects Monitoring Systems Annual Report 2003. Rome, Italy: International Centre on Birth Defects	Nonfatal Health Outcomes	Urogenital congenital anomalies
Wu Z, Rotheram-Borus MJ, Li L, Guan J, Detels R, Yin Y, Wu S, Liu Z, Lin C, Hsieh J, NIMH Collaborative HIV/STD Prevention Trial Group. Sexually transmitted diseases and risk behaviors among market vendors in China. Sex Transm Dis. 2007; 34(12): 1030-4	Nonfatal Health Outcomes	Genital herpes
Xuan S-Y, Xin Y-N, Chen H, Shi G-J, Guan H-S, Li Y. Significance of hepatitis B virus surface antigen, hepatitis C virus expression in hepatocellular carcinoma and pericarcinomatous tissues . World J Gastroenterol. 2007; 13(12): 1870	Nonfatal Health Outcomes	Liver cancer due to NASH
Kong F-Y, Tao W-D, Hao Z-L, Liu M. Predictors of one-year disability and death in Chinese hospitalized women after ischemic stroke. Cerebrovasc Dis. 2010; 29(3): 255Â–62	Nonfatal Health Outcomes	Stroke
Dongfeng G, Guangyong H, Jiang H, Xigui W, Xiufang D, Macmahon S, Whelton PK. Investigation of Prevalence and Distributing Feature of Chronic Heart Failure in Chinese Adult Population. Chin J Cardiol. 2003; 31(1): 3Â–6	Nonfatal Health Outcomes	Heart failure

Tao LL, Hu BJ, He LX, Wei L, Xie HM, Wang BQ, Li HY, Chen XH, Zhou CM, Deng WW. Etiology and antimicrobial resistance of community-acquired pneumonia in adult patients in China. Chin Med J (Engl). 2012; 125(17): 2967-72	Nonfatal Health Outcomes	Lower respiratory infections
Jia T-W, Zhou X-N, Wang X-H, Utzinger J, Steinmann P, Wu X-H. Assessment of the age-specific disability weight of chronic schistosomiasis japonica. Bull World Health Organ. 2007; 85(6): 458-65	Nonfatal Health Outcomes	Schistosomiasis
United Nations Children's Fund (UNICEF). Child Mortality and Injury in Asia. New York, United States: United Nations Children's Fund (UNICEF), 2008. (Innocenti Working Papers: Special Series on Child Injuries Nos. 1-4)	Nonfatal Health Outcomes	Road injuries
Ministry of Health (China). Report on the National Survey of Current Situation of Major Human Parasitic Diseases in China. Beijing, China: Chinese Center for Disease Control and Prevention (CCDC), 2005	Nonfatal Health Outcomes	Food-borne trematodiases
Detels R, Wu Z, Rotheram MJ, Li L, Guan J, Yin Y, Liang G, Lee M, Hu L, National Institute of Mental Health (NIMH) Collaborative HIV Prevention Trial Group. Sexually transmitted disease prevalence and characteristics of market vendors in eastern China. Sex Transm Dis. 2003; 30(11): 803-8	Nonfatal Health Outcomes	Chlamydial infection
Detels R, Wu Z, Rotheram MJ, Li L, Guan J, Yin Y, Liang G, Lee M, Hu L, National Institute of Mental Health (NIMH) Collaborative HIV Prevention Trial Group. Sexually transmitted disease prevalence and characteristics of market vendors in eastern China. Sex Transm Dis. 2003; 30(11): 803-8	Nonfatal Health Outcomes	Gonococcal infection
Fan S, Dong Y. Screening report for STD in 1000 pregnant women. Beijing Med J. 1991; 76	Nonfatal Health Outcomes	Gonococcal infection
Detels R, Wu Z, Rotheram MJ, Li L, Guan J, Yin Y, Liang G, Lee M, Hu L, National Institute of Mental Health (NIMH) Collaborative HIV Prevention Trial Group. Sexually transmitted disease prevalence and characteristics of market vendors in eastern China. Sex Transm Dis. 2003; 30(11): 803-8	Nonfatal Health Outcomes	Trichomoniasis
Hong Kong Cancer Registry. Hong Kong Cancer Registry Annual Tables. Kowloon, Hong Kong: Hong Kong Cancer Registry	Nonfatal Health Outcomes	Thyroid cancer
World Health Organization (WHO). China World Health Survey 2002. Geneva, Switzerland: World Health Organization (WHO), 2005	Nonfatal Health Outcomes	Asthma
Chen Y-D, Liu M-Y, Yu W-L, Li J-Q, Peng M, Dai Q, Liu X, Zhou Z-Q. Hepatitis C virus infections and genotypes in China. Hepatobiliary Pancreat Dis Int. 2002; 1(2): 194-201	Nonfatal Health Outcomes	Cirrhosis and other chronic liver diseases due to hepatitis C
Institute of Social Medicine and Health Policy, Shandong University, Shandong University School of Medicine, World Health Organization (WHO). China WHO Multi-country Survey Study on Health and Health System Responsiveness 2000-2001	Nonfatal Health Outcomes	Endocrine, metabolic, blood, and immune disorders
Xuan S-Y, Xin Y-N, Chen H, Shi G-J, Guan H-S, Li Y. Significance of hepatitis B virus surface antigen, hepatitis C virus expression in hepatocellular carcinoma and pericarcinomatous tissues . World J Gastroenterol. 2007; 13(12): 1870	Nonfatal Health Outcomes	Liver cancer due to hepatitis B
Xiang Y-T, Ma X, Lu J-Y, Cai Z-J, Li S-R, Xiang Y-Q, Guo H-L, Hou Y-Z, Li Z-B, Li Z-J, Tao Y-F, Dang W-M, Wu X-M, Deng J, Lai KYC, Ungvari GS. Alcohol-related disorders in Beijing, China: prevalence, socio-demographic correlates, and unmet need for treatment. Alcohol Clin Exp Res. 2009; 33(6): 1111-8	Nonfatal Health Outcomes	Alcohol use disorders
Liu S-M, Wei G, Zhang W, Xiang Y, Huang X-Q, Yang C, Huang W-J, Xie W-J, He X, Su X-F, Wang J, Ciren P, Bima Z, Ci P, Za S, Liu X-H. [Epidemiological Survey on Neuropsychiatric Disorders in Tibet of China: Neuroses, Alcohol-related Disorders, Mental Retardation and Epilepsy]. J Sichuan Univ Med Sci Ed. 2012; 43(2): 210-3	Nonfatal Health Outcomes	Alcohol use disorders
Hao W, Su Z, Liu B, Zhang K, Yang H, Chen S, Biao M, Cui C. Drinking and drinking patterns and health status in the general population of five areas of China. Alcohol Alcohol. 2004; 39(1): 43-52	Nonfatal Health Outcomes	Alcohol use disorders
Chen C-C, Yin S-J. Alcohol abuse and related factors in Asia. Int Rev Psychiatry. 2008; 20(5): 425-33	Nonfatal Health Outcomes	Alcohol use disorders
Su Z-H, Hao W, Chen H-X. Alcohol patterns, alcohol consumption and alcohol-related problems in 5 areas in China: 3. problems related to alcohol use in general populationy. Chin Ment Health J. 2003; 17(8): 544-546	Nonfatal Health Outcomes	Alcohol use disorders
Institute of Social Medicine and Health Policy, Shandong University, Shandong University School of Medicine, World Health Organization (WHO). China WHO Multi-country Survey Study on Health and Health System Responsiveness 2000-2001	Nonfatal Health Outcomes	Cocaine use disorders
Institute of Social Medicine and Health Policy, Shandong University, Shandong University School of Medicine, World Health Organization (WHO). China WHO Multi-country Survey Study on Health and Health System Responsiveness 2000-2001	Nonfatal Health Outcomes	Cannabis use disorders

Phillips MR, Zhang J, Shi Q, Song Z, Ding Z, Pang S, Li X, Zhang Y, Wang Z. Prevalence, treatment, and associated disability of mental disorders in four provinces in China during 2001-05: an epidemiological survey. Lancet. 2009; 373(9680): 2041-53	Nonfatal Health Outcomes	Anxiety disorders
Liu S, Wang W, Zhang J, He Y, Yao C, Zeng Z, Piao J, Howard BV, Fabsitz RR, Best L, Yang X, Lee ET. Prevalence of diabetes and impaired fasting glucose in Chinese adults, China National Nutrition and Health Survey, 2002. Prev Chronic Dis. 2011; 8(1): A13	Nonfatal Health Outcomes	Diabetes mellitus
Mezuk B, Chen Y, Yu C, Guo Y, Bian Z, Collins R, Chen J, Pang Z, Wang H, Peto R, Que X, Zhang H, Tan Z, Kendler KS, Li L, Chen Z. Depression, anxiety, and prevalent diabetes in the Chinese population: findings from the China Kadoorie Biobank of 0.5 million people. J Psychosom Res. 2013; 75(6): 511Â–7	Nonfatal Health Outcomes	Diabetes mellitus
Wand W, Xu W, Ding S, Zhou B. Population-based case-control study on risk factors of uterine myomas. Chin J Cancer Prev Treatment. 2007; 14(11): 821-3	Nonfatal Health Outcomes	Uterine fibroids
Lui P. Glucose-6-phosphate dehydrogenase gene mutations in She nationality, Fujian province. Chin J Hematol. 2005; 26(10): 612-5	Nonfatal Health Outcomes	G6PD deficiency
World Health Organization (WHO). China World Health Survey 2002. Geneva, Switzerland: World Health Organization (WHO), 2005	Nonfatal Health Outcomes	Edentulism and severe tooth loss
Zhang Q, MacDonald NE, Guo Z. Vaccine preventable community-acquired pneumonia in hospitalized children in Northwest China. Pediatr Infect Dis J. 2011; 30(1): 7-10	Nonfatal Health Outcomes	Influenza
Zhang Q, MacDonald NE, Guo Z. Vaccine preventable community-acquired pneumonia in hospitalized children in Northwest China. Pediatr Infect Dis J. 2011; 30(1): 7-10	Nonfatal Health Outcomes	Respiratory syncytial virus pneumonia
Xuan S-Y, Xin Y-N, Chen H, Shi G-J, Guan H-S, Li Y. Significance of hepatitis B virus surface antigen, hepatitis C virus expression in hepatocellular carcinoma and pericarcinomatous tissues . World J Gastroenterol. 2007; 13(12): 1870	Nonfatal Health Outcomes	Liver cancer due to hepatitis C
Luo Y, Li X, Li J, Wang X, Xu Y, Qiao Y, Hu D, Ma Y. Peripheral arterial disease, chronic kidney disease, and mortality: the Chinese Ankle Brachial Index Cohort Study. Vasc Med. 2010; 15(2): 107-12	Nonfatal Health Outcomes	Peripheral artery disease
Li J-M, Chen Y-R, Li X-T, Xu W-C. Screening of Herpes simplex virus 2 infection among pregnant women in southern China. J Dermatol. 2011; 38(2): 120Â–4	Nonfatal Health Outcomes	Genital herpes
Ding BG, Fan DM, Mu LN, Yu SZ. A caseÂ–control study of hepatitis B virus, hepatitis C virus and liver cancer. Chin J Epidemiol. 2004; 25(22)	Nonfatal Health Outcomes	Liver cancer due to hepatitis C
Zeng JH, Dai YS, Cao MQ, Hu XK. Serological markers of HCV and HBV and primary hepatocellular carcinoma. Modern Prev Med. 2003; 30: 324-5	Nonfatal Health Outcomes	Liver cancer due to hepatitis C
Chen Q-L, Shi Z-B, Wu L-R, Feng J, Wang Y, Tan N, Li Y-Y. Epidemiological Investiagtion on Opioid Use Disorders in Lanzhou City. J Clin Psychol Med. 2003; 13(5): 285-6	Nonfatal Health Outcomes	Opioid use disorders
Chen H-L, Hu B, Chen X-S, Zou G-H, Lu X-Y, Zhou P-L, Tu Y-L, Wei B, Yu X-H, Li K, Zou S-J, Li Z-H, Wu S-H, Kuang Y-H, Liu P, Liu Z-Y, Chen D-H, Liu K-F, Zhou G-Z, Li C-, Zhu A-X. Epidemiological investigation on mental disorders in Jiangxi province. Chin J Psychiatry. 2004; 37(3): 52-55	Nonfatal Health Outcomes	Alcohol use disorders
Zhang J-X, Lu C-H, Tang J-S, Qiu H-M, Liu L-F, Wang S-B, Wang A-Z, Zhang T-L, Phillips M-R, Li X-Y, Zhang S-D, Jiang Y-H, Zhao C-Y, Hu B-W, Cao X-Y, Zhang Y, Gao C-N. Epidemiological survey of mental disorders in persons aged 18 years and older in Shandong Province. Chin Ment Health J. 2010; 24(3): 161-167	Nonfatal Health Outcomes	Alcohol use disorders
Zhang G, Xu Y, Huang C, Yang H, Lu Y. An epidemiolgoical survey of autism in the Yunyan district of Guiyang city. J Guiyand Med Coll. 2009; 34(4): 463Â–465	Nonfatal Health Outcomes	Autism spectrum disorders
Miller S, Tudor C, Nyima, Thorsten VR, Sonam, Droyoung, Craig S, Le P, Wright LL, Varner MW. Maternal and neonatal outcomes of hospital vaginal deliveries in Tibet. Int J Gynaecol Obstet. 2007; 98(3): 217-21	Nonfatal Health Outcomes	Neonatal preterm birth
Tian H, Song G, Xie H, Zhang H, Tuomilehto J, Hu G. Prevalence of diabetes and impaired fasting glucose among 769,792 rural Chinese adults. Diabetes Res Clin Pract. 2009; 84(3): 273-8	Nonfatal Health Outcomes	Diabetes mellitus
Zhu H, Napravnik S, Eron JJ, Cole SR, Ma Y, Wohl DA, Dou Z, Zhang Y, Liu Z, Zhao D, Yu L, Liu X, Cohen MS, Zhang F. Decreasing excess mortality of HIV-infected patients initiating antiretroviral therapy: comparison with mortality in general population in China, 2003-2009. J Acquir Immune Defic Syndr. 2013; 63(5): e150-157	Nonfatal Health Outcomes	HIV/AIDS
Xueqiang Fang YZ. Prevalence and risk factors of trichomoniasis, bacterial vaginosis, and candidiasis for married women of child-bearing age in rural Shandong. Jpn J Infect Dis. 2007; 60(5): 257-61	Nonfatal Health Outcomes	Sexually transmitted infections excluding HIV
Liao L, Xing H, Su B, Wang Z, Ruan Y, Wang X, Liu Z, Lu Y, Yang S, Zhao Q, Vermund SH, Chen RY, Shao Y. Impact of HIV drug resistance on virologic and immunologic failure and mortality in a cohort of patients on antiretroviral therapy in China. AIDS. 2013; 27(11): 1815-24	Nonfatal Health Outcomes	HIV/AIDS

World Health Organization (WHO). WHO World Mental Health Surveys: Global Perspectives on the Epidemiology of Mental Disorders. Cambridge, United Kingdom: Cambridge University Press, 2008	Nonfatal Health Outcomes	Cannabis use disorders
Liu S, Wang X, Wang D, Zhou Y, LÃ¼ J, Zheng J, Zhong N, Ran P. [Epidemiologic analysis of COPD in Guangdong province]. Nat Med J Chin. 2005; 85(11): 747-52	Nonfatal Health Outcomes	Chronic obstructive pulmonary disease
Han Y-C, Li K-Q, Cui L-J, Huang J, Jiang Q-P, Cui Z, Sun X-L, Gao L-H, Zhang Y. The epidemiological survey on the alcohol abuse and dependence obstacles in the contryside of Hebei province. China J Health Psychol. 2009; 17(4): 460-462	Nonfatal Health Outcomes	Alcohol use disorders
Wang ZF. A study of the relationship between the primary carcinoma of liver and hepatitis B, C and TTV. Henan Med Res. 2003; 12: 43-44	Nonfatal Health Outcomes	Liver cancer due to hepatitis B
Wang ZF. A study of the relationship between the primary carcinoma of liver and hepatitis B, C and TTV. Henan Med Res. 2003; 12: 43-44	Nonfatal Health Outcomes	Liver cancer due to hepatitis C
Zhang S-J, Jiang C, Wang P, Liu M-J, Liu H, Yu C-Y, Hu Z-G, Yu F-Q, Yang X-L, N J, Li N, Yan T-M, Pan G-W. Epidemiological Survey of Mood Disorders Among Urban and Rural Residents in Liaoning Province. Chin J Prev Control Chronic Dis. 2008; 16(4): 378-81	Nonfatal Health Outcomes	Bipolar disorder
Lam KBH, Yin P, Jiang CQ, Zhang WS, Adab P, Miller MR, Thomas GN, Ayres JG, Lam TH, Cheng KK. Past dust and GAS/FUME exposure and COPD in Chinese: the Guangzhou Biobank Cohort Study. Respir Med. 2012; 106(10): 1421-8	Nonfatal Health Outcomes	Chronic obstructive pulmonary disease
Guo C. An epidemiological survey of psychiatric disability among children 2-6 years of age in Dingxi county. Chin J Rehabil Theory Pract. 2004; 10(2): 118Â–119	Nonfatal Health Outcomes	Autism spectrum disorders
Jiang H, Petersen PE, Peng B, Tai B, Bian Z. Self-assessed dental health, oral health practices, and general health behaviors in Chinese urban adolescents. Acta Odontol Scand. 2005; 63(6): 343-52	Nonfatal Health Outcomes	Caries of deciduous teeth
Guo R. Epidemiological investigation analysis of 5000 Chinese between 0 and 6 years old with childhood autism in Tianjin city. Chin J Clin Rehab. 2004; 8(6): 1122Â–1123	Nonfatal Health Outcomes	Autism spectrum disorders
Zhang X, Ji C-Y. Autism and mental retardation of young children in China. Biomed Environ Sci. 2005; 18(5): 334Â–40	Nonfatal Health Outcomes	Autism spectrum disorders
Zhang X, Ji C, Li J, Sun J. The investigation of autism in children aged 2 to 6 years old in Tianjin. Chin J Reprod Health. 2004; 15(4): 206-208	Nonfatal Health Outcomes	Autism spectrum disorders
Han W, Xie Y, Zhou XL, Yin LM, Shu-Ying R. Prognosis of 212 children with asthma: a 5-year follow-up study. Chin J Contemp Pediatr. 2011; 13(11): 870	Nonfatal Health Outcomes	Asthma
Du H, Li L, Bennett D, Guo Y, Key T, Bian Z, Sherliker P, Gao H, Chen Y, Yang L, Chen J, Wang S, Du R, Su H, Collins R, Peto R, Chen Z. Fresh Fruit Consumption and Major Cardiovascular Disease in China. N Engl J Med. 2016; 374(14): 1332-43	Nonfatal Health Outcomes	Intracerebral hemorrhage
Wei G, Liu S-M, Zhang W, Xiang Y, Huang X-Q, Yang C, Huang W-J, Xie W-J, He X, Su X-F, Wang J, Ciren P-C, Baima Z-G, Ci-P, Zha S, Liu X-H. Epidemiological investigation on mental disorders at Tibet in China: I major psychiatric disorders. Chin J Nerv Ment Dis. 2008; 34(10): 601-604	Nonfatal Health Outcomes	Bipolar disorder
Liang X1, Bi S, Yang W, Wang L, Cui G, Cui F, Zhang Y, Liu J, Gong X, Chen Y, Wang F, Zheng H, Wang F, Guo J, Jia Z, Ma J, Wang H, Luo H, Li L, Jin S, Hadler SC, Wang Y. Epidemiological serosurvey of hepatitis B in China--declining HBV prevalence due to hepatitis B vaccination. Vaccine. 2009; 5(27): 6550-7	Nonfatal Health Outcomes	Acute hepatitis B
Chinese Center for Disease Control and Prevention (CCDC), Ministry of Health (China). China National Injury Surveillance System 2006 - China CDC	Nonfatal Health Outcomes	Road injuries
Chinese Center for Disease Control and Prevention (CCDC), Ministry of Health (China). China National Injury Surveillance System 2006 - China CDC	Nonfatal Health Outcomes	Exposure to mechanical forces
Chinese Center for Disease Control and Prevention (CCDC), Ministry of Health (China). China National Injury Surveillance System 2006 - China CDC	Nonfatal Health Outcomes	Interpersonal violence
Xu J, Xu L, Du KF, Shao L, Chen CX, Zhou JQ, Wang YX, You QS, Jonas JB, Wei WB. Subfoveal choroidal thickness in diabetes and diabetic retinopathy [Unpublished data]. Ophthalmology. 2013 Oct;120(10):2023-8	Nonfatal Health Outcomes	
Chinese Center for Disease Control and Prevention (CCDC), Ministry of Health (China). China National Injury Surveillance System 2006 - China CDC	Nonfatal Health Outcomes	Physical violence by sharp object
Chinese Center for Disease Control and Prevention (CCDC). China Disease Surveillance Points 2006 - China CDC	Nonfatal Health Outcomes	Rabies
International Centre for Eye Health (ICEH). China - Kunming Rapid Assessment of Avoidable Blindness 2006. Grootebroek, Netherlands: RAAB Repository	Nonfatal Health Outcomes	Other vision loss
Chinese Center for Disease Control and Prevention (CCDC), Ministry of Health (China). China National Injury Surveillance System 2006 - China CDC	Nonfatal Health Outcomes	Falls
Chinese Center for Disease Control and Prevention (CCDC), Ministry of Health (China). China National Injury Surveillance System 2006 - China CDC	Nonfatal Health Outcomes	Self-harm
Chinese Center for Disease Control and Prevention (CCDC), Ministry of Health (China). China National Injury Surveillance System 2006 - China CDC	Nonfatal Health Outcomes	Non-venomous animal contact

Chinese Center for Disease Control and Prevention (CCDC). China Notifiable Infectious Diseases 2006	Nonfatal Health Outcomes	Rabies
Chinese Center for Disease Control and Prevention (CCDC), Ministry of Health (China). China National Injury Surveillance System 2006 - China CDC	Nonfatal Health Outcomes	Pulmonary aspiration and foreign body in airway
International Clearinghouse for Birth Defects Surveillance and Research. International Clearinghouse for Birth Defects Surveillance and Research Annual Report 2007. Rome, Italy: International Clearinghouse for Birth Defects Surveillance and Research, 2008	Nonfatal Health Outcomes	Orofacial clefts
Chinese Center for Disease Control and Prevention (CCDC), Ministry of Health (China). China National Injury Surveillance System 2006 - China CDC	Nonfatal Health Outcomes	Fire, heat, and hot substances
Chen X, Yang D, Mo Y, Li L, Chen Y, Huang Y. Prevalence of polycystic ovary syndrome in unselected women from southern China. Eur J Obstet Gynecol Reprod Biol. 2008; 139(1): 59-64	Nonfatal Health Outcomes	Polycystic ovarian syndrome
United Nations Population Division. United Nations World Population Prospects 1950-2100 - 2010 Revision. New York City, United States: United Nations Population Division, 2011	Nonfatal Health Outcomes	Premenstrual syndrome
Chinese Center for Disease Control and Prevention (CCDC), Ministry of Health (China). China National Injury Surveillance System 2006 - China CDC	Nonfatal Health Outcomes	Animal contact
Zhao Y, Zhang Q, Tsering T, Sangwan, Hu X, Liu L, Shang H, Chen Q, Liu Y, Yang X, Wang W, Li S, Wu J, Sander JW, Zhou D. Prevalence of convulsive epilepsy and health-related quality of life of the population with convulsive epilepsy in rural areas of Tibet Autonomous Region in China: an initial survey. Epilepsy Behav. 2008; 12(3): 373-81	Nonfatal Health Outcomes	Epilepsy
Hu J-Z, Hu C-Y, Duan W-D, Gao H, Zhang X, Tang Z-R, Lu Y-W, Zhang F-X, Jin D, Yang K-J, Lin X-B, Yang H, Shu M-Y, Zhang Y-H, Liu T-B, Shen Q-J. Survey on Mental Disorders among Registered Residents and Non-registered Residents in Shenzhen. Chin J Epidemiol. 2009; 30(6): 543-8	Nonfatal Health Outcomes	Schizophrenia
Liao ZT, Pan YF, Huang JL, Huang F, Chi WJ, Zhang KX, Lin ZM, Wu YQ, He WZ, Wu J, Xie XJ, Huang JX, Wei QJ, Li TW, Wu Z, Yu BY, Gu JR. An epidemiological survey of low back pain and axial spondyloarthritis in a Chinese Han population. Scand J Rheumatol. 2009; 38(6): 455-9	Nonfatal Health Outcomes	Low back pain
Li F, Zhou Y, Li S, Jiang F, Jin X, Yan C, Tian Y, Zhang Y, Tong S, Shen X. Prevalence and risk factors of childhood allergic diseases in eight metropolitan cities in China: a multicenter study. BMC Public Health. 2011; 11: 437	Nonfatal Health Outcomes	Atopic dermatitis
Flohr C, Weinmayr G, Weiland SK, Addo-Yobo E, Annesi-Maesano I, BjÃ¶rkstÃ©n B, BrÃ¥bÃ¤ck L, BÃ¼chele G, Chico M, Cooper P, Clausen M, El Sharif N, Martinez Gimeno A, Mathur RS, von Mutius E, Morales Suarez-Varela M, Pearce N, Svabe V, Wong GWK, Yu M, Zhong NS, Williams HC, ISAAC Phase Two Study Group. How well do questionnaires perform compared with physical examination in detecting flexural eczema? Findings from the International Study of Asthma and Allergies in Childhood (ISAAC) Phase Two. Br J Dermatol. 2009; 161(4): 846-53	Nonfatal Health Outcomes	Atopic dermatitis
Flohr C, Weiland SK, Weinmayr G, BjÃ¶rkstÃ©n B, BrÃ¥bÃ¤ck L, Brunekreef B, BÃ¼chele G, Clausen M, Cookson WOC, von Mutius E, Strachan DP, Williams HC, ISAAC Phase Two Study Group. The role of atopic sensitization in flexural eczema: findings from the International Study of Asthma and Allergies in Childhood Phase Two. J Allergy Clin Immunol. 2008; 121(1): 141-147	Nonfatal Health Outcomes	Atopic dermatitis
Xie Z-H, Bo S-Y, Zhang X-T, Liu M, Zhang Z-X, Yang X-L, Ji S-R, Yan H, Sui X-L, Na X, Guo S-H, Wu Z-L. Sampling survey on intellectual disability in 0 approximately 6-year-old children in China. J Intellect Disabil Res. 2008; 52(12): 1029-38	Nonfatal Health Outcomes	Developmental intellectual disability
Chinese Center for Disease Control and Prevention (CCDC), Ministry of Health (China). China National Injury Surveillance System 2006 - China CDC	Nonfatal Health Outcomes	Unintentional firearm injuries
He M, Huang W, Zheng Y, Huang L, Ellwein LB. Refractive error and visual impairment in school children in rural southern China. Ophthalmology. 2007; 114(2): 374-82	Nonfatal Health Outcomes	Refraction and accommodation disorders
Chinese Center for Disease Control and Prevention (CCDC), Ministry of Health (China). China National Injury Surveillance System 2006 - China CDC	Nonfatal Health Outcomes	Foreign body in other body part
Chinese Center for Disease Control and Prevention (CCDC), Ministry of Health (China). China National Injury Surveillance System 2006 - China CDC	Nonfatal Health Outcomes	Poisonings
Chinese Center for Disease Control and Prevention (CCDC). China Notifiable Infectious Diseases 2006	Nonfatal Health Outcomes	Syphilis
Chinese Center for Disease Control and Prevention (CCDC), Ministry of Health (China). China National Injury Surveillance System 2006 - China CDC	Nonfatal Health Outcomes	Physical violence by other means
Chinese Center for Disease Control and Prevention (CCDC), Ministry of Health (China). China National Injury Surveillance System 2006 - China CDC	Nonfatal Health Outcomes	Other exposure to mechanical forces
Chinese Center for Disease Control and Prevention (CCDC), Ministry of Health (China). China National Injury Surveillance System 2006 - China CDC	Nonfatal Health Outcomes	Drowning

Gao L-J, Wqu Q-H, Ning N, Sun H, Cui Y. Analysis on the prevalence and causes of mental disabilities of population in Heilongjiang province. Med Soc. 2008; 21(9): 4-6	Nonfatal Health Outcomes	Schizophrenia
Chinese Center for Disease Control and Prevention (CCDC), Ministry of Health (China). China National Injury Surveillance System 2006 - China CDC	Nonfatal Health Outcomes	Adverse effects of medical treatment
Jiangsu Province Hospital, Nanjing Medical University. China - Jiangsu, Sichuan, Guizhou, and Jilin WHO Ear and Hearing Disorders Survey 2005-2006, as provided by the Global Burden of Disease 2010 Hearing Disorders Expert Group. [Unpublished]	Nonfatal Health Outcomes	Hearing loss
Chinese Center for Disease Control and Prevention (CCDC). China Notifiable Infectious Diseases 2005	Nonfatal Health Outcomes	Gonococcal infection
Chinese Center for Disease Control and Prevention (CCDC). China Notifiable Infectious Diseases 2006	Nonfatal Health Outcomes	Gonococcal infection
International Clearinghouse for Birth Defects Surveillance and Research. International Clearinghouse for Birth Defects Surveillance and Research Annual Report 2007. Rome, Italy: International Clearinghouse for Birth Defects Surveillance and Research, 2008	Nonfatal Health Outcomes	Congenital musculoskeletal and limb anomalies
International Clearinghouse for Birth Defects Surveillance and Research. International Clearinghouse for Birth Defects Surveillance and Research Annual Report 2007. Rome, Italy: International Clearinghouse for Birth Defects Surveillance and Research, 2008	Nonfatal Health Outcomes	Neural tube defects
Yangzom Y, Qian L, Shan M, La Y, Meiduo D, Hu X, Da Q, Sun B, ZetterstrÃ¶m R. Outcome of hospital deliveries of women living at high altitude: a study from Lhasa in Tibet. Acta Paediatr. 2008; 97(3): 317-21	Nonfatal Health Outcomes	Neonatal preterm birth
International Clearinghouse for Birth Defects Surveillance and Research. International Clearinghouse for Birth Defects Surveillance and Research Annual Report 2007. Rome, Italy: International Clearinghouse for Birth Defects Surveillance and Research, 2008	Nonfatal Health Outcomes	Urogenital congenital anomalies
Hong Kong Cancer Registry. Hong Kong Cancer Registry Annual Tables. Kowloon, Hong Kong: Hong Kong Cancer Registry	Nonfatal Health Outcomes	Bladder cancer
Chen N, Wang W, Huang Y, Shen P, Pei D, Yu H, Shi H, Zhang Q, Xu J, Lv Y, Fan Q. Community-based study on CKD subjects and the associated risk factors. Nephrol Dial Transplant. 2009; 24(7): 2117-23	Nonfatal Health Outcomes	Chronic kidney disease
He N, Cao H, Yin Y, Gao M, Zhang T, Detels R. Herpes simplex virus-2 infection in male rural migrants in Shanghai, China. Int J STD AIDS. 2009; 20(2): 112Â–4	Nonfatal Health Outcomes	Genital herpes
International Centre for Eye Health (ICEH). China - Kunming Rapid Assessment of Avoidable Blindness 2006. Grootebroek, Netherlands: RAAB Repository	Nonfatal Health Outcomes	Vision loss
Zhang L, Zhang P, Wang F, Zuo L, Zhou Y, Shi Y, Li G, Jiao S, Liu Z, Liang W, Wang H. Prevalence and factors associated with CKD: a population study from Beijing. Am J Kidney Dis. 2008; 51(4): 373-84	Nonfatal Health Outcomes	Chronic kidney disease
National Central Cancer Registry (China). China Cancer Registry Incidence and Mortality 1990-2009	Nonfatal Health Outcomes	Lip and oral cavity cancer
World Health Organization (WHO). WHO Tuberculosis Case Notifications. Geneva, Switzerland: World Health Organization (WHO)	Nonfatal Health Outcomes	HIV/AIDS and tuberculosis
Yu Z, Xi Y, Ding W, Han S, Cao L, Zhu C, Wang X, Guo X. Congenital heart disease in a Chinese hospital: pre- and postnatal detection, incidence, clinical characteristics and outcomes. Pediatr Int. 2011; 53(6): 1059-65	Nonfatal Health Outcomes	Congenital birth defects
Li L, Xu Z, Jin X, Yan C, Jiang F, Tong S, Shen X, Li S. Sleep-disordered breathing and asthma: evidence from a large multicentric epidemiological study in China. Respir Res. 2015; 16: 56	Nonfatal Health Outcomes	Asthma
Chinese Center for Disease Control and Prevention (CCDC), Ministry of Health (China). China National Injury Surveillance System 2006 - China CDC	Nonfatal Health Outcomes	Venomous animal contact
United Nations Children's Fund (UNICEF). Child Mortality and Injury in Asia. New York, United States: United Nations Children's Fund (UNICEF), 2008. (Innocenti Working Papers: Special Series on Child Injuries Nos. 1-4)	Nonfatal Health Outcomes	Self-harm
Chinese Center for Disease Control and Prevention (CCDC), Ministry of Health (China). China National Injury Surveillance System 2006 - China CDC	Nonfatal Health Outcomes	Foreign body in eyes
Wang D, Liu J, Liu M, Lu C, Brainin M, Zhang J. Patterns of Stroke Between University Hospitals and Nonuniversity Hospitals in Mainland China: Prospective Multicenter Hospital-Based Registry Study. World Neurosurg. 2017; 98: 258Â–65	Nonfatal Health Outcomes	Intracerebral hemorrhage
Chinese Center for Disease Control and Prevention (CCDC), Ministry of Health (China). China National Injury Surveillance System 2006 - China CDC	Nonfatal Health Outcomes	Physical violence by firearm
Sung RYT, Chan PKS, Tsen T, Li AM, Lam WY, Yeung ACM, Nelson EAS. Identification of viral and atypical bacterial pathogens in children hospitalized with acute respiratory infections in Hong Kong by multiplex PCR assays. J Med Virol. 2009; 81(1): 153-9	Nonfatal Health Outcomes	Influenza

Sung RYT, Chan PKS, Tsen T, Li AM, Lam WY, Yeung ACM, Nelson EAS. Identification of viral and atypical bacterial pathogens in children hospitalized with acute respiratory infections in Hong Kong by multiplex PCR assays. J Med Virol. 2009; 81(1): 153-9	Nonfatal Health Outcomes	Respiratory syncytial virus pneumonia
World Health Organization Regional Office for the Western Pacific (WPRO-WHO). Number of Reported Cases of Dengue Fever and Dengue Hemorrhagic Fever in the Western Pacific Region, by Country 2006. Manila, Philippines: World Health Organization Regional Office for the Western Pacific (WPRO-WHO)	Nonfatal Health Outcomes	Dengue
Song Z-Q, Du X-B, Han G-L, Jian Y-L, Liu C, Phillips Michael R, Li X-Y, An H-S, Shen B-L, Zheng T, Liu L-X, E H-H. Epidemiological survey of mental disorders in persons aged 18 and older in Qinghai Province. Chin Ment Health J. 2010; 24(3): 168-174	Nonfatal Health Outcomes	Alcohol use disorders
Wu M, Yip JLY, Kuper H. Rapid assessment of avoidable blindness in Kunming, China. Ophthalmology. 2008; 115(6): 969-74	Nonfatal Health Outcomes	Vision loss
Chinese Center for Disease Control and Prevention (CCDC), Ministry of Health (China). China National Injury Surveillance System 2006 - China CDC	Nonfatal Health Outcomes	Injuries
Song Z-Q, Du X-B, Han G-L, Jian Y-L, Liu C, Phillips Michael R, Li X-Y, An H-S, Shen B-L, Zheng T, Liu L-X, E H-H. Epidemiological survey of mental disorders in persons aged 18 and older in Qinghai Province. Chin Ment Health J. 2010; 24(3): 168-174	Nonfatal Health Outcomes	Anxiety disorders
Liang Z, Lin Y, Ma Y, Zhang L, Zhang X, Li L, Zhang S, Cheng Y, Zhou X, Lin H, Miao H, Zhao Q. The association between ambient temperature and preterm birth in Shenzhen, China: a distributed lag non-linear time series analysis. Environ Health. 2016; 15(1): 84	Nonfatal Health Outcomes	Neonatal preterm birth
Ding Z-J, Wang G-P, Pei G-X, Guan R-C, He R-F, Phillips Michael R, Li X-Y, Zhang Y-L, Wang D-M, Hong G-Z, Du H-Y, Bai A-P, Li G, Wang X-Q, Wei C-L, Li H, Wang J. Epidemiological survey of mental disorders in people aged 18 and older in Tianshui City of Gansu Province. Chin Ment Health J. 2010; 24(3): 183-190	Nonfatal Health Outcomes	Major depressive disorder
World Health Organization (WHO). Global leprosy situation, 2006. Wkly Epidemiol Rec. 2006: 81(32): 309-16.	Nonfatal Health Outcomes	Leprosy
World Health Organization (WHO). Global leprosy situation, 2007. Wkly Epidemiol Rec. 2007: 82(25): 225-32	Nonfatal Health Outcomes	Leprosy
International Centre for Eye Health (ICEH). China - Kunming Rapid Assessment of Avoidable Blindness 2006. Grootebroek, Netherlands: RAAB Repository	Nonfatal Health Outcomes	Cataract
Wang R, Yan X, Ma XQ, Cao Y, Wallander MA, Johansson S, He J. Burden of gastroesophageal reflux disease in Shanghai, China. Dig Liver Dis. 2009; 41(2): 110-5	Nonfatal Health Outcomes	Gastroesophageal reflux disease
International Centre for Eye Health (ICEH). China - Kunming Rapid Assessment of Avoidable Blindness 2006. Grootebroek, Netherlands: RAAB Repository	Nonfatal Health Outcomes	
Kim JH, Lee S, Chow J, Lau J, Tsang A, Choi J, Griffiths SM. Prevalence and the factors associated with binge drinking, alcohol abuse, and alcohol dependence: a population-based study of Chinese adults in Hong Kong. Alcohol Alcohol. 2008; 43(3): 360Â–70	Nonfatal Health Outcomes	Alcohol use disorders
Leung P, Hung S, Ho T, Lee C, Liu W, Tang C, Kwong S. Prevalence of DSM-IV disorders in Chinese adolescents and the effects of an impairment criterion: a pilot community study in Hong Kong. Eur Child Adolesc Psychiatry. 2008; 17(7): 452-61	Nonfatal Health Outcomes	Attention-deficit/hyperactivity disorder
Leung P, Hung S, Ho T, Lee C, Liu W, Tang C, Kwong S. Prevalence of DSM-IV disorders in Chinese adolescents and the effects of an impairment criterion: a pilot community study in Hong Kong. Eur Child Adolesc Psychiatry. 2008; 17(7): 452-61	Nonfatal Health Outcomes	Conduct disorder
Li F, Zhou Y, Li S, Jiang F, Jin X, Yan C, Tian Y, Zhang Y, Tong S, Shen X. Prevalence and risk factors of childhood allergic diseases in eight metropolitan cities in China: a multicenter study. BMC Public Health. 2011; 11: 437	Nonfatal Health Outcomes	Dermatitis
Fu Q, Li SZ, Wu WP, Hou YY, Zhang S, Feng Y, Zhang LP, Tang LH. Endemic characteristics of infantile visceral leishmaniasis in the People's Republic of China. Parasites Vectors. 2013; 143	Nonfatal Health Outcomes	Visceral leishmaniasis
Lin H, He N, Su M, Feng J, Chen L, Gao M. Herpes simplex virus infections among rural residents in eastern China. BMC Infect Dis. 2011; 69	Nonfatal Health Outcomes	Genital herpes
Hai J-J, Chan P-H, Huang D, Ho M-H, Ho C-W, Cheung E, Lau C-P, Tse H-F, Siu C-W. Clinical Characteristics, Management, and Outcomes of Hospitalized Heart Failure in a Chinese Population-The Hong Kong Heart Failure Registry. J Card Fail. 2016; 22(8): 600Â–8	Nonfatal Health Outcomes	Heart failure
Liu T, Zhang L, Pang L, Li N, Chen G, Zheng X. Schizophrenia-related disability in China: prevalence, gender, and geographic location. Psychiatr Serv. 2015; 66(3): 249Â–57	Nonfatal Health Outcomes	Schizophrenia
Wu M, Yip JLY, Kuper H. Rapid assessment of avoidable blindness in Kunming, China. Ophthalmology. 2008; 115(6): 969-74	Nonfatal Health Outcomes	Cataract

Ye H-S, Luo X-R, Zhou X, Shen X-Y, Wang X-H. The Epidemiological Investigation of Anxiety Disorders in Urban-rural Primary and Middle School Students in Huaihua. J Clin Psychosom Dis. 2008; 14(6): 501-4	Nonfatal Health Outcomes	Anxiety disorders
Hai J-J, Chan P-H, Huang D, Ho M-H, Ho C-W, Cheung E, Lau C-P, Tse H-F, Siu C-W. Clinical Characteristics, Management, and Outcomes of Hospitalized Heart Failure in a Chinese Population-The Hong Kong Heart Failure Registry. J Card Fail. 2016; 22(8): 600Â–8	Nonfatal Health Outcomes	
Xu J, Xu L, Du KF, Shao L, Chen CX, Zhou JQ, Wang YX, You QS, Jonas JB, Wei WB. Subfoveal choroidal thickness in diabetes and diabetic retinopathy [Unpublished data]. Ophthalmology. 2013 Oct;120(10):2023-8	Nonfatal Health Outcomes	Vision loss
International Centre for Eye Health (ICEH). China - Kunming Rapid Assessment of Avoidable Blindness 2006. Grootebroek, Netherlands: RAAB Repository	Nonfatal Health Outcomes	Refraction and accommodation disorders
Wu M, Yip JLY, Kuper H. Rapid assessment of avoidable blindness in Kunming, China. Ophthalmology. 2008; 115(6): 969-74	Nonfatal Health Outcomes	Age-related macular degeneration
Li F, Zhou Y, Li S, Jiang F, Jin X, Yan C, Tian Y, Zhang Y, Tong S, Shen X. Prevalence and risk factors of childhood allergic diseases in eight metropolitan cities in China: a multicenter study. BMC Public Health. 2011; 11: 437	Nonfatal Health Outcomes	Asthma
Chinese Center for Disease Control and Prevention (CCDC). China Notifiable Infectious Diseases 2005	Nonfatal Health Outcomes	Sexually transmitted infections excluding HIV
Chinese Center for Disease Control and Prevention (CCDC). China Notifiable Infectious Diseases 2006	Nonfatal Health Outcomes	Sexually transmitted infections excluding HIV
Ellis MK, Raso G, Li Y-S, Rong Z, Chen H-G, McManus DP. Familial aggregation of human susceptibility to co- and multiple helminth infections in a population from the Poyang Lake region, China. Int J Parasitol. 2007; 37(10): 1153Â–61	Nonfatal Health Outcomes	Schistosomiasis
International Clearinghouse for Birth Defects Surveillance and Research. International Clearinghouse for Birth Defects Surveillance and Research Annual Report 2007. Rome, Italy: International Clearinghouse for Birth Defects Surveillance and Research, 2008	Nonfatal Health Outcomes	Congenital birth defects
Lu J, Ruan Y, Huang Y, Yao J, Dang W, Gao C. Major depression in Kunming: prevalence, correlates and co-morbidity in a south-western city of China. J Affect Disord. 2008; 111(2-3): 221-6	Nonfatal Health Outcomes	Major depressive disorder
Chinese Center for Disease Control and Prevention (CCDC). China Notifiable Infectious Diseases 2005	Nonfatal Health Outcomes	Syphilis
National Central Cancer Registry (China). China Cancer Registry Incidence and Mortality 1990-2009	Nonfatal Health Outcomes	Malignant skin melanoma
Liu QG, Sun J, Xiao XW, Song GR. Birth defects data from surveillance hospitals in Dalian city, China, 2006-2010. J Matern Fetal Neonatal Med. 2016; 29(22): 3615-21	Nonfatal Health Outcomes	Digestive congenital anomalies
Wu S, Qiu Y, Yan P, Wang W, Xie M, Xia P, Wang Z, Yan Y. Effectiveness of first-line antiretroviral therapy in HIV/AIDS patients: A 5-year longitudinal evaluation in Fujian Province, Southeast China. Arch Virol. 2015; 160(11): 2693Â–701	Nonfatal Health Outcomes	HIV/AIDS resulting in other diseases
Liu QG, Sun J, Xiao XW, Song GR. Birth defects data from surveillance hospitals in Dalian city, China, 2006-2010. J Matern Fetal Neonatal Med. 2016; 29(22): 3615-21	Nonfatal Health Outcomes	Congenital musculoskeletal and limb anomalies
Wei W, Liu S-Y, Zeng F-F, Yao S-P, Zhang H-T, Wan G, Zhong M, Yang Z, Wang B-Y. Type 2 diabetes and impaired glucose tolerance in North-China-based rural community adults. Public Health. 2010; 124(10): 593-601	Nonfatal Health Outcomes	Diabetes mellitus
Liu QG, Sun J, Xiao XW, Song GR. Birth defects data from surveillance hospitals in Dalian city, China, 2006-2010. J Matern Fetal Neonatal Med. 2016; 29(22): 3615-21	Nonfatal Health Outcomes	Congenital birth defects
Xie Z-H, Bo S-Y, Zhang X-T, Liu M, Zhang Z-X, Yang X-L, Ji S-R, Yan H, Sui X-L, Na X, Guo S-H, Wu Z-L. Sampling survey on intellectual disability in 0 approximately 6-year-old children in China. J Intellect Disabil Res. 2008; 52(12): 1029-38	Nonfatal Health Outcomes	Idiopathic developmental intellectual disability
Wang H, Fang C, Cai L, Dong B, Deng J. Chronic kidney disease and cognitive impairment among the very old in China. Aging Clin Exp Res. 2016; 28(3): 475-82	Nonfatal Health Outcomes	Chronic kidney disease
Jiang L, Rong J, Zhang Q, Hu F, Zhang S, Li X, Zhao Y, Tao T. Prevalence and associated factors of knee osteoarthritis in a community-based population in Heilongjiang, Northeast China. Rheumatol Int. 2012; 32(5): 1189-95	Nonfatal Health Outcomes	Osteoarthritis
International Clearinghouse for Birth Defects Surveillance and Research. International Clearinghouse for Birth Defects Surveillance and Research Annual Report 2007. Rome, Italy: International Clearinghouse for Birth Defects Surveillance and Research, 2008	Nonfatal Health Outcomes	Digestive congenital anomalies
Ding Z-J, Wang G-P, Pei G-X, Guan R-C, He R-F, Phillips Michael R, Li X-Y, Zhang Y-L, Wang D-M, Hong G-Z, Du H-Y, Bai A-P, Li G, Wang X-Q, Wei C-L, Li H, Wang J. Epidemiological survey of mental disorders in people aged 18 and older in Tianshui City of Gansu Province. Chin Ment Health J. 2010; 24(3): 183-190	Nonfatal Health Outcomes	Anxiety disorders
Yan J, Huang Y-Q, Ruan Y, Lu J, Gao C-Q, Dang W-M, Luo C. Epidemiological survey of affective disorder in Kunming City. Chin Ment Health J. 2010; 24(2): 110-115	Nonfatal Health Outcomes	Dysthymia

Zeng Q, Zang C, Li X, Dong H, Zhang A, Lin L. Associated risk factors of knee osteoarthritis: a population survey in Taiyuan, China. Chin Med J (Engl). 2006; 119(18): 1522-7	Nonfatal Health Outcomes	Osteoarthritis
Lu D-B, Wang T-P, Rudge JW, Donnelly CA, Fang G-R, Webster JP. Contrasting reservoirs for Schistosoma japonicum between marshland and hilly regions in Anhui, China--a two-year longitudinal parasitological survey. Parasitology. 2010; 137(1): 99Â–110	Nonfatal Health Outcomes	Schistosomiasis
Zhou YJ, Li YY, Nie YQ, Huang CM, Cao CY. Natural course of nonalcoholic fatty liver disease in southern China: a prospective cohort study. J Dig Dis. 2012; 13(3): 153-60	Nonfatal Health Outcomes	Cirrhosis due to NASH
International Centre for Eye Health (ICEH). China - Kunming Rapid Assessment of Avoidable Blindness 2006. Grootebroek, Netherlands: RAAB Repository	Nonfatal Health Outcomes	Diabetes mellitus
Mu J, Liu L, Zhang Q, Si Y, Hu J, Fang J, Gao Y, He J, Li S, Wang W, Wu J, Sander JW, Zhou D. Causes of death among people with convulsive epilepsy in rural West China: a prospective study. Neurology. 2011; 77(2): 132-7	Nonfatal Health Outcomes	Epilepsy
Jiangsu Province Hospital, Nanjing Medical University. China - Jiangsu, Sichuan, Guizhou, and Jilin WHO Ear and Hearing Disorders Survey 2005-2006, as provided by the Global Burden of Disease 2010 Hearing Disorders Expert Group. [Unpublished]	Nonfatal Health Outcomes	Age-related and other hearing loss
International Centre for Eye Health (ICEH). China - Kunming Rapid Assessment of Avoidable Blindness 2006. Grootebroek, Netherlands: RAAB Repository	Nonfatal Health Outcomes	Trachoma
International Centre for Eye Health (ICEH). China - Kunming Rapid Assessment of Avoidable Blindness 2006. Grootebroek, Netherlands: RAAB Repository	Nonfatal Health Outcomes	Age-related macular degeneration
Wu M, Yip JLY, Kuper H. Rapid assessment of avoidable blindness in Kunming, China. Ophthalmology. 2008; 115(6): 969-74	Nonfatal Health Outcomes	Glaucoma
International Centre for Eye Health (ICEH). China - Kunming Rapid Assessment of Avoidable Blindness 2006. Grootebroek, Netherlands: RAAB Repository	Nonfatal Health Outcomes	Glaucoma
Chinese Center for Disease Control and Prevention (CCDC). China Notifiable Infectious Diseases 2005	Nonfatal Health Outcomes	Chlamydial infection
Chinese Center for Disease Control and Prevention (CCDC). China Notifiable Infectious Diseases 2006	Nonfatal Health Outcomes	Chlamydial infection
Wu M, Yip JLY, Kuper H. Rapid assessment of avoidable blindness in Kunming, China. Ophthalmology. 2008; 115(6): 969-74	Nonfatal Health Outcomes	Other vision loss
Jiang Y, Lu J, Zhang L, Tian R, Liu Q, Bi S. [Antibody detection and sequencing analysis of hepatitis E virus in human population, swine and chicken in Sichuan region in China]. Chin J Exper Clin Virol. 2008; 22(6): 468-71	Nonfatal Health Outcomes	Acute hepatitis E
Liao ZT, Pan YF, Huang JL, Huang F, Chi WJ, Zhang KX, Lin ZM, Wu YQ, He WZ, Wu J, Xie XJ, Huang JX, Wei QJ, Li TW, Wu Z, Yu BY, Gu JR. An epidemiological survey of low back pain and axial spondyloarthritis in a Chinese Han population. Scand J Rheumatol. 2009; 38(6): 455-9	Nonfatal Health Outcomes	Low back and neck pain
Flohr C, Weinmayr G, Weiland SK, Addo-Yobo E, Annesi-Maesano I, BjÃ¶rkstÃ©n B, BrÃ¥bÃ¤ck L, BÃ¼chele G, Chico M, Cooper P, Clausen M, El Sharif N, Martinez Gimeno A, Mathur RS, von Mutius E, Morales Suarez-Varela M, Pearce N, Svabe V, Wong GWK, Yu M, Zhong NS, Williams HC, ISAAC Phase Two Study Group. How well do questionnaires perform compared with physical examination in detecting flexural eczema? Findings from the International Study of Asthma and Allergies in Childhood (ISAAC) Phase Two. Br J Dermatol. 2009; 161(4): 846-53	Nonfatal Health Outcomes	Dermatitis
Flohr C, Weiland SK, Weinmayr G, BjÃ¶rkstÃ©n B, BrÃ¥bÃ¤ck L, Brunekreef B, BÃ¼chele G, Clausen M, Cookson WOC, von Mutius E, Strachan DP, Williams HC, ISAAC Phase Two Study Group. The role of atopic sensitization in flexural eczema: findings from the International Study of Asthma and Allergies in Childhood Phase Two. J Allergy Clin Immunol. 2008; 121(1): 141-147	Nonfatal Health Outcomes	Dermatitis
He M, Huang W, Zheng Y, Huang L, Ellwein LB. Refractive error and visual impairment in school children in rural southern China. Ophthalmology. 2007; 114(2): 374-82	Nonfatal Health Outcomes	Refraction disorders
Liang W, Huang R, Lee A, Hu D, Binns C. Hospitalizations for incident stroke in Shunde District, Foshan, South China. Neuroepidemiology. 2008; 30(2): 101-4	Nonfatal Health Outcomes	Ischemic stroke
Liang W, Huang R, Lee A, Hu D, Binns C. Hospitalizations for incident stroke in Shunde District, Foshan, South China. Neuroepidemiology. 2008; 30(2): 101-4	Nonfatal Health Outcomes	Intracerebral hemorrhage
Liu QG, Sun J, Xiao XW, Song GR. Birth defects data from surveillance hospitals in Dalian city, China, 2006-2010. J Matern Fetal Neonatal Med. 2016; 29(22): 3615-21	Nonfatal Health Outcomes	Congenital heart anomalies
Zhang G-Y, Yang Y-C, Huang Y, Liu S-J, Sun X-L. Epidemiological investigation on depression among 6-16 years old children and adolescents in Chengdu. Chin J Psychiatry. 2010; 24(3): 211-214	Nonfatal Health Outcomes	Major depressive disorder

Kang X, Fransen M, Zhang Y, Li H, Ke Y, Lu M, Su S, Song X, Guo Y, Chen J, Niu J, Felson D, Lin J. The high prevalence of knee osteoarthritis in a rural Chinese population: the Wuchuan osteoarthritis study. Arthritis Rheum. 2009; 61(5): 641-7	Nonfatal Health Outcomes	Osteoarthritis
Zhan R-T, Lu W-X, Li S-Z, Xiang X-H, Chen W-M, Li T, Xiao J-M. Epidemiological investigation on mental disorders in Puer city. Med J Chin Peoples Health. 2011; 23(8): 955-1012	Nonfatal Health Outcomes	Schizophrenia
Yang J, Ruan Y, Huang Y, Luo C, Yao J, Gao C, Lu J. Study on tobacco and alcohol induced mental disorders among residents aged over 15 in Kunming city in 2005. Prev Med Trib. 2009; 490Â–2	Nonfatal Health Outcomes	Alcohol use disorders
Yan J, Huang Y-Q, Ruan Y, Lu J, Gao C-Q, Dang W-M, Luo C. Epidemiological survey of affective disorder in Kunming City. Chin Ment Health J. 2010; 24(2): 110-115	Nonfatal Health Outcomes	Bipolar disorder
He S, Thomson WM. An oral epidemiological comparison of Chinese and New Zealand adults in 2 key age groups. Community Dent Oral Epidemiol. 2017	Nonfatal Health Outcomes	Caries of permanent teeth
He S, Thomson WM. An oral epidemiological comparison of Chinese and New Zealand adults in 2 key age groups. Community Dent Oral Epidemiol. 2017	Nonfatal Health Outcomes	Periodontal diseases
He S, Thomson WM. An oral epidemiological comparison of Chinese and New Zealand adults in 2 key age groups. Community Dent Oral Epidemiol. 2017	Nonfatal Health Outcomes	Edentulism and severe tooth loss
Lu B, Yang Z, Wang M, Yang Z, Gong W, Yang Y, Wen J, Zhang Z, Zhao N, Zhu X, Hu R. High prevalence of diabetic neuropathy in population-based patients diagnosed with type 2 diabetes in the Shanghai downtown. Diabetes Res Clin Pract. 2010; 88(3): 289-94	Nonfatal Health Outcomes	Diabetes mellitus
Wu M, Yip JLY, Kuper H. Rapid assessment of avoidable blindness in Kunming, China. Ophthalmology. 2008; 115(6): 969-74	Nonfatal Health Outcomes	
Yang Y, Zhu Y, Li D, Li Z, LÃ¼ H, Wu J, Tang J, Tong S. Characterization of glucose-6-phosphate dehydrogenase deficiency and identification of a novel haplotype 487G>A/IVS5-612(G>C) in the Achang population of Southwestern China. Sci China C Life Sci. 2007; 50(4): 479-85	Nonfatal Health Outcomes	G6PD deficiency
United Nations Children's Fund (UNICEF). Child Mortality and Injury in Asia. New York, United States: United Nations Children's Fund (UNICEF), 2008. (Innocenti Working Papers: Special Series on Child Injuries Nos. 1-4)	Nonfatal Health Outcomes	Exposure to mechanical forces
Li G, de Courten M, Jiao S, Wang Y. Prevalence and characteristics of the metabolic syndrome among adults in Beijing, China. Asia Pac J Clin Nutr. 2010; 19(1): 98Â–102	Nonfatal Health Outcomes	Diabetes mellitus
Huang W, Yang X, Yang JY. Investigation on prevalence of dementia among elderly in urban communities of Guiyang city. Chin J Public Health. 2007; 23(8): 983-6	Nonfatal Health Outcomes	Alzheimer's disease and other dementias
International Clearinghouse for Birth Defects Surveillance and Research. International Clearinghouse for Birth Defects Surveillance and Research Annual Report 2007. Rome, Italy: International Clearinghouse for Birth Defects Surveillance and Research, 2008	Nonfatal Health Outcomes	Down syndrome
Xu G-L,Wang C-H,Yang G-F,Xu G-M,Zhen L. Investigation on children's attention and disruptive behavior disorder in the town of Cangtai, Henan province. J Clin Psychiatry. 2011; 21(4): 255-257	Nonfatal Health Outcomes	Attention-deficit/hyperactivity disorder
Xu G-L,Wang C-H,Yang G-F,Xu G-M,Zhen L. Investigation on children's attention and disruptive behavior disorder in the town of Cangtai, Henan province. J Clin Psychiatry. 2011; 21(4): 255-257	Nonfatal Health Outcomes	Conduct disorder
Pang S-T, Wang G-J, Kong L-L, Zhang Y-D, Sun B, Yin S, Li X-Y, Phillips Michael R. Epidemiological survey of mental illnesses in persons aged 18 years and older in Qingdao City. Chin Ment Health J. 2010; 24(3): 175-182	Nonfatal Health Outcomes	Schizophrenia
Gong Z, Zhang K, Zhang C, Liu X, Cao C, Liang Y, al et. Epidemiological survey on alcohol dependence in Weihai city, Shandong Province. Chin J Drug Abuse Prev Treat. 2008; 207Â–9	Nonfatal Health Outcomes	Alcohol use disorders
Pang S-T, Wang G-J, Kong L-L, Zhang Y-D, Sun B, Yin S, Li X-Y, Phillips Michael R. Epidemiological survey of mental illnesses in persons aged 18 years and older in Qingdao City. Chin Ment Health J. 2010; 24(3): 175-182	Nonfatal Health Outcomes	Alcohol use disorders
Liang X, Bi S, Yang W, Wang L, Cui G, Cui F, Zhang Y, Liu J, Gong X, Chen Y, Wang F, Zheng H, Wang F, Guo J, Jia Z, Ma J, Wang H, Luo H, Li L, Jin S, Hadler SC, Wang Y. Reprint of: Epidemiological serosurvey of Hepatitis B in China--declining HBV prevalence due to Hepatitis B vaccination. Vaccine. 2013; J21-28	Nonfatal Health Outcomes	Acute hepatitis B
Pang S-T, Wang G-J, Kong L-L, Zhang Y-D, Sun B, Yin S, Li X-Y, Phillips Michael R. Epidemiological survey of mental illnesses in persons aged 18 years and older in Qingdao City. Chin Ment Health J. 2010; 24(3): 175-182	Nonfatal Health Outcomes	Anxiety disorders
Hairong Nan, Zengchang Pang, Shaojie Wang, Weiguo Gao, Lei Zhang, Jie Ren, Feng Ning, Tuomilehto J, Qing Qiao. Serum uric acid, plasma glucose and diabetes. Diab Vasc Dis Res. 2010; 7(1): 40Â–6	Nonfatal Health Outcomes	Diabetes mellitus
Hong Kong Cancer Registry. Hong Kong Cancer Registry Annual Tables. Kowloon, Hong Kong: Hong Kong Cancer Registry	Nonfatal Health Outcomes	Nasopharynx cancer

Hong Kong Cancer Registry. Hong Kong Cancer Registry Annual Tables. Kowloon, Hong Kong: Hong Kong Cancer Registry	Nonfatal Health Outcomes	Malignant skin melanoma
Hong Kong Cancer Registry. Hong Kong Cancer Registry Annual Tables. Kowloon, Hong Kong: Hong Kong Cancer Registry	Nonfatal Health Outcomes	Mesothelioma
Guo Y-L, Ou Y, Gao X-X, Li W-W, Li W, Z S-E. Investigation on the Clonorchiasis sinensis infection in Zhaoqing City. J Trop Med. 2007; 7(12): 1221-23	Nonfatal Health Outcomes	Food-borne trematodiases
Cao Y, Yi Z-W, Zhang H, Dang X-Q, Wu X-C, Huang A-W. Etiology and outcomes of acute kidney injury in Chinese children: a prospective multicentre investigation. BMC Urol. 2013; 41	Nonfatal Health Outcomes	Acute glomerulonephritis
Liu QG, Sun J, Xiao XW, Song GR. Birth defects data from surveillance hospitals in Dalian city, China, 2006-2010. J Matern Fetal Neonatal Med. 2016; 29(22): 3615-21	Nonfatal Health Outcomes	Orofacial clefts
Wu S, Qiu Y, Yan P, Wang W, Xie M, Xia P, Wang Z, Yan Y. Effectiveness of first-line antiretroviral therapy in HIV/AIDS patients: A 5-year longitudinal evaluation in Fujian Province, Southeast China. Arch Virol. 2015; 160(11): 2693Â–701	Nonfatal Health Outcomes	HIV/AIDS
Liu QG, Sun J, Xiao XW, Song GR. Birth defects data from surveillance hospitals in Dalian city, China, 2006-2010. J Matern Fetal Neonatal Med. 2016; 29(22): 3615-21	Nonfatal Health Outcomes	Neural tube defects
Liu QG, Sun J, Xiao XW, Song GR. Birth defects data from surveillance hospitals in Dalian city, China, 2006-2010. J Matern Fetal Neonatal Med. 2016; 29(22): 3615-21	Nonfatal Health Outcomes	Urogenital congenital anomalies
Wang D, Liu J, Liu M, Lu C, Brainin M, Zhang J. Patterns of Stroke Between University Hospitals and Nonuniversity Hospitals in Mainland China: Prospective Multicenter Hospital-Based Registry Study. World Neurosurg. 2017; 98: 258Â–65	Nonfatal Health Outcomes	Subarachnoid hemorrhage
Zhang H, Xu W, Dahl AK, Xu Z, Wang H-X, Qi X. Relation of socio-economic status to impaired fasting glucose and Type 2 diabetes: findings based on a large population-based cross-sectional study in Tianjin, China. Diabet Med. 2013; 30(5): e157Â–62	Nonfatal Health Outcomes	Diabetes mellitus
Long J, Wang Y, Li M, Tong W-M, Jia J-D, Huang J. Correlation of TP53 mutations with HCV positivity in hepatocarcinogenesis: identification of a novel TP53 microindel in hepatocellular carcinoma with HCV infection . Oncol Rep. 2013; 30(1): 119-24	Nonfatal Health Outcomes	Liver cancer due to hepatitis B
Ruan Y, Huang Y, Xu Y, Wu M, Yang J, Yao J, Dang W, Gao C, Luo C. Epidemiological Survey of Mental and Behavior Disorders in Kunming. Modern Prev Med. 2010; 37(4): 628-32	Nonfatal Health Outcomes	Schizophrenia
World Health Organization (WHO). WHO Tuberculosis Case Notifications. Geneva, Switzerland: World Health Organization (WHO)	Nonfatal Health Outcomes	None
Ding Z-J, Wang G-P, Pei G-X, Guan R-C, He R-F, Phillips Michael R, Li X-Y, Zhang Y-L, Wang D-M, Hong G-Z, Du H-Y, Bai A-P, Li G, Wang X-Q, Wei C-L, Li H, Wang J. Epidemiological survey of mental disorders in people aged 18 and older in Tianshui City of Gansu Province. Chin Ment Health J. 2010; 24(3): 183-190	Nonfatal Health Outcomes	Schizophrenia
Chinese Center for Disease Control and Prevention (CCDC). China Zhuhai Study 2006-2007 - China CDC	Nonfatal Health Outcomes	Injuries
World Health Organization (WHO). WHO PCT Databank - Soil-transmitted Helminthiases. Geneva, Switzerland: World Health Organization (WHO)	Nonfatal Health Outcomes	Intestinal nematode infections
Ding Z-J, Wang G-P, Pei G-X, Guan R-C, He R-F, Phillips Michael R, Li X-Y, Zhang Y-L, Wang D-M, Hong G-Z, Du H-Y, Bai A-P, Li G, Wang X-Q, Wei C-L, Li H, Wang J. Epidemiological survey of mental disorders in people aged 18 and older in Tianshui City of Gansu Province. Chin Ment Health J. 2010; 24(3): 183-190	Nonfatal Health Outcomes	Alcohol use disorders
Wang T, Bi Y, Xu M, Huang Y, Xu Y, Li X, Wang W, Ning G. Serum uric acid associates with the incidence of type 2 diabetes in a prospective cohort of middle-aged and elderly Chinese. Endocrine. 2011; 40(1): 109Â–16	Nonfatal Health Outcomes	Diabetes mellitus
Yu Z, Xi Y, Ding W, Han S, Cao L, Zhu C, Wang X, Guo X. Congenital heart disease in a Chinese hospital: pre- and postnatal detection, incidence, clinical characteristics and outcomes. Pediatr Int. 2011; 53(6): 1059-65	Nonfatal Health Outcomes	Congenital heart anomalies
Chen H-M, Gongalanzi, Shi X-M, Pu Z. Epidemiological Investigation on Alcohol Dependence in Rural Area of Lhasa. Tibets Sci Technol. 2009; 5: 29-30	Nonfatal Health Outcomes	Alcohol use disorders
Liu W-J, Yu D-N, Luo W-Q, Liang Q-R, Pan H-Z. Analysis of the results of a baseline survey of clonorchiasis in Taishan City, Guangdong, China. China Tropical Medicine. 2007; 7(4): 602-03	Nonfatal Health Outcomes	Food-borne trematodiases
Fan S-Y, Huang C-L, Liu Y, Li L-L, Shi X-H. [Epidemiology study of clonorchiasis in Futian district of Shenzhen]. J Trop Med. 2008; 1: 75-76	Nonfatal Health Outcomes	Food-borne trematodiases
Taniguchi M, Kim SR, Mishiro S, Takahashi K, Shin MH, Yun H, Park MS, Li ZM, Kim MK, Fang J, Hayashi Y. Epidemiology of hepatitis E in Northeastern China, South Korea and Japan. J Infect. 2009; 58(3): 232-7	Nonfatal Health Outcomes	Acute hepatitis E
Long J, Wang Y, Li M, Tong W-M, Jia J-D, Huang J. Correlation of TP53 mutations with HCV positivity in hepatocarcinogenesis: identification of a novel TP53 microindel in hepatocellular carcinoma with HCV infection . Oncol Rep. 2013; 30(1): 119-24	Nonfatal Health Outcomes	Liver cancer due to hepatitis C

Zhao Z-H, Huang Q-Y, Li J, Deng H-H, Huang X-M, Su J-H, Dang W-M, Yang L, Huang J-K, Zhang W-M, Deng Y, Zhou W-C, Qiu C, Lu W-C, Chen Y-W, Zhong S-J, Chen B-Y, Zeng Q-M, Mei F. An Epidemiological survey of mental disorders in Guangzhou area. Chin J Nerv Ment Dis. 2009; 35(9): 530-534	Nonfatal Health Outcomes	Schizophrenia
Sung RYT, Chan PKS, Tsen T, Li AM, Lam WY, Yeung ACM, Nelson EAS. Identification of viral and atypical bacterial pathogens in children hospitalized with acute respiratory infections in Hong Kong by multiplex PCR assays. J Med Virol. 2009; 81(1): 153-9	Nonfatal Health Outcomes	Lower respiratory infections
Zhao Z-H, Huang Q-Y, Li J, Deng H-H, Huang X-M, Su J-H, Dang W-M, Yang L, Huang J-K, Zhang W-M, Deng Y, Zhou W-C, Qiu C, Lu W-C, Chen Y-W, Zhong S-J, Chen B-Y, Zeng Q-M, Mei F. An Epidemiological survey of mental disorders in Guangzhou area. Chin J Nerv Ment Dis. 2009; 35(9): 530-534	Nonfatal Health Outcomes	Alcohol use disorders
Chinese Center for Disease Control and Prevention (CCDC). China Notifiable Infectious Diseases 2007	Nonfatal Health Outcomes	Rabies
Bromet E, Andrade LH, Hwang I, Sampson NA, Alonso J, de Girolamo G, de Graaf R, Demyttenaere K, Hu C, Iwata N, Karam AN, Kaur J, Kostyuchenko S, LÃ©pine J-P, Levinson D, Matschinger H, Mora MEM, Browne MO, Posada-Villa J, Viana MC, Williams DR, Kessler RC. Cross-national epidemiology of DSM-IV major depressive episode. BMC Med. 2011; 9: 90	Nonfatal Health Outcomes	Major depressive disorder
Merikangas KR, Jin R, He J-P, Kessler RC, Lee S, Sampson NA, Viana MC, Andrade LH, Hu C, Karam EG, Ladea M, Medina-Mora ME, Ono Y, Posada-Villa J, Sagar R, Wells JE, Zarkov Z. Prevalence and correlates of bipolar spectrum disorder in the World Mental Health Survey Initiative. Arch Gen Psychiatry. 2011; 68(3): 241-51	Nonfatal Health Outcomes	Bipolar disorder
Yu J-C, Tang M-N. Epidemiological survey of mental disorders in urban and rural areas of Guangzhou. Guangzhou Medical College. 2010; Thesis: 1-47	Nonfatal Health Outcomes	Bulimia nervosa
Ministry of Health (China), National Center for Chronic and Noncommunicable Disease Control and Prevention (China), World Health Organization (WHO). China WHO Study on Global AGEing and Adult Health 2007-2010	Nonfatal Health Outcomes	Low back pain
Chinese Center for Disease Control and Prevention (CCDC). China Notifiable Infectious Diseases 2008	Nonfatal Health Outcomes	Rabies
Wang H, Qiu Q, Tan LL, Liu T, Deng XQ, Chen YM, Chen W, Yu XQ, Hu BJ, Chen WQ. Prevalence and determinants of diabetes and impaired fasting glucose among urban community-dwelling adults in Guangzhou, China. Diabetes Metab. 2009; 35(5): 378-84	Nonfatal Health Outcomes	Diabetes mellitus
Chinese Center for Disease Control and Prevention (CCDC), Ministry of Health (China). China National Injury Surveillance System 2008 - China CDC	Nonfatal Health Outcomes	Other exposure to mechanical forces
Chen W, Chen W, Wang H, Dong X, Liu Q, Mao H, Tan J, Lin J, Zhou F, Luo N, He H, Johnson RJ, Zhou SF, Yu X. Prevalence and risk factors associated with chronic kidney disease in an adult population from southern China. Nephrol Dial Transplant. 2009; 24(4): 1205-12	Nonfatal Health Outcomes	Chronic kidney disease
Chinese Center for Disease Control and Prevention (CCDC), Ministry of Health (China). China National Injury Surveillance System 2009 - China CDC	Nonfatal Health Outcomes	Exposure to mechanical forces
Chinese Center for Disease Control and Prevention (CCDC). China Notifiable Infectious Diseases 2009	Nonfatal Health Outcomes	Rabies
International Society of Nephrology (ISN). China - Guilin International Society of Nephrology Kidney Disease Data Center Chronic Kidney Disease and Cardiovascular Risk Survey 2007-2011	Nonfatal Health Outcomes	Chronic kidney disease
Chinese Center for Disease Control and Prevention (CCDC), Ministry of Health (China). China National Injury Surveillance System 2007 - China CDC	Nonfatal Health Outcomes	Road injuries
Chinese Center for Disease Control and Prevention (CCDC), Ministry of Health (China). China National Injury Surveillance System 2008 - China CDC	Nonfatal Health Outcomes	Road injuries
Yao W, Mai X, Luo C, Ai F, Chen Q. A cross-sectional survey of nonspecific low back pain among 2083 schoolchildren in China. Spine. 2011; 36(22): 1885-90	Nonfatal Health Outcomes	Low back pain
Zhao J, Bai J, Shen K, Xiang L, Huang S, Chen A, Huang Y, Wang J, Ye R. Self-reported prevalence of childhood allergic diseases in three cities of China: a multicenter study. BMC Public Health. 2010; 10: 551	Nonfatal Health Outcomes	Atopic dermatitis
Chinese Center for Disease Control and Prevention (CCDC), Ministry of Health (China). China National Injury Surveillance System 2009 - China CDC	Nonfatal Health Outcomes	Road injuries
Wang H, Zhang Y, Li Z, Wang T, Liu P. Prevalence and causes of corneal blindness. Clin Experiment Ophthalmol. 2013	Nonfatal Health Outcomes	Glaucoma
Wang L, Huang W, He M, Zheng Y, Huang S, Liu B, Jin L, Congdon NG, He M. Causes and five-year incidence of blindness and visual impairment in urban Southern China: the Liwan Eye Study. Invest Ophthalmol Vis Sci. 2013; 54(6): 4117-21	Nonfatal Health Outcomes	Glaucoma
Wang H, Zhang Y, Li Z, Wang T, Liu P. Prevalence and causes of corneal blindness. Clin Experiment Ophthalmol. 2013	Nonfatal Health Outcomes	Cataract

Wang L, Huang W, He M, Zheng Y, Huang S, Liu B, Jin L, Congdon NG, He M. Causes and five-year incidence of blindness and visual impairment in urban Southern China: the Liwan Eye Study. Invest Ophthalmol Vis Sci. 2013; 54(6): 4117-21	Nonfatal Health Outcomes	Cataract
Wang H, Zhang Y, Li Z, Wang T, Liu P. Prevalence and causes of corneal blindness. Clin Experiment Ophthalmol. 2013	Nonfatal Health Outcomes	Age-related macular degeneration
Wang L, Huang W, He M, Zheng Y, Huang S, Liu B, Jin L, Congdon NG, He M. Causes and five-year incidence of blindness and visual impairment in urban Southern China: the Liwan Eye Study. Invest Ophthalmol Vis Sci. 2013; 54(6): 4117-21	Nonfatal Health Outcomes	Age-related macular degeneration
Congdon N, Wang Y, Song Y, Choi K, Zhang M, Zhou Z, Xie Z, Li L, Liu X, Sharma A, Wu B, Lam DSC. Visual disability, visual function, and myopia among rural Chinese secondary school children: the Xichang Pediatric Refractive Error Study (X-PRES)Â–Report 1. Invest Ophthalmol Vis Sci. 2008; 49(7): 2888-94	Nonfatal Health Outcomes	Refraction and accommodation disorders
Zhao J, Ellwein LB, Cui H, Ge J, Guan H, Lv J, Ma X, Yin J, Yin ZQ, Yuan Y, Liu H. Prevalence of vision impairment in older adults in rural China: the China Nine-Province Survey. Ophthalmology. 2010; 117(3): 409-16	Nonfatal Health Outcomes	Refraction and accommodation disorders
Chinese Center for Disease Control and Prevention (CCDC). China Disease Surveillance Points and Death Registration 2008 - China CDC	Nonfatal Health Outcomes	Rabies
Wang H, Zhang Y, Li Z, Wang T, Liu P. Prevalence and causes of corneal blindness. Clin Experiment Ophthalmol. 2013	Nonfatal Health Outcomes	Other vision loss
Chinese Center for Disease Control and Prevention (CCDC), Ministry of Health (China). China National Injury Surveillance System 2007 - China CDC	Nonfatal Health Outcomes	Falls
Chinese Center for Disease Control and Prevention (CCDC), Ministry of Health (China). China National Injury Surveillance System 2008 - China CDC	Nonfatal Health Outcomes	Falls
Chinese Center for Disease Control and Prevention (CCDC), Ministry of Health (China). China National Injury Surveillance System 2009 - China CDC	Nonfatal Health Outcomes	Falls
World Health Organization (WHO). WHO Global Project on Anti-Tuberculosis Drug Resistance Surveillance Data 1988-2015	Nonfatal Health Outcomes	Extensively drug-resistant tuberculosis
World Health Organization (WHO). WHO Global Project on Anti-Tuberculosis Drug Resistance Surveillance Data 1988-2015	Nonfatal Health Outcomes	HIV/AIDS - Extensively drug-resistant Tuberculosis
Chinese Center for Disease Control and Prevention (CCDC), Ministry of Health (China). China National Injury Surveillance System 2007 - China CDC	Nonfatal Health Outcomes	Drowning
Chinese Center for Disease Control and Prevention (CCDC), Ministry of Health (China). China National Injury Surveillance System 2008 - China CDC	Nonfatal Health Outcomes	Drowning
Chinese Center for Disease Control and Prevention (CCDC), Ministry of Health (China). China National Injury Surveillance System 2009 - China CDC	Nonfatal Health Outcomes	Drowning
Chinese Center for Disease Control and Prevention (CCDC), Ministry of Health (China). China National Injury Surveillance System 2007 - China CDC	Nonfatal Health Outcomes	Fire, heat, and hot substances
Chinese Center for Disease Control and Prevention (CCDC), Ministry of Health (China). China National Injury Surveillance System 2008 - China CDC	Nonfatal Health Outcomes	Fire, heat, and hot substances
Chinese Center for Disease Control and Prevention (CCDC), Ministry of Health (China). China National Injury Surveillance System 2009 - China CDC	Nonfatal Health Outcomes	Fire, heat, and hot substances
Chinese Center for Disease Control and Prevention (CCDC), Ministry of Health (China). China National Injury Surveillance System 2007 - China CDC	Nonfatal Health Outcomes	Poisonings
Chinese Center for Disease Control and Prevention (CCDC), Ministry of Health (China). China National Injury Surveillance System 2008 - China CDC	Nonfatal Health Outcomes	Poisonings
Chinese Center for Disease Control and Prevention (CCDC), Ministry of Health (China). China National Injury Surveillance System 2009 - China CDC	Nonfatal Health Outcomes	Poisonings
Chinese Center for Disease Control and Prevention (CCDC), Ministry of Health (China). China National Injury Surveillance System 2007 - China CDC	Nonfatal Health Outcomes	Exposure to mechanical forces
Chinese Center for Disease Control and Prevention (CCDC), Ministry of Health (China). China National Injury Surveillance System 2008 - China CDC	Nonfatal Health Outcomes	Exposure to mechanical forces
Chinese Center for Disease Control and Prevention (CCDC), Ministry of Health (China). China National Injury Surveillance System 2007 - China CDC	Nonfatal Health Outcomes	Adverse effects of medical treatment
Chinese Center for Disease Control and Prevention (CCDC), Ministry of Health (China). China National Injury Surveillance System 2008 - China CDC	Nonfatal Health Outcomes	Adverse effects of medical treatment
Chinese Center for Disease Control and Prevention (CCDC), Ministry of Health (China). China National Injury Surveillance System 2009 - China CDC	Nonfatal Health Outcomes	Adverse effects of medical treatment
Chinese Center for Disease Control and Prevention (CCDC), Ministry of Health (China). China National Injury Surveillance System 2007 - China CDC	Nonfatal Health Outcomes	Animal contact
Chinese Center for Disease Control and Prevention (CCDC), Ministry of Health (China). China National Injury Surveillance System 2008 - China CDC	Nonfatal Health Outcomes	Animal contact
Chinese Center for Disease Control and Prevention (CCDC), Ministry of Health (China). China National Injury Surveillance System 2009 - China CDC	Nonfatal Health Outcomes	Animal contact
International Centre for Eye Health (ICEH). China - Luliang County Rapid Assessment for Avoidable Blindness 2008	Nonfatal Health Outcomes	Cataract

Zhu Y-Z, Jia Y, Liu R, Liu Z-F, Wang X. The Condition of Mental Disorders and the Analysis of the Recovery Management in the Community. Guide Chin Med. 2012; 10(24): 452-4	Nonfatal Health Outcomes	Schizophrenia
Chinese Center for Disease Control and Prevention (CCDC), Ministry of Health (China). China National Injury Surveillance System 2007 - China CDC	Nonfatal Health Outcomes	Pulmonary aspiration and foreign body in airway
Chinese Center for Disease Control and Prevention (CCDC), Ministry of Health (China). China National Injury Surveillance System 2008 - China CDC	Nonfatal Health Outcomes	Pulmonary aspiration and foreign body in airway
Chinese Center for Disease Control and Prevention (CCDC), Ministry of Health (China). China National Injury Surveillance System 2009 - China CDC	Nonfatal Health Outcomes	Pulmonary aspiration and foreign body in airway
Chinese Center for Disease Control and Prevention (CCDC), Ministry of Health (China). China National Injury Surveillance System 2007 - China CDC	Nonfatal Health Outcomes	Self-harm
Chinese Center for Disease Control and Prevention (CCDC), Ministry of Health (China). China National Injury Surveillance System 2008 - China CDC	Nonfatal Health Outcomes	Self-harm
Chinese Center for Disease Control and Prevention (CCDC), Ministry of Health (China). China National Injury Surveillance System 2009 - China CDC	Nonfatal Health Outcomes	Self-harm
Chinese Center for Disease Control and Prevention (CCDC), Ministry of Health (China). China National Injury Surveillance System 2007 - China CDC	Nonfatal Health Outcomes	Interpersonal violence
Chinese Center for Disease Control and Prevention (CCDC), Ministry of Health (China). China National Injury Surveillance System 2008 - China CDC	Nonfatal Health Outcomes	Interpersonal violence
Chinese Center for Disease Control and Prevention (CCDC), Ministry of Health (China). China National Injury Surveillance System 2009 - China CDC	Nonfatal Health Outcomes	Interpersonal violence
Congdon N, Wang Y, Song Y, Choi K, Zhang M, Zhou Z, Xie Z, Li L, Liu X, Sharma A, Wu B, Lam DSC. Visual disability, visual function, and myopia among rural Chinese secondary school children: the Xichang Pediatric Refractive Error Study (X-PRES)Â–Report 1. Invest Ophthalmol Vis Sci. 2008; 49(7): 2888-94	Nonfatal Health Outcomes	Vision loss
Wang L, Huang W, He M, Zheng Y, Huang S, Liu B, Jin L, Congdon NG, He M. Causes and five-year incidence of blindness and visual impairment in urban Southern China: the Liwan Eye Study. Invest Ophthalmol Vis Sci. 2013; 54(6): 4117-21	Nonfatal Health Outcomes	Vision loss
Zhao J, Ellwein LB, Cui H, Ge J, Guan H, Lv J, Ma X, Yin J, Yin ZQ, Yuan Y, Liu H. Prevalence of vision impairment in older adults in rural China: the China Nine-Province Survey. Ophthalmology. 2010; 117(3): 409-16	Nonfatal Health Outcomes	Vision loss
Xiu-Xiang Li, Yan Yang. Epidemiology survey of epilepsy in Chengde. Hebei Med. 2012; 18(11): 1676-8	Nonfatal Health Outcomes	Epilepsy
Ministry of Health (China), National Center for Chronic and Noncommunicable Disease Control and Prevention (China), World Health Organization (WHO). China WHO Study on Global AGEing and Adult Health 2007-2010	Nonfatal Health Outcomes	Low back and neck pain
Chinese Center for Disease Control and Prevention (CCDC). China Disease Surveillance Points and Death Registration 2009 - China CDC	Nonfatal Health Outcomes	Rabies
Chinese Center for Disease Control and Prevention (CCDC), Ministry of Health (China), World Health Organization (WHO). China National Tuberculosis Prevalence Survey 2010	Nonfatal Health Outcomes	Tuberculosis
Zhu M, Tong X, Zhao R, He X, Zhao H, Liu M, Zhu J. Visual impairment and spectacle coverage rate in Baoshan district, China: population-based study. BMC Public Health. 2013; 13(1): 311	Nonfatal Health Outcomes	Refraction disorders
Zhu M, Tong X, Zhao R, He X, Zhao H, Liu M, Zhu J. Visual impairment and spectacle coverage rate in Baoshan district, China: population-based study. BMC Public Health. 2013; 13(1): 311	Nonfatal Health Outcomes	Refraction and accommodation disorders
Chinese Center for Disease Control and Prevention (CCDC), Ministry of Health (China). China National Injury Surveillance System 2007 - China CDC	Nonfatal Health Outcomes	Unintentional firearm injuries
Chinese Center for Disease Control and Prevention (CCDC), Ministry of Health (China). China National Injury Surveillance System 2008 - China CDC	Nonfatal Health Outcomes	Unintentional firearm injuries
Chinese Center for Disease Control and Prevention (CCDC), Ministry of Health (China). China National Injury Surveillance System 2009 - China CDC	Nonfatal Health Outcomes	Unintentional firearm injuries
Chinese Center for Disease Control and Prevention (CCDC), Ministry of Health (China). China National Injury Surveillance System 2007 - China CDC	Nonfatal Health Outcomes	Other exposure to mechanical forces
Chinese Center for Disease Control and Prevention (CCDC), Ministry of Health (China). China National Injury Surveillance System 2009 - China CDC	Nonfatal Health Outcomes	Other exposure to mechanical forces
Chinese Center for Disease Control and Prevention (CCDC), Ministry of Health (China). China National Injury Surveillance System 2007 - China CDC	Nonfatal Health Outcomes	Venomous animal contact
Chinese Center for Disease Control and Prevention (CCDC), Ministry of Health (China). China National Injury Surveillance System 2008 - China CDC	Nonfatal Health Outcomes	Venomous animal contact
Chinese Center for Disease Control and Prevention (CCDC), Ministry of Health (China). China National Injury Surveillance System 2009 - China CDC	Nonfatal Health Outcomes	Venomous animal contact
Chinese Center for Disease Control and Prevention (CCDC), Ministry of Health (China). China National Injury Surveillance System 2007 - China CDC	Nonfatal Health Outcomes	Non-venomous animal contact

Chinese Center for Disease Control and Prevention (CCDC), Ministry of Health (China). China National Injury Surveillance System 2008 - China CDC	Nonfatal Health Outcomes	Non-venomous animal contact
Chinese Center for Disease Control and Prevention (CCDC), Ministry of Health (China). China National Injury Surveillance System 2009 - China CDC	Nonfatal Health Outcomes	Non-venomous animal contact
Chinese Center for Disease Control and Prevention (CCDC), Ministry of Health (China). China National Injury Surveillance System 2007 - China CDC	Nonfatal Health Outcomes	Foreign body in eyes
Chinese Center for Disease Control and Prevention (CCDC), Ministry of Health (China). China National Injury Surveillance System 2008 - China CDC	Nonfatal Health Outcomes	Foreign body in eyes
Chinese Center for Disease Control and Prevention (CCDC), Ministry of Health (China). China National Injury Surveillance System 2009 - China CDC	Nonfatal Health Outcomes	Foreign body in eyes
Chinese Center for Disease Control and Prevention (CCDC), Ministry of Health (China). China National Injury Surveillance System 2007 - China CDC	Nonfatal Health Outcomes	Foreign body in other body part
Chinese Center for Disease Control and Prevention (CCDC), Ministry of Health (China). China National Injury Surveillance System 2008 - China CDC	Nonfatal Health Outcomes	Foreign body in other body part
Chinese Center for Disease Control and Prevention (CCDC), Ministry of Health (China). China National Injury Surveillance System 2009 - China CDC	Nonfatal Health Outcomes	Foreign body in other body part
Chinese Center for Disease Control and Prevention (CCDC), Ministry of Health (China). China National Injury Surveillance System 2007 - China CDC	Nonfatal Health Outcomes	Physical violence by firearm
Chinese Center for Disease Control and Prevention (CCDC), Ministry of Health (China). China National Injury Surveillance System 2008 - China CDC	Nonfatal Health Outcomes	Physical violence by firearm
Chinese Center for Disease Control and Prevention (CCDC), Ministry of Health (China). China National Injury Surveillance System 2009 - China CDC	Nonfatal Health Outcomes	Physical violence by firearm
Chinese Center for Disease Control and Prevention (CCDC), Ministry of Health (China). China National Injury Surveillance System 2007 - China CDC	Nonfatal Health Outcomes	Physical violence by sharp object
Chinese Center for Disease Control and Prevention (CCDC), Ministry of Health (China). China National Injury Surveillance System 2008 - China CDC	Nonfatal Health Outcomes	Physical violence by sharp object
Chinese Center for Disease Control and Prevention (CCDC), Ministry of Health (China). China National Injury Surveillance System 2009 - China CDC	Nonfatal Health Outcomes	Physical violence by sharp object
Chinese Center for Disease Control and Prevention (CCDC), Ministry of Health (China). China National Injury Surveillance System 2007 - China CDC	Nonfatal Health Outcomes	Physical violence by other means
Chinese Center for Disease Control and Prevention (CCDC), Ministry of Health (China). China National Injury Surveillance System 2008 - China CDC	Nonfatal Health Outcomes	Physical violence by other means
Chinese Center for Disease Control and Prevention (CCDC), Ministry of Health (China). China National Injury Surveillance System 2009 - China CDC	Nonfatal Health Outcomes	Physical violence by other means
Wang H, Zhang Y, Li Z, Wang T, Liu P. Prevalence and causes of corneal blindness. Clin Experiment Ophthalmol. 2013	Nonfatal Health Outcomes	Diabetes mellitus
Meng H-M, Li Na CL. A survey on the epidemiology of epilepsy in partial rural areas of Jilin Province. J Apoplexy Nerv Dis. 2010; 12	Nonfatal Health Outcomes	Epilepsy
Zhao Y-H, Zhang Q, Long N, Yang C, Hong J, Mu L, Zhou D. Prevalence of epilepsy and alcohol-related risk in Zayul County, Tibet Autonomous Region in China: an initial survey. Epilepsy Behav. 2010; 19(4): 635-8	Nonfatal Health Outcomes	Epilepsy
Leung TF, Lam DS, Miu TY, Hon KL, Chau CS, Ku SW, Lee RS, Chow PY, Chiu WK, Ng DK. Epidemiology and risk factors for severe respiratory syncytial virus infections requiring pediatric intensive care admission in Hong Kong children. Infection. 2014; 42(2): 343-50	Nonfatal Health Outcomes	Respiratory syncytial virus pneumonia
China National Maternal and Child Health Surveillance System Congenital Anomalies 1996-2012 - MCHS	Nonfatal Health Outcomes	Turner syndrome
China National Maternal and Child Health Surveillance System Congenital Anomalies 1996-2012 - MCHS	Nonfatal Health Outcomes	Klinefelter syndrome
World Health Organization Regional Office for the Western Pacific (WPRO-WHO). Number of Reported Cases of Dengue Fever and Dengue Hemorrhagic Fever in the Western Pacific Region, by Country 2007. Manila, Philippines: World Health Organization Regional Office for the Western Pacific (WPRO-WHO)	Nonfatal Health Outcomes	Dengue
World Health Organization Regional Office for the Western Pacific (WPRO-WHO). Number of Reported Cases of Dengue Fever and Dengue Hemorrhagic Fever in the Western Pacific Region, by Country 2008. Manila, Philippines: World Health Organization Regional Office for the Western Pacific (WPRO-WHO)	Nonfatal Health Outcomes	Dengue
World Health Organization Regional Office for the Western Pacific (WPRO-WHO). Number of Reported Cases of Dengue Fever and Dengue Hemorrhagic Fever in the Western Pacific Region, by Country 2009. Manila, Philippines: World Health Organization Regional Office for the Western Pacific (WPRO-WHO)	Nonfatal Health Outcomes	Dengue
World Health Organization (WHO). Global leprosy situation, 2009. Wkly Epidemiol Rec. 2009: 84(33): 333-40.	Nonfatal Health Outcomes	Leprosy
World Health Organization (WHO). Global leprosy situation, 2010. Wkly Epidemiol Rec. 2010: 85(35): 337-48.	Nonfatal Health Outcomes	Leprosy

World Health Organization (WHO). Global leprosy situation, beginning of 2008. Wkly Epidemiol Rec. 2008: 83(33): 293-300.	Nonfatal Health Outcomes	Leprosy
Ministry of Health (China), National Center for Chronic and Noncommunicable Disease Control and Prevention (China), World Health Organization (WHO). China WHO Study on Global AGEing and Adult Health 2007-2010	Nonfatal Health Outcomes	Ischemic stroke
Feng L, Li Z, Zhao S, Nair H, Lai S, Xu W, Li M, Wu J, Ren L, Liu W, Yuan Z, Chen Y, Wang X, Zhao Z, Zhang H, Li F, Ye X, Li S, Feikin D, Yu H, Yang W. Viral etiologies of hospitalized acute lower respiratory infection patients in China, 2009-2013. PLoS One. 2014; 9(6): e99419	Nonfatal Health Outcomes	Influenza
Lee S, Ng KL, Tsang A. A community survey of the twelve-month prevalence and correlates of bipolar spectrum disorder in Hong Kong. J Affect Disord. 2009; 117(1-2): 79-86	Nonfatal Health Outcomes	Bipolar disorder
Li Z, Cui H, Liu P, Zhang L, Yang H, Zhang L. Prevalence and causes of blindness and visual impairment among the elderly in rural southern Harbin, China. Ophthalmic Epidemiol. 2008; 15(5): 334-8	Nonfatal Health Outcomes	Cataract
Li Z, Cui H, Liu P, Zhang L, Yang H, Zhang L. Prevalence and causes of blindness and visual impairment among the elderly in rural southern Harbin, China. Ophthalmic Epidemiol. 2008; 15(5): 334-8	Nonfatal Health Outcomes	Age-related macular degeneration
Chu C-H, Ho P-L, Lo ECM. Oral health status and behaviours of preschool children in Hong Kong. BMC Public Health. 2012; 12(1): 767	Nonfatal Health Outcomes	Caries of deciduous teeth
Wong MCM, Lu HX, Lo ECM. Caries increment over 2 years in preschool children: a life course approach. Int J Paediatr Dent. 2012; 22(2): 77-84	Nonfatal Health Outcomes	Caries of deciduous teeth
Song W, Sun X, Shao Z, Zhou X, Kang Y, Sui H, Yuan H. Prevalence and causes of visual impairment in a rural North-east China adult population: a population-based survey in Bin County, Harbin. Acta Ophthalmol. 2010; 88(6): 669-74	Nonfatal Health Outcomes	Refraction and accommodation disorders
Department of Health (Hong Kong). Hong Kong Injury Survey 2008	Nonfatal Health Outcomes	Falls
Li Z, Cui H, Liu P, Zhang L, Yang H, Zhang L. Prevalence and causes of blindness and visual impairment among the elderly in rural southern Harbin, China. Ophthalmic Epidemiol. 2008; 15(5): 334-8	Nonfatal Health Outcomes	Other vision loss
Gu DF, Shi YL, Chen YM, Liu HM, Ding YN, Liu XY, Li YQ, Shao XF, Liang Y, Chen YS, Yuan ZY, Zou HQ. Prevalence of chronic kidney disease and prediabetes and associated risk factors: a community-based screening in Zhuhai, Southern China. Chin Med J. 2013; 126(7): 1213-9	Nonfatal Health Outcomes	Chronic kidney disease
Chinese Center for Disease Control and Prevention (CCDC). China Disease Surveillance Points 2007 - China CDC	Nonfatal Health Outcomes	Rabies
Chinese Center for Disease Control and Prevention (CCDC), Ministry of Health (China). China National Injury Surveillance System 2009 - China CDC	Nonfatal Health Outcomes	Injuries
International Centre for Eye Health (ICEH). China - Inner Mongolia Shangdu Rapid Assessment of Avoidable Blindness 2010	Nonfatal Health Outcomes	Vision loss
Ministry of Health (China), National Center for Chronic and Noncommunicable Disease Control and Prevention (China), World Health Organization (WHO). China WHO Study on Global AGEing and Adult Health 2007-2010	Nonfatal Health Outcomes	Intracerebral hemorrhage
Zhang J, Liu G, Arima H, Li Y, Cheng G, Shiue I, Lv L, Wang H, Zhang C, Zhao J, Anderson C. Incidence and risks of subarachnoid hemorrhage in China. Stroke. 2013; 44(10): 2891-3	Nonfatal Health Outcomes	Subarachnoid hemorrhage
Ministry of Health (China), National Center for Chronic and Noncommunicable Disease Control and Prevention (China), World Health Organization (WHO). China WHO Study on Global AGEing and Adult Health 2007-2010	Nonfatal Health Outcomes	Subarachnoid hemorrhage
Meng L, Luo N, Mi J. Impacts of types and degree of obesity on non-alcoholic fatty liver disease and related dyslipidemia in Chinese school-age children?. Biomed Environ Sci. 2011; 24(1): 22-30	Nonfatal Health Outcomes	Cirrhosis due to NASH
He J, Ma X, Zhao Y, Wang R, Yan X, Yan H, Yin P, Kang X, Fang J, Hao Y, Li Q, Dent J, Sung JJ, Zou D, Wallander MA, Johansson S, Liu W, Li Z. A population-based survey of the epidemiology of symptom-defined gastroesophageal reflux disease: the Systematic Investigation of Gastrointestinal Diseases in China. BMC Gastroenterol. 2010; 10: 94	Nonfatal Health Outcomes	Gastroesophageal reflux disease
Ministry of Health (China), National Center for Chronic and Noncommunicable Disease Control and Prevention (China), World Health Organization (WHO). China WHO Study on Global AGEing and Adult Health 2007-2010	Nonfatal Health Outcomes	Vision loss
Wang X, Yang W-H, Jin Y, Jing J, Huang X, Li X-H, Wei W, Bao P, Wang H, Hu M, Li S-Y, Xiu L-J, Guo J, Liang H-N, Ning J, Cheng S-Y, Fan Y-B. Prevalence of autism spectrum disorders in preschool children of Guangzhou kindergartens. Chin Ment Health J. 2011; 25(6): 401Â–408	Nonfatal Health Outcomes	Autism spectrum disorders
Liang M, Chen Q, Liu C, Gu Z, Mai M, Jiang Y. Analysis of screening for autism in preschool children in Maoming. Matern Child Health Care China. 2011; 26: 1164-1165	Nonfatal Health Outcomes	Autism spectrum disorders

Wei Z, Ren L, Weng L, Gao W, He H, Wang W, Wan G. Early screening on autism spectrum disorders in 18-24 months old children in Shenzhen: 3 years follow up study. Chin J Child Health Care. 2012; 20(4): 354Â–357	Nonfatal Health Outcomes	Autism spectrum disorders
Li Y, Yin Z, Shao Z, Li M, Liang X, Sandhu HS, Hadler SC, Li J, Sun Y, Li J, Zou W, Lin M, Zuo S, Mayer LW, Novak RT, Zhu B, Xu L, Luo H, Acute Meningitis and Encephalitis Syndrome Study Group. Population-based surveillance for bacterial meningitis in China, September 2006-December 2009. Emerg Infect Dis. 2014; 20(1): 61-9	Nonfatal Health Outcomes	Meningitis
Chronic Kidney Disease Prognosis Consortium (CKD-PC). China ChinaNS Chronic Kidney Disease Prevalence Data 2006-2010 [Unpublished]	Nonfatal Health Outcomes	Chronic kidney disease
Li X, Yang Y, Huang K, Xu H, Xie Z, Mai F, Shang S, Su A, Li S, Ou F, Wu Q, Wu W, Zhu X, Jiange Z. [Discovery of a residual focus of bancroftian filariasis after declaration of its transmission interruption in Guangxi]. Chin J Parasitol Parasit Dis. 2008; 26(6): 404-8	Nonfatal Health Outcomes	Lymphatic filariasis
Huang TR, Yu JH, Li JL, Zhang ZQ, Deng W, Zhang CY, Zhao SF. A cross-sectional study on liver diseases in the rural residents in southern Guangxi, China. Chin J Prev Med. 2007; 41: 123-26	Nonfatal Health Outcomes	Food-borne trematodiases
International Centre for Eye Health (ICEH). China - Wanzai Rapid Assessment of Avoidable Blindness 2007. Grootebroek, Netherlands: RAAB Repository	Nonfatal Health Outcomes	Refraction disorders
International Centre for Eye Health (ICEH). China - Xingan Rapid Assessment of Avoidable Blindness 2007. Grootebroek, Netherlands: RAAB Repository	Nonfatal Health Outcomes	Refraction disorders
Chan R, Wong VW, Chu WC, Wong GL, Li LS, Leung J, Chim AM, Yeung DK, Sea MM, Woo J, Chan FK, Chan HL. Diet-Quality Scores and Prevalence of Nonalcoholic Fatty Liver Disease: A Population Study Using Proton-Magnetic Resonance Spectroscopy. PLoS One. 2015; 10(9): e0139310	Nonfatal Health Outcomes	Cirrhosis due to NASH
Dong C, Dai X, Liang J, Dong M, Meng J. Seroprevalence of Hepatitis E virus varies considerably among Chinese provinces. Hepat Mon. 2012; 12(6): 386-90	Nonfatal Health Outcomes	Acute hepatitis E
International Centre for Eye Health (ICEH). China - Gao'an Rapid Assessment of Avoidable Blindness 2007. Grootebroek, Netherlands: RAAB Repository	Nonfatal Health Outcomes	Refraction and accommodation disorders
International Centre for Eye Health (ICEH). China - Xingan Rapid Assessment of Avoidable Blindness 2007. Grootebroek, Netherlands: RAAB Repository	Nonfatal Health Outcomes	Glaucoma
Song W, Sun X, Shao Z, Zhou X, Kang Y, Sui H, Yuan H. Prevalence and causes of visual impairment in a rural North-east China adult population: a population-based survey in Bin County, Harbin. Acta Ophthalmol. 2010; 88(6): 669-74	Nonfatal Health Outcomes	Vision loss
Zhang Y, Wang H, Liu J, Wang T, Cao S, Zhou D, Du L, Li Z, Liu P. Prevalence of blindness and low vision: a study in the rural Heilongjiang Province of China. Clin Experiment Ophthalmol. 2012; 40(5): 484-9	Nonfatal Health Outcomes	Vision loss
Li Z, Cui H, Liu P, Zhang L, Yang H, Zhang L. Prevalence and causes of blindness and visual impairment among the elderly in rural southern Harbin, China. Ophthalmic Epidemiol. 2008; 15(5): 334-8	Nonfatal Health Outcomes	Vision loss
Tao L, Huang F, Zhou Y, Li Q, Liang H, Li G, Wei H, Zhou L, Feng Q. Epidemiological survey of schizophrenia in urban and rural in Guilin, China. J Guangxi Med Univ. 2011; 6	Nonfatal Health Outcomes	Schizophrenia
Wei B, Chen Q, Feng Q. Prevalence of schizophrenia in residents of Guangxi Zhuang Autonomous Region. Chin J Public Health. 2011; 1245Â–7	Nonfatal Health Outcomes	Schizophrenia
Wei B. CQ Feng Q., Pan R., Chen Q., Huang G. et al. [Epidemiological survey on mental disorders in urban and rural in Guangxi Zhuang autonomous region]. J Guangxi Med Univ. 2010; 951Â–6	Nonfatal Health Outcomes	Alcohol use disorders
Feng Q-M, Luo H-Y, Wei B, Chen Q, Pang R-D, Chen Q-M, Huang G-G, Tang H-N, Chen F-Q, Li H-J. Epidemiological Survey of Alcohol Dependence in Rural Residents in Guangxi Zhaung Autonomous Region. Chin J Prev Control Chronic Dis. 2012; 20(5): 517-9	Nonfatal Health Outcomes	Alcohol use disorders
Wei B, Chen Q, Pan R-D, Feng Q-M, Chen Q-M, Huang G-G, Luo H-Y, Su L, Tang Z-H, Tang H-N, Chen N-S, Chen F-Q, Li H-J. Prevalence of major depressive disorder in urban and rural residents of Guangxi Zhuang Autonomous Region, China. Chin J Public Health. 2011; 27(4): 399-401	Nonfatal Health Outcomes	Major depressive disorder
Tao L-G, Huang F, Zhou Y, Li Q-B, Liang H-M, Li Guang L, Wei H-R, Zhou L-J, Feng Q-M. Prevalence of major depression disorder among urban and rural residents in Guilin municipality, China. Chin J Public Health. 2012; 28(10): 1268-1271	Nonfatal Health Outcomes	Major depressive disorder
Tao L-G, Huang F, Zhou Y, Li Q-B, Liang H-M, Li G-L, Wei H-R, Zhou L-J, Feng Q-M. Epidemiological survey of mental disorders in rural in Guilin. J Clin Psychiatry. 2011; 21(6): 381-383	Nonfatal Health Outcomes	Bipolar disorder
Ma Z-Y, Feng Q-M, Chen Q, Pan Y-D, Luo H-Y, Su L, Chen F-Q, Li H-J, Wei B. Prevalence of mood disorders among residents in Guangxi Zhuang Autonomous region. Chin J Public Health. 2012; 28(2): 1565-1568	Nonfatal Health Outcomes	Bipolar disorder

Tao L-G, Huang F, Zhou Y, Li Q-B, Liang H-M, Li G-L, Wei H-R, Zhou L-J, Feng Q-M. Epidemiological survey of mental disorders in rural in Guilin. J Clin Psychiatry. 2011; 21(6): 381-383	Nonfatal Health Outcomes	Anxiety disorders
International Centre for Eye Health (ICEH). China - Xingan Rapid Assessment of Avoidable Blindness 2007. Grootebroek, Netherlands: RAAB Repository	Nonfatal Health Outcomes	Age-related macular degeneration
International Centre for Eye Health (ICEH). China - Gao'an Rapid Assessment of Avoidable Blindness 2007. Grootebroek, Netherlands: RAAB Repository	Nonfatal Health Outcomes	
Chinese Center for Disease Control and Prevention (CCDC). China Notifiable Infectious Diseases 2008	Nonfatal Health Outcomes	Chlamydial infection
Ministry of Health (China), National Center for Chronic and Noncommunicable Disease Control and Prevention (China), World Health Organization (WHO). China WHO Study on Global AGEing and Adult Health 2007-2010	Nonfatal Health Outcomes	Ischemic heart disease
Ministry of Health (China), National Center for Chronic and Noncommunicable Disease Control and Prevention (China), World Health Organization (WHO). China WHO Study on Global AGEing and Adult Health 2007-2010	Nonfatal Health Outcomes	Asthma
Xu S, Ming J, Xing Y, Gao B, Yang C, Ji Q, Chen G. Regional differences in diabetes prevalence and awareness between coastal and interior provinces in China: a population-based cross-sectional study. BMC Public Health. 2013; 299	Nonfatal Health Outcomes	Diabetes mellitus
International Centre for Eye Health (ICEH). China - Wanzai Rapid Assessment of Avoidable Blindness 2007. Grootebroek, Netherlands: RAAB Repository	Nonfatal Health Outcomes	Other vision loss
Liu J, Huang Y, Wang J, Guo N, Li J, Dong X, Ma H, Tiemuer M, Huang M, Wright DJ, Ness, P, Shan, H. The increasing prevalence of serologic markers for syphilis among Chinese blood donors in 2008 through 2010 during a syphilis epidemic. Transfusion. 2012; 52(8): 1741-9	Nonfatal Health Outcomes	Syphilis
Yu S, Liu R, Zhao G, Yang X, Qiao X, Feng J, Fang Y, Cao X, He M, Steiner T. The prevalence and burden of primary headaches in China: a population-based door-to-door survey. Headache. 2012; 52(4): 582-91	Nonfatal Health Outcomes	Tension-type headache
LI Zong-hua, XU Zhan, CHEN Yang, et al. Hearing status among middle school students in Xi'an Â– an epidemiological survey. Chin J Otol. 2009; 7(1): 30-34	Nonfatal Health Outcomes	Hearing loss
He Y, Lin GY, Wang Q, Cai XY, Zhang YH, Lin CX, Lu CD, Lu XD. A 3-year prospective study of the epidemiology of acute respiratory viral infections in hospitalized children in Shenzhen, China. Influenza Other Respir Viruses. 2014; 8(4): 443-51	Nonfatal Health Outcomes	Lower respiratory infections
Liu WK, Liu Q, Chen de H, Liang HX, Chen XK, Chen MX, Qiu SY, Yang ZY, Zhou R. Epidemiology of acute respiratory infections in children in Guangzhou: a three-year study. PLoS One. 2014; 9(5): e96674	Nonfatal Health Outcomes	Lower respiratory infections
Guo RN, Zheng HZ, Huang LQ, Zhou Y, Zhang X, Liang CK, Lin JY, He JF, Zhang JQ. Epidemiologic and economic burden of influenza in the outpatient setting: a prospective study in a subtropical area of China. PLoS One. 2012; 7(7): e41403	Nonfatal Health Outcomes	Lower respiratory infections
LI Zong-hua, XU Zhan, CHEN Yang, et al. Hearing status among middle school students in Xi'an Â– an epidemiological survey. Chin J Otol. 2009; 7(1): 30-34	Nonfatal Health Outcomes	Age-related and other hearing loss
Hu J, Si Y, Zhou D, Mu J, Li J, Liu L, Zhu CR, Deng Y, He J, Zhang NM, Chen XF. Prevalence and treatment gap of active convulsive epilepsy: a large community-based survey in rural West China. Seizure. 2014; 23(5): 333-7	Nonfatal Health Outcomes	Epilepsy
Zhao J, Ellwein LB, Cui H, Ge J, Guan H, Lv J, Ma X, Yin J, Yin ZQ, Yuan Y, Liu H. Prevalence of vision impairment in older adults in rural China: the China Nine-Province Survey. Ophthalmology. 2010; 117(3): 409-16	Nonfatal Health Outcomes	Refraction disorders
Congdon N, Wang Y, Song Y, Choi K, Zhang M, Zhou Z, Xie Z, Li L, Liu X, Sharma A, Wu B, Lam DSC. Visual disability, visual function, and myopia among rural Chinese secondary school children: the Xichang Pediatric Refractive Error Study (X-PRES)Â–Report 1. Invest Ophthalmol Vis Sci. 2008; 49(7): 2888-94	Nonfatal Health Outcomes	Refraction disorders
International Centre for Eye Health (ICEH). China - Inner Mongolia Shangdu Rapid Assessment of Avoidable Blindness 2010	Nonfatal Health Outcomes	
International Centre for Eye Health (ICEH). China - Inner Mongolia Tuoketuo Rapid Assessment of Avoidable Blindness 2010	Nonfatal Health Outcomes	
Lu W-P, Lin G-X, Shi S, Dong J-H. Simultaneously high prevalences of hepatitis B and C virus infections in a population in Putian County, China. J Clin Microbiol. 2012; 50(6): 2142-4	Nonfatal Health Outcomes	Acute hepatitis C
Jiang L, Liang Y, Qiu B, Wang F, Duan X, Yang X, Huang W, Wang N. Prevalence of chronic kidney disease in a rural Chinese adult population: the Handan Eye Study. Nephron Clin Pract. 2010; 114(4): c295-302	Nonfatal Health Outcomes	Chronic kidney disease
Dai Q, Hu L, Jiang Y, Shi H, Liu J, Zhou W, Shen C, Yang H. An epidemiological survey of bacterial vaginosis, vulvovaginal candidiasis and trichomoniasis in the Tibetan area of Sichuan Province, China. Eur J Obstet Gynecol Reprod Biol. 2010; 150(2): 207-9	Nonfatal Health Outcomes	Trichomoniasis

Liang YB, Friedman DS, Wong TY, Zhan SY, Sun LP, Wang JJ, Duan XR, Yang XH, Wang FH, Zhou Q, Wang NL. Prevalence and causes of low vision and blindness in a rural chinese adult population: the Handan Eye Study. Ophthalmology. 2008; 115(11): 1965-72	Nonfatal Health Outcomes	Refraction and accommodation disorders
Liang YB, Friedman DS, Wong TY, Zhan SY, Sun LP, Wang JJ, Duan XR, Yang XH, Wang FH, Zhou Q, Wang NL. Prevalence and causes of low vision and blindness in a rural chinese adult population: the Handan Eye Study. Ophthalmology. 2008; 115(11): 1965-72	Nonfatal Health Outcomes	Refraction disorders
Wang H, De Steur H, Chen G, Zhang X, Pei L, Gellynck X, Zheng X. Effectiveness of Folic Acid Fortified Flour for Prevention of Neural Tube Defects in a High Risk Region. Nutrients. 2016; 8(3): 152	Nonfatal Health Outcomes	Neural tube defects
Wang H, De Steur H, Chen G, Zhang X, Pei L, Gellynck X, Zheng X. Effectiveness of Folic Acid Fortified Flour for Prevention of Neural Tube Defects in a High Risk Region. Nutrients. 2016; 8(3): 152	Nonfatal Health Outcomes	Digestive congenital anomalies
Ding X, Wang T, Shen Y, Wang X, Zhou C, Tian S, Liu Y, Peng G, Zhou J, Xue S, Wang R, Tang Y, Meng X, Pei G, Bai Y, Liu Q, Li H, Zhang J. Prevalence of psoriasis in China: a population-based study in six cities. Eur J Dermatol. 2012; 22(5): 663-7	Nonfatal Health Outcomes	Psoriasis
Congdon N, Wang Y, Song Y, Choi K, Zhang M, Zhou Z, Xie Z, Li L, Liu X, Sharma A, Wu B, Lam DSC. Visual disability, visual function, and myopia among rural Chinese secondary school children: the Xichang Pediatric Refractive Error Study (X-PRES)Â–Report 1. Invest Ophthalmol Vis Sci. 2008; 49(7): 2888-94	Nonfatal Health Outcomes	
Wang L, Huang W, He M, Zheng Y, Huang S, Liu B, Jin L, Congdon NG, He M. Causes and five-year incidence of blindness and visual impairment in urban Southern China: the Liwan Eye Study. Invest Ophthalmol Vis Sci. 2013; 54(6): 4117-21	Nonfatal Health Outcomes	
Zhao J, Ellwein LB, Cui H, Ge J, Guan H, Lv J, Ma X, Yin J, Yin ZQ, Yuan Y, Liu H. Prevalence of vision impairment in older adults in rural China: the China Nine-Province Survey. Ophthalmology. 2010; 117(3): 409-16	Nonfatal Health Outcomes	
Guo RN, Zheng HZ, Huang LQ, Zhou Y, Zhang X, Liang CK, Lin JY, He JF, Zhang JQ. Epidemiologic and economic burden of influenza in the outpatient setting: a prospective study in a subtropical area of China. PLoS One. 2012; 7(7): e41403	Nonfatal Health Outcomes	Influenza
Tu Z-W, Huang X-B, Cai S-X, Fan H-P, Wang K-Y, Liu X-G. [Surveillance of schistosomiasis japonica in potential endemic areas in Hubei Province from 2008 to 2012]. Chin J Schistosomiasis Control. 2014; 26(1): 56Â–8	Nonfatal Health Outcomes	Schistosomiasis
International Society of Nephrology (ISN). China - Beijing International Society of Nephrology Kidney Disease Data Center Chronic Kidney Disease and Cardiovascular Risk Survey 2006-2007	Nonfatal Health Outcomes	Chronic kidney disease
Shan Y, Zhang Q, Liu Z, Hu X, Liu D. Prevalence and risk factors associated with chronic kidney disease in adults over 40 years: a population study from Central China. Nephrology (Carlton). 2010; 15(3): 354-61	Nonfatal Health Outcomes	Chronic kidney disease
Liu DW, Wan J, Liu ZS, Wang P, Cheng GY, Shi XZ. Association between dyslipidemia and chronic kidney disease: a cross-sectional study in the middle-aged and elderly Chinese population. Chin Med J. 2013; 126(7): 1207-12	Nonfatal Health Outcomes	Chronic kidney disease
Jia J, Wang F, Wei C, Zhou A, Jia X, Li F, Tang M, Chu L, Zhou Y, Zhou C, Cui Y, Wang Q, Wang W, Yin P, Hu N, Zuo X, Song H, Qin W, Wu L, Li D, Jia L, Song J, Han Y, Xing Y, Yang P, Li Y, Qiao Y, Tang Y, Lv J, Dong X. The prevalence of dementia in urban and rural areas of China. Alzheimers Dement. 2014; 10(1): 1-9	Nonfatal Health Outcomes	Alzheimer's disease and other dementias
Wang H, De Steur H, Chen G, Zhang X, Pei L, Gellynck X, Zheng X. Effectiveness of Folic Acid Fortified Flour for Prevention of Neural Tube Defects in a High Risk Region. Nutrients. 2016; 8(3): 152	Nonfatal Health Outcomes	Congenital birth defects
Zhang B-C, Zhu Y, Deng B, Wen Q-S, Sun Y, Wang X-R. Survey of ADHD among primary and middle school students in urban area of Guiyang city. Matern Child Health Care China. 2011; 26: 4892-4894	Nonfatal Health Outcomes	Attention-deficit/hyperactivity disorder
Gao HL, Lin SQ, Wei Y, Chen Y, Wu ZL. The effect of age and menopausal status on musculoskeletal symptoms in Chinese women aged 35-64 years. Climacteric. 2013; 16(6): 639-45	Nonfatal Health Outcomes	Low back and neck pain
Peng J, Yuan Y, Shen F, Wang Y, Chen L, Liao DJ, Tan Y. Cervical Cancers Manifest a High Rate of Infection by a High-Risk Human Papilloma Virus Subtype but a Very Low Rate of Infection by a Low-Risk Subtype in the Guiyang District of China. J Cancer. 2017; 8(7): 1263-70	Nonfatal Health Outcomes	Viral skin diseases
Chinese Center for Disease Control and Prevention (CCDC). China Notifiable Infectious Diseases 2009	Nonfatal Health Outcomes	Chlamydial infection
Chen Z-S, Zhu W-F, Hu G-T, Qi B-Z, Zhu Q-K, Dong G. Epidemiology of psychiatric disease in residents in Qiaoxia community in Yongjia county, Zhejiang Province. Dis Surveillance. 2012; 27(2): 131-133	Nonfatal Health Outcomes	Schizophrenia
Chinese Center for Disease Control and Prevention (CCDC). China Notifiable Infectious Diseases 2007	Nonfatal Health Outcomes	Chlamydial infection

Chinese Center for Disease Control and Prevention (CCDC). China Notifiable Infectious Diseases 2007	Nonfatal Health Outcomes	Gonococcal infection
Fang Y. The Analysis of the Result of the Census of Mental Disorders in Fenghua City. Mod Pract Med. 2008; 20(10): 816-20	Nonfatal Health Outcomes	Schizophrenia
Wang D, Xiao W, Ma D, Zhang Y, Wang Q, Wang C, Ji X, He B, Wu X, Chen H, Zhang Y, Jiang Y, Yin J. Cross-sectional epidemiological survey of asthma in Jinan, China. Respirology. 2013; 18(2): 313-22	Nonfatal Health Outcomes	Asthma
Dong A-L, Zhang G, Wang S-S, Sun J-G, Zhang L-X, Zhang C-H, Gong Z-Y, Tang S-J, Bi J-H, Gu D-H, Zhang X-R, Niu N, Mu J-M, Cui S-K. Epidemiological survey of mental disorders in Weihai. J Clin Psychiatry. 2008; 18(4): 241-243	Nonfatal Health Outcomes	Schizophrenia
Dong A-L, Zhang G, Wang S-S, Sun J-G, Zhang L-X, Zhang C-H, Gong Z-Y, Tang S-J, Bi J-H, Gu D-H, Zhang X-R, Niu N, Mu J-M, Cui S-K. Epidemiological survey of mental disorders in Weihai. J Clin Psychiatry. 2008; 18(4): 241-243	Nonfatal Health Outcomes	Alcohol use disorders
Dong A-L, Zhang G, Wang S-S, Sun J-G, Zhang L-X, Zhang C-H, Gong Z-Y, Tang S-J, Bi J-H, Gu D-H, Zhang X-R, Niu N, Mu J-M, Cui S-K. Epidemiological survey of mental disorders in Weihai. J Clin Psychiatry. 2008; 18(4): 241-243	Nonfatal Health Outcomes	Bipolar disorder
Sun D-F, Yi J-M, Li M, Li Y-L. An investigation of ADHD and family environment in 8235 school children aged 4-16 years in Northern Shangdong. Chin J Nerv Ment Dis. 2009; 35(11): 650-654	Nonfatal Health Outcomes	Attention-deficit/hyperactivity disorder
Chinese Center for Disease Control and Prevention (CCDC). China Notifiable Infectious Diseases 2007	Nonfatal Health Outcomes	Syphilis
Chinese Center for Disease Control and Prevention (CCDC). China Notifiable Infectious Diseases 2009	Nonfatal Health Outcomes	Syphilis
Zhang L, Wang F, Wang L, Wang W, Liu B, Liu J, Chen M, He Q, Liao Y, Yu X, Chen N, Zhang J, Hu Z, Liu F, Hong D, Ma L, Liu H, Zhou X, Chen J, Pan L, Chen W, Wang W, Li X, Wang H. Prevalence of chronic kidney disease in China: a cross-sectional survey. Lancet. 2012; 379(9818): 815-22	Nonfatal Health Outcomes	Chronic kidney disease
Ministry of Health (China), National Center for Chronic and Noncommunicable Disease Control and Prevention (China), World Health Organization (WHO). China WHO Study on Global AGEing and Adult Health 2007-2010	Nonfatal Health Outcomes	Near vision loss
Li Y, Yin Z, Shao Z, Li M, Liang X, Sandhu HS, Hadler SC, Li J, Sun Y, Li J, Zou W, Lin M, Zuo S, Mayer LW, Novak RT, Zhu B, Xu L, Luo H, Acute Meningitis and Encephalitis Syndrome Study Group. Population-based surveillance for bacterial meningitis in China, September 2006-December 2009. Emerg Infect Dis. 2014; 20(1): 61-9	Nonfatal Health Outcomes	Hearing loss
Li Y, Yin Z, Shao Z, Li M, Liang X, Sandhu HS, Hadler SC, Li J, Sun Y, Li J, Zou W, Lin M, Zuo S, Mayer LW, Novak RT, Zhu B, Xu L, Luo H, Acute Meningitis and Encephalitis Syndrome Study Group. Population-based surveillance for bacterial meningitis in China, September 2006-December 2009. Emerg Infect Dis. 2014; 20(1): 61-9	Nonfatal Health Outcomes	Vision loss
Ming J, Xu S, Yang C, Gao B, Wan Y, Xing Y, Zhang L, Yang W, Ji Q, China National Diabetes and Metabolic Disorders Study Group. Metabolic syndrome and chronic kidney disease in general Chinese adults: results from the 2007-08 China National Diabetes and Metabolic Disorders Study. Clin Chim Acta. 2014; 430: 115-20	Nonfatal Health Outcomes	Chronic kidney disease
GUO Chaobin, GAO Xian, PANG Zengchang. Analyses on hearing loss and its related factors in middle and old aged persons in Jiaonan of QingDao. Med J Qilu. 2012; 27(5): 426-29	Nonfatal Health Outcomes	Age-related and other hearing loss
Ji W, Zhang T, Zhang X, Jiang L, Ding Y, Hao C, Ju L, Wang Y, Jiang Q, Steinhoff M, Black S, Zhao G. The epidemiology of hospitalized influenza in children, a two year population-based study in the PeopleÂ’s Republic of China. BMC Health Serv Res. 2010; 10: 82	Nonfatal Health Outcomes	Lower respiratory infections
Lin B, Shao L, Luo Q, Ou-yang L, Zhou F, Du B, He Q, Wu J, Xu N, Chen J. Prevalence of chronic kidney disease and its association with metabolic diseases: a cross-sectional survey in Zhejiang province, Eastern China. BMC Nephrol. 2014; 36	Nonfatal Health Outcomes	Chronic kidney disease
GUO Chaobin, GAO Xian, PANG Zengchang. Analyses on hearing loss and its related factors in middle and old aged persons in Jiaonan of QingDao. Med J Qilu. 2012; 27(5): 426-29	Nonfatal Health Outcomes	Hearing loss
Lu N-W, Zhou G-Y. Status analysis of patients with schizophrenia in Nantong economic development zone. Med J Chin Peoples Health. 2014; 26(24): 92-93+96	Nonfatal Health Outcomes	Schizophrenia
Chen Y, Ma F, Zhang J, Chu X, Xu Y. Population incidence of Guillain-Barre syndrome in parts of China: three large populations in Jiangsu province, 2008-2010. Eur J Neurol. 2014; 21(1): 124-9	Nonfatal Health Outcomes	Guillain-BarrÃ© syndrome
Fang X, Chen Y, Chen C, Xiong D, Ji J, Shi G, Zheng J, Chen B, Chen T, Weng S. Epidemiological survey of mental disorder in Fujian province. Chin J Psychiatry. 2011; 44(2): 103Â–7	Nonfatal Health Outcomes	Schizophrenia
Chinese Center for Disease Control and Prevention (CCDC). China Notifiable Infectious Diseases 2008	Nonfatal Health Outcomes	Syphilis

Liu J, Huang Y, Wang J, Guo N, Li J, Dong X, Ma H, Tiemuer M, Huang M, Wright DJ, Ness, P, Shan, H. The increasing prevalence of serologic markers for syphilis among Chinese blood donors in 2008 through 2010 during a syphilis epidemic. Transfusion. 2012; 52(8): 1741-9	Nonfatal Health Outcomes	Sexually transmitted infections excluding HIV
Pu Y, Zhang J, Luo Y, Xie X, Wu P, Zhang Q, Yang QY. Prevalence of autism among young children aged 2-6 years in Yunyan district of Guiyang city. Chin J Public Health. 2014; 30(1): 89Â–91	Nonfatal Health Outcomes	Autism spectrum disorders
Ministry of Health (China), National Center for Chronic and Noncommunicable Disease Control and Prevention (China), World Health Organization (WHO). China WHO Study on Global AGEing and Adult Health 2007-2010	Nonfatal Health Outcomes	
Chinese Center for Disease Control and Prevention (CCDC). China Notifiable Infectious Diseases 2009	Nonfatal Health Outcomes	Gonococcal infection
Li EY, Liu Y, Zhan X, Liang YB, Zhang X, Zheng C, Jhanji V, Xu P, Chang DF, Lam DS. Prevalence of blindness and outcomes of cataract surgery in Hainan Province in South China. Ophthalmology. 2013; 120(11.0): 2176-83	Nonfatal Health Outcomes	Vision loss
Zhu Y, Yuan Z, Zhao Q, Chen G, Xu B. Seroprevalence of hepatitis A virus antibody in a population aged 0-30 years in Shanghai, China: implications for hepatitis A immunization. Epidemiol Infect. 2013; 141(3): 556-62	Nonfatal Health Outcomes	Acute hepatitis A
Zhong B, Ding J, Chen H, Li Y, Xu H, Tong J, Wang A, Tang G, Zhu J, Yang D, Liu B, Wang Q, Cheng W, Yin E, Xu M, Zhang T, Hu T, Feng X, Li H, Dan T, Cheng G, Zhang J, Li H, Zhu J. Depressive disorders among children in the transforming China: an epidemiological survey of prevalence, correlates, and service use. Depress Anxiety. 2013; 30(9): 881-92	Nonfatal Health Outcomes	Major depressive disorder
Zhou Y, Echouffo-Tcheugui JB, Gu JJ, Ruan XN, Zhao GM, Xu WH, Yang LM, Zhang H, Qiu H, Narayan KM, Sun Q. Prevalence of chronic kidney disease across levels of glycemia among adults in Pudong New Area, Shanghai, China. BMC Nephrol. 2013; 14: 253	Nonfatal Health Outcomes	Chronic kidney disease
Zhang F, Hang J, Zheng B, Su L, Christiani DC. The changing epidemiology of asthma in Shanghai, China. J Asthma. 2015; 52(5): 465-70	Nonfatal Health Outcomes	Asthma
Jin W, Du Y, Zhong X, David C. Prevalence and contributing factors to attention deficit hyperactivity disorder: a study of five- to fifteen-year-old children in Zhabei District, Shanghai. Asia Pac Psychiatr. 2014; 6(4): 397Â–404	Nonfatal Health Outcomes	Attention-deficit/hyperactivity disorder
Sheng C-S, Liu M, Kang Y-Y, Wei F-F, Zhang L, Li G-L, Dong Q, Huang Q-F, Li Y, Wang J-G. Prevalence, awareness, treatment and control of hypertension in elderly Chinese. Hypertens Res. 2013; 36(9): 824Â–8	Nonfatal Health Outcomes	Diabetes mellitus
Qin Y, Wang R, Ma X, Zhao Y, Lu J, Wu C, He J. Prevalence, Awareness, Treatment and Control of Diabetes Mellitus-A Population Based Study in Shanghai, China. Int J Environ Res Public Health. 2016; 13(5)	Nonfatal Health Outcomes	Diabetes mellitus
Liang YB, Friedman DS, Wong TY, Zhan SY, Sun LP, Wang JJ, Duan XR, Yang XH, Wang FH, Zhou Q, Wang NL. Prevalence and causes of low vision and blindness in a rural chinese adult population: the Handan Eye Study. Ophthalmology. 2008; 115(11): 1965-72	Nonfatal Health Outcomes	
Li X, Zhou Q, Sun L, Wang Z, Han S, Wu S, Wang N. Prevalence of blindness and low vision in a rural population in northern China: preliminary results from a population-based survey. Ophthalmic Epidemiol. 2012; 19(5): 272-7	Nonfatal Health Outcomes	
Du Q, Yu M, Li Y, Du H, Gao W, Mei H, Liu S. Permanent caries experience is associated with primary caries experience: a 7-year longitudinal study in China. Community Dent Oral Epidemiol. 2016	Nonfatal Health Outcomes	Caries of deciduous teeth
Zhu M, Tong X, Zhao R, He X, Zhao H, Liu M, Zhu J. Visual impairment and spectacle coverage rate in Baoshan district, China: population-based study. BMC Public Health. 2013; 13(1): 311	Nonfatal Health Outcomes	Vision loss
Qiao C-X. Health-related quality of life evaluated by tumor node metastasis staging system in patients with hepatocellular carcinoma . World J Gastroenterol. 2012; 18(21): 2689	Nonfatal Health Outcomes	Liver cancer due to hepatitis B
Qiao C-X. Health-related quality of life evaluated by tumor node metastasis staging system in patients with hepatocellular carcinoma . World J Gastroenterol. 2012; 18(21): 2689	Nonfatal Health Outcomes	Liver cancer due to hepatitis C
Qiao C-X. Health-related quality of life evaluated by tumor node metastasis staging system in patients with hepatocellular carcinoma . World J Gastroenterol. 2012; 18(21): 2689	Nonfatal Health Outcomes	Liver cancer due to alcohol use
Zhu M, Tong X, Zhao R, He X, Zhao H, Liu M, Zhu J. Visual impairment and spectacle coverage rate in Baoshan district, China: population-based study. BMC Public Health. 2013; 13(1): 311	Nonfatal Health Outcomes	

Leung TF, Lam DS, Miu TY, Hon KL, Chau CS, Ku SW, Lee RS, Chow PY, Chiu WK, Ng DK. Epidemiology and risk factors for severe respiratory syncytial virus infections requiring pediatric intensive care admission in Hong Kong children. Infection. 2014; 42(2): 343-50	Nonfatal Health Outcomes	Lower respiratory infections
Li Z, Zou D, Ma X, Chen J, Shi X, Gong Y, Man X, Gao L, Zhao Y, Wang R, Yan X, Dent J, Sung JJ, Wernersson B, Johansson S, Liu W, He J. Epidemiology of peptic ulcer disease: endoscopic results of the systematic investigation of gastrointestinal disease in China. Am J Gastroenterol. 2010; 105(12): 2570-7	Nonfatal Health Outcomes	Peptic ulcer disease
Zou D, He J, Ma X, Liu W, Chen J, Shi X, Ye P, Gong Y, Zhao Y, Wang R, Yan X, Man X, Gao L, Dent J, Sung J, Wernersson B, Johansson S, Li Z. Helicobacter pylori infection and gastritis: the Systematic Investigation of gastrointestinaL diseases in China (SILC). J Gastroenterol Hepatol. 2011; 26(5): 908-15	Nonfatal Health Outcomes	Gastritis and duodenitis
Liang YB, Friedman DS, Wong TY, Zhan SY, Sun LP, Wang JJ, Duan XR, Yang XH, Wang FH, Zhou Q, Wang NL. Prevalence and causes of low vision and blindness in a rural chinese adult population: the Handan Eye Study. Ophthalmology. 2008; 115(11): 1965-72	Nonfatal Health Outcomes	Vision loss
Li X, Zhou Q, Sun L, Wang Z, Han S, Wu S, Wang N. Prevalence of blindness and low vision in a rural population in northern China: preliminary results from a population-based survey. Ophthalmic Epidemiol. 2012; 19(5): 272-7	Nonfatal Health Outcomes	Vision loss
Zhang X, Wan Y, Zhang S, Lu L, Chen Z, Liu H, Jiang X, Luo K, Cai W. Nonalcoholic fatty liver disease prevalence in urban school-aged children and adolescents from the Yangtze River delta region: a cross-sectional study. Asia Pac J Clin Nutr. 2015; 24(2): 281-8	Nonfatal Health Outcomes	Cirrhosis due to NASH
Liu J. Early screening and a clinical records study of autism spectrum disorder [master's thesis]. Shanghai, China: Fudan University Shanghai Medical College, 2011	Nonfatal Health Outcomes	Autism spectrum disorders
Bao Z, Yuan X, Wang L, Sun Y, Dong X. The incidence and etiology of community-acquired pneumonia in fever outpatients. Exp Biol Med (Maywood). 2012; 237(11): 1256-61	Nonfatal Health Outcomes	Respiratory syncytial virus pneumonia
Qiao C-X. Health-related quality of life evaluated by tumor node metastasis staging system in patients with hepatocellular carcinoma . World J Gastroenterol. 2012; 18(21): 2689	Nonfatal Health Outcomes	Liver cancer due to NASH
Feng ML, Li SG, Wu ZY, Yin CH, Zhang XJ, Zhai JG, Chen J, Han SF, Zhang XX, Shang YL, Yan XC. [An epidemiological survey on paragonimiasis in Jin Miaopu township in Shanxi province]. Chin J Prev Med. 2007; 41(Suppl): 131-33	Nonfatal Health Outcomes	Food-borne trematodiases
Huang X, Chen M, Tan H, Xiao S, Deng J. The morbidity rate of chronic disease among Chinese rural residents: results from Liuyang cohort. Med Princ Pract. 2013; 22(4): 362-7	Nonfatal Health Outcomes	Urolithiasis
Ma L, Liu C, Zheng X, Xin S, Ji Z, Li Y, Yan G, Tian C, Tang L, Sun B. A 12-month prospective survey of perinatal outcome of liveborn neonates in Julu County, China. Chin Med J (Engl). 2010; 123(20): 2781-5	Nonfatal Health Outcomes	Neonatal preterm birth
Qing-Hua Fan. Study and Analysis of Impact Factors and Health Service of Dementia in Shanxi Province [dissertation]. Shanxi, China: Shanxi Medical University; 2012. 115 p	Nonfatal Health Outcomes	Alzheimer's disease and other dementias
Li EY, Liu Y, Zhan X, Liang YB, Zhang X, Zheng C, Jhanji V, Xu P, Chang DF, Lam DS. Prevalence of blindness and outcomes of cataract surgery in Hainan Province in South China. Ophthalmology. 2013; 120(11.0): 2176-83	Nonfatal Health Outcomes	
Tian R-G, Lu J, Zhang B-C, Jiang Y-Z, Bi S-L. [Antibody detection of hepatitis E virus in some human population, swine and chicken in Beijing in China]. Chin J Exper Clin Virol. 2009; 23(1): 14-6	Nonfatal Health Outcomes	Acute hepatitis E
Liu F, Ma Y-T, Yang Y-N, Zhen Y-J, Xie X, Li X-M, Ma X, Chen B-D, Huang Y, Shan C-F, Gao X-M. The prevalence of isolated systolic hypertension in adult populations from the Han, Uygur and Kazakh ethnic groups in Xinjiang, China. Blood Press. 2014; 23(3): 154Â–9	Nonfatal Health Outcomes	Diabetes mellitus
Yu C, Xia W, Sun A, Zhou X, Liu L, Li J, Zhou X, Wu L. Survey on autistic spectrum disorders in 2 to 6 years old children in Harbin city. Chin J Child Health Care. 2010; 18: 750-753	Nonfatal Health Outcomes	Autism spectrum disorders
Pi X, Cui L, Liu A, Zhang J, Ma Y, Liu B, Cai C, Zhu C, Zhou T, Chen J, Zhou Z, Wang C, Li L, Li S, Wu J, Xiao B. Investigation of prevalence, clinical characteristics and management of epilepsy in Yueyang city of China by a door-to-door survey. Epilepsy Res. 2012; 101(1-2): 129-34	Nonfatal Health Outcomes	Epilepsy
Li Z, Cui H, Liu P, Zhang L, Yang H, Zhang L. Prevalence and causes of blindness and visual impairment among the elderly in rural southern Harbin, China. Ophthalmic Epidemiol. 2008; 15(5): 334-8	Nonfatal Health Outcomes	Glaucoma

Zhang Y, Wang H, Liu J, Wang T, Cao S, Zhou D, Du L, Li Z, Liu P. Prevalence of blindness and low vision: a study in the rural Heilongjiang Province of China. Clin Experiment Ophthalmol. 2012; 40(5): 484-9	Nonfatal Health Outcomes	Glaucoma
Li Z, Xu K, Wu S, Lv J, Jin D, Song Z, Wang Z, Liu P. Population-based survey of refractive error among school-aged children in rural northern China: the Heilongjiang eye study. Clin Experiment Ophthalmol. 2014; 42(4.0): 379-84	Nonfatal Health Outcomes	Refraction and accommodation disorders
Zhang Y, Wang H, Liu J, Wang T, Cao S, Zhou D, Du L, Li Z, Liu P. Prevalence of blindness and low vision: a study in the rural Heilongjiang Province of China. Clin Experiment Ophthalmol. 2012; 40(5): 484-9	Nonfatal Health Outcomes	Other vision loss
Tan CE, Li HJ, Zhang XG, Zhang H, Han PY, An Q, Ding WJ, Wang MQ. The impact of the Wenchuan earthquake on birth outcomes. PLoS One. 2009; 4(12): e8200	Nonfatal Health Outcomes	Neonatal preterm birth
Chen B-J, Li L-S, Lin M-X, Xu M-C, Chen Z-L, Fang Y-E, Yang H. [Surveillance of schistosomiasis in national surveillance sites of Fuqing City and Xiapu County, 2006 - 2009]. Chin J Schistosomiasis Control. 2011; 23(1): 89Â–92	Nonfatal Health Outcomes	Schistosomiasis
Li Z, Xu K, Wu S, Lv J, Jin D, Song Z, Wang Z, Liu P. Population-based survey of refractive error among school-aged children in rural northern China: the Heilongjiang eye study. Clin Experiment Ophthalmol. 2014; 42(4.0): 379-84	Nonfatal Health Outcomes	Vision loss
Dai Q, Hu L, Jiang Y, Shi H, Liu J, Zhou W, Shen C, Yang H. An epidemiological survey of bacterial vaginosis, vulvovaginal candidiasis and trichomoniasis in the Tibetan area of Sichuan Province, China. Eur J Obstet Gynecol Reprod Biol. 2010; 150(2): 207-9	Nonfatal Health Outcomes	Sexually transmitted infections excluding HIV
Liu D-M, Yang Y-H, Wang Z-Q, Xiang D-H, Wang S-Y, Yang C-M, Li H-M, Lin J. Epidemiological survey of severe depression among adults in Yibin city. Chin Prev Med. 2012; 13(7): 507-510	Nonfatal Health Outcomes	Major depressive disorder
Liu Z, Liu L. Hearing screening and diagnosis in a large sample of infants in Central China. J Med Screen. 2013; 20(1): 21-6	Nonfatal Health Outcomes	Age-related and other hearing loss
Song W, Sun X, Shao Z, Zhou X, Kang Y, Sui H, Yuan H. Prevalence and causes of visual impairment in a rural North-east China adult population: a population-based survey in Bin County, Harbin. Acta Ophthalmol. 2010; 88(6): 669-74	Nonfatal Health Outcomes	
Zhao Y, Yao Z, D'Souza W, Zhu C, Chun H, Zhuoga C, Zhang Q, Hu X, Zhou D. An Epidemiological Survey of Stroke in Lhasa, Tibet, China. Stroke. 2010; 41(12): 2739-43	Nonfatal Health Outcomes	Ischemic stroke
Zhang Y, Wang H, Liu J, Wang T, Cao S, Zhou D, Du L, Li Z, Liu P. Prevalence of blindness and low vision: a study in the rural Heilongjiang Province of China. Clin Experiment Ophthalmol. 2012; 40(5): 484-9	Nonfatal Health Outcomes	
Zhang L, Zhang W-H, Zhang L, Wang P-Y. Prevalence of overweight/obesity and its associations with hypertension, diabetes, dyslipidemia, and metabolic syndrome: a survey in the suburban area of Beijing, 2007. Obes Facts. 2011; 4(4): 284-9	Nonfatal Health Outcomes	Diabetes mellitus
Zhang L, Qin L-Q, Liu A-P, Wang P-Y. Prevalence of risk factors for cardiovascular disease and their associations with diet and physical activity in suburban Beijing, China. J Epidemiol. 2010; 20(3): 237Â–43	Nonfatal Health Outcomes	Diabetes mellitus
Zhao Y, Yao Z, D'Souza W, Zhu C, Chun H, Zhuoga C, Zhang Q, Hu X, Zhou D. An Epidemiological Survey of Stroke in Lhasa, Tibet, China. Stroke. 2010; 41(12): 2739-43	Nonfatal Health Outcomes	Subarachnoid hemorrhage
Chen Q-L, Wang J, Liu Q-G, Li Y, Xie S-F, Huang Q-Y, Xu J-T. Prevalence of alcohol use disorders and its influence factors among residents in Dalian city. Chin J Public Health. 2012; 28(5): 614-615	Nonfatal Health Outcomes	Alcohol use disorders
Zhou X, Ji L, Luo Y, Han X, Zhang X, Sun X, Ren Q, Qiao Q. Risk factors associated with the presence of diabetes in Chinese communities in Beijing. Diabetes Res Clin Pract. 2009; 86(3): 233Â–8	Nonfatal Health Outcomes	Diabetes mellitus
Yang H, Wang L, Shi YH, Sui GT, Wu YF, Lu XQ, Li MY, Xia Q, Bian XX, Li HH, Qian JM. Risk factors of acute pancreatitis in the elderly Chinese population: a population-based cross-sectional study. J Dig Dis. 2014; 15(9): 501-7	Nonfatal Health Outcomes	Pancreatitis
Chen Y, Huang H, Zhao Y, Li D, Li J, Rao L, Zhu M, Ren X. Epidemiological survey on autism spectrum disorders in 2 to 6 years old children in Ranghulu district in Daqing city. China J Child Health Care. 2010; 18(4): 331Â–333	Nonfatal Health Outcomes	Autism spectrum disorders
Li R, Sun J, Ren L-M, Wang H-Y, Liu W-H, Zhang X-W, Chen S, Mu R, He J, Zhao Y, Long L, Liu Y-Y, Liu X, Lu X-L, Li Y-H, Wang S-Y, Pan S-S, Li C, Wang H-Y, Li Z-G. Epidemiology of eight common rheumatic diseases in China: a large-scale cross-sectional survey in Beijing. Rheumatology (Oxford). 2012; 51(4): 721-9	Nonfatal Health Outcomes	Rheumatoid arthritis
Gao HL, Lin SQ, Wei Y, Chen Y, Wu ZL. The effect of age and menopausal status on musculoskeletal symptoms in Chinese women aged 35-64 years. Climacteric. 2013; 16(6): 639-45	Nonfatal Health Outcomes	Low back pain
Lu X, Li X, Zhao Y, Zheng Z, Guan S, Chan P. Contemporary epidemiology of gout and hyperuricemia in community elderly in Beijing. Int J Rheum Dis. 2013	Nonfatal Health Outcomes	Gout

Zhou L, Conner KR, Phillips MR, Caine ED, Xiao S, Zhang R, Gong Y. Epidemiology of alcohol abuse and dependence in rural Chinese men. Alcohol Clin Exp Res. 2009; 33(10): 1770-6	Nonfatal Health Outcomes	Alcohol use disorders
Shan Y, Zhang Q, Liu Z, Hu X, Liu D. Prevalence and risk factors associated with chronic kidney disease in adults over 40 years: a population study from Central China. Nephrology (Carlton). 2010; 15(3): 354-61	Nonfatal Health Outcomes	Diabetes mellitus
Hong Kong Cancer Registry. Hong Kong Cancer Registry Annual Tables. Kowloon, Hong Kong: Hong Kong Cancer Registry	Nonfatal Health Outcomes	Ovarian cancer
Yu Q, Wan X-X, Liu Q, Cao C-L, Bao Z-P, Zhu H-Q, Zhong B, Guo J-G. [Cost-effectiveness evaluation and investigation of control measure changes in areas of schistosomiasis transmission control in hilly regions of mountain areas I epidemiological investigation and analysis of prevalence factors of schistosomiasis]. Chin J Schistosomiasis Control. 2012; 24(3): 250Â–4, 365	Nonfatal Health Outcomes	Schistosomiasis
Hong Z, Yanfang J, Shumei H, Jie S, Qing G, Xiangwei F, Bing D, Wei W, Xiaodong S, Chunyan W, Siqi Z, Wanyu L, Junyan F, Junqi N. Relationship between serum aminotransferase levels and metabolic disorders in northern China. Turk J Gastroenterol. 2012; 23(6): 699Â–707	Nonfatal Health Outcomes	Cirrhosis due to NASH
Song W, Sun X, Shao Z, Zhou X, Kang Y, Sui H, Yuan H. Prevalence and causes of visual impairment in a rural North-east China adult population: a population-based survey in Bin County, Harbin. Acta Ophthalmol. 2010; 88(6): 669-74	Nonfatal Health Outcomes	Refraction disorders
Li Z, Xu K, Wu S, Lv J, Jin D, Song Z, Wang Z, Liu P. Population-based survey of refractive error among school-aged children in rural northern China: the Heilongjiang eye study. Clin Experiment Ophthalmol. 2014; 42(4.0): 379-84	Nonfatal Health Outcomes	Refraction disorders
Xu L, Jonas JB, Cui TT, You QS, Wang YX, Yang H, Li JJ, Wei WB, Liang QF, Wang S, Yang XH, Zhang L, Beijing Institute of Ophthalmology, Beijing Tongren Hospital, Capital Medical University. Beijing Eye Public Health Care Project. Ophthalmology. 2012; 119(6): 1167-74	Nonfatal Health Outcomes	
Huang X, Chen M, Tan H, Xiao S, Deng J. The morbidity rate of chronic disease among Chinese rural residents: results from Liuyang cohort. Med Princ Pract. 2013; 22(4): 362-7	Nonfatal Health Outcomes	Gastritis and duodenitis
Chinese Center for Disease Control and Prevention (CCDC). China Notifiable Infectious Diseases 2009	Nonfatal Health Outcomes	Sexually transmitted infections excluding HIV
DananchÃ© C, SÃ¡nchez Picot V, BÃ©net T, Messaoudi M, Chou M, Wang J, Pape J-W, Awasthi S, Bavdekar A, Rakoto-Andrianarivelo M, Sylla M, Nymadawa P, Russomando G, Komurian-Pradel F, Endtz H, Paranhos-BaccalÃ G, Vanhems P, For The Gabriel Network null. Burden of Influenza in Less Than 5-Year-Old Children Admitted to Hospital with Pneumonia in Developing and Emerging Countries: A Descriptive, Multicenter Study. Am J Trop Med Hyg. 2018; 98(6): 1805Â–10	Nonfatal Health Outcomes	Influenza
Feng L, Lai S, Li F, Ye X, Li S, Ren X, Zhang H, Li Z, Yu H, Yang W. [Viral etiologies of hospitalized pneumonia patients aged less than five years in six provinces, 2009-2012]. Chin J Epidemiol. 2014; 35(6): 646Â–9	Nonfatal Health Outcomes	Respiratory syncytial virus pneumonia
Feng L, Li Z, Zhao S, Nair H, Lai S, Xu W, Li M, Wu J, Ren L, Liu W, Yuan Z, Chen Y, Wang X, Zhao Z, Zhang H, Li F, Ye X, Li S, Feikin D, Yu H, Yang W. Viral etiologies of hospitalized acute lower respiratory infection patients in China, 2009-2013. PLoS One. 2014; 9(6): e99419	Nonfatal Health Outcomes	Respiratory syncytial virus pneumonia
Jin Y. Guangxi monitoring network of acute encephalitis, meningitis epidemiological surveillance studies. Dis Surveillance. 2010; 25(3)	Nonfatal Health Outcomes	Meningitis
Li Z, Xu K, Wu S, Lv J, Jin D, Song Z, Wang Z, Liu P. Population-based survey of refractive error among school-aged children in rural northern China: the Heilongjiang eye study. Clin Experiment Ophthalmol. 2014; 42(4.0): 379-84	Nonfatal Health Outcomes	
Li Z, Cui H, Liu P, Zhang L, Yang H, Zhang L. Prevalence and causes of blindness and visual impairment among the elderly in rural southern Harbin, China. Ophthalmic Epidemiol. 2008; 15(5): 334-8	Nonfatal Health Outcomes	
International Centre for Eye Health (ICEH). China - Wanzai Rapid Assessment of Avoidable Blindness 2007. Grootebroek, Netherlands: RAAB Repository	Nonfatal Health Outcomes	
Chinese Center for Disease Control and Prevention (CCDC). China Notifiable Infectious Diseases 2007	Nonfatal Health Outcomes	Sexually transmitted infections excluding HIV
Peng J, Kong W, Guo D, Liu M, Wang Y, Zhu H, Pang B, Miao X, Yu B, Luo T, Hu Q, Zhou D. The epidemiology and etiology of influenza-like illness in Chinese children from 2008 to 2010. J Med Virol. 2012; 84(4): 672-8	Nonfatal Health Outcomes	Influenza
Peng J, Kong W, Guo D, Liu M, Wang Y, Zhu H, Pang B, Miao X, Yu B, Luo T, Hu Q, Zhou D. The epidemiology and etiology of influenza-like illness in Chinese children from 2008 to 2010. J Med Virol. 2012; 84(4): 672-8	Nonfatal Health Outcomes	Respiratory syncytial virus pneumonia

Zhou H, Jia Y, Shen J, Wang S, Li X, Yang R, Huang K, Hu T, Tang F, Zhou J, Yuan J, Huang L, Tian X, Chen Z, Zhang Q, Wang C, Xi L, Deng D, Wang H, Ma D, Li S. Gynecologic infections seen in ThinPrep cytological test in Wuhan, China. Front Med. 2014; 8(2): 236Â–40	Nonfatal Health Outcomes	Trichomoniasis
Chinese Center for Disease Control and Prevention (CCDC). China Notifiable Infectious Diseases 2008	Nonfatal Health Outcomes	Sexually transmitted infections excluding HIV
Zhong B, Ding J, Chen H, Li Y, Xu H, Tong J, Wang A, Tang G, Zhu J, Yang D, Liu B, Wang Q, Cheng W, Yin E, Xu M, Zhang T, Hu T, Feng X, Li H, Dan T, Cheng G, Zhang J, Li H, Zhu J. Depressive disorders among children in the transforming China: an epidemiological survey of prevalence, correlates, and service use. Depress Anxiety. 2013; 30(9): 881-92	Nonfatal Health Outcomes	Dysthymia
Chen W, Liu Q, Wang H, Chen W, Johnson RJ, Dong X, Li H, Ba S, Tan J, Luo N, Liu T, He H, Yu X. Prevalence and risk factors of chronic kidney disease: a population study in the Tibetan population. Nephrol Dial Transplant. 2011; 26(5): 1592-9	Nonfatal Health Outcomes	Chronic kidney disease
Hou X, Lu J, Weng J, Ji L, Shan Z, Liu J, Tian H, Ji Q, Zhu D, Ge J, Lin L, Chen L, Guo X, Zhao Z, Li Q, Zhou Z, Shan G, Yang Z, Yang W, Jia W, China National Diabetes and Metabolic Disorders Study Group. Impact of waist circumference and body mass index on risk of cardiometabolic disorder and cardiovascular disease in Chinese adults: a national diabetes and metabolic disorders survey. PLoS One. 2013; 8(3): e57319	Nonfatal Health Outcomes	Diabetes mellitus
Chen G, Yi X, Chen P, Dong J, Yang G, Fu S. A large-scale newborn hearing screening in rural areas in China. Int J Pediatr Otorhinolaryngol. 2012; 76(12): 1771-4	Nonfatal Health Outcomes	Hearing loss
Liu Z, Liu L. Hearing screening and diagnosis in a large sample of infants in Central China. J Med Screen. 2013; 20(1): 21-6	Nonfatal Health Outcomes	Hearing loss
Shen Y, Wang T, Zhou C, Wang X, Ding X, Tian S, Liu Y, Peng G, Xue S, Zhou J, Wang R, Meng X, Pei G, Bai Y, Liu Q, Li H, Zhang J. Prevalence of acne vulgaris in Chinese adolescents and adults: a community-based study of 17,345 subjects in six cities. Acta Derm Venereol. 2012; 92(1): 40-4	Nonfatal Health Outcomes	Acne vulgaris
HUANG JP, CUI SS, HAN Y, IRVA H-P, QI LH, ZHANG X. Prevalence and Early Signs of Autism Spectrum Disorder (ASD) among 18Â–36 Month Old Children in Tianjin of China. Biomed Environ Sci. 2014; 27(6): 453Â–61	Nonfatal Health Outcomes	Autism spectrum disorders
Su Y.Y., Zhang X., Li A.Y., Li Y.M., Liu G.M. Prevalence and risk factors of infantile autism in Tianjin. Matern Child Health Care China. 2011; 26: 5004-5007	Nonfatal Health Outcomes	Autism spectrum disorders
Wang Y-J, Pan K-L, Zhao X-L, Qiang H, Cheng S-Q. [Therapeutic effects of erythropoietin on hypoxic-ischemic encephalopathy in neonates]. Chin J Contemp Pediatr. 2011; 13(11): 855-8	Nonfatal Health Outcomes	Neonatal encephalopathy due to birth asphyxia and trauma
Kailembo A, Preet R, Stewart Williams J. Common risk factors and edentulism in adults, aged 50 years and over, in China, Ghana, India and South Africa: results from the WHO Study on global AGEing and adult health (SAGE). BMC Oral Health. 2016; 17(1): 29	Nonfatal Health Outcomes	Edentulism and severe tooth loss
Ministry of Health (China), National Center for Chronic and Noncommunicable Disease Control and Prevention (China), World Health Organization (WHO). China WHO Study on Global AGEing and Adult Health 2007-2010	Nonfatal Health Outcomes	Road injuries
Zeng Q, Gong Y, Dong S, Xiang H, Wu Q. Association between exposure to hepatitis B virus and chronic kidney disease in China. J Int Med Res. 2014; 42(5): 1178-84	Nonfatal Health Outcomes	Chronic kidney disease
Ministry of Health (China), National Center for Chronic and Noncommunicable Disease Control and Prevention (China), World Health Organization (WHO). China WHO Study on Global AGEing and Adult Health 2007-2010	Nonfatal Health Outcomes	Falls
Ministry of Health (China), National Center for Chronic and Noncommunicable Disease Control and Prevention (China), World Health Organization (WHO). China WHO Study on Global AGEing and Adult Health 2007-2010	Nonfatal Health Outcomes	Fire, heat, and hot substances
Qh Z, Zw L, Zx Z. An epidemiological study on etiology of mental retardation. Nat Med J Chin. 1994; 74(3): 134-7	Nonfatal Health Outcomes	Developmental intellectual disability
Liu J-M, Liao X-C. [Effect of integrated control of schistosomiasis in Shashi District from 2009 to 2013]. Chin J Schistosomiasis Control. 2014; 26(5): 581Â–2, 585	Nonfatal Health Outcomes	Schistosomiasis
Wu HB, Zhong JM, Hu RY, Wang H, Gong WW, Pan J, Fei FR, Wang M, Guo LH, Yang L, Yu M. Rapidly rising incidence of Type 1 diabetes in children and adolescents aged 0-19 years in Zhejiang, China, 2007 to 2013. Diabet Med. 2016; 33(10): 1339Â–46	Nonfatal Health Outcomes	Diabetes mellitus type 1
Shan C, Chen Y, Ma Y, Yang Y, Liu F, Xie X, Li X, Huang Y, Chen B, Ma X, He C. [Incidence and distribution of chronic heart failure in the elderly from Xinjiang]. Chin J Epidemiol. 2014; 35(9): 1007Â–10	Nonfatal Health Outcomes	
Peng J, Kong W, Guo D, Liu M, Wang Y, Zhu H, Pang B, Miao X, Yu B, Luo T, Hu Q, Zhou D. The epidemiology and etiology of influenza-like illness in Chinese children from 2008 to 2010. J Med Virol. 2012; 84(4): 672-8	Nonfatal Health Outcomes	Lower respiratory infections

Zhou H, Jia Y, Shen J, Wang S, Li X, Yang R, Huang K, Hu T, Tang F, Zhou J, Yuan J, Huang L, Tian X, Chen Z, Zhang Q, Wang C, Xi L, Deng D, Wang H, Ma D, Li S. Gynecologic infections seen in ThinPrep cytological test in Wuhan, China. Front Med. 2014; 8(2): 236Â–40	Nonfatal Health Outcomes	Sexually transmitted infections excluding HIV
Chen G, Yi X, Chen P, Dong J, Yang G, Fu S. A large-scale newborn hearing screening in rural areas in China. Int J Pediatr Otorhinolaryngol. 2012; 76(12): 1771-4	Nonfatal Health Outcomes	Age-related and other hearing loss
Zhang Z, Huang X-C. Epidemiological Survey on Alcohol Dependence in Western Hunan Autonomous Prefecture. China J Health Psychol. 2010; 18(3): 280-1	Nonfatal Health Outcomes	Alcohol use disorders
Guan B-Q, Luo X-R, Deng Y-L, Wei Z, Ye H-S, Yuan X-H, Ning Z-J, Yang W, Ding J. [Prevalence of psychiatric disorders in primary and middle school students in Hunan Province]. Chin J Contemp Pediatr. 2010; 12(2): 123-7	Nonfatal Health Outcomes	Anorexia nervosa
Guan B-Q, Luo X-R, Deng Y-L, Wei Z, Ye H-S, Yuan X-H, Ning Z-J, Yang W, Ding J. [Prevalence of psychiatric disorders in primary and middle school students in Hunan Province]. Chin J Contemp Pediatr. 2010; 12(2): 123-7	Nonfatal Health Outcomes	Attention-deficit/hyperactivity disorder
Guan B-Q, Luo X-R, Deng Y-L, Wei Z, Ye H-S, Yuan X-H, Ning Z-J, Yang W, Ding J. [Prevalence of psychiatric disorders in primary and middle school students in Hunan Province]. Chin J Contemp Pediatr. 2010; 12(2): 123-7	Nonfatal Health Outcomes	Conduct disorder
Yan L, Xiao-Ying Z, Ying HE. Investigation on prevalence rate of Parkinson's disease in population aged 55 years old and above in Kashi, Xinjiang between 2008 and 2009. Chin J Neurol. 2010; 12(43): 863-5	Nonfatal Health Outcomes	Parkinson's disease
Gao L-L. Epidemiological investigation on schizophrenia in shihezi city, Xinjiang province. Med Innov China. 2010; 7(12): 139-40	Nonfatal Health Outcomes	Schizophrenia
Ziao-Rong Pi, Ai-Zhong Liu, Jie Zhang. Epidemiological survey of epilepsy in Yueyang of Hunan Province. J Int Neurol Neurosurg. 2012; 39(2): 103-7	Nonfatal Health Outcomes	Epilepsy
Xiao B, Fan J, Deng Y, Ding Y, Muhit M, Kuper H. Using key informant method to assess the prevalence and causes of childhood blindness in Xiu'shui County, Jiangxi Province, Southeast China. Ophthalmic Epidemiol. 2011; 18(1): 30-5	Nonfatal Health Outcomes	
Chen J, Yu B, Wang Y, Tang M, Hu Y, Cai T, Zhang F, Zinkernagel D von, Harwell JI, Huang ZJ. Expansion of HIV care and treatment in Yunnan Province, China: Treatment outcomes with scale up of combination antiretroviral therapy. AIDS Care. 2014; 26(5): 633Â–41	Nonfatal Health Outcomes	HIV/AIDS resulting in other diseases
Awuti G, Younusi K, Li L, Upur H, Ren J. Epidemiological survey on the prevalence of periodontitis and diabetes mellitus in Uyghur adults from rural Hotan area in Xinjiang. Exp Diabetes Res. 2012; 758921	Nonfatal Health Outcomes	Periodontal diseases
Shan C, Chen Y, Ma Y, Yang Y, Liu F, Xie X, Li X, Huang Y, Chen B, Ma X, He C. [Incidence and distribution of chronic heart failure in the elderly from Xinjiang]. Chin J Epidemiol. 2014; 35(9): 1007Â–10	Nonfatal Health Outcomes	Heart failure
Xiao B, Fan J, Deng Y, Ding Y, Muhit M, Kuper H. Using key informant method to assess the prevalence and causes of childhood blindness in Xiu'shui County, Jiangxi Province, Southeast China. Ophthalmic Epidemiol. 2011; 18(1): 30-5	Nonfatal Health Outcomes	Vision loss
World Health Organization (WHO). WHO Tuberculosis Case Notifications. Geneva, Switzerland: World Health Organization (WHO)	Nonfatal Health Outcomes	HIV/AIDS - Multidrug-resistant Tuberculosis without extensive drug resistance
Vogel JP, Lee ACC, Souza JP. Maternal morbidity and preterm birth in 22 low- and middle-income countries: a secondary analysis of the WHO Global Survey dataset. BMC Pregnancy Childbirth. 2014; 56	Nonfatal Health Outcomes	Maternal hypertensive disorders
Huang DH, Zheng JO, Chen J, Yu L. Treatment gaps of epilepsy and retention rates of sodium valproate in rural Guangxi, China. Genet Mol Res. 2014; 13(3): 6202-12	Nonfatal Health Outcomes	Epilepsy
Li G, Chen Y, Hu H, Liu L, Hu X, Wang J, Shi W, Yin D. Association between age-related decline of kidney function and plasma malondialdehyde. Rejuvenation Res. 2012; 15(3): 257-64	Nonfatal Health Outcomes	Chronic kidney disease
Zhu Q, Wang L, Lin W, Bulterys M, Yang W, Sun D, Cui Z, Kaplan J, Kleinman N, Wei X, Chung J, Wang Z. Improved survival with co-trimoxazole prophylaxis among people living with HIV/AIDS who initiated antiretroviral treatment in Henan Province, China. Curr HIV Res. 2014; 12(5): 359Â–65	Nonfatal Health Outcomes	HIV/AIDS resulting in other diseases
Onozaki I. Prevalence Survey Global Overview: Background, Survey results since 2007, Lessons and Implications to the Program. Presented at: WHO Multi-country Global Workshop on TB Prevalence Surveys and TB Surveillance; 2013 April 29 - May 3; Accra, Ghana. PowerPoint presentation	Nonfatal Health Outcomes	Tuberculosis
Yu S, Liu R, Zhao G, Yang X, Qiao X, Feng J, Fang Y, Cao X, He M, Steiner T. The prevalence and burden of primary headaches in China: a population-based door-to-door survey. Headache. 2012; 52(4): 582-91	Nonfatal Health Outcomes	Migraine
Le C, Jun D, Zhankun S, Yichun L, Jie T. Socioeconomic differences in diabetes prevalence, awareness, and treatment in rural southwest China. Trop Med Int Health. 2011; 16(9): 1070-6	Nonfatal Health Outcomes	Diabetes mellitus

International Centre for Eye Health (ICEH). China - Kunming Rapid Assessment of Avoidable Blindness 2006. Grootebroek, Netherlands: RAAB Repository	Nonfatal Health Outcomes	Refraction disorders
International Centre for Eye Health (ICEH). China - Gao'an Rapid Assessment of Avoidable Blindness 2007. Grootebroek, Netherlands: RAAB Repository	Nonfatal Health Outcomes	Cataract
International Centre for Eye Health (ICEH). China - Xingan Rapid Assessment of Avoidable Blindness 2007. Grootebroek, Netherlands: RAAB Repository	Nonfatal Health Outcomes	Cataract
International Centre for Eye Health (ICEH). China - Gao'an Rapid Assessment of Avoidable Blindness 2007. Grootebroek, Netherlands: RAAB Repository	Nonfatal Health Outcomes	Age-related macular degeneration
International Centre for Eye Health (ICEH). China - Xingan Rapid Assessment of Avoidable Blindness 2007. Grootebroek, Netherlands: RAAB Repository	Nonfatal Health Outcomes	Refraction and accommodation disorders
International Centre for Eye Health (ICEH). China - Wanzai Rapid Assessment of Avoidable Blindness 2007. Grootebroek, Netherlands: RAAB Repository	Nonfatal Health Outcomes	Refraction and accommodation disorders
International Centre for Eye Health (ICEH). China - Xingan Rapid Assessment of Avoidable Blindness 2007. Grootebroek, Netherlands: RAAB Repository	Nonfatal Health Outcomes	Other vision loss
International Centre for Eye Health (ICEH). China - Gao'an Rapid Assessment of Avoidable Blindness 2007. Grootebroek, Netherlands: RAAB Repository	Nonfatal Health Outcomes	Other vision loss
Li J, Zhong H, Cai N, Luo T, Li J, Su X, Li X, Qiu X, Yang Y, Yuan Y, Yu M. The prevalence and causes of visual impairment in an elderly Chinese Bai ethnic rural population: the Yunnan minority eye study. Invest Ophthalmol Vis Sci. 2012; 53(8): 4498-504	Nonfatal Health Outcomes	
International Centre for Eye Health (ICEH). China - Inner Mongolia Tuoketuo Rapid Assessment of Avoidable Blindness 2010	Nonfatal Health Outcomes	Vision loss
Yao W, Mai X, Luo C, Ai F, Chen Q. A cross-sectional survey of nonspecific low back pain among 2083 schoolchildren in China. Spine. 2011; 36(22): 1885-90	Nonfatal Health Outcomes	Low back and neck pain
Zhao J, Bai J, Shen K, Xiang L, Huang S, Chen A, Huang Y, Wang J, Ye R. Self-reported prevalence of childhood allergic diseases in three cities of China: a multicenter study. BMC Public Health. 2010; 10: 551	Nonfatal Health Outcomes	Dermatitis
Chen Z, Du J, Shao L, Zheng L, Wu M, Ai M, Zhang Y. Prepregnancy body mass index, gestational weight gain, and pregnancy outcomes in China. Int J Gynaecol Obstet. 2010; 109(1): 41-4	Nonfatal Health Outcomes	Neonatal preterm birth
Li J, Zhong H, Cai N, Luo T, Li J, Su X, Li X, Qiu X, Yang Y, Yuan Y, Yu M. The prevalence and causes of visual impairment in an elderly Chinese Bai ethnic rural population: the Yunnan minority eye study. Invest Ophthalmol Vis Sci. 2012; 53(8): 4498-504	Nonfatal Health Outcomes	Vision loss
International Centre for Eye Health (ICEH). China - Xingan Rapid Assessment of Avoidable Blindness 2007. Grootebroek, Netherlands: RAAB Repository	Nonfatal Health Outcomes	
International Centre for Eye Health (ICEH). China - Xingan Rapid Assessment of Avoidable Blindness 2007. Grootebroek, Netherlands: RAAB Repository	Nonfatal Health Outcomes	Trachoma
Zhu P, Tao F, Hao J, Sun Y, Jiang X. Prenatal life events stress: implications for preterm birth and infant birthweight. Am J Obstet Gynecol. 2010; 203(1): 34	Nonfatal Health Outcomes	Neonatal preterm birth
Zhang X-J, Shen Q, Wang G-Y, Yu Y-L, Sun Y-H, Yu G-B, Zhao D, Ye D-Q. Risk factors for reproductive tract infections among married women in rural areas of Anhui Province, China. Eur J Obstet Gynecol Reprod Biol. 2009; 147(2): 187-91	Nonfatal Health Outcomes	Trichomoniasis
Shen M, Huang N, Feng X, Zhang Y, Wang L, Xiong M, Li T, Yang W, Yang W, Chen S. [Analysis of schistosomiasis endemic status of national surveillance sites in Yunnan Province, 2014]. Chin J Schistosomiasis Control. 2015; 27(5): 463Â–6	Nonfatal Health Outcomes	Schistosomiasis
Li LL, Ma Y, Chen R, Liu ZM, Qin X et al. Prevalence of dementia among the elderly in rural community of Anhui. Chin J Dis Control Prev. 2011; 15(4): 292-4	Nonfatal Health Outcomes	Alzheimer's disease and other dementias
Zhang Q, Guo Z, Bai Z, MacDonald NE. A 4 year prospective study to determine risk factors for severe community acquired pneumonia in children in southern China. Pediatr Pulmonol. 2013; 48(4): 390-7	Nonfatal Health Outcomes	Lower respiratory infections
Zhang Q, Guo Z, Bai Z, MacDonald NE. A 4 year prospective study to determine risk factors for severe community acquired pneumonia in children in southern China. Pediatr Pulmonol. 2013; 48(4): 390-7	Nonfatal Health Outcomes	Influenza
Zhang Q, Guo Z, Langley JM, Bai Z. Respiratory syncytial virus-associated intensive care unit admission in children in Southern China. BMC Res Notes. 2013; 447	Nonfatal Health Outcomes	Respiratory syncytial virus pneumonia
Zhang Q, Guo Z, Bai Z, MacDonald NE. A 4 year prospective study to determine risk factors for severe community acquired pneumonia in children in southern China. Pediatr Pulmonol. 2013; 48(4): 390-7	Nonfatal Health Outcomes	Respiratory syncytial virus pneumonia
Chen J, Yu B, Wang Y, Tang M, Hu Y, Cai T, Zhang F, Zinkernagel D von, Harwell JI, Huang ZJ. Expansion of HIV care and treatment in Yunnan Province, China: Treatment outcomes with scale up of combination antiretroviral therapy. AIDS Care. 2014; 26(5): 633Â–41	Nonfatal Health Outcomes	HIV/AIDS
Bai J, Zhao J, Shen K-L, Xiang L, Chen A-H, Huang S, Huang Y, Wang J-S, Ye R-W. Current trends of the prevalence of childhood asthma in three Chinese cities: a multicenter epidemiological survey. Biomed Environ Sci. 2010; 23(6): 453-7	Nonfatal Health Outcomes	Asthma

Chinese Center for Disease Control and Prevention (CCDC), Ministry of Health (China). China National Injury Surveillance System 2007 - China CDC	Nonfatal Health Outcomes	Injuries
Chinese Center for Disease Control and Prevention (CCDC), Ministry of Health (China). China National Injury Surveillance System 2008 - China CDC	Nonfatal Health Outcomes	Injuries
Zhu Q, Wang L, Lin W, Bulterys M, Yang W, Sun D, Cui Z, Kaplan J, Kleinman N, Wei X, Chung J, Wang Z. Improved survival with co-trimoxazole prophylaxis among people living with HIV/AIDS who initiated antiretroviral treatment in Henan Province, China. Curr HIV Res. 2014; 12(5): 359Â–65	Nonfatal Health Outcomes	HIV/AIDS
Zhou J, Yuan Y, Zhang X, Guan HJ. [A prevalence investigation of blindness and low vision in 2008 among adults aged 60 years or above in 2 villages of Nantong]. Chin J Ophthalmol. 2012; 48(10): 908-14	Nonfatal Health Outcomes	Vision loss
Lu J, Li H, Tian R, Jiang Y, Xu Y, Yu D, Jiang J, Bi S. [Hepatitis viruses infection situation in human population of the Gansu province]. Chin J Exper Clin Virol. 2009; 23(5): 349-51	Nonfatal Health Outcomes	Acute hepatitis E
Cao B, Song S-F, Bai L, Yin YD, Zhang Y-Y, Liu Y-M, Guo P, Wang C, Ren L-L, Wang J-W, Zhao F, Zhang J-Z, Gonzalez R. Viral and Mycoplasma pneumoniae community-acquired pneumonia and novel clinical outcome evaluation in ambulatory adult patients in China. Eur J Clin Microbiol Infect Dis. 2010; 29(11): 1443-8	Nonfatal Health Outcomes	Influenza
Lu G, Li J, Xie Z, Liu C, Guo L, Vernet G, Shen K, Wang J. Human metapneumovirus associated with community-acquired pneumonia in children in Beijing, China. J Med Virol. 2013; 85(1): 138-43	Nonfatal Health Outcomes	Influenza
Zhang C, Zhu N, Xie Z, Lu R, He B, Liu C, Ma X, Tan W. Viral etiology and clinical profiles of children with severe acute respiratory infections in China. PLoS One. 2013; 8(8): e72606	Nonfatal Health Outcomes	Influenza
Yang X, Yao Y, Chen M, Yang X, Xie Y, Liu Y, Zhao X, Gao Y, Wei L. Etiology and clinical characteristics of influenza-like illness (ILI) in outpatients in Beijing, June 2010 to May 2011. PLoS One. 2012; 7(1): e28786	Nonfatal Health Outcomes	Influenza
Cao B, Song S-F, Bai L, Yin YD, Zhang Y-Y, Liu Y-M, Guo P, Wang C, Ren L-L, Wang J-W, Zhao F, Zhang J-Z, Gonzalez R. Viral and Mycoplasma pneumoniae community-acquired pneumonia and novel clinical outcome evaluation in ambulatory adult patients in China. Eur J Clin Microbiol Infect Dis. 2010; 29(11): 1443-8	Nonfatal Health Outcomes	Respiratory syncytial virus pneumonia
Lu G, Li J, Xie Z, Liu C, Guo L, Vernet G, Shen K, Wang J. Human metapneumovirus associated with community-acquired pneumonia in children in Beijing, China. J Med Virol. 2013; 85(1): 138-43	Nonfatal Health Outcomes	Respiratory syncytial virus pneumonia
Zhang C, Zhu N, Xie Z, Lu R, He B, Liu C, Ma X, Tan W. Viral etiology and clinical profiles of children with severe acute respiratory infections in China. PLoS One. 2013; 8(8): e72606	Nonfatal Health Outcomes	Respiratory syncytial virus pneumonia
Liu C, Xiao Y, Zhang J, Ren L, Li J, Xie Z, Xu B, Yang Y, Qian S, Wang J, Shen K. Adenovirus infection in children with acute lower respiratory tract infections in Beijing, China, 2007 to 2012. BMC Infect Dis. 2015; 15: 408	Nonfatal Health Outcomes	Respiratory syncytial virus pneumonia
Li C, Wu M, Wang J, Zhang S, Zhu L, Pan J, Zhang W. A population-based study on the risks of cervical lesion and human papillomavirus infection among women in Beijing, People's Republic of China. Cancer Epidemiol Biomark Prev. 2010; 19(10): 2655-64	Nonfatal Health Outcomes	Trichomoniasis
Wu HB, Zhong JM, Hu RY, Wang H, Gong WW, Pan J, Fei FR, Wang M, Guo LH, Yang L, Yu M. Rapidly rising incidence of Type 1 diabetes in children and adolescents aged 0-19 years in Zhejiang, China, 2007 to 2013. Diabet Med. 2016; 33(10): 1339Â–46	Nonfatal Health Outcomes	Diabetes mellitus
Zhao J, Bai J, Shen K, Xiang L, Huang S, Chen A, Huang Y, Wang J, Ye R. Self-reported prevalence of childhood allergic diseases in three cities of China: a multicenter study. BMC Public Health. 2010; 10: 551	Nonfatal Health Outcomes	Asthma
Zhang Q, Guo Z, Langley JM, Bai Z. Respiratory syncytial virus-associated intensive care unit admission in children in Southern China. BMC Res Notes. 2013; 447	Nonfatal Health Outcomes	Lower respiratory infections
Prince M, Acosta D, Ferri CP, Guerra M, Huang Y, Llibre Rodriguez JJ, Salas A, Sosa AL, Williams JD, Dewey ME, Acosta I, Jotheeswaran AT, Liu Z. Dementia incidence and mortality in middle-income countries, and associations with indicators of cognitive reserve: a 10/66 Dementia Research Group population-based cohort study. Lancet. 2012; 380(9836): 50-8	Nonfatal Health Outcomes	Alzheimer's disease and other dementias
Zhou XH, Ji LN, Luo YY, Zhang XY, Han XY, Qiao Q. Performance of HbA(1c) for detecting newly diagnosed diabetes and pre-diabetes in Chinese communities living in Beijing. Diabet Med. 2009; 26(12): 1262-8	Nonfatal Health Outcomes	Diabetes mellitus
Cai L, Liu A, Zhang L, Li S, Wang P. Prevalence, awareness, treatment, and control of hypertension among adults in Beijing, China. Clin Exp Hypertens. 2012; 34(1): 45Â–52	Nonfatal Health Outcomes	Diabetes mellitus

Wang F, Ye P, Luo L, Xiao W, Qi L, Bian S, Wu H, Sheng L, Xiao T, Xu R. Association of serum lipids with arterial stiffness in a population-based study in Beijing. Eur J Clin Invest. 2011; 41(9): 929Â–36	Nonfatal Health Outcomes	Diabetes mellitus
Zhan Y, Zhuang J, Dong Y, Xu H, Hu D, Yu J. Predicting the prevalence of peripheral arterial diseases: modelling and validation in different cohorts. VASA. 2016; 45(1): 31-9	Nonfatal Health Outcomes	Diabetes mellitus
Zhuang J, Zhan Y, Zhang F, Tang Z, Wang J, Sun Y, Ding R, Hu D, Yu J. Self-reported insomnia and coronary heart disease in the elderly. Clin Exp Hypertens. 2016; 38(1): 51Â–5	Nonfatal Health Outcomes	Diabetes mellitus
Gao HL, Lin SQ, Wei Y, Chen Y, Wu ZL. The effect of age and menopausal status on musculoskeletal symptoms in Chinese women aged 35-64 years. Climacteric. 2013; 16(6): 639-45	Nonfatal Health Outcomes	Neck pain
Li R, Sun J, Ren L-M, Wang H-Y, Liu W-H, Zhang X-W, Chen S, Mu R, He J, Zhao Y, Long L, Liu Y-Y, Liu X, Lu X-L, Li Y-H, Wang S-Y, Pan S-S, Li C, Wang H-Y, Li Z-G. Epidemiology of eight common rheumatic diseases in China: a large-scale cross-sectional survey in Beijing. Rheumatology (Oxford). 2012; 51(4): 721-9	Nonfatal Health Outcomes	Gout
Li R, Sun J, Ren L-M, Wang H-Y, Liu W-H, Zhang X-W, Chen S, Mu R, He J, Zhao Y, Long L, Liu Y-Y, Liu X, Lu X-L, Li Y-H, Wang S-Y, Pan S-S, Li C, Wang H-Y, Li Z-G. Epidemiology of eight common rheumatic diseases in China: a large-scale cross-sectional survey in Beijing. Rheumatology (Oxford). 2012; 51(4): 721-9	Nonfatal Health Outcomes	Psoriasis
You QS, Xu L, Wang YX, Liang QF, Cui TT, Yang XH, Yang H, Jonas JB. Prevalence of diabetic retinopathy as cause for visual impairment: the Beijing Public Health Care Project. Clin Experiment Ophthalmol. 2013; 41(6.0): 608-9	Nonfatal Health Outcomes	Cataract
Liu Z, Huang J, Qian D, Chen F, Xu J, Li S, Jin L, Chu X, Wang X. Prevalence and related factors of chronic kidney disease (CKD) among long-lived individuals (LLI) over 95 years of age. Arch Gerontol Geriatr. 2015; 60(2): 354-8	Nonfatal Health Outcomes	Chronic kidney disease
ZHU Yu-hua, JI Fei, YU Ning, et al. The Distribution of Hearing Levels and the Self-cognitive Abilities of the Elderly People in a Community in Beijing. Chin Sci J Hear Speech Rehabil. 2013; 58: 185-87	Nonfatal Health Outcomes	Hearing loss
Xu L, Jonas JB, Cui TT, You QS, Wang YX, Yang H, Li JJ, Wei WB, Liang QF, Wang S, Yang XH, Zhang L, Beijing Institute of Ophthalmology, Beijing Tongren Hospital, Capital Medical University. Beijing Eye Public Health Care Project. Ophthalmology. 2012; 119(6): 1167-74	Nonfatal Health Outcomes	Vision loss
You QS, Xu L, Wang YX, Liang QF, Cui TT, Yang XH, Yang H, Jonas JB. Prevalence of diabetic retinopathy as cause for visual impairment: the Beijing Public Health Care Project. Clin Experiment Ophthalmol. 2013; 41(6.0): 608-9	Nonfatal Health Outcomes	Vision loss
Zhou J, Yuan Y, Zhang X, Guan HJ. [A prevalence investigation of blindness and low vision in 2008 among adults aged 60 years or above in 2 villages of Nantong]. Chin J Ophthalmol. 2012; 48(10): 908-14	Nonfatal Health Outcomes	
Zhang F, Sui Q, Wang J, Xue S, Lu X, Ma X, et al. The latest investigation of autism epidemic of children aged from 1 to 6 years old in Wuxi city. Matern Child Health Care China. 2008; 23(27): 3878Â–3880	Nonfatal Health Outcomes	Autism spectrum disorders
Wu X, Lu Y, Wang Y, Zheng Q, Wang T, Lin J, Chen J. Investigation of childhood autism status in Lianyungang city. J Mod Med Health. 2010; 26: 3724Â–3726	Nonfatal Health Outcomes	Autism spectrum disorders
Xiao B, Kuper H, Guan C, Bailey K, Limburg H. Rapid assessment of avoidable blindness in three counties, Jiangxi Province, China. Br J Ophthalmol. 2010; 94(11): 1437-42	Nonfatal Health Outcomes	Trachoma
International Centre for Eye Health (ICEH). China - Gao'an Rapid Assessment of Avoidable Blindness 2007. Grootebroek, Netherlands: RAAB Repository	Nonfatal Health Outcomes	Trachoma
International Centre for Eye Health (ICEH). China - Wanzai Rapid Assessment of Avoidable Blindness 2007. Grootebroek, Netherlands: RAAB Repository	Nonfatal Health Outcomes	Trachoma
International Centre for Eye Health (ICEH). China - Gao'an Rapid Assessment of Avoidable Blindness 2007. Grootebroek, Netherlands: RAAB Repository	Nonfatal Health Outcomes	Glaucoma
International Centre for Eye Health (ICEH). China - Wanzai Rapid Assessment of Avoidable Blindness 2007. Grootebroek, Netherlands: RAAB Repository	Nonfatal Health Outcomes	Glaucoma
International Centre for Eye Health (ICEH). China - Wanzai Rapid Assessment of Avoidable Blindness 2007. Grootebroek, Netherlands: RAAB Repository	Nonfatal Health Outcomes	Cataract
International Centre for Eye Health (ICEH). China - Wanzai Rapid Assessment of Avoidable Blindness 2007. Grootebroek, Netherlands: RAAB Repository	Nonfatal Health Outcomes	Age-related macular degeneration
International Centre for Eye Health (ICEH). China - Gao'an Rapid Assessment of Avoidable Blindness 2007. Grootebroek, Netherlands: RAAB Repository	Nonfatal Health Outcomes	Refraction disorders
Zhou L, Shi J. A clinical diagnostic analysis of infant and child autism. China Mod Dr. 2012; 50(17): 50-51	Nonfatal Health Outcomes	Autism spectrum disorders
Ren L, Xiao Q, Zhou L, Xia Q, Liu E. Molecular characterization of human respiratory syncytial virus subtype B: a novel genotype of subtype B circulating in China. J Med Virol. 2015; 87(1): 1-9	Nonfatal Health Outcomes	Lower respiratory infections

World Health Organization (WHO). WHO Tuberculosis Case Notifications. Geneva, Switzerland: World Health Organization (WHO)	Nonfatal Health Outcomes	HIV/AIDS - Extensively drug-resistant Tuberculosis
Gao B, Zhu L, Pan Y, Yang S, Zhang L, Wang H. Ocular fundus pathology and chronic kidney disease in a Chinese population. BMC Nephrol. 2011; 12: 62	Nonfatal Health Outcomes	Chronic kidney disease
International Centre for Eye Health (ICEH). China - Gao'an Rapid Assessment of Avoidable Blindness 2007. Grootebroek, Netherlands: RAAB Repository	Nonfatal Health Outcomes	Vision loss
International Centre for Eye Health (ICEH). China - Wanzai Rapid Assessment of Avoidable Blindness 2007. Grootebroek, Netherlands: RAAB Repository	Nonfatal Health Outcomes	Vision loss
International Centre for Eye Health (ICEH). China - Xingan Rapid Assessment of Avoidable Blindness 2007. Grootebroek, Netherlands: RAAB Repository	Nonfatal Health Outcomes	Vision loss
International Centre for Eye Health (ICEH). China - Gao'an Rapid Assessment of Avoidable Blindness 2007. Grootebroek, Netherlands: RAAB Repository	Nonfatal Health Outcomes	Diabetes mellitus
International Centre for Eye Health (ICEH). China - Wanzai Rapid Assessment of Avoidable Blindness 2007. Grootebroek, Netherlands: RAAB Repository	Nonfatal Health Outcomes	Diabetes mellitus
International Centre for Eye Health (ICEH). China - Xingan Rapid Assessment of Avoidable Blindness 2007. Grootebroek, Netherlands: RAAB Repository	Nonfatal Health Outcomes	Diabetes mellitus
Jiang C, Shen YH, Qin XY, Li Y, Cai W, Li YL, Wu X, Hu YH. [A Community-based Epidemiologic Study on Gastroesophageal Reflux Disease in Haidian District of Beijing]. Chin J Prev Med. 2010; 44(6): 516-21	Nonfatal Health Outcomes	Gastroesophageal reflux disease
Ren L, Xiao Q, Zhou L, Xia Q, Liu E. Molecular characterization of human respiratory syncytial virus subtype B: a novel genotype of subtype B circulating in China. J Med Virol. 2015; 87(1): 1-9	Nonfatal Health Outcomes	Respiratory syncytial virus pneumonia
Zhao SM, Jiang TL, Gao FX, Lu L, Zheng HQ, Hu J, Fan YH, Li B, Xiao RR, Yury K. Analysis of true voluntary blood donors with anti-HCV prevalence and implications for donor management in Chongqing, China. Transfus Med. 2007; 17(3): 210-1	Nonfatal Health Outcomes	Acute hepatitis C
Zhang X-J, Shen Q, Wang G-Y, Yu Y-L, Sun Y-H, Yu G-B, Zhao D, Ye D-Q. Risk factors for reproductive tract infections among married women in rural areas of Anhui Province, China. Eur J Obstet Gynecol Reprod Biol. 2009; 147(2): 187-91	Nonfatal Health Outcomes	Sexually transmitted infections excluding HIV
Yao XY, Yu J, Chen SP, Xiao JW, Zheng QC, Liu HY, Zhang L, Xian Y, Zou L. Prevalence and genetic analysis of a-thalassemia and b-thalassemia in Chongqing area of China. Gene. 2013; 532(1): 120-4	Nonfatal Health Outcomes	Thalassemias
Liu X, Du J, Wang G, Chen Z, Wang W, Xi Q. Effect of pre-pregnancy body mass index on adverse pregnancy outcome in north of China. Arch Gynecol Obstet. 2011; 283(1): 65-70	Nonfatal Health Outcomes	Maternal disorders
Zhang Z, Huang Y, Su P, Wang D, Wang L. Experience in treating congenital esophageal atresia in China. J Pediatr Surg. 2010; 45(10): 2009-14	Nonfatal Health Outcomes	Congenital birth defects
Ho TC, Ouyang H, Lu Y, Young AH, Chintala K, Detrano RC. Postprocedural outcomes of rural children undergoing correction of congenital heart lesions in Yunnan Province, China. Pediatr Cardiol. 2011; 32(6): 811-4	Nonfatal Health Outcomes	Congenital birth defects
Wang Y, Liang D, Tang C-H, Zhang W-L, Zhou F-J. Epidemiological Investigation on ADHD among Students aged from 6-8 in Changchun City. Aerosp Med. 2010; 21(2): 239-40	Nonfatal Health Outcomes	Attention-deficit/hyperactivity disorder
Feng L, Lai S, Li F, Ye X, Li S, Ren X, Zhang H, Li Z, Yu H, Yang W. [Viral etiologies of hospitalized pneumonia patients aged less than five years in six provinces, 2009-2012]. Chin J Epidemiol. 2014; 35(6): 646Â–9	Nonfatal Health Outcomes	Lower respiratory infections
Zhang PJ, Cao B, Li XL, Liang LR, Yang SG, Gu L, Xu Z, Hu K, Zhang HY, Yan XX, Huang WB, Chen W, Zhang JX, Li LJ, Wang C. Risk factors for adult death due to 2009 pandemic influenza A (H1N1) virus infection: a 2151 severe and critical cases analysis. Chin Med J (Engl). 2013; 126(12): 2222-8	Nonfatal Health Outcomes	Lower respiratory infections
Feng L, Li Z, Zhao S, Nair H, Lai S, Xu W, Li M, Wu J, Ren L, Liu W, Yuan Z, Chen Y, Wang X, Zhao Z, Zhang H, Li F, Ye X, Li S, Feikin D, Yu H, Yang W. Viral etiologies of hospitalized acute lower respiratory infection patients in China, 2009-2013. PLoS One. 2014; 9(6): e99419	Nonfatal Health Outcomes	Lower respiratory infections
DananchÃ© C, SÃ¡nchez Picot V, BÃ©net T, Messaoudi M, Chou M, Wang J, Pape J-W, Awasthi S, Bavdekar A, Rakoto-Andrianarivelo M, Sylla M, Nymadawa P, Russomando G, Komurian-Pradel F, Endtz H, Paranhos-BaccalÃ G, Vanhems P, For The Gabriel Network null. Burden of Influenza in Less Than 5-Year-Old Children Admitted to Hospital with Pneumonia in Developing and Emerging Countries: A Descriptive, Multicenter Study. Am J Trop Med Hyg. 2018; 98(6): 1805Â–10	Nonfatal Health Outcomes	Lower respiratory infections
Jin Y. Guangxi monitoring network of acute encephalitis, meningitis epidemiological surveillance studies. Dis Surveillance. 2010; 25(3)	Nonfatal Health Outcomes	Hearing loss
Jin Y. Guangxi monitoring network of acute encephalitis, meningitis epidemiological surveillance studies. Dis Surveillance. 2010; 25(3)	Nonfatal Health Outcomes	Vision loss
Chinese Center for Disease Control and Prevention (CCDC). China Notifiable Infectious Diseases 2008	Nonfatal Health Outcomes	Gonococcal infection

Lu W-P, Lin G-X, Shi S, Dong J-H. Simultaneously high prevalences of hepatitis B and C virus infections in a population in Putian County, China. J Clin Microbiol. 2012; 50(6): 2142-4	Nonfatal Health Outcomes	Acute hepatitis B
Cao B, Song S-F, Bai L, Yin YD, Zhang Y-Y, Liu Y-M, Guo P, Wang C, Ren L-L, Wang J-W, Zhao F, Zhang J-Z, Gonzalez R. Viral and Mycoplasma pneumoniae community-acquired pneumonia and novel clinical outcome evaluation in ambulatory adult patients in China. Eur J Clin Microbiol Infect Dis. 2010; 29(11): 1443-8	Nonfatal Health Outcomes	Lower respiratory infections
Lu G, Li J, Xie Z, Liu C, Guo L, Vernet G, Shen K, Wang J. Human metapneumovirus associated with community-acquired pneumonia in children in Beijing, China. J Med Virol. 2013; 85(1): 138-43	Nonfatal Health Outcomes	Lower respiratory infections
Zhang C, Zhu N, Xie Z, Lu R, He B, Liu C, Ma X, Tan W. Viral etiology and clinical profiles of children with severe acute respiratory infections in China. PLoS One. 2013; 8(8): e72606	Nonfatal Health Outcomes	Lower respiratory infections
Yang X, Yao Y, Chen M, Yang X, Xie Y, Liu Y, Zhao X, Gao Y, Wei L. Etiology and clinical characteristics of influenza-like illness (ILI) in outpatients in Beijing, June 2010 to May 2011. PLoS One. 2012; 7(1): e28786	Nonfatal Health Outcomes	Lower respiratory infections
Bao Z, Yuan X, Wang L, Sun Y, Dong X. The incidence and etiology of community-acquired pneumonia in fever outpatients. Exp Biol Med (Maywood). 2012; 237(11): 1256-61	Nonfatal Health Outcomes	Lower respiratory infections
Liu C, Xiao Y, Zhang J, Ren L, Li J, Xie Z, Xu B, Yang Y, Qian S, Wang J, Shen K. Adenovirus infection in children with acute lower respiratory tract infections in Beijing, China, 2007 to 2012. BMC Infect Dis. 2015; 15: 408	Nonfatal Health Outcomes	Lower respiratory infections
Li C, Wu M, Wang J, Zhang S, Zhu L, Pan J, Zhang W. A population-based study on the risks of cervical lesion and human papillomavirus infection among women in Beijing, People's Republic of China. Cancer Epidemiol Biomark Prev. 2010; 19(10): 2655-64	Nonfatal Health Outcomes	Sexually transmitted infections excluding HIV
Liu X, Du J, Wang G, Chen Z, Wang W, Xi Q. Effect of pre-pregnancy body mass index on adverse pregnancy outcome in north of China. Arch Gynecol Obstet. 2011; 283(1): 65-70	Nonfatal Health Outcomes	Maternal hemorrhage
Liu X, Du J, Wang G, Chen Z, Wang W, Xi Q. Effect of pre-pregnancy body mass index on adverse pregnancy outcome in north of China. Arch Gynecol Obstet. 2011; 283(1): 65-70	Nonfatal Health Outcomes	Maternal hypertensive disorders
Zhang Z, Huang Y, Su P, Wang D, Wang L. Experience in treating congenital esophageal atresia in China. J Pediatr Surg. 2010; 45(10): 2009-14	Nonfatal Health Outcomes	Digestive congenital anomalies
Fang X, Chen Y, Chen C, Xiong D, Ji J, Shi G, Zheng J, Chen B, Chen T, Weng S. Epidemiological survey of mental disorder in Fujian province. Chin J Psychiatry. 2011; 44(2): 103Â–7	Nonfatal Health Outcomes	Anorexia nervosa
Fang X, Chen Y, Chen C, Xiong D, Ji J, Shi G, Zheng J, Chen B, Chen T, Weng S. Epidemiological survey of mental disorder in Fujian province. Chin J Psychiatry. 2011; 44(2): 103Â–7	Nonfatal Health Outcomes	Bulimia nervosa
Lin L, Chen G, Zou X, Zhao J, Zhu F, Tu M, Xu S, Lin W, Yang S, Zhang Y, Lin M, Chen N, Huang H, Liang J, Li L, Yao J. Diabetes, pre-diabetes and associated risks on Minnesota code-indicated major electrocardiogram abnormality among Chinese: a cross-sectional diabetic study in Fujian province, southeast China. Obes Rev. 2009; 10(4): 420-30	Nonfatal Health Outcomes	Diabetes mellitus
Qh Z, Zw L, Zx Z. An epidemiological study on etiology of mental retardation. Nat Med J Chin. 1994; 74(3): 134-7	Nonfatal Health Outcomes	Idiopathic developmental intellectual disability
Chen Q, Li M, Li M, Lu X, LÃ¼ R, Sun J, Liu Z. Molecular basis of weak D and DEL in Han population in Anhui Province, China. Chin Med J (Engl). 2012; 125(18): 3251-5	Nonfatal Health Outcomes	Neonatal disorders
Li Q, Hou L, Guo Z-H, Ye L-Y, Yue D-Q, Zhu Z-Y. Molecular basis of the RHD gene in blood donors with DEL phenotypes in Shanghai. Vox Sang. 2009; 97(2): 139-46	Nonfatal Health Outcomes	Neonatal disorders
Zhao Y, Yao Z, D'Souza W, Zhu C, Chun H, Zhuoga C, Zhang Q, Hu X, Zhou D. An Epidemiological Survey of Stroke in Lhasa, Tibet, China. Stroke. 2010; 41(12): 2739-43	Nonfatal Health Outcomes	Intracerebral hemorrhage
Ho TC, Ouyang H, Lu Y, Young AH, Chintala K, Detrano RC. Postprocedural outcomes of rural children undergoing correction of congenital heart lesions in Yunnan Province, China. Pediatr Cardiol. 2011; 32(6): 811-4	Nonfatal Health Outcomes	Congenital heart anomalies
Hu WS, Feng Y, Dong MY, He J. Comparing maternal and perinatal outcomes in pregnancies complicated by preeclampsia superimposed chronic hypertension and preeclampsia alone. Clin Exp Obstet Gynecol. 2016; 43(2): 212-5	Nonfatal Health Outcomes	Maternal hypertensive disorders
Wang J, Zhu W, Huang S, Xu L, Miao M, Wu C, Yu C, Li Y, Xu C. Serum apoB levels independently predict the development of non-alcoholic fatty liver disease: A 7-year prospective study. Liver Int. 2017; 37(8): 1202-1208	Nonfatal Health Outcomes	Cirrhosis due to NASH
You QS, Xu L, Wang YX, Liang QF, Cui TT, Yang XH, Yang H, Jonas JB. Prevalence of diabetic retinopathy as cause for visual impairment: the Beijing Public Health Care Project. Clin Experiment Ophthalmol. 2013; 41(6.0): 608-9	Nonfatal Health Outcomes	

ZHU Yu-hua, JI Fei, YU Ning, et al. The Distribution of Hearing Levels and the Self-cognitive Abilities of the Elderly People in a Community in Beijing. Chin Sci J Hear Speech Rehabil. 2013; 58: 185-87	Nonfatal Health Outcomes	Age-related and other hearing loss
Wu J, Xu H, He X, Yuan Y, Wang C, Sun J, He S, Niu J. Six-year changes in the prevalence of obesity and obesity-related diseases in Northeastern China from 2007 to 2013. Sci Rep. 2017; 7: 41518	Nonfatal Health Outcomes	Cirrhosis due to NASH
Xiaoli Y, Chao J, Wen P, Wenming X, Fang L, Ning L, Huijuan M, Jun N, Ming L, Xiaoxia A, Chuanyou Y, Zenguo F, Lili L, Lianzheng Y, Lijuan T, Guowei P. Prevalence of psychiatric disorders among children and adolescents in northeast China. PLoS One. 2014; 9(10): e111223	Nonfatal Health Outcomes	Major depressive disorder
He Y, Lin GY, Wang Q, Cai XY, Zhang YH, Lin CX, Lu CD, Lu XD. A 3-year prospective study of the epidemiology of acute respiratory viral infections in hospitalized children in Shenzhen, China. Influenza Other Respir Viruses. 2014; 8(4): 443-51	Nonfatal Health Outcomes	Respiratory syncytial virus pneumonia
Hu Y, Teng W, Liu L, Chen K, Liu L, Hua R, Chen J, Zhou Y, Chen L. Prevalence and risk factors of diabetes and diabetic retinopathy in Liaoning province, China: a population-based cross-sectional study. PLoS One. 2015; 10(3): e0121477	Nonfatal Health Outcomes	Diabetes mellitus
Feng L, Lai S, Li F, Ye X, Li S, Ren X, Zhang H, Li Z, Yu H, Yang W. [Viral etiologies of hospitalized pneumonia patients aged less than five years in six provinces, 2009-2012]. Chin J Epidemiol. 2014; 35(6): 646Â–9	Nonfatal Health Outcomes	Influenza
Zhang PJ, Cao B, Li XL, Liang LR, Yang SG, Gu L, Xu Z, Hu K, Zhang HY, Yan XX, Huang WB, Chen W, Zhang JX, Li LJ, Wang C. Risk factors for adult death due to 2009 pandemic influenza A (H1N1) virus infection: a 2151 severe and critical cases analysis. Chin Med J (Engl). 2013; 126(12): 2222-8	Nonfatal Health Outcomes	Influenza
Greaves K, Chen Y, Appadurai V, Hu Z, Schofield P, Chen R. The prevalence of doctor-diagnosed angina in 4314 older adults in China and comparison with the Rose angina questionnaire: the 4 province study. Int J Cardiol. 2014; 177(2): 627Â–8	Nonfatal Health Outcomes	Ischemic heart disease
Hong Kong Cancer Registry. Hong Kong Cancer Registry Annual Tables. Kowloon, Hong Kong: Hong Kong Cancer Registry	Nonfatal Health Outcomes	Gallbladder and biliary tract cancer
Lv Y-B, Yin Z-X, Chei C-L, Qian H-Z, Kraus VB, Zhang J, Brasher MS, Shi X-M, Matchar DB, Zeng Y. Low-density lipoprotein cholesterol was inversely associated with 3-year all-cause mortality among Chinese oldest old: data from the Chinese Longitudinal Healthy Longevity Survey. Atherosclerosis. 2015; 239(1): 137Â–42	Nonfatal Health Outcomes	Diabetes mellitus
Zeng Q, He Y. Age-specific prevalence of kidney stones in Chinese urban inhabitants. Urolithiasis. 2013; 41(1): 91-3	Nonfatal Health Outcomes	Urolithiasis
Qiao M, Zhang H, Liu H, Luo S, Wang T, Zhang J, Ji L. Prevalence of premenstrual syndrome and premenstrual dysphoric disorder in a population-based sample in China. Eur J Obstet Gynecol Reprod Biol. 2012; 162(1): 83-6	Nonfatal Health Outcomes	Premenstrual syndrome
Ministry of Health (China), National Center for Chronic and Noncommunicable Disease Control and Prevention (China), World Health Organization (WHO). China WHO Study on Global AGEing and Adult Health 2007-2010	Nonfatal Health Outcomes	Drowning
Ministry of Health (China), National Center for Chronic and Noncommunicable Disease Control and Prevention (China), World Health Organization (WHO). China WHO Study on Global AGEing and Adult Health 2007-2010	Nonfatal Health Outcomes	Poisonings
Ministry of Health (China), National Center for Chronic and Noncommunicable Disease Control and Prevention (China), World Health Organization (WHO). China WHO Study on Global AGEing and Adult Health 2007-2010	Nonfatal Health Outcomes	Animal contact
Ministry of Health (China), National Center for Chronic and Noncommunicable Disease Control and Prevention (China), World Health Organization (WHO). China WHO Study on Global AGEing and Adult Health 2007-2010	Nonfatal Health Outcomes	Self-harm
Ministry of Health (China), National Center for Chronic and Noncommunicable Disease Control and Prevention (China), World Health Organization (WHO). China WHO Study on Global AGEing and Adult Health 2007-2010	Nonfatal Health Outcomes	Interpersonal violence
Liu C, Xiao G-Q, Yan L-N, Li B, Li J, Wen T-F, Wang W-T, Xu M-Q, Yang J-Y. Value of ?-fetoprotein in association with clinicopathological features of hepatocellular carcinoma . World J Gastroenterol. 2013; 19(11): 1811	Nonfatal Health Outcomes	Liver cancer due to hepatitis B
Liu C, Xiao G-Q, Yan L-N, Li B, Li J, Wen T-F, Wang W-T, Xu M-Q, Yang J-Y. Value of ?-fetoprotein in association with clinicopathological features of hepatocellular carcinoma . World J Gastroenterol. 2013; 19(11): 1811	Nonfatal Health Outcomes	Liver cancer due to hepatitis C
Wang Y, Xu Y, Li J, Wei Y, Zhao D, Hou L, Hasimu B, Yang J, Yuan H, Hu D. Characteristics of prevalence in peripheral arterial disease and correlative risk factors and comorbidities among female natural population in China. VASA. 2010; 39(4): 305Â–11	Nonfatal Health Outcomes	Peripheral artery disease
Chen Q, Li M, Li M, Lu X, LÃ¼ R, Sun J, Liu Z. Molecular basis of weak D and DEL in Han population in Anhui Province, China. Chin Med J (Engl). 2012; 125(18): 3251-5	Nonfatal Health Outcomes	Hemolytic disease and other neonatal jaundice

Li Q, Hou L, Guo Z-H, Ye L-Y, Yue D-Q, Zhu Z-Y. Molecular basis of the RHD gene in blood donors with DEL phenotypes in Shanghai. Vox Sang. 2009; 97(2): 139-46	Nonfatal Health Outcomes	Hemolytic disease and other neonatal jaundice
Liu QG, Sun J, Xiao XW, Song GR. Birth defects data from surveillance hospitals in Dalian city, China, 2006-2010. J Matern Fetal Neonatal Med. 2016; 29(22): 3615-21	Nonfatal Health Outcomes	Down syndrome
Ma Z, Feng R, Zhao C, Harrison TJ, Li M, Qiao Z, Feng Y, Wang Y. Seroprevalence and distribution of hepatitis E virus in various ethnic groups in Gansu province, China. Infect Genet Evol. 2010; 10(5): 614-9	Nonfatal Health Outcomes	Acute hepatitis E
World Health Organization (WHO). WHO Tuberculosis Case Notifications. Geneva, Switzerland: World Health Organization (WHO)	Nonfatal Health Outcomes	HIV/AIDS - Drug-susceptible Tuberculosis
Xiaoli Y, Chao J, Wen P, Wenming X, Fang L, Ning L, Huijuan M, Jun N, Ming L, Xiaoxia A, Chuanyou Y, Zenguo F, Lili L, Lianzheng Y, Lijuan T, Guowei P. Prevalence of psychiatric disorders among children and adolescents in northeast China. PLoS One. 2014; 9(10): e111223	Nonfatal Health Outcomes	Anxiety disorders
He Y, Lin GY, Wang Q, Cai XY, Zhang YH, Lin CX, Lu CD, Lu XD. A 3-year prospective study of the epidemiology of acute respiratory viral infections in hospitalized children in Shenzhen, China. Influenza Other Respir Viruses. 2014; 8(4): 443-51	Nonfatal Health Outcomes	Influenza
Liu WK, Liu Q, Chen de H, Liang HX, Chen XK, Chen MX, Qiu SY, Yang ZY, Zhou R. Epidemiology of acute respiratory infections in children in Guangzhou: a three-year study. PLoS One. 2014; 9(5): e96674	Nonfatal Health Outcomes	Influenza
Liu WK, Liu Q, Chen de H, Liang HX, Chen XK, Chen MX, Qiu SY, Yang ZY, Zhou R. Epidemiology of acute respiratory infections in children in Guangzhou: a three-year study. PLoS One. 2014; 9(5): e96674	Nonfatal Health Outcomes	Respiratory syncytial virus pneumonia
International Centre for Eye Health (ICEH). China - Inner Mongolia Tuoketuo Rapid Assessment of Avoidable Blindness 2010	Nonfatal Health Outcomes	Other vision loss
Chinese Center for Disease Control and Prevention (CCDC). China Disease Surveillance Points and Death Registration 2010 - China CDC	Nonfatal Health Outcomes	Rabies
Li EY, Liu Y, Zhan X, Liang YB, Zhang X, Zheng C, Jhanji V, Xu P, Chang DF, Lam DS. Prevalence of blindness and outcomes of cataract surgery in Hainan Province in South China. Ophthalmology. 2013; 120(11.0): 2176-83	Nonfatal Health Outcomes	Other vision loss
International Centre for Eye Health (ICEH). China - Inner Mongolia Shangdu Rapid Assessment of Avoidable Blindness 2010	Nonfatal Health Outcomes	Other vision loss
Chinese Center for Disease Control and Prevention (CCDC), Ministry of Health (China), World Health Organization (WHO). China National Tuberculosis Prevalence Survey 2010	Nonfatal Health Outcomes	HIV/AIDS - Multidrug-resistant Tuberculosis without extensive drug resistance
Zhou K-Y, Gao M-H, Yang C-H, Zhang J-N, Chen Y-Z, Song J-Z, Zhuang Y-Y, Zhang X-Y, Zhang W, Wen F-Q. An Epidemiological Survey of Attention Deficit Hyperactivity Disorder in School-age Children in Shenzhen. Chin J Contemp Pediatr. 2012; 14(9): 689-92	Nonfatal Health Outcomes	Attention-deficit/hyperactivity disorder
Chinese Center for Disease Control and Prevention (CCDC). China Notifiable Infectious Diseases 2010	Nonfatal Health Outcomes	Rabies
Chinese Center for Disease Control and Prevention (CCDC). China Chronic Disease and Risk Factor Surveillance 2010	Nonfatal Health Outcomes	Diabetes mellitus
Zhang YH, Ma WJ, Thomas GN, Xu YJ, Lao XQ, Xu XJ, Song XL, Xu HF, Cai QM, Xia L, Nie SP, Deng HH, Yu IT. Diabetes and pre-diabetes as determined by glycated haemoglobin A1c and glucose levels in a developing southern Chinese population. PLoS One. 2012; 7(5): e37260	Nonfatal Health Outcomes	Diabetes mellitus
Lao XQ, Ma WJ, Sobko T, Zhang YH, Xu YJ, Xu XJ, Yu DM, Nie SP, Cai QM, Wei XL, Xia L, Wong MC-S. Dramatic escalation in metabolic syndrome and cardiovascular risk in a Chinese population experiencing rapid economic development. BMC Public Health. 2014; 14: 983	Nonfatal Health Outcomes	Diabetes mellitus
Chinese Center for Disease Control and Prevention (CCDC), Ministry of Health (China), World Health Organization (WHO). China National Tuberculosis Prevalence Survey 2010	Nonfatal Health Outcomes	HIV/AIDS - Drug-susceptible Tuberculosis
Chinese Center for Disease Control and Prevention (CCDC), Ministry of Health (China). China National Injury Surveillance System 2010 - China CDC	Nonfatal Health Outcomes	Animal contact
Chinese Center for Disease Control and Prevention (CCDC), Ministry of Health (China), World Health Organization (WHO). China National Tuberculosis Prevalence Survey 2010	Nonfatal Health Outcomes	Multidrug-resistant tuberculosis without extensive drug resistance
Chinese Center for Disease Control and Prevention (CCDC), Ministry of Health (China). China National Injury Surveillance System 2010 - China CDC	Nonfatal Health Outcomes	Other exposure to mechanical forces
Chinese Center for Disease Control and Prevention (CCDC), Ministry of Health (China). China National Injury Surveillance System 2010 - China CDC	Nonfatal Health Outcomes	Road injuries
International Centre for Eye Health (ICEH). China - Inner Mongolia Tuoketuo Rapid Assessment of Avoidable Blindness 2010	Nonfatal Health Outcomes	Refraction and accommodation disorders
Chinese Center for Disease Control and Prevention (CCDC), Ministry of Health (China). China National Injury Surveillance System 2010 - China CDC	Nonfatal Health Outcomes	Falls

National Central Cancer Registry (China). China Cancer Registry Incidence and Mortality 2011	Nonfatal Health Outcomes	Other leukemia
Chinese Center for Disease Control and Prevention (CCDC), Ministry of Health (China). China National Injury Surveillance System 2010 - China CDC	Nonfatal Health Outcomes	Fire, heat, and hot substances
Chinese Center for Disease Control and Prevention (CCDC), Ministry of Health (China). China National Injury Surveillance System 2010 - China CDC	Nonfatal Health Outcomes	Poisonings
Chinese Center for Disease Control and Prevention (CCDC), Ministry of Health (China). China National Injury Surveillance System 2010 - China CDC	Nonfatal Health Outcomes	Exposure to mechanical forces
Chinese Center for Disease Control and Prevention (CCDC), Ministry of Health (China). China National Injury Surveillance System 2010 - China CDC	Nonfatal Health Outcomes	Interpersonal violence
International Centre for Eye Health (ICEH). China - Inner Mongolia Tuoketuo Rapid Assessment of Avoidable Blindness 2010	Nonfatal Health Outcomes	Age-related macular degeneration
Chinese Center for Disease Control and Prevention (CCDC), Ministry of Health (China). China National Injury Surveillance System 2010 - China CDC	Nonfatal Health Outcomes	Foreign body in other body part
Chinese Center for Disease Control and Prevention (CCDC), Ministry of Health (China). China National Injury Surveillance System 2010 - China CDC	Nonfatal Health Outcomes	Self-harm
Chinese Center for Disease Control and Prevention (CCDC), Ministry of Health (China), World Health Organization (WHO). China National Tuberculosis Prevalence Survey 2010	Nonfatal Health Outcomes	Extensively drug-resistant tuberculosis
Chinese Center for Disease Control and Prevention (CCDC), Ministry of Health (China), World Health Organization (WHO). China National Tuberculosis Prevalence Survey 2010	Nonfatal Health Outcomes	HIV/AIDS - Extensively drug-resistant Tuberculosis
Chinese Center for Disease Control and Prevention (CCDC), Ministry of Health (China). China National Injury Surveillance System 2010 - China CDC	Nonfatal Health Outcomes	Physical violence by sharp object
Chinese Center for Disease Control and Prevention (CCDC), Ministry of Health (China). China National Injury Surveillance System 2010 - China CDC	Nonfatal Health Outcomes	Pulmonary aspiration and foreign body in airway
Chinese Center for Disease Control and Prevention (CCDC), Ministry of Health (China). China National Injury Surveillance System 2010 - China CDC	Nonfatal Health Outcomes	Venomous animal contact
Chinese Center for Disease Control and Prevention (CCDC). China Notifiable Infectious Diseases 2010	Nonfatal Health Outcomes	Syphilis
Chinese Center for Disease Control and Prevention (CCDC), Ministry of Health (China). China National Injury Surveillance System 2010 - China CDC	Nonfatal Health Outcomes	Other transport injuries
Chinese Center for Disease Control and Prevention (CCDC), Ministry of Health (China). China National Injury Surveillance System 2010 - China CDC	Nonfatal Health Outcomes	Unintentional firearm injuries
Chinese Center for Disease Control and Prevention (CCDC). China Injury Comprehensive Surveillance Study 2009-2011 - China CDC	Nonfatal Health Outcomes	Falls
Chinese Center for Disease Control and Prevention (CCDC), Ministry of Health (China). China National Injury Surveillance System 2010 - China CDC	Nonfatal Health Outcomes	Non-venomous animal contact
Chinese Center for Disease Control and Prevention (CCDC), Ministry of Health (China). China National Injury Surveillance System 2010 - China CDC	Nonfatal Health Outcomes	Drowning
Chinese Center for Disease Control and Prevention (CCDC). China Injury Comprehensive Surveillance Study 2009-2011 - China CDC	Nonfatal Health Outcomes	Drowning
Chinese Center for Disease Control and Prevention (CCDC), Ministry of Health (China). China National Injury Surveillance System 2010 - China CDC	Nonfatal Health Outcomes	Physical violence by firearm
Chinese Center for Disease Control and Prevention (CCDC). China Injury Comprehensive Surveillance Study 2009-2011 - China CDC	Nonfatal Health Outcomes	Fire, heat, and hot substances
International Centre for Eye Health (ICEH). China - Inner Mongolia Shangdu Rapid Assessment of Avoidable Blindness 2010	Nonfatal Health Outcomes	Refraction and accommodation disorders
Chinese Center for Disease Control and Prevention (CCDC), Ministry of Health (China). China National Injury Surveillance System 2010 - China CDC	Nonfatal Health Outcomes	Physical violence by other means
Chinese Center for Disease Control and Prevention (CCDC). China Injury Comprehensive Surveillance Study 2009-2011 - China CDC	Nonfatal Health Outcomes	Poisonings
Chinese Center for Disease Control and Prevention (CCDC). China Injury Comprehensive Surveillance Study 2009-2011 - China CDC	Nonfatal Health Outcomes	Exposure to mechanical forces
Chinese Center for Disease Control and Prevention (CCDC), Ministry of Health (China). China National Injury Surveillance System 2010 - China CDC	Nonfatal Health Outcomes	Adverse effects of medical treatment
Chinese Center for Disease Control and Prevention (CCDC), Ministry of Health (China), World Health Organization (WHO). China National Tuberculosis Prevalence Survey 2010	Nonfatal Health Outcomes	Drug-susceptible tuberculosis
Chinese Center for Disease Control and Prevention (CCDC). China Injury Comprehensive Surveillance Study 2009-2011 - China CDC	Nonfatal Health Outcomes	Animal contact
Chinese Center for Disease Control and Prevention (CCDC). China Injury Comprehensive Surveillance Study 2009-2011 - China CDC	Nonfatal Health Outcomes	Pulmonary aspiration and foreign body in airway
Chinese Center for Disease Control and Prevention (CCDC). China Injury Comprehensive Surveillance Study 2009-2011 - China CDC	Nonfatal Health Outcomes	Self-harm

Chinese Center for Disease Control and Prevention (CCDC). China Injury Comprehensive Surveillance Study 2009-2011 - China CDC	Nonfatal Health Outcomes	Interpersonal violence
Li H, Wei Q, Tan A, Wang L. Epidemiological analysis of respiratory viral etiology for influenza-like illness during 2010 in Zhuhai, China. Virol J. 2013; 143	Nonfatal Health Outcomes	Respiratory syncytial virus pneumonia
Li Keqing, Jiang Qinpu, Cui Lijun. Epidemiological Investigation of Alzheimer Disease and Rural Areas in Hebei Province. China J Health Psychol. 2008; 16(11): 1251-3	Nonfatal Health Outcomes	Alzheimer's disease and other dementias
Kang M, Gao YM, Huo HQ. Prevalence of Alzheimer's disease and its related factors among elderly people in Hebei province. Chin J Public Health. 2011; 27(9): 1123-5	Nonfatal Health Outcomes	Alzheimer's disease and other dementias
Chen H-M, Guo H, Pan H-J, Zou Y-J, Luo A-J, Zhu H-Y, Liu X-J, Sun X-L. Qinhuangdao Epidemiological Survey of 18 Years of Age and People Over Mental Illness. Shanxi Med J. 2012; 41(21): 1191-4	Nonfatal Health Outcomes	Schizophrenia
Ministry of Health (China), National Center for Chronic and Noncommunicable Disease Control and Prevention (China), World Health Organization (WHO). China WHO Study on Global AGEing and Adult Health 2007-2010	Nonfatal Health Outcomes	Chronic obstructive pulmonary disease
Chen H-M, Guo H, Pan H-J, Zou Y-J, Luo A-J, Zhu H-Y, Liu X-J, Sun X-L. Qinhuangdao Epidemiological Survey of 18 Years of Age and People Over Mental Illness. Shanxi Med J. 2012; 41(21): 1191-4	Nonfatal Health Outcomes	Anxiety disorders
Li H, Wei Q, Tan A, Wang L. Epidemiological analysis of respiratory viral etiology for influenza-like illness during 2010 in Zhuhai, China. Virol J. 2013; 143	Nonfatal Health Outcomes	Influenza
National Central Cancer Registry (China). China Cancer Registry Incidence and Mortality 2011	Nonfatal Health Outcomes	Nasopharynx cancer
National Central Cancer Registry (China). China Cancer Registry Incidence and Mortality 2011	Nonfatal Health Outcomes	Multiple myeloma
National Central Cancer Registry (China). China Cancer Registry Incidence and Mortality 2011	Nonfatal Health Outcomes	Leukemia
Li X, Zhou Q, Sun L, Wang Z, Han S, Wu S, Wang N. Prevalence of blindness and low vision in a rural population in northern China: preliminary results from a population-based survey. Ophthalmic Epidemiol. 2012; 19(5): 272-7	Nonfatal Health Outcomes	Refraction and accommodation disorders
Chinese Center for Disease Control and Prevention (CCDC), Ministry of Health (China). China National Injury Surveillance System 2010 - China CDC	Nonfatal Health Outcomes	Pedestrian road injuries
Chinese Center for Disease Control and Prevention (CCDC), Ministry of Health (China). China National Injury Surveillance System 2010 - China CDC	Nonfatal Health Outcomes	Cyclist road injuries
Chinese Center for Disease Control and Prevention (CCDC), Ministry of Health (China). China National Injury Surveillance System 2010 - China CDC	Nonfatal Health Outcomes	Motorcyclist road injuries
Chinese Center for Disease Control and Prevention (CCDC), Ministry of Health (China). China National Injury Surveillance System 2010 - China CDC	Nonfatal Health Outcomes	Motor vehicle road injuries
Chinese Center for Disease Control and Prevention (CCDC). China Injury Comprehensive Surveillance Study 2009-2011 - China CDC	Nonfatal Health Outcomes	Venomous animal contact
Chinese Center for Disease Control and Prevention (CCDC). China Injury Comprehensive Surveillance Study 2009-2011 - China CDC	Nonfatal Health Outcomes	Non-venomous animal contact
Chinese Center for Disease Control and Prevention (CCDC), Ministry of Health (China). China National Injury Surveillance System 2010 - China CDC	Nonfatal Health Outcomes	Foreign body in eyes
Chinese Center for Disease Control and Prevention (CCDC). China Notifiable Infectious Diseases 2010	Nonfatal Health Outcomes	Chlamydial infection
World Health Organization (WHO). Leprosy update, 2011. Wkly Epidemiol Rec. 2011: 86(36): 389-99.	Nonfatal Health Outcomes	Leprosy
Li LW, Wong HM, McGrath CP. Longitudinal Association between Obesity and Dental Caries in Adolescents. J Pediatr. 2017; 189: 149-154e5	Nonfatal Health Outcomes	Caries of permanent teeth
Chinese Center for Disease Control and Prevention (CCDC). China Notifiable Infectious Diseases 2010	Nonfatal Health Outcomes	Sexually transmitted infections excluding HIV
Li EY, Liu Y, Zhan X, Liang YB, Zhang X, Zheng C, Jhanji V, Xu P, Chang DF, Lam DS. Prevalence of blindness and outcomes of cataract surgery in Hainan Province in South China. Ophthalmology. 2013; 120(11.0): 2176-83	Nonfatal Health Outcomes	Glaucoma
Li EY, Liu Y, Zhan X, Liang YB, Zhang X, Zheng C, Jhanji V, Xu P, Chang DF, Lam DS. Prevalence of blindness and outcomes of cataract surgery in Hainan Province in South China. Ophthalmology. 2013; 120(11.0): 2176-83	Nonfatal Health Outcomes	Cataract
Li EY, Liu Y, Zhan X, Liang YB, Zhang X, Zheng C, Jhanji V, Xu P, Chang DF, Lam DS. Prevalence of blindness and outcomes of cataract surgery in Hainan Province in South China. Ophthalmology. 2013; 120(11.0): 2176-83	Nonfatal Health Outcomes	Age-related macular degeneration
Li EY, Liu Y, Zhan X, Liang YB, Zhang X, Zheng C, Jhanji V, Xu P, Chang DF, Lam DS. Prevalence of blindness and outcomes of cataract surgery in Hainan Province in South China. Ophthalmology. 2013; 120(11.0): 2176-83	Nonfatal Health Outcomes	Refraction and accommodation disorders
Zhang J, Song L, Wei J, Zhang A, Dong H, Wen H, Luo J, Liu G. Prevalence of and risk factors for the occurrence of symptomatic osteoarthritis in rural regions of Shanxi Province, China. Int J Rheum Dis. 2016; 19(8): 781-789	Nonfatal Health Outcomes	Osteoarthritis

Zhang J, Song L, Liu G, Zhang A, Dong H, Liu Z, Li X, Luo J. Risk factors for and prevalence of knee osteoarthritis in the rural areas of Shanxi Province, North China: a COPCORD study. Rheumatol Int. 2013; 33(11): 2783-2788	Nonfatal Health Outcomes	Osteoarthritis
Pew Research Center. The Future of the Global Muslim Population. Washington, DC, United States: Pew Research Center, 2011	Nonfatal Health Outcomes	Cysticercosis
World Health Organization Regional Office for the Western Pacific (WPRO-WHO). Number of Reported Cases of Dengue Fever and Dengue Hemorrhagic Fever in the Western Pacific Region, by Country 2010. Manila, Philippines: World Health Organization Regional Office for the Western Pacific (WPRO-WHO)	Nonfatal Health Outcomes	Dengue
Yuan J, Qu, Z-F, Jiang Q, Yang Z-D, Bo W-Z. The epidemiological survey of depression disorder and cognitive disorder of the elderly in Shanghai Pudong community. J Clin Psychiatry. 2013; 23(3): 86-88	Nonfatal Health Outcomes	Bipolar disorder
Li J, Zhong H, Cai N, Luo T, Li J, Su X, Li X, Qiu X, Yang Y, Yuan Y, Yu M. The prevalence and causes of visual impairment in an elderly Chinese Bai ethnic rural population: the Yunnan minority eye study. Invest Ophthalmol Vis Sci. 2012; 53(8): 4498-504	Nonfatal Health Outcomes	Glaucoma
Li J, Zhong H, Cai N, Luo T, Li J, Su X, Li X, Qiu X, Yang Y, Yuan Y, Yu M. The prevalence and causes of visual impairment in an elderly Chinese Bai ethnic rural population: the Yunnan minority eye study. Invest Ophthalmol Vis Sci. 2012; 53(8): 4498-504	Nonfatal Health Outcomes	Age-related macular degeneration
Li H, Wei Q, Tan A, Wang L. Epidemiological analysis of respiratory viral etiology for influenza-like illness during 2010 in Zhuhai, China. Virol J. 2013; 143	Nonfatal Health Outcomes	Lower respiratory infections
Li J, Zhong H, Cai N, Luo T, Li J, Su X, Li X, Qiu X, Yang Y, Yuan Y, Yu M. The prevalence and causes of visual impairment in an elderly Chinese Bai ethnic rural population: the Yunnan minority eye study. Invest Ophthalmol Vis Sci. 2012; 53(8): 4498-504	Nonfatal Health Outcomes	Cataract
Li J, Zhong H, Cai N, Luo T, Li J, Su X, Li X, Qiu X, Yang Y, Yuan Y, Yu M. The prevalence and causes of visual impairment in an elderly Chinese Bai ethnic rural population: the Yunnan minority eye study. Invest Ophthalmol Vis Sci. 2012; 53(8): 4498-504	Nonfatal Health Outcomes	Refraction and accommodation disorders
Chinese Center for Disease Control and Prevention (CCDC). China Notifiable Infectious Diseases 2010	Nonfatal Health Outcomes	Gonococcal infection
International Centre for Eye Health (ICEH). China - Inner Mongolia Shangdu Rapid Assessment of Avoidable Blindness 2010	Nonfatal Health Outcomes	Diabetes mellitus
International Centre for Eye Health (ICEH). China - Inner Mongolia Tuoketuo Rapid Assessment of Avoidable Blindness 2010	Nonfatal Health Outcomes	Diabetes mellitus
Wang W-Q, Ding L-J, Wen C, Liao Z-H, Hong X, Chen Y, et al. Epidemiological Survey of Mental Disorders in People aged 18 years and older in Xiamen City. Chin J Psychiatry. 2013; 46(1): 43-9	Nonfatal Health Outcomes	Schizophrenia
Chen X, Cheng HG, Huang Y, Liu Z, Luo X. Depression symptoms and chronic pain in the community population in Beijing, China. Psychiatry Res. 2012; 200(2-3): 313-7	Nonfatal Health Outcomes	Major depressive disorder
Sun X-R, Qu Z-W, Jiang Q, Yuan J. Min J, Qin H-Y. Investigation into the epidemiology of severe depression in a commmunity in Pudong New area Shanghai Province. Med J Chin Peoples Health. 2011; 23(9): 1078-80	Nonfatal Health Outcomes	Major depressive disorder
Qu Z-W, Yang Z-D, Jiang Q, Yuan J, Qin J, Tang Q, Lu Y, Sun Y-Y, Xi Y, Min J, Liu Y-J, Lu W, Qin H-Y, Pei Y, Wang H-J. Epidemological Characteristics of Anxiety Disorders in Elderly in Pudong New District, Shanghai: A Community-based Study. Chin J Clin (Electron Edit). 2011; 5(5): 1346-50	Nonfatal Health Outcomes	Anxiety disorders
International Centre for Eye Health (ICEH). China - Inner Mongolia Shangdu Rapid Assessment of Avoidable Blindness 2010	Nonfatal Health Outcomes	Glaucoma
International Centre for Eye Health (ICEH). China - Inner Mongolia Tuoketuo Rapid Assessment of Avoidable Blindness 2010	Nonfatal Health Outcomes	Glaucoma
International Centre for Eye Health (ICEH). China - Inner Mongolia Shangdu Rapid Assessment of Avoidable Blindness 2010	Nonfatal Health Outcomes	Cataract
International Centre for Eye Health (ICEH). China - Inner Mongolia Tuoketuo Rapid Assessment of Avoidable Blindness 2010	Nonfatal Health Outcomes	Cataract
International Centre for Eye Health (ICEH). China - Inner Mongolia Shangdu Rapid Assessment of Avoidable Blindness 2010	Nonfatal Health Outcomes	Age-related macular degeneration
Zhao X, Zhu X, Zhang H, Zhao W, Li J, Shu Y, Li S, Yang M, Cai L, Zhou J, Li Y. Prevalence of diabetes and predictions of its risks using anthropometric measures in southwest rural areas of China. BMC Public Health. 2012; 821	Nonfatal Health Outcomes	Diabetes mellitus
Liu J, Yan F, Ma X, Guo H-L, Tang Y-L, Rakofsky JJ, Wu X-M, Li X-Q, Zhu H, Guo X-B, Yang Y, Li P, Cao X-D, Li H-Y, Li Z-B, Wang P, Xu Q-Y. Prevalence of major depressive disorder and socio-demographic correlates: Results of a representative household epidemiological survey in Beijing, China. J Affect Disord. 2015; 179: 74Â–81	Nonfatal Health Outcomes	Major depressive disorder

Liu Z, Huang Y, Chen X, Cheng H, Luo X. The prevalence of mood disorder, anxiety disorder and substance use disorder in community residents in Beijing: A cross-sectional study. Chin Ment Health J. 2013; 27(2): 102-10	Nonfatal Health Outcomes	Anxiety disorders
Han L-T, Han J-Y, Huang G-Y, Li J-W. Epidemiological investigation on adhd in Liaoyang city, Liaoning. Med J Chin Peoples Health. 2011; 23(7): 883-7	Nonfatal Health Outcomes	Attention-deficit/hyperactivity disorder
Liu Y, Zhang H, Liang N, Fan W, Li J, Huang Z, Yin Z, Wu Z, Hu J. Prevalence and associated factors of knee osteoarthritis in a rural Chinese adult population: an epidemiological survey. BMC Public Health. 2016; 16: 94	Nonfatal Health Outcomes	Osteoarthritis
Wang Feng, Xu Pin, Zhu Sihui. Survey on the awareness rate and prevalence of Alzheimer's disease in the urban area of Zaozhuang City. Chin J Practical Nerv Dis. 2010; 13(23): 9-11	Nonfatal Health Outcomes	Alzheimer's disease and other dementias
Xu Y, Wang L, He J, Bi Y, Li M, Wang T, Wang L, Jiang Y, Dai M, Lu J, Xu M, Li Y, Hu N, Li J, Mi S, Chen CS, Li G, Mu Y, Zhao J, Kong L, Chen J, Lai S, Wang W, Zhao W, Ning G. Prevalence and control of diabetes in Chinese adults. JAMA. 2013; 310(9): 948-59	Nonfatal Health Outcomes	Diabetes mellitus
Sun L, Yue H, Sun B, Han L, Qi M, Tian Z, Lu S, Shan C, Luo J, Fan Y, Li S, Dong M, Zuo X, Zhang Y, Lin W, Xu J, Heng Y, Huai'an Perinatal-Neonatal Study Group. Estimation of birth population-based perinatal-neonatal mortality and preterm rate in China from a regional survey in 2010. J Matern Fetal Neonatal Med. 2013; 26(16): 1641-8	Nonfatal Health Outcomes	Neonatal sepsis and other neonatal infections
Li Z, Zhu L, Zhang B, Si Y, Liu M. [Periodontal health status assessed by community periodontal index and related factors in adult population of Beijing urban community]. J Peking Univ Health Sci. 2012; 44(1): 130-4	Nonfatal Health Outcomes	Edentulism and severe tooth loss
Chinese Center for Disease Control and Prevention (CCDC). China Injury Comprehensive Surveillance Study 2009-2011 - China CDC	Nonfatal Health Outcomes	Injuries
Bhutani VK, Zipursky A, Blencowe H, Khanna R, Sgro M, Ebbesen F, Bell J, Mori R, Slusher TM, Fahmy N, Paul VK, Du L, Okolo AA, de Almeida MF, Olusanya BO, Kumar P, Cousens S, Lawn JE. Neonatal hyperbilirubinemia and Rhesus disease of the newborn: incidence and impairment estimates for 2010 at regional and global levels. Pediatr Res. 2013; 74(Suppl 1): 86-100	Nonfatal Health Outcomes	Hemolytic disease and other neonatal jaundice
Chinese Center for Disease Control and Prevention (CCDC), Ministry of Health (China). China National Injury Surveillance System 2010 - China CDC	Nonfatal Health Outcomes	Injuries
Wei X, Zhang X, Yin J, Walley J, Beanland R, Zou G, Zhang H, Li F, Liu Z, Zee BCY, Griffiths SM. Changes in pulmonary tuberculosis prevalence: evidence from the 2010 population survey in a populous province of China. BMC Infect Dis. 2014; 21	Nonfatal Health Outcomes	Tuberculosis
Cao Z, Wang T, Zhang S, Sha J, Huang H, Zhu L. [Risk evaluation of schistosomiasis japonica input to potential endemic areas in Anhui province]. Chin J Prev Med. 2014; 48(1): 58Â–61	Nonfatal Health Outcomes	Schistosomiasis
Chinese Center for Disease Control and Prevention (CCDC), Ministry of Health (China). China National Injury Surveillance System 2010 - China CDC	Nonfatal Health Outcomes	Other road injuries
Chinese Center for Disease Control and Prevention (CCDC), Ministry of Health (China). China National Injury Surveillance System 2013	Nonfatal Health Outcomes	Animal contact
Chinese Center for Disease Control and Prevention (CCDC). China Disease Surveillance Points and Death Registration 2011 - China CDC	Nonfatal Health Outcomes	Rabies
Chinese Center for Disease Control and Prevention (CCDC). China Disease Surveillance Points 2013 - China CDC	Nonfatal Health Outcomes	Rabies
Center for Health Statistics and Information, National Health and Family Planning Commission (China), Shanghai Municipal Center for Disease Control and Prevention (Shanghai CDC), Shanghai Health Information Center. China Hospital Inpatient Data 2013-2016	Nonfatal Health Outcomes	Diarrheal diseases
Liu L, Yang G-J, Zhu H-R, Yang K, Ai L. Knowledge of, attitudes towards, and practice relating to schistosomiasis in two subtypes of a mountainous region of the PeopleÂ’s Republic of China. Infect Dis Poverty. 2014; 3(1)	Nonfatal Health Outcomes	Schistosomiasis
Center for Health Statistics and Information, National Health and Family Planning Commission (China), Shanghai Municipal Center for Disease Control and Prevention (Shanghai CDC), Shanghai Health Information Center. China Hospital Inpatient Data 2013-2016	Nonfatal Health Outcomes	Pelvic inflammatory disease
National Central Cancer Registry (China). China Cancer Registry Incidence and Mortality 2011	Nonfatal Health Outcomes	Non-melanoma skin cancer (basal-cell carcinoma)
Chinese Center for Disease Control and Prevention (CCDC), Ministry of Health (China). China National Injury Surveillance System 2011 - China CDC	Nonfatal Health Outcomes	Exposure to mechanical forces
Chinese Center for Disease Control and Prevention (CCDC). China Disease Surveillance Points and Death Registration 2012 - China CDC	Nonfatal Health Outcomes	Rabies
National Central Cancer Registry (China). China Cancer Registry Incidence and Mortality 2011	Nonfatal Health Outcomes	Non-melanoma skin cancer (squamous-cell carcinoma)
Chinese Center for Disease Control and Prevention (CCDC). China Chronic Disease and Risk Factor Surveillance 2013	Nonfatal Health Outcomes	Diabetes mellitus

Chinese Center for Disease Control and Prevention (CCDC). China Notifiable Infectious Diseases 2011	Nonfatal Health Outcomes	Rabies
Weng J, Zhou Z, Guo L, Zhu D, Ji L, Luo X, Mu Y, Jia W. Incidence of type 1 diabetes in China, 2010-13: population based study. BMJ. 2018; 360: j5295	Nonfatal Health Outcomes	Diabetes mellitus
Chinese Center for Disease Control and Prevention (CCDC). China Notifiable Infectious Diseases 2013 - China CDC	Nonfatal Health Outcomes	Rabies
United Nations Population Division. World Abortion Policies 2013. New York, United States: United Nations (UN), 2013	Nonfatal Health Outcomes	Maternal abortion and miscarriage
Chinese Center for Disease Control and Prevention (CCDC), Ministry of Health (China). China National Injury Surveillance System 2011 - China CDC	Nonfatal Health Outcomes	Foreign body in eyes
Carter Center, International Trachoma Initiative, London School of Hygiene and Tropical Medicine. Global Atlas of Trachoma. Decatur, United States: International Trachoma Initiative	Nonfatal Health Outcomes	Trachoma
Chinese Center for Disease Control and Prevention (CCDC), Ministry of Health (China). China National Injury Surveillance System 2012 - China CDC	Nonfatal Health Outcomes	Other exposure to mechanical forces
Chinese Center for Disease Control and Prevention (CCDC), Ministry of Health (China). China National Injury Surveillance System 2012 - China CDC	Nonfatal Health Outcomes	Physical violence by sharp object
Chinese Center for Disease Control and Prevention (CCDC), Ministry of Health (China). China National Injury Surveillance System 2011 - China CDC	Nonfatal Health Outcomes	Animal contact
Center for Health Statistics and Information, National Health and Family Planning Commission (China), Shanghai Municipal Center for Disease Control and Prevention (Shanghai CDC), Shanghai Health Information Center. China Hospital Inpatient Data 2013-2016	Nonfatal Health Outcomes	Lower respiratory infections
Chinese Center for Disease Control and Prevention (CCDC), Ministry of Health (China). China National Injury Surveillance System 2012 - China CDC	Nonfatal Health Outcomes	Animal contact
National Central Cancer Registry (China). China Cancer Registry Incidence and Mortality 2011	Nonfatal Health Outcomes	Prostate cancer
National Central Cancer Registry (China). China Cancer Registry Incidence and Mortality 2011	Nonfatal Health Outcomes	Non-Hodgkin lymphoma
Chinese Center for Disease Control and Prevention (CCDC), Ministry of Health (China). China National Injury Surveillance System 2013	Nonfatal Health Outcomes	Pulmonary aspiration and foreign body in airway
Li QH, Gao WJ, Li JY, Shi LA, Hao XJ, Ge SW, An SH. [Detection of respiratory viruses in children with acute lower respiratory tract infection: an analysis of 5,150 children]. Chin J Contemp Pediatr. 2016; 18(1): 51-4	Nonfatal Health Outcomes	Influenza
Ng SC, Tang W, Ching JY, Wong M, Chow CM, Hui AJ, Wong TC, Leung VK, Tsang SW, Yu HH, Li MF, Ng KK, Kamm MA, Studd C, Bell S, Leong R, de Silva HJ, Kasturiratne A, Mufeena MNF, Ling KL, Ooi CJ, Tan PS, Ong D, Goh KL, Hilmi I, Pisespongsa P, Manatsathit S, Rerknimitr R, Aniwan S, Wang YF, Ouyang Q, Zeng Z, Zhu Z, Chen MH, Hu PJ, Wu K, Wang X, Simadibrata M, Abdullah M, Wu JC, Sung JJY, Chan FKL, AsiaÂ–Pacific CrohnÂ's and Colitis Epidemiologic Study (ACCESS) Study Group. Incidence and phenotype of inflammatory bowel disease based on results from the Asia-pacific CrohnÂ's and colitis epidemiology study. Gastroenterology. 2013; 145(1): 158Â–65e2	Nonfatal Health Outcomes	Inflammatory bowel disease
National Central Cancer Registry (China). China Cancer Registry Incidence and Mortality 2011	Nonfatal Health Outcomes	Esophageal cancer
Li QH, Gao WJ, Li JY, Shi LA, Hao XJ, Ge SW, An SH. [Detection of respiratory viruses in children with acute lower respiratory tract infection: an analysis of 5,150 children]. Chin J Contemp Pediatr. 2016; 18(1): 51-4	Nonfatal Health Outcomes	Respiratory syncytial virus pneumonia
Chinese Center for Disease Control and Prevention (CCDC), Ministry of Health (China). China National Injury Surveillance System 2011 - China CDC	Nonfatal Health Outcomes	Road injuries
Chinese Center for Disease Control and Prevention (CCDC), Ministry of Health (China). China National Injury Surveillance System 2012 - China CDC	Nonfatal Health Outcomes	Road injuries
Chinese Center for Disease Control and Prevention (CCDC), Ministry of Health (China). China National Injury Surveillance System 2011 - China CDC	Nonfatal Health Outcomes	Falls
Chinese Center for Disease Control and Prevention (CCDC), Ministry of Health (China). China National Injury Surveillance System 2012 - China CDC	Nonfatal Health Outcomes	Falls
Chinese Center for Disease Control and Prevention (CCDC), Ministry of Health (China). China National Injury Surveillance System 2012 - China CDC	Nonfatal Health Outcomes	Drowning
Chinese Center for Disease Control and Prevention (CCDC), Ministry of Health (China). China National Injury Surveillance System 2012 - China CDC	Nonfatal Health Outcomes	Fire, heat, and hot substances
Chinese Center for Disease Control and Prevention (CCDC), Ministry of Health (China). China National Injury Surveillance System 2011 - China CDC	Nonfatal Health Outcomes	Fire, heat, and hot substances
Chinese Center for Disease Control and Prevention (CCDC), Ministry of Health (China). China National Injury Surveillance System 2012 - China CDC	Nonfatal Health Outcomes	Poisonings
Chinese Center for Disease Control and Prevention (CCDC), Ministry of Health (China). China National Injury Surveillance System 2012 - China CDC	Nonfatal Health Outcomes	Exposure to mechanical forces

Yang H, Wang Q, Zheng L, Zhan XF, Lin M, Lin F, Tong X, Luo ZY, Huang Y, Yang LY. Incidence and molecular characterization of Glucose-6-Phosphate Dehydrogenase deficiency among neonates for newborn screening in Chaozhou, China. Int J Lab Hematol. 2015; 37(3): 410-9	Nonfatal Health Outcomes	G6PD deficiency
Peng Q, Li S, Ma K, Li W, Ma Q, He X, He Y, He T, Lu X. Large cohort screening of G6PD deficiency and the mutational spectrum in the Dongguan District in Southern China. PLoS One. 2015; 10(3): e0120683	Nonfatal Health Outcomes	G6PD deficiency
Chinese Center for Disease Control and Prevention (CCDC), Ministry of Health (China). China National Injury Surveillance System 2013	Nonfatal Health Outcomes	Self-harm
Chinese Center for Disease Control and Prevention (CCDC), Ministry of Health (China). China National Injury Surveillance System 2012 - China CDC	Nonfatal Health Outcomes	Pulmonary aspiration and foreign body in airway
Chinese Center for Disease Control and Prevention (CCDC), Ministry of Health (China). China National Injury Surveillance System 2011 - China CDC	Nonfatal Health Outcomes	Self-harm
Chinese Center for Disease Control and Prevention (CCDC), Ministry of Health (China). China National Injury Surveillance System 2012 - China CDC	Nonfatal Health Outcomes	Self-harm
Chinese Center for Disease Control and Prevention (CCDC), Ministry of Health (China). China National Injury Surveillance System 2011 - China CDC	Nonfatal Health Outcomes	Foreign body in other body part
Chinese Center for Disease Control and Prevention (CCDC), Ministry of Health (China). China National Injury Surveillance System 2012 - China CDC	Nonfatal Health Outcomes	Foreign body in other body part
National Central Cancer Registry (China). China Cancer Registry Incidence and Mortality 2011	Nonfatal Health Outcomes	Gallbladder and biliary tract cancer
Chinese Center for Disease Control and Prevention (CCDC), Ministry of Health (China). China National Injury Surveillance System 2012 - China CDC	Nonfatal Health Outcomes	Foreign body in eyes
Chinese Center for Disease Control and Prevention (CCDC). China Notifiable Infectious Diseases 2012	Nonfatal Health Outcomes	Rabies
Chinese Center for Disease Control and Prevention (CCDC), Ministry of Health (China). China National Injury Surveillance System 2011 - China CDC	Nonfatal Health Outcomes	Interpersonal violence
Chinese Center for Disease Control and Prevention (CCDC), Ministry of Health (China). China National Injury Surveillance System 2012 - China CDC	Nonfatal Health Outcomes	Interpersonal violence
Zeng S-Y, Gong Y, Zhang Y-P, Chen S-B, Chen J-Y, Lin C-Q, Peng J-H, Hou Z-D, Zhong J-Q, Liang H-J, Huang G-H, Wang D-M, Lai H-Y, Li L-P, Zeng QY. Changes in the Prevalence of Rheumatic Diseases in Shantou, China, in the Past Three Decades: A COPCORD Study. PLoS One. 2015; 10(9): e0138492	Nonfatal Health Outcomes	Rheumatoid arthritis
Zeng S-Y, Gong Y, Zhang Y-P, Chen S-B, Chen J-Y, Lin C-Q, Peng J-H, Hou Z-D, Zhong J-Q, Liang H-J, Huang G-H, Wang D-M, Lai H-Y, Li L-P, Zeng QY. Changes in the Prevalence of Rheumatic Diseases in Shantou, China, in the Past Three Decades: A COPCORD Study. PLoS One. 2015; 10(9): e0138492	Nonfatal Health Outcomes	Osteoarthritis
Tang X, Wang S, Zhan S, Niu J, Tao K, Zhang Y, Lin J. The Prevalence of Symptomatic Knee Osteoarthritis in China: Results From the China Health and Retirement Longitudinal Study. Arthritis Rheumatol. 2016; 68(3): 648-53	Nonfatal Health Outcomes	Osteoarthritis
Chinese Center for Disease Control and Prevention (CCDC), Ministry of Health (China). China National Injury Surveillance System 2013	Nonfatal Health Outcomes	Foreign body in other body part
China Center for Economic Research, Peking University. China Health and Retirement Longitudinal Study Pilot Resurvey 2012. Beijing, China: China Center for Economic Research, Peking University	Nonfatal Health Outcomes	Cutaneous and mucocutaneous leishmaniasis
National Central Cancer Registry (China). China Cancer Registry Incidence and Mortality 2011	Nonfatal Health Outcomes	Pancreatic cancer
National Central Cancer Registry (China). China Cancer Registry Incidence and Mortality 2011	Nonfatal Health Outcomes	Liver cancer
Chinese Center for Disease Control and Prevention (CCDC), Ministry of Health (China). China National Injury Surveillance System 2013	Nonfatal Health Outcomes	Other unintentional injuries
International Centre for Eye Health (ICEH). China - Inner Mongolia Tuoketuo Rapid Assessment of Avoidable Blindness 2010	Nonfatal Health Outcomes	Refraction disorders
National Central Cancer Registry (China). China Cancer Registry Incidence and Mortality 2011	Nonfatal Health Outcomes	Stomach cancer
National Central Cancer Registry (China). China Cancer Registry Incidence and Mortality 2011	Nonfatal Health Outcomes	Tracheal, bronchus, and lung cancer
Chinese Center for Disease Control and Prevention (CCDC), Ministry of Health (China). China National Injury Surveillance System 2013	Nonfatal Health Outcomes	Interpersonal violence
China Center for Economic Research, Peking University. China Health and Retirement Longitudinal Study Pilot Resurvey 2012. Beijing, China: China Center for Economic Research, Peking University	Nonfatal Health Outcomes	Endocrine, metabolic, blood, and immune disorders
Chinese Center for Disease Control and Prevention (CCDC), Ministry of Health (China). China National Injury Surveillance System 2011 - China CDC	Nonfatal Health Outcomes	Physical violence by sharp object

China Center for Economic Research, Peking University. China Health and Retirement Longitudinal Study Pilot Resurvey 2012. Beijing, China: China Center for Economic Research, Peking University	Nonfatal Health Outcomes	Inguinal, femoral, and abdominal hernia
National Central Cancer Registry (China). China Cancer Registry Incidence and Mortality 2011	Nonfatal Health Outcomes	Colon and rectum cancer
Liang JJ, Zhang ZQ, Chen YJ, Mai JC, Ma J, Yang WH, Jing J. Dental caries is negatively correlated with body mass index among 7-9 years old children in Guangzhou, China. BMC Public Health. 2016; 16: 638	Nonfatal Health Outcomes	Caries of deciduous teeth
Yang, LG, Tucker, JD, Liu, FY, Ren, XQ, Hong, X, Wang, C, McLaughlin, MM, Bien, CH, Chen, XS, Yang, B. Syphilis screening among 27,150 pregnant women in South Chinese rural areas using point-of-care tests. PLoS One. 2013; 8(8): e72149	Nonfatal Health Outcomes	Sexually transmitted infections excluding HIV
National Central Cancer Registry (China). China Cancer Registry Incidence and Mortality 2011	Nonfatal Health Outcomes	Ovarian cancer
Chinese Center for Disease Control and Prevention (CCDC), Ministry of Health (China). China National Injury Surveillance System 2013	Nonfatal Health Outcomes	Physical violence by sharp object
National Central Cancer Registry (China). China Cancer Registry Incidence and Mortality 2011	Nonfatal Health Outcomes	Kidney cancer
Chinese Center for Disease Control and Prevention (CCDC), Ministry of Health (China). China National Injury Surveillance System 2013	Nonfatal Health Outcomes	Road injuries
Chinese Center for Disease Control and Prevention (CCDC), Ministry of Health (China). China National Injury Surveillance System 2011 - China CDC	Nonfatal Health Outcomes	Other transport injuries
International Centre for Eye Health (ICEH). China - Jianchuan Rapid Assessment of Avoidable Blindness 2012. Grootebroek, Netherlands: RAAB Repository	Nonfatal Health Outcomes	Age-related macular degeneration
Chinese Center for Disease Control and Prevention (CCDC), Ministry of Health (China). China National Injury Surveillance System 2011 - China CDC	Nonfatal Health Outcomes	Unintentional firearm injuries
Chinese Center for Disease Control and Prevention (CCDC), Ministry of Health (China). China National Injury Surveillance System 2012 - China CDC	Nonfatal Health Outcomes	Unintentional firearm injuries
Chinese Center for Disease Control and Prevention (CCDC), Ministry of Health (China). China National Injury Surveillance System 2011 - China CDC	Nonfatal Health Outcomes	Other exposure to mechanical forces
Chinese Center for Disease Control and Prevention (CCDC), Ministry of Health (China). China National Injury Surveillance System 2013	Nonfatal Health Outcomes	Falls
Chinese Center for Disease Control and Prevention (CCDC), Ministry of Health (China). China National Injury Surveillance System 2013	Nonfatal Health Outcomes	Drowning
Chinese Center for Disease Control and Prevention (CCDC), Ministry of Health (China). China National Injury Surveillance System 2011 - China CDC	Nonfatal Health Outcomes	Non-venomous animal contact
Chinese Center for Disease Control and Prevention (CCDC), Ministry of Health (China). China National Injury Surveillance System 2012 - China CDC	Nonfatal Health Outcomes	Non-venomous animal contact
Chinese Center for Disease Control and Prevention (CCDC), Ministry of Health (China). China National Injury Surveillance System 2011 - China CDC	Nonfatal Health Outcomes	Drowning
Chinese Center for Disease Control and Prevention (CCDC), Ministry of Health (China). China National Injury Surveillance System 2013	Nonfatal Health Outcomes	Poisonings
Chinese Center for Disease Control and Prevention (CCDC), Ministry of Health (China). China National Injury Surveillance System 2011 - China CDC	Nonfatal Health Outcomes	Physical violence by other means
Chinese Center for Disease Control and Prevention (CCDC), Ministry of Health (China). China National Injury Surveillance System 2012 - China CDC	Nonfatal Health Outcomes	Physical violence by other means
Chinese Center for Disease Control and Prevention (CCDC), Ministry of Health (China). China National Injury Surveillance System 2011 - China CDC	Nonfatal Health Outcomes	Poisonings
Chinese Center for Disease Control and Prevention (CCDC), Ministry of Health (China). China National Injury Surveillance System 2013	Nonfatal Health Outcomes	Exposure to mechanical forces
Chinese Center for Disease Control and Prevention (CCDC), Ministry of Health (China). China National Injury Surveillance System 2011 - China CDC	Nonfatal Health Outcomes	Adverse effects of medical treatment
Chinese Center for Disease Control and Prevention (CCDC), Ministry of Health (China). China National Injury Surveillance System 2012 - China CDC	Nonfatal Health Outcomes	Adverse effects of medical treatment
Chinese Center for Disease Control and Prevention (CCDC), Ministry of Health (China). China National Injury Surveillance System 2013	Nonfatal Health Outcomes	Adverse effects of medical treatment
Chinese Center for Disease Control and Prevention (CCDC), Ministry of Health (China). China National Injury Surveillance System 2011 - China CDC	Nonfatal Health Outcomes	Pulmonary aspiration and foreign body in airway
Mijiti P, Yuehua L, Feng X, Milligan PJ, Merle C, Gang W, Nianqiang L, Upur H. Prevalence of pulmonary tuberculosis in western China in 2010Â–11: a population-based, cross-sectional survey. Lancet Glob Health. 2016; 4(7): e485Â–e494	Nonfatal Health Outcomes	HIV/AIDS - Drug-susceptible Tuberculosis
China Center for Economic Research, Peking University. China Health and Retirement Longitudinal Study Pilot Resurvey 2012. Beijing, China: China Center for Economic Research, Peking University	Nonfatal Health Outcomes	Syphilis
Xue Q, Shen F, Gao Y, Tong M, Zhao M, Chen Q. An analysis of the medical indications for preterm birth in an obstetrics and gynaecology teaching hospital in Shanghai, China. Midwifery. 2016; 35: 17-21	Nonfatal Health Outcomes	Neonatal preterm birth

Chinese Center for Disease Control and Prevention (CCDC), Ministry of Health (China). China National Injury Surveillance System 2013	Nonfatal Health Outcomes	Physical violence by firearm
National Central Cancer Registry (China). China Cancer Registry Incidence and Mortality 2011	Nonfatal Health Outcomes	Breast cancer
Mijiti P, Yuehua L, Feng X, Milligan PJ, Merle C, Gang W, Nianqiang L, Upur H. Prevalence of pulmonary tuberculosis in western China in 2010Â–11: a population-based, cross-sectional survey. Lancet Glob Health. 2016; 4(7): e485Â–e494	Nonfatal Health Outcomes	Multidrug-resistant tuberculosis without extensive drug resistance
China Center for Economic Research, Peking University. China Health and Retirement Longitudinal Study Pilot Resurvey 2012. Beijing, China: China Center for Economic Research, Peking University	Nonfatal Health Outcomes	Cirrhosis and other chronic liver diseases
Li S-Z, Zheng H, Gao J, Zhang L-J, Zhu R, Xu J, Guo J-G, Xiao N, Zhou X-N. [Endemic status of schistosomiasis in PeopleÂ's Republic of China in 2012]. Chin J Schistosomiasis Control. 2013; 25(6): 557Â–63	Nonfatal Health Outcomes	Schistosomiasis
Mijiti P, Yuehua L, Feng X, Milligan PJ, Merle C, Gang W, Nianqiang L, Upur H. Prevalence of pulmonary tuberculosis in western China in 2010Â–11: a population-based, cross-sectional survey. Lancet Glob Health. 2016; 4(7): e485Â–e494	Nonfatal Health Outcomes	HIV/AIDS - Multidrug-resistant Tuberculosis without extensive drug resistance
Mijiti P, Yuehua L, Feng X, Milligan PJ, Merle C, Gang W, Nianqiang L, Upur H. Prevalence of pulmonary tuberculosis in western China in 2010Â–11: a population-based, cross-sectional survey. Lancet Glob Health. 2016; 4(7): e485Â–e494	Nonfatal Health Outcomes	Drug-susceptible tuberculosis
National Central Cancer Registry (China). China Cancer Registry Incidence and Mortality 2011	Nonfatal Health Outcomes	Cervical cancer
National Central Cancer Registry (China). China Cancer Registry Incidence and Mortality 2011	Nonfatal Health Outcomes	Uterine cancer
National Central Cancer Registry (China). China Cancer Registry Incidence and Mortality 2011	Nonfatal Health Outcomes	Bladder cancer
Chinese Center for Disease Control and Prevention (CCDC), Ministry of Health (China). China National Injury Surveillance System 2013	Nonfatal Health Outcomes	Non-venomous animal contact
China Center for Economic Research, Peking University. China Health and Retirement Longitudinal Study Pilot Resurvey 2012. Beijing, China: China Center for Economic Research, Peking University	Nonfatal Health Outcomes	Decubitus ulcer
Mijiti P, Yuehua L, Feng X, Milligan PJ, Merle C, Gang W, Nianqiang L, Upur H. Prevalence of pulmonary tuberculosis in western China in 2010Â–11: a population-based, cross-sectional survey. Lancet Glob Health. 2016; 4(7): e485Â–e494	Nonfatal Health Outcomes	Extensively drug-resistant tuberculosis
Chinese Center for Disease Control and Prevention (CCDC), Ministry of Health (China). China National Injury Surveillance System 2013	Nonfatal Health Outcomes	Physical violence by other means
Li X, Zhou Q, Sun L, Wang Z, Han S, Wu S, Wang N. Prevalence of blindness and low vision in a rural population in northern China: preliminary results from a population-based survey. Ophthalmic Epidemiol. 2012; 19(5): 272-7	Nonfatal Health Outcomes	Refraction disorders
Mijiti P, Yuehua L, Feng X, Milligan PJ, Merle C, Gang W, Nianqiang L, Upur H. Prevalence of pulmonary tuberculosis in western China in 2010Â–11: a population-based, cross-sectional survey. Lancet Glob Health. 2016; 4(7): e485Â–e494	Nonfatal Health Outcomes	HIV/AIDS - Extensively drug-resistant Tuberculosis
Zheng J-Y, Tian H-T, Zhu Z-M, Li B, Han L, Jiang S-L, Chen Y, Li D-T, He J-C, Zhao Z, Cao Y, Qiu Y-G, Li T-C. Prevalence of symptomatic congenital heart disease in Tibetan school children. Am J Cardiol. 2013; 112(9): 1468-70	Nonfatal Health Outcomes	Congenital birth defects
Luo W-Y, Huang X-M, Gong Z-Y, Huang W-J. Analysis on the epidemic condition about severe psychiatric diseases in Houjie Town of Dongguan in 2012. Chin Prim Health Care. 2014; 28(1): 100-102	Nonfatal Health Outcomes	Schizophrenia
International Centre for Eye Health (ICEH). China - Sichuan Dechang Rapid Assessment of Avoidable Blindness 2011	Nonfatal Health Outcomes	Refraction and accommodation disorders
China Center for Economic Research, Peking University. China Health and Retirement Longitudinal Study Pilot Resurvey 2012. Beijing, China: China Center for Economic Research, Peking University	Nonfatal Health Outcomes	Non-venomous animal contact
Weng J, Zhou Z, Guo L, Zhu D, Ji L, Luo X, Mu Y, Jia W. Incidence of type 1 diabetes in China, 2010-13: population based study. BMJ. 2018; 360: j5295	Nonfatal Health Outcomes	Diabetes mellitus type 1
China Center for Economic Research, Peking University. China Health and Retirement Longitudinal Study Pilot Resurvey 2012. Beijing, China: China Center for Economic Research, Peking University	Nonfatal Health Outcomes	Road injuries
National Central Cancer Registry (China). China Cancer Registry Incidence and Mortality 2011	Nonfatal Health Outcomes	Lip and oral cavity cancer
Kang G, Xiao J, Wang J, Chen J, Li W, Wang Y, Liu Q, Wang Z, Xia J, Huang J, Cheng L, Chen Y, Chen Q, Yang F. Congenital Heart Disease in Local and Migrant Elementary Schoolchildren in Dongguan, China. Am J Cardiol. 2016; 117(3): 461-4	Nonfatal Health Outcomes	Congenital birth defects
National Central Cancer Registry (China). China Cancer Registry Incidence and Mortality 2011	Nonfatal Health Outcomes	Larynx cancer
National Central Cancer Registry (China). China Cancer Registry Incidence and Mortality 2011	Nonfatal Health Outcomes	Brain and central nervous system cancer

National Central Cancer Registry (China). China Cancer Registry Incidence and Mortality 2011	Nonfatal Health Outcomes	Thyroid cancer
International Centre for Eye Health (ICEH). China - Sichuan Dechang Rapid Assessment of Avoidable Blindness 2011	Nonfatal Health Outcomes	Cataract
Chinese Center for Disease Control and Prevention (CCDC), Ministry of Health (China). China National Injury Surveillance System 2011 - China CDC	Nonfatal Health Outcomes	Pedestrian road injuries
Chinese Center for Disease Control and Prevention (CCDC), Ministry of Health (China). China National Injury Surveillance System 2011 - China CDC	Nonfatal Health Outcomes	Cyclist road injuries
Chinese Center for Disease Control and Prevention (CCDC), Ministry of Health (China). China National Injury Surveillance System 2011 - China CDC	Nonfatal Health Outcomes	Motorcyclist road injuries
Chinese Center for Disease Control and Prevention (CCDC), Ministry of Health (China). China National Injury Surveillance System 2011 - China CDC	Nonfatal Health Outcomes	Motor vehicle road injuries
Chinese Center for Disease Control and Prevention (CCDC), Ministry of Health (China). China National Injury Surveillance System 2013	Nonfatal Health Outcomes	Unintentional firearm injuries
Chinese Center for Disease Control and Prevention (CCDC), Ministry of Health (China). China National Injury Surveillance System 2013	Nonfatal Health Outcomes	Other exposure to mechanical forces
Chinese Center for Disease Control and Prevention (CCDC), Ministry of Health (China). China National Injury Surveillance System 2013	Nonfatal Health Outcomes	Venomous animal contact
Chinese Center for Disease Control and Prevention (CCDC), Ministry of Health (China). China National Injury Surveillance System 2011 - China CDC	Nonfatal Health Outcomes	Venomous animal contact
Chinese Center for Disease Control and Prevention (CCDC), Ministry of Health (China). China National Injury Surveillance System 2012 - China CDC	Nonfatal Health Outcomes	Venomous animal contact
Center for Health Statistics and Information, National Health and Family Planning Commission (China), Shanghai Municipal Center for Disease Control and Prevention (Shanghai CDC), Shanghai Health Information Center. China Hospital Inpatient Data 2013-2016	Nonfatal Health Outcomes	Meningitis
Wang W, Jiang B, Sun H, Ru X, Sun D, Wang L, Wang L, Jiang Y, Li Y, Wang Y, Chen Z, Wu S, Zhang Y, Wang D, Wang Y, Feigin VL; NESS-China investigators. Prevalence, Incidence and Mortality of Stroke in China: Results from a Nationwide Population-Based Survey of 480,687 Adults. Circulation. 2017; 135(8): 759-71	Nonfatal Health Outcomes	Ischemic stroke
Chinese Center for Disease Control and Prevention (CCDC), Ministry of Health (China). China National Injury Surveillance System 2013	Nonfatal Health Outcomes	Foreign body in eyes
Wang W, Jiang B, Sun H, Ru X, Sun D, Wang L, Wang L, Jiang Y, Li Y, Wang Y, Chen Z, Wu S, Zhang Y, Wang D, Wang Y, Feigin VL; NESS-China investigators. Prevalence, Incidence and Mortality of Stroke in China: Results from a Nationwide Population-Based Survey of 480,687 Adults. Circulation. 2017; 135(8): 759-71	Nonfatal Health Outcomes	Intracerebral hemorrhage
Zheng J-Y, Tian H-T, Zhu Z-M, Li B, Han L, Jiang S-L, Chen Y, Li D-T, He J-C, Zhao Z, Cao Y, Qiu Y-G, Li T-C. Prevalence of symptomatic congenital heart disease in Tibetan school children. Am J Cardiol. 2013; 112(9): 1468-70	Nonfatal Health Outcomes	Congenital heart anomalies
Chinese Center for Disease Control and Prevention (CCDC), Ministry of Health (China). China National Injury Surveillance System 2011 - China CDC	Nonfatal Health Outcomes	Physical violence by firearm
Chinese Center for Disease Control and Prevention (CCDC), Ministry of Health (China). China National Injury Surveillance System 2012 - China CDC	Nonfatal Health Outcomes	Physical violence by firearm
China Center for Economic Research, Peking University. China Health and Retirement Longitudinal Study Pilot Resurvey 2012. Beijing, China: China Center for Economic Research, Peking University	Nonfatal Health Outcomes	Orofacial clefts
International Centre for Eye Health (ICEH). China - Sichuan Dechang Rapid Assessment of Avoidable Blindness 2011	Nonfatal Health Outcomes	
Shu C, Chen S, Qin T, Fu Z, Sun T, Xie M, Zhang L, Dong N, Yin P. Prevalence and correlates of valvular heart diseases in the elderly population in Hubei, China. Sci Rep. 2016; 6: 27253	Nonfatal Health Outcomes	Non-rheumatic calcific aortic valve disease
Shu C, Chen S, Qin T, Fu Z, Sun T, Xie M, Zhang L, Dong N, Yin P. Prevalence and correlates of valvular heart diseases in the elderly population in Hubei, China. Sci Rep. 2016; 6: 27253	Nonfatal Health Outcomes	Non-rheumatic degenerative mitral valve disease
China Center for Economic Research, Peking University. China Health and Retirement Longitudinal Study Pilot Resurvey 2012. Beijing, China: China Center for Economic Research, Peking University	Nonfatal Health Outcomes	Lower respiratory infections
China Center for Economic Research, Peking University. China Health and Retirement Longitudinal Study Pilot Resurvey 2012. Beijing, China: China Center for Economic Research, Peking University	Nonfatal Health Outcomes	Interpersonal violence
China Center for Economic Research, Peking University. China Health and Retirement Longitudinal Study Pilot Resurvey 2012. Beijing, China: China Center for Economic Research, Peking University	Nonfatal Health Outcomes	Endocarditis
International Centre for Eye Health (ICEH). China - Sichuan Mianning Rapid Assessment of Avoidable Blindness 2011	Nonfatal Health Outcomes	Other vision loss

Yang, LG, Tucker, JD, Liu, FY, Ren, XQ, Hong, X, Wang, C, McLaughlin, MM, Bien, CH, Chen, XS, Yang, B. Syphilis screening among 27,150 pregnant women in South Chinese rural areas using point-of-care tests. PLoS One. 2013; 8(8): e72149	Nonfatal Health Outcomes	Syphilis
Gao L, Lu W, Bai L, Wang X, Xu J, Catanzaro A, CÃ¡rdenas V, Li X, Yang Y, Du J, Sui H, Xia Y, Li M, Feng B, Li Z, Xin H, Zhao R, Liu J, Pan S, Shen F, He J, Yang S, Si H, Wang Y, Xu Z, Tan Y, Chen T, Xu W, Peng H, Wang Z, Zhu T, Zhou F, Liu H, Zhao Y, Cheng S, Jin Q; LATENTTB-NSTM study team. Latent tuberculosis infection in rural China: baseline results of a population-based, multicentre, prospective cohort study. Lancet Infect Dis. 2015; 15(3): 310-9	Nonfatal Health Outcomes	Tuberculosis
World Health Organization (WHO). Global leprosy situation, 2012. Wkly Epidemiol Rec. 2012: 87(34): 317-28.	Nonfatal Health Outcomes	Leprosy
World Health Organization (WHO). Global leprosy: update on the 2012 situation. Wkly Epidemiol Rec. 2013: 88(35): 365-79.	Nonfatal Health Outcomes	Leprosy
Wang Z, Xu M, Hu Z, Shrestha UK. Prevalence of nonalcoholic fatty liver disease and its metabolic risk factors in women of different ages and body mass index. Menopause. 2015; 22(6): 667-73	Nonfatal Health Outcomes	Cirrhosis due to NASH
Ji Y, Shi Z, Zhang Y, Liu S, Liu S, Yue W, Liu M, Huo YR, Wang J, Wisniewski T. Prevalence of dementia and main subtypes in rural northern China. Dement Geriatr Cogn Disord. 2015; 39(5-6): 294Â–302	Nonfatal Health Outcomes	Alzheimer's disease and other dementias
Chinese Center for Disease Control and Prevention (CCDC), Ministry of Health (China). China National Injury Surveillance System 2011 - China CDC	Nonfatal Health Outcomes	Injuries
Chinese Center for Disease Control and Prevention (CCDC). China Notifiable Infectious Diseases 2012	Nonfatal Health Outcomes	Gonococcal infection
Ju X, Fang Q, Zhang J, Xu A, Liang L, Ke C. Viral etiology of influenza-like illnesses in Huizhou, China, from 2011 to 2013. Arch Virol. 2014; 159(8): 2003-10	Nonfatal Health Outcomes	Lower respiratory infections
Zhu Q, Zheng H, Qu H, Deng H, Zhang J, Ma W, Lin Y, Xie X, Qiu Q, Huang Z. Epidemiology of herpes zoster among adults aged 50 and above in Guangdong, China. Hum Vaccin Immunother. 2015; 11(8): 2113-8	Nonfatal Health Outcomes	Varicella and herpes zoster
International Centre for Eye Health (ICEH). China - Sichuan Mianning Rapid Assessment of Avoidable Blindness 2011	Nonfatal Health Outcomes	Refraction and accommodation disorders
China Center for Economic Research, Peking University. China Health and Retirement Longitudinal Study Pilot Resurvey 2012. Beijing, China: China Center for Economic Research, Peking University	Nonfatal Health Outcomes	Benign prostatic hyperplasia
Wen H, Wang D, Wang W, Yang X-M, Peng R, Yao F-Q. The State of Death and the Cause of Death in Severe Psychotic Patients who were Managed in Sichuan, 2011-2013. Sichuan Ment Health. 2014; 4: 337-40	Nonfatal Health Outcomes	Schizophrenia
China Center for Economic Research, Peking University. China Health and Retirement Longitudinal Study Pilot Resurvey 2012. Beijing, China: China Center for Economic Research, Peking University	Nonfatal Health Outcomes	Peptic ulcer disease
China Center for Economic Research, Peking University. China Health and Retirement Longitudinal Study Pilot Resurvey 2012. Beijing, China: China Center for Economic Research, Peking University	Nonfatal Health Outcomes	Congenital musculoskeletal and limb anomalies
China Center for Economic Research, Peking University. China Health and Retirement Longitudinal Study Pilot Resurvey 2012. Beijing, China: China Center for Economic Research, Peking University	Nonfatal Health Outcomes	Cyclist road injuries
Chung VCH, Yip BHK, Griffiths SM, Yu ELM, Kim JH, Tam WWS, Wong AHC, Chan IWT, Lau JTF. The impact of cutting alcohol duties on drinking patterns in Hong Kong. Alcohol Alcohol. 2013; 48(6): 720Â–8	Nonfatal Health Outcomes	Alcohol use disorders
Wong CKM, Liang J, Chan ML, Chan YH, Chan L, Wan KY, Ng MS, Chan DCC, Wong SYS, Wong MCS. Prevalence and psychosocial correlates of depressive symptoms in urban Chinese women during midlife. PLoS One. 2014; 9(11): e110877	Nonfatal Health Outcomes	Major depressive disorder
Lam LC-W, Wong CS-M, Wang M-J, Chan W-C, Chen EY-H, Ng RM-K, Hung S-F, Cheung EF-C, Sham P-C, Chiu HF-K, Lam M, Chang W-C, Lee EH-M, Chiang T-P, Lau JT-F, van Os J, Lewis G, Bebbington P. Prevalence, psychosocial correlates and service utilization of depressive and anxiety disorders in Hong Kong: the Hong Kong Mental Morbidity Survey (HKMMS). Soc Psychiatry Psychiatr Epidemiol. 2015; 50(9): 1379Â–88	Nonfatal Health Outcomes	Major depressive disorder
Lam LC-W, Wong CS-M, Wang M-J, Chan W-C, Chen EY-H, Ng RM-K, Hung S-F, Cheung EF-C, Sham P-C, Chiu HF-K, Lam M, Chang W-C, Lee EH-M, Chiang T-P, Lau JT-F, van Os J, Lewis G, Bebbington P. Prevalence, psychosocial correlates and service utilization of depressive and anxiety disorders in Hong Kong: the Hong Kong Mental Morbidity Survey (HKMMS). Soc Psychiatry Psychiatr Epidemiol. 2015; 50(9): 1379Â–88	Nonfatal Health Outcomes	Anxiety disorders
China Center for Economic Research, Peking University. China Health and Retirement Longitudinal Study Pilot Resurvey 2012. Beijing, China: China Center for Economic Research, Peking University	Nonfatal Health Outcomes	Encephalitis

International Centre for Eye Health (ICEH). China - Sichuan Dechang Rapid Assessment of Avoidable Blindness 2011	Nonfatal Health Outcomes	Vision loss
China Center for Economic Research, Peking University. China Health and Retirement Longitudinal Study Pilot Resurvey 2012. Beijing, China: China Center for Economic Research, Peking University	Nonfatal Health Outcomes	Pulmonary aspiration and foreign body in airway
Huang G, Yu D, Mao N, Zhu Z, Zhang H, Jiang Z, Li H, Zhang Y, Shi J, Zhang S, Wang X, Xu W. Viral etiology of acute respiratory infection in Gansu Province, China, 2011. PLoS One. 2013; 8(5): e64254	Nonfatal Health Outcomes	Lower respiratory infections
China Center for Economic Research, Peking University. China Health and Retirement Longitudinal Study Pilot Resurvey 2012. Beijing, China: China Center for Economic Research, Peking University	Nonfatal Health Outcomes	Poisonings
International Centre for Eye Health (ICEH). China - Inner Mongolia Shangdu Rapid Assessment of Avoidable Blindness 2010	Nonfatal Health Outcomes	Refraction disorders
China Center for Economic Research, Peking University. China Health and Retirement Longitudinal Study Pilot Resurvey 2012. Beijing, China: China Center for Economic Research, Peking University	Nonfatal Health Outcomes	Pyoderma
Kang G, Xiao J, Wang J, Chen J, Li W, Wang Y, Liu Q, Wang Z, Xia J, Huang J, Cheng L, Chen Y, Chen Q, Yang F. Congenital Heart Disease in Local and Migrant Elementary Schoolchildren in Dongguan, China. Am J Cardiol. 2016; 117(3): 461-4	Nonfatal Health Outcomes	Congenital heart anomalies
Li C, Zhao K, Li H, Farah OI, Wang J, Sun R, Zhang H. Free preconceptual screening examination service in rural areas of Hubei Province, China in 2012. PLoS One. 2014; 9(11): e111918	Nonfatal Health Outcomes	Sexually transmitted infections excluding HIV
China Center for Economic Research, Peking University. China Health and Retirement Longitudinal Study Pilot Resurvey 2012. Beijing, China: China Center for Economic Research, Peking University	Nonfatal Health Outcomes	Self-harm by other specified means
China Center for Economic Research, Peking University. China Health and Retirement Longitudinal Study Pilot Resurvey 2012. Beijing, China: China Center for Economic Research, Peking University	Nonfatal Health Outcomes	Maternal sepsis and other maternal infections
Ge X-L, Zhou J-L. Survey on schizophrenia among people over 15 years old in Changsha. J Clin Psychiatry. 2014; 24(5): 292-295	Nonfatal Health Outcomes	Schizophrenia
Tan B-T, Yang M, Guo T-S, Zhou X-H, Liu Y-J, Zhou J-L, Yu A-Z, Song D-M, Zhou L. Comorbidity of alcohol use disorders and mood disorders in residents in Changsha area. Chin Ment Health J. 2014; 28(5): 349Â–355	Nonfatal Health Outcomes	Alcohol use disorders
China Center for Economic Research, Peking University. China Health and Retirement Longitudinal Study Pilot Resurvey 2012. Beijing, China: China Center for Economic Research, Peking University	Nonfatal Health Outcomes	Ischemic heart disease
China Center for Economic Research, Peking University. China Health and Retirement Longitudinal Study Pilot Resurvey 2012. Beijing, China: China Center for Economic Research, Peking University	Nonfatal Health Outcomes	Acute glomerulonephritis
Chinese Center for Disease Control and Prevention (CCDC). China Notifiable Infectious Diseases 2012	Nonfatal Health Outcomes	Syphilis
Cao X, Wang D, Zhou J, Yuan H, Chen Z. Relationship between dental caries and metabolic syndrome among 13 998 middle-aged urban Chinese. J Diabetes. 2017; 9(4): 378-385	Nonfatal Health Outcomes	Caries of permanent teeth
China Center for Economic Research, Peking University. China Health and Retirement Longitudinal Study Pilot Resurvey 2012. Beijing, China: China Center for Economic Research, Peking University	Nonfatal Health Outcomes	Pancreatitis
Fu QL, Du Y, Xu G, Zhang H, Cheng L, Wang YJ, Zhu DD, Lv W, Liu SX, Li PZ, Shi JB, Ou CQ. Prevalence and Occupational and Environmental Risk Factors of Self-Reported Asthma: Evidence from a Cross-Sectional Survey in Seven Chinese Cities. Int J Environ Res Public Health. 2016; 13(11)	Nonfatal Health Outcomes	Asthma
Jian-Long Z, Yu-Ling W, Ya-Ni Y. Analysis of prevalence and related factors in different national Parkinson's disease in Yili of Xinjiang area. J Xinjiang Med Univ. 2013; 36(3): 278-281	Nonfatal Health Outcomes	Parkinson's disease
Lu-Ning W, Tai-Fang Z, Yu-Ling W. Study on the prevalence and risk factors of the Parkinson's disease in residents aged 35 years or older in Urumqi city. J Xinjiang Med Univ. 2013; 3	Nonfatal Health Outcomes	Parkinson's disease
Li Z, Zhu L, Zhang B, Si Y, Liu M. [Periodontal health status assessed by community periodontal index and related factors in adult population of Beijing urban community]. J Peking Univ Health Sci. 2012; 44(1): 130-4	Nonfatal Health Outcomes	Periodontal diseases
Feng L, Li P, Lu C, Tang W, Mahapatra T, Wang Y, Wang X, Ma Y, Ben Y, Cao X, Mahapatra S, Ling M, Gou A, Wang Y, Xiao J, Hou M, Wang X, Lin B, Chen R, Wang F, Hu Z. Burden and correlates of geriatric depression in the Uyghur elderly population, observation from Xinjiang, China. PLoS One. 2014; 9(12): e114139	Nonfatal Health Outcomes	Major depressive disorder

Pi X, Zhou L, Cui L, Liu A, Zhang J, Ma Y, Liu B, Cai C, Zhu C, Zhou T, Chen J, Zhou Z, Wang C, Li L, Li S, Wu J, Xiao B. Prevalence and clinical characteristics of active epilepsy in southern Han Chinese. Seizure. 2014; 23(8): 636-40	Nonfatal Health Outcomes	Epilepsy
United Nations Population Division. World Abortion Policies 2013. New York, United States: United Nations (UN), 2013	Nonfatal Health Outcomes	Ectopic pregnancy
Chinese Center for Disease Control and Prevention (CCDC). China Notifiable Infectious Diseases 2012	Nonfatal Health Outcomes	Sexually transmitted infections excluding HIV
Sun G-Z, Guo L, Wang X-Z, Song H-J, Li Z, Wang J, Sun Y-X. Prevalence of atrial fibrillation and its risk factors in rural China: a cross-sectional study. Int J Cardiol. 2015; 13Â–7	Nonfatal Health Outcomes	Atrial fibrillation and flutter
Bellizzi S, Ali MM, Abalos E, Betran AP, Kapila J, Pileggi-Castro C, Vogel JP, Merialdi M. Are hypertensive disorders in pregnancy associated with congenital malformations in offspring? Evidence from the WHO Multicountry cross sectional survey on maternal and newborn health. BMC Pregnancy Childbirth. 2016; 16(1): 198	Nonfatal Health Outcomes	Congenital heart anomalies
Cheng W, Wu Y, Wen Y, Ma Y, Zhao D, Dou Z, Zhang W, Bulterys M, Zhang F. Cotrimoxazole prophylaxis and antiretroviral therapy: an observational cohort study in China. Bull World Health Organ. 2015; 93(3): 152Â–60	Nonfatal Health Outcomes	HIV/AIDS resulting in other diseases
Li Y, Wulaerhan J, Liu Y, Abudureyimu A, Zhao J. Prevalence of severe early childhood caries and associated socioeconomic and behavioral factors in Xinjiang, China: a cross-sectional study. BMC Oral Health. 2017; 17(1): 144	Nonfatal Health Outcomes	Caries of deciduous teeth
Chen Q, Huang LX, Xu WJ, Chen H, Zhong JQ, Zeng CZ. Research on the prevalence and the risk factors of autism sepctrum disorder from 1.5 to 3 years old in Zhuhai city. Chin J Child Health Care. 2014; 22(6): 649-651	Nonfatal Health Outcomes	Autism spectrum disorders
Li RC, Huang T, Li Y, Luo D, Tao J, Fu B, Si G, Nong Y, Mo Z, Liao X, Luan I, Tang H, Rathi N, Karkada N, Han HH. Human rotavirus vaccine (RIX4414) efficacy in the first two years of life: a randomized, placebo-controlled trial in China. Hum Vaccin Immunother. 2014; 10(1): 8-Nov	Nonfatal Health Outcomes	Diarrheal diseases
Chinese Center for Disease Control and Prevention (CCDC), Ministry of Health (China). China National Injury Surveillance System 2012 - China CDC	Nonfatal Health Outcomes	Injuries
Gao L, Lu W, Bai L, Wang X, Xu J, Catanzaro A, CÃ¡rdenas V, Li X, Yang Y, Du J, Sui H, Xia Y, Li M, Feng B, Li Z, Xin H, Zhao R, Liu J, Pan S, Shen F, He J, Yang S, Si H, Wang Y, Xu Z, Tan Y, Chen T, Xu W, Peng H, Wang Z, Zhu T, Zhou F, Liu H, Zhao Y, Cheng S, Jin Q; LATENTTB-NSTM study team. Latent tuberculosis infection in rural China: baseline results of a population-based, multicentre, prospective cohort study. Lancet Infect Dis. 2015; 15(3): 310-9	Nonfatal Health Outcomes	Latent tuberculosis infection
Gao L, Lu W, Bai L, Wang X, Xu J, Catanzaro A, CÃ¡rdenas V, Li X, Yang Y, Du J, Sui H, Xia Y, Li M, Feng B, Li Z, Xin H, Zhao R, Liu J, Pan S, Shen F, He J, Yang S, Si H, Wang Y, Xu Z, Tan Y, Chen T, Xu W, Peng H, Wang Z, Zhu T, Zhou F, Liu H, Zhao Y, Cheng S, Jin Q; LATENTTB-NSTM study team. Latent tuberculosis infection in rural China: baseline results of a population-based, multicentre, prospective cohort study. Lancet Infect Dis. 2015; 15(3): 310-9	Nonfatal Health Outcomes	HIV/AIDS - Multidrug-resistant Tuberculosis without extensive drug resistance
Gao L, Lu W, Bai L, Wang X, Xu J, Catanzaro A, CÃ¡rdenas V, Li X, Yang Y, Du J, Sui H, Xia Y, Li M, Feng B, Li Z, Xin H, Zhao R, Liu J, Pan S, Shen F, He J, Yang S, Si H, Wang Y, Xu Z, Tan Y, Chen T, Xu W, Peng H, Wang Z, Zhu T, Zhou F, Liu H, Zhao Y, Cheng S, Jin Q; LATENTTB-NSTM study team. Latent tuberculosis infection in rural China: baseline results of a population-based, multicentre, prospective cohort study. Lancet Infect Dis. 2015; 15(3): 310-9	Nonfatal Health Outcomes	Multidrug-resistant tuberculosis without extensive drug resistance
Gao L, Lu W, Bai L, Wang X, Xu J, Catanzaro A, CÃ¡rdenas V, Li X, Yang Y, Du J, Sui H, Xia Y, Li M, Feng B, Li Z, Xin H, Zhao R, Liu J, Pan S, Shen F, He J, Yang S, Si H, Wang Y, Xu Z, Tan Y, Chen T, Xu W, Peng H, Wang Z, Zhu T, Zhou F, Liu H, Zhao Y, Cheng S, Jin Q; LATENTTB-NSTM study team. Latent tuberculosis infection in rural China: baseline results of a population-based, multicentre, prospective cohort study. Lancet Infect Dis. 2015; 15(3): 310-9	Nonfatal Health Outcomes	Drug-susceptible tuberculosis
Gao L, Lu W, Bai L, Wang X, Xu J, Catanzaro A, CÃ¡rdenas V, Li X, Yang Y, Du J, Sui H, Xia Y, Li M, Feng B, Li Z, Xin H, Zhao R, Liu J, Pan S, Shen F, He J, Yang S, Si H, Wang Y, Xu Z, Tan Y, Chen T, Xu W, Peng H, Wang Z, Zhu T, Zhou F, Liu H, Zhao Y, Cheng S, Jin Q; LATENTTB-NSTM study team. Latent tuberculosis infection in rural China: baseline results of a population-based, multicentre, prospective cohort study. Lancet Infect Dis. 2015; 15(3): 310-9	Nonfatal Health Outcomes	Extensively drug-resistant tuberculosis
Gao L, Lu W, Bai L, Wang X, Xu J, Catanzaro A, CÃ¡rdenas V, Li X, Yang Y, Du J, Sui H, Xia Y, Li M, Feng B, Li Z, Xin H, Zhao R, Liu J, Pan S, Shen F, He J, Yang S, Si H, Wang Y, Xu Z, Tan Y, Chen T, Xu W, Peng H, Wang Z, Zhu T, Zhou F, Liu H, Zhao Y, Cheng S, Jin Q; LATENTTB-NSTM study team. Latent tuberculosis infection in rural China: baseline results of a population-based, multicentre, prospective cohort study. Lancet Infect Dis. 2015; 15(3): 310-9	Nonfatal Health Outcomes	HIV/AIDS - Drug-susceptible Tuberculosis

Fung J, Lee CK, Chan M, Seto WK, Lai CL, Yuen MF, Hong Kong Liver Health Census Study Group. High prevalence of non-alcoholic fatty liver disease in the Chinese - results from the Hong Kong liver health census. Liver Int. 2015; 35(2): 542-9	Nonfatal Health Outcomes	Cirrhosis due to NASH
Gao L, Lu W, Bai L, Wang X, Xu J, Catanzaro A, CÃ¡rdenas V, Li X, Yang Y, Du J, Sui H, Xia Y, Li M, Feng B, Li Z, Xin H, Zhao R, Liu J, Pan S, Shen F, He J, Yang S, Si H, Wang Y, Xu Z, Tan Y, Chen T, Xu W, Peng H, Wang Z, Zhu T, Zhou F, Liu H, Zhao Y, Cheng S, Jin Q; LATENTTB-NSTM study team. Latent tuberculosis infection in rural China: baseline results of a population-based, multicentre, prospective cohort study. Lancet Infect Dis. 2015; 15(3): 310-9	Nonfatal Health Outcomes	HIV/AIDS - Extensively drug-resistant Tuberculosis
International Centre for Eye Health (ICEH). China - Sichuan Mianning Rapid Assessment of Avoidable Blindness 2011	Nonfatal Health Outcomes	
China Center for Economic Research, Peking University. China Health and Retirement Longitudinal Study Pilot Resurvey 2012. Beijing, China: China Center for Economic Research, Peking University	Nonfatal Health Outcomes	Coal workers pneumoconiosis
China Center for Economic Research, Peking University. China Health and Retirement Longitudinal Study Pilot Resurvey 2012. Beijing, China: China Center for Economic Research, Peking University	Nonfatal Health Outcomes	Pedestrian road injuries
International Centre for Eye Health (ICEH). China - Sichuan Dechang Rapid Assessment of Avoidable Blindness 2011	Nonfatal Health Outcomes	Other vision loss
United Nations Population Division. World Abortion Policies 2013. New York, United States: United Nations (UN), 2013	Nonfatal Health Outcomes	None
Carter Center, International Trachoma Initiative, London School of Hygiene and Tropical Medicine. Global Atlas of Trachoma. Decatur, United States: International Trachoma Initiative	Nonfatal Health Outcomes	
National Central Cancer Registry (China). China Cancer Registry Incidence and Mortality 2011	Nonfatal Health Outcomes	Other pharynx cancer
China Center for Economic Research, Peking University. China Health and Retirement Longitudinal Study Pilot Resurvey 2012. Beijing, China: China Center for Economic Research, Peking University	Nonfatal Health Outcomes	Interstitial lung disease and pulmonary sarcoidosis
Liu F, Liao L-H, Jiang Z-S. Epidemiological Investigation on ADHD Among Children aged 6-12 in Liuzhou City. Contemp Med Forum. 2014; 12(16): 179-181	Nonfatal Health Outcomes	Attention-deficit/hyperactivity disorder
Xiu-Shan He. Analysis of the Status and Influence of Alzheimer's Disease in Rural Communities. J Jiujiang Univ Natural Sci. 2012; 1: 29-32	Nonfatal Health Outcomes	Alzheimer's disease and other dementias
China Center for Economic Research, Peking University. China Health and Retirement Longitudinal Study Pilot Resurvey 2012. Beijing, China: China Center for Economic Research, Peking University	Nonfatal Health Outcomes	Visceral leishmaniasis
Chinese Center for Disease Control and Prevention (CCDC). China Notifiable Infectious Diseases 2011	Nonfatal Health Outcomes	Sexually transmitted infections excluding HIV
China Center for Economic Research, Peking University. China Health and Retirement Longitudinal Study Pilot Resurvey 2012. Beijing, China: China Center for Economic Research, Peking University	Nonfatal Health Outcomes	Foreign body in other body part
China Center for Economic Research, Peking University. China Health and Retirement Longitudinal Study Pilot Resurvey 2012. Beijing, China: China Center for Economic Research, Peking University	Nonfatal Health Outcomes	Vascular intestinal disorders
International Centre for Eye Health (ICEH). China - Jianchuan Rapid Assessment of Avoidable Blindness 2012. Grootebroek, Netherlands: RAAB Repository	Nonfatal Health Outcomes	Cataract
China Center for Economic Research, Peking University. China Health and Retirement Longitudinal Study Pilot Resurvey 2012. Beijing, China: China Center for Economic Research, Peking University	Nonfatal Health Outcomes	Turner syndrome
Chinese Center for Disease Control and Prevention (CCDC). China Notifiable Infectious Diseases 2011	Nonfatal Health Outcomes	Syphilis
China Center for Economic Research, Peking University. China Health and Retirement Longitudinal Study Pilot Resurvey 2012. Beijing, China: China Center for Economic Research, Peking University	Nonfatal Health Outcomes	Unintentional suffocation
Chinese Center for Disease Control and Prevention (CCDC). China Notifiable Infectious Diseases 2012	Nonfatal Health Outcomes	Chlamydial infection
China Center for Economic Research, Peking University. China Health and Retirement Longitudinal Study Pilot Resurvey 2012. Beijing, China: China Center for Economic Research, Peking University	Nonfatal Health Outcomes	Environmental heat and cold exposure
Mijiti P, Yuehua L, Feng X, Milligan PJ, Merle C, Gang W, Nianqiang L, Upur H. Prevalence of pulmonary tuberculosis in western China in 2010Â–11: a population-based, cross-sectional survey. Lancet Glob Health. 2016; 4(7): e485Â–e494	Nonfatal Health Outcomes	Tuberculosis
Ren C, McGrath C, Yang Y. Edentulism and associated factors among community-dwelling middle-aged and elderly adults in China. Gerodontology. 2017; 34(2): 195-207	Nonfatal Health Outcomes	Edentulism and severe tooth loss

China Center for Economic Research, Peking University. China Health and Retirement Longitudinal Study Pilot Resurvey 2012. Beijing, China: China Center for Economic Research, Peking University	Nonfatal Health Outcomes	Motor vehicle road injuries
International Centre for Eye Health (ICEH). China - Sichuan Mianning Rapid Assessment of Avoidable Blindness 2011	Nonfatal Health Outcomes	Cataract
Ju X, Fang Q, Zhang J, Xu A, Liang L, Ke C. Viral etiology of influenza-like illnesses in Huizhou, China, from 2011 to 2013. Arch Virol. 2014; 159(8): 2003-10	Nonfatal Health Outcomes	Respiratory syncytial virus pneumonia
China Center for Economic Research, Peking University. China Health and Retirement Longitudinal Study Pilot Resurvey 2012. Beijing, China: China Center for Economic Research, Peking University	Nonfatal Health Outcomes	Diarrheal diseases
International Centre for Eye Health (ICEH). China - Sichuan Mianning Rapid Assessment of Avoidable Blindness 2011	Nonfatal Health Outcomes	Glaucoma
Deng QW, Chen YG, Huang QS, Liao B, Wang ML. Investigation of Autism Spectrum Disorders for Preschool Children in Hengyang. Chin Prim Health Care. 2014; 28(3): 92-4	Nonfatal Health Outcomes	Autism spectrum disorders
Deng QW. The Research of Prevalence and Correlative Factors of Autism Disorders Among Preschool Children in Hengyang [dissertation]. Hengyang, China: Nanhua Univeristy, 2014	Nonfatal Health Outcomes	Autism spectrum disorders
China Center for Economic Research, Peking University. China Health and Retirement Longitudinal Study Pilot Resurvey 2012. Beijing, China: China Center for Economic Research, Peking University	Nonfatal Health Outcomes	Urolithiasis
Li QH, Gao WJ, Li JY, Shi LA, Hao XJ, Ge SW, An SH. [Detection of respiratory viruses in children with acute lower respiratory tract infection: an analysis of 5,150 children]. Chin J Contemp Pediatr. 2016; 18(1): 51-4	Nonfatal Health Outcomes	Lower respiratory infections
International Centre for Eye Health (ICEH). China - Sichuan Mianning Rapid Assessment of Avoidable Blindness 2011	Nonfatal Health Outcomes	Age-related macular degeneration
Wang W, Jiang B, Sun H, Ru X, Sun D, Wang L, Wang L, Jiang Y, Li Y, Wang Y, Chen Z, Wu S, Zhang Y, Wang D, Wang Y, Feigin VL; NESS-China investigators. Prevalence, Incidence and Mortality of Stroke in China: Results from a Nationwide Population-Based Survey of 480,687 Adults. Circulation. 2017; 135(8): 759-71	Nonfatal Health Outcomes	Subarachnoid hemorrhage
International Centre for Eye Health (ICEH). China - Sichuan Dechang Rapid Assessment of Avoidable Blindness 2011	Nonfatal Health Outcomes	Age-related macular degeneration
International Centre for Eye Health (ICEH). China - Lancang Rapid Assessment of Avoidable Blindness 2012. Grootebroek, Netherlands: RAAB Repository	Nonfatal Health Outcomes	Trachoma
International Centre for Eye Health (ICEH). China - Jianchuan Rapid Assessment of Avoidable Blindness 2012. Grootebroek, Netherlands: RAAB Repository	Nonfatal Health Outcomes	Trachoma
International Centre for Eye Health (ICEH). China - Sichuan Mianning Rapid Assessment of Avoidable Blindness 2011	Nonfatal Health Outcomes	Refraction disorders
China Center for Economic Research, Peking University. China Health and Retirement Longitudinal Study Pilot Resurvey 2012. Beijing, China: China Center for Economic Research, Peking University	Nonfatal Health Outcomes	Other road injuries
International Centre for Eye Health (ICEH). China - Lancang Rapid Assessment of Avoidable Blindness 2012. Grootebroek, Netherlands: RAAB Repository	Nonfatal Health Outcomes	Age-related macular degeneration
International Centre for Eye Health (ICEH). China - Lancang Rapid Assessment of Avoidable Blindness 2012. Grootebroek, Netherlands: RAAB Repository	Nonfatal Health Outcomes	Glaucoma
International Centre for Eye Health (ICEH). China - Jianchuan Rapid Assessment of Avoidable Blindness 2012. Grootebroek, Netherlands: RAAB Repository	Nonfatal Health Outcomes	Glaucoma
International Centre for Eye Health (ICEH). China - Lancang Rapid Assessment of Avoidable Blindness 2012. Grootebroek, Netherlands: RAAB Repository	Nonfatal Health Outcomes	Cataract
Li J, Zhong H, Cai N, Luo T, Li J, Su X, Li X, Qiu X, Yang Y, Yuan Y, Yu M. The prevalence and causes of visual impairment in an elderly Chinese Bai ethnic rural population: the Yunnan minority eye study. Invest Ophthalmol Vis Sci. 2012; 53(8): 4498-504	Nonfatal Health Outcomes	Refraction disorders
International Centre for Eye Health (ICEH). China - Lancang Rapid Assessment of Avoidable Blindness 2012. Grootebroek, Netherlands: RAAB Repository	Nonfatal Health Outcomes	Refraction and accommodation disorders
International Centre for Eye Health (ICEH). China - Lancang Rapid Assessment of Avoidable Blindness 2012. Grootebroek, Netherlands: RAAB Repository	Nonfatal Health Outcomes	Refraction disorders
International Centre for Eye Health (ICEH). China - Jianchuan Rapid Assessment of Avoidable Blindness 2012. Grootebroek, Netherlands: RAAB Repository	Nonfatal Health Outcomes	Refraction and accommodation disorders
International Centre for Eye Health (ICEH). China - Jianchuan Rapid Assessment of Avoidable Blindness 2012. Grootebroek, Netherlands: RAAB Repository	Nonfatal Health Outcomes	Refraction disorders
International Centre for Eye Health (ICEH). China - Lancang Rapid Assessment of Avoidable Blindness 2012. Grootebroek, Netherlands: RAAB Repository	Nonfatal Health Outcomes	Other vision loss
International Centre for Eye Health (ICEH). China - Jianchuan Rapid Assessment of Avoidable Blindness 2012. Grootebroek, Netherlands: RAAB Repository	Nonfatal Health Outcomes	Other vision loss

Zhou X, Guan H, Zheng L, Li Z, Guo X, Yang H, Yu S, Sun G, Li W, Hu W, Guo L, Pan G, Xing L, Zhang Y, Sun Y. Prevalence and awareness of diabetes mellitus among a rural population in China: results from Liaoning Province. Diabet Med. 2015; 32(3): 332-42	Nonfatal Health Outcomes	Diabetes mellitus
Wang S, Chen R, Liu Q, Shu Z, Zhan S, Li L. Prevalence, awareness and treatment of chronic kidney disease among middle-aged and elderly: The China Health and Retirement Longitudinal Study. Nephrology (Carlton). 2015; 20(7): 474-84	Nonfatal Health Outcomes	Chronic kidney disease
China Center for Economic Research, Peking University. China Health and Retirement Longitudinal Study Pilot Resurvey 2012. Beijing, China: China Center for Economic Research, Peking University	Nonfatal Health Outcomes	Exposure to mechanical forces
International Centre for Eye Health (ICEH). China - Sichuan Mianning Rapid Assessment of Avoidable Blindness 2011	Nonfatal Health Outcomes	Vision loss
China Center for Economic Research, Peking University. China Health and Retirement Longitudinal Study Pilot Resurvey 2012. Beijing, China: China Center for Economic Research, Peking University	Nonfatal Health Outcomes	Inflammatory bowel disease
International Centre for Eye Health (ICEH). China - Sichuan Dechang Rapid Assessment of Avoidable Blindness 2011	Nonfatal Health Outcomes	Refraction disorders
Chinese Center for Disease Control and Prevention (CCDC). China Notifiable Infectious Diseases 2011	Nonfatal Health Outcomes	Chlamydial infection
China Center for Economic Research, Peking University. China Health and Retirement Longitudinal Study Pilot Resurvey 2012. Beijing, China: China Center for Economic Research, Peking University	Nonfatal Health Outcomes	Motorcyclist road injuries
China Center for Economic Research, Peking University. China Health and Retirement Longitudinal Study Pilot Resurvey 2012. Beijing, China: China Center for Economic Research, Peking University	Nonfatal Health Outcomes	Physical violence by other means
Liu YF, Gao Y, Chen MF, Cao B, Yang XH, Wei L. Etiological analysis and predictive diagnostic model building of community-acquired pneumonia in adult outpatients in Beijing, China. BMC Infect Dis. 2013; 309	Nonfatal Health Outcomes	Lower respiratory infections
Yu X, Lu R, Wang Z, Zhu N, Wang W, Julian D, Chris B, Lu J, Lv J, Tan W. Etiology and clinical characterization of respiratory virus infections in adult patients attending an emergency department in Beijing. PLoS One. 2012; 7(2): e32174	Nonfatal Health Outcomes	Lower respiratory infections
Qu J-X, Gu L, Pu Z-H, Yu X-M, Liu Y-M, Li R, Wang Y-M, Cao B, Wang C, Beijing Network for Adult Community-Acquired Pneumonia (BNACAP). Viral etiology of community-acquired pneumonia among adolescents and adults with mild or moderate severity and its relation to age and severity. BMC Infect Dis. 2015; 89	Nonfatal Health Outcomes	Lower respiratory infections
Jin Z, Shi L, Wang Y-J, Yang L-G, Shi Y-H, Shen L-W, Ren C-C. Prevalence of headache among children and adolescents in Shanghai, China. J Clin Neurosci. 2013; 20(1): 117-21	Nonfatal Health Outcomes	Migraine
International Centre for Eye Health (ICEH). China - Lancang Rapid Assessment of Avoidable Blindness 2012. Grootebroek, Netherlands: RAAB Repository	Nonfatal Health Outcomes	
International Centre for Eye Health (ICEH). China - Jianchuan Rapid Assessment of Avoidable Blindness 2012. Grootebroek, Netherlands: RAAB Repository	Nonfatal Health Outcomes	
International Centre for Eye Health (ICEH). China - Lancang Rapid Assessment of Avoidable Blindness 2012. Grootebroek, Netherlands: RAAB Repository	Nonfatal Health Outcomes	Diabetes mellitus
International Centre for Eye Health (ICEH). China - Jianchuan Rapid Assessment of Avoidable Blindness 2012. Grootebroek, Netherlands: RAAB Repository	Nonfatal Health Outcomes	Diabetes mellitus
China Center for Economic Research, Peking University. China Health and Retirement Longitudinal Study Pilot Resurvey 2012. Beijing, China: China Center for Economic Research, Peking University	Nonfatal Health Outcomes	Venomous animal contact
Chinese Center for Disease Control and Prevention (CCDC). China Notifiable Infectious Diseases 2011	Nonfatal Health Outcomes	Gonococcal infection
International Centre for Eye Health (ICEH). China - Lancang Rapid Assessment of Avoidable Blindness 2012. Grootebroek, Netherlands: RAAB Repository	Nonfatal Health Outcomes	Vision loss
International Centre for Eye Health (ICEH). China - Jianchuan Rapid Assessment of Avoidable Blindness 2012. Grootebroek, Netherlands: RAAB Repository	Nonfatal Health Outcomes	Vision loss
China Center for Economic Research, Peking University. China Health and Retirement Longitudinal Study Pilot Resurvey 2012. Beijing, China: China Center for Economic Research, Peking University	Nonfatal Health Outcomes	Physical violence by sharp object
Xu J, Xu L, Du KF, Shao L, Chen CX, Zhou JQ, Wang YX, You QS, Jonas JB, Wei WB. Subfoveal choroidal thickness in diabetes and diabetic retinopathy [Unpublished data]. Ophthalmology. 2013 Oct;120(10):2023-8	Nonfatal Health Outcomes	Chronic kidney disease
China Center for Economic Research, Peking University. China Health and Retirement Longitudinal Study Pilot Resurvey 2012. Beijing, China: China Center for Economic Research, Peking University	Nonfatal Health Outcomes	Polycystic ovarian syndrome

China Center for Economic Research, Peking University. China Health and Retirement Longitudinal Study Pilot Resurvey 2012. Beijing, China: China Center for Economic Research, Peking University	Nonfatal Health Outcomes	Down syndrome
Ju X, Fang Q, Zhang J, Xu A, Liang L, Ke C. Viral etiology of influenza-like illnesses in Huizhou, China, from 2011 to 2013. Arch Virol. 2014; 159(8): 2003-10	Nonfatal Health Outcomes	Influenza
China Center for Economic Research, Peking University. China Health and Retirement Longitudinal Study Pilot Resurvey 2012. Beijing, China: China Center for Economic Research, Peking University	Nonfatal Health Outcomes	Atrial fibrillation and flutter
China Center for Economic Research, Peking University. China Health and Retirement Longitudinal Study Pilot Resurvey 2012. Beijing, China: China Center for Economic Research, Peking University	Nonfatal Health Outcomes	Gastritis and duodenitis
China Center for Economic Research, Peking University. China Health and Retirement Longitudinal Study Pilot Resurvey 2012. Beijing, China: China Center for Economic Research, Peking University	Nonfatal Health Outcomes	Congenital heart anomalies
Zhou X, Bi B, Zheng L, Li Z, Yang H, Song H, Sun Y. The prevalence and risk factors for depression symptoms in a rural Chinese sample population. PLoS One. 2014; 9(6): e99692	Nonfatal Health Outcomes	Major depressive disorder
Guo-ying Ye, Jian-er Chen, Kai-Zhong Shen. Dementia in people over 60 years old and related risk factors. Chin Rural Health Serv Adm. 2011; 31(2): 152	Nonfatal Health Outcomes	Alzheimer's disease and other dementias
Yang H, Li Y, Wu W, Sun Q, Zhang Y, Zhao W, Lv H, Xia Q, Hu P, Li H, Qian J. The incidence of inflammatory bowel disease in Northern China: a prospective population-based study. PLoS One. 2014; 9(7): e101296	Nonfatal Health Outcomes	Inflammatory bowel disease
China Center for Economic Research, Peking University. China Health and Retirement Longitudinal Study Pilot Resurvey 2012. Beijing, China: China Center for Economic Research, Peking University	Nonfatal Health Outcomes	Falls
China Center for Economic Research, Peking University. China Health and Retirement Longitudinal Study Pilot Resurvey 2012. Beijing, China: China Center for Economic Research, Peking University	Nonfatal Health Outcomes	Intracerebral hemorrhage
China Center for Economic Research, Peking University. China Health and Retirement Longitudinal Study Pilot Resurvey 2012. Beijing, China: China Center for Economic Research, Peking University	Nonfatal Health Outcomes	Foreign body in eyes
Wang S, Ma W, Yuan Z, Wang S-M, Yi X, Jia H, Xue F. Association between obesity indices and type 2 diabetes mellitus among middle-aged and elderly people in Jinan, China: a cross-sectional study. BMJ Open. 2016; 6(11): e012742	Nonfatal Health Outcomes	Diabetes mellitus
China Center for Economic Research, Peking University. China Health and Retirement Longitudinal Study Pilot Resurvey 2012. Beijing, China: China Center for Economic Research, Peking University	Nonfatal Health Outcomes	Klinefelter syndrome
Yu X, Kou Y, Xia D, Li J, Yang X, Zhou Y, He X. Human respiratory syncytial virus in children with lower respiratory tract infections or influenza-like illness and its co-infection characteristics with viruses and atypical bacteria in Hangzhou, China. J Clin Virol. 2015; 69: 1-6	Nonfatal Health Outcomes	Lower respiratory infections
China Center for Economic Research, Peking University. China Health and Retirement Longitudinal Study Pilot Resurvey 2012. Beijing, China: China Center for Economic Research, Peking University	Nonfatal Health Outcomes	Uterine fibroids
Huo X, Qin Y, Qi X, Zu R, Tang F, Li L, Hu Z, Zhu F. Surveillance of 16 respiratory viruses in patients with influenza-like illness in Nanjing, China. J Med Virol. 2012; 84(12): 1980-4	Nonfatal Health Outcomes	Influenza
Chen K, Jia R, Li L, Yang C, Shi Y. The aetiology of community associated pneumonia in children in Nanjing, China and aetiological patterns associated with age and season. BMC Public Health. 2015; 113	Nonfatal Health Outcomes	Influenza
Huo X, Qin Y, Qi X, Zu R, Tang F, Li L, Hu Z, Zhu F. Surveillance of 16 respiratory viruses in patients with influenza-like illness in Nanjing, China. J Med Virol. 2012; 84(12): 1980-4	Nonfatal Health Outcomes	Respiratory syncytial virus pneumonia
Chen K, Jia R, Li L, Yang C, Shi Y. The aetiology of community associated pneumonia in children in Nanjing, China and aetiological patterns associated with age and season. BMC Public Health. 2015; 113	Nonfatal Health Outcomes	Respiratory syncytial virus pneumonia
Lu L, Yan Y, Yang B, Xiao Z, Feng X, Wang Y, Ji W, Mize M, Hao C, Chen Z. Epidemiological and clinical profiles of respiratory syncytial virus infection in hospitalized neonates in Suzhou, China. BMC Infect Dis. 2015; 15: 431	Nonfatal Health Outcomes	Respiratory syncytial virus pneumonia
Pan American Health Organization (PAHO). Number of Reported Cases of Dengue and Severe Dengue (DS) in the Americas, by Country 2011. Washington, D.C., United States: Pan American Health Organization (PAHO), 2012	Nonfatal Health Outcomes	Dengue
China Center for Economic Research, Peking University. China Health and Retirement Longitudinal Study Pilot Resurvey 2012. Beijing, China: China Center for Economic Research, Peking University	Nonfatal Health Outcomes	Myocarditis

Yu X, Kou Y, Xia D, Li J, Yang X, Zhou Y, He X. Human respiratory syncytial virus in children with lower respiratory tract infections or influenza-like illness and its co-infection characteristics with viruses and atypical bacteria in Hangzhou, China. J Clin Virol. 2015; 69: 1-6	Nonfatal Health Outcomes	Respiratory syncytial virus pneumonia
Qu Y, Jiang H, Zhang N, Wang D, Guo L. Prevalence of Mental Disorders in 6Â–16-Year-Old Students in Sichuan Province, China. Int J Environ Res Public Health. 2015; 12(5): 5090Â–107	Nonfatal Health Outcomes	Anxiety disorders
Qiu P, Caine E-D, Hou F, Cerulli C, Wittink M-N, Li J. The Prevalence of Distress and Depression among Women in Rural Sichuan Province. PLoS One. 2016; 11(8): e0161099	Nonfatal Health Outcomes	Anxiety disorders
Bellizzi S, Ali MM, Abalos E, Betran AP, Kapila J, Pileggi-Castro C, Vogel JP, Merialdi M. Are hypertensive disorders in pregnancy associated with congenital malformations in offspring? Evidence from the WHO Multicountry cross sectional survey on maternal and newborn health. BMC Pregnancy Childbirth. 2016; 16(1): 198	Nonfatal Health Outcomes	Congenital musculoskeletal and limb anomalies
Wang X, Xing Y, Sun J, Zhou H, Yu H, Zhao Y, Yan S. Prevalence, Associated Factors, and Impact on Quality of Life of Migraine in a Community in Northeast China. J Oral Facial Pain Headache. 2016; 30(2): 139-49	Nonfatal Health Outcomes	Migraine
Luo XL, Zhang WY. Obstetrical disease spectrum in China: an epidemiological study of 111,767 cases in 2011. Chin Med J (Engl). 2015; 128(9): 1137-46	Nonfatal Health Outcomes	Maternal disorders
China Center for Economic Research, Peking University. China Health and Retirement Longitudinal Study Pilot Resurvey 2012. Beijing, China: China Center for Economic Research, Peking University	Nonfatal Health Outcomes	Cystic echinococcosis
Lin M, Han ZJ, Wang Q, Zheng L, Wang Y, Yang H, Huang Y, Lin F, Zhan XF, Lin CP, Wu JR, Luo ZY, Liu JB, Yan ZH, Zheng SY, Zheng JK, Lu M, Zhu JJ, Xie LX, Yang LY. Molecular epidemiological survey of hemoglobinopathies in the Wuxi region of Jiangsu Province, eastern China. Hemoglobin. 2013; 37(5): 454-66	Nonfatal Health Outcomes	Thalassemias
Li W, Hussein Musa T, Gao R, Li XS, Wang WX, Hong L, Wei PM. Association between BMI and Dental Caries among School Children and Adolescents in Jiangsu Province, China. Biomed Environ Sci. 2017; 30(10): 758-761	Nonfatal Health Outcomes	Caries of permanent teeth
China Center for Economic Research, Peking University. China Health and Retirement Longitudinal Study Pilot Resurvey 2012. Beijing, China: China Center for Economic Research, Peking University	Nonfatal Health Outcomes	Other transport injuries
China Center for Economic Research, Peking University. China Health and Retirement Longitudinal Study Pilot Resurvey 2012. Beijing, China: China Center for Economic Research, Peking University	Nonfatal Health Outcomes	Meningitis
China Center for Economic Research, Peking University. China Health and Retirement Longitudinal Study Pilot Resurvey 2012. Beijing, China: China Center for Economic Research, Peking University	Nonfatal Health Outcomes	Neonatal encephalopathy due to birth asphyxia and trauma
China Center for Economic Research, Peking University. China Health and Retirement Longitudinal Study Pilot Resurvey 2012. Beijing, China: China Center for Economic Research, Peking University	Nonfatal Health Outcomes	Neonatal preterm birth
China Center for Economic Research, Peking University. China Health and Retirement Longitudinal Study Pilot Resurvey 2012. Beijing, China: China Center for Economic Research, Peking University	Nonfatal Health Outcomes	Self-harm
China Center for Economic Research, Peking University. China Health and Retirement Longitudinal Study Pilot Resurvey 2012. Beijing, China: China Center for Economic Research, Peking University	Nonfatal Health Outcomes	Unintentional firearm injuries
Lin H, Li Q, Liu X, Ma H, Xia M, Wang D, Li X, Wu J, Zhao N, Pan B, Gao X. Liver Fat Content Is Associated with Elevated Serum Uric Acid in the Chinese Middle-Aged and Elderly Populations: Shanghai Changfeng Study. PLoS One. 2015; 10(10): e0140379	Nonfatal Health Outcomes	Cirrhosis due to NASH
China Center for Economic Research, Peking University. China Health and Retirement Longitudinal Study Pilot Resurvey 2012. Beijing, China: China Center for Economic Research, Peking University	Nonfatal Health Outcomes	Other unintentional injuries
Wang X, Wei Z, Li Q, Mei L. A longitudinal study of early childhood caries incidence in Wenzhou preschool children. BMC Oral Health. 2017; 17(1): 105	Nonfatal Health Outcomes	Caries of deciduous teeth
China Center for Economic Research, Peking University. China Health and Retirement Longitudinal Study Pilot Resurvey 2012. Beijing, China: China Center for Economic Research, Peking University	Nonfatal Health Outcomes	Neural tube defects
China Center for Economic Research, Peking University. China Health and Retirement Longitudinal Study Pilot Resurvey 2012. Beijing, China: China Center for Economic Research, Peking University	Nonfatal Health Outcomes	Self-harm by firearm
China Center for Economic Research, Peking University. China Health and Retirement Longitudinal Study Pilot Resurvey 2012. Beijing, China: China Center for Economic Research, Peking University	Nonfatal Health Outcomes	Asbestosis

China Center for Economic Research, Peking University. China Health and Retirement Longitudinal Study Pilot Resurvey 2012. Beijing, China: China Center for Economic Research, Peking University	Nonfatal Health Outcomes	Maternal abortion, miscarriage, and ectopic pregnancy
China Center for Economic Research, Peking University. China Health and Retirement Longitudinal Study Pilot Resurvey 2012. Beijing, China: China Center for Economic Research, Peking University	Nonfatal Health Outcomes	Paralytic ileus and intestinal obstruction
Xue L, Lou Y, Feng X, Wang C, Ran Z, Zhang X. Prevalence of chronic kidney disease and associated factors among the Chinese population in Taian, China. BMC Nephrol. 2014; 15: 205	Nonfatal Health Outcomes	Chronic kidney disease
China Center for Economic Research, Peking University. China Health and Retirement Longitudinal Study Pilot Resurvey 2012. Beijing, China: China Center for Economic Research, Peking University	Nonfatal Health Outcomes	Physical violence by firearm
Xia Z, Wang Z, Cai Q, Yang J, Zhang X, Yang T. Prevalence and risk factors of type 2 diabetes in the adults in haikou city, hainan island, china. Iran J Public Health. 2013; 42(3): 222-30	Nonfatal Health Outcomes	Diabetes mellitus
China Center for Economic Research, Peking University. China Health and Retirement Longitudinal Study Pilot Resurvey 2012. Beijing, China: China Center for Economic Research, Peking University	Nonfatal Health Outcomes	Urinary tract infections
China Center for Economic Research, Peking University. China Health and Retirement Longitudinal Study Pilot Resurvey 2012. Beijing, China: China Center for Economic Research, Peking University	Nonfatal Health Outcomes	Appendicitis
China Center for Economic Research, Peking University. China Health and Retirement Longitudinal Study Pilot Resurvey 2012. Beijing, China: China Center for Economic Research, Peking University	Nonfatal Health Outcomes	Cellulitis
China Center for Economic Research, Peking University. China Health and Retirement Longitudinal Study Pilot Resurvey 2012. Beijing, China: China Center for Economic Research, Peking University	Nonfatal Health Outcomes	Other exposure to mechanical forces
China Center for Economic Research, Peking University. China Health and Retirement Longitudinal Study Pilot Resurvey 2012. Beijing, China: China Center for Economic Research, Peking University	Nonfatal Health Outcomes	Endometriosis
International Centre for Eye Health (ICEH). China - Sichuan Dechang Rapid Assessment of Avoidable Blindness 2011	Nonfatal Health Outcomes	Glaucoma
China Center for Economic Research, Peking University. China Health and Retirement Longitudinal Study Pilot Resurvey 2012. Beijing, China: China Center for Economic Research, Peking University	Nonfatal Health Outcomes	Animal contact
China Center for Economic Research, Peking University. China Health and Retirement Longitudinal Study Pilot Resurvey 2012. Beijing, China: China Center for Economic Research, Peking University	Nonfatal Health Outcomes	Maternal hypertensive disorders
China Center for Economic Research, Peking University. China Health and Retirement Longitudinal Study Pilot Resurvey 2012. Beijing, China: China Center for Economic Research, Peking University	Nonfatal Health Outcomes	Gallbladder and biliary diseases
China Center for Economic Research, Peking University. China Health and Retirement Longitudinal Study Pilot Resurvey 2012. Beijing, China: China Center for Economic Research, Peking University	Nonfatal Health Outcomes	Ischemic stroke
China Center for Economic Research, Peking University. China Health and Retirement Longitudinal Study Pilot Resurvey 2012. Beijing, China: China Center for Economic Research, Peking University	Nonfatal Health Outcomes	Neonatal sepsis and other neonatal infections
China Center for Economic Research, Peking University. China Health and Retirement Longitudinal Study Pilot Resurvey 2012. Beijing, China: China Center for Economic Research, Peking University	Nonfatal Health Outcomes	Adverse effects of medical treatment
China Center for Economic Research, Peking University. China Health and Retirement Longitudinal Study Pilot Resurvey 2012. Beijing, China: China Center for Economic Research, Peking University	Nonfatal Health Outcomes	Fire, heat, and hot substances
China Center for Economic Research, Peking University. China Health and Retirement Longitudinal Study Pilot Resurvey 2012. Beijing, China: China Center for Economic Research, Peking University	Nonfatal Health Outcomes	Silicosis
China Center for Economic Research, Peking University. China Health and Retirement Longitudinal Study Pilot Resurvey 2012. Beijing, China: China Center for Economic Research, Peking University	Nonfatal Health Outcomes	Genital prolapse
Wang GX, Li X, Fang LY, Xu H. Correlation analysis and investigation of autism spectrum disorder in 2-6 years old children of Zaozhuang. Chin J Child Health Care. 2015; 23(12): 1322-1324	Nonfatal Health Outcomes	Autism spectrum disorders
China Center for Economic Research, Peking University. China Health and Retirement Longitudinal Study Pilot Resurvey 2012. Beijing, China: China Center for Economic Research, Peking University	Nonfatal Health Outcomes	Rheumatic heart disease

Lin Y, Fu Y, Xu M, Su L, Cao L, Xu J, Cheng X. Evaluation of a PCR/ESI-MS platform to identify respiratory viruses from nasopharyngeal aspirates. J Med Virol. 2015; 87.0(11): 1867-71	Nonfatal Health Outcomes	Influenza
Zhang X, Liu L, Shi P, Jiang G, Jia P, Wang C, Wang L, Qian L. [Risk factors for acute respiratory syncytial virus infection of lower respiratory tract in hospitalized infants]. Chin J Pediatr. 2014; 52(5): 373-7	Nonfatal Health Outcomes	Respiratory syncytial virus pneumonia
Lin Y, Fu Y, Xu M, Su L, Cao L, Xu J, Cheng X. Evaluation of a PCR/ESI-MS platform to identify respiratory viruses from nasopharyngeal aspirates. J Med Virol. 2015; 87.0(11): 1867-71	Nonfatal Health Outcomes	Respiratory syncytial virus pneumonia
Ma C, Wu S, Yang P, Li H, Tang S, Wang Q. Behavioural factors associated with diarrhea among adults over 18 years of age in Beijing, China. BMC Public Health. 2014; 14: 451	Nonfatal Health Outcomes	Diarrheal diseases
Qu J-X, Gu L, Pu Z-H, Yu X-M, Liu Y-M, Li R, Wang Y-M, Cao B, Wang C, Beijing Network for Adult Community-Acquired Pneumonia (BNACAP). Viral etiology of community-acquired pneumonia among adolescents and adults with mild or moderate severity and its relation to age and severity. BMC Infect Dis. 2015; 89	Nonfatal Health Outcomes	Influenza
Yu X, Lu R, Wang Z, Zhu N, Wang W, Julian D, Chris B, Lu J, Lv J, Tan W. Etiology and clinical characterization of respiratory virus infections in adult patients attending an emergency department in Beijing. PLoS One. 2012; 7(2): e32174	Nonfatal Health Outcomes	Respiratory syncytial virus pneumonia
Qu J-X, Gu L, Pu Z-H, Yu X-M, Liu Y-M, Li R, Wang Y-M, Cao B, Wang C, Beijing Network for Adult Community-Acquired Pneumonia (BNACAP). Viral etiology of community-acquired pneumonia among adolescents and adults with mild or moderate severity and its relation to age and severity. BMC Infect Dis. 2015; 89	Nonfatal Health Outcomes	Respiratory syncytial virus pneumonia
Zhang X, Liu L, Shi P, Jiang G, Jia P, Wang C, Wang L, Qian L. [Risk factors for acute respiratory syncytial virus infection of lower respiratory tract in hospitalized infants]. Chin J Pediatr. 2014; 52(5): 373-7	Nonfatal Health Outcomes	Lower respiratory infections
International Centre for Eye Health (ICEH). China - Sichuan Dechang Rapid Assessment of Avoidable Blindness 2011	Nonfatal Health Outcomes	Diabetes mellitus
International Centre for Eye Health (ICEH). China - Sichuan Mianning Rapid Assessment of Avoidable Blindness 2011	Nonfatal Health Outcomes	Diabetes mellitus
China Center for Economic Research, Peking University. China Health and Retirement Longitudinal Study Pilot Resurvey 2012. Beijing, China: China Center for Economic Research, Peking University	Nonfatal Health Outcomes	Other pneumoconiosis
China Center for Economic Research, Peking University. China Health and Retirement Longitudinal Study Pilot Resurvey 2012. Beijing, China: China Center for Economic Research, Peking University	Nonfatal Health Outcomes	Drowning
Shan Z, Deng G, Li J, Li Y, Zhang Y, Zhao Q. Correlational analysis of neck/shoulder pain and low back pain with the use of digital products, physical activity and psychological status among adolescents in Shanghai. PLoS One. 2013; 8(10): e78109	Nonfatal Health Outcomes	Low back and neck pain
Huang C, Liu W, Hu Y, Zou Z, Zhao Z, Shen L, Weschler LB, Sundell J. Updated prevalences of asthma, allergy, and airway symptoms, and a systematic review of trends over time for childhood asthma in Shanghai, China. PLoS One. 2015; 10(4): e0121577	Nonfatal Health Outcomes	Asthma
Huang C, Liu W, Hu Y, Zou Z, Zhao Z, Shen L, Weschler LB, Sundell J. Updated prevalences of asthma, allergy, and airway symptoms, and a systematic review of trends over time for childhood asthma in Shanghai, China. PLoS One. 2015; 10(4): e0121577	Nonfatal Health Outcomes	Dermatitis
Zhu WJ, Ma HX, Cui HY, Lu X, Shao MJ, Li S, Luo YQ, Wang Q, Xu CY, Xu DQ, Liu CH, Chen YZ. Prevalence and Treatment of Children's Asthma in Rural Areas Compared with Urban Areas in Beijing. Chin Med J (Engl). 2015; 128(17): 2273-7	Nonfatal Health Outcomes	Asthma
Xu J, Xu L, Du KF, Shao L, Chen CX, Zhou JQ, Wang YX, You QS, Jonas JB, Wei WB. Subfoveal choroidal thickness in diabetes and diabetic retinopathy [Unpublished data]. Ophthalmology. 2013 Oct;120(10):2023-8	Nonfatal Health Outcomes	Asthma
Cheng Q, Sun H-X, Ye F-L, Wang G, Ling H-W, Chen S-D, Jiang G-X. Dementia among elderly in Shanghai suburb: a rural community survey. J Alzheimers Dis. 2014; 39(4): 883Â–9	Nonfatal Health Outcomes	Alzheimer's disease and other dementias
Xu X, Xiao S, Rahardjo TB, Hogervorst E. Risk Factors for Possible Dementia Using the Hopkins Verbal Learning Test and the Mini-Mental State Examination in Shanghai. Diagnostics (Basel). 2015; 5(4): 487-96	Nonfatal Health Outcomes	Alzheimer's disease and other dementias
Xiao S, Lewis M, Mellor D, McCabe M, Byrne L, Wang T, Wang J, Zhu M, Cheng Y, Yang C, Dong S. The China longitudinal ageing study: overview of the demographic, psychosocial and cognitive data of the Shanghai sample. J Ment Health. 2016; 25(2): 131-6	Nonfatal Health Outcomes	Alzheimer's disease and other dementias

Ban CX, Zhong L, Wang T, Zhu MJ, Wang JH, Zhang ZL, Wang Z, Su N, Liu YY, Shi YC, Xiao SF, Li X. Enhanced Diabetes Susceptibility in Community Dwelling Han Elders Carrying the Apolipoprotein E 3/3 Genotype. PLoS One. 2016; 11(3): e0151336	Nonfatal Health Outcomes	Alzheimer's disease and other dementias
Chen K, Jia R, Li L, Yang C, Shi Y. The aetiology of community associated pneumonia in children in Nanjing, China and aetiological patterns associated with age and season. BMC Public Health. 2015; 113	Nonfatal Health Outcomes	Lower respiratory infections
Huo X, Qin Y, Qi X, Zu R, Tang F, Li L, Hu Z, Zhu F. Surveillance of 16 respiratory viruses in patients with influenza-like illness in Nanjing, China. J Med Virol. 2012; 84(12): 1980-4	Nonfatal Health Outcomes	Lower respiratory infections
Xin Z, Yuan M-X, Li H-X, Hua L, Feng J-P, Shi J, Zhu X-R, Cao X, Yang J-K. Evaluation for fasting and 2-hour glucose and HbA1c for diagnosing diabetes based on prevalence of retinopathy in a Chinese population. PLoS One. 2012; 7(7): e40610	Nonfatal Health Outcomes	Diabetes mellitus
Shan Z, Deng G, Li J, Li Y, Zhang Y, Zhao Q. Correlational analysis of neck/shoulder pain and low back pain with the use of digital products, physical activity and psychological status among adolescents in Shanghai. PLoS One. 2013; 8(10): e78109	Nonfatal Health Outcomes	Low back pain
Shan Z, Deng G, Li J, Li Y, Zhang Y, Zhao Q. Correlational analysis of neck/shoulder pain and low back pain with the use of digital products, physical activity and psychological status among adolescents in Shanghai. PLoS One. 2013; 8(10): e78109	Nonfatal Health Outcomes	Neck pain
Huang C, Liu W, Hu Y, Zou Z, Zhao Z, Shen L, Weschler LB, Sundell J. Updated prevalences of asthma, allergy, and airway symptoms, and a systematic review of trends over time for childhood asthma in Shanghai, China. PLoS One. 2015; 10(4): e0121577	Nonfatal Health Outcomes	Atopic dermatitis
Du Q, Yu M, Li Y, Du H, Gao W, Mei H, Liu S. Permanent caries experience is associated with primary caries experience: a 7-year longitudinal study in China. Community Dent Oral Epidemiol. 2016	Nonfatal Health Outcomes	Caries of permanent teeth
Fan C, Wang W, Xu T, Zheng S. Risk factors of early childhood caries among children in Beijing: a case-control study. BMC Oral Health. 2016; 16(1): 98	Nonfatal Health Outcomes	Caries of deciduous teeth
Xiong X-Z. Epidemiological investigation on severe mental disorders in Ziyang city Sichuan province. Med Innov China. 2013; 10(5): 117-9	Nonfatal Health Outcomes	Schizophrenia
Center for Health Statistics and Information, National Health and Family Planning Commission (China), Shanghai Municipal Center for Disease Control and Prevention (Shanghai CDC), Shanghai Health Information Center. China Hospital Inpatient Data 2013-2016	Nonfatal Health Outcomes	Guillain-BarrÃ© syndrome
Wang YJ, Wang WR. Analysis on Application of DSM-V in Early Screening of Autism Spectrum Disorder Children. Matern Child Health Care China. 2015; 13: 2011-4	Nonfatal Health Outcomes	Autism spectrum disorders
Guan WD, Gong XY, Mok CK, Chen TT, Wu SG, Pan SH, Cowling BJ, Yang ZF, Chen de H. Surveillance for seasonal influenza virus prevalence in hospitalized children with lower respiratory tract infection in Guangzhou, China during the post-pandemic era. PLoS One. 2015; 10(4): e0120983	Nonfatal Health Outcomes	Lower respiratory infections
Li EY, Liu Y, Zhan X, Liang YB, Zhang X, Zheng C, Jhanji V, Xu P, Chang DF, Lam DS. Prevalence of blindness and outcomes of cataract surgery in Hainan Province in South China. Ophthalmology. 2013; 120(11.0): 2176-83	Nonfatal Health Outcomes	Refraction disorders
Zhang, T, Yang, Y, Yu, F, Zhao, Y, Lin, F, Minhas, V, Wood, C, He, N. Kaposi`s sarcoma associated herpesvirus infection among female sex workers and general population women in Shanghai, China: a cross-sectional study. BMC Infect Dis. 2014; 58	Nonfatal Health Outcomes	Syphilis
Sun J, Liu D, Liu Z, Li W, Li G, Zhang J. [Epidemiology of rheumatoid arthritis in middle-aged and elderly population in Luohe City, Henan Province]. Chin Med J (Taipei). 2013; 93(41): 3309Â–11	Nonfatal Health Outcomes	Rheumatoid arthritis
Zhao Q-M, Ma X-J, Jia B, Huang G-Y. Prevalence of congenital heart disease at live birth: an accurate assessment by echocardiographic screening. Acta Paediatr. 2013; 102(4): 397-402	Nonfatal Health Outcomes	Congenital heart anomalies
Zhao Q-M, Ma X-J, Jia B, Huang G-Y. Prevalence of congenital heart disease at live birth: an accurate assessment by echocardiographic screening. Acta Paediatr. 2013; 102(4): 397-402	Nonfatal Health Outcomes	Congenital birth defects
Li Y, Huang M-X, Yu Q, Zhao B. Investigation of Children and Adolescents with ADHD Combined with Conduct Disorder. J Int Psychiatry. 2015; 42(5): 32-5	Nonfatal Health Outcomes	Attention-deficit/hyperactivity disorder
Lu K, Chen J, Wu S, Chen J, Hu D. Interaction of Sleep Duration and Sleep Quality on Hypertension Prevalence in Adult Chinese Males. J Epidemiol. 2015; 25(6): 415Â–22	Nonfatal Health Outcomes	Diabetes mellitus
Song N, Shamssain M, Mohammed S, Zhang J, Wu J, Fu C, Hao S, Guan J, Yan X. Prevalence, severity and risk factors of asthma, rhinitis and eczema in a large group of Chinese schoolchildren. J Asthma. 2014; 51(3): 232-42	Nonfatal Health Outcomes	Atopic dermatitis

Sun K, Lu J, Jiang Y, Xu M, Xu Y, Zhang J, Xu B, Sun J, Sun W, Ren C, Liu J, Wang W, Bi Y, Ning G. Low serum potassium level is associated with nonalcoholic fatty liver disease and its related metabolic disorders. Clin Endocrinol (Oxf). 2014; 80(3): 348Â–55	Nonfatal Health Outcomes	Cirrhosis due to NASH
Sun W, Zhang D, Sun J, Xu B, Sun K, Wang T, Ren C, Li J, Chen Y, Xu M, Bi Y, Xu Q, Wang W, Gu Y, Ning G. Association between non-alcoholic fatty liver disease and autonomic dysfunction in a Chinese population. QJM. 2015; 108(8): 617-24	Nonfatal Health Outcomes	Cirrhosis due to NASH
Yang Z, Zhang H, Niu Y, Zhang W, Zhu L, Li X, Lu S, Fan J, Li X, Ning G, Qin L, Su Q. Circulating periostin in relation to insulin resistance and nonalcoholic fatty liver disease among overweight and obese subjects. Sci Rep. 2016; 6: 37886	Nonfatal Health Outcomes	Cirrhosis due to NASH
Sun X, Allison C, Matthews FE, Zhang Z, Auyeung B, Baron-Cohen S, Brayne C. Exploring the Underdiagnosis and Prevalence of Autism Spectrum Conditions in Beijing. Autism Res. 2015; 8(3): 250Â–60	Nonfatal Health Outcomes	Autism spectrum disorders
Wei Q, Sun J, Huang J, Zhou HY, Ding YM, Tao YC, He SM, Liu YL, Niu JQ. Prevalence of hypertension and associated risk factors in Dehui City of Jilin Province in China. J Hum Hypertens. 2015; 29(1): 64Â–8	Nonfatal Health Outcomes	Diabetes mellitus
Bellizzi S, Ali MM, Abalos E, Betran AP, Kapila J, Pileggi-Castro C, Vogel JP, Merialdi M. Are hypertensive disorders in pregnancy associated with congenital malformations in offspring? Evidence from the WHO Multicountry cross sectional survey on maternal and newborn health. BMC Pregnancy Childbirth. 2016; 16(1): 198	Nonfatal Health Outcomes	Congenital birth defects
Lu B, Hu J, Wen J, Zhang Z, Zhou L, Li Y, Hu R. Determination of peripheral neuropathy prevalence and associated factors in Chinese subjects with diabetes and pre-diabetes - ShangHai Diabetic neuRopathy Epidemiology and Molecular Genetics Study (SH-DREAMS). PLoS One. 2013; 8(4): e61053	Nonfatal Health Outcomes	Diabetes mellitus
Zhu YC, Yang HX, Wei YM, Zhu WW, Meng WY, Wang YQ, Shang LX, Cai ZY, Ji LP, Wang YF, Sun Y, Liu JX, Wei L, Sun YF, Zhang XY, Luo TX, Chen HX, Yu LJ. Analysis of correlation factors and pregnancy outcomes of hypertensive disorders of pregnancy - a secondary analysis of a random sampling in Beijing, China. J Matern Fetal Neonatal Med. 2017; 30(6): 751-754	Nonfatal Health Outcomes	Maternal hypertensive disorders
Song N, Shamssain M, Mohammed S, Zhang J, Wu J, Fu C, Hao S, Guan J, Yan X. Prevalence, severity and risk factors of asthma, rhinitis and eczema in a large group of Chinese schoolchildren. J Asthma. 2014; 51(3): 232-42	Nonfatal Health Outcomes	Dermatitis
Lu L, Yan Y, Yang B, Xiao Z, Feng X, Wang Y, Ji W, Mize M, Hao C, Chen Z. Epidemiological and clinical profiles of respiratory syncytial virus infection in hospitalized neonates in Suzhou, China. BMC Infect Dis. 2015; 15: 431	Nonfatal Health Outcomes	Lower respiratory infections
Lin L, Lu J, Chen L, Mu Y, Ye Z, Liu C, Chen G, Shi L, Zhao J, Li Q, Yang T, Yan L, Wan Q, Wu S, Liu Y, Wang G, Luo Z, Tang X, Huo Y, Gao Z, Su Q, Wang Y, Qin G, Deng H, Yu X, Shen F, Chen L, Zhao L, Xu Y, Xu M, Peng K, Du R, Dai M, Li M, Wang T, Zhao Z, Lai S, Li D, Bi Y, Wang W, Ning G, REACTION Study Group. Glycemic status and chronic kidney disease in Chinese adults: Findings from the REACTION study. J Diabetes. 2017; 9(9): 837-845	Nonfatal Health Outcomes	Chronic kidney disease
Hou F, Cerulli C, Wittink MN, Caine ED, Qiu P. Depression, social support and associated factors among women living in rural China: a cross-sectional study. BMC Womens Health. 2015; 15: 28	Nonfatal Health Outcomes	Major depressive disorder
Qiu P, Caine E-D, Hou F, Cerulli C, Wittink M-N, Li J. The Prevalence of Distress and Depression among Women in Rural Sichuan Province. PLoS One. 2016; 11(8): e0161099	Nonfatal Health Outcomes	Major depressive disorder
Qu Y, Jiang H, Zhang N, Wang D, Guo L. Prevalence of Mental Disorders in 6Â–16-Year-Old Students in Sichuan Province, China. Int J Environ Res Public Health. 2015; 12(5): 5090Â–107	Nonfatal Health Outcomes	Dysthymia
Qiu P, Caine E-D, Hou F, Cerulli C, Wittink M-N, Li J. The Prevalence of Distress and Depression among Women in Rural Sichuan Province. PLoS One. 2016; 11(8): e0161099	Nonfatal Health Outcomes	Bipolar disorder
Qu Y, Jiang H, Zhang N, Wang D, Guo L. Prevalence of Mental Disorders in 6Â–16-Year-Old Students in Sichuan Province, China. Int J Environ Res Public Health. 2015; 12(5): 5090Â–107	Nonfatal Health Outcomes	Anorexia nervosa
Qu Y, Jiang H, Zhang N, Wang D, Guo L. Prevalence of Mental Disorders in 6Â–16-Year-Old Students in Sichuan Province, China. Int J Environ Res Public Health. 2015; 12(5): 5090Â–107	Nonfatal Health Outcomes	Bulimia nervosa
Qu Y, Jiang H, Zhang N, Wang D, Guo L. Prevalence of Mental Disorders in 6Â–16-Year-Old Students in Sichuan Province, China. Int J Environ Res Public Health. 2015; 12(5): 5090Â–107	Nonfatal Health Outcomes	Attention-deficit/hyperactivity disorder
Fang M, Yang Y, Wang X-Q, Tang Y, Zhuang X-L. Diagnosis of puerile attention deficit hyperactivity disorder in Xichang City, Liangshan Yi Autonomous Prefecture, Sichuan Privince. Chin J Obstet Gynecol Pediatr (Electron Ed). 2012; 8(6): 721-724	Nonfatal Health Outcomes	Attention-deficit/hyperactivity disorder

Qu Y, Jiang H, Zhang N, Wang D, Guo L. Prevalence of Mental Disorders in 6Â–16-Year-Old Students in Sichuan Province, China. Int J Environ Res Public Health. 2015; 12(5): 5090Â–107	Nonfatal Health Outcomes	Conduct disorder
Joint United Nations Program on HIV/AIDS (UNAIDS), United Nations Children's Fund (UNICEF), World Health Organization (WHO). China Global AIDS Response Progress Reporting (GARPR) System - Antenatal Care Attendees Positive for Syphilis	Nonfatal Health Outcomes	Sexually transmitted infections excluding HIV
Liu Y, Li G, Zhang W. Effect of fetal gender on pregnancy outcomes in Northern China. J Matern Fetal Neonatal Med. 2017; 30(7): 858-863	Nonfatal Health Outcomes	Neonatal preterm birth
Yu X, Lu R, Wang Z, Zhu N, Wang W, Julian D, Chris B, Lu J, Lv J, Tan W. Etiology and clinical characterization of respiratory virus infections in adult patients attending an emergency department in Beijing. PLoS One. 2012; 7(2): e32174	Nonfatal Health Outcomes	Influenza
Tian J, Wang L, Zhang X. Prevalence and Influencing Factors of Autism Spectrum Disorders in Public Primary School. Chin J Clin Psychol. 2016; 24(2): 277Â–81	Nonfatal Health Outcomes	Autism spectrum disorders
Xiaoli Y, Chao J, Wen P, Wenming X, Fang L, Ning L, Huijuan M, Jun N, Ming L, Xiaoxia A, Chuanyou Y, Zenguo F, Lili L, Lianzheng Y, Lijuan T, Guowei P. Prevalence of psychiatric disorders among children and adolescents in northeast China. PLoS One. 2014; 9(10): e111223	Nonfatal Health Outcomes	Attention-deficit/hyperactivity disorder
Li Y, An Z, Yin D, Liu Y, Huang Z, Xu J, Ma Y, Tu Q, Li Q, Wang H. Disease Burden Due to Herpes Zoster among Population Aged >=50 Years Old in China: A Community Based Retrospective Survey. PLoS One. 2016; 11(4): e0152660	Nonfatal Health Outcomes	Varicella and herpes zoster
Liu L, Zhou C, Du H, Zhang K, Huang D, Wu J. The prevalences of impaired fasting glucose and diabetes mellitus in working age men of North China: Anshan Worker Health Survey. Sci Rep. 2014; 4835	Nonfatal Health Outcomes	Diabetes mellitus
Yu S, Guo X, Yang H, Zheng L, Sun Y. An update on the prevalence of metabolic syndrome and its associated factors in rural northeast China. BMC Public Health. 2014; 14: 877	Nonfatal Health Outcomes	Diabetes mellitus
Feng RN, Du SS, Wang C, Li YC, Liu LY, Guo FC, Sun CH. Lean-non-alcoholic fatty liver disease increases risk for metabolic disorders in a normal weight Chinese population. World J Gastroenterol. 2014; 20(47): 17932-40	Nonfatal Health Outcomes	Cirrhosis due to NASH
Liu J, Wang Q, Ji X, Guo S, Dai Y, Zhang Z, Jia L, Shi Y, Tai S, Lee Y. Prevalence of Ureaplasma urealyticum, Mycoplasma hominis, Chlamydia trachomatis infections, and semen quality in infertile and fertile men in China. Urology. 2014; 83(4): 795Â–9	Nonfatal Health Outcomes	Chlamydial infection
Xiaoli Y, Chao J, Wen P, Wenming X, Fang L, Ning L, Huijuan M, Jun N, Ming L, Xiaoxia A, Chuanyou Y, Zenguo F, Lili L, Lianzheng Y, Lijuan T, Guowei P. Prevalence of psychiatric disorders among children and adolescents in northeast China. PLoS One. 2014; 9(10): e111223	Nonfatal Health Outcomes	Conduct disorder
Lin Y, Fu Y, Xu M, Su L, Cao L, Xu J, Cheng X. Evaluation of a PCR/ESI-MS platform to identify respiratory viruses from nasopharyngeal aspirates. J Med Virol. 2015; 87.0(11): 1867-71	Nonfatal Health Outcomes	Lower respiratory infections
World Health Organization (WHO). WHO Global Health Observatory Interactive Graph - Number of Cases of Visceral Leishmaniasis Reported 2013. Geneva, Switzerland: World Health Organization (WHO)	Nonfatal Health Outcomes	Visceral leishmaniasis
World Health Organization (WHO). WHO Global Health Observatory - Visceral Leishmaniasis: Number of Reported Cases by Country. Geneva, Switzerland: World Health Organization (WHO)	Nonfatal Health Outcomes	Cutaneous and mucocutaneous leishmaniasis
Pan American Health Organization (PAHO). Number of Reported Cases of Dengue and Severe Dengue (DS) in the Americas, by Country 2012. Washington, D.C., United States: Pan American Health Organization (PAHO), 2013	Nonfatal Health Outcomes	Dengue
Pan American Health Organization (PAHO). Number of Reported Cases of Dengue and Severe Dengue (DS) in the Americas, by Country 2013. Washington, D.C., United States: Pan American Health Organization (PAHO), 2014	Nonfatal Health Outcomes	Dengue
Li Z, Zhao LL, Zhong YF. Epidemiolgical Investigation on 10,000 Children of 0-6 Years with Autism in Changchun city. J Community Med. 2014; 12(18): 11Â–3	Nonfatal Health Outcomes	Autism spectrum disorders
Xu Y, Yang J, Gao J, Zhou Z, Zhang T, Ren J, Li Y, Qian Y, Lai S, Chen G. Decomposing socioeconomic inequalities in depressive symptoms among the elderly in China. BMC Public Health. 2016; 16: 1214	Nonfatal Health Outcomes	Major depressive disorder
Qi L, Feng L, Ding X, Mao D, Wang Y, Xiong H. Prevalence of diabetes and impaired fasting glucose among residents in the Three Gorges Reservoir Region, China. BMC Public Health. 2014; 14: 1152	Nonfatal Health Outcomes	Diabetes mellitus
Ding Z-J, Wang G-P, Pei G-X, Zhang Y-L, Wang D-M, Hong G-Z, Du H-Y, Bai A-P, Li G. Epidemiological investigation of mental disorders among adults in Tianshui City, Gansu Province. Health Occupational Education. 2015; 33(8): 128-131	Nonfatal Health Outcomes	Alcohol use disorders
Huang G, Yu D, Mao N, Zhu Z, Zhang H, Jiang Z, Li H, Zhang Y, Shi J, Zhang S, Wang X, Xu W. Viral etiology of acute respiratory infection in Gansu Province, China, 2011. PLoS One. 2013; 8(5): e64254	Nonfatal Health Outcomes	Respiratory syncytial virus pneumonia

Bellizzi S, Ali MM, Abalos E, Betran AP, Kapila J, Pileggi-Castro C, Vogel JP, Merialdi M. Are hypertensive disorders in pregnancy associated with congenital malformations in offspring? Evidence from the WHO Multicountry cross sectional survey on maternal and newborn health. BMC Pregnancy Childbirth. 2016; 16(1): 198	Nonfatal Health Outcomes	Orofacial clefts
Qu Y, Jiang H, Zhang N, Wang D, Guo L. Prevalence of Mental Disorders in 6Â–16-Year-Old Students in Sichuan Province, China. Int J Environ Res Public Health. 2015; 12(5): 5090Â–107	Nonfatal Health Outcomes	Autism spectrum disorders
Joint United Nations Program on HIV/AIDS (UNAIDS), United Nations Children's Fund (UNICEF), World Health Organization (WHO). China Global AIDS Response Progress Reporting (GARPR) System - Antenatal Care Attendees Positive for Syphilis	Nonfatal Health Outcomes	Syphilis
China Center for Economic Research, Peking University. China Health and Retirement Longitudinal Study Pilot Resurvey 2012. Beijing, China: China Center for Economic Research, Peking University	Nonfatal Health Outcomes	None
Li D, Long Y, Wang T, Xiao D, Zhang J, Guo Z, Wang B, Yan Y. Epidemiology of Hepatitis C Virus Infection in Highly Endemic HBV Areas in China. PLoS One. 2013; 8(1): e54815	Nonfatal Health Outcomes	Acute hepatitis C
Bellizzi S, Ali MM, Abalos E, Betran AP, Kapila J, Pileggi-Castro C, Vogel JP, Merialdi M. Are hypertensive disorders in pregnancy associated with congenital malformations in offspring? Evidence from the WHO Multicountry cross sectional survey on maternal and newborn health. BMC Pregnancy Childbirth. 2016; 16(1): 198	Nonfatal Health Outcomes	Neural tube defects
Bellizzi S, Ali MM, Abalos E, Betran AP, Kapila J, Pileggi-Castro C, Vogel JP, Merialdi M. Are hypertensive disorders in pregnancy associated with congenital malformations in offspring? Evidence from the WHO Multicountry cross sectional survey on maternal and newborn health. BMC Pregnancy Childbirth. 2016; 16(1): 198	Nonfatal Health Outcomes	Urogenital congenital anomalies
Qu Y, Jiang H, Zhang N, Wang D, Guo L. Prevalence of Mental Disorders in 6Â–16-Year-Old Students in Sichuan Province, China. Int J Environ Res Public Health. 2015; 12(5): 5090Â–107	Nonfatal Health Outcomes	Schizophrenia
Qu Y, Jiang H, Zhang N, Wang D, Guo L. Prevalence of Mental Disorders in 6Â–16-Year-Old Students in Sichuan Province, China. Int J Environ Res Public Health. 2015; 12(5): 5090Â–107	Nonfatal Health Outcomes	Alcohol use disorders
Qu Y, Jiang H, Zhang N, Wang D, Guo L. Prevalence of Mental Disorders in 6Â–16-Year-Old Students in Sichuan Province, China. Int J Environ Res Public Health. 2015; 12(5): 5090Â–107	Nonfatal Health Outcomes	Major depressive disorder
Liu YF, Gao Y, Chen MF, Cao B, Yang XH, Wei L. Etiological analysis and predictive diagnostic model building of community-acquired pneumonia in adult outpatients in Beijing, China. BMC Infect Dis. 2013; 309	Nonfatal Health Outcomes	Influenza
Liu YF, Gao Y, Chen MF, Cao B, Yang XH, Wei L. Etiological analysis and predictive diagnostic model building of community-acquired pneumonia in adult outpatients in Beijing, China. BMC Infect Dis. 2013; 309	Nonfatal Health Outcomes	Respiratory syncytial virus pneumonia
Yu S, Yang H, Guo X, Zheng L, Sun Y. Association between Obese Phenotype and Mildly Reduced eGFR among the General Population from Rural Northeast China. Int J Environ Res Public Health. 2016; 13(6)	Nonfatal Health Outcomes	Chronic kidney disease
Cheng W, Wu Y, Wen Y, Ma Y, Zhao D, Dou Z, Zhang W, Bulterys M, Zhang F. Cotrimoxazole prophylaxis and antiretroviral therapy: an observational cohort study in China. Bull World Health Organ. 2015; 93(3): 152Â–60	Nonfatal Health Outcomes	HIV/AIDS
Huang G, Yu D, Mao N, Zhu Z, Zhang H, Jiang Z, Li H, Zhang Y, Shi J, Zhang S, Wang X, Xu W. Viral etiology of acute respiratory infection in Gansu Province, China, 2011. PLoS One. 2013; 8(5): e64254	Nonfatal Health Outcomes	Influenza
Li C, Zhao K, Li H, Farah OI, Wang J, Sun R, Zhang H. Free preconceptual screening examination service in rural areas of Hubei Province, China in 2012. PLoS One. 2014; 9(11): e111918	Nonfatal Health Outcomes	Chlamydial infection
Li C, Zhao K, Li H, Farah OI, Wang J, Sun R, Zhang H. Free preconceptual screening examination service in rural areas of Hubei Province, China in 2012. PLoS One. 2014; 9(11): e111918	Nonfatal Health Outcomes	Gonococcal infection
Wang R, Zhang P, Lv X, Jiang L, Gao C, Song Y, Yu Y, Li B. Situation of Diabetes and Related Disease Surveillance in Rural Areas of Jilin Province, Northeast China. Int J Environ Res Public Health. 2016; 13(6)	Nonfatal Health Outcomes	Diabetes mellitus
Zhang, T, Yang, Y, Yu, F, Zhao, Y, Lin, F, Minhas, V, Wood, C, He, N. Kaposi`s sarcoma associated herpesvirus infection among female sex workers and general population women in Shanghai, China: a cross-sectional study. BMC Infect Dis. 2014; 58	Nonfatal Health Outcomes	Sexually transmitted infections excluding HIV

Shu C-B, Chen C-A, Hu A-R, Geng D-L. Epidemiological Analysis of Severe Mental Illness in Suizhou Economic Development District. Pract Prev Med. 2013; 20(3): 305-7	Nonfatal Health Outcomes	Schizophrenia
Liu J, Wang Q, Ji X, Guo S, Dai Y, Zhang Z, Jia L, Shi Y, Tai S, Lee Y. Prevalence of Ureaplasma urealyticum, Mycoplasma hominis, Chlamydia trachomatis infections, and semen quality in infertile and fertile men in China. Urology. 2014; 83(4): 795Â–9	Nonfatal Health Outcomes	Sexually transmitted infections excluding HIV
Guan WD, Gong XY, Mok CK, Chen TT, Wu SG, Pan SH, Cowling BJ, Yang ZF, Chen de H. Surveillance for seasonal influenza virus prevalence in hospitalized children with lower respiratory tract infection in Guangzhou, China during the post-pandemic era. PLoS One. 2015; 10(4): e0120983	Nonfatal Health Outcomes	Influenza
Shen Y, Tang M. The epidemiologic survey of mild cognitive impairment and dementia among residents aged 65 years and over in the urban and the rural areas in Guangzhou [dissertation]. [Guangzhou, China]: Guangzhou Medical University; 2011. 72 p	Nonfatal Health Outcomes	Alzheimer's disease and other dementias
Luo XL, Zhang WY. Obstetrical disease spectrum in China: an epidemiological study of 111,767 cases in 2011. Chin Med J (Engl). 2015; 128(9): 1137-46	Nonfatal Health Outcomes	Maternal hemorrhage
Luo XL, Zhang WY. Obstetrical disease spectrum in China: an epidemiological study of 111,767 cases in 2011. Chin Med J (Engl). 2015; 128(9): 1137-46	Nonfatal Health Outcomes	Maternal hypertensive disorders
Zeng Z, Zhu Z, Yang Y, Ruan W, Peng X, Su Y, Peng L, Chen J, Yin Q, Zhao C, Zhou H, Yuan S, Hao Y, Qian J, Ng SC, Chen M, Hu P. Incidence and clinical characteristics of inflammatory bowel disease in a developed region of Guangdong Province, China: a prospective population-based study. J Gastroenterol Hepatol. 2013; 28(7): 1148-53	Nonfatal Health Outcomes	Inflammatory bowel disease
Center for Health Statistics and Information, National Health and Family Planning Commission (China), Shanghai Municipal Center for Disease Control and Prevention (Shanghai CDC), Shanghai Health Information Center. China Hospital Inpatient Data 2013-2016	Nonfatal Health Outcomes	Congenital birth defects
Center for Health Statistics and Information, National Health and Family Planning Commission (China), Shanghai Municipal Center for Disease Control and Prevention (Shanghai CDC), Shanghai Health Information Center. China Hospital Inpatient Data 2013-2016	Nonfatal Health Outcomes	Cirrhosis and other chronic liver diseases
Center for Health Statistics and Information, National Health and Family Planning Commission (China), Shanghai Municipal Center for Disease Control and Prevention (Shanghai CDC), Shanghai Health Information Center. China Hospital Inpatient Data 2013-2016	Nonfatal Health Outcomes	Acute glomerulonephritis
Center for Health Statistics and Information, National Health and Family Planning Commission (China), Shanghai Municipal Center for Disease Control and Prevention (Shanghai CDC), Shanghai Health Information Center. China Hospital Inpatient Data 2013-2016	Nonfatal Health Outcomes	Neonatal preterm birth
Center for Health Statistics and Information, National Health and Family Planning Commission (China), Shanghai Municipal Center for Disease Control and Prevention (Shanghai CDC), Shanghai Health Information Center. China Hospital Inpatient Data 2013-2016	Nonfatal Health Outcomes	Inguinal, femoral, and abdominal hernia
Center for Health Statistics and Information, National Health and Family Planning Commission (China), Shanghai Municipal Center for Disease Control and Prevention (Shanghai CDC), Shanghai Health Information Center. China Hospital Inpatient Data 2013-2016	Nonfatal Health Outcomes	Gynecological diseases
Center for Health Statistics and Information, National Health and Family Planning Commission (China), Shanghai Municipal Center for Disease Control and Prevention (Shanghai CDC), Shanghai Health Information Center. China Hospital Inpatient Data 2013-2016	Nonfatal Health Outcomes	Neural tube defects
Center for Health Statistics and Information, National Health and Family Planning Commission (China). China National Health Statistical Information Reporting System 2013-2015	Nonfatal Health Outcomes	Inguinal, femoral, and abdominal hernia
Center for Health Statistics and Information, National Health and Family Planning Commission (China), Shanghai Municipal Center for Disease Control and Prevention (Shanghai CDC), Shanghai Health Information Center. China Hospital Inpatient Data 2013-2016	Nonfatal Health Outcomes	Urinary tract infections
Center for Health Statistics and Information, National Health and Family Planning Commission (China), Shanghai Municipal Center for Disease Control and Prevention (Shanghai CDC), Shanghai Health Information Center. China Hospital Inpatient Data 2013-2016	Nonfatal Health Outcomes	Urolithiasis

Center for Health Statistics and Information, National Health and Family Planning Commission (China), Shanghai Municipal Center for Disease Control and Prevention (Shanghai CDC), Shanghai Health Information Center. China Hospital Inpatient Data 2013-2016	Nonfatal Health Outcomes	Benign prostatic hyperplasia
Center for Health Statistics and Information, National Health and Family Planning Commission (China), Shanghai Municipal Center for Disease Control and Prevention (Shanghai CDC), Shanghai Health Information Center. China Hospital Inpatient Data 2013-2016	Nonfatal Health Outcomes	Uterine fibroids
Center for Health Statistics and Information, National Health and Family Planning Commission (China), Shanghai Municipal Center for Disease Control and Prevention (Shanghai CDC), Shanghai Health Information Center. China Hospital Inpatient Data 2013-2016	Nonfatal Health Outcomes	Endometriosis
Center for Health Statistics and Information, National Health and Family Planning Commission (China), Shanghai Municipal Center for Disease Control and Prevention (Shanghai CDC), Shanghai Health Information Center. China Hospital Inpatient Data 2013-2016	Nonfatal Health Outcomes	Genital prolapse
Center for Health Statistics and Information, National Health and Family Planning Commission (China), Shanghai Municipal Center for Disease Control and Prevention (Shanghai CDC), Shanghai Health Information Center. China Hospital Inpatient Data 2013-2016	Nonfatal Health Outcomes	Sickle cell disorders
Center for Health Statistics and Information, National Health and Family Planning Commission (China), Shanghai Municipal Center for Disease Control and Prevention (Shanghai CDC), Shanghai Health Information Center. China Hospital Inpatient Data 2013-2016	Nonfatal Health Outcomes	Endocrine, metabolic, blood, and immune disorders
Center for Health Statistics and Information, National Health and Family Planning Commission (China). China National Health Statistical Information Reporting System 2013-2015	Nonfatal Health Outcomes	Inflammatory bowel disease
Center for Health Statistics and Information, National Health and Family Planning Commission (China), Shanghai Municipal Center for Disease Control and Prevention (Shanghai CDC), Shanghai Health Information Center. China Hospital Inpatient Data 2013-2016	Nonfatal Health Outcomes	Coal workers pneumoconiosis
Center for Health Statistics and Information, National Health and Family Planning Commission (China), Shanghai Municipal Center for Disease Control and Prevention (Shanghai CDC), Shanghai Health Information Center. China Hospital Inpatient Data 2013-2016	Nonfatal Health Outcomes	Vascular intestinal disorders
Center for Health Statistics and Information, National Health and Family Planning Commission (China), Shanghai Municipal Center for Disease Control and Prevention (Shanghai CDC), Shanghai Health Information Center. China Hospital Inpatient Data 2013-2016	Nonfatal Health Outcomes	Gallbladder and biliary diseases
Center for Health Statistics and Information, National Health and Family Planning Commission (China), Shanghai Municipal Center for Disease Control and Prevention (Shanghai CDC), Shanghai Health Information Center. China Hospital Inpatient Data 2013-2016	Nonfatal Health Outcomes	Hearing loss
Center for Health Statistics and Information, National Health and Family Planning Commission (China), Shanghai Municipal Center for Disease Control and Prevention (Shanghai CDC), Shanghai Health Information Center. China Hospital Inpatient Data 2013-2016	Nonfatal Health Outcomes	Pancreatitis
Center for Health Statistics and Information, National Health and Family Planning Commission (China), Shanghai Municipal Center for Disease Control and Prevention (Shanghai CDC), Shanghai Health Information Center. China Hospital Inpatient Data 2013-2016	Nonfatal Health Outcomes	Cirrhosis and other chronic liver diseases due to hepatitis C
Center for Health Statistics and Information, National Health and Family Planning Commission (China), Shanghai Municipal Center for Disease Control and Prevention (Shanghai CDC), Shanghai Health Information Center. China Hospital Inpatient Data 2013-2016	Nonfatal Health Outcomes	Other chromosomal abnormalities
Center for Health Statistics and Information, National Health and Family Planning Commission (China), Shanghai Municipal Center for Disease Control and Prevention (Shanghai CDC), Shanghai Health Information Center. China Hospital Inpatient Data 2013-2016	Nonfatal Health Outcomes	Congenital musculoskeletal and limb anomalies
Center for Health Statistics and Information, National Health and Family Planning Commission (China), Shanghai Municipal Center for Disease Control and Prevention (Shanghai CDC), Shanghai Health Information Center. China Hospital Inpatient Data 2013-2016	Nonfatal Health Outcomes	Interstitial lung disease and pulmonary sarcoidosis

Center for Health Statistics and Information, National Health and Family Planning Commission (China), Shanghai Municipal Center for Disease Control and Prevention (Shanghai CDC), Shanghai Health Information Center. China Hospital Inpatient Data 2013-2016	Nonfatal Health Outcomes	Congenital heart anomalies
Center for Health Statistics and Information, National Health and Family Planning Commission (China), Shanghai Municipal Center for Disease Control and Prevention (Shanghai CDC), Shanghai Health Information Center. China Hospital Inpatient Data 2013-2016	Nonfatal Health Outcomes	Subarachnoid hemorrhage
Center for Health Statistics and Information, National Health and Family Planning Commission (China), Shanghai Municipal Center for Disease Control and Prevention (Shanghai CDC), Shanghai Health Information Center. China Hospital Inpatient Data 2013-2016	Nonfatal Health Outcomes	Endocarditis
Center for Health Statistics and Information, National Health and Family Planning Commission (China), Shanghai Municipal Center for Disease Control and Prevention (Shanghai CDC), Shanghai Health Information Center. China Hospital Inpatient Data 2013-2016	Nonfatal Health Outcomes	Other gynecological diseases
Center for Health Statistics and Information, National Health and Family Planning Commission (China), Shanghai Municipal Center for Disease Control and Prevention (Shanghai CDC), Shanghai Health Information Center. China Hospital Inpatient Data 2013-2016	Nonfatal Health Outcomes	Urogenital congenital anomalies
Center for Health Statistics and Information, National Health and Family Planning Commission (China), Shanghai Municipal Center for Disease Control and Prevention (Shanghai CDC), Shanghai Health Information Center. China Hospital Inpatient Data 2013-2016	Nonfatal Health Outcomes	Neonatal encephalopathy due to birth asphyxia and trauma
Center for Health Statistics and Information, National Health and Family Planning Commission (China), Shanghai Municipal Center for Disease Control and Prevention (Shanghai CDC), Shanghai Health Information Center. China Hospital Inpatient Data 2013-2016	Nonfatal Health Outcomes	Peptic ulcer disease
Center for Health Statistics and Information, National Health and Family Planning Commission (China), Shanghai Municipal Center for Disease Control and Prevention (Shanghai CDC), Shanghai Health Information Center. China Hospital Inpatient Data 2013-2016	Nonfatal Health Outcomes	Encephalitis
Center for Health Statistics and Information, National Health and Family Planning Commission (China), Shanghai Municipal Center for Disease Control and Prevention (Shanghai CDC), Shanghai Health Information Center. China Hospital Inpatient Data 2013-2016	Nonfatal Health Outcomes	Cystic echinococcosis
Center for Health Statistics and Information, National Health and Family Planning Commission (China), Shanghai Municipal Center for Disease Control and Prevention (Shanghai CDC), Shanghai Health Information Center. China Hospital Inpatient Data 2013-2016	Nonfatal Health Outcomes	Maternal hemorrhage
Center for Health Statistics and Information, National Health and Family Planning Commission (China), Shanghai Municipal Center for Disease Control and Prevention (Shanghai CDC), Shanghai Health Information Center. China Hospital Inpatient Data 2013-2016	Nonfatal Health Outcomes	Maternal hypertensive disorders
Center for Health Statistics and Information, National Health and Family Planning Commission (China), Shanghai Municipal Center for Disease Control and Prevention (Shanghai CDC), Shanghai Health Information Center. China Hospital Inpatient Data 2013-2016	Nonfatal Health Outcomes	Maternal obstructed labor and uterine rupture
Center for Health Statistics and Information, National Health and Family Planning Commission (China), Shanghai Municipal Center for Disease Control and Prevention (Shanghai CDC), Shanghai Health Information Center. China Hospital Inpatient Data 2013-2016	Nonfatal Health Outcomes	Cirrhosis and other chronic liver diseases due to other causes
Center for Health Statistics and Information, National Health and Family Planning Commission (China), Shanghai Municipal Center for Disease Control and Prevention (Shanghai CDC), Shanghai Health Information Center. China Hospital Inpatient Data 2013-2016	Nonfatal Health Outcomes	Cirrhosis and other chronic liver diseases due to hepatitis B
Center for Health Statistics and Information, National Health and Family Planning Commission (China), Shanghai Municipal Center for Disease Control and Prevention (Shanghai CDC), Shanghai Health Information Center. China Hospital Inpatient Data 2013-2016	Nonfatal Health Outcomes	Syphilis
Center for Health Statistics and Information, National Health and Family Planning Commission (China), Shanghai Municipal Center for Disease Control and Prevention (Shanghai CDC), Shanghai Health Information Center. China Hospital Inpatient Data 2013-2016	Nonfatal Health Outcomes	Cirrhosis and other chronic liver diseases due to alcohol use

Center for Health Statistics and Information, National Health and Family Planning Commission (China), Shanghai Municipal Center for Disease Control and Prevention (Shanghai CDC), Shanghai Health Information Center. China Hospital Inpatient Data 2013-2016	Nonfatal Health Outcomes	Rheumatic heart disease
Center for Health Statistics and Information, National Health and Family Planning Commission (China), Shanghai Municipal Center for Disease Control and Prevention (Shanghai CDC), Shanghai Health Information Center. China Hospital Inpatient Data 2013-2016	Nonfatal Health Outcomes	Ischemic heart disease
Center for Health Statistics and Information, National Health and Family Planning Commission (China), Shanghai Municipal Center for Disease Control and Prevention (Shanghai CDC), Shanghai Health Information Center. China Hospital Inpatient Data 2013-2016	Nonfatal Health Outcomes	Silicosis
Center for Health Statistics and Information, National Health and Family Planning Commission (China), Shanghai Municipal Center for Disease Control and Prevention (Shanghai CDC), Shanghai Health Information Center. China Hospital Inpatient Data 2013-2016	Nonfatal Health Outcomes	Asbestosis
Center for Health Statistics and Information, National Health and Family Planning Commission (China), Shanghai Municipal Center for Disease Control and Prevention (Shanghai CDC), Shanghai Health Information Center. China Hospital Inpatient Data 2013-2016	Nonfatal Health Outcomes	Other pneumoconiosis
Center for Health Statistics and Information, National Health and Family Planning Commission (China), Shanghai Municipal Center for Disease Control and Prevention (Shanghai CDC), Shanghai Health Information Center. China Hospital Inpatient Data 2013-2016	Nonfatal Health Outcomes	Maternal sepsis and other maternal infections
Center for Health Statistics and Information, National Health and Family Planning Commission (China), Shanghai Municipal Center for Disease Control and Prevention (Shanghai CDC), Shanghai Health Information Center. China Hospital Inpatient Data 2013-2016	Nonfatal Health Outcomes	Appendicitis
Center for Health Statistics and Information, National Health and Family Planning Commission (China), Shanghai Municipal Center for Disease Control and Prevention (Shanghai CDC), Shanghai Health Information Center. China Hospital Inpatient Data 2013-2016	Nonfatal Health Outcomes	Paralytic ileus and intestinal obstruction
Center for Health Statistics and Information, National Health and Family Planning Commission (China), Shanghai Municipal Center for Disease Control and Prevention (Shanghai CDC), Shanghai Health Information Center. China Hospital Inpatient Data 2013-2016	Nonfatal Health Outcomes	Orofacial clefts
Center for Health Statistics and Information, National Health and Family Planning Commission (China), Shanghai Municipal Center for Disease Control and Prevention (Shanghai CDC), Shanghai Health Information Center. China Hospital Inpatient Data 2013-2016	Nonfatal Health Outcomes	Ischemic stroke
Center for Health Statistics and Information, National Health and Family Planning Commission (China), Shanghai Municipal Center for Disease Control and Prevention (Shanghai CDC), Shanghai Health Information Center. China Hospital Inpatient Data 2013-2016	Nonfatal Health Outcomes	Vision loss
Center for Health Statistics and Information, National Health and Family Planning Commission (China), Shanghai Municipal Center for Disease Control and Prevention (Shanghai CDC), Shanghai Health Information Center. China Hospital Inpatient Data 2013-2016	Nonfatal Health Outcomes	Intracerebral hemorrhage
Center for Health Statistics and Information, National Health and Family Planning Commission (China), Shanghai Municipal Center for Disease Control and Prevention (Shanghai CDC), Shanghai Health Information Center. China Hospital Inpatient Data 2013-2016	Nonfatal Health Outcomes	Atrial fibrillation and flutter
Center for Health Statistics and Information, National Health and Family Planning Commission (China), Shanghai Municipal Center for Disease Control and Prevention (Shanghai CDC), Shanghai Health Information Center. China Hospital Inpatient Data 2013-2016	Nonfatal Health Outcomes	Gastritis and duodenitis
Center for Health Statistics and Information, National Health and Family Planning Commission (China), Shanghai Municipal Center for Disease Control and Prevention (Shanghai CDC), Shanghai Health Information Center. China Hospital Inpatient Data 2013-2016	Nonfatal Health Outcomes	Maternal disorders
Center for Health Statistics and Information, National Health and Family Planning Commission (China), Shanghai Municipal Center for Disease Control and Prevention (Shanghai CDC), Shanghai Health Information Center. China Hospital Inpatient Data 2013-2016	Nonfatal Health Outcomes	Sexually transmitted infections excluding HIV

Center for Health Statistics and Information, National Health and Family Planning Commission (China), Shanghai Municipal Center for Disease Control and Prevention (Shanghai CDC), Shanghai Health Information Center. China Hospital Inpatient Data 2013-2016	Nonfatal Health Outcomes	Down syndrome
Center for Health Statistics and Information, National Health and Family Planning Commission (China), Shanghai Municipal Center for Disease Control and Prevention (Shanghai CDC), Shanghai Health Information Center. China Hospital Inpatient Data 2013-2016	Nonfatal Health Outcomes	Turner syndrome
Center for Health Statistics and Information, National Health and Family Planning Commission (China), Shanghai Municipal Center for Disease Control and Prevention (Shanghai CDC), Shanghai Health Information Center. China Hospital Inpatient Data 2013-2016	Nonfatal Health Outcomes	Klinefelter syndrome
Center for Health Statistics and Information, National Health and Family Planning Commission (China), Shanghai Municipal Center for Disease Control and Prevention (Shanghai CDC), Shanghai Health Information Center. China Hospital Inpatient Data 2013-2016	Nonfatal Health Outcomes	Neonatal disorders
Center for Health Statistics and Information, National Health and Family Planning Commission (China), Shanghai Municipal Center for Disease Control and Prevention (Shanghai CDC), Shanghai Health Information Center. China Hospital Inpatient Data 2013-2016	Nonfatal Health Outcomes	Neonatal sepsis and other neonatal infections
Center for Health Statistics and Information, National Health and Family Planning Commission (China), Shanghai Municipal Center for Disease Control and Prevention (Shanghai CDC), Shanghai Health Information Center. China Hospital Inpatient Data 2013-2016	Nonfatal Health Outcomes	Polycystic ovarian syndrome
Center for Health Statistics and Information, National Health and Family Planning Commission (China), Shanghai Municipal Center for Disease Control and Prevention (Shanghai CDC), Shanghai Health Information Center. China Hospital Inpatient Data 2013-2016	Nonfatal Health Outcomes	Thalassemias
National Bureau of Statistics of China. China Statistical Yearbook 2015. Beijing, China: National Bureau of Statistics of China	Nonfatal Health Outcomes	Maternal abortion, miscarriage, and ectopic pregnancy
National Bureau of Statistics of China. China Statistical Yearbook 2015. Beijing, China: National Bureau of Statistics of China	Nonfatal Health Outcomes	Cystic echinococcosis
National Bureau of Statistics of China. China Statistical Yearbook 2015. Beijing, China: National Bureau of Statistics of China	Nonfatal Health Outcomes	Endocarditis
National Bureau of Statistics of China. China Statistical Yearbook 2015. Beijing, China: National Bureau of Statistics of China	Nonfatal Health Outcomes	Gastritis and duodenitis
National Bureau of Statistics of China. China Statistical Yearbook 2015. Beijing, China: National Bureau of Statistics of China	Nonfatal Health Outcomes	Acute glomerulonephritis
National Bureau of Statistics of China. China Statistical Yearbook 2015. Beijing, China: National Bureau of Statistics of China	Nonfatal Health Outcomes	Endocrine, metabolic, blood, and immune disorders
National Bureau of Statistics of China. China Statistical Yearbook 2015. Beijing, China: National Bureau of Statistics of China	Nonfatal Health Outcomes	Maternal sepsis and other maternal infections
National Bureau of Statistics of China. China Statistical Yearbook 2015. Beijing, China: National Bureau of Statistics of China	Nonfatal Health Outcomes	Encephalitis
National Bureau of Statistics of China. China Statistical Yearbook 2015. Beijing, China: National Bureau of Statistics of China	Nonfatal Health Outcomes	Ischemic heart disease
National Bureau of Statistics of China. China Statistical Yearbook 2015. Beijing, China: National Bureau of Statistics of China	Nonfatal Health Outcomes	Interstitial lung disease and pulmonary sarcoidosis
National Bureau of Statistics of China. China Statistical Yearbook 2015. Beijing, China: National Bureau of Statistics of China	Nonfatal Health Outcomes	Vascular intestinal disorders
National Bureau of Statistics of China. China Statistical Yearbook 2015. Beijing, China: National Bureau of Statistics of China	Nonfatal Health Outcomes	Polycystic ovarian syndrome
National Bureau of Statistics of China. China Statistical Yearbook 2015. Beijing, China: National Bureau of Statistics of China	Nonfatal Health Outcomes	Diarrheal diseases
National Bureau of Statistics of China. China Statistical Yearbook 2015. Beijing, China: National Bureau of Statistics of China	Nonfatal Health Outcomes	Neonatal preterm birth
National Bureau of Statistics of China. China Statistical Yearbook 2015. Beijing, China: National Bureau of Statistics of China	Nonfatal Health Outcomes	Asbestosis
National Bureau of Statistics of China. China Statistical Yearbook 2015. Beijing, China: National Bureau of Statistics of China	Nonfatal Health Outcomes	Paralytic ileus and intestinal obstruction
National Bureau of Statistics of China. China Statistical Yearbook 2015. Beijing, China: National Bureau of Statistics of China	Nonfatal Health Outcomes	Urolithiasis
National Bureau of Statistics of China. China Statistical Yearbook 2015. Beijing, China: National Bureau of Statistics of China	Nonfatal Health Outcomes	Down syndrome
National Bureau of Statistics of China. China Statistical Yearbook 2015. Beijing, China: National Bureau of Statistics of China	Nonfatal Health Outcomes	Congenital heart anomalies

National Bureau of Statistics of China. China Statistical Yearbook 2015. Beijing, China: National Bureau of Statistics of China	Nonfatal Health Outcomes	Cutaneous and mucocutaneous leishmaniasis
National Bureau of Statistics of China. China Statistical Yearbook 2015. Beijing, China: National Bureau of Statistics of China	Nonfatal Health Outcomes	Atrial fibrillation and flutter
National Bureau of Statistics of China. China Statistical Yearbook 2015. Beijing, China: National Bureau of Statistics of China	Nonfatal Health Outcomes	Peptic ulcer disease
National Bureau of Statistics of China. China Statistical Yearbook 2015. Beijing, China: National Bureau of Statistics of China	Nonfatal Health Outcomes	Pancreatitis
National Bureau of Statistics of China. China Statistical Yearbook 2015. Beijing, China: National Bureau of Statistics of China	Nonfatal Health Outcomes	Genital prolapse
National Bureau of Statistics of China. China Statistical Yearbook 2015. Beijing, China: National Bureau of Statistics of China	Nonfatal Health Outcomes	Rheumatic heart disease
National Bureau of Statistics of China. China Statistical Yearbook 2015. Beijing, China: National Bureau of Statistics of China	Nonfatal Health Outcomes	Intracerebral hemorrhage
National Bureau of Statistics of China. China Statistical Yearbook 2015. Beijing, China: National Bureau of Statistics of China	Nonfatal Health Outcomes	Meningitis
National Bureau of Statistics of China. China Statistical Yearbook 2015. Beijing, China: National Bureau of Statistics of China	Nonfatal Health Outcomes	Syphilis
National Bureau of Statistics of China. China Statistical Yearbook 2015. Beijing, China: National Bureau of Statistics of China	Nonfatal Health Outcomes	Other pneumoconiosis
National Bureau of Statistics of China. China Statistical Yearbook 2015. Beijing, China: National Bureau of Statistics of China	Nonfatal Health Outcomes	Inflammatory bowel disease
National Bureau of Statistics of China. China Statistical Yearbook 2015. Beijing, China: National Bureau of Statistics of China	Nonfatal Health Outcomes	Uterine fibroids
National Bureau of Statistics of China. China Statistical Yearbook 2015. Beijing, China: National Bureau of Statistics of China	Nonfatal Health Outcomes	Maternal hypertensive disorders
National Bureau of Statistics of China. China Statistical Yearbook 2015. Beijing, China: National Bureau of Statistics of China	Nonfatal Health Outcomes	Silicosis
National Bureau of Statistics of China. China Statistical Yearbook 2015. Beijing, China: National Bureau of Statistics of China	Nonfatal Health Outcomes	Appendicitis
National Bureau of Statistics of China. China Statistical Yearbook 2015. Beijing, China: National Bureau of Statistics of China	Nonfatal Health Outcomes	Urinary tract infections
National Bureau of Statistics of China. China Statistical Yearbook 2015. Beijing, China: National Bureau of Statistics of China	Nonfatal Health Outcomes	Neonatal encephalopathy due to birth asphyxia and trauma
National Bureau of Statistics of China. China Statistical Yearbook 2015. Beijing, China: National Bureau of Statistics of China	Nonfatal Health Outcomes	Orofacial clefts
National Bureau of Statistics of China. China Statistical Yearbook 2015. Beijing, China: National Bureau of Statistics of China	Nonfatal Health Outcomes	Neural tube defects
National Bureau of Statistics of China. China Statistical Yearbook 2015. Beijing, China: National Bureau of Statistics of China	Nonfatal Health Outcomes	Visceral leishmaniasis
National Bureau of Statistics of China. China Statistical Yearbook 2015. Beijing, China: National Bureau of Statistics of China	Nonfatal Health Outcomes	Cirrhosis and other chronic liver diseases
National Bureau of Statistics of China. China Statistical Yearbook 2015. Beijing, China: National Bureau of Statistics of China	Nonfatal Health Outcomes	Gallbladder and biliary diseases
National Bureau of Statistics of China. China Statistical Yearbook 2015. Beijing, China: National Bureau of Statistics of China	Nonfatal Health Outcomes	Endometriosis
National Bureau of Statistics of China. China Statistical Yearbook 2015. Beijing, China: National Bureau of Statistics of China	Nonfatal Health Outcomes	Ischemic stroke
National Bureau of Statistics of China. China Statistical Yearbook 2015. Beijing, China: National Bureau of Statistics of China	Nonfatal Health Outcomes	Lower respiratory infections
National Bureau of Statistics of China. China Statistical Yearbook 2015. Beijing, China: National Bureau of Statistics of China	Nonfatal Health Outcomes	Neonatal sepsis and other neonatal infections
National Bureau of Statistics of China. China Statistical Yearbook 2015. Beijing, China: National Bureau of Statistics of China	Nonfatal Health Outcomes	Coal workers pneumoconiosis
National Bureau of Statistics of China. China Statistical Yearbook 2015. Beijing, China: National Bureau of Statistics of China	Nonfatal Health Outcomes	Inguinal, femoral, and abdominal hernia
National Bureau of Statistics of China. China Statistical Yearbook 2015. Beijing, China: National Bureau of Statistics of China	Nonfatal Health Outcomes	Benign prostatic hyperplasia
Zhu J-Y. An Investigation of Mental Disorders for 6-16 years old Children and Adolescents in Jiangmen City. J Psychiatry. 2016; 29(1): 16-8	Nonfatal Health Outcomes	Attention-deficit/hyperactivity disorder
Zhu J-Y. An Investigation of Mental Disorders for 6-16 years old Children and Adolescents in Jiangmen City. J Psychiatry. 2016; 29(1): 16-8	Nonfatal Health Outcomes	Conduct disorder
Chinese Center for Disease Control and Prevention (CCDC). China Notifiable Infectious Diseases 2014 - China CDC	Nonfatal Health Outcomes	Rabies

Center for Health Statistics and Information, National Health and Family Planning Commission (China), Shanghai Municipal Center for Disease Control and Prevention (Shanghai CDC), Shanghai Health Information Center. China Hospital Inpatient Data 2013-2016	Nonfatal Health Outcomes	Cellulitis
Center for Health Statistics and Information, National Health and Family Planning Commission (China), Shanghai Municipal Center for Disease Control and Prevention (Shanghai CDC), Shanghai Health Information Center. China Hospital Inpatient Data 2013-2016	Nonfatal Health Outcomes	Pyoderma
Center for Health Statistics and Information, National Health and Family Planning Commission (China), Shanghai Municipal Center for Disease Control and Prevention (Shanghai CDC), Shanghai Health Information Center. China Hospital Inpatient Data 2013-2016	Nonfatal Health Outcomes	Decubitus ulcer
Chinese Center for Disease Control and Prevention (CCDC), Ministry of Health (China). China National Injury Surveillance System 2014	Nonfatal Health Outcomes	Road injuries
Chinese Center for Disease Control and Prevention (CCDC). China Disease Surveillance Points 2014 - China CDC	Nonfatal Health Outcomes	Rabies
Chinese Center for Disease Control and Prevention (CCDC), Ministry of Health (China). China National Injury Surveillance System 2014	Nonfatal Health Outcomes	Falls
Chinese Center for Disease Control and Prevention (CCDC), Ministry of Health (China). China National Injury Surveillance System 2014	Nonfatal Health Outcomes	Drowning
Center for Health Statistics and Information, National Health and Family Planning Commission (China), Shanghai Municipal Center for Disease Control and Prevention (Shanghai CDC), Shanghai Health Information Center. China Hospital Inpatient Data 2013-2016	Nonfatal Health Outcomes	Non-rheumatic calcific aortic valve disease
National Bureau of Statistics of China. China Statistical Yearbook 2015. Beijing, China: National Bureau of Statistics of China	Nonfatal Health Outcomes	Turner syndrome
Chinese Center for Disease Control and Prevention (CCDC), Ministry of Health (China). China National Injury Surveillance System 2014	Nonfatal Health Outcomes	Poisonings
Chinese Center for Disease Control and Prevention (CCDC), Ministry of Health (China). China National Injury Surveillance System 2014	Nonfatal Health Outcomes	Exposure to mechanical forces
Center for Health Statistics and Information, National Health and Family Planning Commission (China), Shanghai Municipal Center for Disease Control and Prevention (Shanghai CDC), Shanghai Health Information Center. China Hospital Inpatient Data 2013-2016	Nonfatal Health Outcomes	Digestive congenital anomalies
Center for Health Statistics and Information, National Health and Family Planning Commission (China), Shanghai Municipal Center for Disease Control and Prevention (Shanghai CDC), Shanghai Health Information Center. China Hospital Inpatient Data 2013-2016	Nonfatal Health Outcomes	Non-rheumatic degenerative mitral valve disease
Chinese Center for Disease Control and Prevention (CCDC), Ministry of Health (China). China National Injury Surveillance System 2014	Nonfatal Health Outcomes	Adverse effects of medical treatment
Center for Health Statistics and Information, National Health and Family Planning Commission (China), Shanghai Municipal Center for Disease Control and Prevention (Shanghai CDC), Shanghai Health Information Center. China Hospital Inpatient Data 2013-2016	Nonfatal Health Outcomes	Adverse effects of medical treatment
Chinese Center for Disease Control and Prevention (CCDC), Ministry of Health (China). China National Injury Surveillance System 2014	Nonfatal Health Outcomes	Animal contact
Chinese Center for Disease Control and Prevention (CCDC), Ministry of Health (China). China National Injury Surveillance System 2014	Nonfatal Health Outcomes	Pulmonary aspiration and foreign body in airway
Chinese Center for Disease Control and Prevention (CCDC), Ministry of Health (China). China National Injury Surveillance System 2014	Nonfatal Health Outcomes	Other unintentional injuries
Chinese Center for Disease Control and Prevention (CCDC), Ministry of Health (China). China National Injury Surveillance System 2014	Nonfatal Health Outcomes	Self-harm
Chinese Center for Disease Control and Prevention (CCDC), Ministry of Health (China). China National Injury Surveillance System 2014	Nonfatal Health Outcomes	Interpersonal violence
Center for Health Statistics and Information, National Health and Family Planning Commission (China), Shanghai Municipal Center for Disease Control and Prevention (Shanghai CDC), Shanghai Health Information Center. China Hospital Inpatient Data 2013-2016	Nonfatal Health Outcomes	Myelodysplastic, myeloproliferative, and other hematopoietic neoplasms
Center for Health Statistics and Information, National Health and Family Planning Commission (China), Shanghai Municipal Center for Disease Control and Prevention (Shanghai CDC), Shanghai Health Information Center. China Hospital Inpatient Data 2013-2016	Nonfatal Health Outcomes	Benign and in situ intestinal neoplasms
Center for Health Statistics and Information, National Health and Family Planning Commission (China), Shanghai Municipal Center for Disease Control and Prevention (Shanghai CDC), Shanghai Health Information Center. China Hospital Inpatient Data 2013-2016	Nonfatal Health Outcomes	Benign and in situ cervical and uterine neoplasms

Center for Health Statistics and Information, National Health and Family Planning Commission (China), Shanghai Municipal Center for Disease Control and Prevention (Shanghai CDC), Shanghai Health Information Center. China Hospital Inpatient Data 2013-2016	Nonfatal Health Outcomes	Other benign and in situ neoplasms
Center for Health Statistics and Information, National Health and Family Planning Commission (China), Shanghai Municipal Center for Disease Control and Prevention (Shanghai CDC), Shanghai Health Information Center. China Hospital Inpatient Data 2013-2016	Nonfatal Health Outcomes	Maternal abortion and miscarriage
Chinese Center for Disease Control and Prevention (CCDC), Ministry of Health (China). China National Injury Surveillance System 2014	Nonfatal Health Outcomes	Unintentional firearm injuries
Chinese Center for Disease Control and Prevention (CCDC), Ministry of Health (China). China National Injury Surveillance System 2014	Nonfatal Health Outcomes	Other exposure to mechanical forces
Chinese Center for Disease Control and Prevention (CCDC), Ministry of Health (China). China National Injury Surveillance System 2014	Nonfatal Health Outcomes	Venomous animal contact
Chinese Center for Disease Control and Prevention (CCDC), Ministry of Health (China). China National Injury Surveillance System 2014	Nonfatal Health Outcomes	Non-venomous animal contact
Chinese Center for Disease Control and Prevention (CCDC), Ministry of Health (China). China National Injury Surveillance System 2014	Nonfatal Health Outcomes	Foreign body in eyes
Chinese Center for Disease Control and Prevention (CCDC), Ministry of Health (China). China National Injury Surveillance System 2014	Nonfatal Health Outcomes	Foreign body in other body part
Chinese Center for Disease Control and Prevention (CCDC), Ministry of Health (China). China National Injury Surveillance System 2014	Nonfatal Health Outcomes	Physical violence by firearm
Chinese Center for Disease Control and Prevention (CCDC), Ministry of Health (China). China National Injury Surveillance System 2014	Nonfatal Health Outcomes	Physical violence by sharp object
Chinese Center for Disease Control and Prevention (CCDC), Ministry of Health (China). China National Injury Surveillance System 2014	Nonfatal Health Outcomes	Physical violence by other means
Center for Health Statistics and Information, National Health and Family Planning Commission (China), Shanghai Municipal Center for Disease Control and Prevention (Shanghai CDC), Shanghai Health Information Center. China Hospital Inpatient Data 2013-2016	Nonfatal Health Outcomes	Myocarditis
Zhang Y, Li H-H, Li C-J, Yang Z, Xiong L, Chen L, Huang Y, Nong Z, Zeng D-Y. Analysis of Risk Factors and Prevalence of ADHD among Elementary School Students in Liuzhou City. Chin J Sch Health. 2017; 38(2): 242-8	Nonfatal Health Outcomes	Attention-deficit/hyperactivity disorder
Yang L, Jin X, Yan J, Jin Y, Yu W, Wu H, Xu S. Prevalence of dementia, cognitive status and associated risk factors among elderly of Zhejiang province, China in 2014. Age Ageing. 2016; 45(5): 708-12	Nonfatal Health Outcomes	Alzheimer's disease and other dementias
Jiang YY. Prevalence of Early Childhood Caries Among 2- to 5-year-old Preschoolers in Kindergartens of Weifang City, China: A Cross-sectional Study. Oral Health Prev Dent. 2017; 15(1): 89-97	Nonfatal Health Outcomes	Caries of deciduous teeth
Shu LH, Xu JJ, Wang S, Zhong HQ, Dong XY, Jiang K, Zhang HY, Xiong Q, Wang C, Sun T, Sun C, Lu Q. [Distribution of pathogenic microorganisms and its relationship with clinical features in children with community-acquired pneumonia]. Chin J Contemp Pediatr. 2015; 17(10): 1056-61	Nonfatal Health Outcomes	Influenza
Shu LH, Xu JJ, Wang S, Zhong HQ, Dong XY, Jiang K, Zhang HY, Xiong Q, Wang C, Sun T, Sun C, Lu Q. [Distribution of pathogenic microorganisms and its relationship with clinical features in children with community-acquired pneumonia]. Chin J Contemp Pediatr. 2015; 17(10): 1056-61	Nonfatal Health Outcomes	Respiratory syncytial virus pneumonia
Shu LH, Xu JJ, Wang S, Zhong HQ, Dong XY, Jiang K, Zhang HY, Xiong Q, Wang C, Sun T, Sun C, Lu Q. [Distribution of pathogenic microorganisms and its relationship with clinical features in children with community-acquired pneumonia]. Chin J Contemp Pediatr. 2015; 17(10): 1056-61	Nonfatal Health Outcomes	Lower respiratory infections
Han H-Y, Zhang H-Q, Zhang H-M, Gao X-N, Zhang W. Study on the correlation between the incidence of anxiety disorder and family factors in Shijiazhuang. Chin J Practical Nerv Dis. 2016; 19(3): 8-10	Nonfatal Health Outcomes	Anxiety disorders
Jiang L, Li G, Hao L, Guo R, Yang C, Du Y. [Epidemiological investigation on autism spectrum disorders among preschool children in Shanghai]. Chin J Epidemiol. 2015; 36(12): 1365Â–8	Nonfatal Health Outcomes	Autism spectrum disorders
Zhang Y, Shi Z, Hock D, Yue W, Liu S, Zhang Y, Liu S, Zhao L, Lu H, Guan Y, Wang X, Wsiniewski T, Ji Y. Prevalence of primary headache disorders in a population aged 60 years and older in a rural area of Northern China. J Headache Pain. 2016; 17(1): 83	Nonfatal Health Outcomes	Migraine
Zhang Y, Shi Z, Hock D, Yue W, Liu S, Zhang Y, Liu S, Zhao L, Lu H, Guan Y, Wang X, Wsiniewski T, Ji Y. Prevalence of primary headache disorders in a population aged 60 years and older in a rural area of Northern China. J Headache Pain. 2016; 17(1): 83	Nonfatal Health Outcomes	Tension-type headache

Wang S-Y, Ye X-H, Chen H, Ou G-S, Meng N-N, Mu J, Zhang Q. Prevalence of Attention Deficit Hypperactivity Disorder and its Related Factors among 6-13 years old School Children in Lanzhou City. Pract Clin Med. 2015; 16(5): 90-5	Nonfatal Health Outcomes	Attention-deficit/hyperactivity disorder
Dai H, Wang W, Chen R, Chen Z, Lu Y, Yuan H. Lipid Accumulation Product is a Powerful Tool to Predict Non-alcoholic Fatty Liver Disease in Chinese Adults. Nutr Metab (Lond). 2017; 14(49): 1-9	Nonfatal Health Outcomes	Cirrhosis due to NASH
Hu Y, Li R, Ward MP, Chen Y, Lynn H, Wang D, Chen G, He Z, Sun L, Xiong C, Zhang Z, Jiang Q. Human infections and co-infections with helminths in a rural population in Guichi, Anhui Province, China. Geospat Health. 2015; 10(2): 374	Nonfatal Health Outcomes	Schistosomiasis
Cheng W, Lue LQ, Qian YY, Hu YL, Hu ZJ, Ling CY, Gao YJ, Hu ZY. Epidemiological investigation on autism spectrum disorders in children aged from 1 to 6 years old in Ningbo city. Prev Med. 2016; 28(11): 1168-1171	Nonfatal Health Outcomes	Autism spectrum disorders
Peng Y, Shu C, Fu Z, Li QB, Liu Z, Yan L. [Pathogen detection of 1 613 cases of hospitalized children with community acquired pneumonia]. Chin J Contemp Pediatr. 2015; 17.0(11): 1193-9	Nonfatal Health Outcomes	Influenza
Peng Y, Shu C, Fu Z, Li QB, Liu Z, Yan L. [Pathogen detection of 1 613 cases of hospitalized children with community acquired pneumonia]. Chin J Contemp Pediatr. 2015; 17.0(11): 1193-9	Nonfatal Health Outcomes	Respiratory syncytial virus pneumonia
Peng Y, Shu C, Fu Z, Li QB, Liu Z, Yan L. [Pathogen detection of 1 613 cases of hospitalized children with community acquired pneumonia]. Chin J Contemp Pediatr. 2015; 17.0(11): 1193-9	Nonfatal Health Outcomes	Lower respiratory infections
National Bureau of Statistics of China. China Statistical Yearbook 2015. Beijing, China: National Bureau of Statistics of China	Nonfatal Health Outcomes	Cyclist road injuries
National Bureau of Statistics of China. China Statistical Yearbook 2015. Beijing, China: National Bureau of Statistics of China	Nonfatal Health Outcomes	Non-venomous animal contact
National Bureau of Statistics of China. China Statistical Yearbook 2015. Beijing, China: National Bureau of Statistics of China	Nonfatal Health Outcomes	Drowning
National Bureau of Statistics of China. China Statistical Yearbook 2015. Beijing, China: National Bureau of Statistics of China	Nonfatal Health Outcomes	Self-harm
National Bureau of Statistics of China. China Statistical Yearbook 2015. Beijing, China: National Bureau of Statistics of China	Nonfatal Health Outcomes	Unintentional firearm injuries
National Bureau of Statistics of China. China Statistical Yearbook 2015. Beijing, China: National Bureau of Statistics of China	Nonfatal Health Outcomes	Physical violence by sharp object
National Bureau of Statistics of China. China Statistical Yearbook 2015. Beijing, China: National Bureau of Statistics of China	Nonfatal Health Outcomes	Fire, heat, and hot substances
National Bureau of Statistics of China. China Statistical Yearbook 2015. Beijing, China: National Bureau of Statistics of China	Nonfatal Health Outcomes	Interpersonal violence
National Bureau of Statistics of China. China Statistical Yearbook 2015. Beijing, China: National Bureau of Statistics of China	Nonfatal Health Outcomes	Unintentional suffocation
National Bureau of Statistics of China. China Statistical Yearbook 2015. Beijing, China: National Bureau of Statistics of China	Nonfatal Health Outcomes	Congenital musculoskeletal and limb anomalies
National Bureau of Statistics of China. China Statistical Yearbook 2015. Beijing, China: National Bureau of Statistics of China	Nonfatal Health Outcomes	Road injuries
National Bureau of Statistics of China. China Statistical Yearbook 2015. Beijing, China: National Bureau of Statistics of China	Nonfatal Health Outcomes	Animal contact
National Bureau of Statistics of China. China Statistical Yearbook 2015. Beijing, China: National Bureau of Statistics of China	Nonfatal Health Outcomes	Motorcyclist road injuries
National Bureau of Statistics of China. China Statistical Yearbook 2015. Beijing, China: National Bureau of Statistics of China	Nonfatal Health Outcomes	Other transport injuries
National Bureau of Statistics of China. China Statistical Yearbook 2015. Beijing, China: National Bureau of Statistics of China	Nonfatal Health Outcomes	Pulmonary aspiration and foreign body in airway
National Bureau of Statistics of China. China Statistical Yearbook 2015. Beijing, China: National Bureau of Statistics of China	Nonfatal Health Outcomes	Motor vehicle road injuries
National Bureau of Statistics of China. China Statistical Yearbook 2015. Beijing, China: National Bureau of Statistics of China	Nonfatal Health Outcomes	Foreign body in other body part
National Bureau of Statistics of China. China Statistical Yearbook 2015. Beijing, China: National Bureau of Statistics of China	Nonfatal Health Outcomes	Environmental heat and cold exposure
National Bureau of Statistics of China. China Statistical Yearbook 2015. Beijing, China: National Bureau of Statistics of China	Nonfatal Health Outcomes	Self-harm by other specified means
National Bureau of Statistics of China. China Statistical Yearbook 2015. Beijing, China: National Bureau of Statistics of China	Nonfatal Health Outcomes	Cellulitis
National Bureau of Statistics of China. China Statistical Yearbook 2015. Beijing, China: National Bureau of Statistics of China	Nonfatal Health Outcomes	Poisonings
National Bureau of Statistics of China. China Statistical Yearbook 2015. Beijing, China: National Bureau of Statistics of China	Nonfatal Health Outcomes	Other exposure to mechanical forces

National Bureau of Statistics of China. China Statistical Yearbook 2015. Beijing, China: National Bureau of Statistics of China	Nonfatal Health Outcomes	Pyoderma
National Bureau of Statistics of China. China Statistical Yearbook 2015. Beijing, China: National Bureau of Statistics of China	Nonfatal Health Outcomes	Exposure to mechanical forces
National Bureau of Statistics of China. China Statistical Yearbook 2015. Beijing, China: National Bureau of Statistics of China	Nonfatal Health Outcomes	Pedestrian road injuries
National Bureau of Statistics of China. China Statistical Yearbook 2015. Beijing, China: National Bureau of Statistics of China	Nonfatal Health Outcomes	Venomous animal contact
Chinese Center for Disease Control and Prevention (CCDC). China Disease Surveillance Points 2016 - China CDC	Nonfatal Health Outcomes	Rabies
National Bureau of Statistics of China. China Statistical Yearbook 2015. Beijing, China: National Bureau of Statistics of China	Nonfatal Health Outcomes	Falls
National Bureau of Statistics of China. China Statistical Yearbook 2015. Beijing, China: National Bureau of Statistics of China	Nonfatal Health Outcomes	Other unintentional injuries
National Bureau of Statistics of China. China Statistical Yearbook 2015. Beijing, China: National Bureau of Statistics of China	Nonfatal Health Outcomes	Other road injuries
National Bureau of Statistics of China. China Statistical Yearbook 2015. Beijing, China: National Bureau of Statistics of China	Nonfatal Health Outcomes	Physical violence by firearm
National Bureau of Statistics of China. China Statistical Yearbook 2015. Beijing, China: National Bureau of Statistics of China	Nonfatal Health Outcomes	Decubitus ulcer
National Bureau of Statistics of China. China Statistical Yearbook 2015. Beijing, China: National Bureau of Statistics of China	Nonfatal Health Outcomes	Adverse effects of medical treatment
National Bureau of Statistics of China. China Statistical Yearbook 2015. Beijing, China: National Bureau of Statistics of China	Nonfatal Health Outcomes	Foreign body in eyes
National Bureau of Statistics of China. China Statistical Yearbook 2015. Beijing, China: National Bureau of Statistics of China	Nonfatal Health Outcomes	Klinefelter syndrome
National Bureau of Statistics of China. China Statistical Yearbook 2015. Beijing, China: National Bureau of Statistics of China	Nonfatal Health Outcomes	Self-harm by firearm
National Bureau of Statistics of China. China Statistical Yearbook 2015. Beijing, China: National Bureau of Statistics of China	Nonfatal Health Outcomes	Myocarditis
National Bureau of Statistics of China. China Statistical Yearbook 2015. Beijing, China: National Bureau of Statistics of China	Nonfatal Health Outcomes	Physical violence by other means
National Bureau of Statistics of China. China Statistical Yearbook 2015. Beijing, China: National Bureau of Statistics of China	Nonfatal Health Outcomes	None
Chinese Center for Disease Control and Prevention (CCDC). China Disease Surveillance Points 2015 - China CDC	Nonfatal Health Outcomes	Rabies
Centre for Health Protection (Hong Kong, China). Hong Kong Communicable Diseases Watch 2018. Kowloon, China: Centre for Health Protection (Hong Kong, China), 2018	Nonfatal Health Outcomes	Dengue
Zhou H, Zhao M, Li X, Zhang D, Zhou S, Chen C, Feng Z, Ma X. Clinical evaluation of the isothermal amplification assays for the detection of four common respiratory viruses in children with pneumonia. Arch Virol. 2017; 162(5): 1311-1318	Nonfatal Health Outcomes	Respiratory syncytial virus pneumonia
International Centre for Eye Health (ICEH). China - Xinjiang Rapid Assessment of Avoidable Blindness 2015. Grootebroek, Netherlands: RAAB Repository	Nonfatal Health Outcomes	Glaucoma
International Centre for Eye Health (ICEH). China - Xinjiang Rapid Assessment of Avoidable Blindness 2015. Grootebroek, Netherlands: RAAB Repository	Nonfatal Health Outcomes	Cataract
International Centre for Eye Health (ICEH). China - Xinjiang Rapid Assessment of Avoidable Blindness 2015. Grootebroek, Netherlands: RAAB Repository	Nonfatal Health Outcomes	Age-related macular degeneration
International Centre for Eye Health (ICEH). China - Xinjiang Rapid Assessment of Avoidable Blindness 2015. Grootebroek, Netherlands: RAAB Repository	Nonfatal Health Outcomes	Refraction and accommodation disorders
International Centre for Eye Health (ICEH). China - Xinjiang Rapid Assessment of Avoidable Blindness 2015. Grootebroek, Netherlands: RAAB Repository	Nonfatal Health Outcomes	Refraction disorders
Chen KJ, Gao SS, Duangthip D, Li SKY, Lo ECM, Chu CH. Dental caries status and its associated factors among 5-year-old Hong Kong children: a cross-sectional study. BMC Oral Health. 2017; 17(1): 121	Nonfatal Health Outcomes	Caries of deciduous teeth
Zhang S, Lo ECM, Chu CH. Occlusal Features and Caries Experience of Hong Kong Chinese Preschool Children: A Cross-Sectional Study. Int J Environ Res Public Health. 2017; 14(6)	Nonfatal Health Outcomes	Caries of deciduous teeth
Yan B, Chan L, Lee V, Freedman B. Medical outpatient clinics an ideal setting for atrial fibrillation screening using a handheld single-lead ECG with automated diagnosis. Eur Heart J. 2016; 37: 888	Nonfatal Health Outcomes	Atrial fibrillation and flutter
International Centre for Eye Health (ICEH). China - Xinjiang Rapid Assessment of Avoidable Blindness 2015. Grootebroek, Netherlands: RAAB Repository	Nonfatal Health Outcomes	
International Centre for Eye Health (ICEH). China - Xinjiang Rapid Assessment of Avoidable Blindness 2015. Grootebroek, Netherlands: RAAB Repository	Nonfatal Health Outcomes	Diabetes mellitus

International Centre for Eye Health (ICEH). China - Xinjiang Rapid Assessment of Avoidable Blindness 2015. Grootebroek, Netherlands: RAAB Repository	Nonfatal Health Outcomes	Vision loss
Centre for Health Protection (Hong Kong, China). Hong Kong Update on Number of Dengue Fever Cases 2017. 2017	Nonfatal Health Outcomes	Dengue
Zhou H, Zhao M, Li X, Zhang D, Zhou S, Chen C, Feng Z, Ma X. Clinical evaluation of the isothermal amplification assays for the detection of four common respiratory viruses in children with pneumonia. Arch Virol. 2017; 162(5): 1311-1318	Nonfatal Health Outcomes	Lower respiratory infections
Yang X-C, He C-G, Su J, Zhang Q-H, Qi W-G, Wang Q-F, Zhang J-G, Zhu C-Q, Shi W-F, Zhong M. Epidemiological survey of mental disorders among people aged 15 and over in Laiwu city of Shandong province. J Clin Psychiatry. 2017; 27(1): 56-58	Nonfatal Health Outcomes	Schizophrenia
Yang X-C, He C-G, Su J, Zhang Q-H, Qi W-G, Wang Q-F, Zhang J-G, Zhu C-Q, Shi W-F, Zhong M. Epidemiological survey of mental disorders among people aged 15 and over in Laiwu city of Shandong province. J Clin Psychiatry. 2017; 27(1): 56-58	Nonfatal Health Outcomes	Alcohol use disorders
Yang X-C, He C-G, Su J, Zhang Q-H, Qi W-G, Wang Q-F, Zhang J-G, Zhu C-Q, Shi W-F, Zhong M. Epidemiological survey of mental disorders among people aged 15 and over in Laiwu city of Shandong province. J Clin Psychiatry. 2017; 27(1): 56-58	Nonfatal Health Outcomes	Anxiety disorders
Li X-D, Qiu B-H, Su F-C, Sun S-X. Gender impacts on the correlations between nonalcoholic fatty liver disease and hypertension in a Chinese population aged 45-60 y. Clin Exp Hypertens. 2016; 38(7): 639Â–43	Nonfatal Health Outcomes	Cirrhosis due to NASH
Yin W, Yang YM, Chen H, Li X, Wang Z, Cheng L, Yin QD, Fang HZ, Fei W, Mi FL, Nie MH, Hu T, Zhou XD. Oral health status in Sichuan Province: findings from the oral health survey of Sichuan, 2015-2016. Int J Oral Sci. 2017; 9(1): 10-15	Nonfatal Health Outcomes	Caries of deciduous teeth
Yin W, Yang YM, Chen H, Li X, Wang Z, Cheng L, Yin QD, Fang HZ, Fei W, Mi FL, Nie MH, Hu T, Zhou XD. Oral health status in Sichuan Province: findings from the oral health survey of Sichuan, 2015-2016. Int J Oral Sci. 2017; 9(1): 10-15	Nonfatal Health Outcomes	Caries of permanent teeth
Peng R, Li S, Zhang H, Zeng H, Jiang B, Liu Y, Yi X, Xu M, Zhu L, Zhang Z. Weight Status Is Associated with Blood Pressure, Vital Capacity, Dental Decay, and Visual Acuity among School-Age Children in Chengdu, China. Ann Nutr Metab. 2016; 69(3-4): 237-245	Nonfatal Health Outcomes	Caries of permanent teeth
Yin W, Yang YM, Chen H, Li X, Wang Z, Cheng L, Yin QD, Fang HZ, Fei W, Mi FL, Nie MH, Hu T, Zhou XD. Oral health status in Sichuan Province: findings from the oral health survey of Sichuan, 2015-2016. Int J Oral Sci. 2017; 9(1): 10-15	Nonfatal Health Outcomes	Periodontal diseases
Yin W, Yang YM, Chen H, Li X, Wang Z, Cheng L, Yin QD, Fang HZ, Fei W, Mi FL, Nie MH, Hu T, Zhou XD. Oral health status in Sichuan Province: findings from the oral health survey of Sichuan, 2015-2016. Int J Oral Sci. 2017; 9(1): 10-15	Nonfatal Health Outcomes	Edentulism and severe tooth loss
World Health Organization Regional Office for the Western Pacific (WPRO-WHO). WPRO Dengue Situation Update Number 539, 16 January 2018. Manila, Philippines: World Health Organization Regional Office for the Western Pacific (WPRO-WHO), 2018	Nonfatal Health Outcomes	Dengue
World Health Organization Regional Office for the Western Pacific (WPRO-WHO). WPRO Dengue Situation Update Number 526, 26 Sep 2017. Manila, Philippines: World Health Organization Regional Office for the Western Pacific (WPRO-WHO), 2017	Nonfatal Health Outcomes	Dengue
World Health Organization Regional Office for the Western Pacific (WPRO-WHO). WPRO Dengue Situation Update Number 528, 24 Oct 2017. Manila, Philippines: World Health Organization Regional Office for the Western Pacific (WPRO-WHO), 2017	Nonfatal Health Outcomes	Dengue
World Health Organization Regional Office for the Western Pacific (WPRO-WHO). WPRO Dengue Situation Update Number 530, 21 Nov 2017. Manila, Philippines: World Health Organization Regional Office for the Western Pacific (WPRO-WHO), 2017	Nonfatal Health Outcomes	Dengue
World Health Organization Regional Office for the Western Pacific (WPRO-WHO). WPRO Dengue Situation Update Number 532, 19 Dec 2017. Manila, Philippines: World Health Organization Regional Office for the Western Pacific (WPRO-WHO), 2017	Nonfatal Health Outcomes	Dengue
Hong Kong Vital Registration - Deaths 1991 ICD9 as it appears in World Health Organization (WHO). WHO Mortality Database Version September 2016. Geneva, Switzerland: World Health Organization (WHO), 2016	Nonfatal Health Outcomes	Rabies
Hong Kong Vital Registration - Deaths 1992 ICD9 as it appears in World Health Organization (WHO). WHO Mortality Database Version September 2016. Geneva, Switzerland: World Health Organization (WHO), 2016	Nonfatal Health Outcomes	Rabies
Hong Kong Vital Registration - Deaths 1993 ICD9 as it appears in World Health Organization (WHO). WHO Mortality Database Version September 2016. Geneva, Switzerland: World Health Organization (WHO), 2016	Nonfatal Health Outcomes	Rabies

Hong Kong Vital Registration - Deaths 1994 ICD9 as it appears in World Health Organization (WHO). WHO Mortality Database Version November 2015. Geneva, Switzerland: World Health Organization (WHO), 2015	Nonfatal Health Outcomes	Rabies
Hong Kong Vital Registration - Deaths 1995 ICD9 as it appears in World Health Organization (WHO). WHO Mortality Database Version November 2015. Geneva, Switzerland: World Health Organization (WHO), 2015	Nonfatal Health Outcomes	Rabies
Hong Kong Vital Registration - Deaths 1996 ICD9 as it appears in World Health Organization (WHO). WHO Mortality Database Version November 2015. Geneva, Switzerland: World Health Organization (WHO), 2015	Nonfatal Health Outcomes	Rabies
Hong Kong Vital Registration - Deaths 1997 ICD9 as it appears in World Health Organization (WHO). WHO Mortality Database Version November 2015. Geneva, Switzerland: World Health Organization (WHO), 2015	Nonfatal Health Outcomes	Rabies
Hong Kong Vital Registration - Deaths 1998 ICD9 as it appears in World Health Organization (WHO). WHO Mortality Database Version November 2015. Geneva, Switzerland: World Health Organization (WHO), 2015	Nonfatal Health Outcomes	Rabies
Hong Kong Vital Registration - Deaths 1999 ICD9 as it appears in World Health Organization (WHO). WHO Mortality Database Version November 2015. Geneva, Switzerland: World Health Organization (WHO), 2015	Nonfatal Health Outcomes	Rabies
Hong Kong Vital Registration - Deaths 2000 ICD9 as it appears in World Health Organization (WHO). WHO Mortality Database Version November 2015. Geneva, Switzerland: World Health Organization (WHO), 2015	Nonfatal Health Outcomes	Rabies
Macau Vital Registration - Deaths 1994 ICD9 as it appears in World Health Organization (WHO). WHO Mortality Database Version November 2015. Geneva, Switzerland: World Health Organization (WHO), 2015	Nonfatal Health Outcomes	Rabies
Hong Kong Vital Registration - Deaths 2001 ICD10 as it appears in World Health Organization (WHO). WHO Mortality Database Version October 2017. Geneva, Switzerland: World Health Organization (WHO), 2017	Nonfatal Health Outcomes	Rabies
Hong Kong Vital Registration - Deaths 2002 ICD10 as it appears in World Health Organization (WHO). WHO Mortality Database Version October 2017. Geneva, Switzerland: World Health Organization (WHO), 2017	Nonfatal Health Outcomes	Rabies
Hong Kong Vital Registration - Deaths 2003 ICD10 as it appears in World Health Organization (WHO). WHO Mortality Database Version October 2017. Geneva, Switzerland: World Health Organization (WHO), 2017	Nonfatal Health Outcomes	Rabies
Hong Kong Vital Registration - Deaths 2004 ICD10 as it appears in World Health Organization (WHO). WHO Mortality Database Version October 2017. Geneva, Switzerland: World Health Organization (WHO), 2017	Nonfatal Health Outcomes	Rabies
Hong Kong Vital Registration - Deaths 2005 ICD10 as it appears in World Health Organization (WHO). WHO Mortality Database Version October 2017. Geneva, Switzerland: World Health Organization (WHO), 2017	Nonfatal Health Outcomes	Rabies
Hong Kong Vital Registration - Deaths 2006 ICD10 as it appears in World Health Organization (WHO). WHO Mortality Database Version October 2017. Geneva, Switzerland: World Health Organization (WHO), 2017	Nonfatal Health Outcomes	Rabies
Hong Kong Vital Registration - Deaths 2007 ICD10 as it appears in World Health Organization (WHO). WHO Mortality Database Version October 2017. Geneva, Switzerland: World Health Organization (WHO), 2017	Nonfatal Health Outcomes	Rabies
China - Beijing Eye Study 2001 as it appears in Xu J, Xu L, Du KF, Shao L, Chen CX, Zhou JQ, Wang YX, You QS, Jonas JB, Wei WB. Subfoveal choroidal thickness in diabetes and diabetic retinopathy [Unpublished data]. Ophthalmology. 2013 Oct;120(10):2023-8	Nonfatal Health Outcomes	
Hong Kong Vital Registration - Deaths 2010 ICD10 as it appears in World Health Organization (WHO). WHO Mortality Database Version October 2017. Geneva, Switzerland: World Health Organization (WHO), 2017	Nonfatal Health Outcomes	Rabies
China National Nutrition Survey 1992 - China CDC as it appears in World Health Organization (WHO). WHO Global Database on Anemia, Nutrition Landscape Information System. Geneva, Switzerland: World Health Organization (WHO)	Nonfatal Health Outcomes	Anemia
Zhou LM, Yang WW, Hua JZ, Deng CQ, Tao X, Stoltzfus RJ. Relation of hemoglobin measured at different times in pregnancy to preterm birth and low birth weight in Shanghai, China. Am J Epidemiol. 1998; 148(10): 998-1006 as it appears in World Health Organization (WHO). WHO Global Database on Anemia, Nutrition Landscape Information System. Geneva, Switzerland: World Health Organization (WHO)	Nonfatal Health Outcomes	Anemia
Cai MQ, Yan WY. Study on iron nutritional status in adolescence. Biomed Environ Sci. 1990; 3(1): 113-9 as it appears in World Health Organization (WHO). WHO Global Database on Anemia, Nutrition Landscape Information System. Geneva, Switzerland: World Health Organization (WHO)	Nonfatal Health Outcomes	Anemia

Hong Kong Vital Registration - Deaths 2014 ICD10 as it appears in World Health Organization (WHO). WHO Mortality Database Version April 2018. Geneva, Switzerland: World Health Organization (WHO), 2018	Nonfatal Health Outcomes	Rabies
Hong Kong Vital Registration - Deaths 2015 ICD10 as it appears in World Health Organization (WHO). WHO Mortality Database Version April 2018. Geneva, Switzerland: World Health Organization (WHO), 2018	Nonfatal Health Outcomes	Rabies
Hong Kong Vital Registration - Deaths 2012 ICD10 as it appears in World Health Organization (WHO). WHO Mortality Database Version October 2017. Geneva, Switzerland: World Health Organization (WHO), 2017	Nonfatal Health Outcomes	Rabies
Hong Kong Vital Registration - Deaths 2013 ICD10 as it appears in World Health Organization (WHO). WHO Mortality Database Version October 2017. Geneva, Switzerland: World Health Organization (WHO), 2017	Nonfatal Health Outcomes	Rabies
China National Iodine Deficiency Disorders Surveillance 1995 as it appears in World Health Organization (WHO). WHO Global Database on Iodine Deficiency. Geneva, Switzerland: World Health Organization (WHO)	Nonfatal Health Outcomes	Iodine deficiency
China National Iodine Deficiency Disorders Surveillance 1997 as it appears in World Health Organization (WHO). WHO Global Database on Iodine Deficiency. Geneva, Switzerland: World Health Organization (WHO)	Nonfatal Health Outcomes	Iodine deficiency
China National Iodine Deficiency Disorders Surveillance 1999 as it appears in World Health Organization (WHO). WHO Global Database on Iodine Deficiency. Geneva, Switzerland: World Health Organization (WHO)	Nonfatal Health Outcomes	Iodine deficiency
Yan YQ, Chen ZP, Yang XM, Liu H, Zhang JX, Zhong W, Yao W, Zhao JK, Zhang ZZ, Hua JL, Li JS, Yu XQ, Wang FR. Attention to the hiding iodine deficiency in pregnant and lactating women after universal salt iodization: A multi-community study in China. J Endocrinol Invest. 2005; 28(6): 547-53 as it appears in World Health Organization (WHO). WHO Global Database on Iodine Deficiency. Geneva, Switzerland: World Health Organization (WHO)	Nonfatal Health Outcomes	Iodine deficiency
China Plasmodium Falciparum Parasite Rate Data, Personal Communication with I. Carneiro and N. Hill 2009 as it appears in Malaria Atlas Project. Malaria Atlas Project Plasmodium Falciparum Parasite Rate Database. Oxford, United Kingdom: Malaria Atlas Project	Nonfatal Health Outcomes	Malaria
Hong Kong Vital Registration - Deaths 1986 ICD9 as it appears in World Health Organization (WHO). WHO Mortality Database Version September 2016. Geneva, Switzerland: World Health Organization (WHO), 2016	Nonfatal Health Outcomes	Rabies
Hong Kong Vital Registration - Deaths 2009 ICD10 as it appears in World Health Organization (WHO). WHO Mortality Database Version October 2017. Geneva, Switzerland: World Health Organization (WHO), 2017	Nonfatal Health Outcomes	Rabies
Hong Kong Vital Registration - Deaths 2008 ICD10 as it appears in World Health Organization (WHO). WHO Mortality Database Version October 2017. Geneva, Switzerland: World Health Organization (WHO), 2017	Nonfatal Health Outcomes	Rabies
Hong Kong Vital Registration - Deaths 2011 ICD10 as it appears in World Health Organization (WHO). WHO Mortality Database Version October 2017. Geneva, Switzerland: World Health Organization (WHO), 2017	Nonfatal Health Outcomes	Rabies
China - Luliang County Rapid Assessment for Avoidable Blindness 2008 as it appears in Bourne R, Global Burden of Disease Vision Loss Expert Group. Vision Loss Database - Survey Data on Vision Loss by Severity and Etiology. [Unpublished]	Nonfatal Health Outcomes	Vision loss
Dang S, Yan H, Wang X. [Study on the hemoglobin levels of children under the age of three years and the prevalence of anemia at high altitude in Tibet of China]. Chin J Epidemiol. 2003; 24(12): 1108-11 as it appears in World Health Organization (WHO). WHO Global Database on Anemia, Nutrition Landscape Information System. Geneva, Switzerland: World Health Organization (WHO)	Nonfatal Health Outcomes	Anemia
Dang S, Yan H, Yamamoto S, Wang X, Zeng L. Poor nutritional status of younger Tibetan children living at high altitudes. Eur J Clin Nutr. 2004; 58(6): 938-46 as it appears in World Health Organization (WHO). WHO Global Database on Anemia, Nutrition Landscape Information System. Geneva, Switzerland: World Health Organization (WHO)	Nonfatal Health Outcomes	Anemia
China - Beijing Eye Study 2001 as it appears in Bourne R, Global Burden of Disease Vision Loss Expert Group. Vision Loss Database - Survey Data on Vision Loss by Severity and Etiology. [Unpublished]	Nonfatal Health Outcomes	Glaucoma
China National Iodine Deficiency Disorders Surveillance 1995 as it appears in World Health Organization (WHO). WHO Global Database on Iodine Deficiency. Geneva, Switzerland: World Health Organization (WHO)	Nonfatal Health Outcomes	Nutritional deficiencies
China National Iodine Deficiency Disorders Surveillance 1997 as it appears in World Health Organization (WHO). WHO Global Database on Iodine Deficiency. Geneva, Switzerland: World Health Organization (WHO)	Nonfatal Health Outcomes	Nutritional deficiencies

China National Iodine Deficiency Disorders Surveillance 1999 as it appears in World Health Organization (WHO). WHO Global Database on Iodine Deficiency. Geneva, Switzerland: World Health Organization (WHO)	Nonfatal Health Outcomes	Nutritional deficiencies
Yan YQ, Chen ZP, Yang XM, Liu H, Zhang JX, Zhong W, Yao W, Zhao JK, Zhang ZZ, Hua JL, Li JS, Yu XQ, Wang FR. Attention to the hiding iodine deficiency in pregnant and lactating women after universal salt iodization: A multi-community study in China. J Endocrinol Invest. 2005; 28(6): 547-53 as it appears in World Health Organization (WHO). WHO Global Database on Iodine Deficiency. Geneva, Switzerland: World Health Organization (WHO)	Nonfatal Health Outcomes	Nutritional deficiencies
Zeng L, Yan H, Chen Z, Dang S, Xie H. [Analysis on the prevalence of anemia among children under 3-year-old in 5 provinces in Western China]. Chin J Epidemiol. 2004; 25(3): 225-8 as it appears in World Health Organization (WHO). WHO Global Database on Anemia, Nutrition Landscape Information System. Geneva, Switzerland: World Health Organization (WHO)	Nonfatal Health Outcomes	Anemia
Hong Kong Vital Registration - Deaths 1980 ICD9 as it appears in World Health Organization (WHO). WHO Mortality Database Version September 2016. Geneva, Switzerland: World Health Organization (WHO), 2016	Nonfatal Health Outcomes	Rabies
Hong Kong Vital Registration - Deaths 1981 ICD9 as it appears in World Health Organization (WHO). WHO Mortality Database Version September 2016. Geneva, Switzerland: World Health Organization (WHO), 2016	Nonfatal Health Outcomes	Rabies
Hong Kong Vital Registration - Deaths 1982 ICD9 as it appears in World Health Organization (WHO). WHO Mortality Database Version September 2016. Geneva, Switzerland: World Health Organization (WHO), 2016	Nonfatal Health Outcomes	Rabies
Hong Kong Vital Registration - Deaths 1983 ICD9 as it appears in World Health Organization (WHO). WHO Mortality Database Version September 2016. Geneva, Switzerland: World Health Organization (WHO), 2016	Nonfatal Health Outcomes	Rabies
Hong Kong Vital Registration - Deaths 1984 ICD9 as it appears in World Health Organization (WHO). WHO Mortality Database Version September 2016. Geneva, Switzerland: World Health Organization (WHO), 2016	Nonfatal Health Outcomes	Rabies
Hong Kong Vital Registration - Deaths 1985 ICD9 as it appears in World Health Organization (WHO). WHO Mortality Database Version September 2016. Geneva, Switzerland: World Health Organization (WHO), 2016	Nonfatal Health Outcomes	Rabies
Hong Kong Vital Registration - Deaths 1987 ICD9 as it appears in World Health Organization (WHO). WHO Mortality Database Version September 2016. Geneva, Switzerland: World Health Organization (WHO), 2016	Nonfatal Health Outcomes	Rabies
Hong Kong Vital Registration - Deaths 1988 ICD9 as it appears in World Health Organization (WHO). WHO Mortality Database Version September 2016. Geneva, Switzerland: World Health Organization (WHO), 2016	Nonfatal Health Outcomes	Rabies
Hong Kong Vital Registration - Deaths 1989 ICD9 as it appears in World Health Organization (WHO). WHO Mortality Database Version September 2016. Geneva, Switzerland: World Health Organization (WHO), 2016	Nonfatal Health Outcomes	Rabies
Hong Kong Vital Registration - Deaths 1990 ICD9 as it appears in World Health Organization (WHO). WHO Mortality Database Version September 2016. Geneva, Switzerland: World Health Organization (WHO), 2016	Nonfatal Health Outcomes	Rabies
China - Luliang County Rapid Assessment for Avoidable Blindness 2008 as it appears in Bourne R, Global Burden of Disease Vision Loss Expert Group. Vision Loss Database - Survey Data on Vision Loss by Severity and Etiology. [Unpublished]	Nonfatal Health Outcomes	Trachoma
China - Sichuan Province Birth Defects Monitoring Program Data 1987 - ICBDMS as it appears in International Clearinghouse for Birth Defects Monitoring Systems. Congenital Malformations Worldwide: A Report from the International Clearinghouse for Birth Defects Monitoring Systems. Amsterdam, Netherlands: Elsevier, 1991	Nonfatal Health Outcomes	Congenital musculoskeletal and limb anomalies
Hesketh T, Ding QJ, Tomkins AM. Disparities in economic development in Eastern China: impact on nutritional status of adolescents. Public Health Nutr. 2002; 5(2): 313-8 as it appears in World Health Organization (WHO). WHO Global Database on Anemia, Nutrition Landscape Information System. Geneva, Switzerland: World Health Organization (WHO)	Nonfatal Health Outcomes	Anemia
China Beijing Birth Defect Surveillance System in Thirty Counties of Four Provinces Data 2005 - ICBDSR as it appears in International Clearinghouse for Birth Defects Surveillance and Research. International Clearinghouse for Birth Defects Surveillance and Research Annual Report 2007. Rome, Italy: International Clearinghouse for Birth Defects Surveillance and Research, 2008	Nonfatal Health Outcomes	Congenital musculoskeletal and limb anomalies
Jia F, Fu Z. [Preliminary analysis of the hemoglobin levels of children under 6 years of age]. J Hyg Res. 2000; 29(5): 298-9 as it appears in World Health Organization (WHO). WHO Global Database on Anemia, Nutrition Landscape Information System. Geneva, Switzerland: World Health Organization (WHO)	Nonfatal Health Outcomes	Anemia

Wang W, Wu Y, Chen X, Liu D, Feng L, Yu S, Sun A, Jin Y. Studies on iron deficiency anemia of preschool children, I: a survey of iron deficiency anemia among preschool children in Beijing [in Chinese]. Acta Nutri Sin. 1983; 73-8 as it appears in World Health Organization (WHO). WHO Global Database on Anemia, Nutrition Landscape Information System. Geneva, Switzerland: World Health Organization (WHO)	Nonfatal Health Outcomes	Anemia
Macao Vital Registration Birth Data 1990 as it appears in United Nations Statistics Division (UNSD). United Nations Demographic Yearbook - Historical Supplement 1997. New York, United States: United Nations (UN)	Nonfatal Health Outcomes	Premenstrual syndrome
China Beijing Birth Defect Surveillance System in Thirty Counties of Four Provinces Data 2005 - ICBDSR as it appears in International Clearinghouse for Birth Defects Surveillance and Research. International Clearinghouse for Birth Defects Surveillance and Research Annual Report 2007. Rome, Italy: International Clearinghouse for Birth Defects Surveillance and Research, 2008	Nonfatal Health Outcomes	Congenital birth defects
China's National Group On Students' Constitution And Health Survey, Ji C. Dynamic analysis on the detecting rate of iron-deficient anemia of Chinese students. Chin J Prev Med. 2002; 36(2): 81-3 as it appears in World Health Organization (WHO). WHO Global Database on Anemia, Nutrition Landscape Information System. Geneva, Switzerland: World Health Organization (WHO)	Nonfatal Health Outcomes	Dietary iron deficiency
China's National Group On Students' Constitution And Health Survey, Ji C. Dynamic analysis on the detecting rate of iron-deficient anemia of Chinese students. Chin J Prev Med. 2002; 36(2): 81-3 as it appears in World Health Organization (WHO). WHO Global Database on Anemia, Nutrition Landscape Information System. Geneva, Switzerland: World Health Organization (WHO)	Nonfatal Health Outcomes	Anemia
China Prevalence of Anemia in China by Age and Gender 2002 as it appears in World Health Organization (WHO). WHO Global Database on Anemia, Nutrition Landscape Information System. Geneva, Switzerland: World Health Organization (WHO)	Nonfatal Health Outcomes	Dietary iron deficiency
Hong Kong Vital Registration Birth Data 1990 as it appears in United Nations Statistics Division (UNSD). United Nations Demographic Yearbook - Historical Supplement 1997. New York, United States: United Nations (UN)	Nonfatal Health Outcomes	Premenstrual syndrome
China Vital Registration Birth Data 1989 as it appears in United Nations Statistics Division (UNSD). United Nations Demographic Yearbook - Historical Supplement 1997. New York, United States: United Nations (UN)	Nonfatal Health Outcomes	Premenstrual syndrome
China - Luliang County Rapid Assessment for Avoidable Blindness 2008 as it appears in Bourne R, Global Burden of Disease Vision Loss Expert Group. Vision Loss Database - Survey Data on Vision Loss by Severity and Etiology. [Unpublished]	Nonfatal Health Outcomes	Cataract
China - Luliang County Rapid Assessment for Avoidable Blindness 2008 as it appears in Bourne R, Global Burden of Disease Vision Loss Expert Group. Vision Loss Database - Survey Data on Vision Loss by Severity and Etiology. [Unpublished]	Nonfatal Health Outcomes	Refraction and accommodation disorders
China - Luliang County Rapid Assessment for Avoidable Blindness 2008 as it appears in Bourne R, Global Burden of Disease Vision Loss Expert Group. Vision Loss Database - Survey Data on Vision Loss by Severity and Etiology. [Unpublished]	Nonfatal Health Outcomes	Refraction disorders
Chang Y, Zhai F, Li W, Ge K, Jin D, de Onis M. Nutritional status of preschool children in poor rural areas of China. Bull World Health Organ. 1994; 72(1): 105-12 as it appears in World Health Organization (WHO). WHO Global Database on Anemia, Nutrition Landscape Information System. Geneva, Switzerland: World Health Organization (WHO)	Nonfatal Health Outcomes	Anemia
He Y, Zhai F, Li Y, Jia F. [Effect of nutrition on the health improvement of preschool children in rural areas]. J Hyg Res. 1999; 28(3): 172-5 as it appears in World Health Organization (WHO). WHO Global Database on Anemia, Nutrition Landscape Information System. Geneva, Switzerland: World Health Organization (WHO)	Nonfatal Health Outcomes	Anemia
Ma A-G, Chen X-C, Wang Y, Xu R-X, Zheng M-C, Li J-S. The multiple vitamin status of Chinese pregnant women with anemia and nonanemia in the last trimester. J Nutri Sci Vitaminol. 2004; 50(2): 87-92 as it appears in World Health Organization (WHO). WHO Global Database on Anemia, Nutrition Landscape Information System. Geneva, Switzerland: World Health Organization (WHO)	Nonfatal Health Outcomes	Anemia
Shi Z, Lien N, Kumar BN, Dalen I, Holmboe-Ottesen G. The sociodemographic correlates of nutritional status of school adolescents in Jiangsu Province, China. J Adolesc Health. 2005; 37(4): 313-22 as it appears in World Health Organization (WHO). WHO Global Database on Anemia, Nutrition Landscape Information System. Geneva, Switzerland: World Health Organization (WHO)	Nonfatal Health Outcomes	Anemia
China Prevalence of Anemia in China by Age and Gender 2002 as it appears in World Health Organization (WHO). WHO Global Database on Anemia, Nutrition Landscape Information System. Geneva, Switzerland: World Health Organization (WHO)	Nonfatal Health Outcomes	Anemia

Chinese Birth Defects Monitoring Network Data 2001 - ICBDMS as it appears in International Clearinghouse for Birth Defects Monitoring Systems. International Clearinghouse for Birth Defects Monitoring Systems Annual Report 2003. Rome, Italy: International Centre on Birth Defects	Nonfatal Health Outcomes	Congenital musculoskeletal and limb anomalies
China Beijing Birth Defect Surveillance System in Thirty Counties of Four Provinces Data 2002 - ICBDMS as it appears in International Clearinghouse for Birth Defects Monitoring Systems. International Clearinghouse for Birth Defects Monitoring Systems Annual Report 2004. Rome, Italy: International Centre on Birth Defects, 2006	Nonfatal Health Outcomes	Congenital musculoskeletal and limb anomalies
Chinese Birth Defects Monitoring Network Data 2003 - ICBDSR as it appears in International Clearinghouse for Birth Defects Surveillance and Research. International Clearinghouse for Birth Defects Surveillance and Research Annual Report 2005. Rome, Italy: International Clearinghouse for Birth Defects Surveillance and Research, 2007	Nonfatal Health Outcomes	Congenital musculoskeletal and limb anomalies
Chinese Birth Defects Monitoring Network Data 2004 - ICBDSR as it appears in International Clearinghouse for Birth Defects Surveillance and Research. International Clearinghouse for Birth Defects Surveillance and Research Annual Report 2006. Rome, Italy: International Clearinghouse for Birth Defects Surveillance and Research, 2007	Nonfatal Health Outcomes	Congenital heart anomalies
Wang W, Chen X, Liu D. Hematologic response to iron and ascorbic acid administration in preschool children with anemia. Nutr Res. 1986; 241-8 as it appears in World Health Organization (WHO). WHO Global Database on Anemia, Nutrition Landscape Information System. Geneva, Switzerland: World Health Organization (WHO)	Nonfatal Health Outcomes	Anemia
Hu L, Jin X. [Epidemiology of Malaria in Jiangsu, Shangdong, Henan, Anhui and Hebei Provinces in 2004 and 2005]. J Public Health Prev Med. 2006; 17(6): 54-9 as it appears in Malaria Atlas Project. Malaria Atlas Project Plasmodium Falciparum Parasite Rate Database. Oxford, United Kingdom: Malaria Atlas Project	Nonfatal Health Outcomes	Malaria
China Beijing Birth Defect Surveillance System in Thirty Counties of Four Provinces Data 1997-1998 - WHO as it appears in European Surveillance of Congenital Anomalies (EUROCAT), International Centre on Birth Defects, World Health Organization (WHO). World Atlas of Birth Defects. 2nd ed. Geneva, Switzerland: World Health Organization (WHO), 2003	Nonfatal Health Outcomes	Congenital musculoskeletal and limb anomalies
China Beijing Birth Defect Surveillance System in Thirty Counties of Four Provinces Data 2001 - ICBDMS as it appears in International Clearinghouse for Birth Defects Monitoring Systems. International Clearinghouse for Birth Defects Monitoring Systems Annual Report 2003. Rome, Italy: International Centre on Birth Defects	Nonfatal Health Outcomes	Congenital musculoskeletal and limb anomalies
China Beijing Birth Defect Surveillance System in Thirty Counties of Four Provinces Data 2003 - ICBDSR as it appears in International Clearinghouse for Birth Defects Surveillance and Research. International Clearinghouse for Birth Defects Surveillance and Research Annual Report 2005. Rome, Italy: International Clearinghouse for Birth Defects Surveillance and Research, 2007	Nonfatal Health Outcomes	Congenital musculoskeletal and limb anomalies
China Beijing Birth Defect Surveillance System in Thirty Counties of Four Provinces Data 2004 - ICBDSR as it appears in International Clearinghouse for Birth Defects Surveillance and Research. International Clearinghouse for Birth Defects Surveillance and Research Annual Report 2006. Rome, Italy: International Clearinghouse for Birth Defects Surveillance and Research, 2007	Nonfatal Health Outcomes	Congenital musculoskeletal and limb anomalies
Chinese Birth Defects Monitoring Network Data 2004 - ICBDSR as it appears in International Clearinghouse for Birth Defects Surveillance and Research. International Clearinghouse for Birth Defects Surveillance and Research Annual Report 2006. Rome, Italy: International Clearinghouse for Birth Defects Surveillance and Research, 2007	Nonfatal Health Outcomes	Congenital musculoskeletal and limb anomalies
China - Sichuan Province Birth Defects Monitoring Program Data 1985 - ICBDMS as it appears in International Clearinghouse for Birth Defects Monitoring Systems. Congenital Malformations Worldwide: A Report from the International Clearinghouse for Birth Defects Monitoring Systems. Amsterdam, Netherlands: Elsevier, 1991	Nonfatal Health Outcomes	Congenital birth defects
China - Sichuan Province Birth Defects Monitoring Program Data 1986 - ICBDMS as it appears in International Clearinghouse for Birth Defects Monitoring Systems. Congenital Malformations Worldwide: A Report from the International Clearinghouse for Birth Defects Monitoring Systems. Amsterdam, Netherlands: Elsevier, 1991	Nonfatal Health Outcomes	Congenital birth defects

China - Sichuan Province Birth Defects Monitoring Program Data 1987 - ICBDMS as it appears in International Clearinghouse for Birth Defects Monitoring Systems. Congenital Malformations Worldwide: A Report from the International Clearinghouse for Birth Defects Monitoring Systems. Amsterdam, Netherlands: Elsevier, 1991	Nonfatal Health Outcomes	Congenital birth defects
China - Sichuan Province Birth Defects Monitoring Program Data 1988 - ICBDMS as it appears in International Clearinghouse for Birth Defects Monitoring Systems. Congenital Malformations Worldwide: A Report from the International Clearinghouse for Birth Defects Monitoring Systems. Amsterdam, Netherlands: Elsevier, 1991	Nonfatal Health Outcomes	Congenital birth defects
Chinese Birth Defects Monitoring Network Data 2001 - ICBDMS as it appears in International Clearinghouse for Birth Defects Monitoring Systems. International Clearinghouse for Birth Defects Monitoring Systems Annual Report 2003. Rome, Italy: International Centre on Birth Defects	Nonfatal Health Outcomes	Congenital birth defects
Chinese Birth Defects Monitoring Network Data 2003 - ICBDSR as it appears in International Clearinghouse for Birth Defects Surveillance and Research. International Clearinghouse for Birth Defects Surveillance and Research Annual Report 2005. Rome, Italy: International Clearinghouse for Birth Defects Surveillance and Research, 2007	Nonfatal Health Outcomes	Congenital birth defects
China - Beijing Eye Study 2001 as it appears in Bourne R, Global Burden of Disease Vision Loss Expert Group. Vision Loss Database - Survey Data on Vision Loss by Severity and Etiology. [Unpublished]	Nonfatal Health Outcomes	Cataract
China - Beijing Eye Study 2001 as it appears in Bourne R, Global Burden of Disease Vision Loss Expert Group. Vision Loss Database - Survey Data on Vision Loss by Severity and Etiology. [Unpublished]	Nonfatal Health Outcomes	Age-related macular degeneration
Luo D, Lu D, Yao R, Li P, Huo X, Li A, Wen L, Ge C, Zhang S, Huo H. Alphamethrin-impregnated bed nets for malaria and mosquito control in China. Trans R Soc Trop Med Hyg. 1994; 88(6): 625-8 as it appears in Malaria Atlas Project. Malaria Atlas Project Plasmodium Falciparum Parasite Rate Database. Oxford, United Kingdom: Malaria Atlas Project	Nonfatal Health Outcomes	Malaria
Chinese Birth Defects Monitoring Network Data 2002 - ICBDMS as it appears in International Clearinghouse for Birth Defects Monitoring Systems. International Clearinghouse for Birth Defects Monitoring Systems Annual Report 2004. Rome, Italy: International Centre on Birth Defects, 2006	Nonfatal Health Outcomes	Congenital birth defects
China Plasmodium Falciparum Parasite Rate Data, Personal Communication with H. Zhang 2006 as it appears in Malaria Atlas Project. Malaria Atlas Project Plasmodium Falciparum Parasite Rate Database. Oxford, United Kingdom: Malaria Atlas Project	Nonfatal Health Outcomes	Malaria
Liu J, Meng F, Hua D. [Malaria survey in Nangiao Township of Hainan Province]. Chin J Parasitol Parasit Dis. 2002; 20(2): 128-9 as it appears in Malaria Atlas Project. Malaria Atlas Project Plasmodium Falciparum Parasite Rate Database. Oxford, United Kingdom: Malaria Atlas Project	Nonfatal Health Outcomes	Malaria
Li C, Zhang Z. [Malaria surveillance in newly developed areas in Jinghong County]. Chin J Parasit Dis Cont. 2006; 15(3): 140 as it appears in Malaria Atlas Project. Malaria Atlas Project Plasmodium Falciparum Parasite Rate Database. Oxford, United Kingdom: Malaria Atlas Project	Nonfatal Health Outcomes	Malaria
Chinese Birth Defects Monitoring Network Data 2004 - ICBDSR as it appears in International Clearinghouse for Birth Defects Surveillance and Research. International Clearinghouse for Birth Defects Surveillance and Research Annual Report 2006. Rome, Italy: International Clearinghouse for Birth Defects Surveillance and Research, 2007	Nonfatal Health Outcomes	Congenital birth defects
Chinese Birth Defects Monitoring Network Data 2005 - ICBDSR as it appears in International Clearinghouse for Birth Defects Surveillance and Research. International Clearinghouse for Birth Defects Surveillance and Research Annual Report 2007. Rome, Italy: International Clearinghouse for Birth Defects Surveillance and Research, 2008	Nonfatal Health Outcomes	Congenital birth defects
He R. The reason of Plasmodium vivax outbreaks and its prevention at the later anti-malarial stage. Chin J Pest Control. 1995; 11(4): 394-5 as it appears in Malaria Atlas Project. Malaria Atlas Project Plasmodium Falciparum Parasite Rate Database. Oxford, United Kingdom: Malaria Atlas Project	Nonfatal Health Outcomes	Malaria
Li C, Ce L, Yang T. Malaria survey of children in Beihe Village, Mengla County. J Pract Parasit Dis. 1998; 6(2): 71 as it appears in Malaria Atlas Project. Malaria Atlas Project Plasmodium Falciparum Parasite Rate Database. Oxford, United Kingdom: Malaria Atlas Project	Nonfatal Health Outcomes	Malaria

China Beijing Birth Defect Surveillance System in Thirty Counties of Four Provinces Data 2002 - ICBDMS as it appears in International Clearinghouse for Birth Defects Monitoring Systems. International Clearinghouse for Birth Defects Monitoring Systems Annual Report 2004. Rome, Italy: International Centre on Birth Defects, 2006	Nonfatal Health Outcomes	Digestive congenital anomalies
Luo MZ, Zheng X, Shang LY, Chen JF, Zhu WD, Tang LH. [Practicability of IFAT using Plasmodium cynomolgi and Plasmodium falciparum antigens in different malarious areas]. Chin J Parasitol Parasit Dis. 2000; 18(1): 49-51 as it appears in Malaria Atlas Project. Malaria Atlas Project Plasmodium Falciparum Parasite Rate Database. Oxford, United Kingdom: Malaria Atlas Project	Nonfatal Health Outcomes	Malaria
Huang G, Gu Z, Huang X, Zheng X, Xia Z, Yang Q, Ye Z, S T, Li H, Tang L. Study on the transmission threshold of malaria by Anopheles anthropophagus in hilly land of Hubei Province. Chin J Parasit Dis Cont. 2003; 16(3): 131-3 as it appears in Malaria Atlas Project. Malaria Atlas Project Plasmodium Falciparum Parasite Rate Database. Oxford, United Kingdom: Malaria Atlas Project	Nonfatal Health Outcomes	Malaria
Li C, Liu H. [Epidemiologic report of malaria at Nuozha Power Station area]. Chin J Parasit Dis Cont. 2003; 16(5): 7 as it appears in Malaria Atlas Project. Malaria Atlas Project Plasmodium Falciparum Parasite Rate Database. Oxford, United Kingdom: Malaria Atlas Project	Nonfatal Health Outcomes	Malaria
Jin X, Gao Q. [Analysis of basline survey of malaria prevalent status in Sihong Town, Jiangsu Province]. China Tropical Medicine. 2004; 4(5): 714-6 as it appears in Malaria Atlas Project. Malaria Atlas Project Plasmodium Falciparum Parasite Rate Database. Oxford, United Kingdom: Malaria Atlas Project	Nonfatal Health Outcomes	Malaria
Zhang Y, Fan ML. [Investigation of rising malaria prevalence in local areas of Fuyang City]. Anhui J Prev Med. 2002; 8(6): 373-4 as it appears in Malaria Atlas Project. Malaria Atlas Project Plasmodium Falciparum Parasite Rate Database. Oxford, United Kingdom: Malaria Atlas Project	Nonfatal Health Outcomes	Malaria
Li G. [Characteristics of endogenic point endemic malaria in Wandien Township, Changning County]. Chin J Parasit Dis Cont. 2004; 17(6): 3 as it appears in Malaria Atlas Project. Malaria Atlas Project Plasmodium Falciparum Parasite Rate Database. Oxford, United Kingdom: Malaria Atlas Project	Nonfatal Health Outcomes	Malaria
Zhang Z-X, Zhou H-N, Zhao X-T, Chang F-X, Wang H-J, Li X-J, Zhuoma Y-J, Ciren Q, Bianma Z, Sangdan L, Zhang W, Yong J, Xu H-M, Bian J, Wang L-Y. [Epidemiological survey on malaria situation in Motuo County of Tibet, China]. Chin J Parasitol Parasit Dis. 2008; 26(5): 343-8 as it appears in Malaria Atlas Project. Malaria Atlas Project Plasmodium Falciparum Parasite Rate Database. Oxford, United Kingdom: Malaria Atlas Project	Nonfatal Health Outcomes	Malaria
China Beijing Birth Defect Surveillance System in Thirty Counties of Four Provinces Data 2001 - ICBDMS as it appears in International Clearinghouse for Birth Defects Monitoring Systems. International Clearinghouse for Birth Defects Monitoring Systems Annual Report 2003. Rome, Italy: International Centre on Birth Defects	Nonfatal Health Outcomes	Digestive congenital anomalies
China Beijing Birth Defect Surveillance System in Thirty Counties of Four Provinces Data 2004 - ICBDSR as it appears in International Clearinghouse for Birth Defects Surveillance and Research. International Clearinghouse for Birth Defects Surveillance and Research Annual Report 2006. Rome, Italy: International Clearinghouse for Birth Defects Surveillance and Research, 2007	Nonfatal Health Outcomes	Digestive congenital anomalies
China - Beijing Eye Study 2001 as it appears in Xu J, Xu L, Du KF, Shao L, Chen CX, Zhou JQ, Wang YX, You QS, Jonas JB, Wei WB. Subfoveal choroidal thickness in diabetes and diabetic retinopathy [Unpublished data]. Ophthalmology. 2013 Oct;120(10):2023-8	Nonfatal Health Outcomes	Vision loss
Xiong X, Buekens P, Fraser WD, Guo Z. Anemia during pregnancy in a Chinese population. Int J Gynaecol Obstet. 2003; 83(2): 159-64 as it appears in World Health Organization (WHO). WHO Global Database on Anemia, Nutrition Landscape Information System. Geneva, Switzerland: World Health Organization (WHO)	Nonfatal Health Outcomes	Anemia
China Beijing Birth Defect Surveillance System in Thirty Counties of Four Provinces Data 2001 - ICBDMS as it appears in International Clearinghouse for Birth Defects Monitoring Systems. International Clearinghouse for Birth Defects Monitoring Systems Annual Report 2003. Rome, Italy: International Centre on Birth Defects	Nonfatal Health Outcomes	Congenital birth defects
China Beijing Birth Defect Surveillance System in Thirty Counties of Four Provinces Data 2003 - ICBDSR as it appears in International Clearinghouse for Birth Defects Surveillance and Research. International Clearinghouse for Birth Defects Surveillance and Research Annual Report 2005. Rome, Italy: International Clearinghouse for Birth Defects Surveillance and Research, 2007	Nonfatal Health Outcomes	Congenital heart anomalies

China Beijing Birth Defect Surveillance System in Thirty Counties of Four Provinces Data 2004 - ICBDSR as it appears in International Clearinghouse for Birth Defects Surveillance and Research. International Clearinghouse for Birth Defects Surveillance and Research Annual Report 2006. Rome, Italy: International Clearinghouse for Birth Defects Surveillance and Research, 2007	Nonfatal Health Outcomes	Congenital heart anomalies
China Beijing Birth Defect Surveillance System in Thirty Counties of Four Provinces Data 1997-1998 - WHO as it appears in European Surveillance of Congenital Anomalies (EUROCAT), International Centre on Birth Defects, World Health Organization (WHO). World Atlas of Birth Defects. 2nd ed. Geneva, Switzerland: World Health Organization (WHO), 2003	Nonfatal Health Outcomes	Congenital birth defects
China Beijing Birth Defect Surveillance System in Thirty Counties of Four Provinces Data 2002 - ICBDMS as it appears in International Clearinghouse for Birth Defects Monitoring Systems. International Clearinghouse for Birth Defects Monitoring Systems Annual Report 2004. Rome, Italy: International Centre on Birth Defects, 2006	Nonfatal Health Outcomes	Congenital birth defects
China Beijing Birth Defect Surveillance System in Thirty Counties of Four Provinces Data 2003 - ICBDSR as it appears in International Clearinghouse for Birth Defects Surveillance and Research. International Clearinghouse for Birth Defects Surveillance and Research Annual Report 2005. Rome, Italy: International Clearinghouse for Birth Defects Surveillance and Research, 2007	Nonfatal Health Outcomes	Congenital birth defects
China Beijing Birth Defect Surveillance System in Thirty Counties of Four Provinces Data 2004 - ICBDSR as it appears in International Clearinghouse for Birth Defects Surveillance and Research. International Clearinghouse for Birth Defects Surveillance and Research Annual Report 2006. Rome, Italy: International Clearinghouse for Birth Defects Surveillance and Research, 2007	Nonfatal Health Outcomes	Congenital birth defects
Ou C, Wang X. Children infected with malaria parasites detected by PRC method in the epidemic area of malaria in Hainan. Hainan Med J. 1998; 2(13): 73-4 as it appears in Malaria Atlas Project. Malaria Atlas Project Plasmodium Falciparum Parasite Rate Database. Oxford, United Kingdom: Malaria Atlas Project	Nonfatal Health Outcomes	Malaria
China Beijing Birth Defect Surveillance System in Thirty Counties of Four Provinces Data 2003 - ICBDSR as it appears in International Clearinghouse for Birth Defects Surveillance and Research. International Clearinghouse for Birth Defects Surveillance and Research Annual Report 2005. Rome, Italy: International Clearinghouse for Birth Defects Surveillance and Research, 2007	Nonfatal Health Outcomes	Digestive congenital anomalies
China Beijing Birth Defect Surveillance System in Thirty Counties of Four Provinces Data 2005 - ICBDSR as it appears in International Clearinghouse for Birth Defects Surveillance and Research. International Clearinghouse for Birth Defects Surveillance and Research Annual Report 2007. Rome, Italy: International Clearinghouse for Birth Defects Surveillance and Research, 2008	Nonfatal Health Outcomes	Digestive congenital anomalies
Chinese Birth Defects Monitoring Network Data 1997-1998 - WHO as it appears in European Surveillance of Congenital Anomalies (EUROCAT), International Centre on Birth Defects, World Health Organization (WHO). World Atlas of Birth Defects. 2nd ed. Geneva, Switzerland: World Health Organization (WHO), 2003	Nonfatal Health Outcomes	Congenital musculoskeletal and limb anomalies
Chinese Birth Defects Monitoring Network Data 1997-1998 - WHO as it appears in European Surveillance of Congenital Anomalies (EUROCAT), International Centre on Birth Defects, World Health Organization (WHO). World Atlas of Birth Defects. 2nd ed. Geneva, Switzerland: World Health Organization (WHO), 2003	Nonfatal Health Outcomes	Digestive congenital anomalies
China - Sichuan Province Birth Defects Monitoring Program Data 1985 - ICBDMS as it appears in International Clearinghouse for Birth Defects Monitoring Systems. Congenital Malformations Worldwide: A Report from the International Clearinghouse for Birth Defects Monitoring Systems. Amsterdam, Netherlands: Elsevier, 1991	Nonfatal Health Outcomes	Congenital musculoskeletal and limb anomalies
China - Sichuan Province Birth Defects Monitoring Program Data 1986 - ICBDMS as it appears in International Clearinghouse for Birth Defects Monitoring Systems. Congenital Malformations Worldwide: A Report from the International Clearinghouse for Birth Defects Monitoring Systems. Amsterdam, Netherlands: Elsevier, 1991	Nonfatal Health Outcomes	Congenital musculoskeletal and limb anomalies
China - Sichuan Province Birth Defects Monitoring Program Data 1988 - ICBDMS as it appears in International Clearinghouse for Birth Defects Monitoring Systems. Congenital Malformations Worldwide: A Report from the International Clearinghouse for Birth Defects Monitoring Systems. Amsterdam, Netherlands: Elsevier, 1991	Nonfatal Health Outcomes	Congenital musculoskeletal and limb anomalies
Chinese Birth Defects Monitoring Network Data 2002 - ICBDMS as it appears in International Clearinghouse for Birth Defects Monitoring Systems. International Clearinghouse for Birth Defects Monitoring Systems Annual Report 2004. Rome, Italy: International Centre on Birth Defects, 2006	Nonfatal Health Outcomes	Congenital musculoskeletal and limb anomalies

Chinese Birth Defects Monitoring Network Data 2005 - ICBDSR as it appears in International Clearinghouse for Birth Defects Surveillance and Research. International Clearinghouse for Birth Defects Surveillance and Research Annual Report 2007. Rome, Italy: International Clearinghouse for Birth Defects Surveillance and Research, 2008	Nonfatal Health Outcomes	Congenital musculoskeletal and limb anomalies
Gao J, Zeng S, Sun BL, Fan HM, Han LH. Menstrual blood loss and hematologic indices in healthy Chinese women. J Reprod Med. 1987; 32(11): 822-6 as it appears in World Health Organization (WHO). WHO Global Database on Anemia, Nutrition Landscape Information System. Geneva, Switzerland: World Health Organization (WHO)	Nonfatal Health Outcomes	Anemia
Leung SS, Davies DP, Lui S, Lo L, Yuen P, Swaminathan R. Iron deficiency is uncommon in healthy Hong Kong infants at 18 months. J Trop Pediatr. 1988; 34(3): 100-3 as it appears in World Health Organization (WHO). WHO Global Database on Anemia, Nutrition Landscape Information System. Geneva, Switzerland: World Health Organization (WHO)	Nonfatal Health Outcomes	Anemia
Todd D, Kan PS. Anaemia in pregnancy in Hong Kong. J Obstet Gynaecol Br Commonw. 1965; 72(5): 738-44 as it appears in World Health Organization (WHO). WHO Global Database on Anemia, Nutrition Landscape Information System. Geneva, Switzerland: World Health Organization (WHO)	Nonfatal Health Outcomes	Anemia
Li AM, Cheng MY. Anaemia and thalassaemia in healthy adolescents from southern Chinese families. J Paediatr Child Health. 1990; 26(6): 339-42 as it appears in World Health Organization (WHO). WHO Global Database on Anemia, Nutrition Landscape Information System. Geneva, Switzerland: World Health Organization (WHO)	Nonfatal Health Outcomes	Anemia
Lao TT, Pun TC. Anaemia in pregnancy--is the current definition meaningful?. Eur J Obstet Gynecol Reprod Biol. 1996; 68(1-2): 53-8 as it appears in World Health Organization (WHO). WHO Global Database on Anemia, Nutrition Landscape Information System. Geneva, Switzerland: World Health Organization (WHO)	Nonfatal Health Outcomes	Anemia
China - Sichuan Province Birth Defects Monitoring Program Data 1985 - ICBDMS as it appears in International Clearinghouse for Birth Defects Monitoring Systems. Congenital Malformations Worldwide: A Report from the International Clearinghouse for Birth Defects Monitoring Systems. Amsterdam, Netherlands: Elsevier, 1991	Nonfatal Health Outcomes	Digestive congenital anomalies
China - Sichuan Province Birth Defects Monitoring Program Data 1986 - ICBDMS as it appears in International Clearinghouse for Birth Defects Monitoring Systems. Congenital Malformations Worldwide: A Report from the International Clearinghouse for Birth Defects Monitoring Systems. Amsterdam, Netherlands: Elsevier, 1991	Nonfatal Health Outcomes	Digestive congenital anomalies
China - Sichuan Province Birth Defects Monitoring Program Data 1987 - ICBDMS as it appears in International Clearinghouse for Birth Defects Monitoring Systems. Congenital Malformations Worldwide: A Report from the International Clearinghouse for Birth Defects Monitoring Systems. Amsterdam, Netherlands: Elsevier, 1991	Nonfatal Health Outcomes	Digestive congenital anomalies
China - Sichuan Province Birth Defects Monitoring Program Data 1988 - ICBDMS as it appears in International Clearinghouse for Birth Defects Monitoring Systems. Congenital Malformations Worldwide: A Report from the International Clearinghouse for Birth Defects Monitoring Systems. Amsterdam, Netherlands: Elsevier, 1991	Nonfatal Health Outcomes	Digestive congenital anomalies
Chinese Birth Defects Monitoring Network Data 2001 - ICBDMS as it appears in International Clearinghouse for Birth Defects Monitoring Systems. International Clearinghouse for Birth Defects Monitoring Systems Annual Report 2003. Rome, Italy: International Centre on Birth Defects	Nonfatal Health Outcomes	Digestive congenital anomalies
Chinese Birth Defects Monitoring Network Data 2002 - ICBDMS as it appears in International Clearinghouse for Birth Defects Monitoring Systems. International Clearinghouse for Birth Defects Monitoring Systems Annual Report 2004. Rome, Italy: International Centre on Birth Defects, 2006	Nonfatal Health Outcomes	Digestive congenital anomalies
Chinese Birth Defects Monitoring Network Data 2003 - ICBDSR as it appears in International Clearinghouse for Birth Defects Surveillance and Research. International Clearinghouse for Birth Defects Surveillance and Research Annual Report 2005. Rome, Italy: International Clearinghouse for Birth Defects Surveillance and Research, 2007	Nonfatal Health Outcomes	Digestive congenital anomalies
Chinese Birth Defects Monitoring Network Data 2004 - ICBDSR as it appears in International Clearinghouse for Birth Defects Surveillance and Research. International Clearinghouse for Birth Defects Surveillance and Research Annual Report 2006. Rome, Italy: International Clearinghouse for Birth Defects Surveillance and Research, 2007	Nonfatal Health Outcomes	Digestive congenital anomalies

Chinese Birth Defects Monitoring Network Data 2005 - ICBDSR as it appears in International Clearinghouse for Birth Defects Surveillance and Research. International Clearinghouse for Birth Defects Surveillance and Research Annual Report 2007. Rome, Italy: International Clearinghouse for Birth Defects Surveillance and Research, 2008	Nonfatal Health Outcomes	Digestive congenital anomalies
China - Luliang County Rapid Assessment for Avoidable Blindness 2008 as it appears in Bourne R, Global Burden of Disease Vision Loss Expert Group. Vision Loss Database - Survey Data on Vision Loss by Severity and Etiology. [Unpublished]	Nonfatal Health Outcomes	
Chinese Birth Defects Monitoring Network Data 2003 - ICBDSR as it appears in International Clearinghouse for Birth Defects Surveillance and Research. International Clearinghouse for Birth Defects Surveillance and Research Annual Report 2005. Rome, Italy: International Clearinghouse for Birth Defects Surveillance and Research, 2007	Nonfatal Health Outcomes	Congenital heart anomalies
Chinese Birth Defects Monitoring Network Data 2001 - ICBDMS as it appears in International Clearinghouse for Birth Defects Monitoring Systems. International Clearinghouse for Birth Defects Monitoring Systems Annual Report 2003. Rome, Italy: International Centre on Birth Defects	Nonfatal Health Outcomes	Congenital heart anomalies
Chinese Birth Defects Monitoring Network Data 1997-1998 - WHO as it appears in European Surveillance of Congenital Anomalies (EUROCAT), International Centre on Birth Defects, World Health Organization (WHO). World Atlas of Birth Defects. 2nd ed. Geneva, Switzerland: World Health Organization (WHO), 2003	Nonfatal Health Outcomes	Congenital birth defects
China Beijing Birth Defect Surveillance System in Thirty Counties of Four Provinces Data 2001 - ICBDMS as it appears in International Clearinghouse for Birth Defects Monitoring Systems. International Clearinghouse for Birth Defects Monitoring Systems Annual Report 2003. Rome, Italy: International Centre on Birth Defects	Nonfatal Health Outcomes	Congenital heart anomalies

Summary Exposure Values for the top 10 risk factors in China in 1990 and 2017 for all ages, both sexes

1990

Location name	Ambient particulate matter pollution	Smoking	Alcohol use	High fasting plasma glucose	High systolic blood pressure	High body-mass index	Diet low in fruits	Diet low in whole grains	Diet high in sodium	High LDL cholesterol
China	29.19 (22.05 -- 38.88)	6.54 (5.58 -- 7.56)	7.29 (5.05 -- 9.98)	1.17 (0.83 -- 1.56)	5.08 (4.5 -- 5.7)	3.25 (2.08 -- 5.01)	49.47 (45.02 -- 51.32)	40.24 (37.88 -- 42.28)	43.62 (39.43 -- 46.84)	9.69 (8.31 -- 11.23)
Taiwan	33.34 (20.29 -- 48.92)	6.57 (5.67 -- 7.52)	7.42 (4.02 -- 11.53)	2.35 (1.56 -- 3.4)	6.08 (5.45 -- 6.72)	9.3 (7.18 -- 12.5)	27.05 (20.99 -- 31.82)	39.22 (36.65 -- 41.35)	8.64 (2.82 -- 15.13)	12.87 (11.4 -- 14.49)
Hong Kong Special Administrative Region of China	33.92 (22.75 -- 50.08)	5.57 (4.19 -- 7.13)	10.16 (5.67 -- 14.63)	1.17 (0.72 -- 1.88)	4.55 (3.99 -- 5.17)	7.44 (5.35 -- 10.32)	32.94 (27.27 -- 36.83)	30.73 (26.18 -- 34.51)	51.64 (49.55 -- 53.12)	14.02 (12.44 -- 15.7)
Macau Special Administrative Region of China	36.12 (25.21 -- 51.8)	7.77 (5.92 -- 9.71)	8.44 (0.93 -- 13.76)	1.28 (0.78 -- 2.09)	5.34 (4.73 -- 6.02)	7.6 (5.63 -- 10.4)	39.95 (34.78 -- 43.08)	30.99 (26.37 -- 34.62)	51.66 (49.79 -- 53.18)	12.72 (11.24 -- 14.38)
Anhui	26.33 (16.9 -- 39.09)	6.8 (5.25 -- 8.42)	6.81 (3.73 -- 10.75)	1.21 (0.79 -- 1.77)	5.54 (4.86 -- 6.24)	3 (1.86 -- 4.72)	50.42 (45.79 -- 52.29)	41.08 (38.49 -- 43.26)	50.78 (47.77 -- 52.89)	9.05 (7.69 -- 10.58)
Beijing	63.03 (45.3 -- 87.18)	4.99 (3.67 -- 6.48)	8.51 (4.74 -- 13.01)	1.29 (0.88 -- 1.84)	5.46 (4.83 -- 6.15)	6.6 (4.49 -- 9.6)	45.66 (40.42 -- 48.51)	36.64 (33.28 -- 39.55)	49.98 (46.31 -- 52.64)	8.46 (7.12 -- 9.93)
Chongqing	29.03 (18.49 -- 42.33)	6.59 (5.07 -- 8.3)	7.38 (4.04 -- 11.27)	1.01 (0.7 -- 1.41)	5.55 (4.85 -- 6.28)	3.07 (1.92 -- 4.82)	50.23 (45.66 -- 52.09)	40.85 (38.2 -- 43.11)	38.74 (32.33 -- 43.89)	9.91 (8.53 -- 11.49)
Fujian	16.88 (10.42 -- 26.72)	6.62 (5.14 -- 8.24)	7.16 (3.76 -- 11.6)	1.15 (0.78 -- 1.57)	4.49 (3.85 -- 5.11)	2.61 (1.6 -- 4.23)	49.65 (44.98 -- 51.7)	40.29 (37.66 -- 42.77)	44.93 (40.15 -- 48.98)	10.76 (9.27 -- 12.38)
Gansu	19.42 (11.04 -- 30.75)	7.25 (5.68 -- 8.98)	6.92 (3.6 -- 10.59)	1.29 (0.85 -- 1.9)	5.75 (5.02 -- 6.51)	2.69 (1.58 -- 4.41)	50.44 (45.86 -- 52.28)	41.08 (38.63 -- 43.33)	20.55 (12.51 -- 27.22)	8.35 (7.01 -- 9.81)
Guangdong	24.71 (16.01 -- 37.9)	6.15 (4.65 -- 7.63)	7.59 (4.39 -- 11.64)	1.71 (1.08 -- 2.53)	3.86 (3.32 -- 4.43)	2.83 (1.76 -- 4.37)	48.71 (43.86 -- 50.85)	39.4 (36.5 -- 41.87)	36.65 (30.35 -- 42.03)	10.22 (8.79 -- 11.8)
Guangxi	22.27 (13.81 -- 33.79)	6.22 (4.85 -- 7.75)	7.21 (3.99 -- 11.28)	1.04 (0.7 -- 1.46)	4.16 (3.59 -- 4.8)	2.54 (1.54 -- 4.18)	50.65 (46.08 -- 52.47)	41.34 (38.68 -- 43.54)	32.82 (25.74 -- 39.12)	10.23 (8.8 -- 11.79)
Guizhou	16.46 (9.29 -- 27.63)	7.81 (6.04 -- 9.81)	6.85 (3.68 -- 11.09)	1.12 (0.76 -- 1.6)	5.59 (4.93 -- 6.28)	2.25 (1.32 -- 3.67)	51.35 (46.88 -- 53.07)	42.14 (39.74 -- 44.07)	45.67 (40.64 -- 49.52)	9.81 (8.41 -- 11.36)
Hainan	11.72 (6.82 -- 18.71)	6.37 (4.81 -- 8.09)	7.54 (4.25 -- 11.51)	1.96 (1.31 -- 2.9)	4.02 (3.49 -- 4.64)	2.87 (1.85 -- 4.47)	49.57 (44.95 -- 51.58)	40.2 (37.54 -- 42.55)	34.83 (27.98 -- 40.49)	10.98 (9.52 -- 12.61)
Hebei	38.1 (19.72 -- 57.21)	6.34 (4.78 -- 7.98)	7.04 (3.79 -- 10.92)	1.11 (0.78 -- 1.57)	5.97 (5.28 -- 6.72)	3.73 (2.24 -- 5.9)	49.77 (45.04 -- 51.75)	40.41 (37.66 -- 42.75)	48.21 (44.49 -- 51.22)	9.7 (8.34 -- 11.32)
Heilongjiang	24.95 (14.51 -- 37.88)	6.43 (4.79 -- 8.21)	7.89 (4.25 -- 12.05)	1.62 (1.07 -- 2.33)	6.45 (5.73 -- 7.3)	4.02 (2.49 -- 6.37)	48.66 (43.73 -- 50.84)	39.33 (36.29 -- 41.94)	43.21 (38.04 -- 47.73)	9.98 (8.54 -- 11.58)
Henan	34.92 (22.28 -- 51.88)	6.48 (5.08 -- 8.17)	7.05 (3.75 -- 10.86)	1.02 (0.69 -- 1.45)	5.64 (4.94 -- 6.42)	3.31 (1.97 -- 5.3)	50.53 (45.97 -- 52.37)	41.22 (38.79 -- 43.3)	47.34 (43.3 -- 50.57)	8.85 (7.5 -- 10.32)
Hubei	33.14 (21.13 -- 49.5)	6.66 (5.15 -- 8.35)	7.37 (3.92 -- 11.87)	0.91 (0.62 -- 1.27)	5.08 (4.42 -- 5.8)	3.1 (1.83 -- 4.88)	49.77 (45.09 -- 51.74)	40.4 (37.62 -- 42.65)	48.34 (44.23 -- 51.21)	9.28 (7.91 -- 10.78)
Hunan	28.59 (18.79 -- 40.97)	6.62 (5.06 -- 8.2)	7.17 (4.16 -- 10.74)	1.2 (0.79 -- 1.68)	4.86 (4.23 -- 5.55)	2.92 (1.83 -- 4.72)	50.18 (45.54 -- 52.05)	40.79 (38.17 -- 43.07)	40.55 (34.67 -- 45.32)	9.53 (8.13 -- 11.13)
Inner Mongolia	20.16 (11.31 -- 32.71)	6.58 (4.88 -- 8.27)	7.43 (4.13 -- 11.35)	1.39 (0.9 -- 2.14)	6.35 (5.59 -- 7.12)	3.13 (1.93 -- 4.9)	49.71 (45.06 -- 51.76)	40.32 (37.54 -- 42.62)	45.46 (40.64 -- 49.18)	9.62 (8.23 -- 11.18)
Jiangsu	33.44 (21.46 -- 51.49)	6.53 (5.19 -- 8.04)	7.6 (4.03 -- 12.08)	1 (0.67 -- 1.42)	5.06 (4.41 -- 5.74)	3.72 (2.38 -- 5.65)	48.74 (43.96 -- 50.91)	39.43 (36.72 -- 41.93)	48.41 (44.24 -- 51.4)	9.42 (8.04 -- 10.9)
Jiangxi	19.96 (12.8 -- 31.49)	6.77 (5.16 -- 8.37)	7.26 (3.73 -- 11.26)	1.12 (0.76 -- 1.6)	4.75 (4.15 -- 5.4)	2.67 (1.64 -- 4.25)	50.32 (45.68 -- 52.21)	40.95 (38.37 -- 43.21)	51.89 (49.38 -- 53.49)	8.95 (7.58 -- 10.43)
Jilin	28.4 (18.58 -- 42.28)	6.05 (4.47 -- 7.65)	7.71 (4.26 -- 11.64)	1.73 (1.11 -- 2.58)	5.71 (5.02 -- 6.44)	4.07 (2.47 -- 6.27)	49.36 (44.54 -- 51.35)	40.01 (37.26 -- 42.4)	50.59 (47.66 -- 52.73)	10.54 (9.13 -- 12.18)
Liaoning	37.96 (25.77 -- 56.22)	5.86 (4.38 -- 7.47)	7.82 (4.32 -- 12)	1.48 (0.94 -- 2.33)	5.79 (5.07 -- 6.54)	4.35 (2.81 -- 6.62)	48.01 (43.2 -- 50.34)	38.68 (35.65 -- 41.25)	36.04 (29.54 -- 41.68)	10.09 (8.71 -- 11.68)
Ningxia	25.28 (16.08 -- 38.89)	7.07 (5.49 -- 8.79)	7.4 (3.82 -- 11.93)	0.95 (0.64 -- 1.34)	5.66 (5.01 -- 6.35)	2.71 (1.62 -- 4.41)	49.75 (45.13 -- 51.76)	40.37 (37.6 -- 42.73)	29.38 (22.02 -- 35.61)	8.34 (7.03 -- 9.8)
Qinghai	22 (10.98 -- 34.68)	6.97 (5.48 -- 8.63)	7.28 (3.99 -- 11.08)	0.98 (0.68 -- 1.37)	5.78 (5.11 -- 6.51)	2.69 (1.65 -- 4.34)	49.56 (44.95 -- 51.62)	40.19 (37.38 -- 42.52)	48.69 (44.9 -- 51.63)	8.34 (7 -- 9.85)
Shaanxi	30.67 (18.75 -- 45.84)	6.69 (5.18 -- 8.26)	6.85 (3.86 -- 10.4)	0.81 (0.54 -- 1.14)	4.56 (3.94 -- 5.22)	2.64 (1.59 -- 4.33)	50.28 (45.6 -- 52.18)	40.91 (38.21 -- 43.21)	45.72 (40.78 -- 49.33)	8.67 (7.37 -- 10.14)
Shandong	35.89 (22.14 -- 53.88)	6.82 (5.24 -- 8.42)	7.46 (3.95 -- 11.6)	0.98 (0.66 -- 1.37)	4.04 (3.48 -- 4.63)	4.04 (2.43 -- 6.4)	49.47 (44.79 -- 51.48)	40.13 (37.39 -- 42.48)	46.71 (42.56 -- 49.87)	10.17 (8.81 -- 11.76)
Shanghai	49.59 (35.46 -- 69.68)	5.31 (3.99 -- 6.7)	8.74 (4.98 -- 12.74)	1.26 (0.85 -- 1.82)	4.83 (4.21 -- 5.47)	5.8 (4.05 -- 8.71)	43.65 (38.28 -- 46.91)	35.19 (31.73 -- 38.18)	48.64 (44.79 -- 51.65)	11.1 (9.67 -- 12.67)
Shanxi	30.76 (18.47 -- 48.69)	6.42 (4.91 -- 8.12)	7.15 (3.91 -- 10.98)	1.36 (0.91 -- 1.96)	5.74 (5.04 -- 6.5)	4.02 (2.41 -- 6.31)	49.69 (44.94 -- 51.69)	40.33 (37.6 -- 42.65)	27.72 (20.52 -- 33.75)	8.46 (7.12 -- 9.94)
Sichuan	27.45 (13.83 -- 41.15)	6.81 (5.3 -- 8.45)	7.18 (3.97 -- 11.16)	0.94 (0.65 -- 1.31)	4.77 (4.12 -- 5.46)	2.67 (1.56 -- 4.3)	50.56 (46.01 -- 52.37)	41.24 (38.78 -- 43.42)	41.64 (35.83 -- 46.19)	9.94 (8.48 -- 11.5)
Tianjin	52.79 (36.48 -- 76.91)	5.43 (4.06 -- 6.95)	7.52 (4.06 -- 11.12)	1.84 (1.19 -- 2.68)	5.08 (4.38 -- 5.77)	5.63 (3.66 -- 8.29)	42.43 (36.93 -- 45.79)	37.23 (33.96 -- 40.18)	35.28 (29.09 -- 40.73)	10.66 (9.26 -- 12.21)
Tibet	8.2 (3.69 -- 17.75)	6.93 (5.59 -- 8.08)	6.9 (3.62 -- 11.45)	0.98 (0.65 -- 1.37)	6.57 (5.86 -- 7.33)	2.29 (1.37 -- 3.75)	49.95 (45.19 -- 51.88)	40.59 (37.96 -- 42.95)	45.11 (40.05 -- 48.93)	8.67 (7.35 -- 10.22)
Xinjiang	33.01 (10.36 -- 66.96)	5.67 (4.38 -- 6.98)	7.07 (3.95 -- 10.24)	1.8 (1.17 -- 2.68)	5.4 (4.71 -- 6.11)	3.42 (2.07 -- 5.4)	49.65 (45.04 -- 51.56)	40.3 (37.46 -- 42.57)	30.2 (23.25 -- 36.36)	9.11 (7.72 -- 10.65)
Yunnan	13.15 (7.55 -- 22.29)	7.38 (5.62 -- 9.18)	6.81 (3.7 -- 10.78)	1.09 (0.73 -- 1.53)	4.32 (3.72 -- 4.92)	2.13 (1.27 -- 3.52)	50.64 (46.02 -- 52.45)	41.32 (38.85 -- 43.51)	39.52 (33.04 -- 44.6)	10.01 (8.6 -- 11.56)
Zhejiang	28.35 (17.33 -- 43.32)	6.8 (5.34 -- 8.43)	7.76 (4.08 -- 12.55)	1.08 (0.72 -- 1.57)	5.56 (4.88 -- 6.3)	3.13 (1.93 -- 4.97)	48.9 (44.26 -- 51.04)	39.6 (36.73 -- 41.94)	50.7 (47.63 -- 52.88)	9.98 (8.59 -- 11.54)

2017

Location name	Ambient particulate matter pollution	Smoking	Alcohol use	High fasting plasma glucose	High systolic blood pressure	High body-mass index	Diet low in fruits	Diet low in whole grains	Diet high in sodium	High LDL cholesterol
China	55.01 (43.37 -- 70.23)	6.82 (6.05 -- 7.61)	11.22 (7.42 -- 14.83)	1.58 (1.13 -- 2.16)	6.62 (5.93 -- 7.35)	9.27 (7.04 -- 12.58)	36.45 (30.83 -- 40.53)	40.86 (38.51 -- 42.83)	44.44 (40.56 -- 47.34)	10.52 (9.14 -- 12.14)
Taiwan	43.83 (27.89 -- 65.23)	5.76 (5.08 -- 6.48)	11.71 (6.63 -- 17.76)	2.67 (1.74 -- 3.84)	4.51 (3.96 -- 5.09)	15.66 (13.07 -- 19.26)	20.65 (14.41 -- 26.22)	41.51 (39.34 -- 43.4)	5.08 (0.95 -- 11.42)	14.32 (12.82 -- 16.09)
Hong Kong Special Administrative Region of China	42.23 (28.92 -- 60.06)	4.61 (3.64 -- 5.67)	13.35 (8.2 -- 18.89)	1.62 (0.98 -- 2.51)	4.83 (4.24 -- 5.43)	13.17 (10.38 -- 17.19)	27.57 (21.37 -- 32.41)	39.14 (36.05 -- 41.73)	49.82 (46.78 -- 52.04)	14.04 (12.51 -- 15.71)
Macau Special Administrative Region of China	45.31 (32.58 -- 61.68)	7.5 (5.72 -- 9.27)	10.4 (1.22 -- 17)	2.49 (1.44 -- 4.86)	7.09 (6.36 -- 7.88)	14.06 (11.1 -- 18.1)	32.13 (26.31 -- 36.55)	36.67 (33.16 -- 39.6)	50.99 (48.35 -- 52.82)	13.2 (11.71 -- 14.97)
Anhui	53.85 (34.75 -- 77.34)	7.33 (5.99 -- 8.67)	10.43 (5.67 -- 15.48)	1.5 (1.04 -- 2.12)	6.45 (5.68 -- 7.2)	9.32 (6.85 -- 12.94)	40.72 (35.01 -- 44.45)	42.29 (40.04 -- 44.29)	48.84 (44.81 -- 51.65)	9.81 (8.44 -- 11.43)
Beijing	86.01 (65.93 -- 99.76)	5.59 (4.29 -- 7.1)	14.66 (9.05 -- 20.95)	1.98 (1.33 -- 2.83)	6.79 (5.99 -- 7.64)	15.58 (12.27 -- 20.01)	27.89 (21.21 -- 33.9)	38 (34.78 -- 40.72)	27.04 (19.44 -- 33.3)	10.89 (9.48 -- 12.55)
Chongqing	53.42 (38.59 -- 75.58)	6.93 (5.69 -- 8.35)	10.09 (5.72 -- 13.94)	1.45 (1 -- 2.08)	6.66 (5.91 -- 7.39)	8.3 (5.86 -- 11.77)	37.81 (31.7 -- 42.17)	41.3 (38.66 -- 43.46)	46.9 (42.62 -- 50.34)	10.75 (9.3 -- 12.37)
Fujian	43.06 (31.09 -- 60.32)	7.28 (5.88 -- 8.7)	11.89 (6.18 -- 17.48)	1.51 (1.03 -- 2.15)	5.54 (4.86 -- 6.21)	8.49 (6.17 -- 11.94)	32.74 (26.25 -- 37.87)	39.69 (36.85 -- 42.16)	48.52 (44.55 -- 51.46)	11.72 (10.24 -- 13.39)
Gansu	36.09 (23.85 -- 52.88)	7.8 (6.26 -- 9.22)	9.41 (5.11 -- 13.36)	1.3 (0.89 -- 1.85)	6.66 (5.89 -- 7.42)	6.74 (4.68 -- 9.69)	42.6 (36.99 -- 46.1)	43 (40.8 -- 44.88)	33.99 (26.67 -- 39.81)	9.04 (7.76 -- 10.55)
Guangdong	51.19 (36.38 -- 71.32)	6.64 (5.32 -- 8.03)	12.21 (7.01 -- 17.3)	1.99 (1.35 -- 2.89)	5.97 (5.25 -- 6.72)	8.37 (6.13 -- 11.76)	31.59 (25 -- 37.08)	39.25 (36.27 -- 41.73)	43.28 (37.94 -- 47.56)	11.1 (9.66 -- 12.72)
Guangxi	41.55 (28.02 -- 60.01)	6.42 (5.13 -- 7.73)	10.16 (5.21 -- 15.01)	1.34 (0.93 -- 1.9)	5 (4.34 -- 5.66)	6.53 (4.62 -- 9.29)	40.29 (34.66 -- 44.18)	42.1 (39.75 -- 44.17)	19.29 (10.12 -- 26.38)	11.3 (9.84 -- 12.92)
Guizhou	32.32 (21.01 -- 49.8)	8.41 (6.82 -- 9.97)	9.45 (5.02 -- 14.37)	1.28 (0.87 -- 1.82)	6.9 (6.11 -- 7.69)	6.51 (4.53 -- 9.25)	43.83 (38.35 -- 46.99)	43.47 (41.39 -- 45.3)	44.72 (39.73 -- 48.77)	10.91 (9.47 -- 12.5)
Hainan	25.91 (16.4 -- 37.91)	7 (5.52 -- 8.53)	10.47 (5.31 -- 15.8)	1.92 (1.28 -- 2.66)	4.76 (4.11 -- 5.4)	6.96 (5 -- 9.91)	39.27 (33.71 -- 43.32)	41.74 (39.19 -- 43.76)	37.32 (30.96 -- 42.71)	11.96 (10.45 -- 13.64)
Hebei	73.59 (40.69 -- 97.88)	6.5 (5.25 -- 7.93)	10.87 (5.87 -- 15.49)	1.72 (1.17 -- 2.34)	7.66 (6.81 -- 8.46)	11.14 (8.32 -- 15.03)	36.9 (31.04 -- 41.24)	41.04 (38.25 -- 43.37)	47.39 (42.88 -- 50.68)	10.63 (9.26 -- 12.19)
Heilongjiang	48.04 (29 -- 71)	6.8 (5.41 -- 8.35)	11.74 (6.17 -- 17.55)	1.89 (1.27 -- 2.65)	7.49 (6.67 -- 8.3)	9.48 (6.9 -- 13.13)	38.77 (32.87 -- 43)	41.62 (39.06 -- 43.75)	36.81 (29.53 -- 42.53)	10.72 (9.31 -- 12.38)
Henan	65.37 (45.23 -- 91.42)	6.51 (5.28 -- 7.71)	10.56 (5.86 -- 15.11)	1.49 (1.03 -- 2.09)	6.81 (6.02 -- 7.59)	10.83 (8.07 -- 14.57)	38.61 (32.68 -- 42.72)	41.59 (39.06 -- 43.7)	47.95 (43.66 -- 51.12)	9.66 (8.32 -- 11.25)
Hubei	59.79 (42.45 -- 82.48)	6.56 (5.32 -- 7.79)	12.37 (6.23 -- 18.85)	1.26 (0.86 -- 1.8)	6.5 (5.75 -- 7.22)	7.65 (5.48 -- 10.82)	38.52 (32.79 -- 42.64)	41.55 (39.07 -- 43.65)	42.89 (37.33 -- 47.44)	9.97 (8.57 -- 11.5)
Hunan	50.61 (35.05 -- 71.65)	7.05 (5.7 -- 8.59)	10.45 (5.7 -- 14.94)	1.4 (0.95 -- 1.99)	6.18 (5.44 -- 6.86)	7.81 (5.62 -- 11.09)	39.38 (33.82 -- 43.35)	41.82 (39.37 -- 43.92)	46.67 (42.08 -- 50.14)	10.36 (8.94 -- 11.96)
Inner Mongolia	41.19 (22.19 -- 61.07)	6.81 (5.52 -- 8.21)	11.55 (6.27 -- 16.94)	1.69 (1.17 -- 2.34)	7.83 (6.99 -- 8.68)	10.58 (7.85 -- 14.23)	36.05 (30.2 -- 40.52)	40.73 (38.07 -- 43)	50.49 (47.52 -- 52.66)	11.06 (9.63 -- 12.75)
Jiangsu	68.41 (49.84 -- 94.81)	6.55 (5.29 -- 7.77)	13.58 (7.42 -- 19.97)	1.59 (1.08 -- 2.26)	6.73 (5.96 -- 7.47)	11.41 (8.58 -- 15.63)	31.35 (24.82 -- 36.58)	39.26 (36.33 -- 41.73)	47.48 (43.03 -- 50.71)	9.88 (8.52 -- 11.47)
Jiangxi	40.09 (27.62 -- 57.15)	7.05 (5.77 -- 8.24)	9.87 (5.21 -- 14.76)	1.27 (0.87 -- 1.8)	5.65 (4.98 -- 6.31)	6.74 (4.76 -- 9.69)	40.9 (35.45 -- 44.65)	42.35 (39.95 -- 44.42)	47.36 (42.79 -- 50.73)	9.71 (8.25 -- 11.29)
Jilin	53.73 (36.52 -- 74.29)	6.3 (4.95 -- 7.67)	11.91 (6.61 -- 17.29)	1.74 (1.22 -- 2.41)	7.25 (6.42 -- 8.07)	10.55 (7.9 -- 14.59)	37.73 (32.01 -- 41.85)	41.28 (38.62 -- 43.4)	42.6 (36.52 -- 47.32)	11.43 (10 -- 13.02)
Liaoning	61.78 (42.5 -- 87.11)	6.34 (4.87 -- 7.78)	12.46 (7.1 -- 18.16)	1.86 (1.27 -- 2.63)	8.92 (8.06 -- 9.73)	11.14 (8.34 -- 15.05)	34.93 (29.07 -- 39.72)	40.38 (37.66 -- 42.59)	43.58 (37.48 -- 48.4)	10.31 (8.9 -- 11.92)
Ningxia	47.92 (33.64 -- 66.85)	7.59 (6.1 -- 8.97)	10.08 (5.12 -- 15.52)	1.24 (0.83 -- 1.76)	6.54 (5.83 -- 7.25)	7.01 (4.79 -- 9.88)	39.88 (34.22 -- 43.88)	41.96 (39.54 -- 44.03)	41.54 (35.33 -- 46.24)	9.02 (7.66 -- 10.57)
Qinghai	41.86 (22.29 -- 62.49)	8.1 (6.65 -- 9.53)	9.4 (5.1 -- 13.87)	1.26 (0.85 -- 1.78)	6.53 (5.8 -- 7.28)	6.41 (4.51 -- 9.3)	40.58 (34.99 -- 44.48)	42.22 (39.79 -- 44.35)	48.6 (44.86 -- 51.5)	9.02 (7.67 -- 10.54)
Shaanxi	53.25 (34.42 -- 75.11)	7.04 (5.69 -- 8.49)	11.06 (5.72 -- 15.86)	1.27 (0.89 -- 1.81)	6.56 (5.78 -- 7.33)	7.86 (5.64 -- 11.05)	39.46 (33.69 -- 43.37)	41.86 (39.42 -- 43.93)	47.94 (43.75 -- 51.29)	9.43 (8.05 -- 10.95)
Shandong	68.61 (41.97 -- 96.48)	6.57 (5.38 -- 7.81)	11.93 (6.55 -- 17.16)	1.54 (1.05 -- 2.21)	6.85 (6.12 -- 7.61)	11.62 (8.78 -- 15.63)	33.04 (26.7 -- 38.1)	39.78 (37 -- 42.15)	50.21 (47.01 -- 52.46)	10.87 (9.45 -- 12.57)
Shanghai	71.43 (53.15 -- 96.73)	5.77 (4.49 -- 7.2)	13.64 (8.4 -- 20.02)	1.69 (1.18 -- 2.38)	6.08 (5.39 -- 6.8)	13.1 (10.06 -- 17.47)	28.62 (21.91 -- 34.72)	38.26 (35.09 -- 40.98)	45.14 (40.1 -- 49.21)	12.44 (10.94 -- 14.14)
Shanxi	55.69 (36.96 -- 79.25)	6.65 (5.31 -- 8.08)	10.37 (5.74 -- 14.48)	1.81 (1.27 -- 2.49)	7.74 (6.89 -- 8.59)	10.73 (7.9 -- 14.79)	39.22 (33.48 -- 43.35)	41.76 (39.18 -- 43.99)	46.99 (42.75 -- 50.35)	8.93 (7.57 -- 10.54)
Sichuan	48.36 (25.62 -- 69.86)	7.23 (5.85 -- 8.7)	10.71 (5.55 -- 15.9)	1.4 (0.96 -- 2)	6.36 (5.62 -- 7.11)	7.88 (5.65 -- 11.2)	39.79 (34.15 -- 43.7)	41.96 (39.64 -- 44.1)	45.78 (40.91 -- 49.46)	10.87 (9.41 -- 12.48)
Tianjin	85.42 (65.74 -- 99.31)	6.29 (4.94 -- 7.81)	13.02 (7.78 -- 18.32)	2.15 (1.49 -- 3.03)	6.82 (5.98 -- 7.66)	14.78 (11.33 -- 19.45)	27.28 (20.18 -- 33.52)	38.77 (35.65 -- 41.46)	25.51 (17.25 -- 32.29)	11.14 (9.7 -- 12.77)
Tibet	16.78 (7.86 -- 35.58)	7.67 (6.78 -- 8.55)	9.52 (4.79 -- 15.68)	1.1 (0.76 -- 1.55)	8.41 (7.57 -- 9.26)	4.99 (3.37 -- 7.46)	42.3 (37.02 -- 45.86)	42.83 (40.42 -- 44.73)	50.54 (47.53 -- 52.78)	9.38 (8 -- 10.93)
Xinjiang	57.51 (19.86 -- 97.99)	5.77 (4.76 -- 6.83)	9.32 (5.29 -- 12.85)	2.05 (1.4 -- 2.88)	6.08 (5.38 -- 6.82)	10.54 (7.84 -- 14.57)	38.74 (32.85 -- 42.8)	41.56 (39.01 -- 43.71)	52.07 (49.88 -- 53.6)	9.85 (8.47 -- 11.42)
Yunnan	29.09 (18.29 -- 44.09)	8.26 (6.57 -- 10)	9.74 (5.24 -- 14.63)	1.32 (0.9 -- 1.88)	5.44 (4.76 -- 6.14)	6.07 (4.28 -- 8.75)	41.39 (35.74 -- 45.07)	42.49 (40.17 -- 44.46)	46.18 (41.43 -- 49.99)	11.13 (9.69 -- 12.74)
Zhejiang	59.47 (41.76 -- 83.97)	7.3 (5.89 -- 8.69)	13.56 (7.41 -- 20.3)	1.44 (0.98 -- 2.09)	6.4 (5.67 -- 7.13)	9.85 (7.29 -- 13.54)	32.24 (25.83 -- 37.34)	38.72 (35.62 -- 41.21)	43.71 (39.59 -- 46.97)	10.58 (9.19 -- 12.16)

Province Name	DSP site code	DSP site name	DSP site population	Total population in the province
Beijing	110101	Dongcheng District	604513.9718	14930135
Beijing	110112	Tongzhou District	780922.6182	14930135
Tianjin	120101	Heping	321750.498	10239996
Tianjin	120223	Jinghai County	572251.1322	10239996
Tianjin	120225	Ji County	832972.0043	10239996
Hebei	130983	Huangye Shi	490601.8175	68090013
Hebei	130481	Wuan Shi	735516.7257	68090013
Hebei	130205	Kaiping District	270359.044	68090013
Hebei	130102	Changan District	506627.5135	68090013
Hebei	130702	Qiaodong District	284763.7609	68090013
Hebei	130826	Fengning Manchu Zizhi County	345475.5537	68090013
Shanxi	140202	Cheng District	554298.9512	33349976
Shanxi	140107	Xinghualing District	536843.4453	33349976
Shanxi	140321	Pingding County	332341.2635	33349976
Shanxi	140826	Jiang County	272714.8795	33349976
Shanxi	141123	Xing County	266924.5567	33349976
Inner Mongolia	152524	Sonid Youqi	77326.63386	23840012
Inner Mongolia	150523	Kairu County	396290.0532	23840012
Inner Mongolia	150103	Huimin District	291293.4733	23840012
Liaoning	210921	Fuxin Mongolia Zizhi County	728648.0976	42169982
Liaoning	211081	Dengta City	512095.8059	42169982
Liaoning	210204	Shahekou District	639936.46	42169982
Liaoning	210703	Linehe District	373932.2973	42169982
Liaoning	210682	Fengcheng City	565861.3818	42169982
Jilin	222405	Longjing City	258825.3265	27089986
Jilin	220183	Dehui City	891926.7159	27089986
Jilin	220102	Nanguan District	579918.0905	27089986
Jilin	220582	Jian City	239689.8508	27089986
Heilongjiang	230523	Baoqing County	415836.3586	38170003
Heilongjiang	230223	Yian County	475424.9103	38170003
Heilongjiang	230103	Nangang District	1008730.103	38170003
Heilongjiang	230606	Datong District	248564.292	38170003
Shanghai	310117	Songjiang District	674830.4892	17419951
Shanghai	310103	Luwan District	363370.6569	17419951
Jiangsu	320831	Jinhu County	369199.4317	74329999
Jiangsu	320684	Haimen City	940764.6227	74329999
Jiangsu	320582	Zhangjiagang City	986744.2549	74329999
Jiangsu	320303	Yunlong District	226251.6228	74329999
Jiangsu	320102	Xuanwu District	494664.7379	74329999
Zhejiang	330703	Jinhua District	483479.8131	47200016
Zhejiang	330702	Wucheng District	436325.3183	47200016
Zhejiang	330324	Yongjia County	755123.6687	47200016
Zhejiang	330283	Fenghua City	484411.8922	47200016
Zhejiang	330183	Fuyang City	648450.716	47200016
Zhejiang	330103	Xiacheng District	431873.585	47200016
Anhui	341823	Jing County	361362.6252	64610023
Anhui	341622	Mengcheng County	1221042.861	64610023
Anhui	341402	Juchao District	848748.9237	64610023
Anhui	341181	Tianchang City	645612.1591	64610023
Anhui	340803	Daguan District	185427.579	64610023
Anhui	340103	Zhongshi District	273057.2185	64610023
Fujian	350822	Yongding County	420664.1364	35109978
Fujian	350784	Jianyang City	326197.5419	35109978
Fujian	350521	Huian County	936376.9722	35109978
Fujian	350402	Meilie District	158389.2931	35109978
Fujian	350104	Cangshan District	485256.0442	35109978
Jiangxi	360923	Shanggao County	351488.059	42840023
Jiangxi	360727	Longnan County	300112.5528	42840023
Jiangxi	360702	Zhanggong District	520470.9649	42840023
Jiangxi	360423	Wuning County	364768.7672	42840023

Jiangxi	360102	Donghu District	515643.2015	42840023
Shandong	370302	Chaochuan District	708686.7739	91800003
Shandong	370203	Shibei District	516837.1719	91800003
Shandong	371327	Junan County	979281.2096	91800003
Shandong	371482	Yucheng City	507025.8388	91800003
Shandong	371202	Laicheng city	1018957.146	91800003
Shandong	370832	Liangshan County	689688.233	91800003
Shandong	370811	Rencheng District	658492.3131	91800003
Shandong	370684	Penglai city	505913.0636	91800003
Shandong	370602	Zhifu District	695678.0787	91800003
Shandong	370323	Yiyuan county	551109.1633	91800003
Henan	411621	Fugou county	706271.413	97170012
Henan	411422	Sui County	804512.9763	97170012
Henan	411328	Tanghe County	1228720.672	97170012
Henan	410782	Hui County	824305.0571	97170012
Henan	410411	Zhanhe District	250907.3698	97170012
Henan	410102	Zhongyuan district	625547.2458	97170012
Hubei	429006	Tianmen city	1630761.587	60159995
Hubei	421002	Sashi District	600098.9136	60159995
Hubei	420625	Gucheng county	555592.7542	60159995
Hubei	420607	XiangYang County	1309968.296	60159995
Hubei	420102	Jiangan District	736985.5689	60159995
Hunan	433123	Fenghuang county	364322.4343	66979974
Hunan	431281	Hongjiang City	509658.5349	66979974
Hunan	431003	Suxian District	361010.1351	66979974
Hunan	430602	Yueyanglou District	576890.7248	66979974
Hunan	430181	Liuyang City	1384440.185	66979974
Hunan	430102	Furong District	410156.0895	66979974
Guangdong	441424	Wuhua county	855973.7544	83040105
Guangdong	440781	Taishan city	922313.802	83040105
Guangdong	440511	Jinping District	739582.7472	83040105
Guangdong	440282	Nanxiong City	360609.5743	83040105
Guangdong	440104	Yuexiu district	376507.9119	83040105
Guangxi	451225	Luocheng Mulao ethnic Zizhi county	359465.1232	48889989
Guangxi	451027	Lingyun county	196991.4298	48889989
Guangxi	450521	Hepu county	960414.506	48889989
Guangxi	450302	Xiufeng District	140114.7633	48889989
Guangxi	450126	binyang County	949401.1372	48889989
Guangxi	450122	Wuming County	670495.2495	48889989
Guangxi	450102	Xinning district	224482.8939	48889989
Hainan	469005	Wenchang city	538346.5915	8180004
Hainan	460108	Meilan district	382984.3343	8180004
Chongqing	500225	Dazu county	928922.1315	31220006
Chongqing	500101	Wanzhou district	1679559.789	31220006
Sichuan	511823	Hanyuan county	368629.1397	87248733
Sichuan	511325	Xichong county	605615.0395	87248733
Sichuan	511025	zizhong county	1359563.272	87248733
Sichuan	510184	Chongzhou city	682941.8637	87248733
Sichuan	510105	Qingyang District	580558.3694	87248733
Guizhou	522726	dushan county	350265.2497	39039986
Guizhou	522623	Shibing county	151078.0978	39039986
Guizhou	522223	Yuping dongzu zizhi county	138117.5563	39039986
Guizhou	520402	Xixiu District	850152.0902	39039986
Guizhou	520328	Meitan county	455361.7885	39039986
Guizhou	520302	Honghuagang district	439383.7538	39039986
Yunnan	532923	Xiangyun county	456196.9266	44149998
Yunnan	532627	Guangnan county	766579.074	44149998
Yunnan	530423	tonghai county	283930.2877	44149998
Yunnan	530103	Panlong District	379306.2377	44149998
Tibet	540127	Mozhu gongka county	40705.46041	2740000
Tibet	540102	Chengguan District	232314.2442	2740000

Shaanxi	610921	hanyin county	248125.5281	37050032
Shaanxi	610629	Luochuan County	212132.9248	37050032
Shaanxi	610582	Hua yin city	254811.1432	37050032
Shaanxi	610326	Mei County	303180.3375	37050032
Shaanxi	610202	Wangyi District	198491.7211	37050032
Shaanxi	610103	Beilin District	723947.8983	37050032
Gansu	623021	Lintan county	147423.0078	26189998
Gansu	620982	Dunhuang city	194399.1384	26189998
Gansu	620702	zhangye city	511784.0377	26189998
Gansu	620503	BeiDao District	568305.0946	26189998
Gansu	620423	Jingtai county	236795.2513	26189998
Gansu	620102	chengguan District	974600.7165	26189998
Qinghai	632221	Menyuan huizu zizhi county	159256.4549	5390007
Qinghai	632126	Muzhu tuzu zizhi county	402006.3447	5390007
Qinghai	630103	Chengzhong District	165360.7961	5390007
Ningxia	640502	Shapotou District	344515.0733	5879999
Ningxia	640323	Yanchi County	165225.2794	5879999
Ningxia	640122	Helan county	190044.8287	5879999
Ningxia	640104	Xingqing district	304917.7427	5879999
Xinjiang	654202	wusu city	203775.1076	19630010
Xinjiang	654025	Xinyuan county	291253.5212	19630010
Xinjiang	653221	Hetian county	279851.3807	19630010
Xinjiang	652302	fukang city	161676.0826	19630010
Xinjiang	650102	Tianshan District	500794.0534	19630010

Province Name	DSP site code	DSP site name	DSP site population	Total population in the province
Beijing	110101	Dongcheng District	644178	15810149
Beijing	110112	Tongzhou District	827231	15810149
Tianjin	120225	Ji County	876540	10750010
Tianjin	120106	Hongqiao district	569186	10750010
Hebei	130227	Qianxi County	363263	68980073
Hebei	130302	Haigang District	598733	68980073
Hebei	130826	Fengning Manchu Zizhi County	347630	68980073
Hebei	130721	Xuanhua County	297947	68980073
Hebei	130427	Ci County	669299	68980073
Hebei	130481	Wuan City	744516	68980073
Hebei	130205	Kaiping District	272439	68980073
Hebei	130702	Zhangjiakou City	286169	68980073
Shanxi	140321	Pingding County	335827	33749905
Shanxi	140427	Huguan County	294721	33749905
Shanxi	140107	Xinghualing District	550353	33749905
Shanxi	140602	Shuocheng District	407305	33749905
Shanxi	141124	Lin County	581092	33749905
Shanxi	140826	Jiang County	276539	33749905
Inner Mongolia	152524	Sonid Youqi	77007	23970100
Inner Mongolia	150802	Linhe District	536610	23970100
Inner Mongolia	150523	Kailu County	400662	23970100
Inner Mongolia	150423	Bairin Youqi	181634	23970100
Inner Mongolia	150103	Huimin District	293924	23970100
Liaoning	210921	Fuxin Mongolia Zizhi County	741181	42709973
Liaoning	210682	Fengcheng City	573261	42709973
Liaoning	210311	Qianshan District	372581	42709973
Liaoning	210204	Shahekou District	647548	42709973
Liaoning	210113	Shenbei New District	312164	42709973
Liaoning	211021	Liaoyang County	586260	42709973
Jilin	220102	Nanguan District	584214	27229991
Jilin	220183	Dehui City	899338	27229991
Jilin	222405	Longjing City	257622	27229991
Jilin	220211	Fengman District	277269	27229991
Jilin	220582	Jian City	239173	27229991
Heilongjiang	230826	Huachuan County	197903	38230046
Heilongjiang	230606	Datong Distrcit	247229	38230046
Heilongjiang	230523	Baoqing County	414208	38230046
Heilongjiang	230305	Lishu District	98866	38230046
Heilongjiang	230223	Yian County	478104	38230046
Heilongjiang	230208	Meiris Daur District	175783	38230046
Heilongjiang	230103	Nangang District	995387	38230046
Shanghai	310117	Songjiang District	706791	18150005
Shanghai	310101	Huangpu District	649355	18150005
Jiangsu	320921	Xiangshui County	592826	75499930
Jiangsu	320831	Jinhu County	373681	75499930
Jiangsu	320582	Zhangjiagang City	1008524	75499930
Jiangsu	320506	Wuzhong District	444447	75499930
Jiangsu	320303	Yunlong District	233002	75499930
Jiangsu	320111	Pukou District	521837	75499930
Zhejiang	331123	Suichang County	219328	49799997
Zhejiang	330702	Wucheng District	462075	49799997
Zhejiang	330523	Anji County	463690	49799997
Zhejiang	330483	Tongxiang City	767615	49799997
Zhejiang	330283	Fenghua City	510852	49799997
Zhejiang	330103	Xiacheng District	455967	49799997
Anhui	341823	Jing County	339567	61099951
Anhui	341622	Mengcheng County	1159980	61099951
Anhui	341181	Tianchang City	607128	61099951
Anhui	340803	Daguan District	175474	61099951

Anhui	340504	Yushan District	272902	61099951
Anhui	340181	Chaohu City	800928	61099951
Fujian	350521	Huian County	956087	35579926
Fujian	350402	Meilie District	159052	35579926
Fujian	350902	Jiaocheng District	420454	35579926
Fujian	350822	Yongaing County	425177	35579926
Fujian	350783	Jianou City	495007	35579926
Jiangxi	360923	Shanggao County	354729	43391301
Jiangxi	360727	Longnan County	303846	43391301
Jiangxi	360702	Zhanggong District	525725	43391301
Jiangxi	360423	Wuning County	369628	43391301
Jiangxi	360102	Donghu District	521743	43391301
Shandong	371327	Junan County	990475	93089983
Shandong	371202	Laicheng District	1030472	93089983
Shandong	370785	Gaomi City	863819	93089983
Shandong	370684	Penglai City	510219	93089983
Shandong	370602	Zhifu District	698362	93089983
Shandong	370403	Xuecheng District	472053	93089983
Shandong	370323	Yiyuan County	556802	93089983
Shandong	370213	Licang District	367994	93089983
Shandong	370203	Shibei District	530881	93089983
Henan	411502	Shihe District	591475	93920033
Henan	411422	Sui County	777492	93920033
Henan	411328	Tanghe County	1188722	93920033
Henan	410782	Hui County	797189	93920033
Henan	410526	Hua County	1231258	93920033
Henan	410323	Xinan County	496686	93920033
Henan	410306	Jilu District	71538	93920033
Henan	410102	Zhongyuan District	606357	93920033
Hubei	429006	Tianmen City	1545144	56930037
Hubei	420923	Yunmeng County	548633	56930037
Hubei	420625	Gucheng County	524987	56930037
Hubei	420503	Wujiagang District	175902	56930037
Hubei	420202	Huangshigang District	187322	56930037
Hubei	420102	Jiangan District	706185	56930037
Hunan	433123	Fenghuang County	345237	63420010
Hunan	431281	Hongjiang City	480392	63420010
Hunan	431003	Suxian District	342164	63420010
Hunan	430702	Wuling District	525964	63420010
Hunan	430626	Pingjiang County	929284	63420010
Hunan	430181	Liuyang City	1312439	63420010
Hunan	430103	Tianxin District	394182	63420010
Guangdong	445302	Yunfu City Yuncheng District	287166	93040103
Guangdong	441502	Shantou City	460796	93040103
Guangdong	441424	Wuhua County	961474	93040103
Guangdong	441284	Sihui City	453739	93040103
Guangdong	440282	Nanxiong City	402101	93040103
Guangdong	440104	Yuexiu District	425027	93040103
Guangxi	451225	Luocheng Mulam County	347121	47190027
Guangxi	451027	Lingyun County	190674	47190027
Guangxi	450521	Hepu County	925875	47190027
Guangxi	450302	Xiufeng District	134684	47190027
Guangxi	450205	Liubei District	423293	47190027
Guangxi	450126	Binyang County	916441	47190027
Hainan	469021	Dingan County	303775	8359981
Hainan	460108	Meilan District	388643	8359981
Chongqing	500111	Dazu District	37634	28079983
Chongqing	500101	Wanzhou District	1502666	28079983
Sichuan	513434	Yuexi County	245385	81690065
Sichuan	513321	Kangding County	113201	81690065

Sichuan	511823	Hanyuan County	345443	81690065
Sichuan	511325	Xichong County	565161	81690065
Sichuan	511025	Zizhong County	1275014	81690065
Sichuan	510411	Renhe District	211402	81690065
Sichuan	510182	Pengzhou City	759463	81690065
Sichuan	510105	Qingyang District	544790	81690065
Guizhou	520328	Meitan County	436366	37571716
Guizhou	520302	Honghuagang District	422032	37571716
Guizhou	522726	Dushan County	336261	37571716
Guizhou	522623	Shibing County	145078	37571716
Guizhou	522223	Yuping Dong Zizhi County	131825	37571716
Yunnan	533325	Lanping Bai Pumi Zizhi County	210809	44830030
Yunnan	532923	Xingyun County	461471	44830030
Yunnan	532823	Mengla County	251738	44830030
Yunnan	532627	Guangnan County	782049	44830030
Yunnan	530423	Tonghai County	286675	44830030
Yunnan	530402	Hongta District	435116	44830030
Tibet	542623	Minlin County	18980	2809878
Tibet	542323	Jiangzi County	67332	2809878
Tibet	542221	Naidong County	64210	2809878
Tibet	540127	Mozhu Gongka County	41718	2809878
Tibet	540102	Chengguan District	237222	2809878
Shanxi	610921	Hanyin County	248003	37349966
Shanxi	610629	Luochuan County	215090	37349966
Shanxi	610582	Huayin County	257112	37349966
Shanxi	610326	Wngyi District	305646	37349966
Shanxi	610202	Tongchuan City	199828	37349966
Gansu	623021	Lintan County	146467	26060171
Gansu	620982	Dunhuang City	192397	26060171
Gansu	620702	Ganzhou District	510916	26060171
Gansu	620503	Maiji District	567950	26060171
Gansu	620423	Jingtai County	236078	26060171
Qinghai	632221	Menyuan Hui Zizhi County	162405	5479964
Qinghai	632121	Pingan County	121500	5479964
Qinghai	630103	Chengzhong District	169142	5479964
Ningxia	640502	Zhongweicheng District	353918	6039993
Ningxia	640104	Xingqing District	282036	6039993
Xinjiang	654025	Xinyuan County	306658	20499990
Xinjiang	653221	Hetian County	294014	20499990
Xinjiang	653125	Shache Cpunty	695813	20499990
Xinjiang	652925	Xinhe County	156543	20499990
Xinjiang	650102	Tianshan District	519743	20499990

Province Name	DSP site code	DSP site name	DSP site population	Total population in the province
Beijing	110101	Dongcheng District	1002090	21148000
Beijing	110106	Fengtai District	2286311	21148000
Beijing	110109	Mentougou District	316329	21148000
Beijing	110112	Tongzhou District	1277827	21148000
Beijing	110114	Changping District	1782728	21148000
Beijing	110228	Miyun County	474856	21148000
Beijing	110229	Yanqing County	319960	21148000
Tianjin	120103	Hexi District	995371	14722100
Tianjin	120104	Nankai District	1160759	14722100
[truncated: 93,709 more chars]
